# Characterization and Small RNA Content of Extracellular Vesicles in Follicular Fluid of Developing Bovine Antral Follicles

**DOI:** 10.1038/srep25486

**Published:** 2016-05-09

**Authors:** Raphatphorn Navakanitworakul, Wei-Ting Hung, Sumedha Gunewardena, John S. Davis, Wilaiwan Chotigeat, Lane K. Christenson

**Affiliations:** 1 Department Molecular and Integrative Physiology, University of Kansas Medical Center, Kansas City, Kansas 66160 USA; 2 Department of Molecular Biotechnology and Bioinformatics, Faculty of Science, Prince of Songkla University, Hat Yai, Songkhla, 90112 Thailand; 3 Department of Biomedical Sciences, Faculty of Medicine, Prince of Songkla University, Hatyai, Songkhla, 90112 Thailand; 4 Department of Biostatistics, University of Kansas Medical Center, Kansas City, Kansas 66160 USA; 5 VA Nebraska-Western Iowa Health Care System and Department of Obstetrics and Gynecology, University of Nebraska Medical Center, Omaha, NE 68198, USA; 6 Center for Genomics and Bioinformatics Research, Faculty of Science, Prince of Songkla University, Hat Yai, Songkhla, 90112 Thailand

## Abstract

Exosomes and microvesicles (i.e., extracellular vesicles: EVs) have been identified within ovarian follicular fluid and recent evidence suggests that EVs are able to elicit profound effects on ovarian cell function. While existence of miRNA within EVs has been reported, whether EV size and concentration as well as their cargos (i.e., proteins and RNA) change during antral follicle growth remains unknown. Extracellular vesicles isolated from follicular fluid of small, medium and large bovine follicles were similar in size, while concentration of EVs decreased progressively as follicle size increased. Electron microscopy indicated a highly purified population of the lipid bilayer enclosed vesicles that were enriched in exosome biomarkers including CD81 and Alix. Small RNA sequencing identified a large number of known and novel miRNAs that changed in the EVs of different size follicles. Ingenuity Pathway Analysis (IPA) indicated that miRNA abundant in small follicle EV preparations were associated with cell proliferation pathways, while those miRNA abundant in large follicle preparations were related to inflammatory response pathways. These studies are the first to demonstrate that EVs change in their levels and makeup during antral follicle development and point to the potential for a unique vesicle-mediated cell-to-cell communication network within the ovarian follicle.

Cell-to-cell communication between somatic cells (theca, granulosa, and cumulus) and germ cells (oocytes) are critical for the process of folliculogenesis^1^,^2^. During folliculogenesis, the coordinated communication and signaling between the different follicular cell types is crucial for growth, maturation, and release of an oocyte that can be fertilized and undergo embryonic development. The endocrine hormones, follicle stimulating hormone (FSH) and luteinizing hormone (LH), play key regulatory roles in all aspects of this developmental progression from a preantral to a periovulatory follicle. Additionally, growth factors within the periovulatory follicle are essential for transmission of these endocrine signals to the deeper residing cumulus cells and oocyte within the follicle^3^,^4^,^5^,^6^. Likewise the oocyte secretes factors (i.e., GDF-9, BMP15) that are able to influence the nearby mural granulosa cells^7^,^8^. The antrum that separates these distinct cells is filled with a complex fluid derived from both blood plasma that arises from increased permeability of the thecal capillaries and release of secretory factors from both types of granulosa cells and oocytes^9^,^10^. Hormones (e.g., FSH, LH, GH, inhibin, activin, estrogens, and androgens), growth factors, proteins, lipoproteins, peptides, amino acids, and nucleotides have been found to be enriched in follicular fluid and provide an important microenvironment for follicle growth and oocyte maturation^11^,^12^,^13^. However, with the exception of several growth factors and steroids, much of the material observed in follicular fluid has not been well characterized and very little is known about the functional role of these molecules.

Recent studies have demonstrated that membrane-enclosed vesicles called microvesicles which are heterogeneous in size (100–1000 nm) contain bioactive molecules such as proteins, messenger RNA (mRNA) and microRNA (miRNA)^14^,^15^ which are released from cells by outward budding and fission of the plasma membrane 16. Similar to microvesicles, exosomes also have a lipid bilayer but are more homogeneous and smaller in size (30–100 nm). Exosomes are derived from intraluminal invagination of the multivesicular bodies (MVBs), followed by fusion of the MVB with the plasma membrane and release of its exosomal cargo into the extracellular space^17^,^18^,^19^,^20^. It should be noted that following cellular release the distinction of exosomes from microvesicles cannot be determined; they will be referred to extracellular vesicles (EVs) from here on. Recent evidence suggests that EVs can play important roles in cell-to-cell communication by transferring proteins, RNA, and miRNA molecules to target cells^14^,^20^. Several studies have now confirmed the existence of EVs in follicular fluid of mares and women at different ages^21^,^22^,^23^,^24^, in women with polycystic ovarian syndrome^25^,^26^, and in cow follicles at a single stage of development^27^. Our recent work has documented that EVs from small and large cow follicles can differentially support cumulus expansion and changes in cumulus-oocyte-complex (COC) gene expression^28^. However, to date no studies have evaluated whether the numbers of vesicles and contents of these vesicles change during ovarian follicular development. The cow was chosen for these studies for several reasons: the cow is a monoovulatory species with a similar temporal follicular growth and regression dynamics to that of the human (endocrine profiles do however differ between these species), the cow follicle provides sufficient levels of readily available fluid from early (small, 3–5 mm) antral follicles, samples that can not be feasibly collected in humans or rodents for comparison to later-staged (medium, 6–9 mm and large >9mm) antral follicles and understanding ovarian physiology in the cow has intrinsic value to the livestock industry.

## Results

### Quantitation and morphological characterization of follicular fluid EV from growing antral follicles

Bovine follicles subdivided by size (small, 3–5 mm; medium, 6–9 mm; and large >9 mm) representing early to late stages of antral follicle growth were pooled by size to ensure enough material for initial EV collection and evaluation. To provide an indication of the relative numbers of healthy follicles versus atretic follicles in each of the 9 pools (i.e., three independent pools at each size), granulosa cells were isolated from a subset (n = 18–20) of individual follicles from each follicle stage. Individual follicles had between 28 and 100% normal intact nuclear staining with the majority (~70%) of the follicles in each group containing less than 25% fragmented nuclei. Compared across the follicular sizes, each pool thus contains between 71% to 81% intact nuclei (Suppl Fig. 1). Analysis of estrogen and progesterone levels in the 9 independent follicular fluid pools indicated that progesterone levels did not vary across the follicle sizes whereas estradiol concentrations increased with size as expected (Suppl Fig. 2).

**Figure 1:**
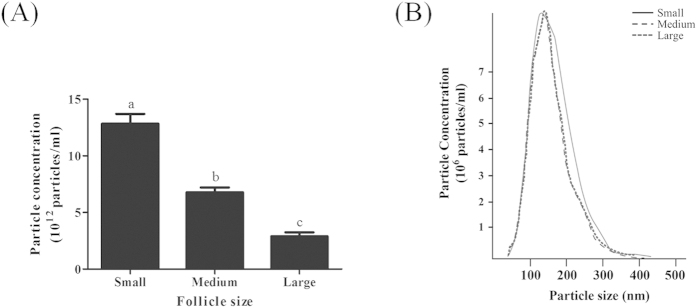
Particle concentrations and size distributions in EV preparations (n = 3) from small, medium and large follicles. (**A**) Mean concentration of particles (x 10^12^ particles per ml of follicular fluid). ^a,b,c^Means ± SEM with different superscripts were statistically different (P < 0.0001). (**B**) Nanoparticle tracking analysis predicted concentration and size distribution of particles in three representative EV preparations from follicular fluid of small, medium and large follicles.

Total EV concentrations (x 10^12^ particles/ml of follicular fluid) were highest in the smallest diameter (3–5 mm) follicles and the number decreased progressively as follicle diameter increased (Fig. 1A). EV size distributions ranged from ~30 to 300 nm as determined by nanoparticle tracking analysis (NTA) and there was no change in size distribution from the three different sized follicles (Fig. 1B). Additionally, the mode and mean size of EVs was not significantly different in the three different follicle diameter groups (Suppl. Table 1).

**Figure 2:**
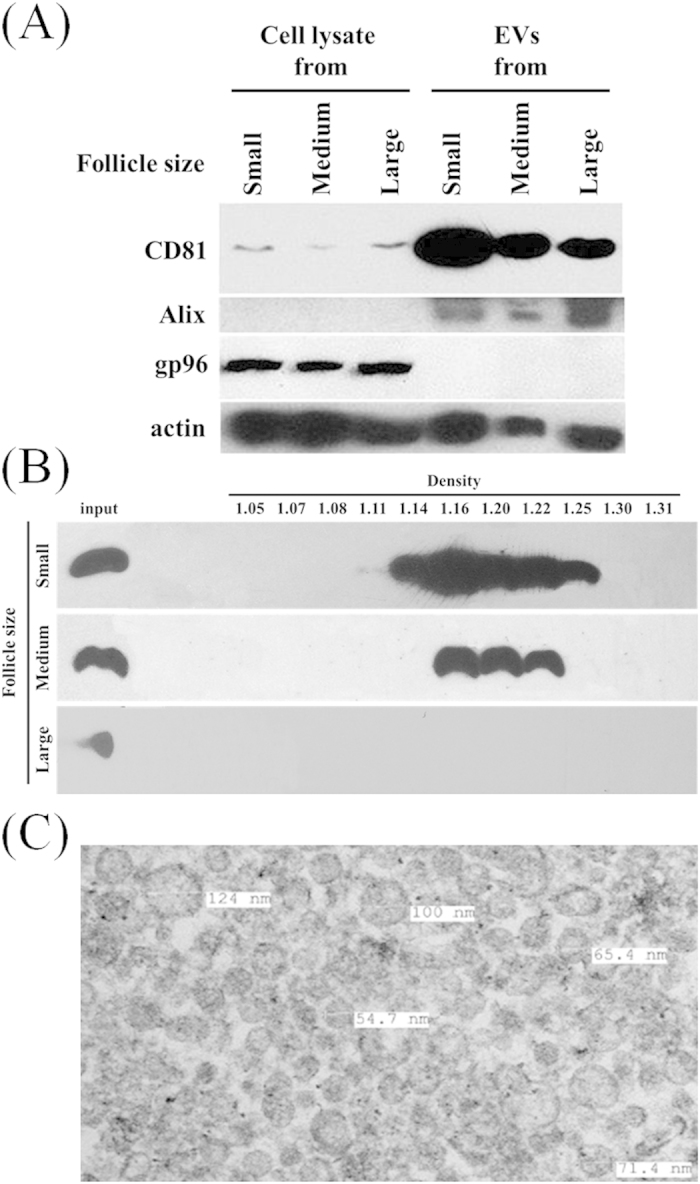
Quantitative and qualitative analysis of the EVs from small, medium and large follicles. (**A**) Western blot analysis of extracellular vesicle (CD81 and Alix), endoplasmic reticulum (GP96) and cellular (actin) markers in EV and cellular lysates. Equal amounts of total protein (10 μg) were loaded into each lane. (**B**) Representative CD81 protein in sucrose gradient fractions of small, medium and large follicles. Each fraction density was determined by refractometry and an equal volume of the resulting resuspended pellets was loaded for western blot analysis (n = 3). Input represents an aliquot of the EV sample that was applied to the sucrose gradient. (**C**) Representative transmission electron microscopy image of a thin section through an EV pellet isolated from a small follicle–several vesicles diameters (nm) are labeled.

Several established exosome markers, tetraspanin CD81 and Alix were used to confirm the presence and enrichment of exosomes in the EV preparations (Fig. 2A). We found that the expression of CD81 was highly expressed in EVs as compared to granulosa cells that were isolated from the initial 800 g centrifugation run in the EV isolation protocol (Fig. 2A). Interestingly, the highest expression of CD81 was found in EVs (10 μg total loaded protein) derived from small follicles and decreased progressively in the EVs as follicle size increased (Fig. 2A). Alix another exosome marker was also abundant in the EV preparations compared to the cell lysates; however it did not show the same decrease in levels as that detected for CD81 as follicle size increased. This later antibody/marker gene exhibited greater variation across samples and was considered less reliable. The endoplasmic reticulum marker GP96 was detected in cell lysates and was not detectable in the EV preparations indicating the absence of other cellular membrane contamination in the EV preparations. Actin was found in both EVs and cells (Fig. 2A). Western blot analysis of CD81 in sucrose gradient fractions of small, medium and large follicle EV preparations indicated that EVs were found in fractions 5–9 of small and medium follicles at densities (1.14 to 1.25 g/cm^3^) that are consistent with extracellular vesicles (i.e, exosomes; Fig. 2B). The tetraspanin CD81 was not readily detectable in the large follicle EV preparations following sucrose gradient analysis; this is likely due the decreased level of CD81 in the starting EV ultracentrifuge pellet “input” which is shown for each preparation (Fig. 2B). Transmission electron microscopy (TEM) revealed that the EV pellets contained a homogeneous population of circular bilayer enclosed vesicles with minimal evidence of protein contamination (very dark staining material that is not enclosed within a lipid bilayer: Fig. 2C). We observed no vesicles >130 nm in diameter by TEM; however because these are cross-sectional views exact sizes are not determinable using this approach.

### Small RNAs within follicular fluid EVs

A representative electropherogram for small, medium and large follicles shows the size distribution of small RNAs derived from the EV RNA isolation in the interval of 6–150 nucleotides (Suppl Fig. 3A). Note that the majority of the small RNA ranged in size between 20 and 40 nucleotides in length consistent with microRNA (miRNA). Moreover, no detectable amounts of the highly abundant 18S and 28S ribosomal RNA (rRNA) were detected in the EV preparations indicating an absence of cellular contamination (data not shown). The percentage of EV RNA that was consistent with the size of miRNA (i.e., 18–24 nucleotides) differed across the follicle size groups with the greatest presence (84%) in the 3–5 mm, slightly lower (75%) amounts in 6–9 mm and the least 58% in the >9 mm diameter follicles (p < 0.0001; Suppl Fig. 3B). The A260/280 and A260/230 values ranged from 1.56–1.74 and 0.13–0.62, respectively. FastQC analysis indicated that all 9 samples had similar high quality sequence data.

**Figure 3:**
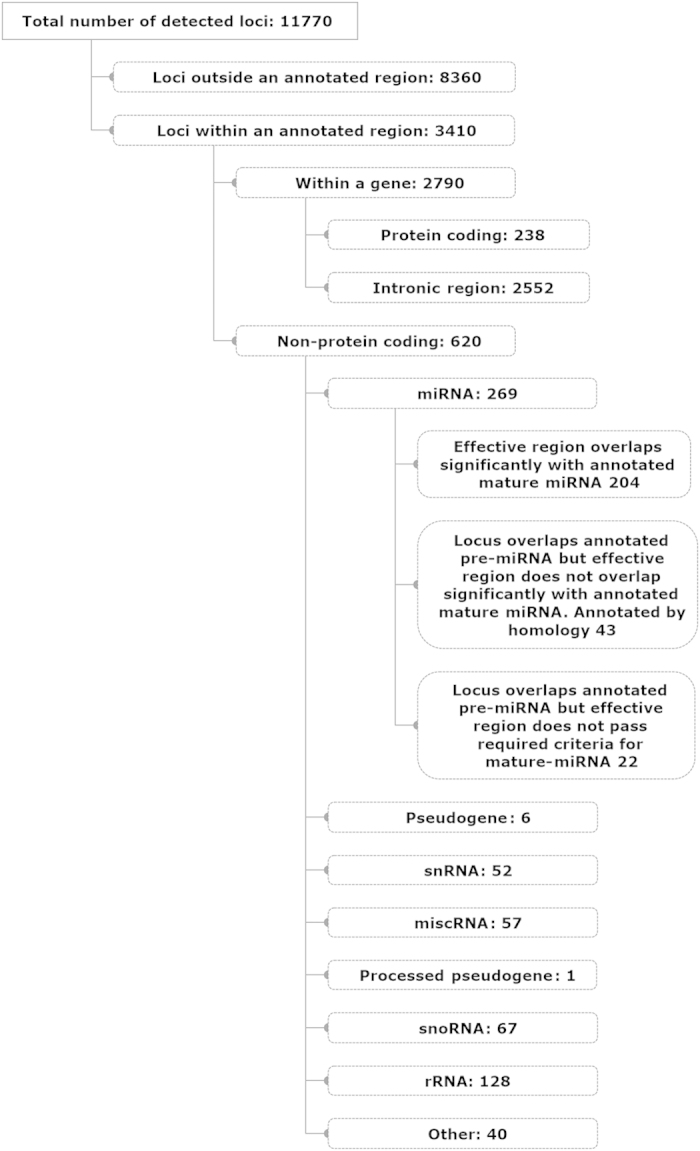
Composite annotation and breakdown of small RNA generated from all small, medium and large EV preparations (n = 9) following comparison to the annotated bovine genome (assembly UMD3.1, release 70).

Three independent small RNA libraries for each of the small, medium and large follicles were generated from the follicular fluid EVs. The total number of reads (Mean ± SEM) was 15,144,579 ± 900,792 reads/sample and this ranged from 12,480,394 to 21,361,359 reads/sample. Of the total reads for the nine independent pools (3 independent pools / size; n = 9) 98.333% ± 0.001% mapped to the bovine genome. Mapping yielded a total of 11,770 different identified loci across all samples with the majority of these loci (8360) being located in unannotated regions (Fig. 3). Breakdown of loci falling within an annotated region (3410) indicated that 74.8% of these loci were detected within intronic regions of genes (2552) with the balance being in protein coding (238) and non-protein coding regions (620), which included miRNA (269), snoRNA (67), rRNA (128) and others (Fig. 3 shows complete breakdown of RNA subtypes). The number of read counts associated with each of these annotation types varied widely (Suppl Table 2). While only 269 bovine miRNA annotated loci were identified as present in EV RNA, the mean read count was 4384 counts/loci indicating that known miRNAs make up a large portion (12.2%) of the total number of reads (Suppl Table 2).

**Figure 4:**
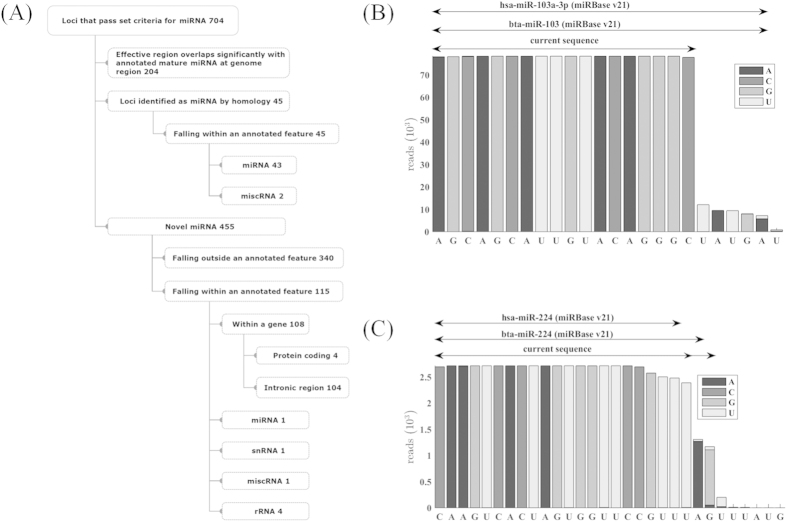
Annotation breakdown and description of miRNA within the EVs using a comprehensive and unbiased approach. (**A**) The flow chart shows the total number of loci and the classification of derivation (i.e., known, homologous to other species, and novel) that were associated with sequence loci with >100 read average within a follicle size group that met the Sanger requirements to be considered a miRNA (see detailed description in Methods). (**B**,**C**) show two representative miRNA sequences derived from EVs and compares them to the established human and bovine annotated sequences. (**B**) The current miR-103 sequence is 5 bases shorter than the previous annotated bovine and human miR-103 transcripts. (**C**) The current miR-224 sequences were of two lengths 22 and 24 bases (denoted by two overlapping arrows) this differs from the previous annotated bovine sequence which was 23 bases.

Following the general alignment to the bovine annotated sequence where we identified 269 miRNA as present within our samples, we then completed an unbiased approach to independently identify known and unknown miRNA within the bovine follicular fluid EVs. Using this unbiased approach we identified 704 loci that met our minimum read count requirements of ≥100 reads and the criteria for miRNA annotation within our EV samples (Fig. 4A). Of these 704 loci, 204 were exact matches for known bovine miRNAs (Ensemble gene annotation file for bovine; release 70). Of the remaining 500 miRNAs, 45 were mapped to a known miRNA of bovine or other species with very strong homology in both the mature and hairpin sequence. Of these 45 loci, 43 fell within a miRNA and 2 fell within a miscRNA annotated region in the bovine genome. Those 43 loci that fell within a miRNA annotated region, however, differed significantly from the previously annotated bovine miRNA. For example, the highly expressed region of the mature bta-miR-103 in the current study is 5 bases shorter than the reported bovine miRNA in miRBase v21 (Fig. 4B). Another example, miRNA-224, was also shown to differ with respect to its 3′ end (the annotated bovine sequence has 23 bases) while the human and murine miR-224 are 21 bases in length (Fig. 4C). Upon closer inspection of the murine sequence (mmu-miR-224) within miRBase, one could easily propose that the mouse sequence is 22 bases in length based on the available data. In our study we found that the EVs contained predominantly a 22 base miR-224 and to a lesser extent a 24 base version of miR-224 versus the bovine 23 base annotated sequence. Another example of a miRNA classification issue is found for a loci between bases 14462439 and 14462462 of chromosome 3 where a highly expressed mature miRNA like sequence aligns to bta-mir-9–5p which is currently annotated (miRBase v21) in two different locations within chromosomes 7 and 21, respectively. While the highly expressed mature sequence in the current study is a single base longer than the reported bta-mir-9–5p sequence, both its mature and stem-loop regions match exactly with the reported miR-9 regions in the four species *tupaia chinensis, canis familiaris, pan troglodytes* and *sus scrofa*. Lastly, the current study identified 455 novel miRNA sequences that met standard criteria as established in detail in the methods. Of these novel miRNA, 115 fell within and 340 fell outside an annotated feature (e.g., exons, etc.). Suppl Fig 4A shows a novel miRNA located in the gene coding for *ADAMTS3* whereas the other novel miRNA in Suppl Fig 4B is located outside any annotated feature. We have included the putative hairpin structures for all known bovine miRNA, miRNA previously identified in other species (i.e., homologous) and novel miRNA in Suppl Fig. 5.

### Differentially abundant miRNA within EVs of increasing sized follicles

Comparison of miRNA content within the EVs of small, medium and large follicles showed that the greatest differences were observed in the small versus large follicles (Table 1). Small (3–5 mm) follicle EVs contained 83 miRNA that were more abundant in small versus large follicle EVs while 42 miRNA were more abundant in large versus small follicle EVs (Table 1). Comparison of small to medium follicles indicated that 28 EV associated miRNA were more abundant in small and 8 more abundant in medium follicle EVs; comparison of medium and large follicles indicated that 42 were greater in medium and 17 more abundant in large follicles (Table 1). The breakdown of known bovine miRNA, homologous miRNA and novel miRNA are shown in (Table 1). The Chi-square test of homogeneity indicates that there is no significant difference in the proportion of up-regulated to down-regulated miRNA in the three comparisons (Yates’ p-value = 0.533). The relative fold changes in miRNA content across these different comparisons for individual miRNA ranged from 1.9 to 419.9.

As the greatest number of miRNA and the biggest differences were observed for the small versus large follicle EV comparison, we have depicted all 80 known differentially detected miRNA for this comparison in Table 2 and all 45 homologous and novel miRNA in Table 3. For a complete breakdown of each of known and homologous miRNA and the novel miRNA that were observed in the different size follicles (see Suppl Table 3 and 4). Of the differentially present miRNA across follicle sizes, only 8 known miRNA exhibited changes across all three classification groups. Five miRNA (miR-204, miR-92b, miR-328a-3p, miR-424–3p and miR-450a) exhibited a significant progressive increase in read counts as follicle size increased (Suppl Table 3). Two miRNA (miR-19a-3p and miR-335) exhibited a progressive decrease in read counts as follicle size increased (Suppl Table 3). Additionally, 9 novel miRNA were shown to progressively decrease in abundance from small to large follicles and none were observed to progressively increase (Suppl Table 4).

### Pathway analysis of miRNAs that differed between large and small follicle sizes

Because the greatest differences existed for the small versus large follicles, we then focused our Ingenuity Pathway Analysis (IPA) upon those miRNA. Because of the nature of IPA, only miRNA (211) which are annotated in human were used. MicroRNA that were more abundant in small and large follicle EVs were both highly linked to the associated Network functions IPA classification called Cancer, Organismal Injury and Abnormalies, Reproductive System Disease, which includes miRNA associated with reproductive tumors, endometriosis, preeclampsia, and azoospermia (Suppl Table 5). Molecular and cellular function predictions identified cellular development, cellular proliferation, cell death and survival and cell cycle as common among those miRNA that differed between these follicle sizes (Suppl Table 5). MicroRNA that are more abundant in large versus small follicles were predicted to be involved in inflammatory responses (Suppl Table 5).

## Discussion

In this study we show for the first time that EVs within follicular fluid change in number and in their small RNA content when examined across different sizes of follicles. Previous work has indicated that small non-coding RNA (i.e., miRNA) exhibit dynamic changes during ovarian follicle development^29^; we discuss several miRNA previously shown to be involved in ovarian function in the context of our new observation that they are now also known to be present in follicular fluid EVs. Our study points to the possibility that like hormones, cytokines and numerous other factors located within the follicular fluid, EVs also change dynamically over time and now must also be considered as a potential mechanism for cell-to-cell communication across the antral fluid barrier that spatially separates the cumulus oocyte complex from the mural granulosa cells. Indeed, we have recently shown that EVs from small and large follicles can elicit differential effects on cumulus-oocyte-complexes^28^.

In order to fully appreciate whether EVs undergo dynamic changes in the developing follicle, the current study took a robust approach to quantify and characterize EVs within the follicular fluid of growing bovine follicles. To confirm the proportion of viable healthy to atretic follicles within the pools, an analysis of 20 individually isolated follicles from each size group indicated that a similar proportion of viable healthy to non-viable atretic follicles (~70 and 30%, respectively). Moreover, analysis of estrogen and progesterone levels in the pools of follicular fluid indicated an increase in estrogen levels as the follicle size increased which is typical of healthy growing follicles. Nanoparticle tracking analysis (NTA) indicated that the size of EVs is consistent with that attributed to the exosomes and small microvesicles. Interestingly, we observed no change in the distribution of particle sizes originating from the different sized follicles. Importantly, it must be pointed out though that our procedure effectively eliminates larger microvesicles (>220 um). Thus, we cannot be absolute in our statement that a population of larger microvesicles does not dynamically change during follicular growth. Analysis of the EV pellets by electron microscopy further indicated that a relatively homogenous population of small vesicles (<130 nm in diameter) was isolated from the follicular fluid. Moreover, and consistent with the NTA, the size and morphology of vesicles by as evaluated by electron microscopy were also not different across the three follicle sizes. These observations might suggest that EVs are not dynamically changing during follicle development. However, the differential enrichment of CD81 within the EV preparations of the small, medium and large follicles suggests that changes within the EV pool are occurring. Conversely, the internal exosomal protein (Alix) was not found to vary in a similar pattern as that of the transmembrane protein CD81 in the EV preps suggesting that either the levels of CD81 vary on individual EVs or that different populations of EVs exist in different size follicles. Furthermore, following differential ultracentrifugation, the EV pellets were subjected to sucrose gradient centrifugation/fractionation and western blot analysis for CD81. Consistent with an exosomal origin as evident by CD81 expression^19^,^20^,^30^, we found those fractions with a density of 1.14–1.22 g/ml to be enriched in CD81 and this was also differentially detected within the three different sizes of follicles. The absence of endoplasmic reticular protein GP96 within the EV prep was further evidence of the purity of these preparations^31^. Ultimately, this detailed characterization of the EVs in the current study is reassuring that the subsequent next generation sequencing results are originating from a population of EVs within the follicular fluid. The importance of this observation is highlighted by the recent observation that serum levels of miRNA are more highly enriched in the non-vesicle fractions of serum following ultracentrifugation^32^. In previous analyses of human and bovine follicular fluid miRNA content, Sang *et al.*^25^ and Sohel *et al.*^27^ both detected miRNA within the pelleted extracellular vesicle component and in the remaining supernatant. In both cases the numbers of miRNA identified were greater in vesicular fraction and in the case of the bovine study, Sohel *et al.*^27^, suggested that the vesicle-mediated pathway may be the predominant mechanism of miRNA transport with the follicle. This clearly differs from observations in other bodily fluids (i.e., serum, urine, salvia) where it appears that non-vesicular miRNA maybe the predominant form^32^. Our current study did not address this issue and additional studies will be required to determine the relative physiologic importance of these two different sources of miRNA within the follicular fluid.

Our study also shows for the first time that the tetraspanin CD81 exhibits differences in levels within the EV preparations of small, medium and large follicles. This protein belongs to a large family of tetraspanin proteins that are found in numerous cell types^33^ and are enriched in exosomes^19^,^20^. Previous studies demonstrated that CD81 was highly expressed by murine cumulus cells which surround oocytes^34^ and by periovulatory bovine mural granulosa cells^35^ before and after the LH surge. Immunohistochemistry indicated that the zona pellucida surrounding mouse oocytes was highly enriched in CD81^36^. A role for CD81 in murine ovarian function was demonstrated when targeted deletion of the gene caused female mice to exhibit a 40% reduction in fertility^37^. Further studies showed that this loss in fertility was due to the inability of oocyte to fuse with sperm^37^. Interestingly, the source of the zona pellucida CD81 does not appear to be of oocyte origin as CD81 was not detected in the oocyte and furthermore oocytes microinjected with exogenous CD81 mRNA failed to produce CD81 or reverse the sperm fusion defect^36^. In our study, the highest level of CD81 was found in EVs derived from 3–5 mm diameter follicles while the level of CD81 decreased in EVs from 6–9 mm and >9 mm follicles. The biological meaning of this change in CD81 within the EVs isolated from the different size follicles as well as the source of the CD81, i.e., whether it is of serum or granulosa/cumulus derivation requires further investigation.

In our evaluation of RNA within the EVs, the preponderance of small RNA species ranged in size from 20 to 40 nucleotides in length, consistent with these small RNA species being of a size consistent with miRNA. Our sequencing results indicated that 12.2% of small RNAs were of miRNA origin which is consistent with that observed (15.4%) in the only other small RNAseq results for follicular fluid^25^. In addition to the overall abundance of miRNA (43.3% of the total of non-protein coding loci), several other small RNA species were identified within EVs including snRNA (8.3%), snoRNA (10.8%), rRNA (20.6%), miscRNA (9.2%). The presence of miRNA within EVs was previously documented in equine^21^, bovine^27^, and human^23^,^24^,^25^ follicular fluid. In the bovine and equine studies, EVs were isolated using the ExoQuick precipitation method followed by qPCR arrays to identify miRNA. Recently it was shown that the Exoquick precipitation method generates a greater range of vesicle sizes and increased protein contamination when compared to the standard differential ultracentrifugation method used here^23^. Additionally, these studies evaluated miRNA abundance using qPCR arrays designed for detection of human miRNA, which limits their observations to those miRNA with 100% homology to the human sequence. It should be noted though that many miRNA are 100% homologus with respect to the mature miRNA sequence across a large number of species^38^. However, consistent with our observation of a large number of miRNA within the bovine follicular fluid, Sohel *et al.*^27^, observed 509 different miRNA in bovine follicular fluid isolated from 4–8 mm follicles. Using our unbiased small RNAseq approach, we detected the presence of 249 previously known miRNA (bovine or other species annotated) and the presence of 455 previously unknown miRNA in bovine EVs. The large number (455) of novel miRNA found within bovine follicular fluid could indicate that this bodily fluid is exceptionally enriched in miRNA or could be a reflection of the incomplete annotation of miRNA species within the bovine. Currently, the bovine has 682 unique annotated miRNA and of these 36.5% were identified within our follicular fluid EVs. In addition to large numbers of novel miRNA, a number of miRNA (45) identified in the bovine EVs, not yet annotated in the bovine genome, were consistent with previously identified miRNA in other species. Recently, Maalouf *et al.*^39^, in a small RNAseq analysis of bovine corpora lutea, identified 590 miRNA of which 46 were novel miRNA. This large difference in numbers of novel miRNA between our study and theirs is likely due to their restriction of identification of novel miRNAs to only regions within introns or antisense exon regions, a criteria we chose not to employ in our current study. Additionally, several novel miRNA structures identified in the current study were found replicated identically or nearly identically in both stem-loop and mature sequence in different locations across the genome; we considered each as a different putative novel miRNA. Recent analysis of miRNA sequences in 13 different human cell lines indicated the presence of a substantial number (3707) of previously unidentified microRNA^40^. Follicular fluid EVs appear to be a rich source of miRNA.

This study is the first to examine the differential abundance of miRNA within EVs across different stages of follicular growth. Previous studies have evaluated follicular fluid derived EVs isolated from follicles of young and old mares^21^, women with and without polycystic ovarian syndrome^25^, IVF patients^23^, and in young (age 31) versus old (age 38) IVF patients. In the previous bovine experiment, follicles within a single range of size 4–8 mm were classified based on oocyte brilliant cresyl blue staining which can define the oocyte as growing or fully grown^27^. Unfortunately, comparisons across these two studies are not possible based on the divergent experimental designs, methods of exosome isolation and miRNA identification methods. Not surprisingly, comparison of the miRNAs within the EVs of large (>9 mm) versus small (3–5 mm) follicles showed the greatest difference in number of differentially present miRNA, with 42 increased and 83 decreased. Changes in miRNA from small to medium and medium to large follicle EVs were intermediate as might also be expected. Five miRNAs exhibited significantly progressive increases in read counts as follicle size increased including miR-204, miR-92b, and miR-328a-3p, miR-424-3p and miR-450a. Mir-204 has been addressed as a cell proliferation inhibitor in several cell types^41^,^42^,^43^,^44^,^45^,^46^,^47^, this could be possibly linked to the decrease in cell proliferation that occurs with increasing follicle size. MiR-92b was more abundant in large follicle than small follicle EVs. Significant changes in miR-92b expression levels have also been noted in the process of neointimal formation in a rat model of vascular injury^48^, in individuals with a cardiac rehabilitation following surgical coronary revascularization^49^ and in individuals with heart failure^50^, indicating that miR-92 is also involved in neovascularization. However, the function of miR-92b in reproduction is still unknown. DNA methyltransferase 3a (Dnmt3a) is known as a target of miR-450a and ectopic miR-450a expression in HepG2 cells decreased Dnmt3a and blocked inhibition of cell proliferation^51^. The roles of miR-328a-3p and miR-424-3p are currently poorly described. Conversely, levels of miR-19a-3p and miR-335 exhibited significant progressive decreases as follicle size increased. Mir-19a-3p has been reported to inhibit breast cancer progression^52^. Deletion of miR-335 is a common event in human breast cancer and also correlates with ovarian cancer recurrence^53^. Others showed that the level of miR-335 is highly elevated in astrocytoma cells and human malignant astrocytoma suggesting that miR-335 might be a tumor promoter by promoting tumorigenic features such as growth and invasion of malignant astrocytoma^54^. Our bioinformatics analyses of pathways also pointed to the changes in vascularization, as evident by the significant association of the different miRNA observed in the small versus large follicle EVs and the inflammatory pathway. Interestingly, miR-150 which exhibited the most profound difference in small versus large follicles (Table 2) was previously reported as an oncomiR because of its promotional effect on vascular endothelial growth factor (VEGF)^55^. During tumor development, tumor-associated macrophages (TAMs) secrete VEGF and other factors to promote angiogenesis. MiR-150 targets TAMs to up-regulate their secretion of VEGF *in vitro*. Angiogenesis is known to increase dramatically during development of follicle with a pronounced increase following selection of the dominant follicle^56^. Human follicular fluid contains elevated VEGF production following induction by the surge of LH in *in vitro* fertilization patients^57^. Thus, miR-150, which was more abundant in the large follicle, might also be involved in the vascular development, follicle growth, and the ovulation of an oocyte through its regulation of VEGF.

The importance of miRNA in the ovary has been illustrated in clinical studies and studies where key elements of the miRNA biogenetic pathway or where specific miRNA have been genetically deleted. Loss of Dicer, the key enzyme of miRNA biogenesis, results in decreased fertility and ovulation rate^58^,^59^,^60^. The change of follicular extracellular associated miRNA was also demonstrated in polycystic ovarian syndrome^25^,^26^. MiR-224 which was discussed above is known to be expressed higher in preantral follicle and is able to regulate estradiol production^61^. MiR-378 which decreased 2.4 fold from small to large antral follicle was also reported to decrease during antral follicle growth^67^ and has the ability to regulate Cyp19A1^68^, critical gene for estradiol production.

Taken together, the results of the present study demonstrate for the first time that EVs differ in number, presence of protein markers and miRNA contents as follicle size changes. Specifically, CD81, one of three major marker proteins used for exosome identification, exhibited decreased abundance as follicle size increased suggesting that changes in EV biogenesis or uptake is occurring during follicle maturation/development. Functional analyses of individual miRNA that are associated with changes in follicle development also await further study, yet the current study provides clear and robust data regarding which miRNA might be first to be evaluated. This in combination with the recent observations that follicular fluid EVs are biologically functional (induce cumulus expansion and gene expression) and differ in their functional activity based on the size of follicle they were derived from is consistent with our current observations. Cumulatively, the observations point to the potential for a unique EV signaling mechanism existing within the ovarian antral follicle.

## Materials and Methods

### Follicular fluid collection

Bovine ovaries were from a local abattoir: because the animals were not used specifically for research purposes our Institutional Animal Care and Use Committee ruled that the collection of ovaries from the abattoir does not constitute animal research and is exempt from further review. Ovaries (~150 ovaries / collection) were placed immediately into PBS and then transported 2.5 h to the University of Kansas Medical Center at 20–24 C temperature in preparation for ovarian follicular fluid aspiration. Follicular diameters were determined by individually measuring follicle diameters; follicles were designated into three different groups based on diameter (3–5 mm - small, 6–9 mm–medium and > 9 mm–large). Follicular fluid was aspirated using a tuberculin syringe (28 gauge needle) for small follicles and a 5 ml syringe with a 20-gauge needle for medium and large follicles. The volume aspirated was used to confirm classification status (small < 50 μl, medium ~100 μl to 350 μl and large > 400 μl). Three independent collections of follicular fluid were conducted over a two-month period for the RNAseq analysis. Each collection provided the approximate volumes of follicular fluid: 20 ml from small, 15 ml from medium and 40 ml from large follicles. To determine relative follicle quality within the small, medium and large pools, 18–20 single follicles at each size were dissected free from the ovaries of a fourth collection of ovaries. The granulosa cells were then isolated by bisecting each follicle and scraping the cells into PBS. Cells were then fixed in 4% PFA, and stained with the DNA dye, Hoechst 33342 (Life Technologies, NY). The nuclei of a hundred granulosa cells from each follicle were then classified as healthy (i.e., intact nucleus) or as apoptotic/atretic (i.e., fragmentized nucleus). Additionally, RIA conducted at the University of Virginia Center for Research in Reproduction Ligand Assay and Analysis Core determined estradiol and progesterone levels for each of the pools. Each follicular fluid sample was diluted 1:15 fold in PBS for estradiol and 1:20 for progesterone prior to RIA. The sensitivity of estradiol assay is 10 pg/ml with intra-assay CV of 6.3% and inter-assay CV of 8.1%. The sensitivity of progesterone assay is 0.15 ng/ml with intra-assay CV of 5.7% and inter-assay CV of 7.1%.

### Isolation of extracellular vesicles

Extracellular vesicles were isolated using a differential ultracentrifugation method developed for serum isolation of exosomes^62^. Follicular fluid (15 ml) was diluted 1:1 with PBS and then spun at 800 g and 2000 g for 10 and 20 min each in an Optima^TM^ L-100XP to pellet all granulosa cells and the oocytes (the cell pellets were snap frozen at −80 C or placed in Trizol). The follicular fluid was then centrifuged at 12000 g for 45 min to remove cellular debris and other large particles (large microvesicles and apoptotic blebs). Samples were then filtered through a 0.22 μm pore filter to further remove vesicles bigger than 220 nm. Ultracentrifugation of the 1:1 mix follicular fluid:PBS was performed at 110000 g for 3 h in an Optima^TM^ L-100XP ultracentrifuge, using a swinging bucket SW32Ti rotor. The resulting EV pellets were then resuspended in 4 ml PBS and spun again for 1 h at 110000 g in a TLA 100.4 fixed angle rotor. A final wash with 1 ml of PBS was then performed and spun at 110000 g for 1 h using a TLA 55 fixed angle rotor. All centrifugations were performed at 4 °C. The obtained pellets were re-suspended in PBS for further analysis.

### Separation of extracellular vesicles on sucrose gradient

Extracellular vesicles isolated from follicular fluid using differential centrifugation were re-suspended in 100 μl of PBS and overlaid on the top of a continuous sucrose gradient (0.2 to 2.5 M sucrose in 20 mM HEPES, pH 7.4) which was made using a Hoefer SG30 gradient marker (GE Healthcare, Piscataway, NJ). The gradient tubes were centrifuged at 210,000 g for 16 h at 4 °C in an SW41Ti swinging bucket rotor with an Optima-Max ultracentrifuge (Beckman Coulter). Eleven 1 ml fractions were collected from the top to bottom of the gradient and the refractive index of each collected fraction was measured using a VEE GEE refractometer (VEE GEE Scientific, Washington, USA). The sucrose density was converted from refractive index to density (g/cm3). Each fraction was washed in 3 ml of PBS by ultracentrifugation at 110,000 g for 1.5 h at 4 C using a TLA-100 rotor in an Optima-Max ultracentrifuge and the pellets were re-suspended in PBS and loaded onto 12% SDS-PAGE for Western blot analysis.

### Western blot Analysis

EV samples (10 μg protein) were lysed in SDS sample buffer with 50 mM DTT, heated for 5 min at 95 °C and subjected to electrophoresis using 12% SDS-PAGE in running buffer at constant 120 V for 1.5 hrs. Proteins were then electrotransferred onto polyvinylidenedifluoride membranes, and the membranes were blocked with 5% (w/v) skim milk powder in Tris-buffered saline with 0.05% (v/v) Tween-20 (TTBS) for 1 h at RT. Membranes were then probed with primary anti-CD81 (sc-166029), anti-Alix (sc49267), anti-GP96 (sc-11402) and anti-Actin (sc-1616) antibodies for 1 h in TTBS (50 mM Tris, 150 mM NaCl, 0.05% Tween20) followed by incubation with the secondary anti-mouse IgG or anti-sheep goat antibodies for 1 h. All primary antibodies were purchased from Santa Cruz Biotechnology Inc. Membranes were washed three times in TTBS for 10 min after each incubation step and detected by enhanced chemiluminescence (ECL) (GE Healthcare Bio-science, PA) per manufacturer’s instructions.

### Nanoparticle tracking analysis

To determine particle size and concentration, nanoparticle-tracking analysis (NTA) was performed with a Nanosight LM10 instrument (Nanosight, Salisbury, UK) outfitted with a LM14C laser. Each EV preparation was sampled three times to generate 3 independent dilutions. These dilutions range from ~1:1000 to 1:10,000 for each sample (the dilution is determined empirically for each sample) and then each of the three dilutions is analyzed 5 times (5–60 video tracks/dilution). The overall average of these three dilutions was used as the experimental result for each sample. If large discrepancies in counts were observed for a set of dilutions or if the range of particle concentrations were outside the optimum range, another dilution was then created from the stock and analyzed. From validation studies using dilution series, we determined that NTA is most accurate between particle concentrations in the range of 2 × 10^8^/ml to 2 × 10^9^/ml. Thus samples were diluted to this level and absolute concentrations were then back calculated according to the dilution factor.

### Transmission electron microscopy

An aliquot of the EV sample was pelleted and the PBS was replaced with 2% gluteraldehyde fixative in 0.1 M cacodylate buffer. Following overnight fixation, the fixative was carefully removed and replaced with 0.1 M sodium cacodylate buffer 2 times for 5 min each. The pellet was then post-fixed in a solution of 1% osmium tetroxide plus with 1% potassium ferric cyanide buffered in 0.1 M cacodylate buffer for 1 h. Osmicated pellets were dehydrated through a series of graded ethanols, rinsed in propylene oxide 2 times for 10 minutes each and then embedded in fresh EMbed 812 resin and cured in a 60 °C oven overnight. Sections were cut at 80 nm using a Leica UC-7 ultramicrotome, mounted on copper thin bar 300 mesh grids, and contrasted with 4% uranyl acetate and Sato’s lead stain. All samples are examined in a JEOL-JEM-1400 transmission electron microscope at an 80 kV with 25,000x magnification.

### RNA preparation

Total RNA was isolated from EVs (20 μl) with Trizol (Invitrogen) according to the manufacturer’s protocol. Briefly, EVs were disrupted and homogenized in 200 μl of Trizol reagent and incubated for 5 min at room temperature. Samples were then spun in a microcentrifuge at 12000 g for 15 min at 4 °C following addition of 40 μl of chloroform. Subsequently, the aqueous solution was mixed with 100 μl of isopropanol. In order to extract small RNA, 1 μl glycogen (20 μg/μl) was added together with RNA-isopropanol mixture solution and incubated at −80 °C for 30 min and spun at 12000 g for 15 min at 4 °C. The pellet was washed with 100 μl of 70% ethanol followed by centrifugation at 7500 g for 5 min at 4 °C. The RNA pellet was dried at room temperature and dissolved in 10 μl of RNase free water. The RNA solution was then stored at −80 °C. RNA quality, yield, and sizes were assessed with the Agilent 2100 Bioanalyzer (Agilent Technologies, Foster City, CA) using the Agilent Small RNA chip. To confirm the absence of 18S and 28S ribosomal RNA we also ran the RNA of the Agilent RNA 6000 Nano chips. Briefly, 1 μl of RNA was analyzed on either the Agilent RNA 6000 Nano or Small RNA chips according to manufacturer’s protocol. Electropherograms were analyzed using the Agilent 2100 Expert B.02.07 software that includes data collection, presentation and interpretation functions. The EV RNA purity was also evaluated using Nanodrop spectrophotometer ND-1000 (Thermo Scientific, Wilmington, DE) at the absorbance 230, 260 and 280 nm. The average A260/230 and A260/280 ratios were used to assess the presence of peptides, phenols, aromatic compounds, or carbohydrates and proteins.

### Next generation sequencing

Small RNA sequencing was performed using the Illumina HiSeq2500 Sequencing System at the University of Kansas Medical Center–Genomics Core (Kansas City, KS). Extracellular vesicle RNA (ranging from 1.8 ng–100 ng) was used to initiate the TruSeq Small RNA library preparation protocol (Illumina #RS200-0012 kit A). The EV RNA was ligated with 3′ and 5′ RNA adapters followed by a modified reverse transcription reaction and modified PCR amplification. Due to low starting RNA quantities, the reverse transcription of the RNA adapter ligated samples was modified by performing two duplicate reactions containing 6 μl of the 3′/5′ RNA ligated RNA. The 12.5 μl yield of each duplicate reverse transcription reaction was then pooled to obtain 25 μl of homogeneous cDNA. The subsequent PCR amplification, with index adapter incorporation, was modified by replacing 8.5 μl of ultra-pure water in the PCR master mix with 8.5 μl of the reverse transcribed and pooled cDNA (21 μl cDNA total). The modified PCR reaction was performed with 15 cycles of amplification. Size selection and purification of the cDNA library construct was conducted using 3% marker H gel cassettes on the Pippin Prep size fractionation system (Sage Science). The Agilent 2100 Bioanalyzer was used with the High Sensitivity DNA kit (Agilent #5067-4626) or the DNA1000 kit (Agilent #5067-1504) to validate the purified libraries. Libraries were quantified on the Illumina ECO Real Time PCR System using KAPA SYBR Universal Library Quant kit–Illumina (KAPA Biosystems KK4824). Following quantification, libraries were adjusted to a 2 nM concentration and pooled for multiplexed sequencing. Libraries are denatured and diluted to the appropriate pM concentration (based on qPCR results) followed by clonal clustering onto the sequencing flow cell using the TruSeq Rapid Single Read (SR) Cluster Kit-HS (Illumina GD402–4001). The clonal clustering procedure was performed using the automated Illumina cBOT Cluster Station. The clustered flow cell was sequenced on the Illumina HiSeq 2500 Sequencing System in Rapid Read mode with a 1×50 cycle read and index read using the TruSeq Rapid SBS kit-HS (Illumina FC402-4002). Following collection, sequence data was converted from .bcl file format to FASTQ files and de-multiplexed into individual sequences for further downstream analysis. FastQC analysis (www.bioinformatics.babraham.ac.uk/projects/fastqc/) was completed to ensure quality of small RNAseq data.

### Bioinformatics

The three groups of EVs (small, medium and large) were analyzed in biological triplicates giving nine samples in total. After 3′ adapter removal, the read sequences were mapped using the bwa software (v 0.7.5a^63^), to the bovine genome (assembly UMD3.1) and annotated for overlapped regions with the Ensemble gene annotation file for bovine (release 70) and miRBase (release 21).

Mapped reads from all 9 samples were merged and loci with an average read count per base (sum of the number of reads mapped at each base of the locus divided by its length) less than 45 were filtered out. These loci were then scanned for high-density regions defined as a contiguous region whose read count at each base is not less than 20% of the highest base read count for the locus. These high-density regions formed the effective region of the locus and its length defined as its effective length. The number of reads mapped to the effective region of each sample formed the effective read counts that were used for down-stream analysis. To further limit the number of loci identified to a more conservative “biologically relevant” number; loci with an average read count (over the three replicate samples) of 100 or greater in at least one of the three conditions (small, medium or large) were included for further analysis.

Known and novel miRNA’s were identified from these loci through a systematic filtering process beginning with a length filter that filtered loci to those with an effective length between 18 and 30 bases. Effective regions that mapped to mature miRNA were first identified, while the remaining effective regions were compared to known miRNA from both bovine and other species found in miRBase (release 21). A region was labeled as a miRNA by homology if it passed the following criteria; a gapless alignment of the effective region to the mature reference miRNA with at most 2 mismatches in the core and at most 1 gap/mismatch at the 5 and 3 prime ends and less than 10% mismatches in the alignment of the reference hair-pin sequence to the locus. Novel miRNAs were identified based on the criteria that the extended effective region should have a predicted pre-miRNA like hairpin structure^64^ with the effective region falling in the stem region with at least 80% pairing. The hybridization free energy cutoff was set at −15 kcal/mol. The raw sequence data available as GEO (GSE74879) has been uploaded to miRBase for annonation, a sortable Excel file format depicting all of the identified loci is available upon request.

Exact statistical methods developed for multi-group experiments available from the edgeR software package^65^ were used to determine significantly differently expressed miRNAs between the different conditions. The edgeR package employs advance empirical Bayes methods to estimate miRNA-specific biological variation under minimal levels of biological replication. The associated p-values were corrected for multiple-hypothesis testing by the Benjamini and Hochberg method^66^.

### MiRNA function prediction

Differentially expressed miRNAs (p < 0.05 and false discovery rate, FDR < 0.1) between two different sizes of follicle groups (large versus small, medium versus small and large versus medium) were used. Selected miRNAs were used to predict potential function through the use of QIAGEN’s Ingenuity^®^ Pathway Analysis (IPA^®^, QIAGEN Redwood City, www.qiagen.com/ingenuity).

### Statistical Analysis

All of the quantitative experiments (concentration of particles, steroid hormones concentrations, ratio of miRNA in total small RNA) were repeated with at least three independent biological replicates. These results were analyzed by one-way ANOVA and Newman-Kuels multiple comparison tests were performed to determine differences amongst the means. The GraphPad Prism version 5.00 for Windows, GraphPad Software, San Diego California USA was used for these analyses. A p < 0.05 was considered statistically significant. The Chi-square test of homogeneity was used to determine if differences in proportions of miRNA significantly up and down regulated differed across the three comparisons of follicular fluid pools. The statistical analysis details for the next generation sequencing results are explained in detail in that section.

## Additional Information

**How to cite this article**: Navakanitworakul, R. *et al.* Characterization and Small RNA Content of Extracellular Vesicles in Follicular Fluid of Developing Bovine Antral Follicles. *Sci. Rep.* **6**, 25486; doi: 10.1038/srep25486 (2016).

## Supplementary Material

Supplementary Table 3

Supplementary Table 4

Supplementary Table and Figures

## Figures and Tables

**Table 1:** Distribution of miRNA that differed between small, medium, and large follicles*.

Comparison		S vs L		S vs M		M vs L	
Increased in		Small	Large	Small	Medium	Medium	Large
miR annotation	Bovine	47	33	5	7	24	15
	Other species (homologous)	5	8	2	1	3	2
	Novel	31	1	21	0	15	0
	All	83	42	28	8	42	17

*The Chi-square test of homogeneity indicates there is no significant difference in the proportion of up-regulated to down-regulated miRNA in the three comparisons (Yates’ p-value = 0.533).

**Table 2:** Normalized read count and fold change of annotated bovine miRNA which differed between small and large follicles*.

Mir name	Normalized read count (Average)		Large vs small	Mir name	Normalized read count (Average)		Large vs small	Mir name	Normalized read count (Average)		Large vs small
	Small	Large	Fold change		Small	Large	Fold change		Small	Large	Fold change
bta-miR-150	**8**	**416**	51.0	bta-miR-542-5p	**42**	**98**	2.3	bta-miR-146b	**946**	**302**	−3.2
bta-miR-132	**51**	**878**	17.1	bta-miR-425-5p	**699**	**1479**	2.1	bta-miR-210	**2438**	**714**	−3.4
bta-miR-92b	**1932**	**24175**	12.5	bta-miR-30b-5p	**735**	**1553**	2.1	bta-miR-20a	**416**	**121**	−3.5
bta-miR-204	**410**	**4117**	10.1	bta-miR-151-5p	**3218**	**6717**	2.1	bta-miR-2284z	**84**	**23**	−3.6
bta-miR-486	**949**	**9245**	9.7	bta-miR-342	**570**	**1124**	2.0	bta-miR-215	**534**	**143**	−3.7
bta-miR-328	**41**	**360**	8.6	bta-miR-450b	**2946**	**5497**	1.9	bta-miR-21-3p	**527**	**133**	−3.9
bta-miR-424-3p	**120**	**838**	7.0	bta-miR-10b	**108030**	**56151**	−1.9	bta-miR-101	**2356**	**611**	−3.9
bta-miR-1343-3p	**60**	**398**	6.6	bta-miR-532	**1867**	**885**	−2.1	bta-miR-1839	**3281**	**818**	−4.0
bta-miR-30f	**155**	**1015**	6.6	bta-miR-27a-3p	**1072**	**504**	−2.1	bta-miR-29a	**6079**	**1506**	−4.0
bta-miR-450a-1	**68**	**444**	6.5	bta-miR-15a	**1534**	**686**	−2.2	bta-miR-1388-5p	**171**	**41**	−4.1
bta-miR-450a-2	**71**	**425**	6.0	bta-miR-103	**8534**	**3785**	−2.3	bta-miR-106b	**752**	**180**	−4.2
bta-miR-222	**140**	**719**	5.2	bta-miR-378	**4819**	**2115**	−2.3	bta-miR-17-3p	**107**	**27**	−4.3
bta-miR-1307	**81**	**389**	4.8	bta-miR-660	**8723**	**3729**	−2.3	bta-miR-2284w	**62**	**12**	−4.5
bta-miR-142-5p	**215**	**1018**	4.8	bta-miR-143	**15992**	**6642**	−2.4	bta-miR-378c	**100**	**20**	−4.7
bta-miR-125b	**335**	**1398**	4.2	bta-miR-152	**972**	**378**	−2.6	bta-miR-192	**6690**	**1393**	−4.8
bta-miR-451	**94**	**378**	4.1	bta-miR-199a-3p	**686**	**252**	−2.7	bta-miR-130a	**6823**	**1411**	−4.8
bta-miR-339a	**517**	**1873**	3.6	bta-miR-17-5p	**1317**	**484**	−2.7	bta-miR-429	**131**	**24**	−5.1
bta-miR-125a	**4165**	**14287**	3.4	bta-miR-23a	**2591**	**949**	−2.7	bta-miR-1271	**56**	**14**	−5.4
bta-miR-2904	**50**	**173**	3.4	bta-miR-873	**1319**	**482**	−2.7	bta-miR-29c	**319**	**54**	−5.8
bta-miR-1468	**416**	**1364**	3.3	bta-miR-452	**150**	**54**	−2.8	bta-miR-19b	**1271**	**212**	−6.1
bta-miR-331-3p	**64**	**210**	3.3	bta-miR-148a	**48363**	**17154**	−2.8	bta-miR-190b	**136**	**18**	−6.2
bta-miR-30d	**12377**	**39580**	3.2	bta-miR-2285k	**68**	**21**	−3.1	bta-miR-193a-3p	**51**	**5**	−7.9
bta-miR-744	**268**	**796**	3.0	bta-miR-18a	**65**	**19**	−3.1	bta-miR-449a	**126**	**17**	−8.7
bta-miR-484	**285**	**828**	2.9	bta-miR-592	**233**	**72**	−3.1	bta-miR-19a	**764**	**52**	−14.6
bta-miR-30c	**2993**	**8015**	2.7	bta-miR-107	**8028**	**2543**	−3.1	bta-miR-335	**4689**	**231**	−20.3
bta-149-5p	**68**	**176**	2.6	bta-miR-130b	**246**	**75**	−3.2	bta-miR-1388-3p	**39**	**0**	−419.9
bta-mIR-182	**287**	**755**	2.6	bta-miR-32	**464**	**147**	−3.2				

*All of the following genes were adjusted for multiple tests by the Benjamini and Hochberg method and had false discovery rates <0.05. Additional details including raw counts, exact FDR values, as well as comparisons to medium sized follicles are present in Supplemental Table 3.

**Table 3:** Normalized read count and fold change of novel and homologous bovine miRNA which differed between small and large follicles*.

Novel miRNA Locus	Normalized read count (Average)		Large vs small
Locus ID (Chromosome:start-end; Strand)	Small	Large	Fold change
22:37936196–37936222 (−)	**600**	**892**	1.5
9:72616256–72616283 (+)	**821**	**453**	−1.8
14:4412105–4412134 (+)	**2352**	**1134**	−2.1
4:46639567–46639595 (−)	**1853**	**802**	−2.3
12:89782137–89782164 (+)	**1115**	**428**	−2.6
X:62079641–62079660 (−)	**4088**	**823**	−5.0
X:62078661–62078681 (−)	**4286**	**779**	−5.5
X:144087437–144087455 (+)	**1817**	**320**	−5.7
X:62080685–62080704 (−)	**2556**	**442**	−5.8
X:62081821–62081840 (−)	**2820**	**444**	−6.3
4:113198367–113198384 (−)	**1558**	**209**	−7.4
18:58243447–58243467 (+)	**1837**	**230**	−8.0
13:13589076–13589092 (−)	**5226**	**621**	−8.4
3:63639301–63639326 (−)	**8378**	**696**	−12.0
7:44900586–44900601 (−)	**2416**	**197**	−12.2
11:72567808–72567823 (+)	**2415**	**163**	−14.7
9:103492721–103492748 (−)	**2209**	**118**	−18.7
10:26814520–26814545 (+)	**16477**	**700**	−23.5
16:71150568–71150591 (+)	**3630**	**133**	−27.3
8:89663122–89663138 (−)	**8049**	**238**	−33.8
X:2676228–2676250 (+)	**3251**	**91**	−35.3
21:60864954–60864973 (+)	**152167**	**3229**	−47.1
28:25631928–25631956 (+)	**2787**	**57**	−48.0
20:55442871–55442887 (−)	**3718**	**69**	−53.2
7:20756731–20756752 (+)	**1908**	**32**	−58.3
X:29130246–29130269 (+)	**1394**	**22**	−62.1
9:94314513–94314529 (−)	**15284**	**232**	−65.9
27:27255989–27256011 (+)	**2563**	**37**	−69.2
8:111841867–111841889 (−)	**2674**	**36**	−71.4
7:64776449–64776465 (+)	**1825**	**23**	−77.7
18:22622903–22622918 (+)	**67609**	**671**	−100.6
X:30338371–30338390 (−)	**14428**	**116**	−123.8

**Homologous miRNA**	**Normalized read count (Average)**		**Large vs small**
**MiRNA name (bovine chromosome:start-end: Strand)**	**Small**	**Large**	**Fold change**
efu-miR-181f	**181**	**826**	4.57
11:95709487-95709508			
chi-let-7b-3p	**41**	**178**	4.24
5:117120245-117120266			
chi-let-7d-3p	**545**	**2010**	3.69
8:86887492-86887513			
chi-miR-28-3p	**2839**	**10157**	3.58
1:79250541-79250563			
ssc-miR-1285	**72**	**231**	3.18
10:42863853-42863882			
chi-miR-361-3p	**167**	**509**	3.05
X:74328465-74328489			
chi-miR-195-3p	**193**	**509**	2.64
19:27441353-27441377			
chi-miR-1307-5p	**1281**	**522**	−2.44
26:24230159-24230179			
eca-miR-199b-3p	**693**	**211**	−3.24
11:98867681-98867702			
chi-miR-335-3p	**530**	**154**	−3.38
4:95071042-95071063			
chi-miR-145-3p	**145**	**36**	−3.69
7:62810788-62810808			
mml-miR-452-3p	**113**	**25**	−4.13
X:34665635-34665656			
chi-miR-708-3p 29:16686296-16686318	**52**	**7**	−5.91

*All of the following genes were adjusted for multiple tests by the Benjamini and Hochberg method and had false discovery rates <0.05. Additional details including raw counts, exact FDR values, as well as comparisons to medium sized follicles are present in Supplemental Table 4.
