# Supplementary material for: Characterization and Small RNA Content of Extracellular Vesicles in Follicular Fluid of Developing Bovine Antral Follicles
Source: Sci Rep. 2016 May 9;6:25486. doi: 10.1038/srep25486 (PMC4860563; doi:10.1038/srep25486)

Characterization and Small RNA Content of Extracellular Vesicles in Follicular Fluid of Developing Bovine Antral Follicles.

Raphatphorn Navakanitworakul, Wei-Ting Hung, Sumedha Gunewardena, John S. Davis, Wilaiwan Chotigeat, and Lane K. Christenson

Supplement table 1 The mode and mean size of EVs from different sized follicles were determined by NTA.

	follicle size (mm)		
	3-5	6-9	>9
Mode (nm)	109	113	106
Mean (nm)	124	134	127
Standard deviation (nm)	43	47	42

Supplement table 2. The number of read counts associated with each of annotation types

Location	Count	Cumulative	Median	Mean	Std. Err.	Max	Min
Total number of detected loci	11770	9,629,592	143	818	63	597,958	37
Loci outside an annotated region	8360	5,207,955	144	623	73	597,958	37
Loci overlapping an annotated region	3410	4,421,637	140	1,297	125	126,917	38
Within a gene	2790	1,332,190	134	477	32	43,014	39
Protein coding	238	40,945	90	172	43	10,255	39
Intronic region	2552	1,291,245	136	506	34	43,014	41
Non-protein coding	620	3,089,447	279	4,983	654	126,917	38
miRNA	269	1,179,225	424	4,384	744	107,513	38
Pseudogene	6	851	53	142	84	561	42
snRNA	52	144,802	113	2,785	902	43,559	47
miscRNA	57	922,140	198	16,178	4,713	126,917	47
Processed pseudogene	1	86	86	86	0	86	86
snoRNA	67	15,568	163	232	34	2,255	59
rRNA	128	812,857	824	6,350	1,585	79,839	63
Mt-rRNA	30	10,579	193	353	142	4,420	72
Mt-tRNA	10	3,340	212	334	101	1,114	64

Supplement table 3 and 4: See uploaded data set

Supplement Table 5. Ingenuity Pathway Analysis predicted functional networks for miRNA enriched in EVs isolated from small or large follicles.*

More in small follicles

Molecular and cellular function

Name	p-value	# Molecules
Cellular Development	3.52E-10 - 4.93E-02	24
Cellular Growth and Proliferation	3.52E-10 - 4.93E-02	24
Cellular Movement	3.39E-08 - 4.82E-02	18
Cell Death and Survival	7.36E-06 - 4.55E-02	20
Cell Cycle	2.49E-05 - 4.82E-02	10

Top networks

ID	Associated Network Functions	Score
1	Cancer, Organismal Injury and Abnormalities, Reproductive System Disease	73
2	Cancer, Organismal Injury and Abnormalities, Reproductive System Disease	32
3	Hereditary Disorder, Skeletal and Muscular Disorders, Developmental Disorder	23
4	Cancer, Gastrointestinal Disease, Inflammatory Response	16
5	Hereditary Disorder, Skeletal and Muscular Disorders, Cancer	8

More in large follicles

Molecular and cellular function

Name	p-value	# Molecules
Cellular Development	1.81E-07 - 4.21E-02	11
Cell-To-Cell Signaling and Interaction	2.69E-03 - 2.69E-03	2
Cell Cycle	4.03E-03 - 3.83E-02	3
Cellular Growth and Proliferation	4.03E-03 - 4.21E-02	8
Cell Death and Survival	5.36E-03 - 4.25E-02	7

Top networks

ID	Associated Network Functions	Score
1	Cancer, Inflammatory Disease, Inflammatory Response	32
2	Organismal Injury and Abnormalities, Reproductive System Disease, Cancer	26
3	Endocrine System Disorders, Organismal Injury and Abnormalities, Reproductive System Disease	3
4	Cancer, Hematological Disease, Immunological Disease	3

*Predicted molecular and cellular functions and top networks are depicted.

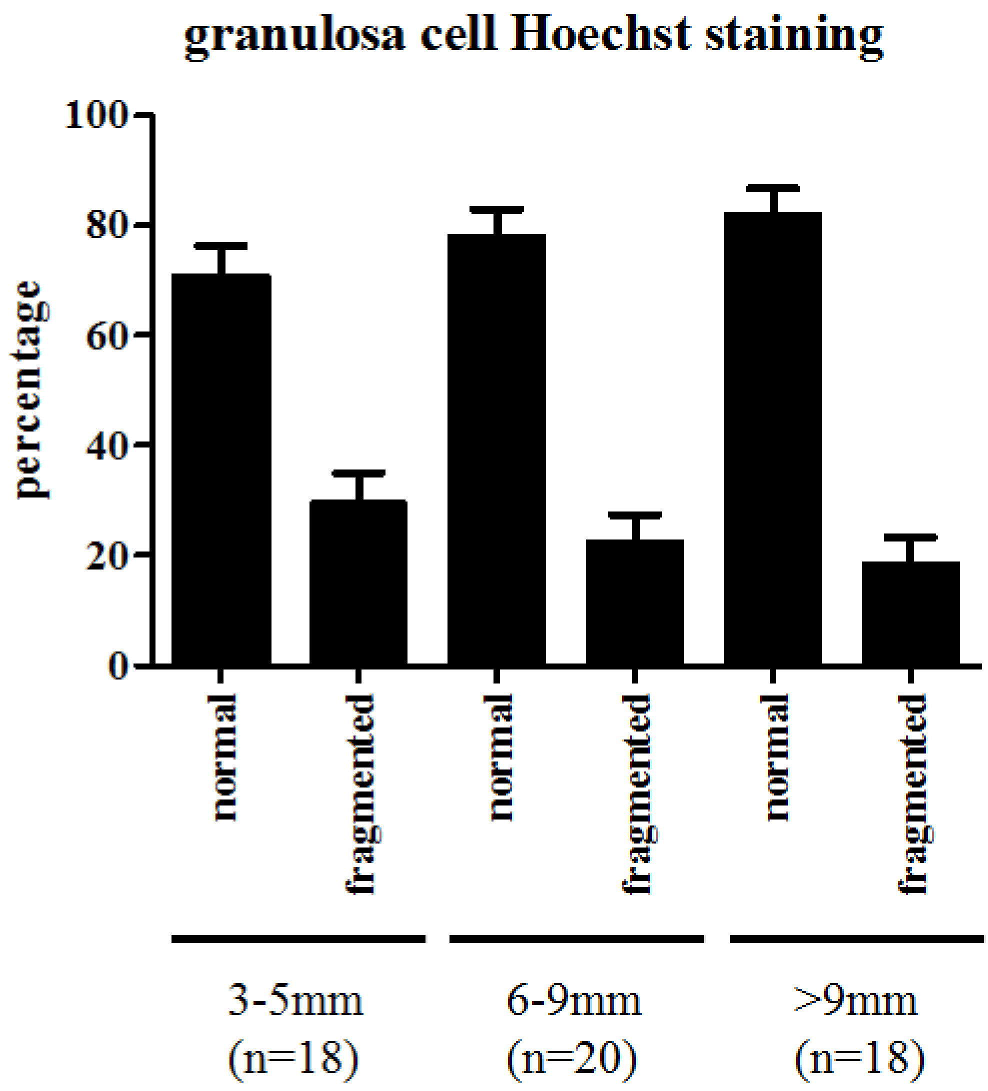

Supplement Figure 1. Percentage of non-apoptotic (normal) and apoptotic (fragmented DNA) granulosa cells from different size follicles. A hundred cells were counted for each follicle. n=follicle numbers analyzed.

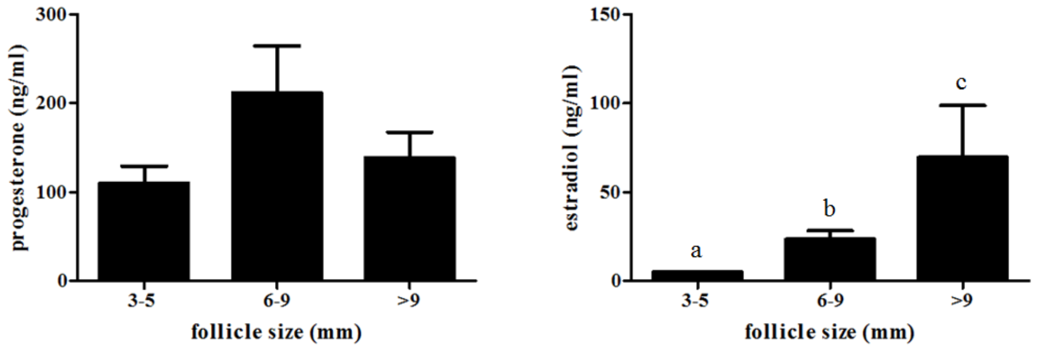

Supplement Figure 2. Progesterone and estradiol concentrations in follicular fluid from different size follicles.

[a,b,c]Means ± SEM with different superscripts were statistically different ($P<0.05$).

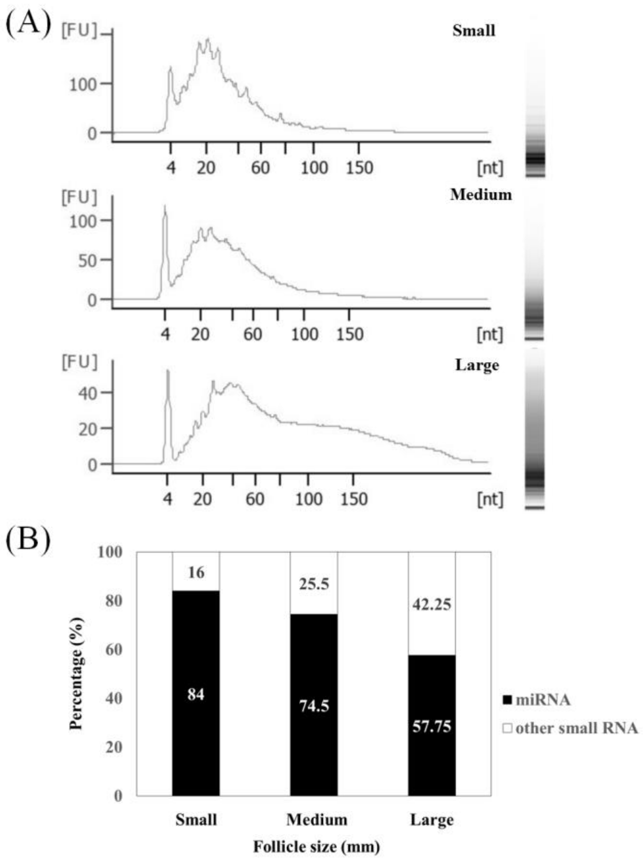

Supplement Figure 3. RNA quality and miRNA percentage using the Agilent Small RNA analysis protocol. A) Isolated RNA from different sized follicles were analyzed using the Agilent Small RNA chip. Distribution of different size of RNA was presented as intensity. The peak at 4 nucleotide represents the spiked in loading control. B) Small RNA with the length from 18-24 nt were considered as potential miRNA (Agilent protocol) and the percentage of miRNA in EVs from different sized follicles were plotted (n=3, $p<0.0001$).

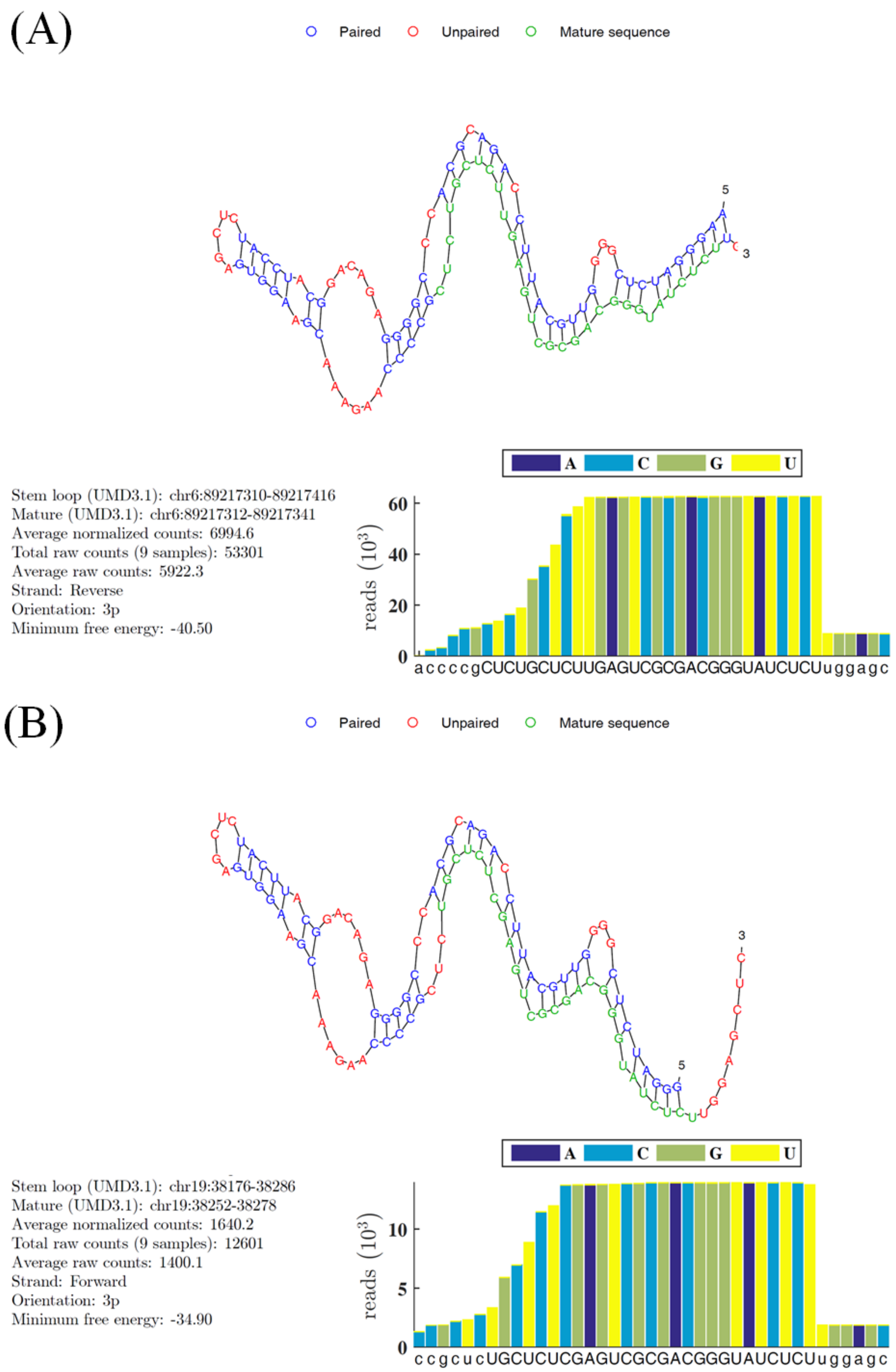

Supplement Figure 4. Stem loop structure for two novel miRNA. Location, read counts, predicted structure, and read intensity of two novel miRNA, A) one located in an annotated region A (ADAMTS3) and B) the other located outside an annotated feature.

Supplement Figure 5.   The 204 known bovine miRNA are followed by 45 (homologous miRNA) and then the list of 455 novel loci that are predicted to encode a miRNA.

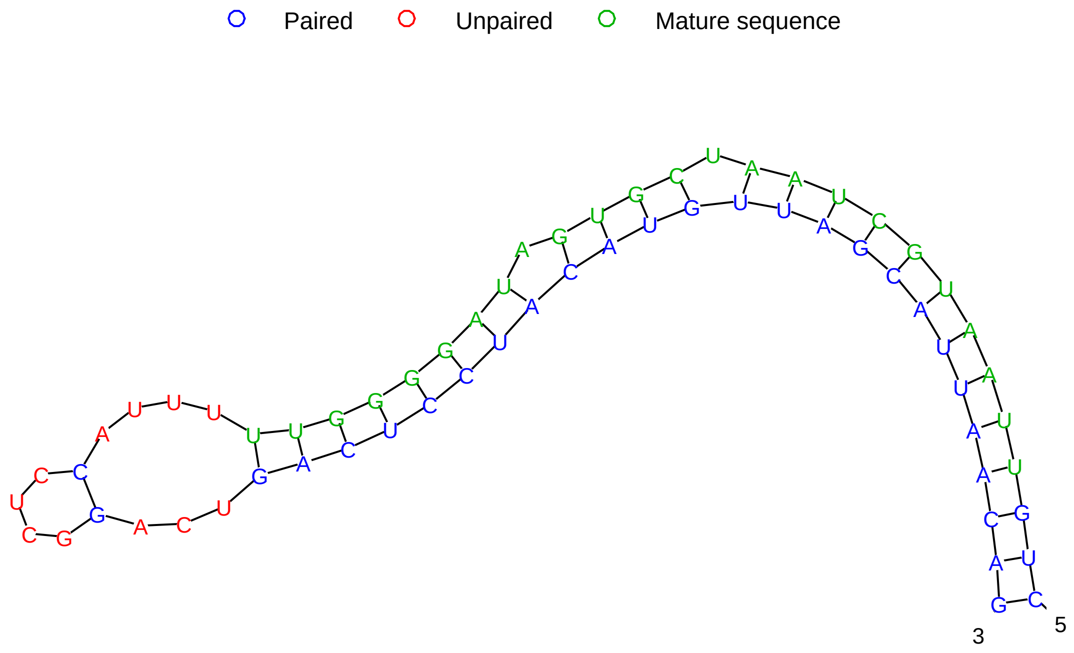

miRNA: bta-miR-155
Stem loop (UMD3.1): chr1:10227276-10227340
Mature (UMD3.1): chr1:10227314-10227337
Mature seq len: 24
Total raw counts (9 samples): 13247
Average raw counts: 1472
Strand: Reverse
Orientation: 5p
Minimum free energy: -30.60

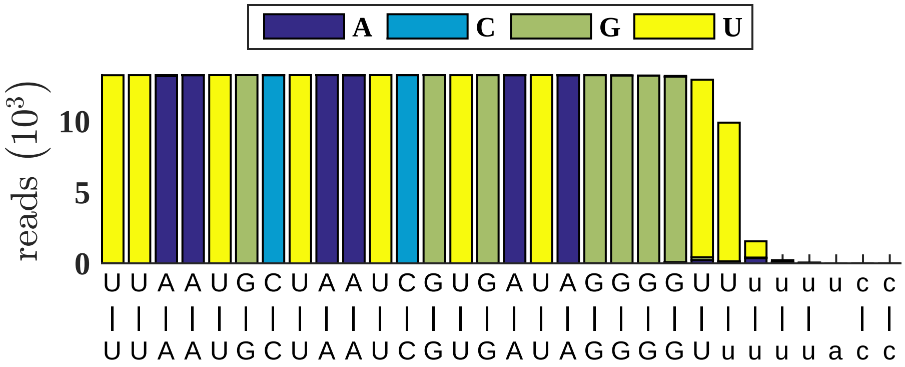

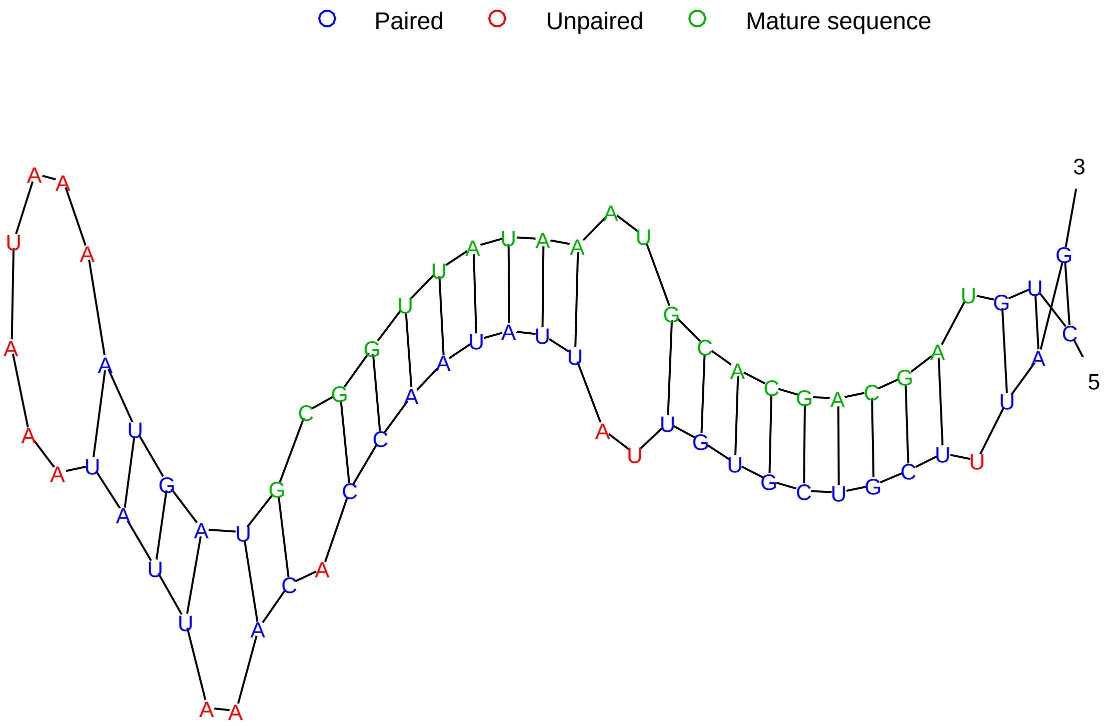

miRNA: bta-miR-16b
Stem loop (UMD3.1): chr1:107923247-107923315
Mature (UMD3.1): chr1:107923291-107923312
Mature seq len: 22
Total raw counts (9 samples): 82716
Average raw counts: 9191
Strand: Reverse
Orientation: 5p
Minimum free energy: -23.70

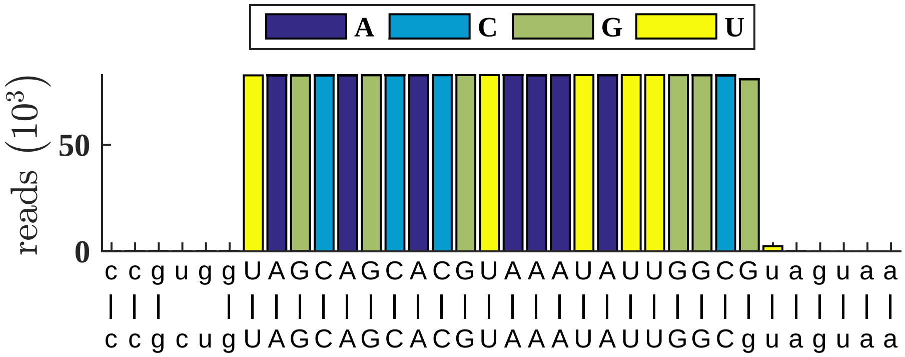

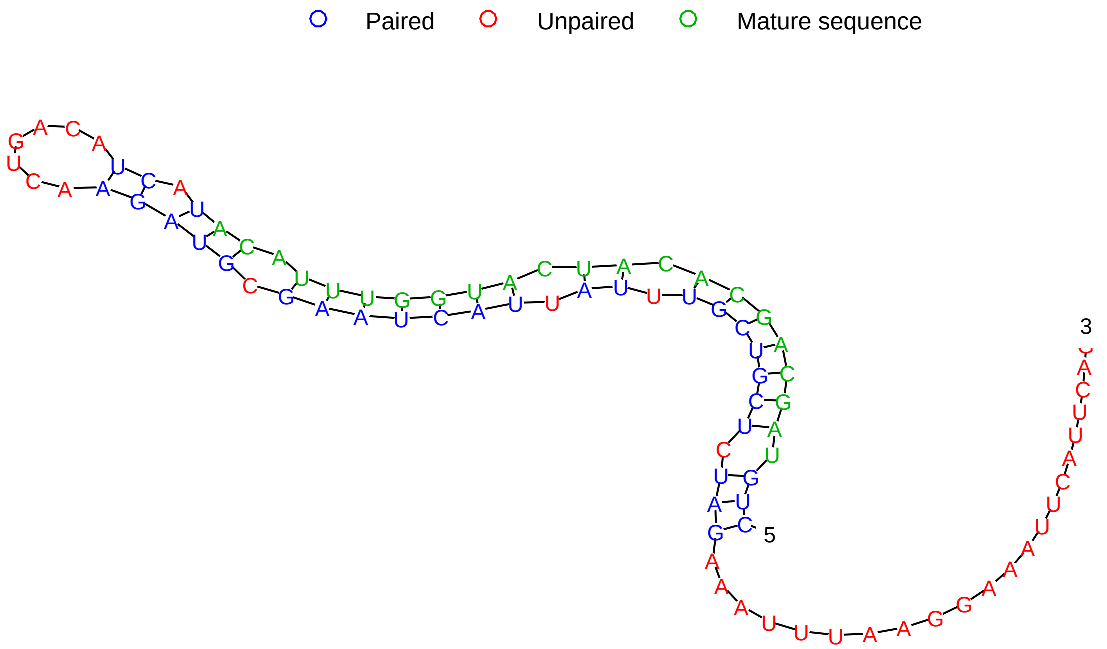

miRNA: bta-miR-15b
Stem loop (UMD3.1): chr1:107923375-107923460
Mature (UMD3.1): chr1:107923436-107923457
Mature seq len: 22
Total raw counts (9 samples): 4916
Average raw counts: 547
Strand: Reverse
Orientation: 5p
Minimum free energy: -20.30

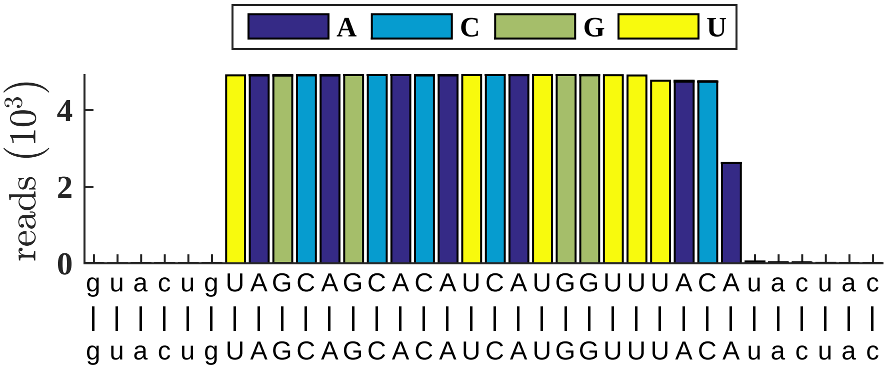

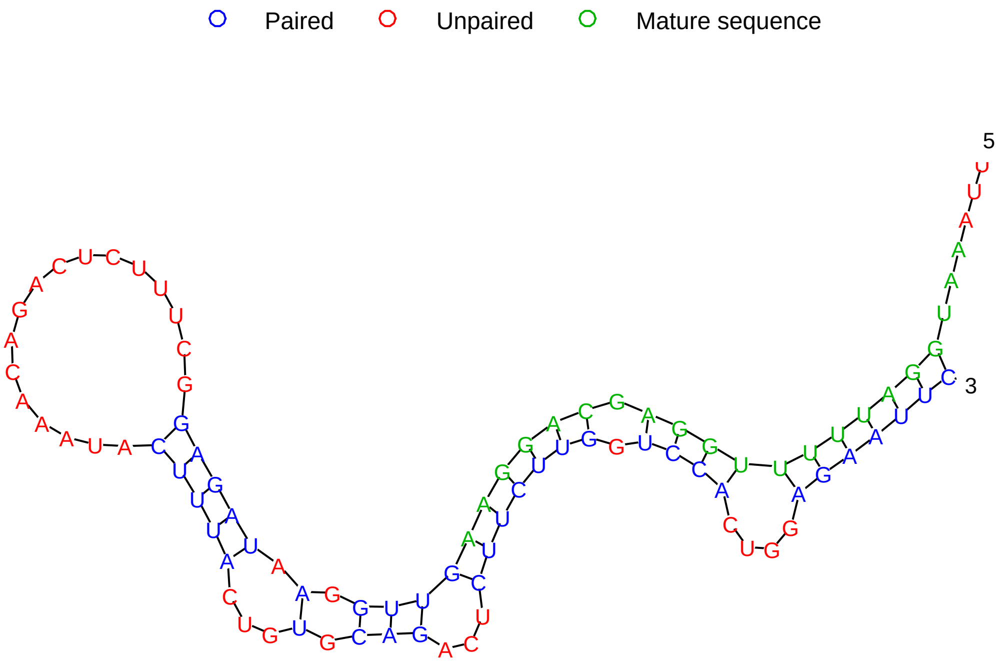

miRNA: bta-miR-1246
Stem loop (UMD3.1): chr1:116822076-116822167
Mature (UMD3.1): chr1:116822079-116822099
Mature seq len: 21
Total raw counts (9 samples): 607
Average raw counts: 68
Strand: Forward
Orientation: 5p
Minimum free energy: -18.90

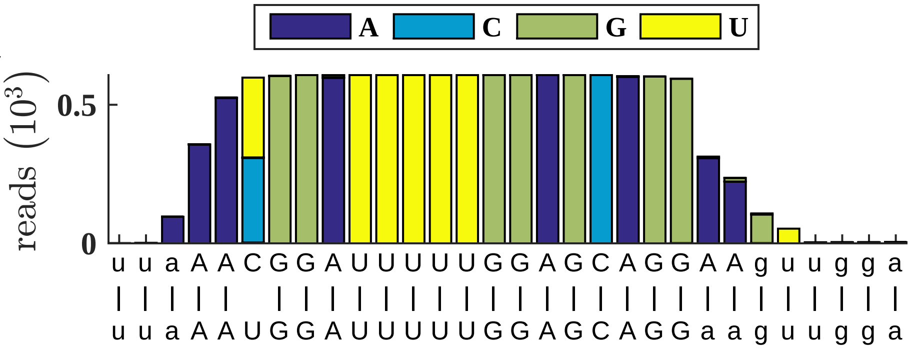

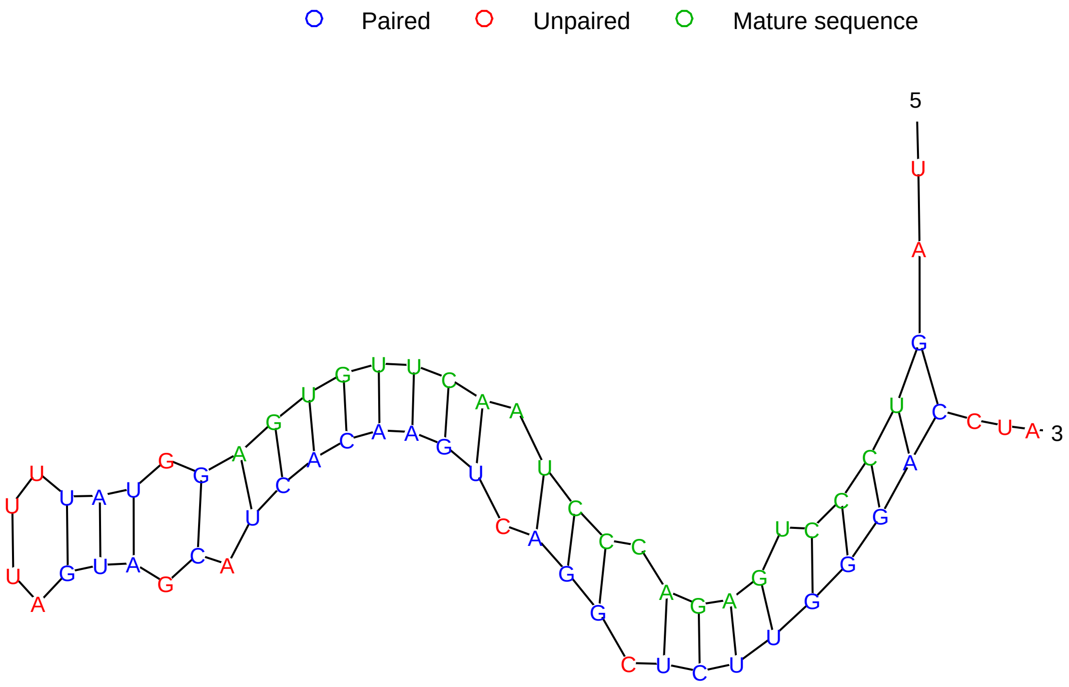

miRNA: bta-miR-125b
Stem loop (UMD3.1): chr1:19881358-19881422
Mature (UMD3.1): chr1:19881398-19881419
Mature seq len: 22
Total raw counts (9 samples): 5085
Average raw counts: 565
Strand: Reverse
Orientation: 5p
Minimum free energy: -28.90

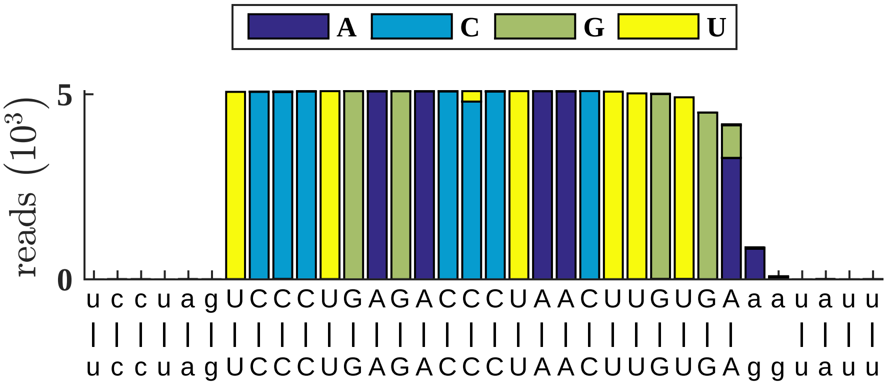

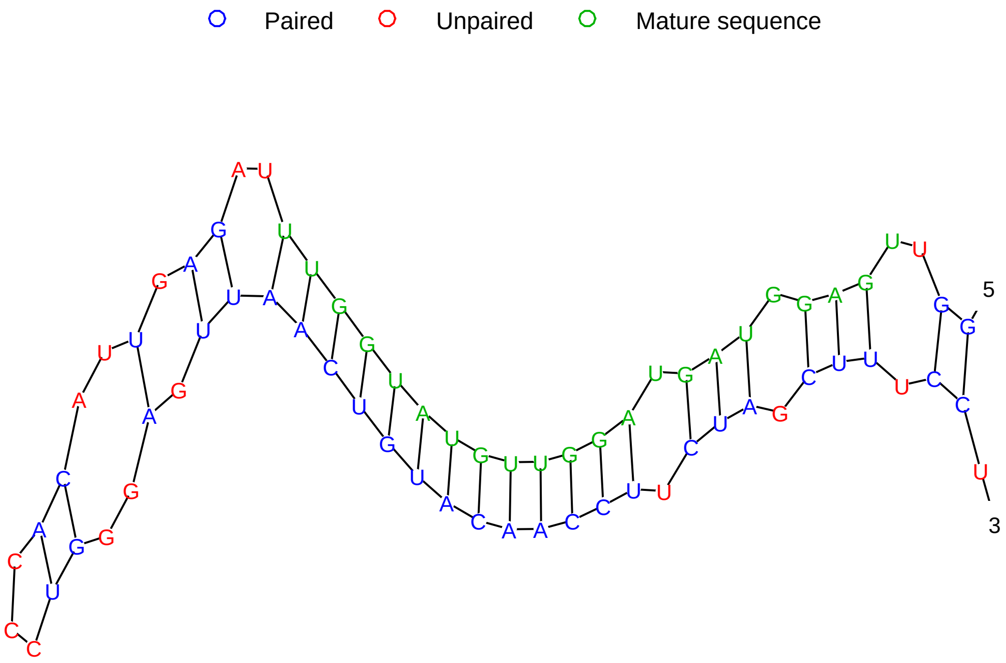

miRNA: bta-let-7c
Stem loop (UMD3.1): chr1:19930465-19930535
Mature (UMD3.1): chr1:19930511-19930532
Mature seq len: 22
Total raw counts (9 samples): 176699
Average raw counts: 19634
Strand: Reverse
Orientation: 5p
Minimum free energy: -24.40

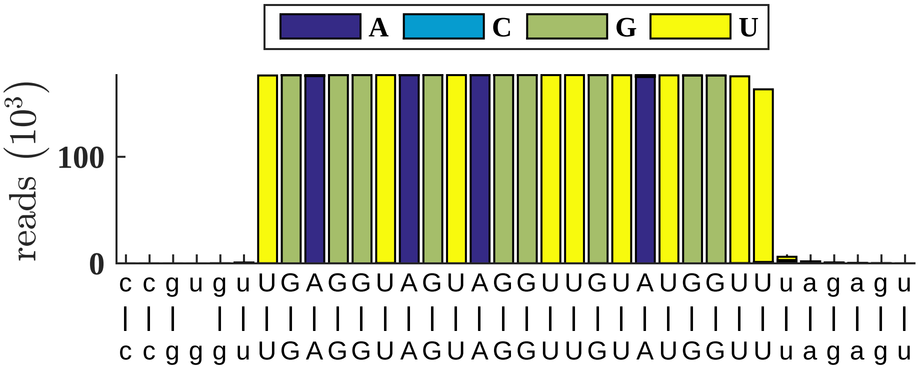

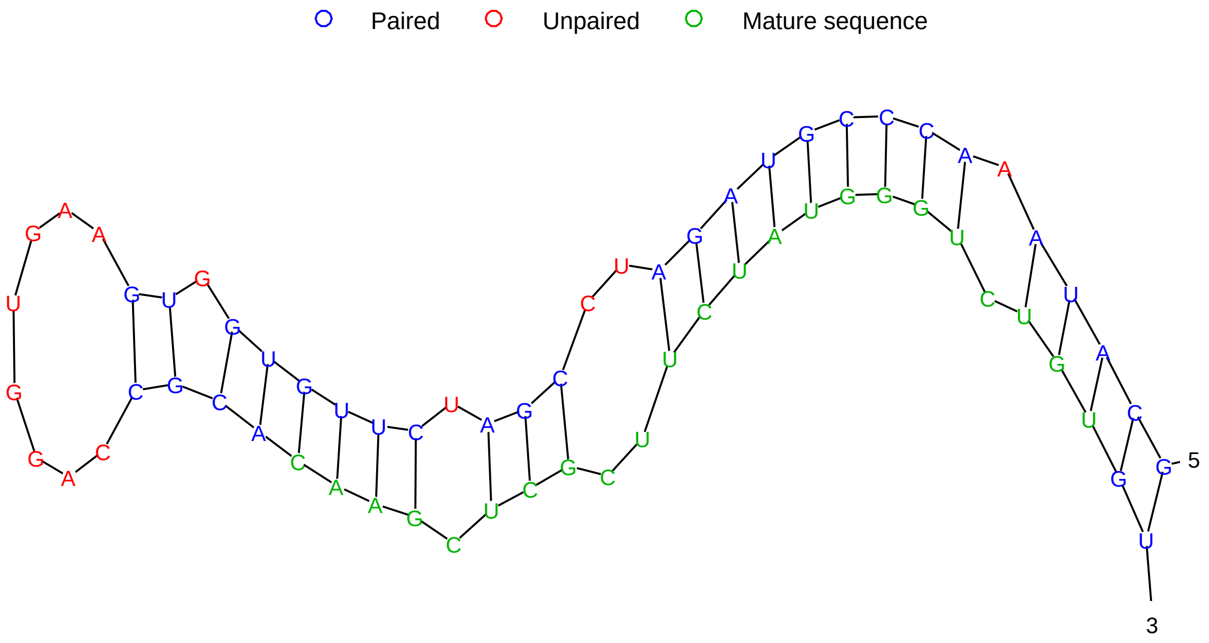

miRNA: bta-miR-99a-3p
Stem loop (UMD3.1): chr1:19931192-19931258
Mature (UMD3.1): chr1:19931194-19931216
Mature seq len: 23
Total raw counts (9 samples): 1761
Average raw counts: 196
Strand: Reverse
Orientation: 3p
Minimum free energy: -30.30

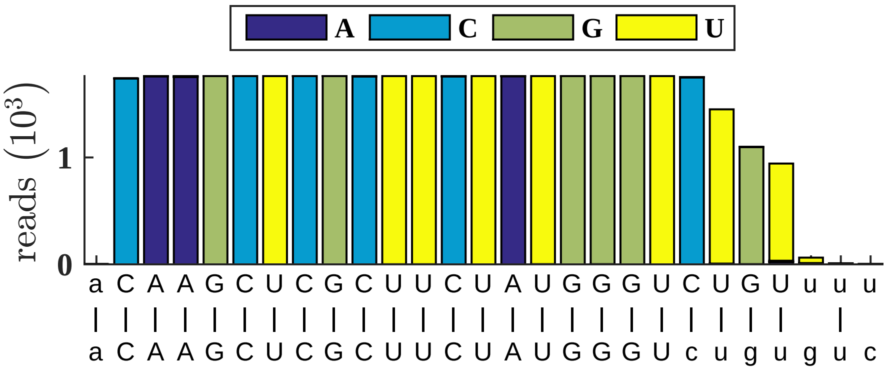

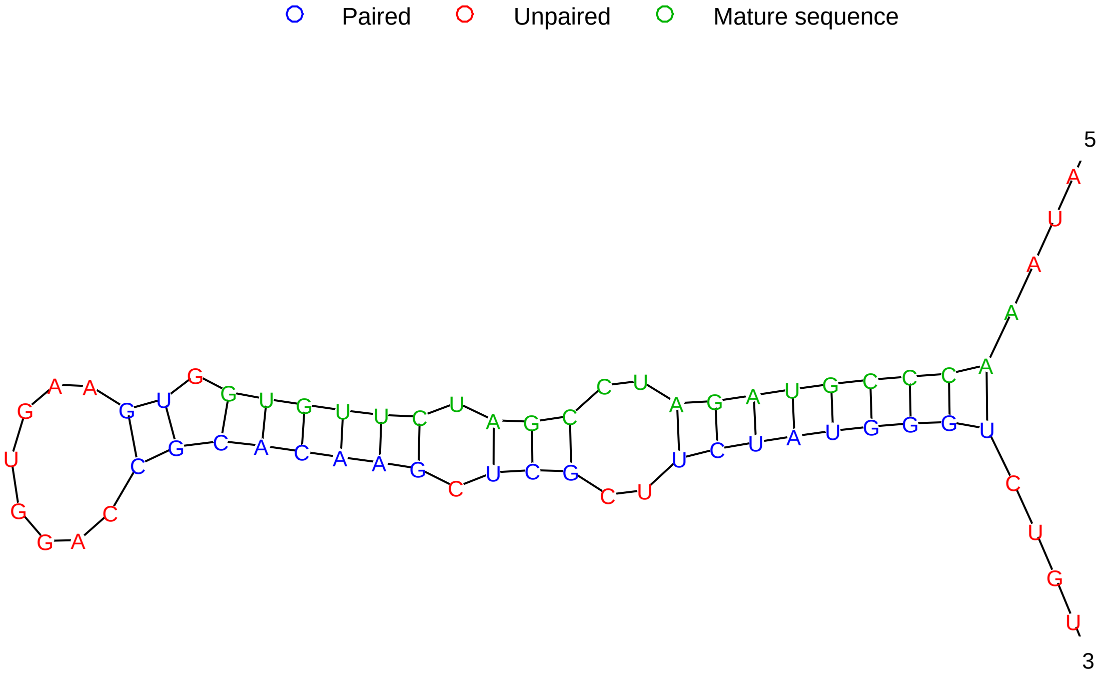

miRNA: bta-miR-99a-5p
Stem loop (UMD3.1): chr1:19931194-19931256
Mature (UMD3.1): chr1:19931232-19931253
Mature seq len: 22
Total raw counts (9 samples): 13472
Average raw counts: 1497
Strand: Reverse
Orientation: 5p
Minimum free energy: -25.90

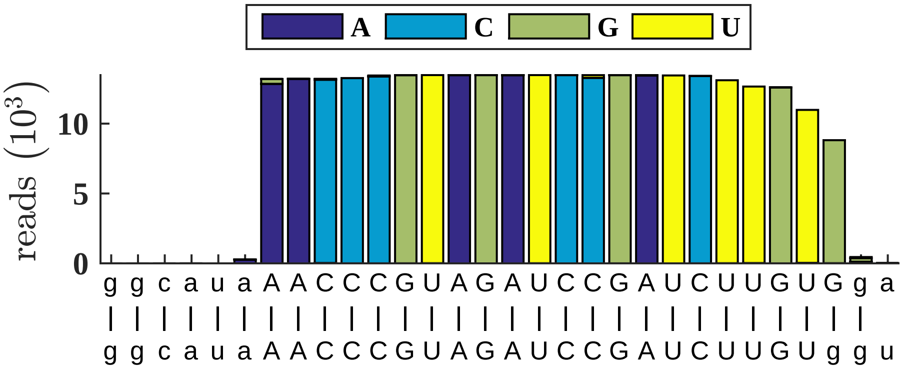

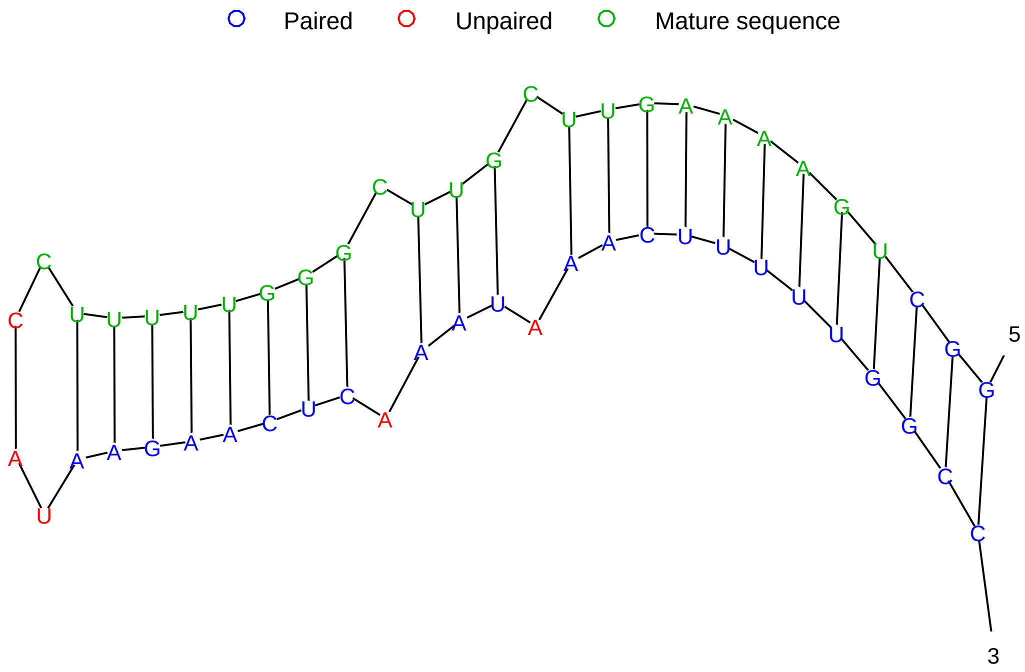

miRNA: bta-miR-2284x
Stem loop (UMD3.1): chr1:3607772-3607825
Mature (UMD3.1): chr1:3607800-3607822
Mature seq len: 23
Total raw counts (9 samples): 55259
Average raw counts: 6140
Strand: Reverse
Orientation: 5p
Minimum free energy: -20.80

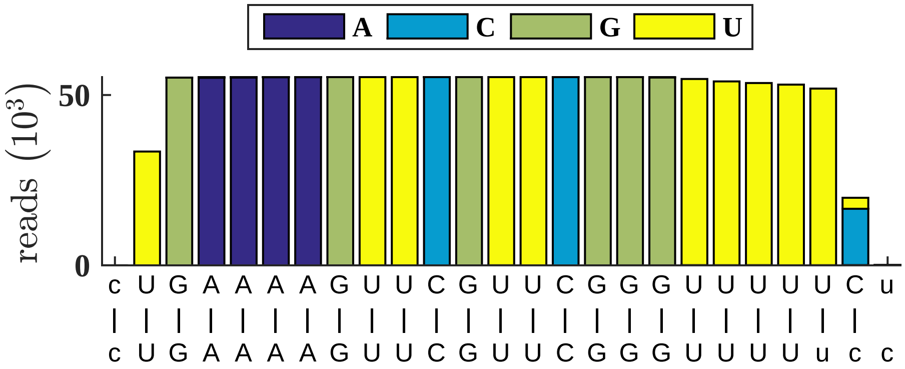

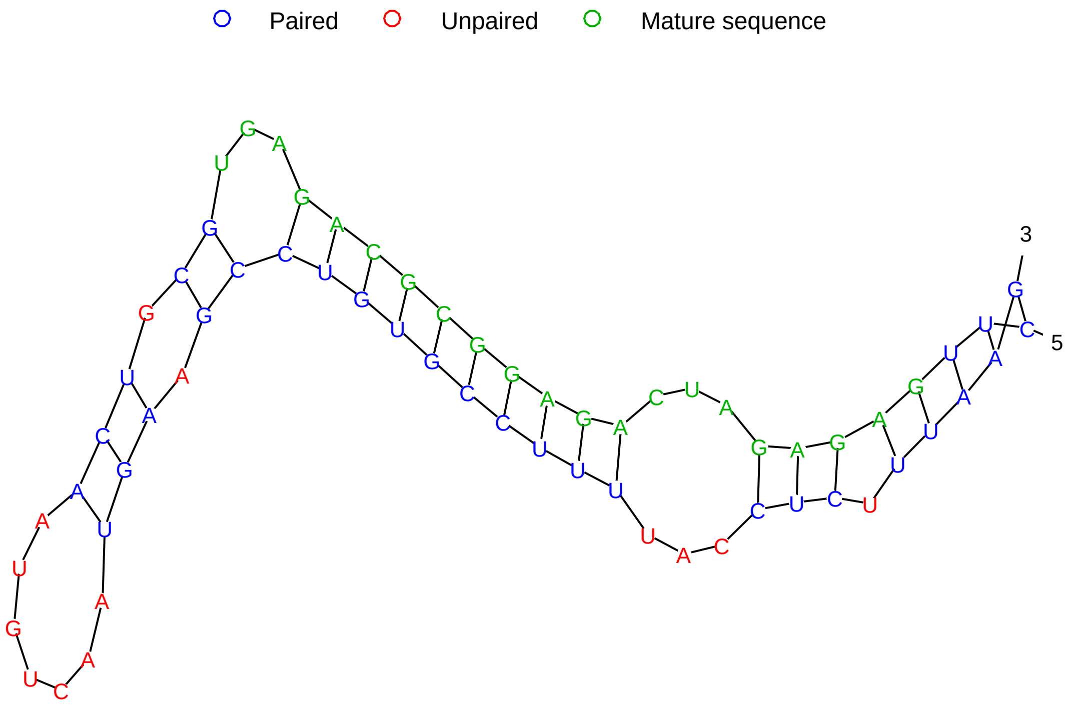

miRNA: bta-miR-6529a
Stem loop (UMD3.1): chr1:65453356-65453420
Mature (UMD3.1): chr1:65453397-65453417
Mature seq len: 21
Total raw counts (9 samples): 22300
Average raw counts: 2478
Strand: Reverse
Orientation: 5p
Minimum free energy: -22.00

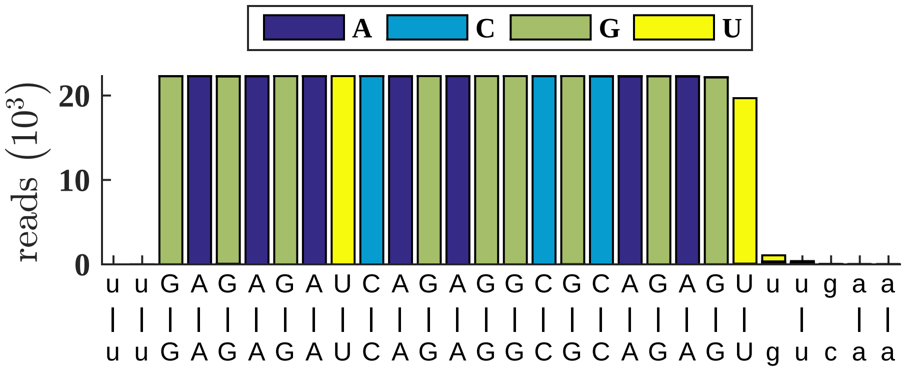

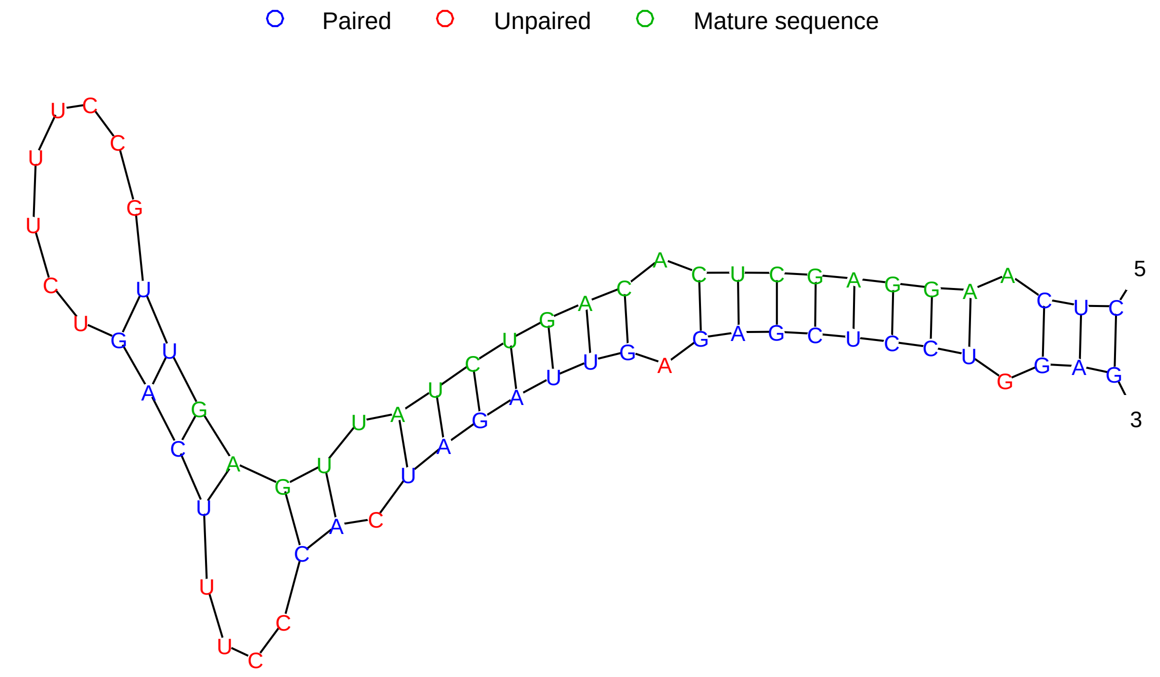

miRNA: bta-miR-28
Stem loop (UMD3.1): chr1:79250541-79250606
Mature (UMD3.1): chr1:79250582-79250603
Mature seq len: 22
Total raw counts (9 samples): 3877
Average raw counts: 431
Strand: Reverse
Orientation: 5p
Minimum free energy: -26.60

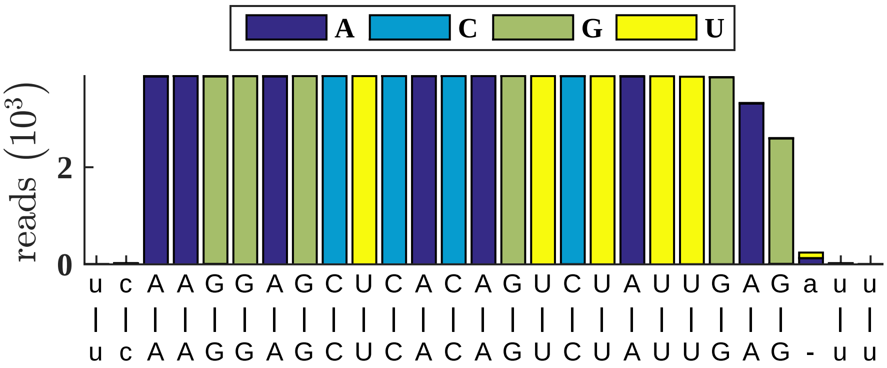

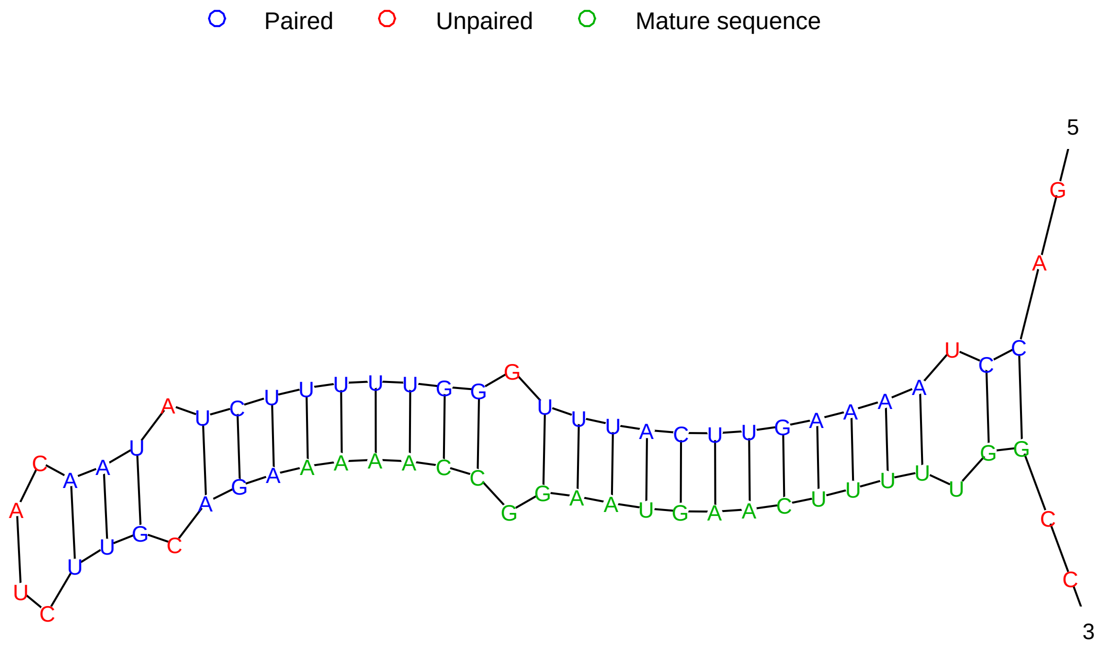

miRNA: bta-miR-2285k
Stem loop (UMD3.1): chr1:80871208-80871273
Mature (UMD3.1): chr1:80871210-80871231
Mature seq len: 22
Total raw counts (9 samples): 570
Average raw counts: 64
Strand: Reverse
Orientation: 3p
Minimum free energy: -28.50

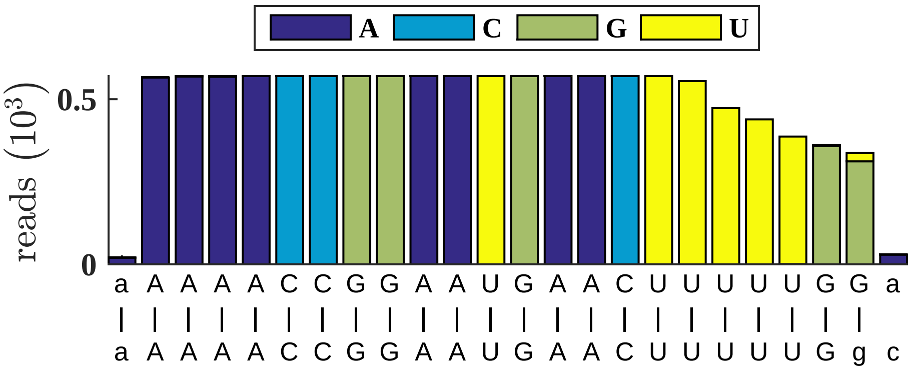

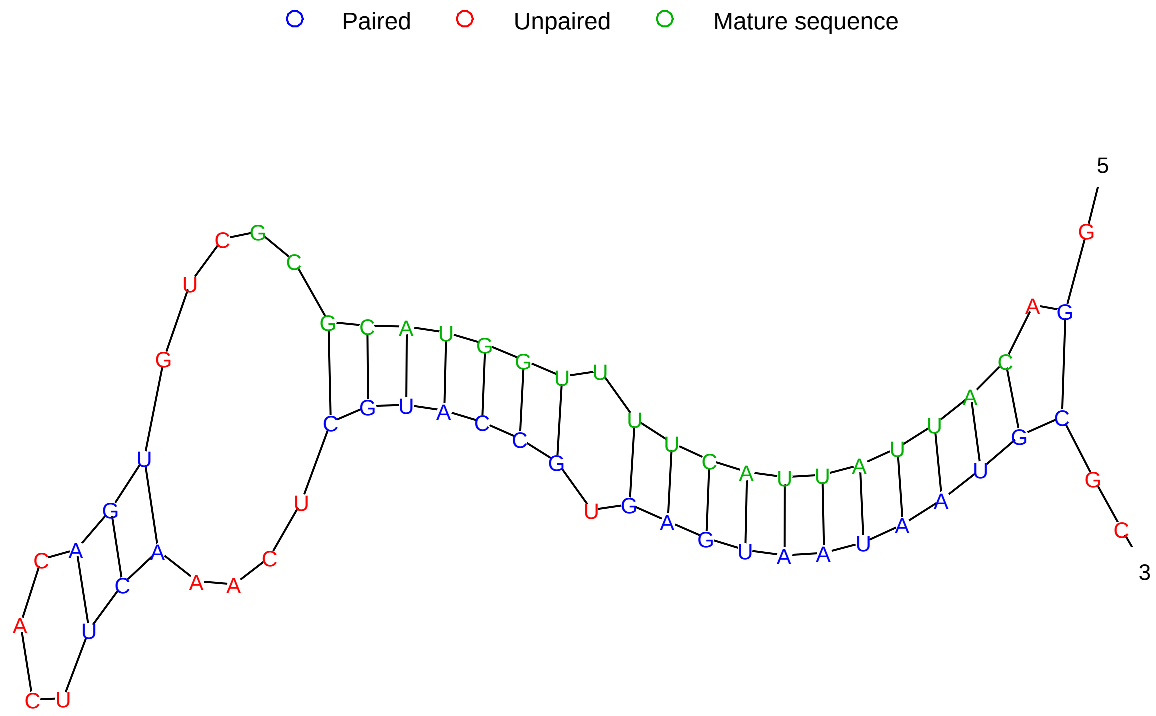

miRNA: bta-miR-126-5p
Stem loop (UMD3.1): chr11:104131414-104131476
Mature (UMD3.1): chr11:104131417-104131437
Mature seq len: 21
Total raw counts (9 samples): 1753
Average raw counts: 195
Strand: Forward
Orientation: 5p
Minimum free energy: -22.90

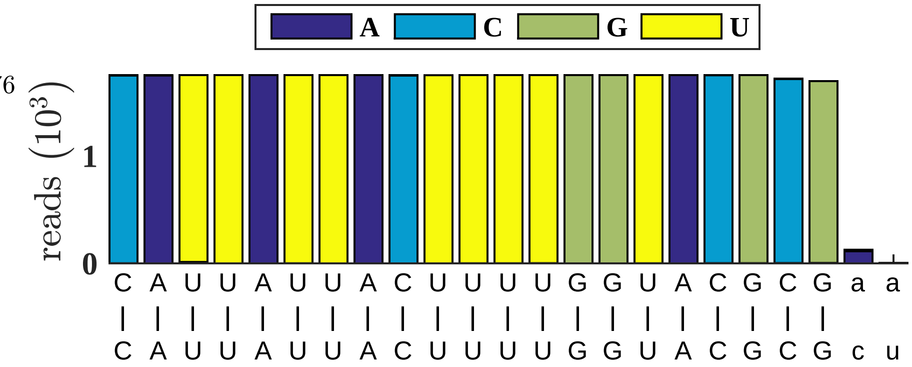

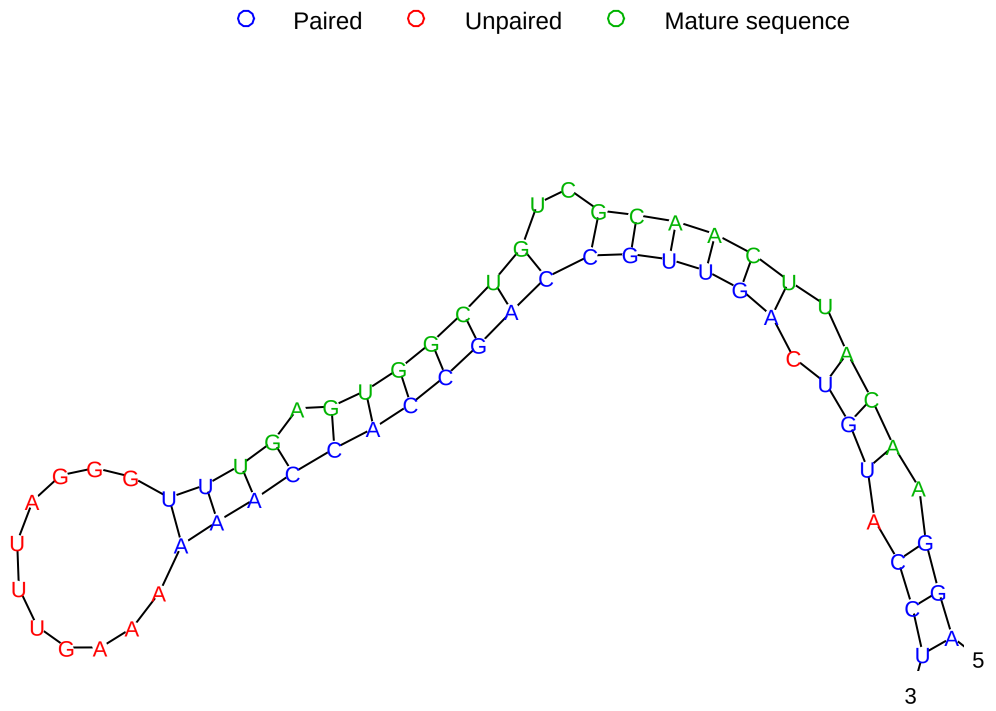

miRNA: bta-miR-181a
Stem loop (UMD3.1): chr11:95709446-95709509
Mature (UMD3.1): chr11:95709449-95709471
Mature seq len: 23
Total raw counts (9 samples): 40506
Average raw counts: 4501
Strand: Forward
Orientation: 5p
Minimum free energy: -26.30

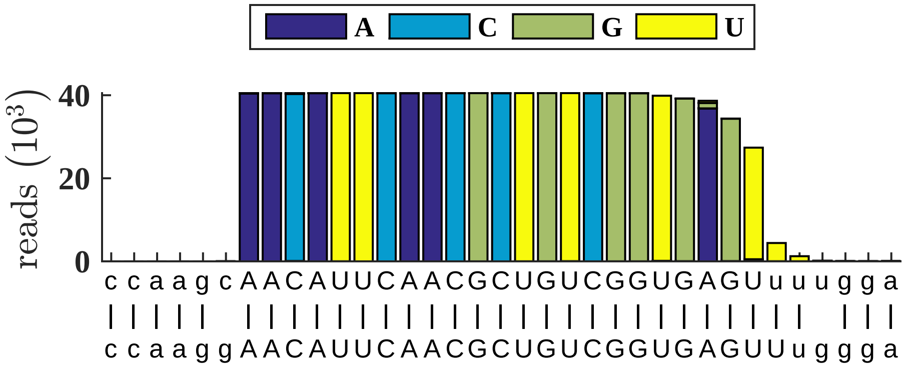

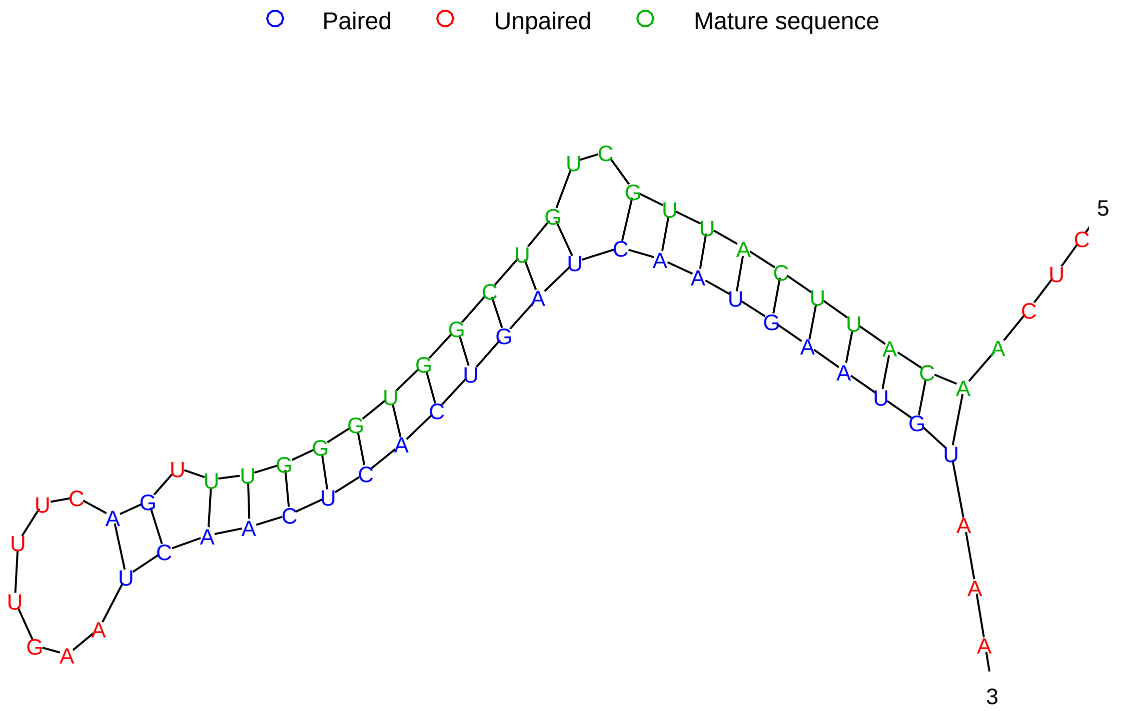

miRNA: bta-miR-181b
Stem loop (UMD3.1): chr11:95710638-95710700
Mature (UMD3.1): chr11:95710641-95710664
Mature seq len: 24
Total raw counts (9 samples): 6787
Average raw counts: 755
Strand: Forward
Orientation: 5p
Minimum free energy: -25.20

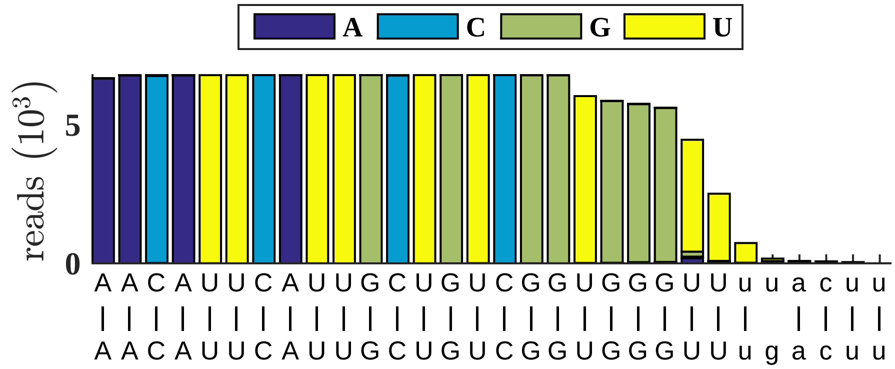

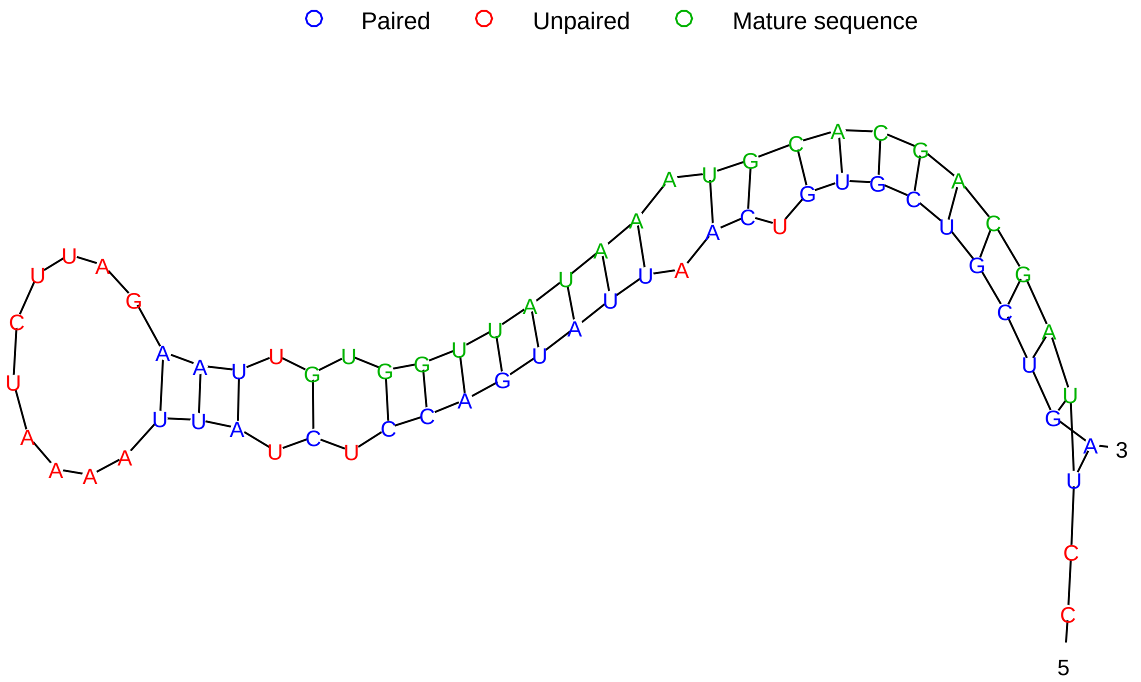

miRNA: bta-miR-16a
Stem loop (UMD3.1): chr12:19596212-19596278
Mature (UMD3.1): chr12:19596254-19596275
Mature seq len: 22
Total raw counts (9 samples): 31840
Average raw counts: 3538
Strand: Reverse
Orientation: 5p
Minimum free energy: -25.10

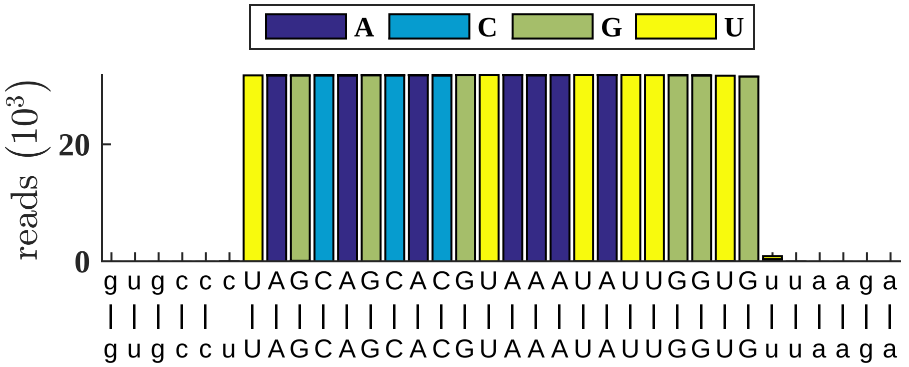

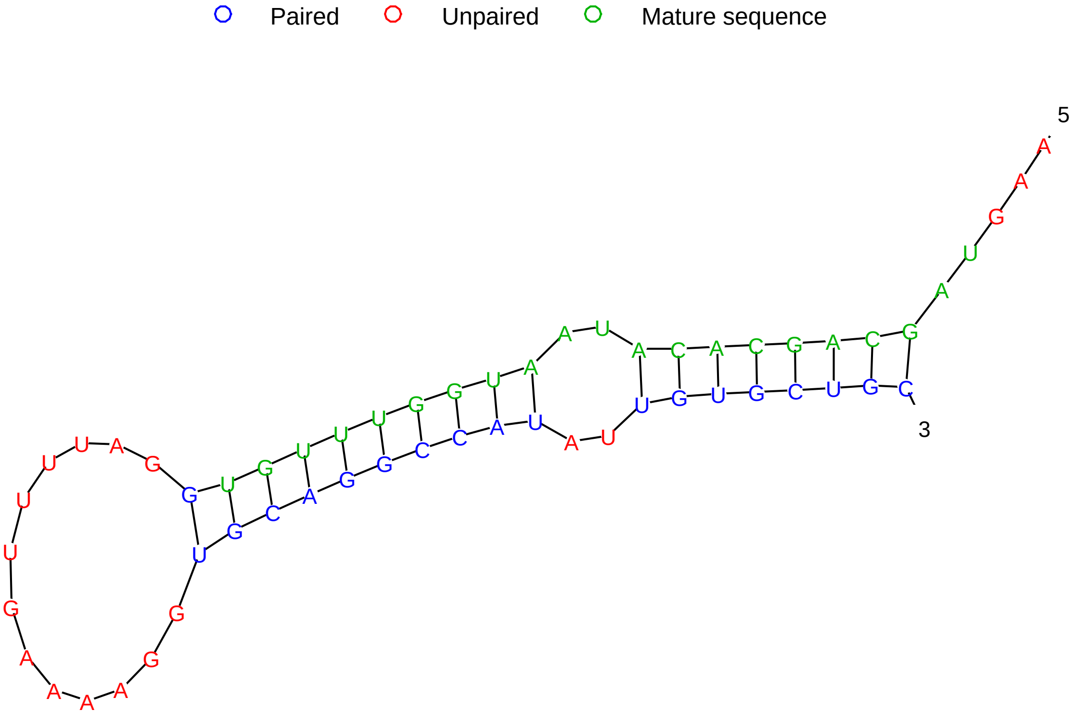

miRNA: bta-miR-15a
Stem loop (UMD3.1): chr12:19596361-19596418
Mature (UMD3.1): chr12:19596395-19596415
Mature seq len: 21
Total raw counts (9 samples): 14669
Average raw counts: 1630
Strand: Reverse
Orientation: 5p
Minimum free energy: -24.00

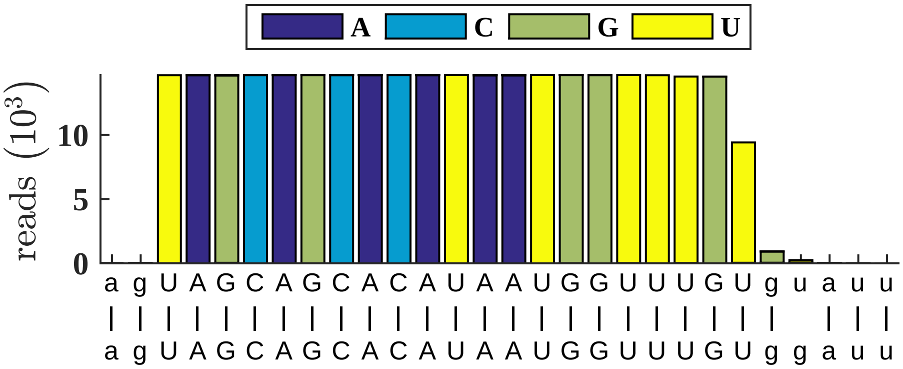

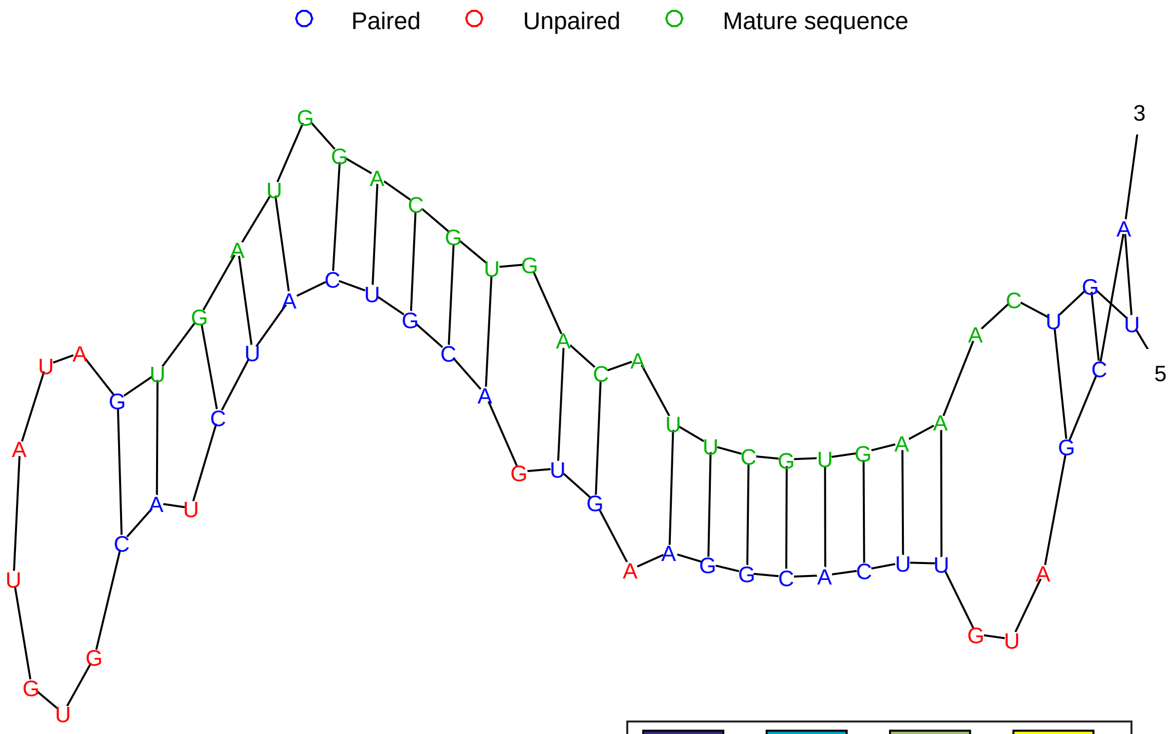

miRNA: bta-miR-17-5p
Stem loop (UMD3.1): chr12:66226564-66226627
Mature (UMD3.1): chr12:66226567-66226590
Mature seq len: 24
Total raw counts (9 samples): 12221
Average raw counts: 1358
Strand: Forward
Orientation: 5p
Minimum free energy: -23.50

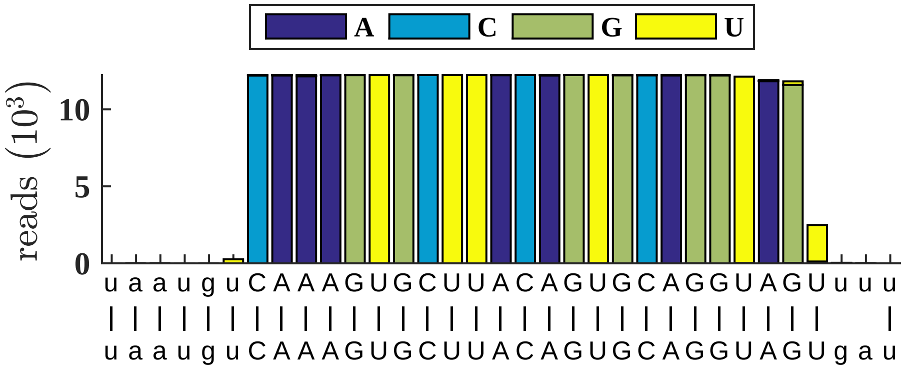

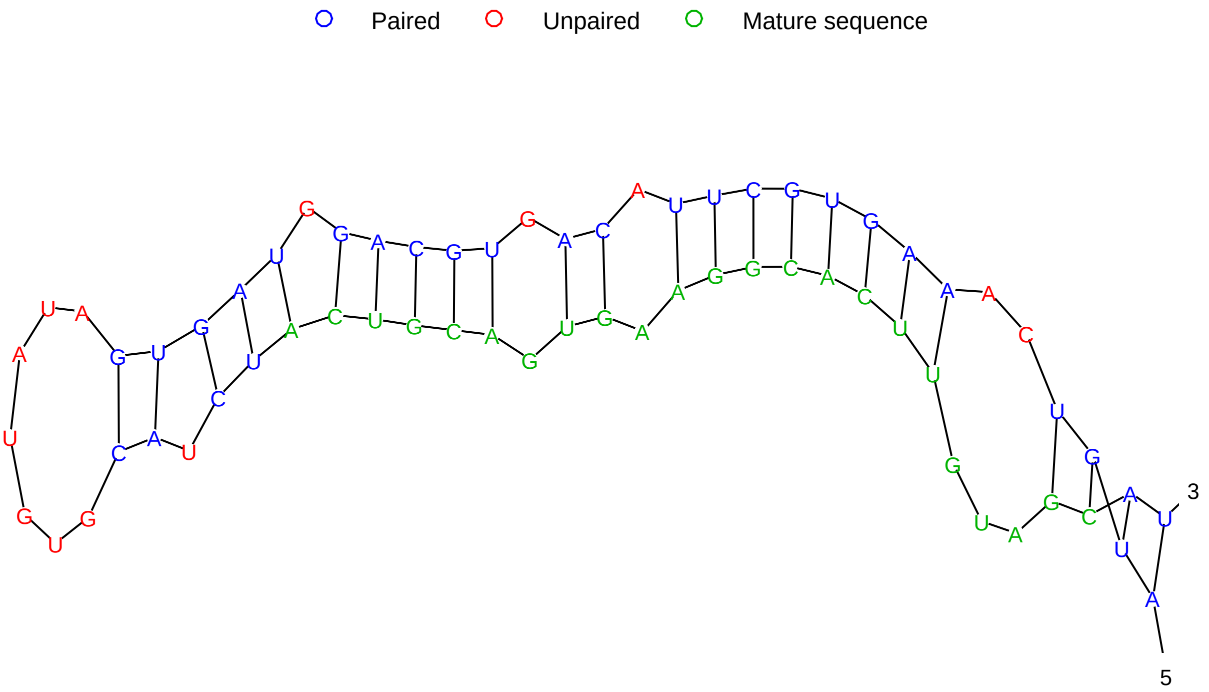

miRNA: bta-miR-17-3p
Stem loop (UMD3.1): chr12:66226563-66226628
Mature (UMD3.1): chr12:66226604-66226626
Mature seq len: 23
Total raw counts (9 samples): 777
Average raw counts: 87
Strand: Forward
Orientation: 3p
Minimum free energy: -24.60

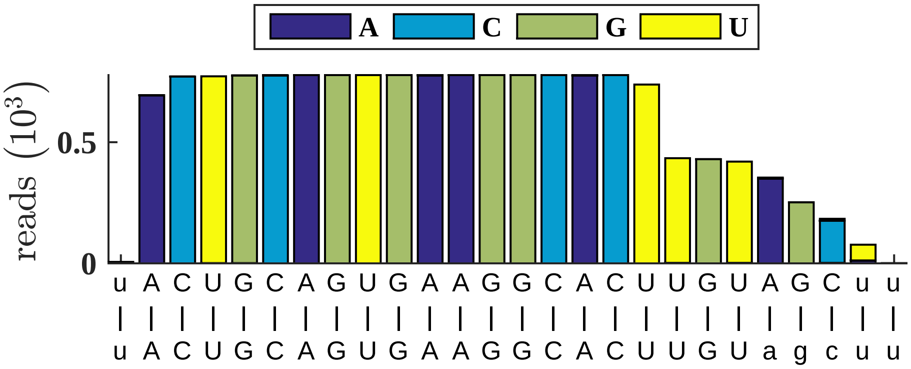

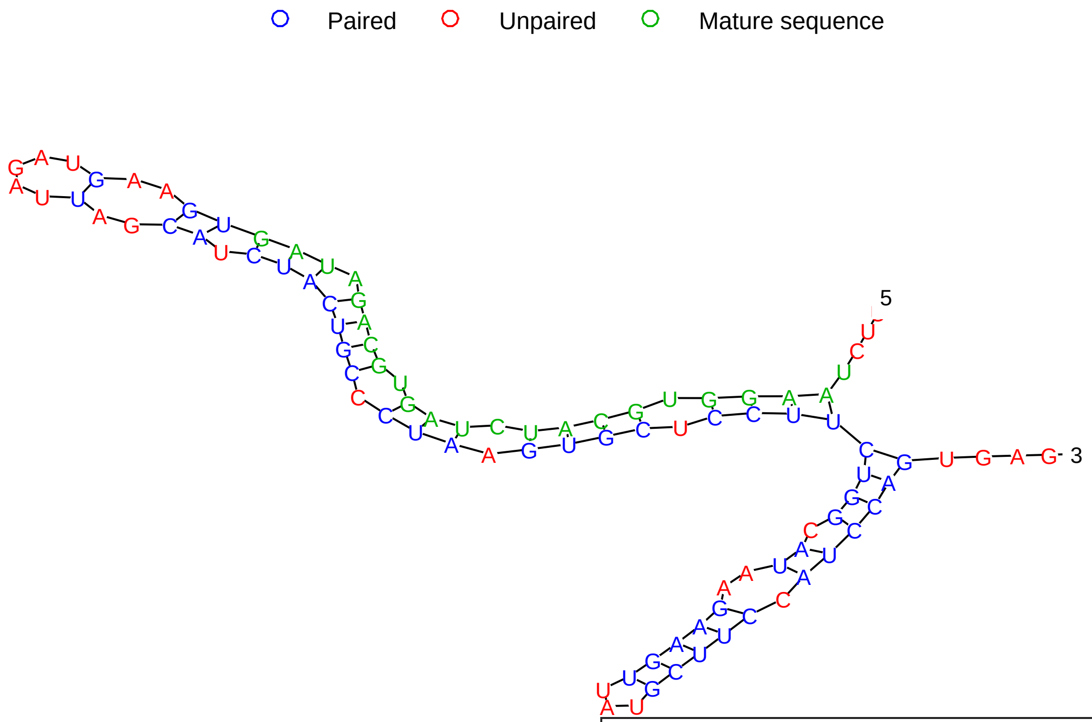

miRNA: bta-miR-18a
Stem loop (UMD3.1): chr12:66226699-66226794
Mature (UMD3.1): chr12:66226702-66226724
Mature seq len: 23
Total raw counts (9 samples): 547
Average raw counts: 61
Strand: Forward
Orientation: 5p
Minimum free energy: -25.90

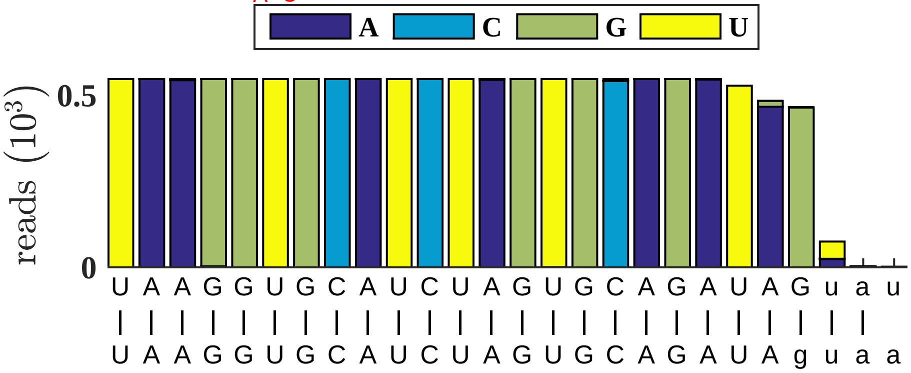

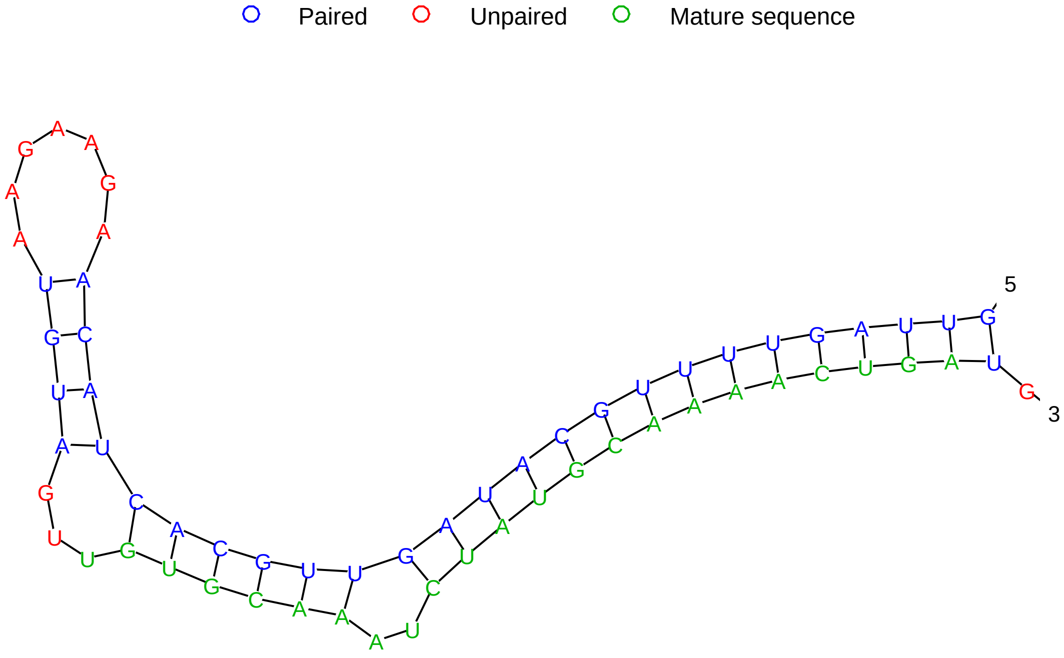

miRNA: bta-miR-19a
Stem loop (UMD3.1): chr12:66226846-66226908
Mature (UMD3.1): chr12:66226884-66226906
Mature seq len: 23
Total raw counts (9 samples): 6400
Average raw counts: 712
Strand: Forward
Orientation: 3p
Minimum free energy: -26.60

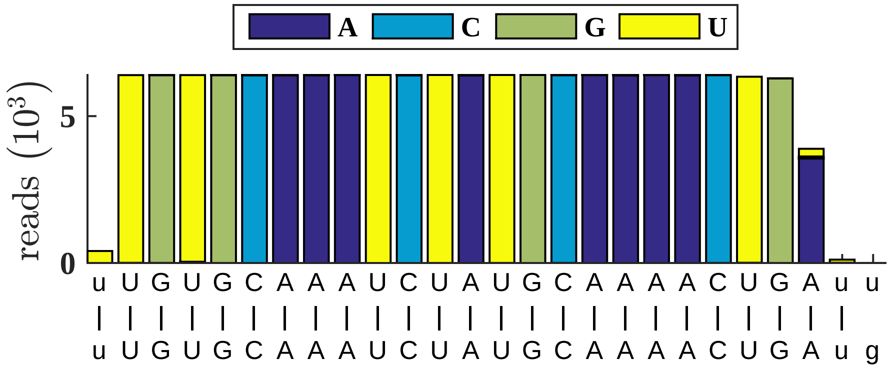

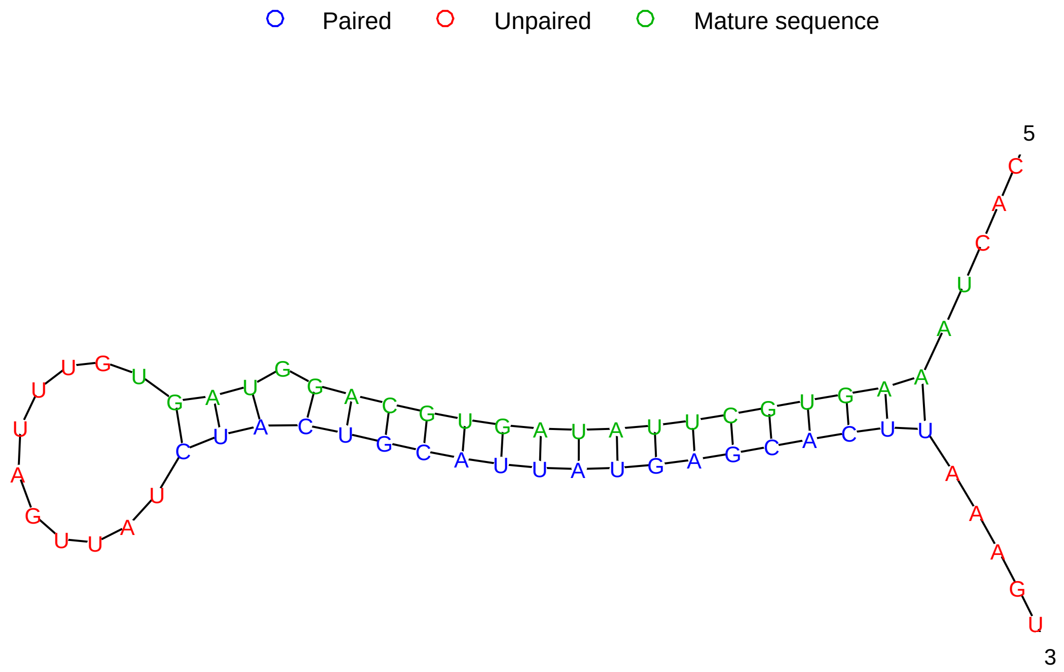

miRNA: bta-miR-20a
Stem loop (UMD3.1): chr12:66227009-66227070
Mature (UMD3.1): chr12:66227012-66227035
Mature seq len: 24
Total raw counts (9 samples): 3426
Average raw counts: 381
Strand: Forward
Orientation: 5p
Minimum free energy: -26.50

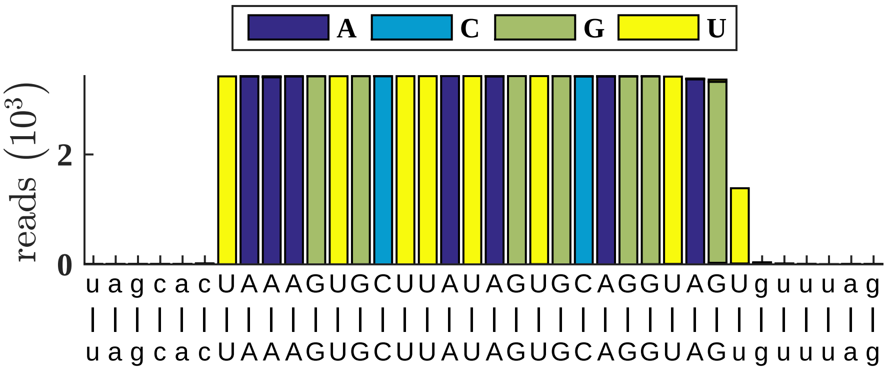

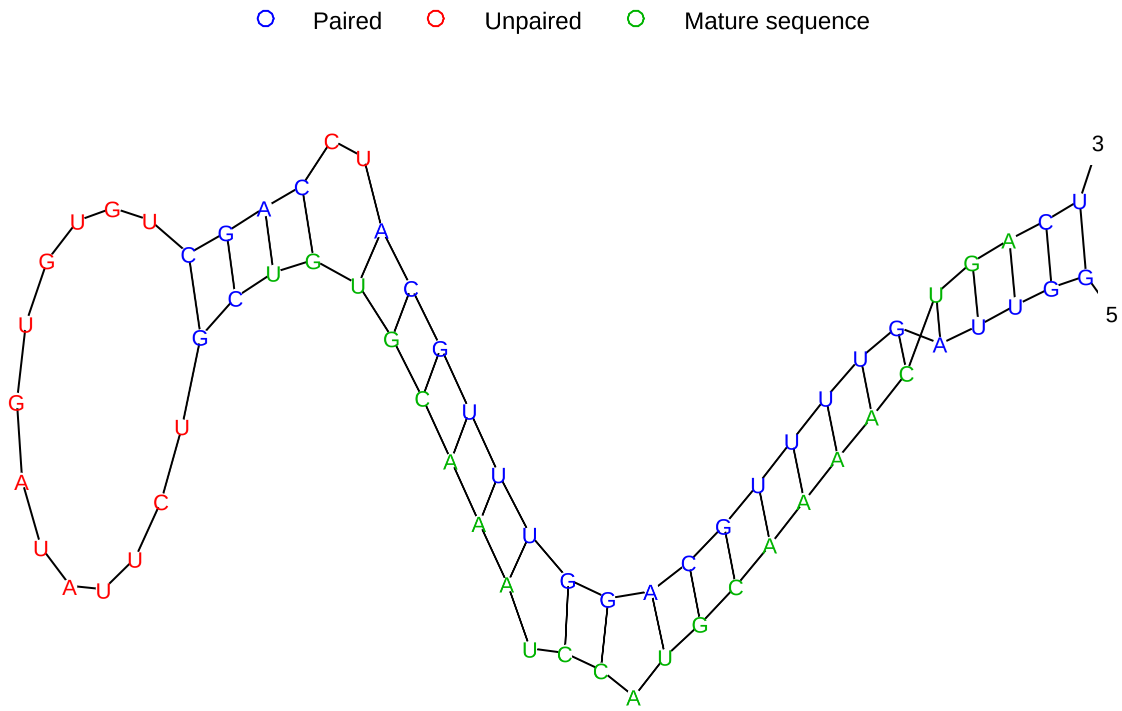

miRNA: bta-miR-19b
Stem loop (UMD3.1): chr12:66227144-66227210
Mature (UMD3.1): chr12:66227186-66227208
Mature seq len: 23
Total raw counts (9 samples): 10432
Average raw counts: 1160
Strand: Forward
Orientation: 3p
Minimum free energy: -29.70

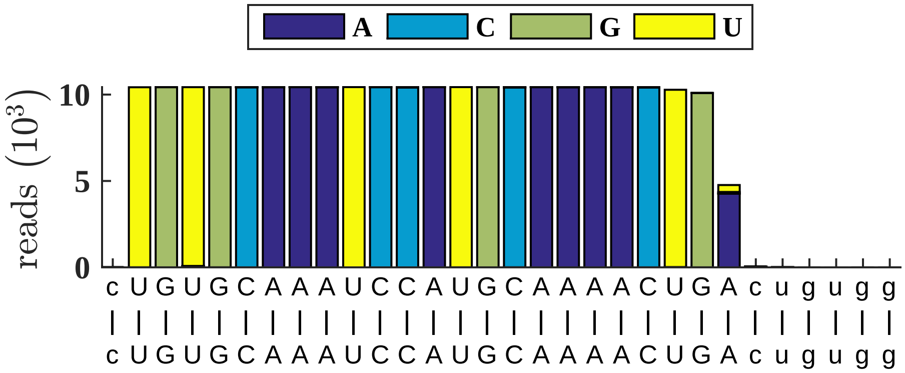

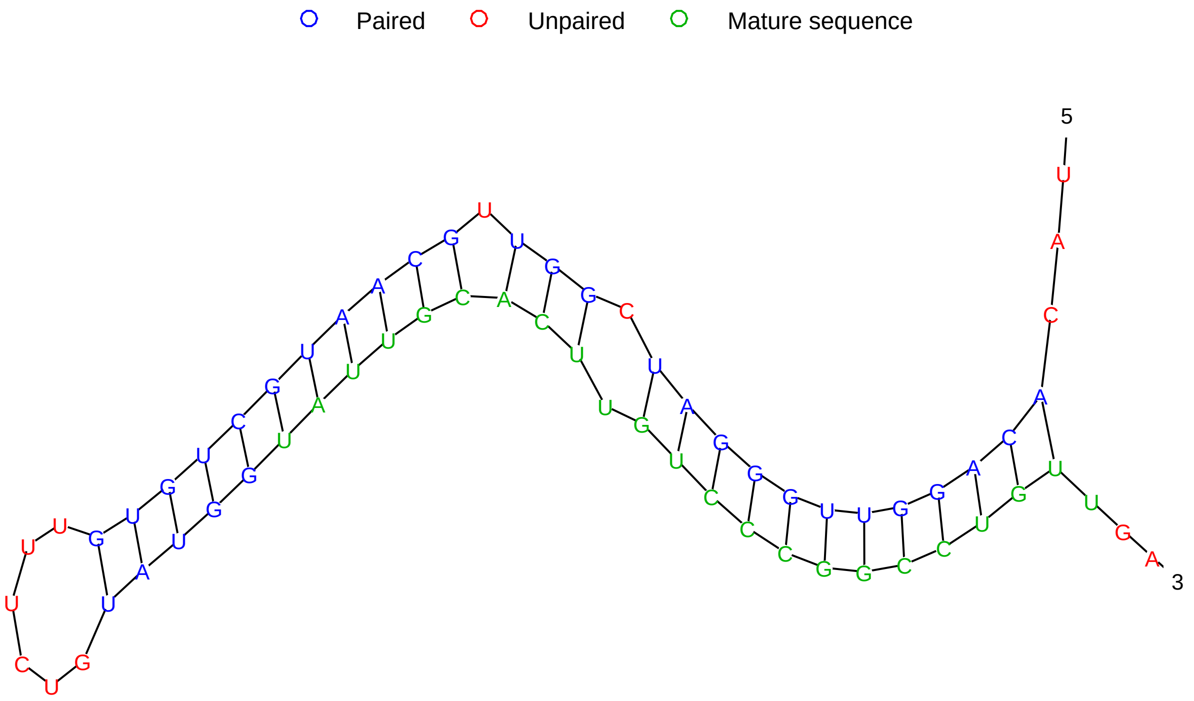

miRNA: bta-miR-92a
Stem loop (UMD3.1): chr12:66227260-66227326
Mature (UMD3.1): chr12:66227302-66227324
Mature seq len: 23
Total raw counts (9 samples): 321784
Average raw counts: 35754
Strand: Forward
Orientation: 3p
Minimum free energy: -35.20

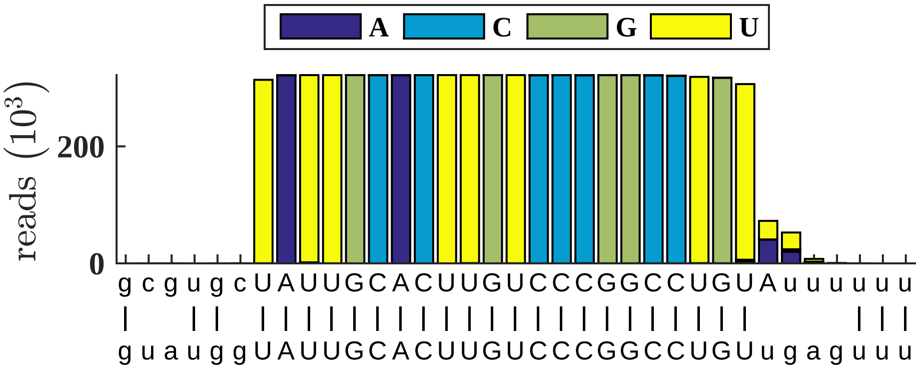

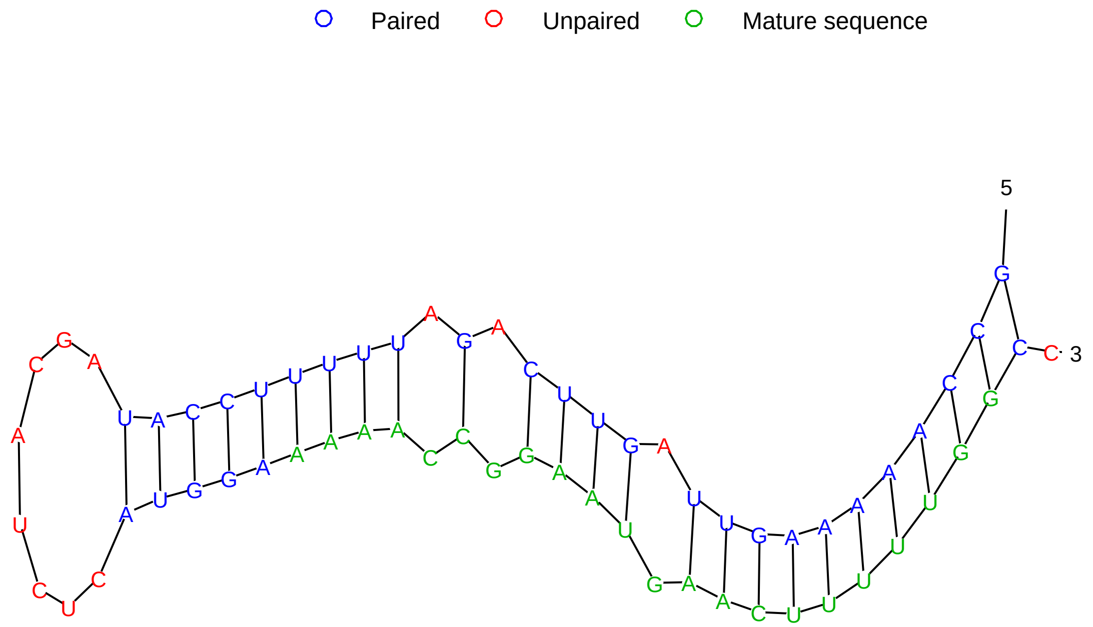

miRNA: bta-miR-2285k
Stem loop (UMD3.1): chr13:23904425-23904489
Mature (UMD3.1): chr13:23904427-23904448
Mature seq len: 22
Total raw counts (9 samples): 616
Average raw counts: 69
Strand: Reverse
Orientation: 3p
Minimum free energy: -26.80

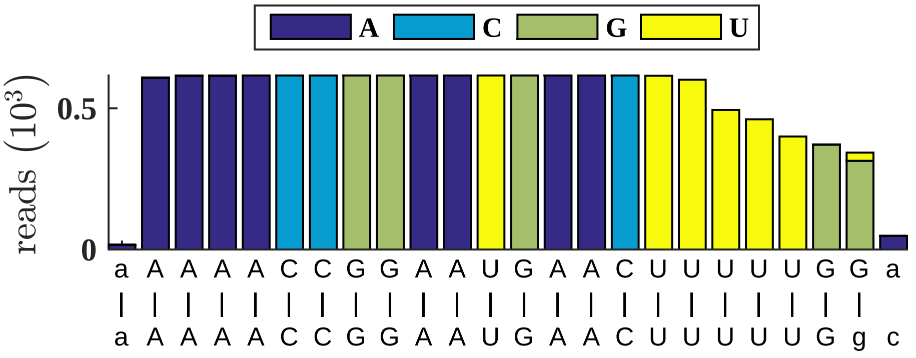

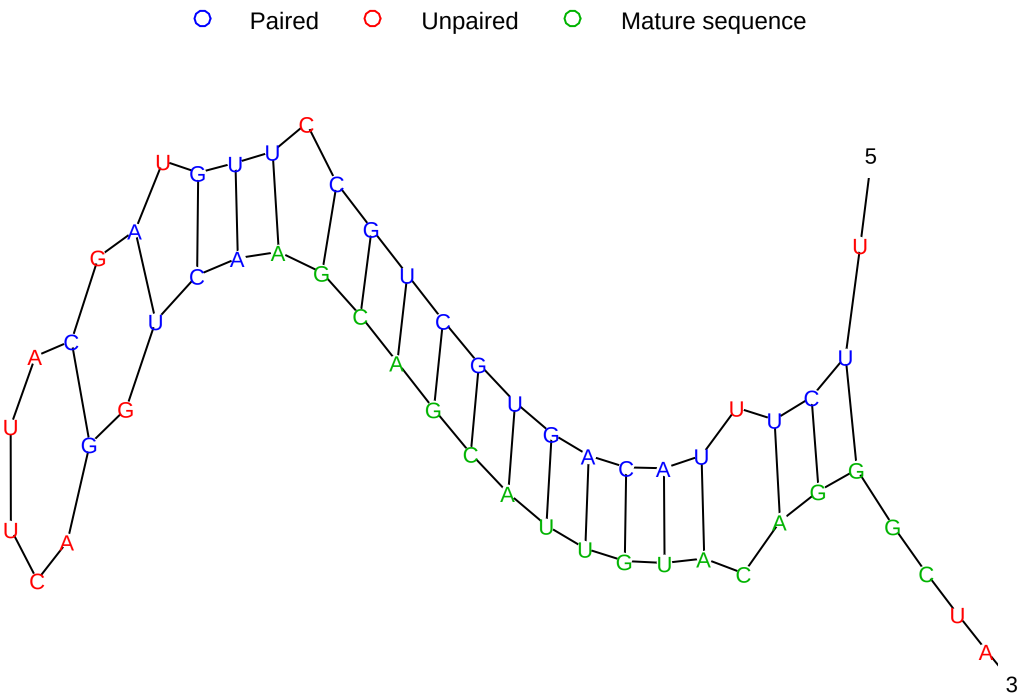

miRNA: bta-miR-103
Stem loop (UMD3.1): chr13:51742431-51742484
Mature (UMD3.1): chr13:51742433-51742450
Mature seq len: 18
Total raw counts (9 samples): 78378
Average raw counts: 8709
Strand: Reverse
Orientation: 3p
Minimum free energy: -20.00

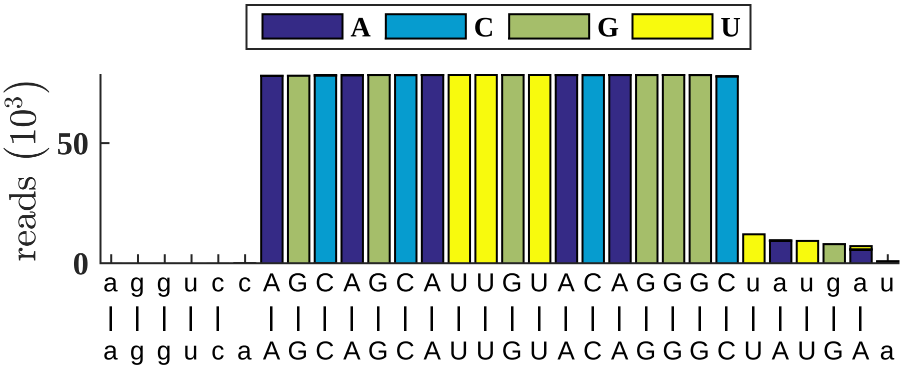

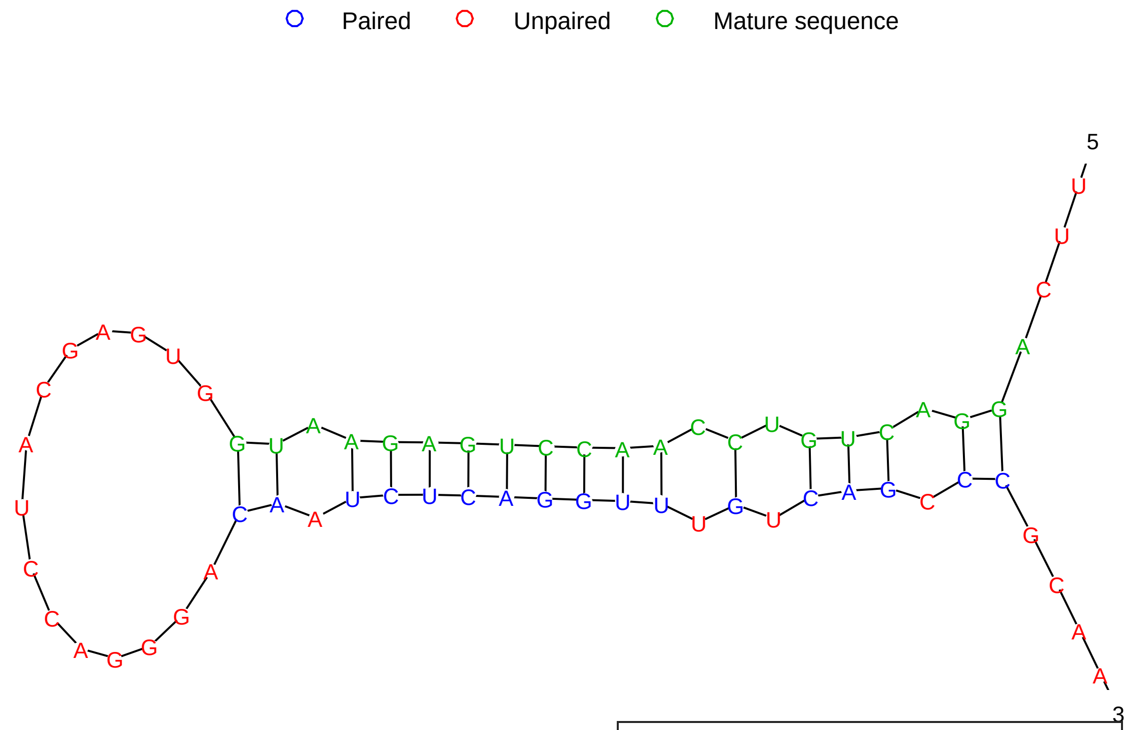

miRNA: bta-miR-1388-5p
Stem loop (UMD3.1): chr13:54375941-54376005
Mature (UMD3.1): chr13:54375944-54375965
Mature seq len: 22
Total raw counts (9 samples): 1493
Average raw counts: 166
Strand: Forward
Orientation: 5p
Minimum free energy: -21.10

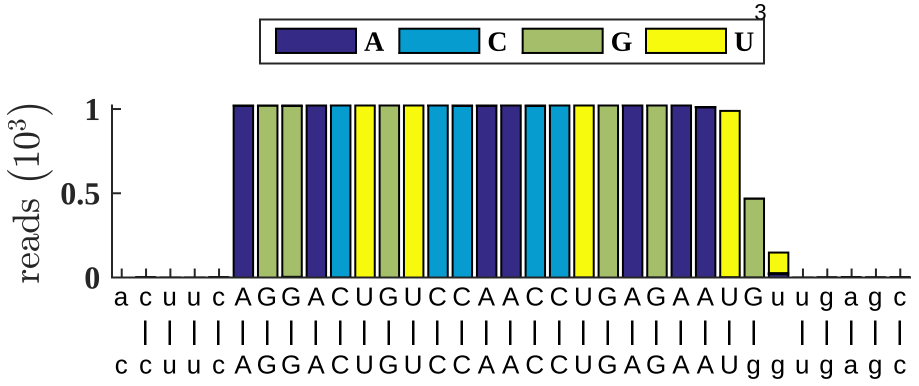

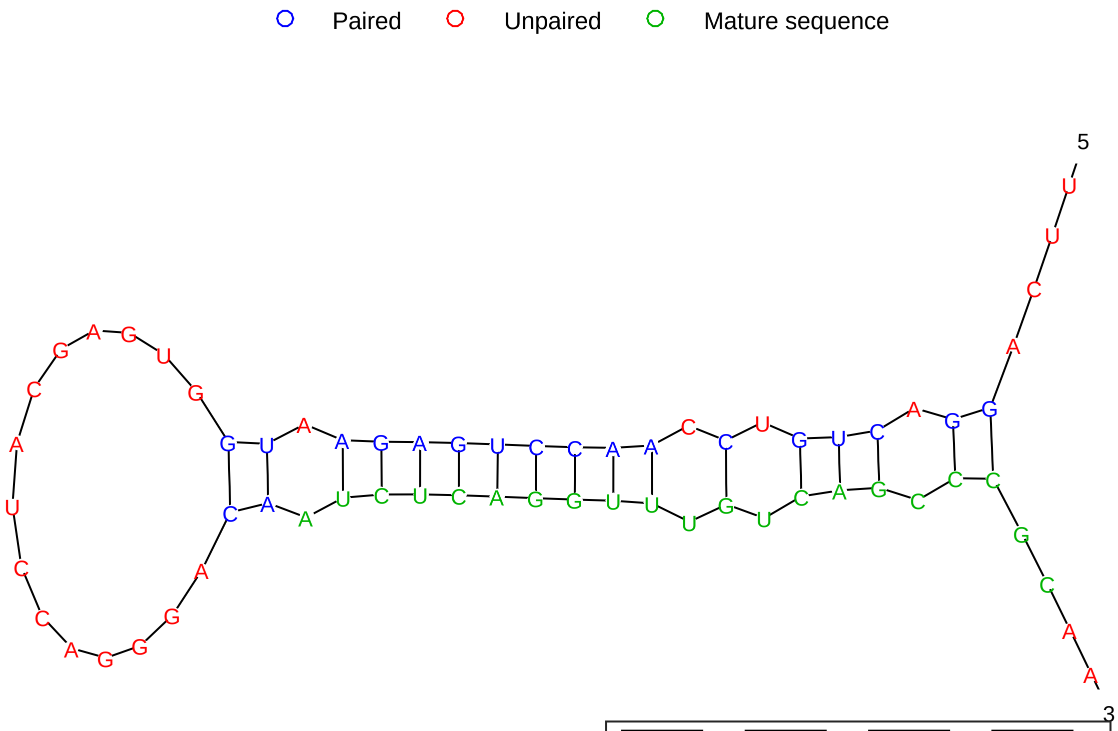

miRNA: bta-miR-1388-3p
Stem loop (UMD3.1): chr13:54375941-54376005
Mature (UMD3.1): chr13:54375983-54376003
Mature seq len: 21
Total raw counts (9 samples): 345
Average raw counts: 39
Strand: Forward
Orientation: 3p
Minimum free energy: -21.10

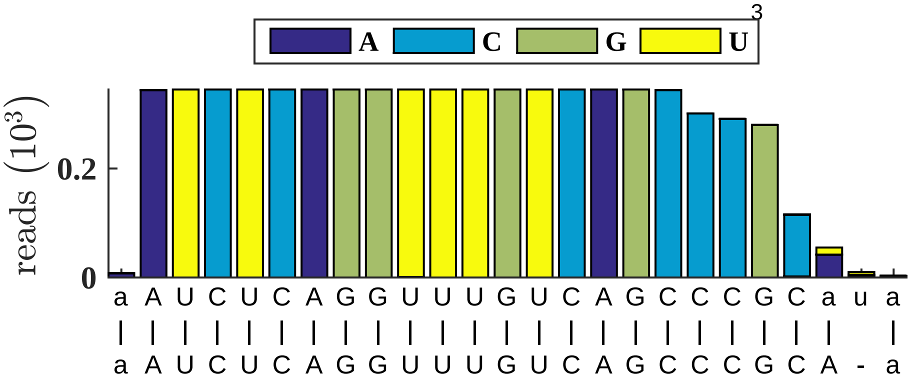

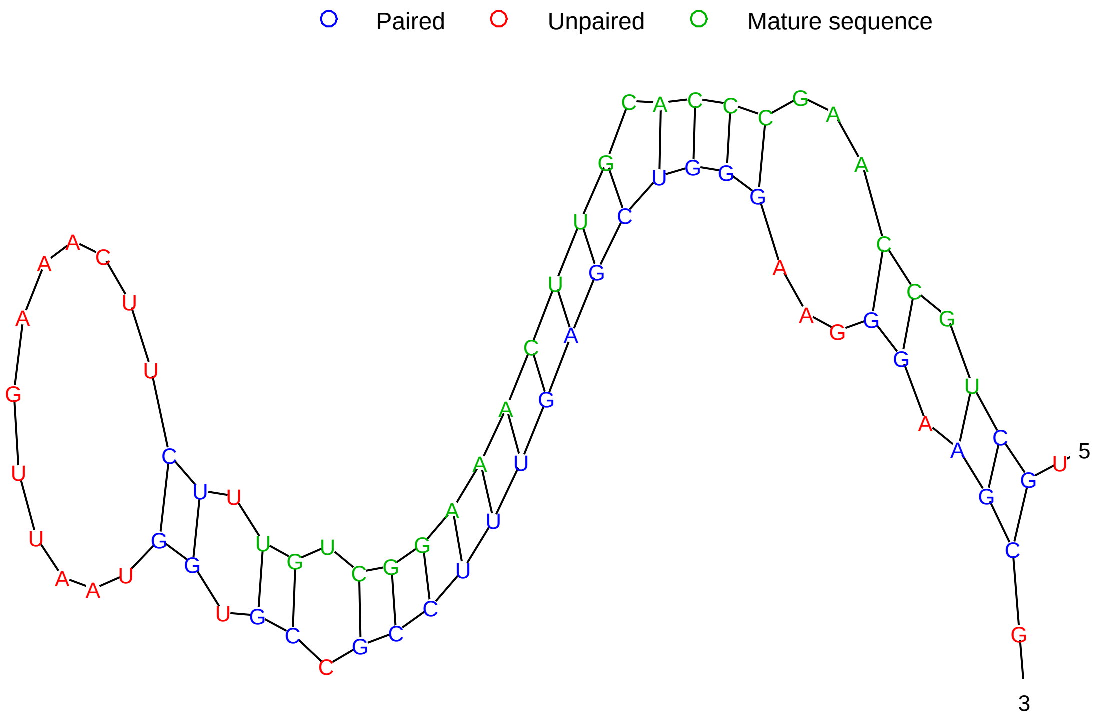

miRNA: bta-miR-6123
Stem loop (UMD3.1): chr13:58091682-58091754
Mature (UMD3.1): chr13:58091685-58091709
Mature seq len: 25
Total raw counts (9 samples): 453
Average raw counts: 51
Strand: Forward
Orientation: 5p
Minimum free energy: -31.20

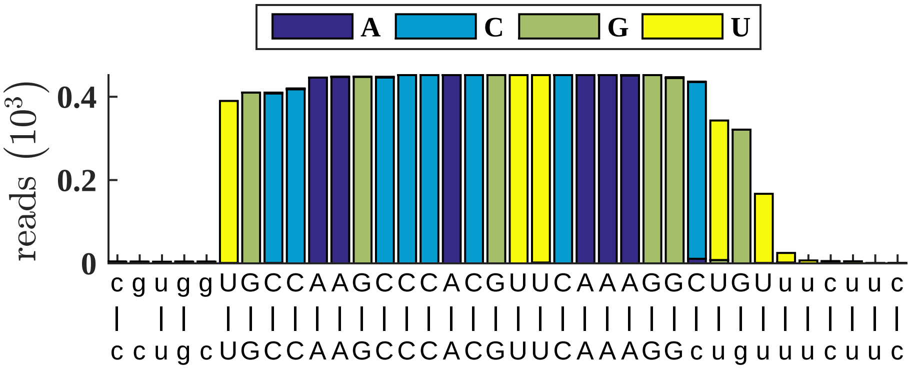

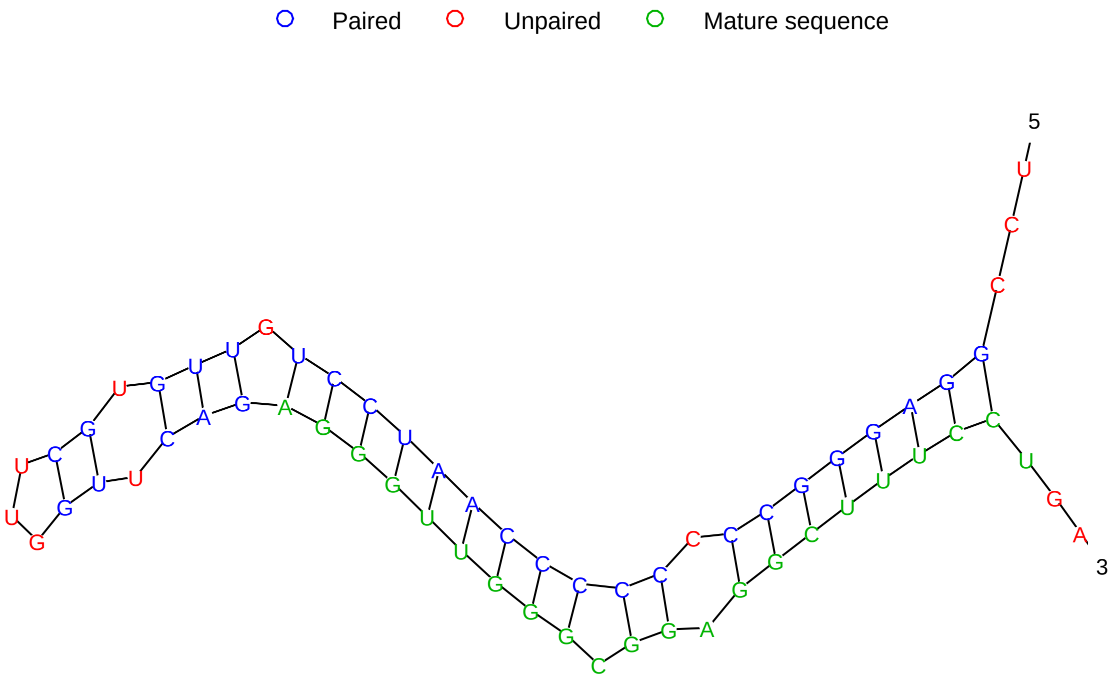

miRNA: bta-miR-296-3p
Stem loop (UMD3.1): chr13:58091944-58092006
Mature (UMD3.1): chr13:58091983-58092004
Mature seq len: 22
Total raw counts (9 samples): 15320
Average raw counts: 1703
Strand: Forward
Orientation: 3p
Minimum free energy: -34.00

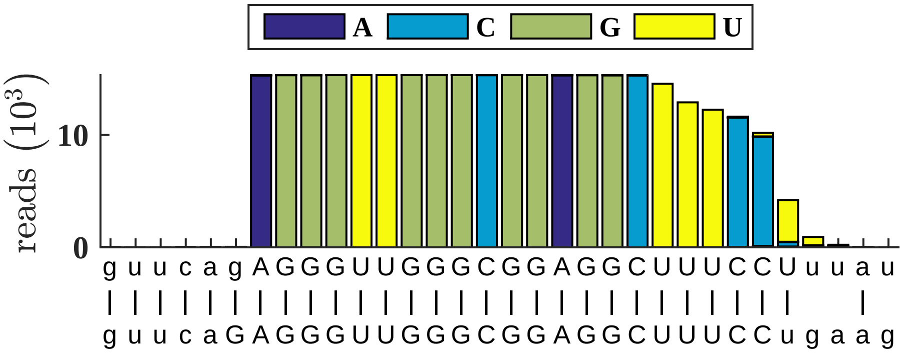

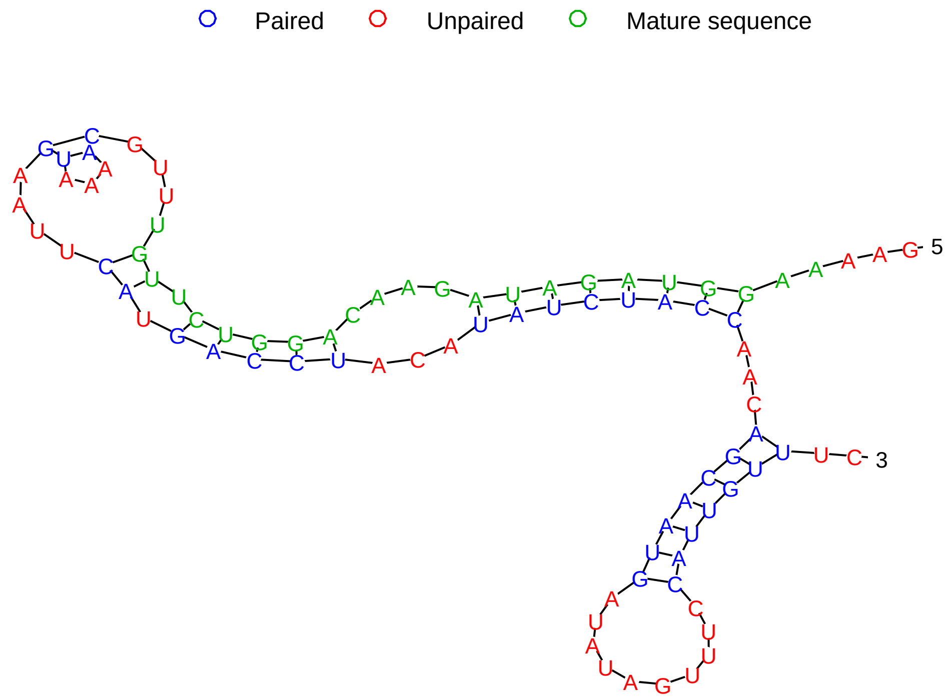

miRNA: bta-miR-1839
Stem loop (UMD3.1): chr14:1883827-1883914
Mature (UMD3.1): chr14:1883889-1883911
Mature seq len: 23
Total raw counts (9 samples): 27379
Average raw counts: 3043
Strand: Reverse
Orientation: 5p
Minimum free energy: -23.10

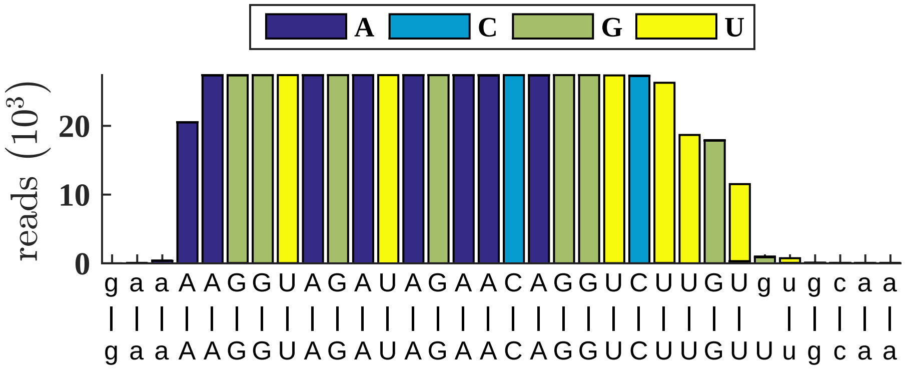

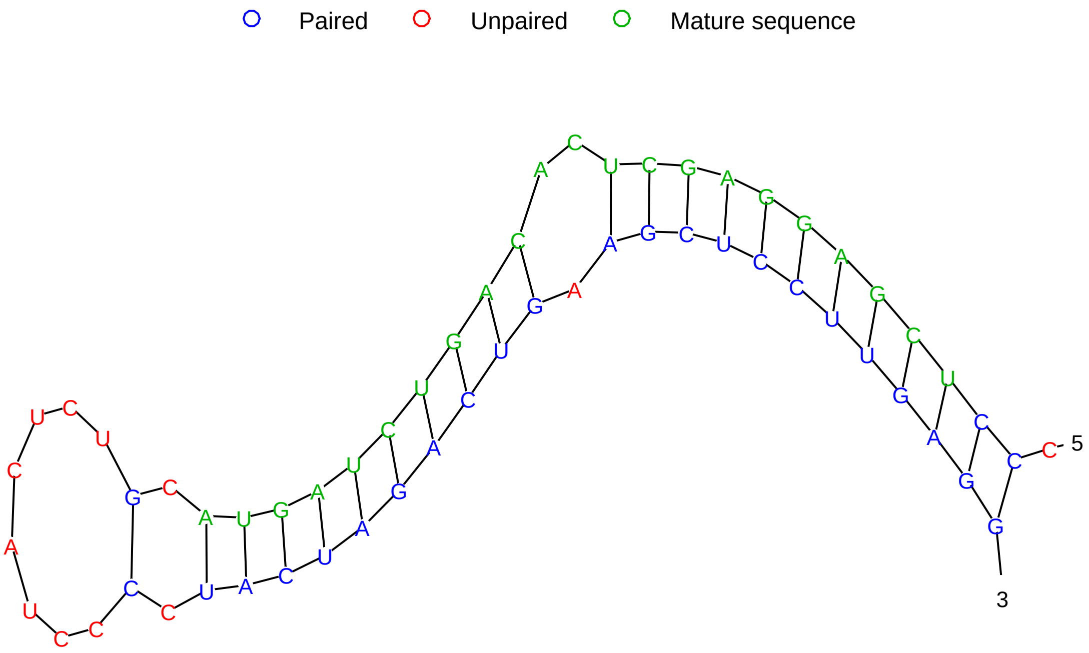

miRNA: bta-miR-151-5p
Stem loop (UMD3.1): chr14:4030323-4030382
Mature (UMD3.1): chr14:4030326-4030347
Mature seq len: 22
Total raw counts (9 samples): 34244
Average raw counts: 3805
Strand: Forward
Orientation: 5p
Minimum free energy: -35.30

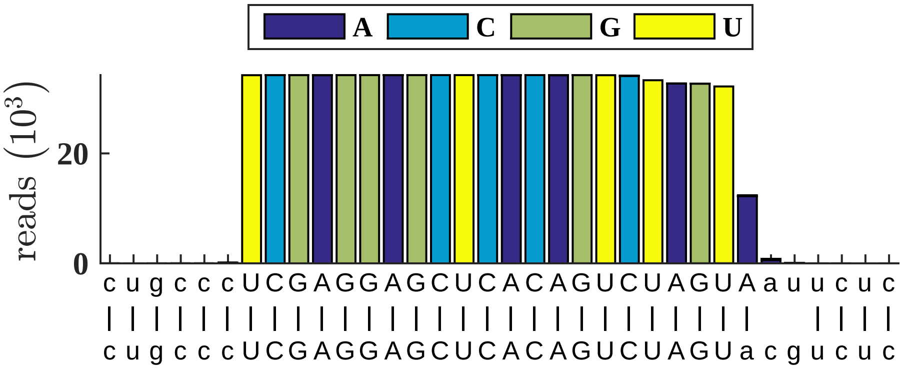

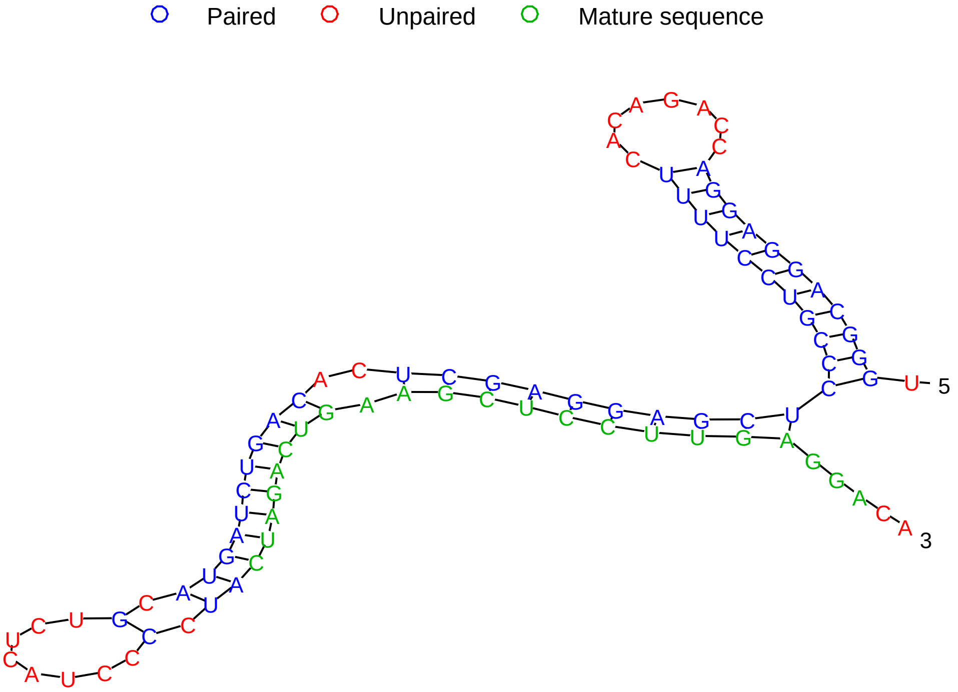

miRNA: bta-miR-151-3p
Stem loop (UMD3.1): chr14:4030295-4030385
Mature (UMD3.1): chr14:4030362-4030383
Mature seq len: 22
Total raw counts (9 samples): 76056
Average raw counts: 8451
Strand: Forward
Orientation: 3p
Minimum free energy: -48.30

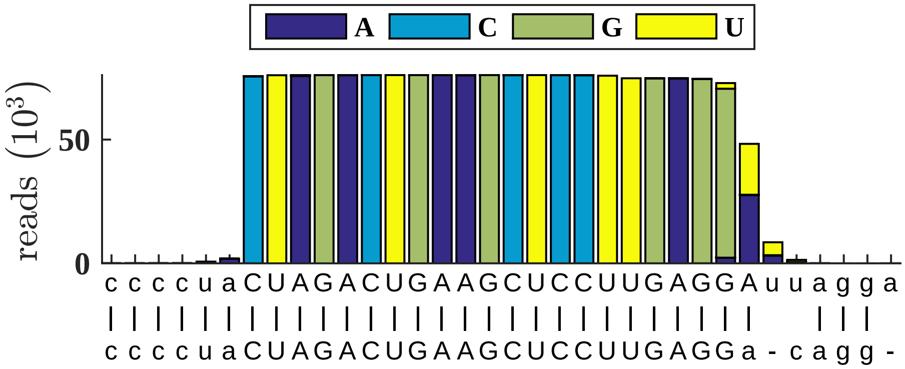

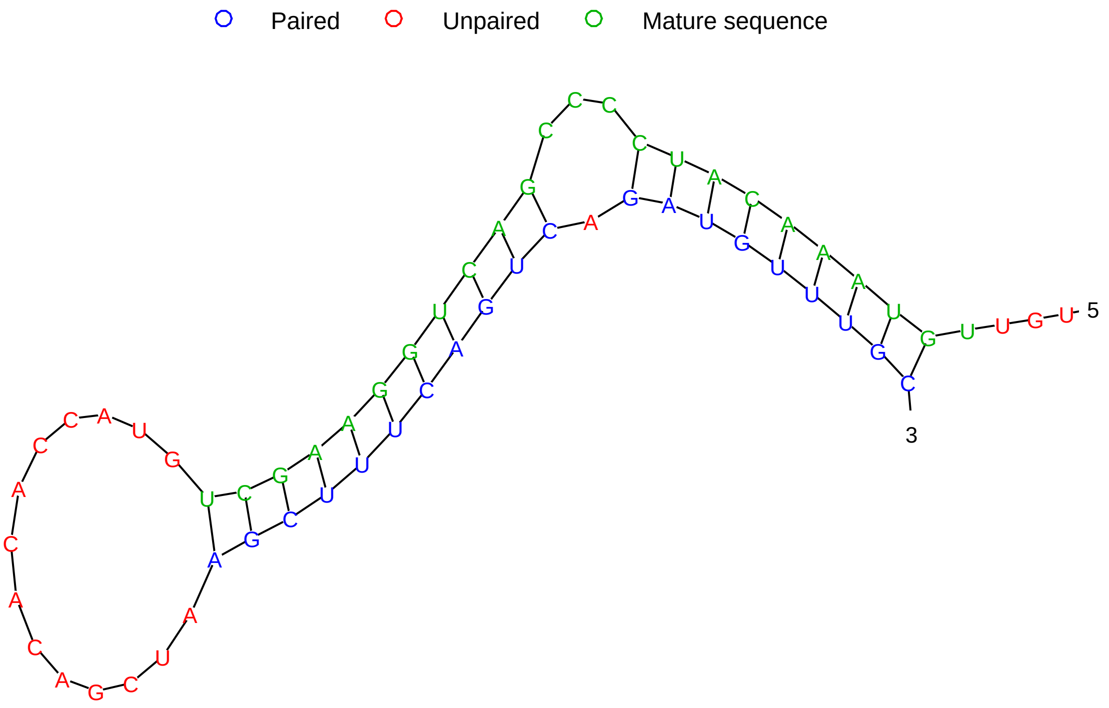

miRNA: bta-miR-30d
Stem loop (UMD3.1): chr14:8080294-8080355
Mature (UMD3.1): chr14:8080297-8080320
Mature seq len: 24
Total raw counts (9 samples): 168088
Average raw counts: 18677
Strand: Forward
Orientation: 5p
Minimum free energy: -25.50

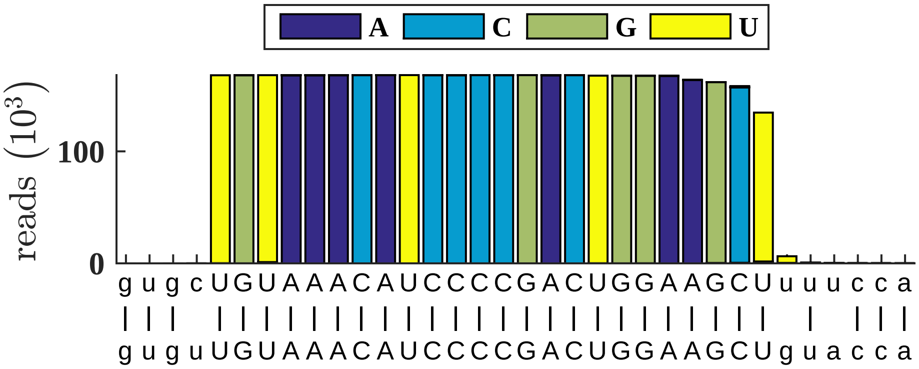

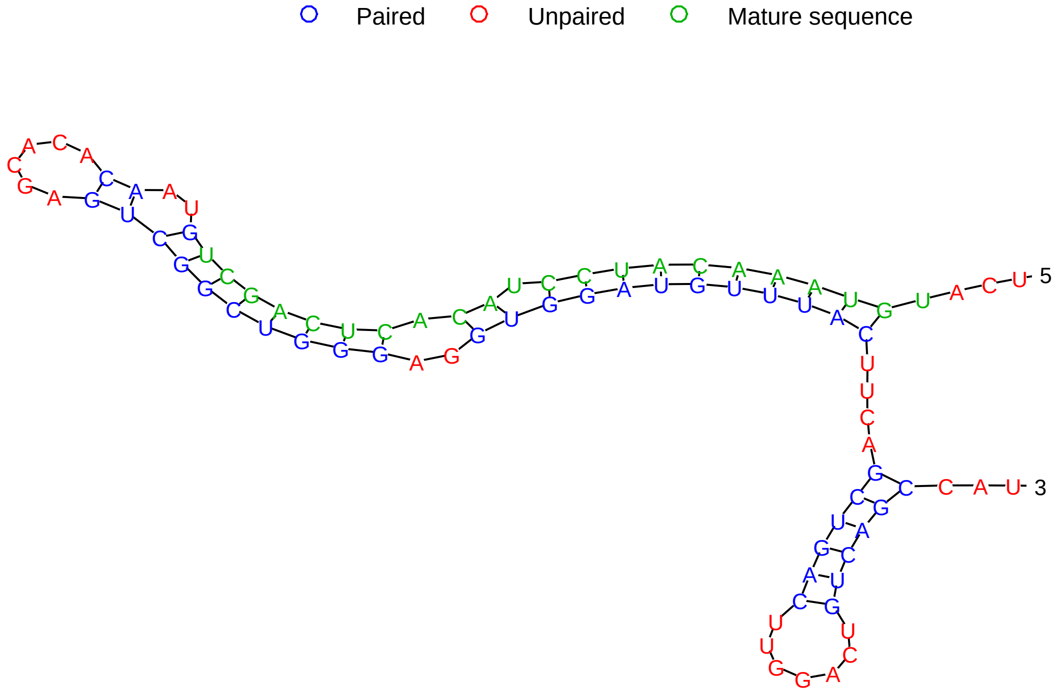

miRNA: bta-miR-30b-5p
Stem loop (UMD3.1): chr14:8084734-8084819
Mature (UMD3.1): chr14:8084737-8084758
Mature seq len: 22
Total raw counts (9 samples): 8510
Average raw counts: 946
Strand: Forward
Orientation: 5p
Minimum free energy: -36.00

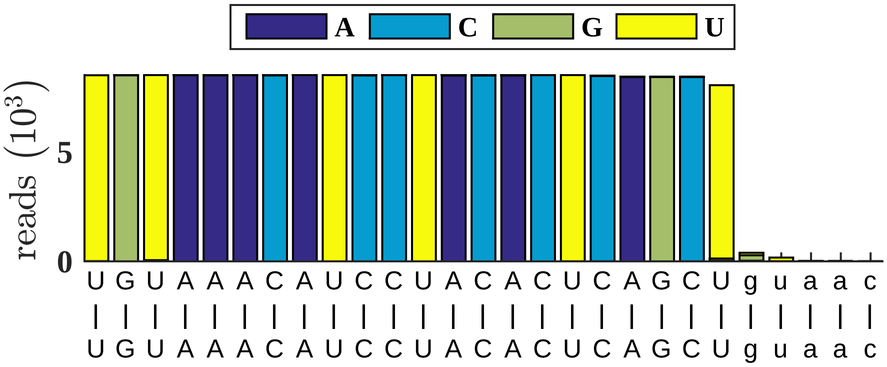

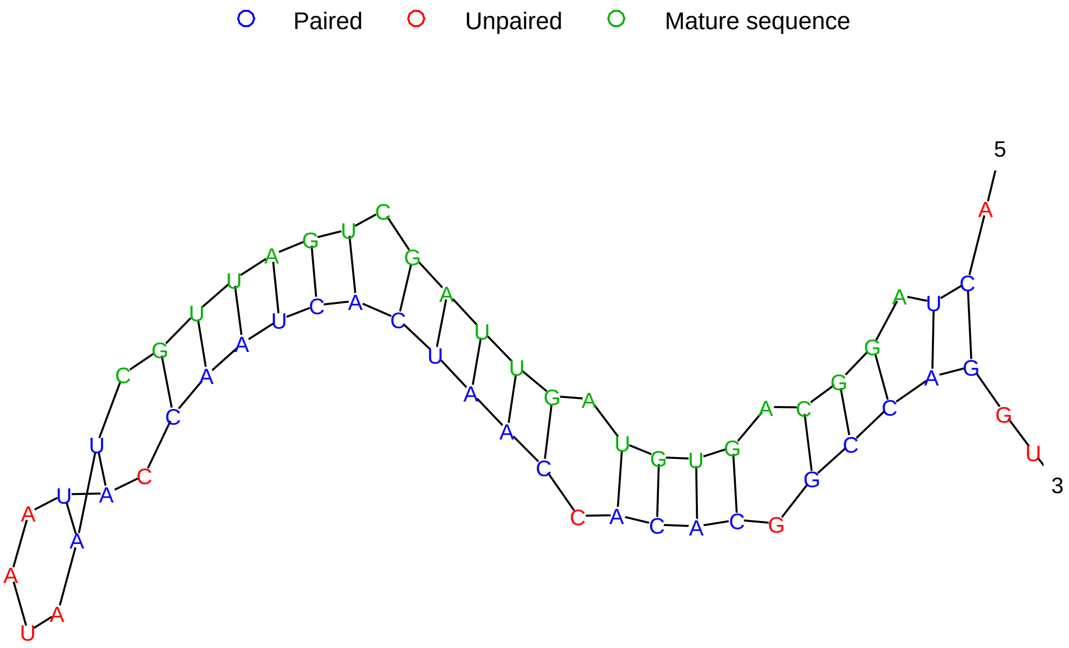

miRNA: bta-miR-34c
Stem loop (UMD3.1): chr15:22135435-22135493
Mature (UMD3.1): chr15:22135438-22135460
Mature seq len: 23
Total raw counts (9 samples): 6393
Average raw counts: 711
Strand: Forward
Orientation: 5p
Minimum free energy: -21.80

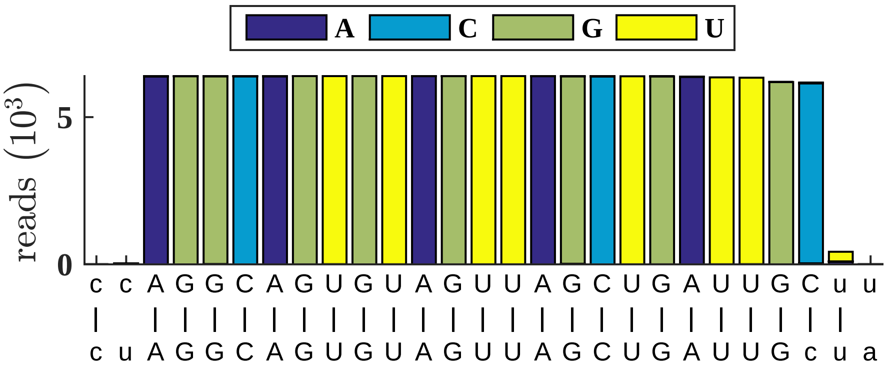

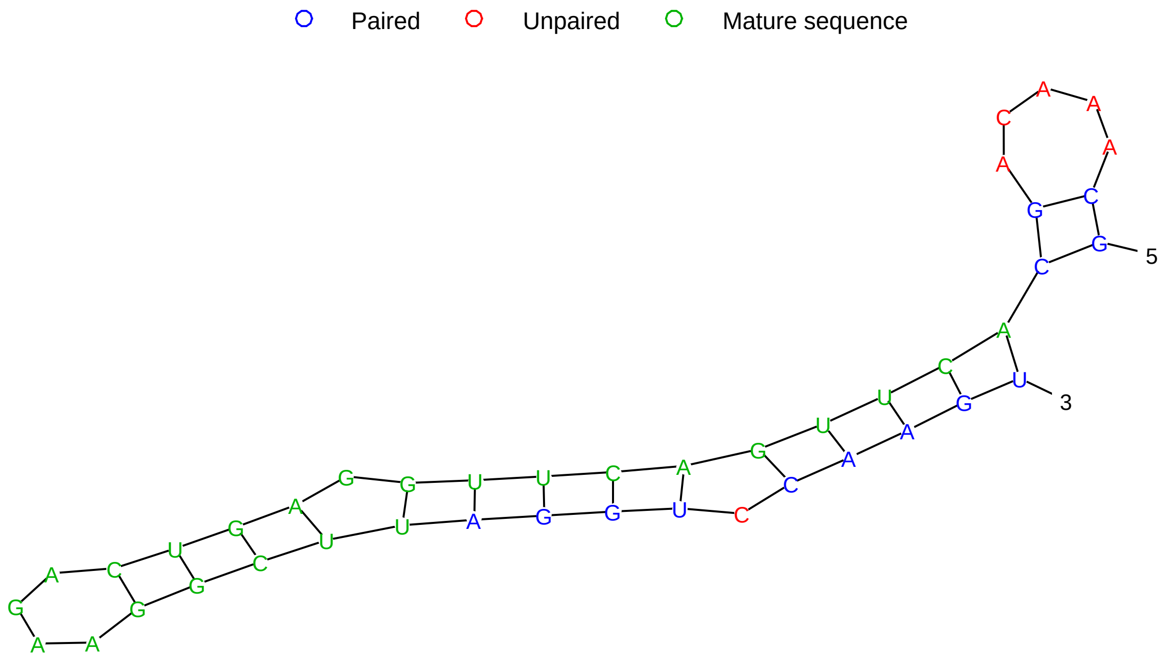

miRNA: bta-miR-378b
Stem loop (UMD3.1): chr15:30727354-30727396
Mature (UMD3.1): chr15:30727363-30727386
Mature seq len: 24
Total raw counts (9 samples): 481
Average raw counts: 54
Strand: Forward
Orientation: 3p
Minimum free energy: -12.00

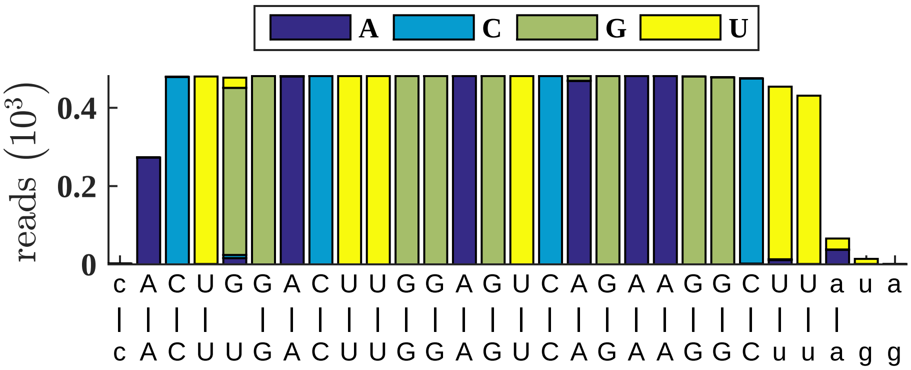

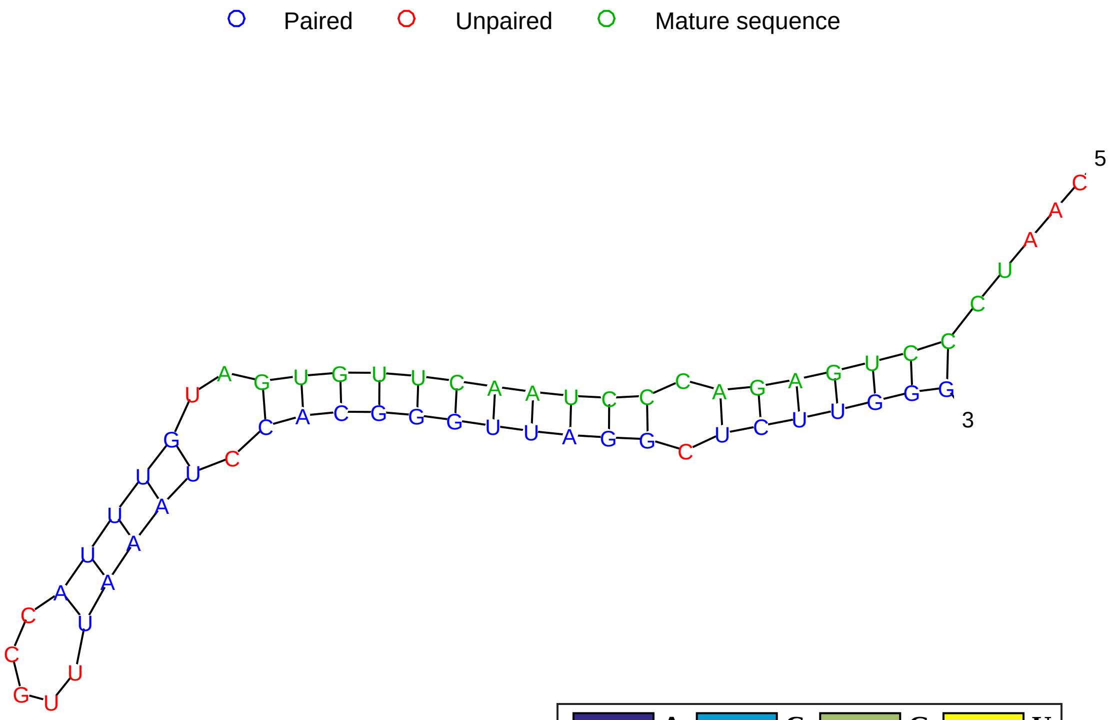

miRNA: bta-miR-125b
Stem loop (UMD3.1): chr15:33298831-33298891
Mature (UMD3.1): chr15:33298867-33298888
Mature seq len: 22
Total raw counts (9 samples): 5041
Average raw counts: 561
Strand: Reverse
Orientation: 5p
Minimum free energy: -24.10

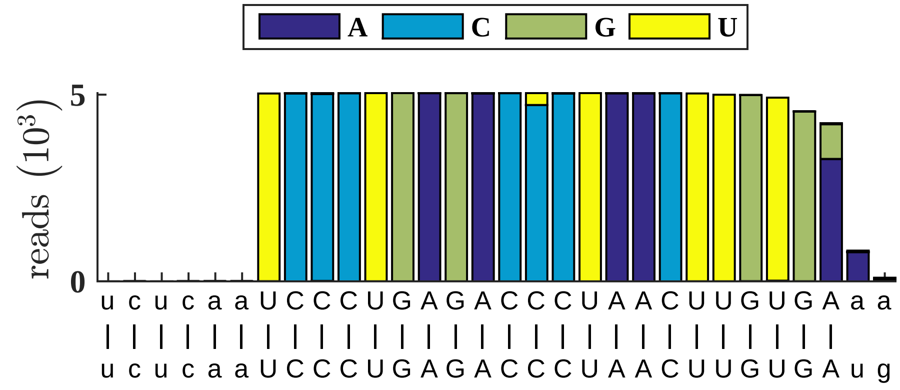

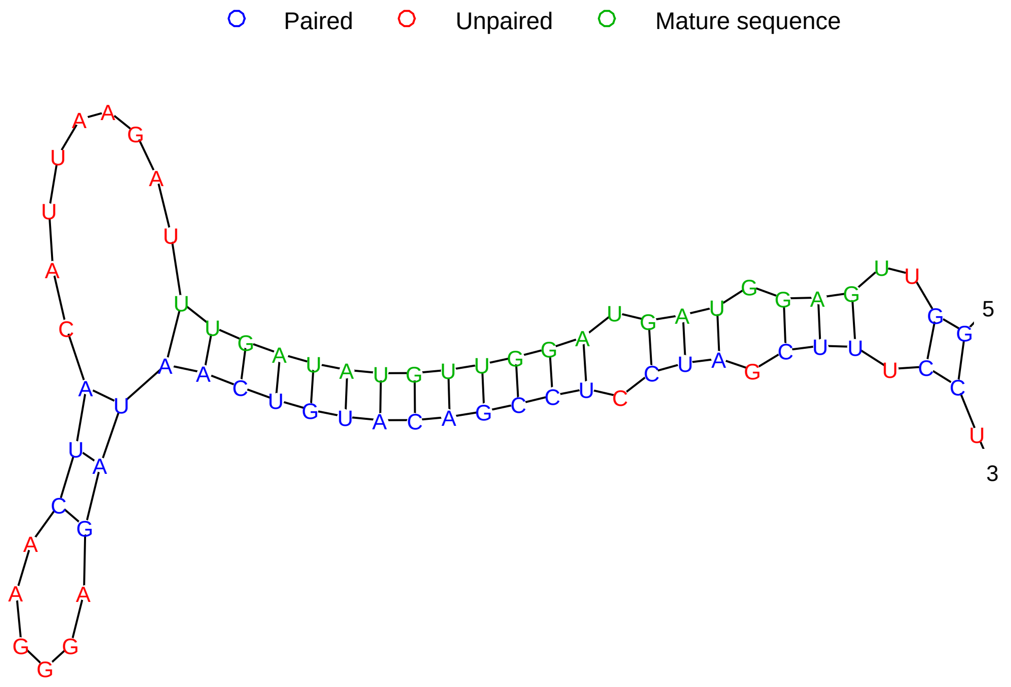

miRNA: bta-let-7a-5p
Stem loop (UMD3.1): chr15:33347569-33347639
Mature (UMD3.1): chr15:33347615-33347636
Mature seq len: 22
Total raw counts (9 samples): 76739
Average raw counts: 8527
Strand: Reverse
Orientation: 5p
Minimum free energy: -24.80

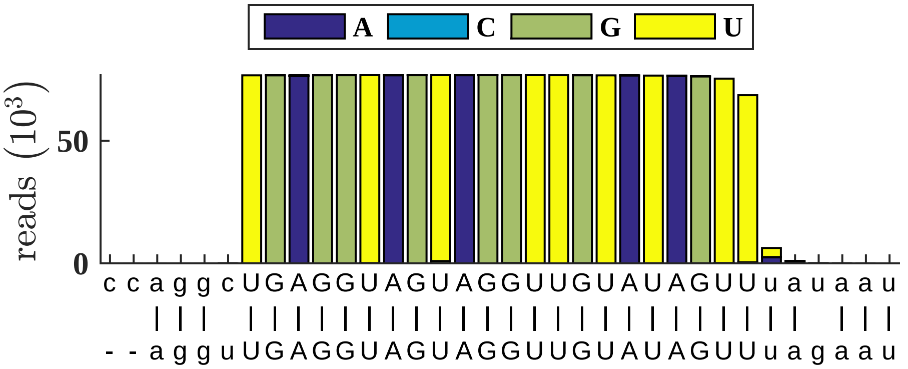

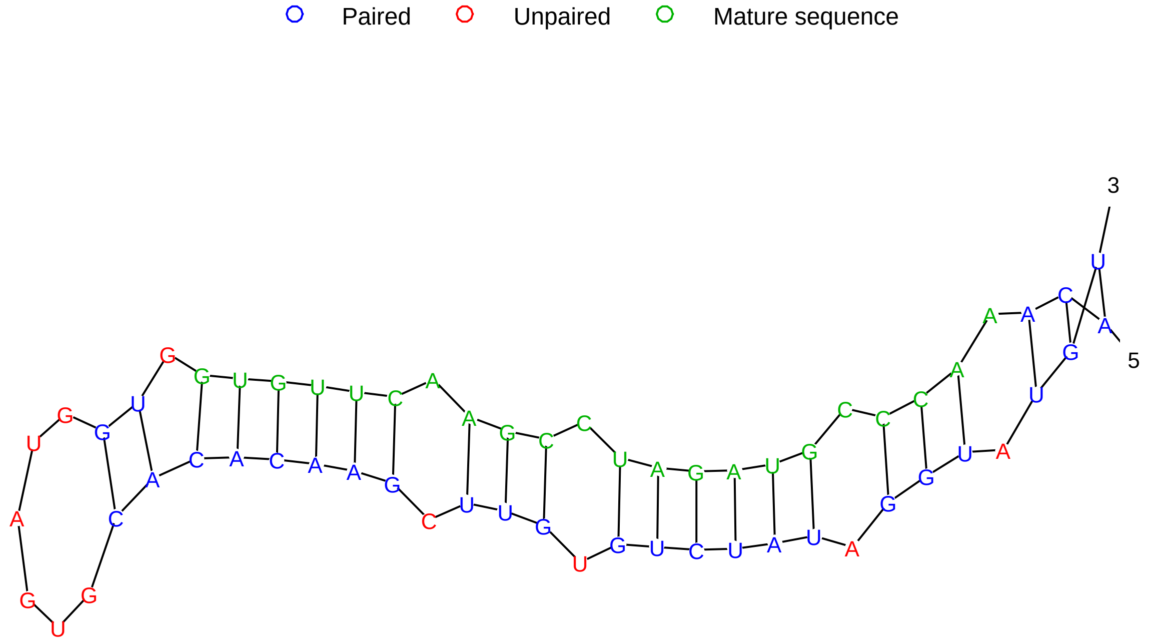

miRNA: bta-miR-100
Stem loop (UMD3.1): chr15:33353401-33353461
Mature (UMD3.1): chr15:33353437-33353458
Mature seq len: 22
Total raw counts (9 samples): 2478
Average raw counts: 276
Strand: Reverse
Orientation: 5p
Minimum free energy: -21.80

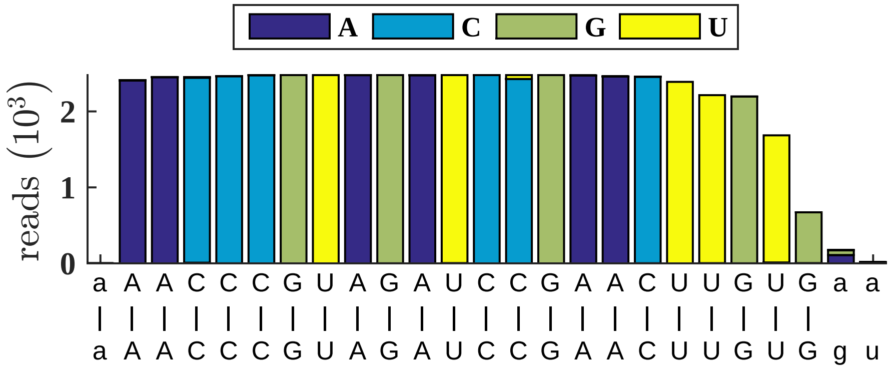

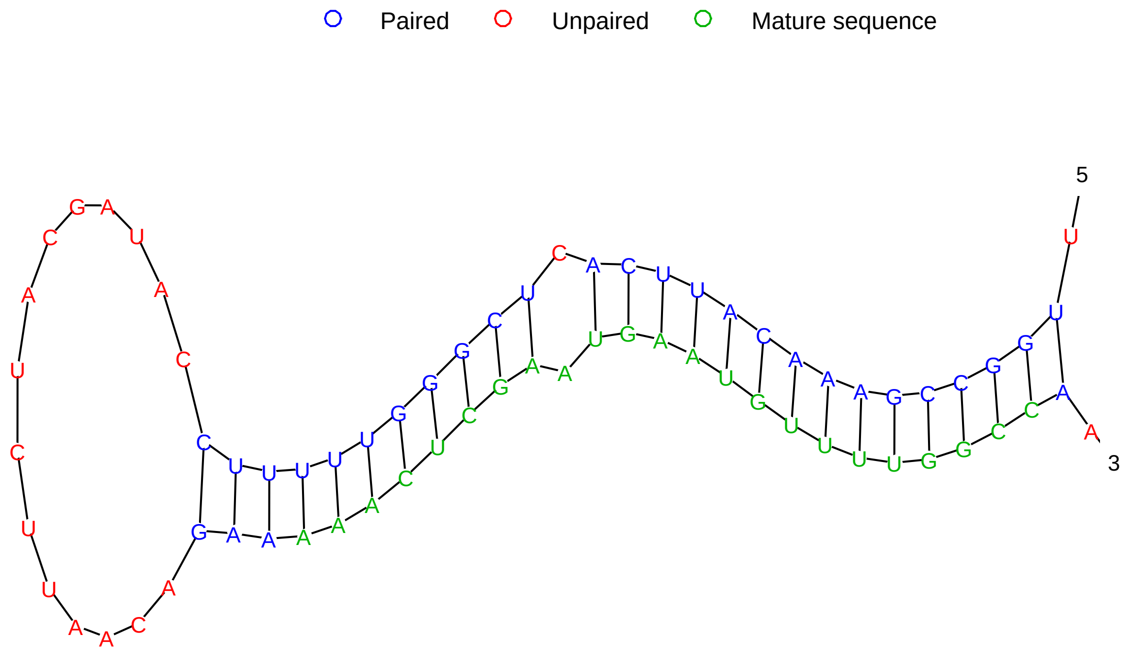

miRNA: bta-miR-2284t-3p
Stem loop (UMD3.1): chr15:39162246-39162316
Mature (UMD3.1): chr15:39162248-39162270
Mature seq len: 23
Total raw counts (9 samples): 537
Average raw counts: 60
Strand: Reverse
Orientation: 3p
Minimum free energy: -36.70

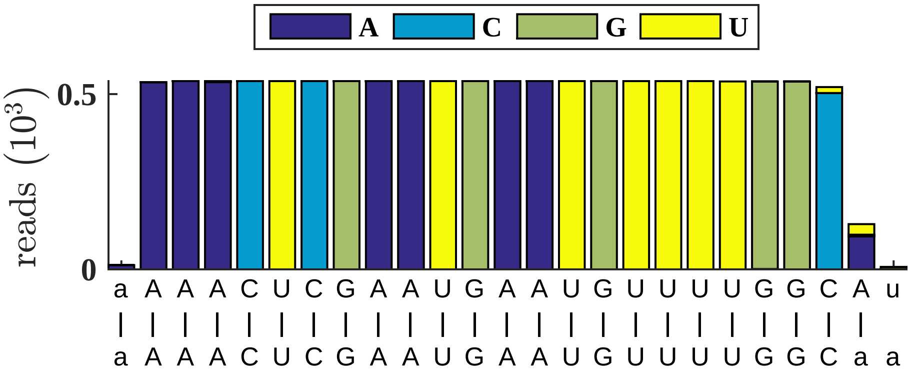

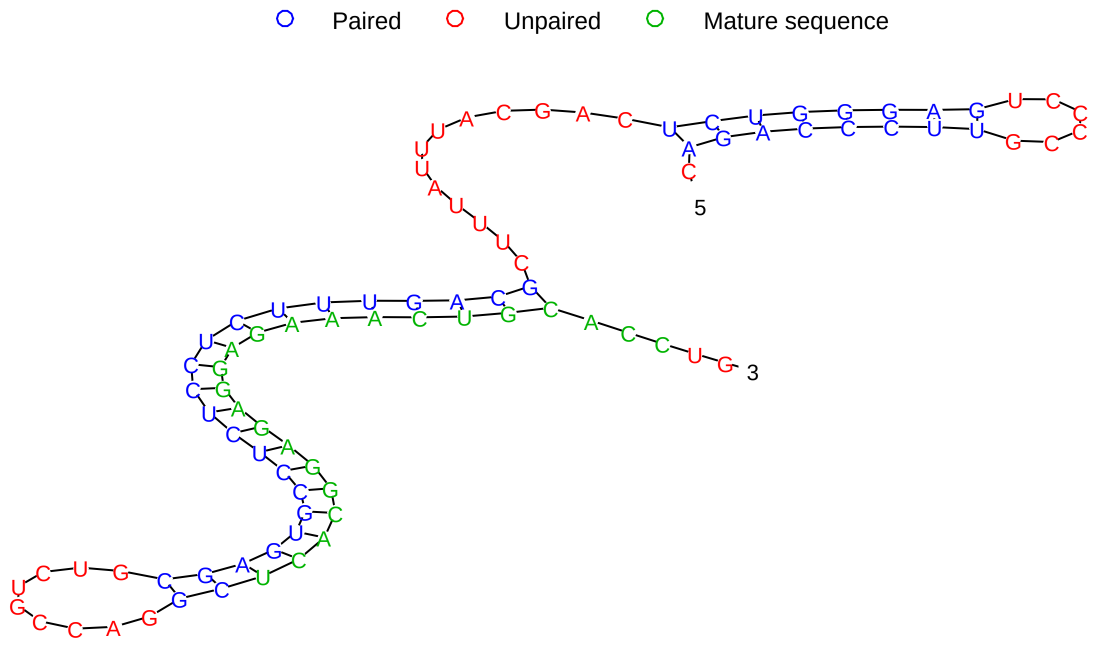

miRNA: bta-miR-6518
Stem loop (UMD3.1): chr15:42911482-42911576
Mature (UMD3.1): chr15:42911552-42911574
Mature seq len: 23
Total raw counts (9 samples): 2041
Average raw counts: 227
Strand: Forward
Orientation: 3p
Minimum free energy: -57.90

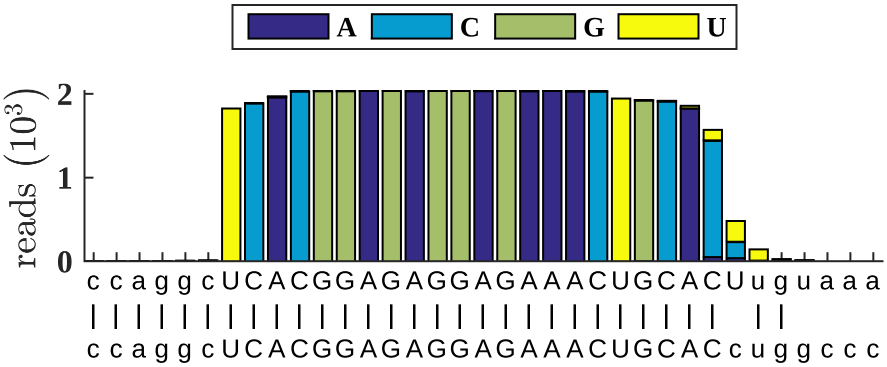

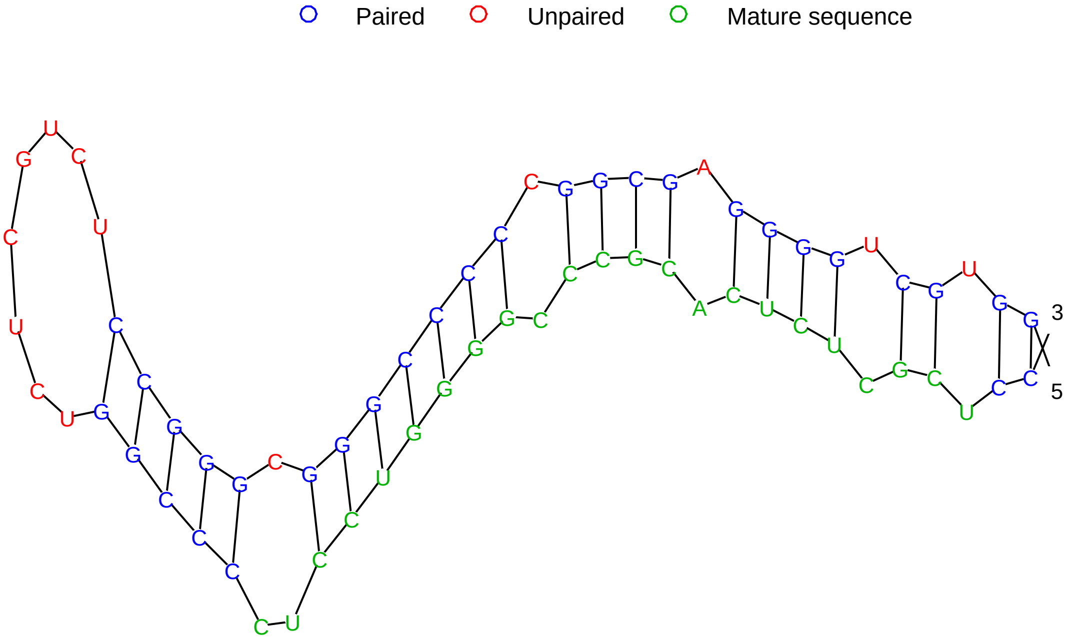

miRNA: bta-miR-1343-3p
Stem loop (UMD3.1): chr15:66264854-66264920
Mature (UMD3.1): chr15:66264896-66264918
Mature seq len: 23
Total raw counts (9 samples): 1265
Average raw counts: 141
Strand: Forward
Orientation: 3p
Minimum free energy: -43.30

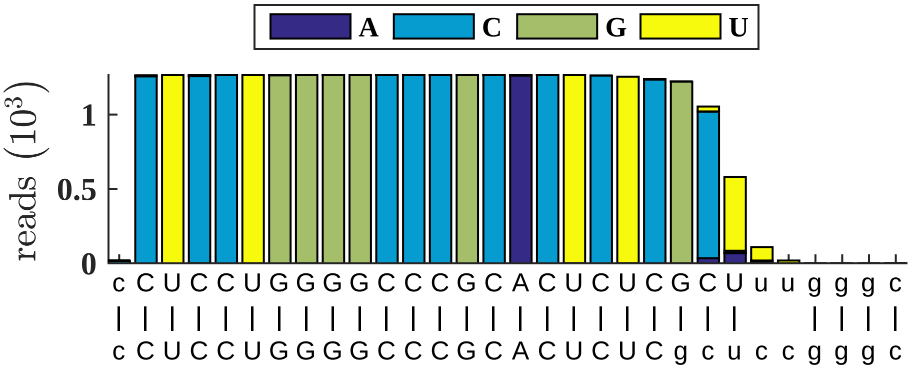

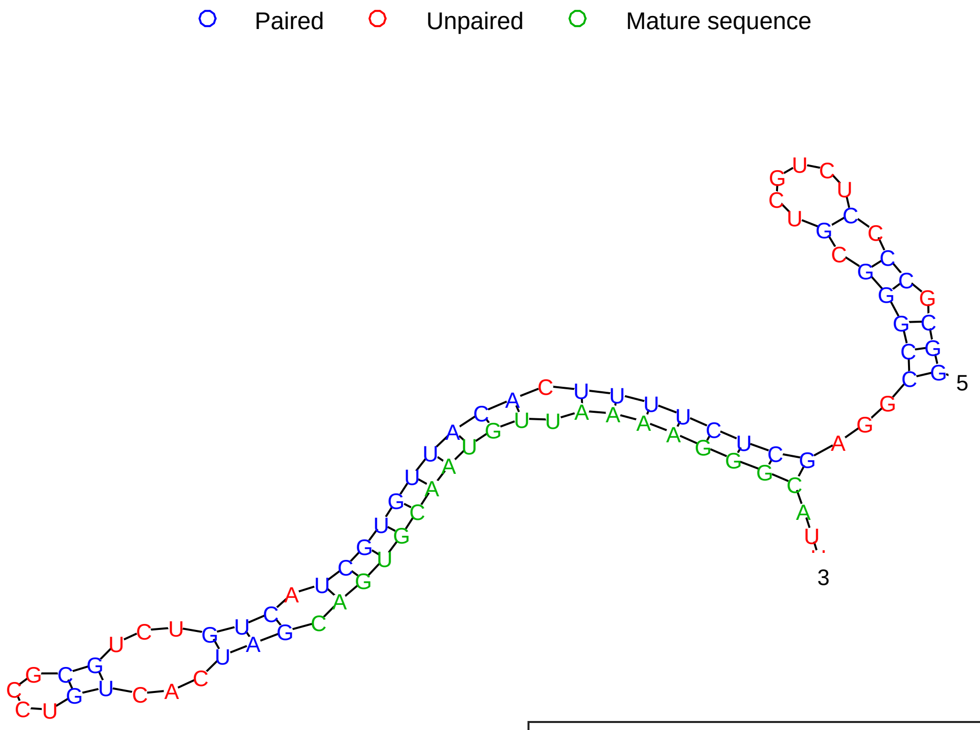

miRNA: bta-miR-130a
Stem loop (UMD3.1): chr15:82197701-82197787
Mature (UMD3.1): chr15:82197765-82197785
Mature seq len: 21
Total raw counts (9 samples): 58124
Average raw counts: 6459
Strand: Forward
Orientation: 3p
Minimum free energy: -30.90

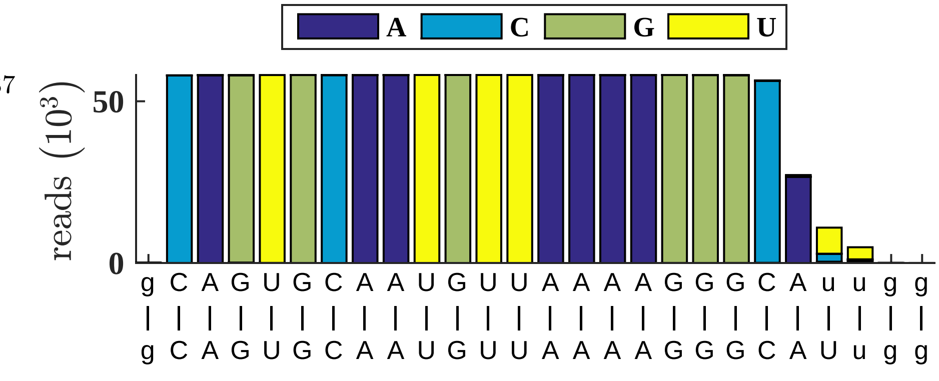

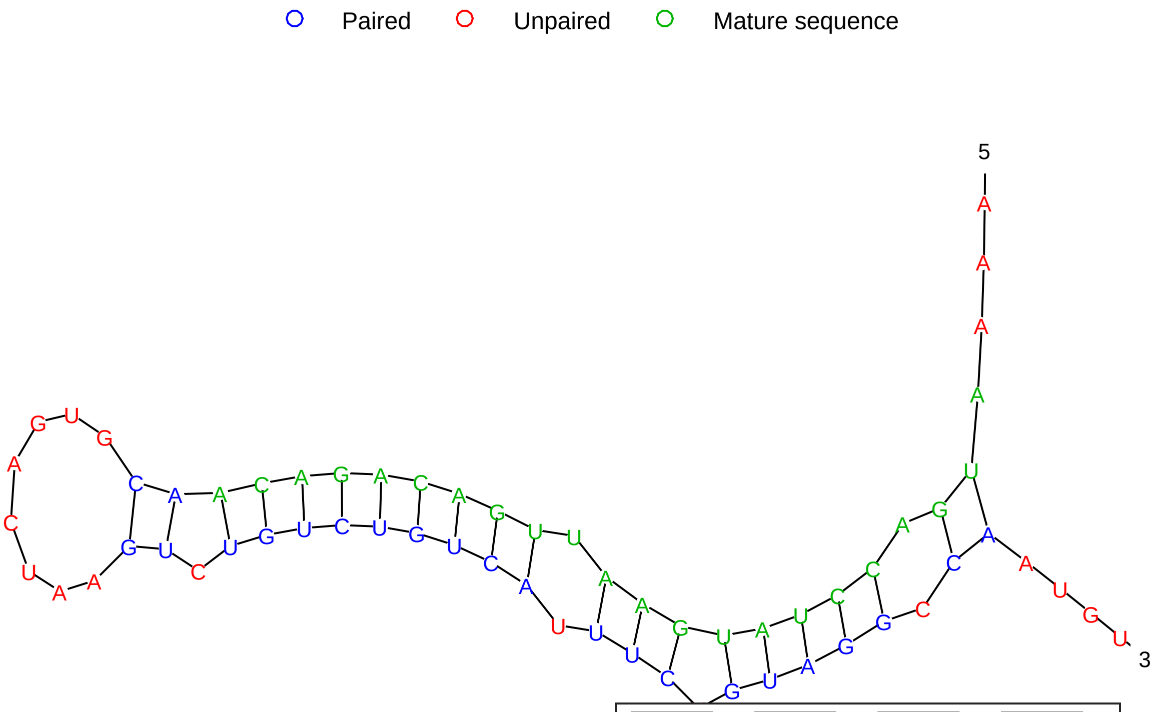

miRNA: bta-miR-215
Stem loop (UMD3.1): chr16:24305744-24305807
Mature (UMD3.1): chr16:24305783-24305804
Mature seq len: 22
Total raw counts (9 samples): 5053
Average raw counts: 562
Strand: Reverse
Orientation: 5p
Minimum free energy: -23.40

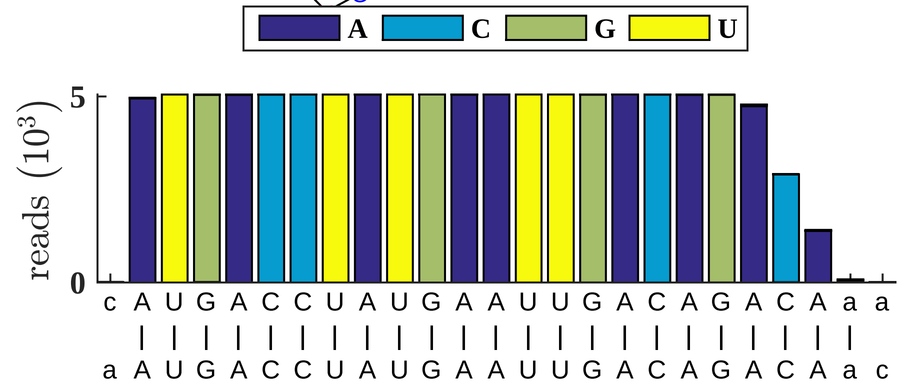

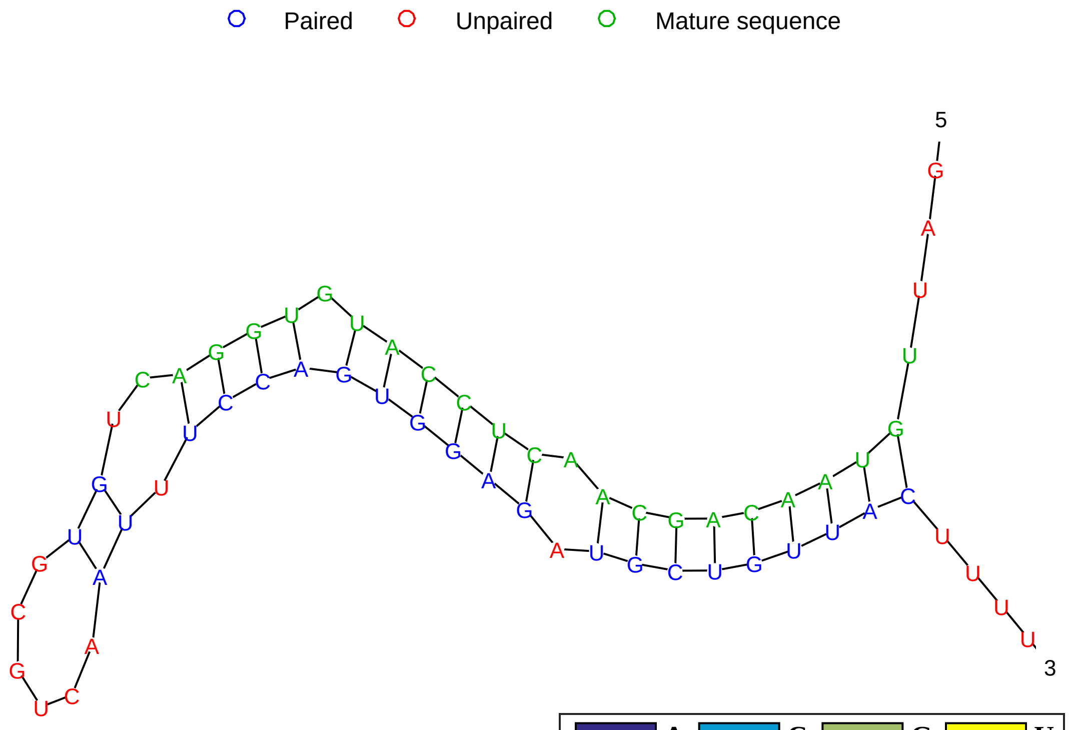

miRNA: bta-miR-194
Stem loop (UMD3.1): chr16:24306042-24306103
Mature (UMD3.1): chr16:24306078-24306100
Mature seq len: 23
Total raw counts (9 samples): 575
Average raw counts: 64
Strand: Reverse
Orientation: 5p
Minimum free energy: -26.70

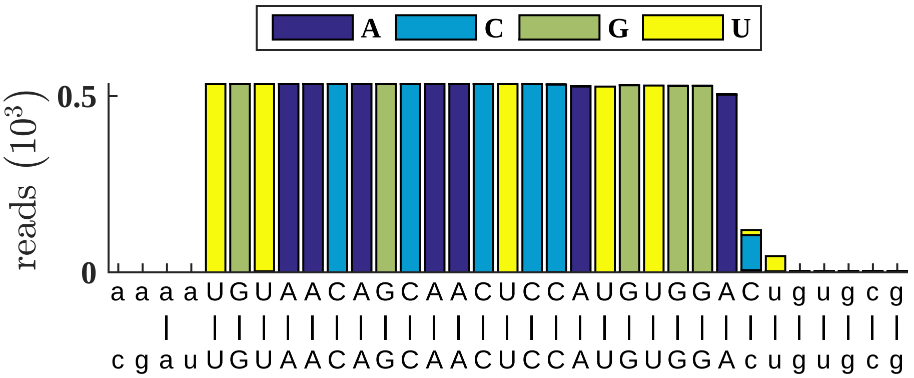

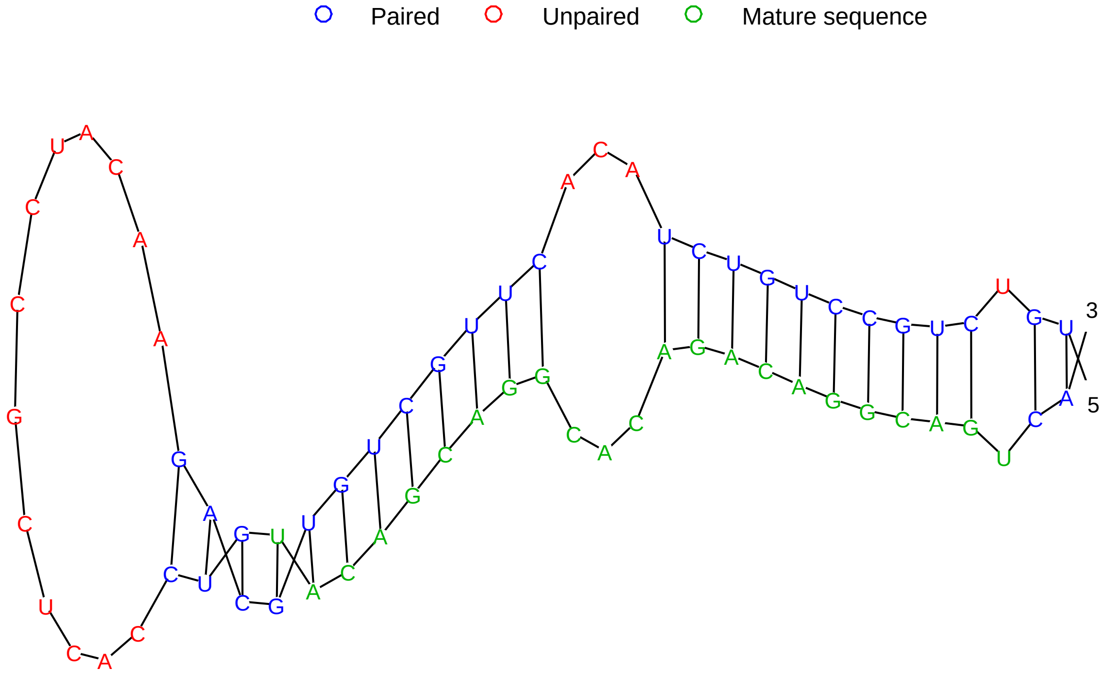

miRNA: bta-miR-214
Stem loop (UMD3.1): chr16:40485823-40485891
Mature (UMD3.1): chr16:40485825-40485847
Mature seq len: 23
Total raw counts (9 samples): 657
Average raw counts: 73
Strand: Reverse
Orientation: 3p
Minimum free energy: -38.30

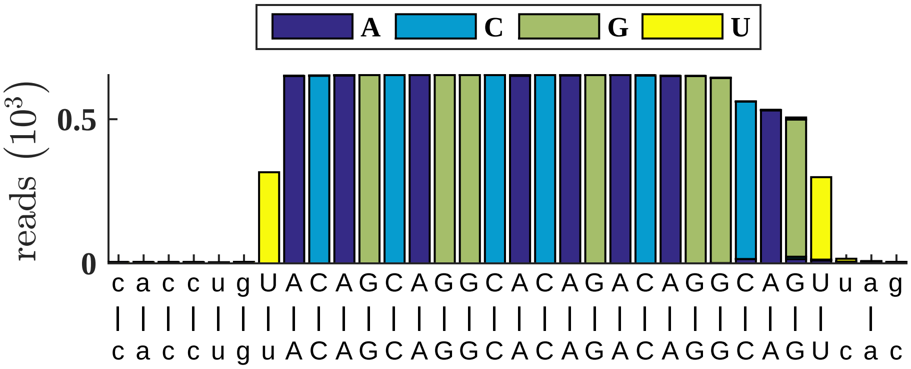

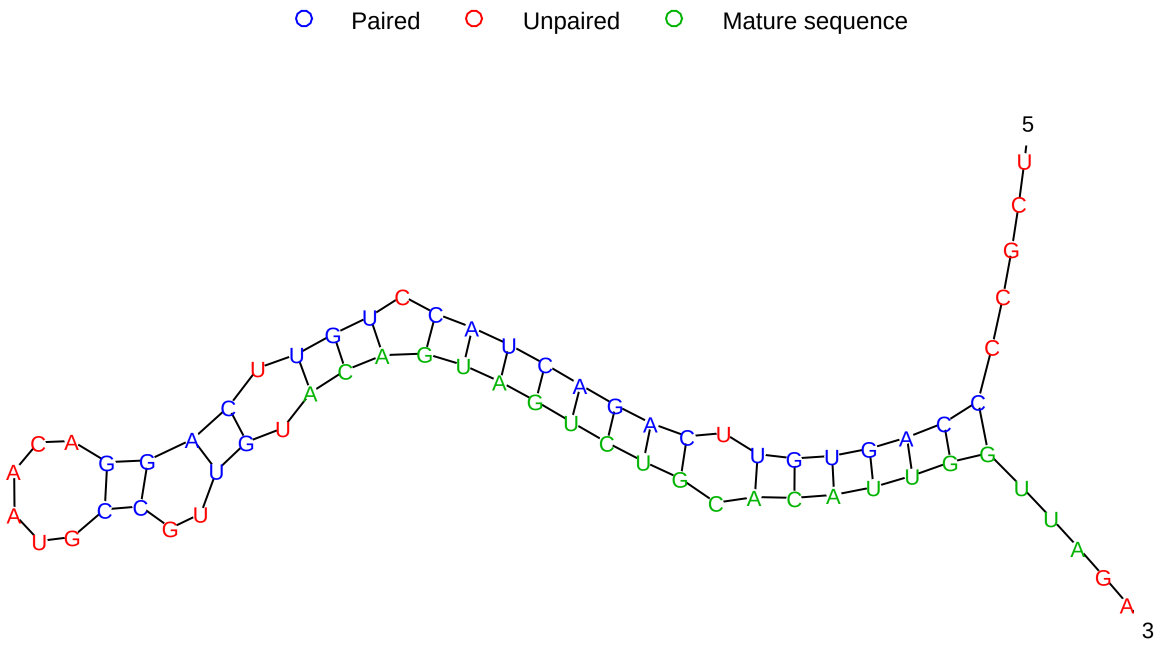

miRNA: bta-miR-199a-3p
Stem loop (UMD3.1): chr16:40491613-40491679
Mature (UMD3.1): chr16:40491615-40491636
Mature seq len: 22
Total raw counts (9 samples): 6261
Average raw counts: 696
Strand: Reverse
Orientation: 3p
Minimum free energy: -25.30

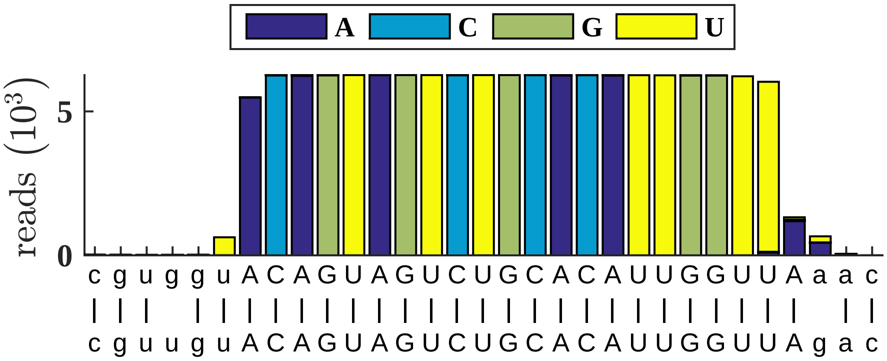

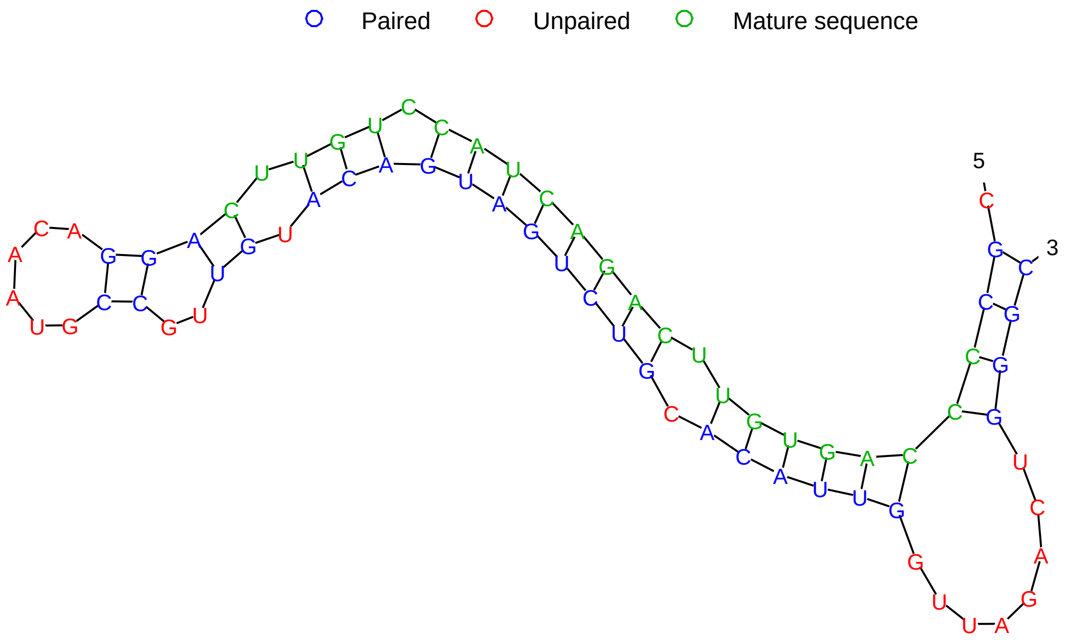

miRNA: bta-miR-199a-5p
Stem loop (UMD3.1): chr16:40491607-40491678
Mature (UMD3.1): chr16:40491653-40491675
Mature seq len: 23
Total raw counts (9 samples): 747
Average raw counts: 83
Strand: Reverse
Orientation: 5p
Minimum free energy: -26.60

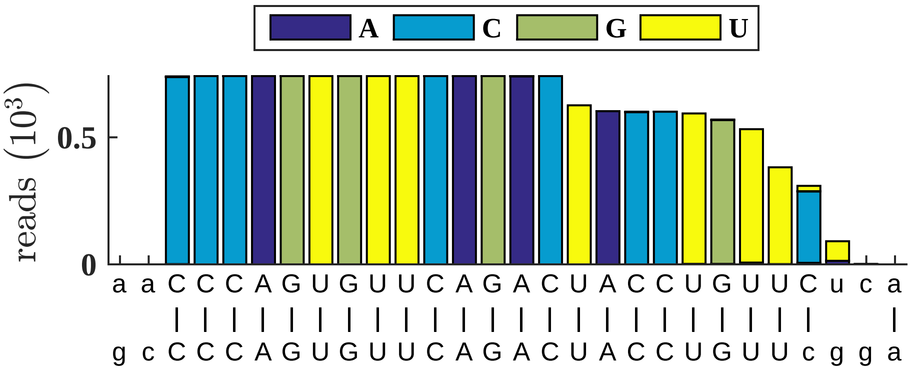

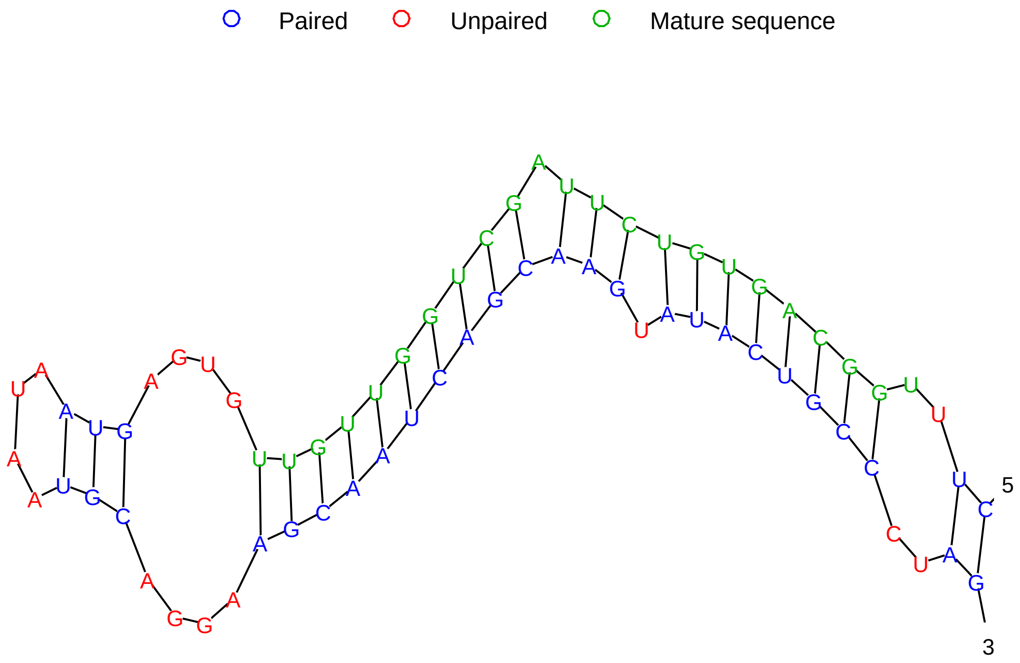

miRNA: bta-miR-34a
Stem loop (UMD3.1): chr16:45197408-45197477
Mature (UMD3.1): chr16:45197411-45197433
Mature seq len: 23
Total raw counts (9 samples): 530
Average raw counts: 59
Strand: Forward
Orientation: 5p
Minimum free energy: -29.10

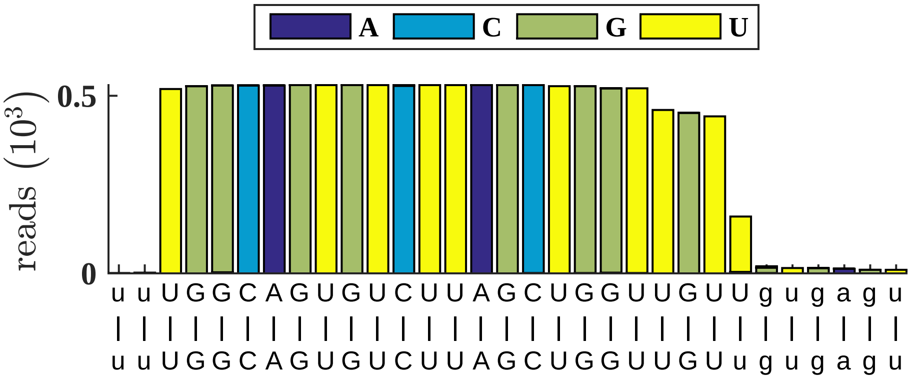

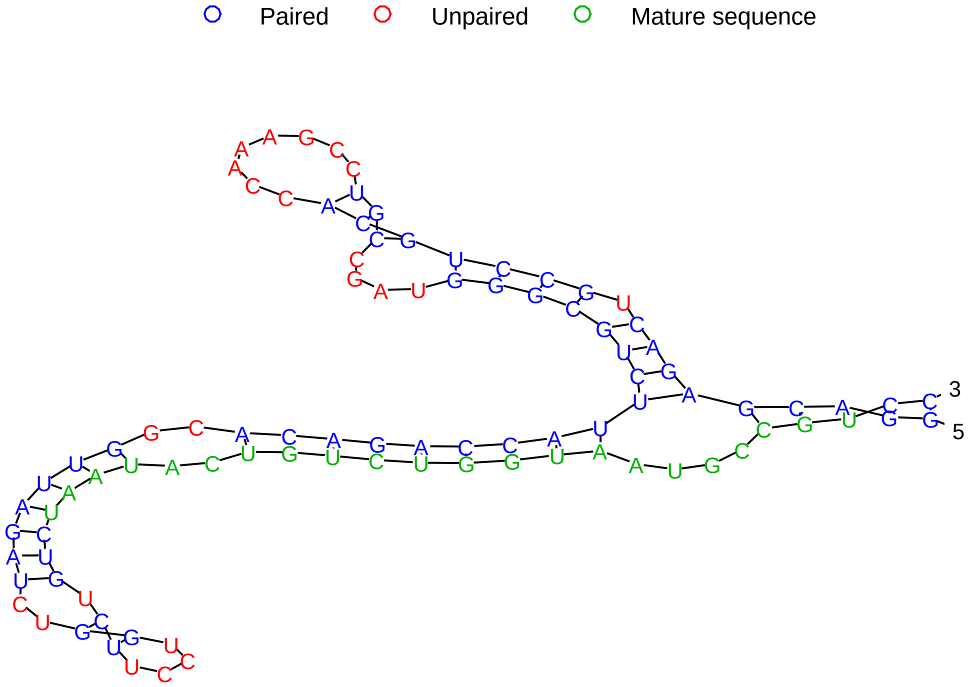

miRNA: bta-miR-429
Stem loop (UMD3.1): chr16:52520098-52520193
Mature (UMD3.1): chr16:52520100-52520121
Mature seq len: 22
Total raw counts (9 samples): 1159
Average raw counts: 129
Strand: Reverse
Orientation: 3p
Minimum free energy: -34.50

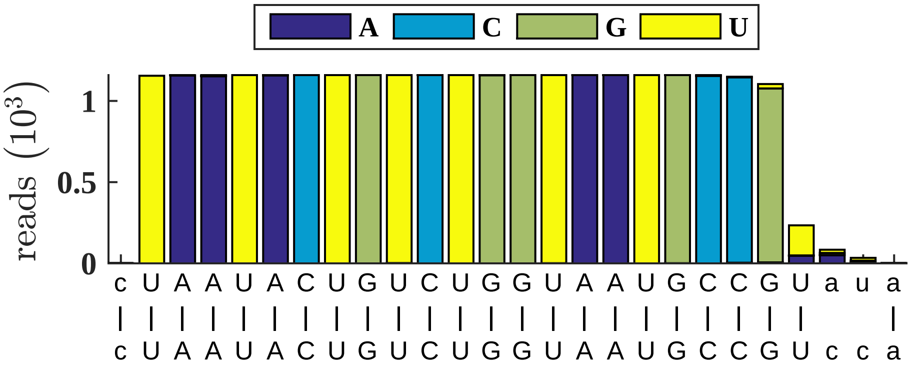

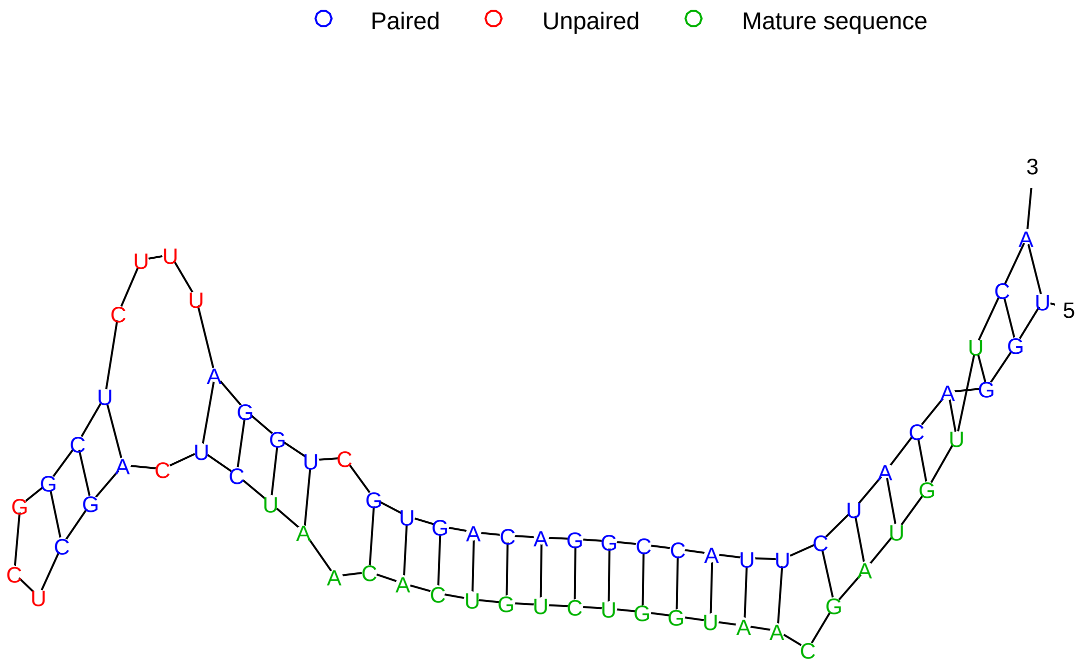

miRNA: bta-miR-200a
Stem loop (UMD3.1): chr16:52521334-52521400
Mature (UMD3.1): chr16:52521336-52521358
Mature seq len: 23
Total raw counts (9 samples): 852
Average raw counts: 95
Strand: Reverse
Orientation: 3p
Minimum free energy: -33.30

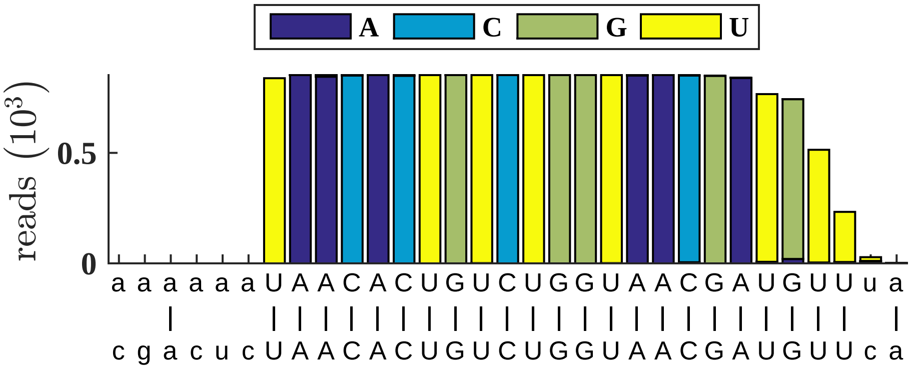

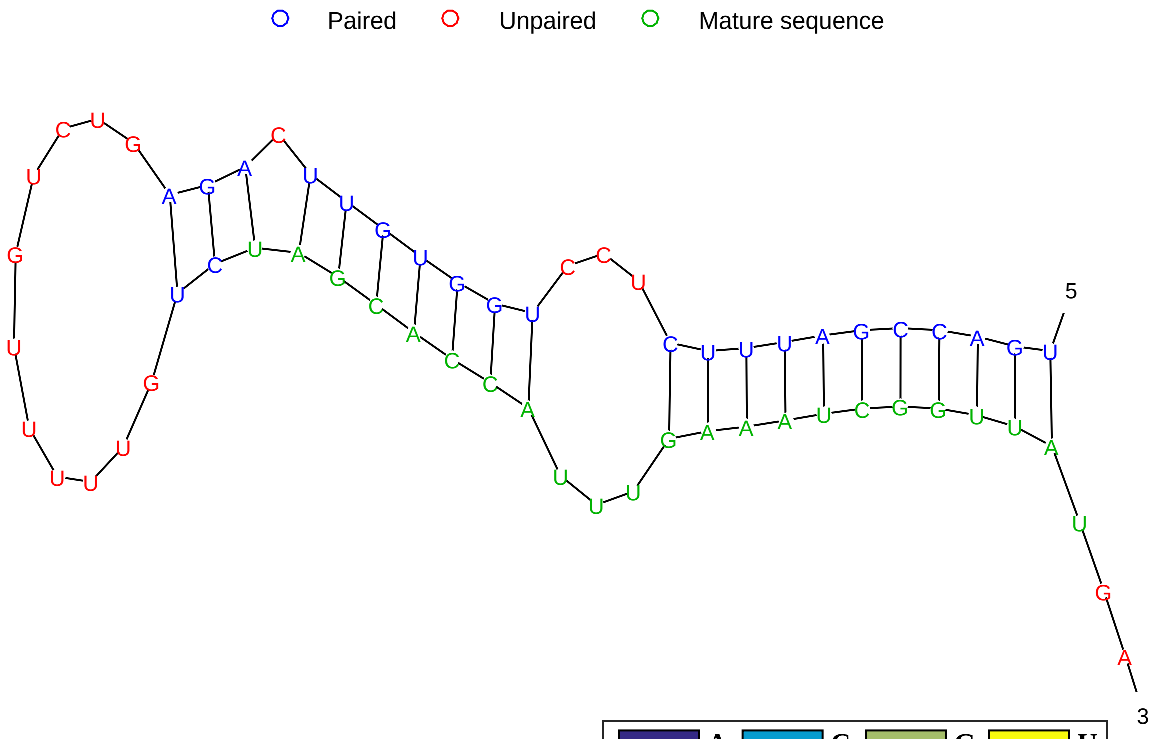

miRNA: bta-miR-29c
Stem loop (UMD3.1): chr16:77478608-77478670
Mature (UMD3.1): chr16:77478646-77478668
Mature seq len: 23
Total raw counts (9 samples): 2833
Average raw counts: 315
Strand: Forward
Orientation: 3p
Minimum free energy: -25.20

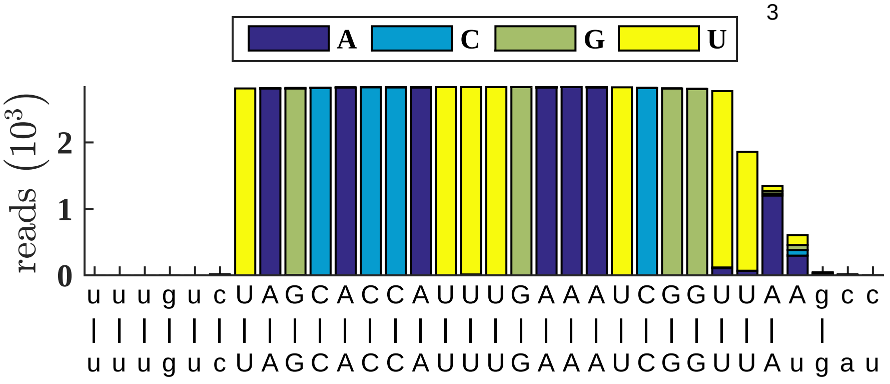

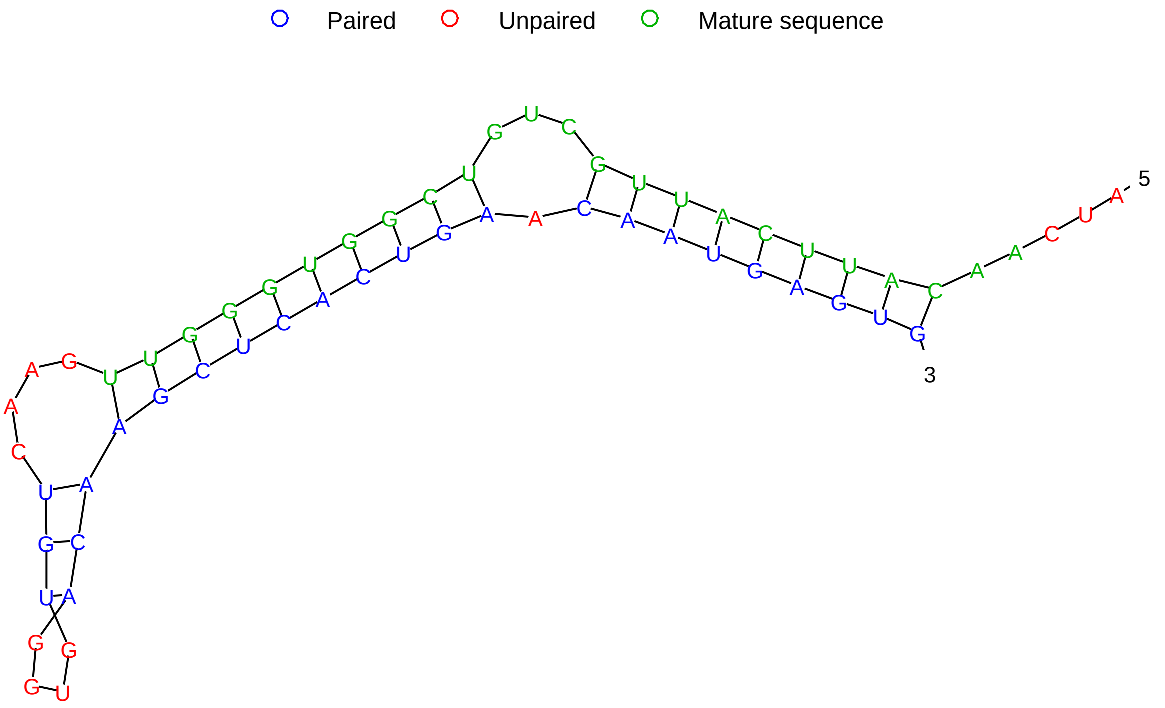

miRNA: bta-miR-181b
Stem loop (UMD3.1): chr16:79685777-79685837
Mature (UMD3.1): chr16:79685811-79685834
Mature seq len: 24
Total raw counts (9 samples): 5958
Average raw counts: 662
Strand: Reverse
Orientation: 5p
Minimum free energy: -22.70

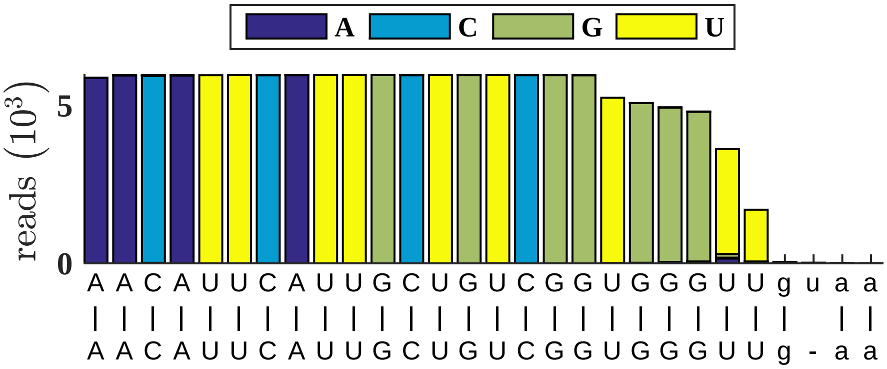

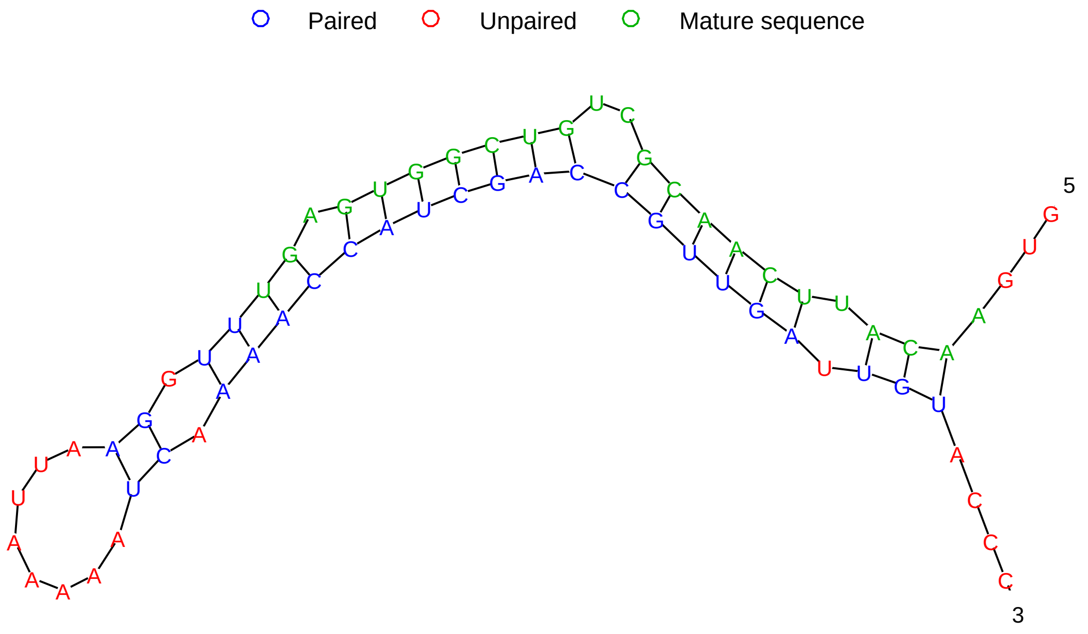

miRNA: bta-miR-181a
Stem loop (UMD3.1): chr16:79685955-79686021
Mature (UMD3.1): chr16:79685996-79686018
Mature seq len: 23
Total raw counts (9 samples): 40493
Average raw counts: 4500
Strand: Reverse
Orientation: 5p
Minimum free energy: -22.30

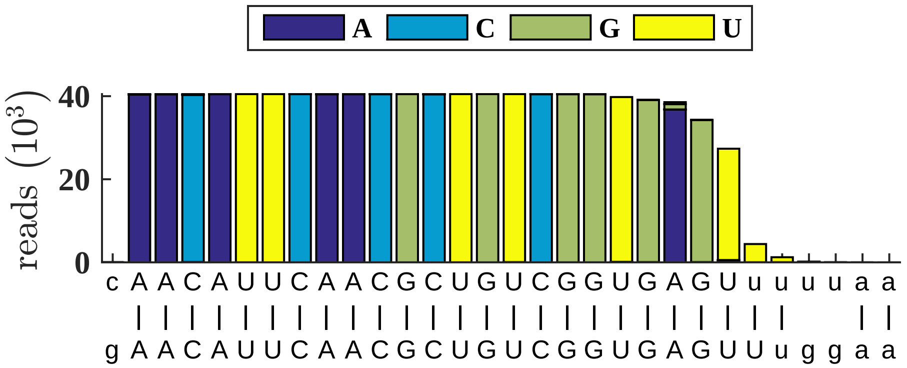

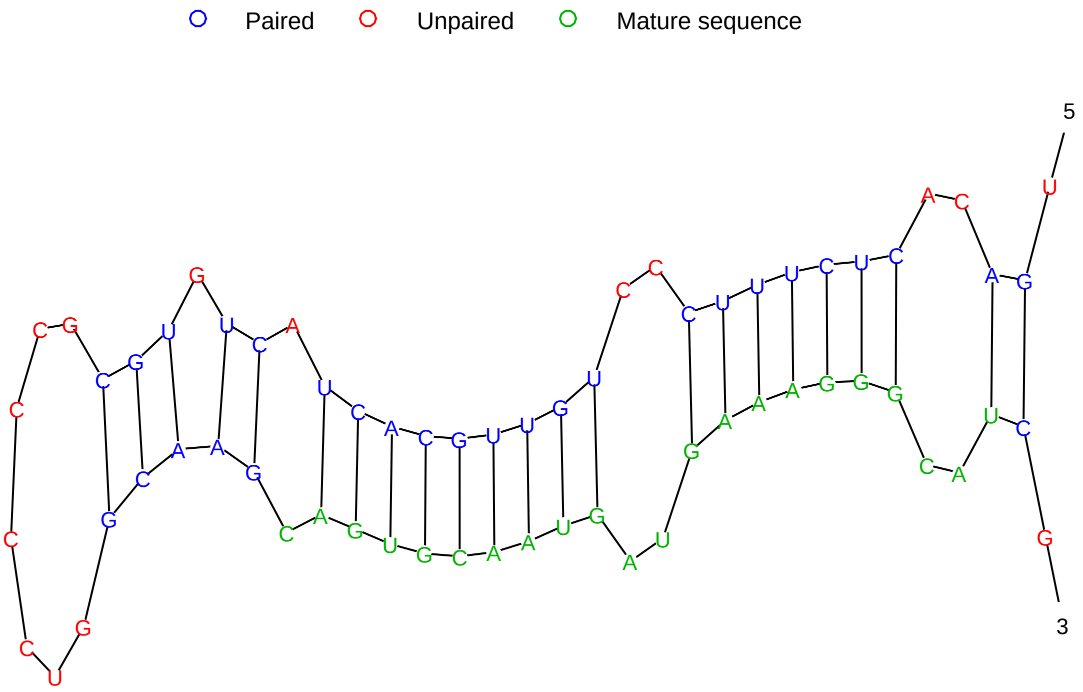

miRNA: bta-miR-130b
Stem loop (UMD3.1): chr17:74100547-74100612
Mature (UMD3.1): chr17:74100549-74100570
Mature seq len: 22
Total raw counts (9 samples): 2183
Average raw counts: 243
Strand: Reverse
Orientation: 3p
Minimum free energy: -23.60

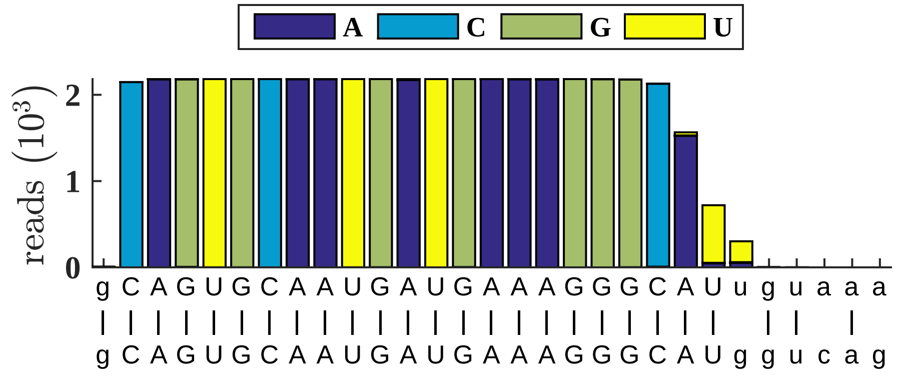

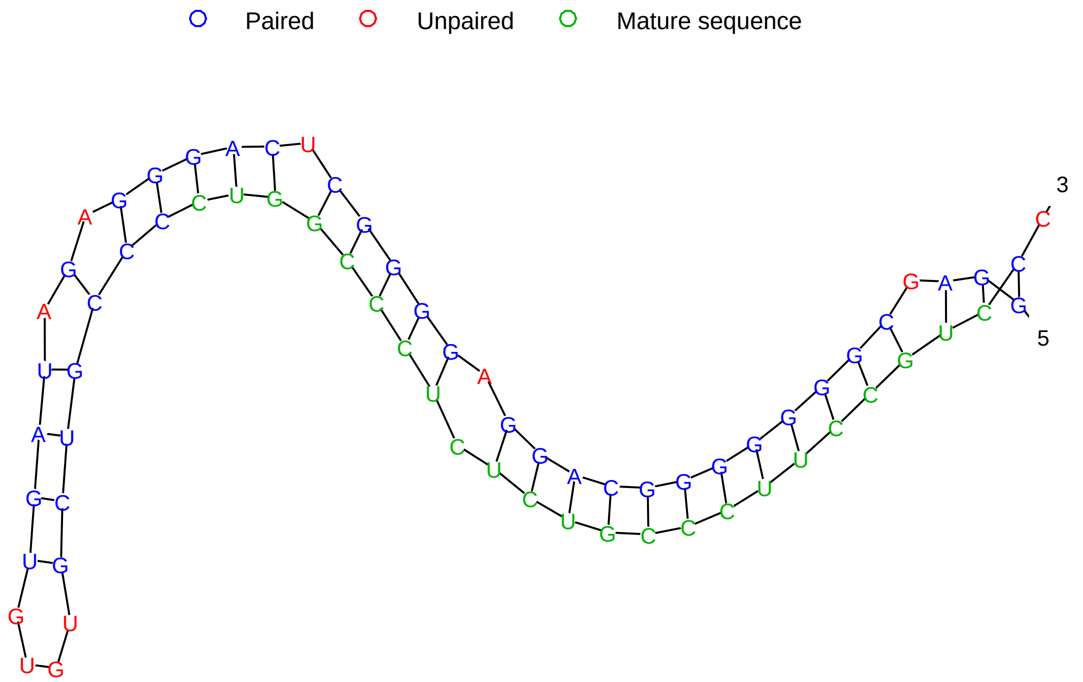

miRNA: bta-miR-328
Stem loop (UMD3.1): chr18:34957617-34957687
Mature (UMD3.1): chr18:34957619-34957641
Mature seq len: 23
Total raw counts (9 samples): 955
Average raw counts: 107
Strand: Reverse
Orientation: 3p
Minimum free energy: -49.40

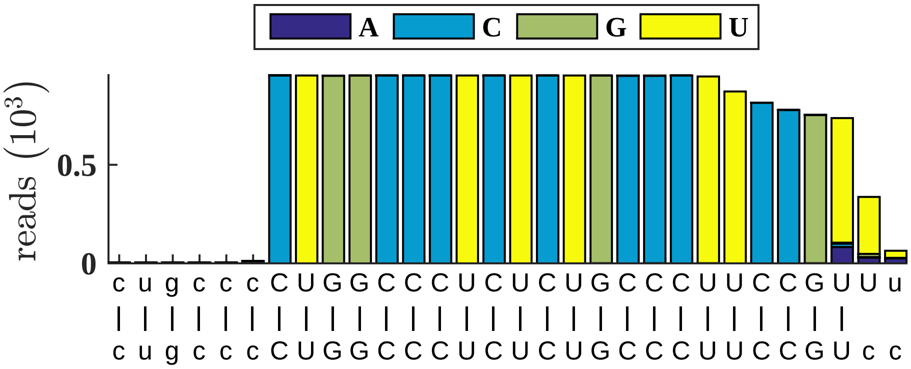

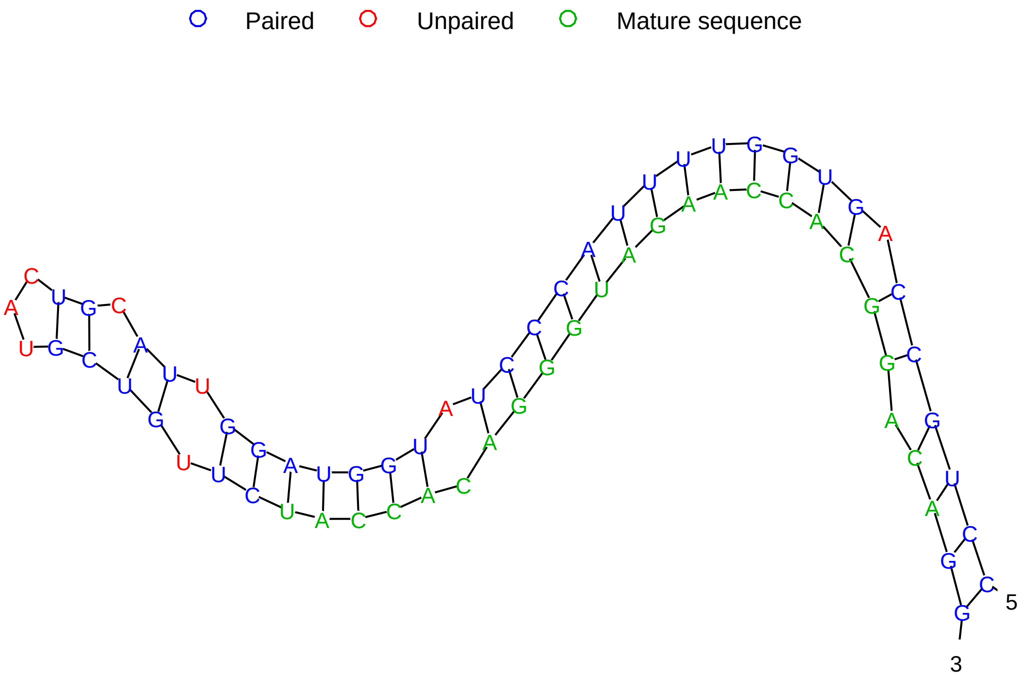

miRNA: bta-miR-140
Stem loop (UMD3.1): chr18:37088148-37088217
Mature (UMD3.1): chr18:37088192-37088215
Mature seq len: 24
Total raw counts (9 samples): 42829
Average raw counts: 4759
Strand: Forward
Orientation: 3p
Minimum free energy: -39.10

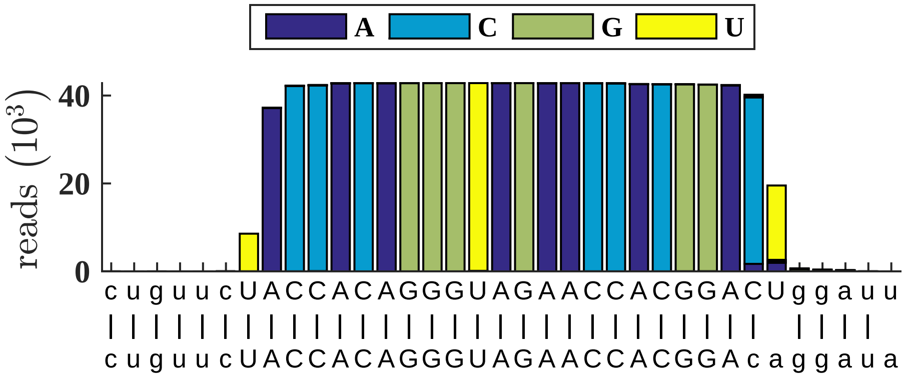

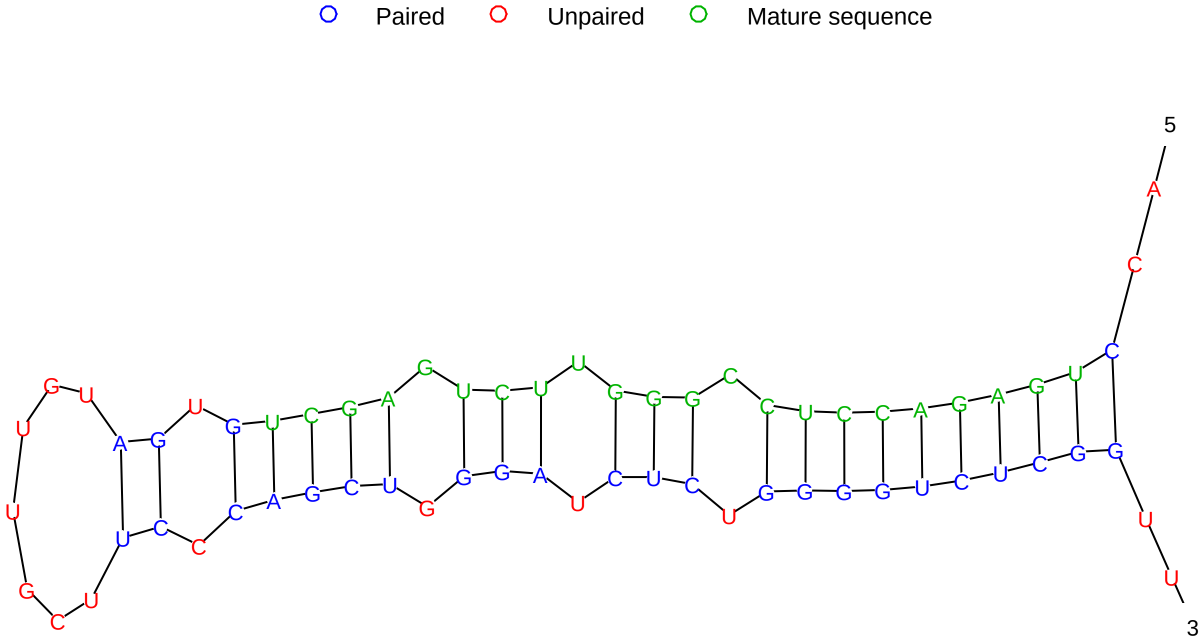

miRNA: bta-miR-769
Stem loop (UMD3.1): chr18:53945903-53945967
Mature (UMD3.1): chr18:53945906-53945927
Mature seq len: 22
Total raw counts (9 samples): 7460
Average raw counts: 829
Strand: Forward
Orientation: 5p
Minimum free energy: -34.70

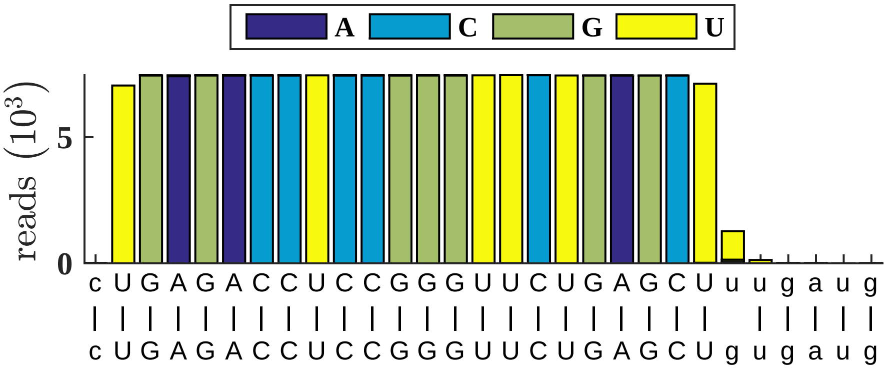

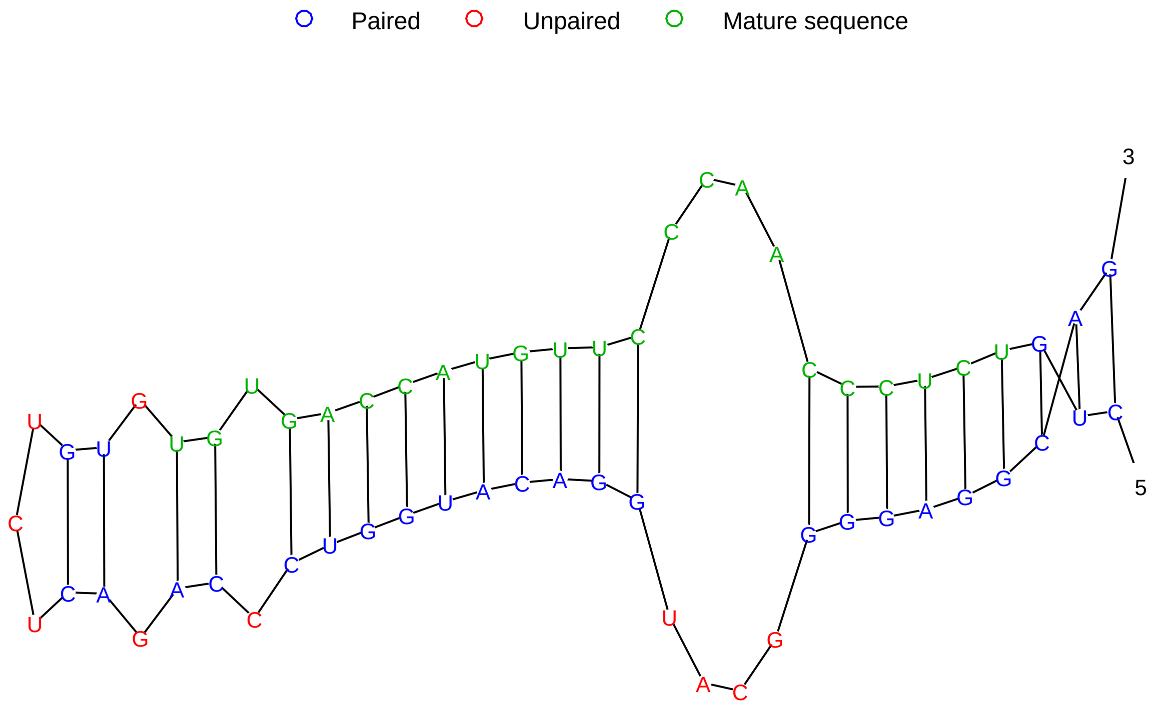

miRNA: bta-miR-150
Stem loop (UMD3.1): chr18:56407865-56407925
Mature (UMD3.1): chr18:56407900-56407922
Mature seq len: 23
Total raw counts (9 samples): 673
Average raw counts: 75
Strand: Reverse
Orientation: 5p
Minimum free energy: -34.80

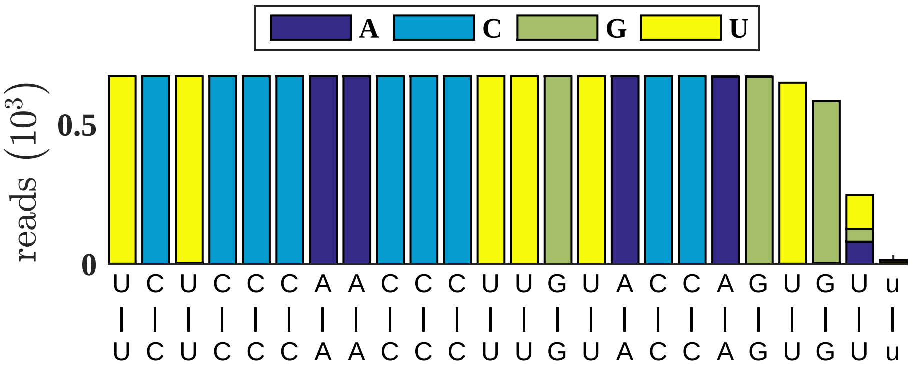

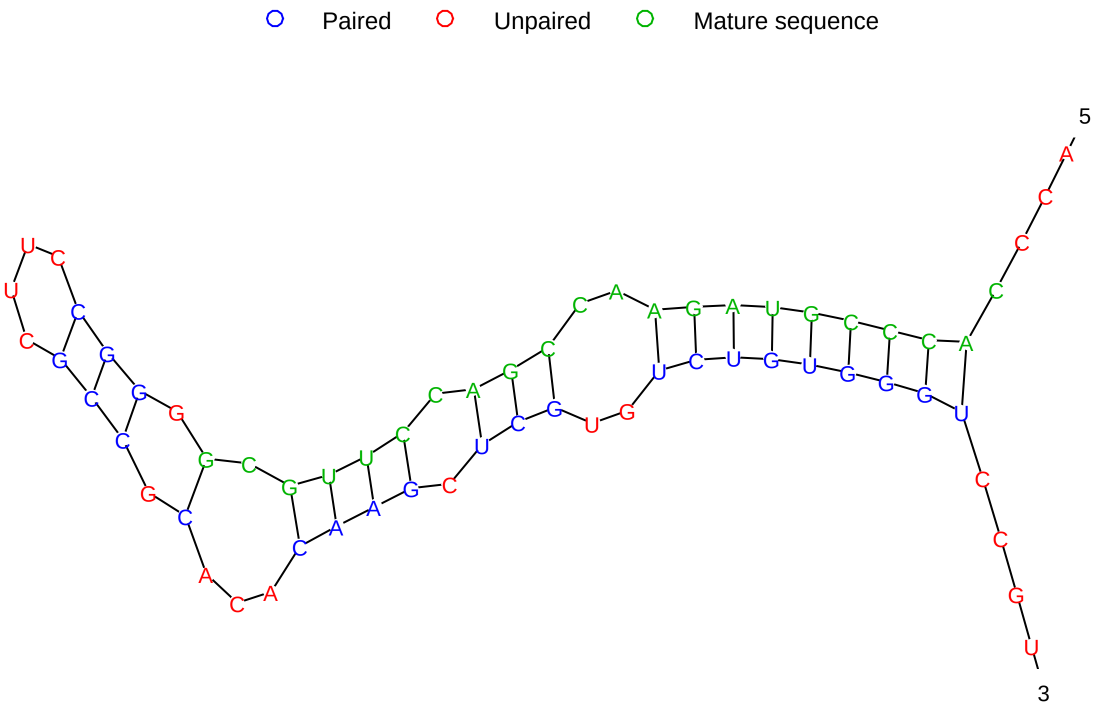

miRNA: bta-miR-99b
Stem loop (UMD3.1): chr18:58014871-58014934
Mature (UMD3.1): chr18:58014874-58014895
Mature seq len: 22
Total raw counts (9 samples): 74998
Average raw counts: 8334
Strand: Forward
Orientation: 5p
Minimum free energy: -23.40

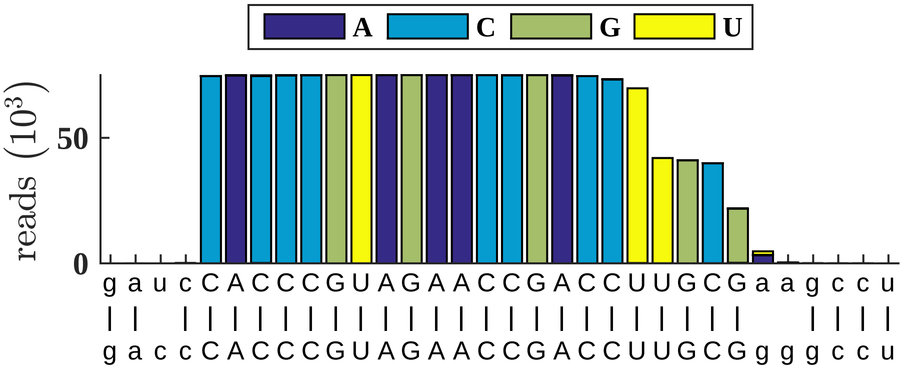

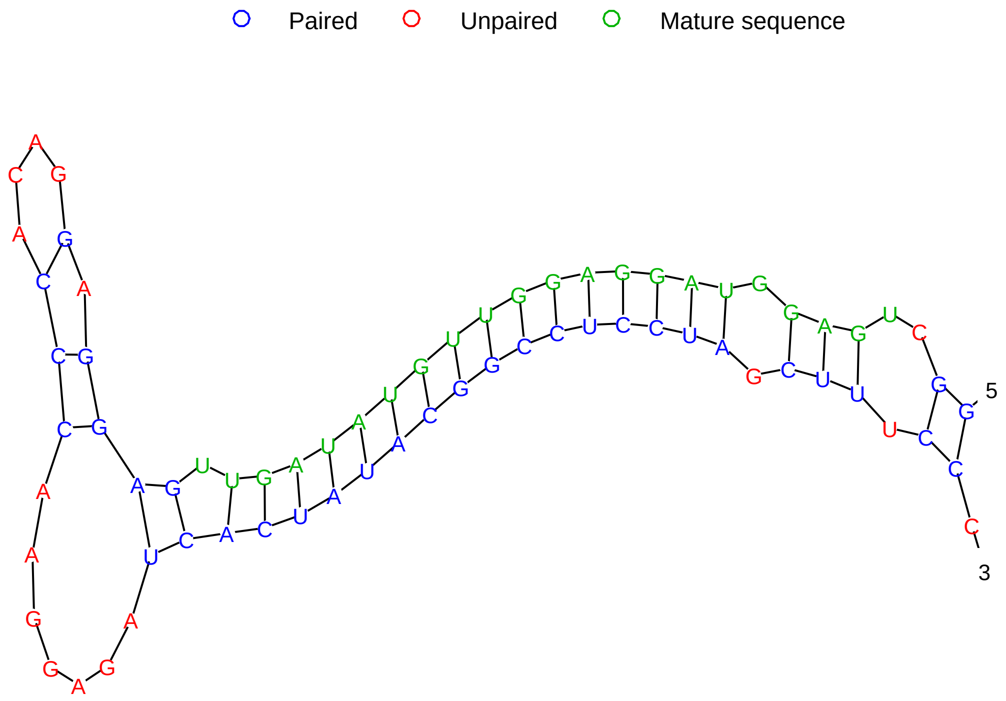

miRNA: bta-let-7e
Stem loop (UMD3.1): chr18:58015040-58015110
Mature (UMD3.1): chr18:58015043-58015064
Mature seq len: 22
Total raw counts (9 samples): 43765
Average raw counts: 4863
Strand: Forward
Orientation: 5p
Minimum free energy: -31.10

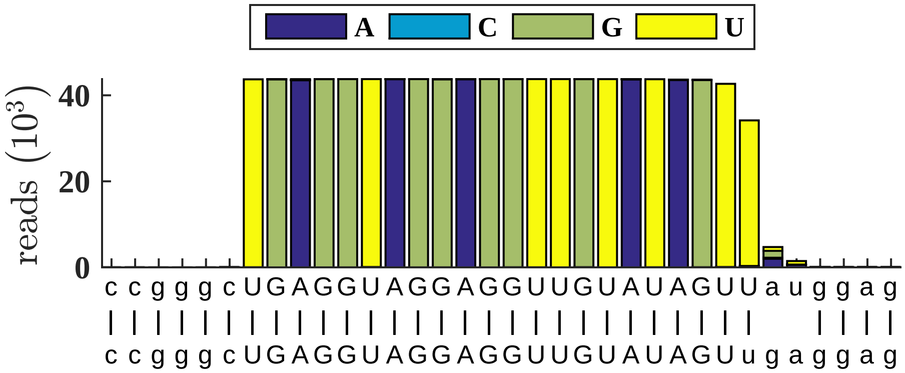

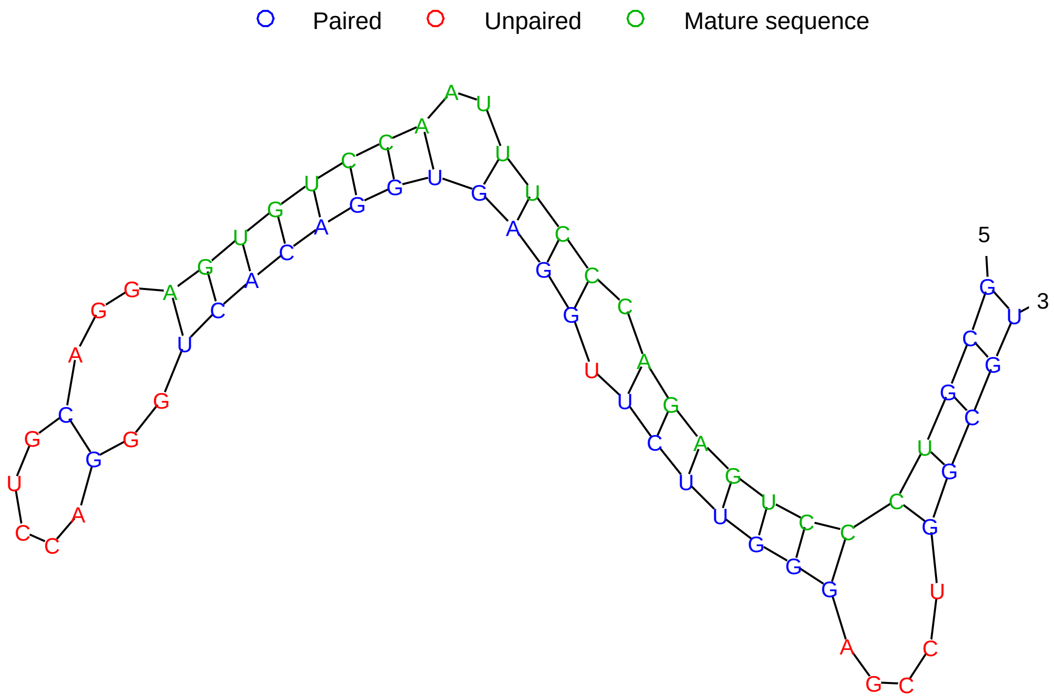

miRNA: bta-miR-125a
Stem loop (UMD3.1): chr18:58015546-58015614
Mature (UMD3.1): chr18:58015549-58015572
Mature seq len: 24
Total raw counts (9 samples): 58910
Average raw counts: 6546
Strand: Forward
Orientation: 5p
Minimum free energy: -28.60

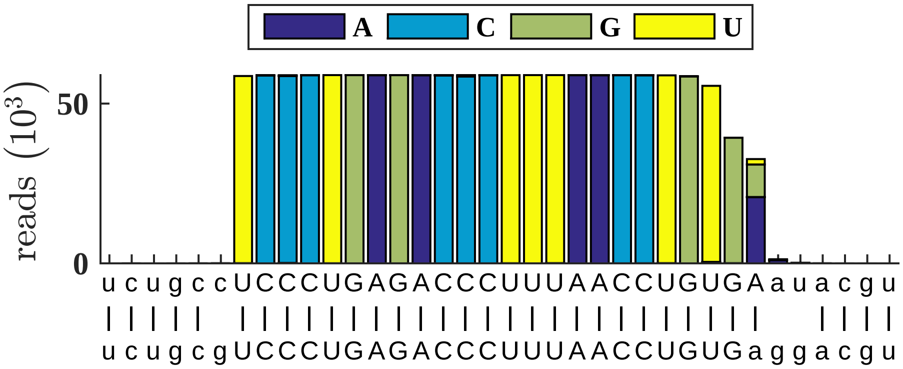

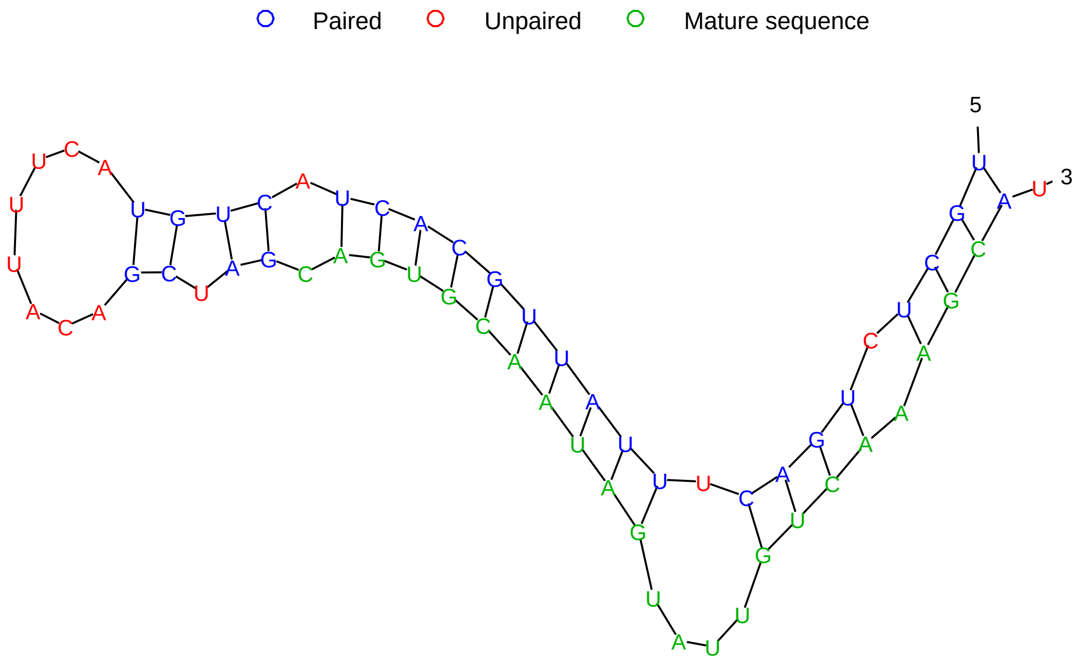

miRNA: bta-miR-301a
Stem loop (UMD3.1): chr19:10349516-10349578
Mature (UMD3.1): chr19:10349518-10349540
Mature seq len: 23
Total raw counts (9 samples): 9196
Average raw counts: 1022
Strand: Reverse
Orientation: 3p
Minimum free energy: -20.50

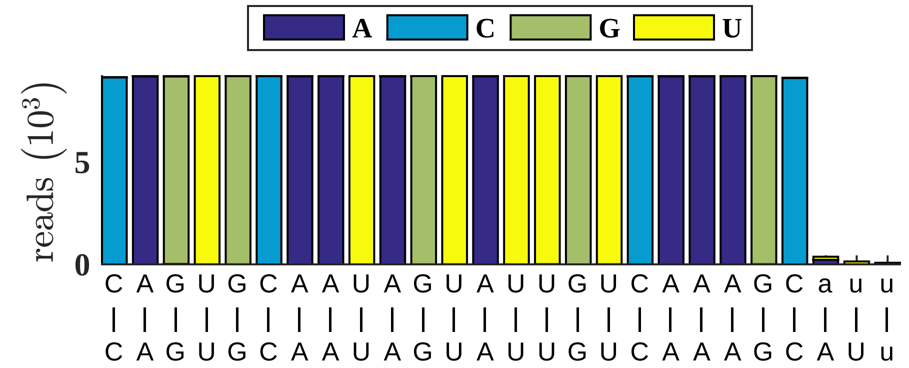

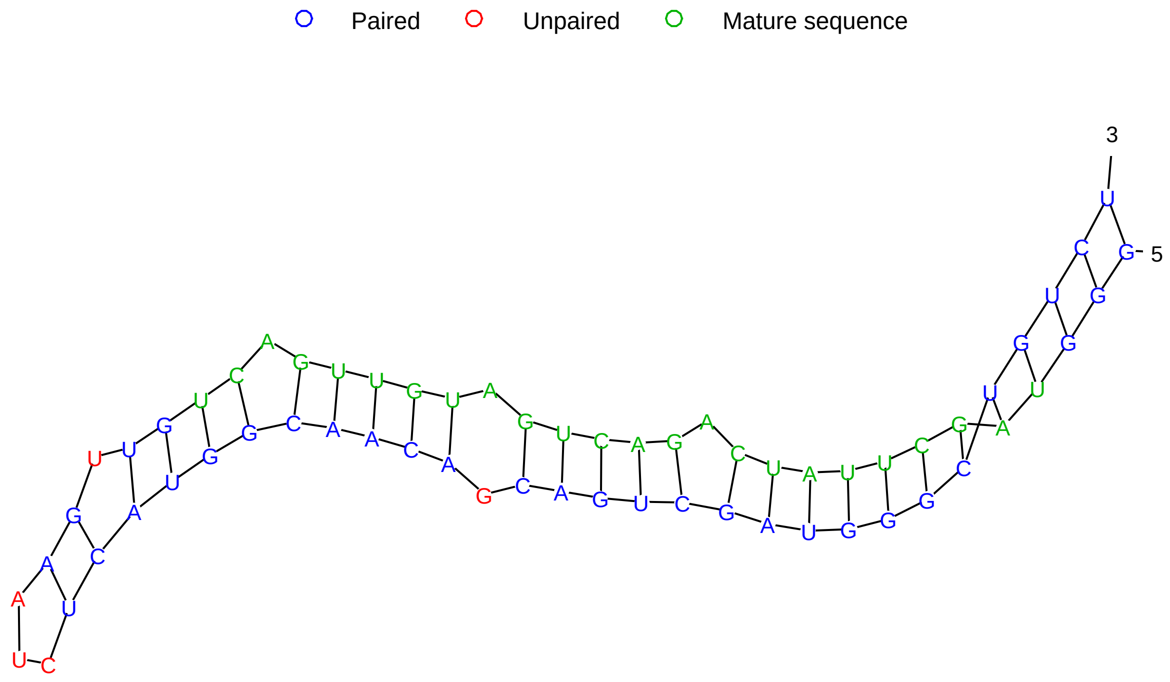

miRNA: bta-miR-21-5p
Stem loop (UMD3.1): chr19:11033076-11033139
Mature (UMD3.1): chr19:11033079-11033102
Mature seq len: 24
Total raw counts (9 samples): 170322
Average raw counts: 18925
Strand: Forward
Orientation: 5p
Minimum free energy: -26.50

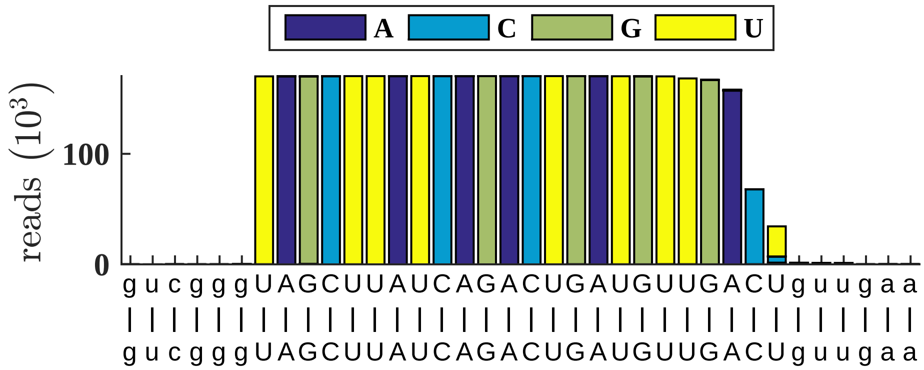

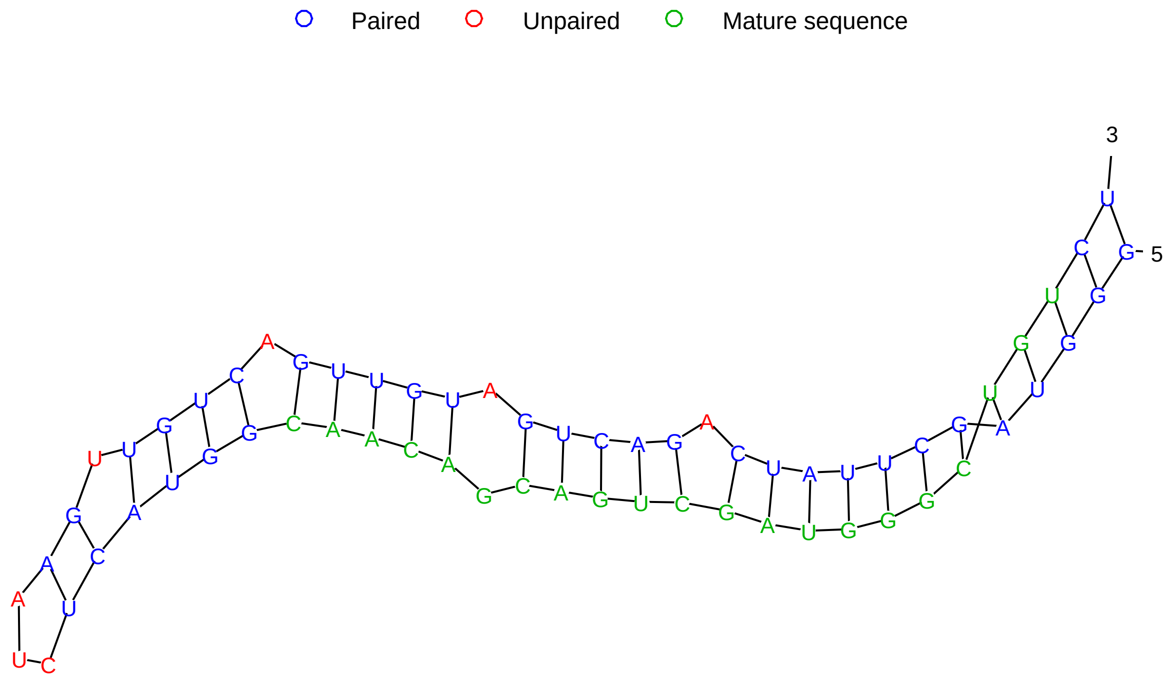

miRNA: bta-miR-21-3p
Stem loop (UMD3.1): chr19:11033076-11033139
Mature (UMD3.1): chr19:11033117-11033137
Mature seq len: 21
Total raw counts (9 samples): 4413
Average raw counts: 491
Strand: Forward
Orientation: 3p
Minimum free energy: -26.50

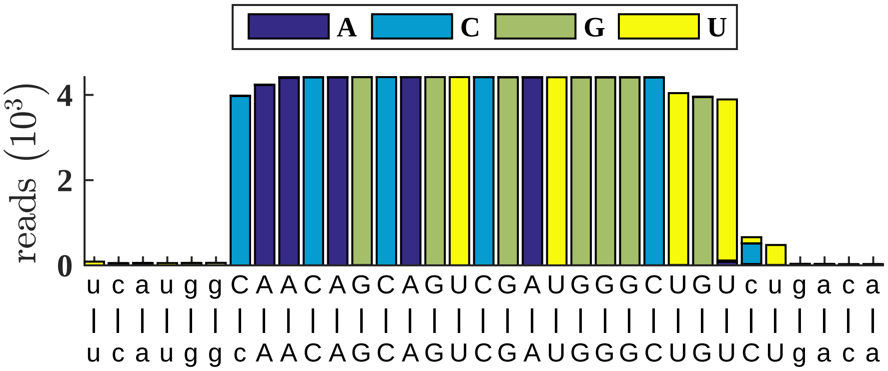

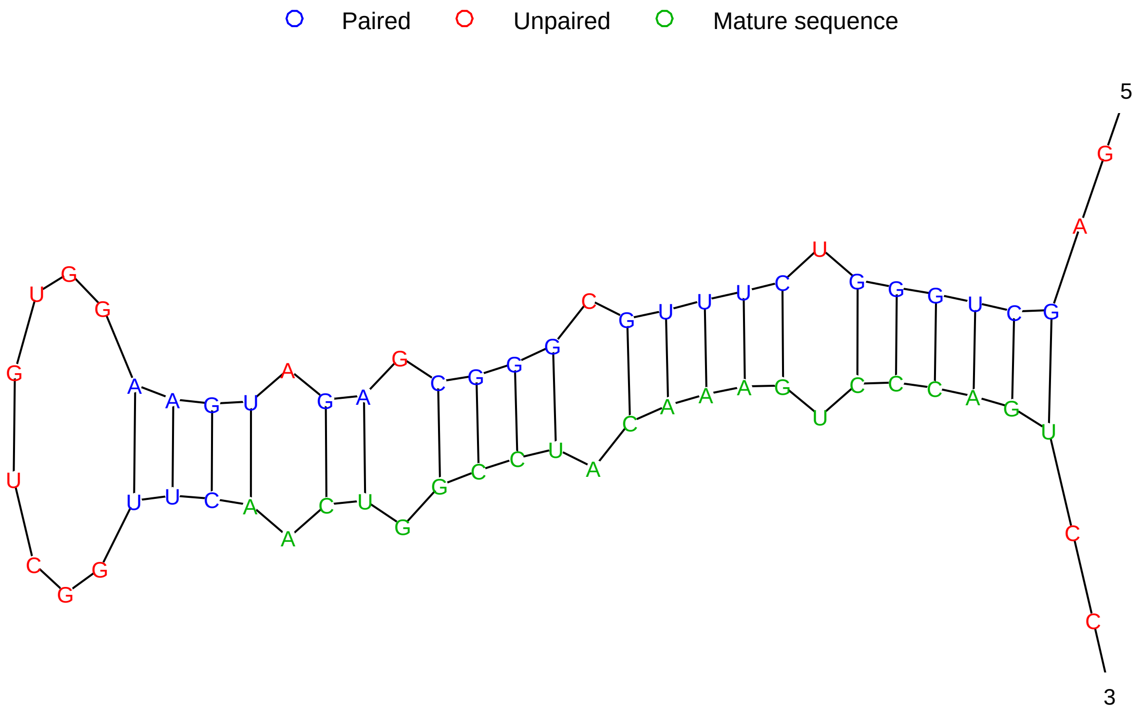

miRNA: bta-miR-193a-3p
Stem loop (UMD3.1): chr19:18824230-18824291
Mature (UMD3.1): chr19:18824232-18824253
Mature seq len: 22
Total raw counts (9 samples): 406
Average raw counts: 46
Strand: Reverse
Orientation: 3p
Minimum free energy: -31.00

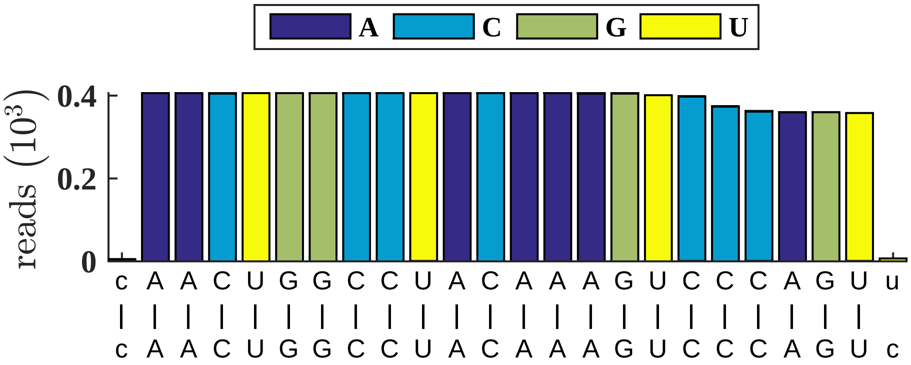

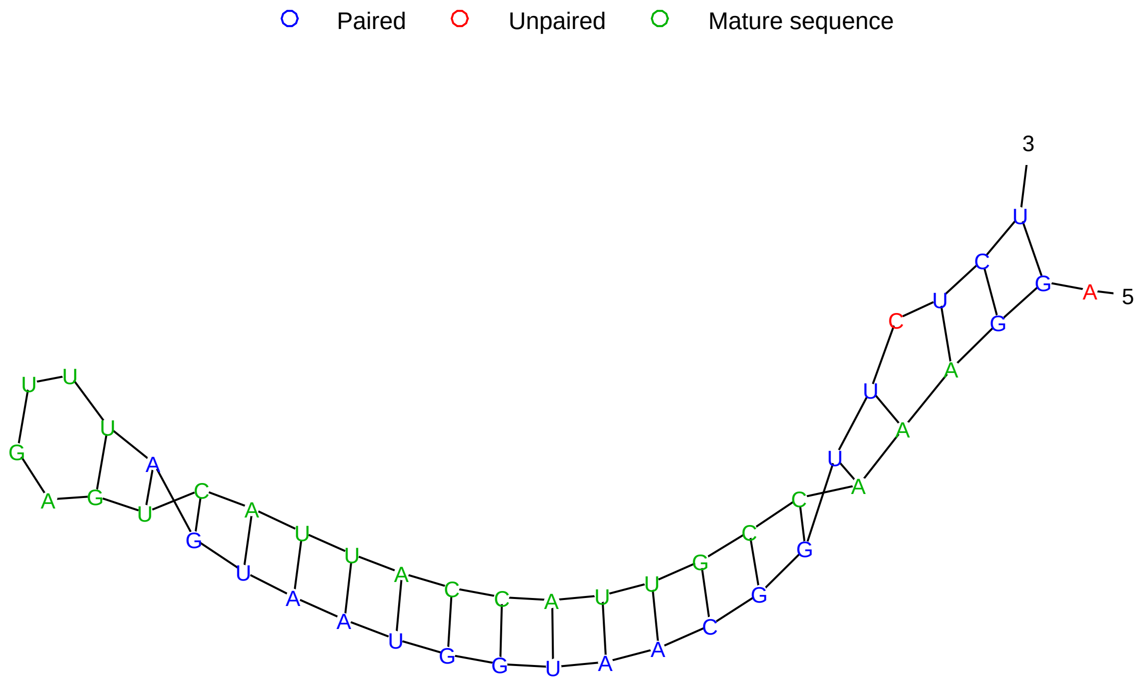

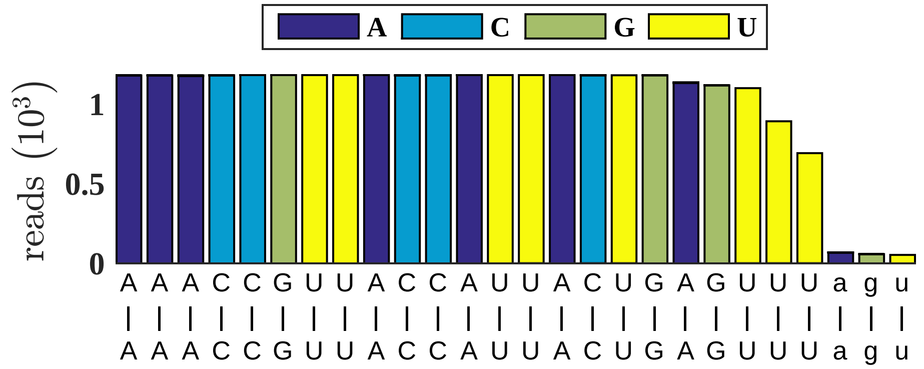

miRNA: bta-miR-451
Stem loop (UMD3.1): chr19:20796873-20796918
Mature (UMD3.1): chr19:20796893-20796915
Mature seq len: 23
Total raw counts (9 samples): 1182
Average raw counts: 132
Strand: Reverse
Orientation: 5p
Minimum free energy: -25.70

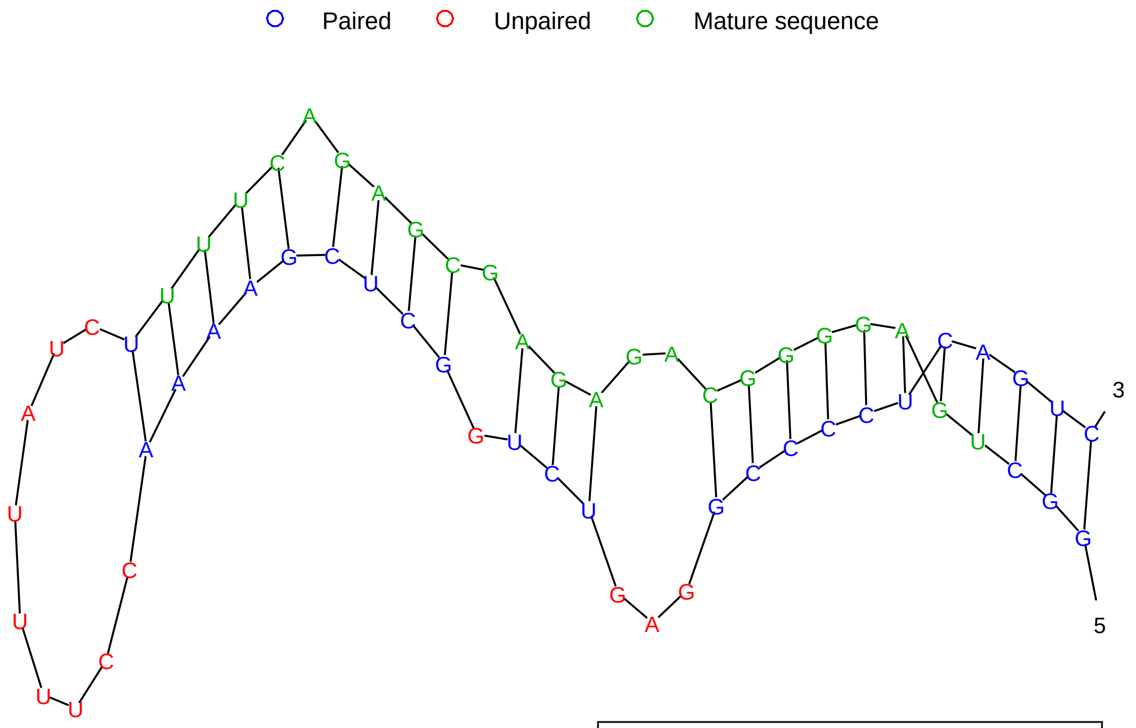

miRNA: bta-miR-423-5p
Stem loop (UMD3.1): chr19:21799497-21799559
Mature (UMD3.1): chr19:21799500-21799522
Mature seq len: 23
Total raw counts (9 samples): 558528
Average raw counts: 62059
Strand: Forward
Orientation: 5p
Minimum free energy: -34.80

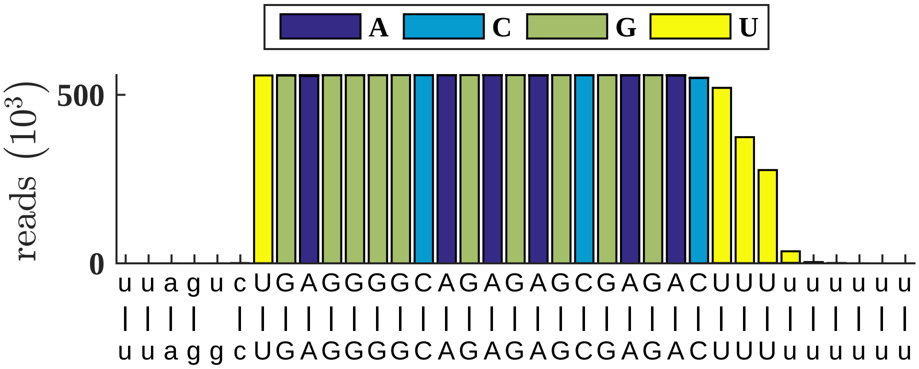

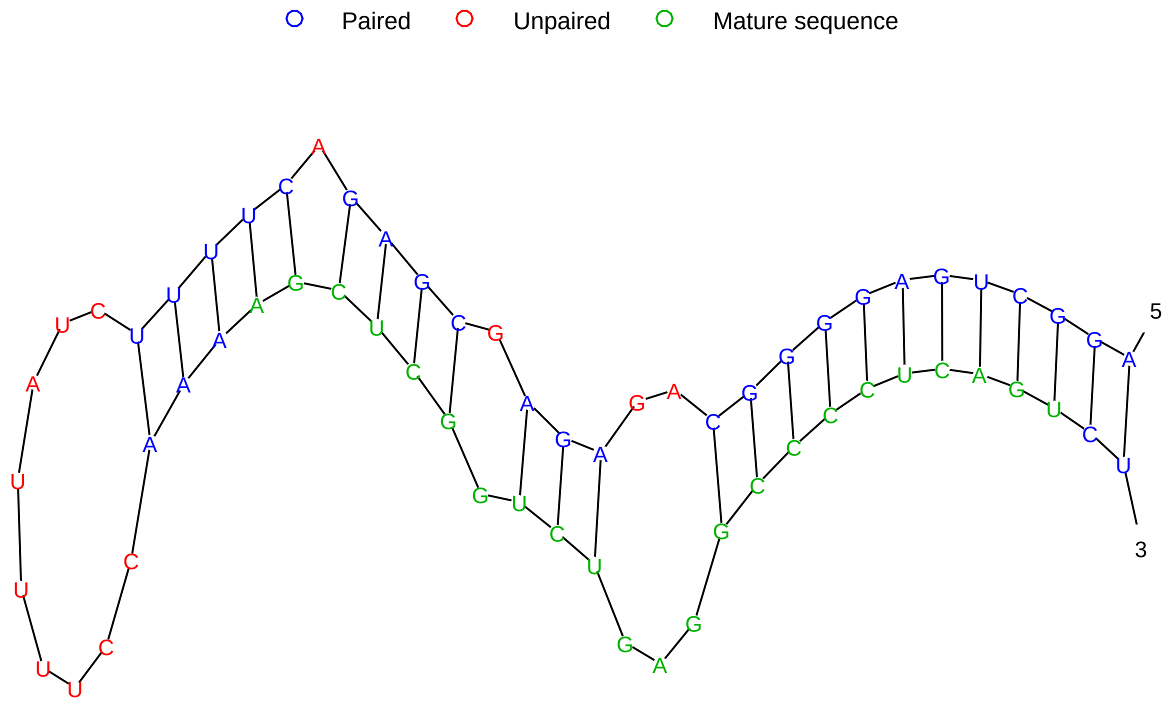

miRNA: bta-miR-423-3p
Stem loop (UMD3.1): chr19:21799496-21799560
Mature (UMD3.1): chr19:21799536-21799558
Mature seq len: 23
Total raw counts (9 samples): 153051
Average raw counts: 17006
Strand: Forward
Orientation: 3p
Minimum free energy: -36.40

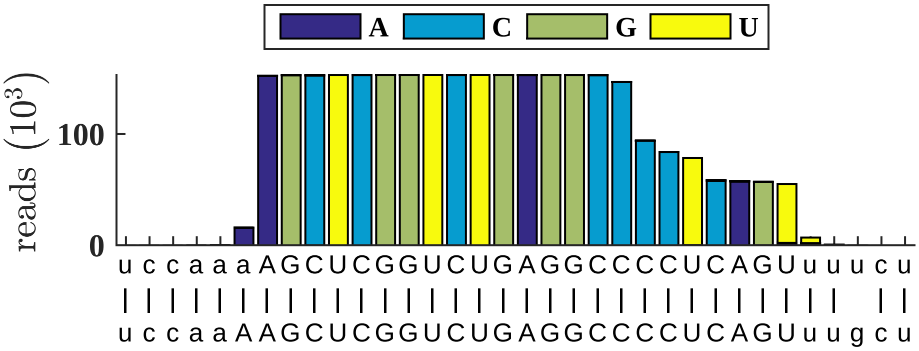

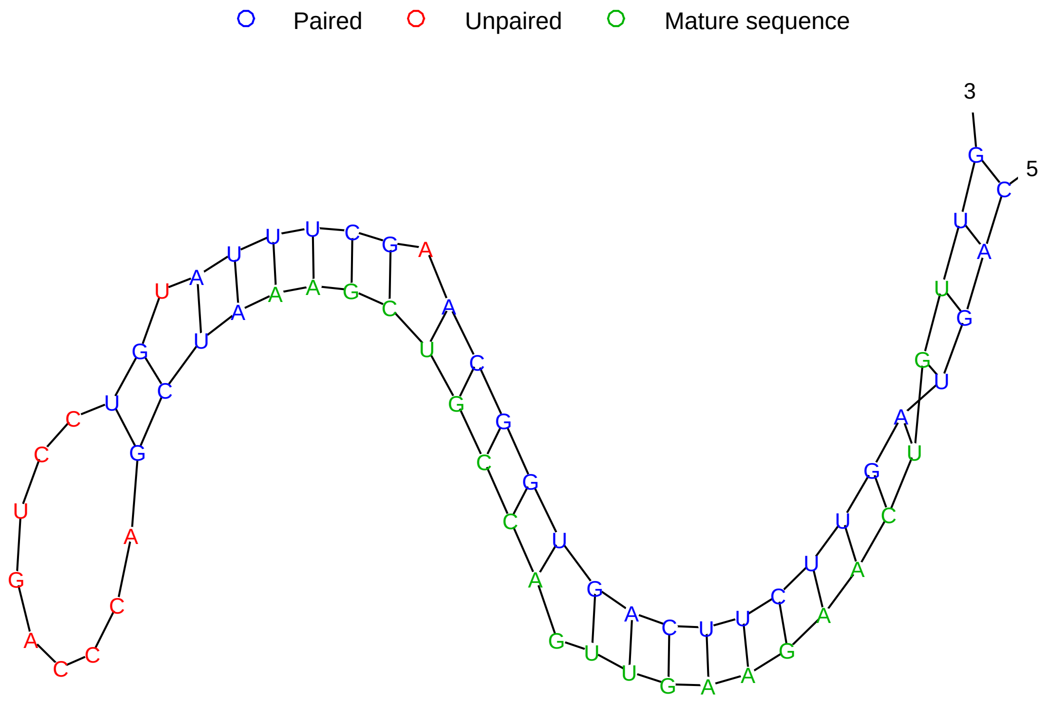

miRNA: bta-miR-22-3p
Stem loop (UMD3.1): chr19:23382444-23382509
Mature (UMD3.1): chr19:23382446-23382467
Mature seq len: 22
Total raw counts (9 samples): 718534
Average raw counts: 79838
Strand: Reverse
Orientation: 3p
Minimum free energy: -28.10

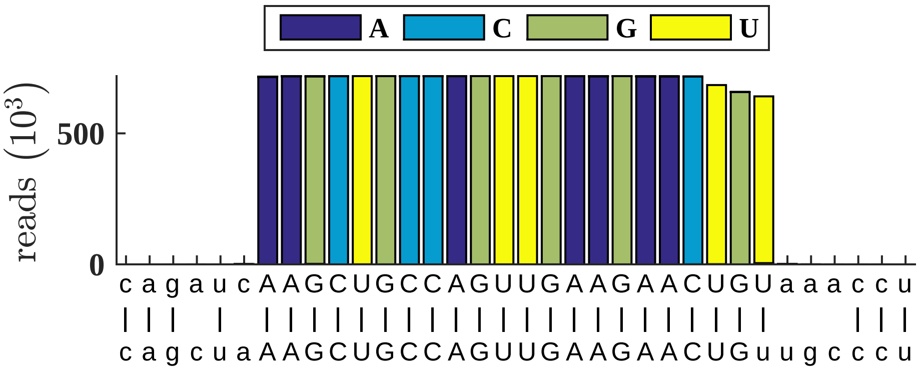

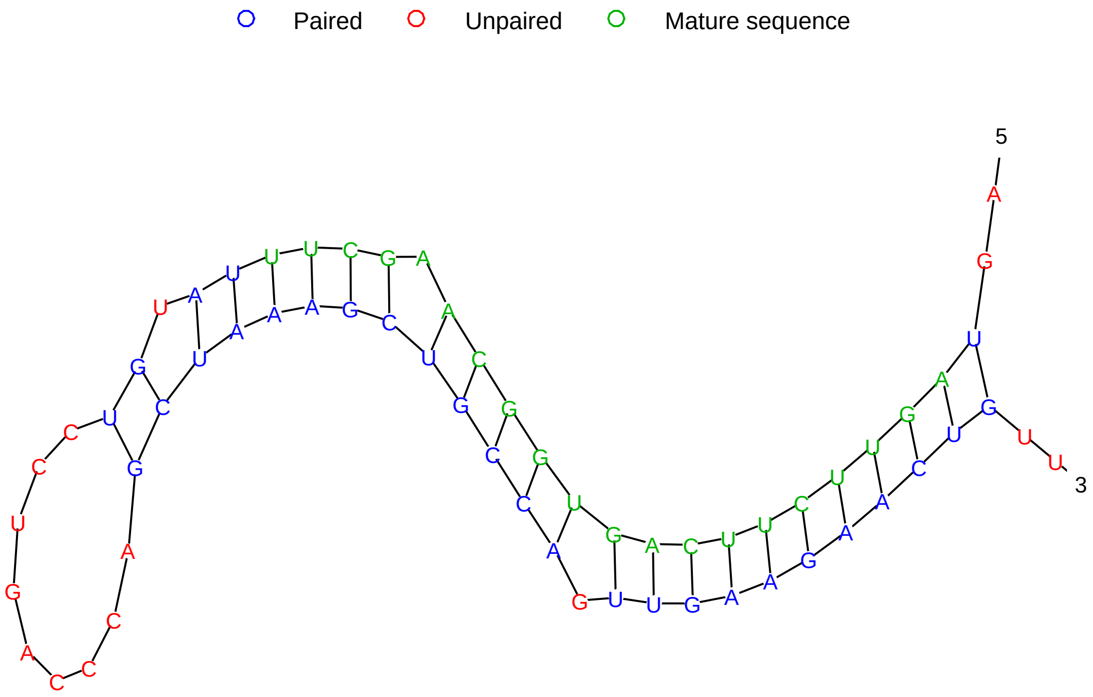

miRNA: bta-miR-22-5p
Stem loop (UMD3.1): chr19:23382445-23382508
Mature (UMD3.1): chr19:23382486-23382505
Mature seq len: 20
Total raw counts (9 samples): 394
Average raw counts: 44
Strand: Reverse
Orientation: 5p
Minimum free energy: -26.80

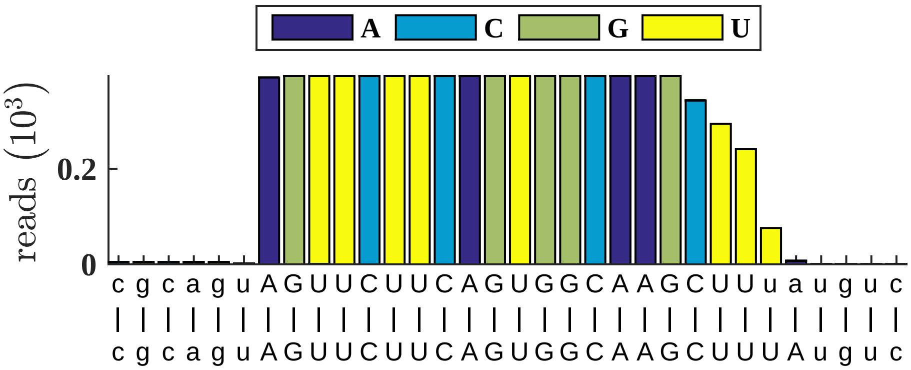

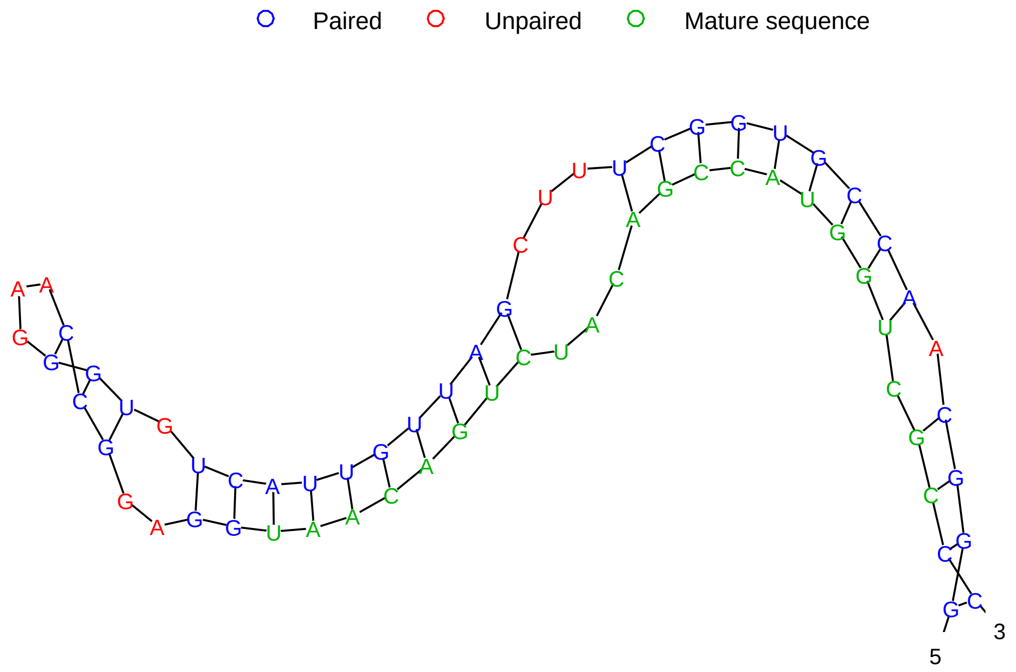

miRNA: bta-miR-132
Stem loop (UMD3.1): chr19:23651158-23651223
Mature (UMD3.1): chr19:23651160-23651182
Mature seq len: 23
Total raw counts (9 samples): 1940
Average raw counts: 216
Strand: Reverse
Orientation: 3p
Minimum free energy: -37.20

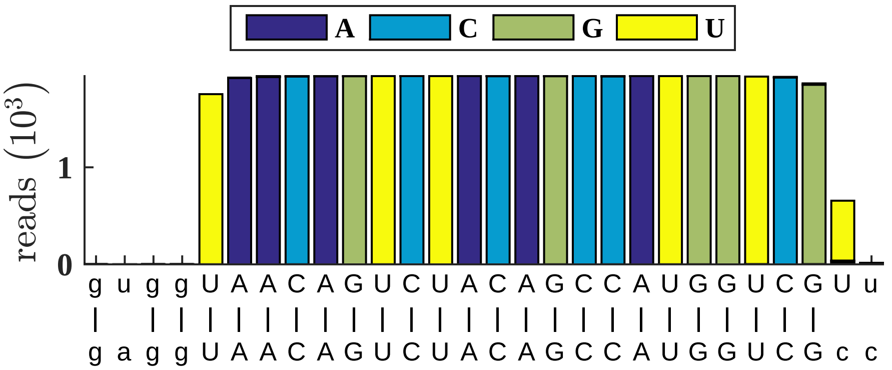

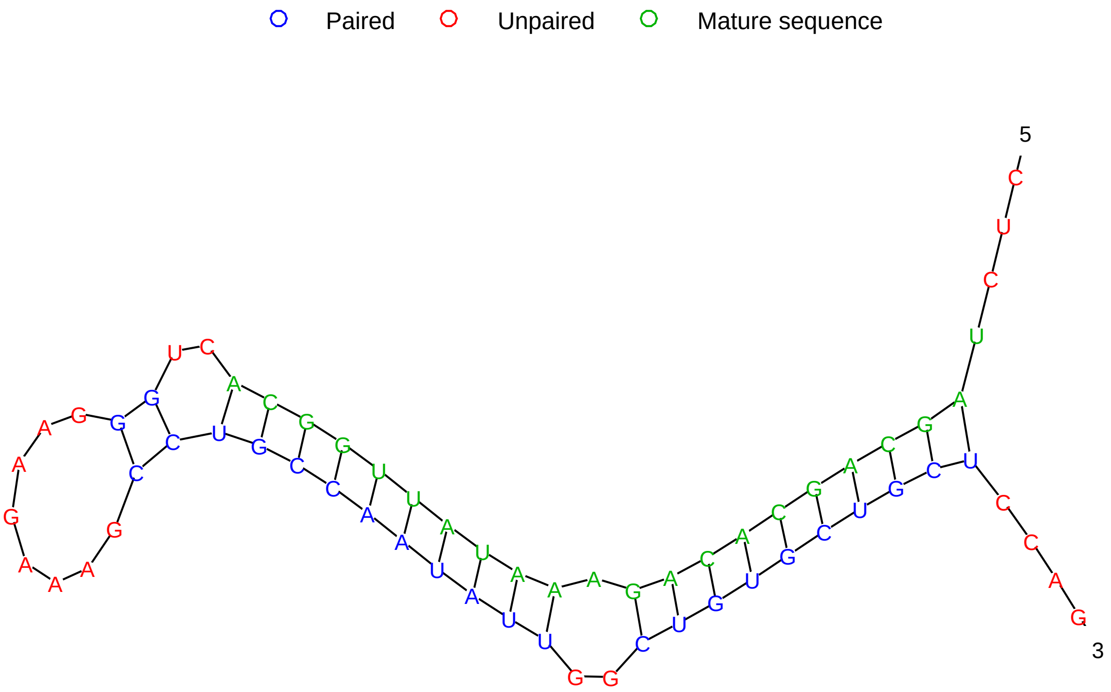

miRNA: bta-miR-195
Stem loop (UMD3.1): chr19:27441354-27441418
Mature (UMD3.1): chr19:27441394-27441415
Mature seq len: 22
Total raw counts (9 samples): 16273
Average raw counts: 1809
Strand: Reverse
Orientation: 5p
Minimum free energy: -32.60

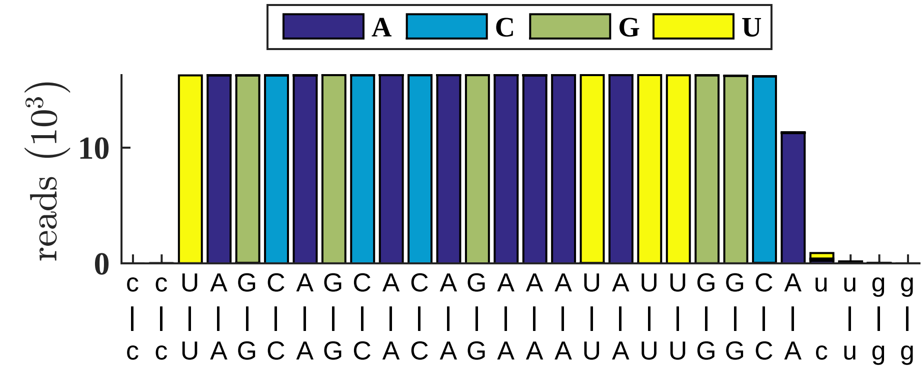

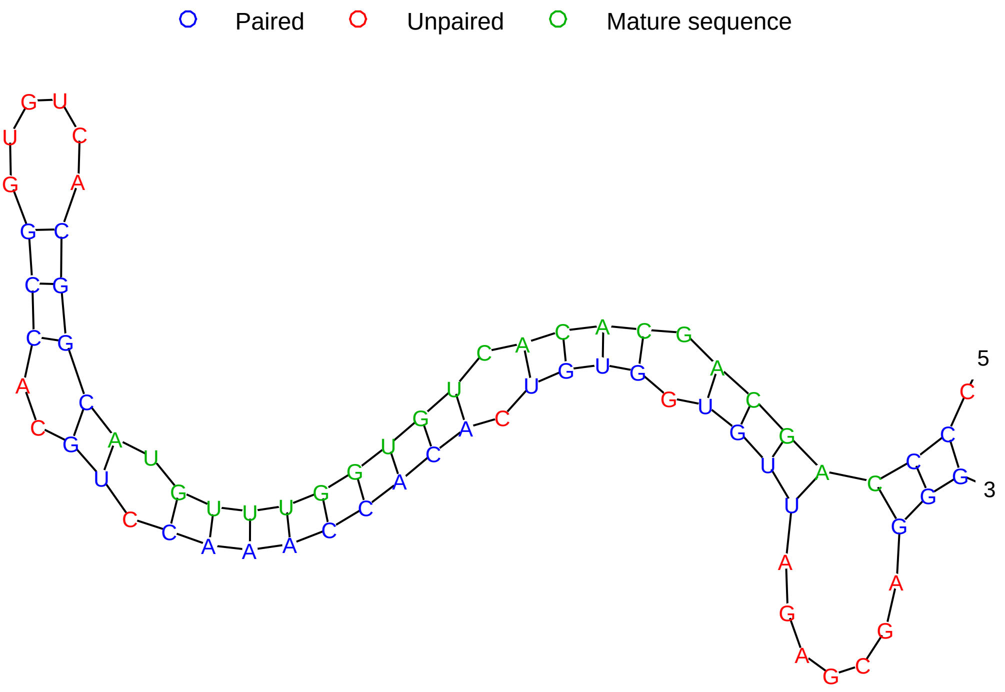

miRNA: bta-miR-497
Stem loop (UMD3.1): chr19:27441672-27441743
Mature (UMD3.1): chr19:27441719-27441740
Mature seq len: 22
Total raw counts (9 samples): 25839
Average raw counts: 2871
Strand: Reverse
Orientation: 5p
Minimum free energy: -29.60

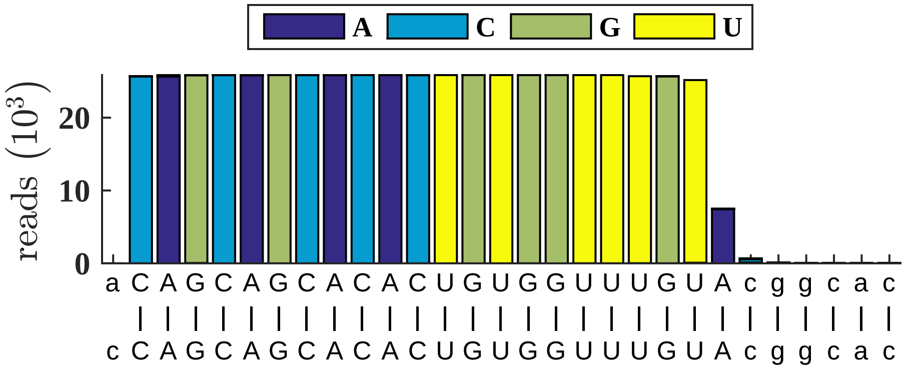

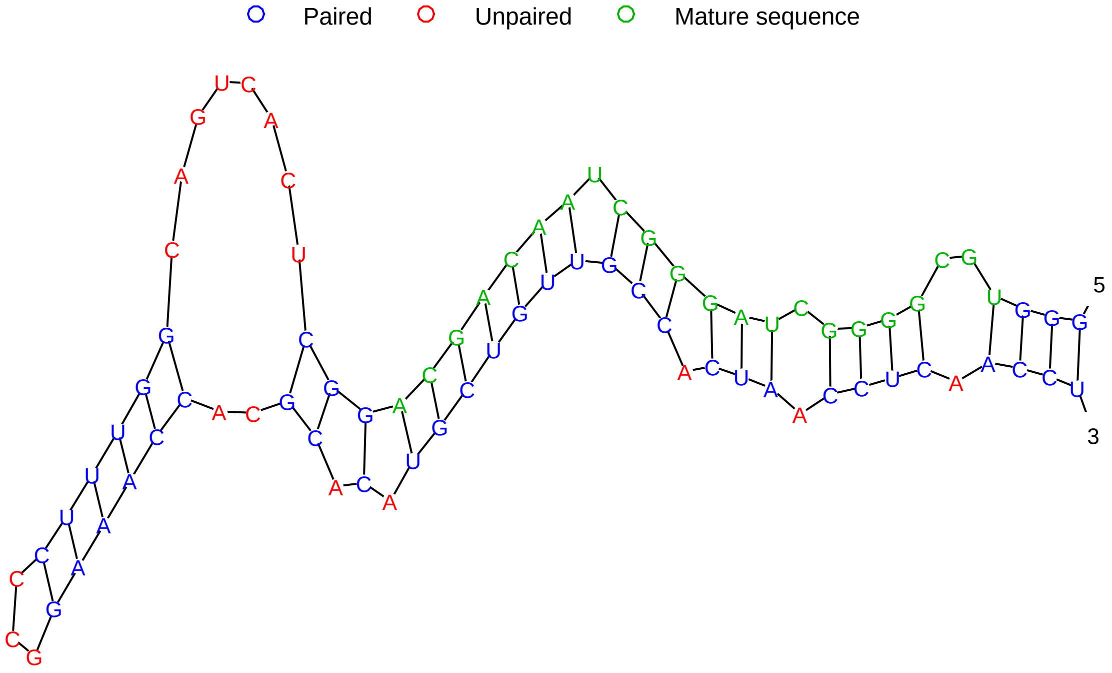

miRNA: bta-miR-744
Stem loop (UMD3.1): chr19:31333521-31333602
Mature (UMD3.1): chr19:31333524-31333545
Mature seq len: 22
Total raw counts (9 samples): 3820
Average raw counts: 425
Strand: Forward
Orientation: 5p
Minimum free energy: -31.60

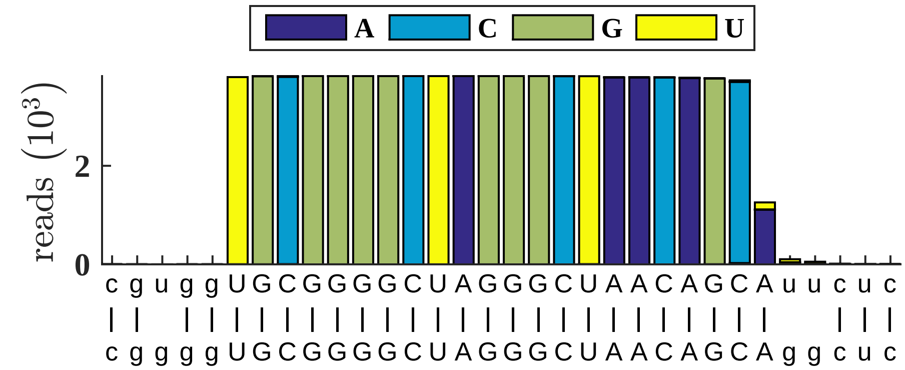

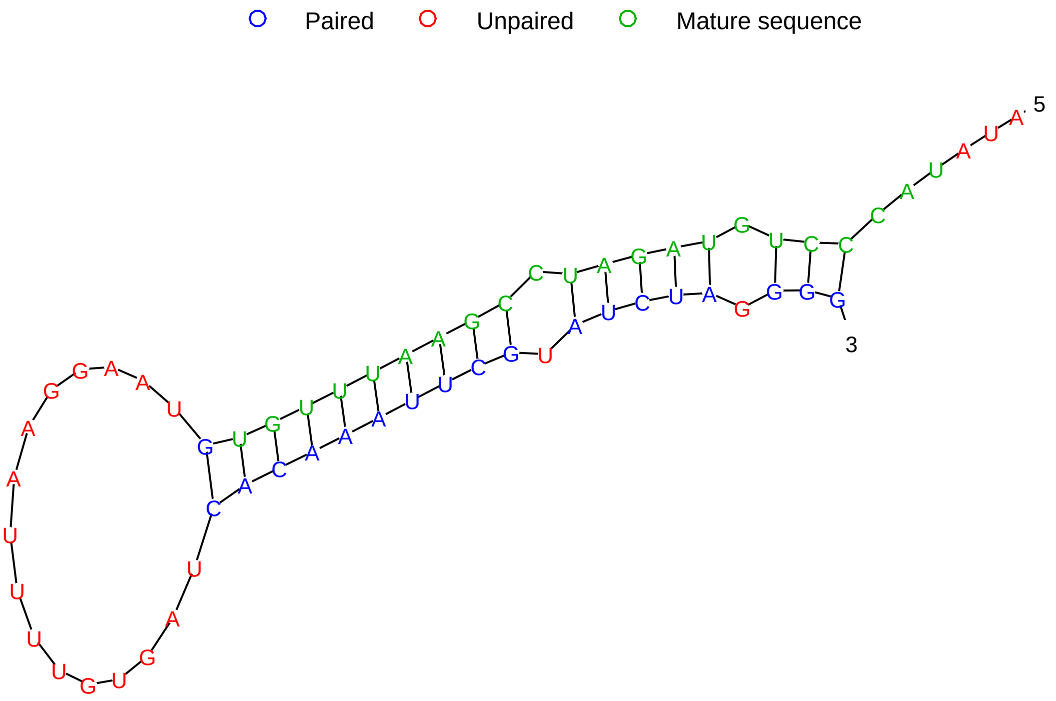

miRNA: bta-miR-10a
Stem loop (UMD3.1): chr19:38549272-38549333
Mature (UMD3.1): chr19:38549275-38549296
Mature seq len: 22
Total raw counts (9 samples): 11453
Average raw counts: 1273
Strand: Forward
Orientation: 5p
Minimum free energy: -21.70

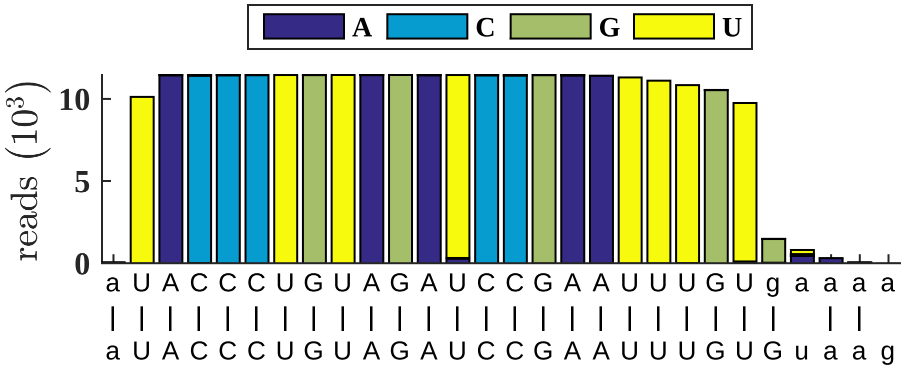

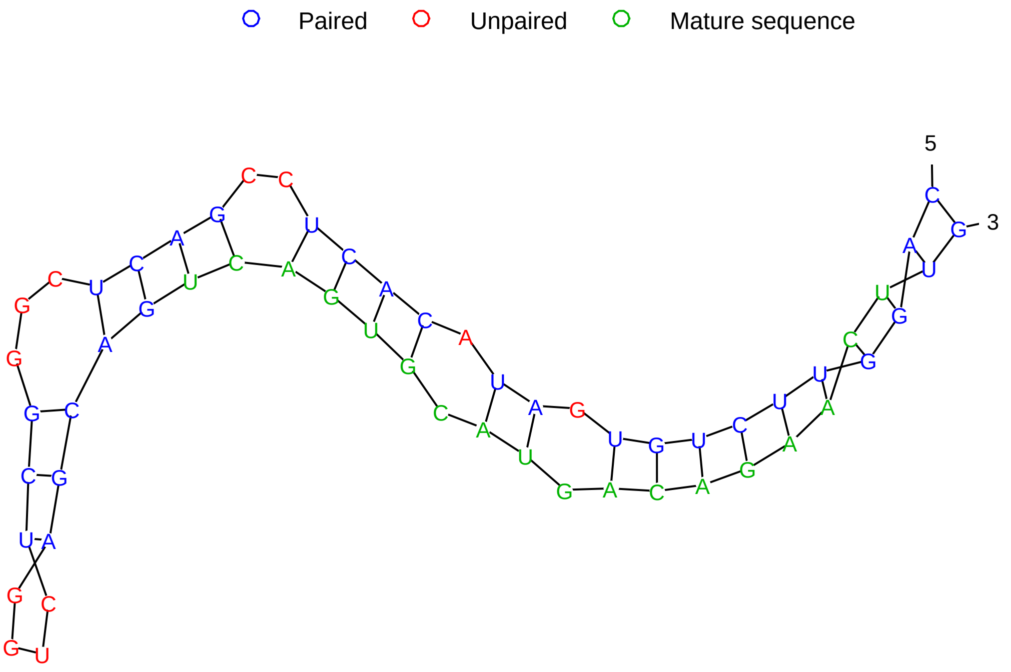

miRNA: bta-miR-152
Stem loop (UMD3.1): chr19:39081178-39081236
Mature (UMD3.1): chr19:39081217-39081234
Mature seq len: 18
Total raw counts (9 samples): 9153
Average raw counts: 1017
Strand: Forward
Orientation: 3p
Minimum free energy: -23.50

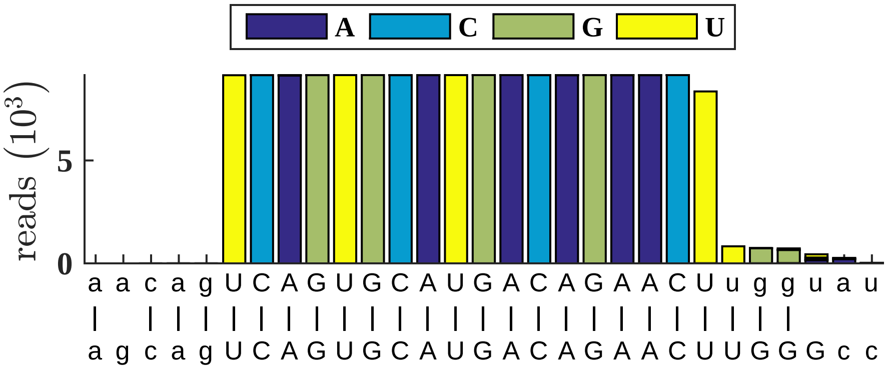

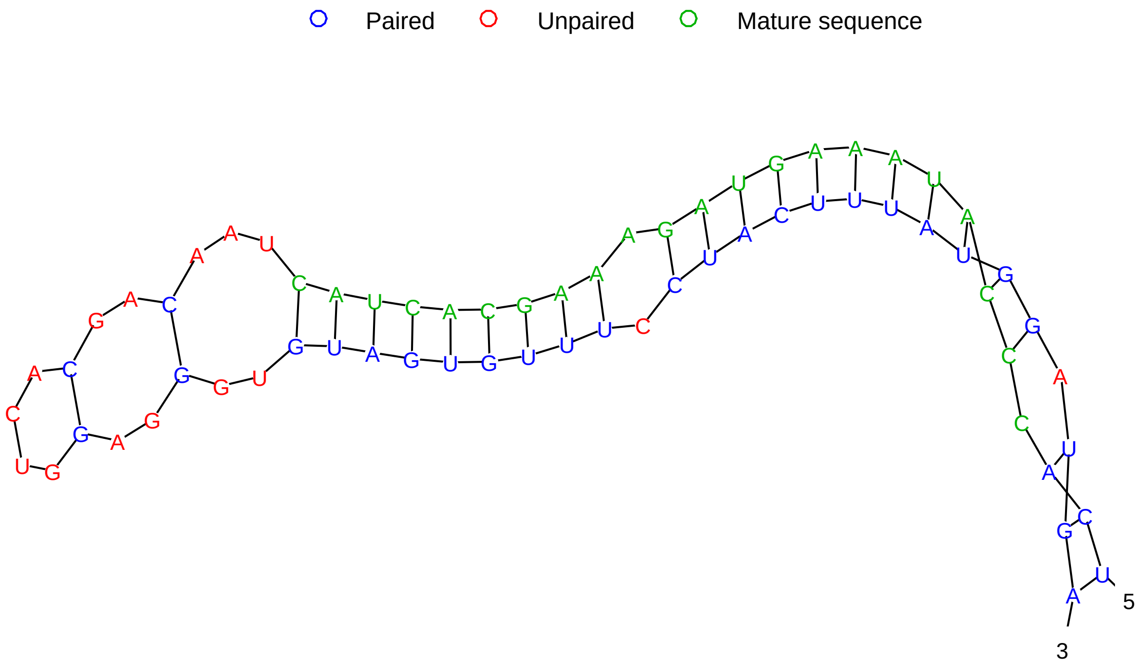

miRNA: bta-miR-142-5p
Stem loop (UMD3.1): chr19:9527315-9527381
Mature (UMD3.1): chr19:9527357-9527378
Mature seq len: 22
Total raw counts (9 samples): 3164
Average raw counts: 352
Strand: Reverse
Orientation: 5p
Minimum free energy: -28.20

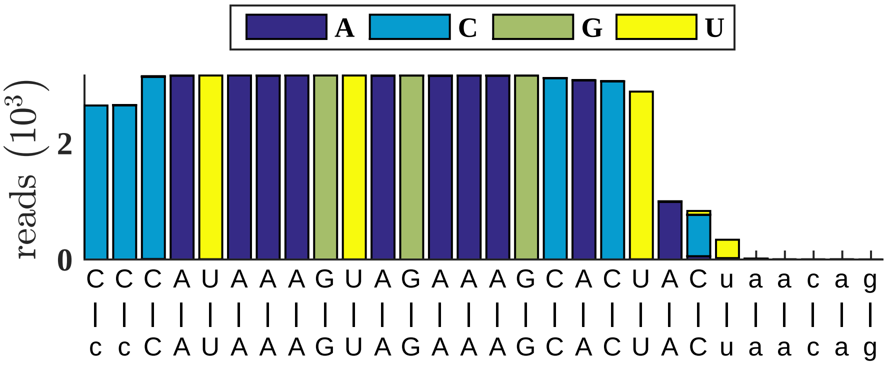

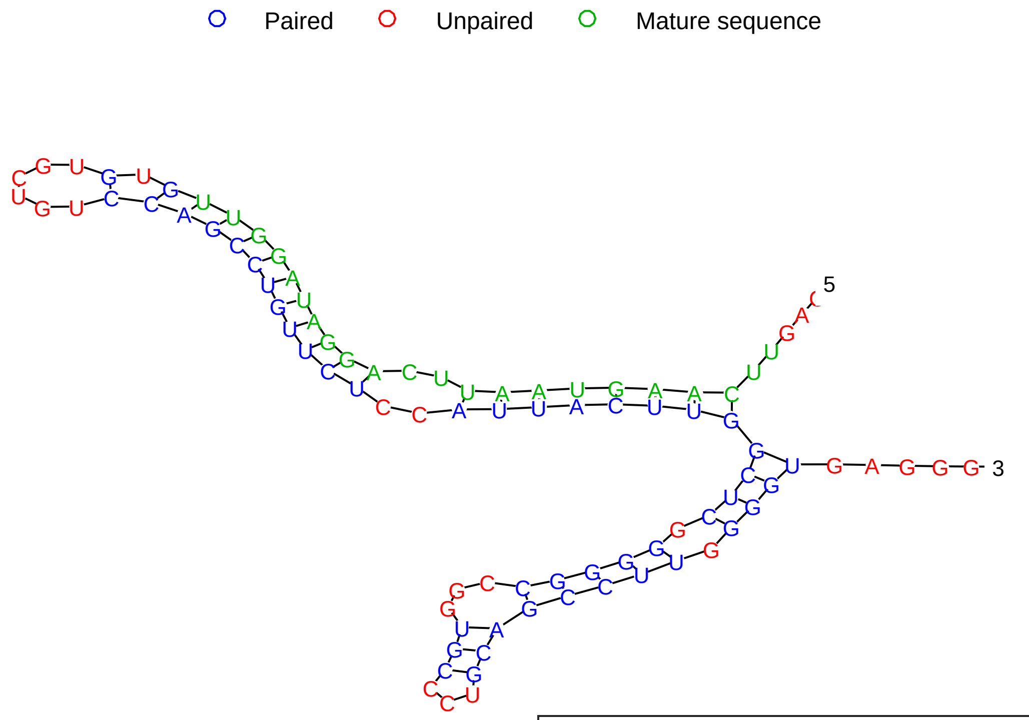

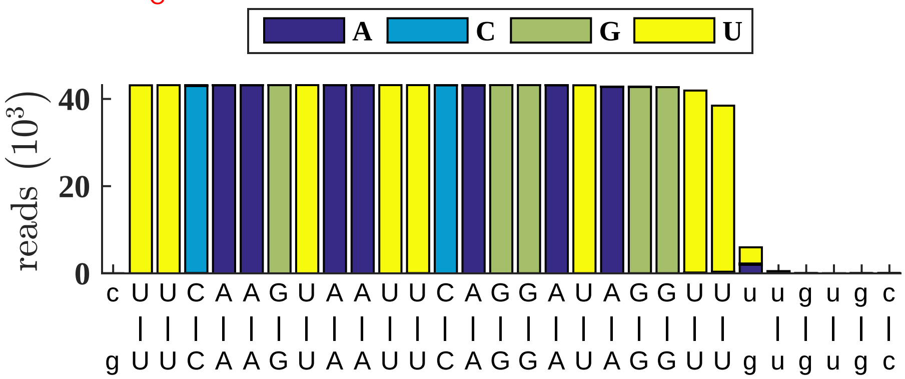

miRNA: bta-miR-26b
Stem loop (UMD3.1): chr2:107133405-107133497
Mature (UMD3.1): chr2:107133408-107133429
Mature seq len: 22
Total raw counts (9 samples): 43170
Average raw counts: 4797
Strand: Forward
Orientation: 5p
Minimum free energy: -36.40

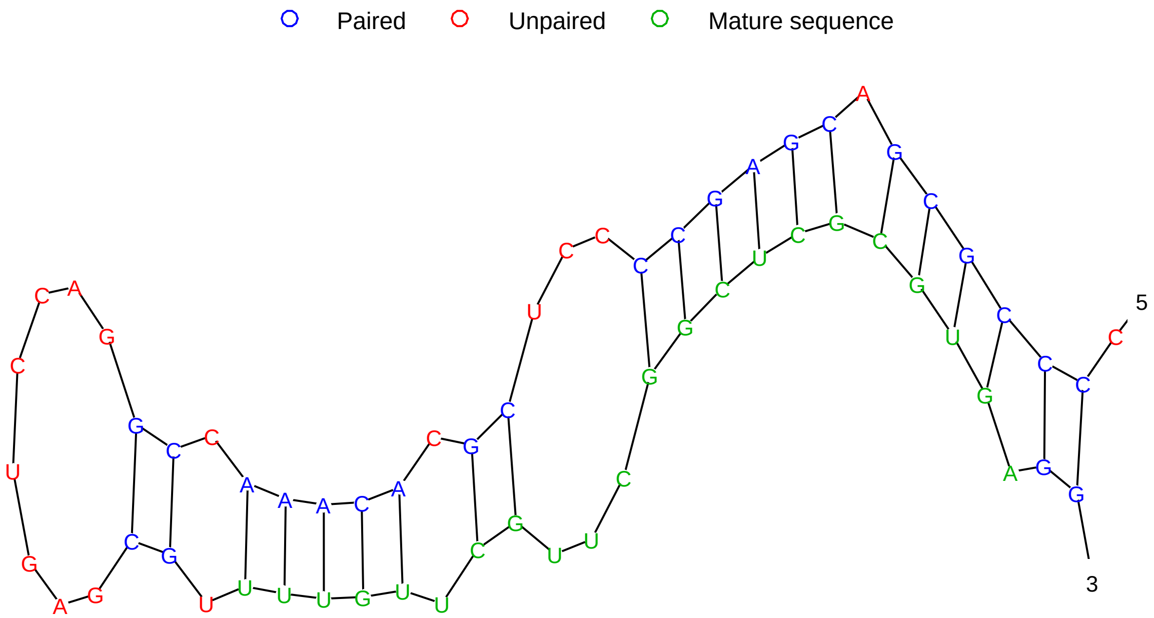

miRNA: bta-miR-375
Stem loop (UMD3.1): chr2:107667522-107667584
Mature (UMD3.1): chr2:107667524-107667545
Mature seq len: 22
Total raw counts (9 samples): 2470
Average raw counts: 275
Strand: Reverse
Orientation: 3p
Minimum free energy: -26.40

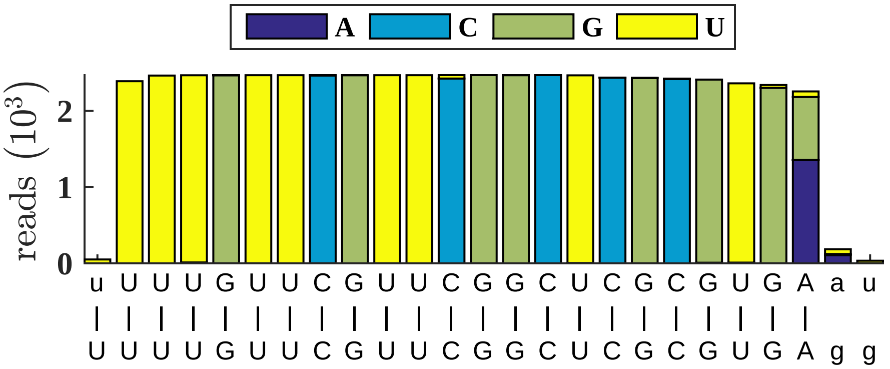

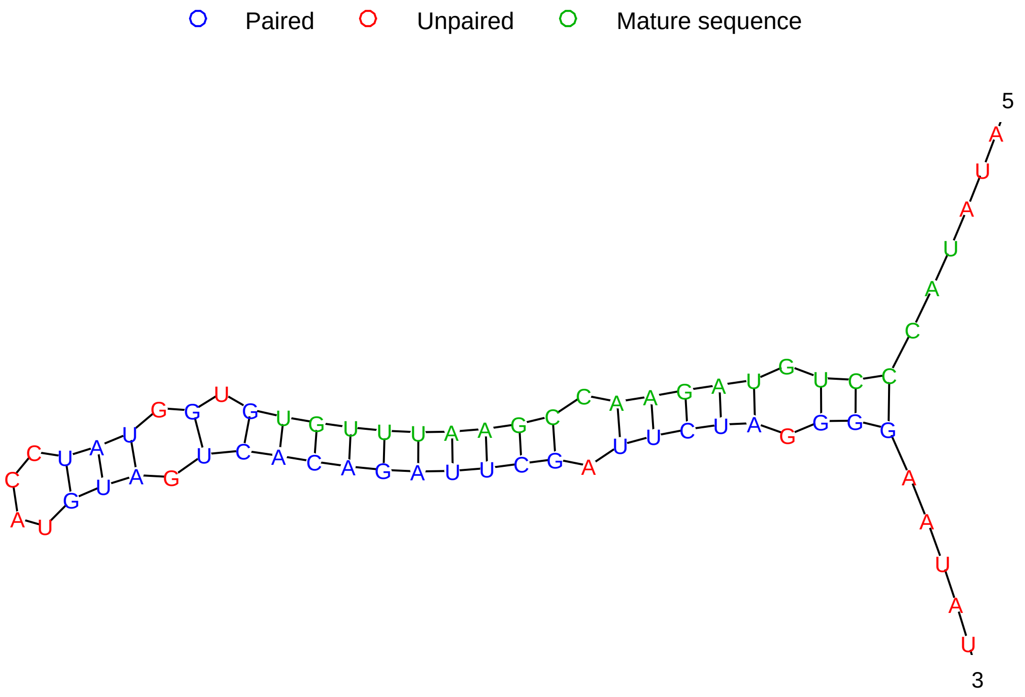

miRNA: bta-miR-10b
Stem loop (UMD3.1): chr2:20797623-20797688
Mature (UMD3.1): chr2:20797664-20797685
Mature seq len: 22
Total raw counts (9 samples): 967617
Average raw counts: 107513
Strand: Reverse
Orientation: 5p
Minimum free energy: -23.70

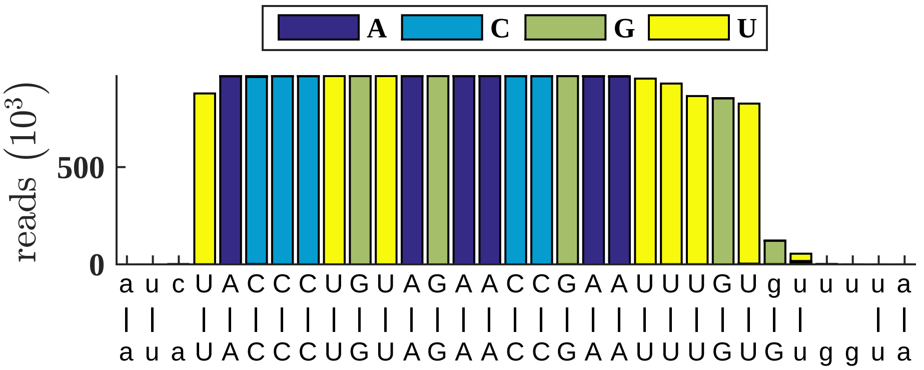

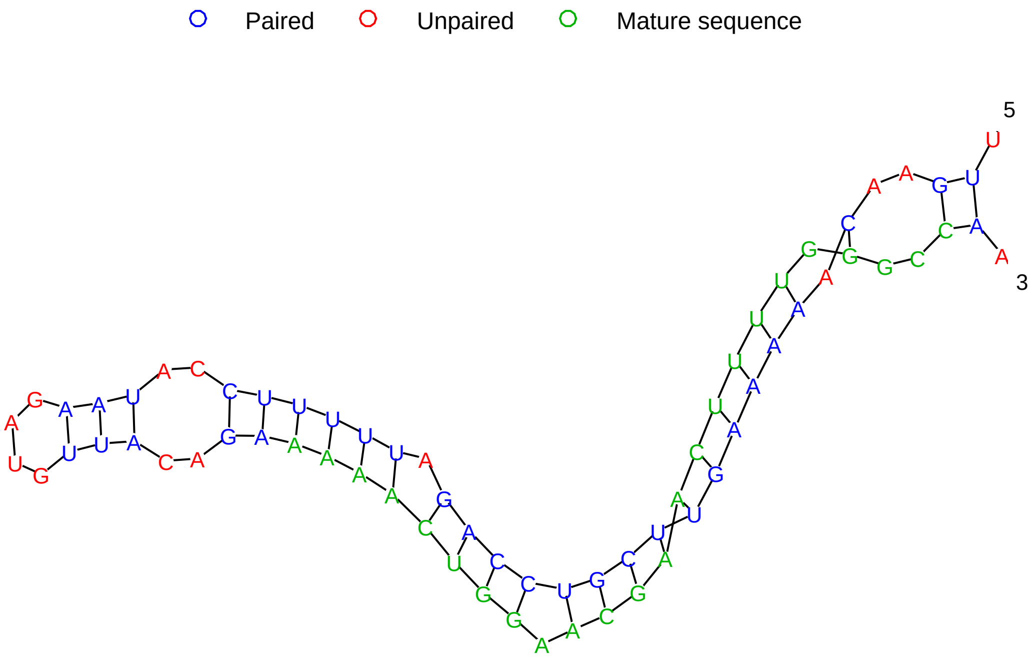

miRNA: bta-miR-2285aa
Stem loop (UMD3.1): chr2:5205388-5205457
Mature (UMD3.1): chr2:5205432-5205455
Mature seq len: 24
Total raw counts (9 samples): 499
Average raw counts: 56
Strand: Forward
Orientation: 3p
Minimum free energy: -23.60

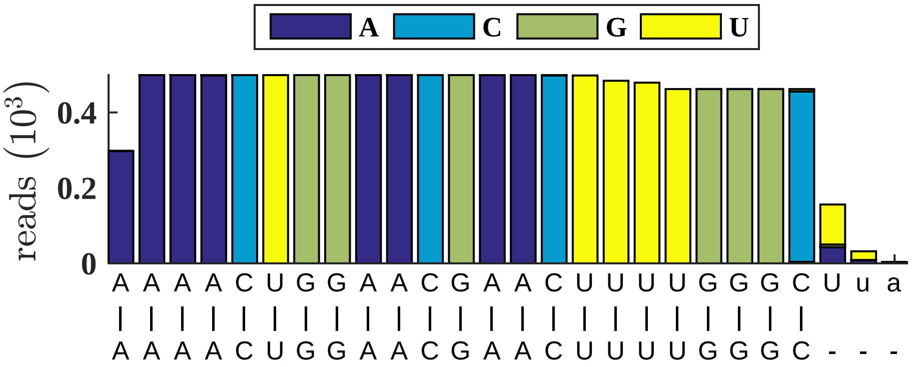

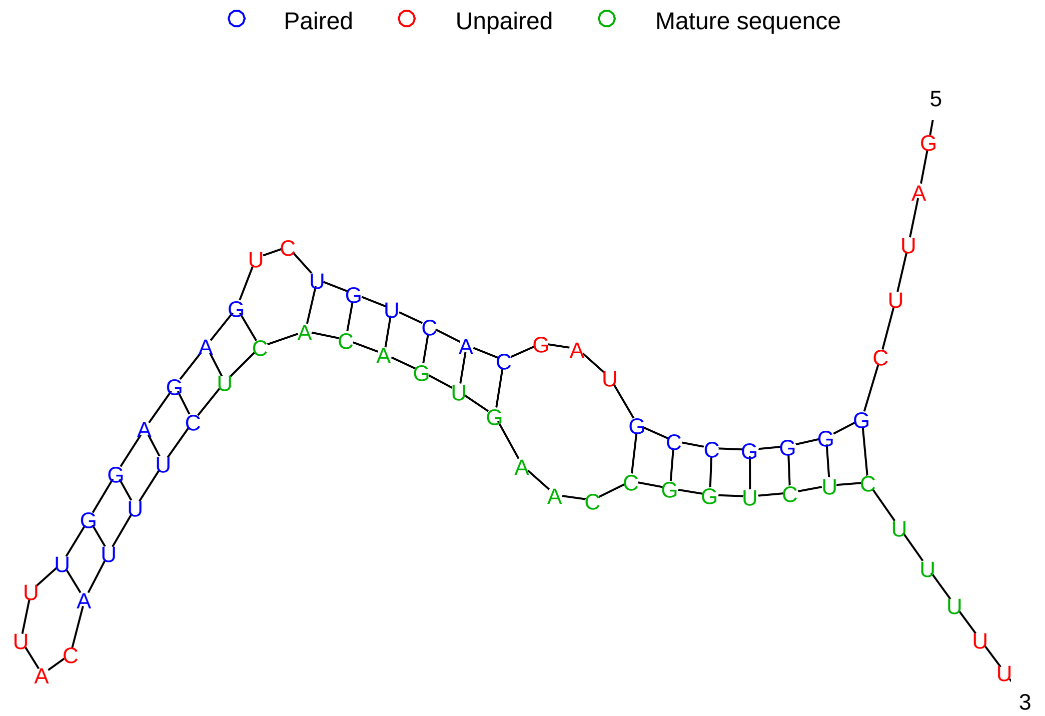

miRNA: bta-miR-128
Stem loop (UMD3.1): chr2:62007762-62007823
Mature (UMD3.1): chr2:62007764-62007784
Mature seq len: 21
Total raw counts (9 samples): 8793
Average raw counts: 977
Strand: Reverse
Orientation: 3p
Minimum free energy: -24.80

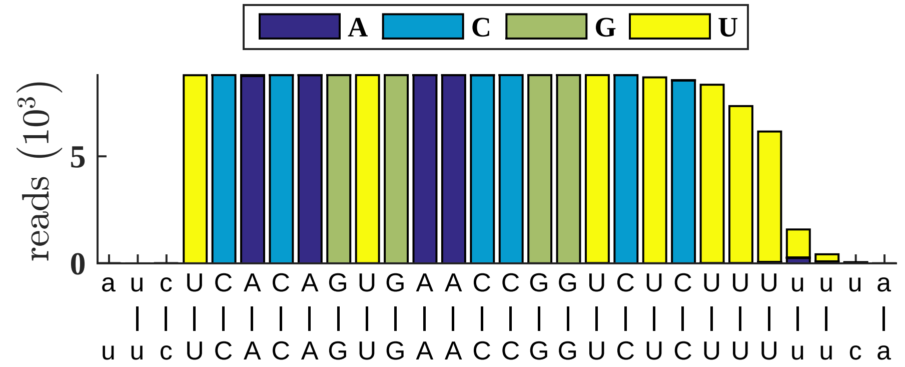

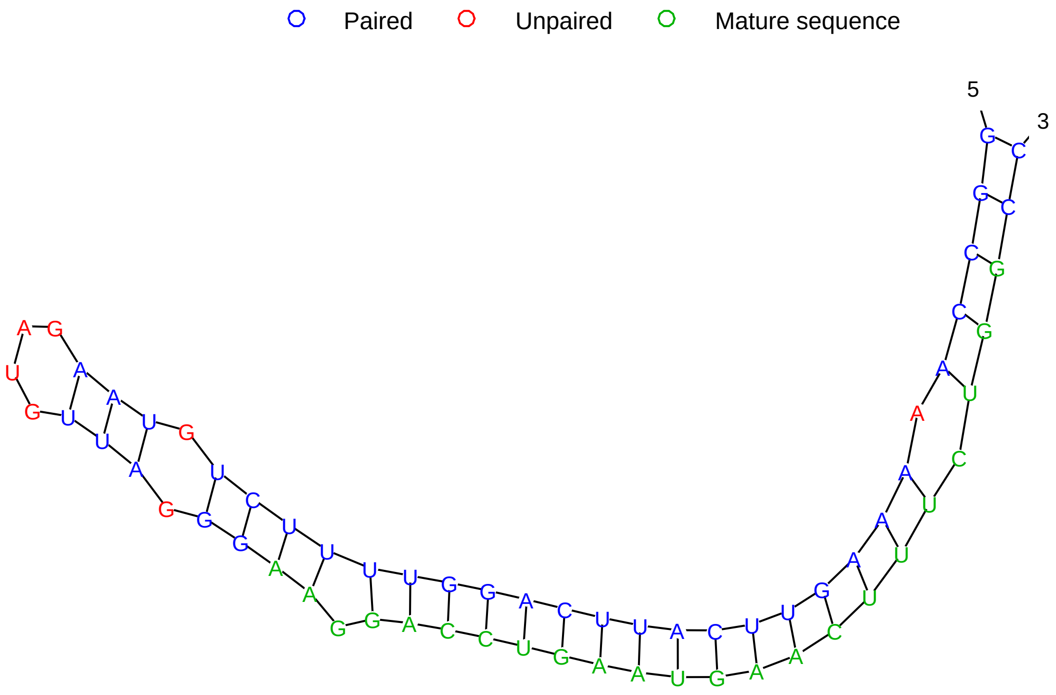

miRNA: bta-miR-2285q
Stem loop (UMD3.1): chr20:13408719-13408783
Mature (UMD3.1): chr20:13408721-13408743
Mature seq len: 23
Total raw counts (9 samples): 1226
Average raw counts: 137
Strand: Reverse
Orientation: 3p
Minimum free energy: -37.70

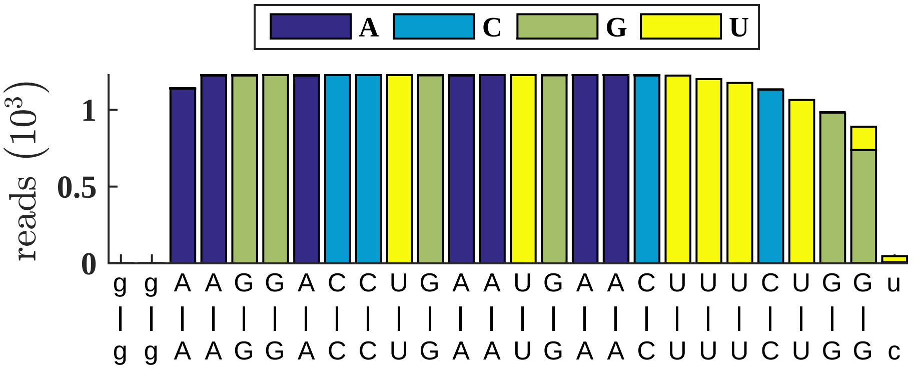

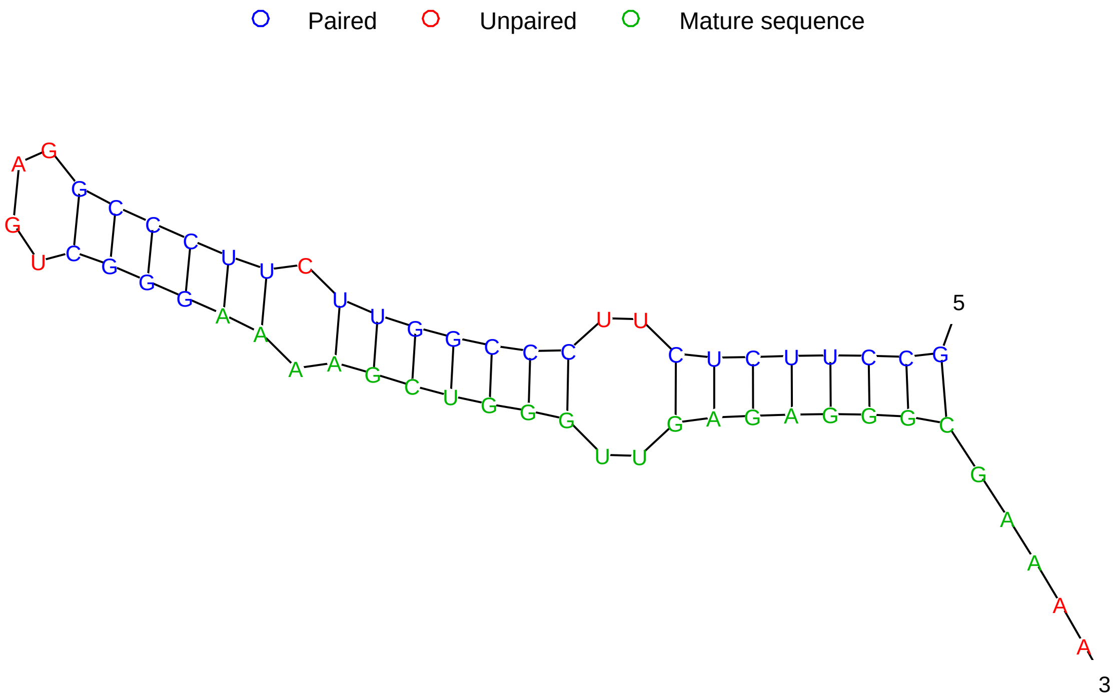

miRNA: bta-miR-320a
Stem loop (UMD3.1): chr20:15213939-15213995
Mature (UMD3.1): chr20:15213971-15213993
Mature seq len: 23
Total raw counts (9 samples): 59289
Average raw counts: 6588
Strand: Forward
Orientation: 3p
Minimum free energy: -38.10

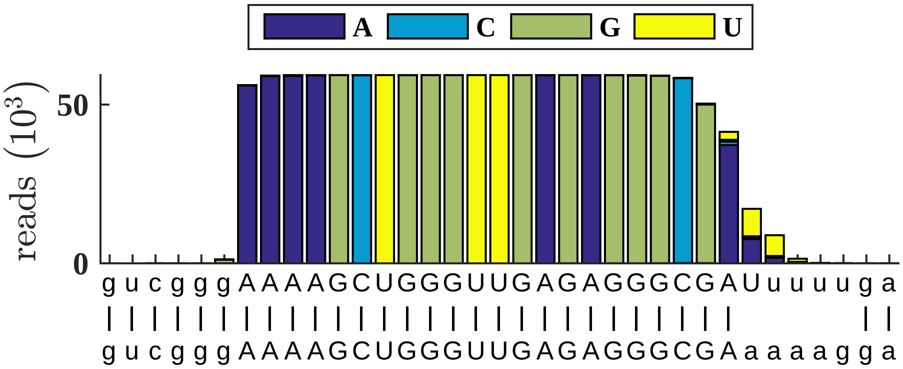

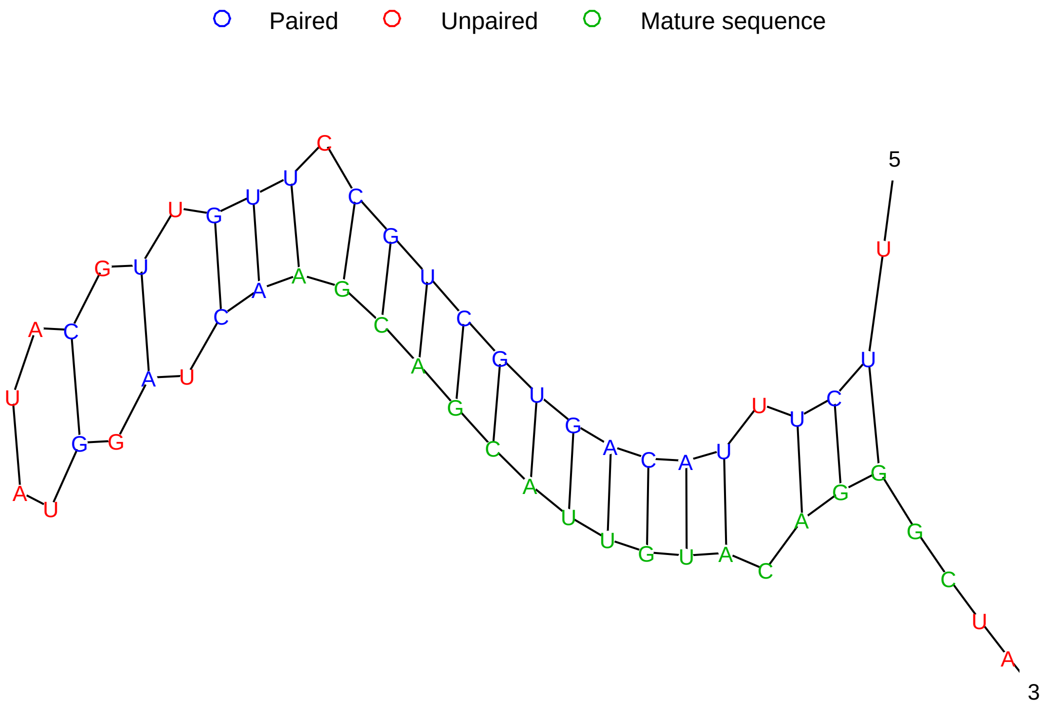

miRNA: bta-miR-103
Stem loop (UMD3.1): chr20:189864-189917
Mature (UMD3.1): chr20:189866-189883
Mature seq len: 18
Total raw counts (9 samples): 78511
Average raw counts: 8724
Strand: Reverse
Orientation: 3p
Minimum free energy: -20.70

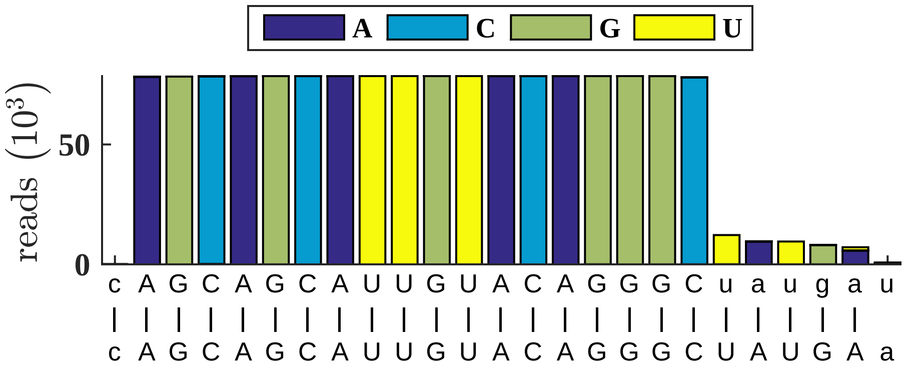

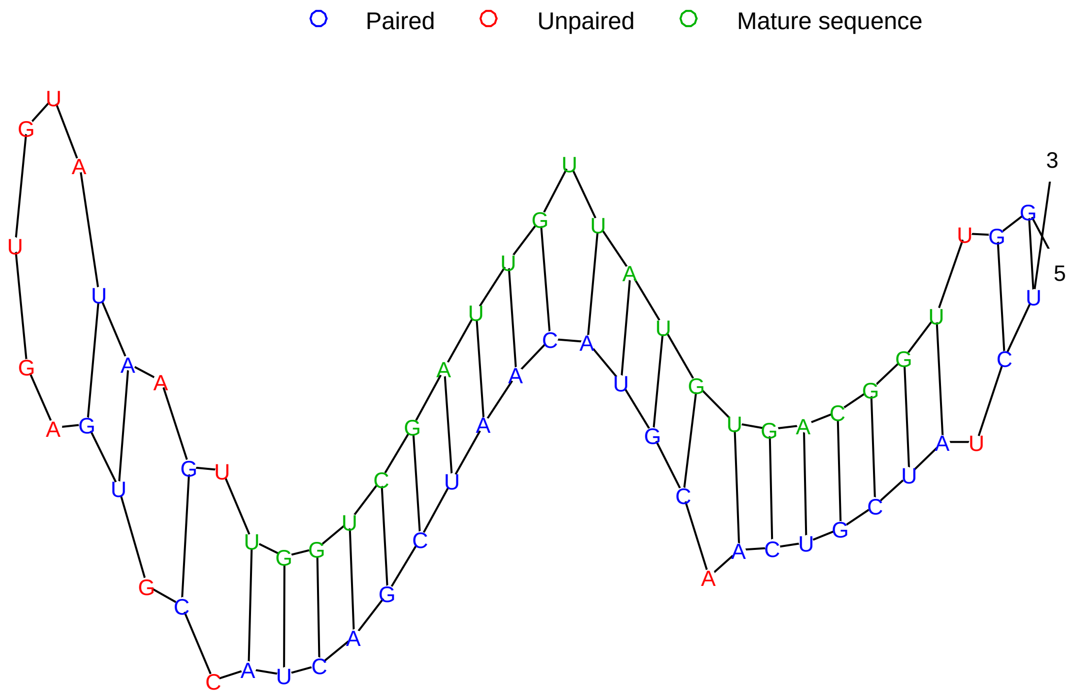

miRNA: bta-miR-449a
Stem loop (UMD3.1): chr20:23967402-23967467
Mature (UMD3.1): chr20:23967405-23967426
Mature seq len: 22
Total raw counts (9 samples): 1053
Average raw counts: 117
Strand: Forward
Orientation: 5p
Minimum free energy: -26.00

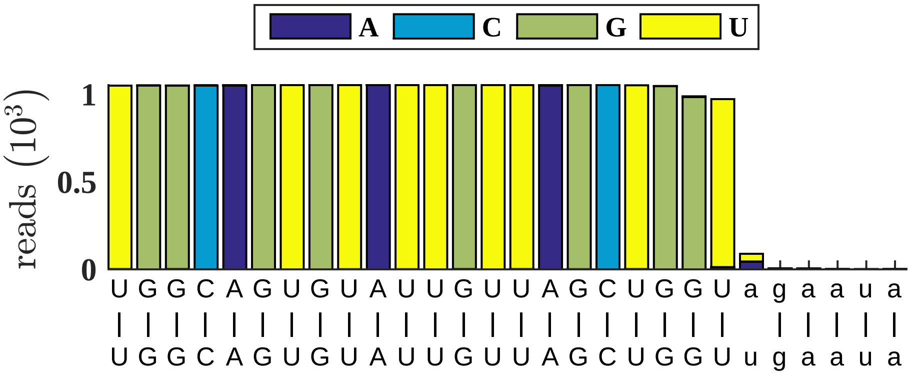

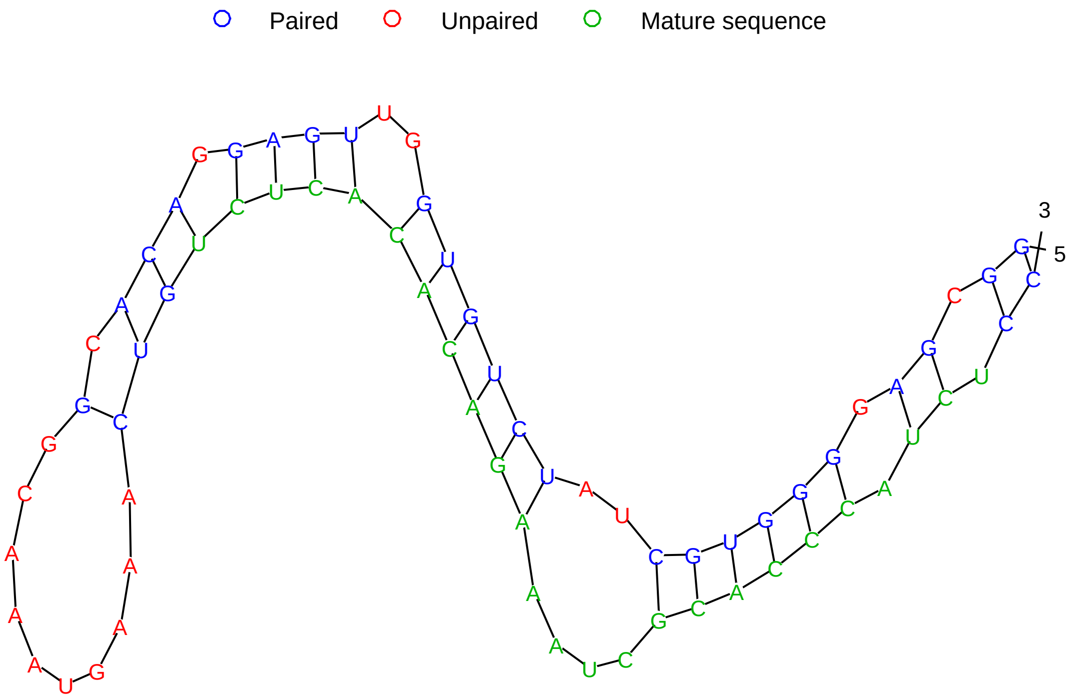

miRNA: bta-miR-342
Stem loop (UMD3.1): chr21:66706863-66706934
Mature (UMD3.1): chr21:66706908-66706932
Mature seq len: 25
Total raw counts (9 samples): 6547
Average raw counts: 728
Strand: Forward
Orientation: 3p
Minimum free energy: -26.30

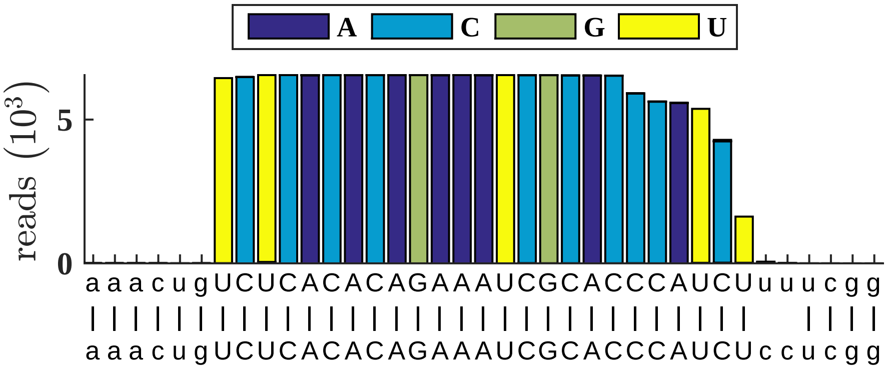

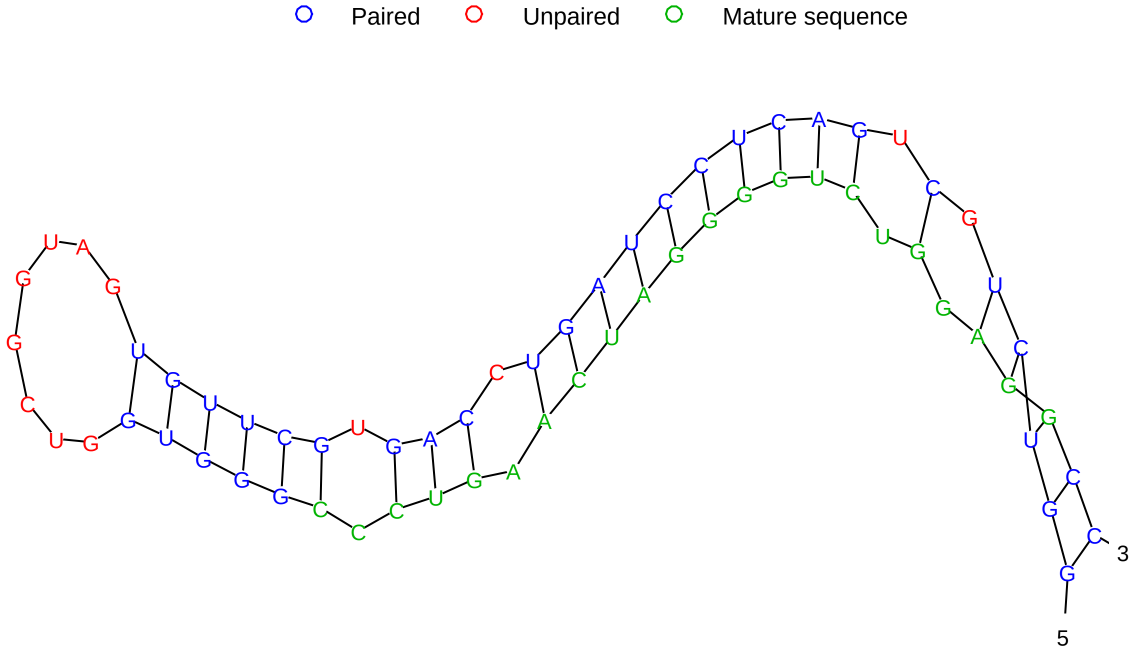

miRNA: bta-miR-345-3p
Stem loop (UMD3.1): chr21:66867097-66867162
Mature (UMD3.1): chr21:66867139-66867160
Mature seq len: 22
Total raw counts (9 samples): 1357
Average raw counts: 151
Strand: Forward
Orientation: 3p
Minimum free energy: -32.00

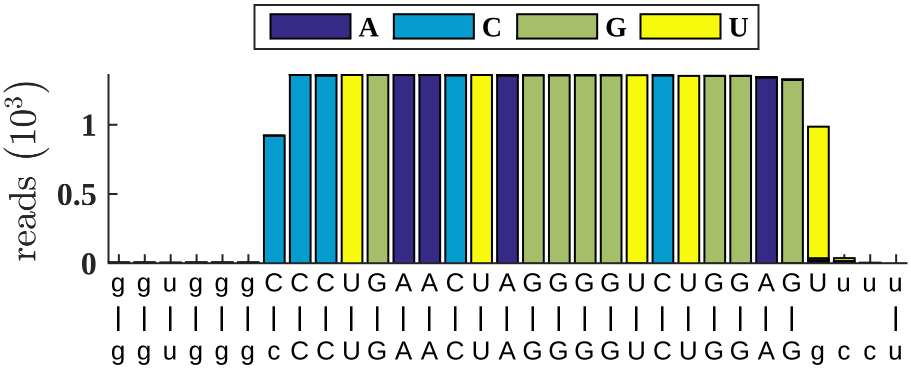

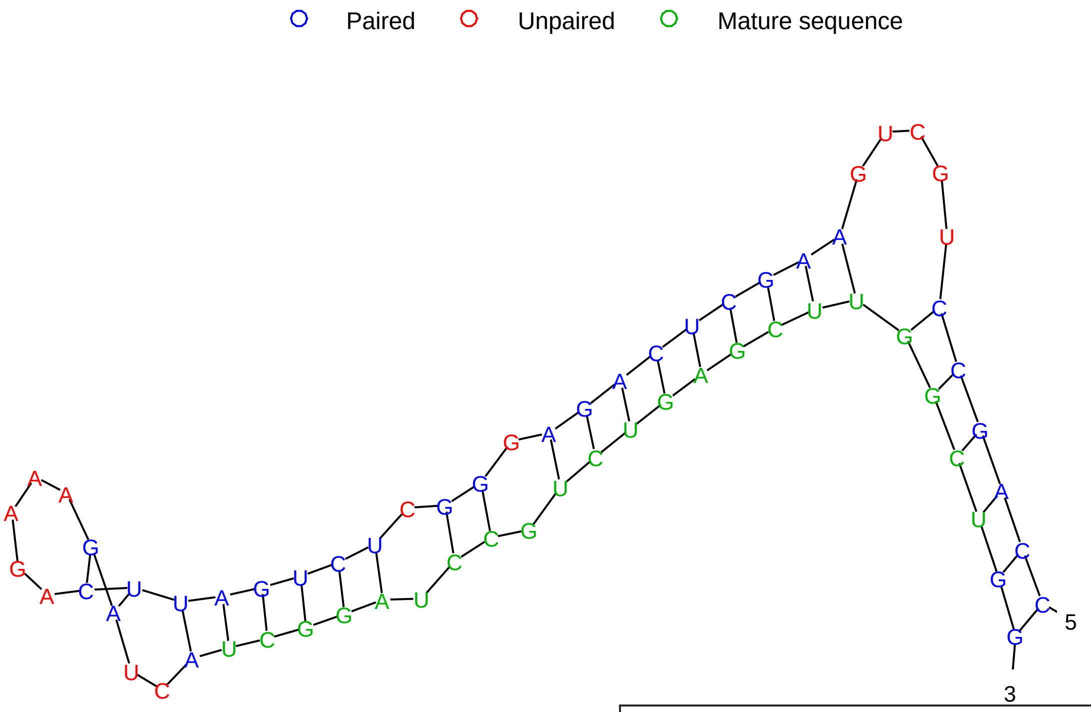

miRNA: bta-miR-127
Stem loop (UMD3.1): chr21:67429756-67429821
Mature (UMD3.1): chr21:67429798-67429819
Mature seq len: 22
Total raw counts (9 samples): 5106
Average raw counts: 568
Strand: Forward
Orientation: 3p
Minimum free energy: -34.50

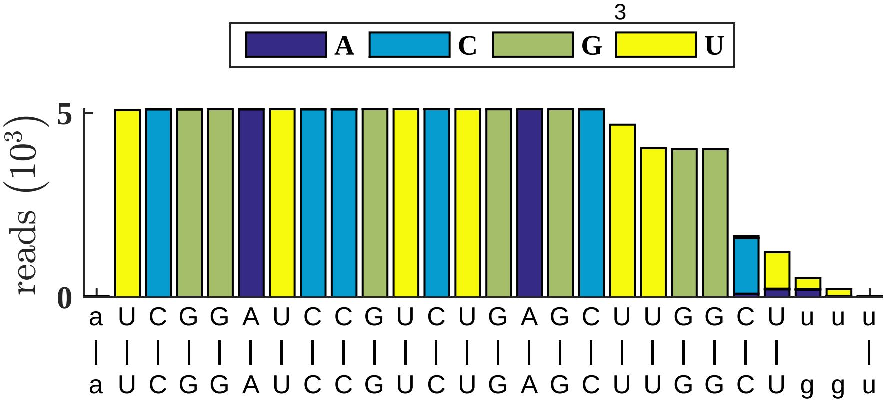

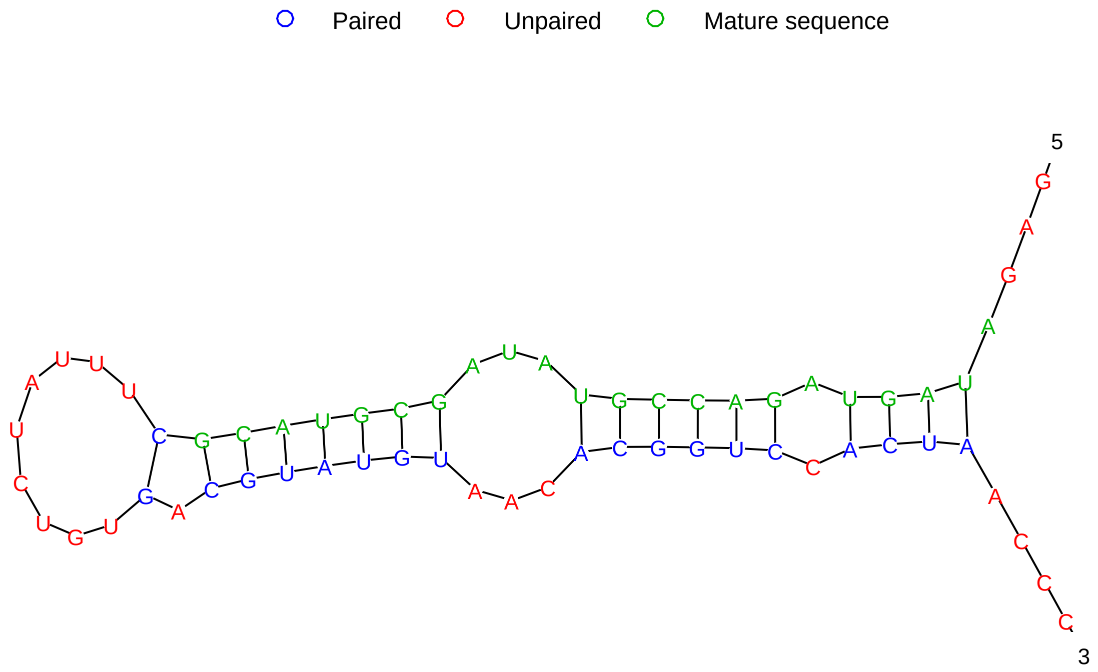

miRNA: bta-miR-411a
Stem loop (UMD3.1): chr21:67563109-67563170
Mature (UMD3.1): chr21:67563112-67563133
Mature seq len: 22
Total raw counts (9 samples): 615
Average raw counts: 69
Strand: Forward
Orientation: 5p
Minimum free energy: -20.30

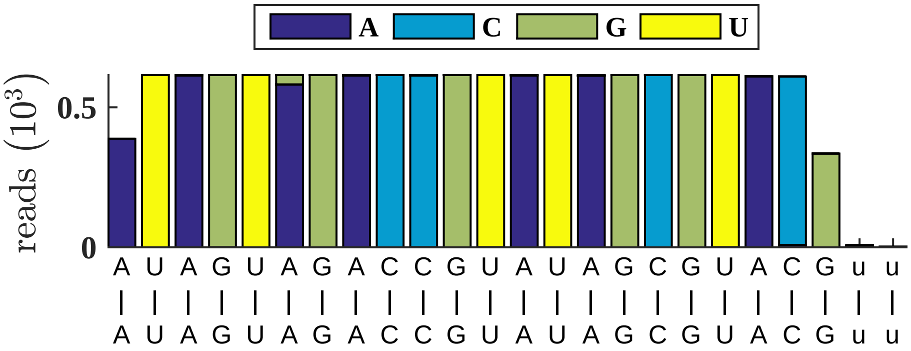

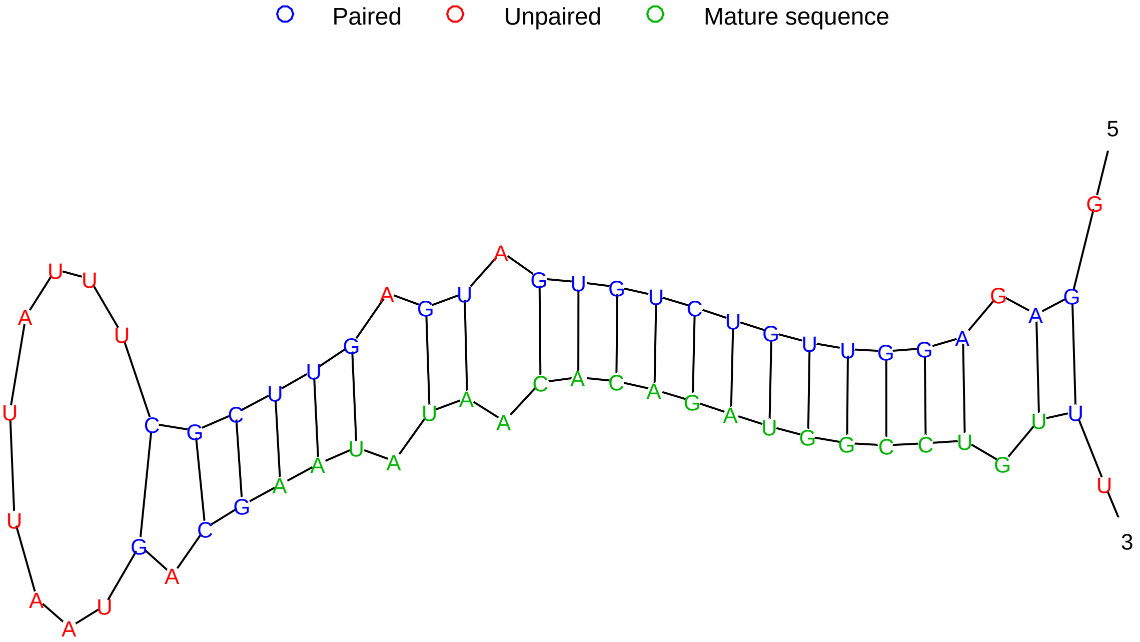

miRNA: bta-miR-410
Stem loop (UMD3.1): chr21:67603875-67603936
Mature (UMD3.1): chr21:67603914-67603934
Mature seq len: 21
Total raw counts (9 samples): 363
Average raw counts: 41
Strand: Forward
Orientation: 3p
Minimum free energy: -22.90

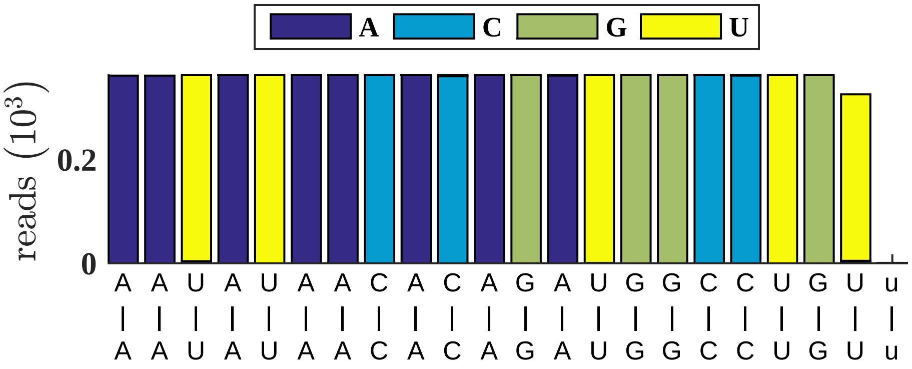

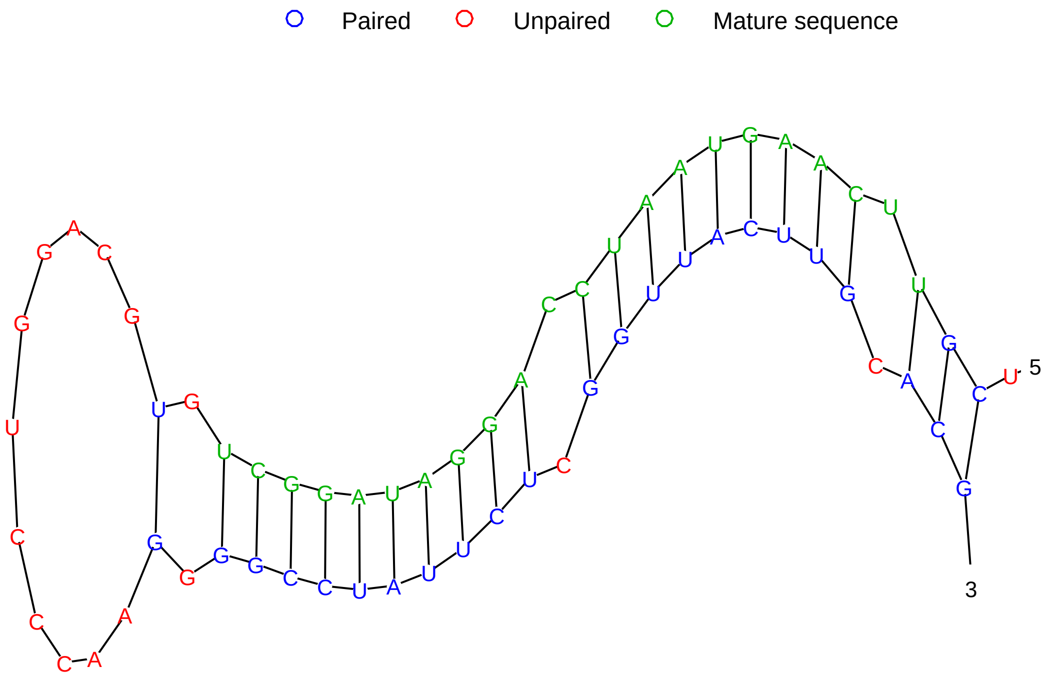

miRNA: bta-miR-26a
Stem loop (UMD3.1): chr22:11457912-11457975
Mature (UMD3.1): chr22:11457915-11457936
Mature seq len: 22
Total raw counts (9 samples): 203155
Average raw counts: 22573
Strand: Forward
Orientation: 5p
Minimum free energy: -27.90

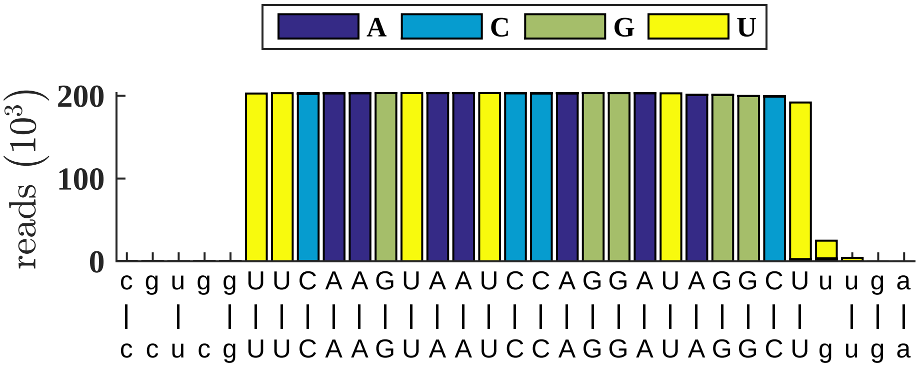

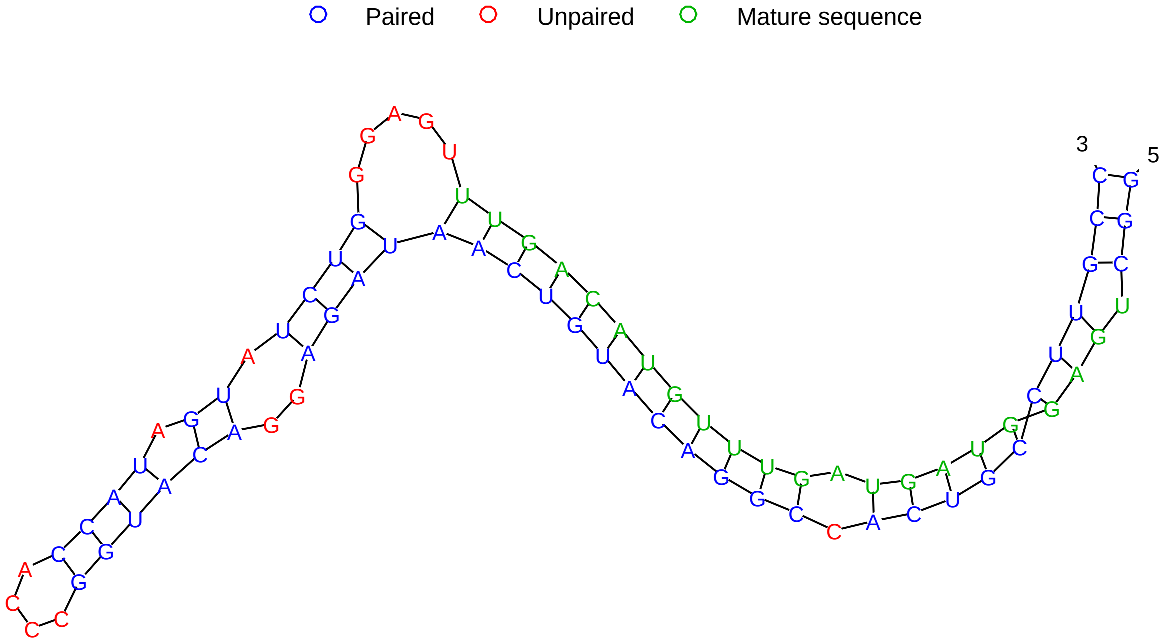

miRNA: bta-let-7g
Stem loop (UMD3.1): chr22:49189341-49189422
Mature (UMD3.1): chr22:49189344-49189365
Mature seq len: 22
Total raw counts (9 samples): 79405
Average raw counts: 8823
Strand: Forward
Orientation: 5p
Minimum free energy: -37.80

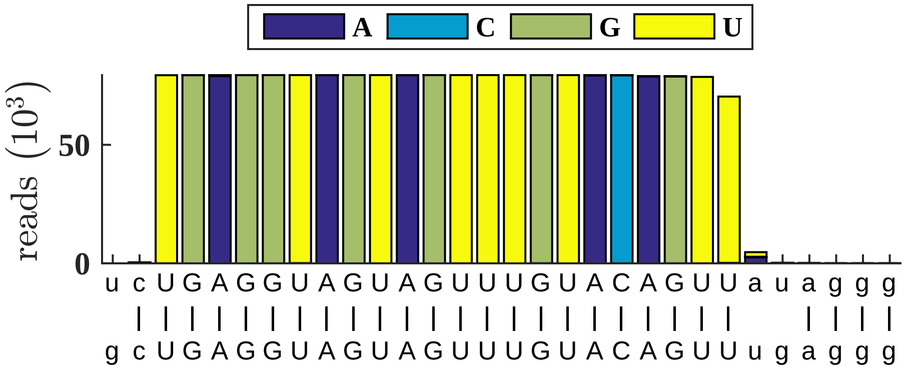

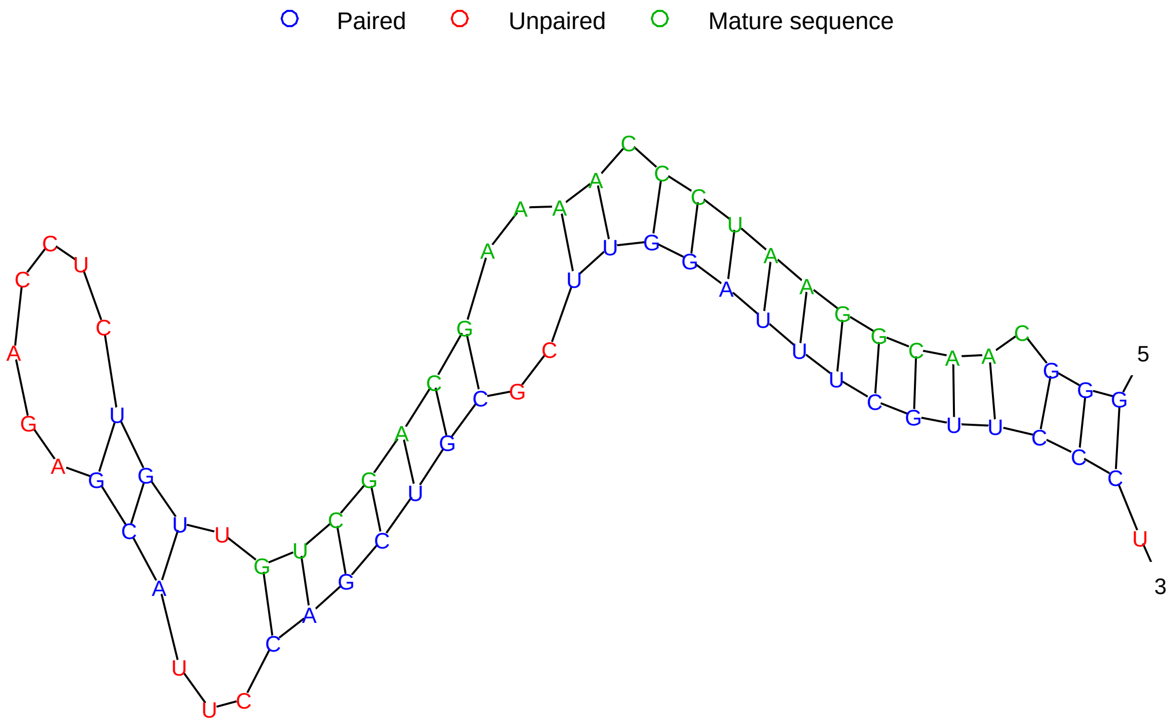

miRNA: bta-miR-191
Stem loop (UMD3.1): chr22:51543481-51543548
Mature (UMD3.1): chr22:51543484-51543506
Mature seq len: 23
Total raw counts (9 samples): 310627
Average raw counts: 34515
Strand: Forward
Orientation: 5p
Minimum free energy: -31.70

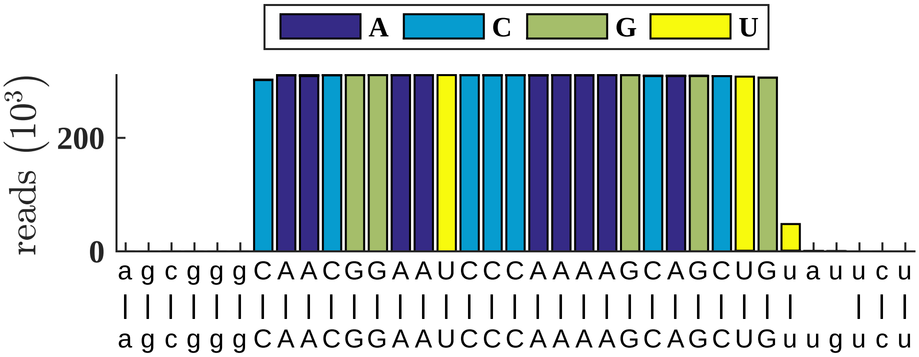

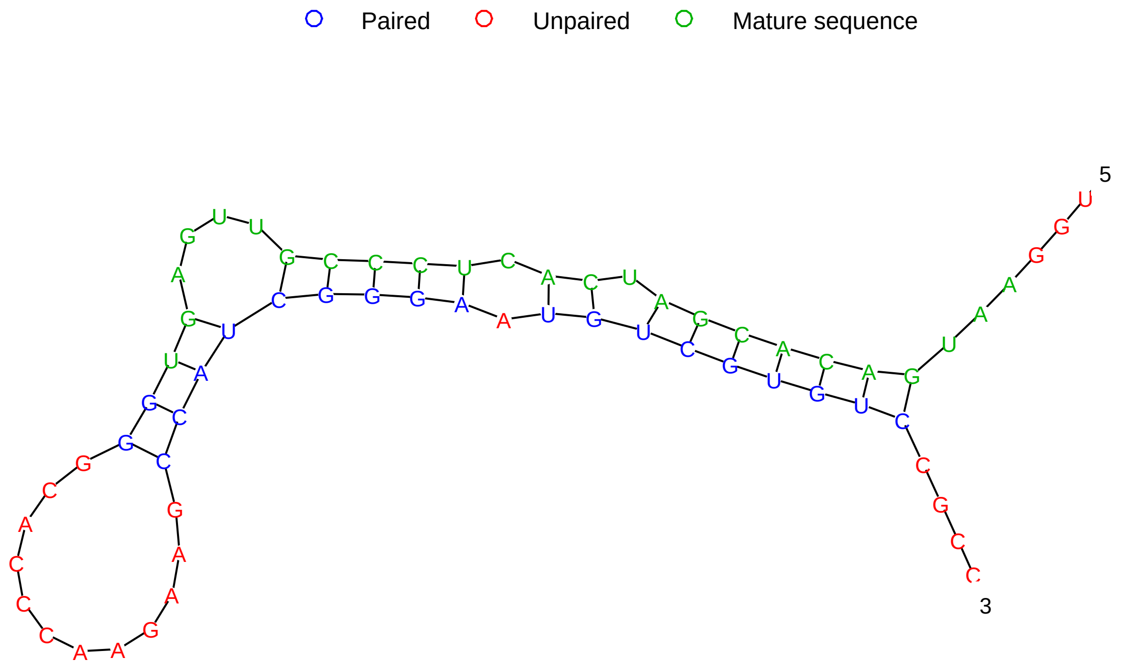

miRNA: bta-miR-425-5p
Stem loop (UMD3.1): chr22:51543962-51544026
Mature (UMD3.1): chr22:51543965-51543989
Mature seq len: 25
Total raw counts (9 samples): 8482
Average raw counts: 943
Strand: Forward
Orientation: 5p
Minimum free energy: -22.70

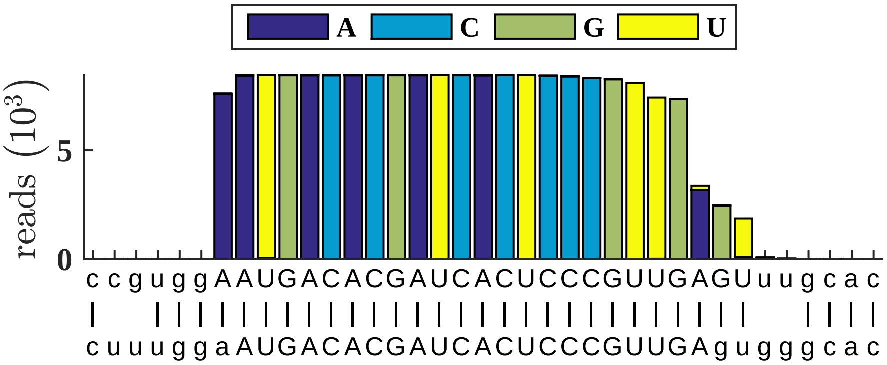

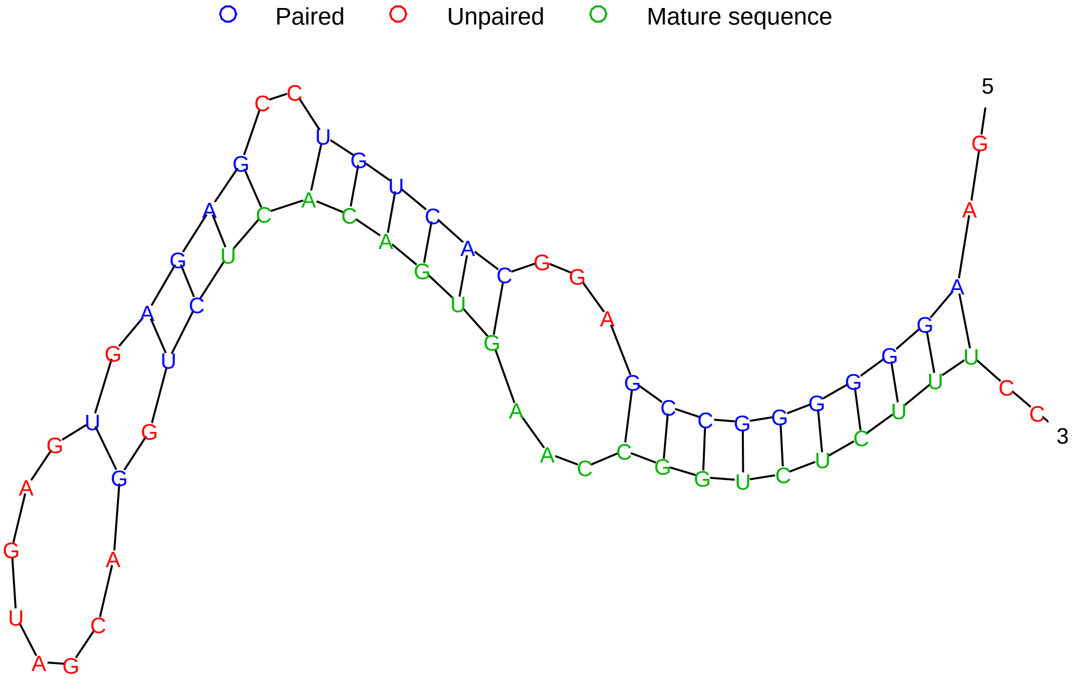

miRNA: bta-miR-128
Stem loop (UMD3.1): chr22:9738608-9738671
Mature (UMD3.1): chr22:9738649-9738669
Mature seq len: 21
Total raw counts (9 samples): 7092
Average raw counts: 788
Strand: Forward
Orientation: 3p
Minimum free energy: -25.90

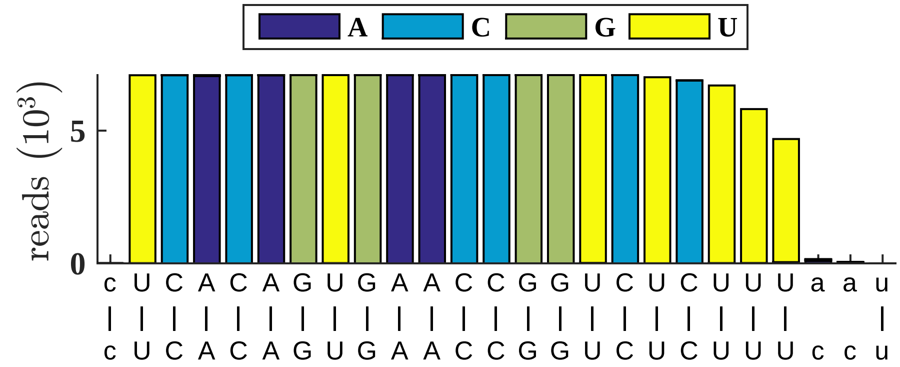

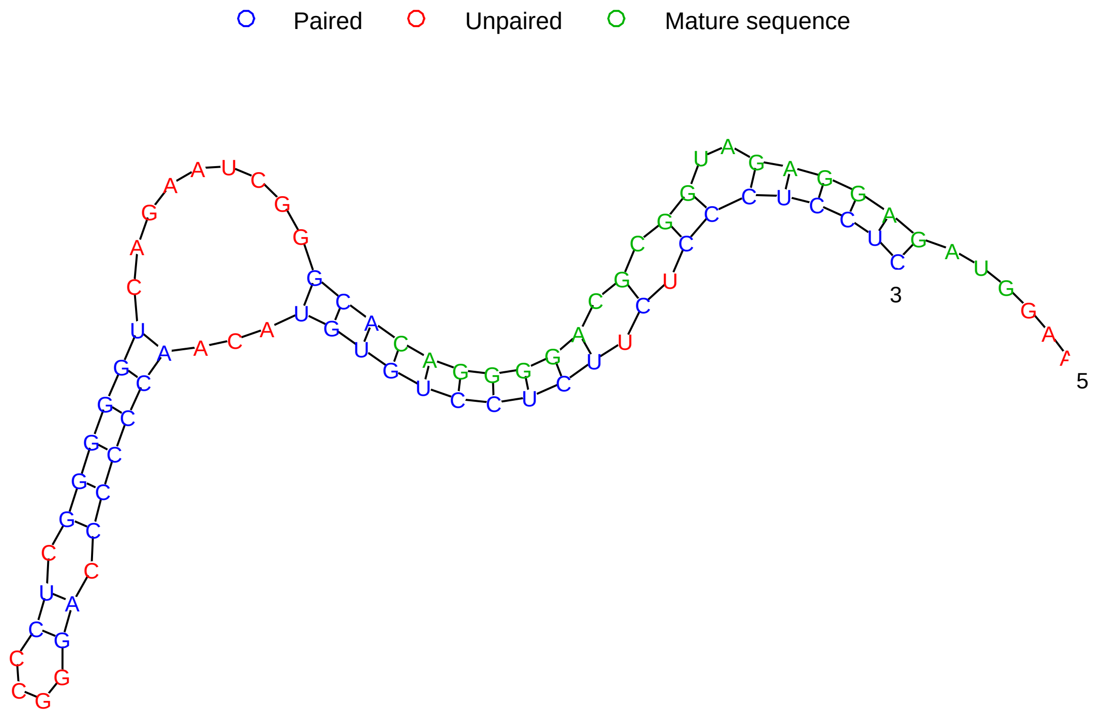

miRNA: bta-miR-877
Stem loop (UMD3.1): chr23:28203162-28203245
Mature (UMD3.1): chr23:28203220-28203242
Mature seq len: 23
Total raw counts (9 samples): 3208
Average raw counts: 357
Strand: Reverse
Orientation: 5p
Minimum free energy: -36.90

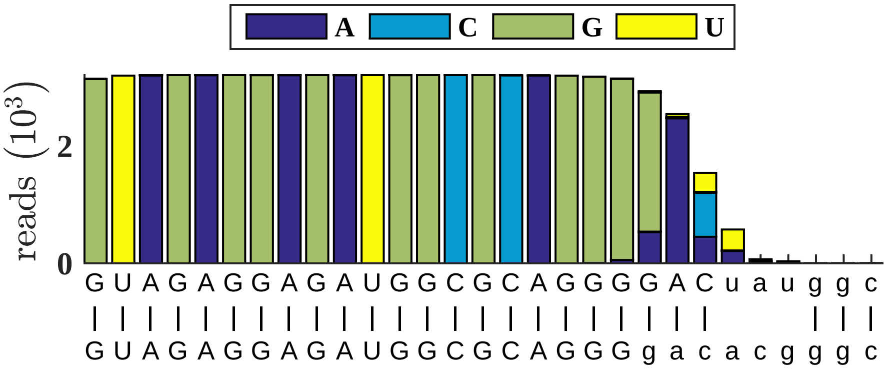

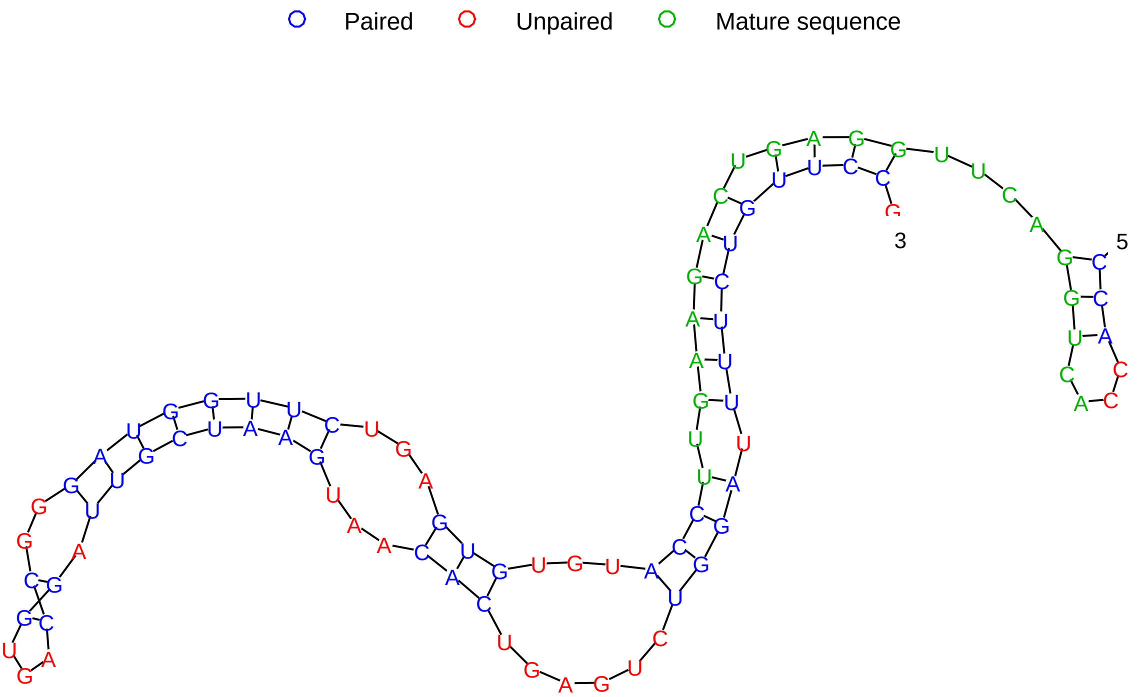

miRNA: bta-miR-378c
Stem loop (UMD3.1): chr24:31729520-31729612
Mature (UMD3.1): chr24:31729586-31729607
Mature seq len: 22
Total raw counts (9 samples): 905
Average raw counts: 101
Strand: Reverse
Orientation: 5p
Minimum free energy: -27.10

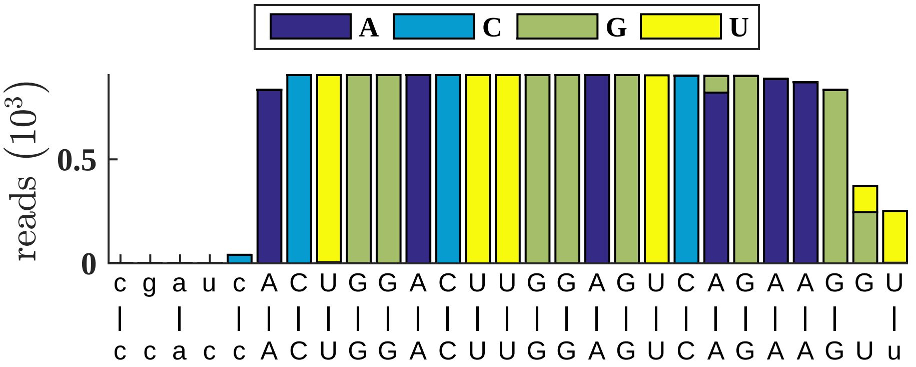

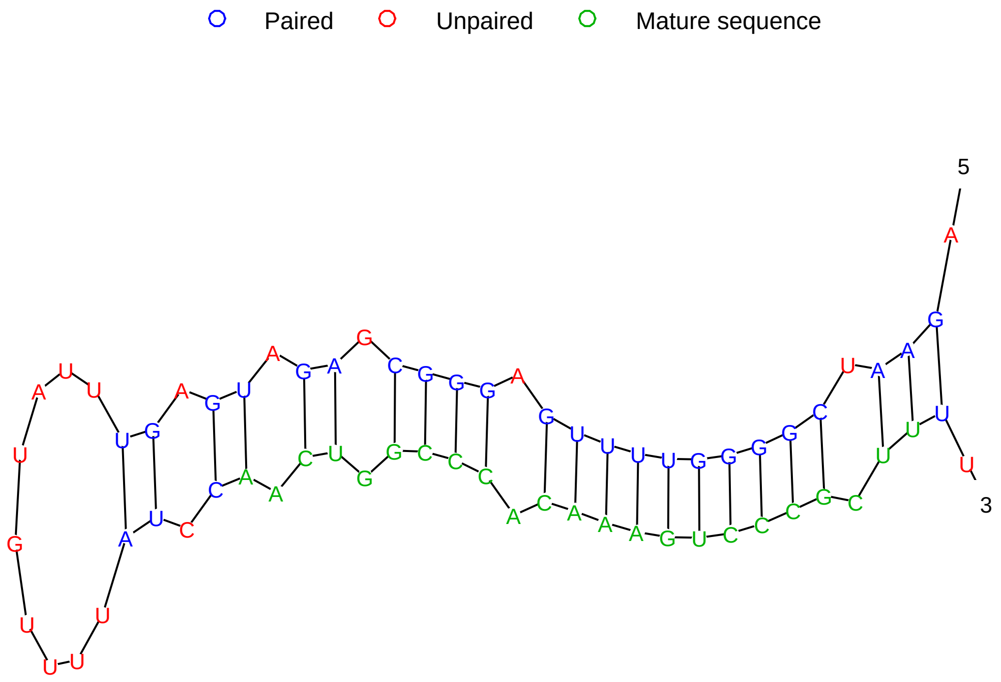

miRNA: bta-miR-193b
Stem loop (UMD3.1): chr25:13339491-13339557
Mature (UMD3.1): chr25:13339533-13339555
Mature seq len: 23
Total raw counts (9 samples): 2372
Average raw counts: 264
Strand: Forward
Orientation: 3p
Minimum free energy: -23.10

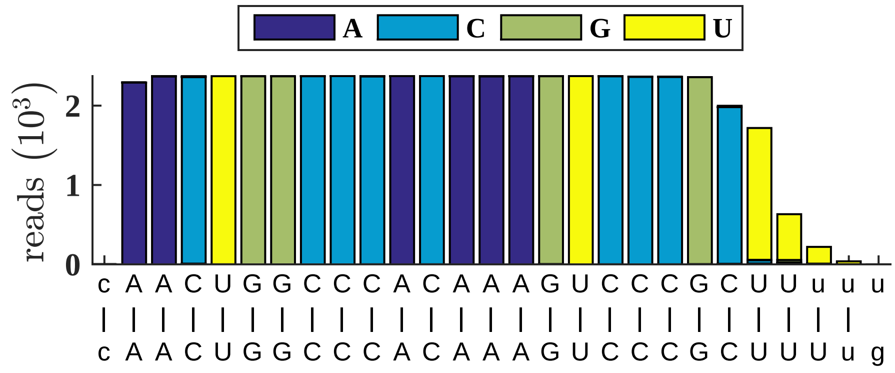

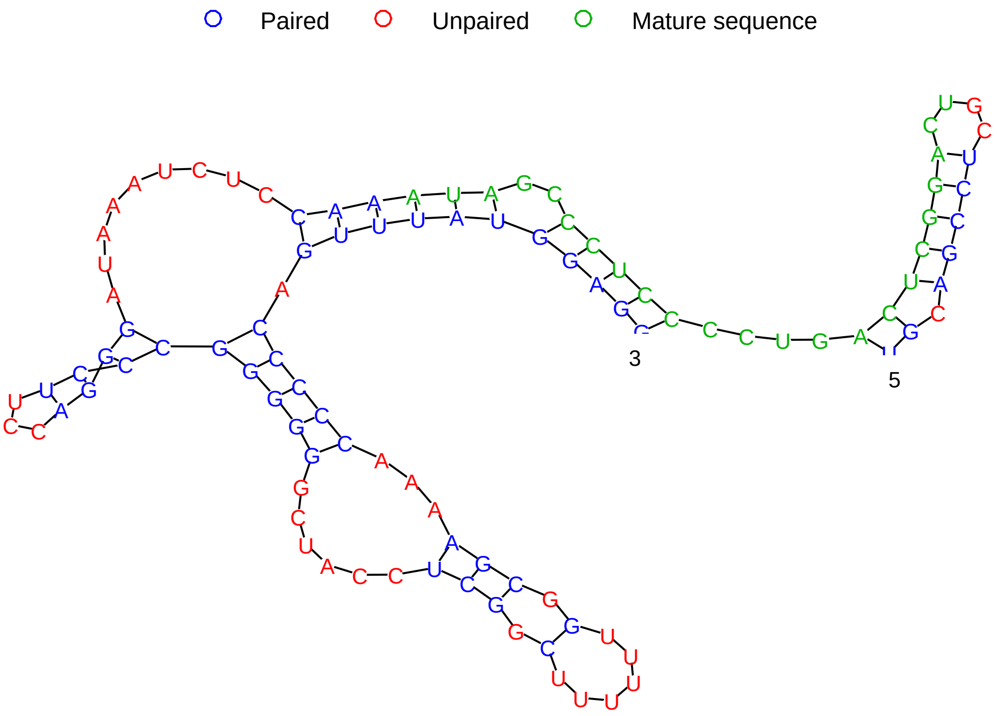

miRNA: bta-miR-484
Stem loop (UMD3.1): chr25:14176039-14176141
Mature (UMD3.1): chr25:14176049-14176071
Mature seq len: 23
Total raw counts (9 samples): 4405
Average raw counts: 490
Strand: Forward
Orientation: 5p
Minimum free energy: -34.40

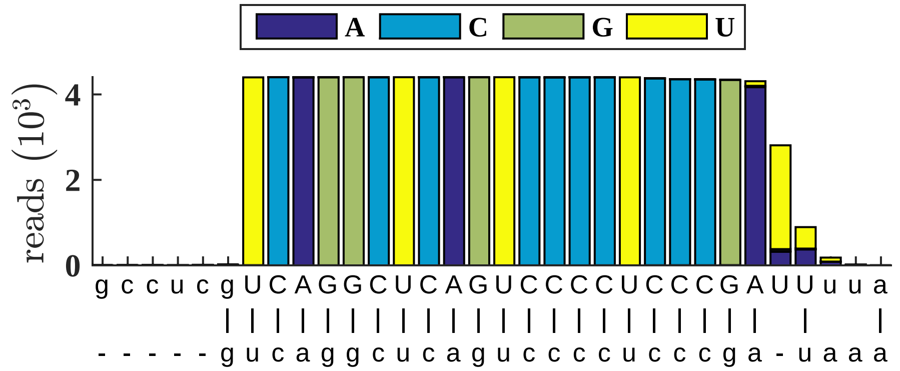

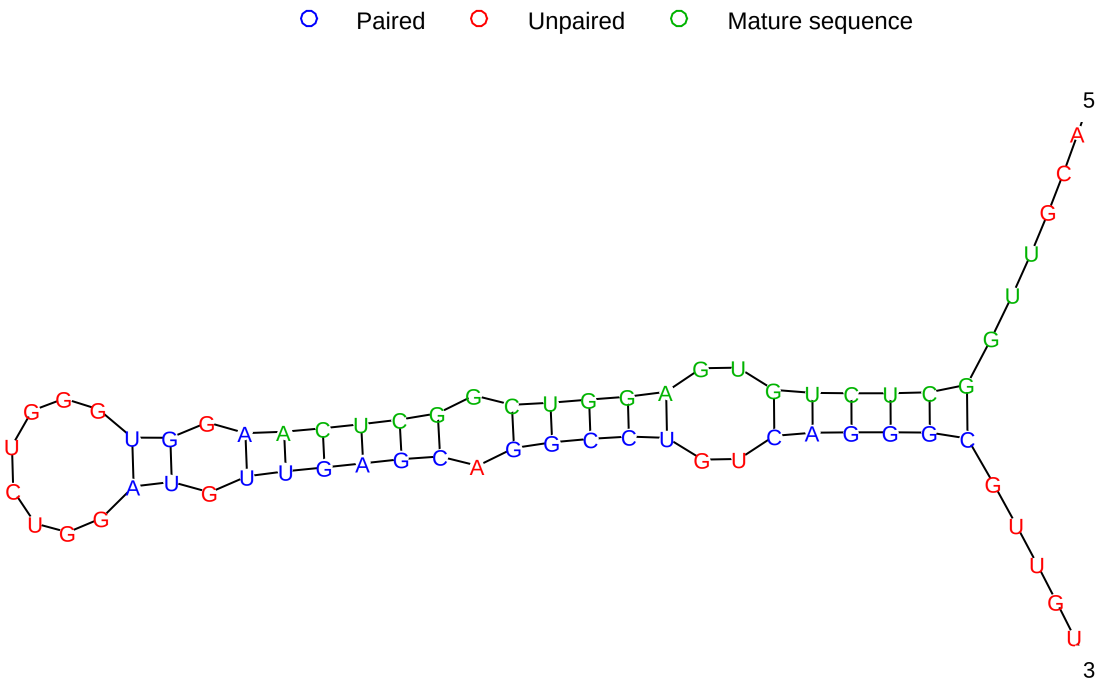

miRNA: bta-miR-1842
Stem loop (UMD3.1): chr25:1744660-1744724
Mature (UMD3.1): chr25:1744700-1744721
Mature seq len: 22
Total raw counts (9 samples): 3348
Average raw counts: 372
Strand: Reverse
Orientation: 5p
Minimum free energy: -29.80

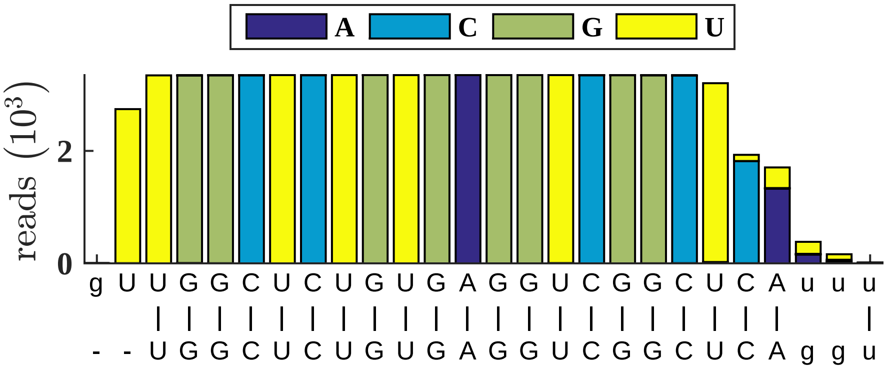

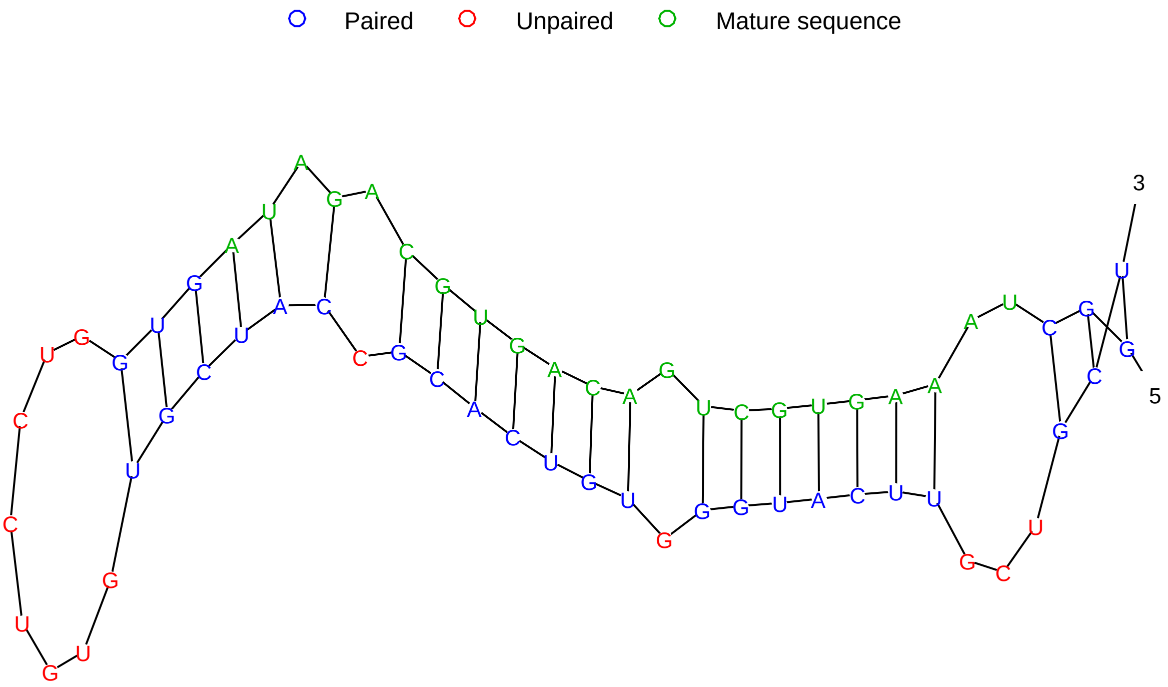

miRNA: bta-miR-106b
Stem loop (UMD3.1): chr25:36892054-36892117
Mature (UMD3.1): chr25:36892057-36892078
Mature seq len: 22
Total raw counts (9 samples): 6463
Average raw counts: 719
Strand: Forward
Orientation: 5p
Minimum free energy: -27.80

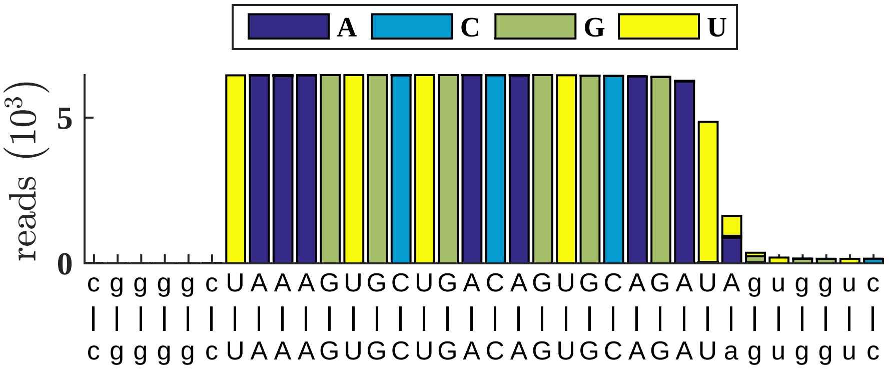

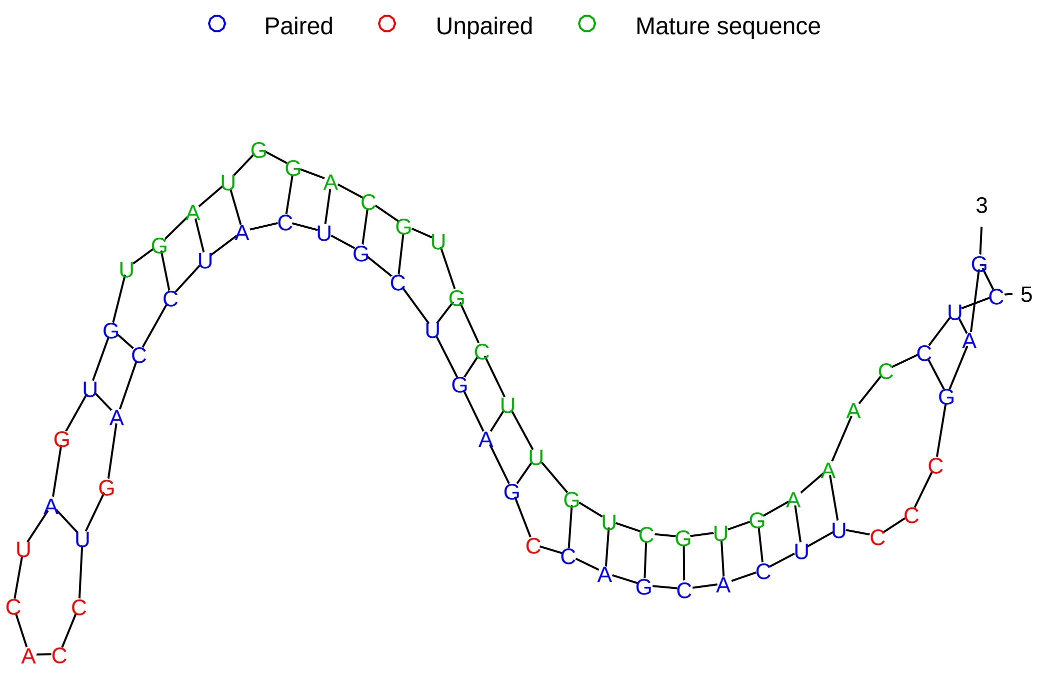

miRNA: bta-miR-93
Stem loop (UMD3.1): chr25:36892256-36892321
Mature (UMD3.1): chr25:36892259-36892282
Mature seq len: 24
Total raw counts (9 samples): 40869
Average raw counts: 4541
Strand: Forward
Orientation: 5p
Minimum free energy: -23.90

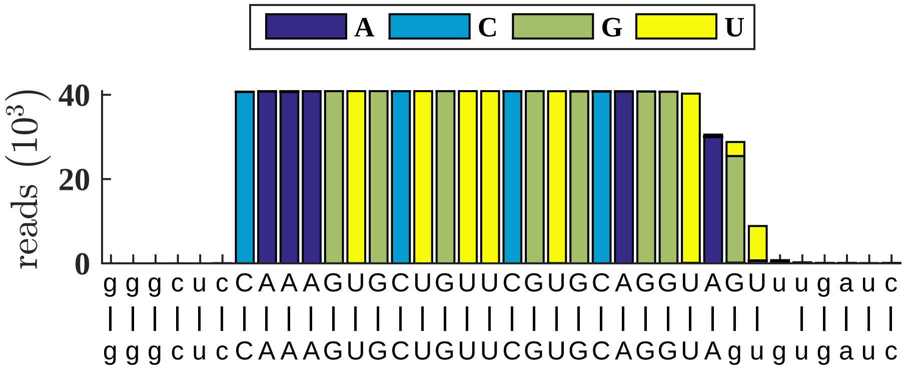

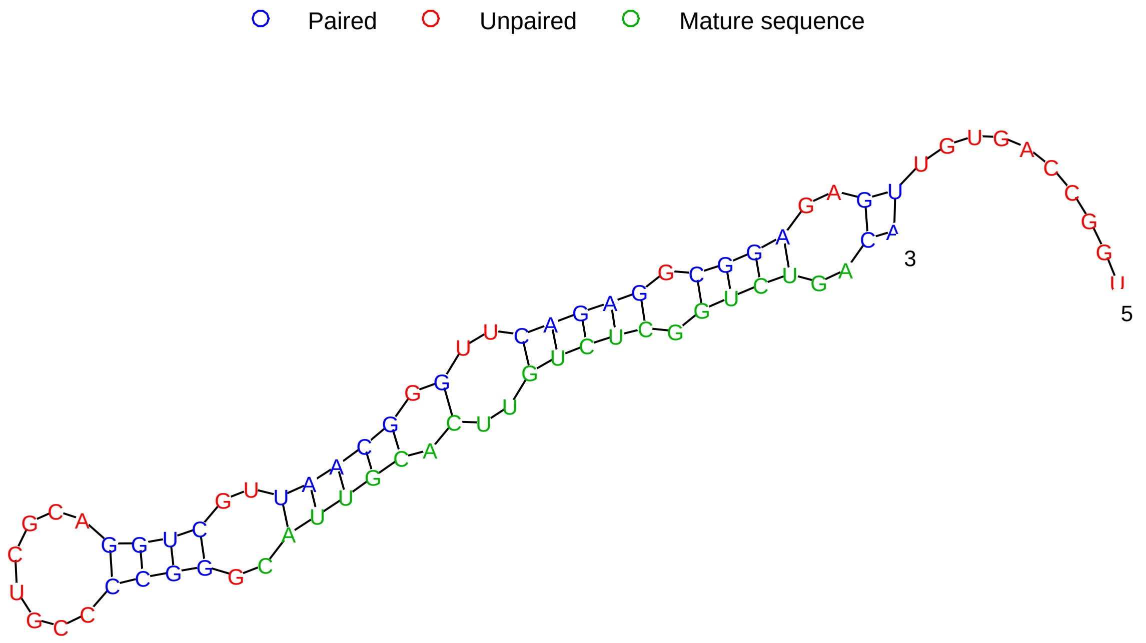

miRNA: bta-miR-25
Stem loop (UMD3.1): chr25:36892448-36892523
Mature (UMD3.1): chr25:36892500-36892521
Mature seq len: 22
Total raw counts (9 samples): 282162
Average raw counts: 31352
Strand: Forward
Orientation: 3p
Minimum free energy: -27.50

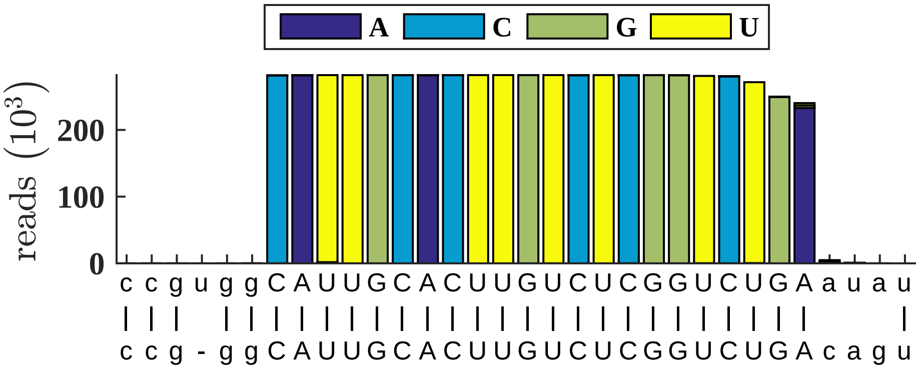

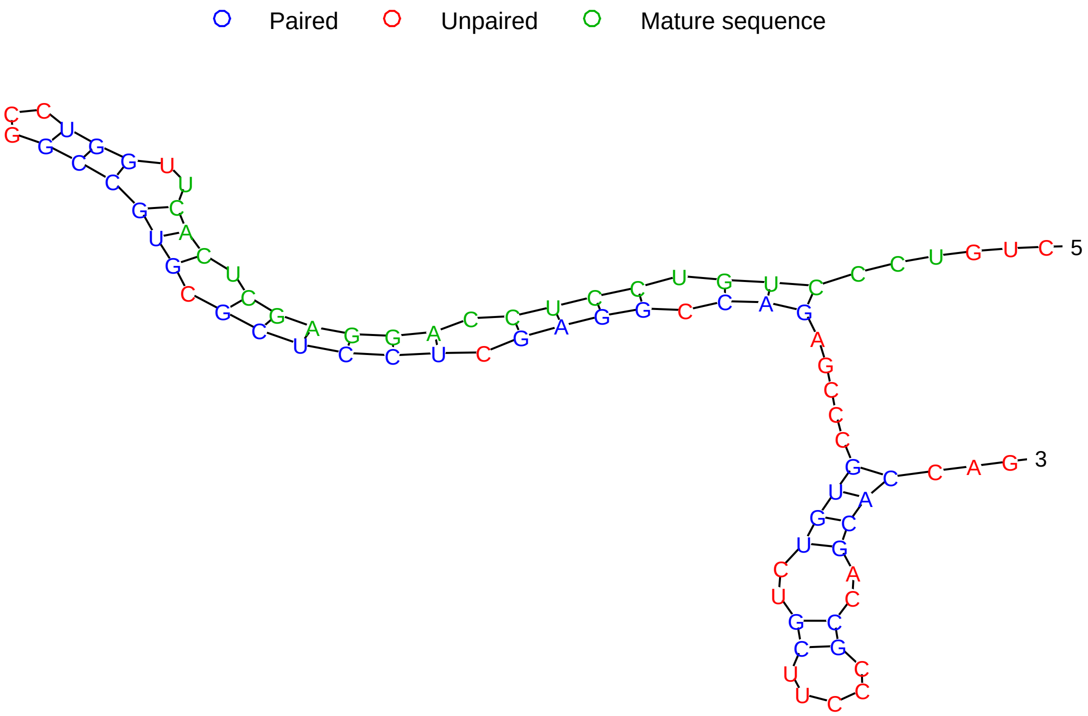

miRNA: bta-miR-339a
Stem loop (UMD3.1): chr25:42294008-42294091
Mature (UMD3.1): chr25:42294011-42294033
Mature seq len: 23
Total raw counts (9 samples): 7981
Average raw counts: 887
Strand: Forward
Orientation: 5p
Minimum free energy: -31.40

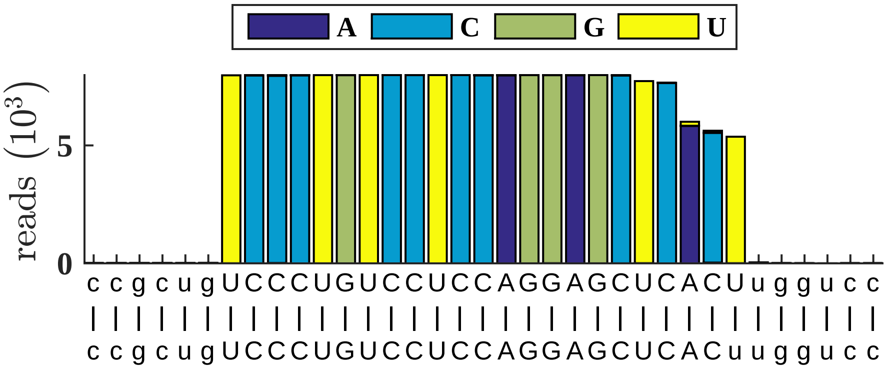

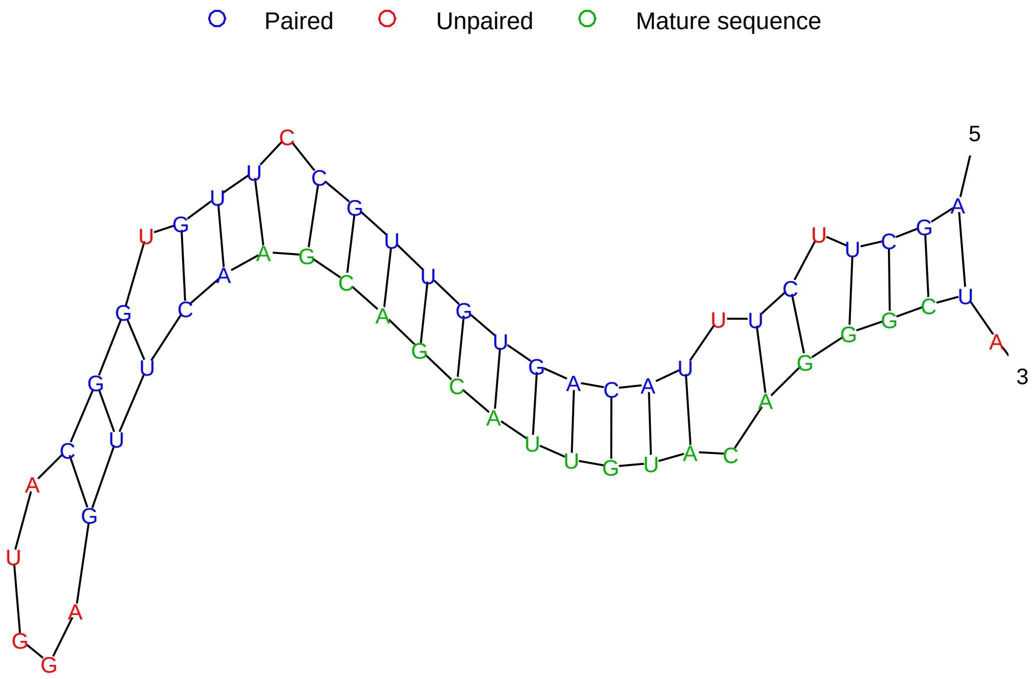

miRNA: bta-miR-107
Stem loop (UMD3.1): chr26:11288006-11288062
Mature (UMD3.1): chr26:11288008-11288025
Mature seq len: 18
Total raw counts (9 samples): 73680
Average raw counts: 8187
Strand: Reverse
Orientation: 3p
Minimum free energy: -21.80

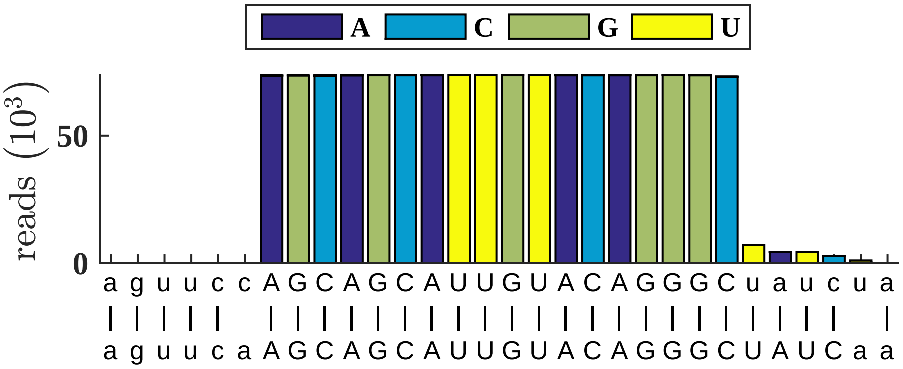

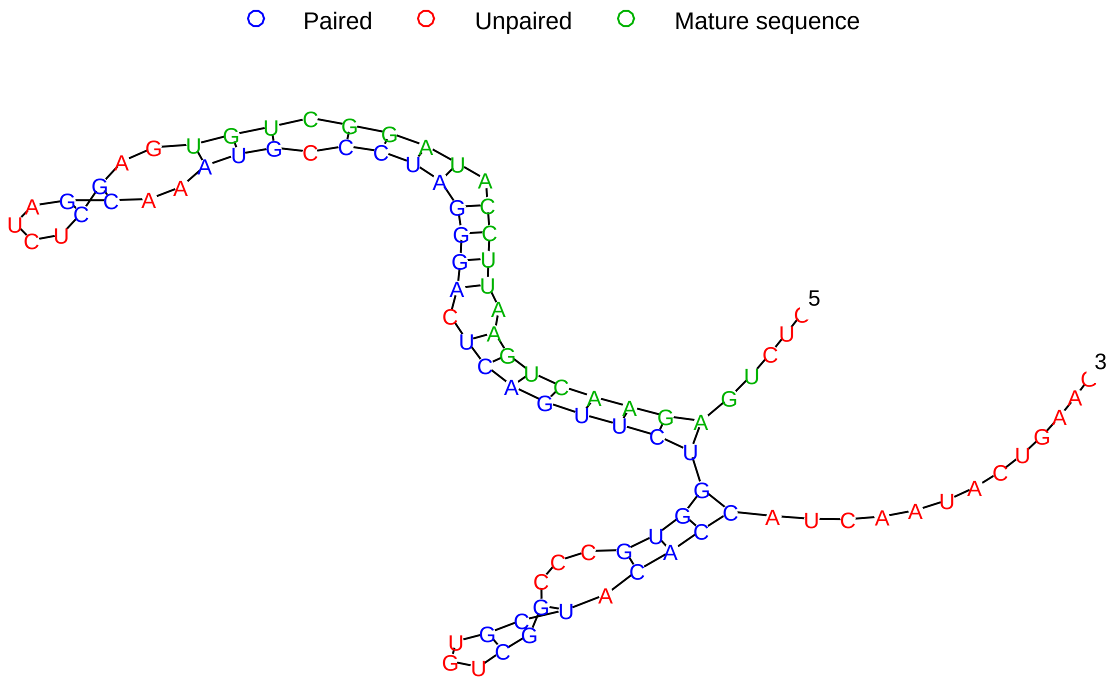

miRNA: bta-miR-146b
Stem loop (UMD3.1): chr26:22930912-22931005
Mature (UMD3.1): chr26:22930915-22930938
Mature seq len: 24
Total raw counts (9 samples): 7981
Average raw counts: 887
Strand: Forward
Orientation: 5p
Minimum free energy: -28.10

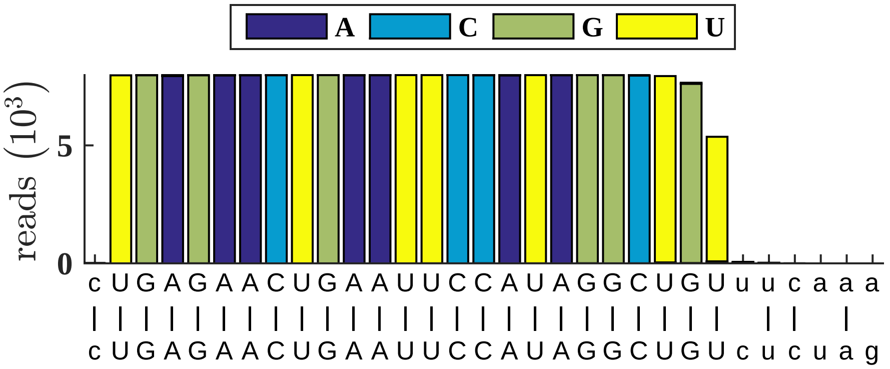

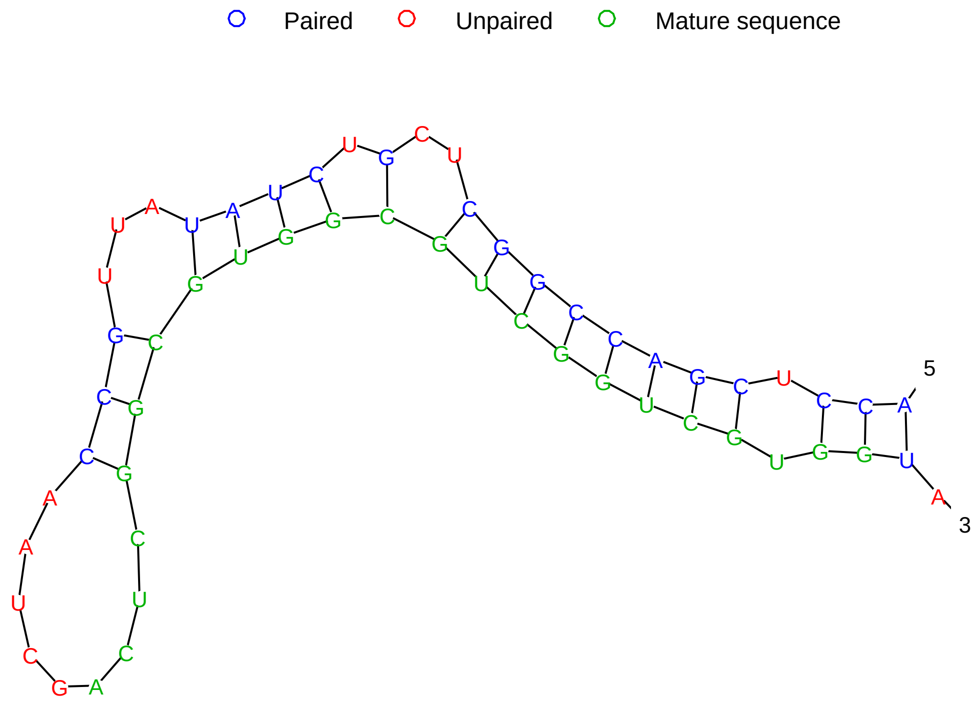

miRNA: bta-miR-1307
Stem loop (UMD3.1): chr26:24230116-24230171
Mature (UMD3.1): chr26:24230118-24230140
Mature seq len: 23
Total raw counts (9 samples): 1552
Average raw counts: 173
Strand: Reverse
Orientation: 3p
Minimum free energy: -21.90

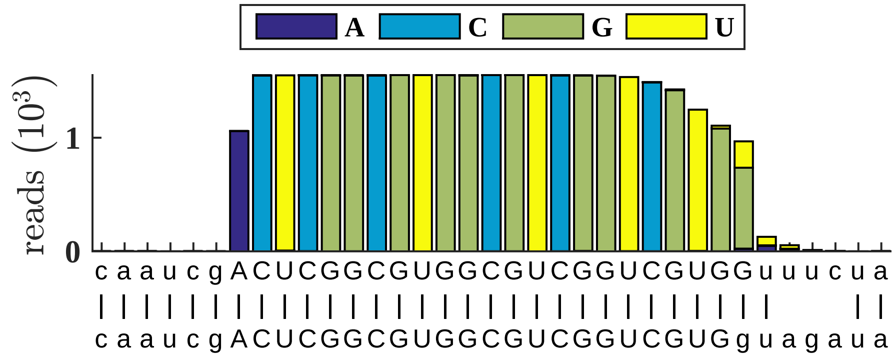

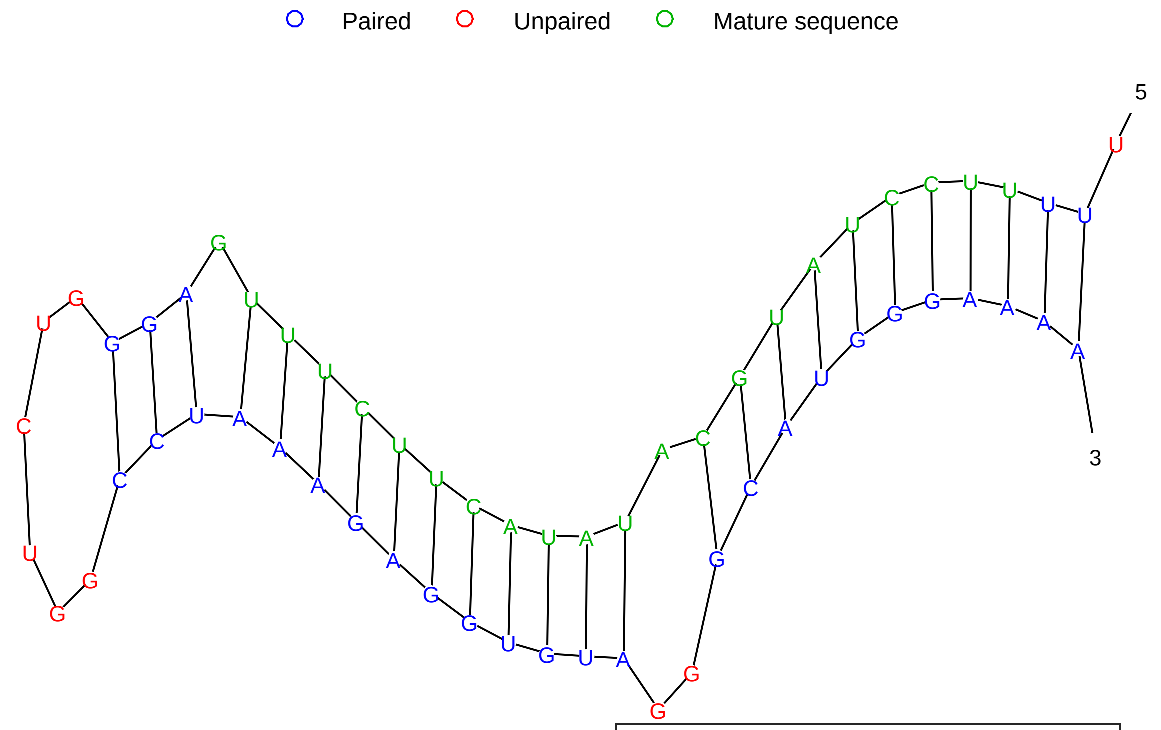

miRNA: bta-miR-202
Stem loop (UMD3.1): chr26:25988255-25988315
Mature (UMD3.1): chr26:25988258-25988279
Mature seq len: 22
Total raw counts (9 samples): 40532
Average raw counts: 4504
Strand: Forward
Orientation: 5p
Minimum free energy: -30.50

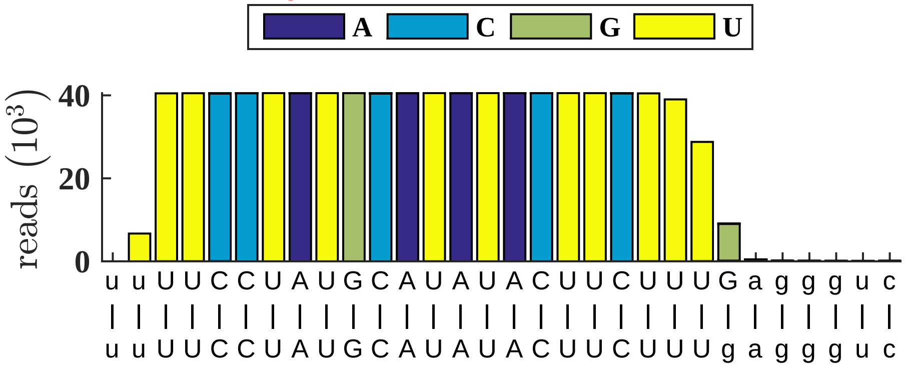

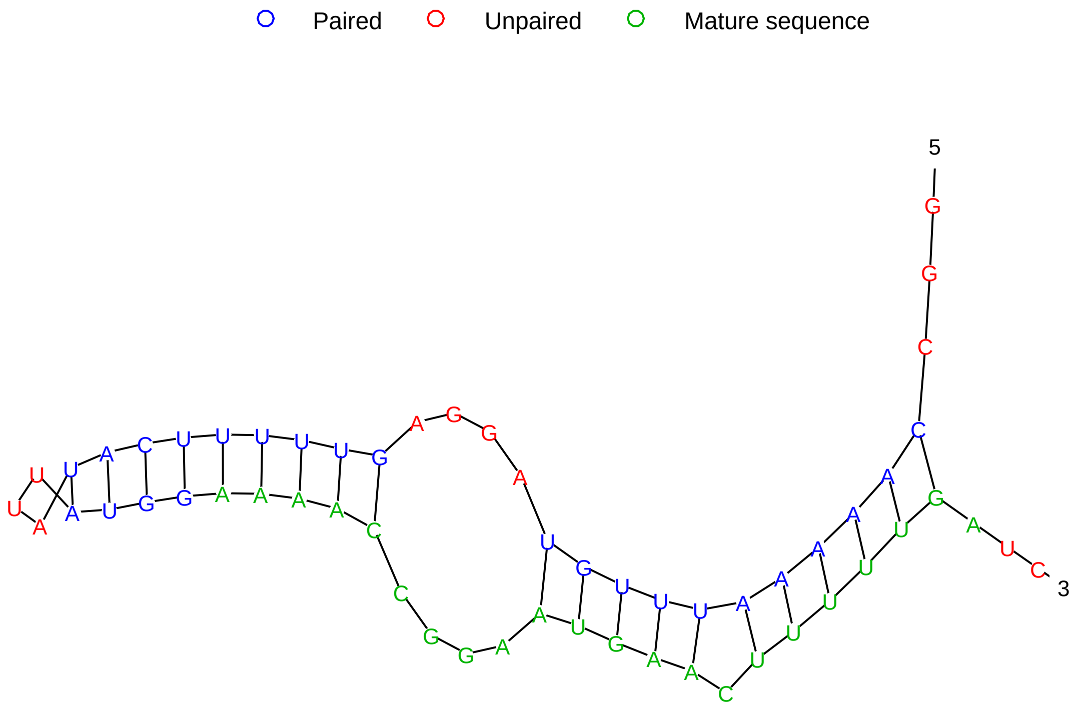

miRNA: bta-miR-2285k
Stem loop (UMD3.1): chr26:29429656-29429713
Mature (UMD3.1): chr26:29429690-29429711
Mature seq len: 22
Total raw counts (9 samples): 457
Average raw counts: 51
Strand: Forward
Orientation: 3p
Minimum free energy: -9.80

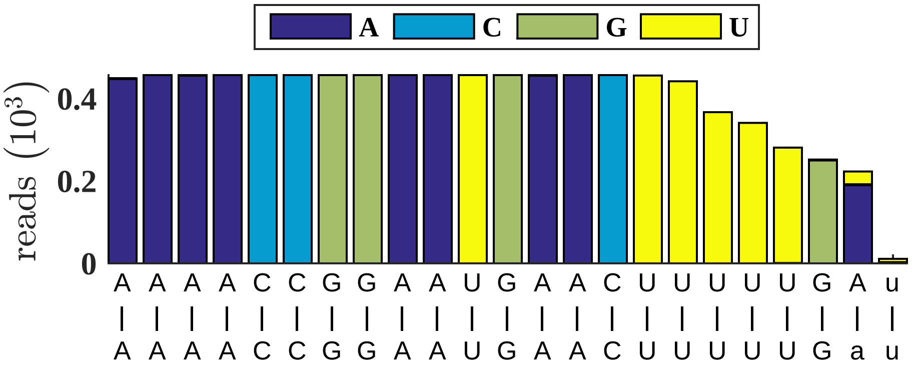

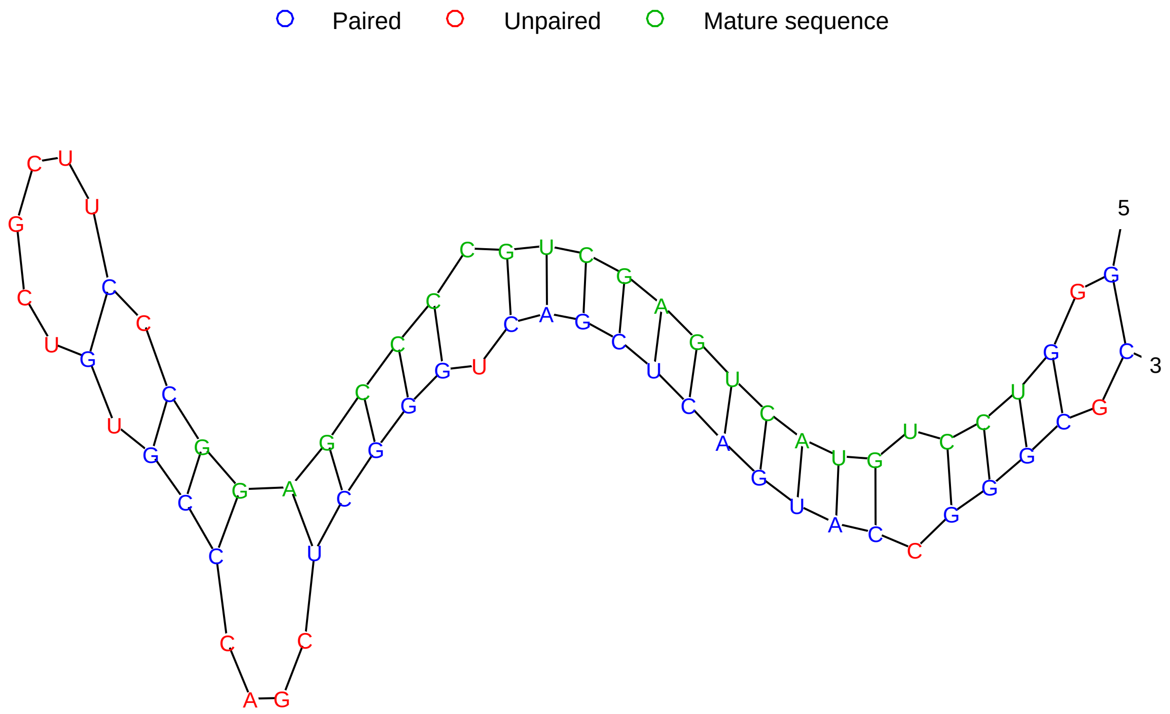

miRNA: bta-miR-486
Stem loop (UMD3.1): chr27:36261846-36261913
Mature (UMD3.1): chr27:36261888-36261910
Mature seq len: 23
Total raw counts (9 samples): 17375
Average raw counts: 1931
Strand: Reverse
Orientation: 5p
Minimum free energy: -40.60

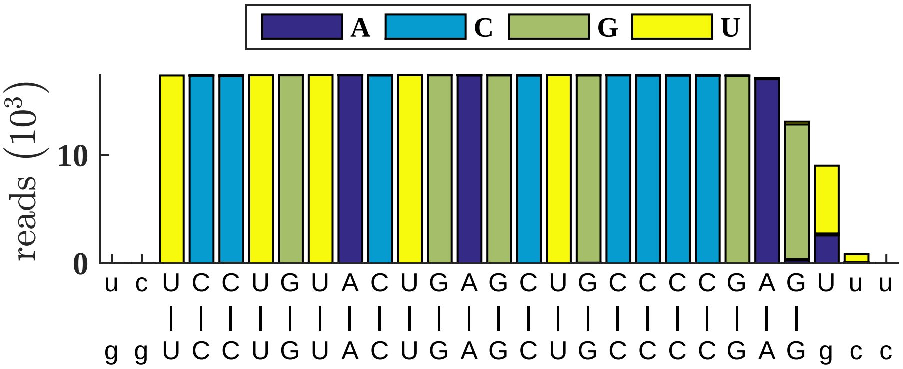

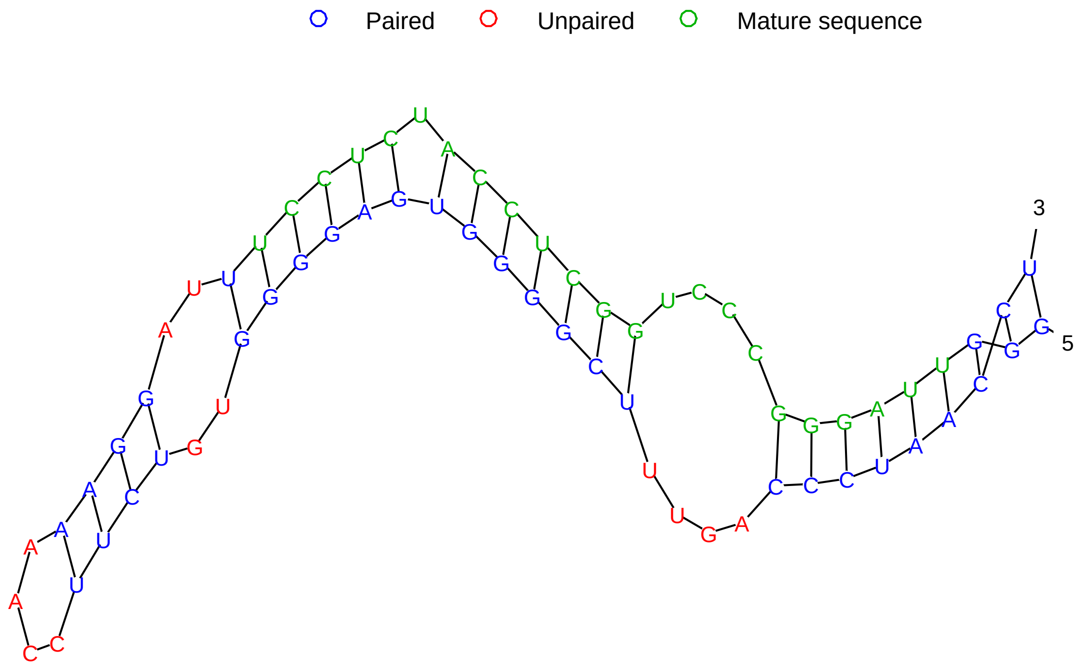

miRNA: bta-miR-1296
Stem loop (UMD3.1): chr28:19569105-19569173
Mature (UMD3.1): chr28:19569148-19569170
Mature seq len: 23
Total raw counts (9 samples): 2929
Average raw counts: 326
Strand: Reverse
Orientation: 5p
Minimum free energy: -37.80

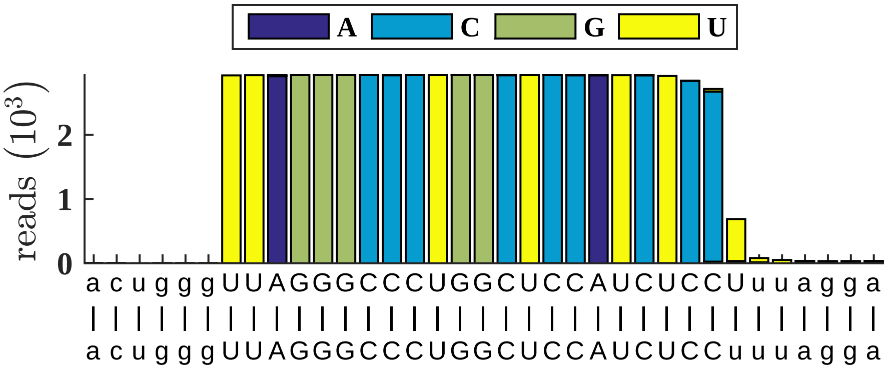

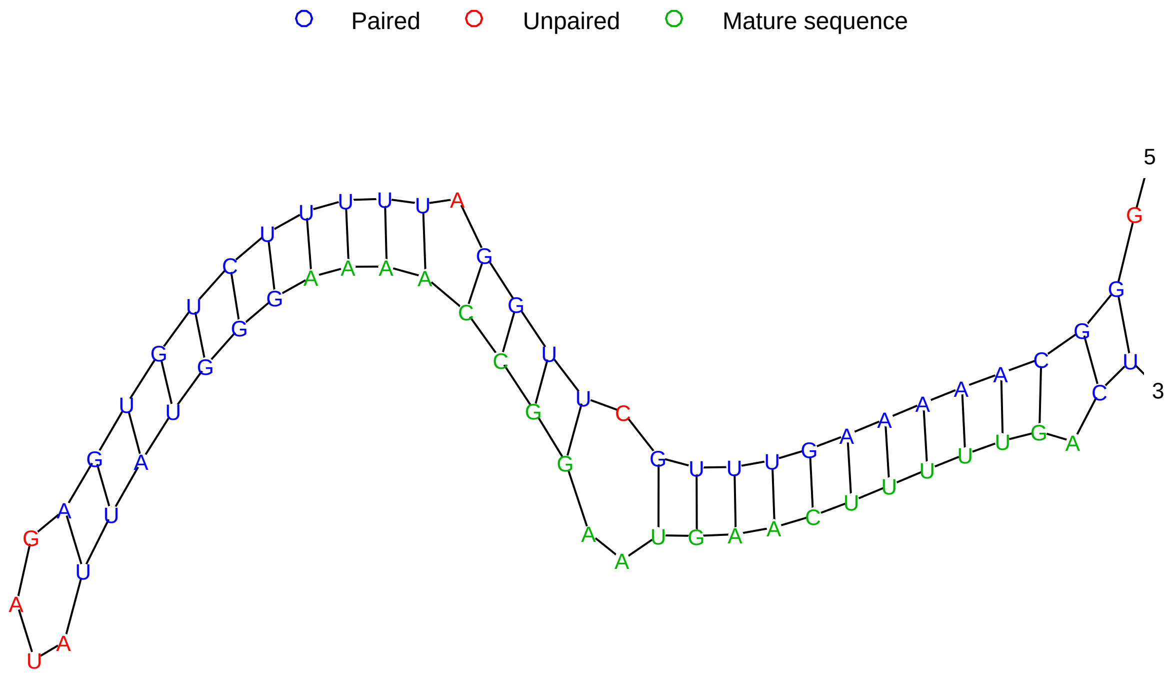

miRNA: bta-miR-2285k
Stem loop (UMD3.1): chr29:10834183-10834248
Mature (UMD3.1): chr29:10834225-10834246
Mature seq len: 22
Total raw counts (9 samples): 449
Average raw counts: 50
Strand: Forward
Orientation: 3p
Minimum free energy: -20.00

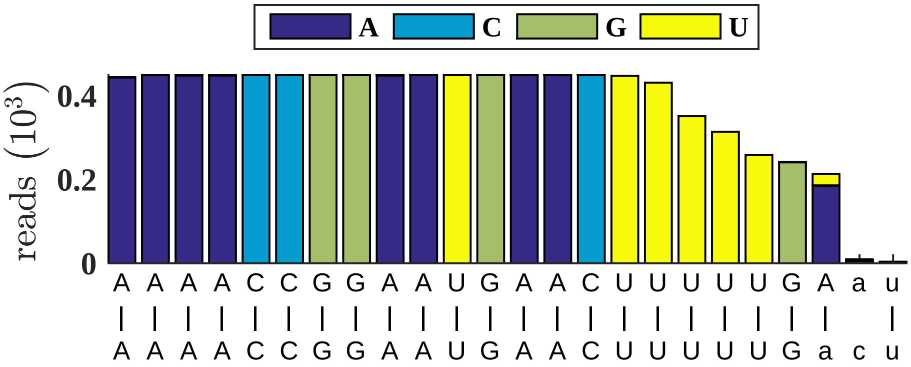

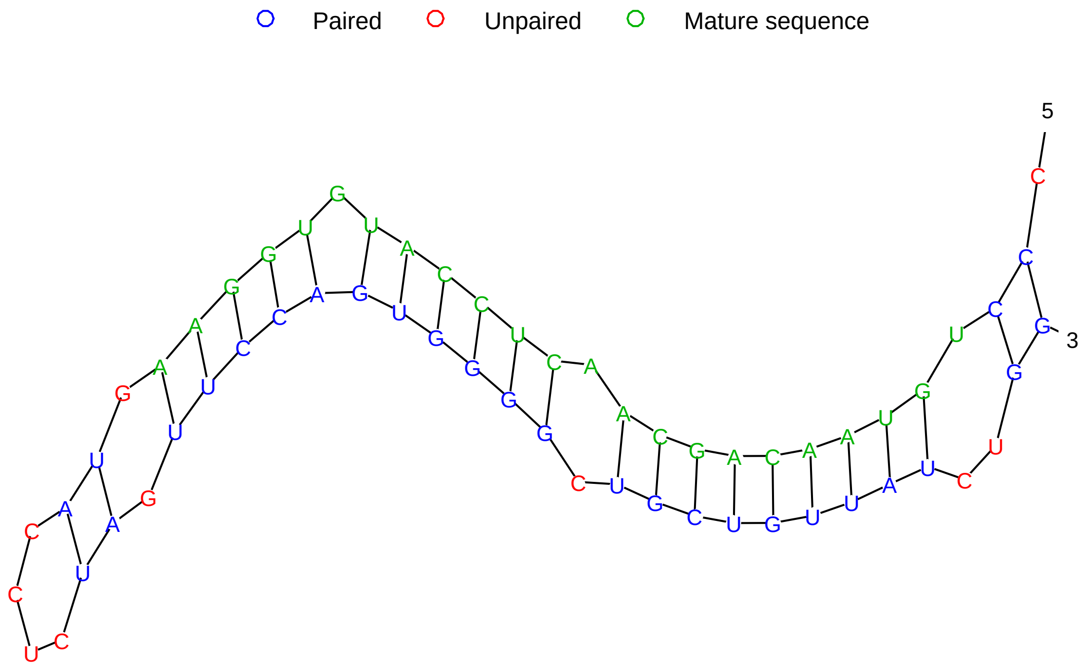

miRNA: bta-miR-194
Stem loop (UMD3.1): chr29:43731471-43731531
Mature (UMD3.1): chr29:43731474-43731496
Mature seq len: 23
Total raw counts (9 samples): 527
Average raw counts: 59
Strand: Forward
Orientation: 5p
Minimum free energy: -28.20

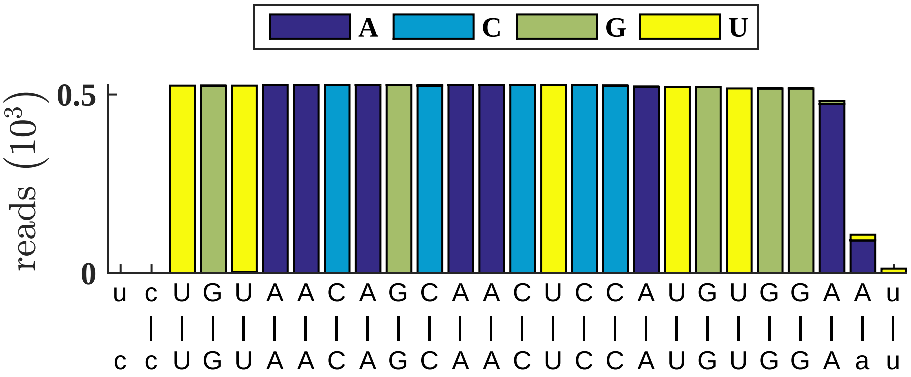

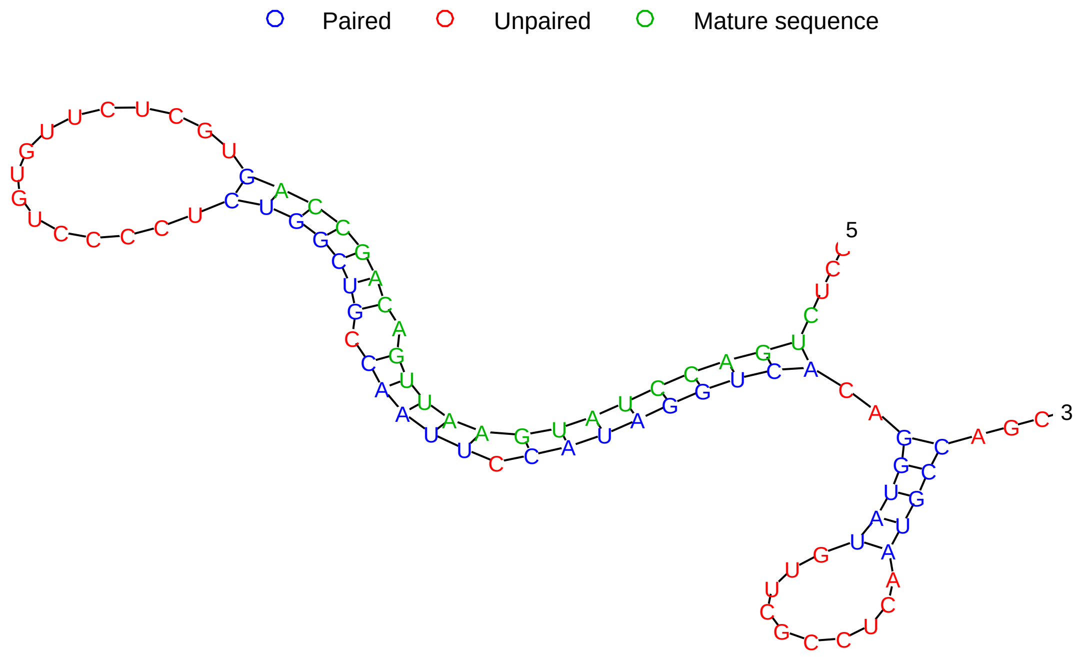

miRNA: bta-miR-192
Stem loop (UMD3.1): chr29:43731676-43731765
Mature (UMD3.1): chr29:43731679-43731700
Mature seq len: 22
Total raw counts (9 samples): 60172
Average raw counts: 6686
Strand: Forward
Orientation: 5p
Minimum free energy: -34.00

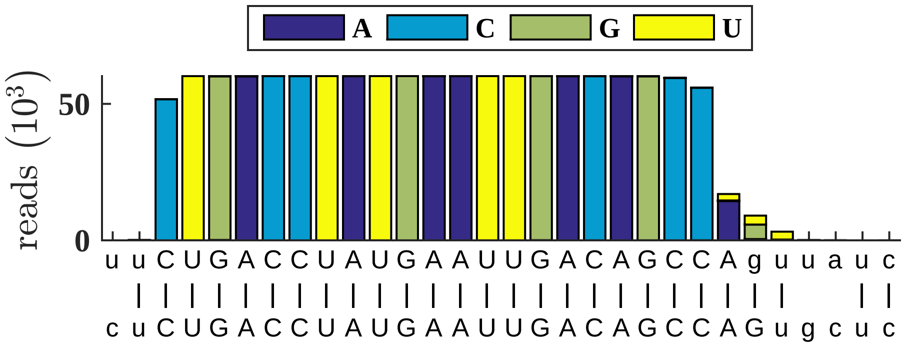

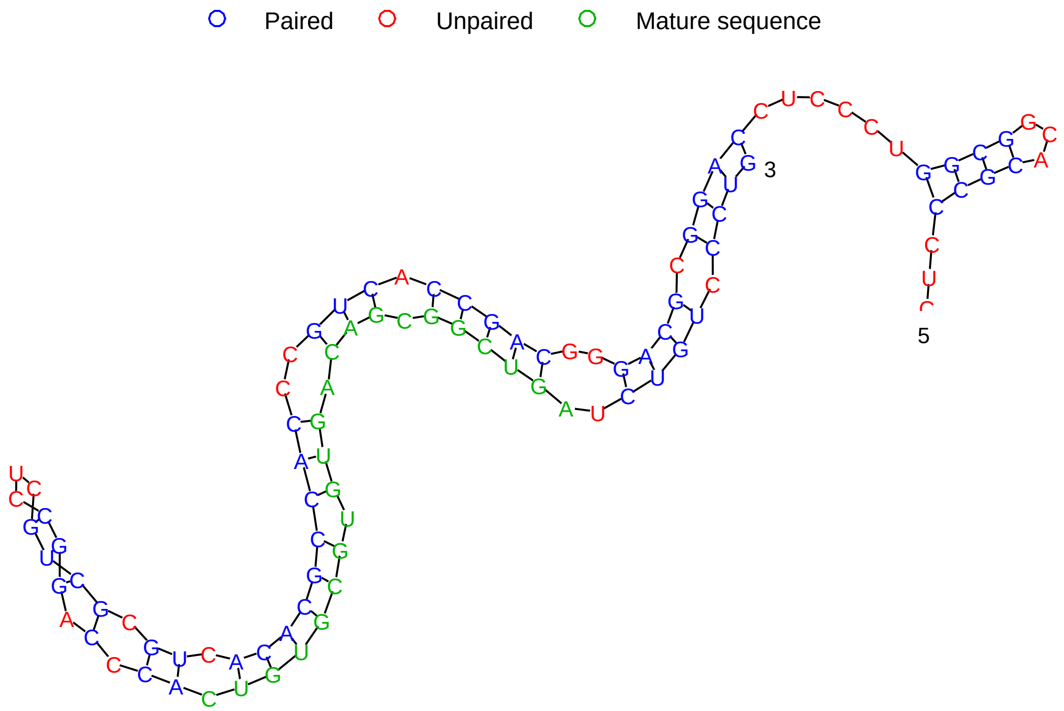

miRNA: bta-miR-210
Stem loop (UMD3.1): chr29:51025417-51025518
Mature (UMD3.1): chr29:51025487-51025508
Mature seq len: 22
Total raw counts (9 samples): 23634
Average raw counts: 2626
Strand: Forward
Orientation: 3p
Minimum free energy: -44.80

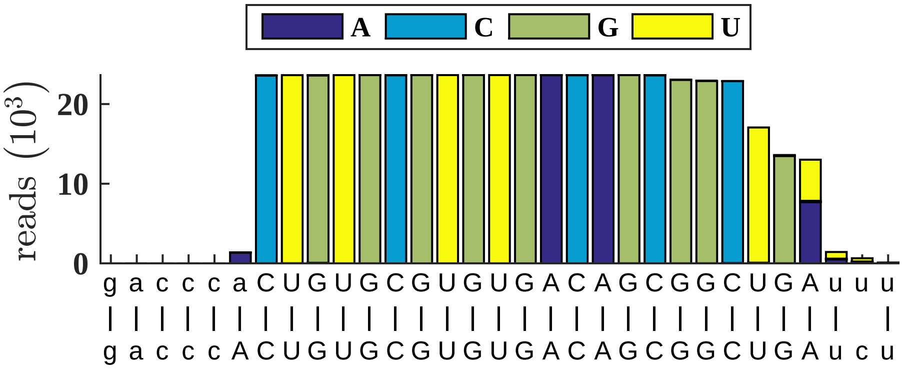

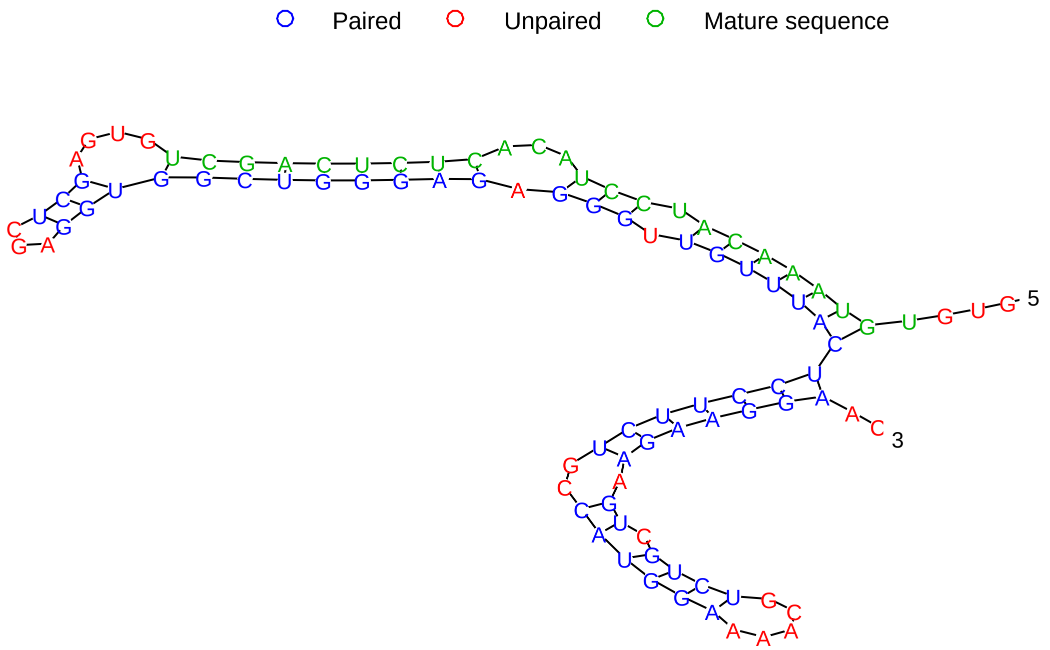

miRNA: bta-miR-30c
Stem loop (UMD3.1): chr3:106059343-106059440
Mature (UMD3.1): chr3:106059414-106059437
Mature seq len: 24
Total raw counts (9 samples): 36188
Average raw counts: 4021
Strand: Reverse
Orientation: 5p
Minimum free energy: -34.60

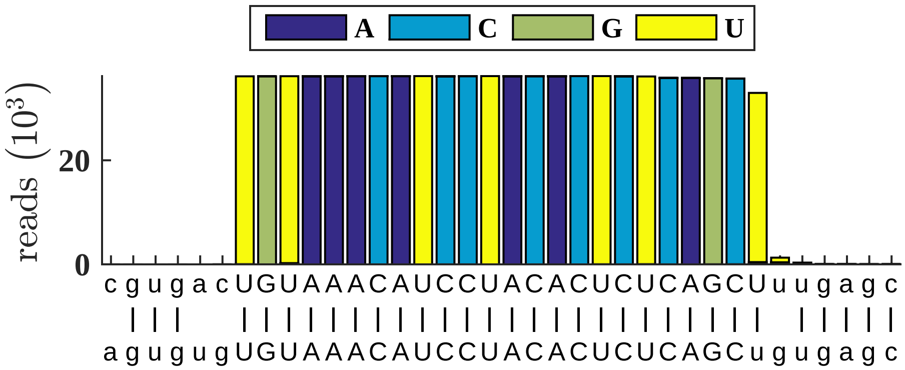

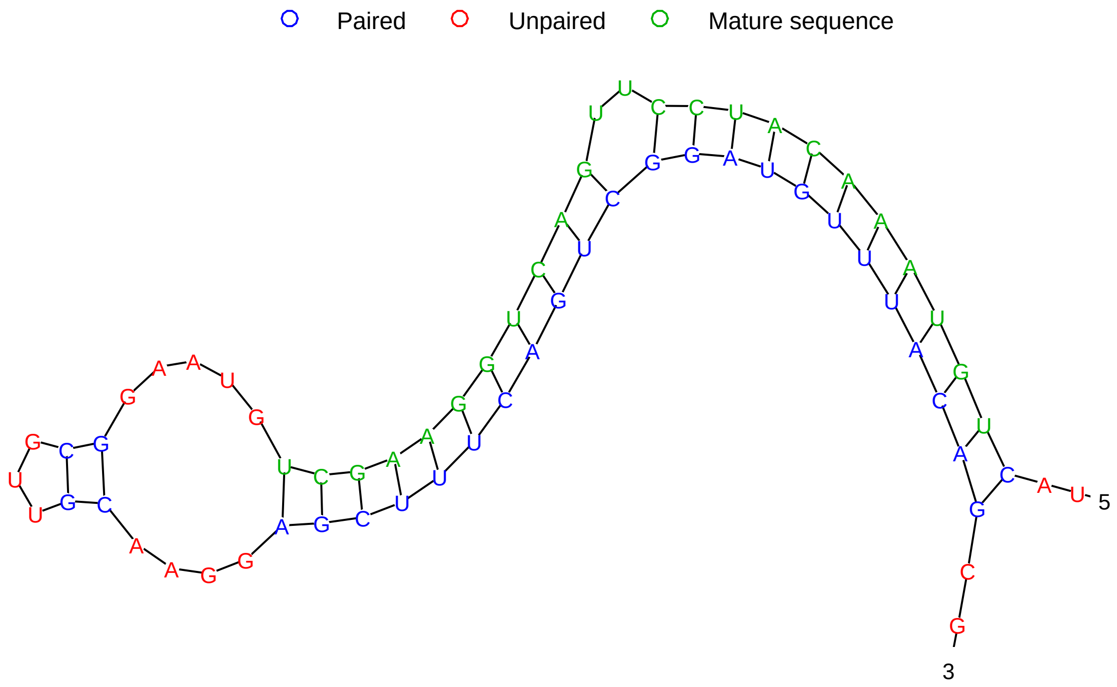

miRNA: bta-miR-30e-5p
Stem loop (UMD3.1): chr3:106062503-106062570
Mature (UMD3.1): chr3:106062544-106062567
Mature seq len: 24
Total raw counts (9 samples): 131145
Average raw counts: 14572
Strand: Reverse
Orientation: 5p
Minimum free energy: -35.10

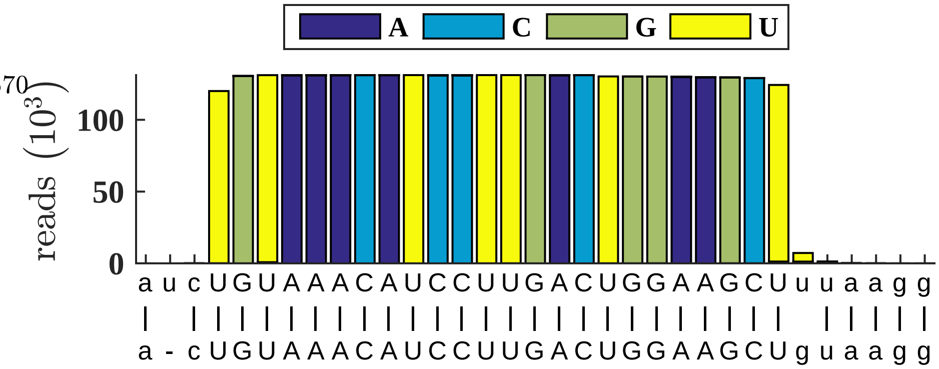

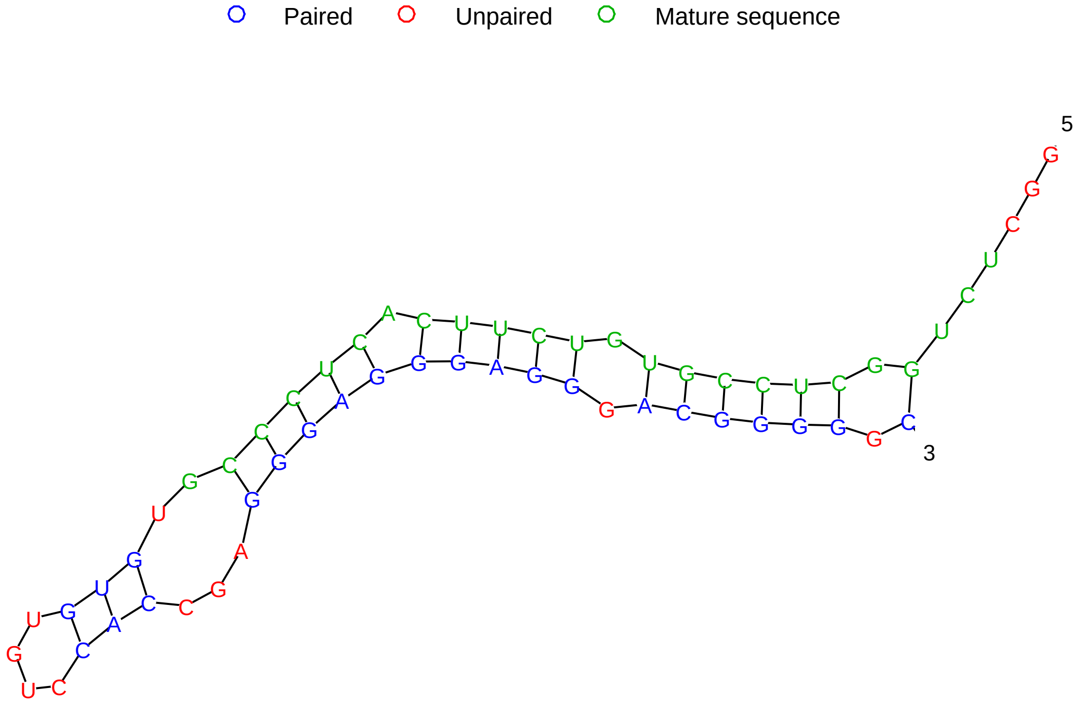

miRNA: bta-miR-149-5p
Stem loop (UMD3.1): chr3:120467959-120468018
Mature (UMD3.1): chr3:120467962-120467985
Mature seq len: 24
Total raw counts (9 samples): 861
Average raw counts: 96
Strand: Forward
Orientation: 5p
Minimum free energy: -29.70

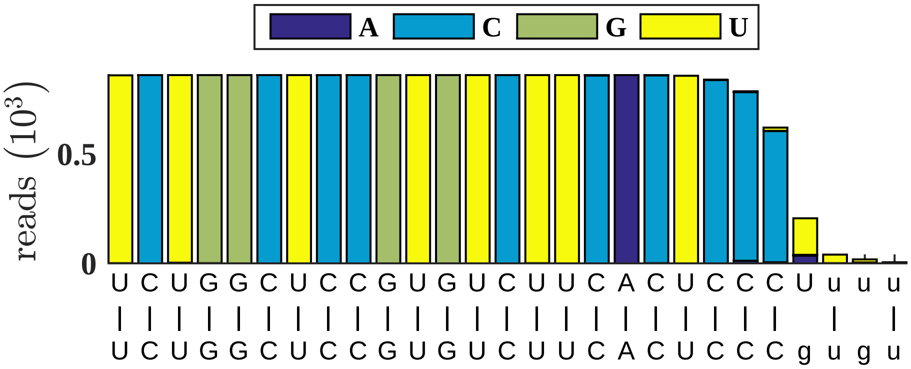

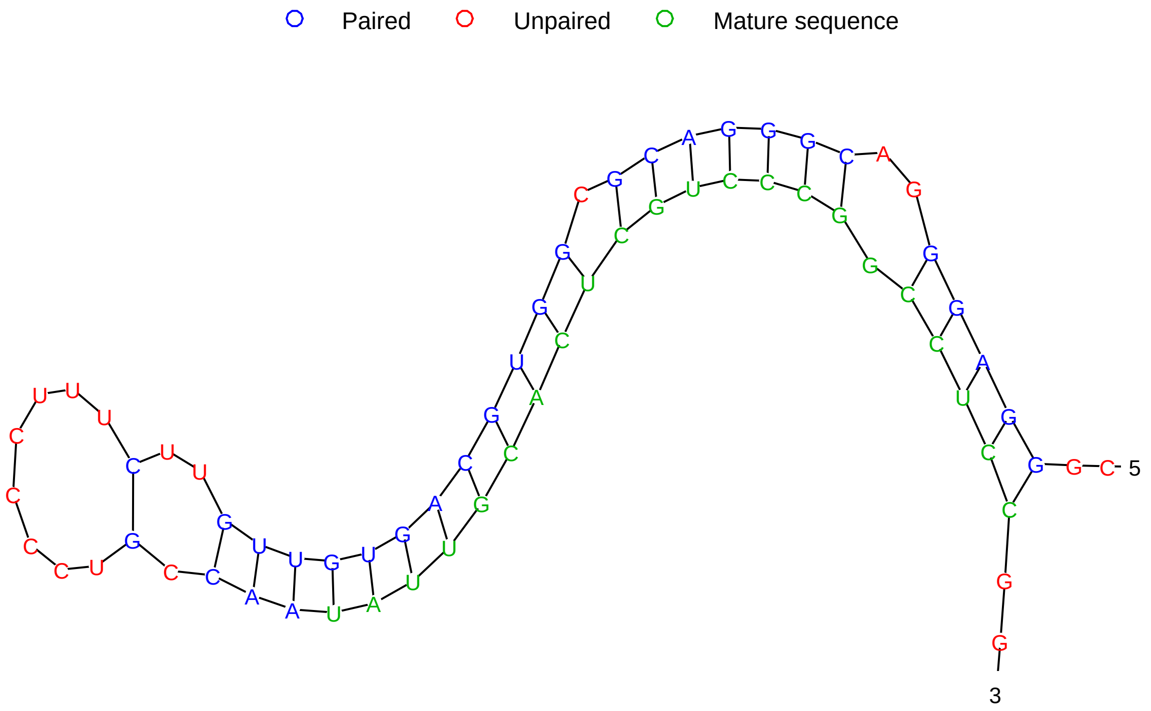

miRNA: bta-miR-92b
Stem loop (UMD3.1): chr3:15480737-15480805
Mature (UMD3.1): chr3:15480739-15480760
Mature seq len: 22
Total raw counts (9 samples): 62244
Average raw counts: 6916
Strand: Reverse
Orientation: 3p
Minimum free energy: -38.90

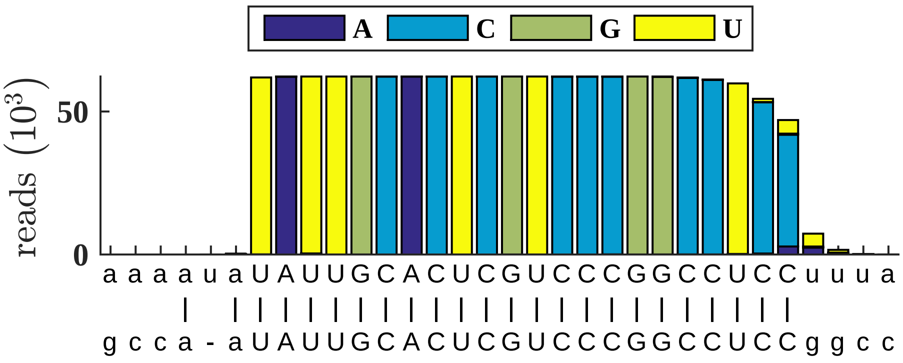

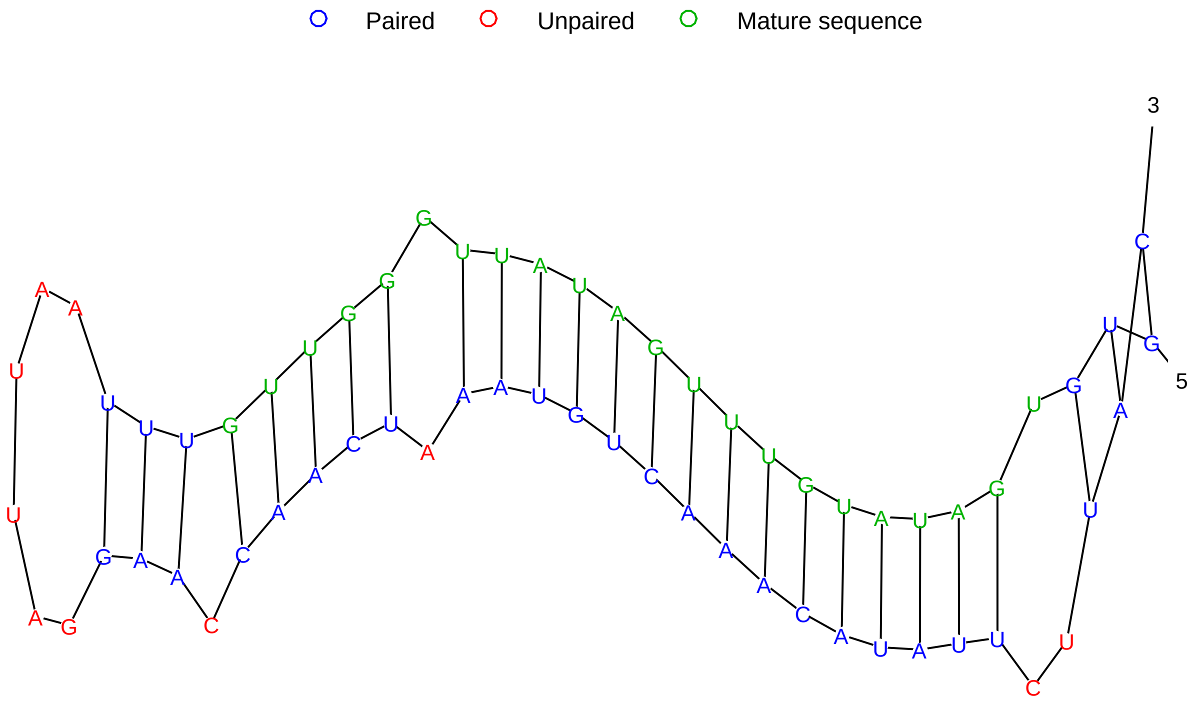

miRNA: bta-miR-190b
Stem loop (UMD3.1): chr3:16374611-16374674
Mature (UMD3.1): chr3:16374614-16374635
Mature seq len: 22
Total raw counts (9 samples): 1206
Average raw counts: 134
Strand: Forward
Orientation: 5p
Minimum free energy: -21.20

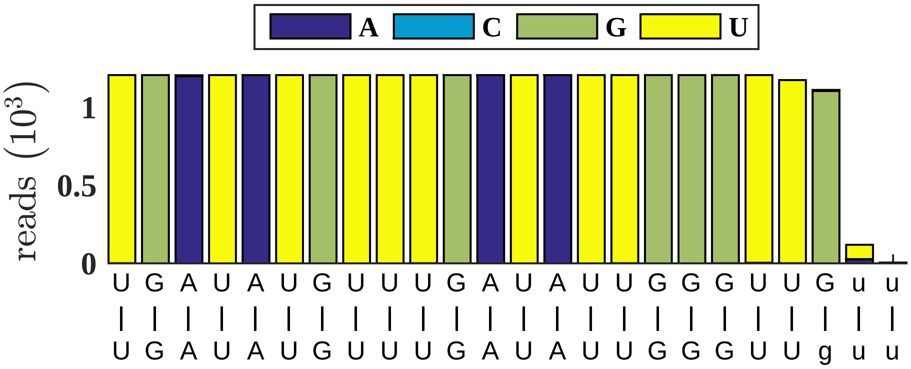

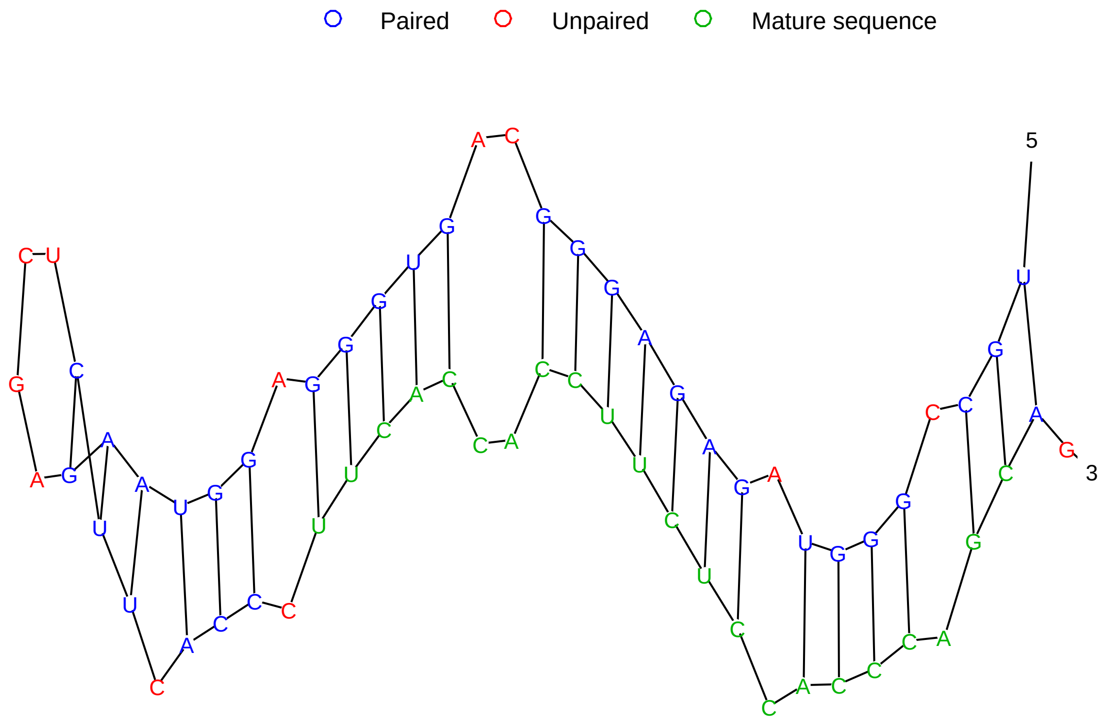

miRNA: bta-miR-197
Stem loop (UMD3.1): chr3:33964972-33965037
Mature (UMD3.1): chr3:33964974-33964995
Mature seq len: 22
Total raw counts (9 samples): 583
Average raw counts: 65
Strand: Reverse
Orientation: 3p
Minimum free energy: -31.80

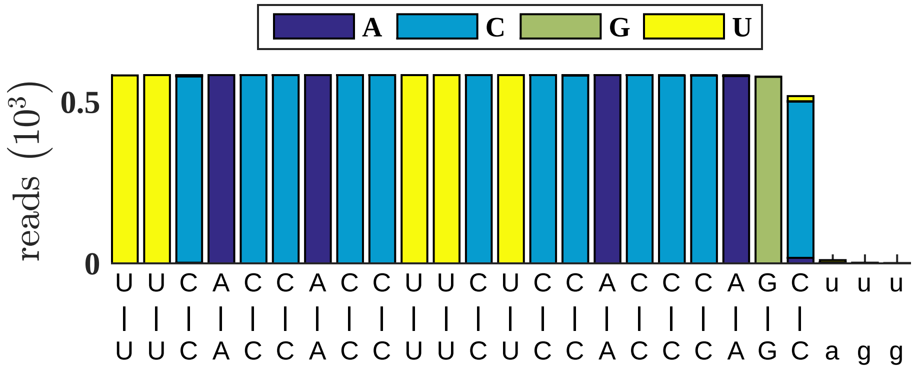

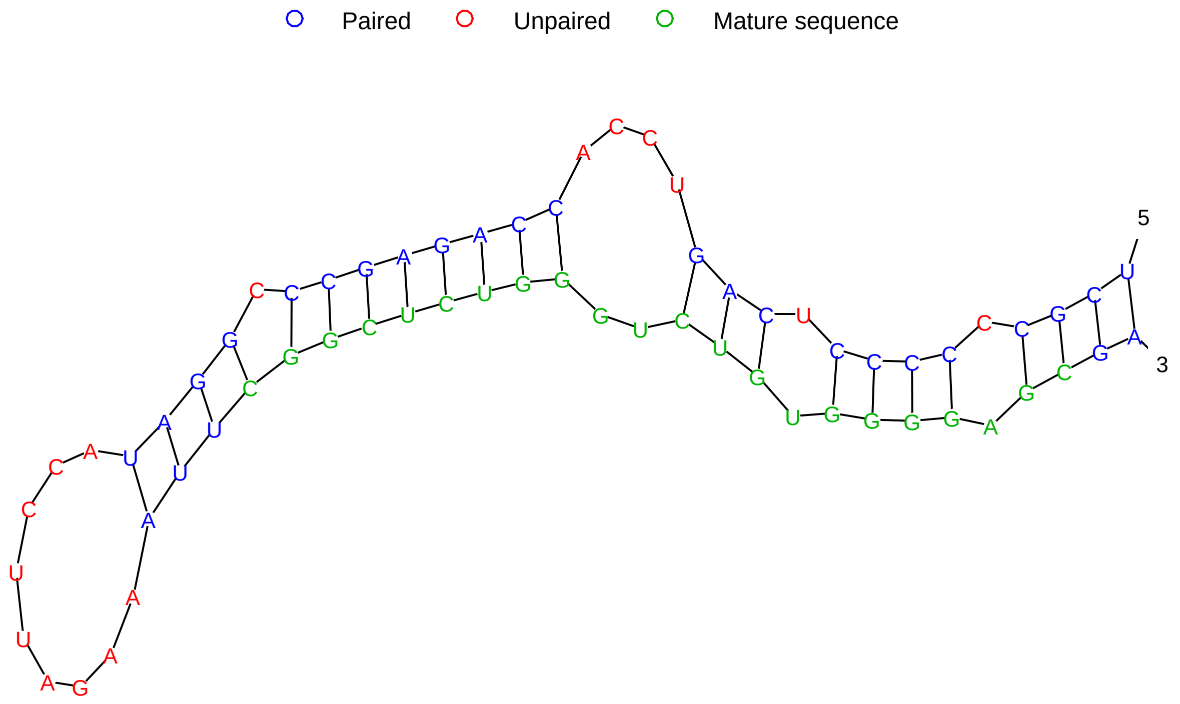

miRNA: bta-miR-760-3p
Stem loop (UMD3.1): chr3:49797116-49797181
Mature (UMD3.1): chr3:49797118-49797139
Mature seq len: 22
Total raw counts (9 samples): 1157
Average raw counts: 129
Strand: Reverse
Orientation: 3p
Minimum free energy: -35.30

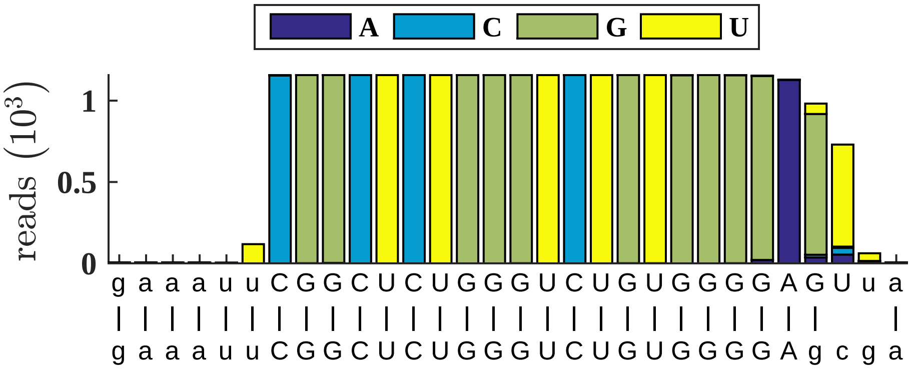

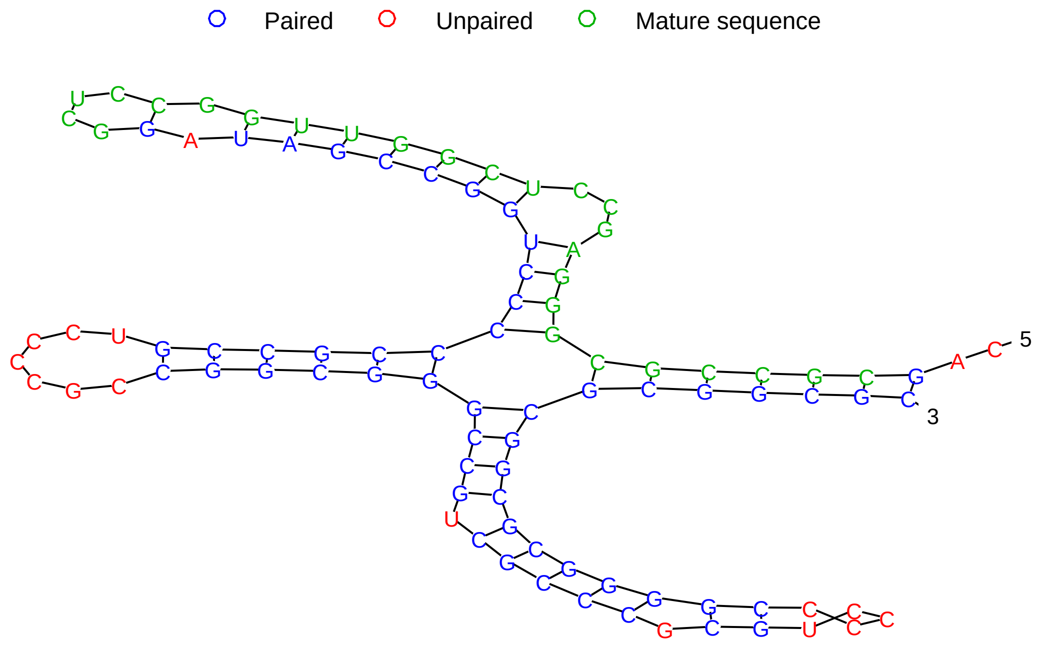

miRNA: bta-miR-2904
Stem loop (UMD3.1): chr3:53471270-53471366
Mature (UMD3.1): chr3:53471273-53471298
Mature seq len: 26
Total raw counts (9 samples): 681
Average raw counts: 76
Strand: Forward
Orientation: 5p
Minimum free energy: -58.70

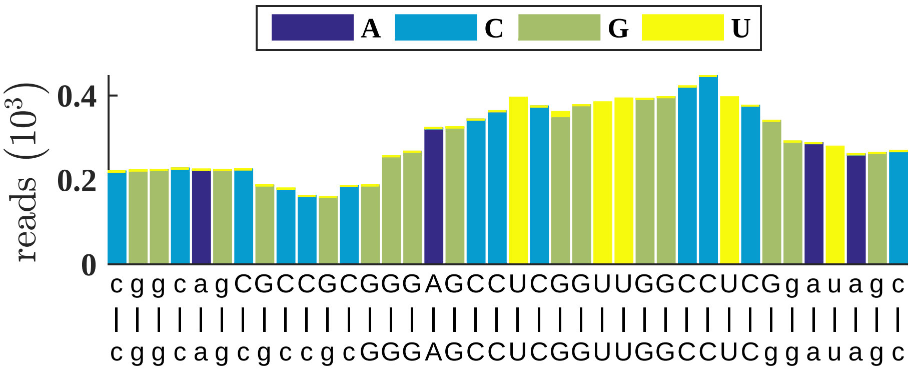

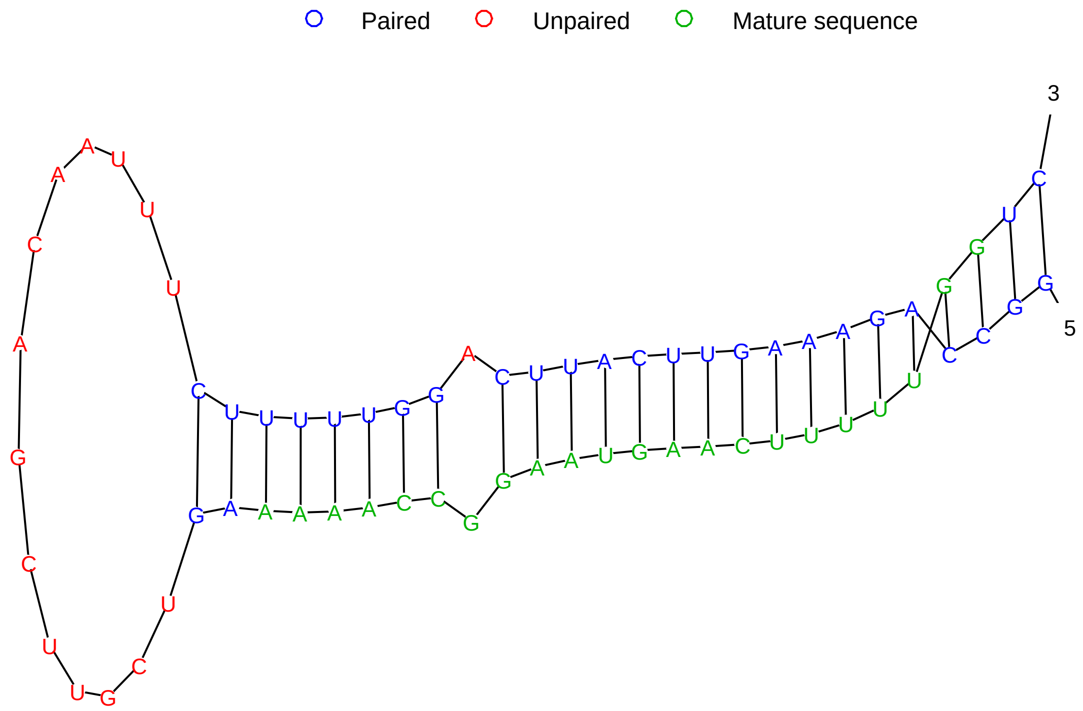

miRNA: bta-miR-2285k
Stem loop (UMD3.1): chr3:53564240-53564305
Mature (UMD3.1): chr3:53564242-53564263
Mature seq len: 22
Total raw counts (9 samples): 663
Average raw counts: 74
Strand: Reverse
Orientation: 3p
Minimum free energy: -33.40

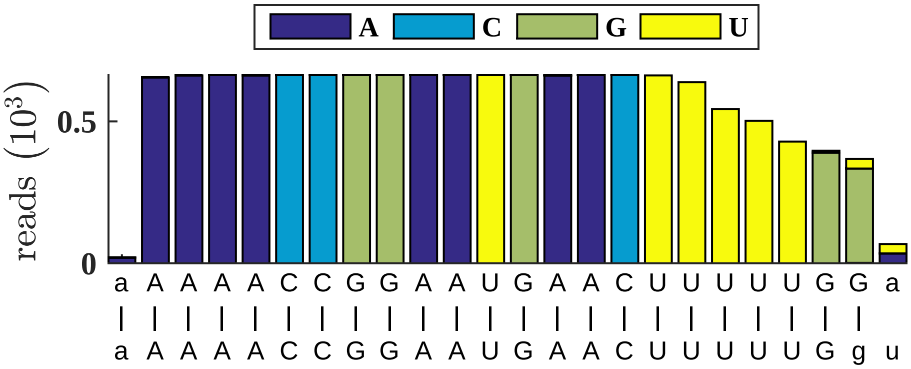

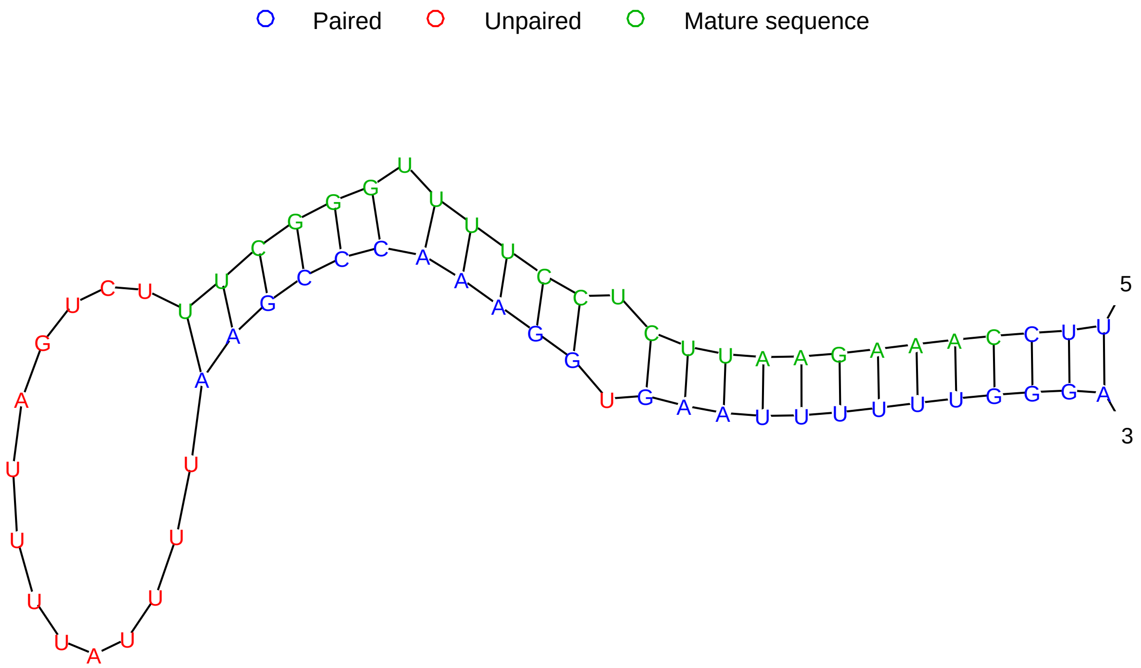

miRNA: bta-miR-186
Stem loop (UMD3.1): chr3:74462306-74462370
Mature (UMD3.1): chr3:74462309-74462331
Mature seq len: 23
Total raw counts (9 samples): 206211
Average raw counts: 22913
Strand: Forward
Orientation: 5p
Minimum free energy: -29.30

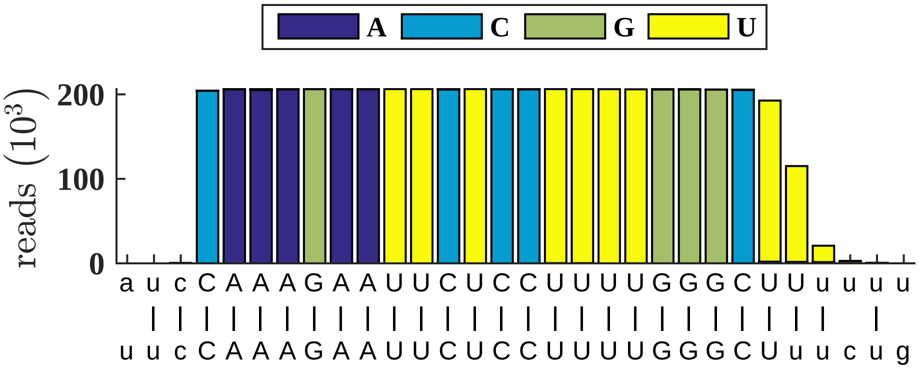

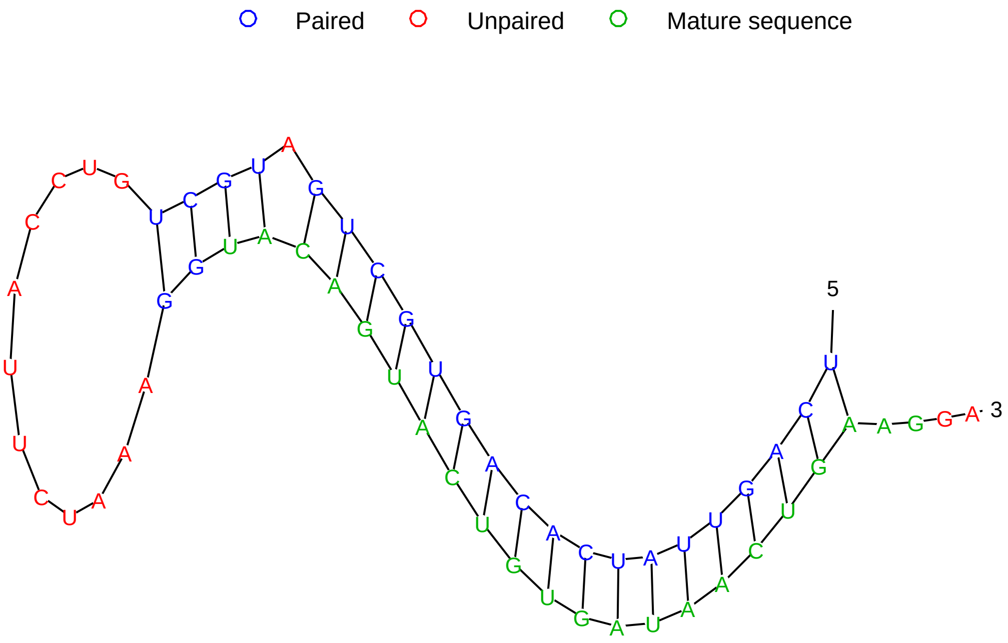

miRNA: bta-miR-101
Stem loop (UMD3.1): chr3:80666430-80666490
Mature (UMD3.1): chr3:80666467-80666488
Mature seq len: 22
Total raw counts (9 samples): 18928
Average raw counts: 2104
Strand: Forward
Orientation: 3p
Minimum free energy: -31.40

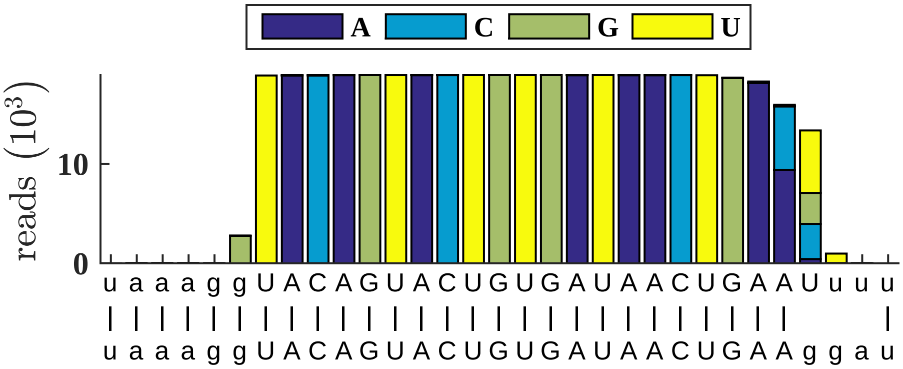

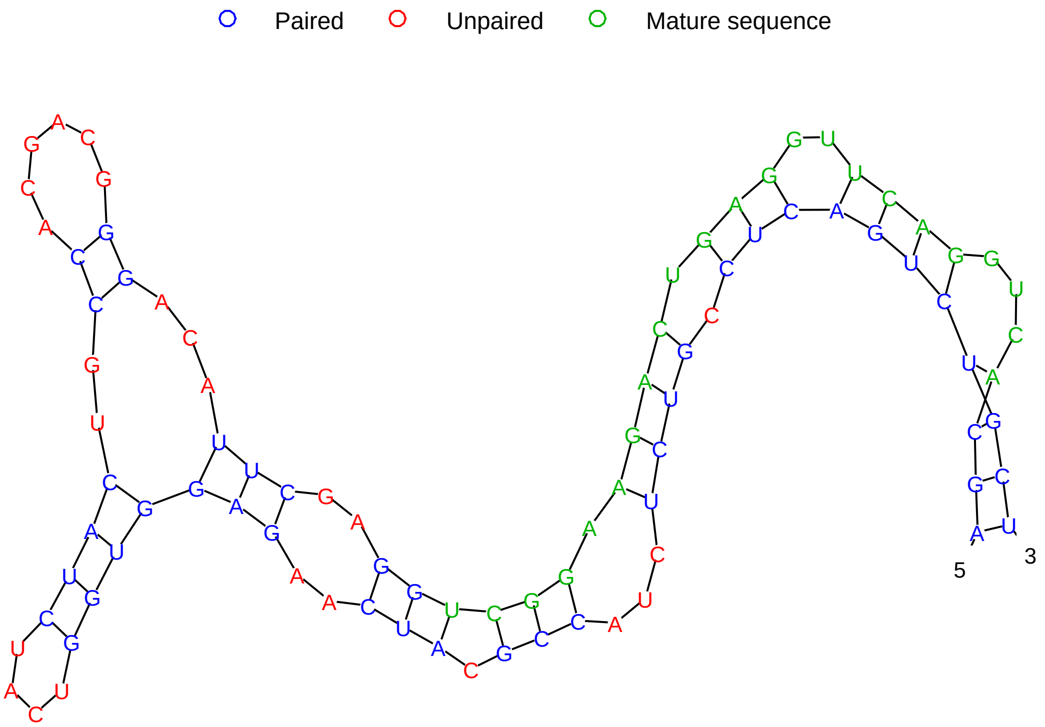

miRNA: bta-miR-378
Stem loop (UMD3.1): chr4:10715302-10715392
Mature (UMD3.1): chr4:10715305-10715327
Mature seq len: 23
Total raw counts (9 samples): 62407
Average raw counts: 6935
Strand: Forward
Orientation: 5p
Minimum free energy: -24.60

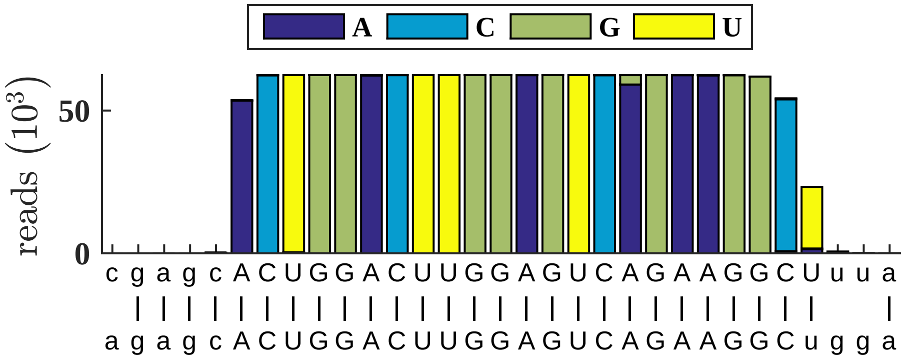

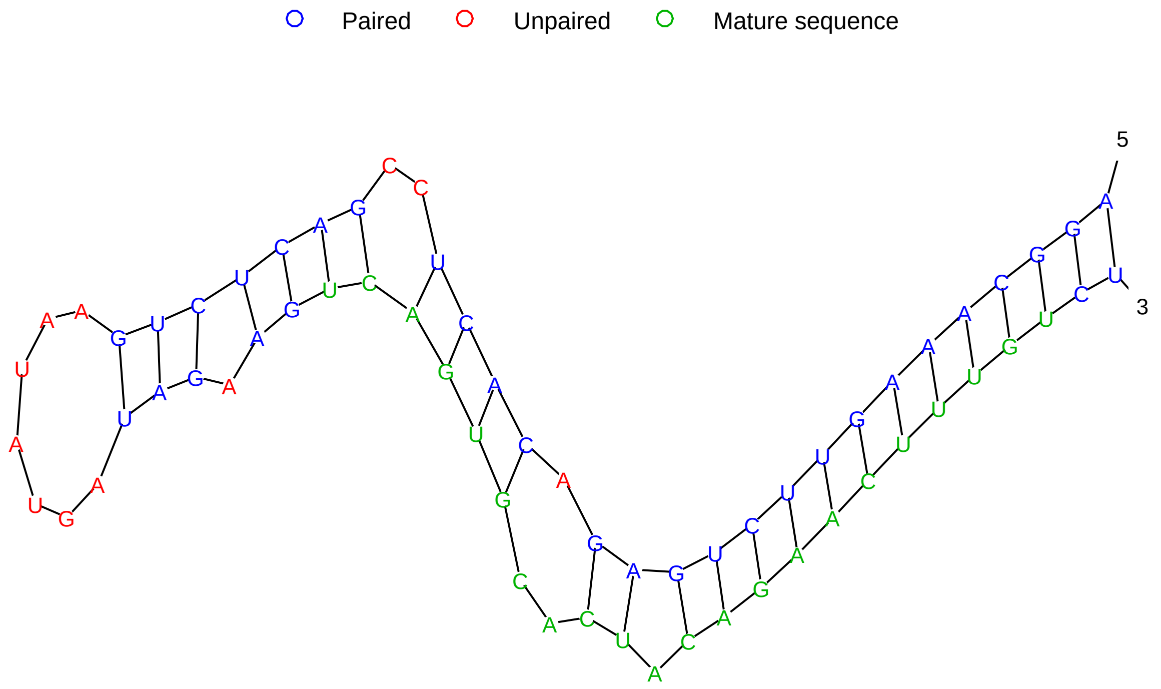

miRNA: bta-miR-148a
Stem loop (UMD3.1): chr4:70414252-70414317
Mature (UMD3.1): chr4:70414294-70414315
Mature seq len: 22
Total raw counts (9 samples): 457075
Average raw counts: 50787
Strand: Forward
Orientation: 3p
Minimum free energy: -25.00

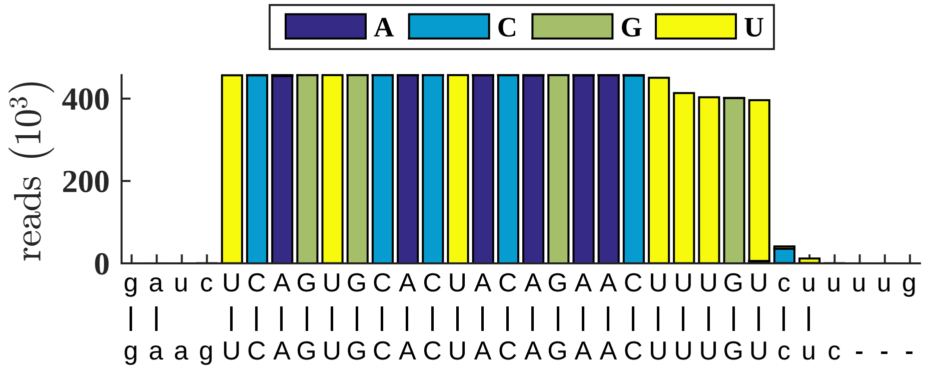

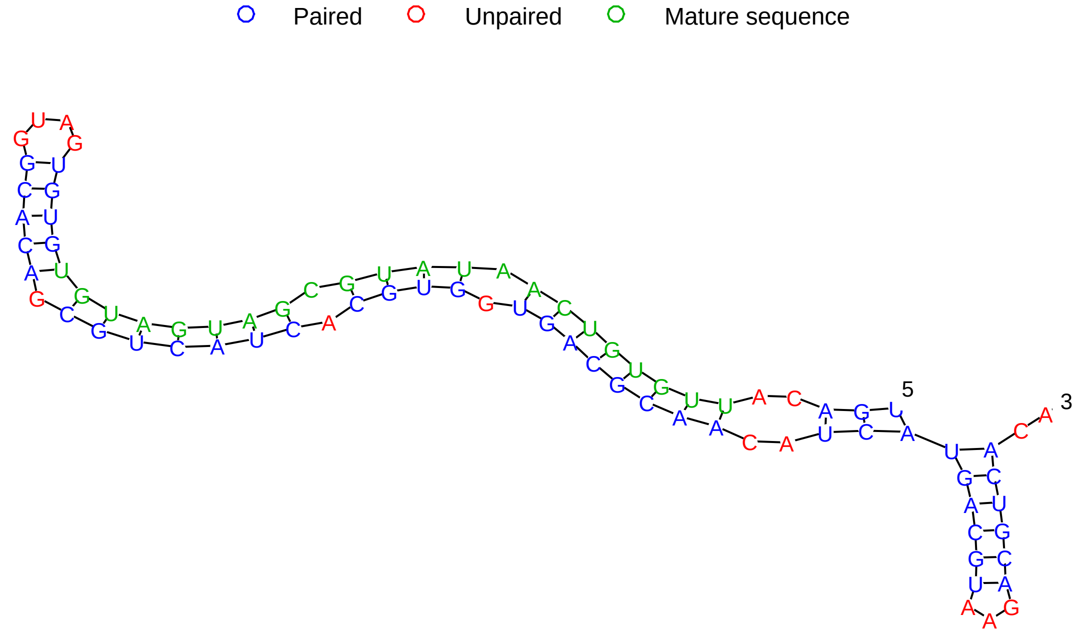

miRNA: bta-miR-592
Stem loop (UMD3.1): chr4:92126806-92126889
Mature (UMD3.1): chr4:92126863-92126884
Mature seq len: 22
Total raw counts (9 samples): 2178
Average raw counts: 242
Strand: Reverse
Orientation: 5p
Minimum free energy: -37.30

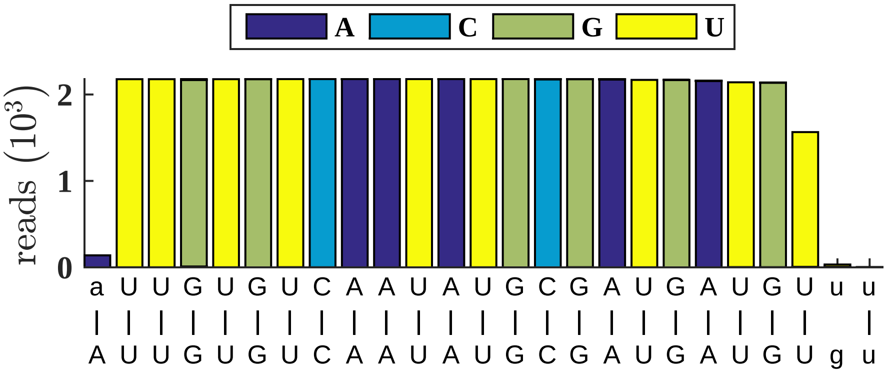

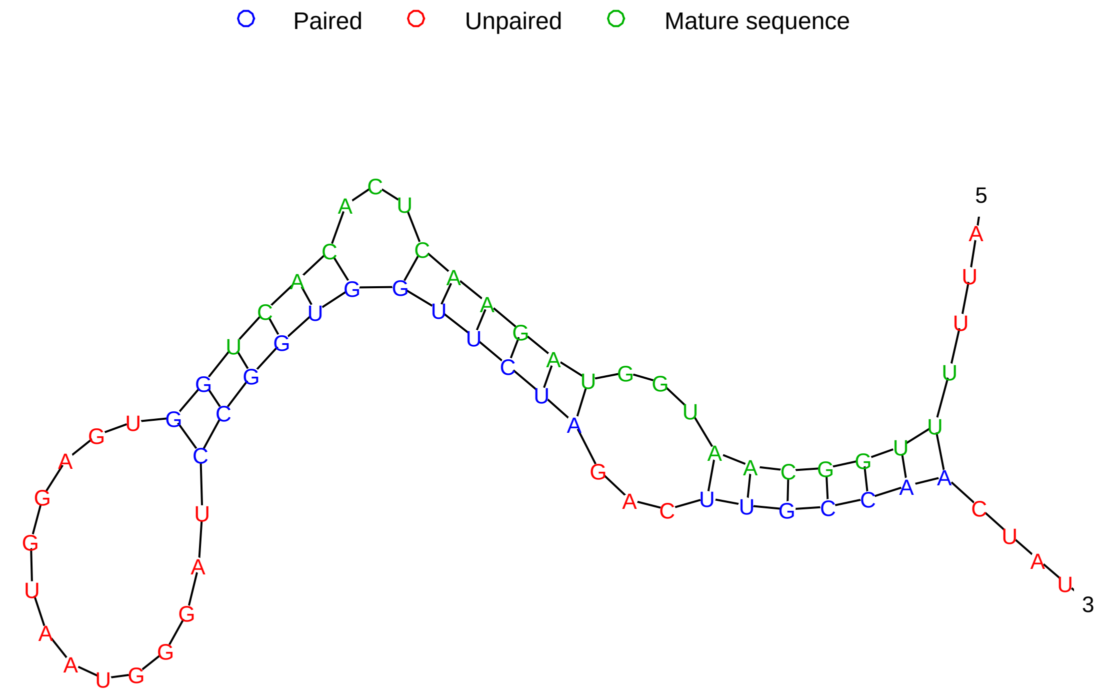

miRNA: bta-miR-182
Stem loop (UMD3.1): chr4:94406721-94406789
Mature (UMD3.1): chr4:94406763-94406786
Mature seq len: 24
Total raw counts (9 samples): 3646
Average raw counts: 406
Strand: Reverse
Orientation: 5p
Minimum free energy: -22.00

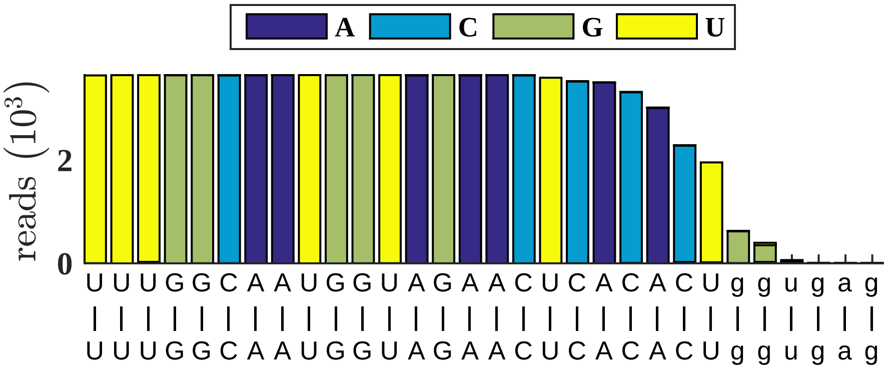

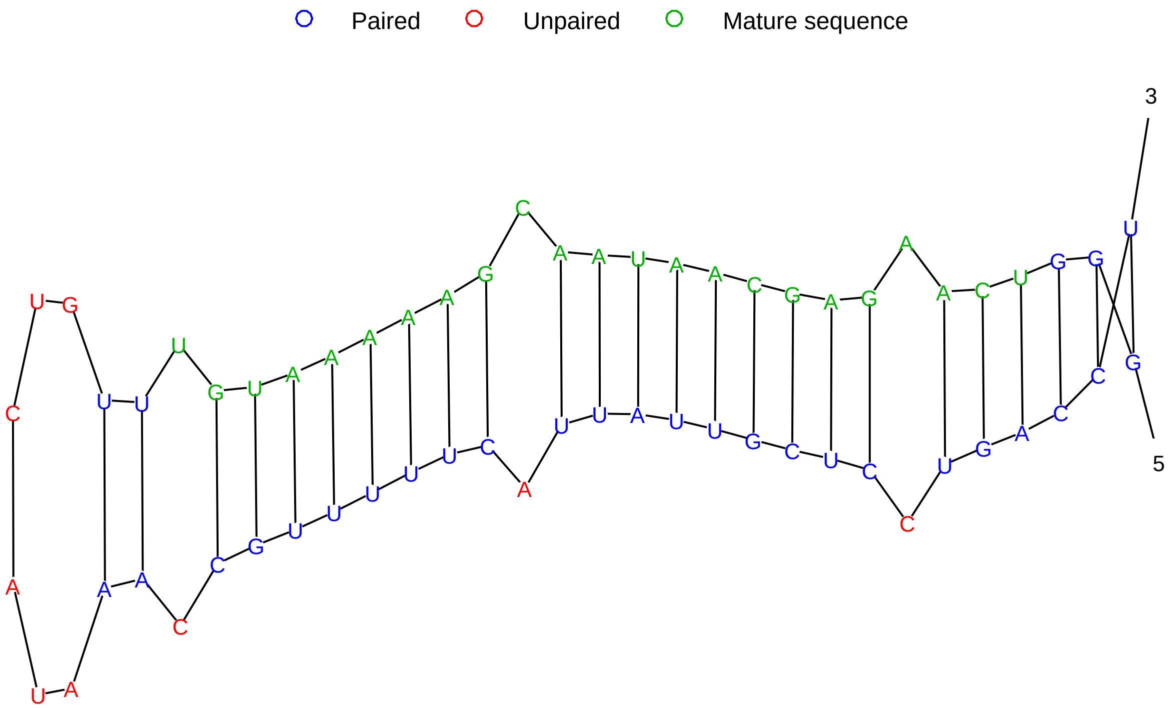

miRNA: bta-miR-335
Stem loop (UMD3.1): chr4:95071003-95071064
Mature (UMD3.1): chr4:95071006-95071028
Mature seq len: 23
Total raw counts (9 samples): 32751
Average raw counts: 3639
Strand: Forward
Orientation: 5p
Minimum free energy: -28.00

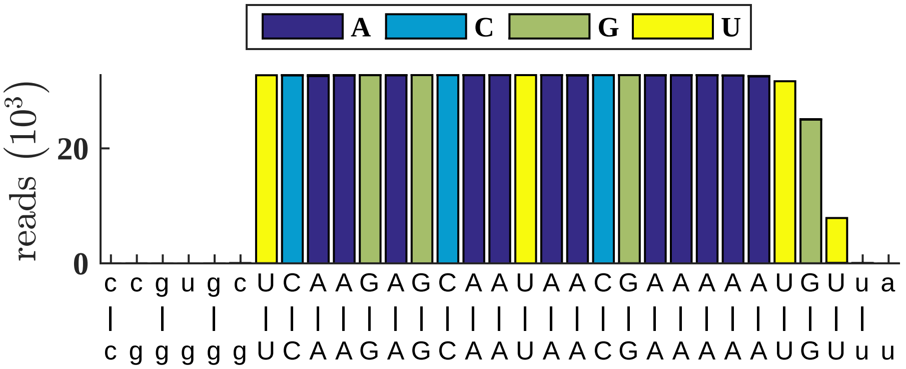

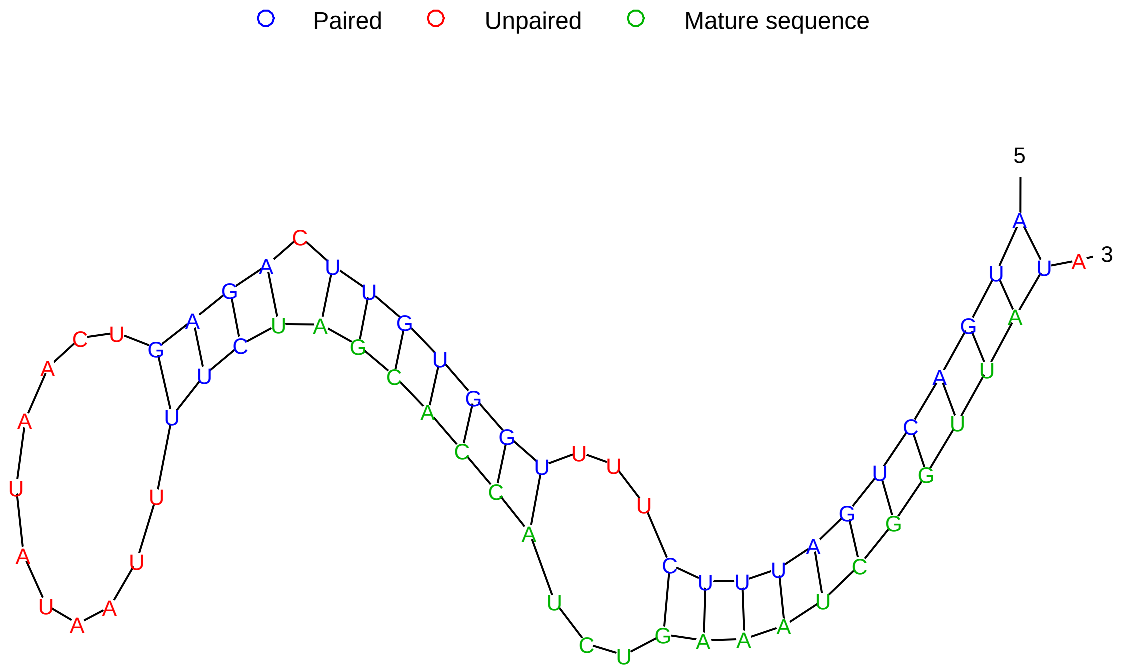

miRNA: bta-miR-29a
Stem loop (UMD3.1): chr4:95402318-95402382
Mature (UMD3.1): chr4:95402320-95402341
Mature seq len: 22
Total raw counts (9 samples): 55662
Average raw counts: 6185
Strand: Reverse
Orientation: 3p
Minimum free energy: -25.30

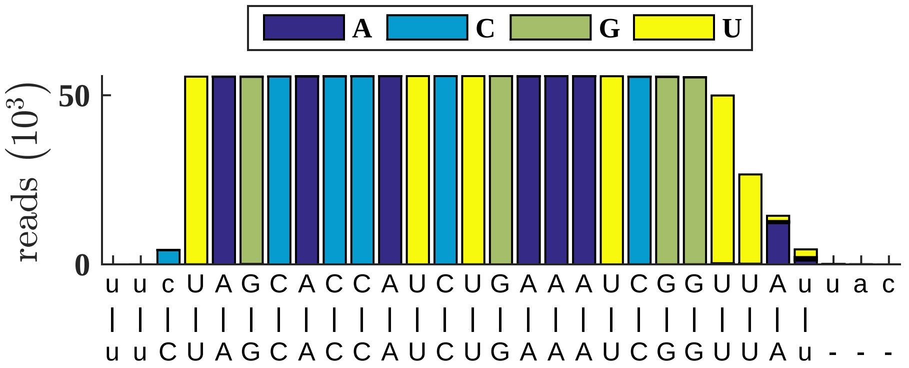

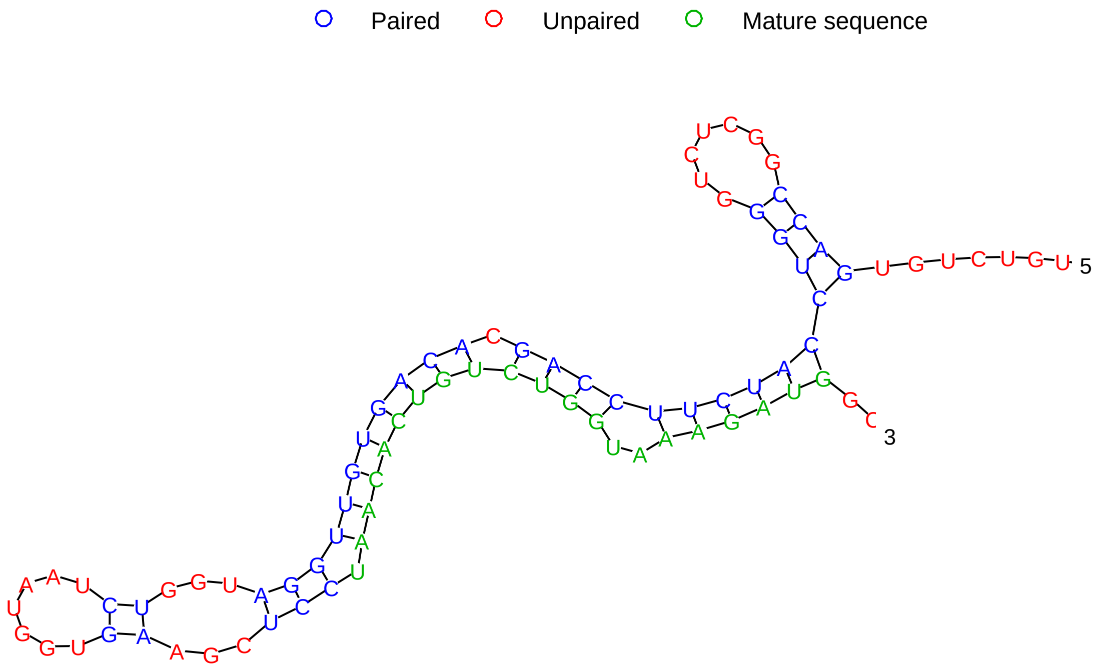

miRNA: bta-miR-141
Stem loop (UMD3.1): chr5:103859015-103859101
Mature (UMD3.1): chr5:103859017-103859037
Mature seq len: 21
Total raw counts (9 samples): 947
Average raw counts: 106
Strand: Reverse
Orientation: 3p
Minimum free energy: -31.70

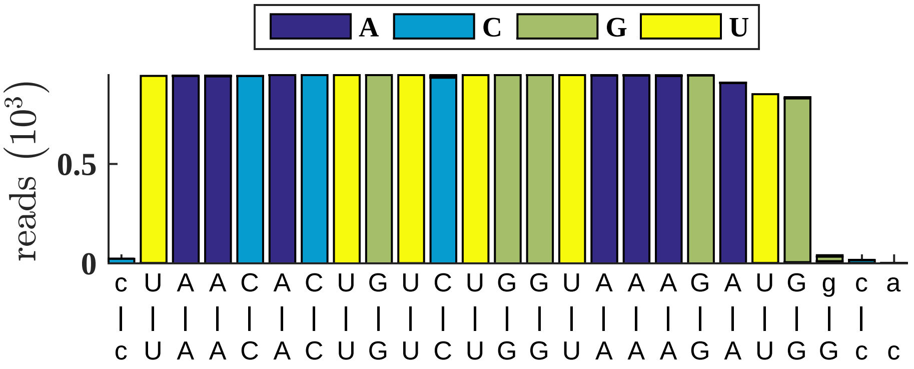

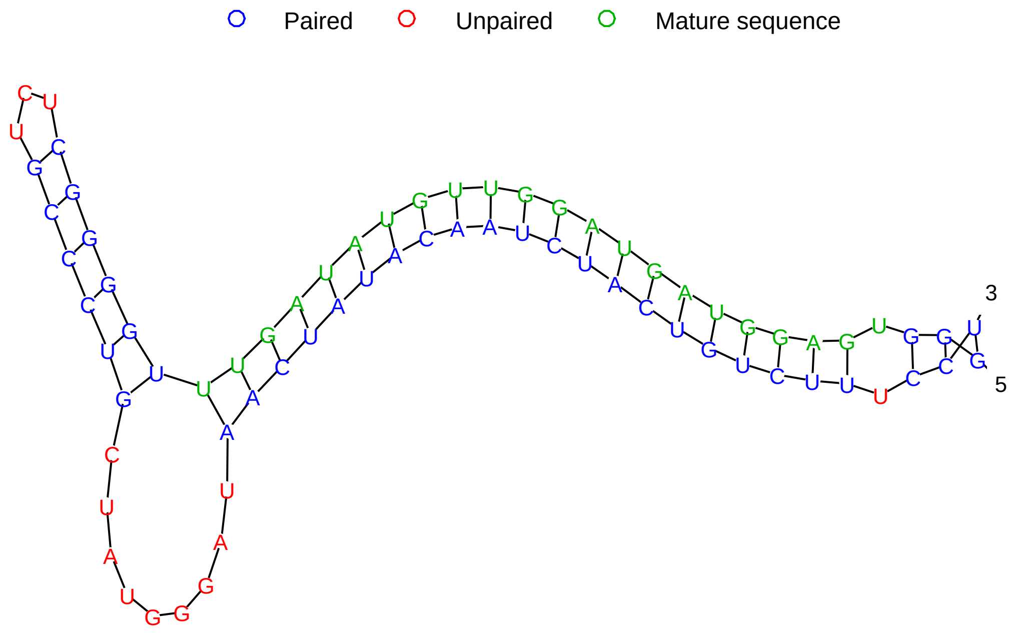

miRNA: bta-let-7a-5p
Stem loop (UMD3.1): chr5:117119385-117119458
Mature (UMD3.1): chr5:117119388-117119409
Mature seq len: 22
Total raw counts (9 samples): 76608
Average raw counts: 8512
Strand: Forward
Orientation: 5p
Minimum free energy: -34.10

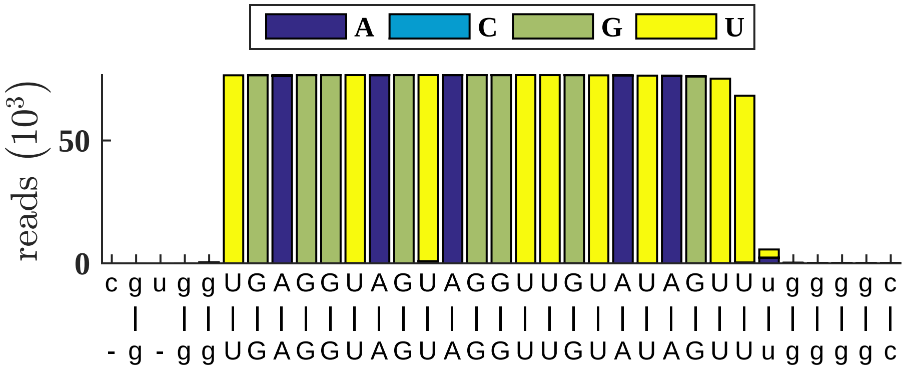

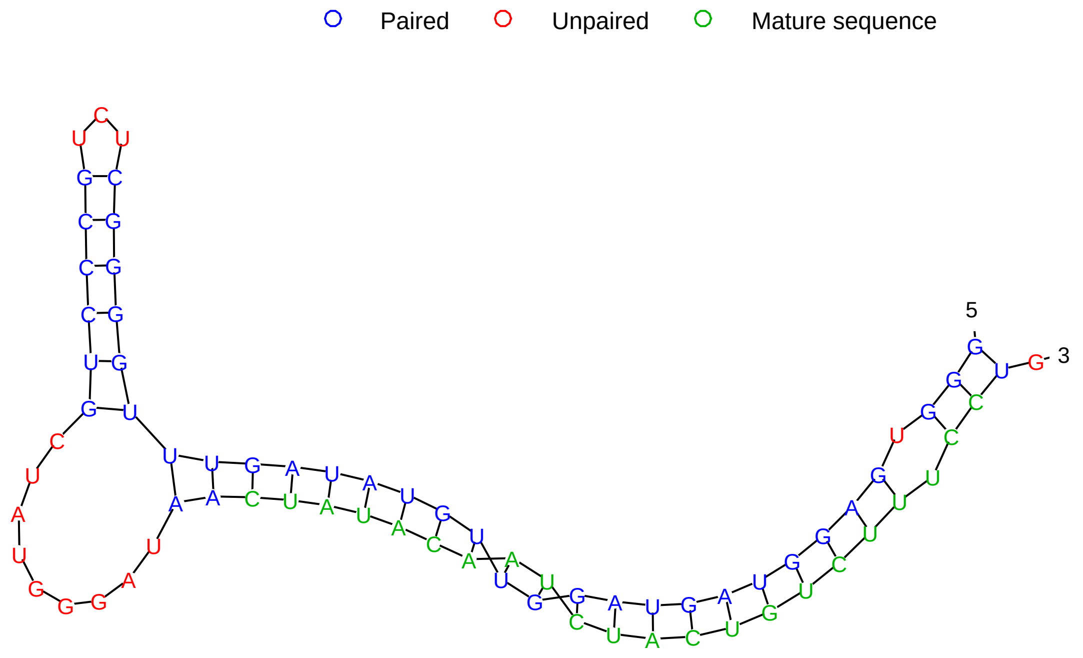

miRNA: bta-let-7a-3p
Stem loop (UMD3.1): chr5:117119385-117119459
Mature (UMD3.1): chr5:117119436-117119457
Mature seq len: 22
Total raw counts (9 samples): 1244
Average raw counts: 139
Strand: Forward
Orientation: 3p
Minimum free energy: -34.80

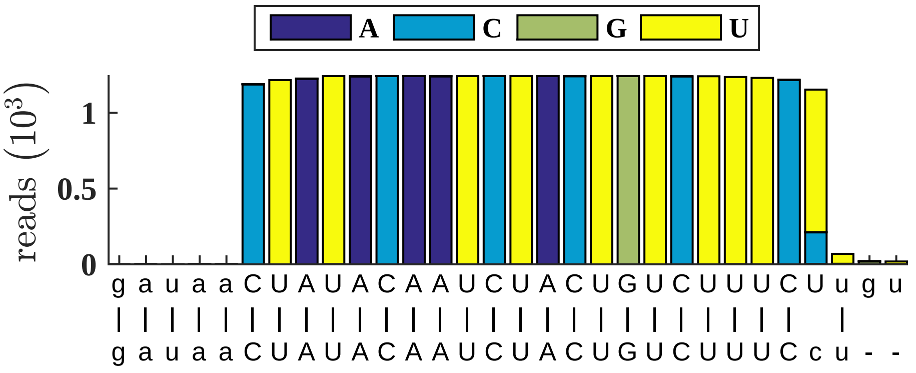

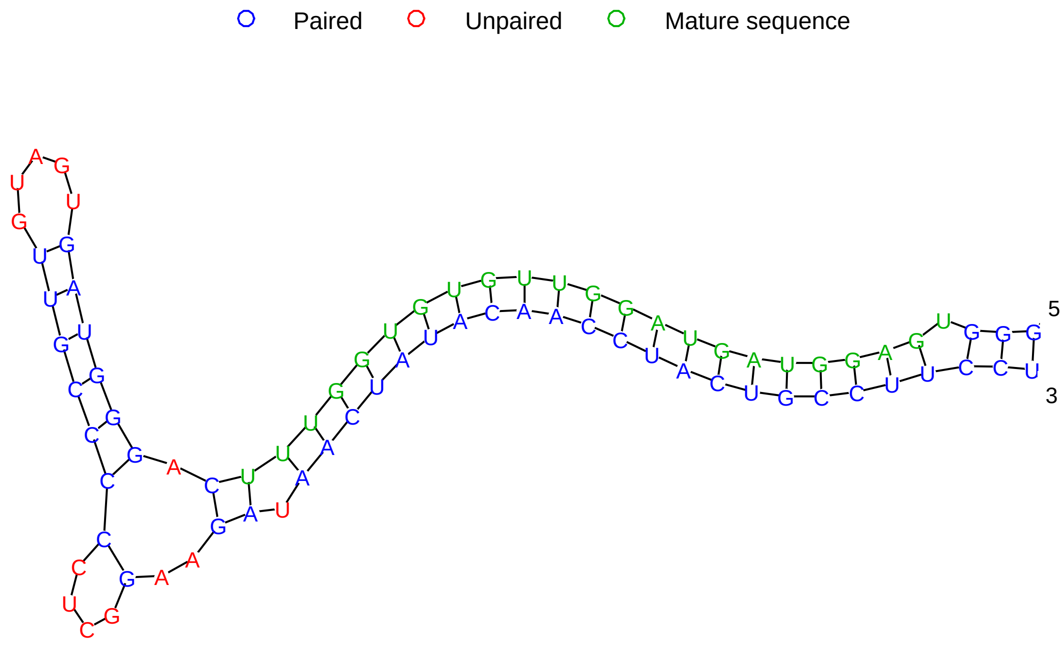

miRNA: bta-let-7b
Stem loop (UMD3.1): chr5:117120187-117120266
Mature (UMD3.1): chr5:117120190-117120212
Mature seq len: 23
Total raw counts (9 samples): 153329
Average raw counts: 17037
Strand: Forward
Orientation: 5p
Minimum free energy: -39.30

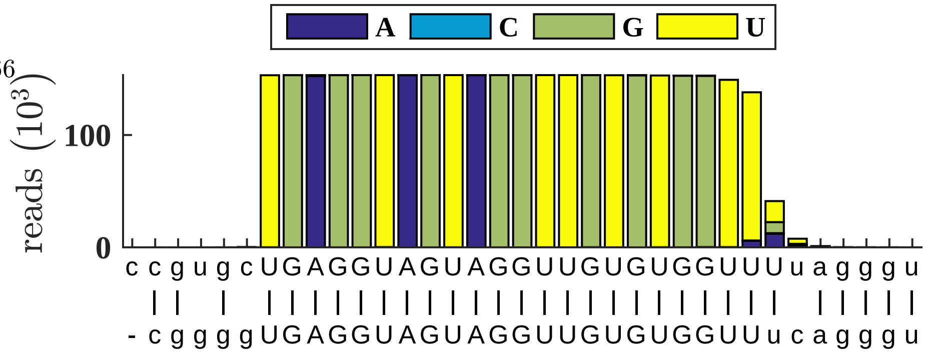

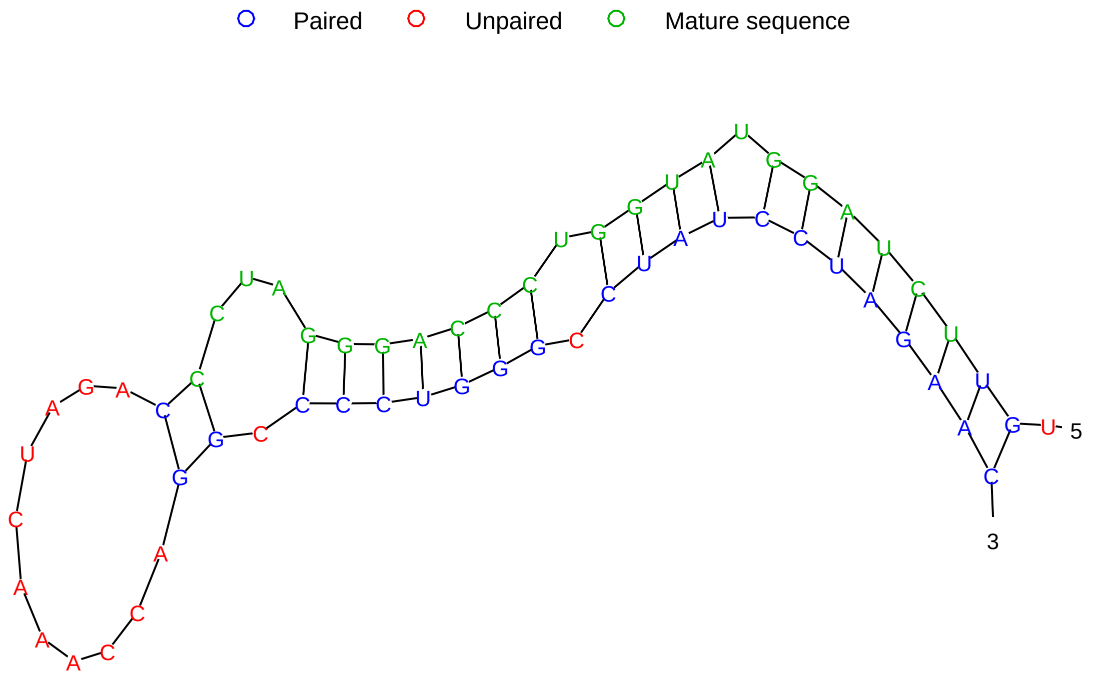

miRNA: bta-miR-331-5p
Stem loop (UMD3.1): chr5:25109078-25109138
Mature (UMD3.1): chr5:25109081-25109103
Mature seq len: 23
Total raw counts (9 samples): 1903
Average raw counts: 212
Strand: Forward
Orientation: 5p
Minimum free energy: -29.90

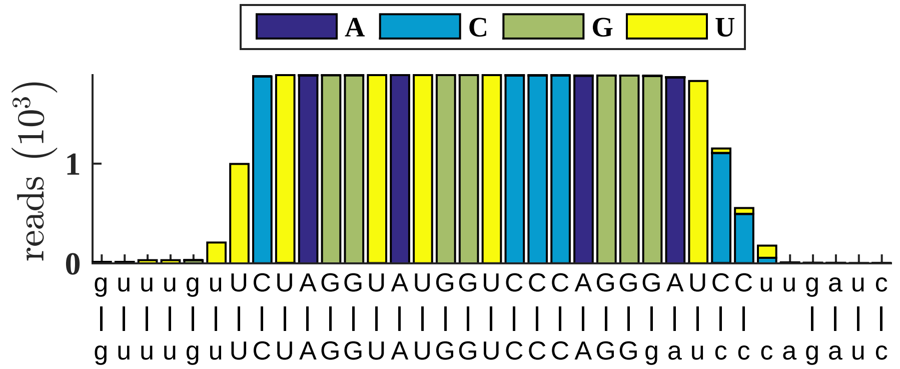

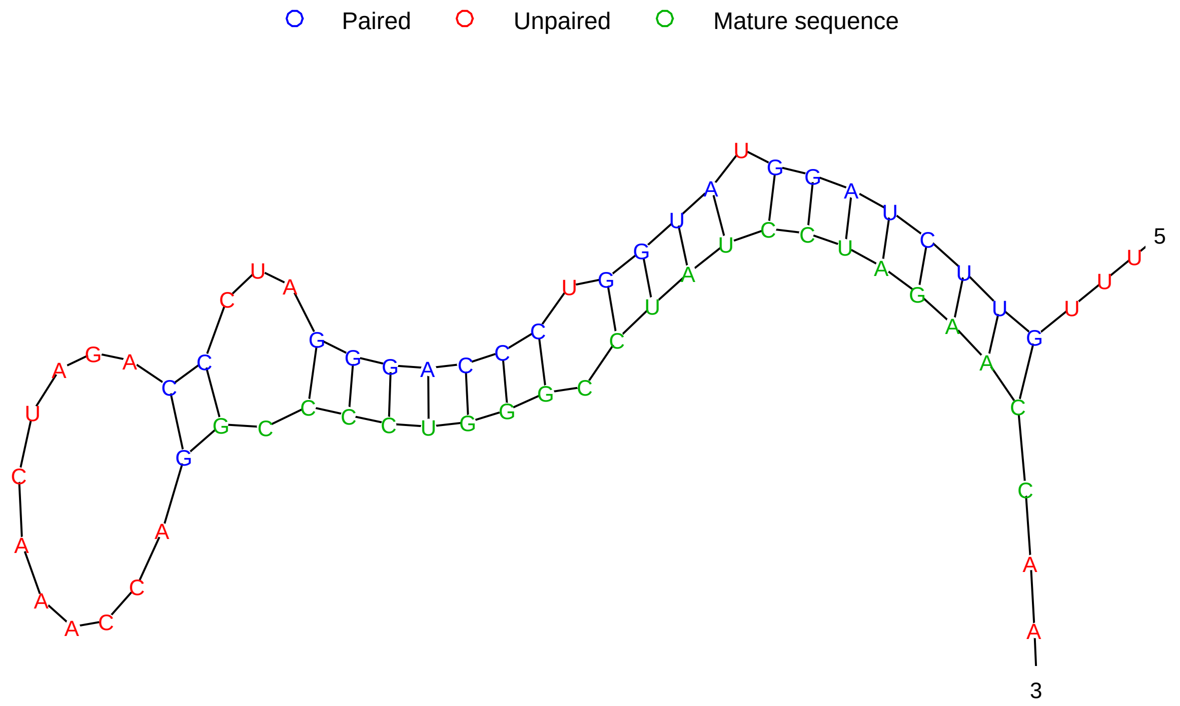

miRNA: bta-miR-331-3p
Stem loop (UMD3.1): chr5:25109076-25109141
Mature (UMD3.1): chr5:25109117-25109139
Mature seq len: 23
Total raw counts (9 samples): 947
Average raw counts: 106
Strand: Forward
Orientation: 3p
Minimum free energy: -30.70

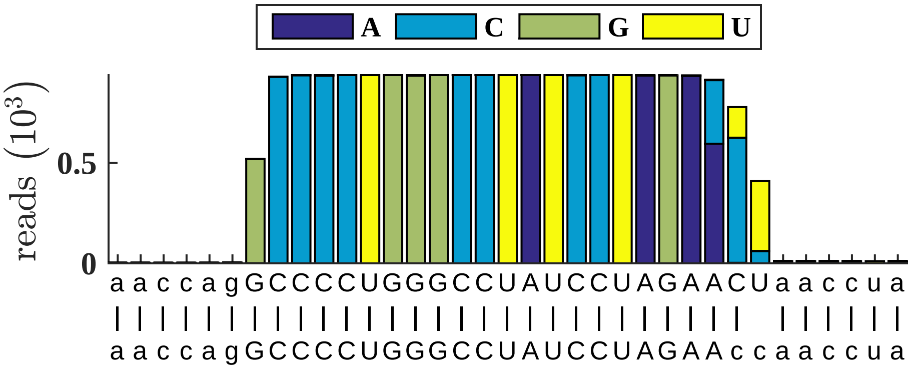

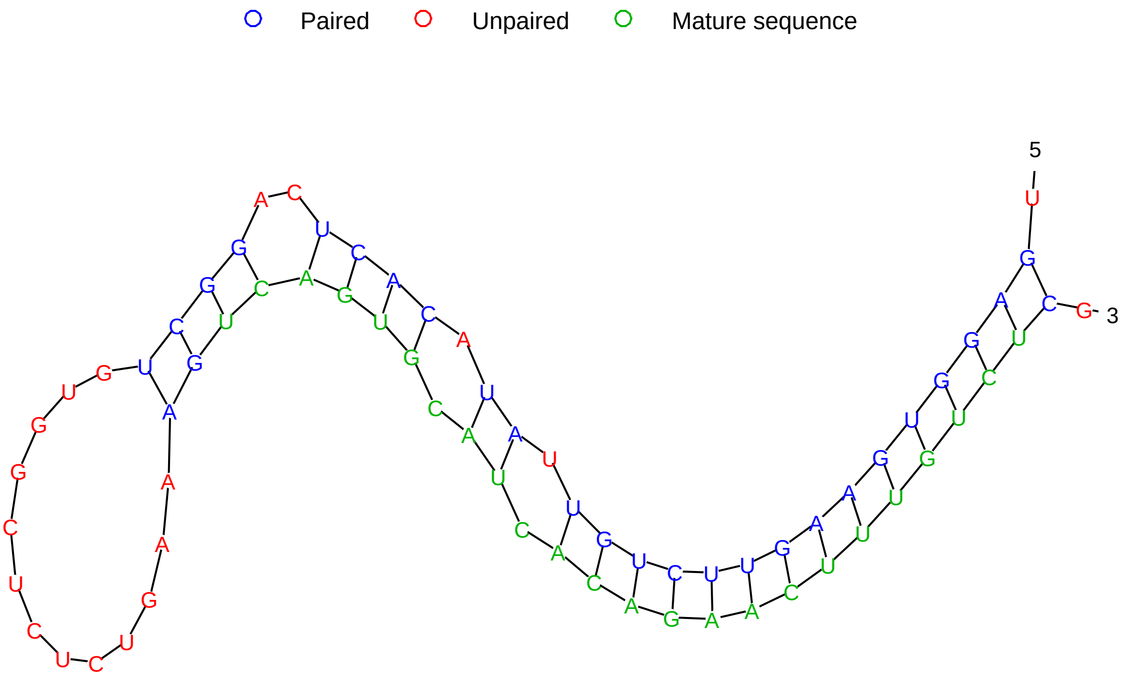

miRNA: bta-miR-148b
Stem loop (UMD3.1): chr5:25849775-25849845
Mature (UMD3.1): chr5:25849777-25849800
Mature seq len: 24
Total raw counts (9 samples): 32934
Average raw counts: 3660
Strand: Reverse
Orientation: 3p
Minimum free energy: -24.00

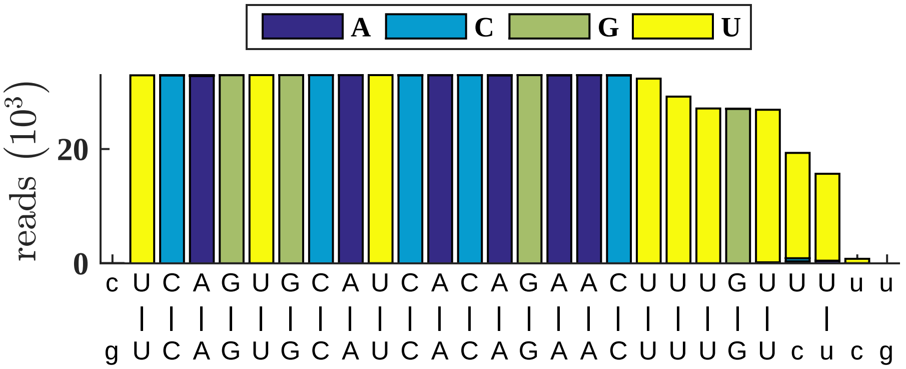

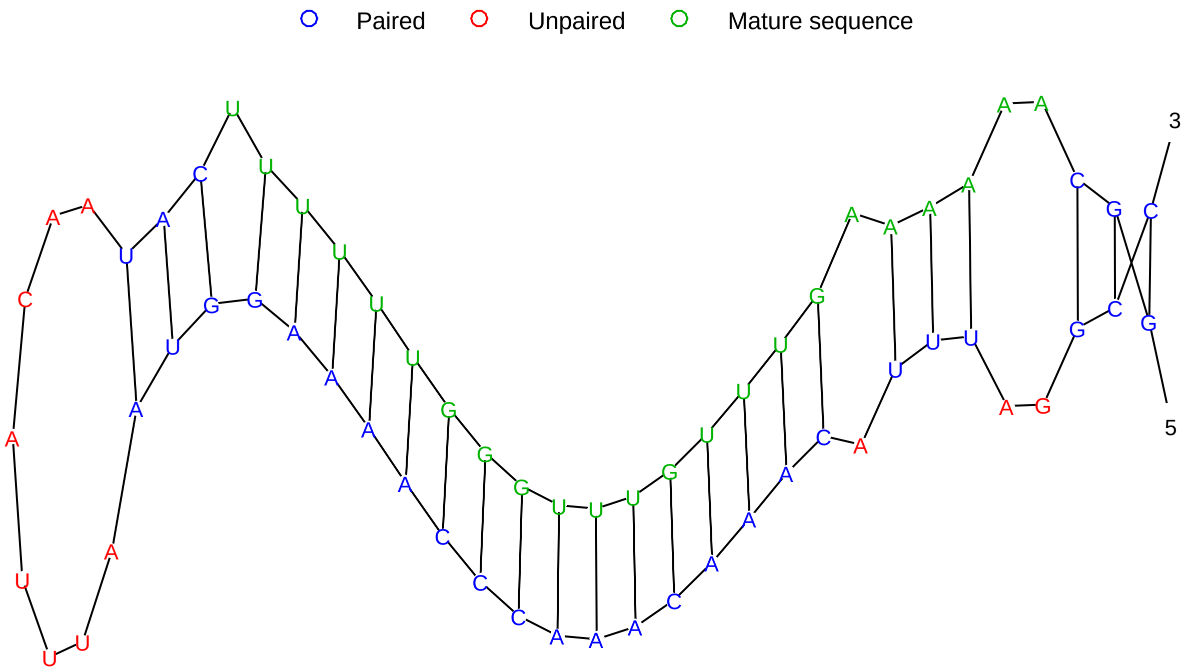

miRNA: bta-miR-2284z
Stem loop (UMD3.1): chr5:43233818-43233882
Mature (UMD3.1): chr5:43233821-43233843
Mature seq len: 23
Total raw counts (9 samples): 782
Average raw counts: 87
Strand: Forward
Orientation: 5p
Minimum free energy: -27.20

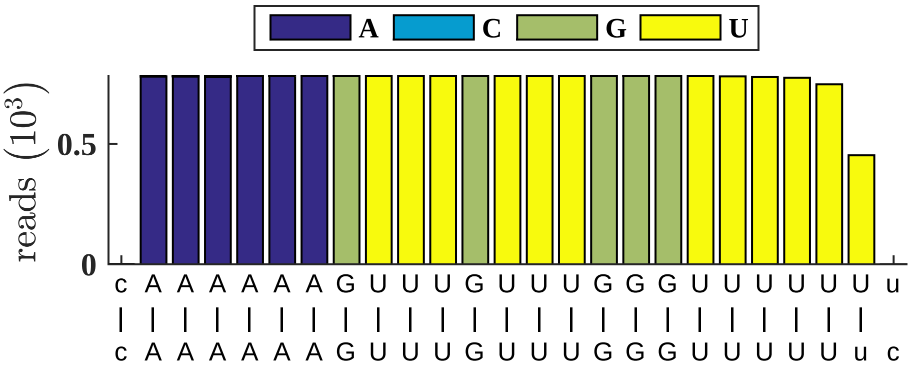

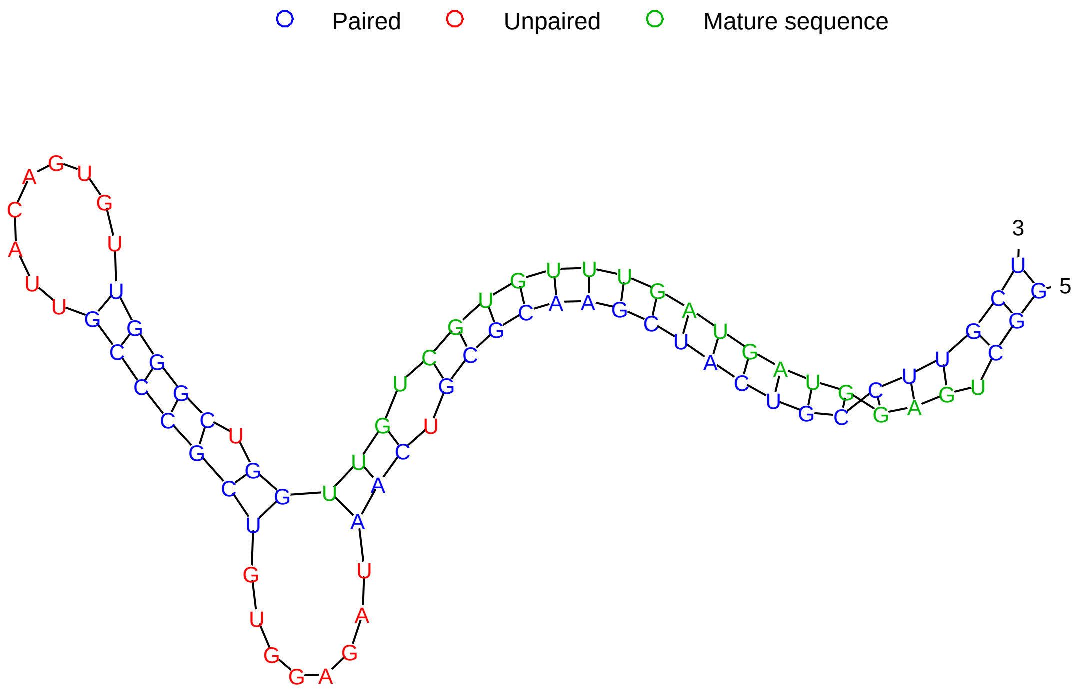

miRNA: bta-let-7i
Stem loop (UMD3.1): chr5:51209082-51209162
Mature (UMD3.1): chr5:51209138-51209159
Mature seq len: 22
Total raw counts (9 samples): 148473
Average raw counts: 16497
Strand: Reverse
Orientation: 5p
Minimum free energy: -38.60

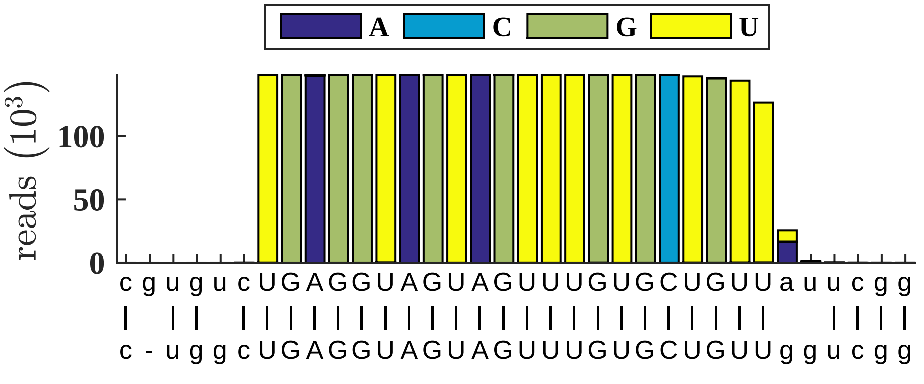

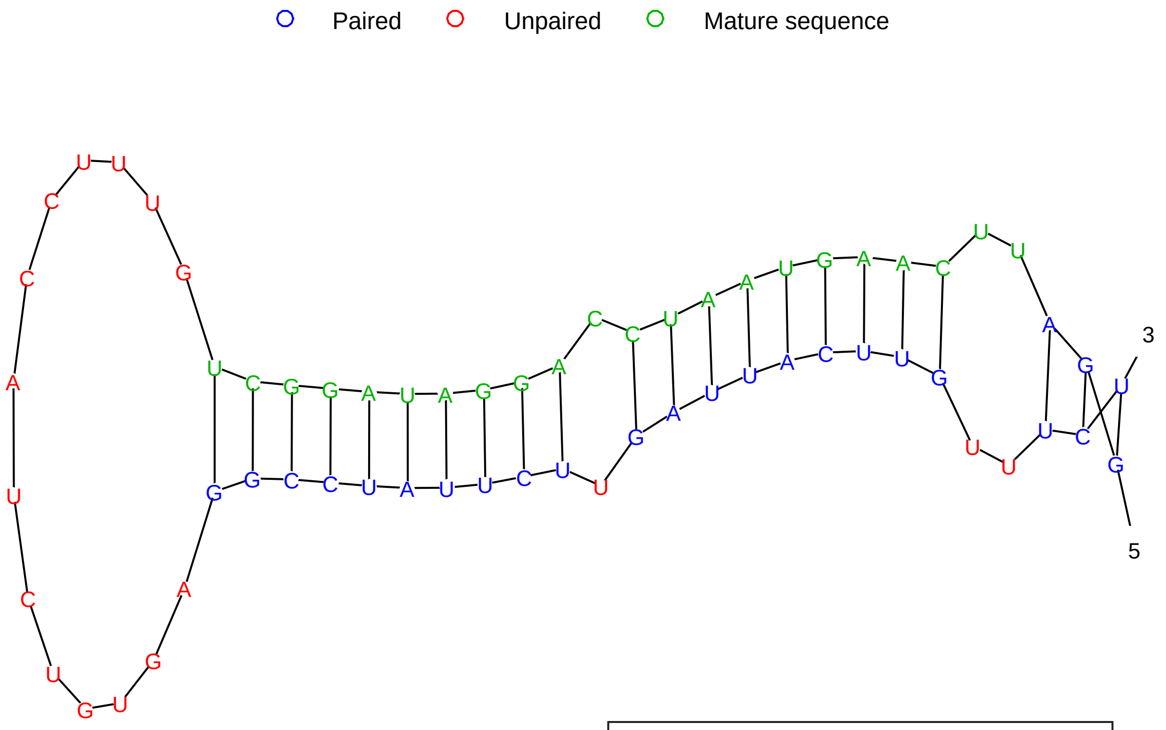

miRNA: bta-miR-26a
Stem loop (UMD3.1): chr5:55977933-55977996
Mature (UMD3.1): chr5:55977936-55977957
Mature seq len: 22
Total raw counts (9 samples): 204261
Average raw counts: 22696
Strand: Forward
Orientation: 5p
Minimum free energy: -28.00

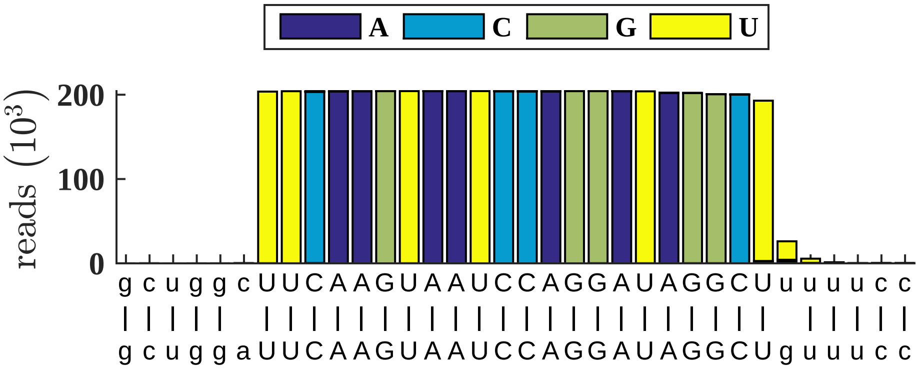

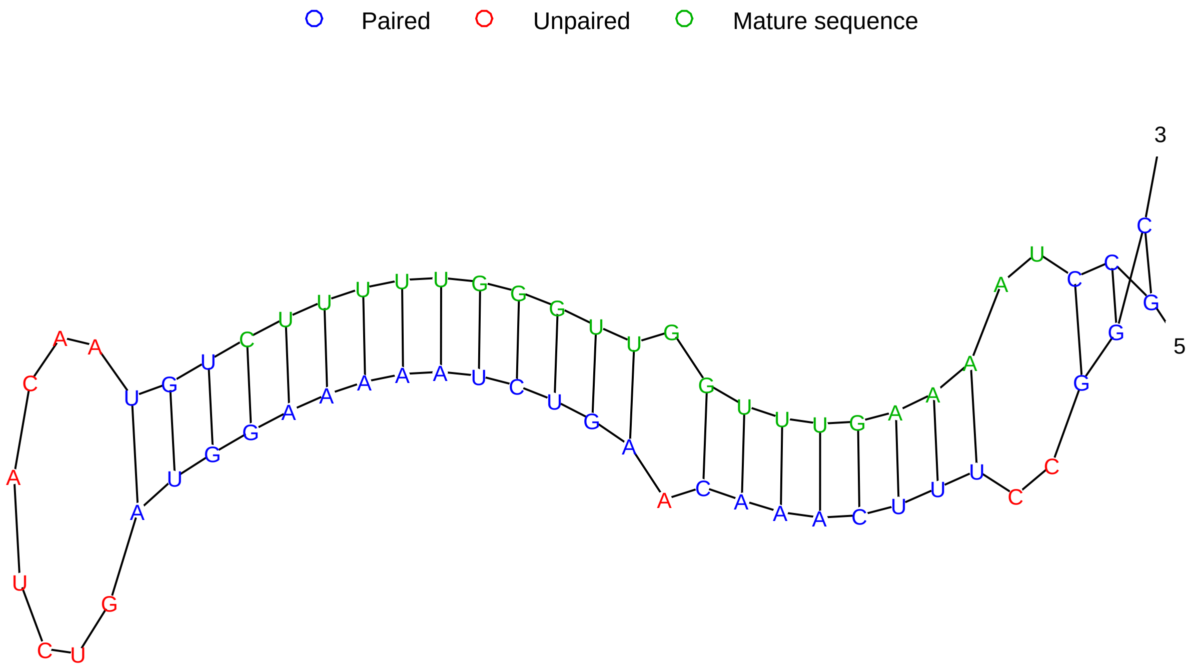

miRNA: bta-miR-2284ab
Stem loop (UMD3.1): chr6:103797775-103797838
Mature (UMD3.1): chr6:103797778-103797799
Mature seq len: 22
Total raw counts (9 samples): 4978
Average raw counts: 554
Strand: Forward
Orientation: 5p
Minimum free energy: -24.10

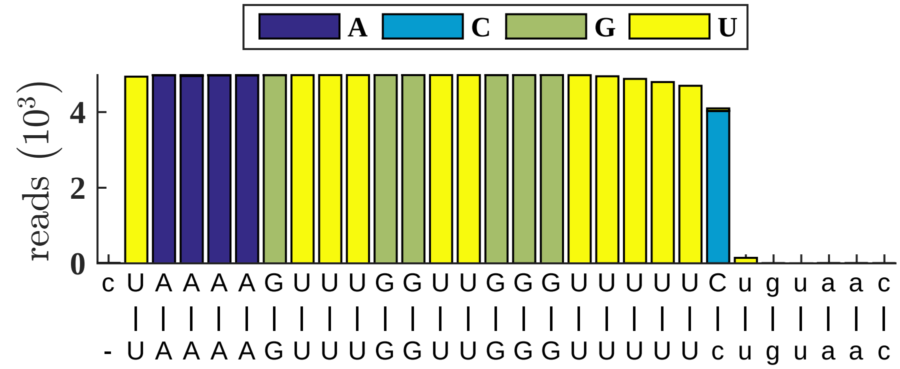

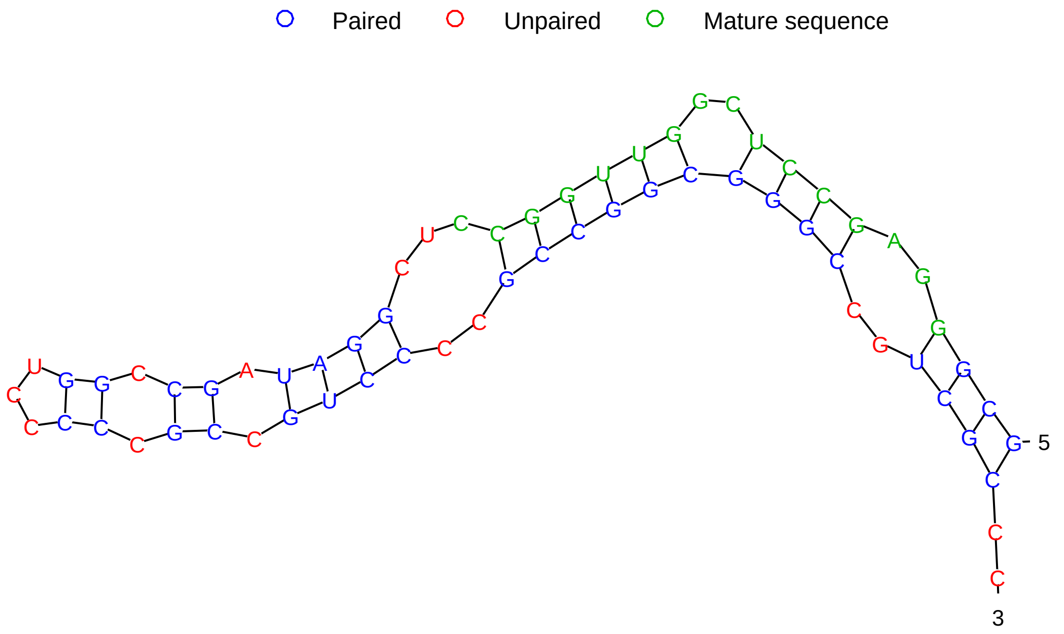

miRNA: bta-miR-2904
Stem loop (UMD3.1): chr6:92539380-92539443
Mature (UMD3.1): chr6:92539383-92539398
Mature seq len: 16
Total raw counts (9 samples): 556
Average raw counts: 62
Strand: Forward
Orientation: 5p
Minimum free energy: -27.90

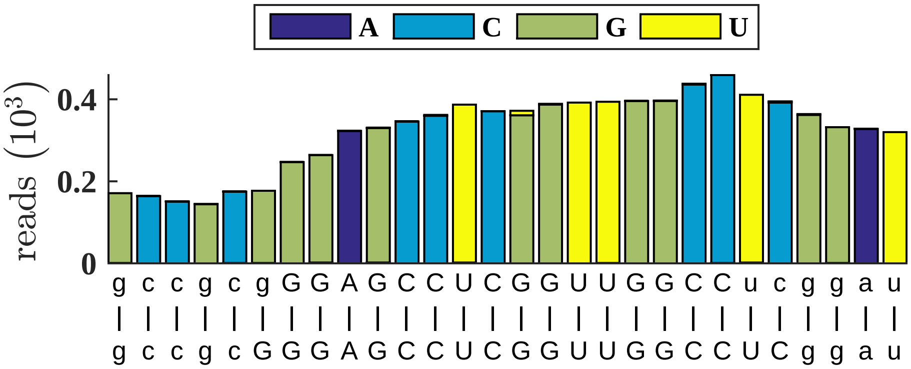

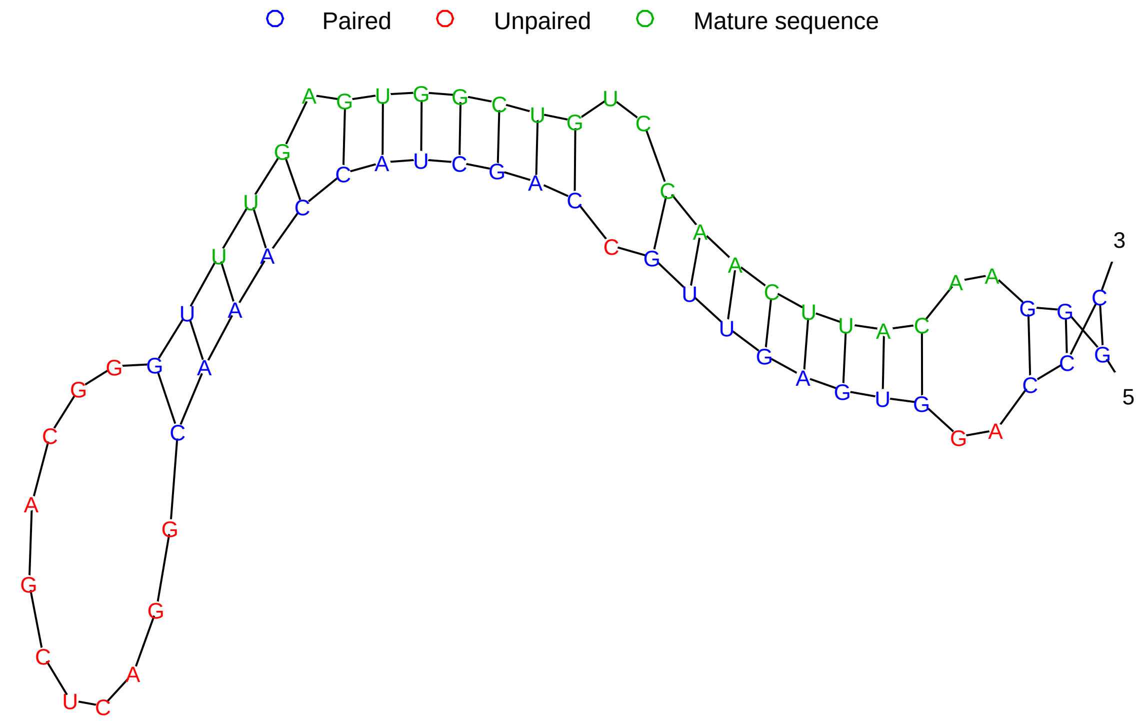

miRNA: bta-miR-181c
Stem loop (UMD3.1): chr7:12915087-12915151
Mature (UMD3.1): chr7:12915090-12915112
Mature seq len: 23
Total raw counts (9 samples): 1852
Average raw counts: 206
Strand: Forward
Orientation: 5p
Minimum free energy: -29.70

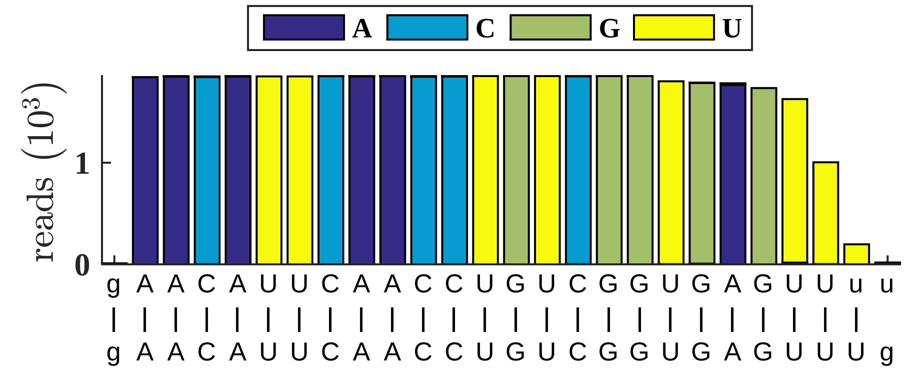

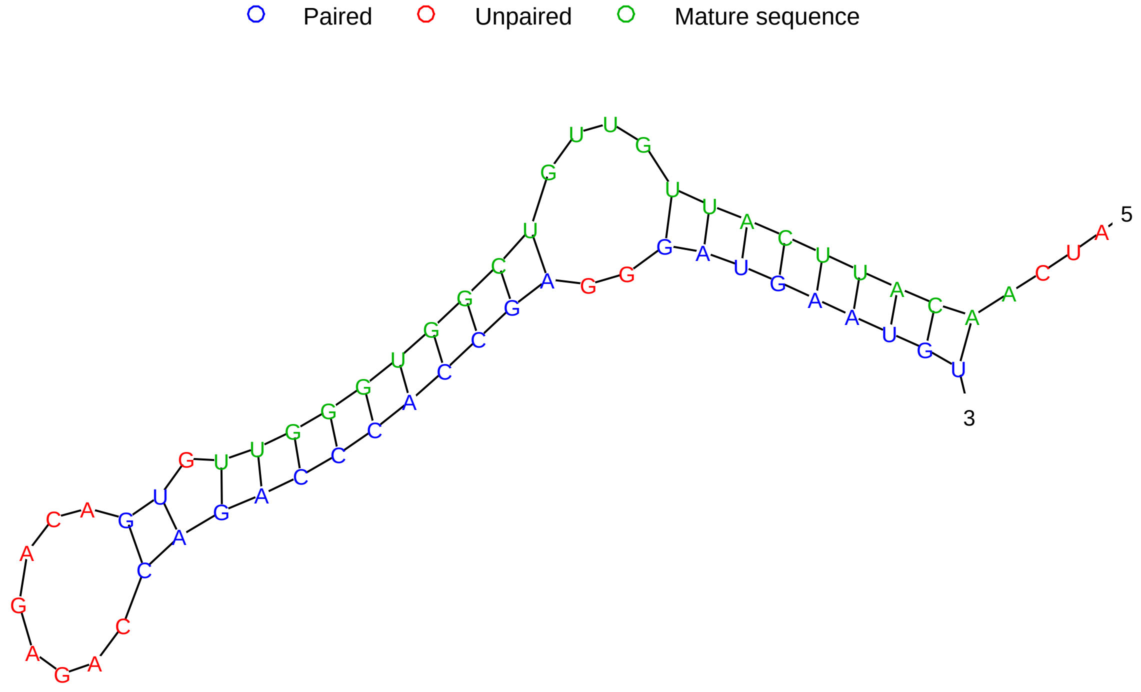

miRNA: bta-miR-181d
Stem loop (UMD3.1): chr7:12915269-12915329
Mature (UMD3.1): chr7:12915272-12915295
Mature seq len: 24
Total raw counts (9 samples): 422
Average raw counts: 47
Strand: Forward
Orientation: 5p
Minimum free energy: -27.80

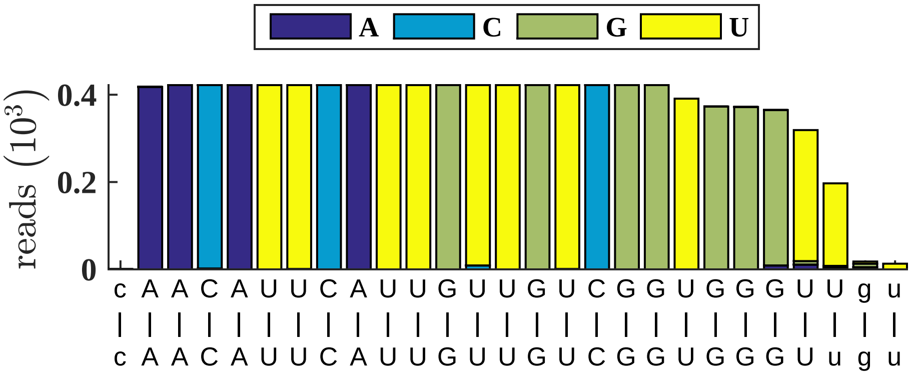

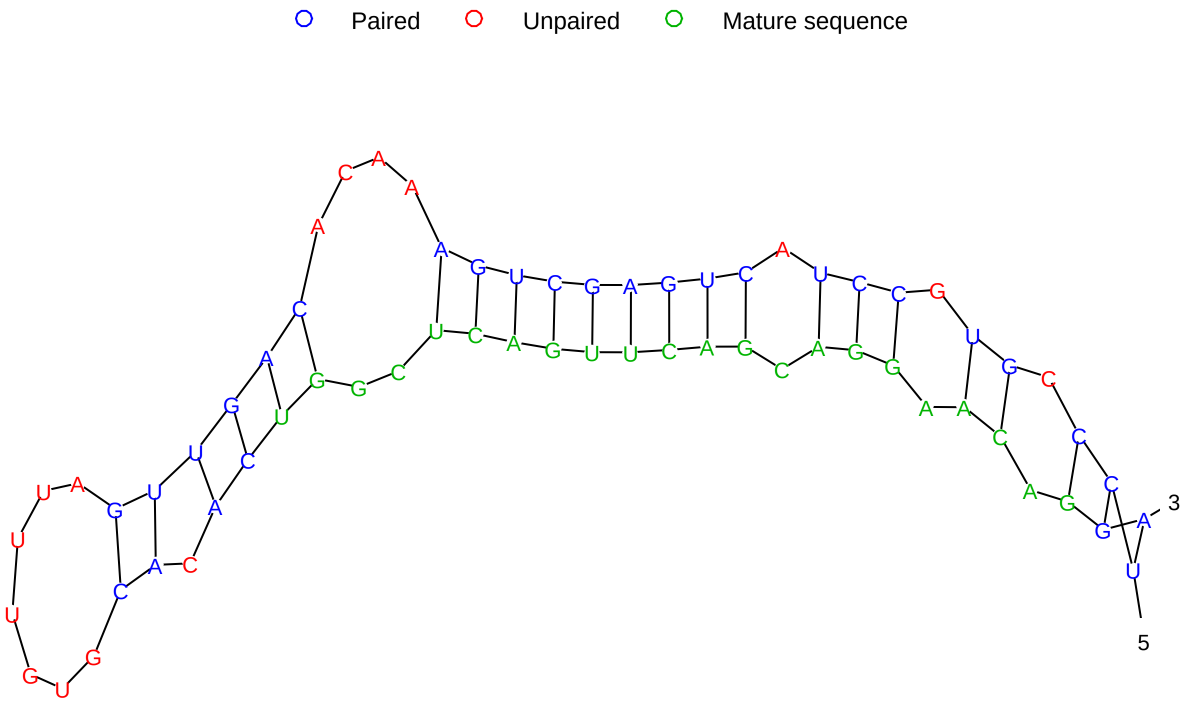

miRNA: bta-miR-24-3p
Stem loop (UMD3.1): chr7:12981641-12981706
Mature (UMD3.1): chr7:12981643-12981664
Mature seq len: 22
Total raw counts (9 samples): 3356
Average raw counts: 373
Strand: Reverse
Orientation: 3p
Minimum free energy: -24.20

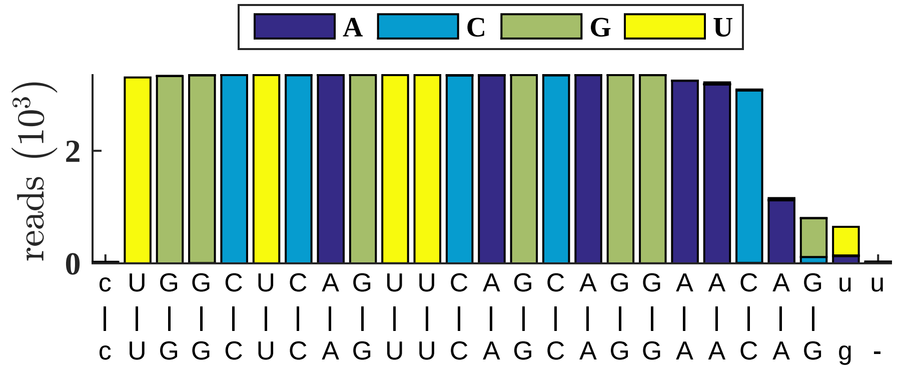

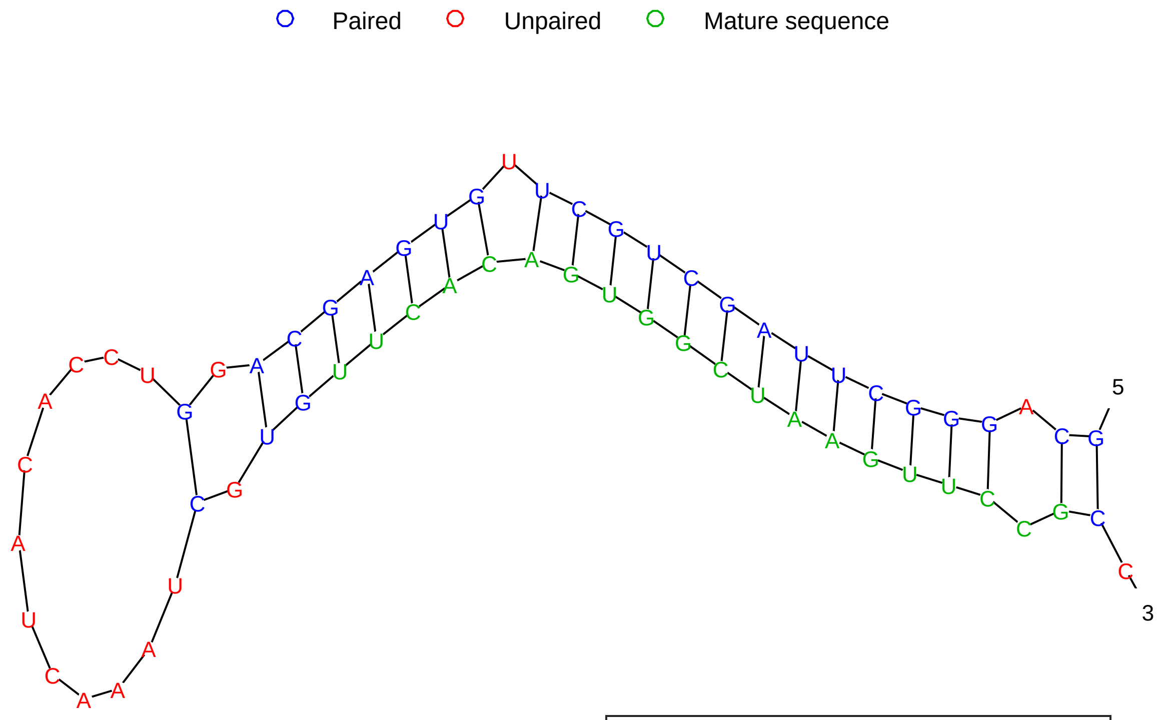

miRNA: bta-miR-27a-3p
Stem loop (UMD3.1): chr7:12981794-12981857
Mature (UMD3.1): chr7:12981796-12981815
Mature seq len: 20
Total raw counts (9 samples): 10533
Average raw counts: 1171
Strand: Reverse
Orientation: 3p
Minimum free energy: -30.90

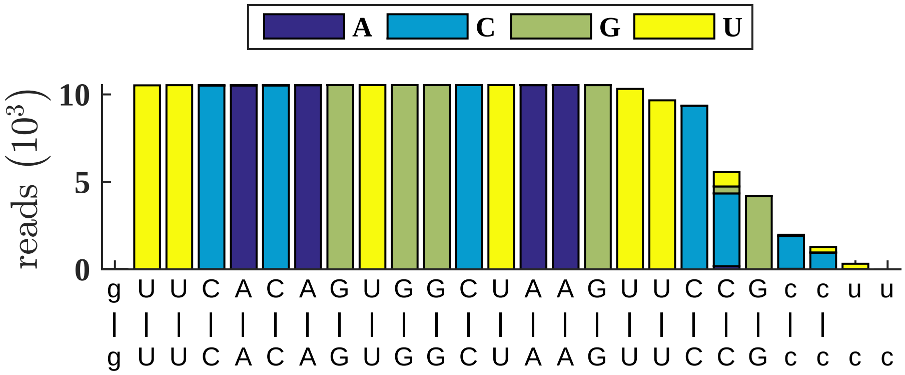

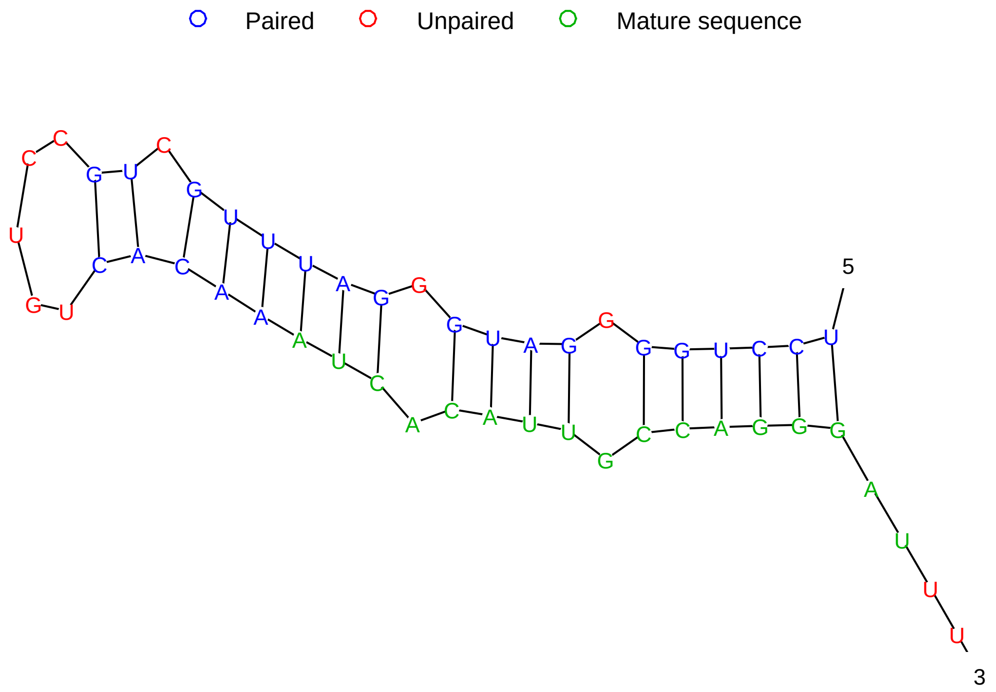

miRNA: bta-miR-23a
Stem loop (UMD3.1): chr7:12981980-12982029
Mature (UMD3.1): chr7:12981982-12981998
Mature seq len: 17
Total raw counts (9 samples): 23278
Average raw counts: 2587
Strand: Reverse
Orientation: 3p
Minimum free energy: -20.70

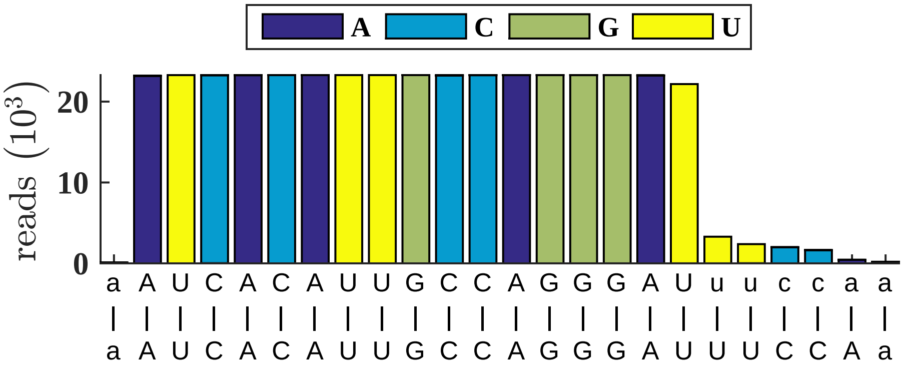

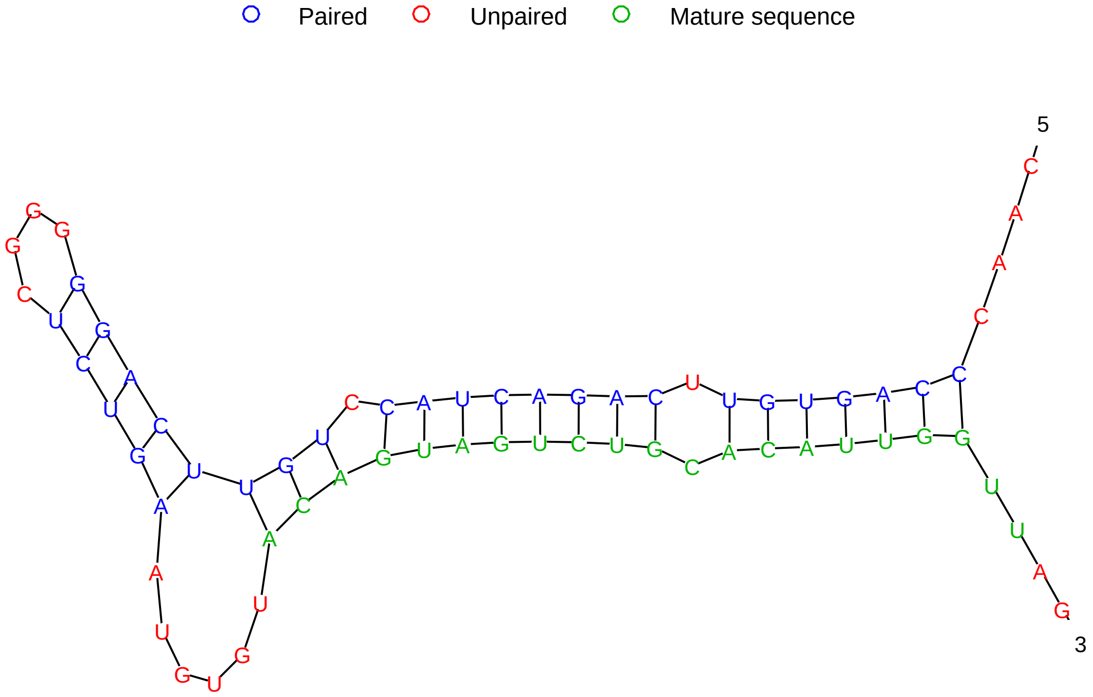

miRNA: bta-miR-199a-3p
Stem loop (UMD3.1): chr7:16508928-16508994
Mature (UMD3.1): chr7:16508930-16508950
Mature seq len: 21
Total raw counts (9 samples): 6321
Average raw counts: 703
Strand: Reverse
Orientation: 3p
Minimum free energy: -26.10

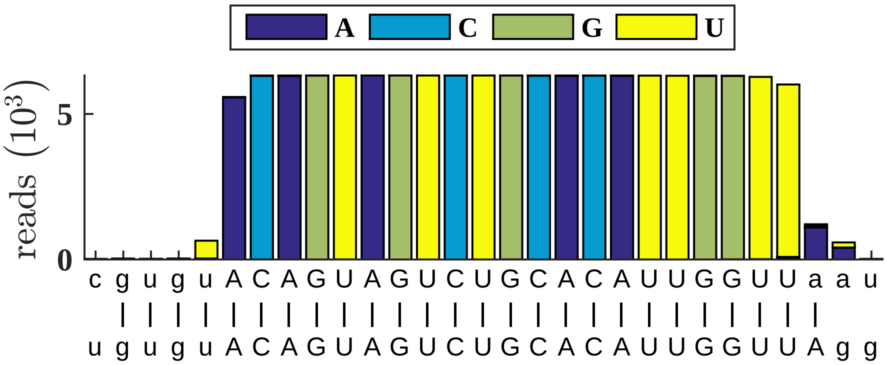

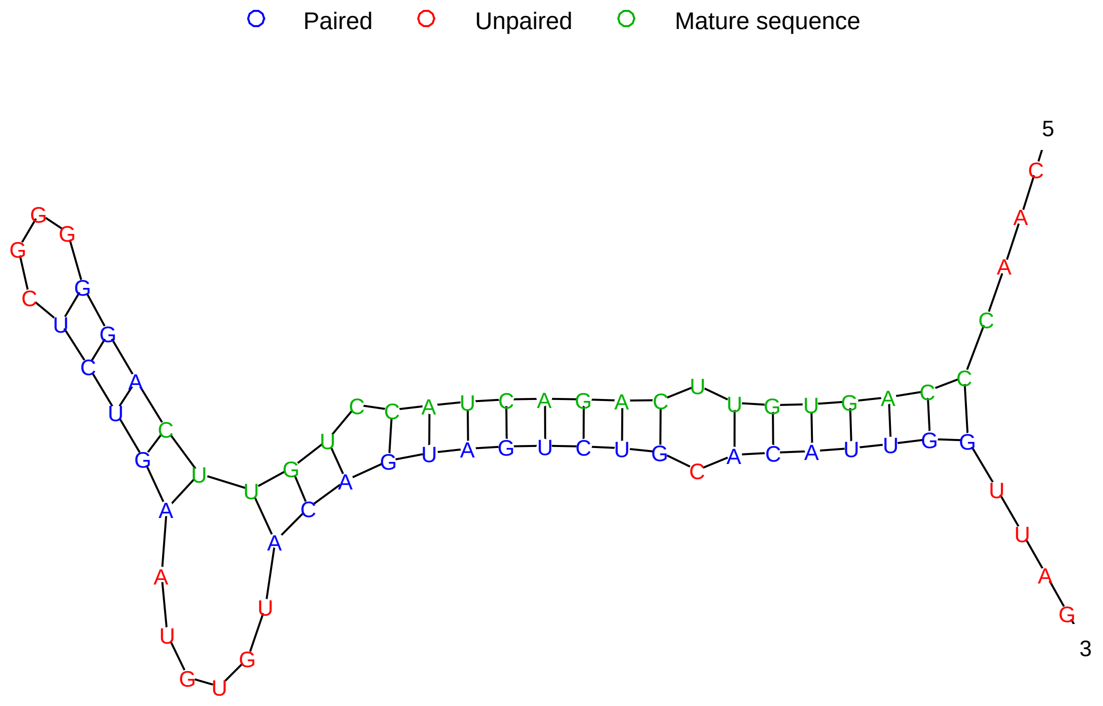

miRNA: bta-miR-199a-5p
Stem loop (UMD3.1): chr7:16508928-16508994
Mature (UMD3.1): chr7:16508969-16508991
Mature seq len: 23
Total raw counts (9 samples): 738
Average raw counts: 82
Strand: Reverse
Orientation: 5p
Minimum free energy: -26.10

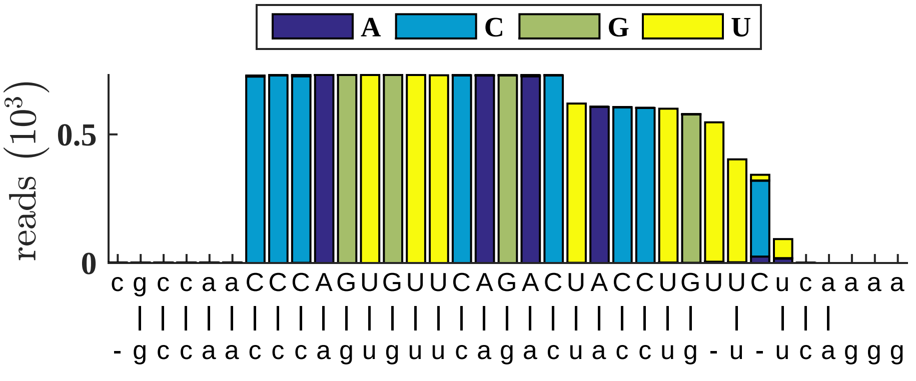

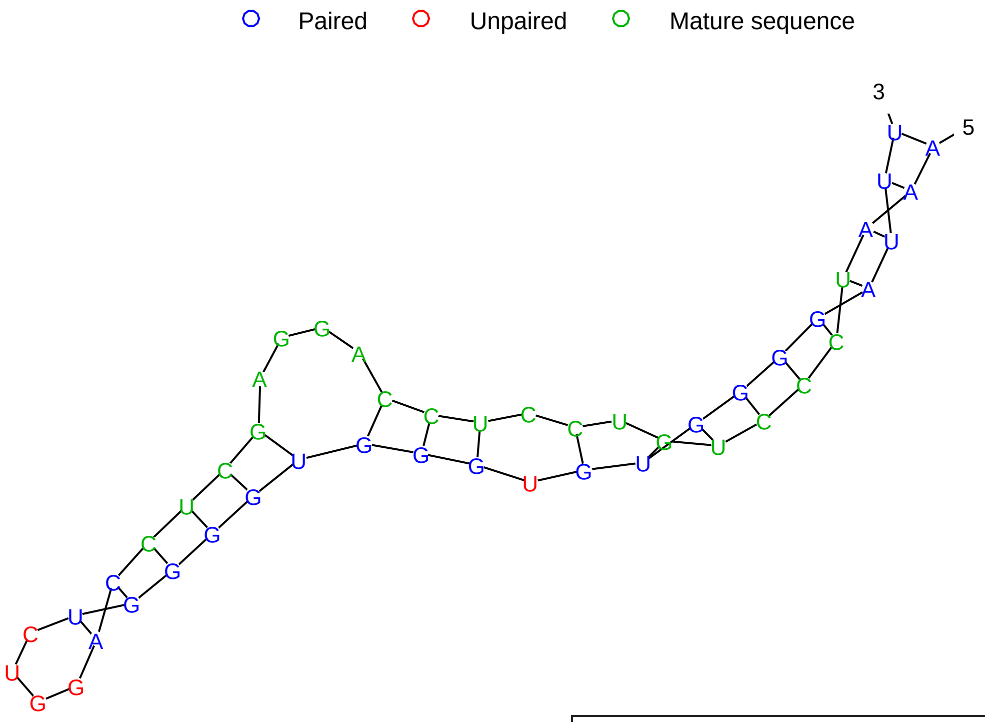

miRNA: bta-miR-339b
Stem loop (UMD3.1): chr7:18379992-18380040
Mature (UMD3.1): chr7:18380018-18380037
Mature seq len: 20
Total raw counts (9 samples): 2128
Average raw counts: 237
Strand: Reverse
Orientation: 5p
Minimum free energy: -17.80

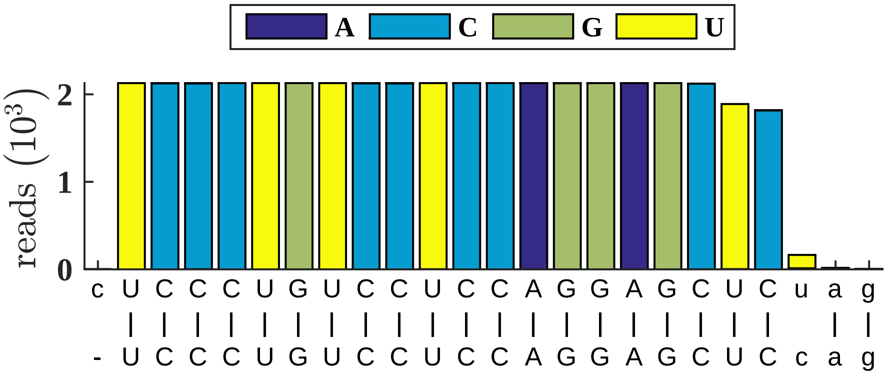

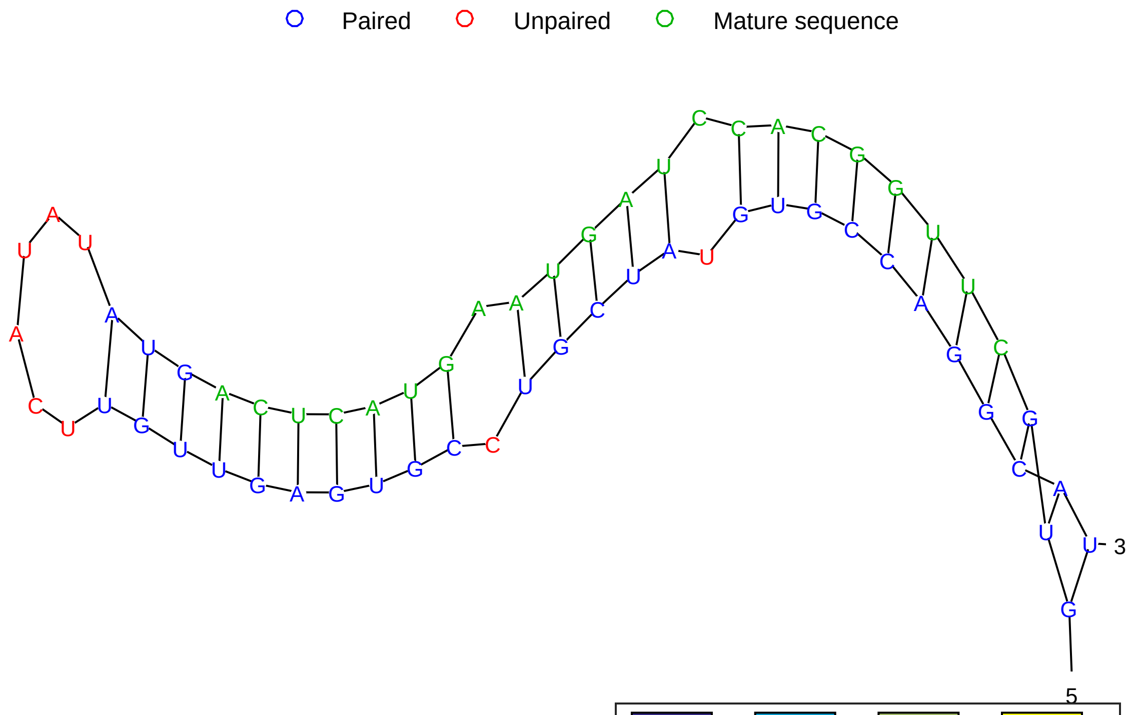

miRNA: bta-miR-1271
Stem loop (UMD3.1): chr7:39194959-39195020
Mature (UMD3.1): chr7:39194962-39194983
Mature seq len: 22
Total raw counts (9 samples): 504
Average raw counts: 56
Strand: Forward
Orientation: 5p
Minimum free energy: -35.10

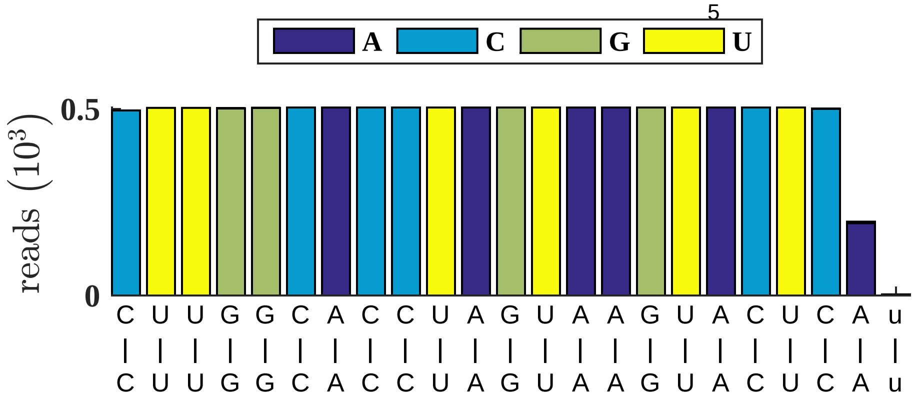

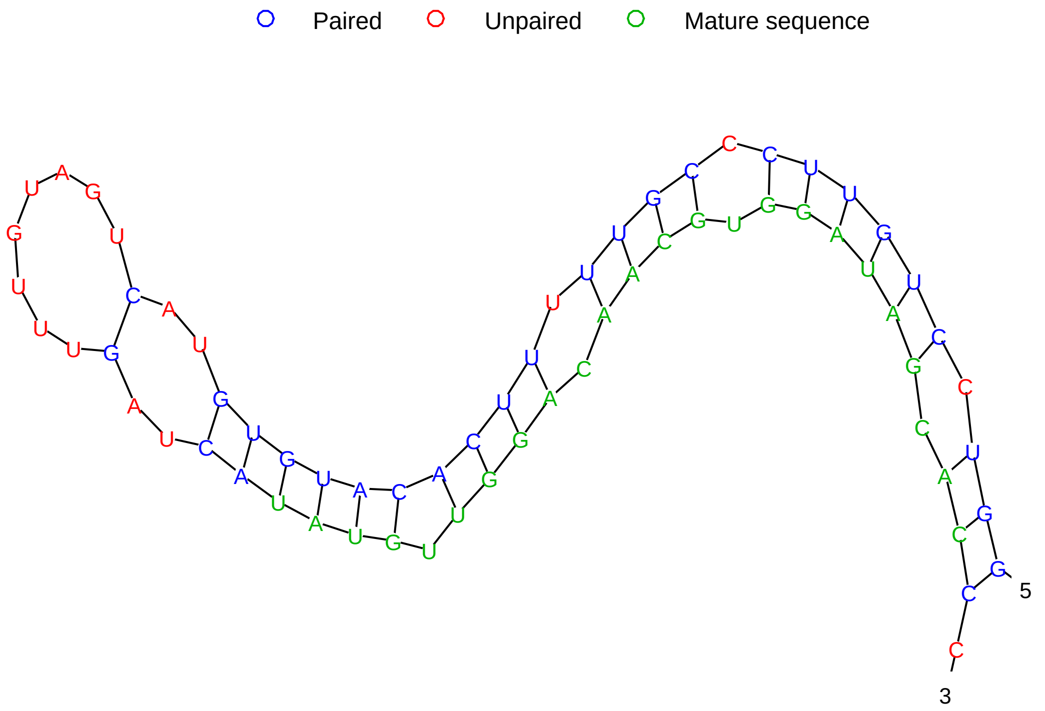

miRNA: bta-miR-6120-3p
Stem loop (UMD3.1): chr7:45882303-45882370
Mature (UMD3.1): chr7:45882345-45882368
Mature seq len: 24
Total raw counts (9 samples): 852
Average raw counts: 95
Strand: Forward
Orientation: 3p
Minimum free energy: -21.90

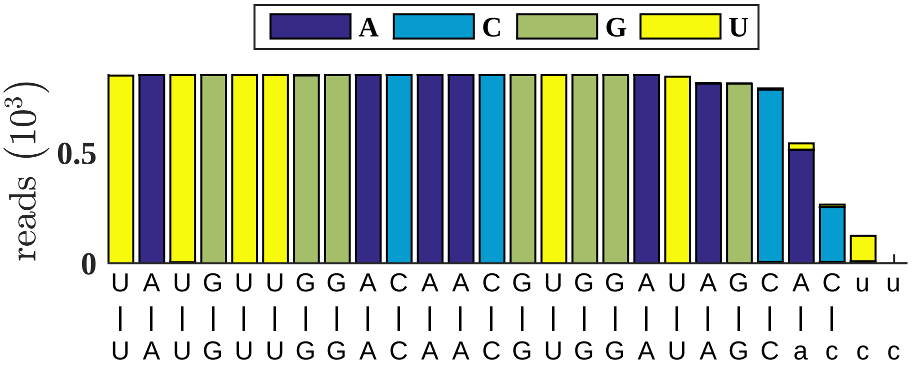

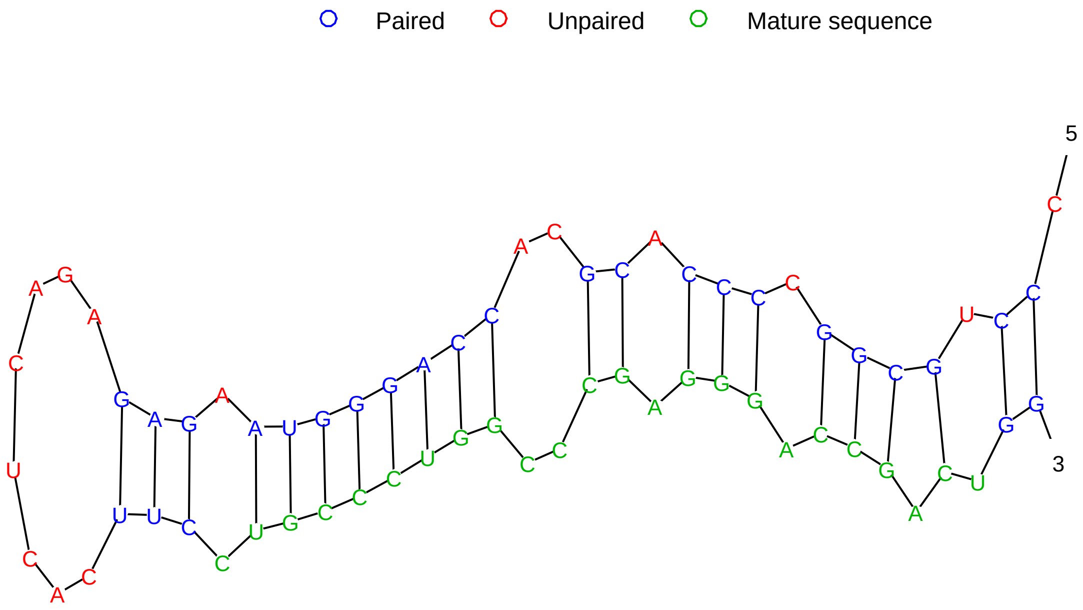

miRNA: bta-miR-874
Stem loop (UMD3.1): chr7:50687430-50687495
Mature (UMD3.1): chr7:50687432-50687455
Mature seq len: 24
Total raw counts (9 samples): 757
Average raw counts: 85
Strand: Reverse
Orientation: 3p
Minimum free energy: -29.50

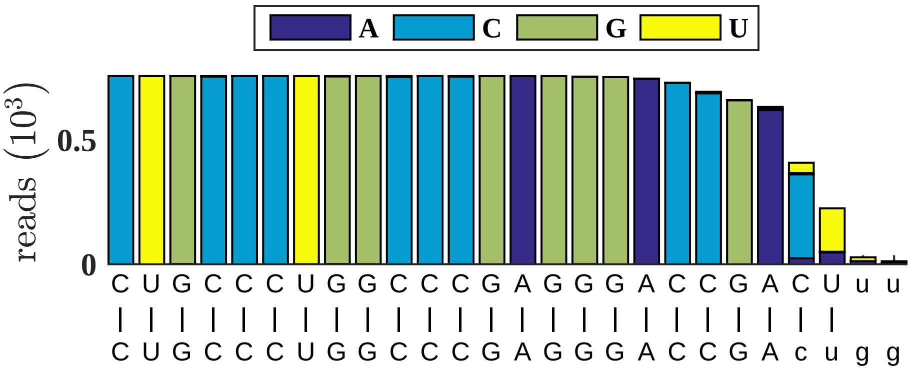

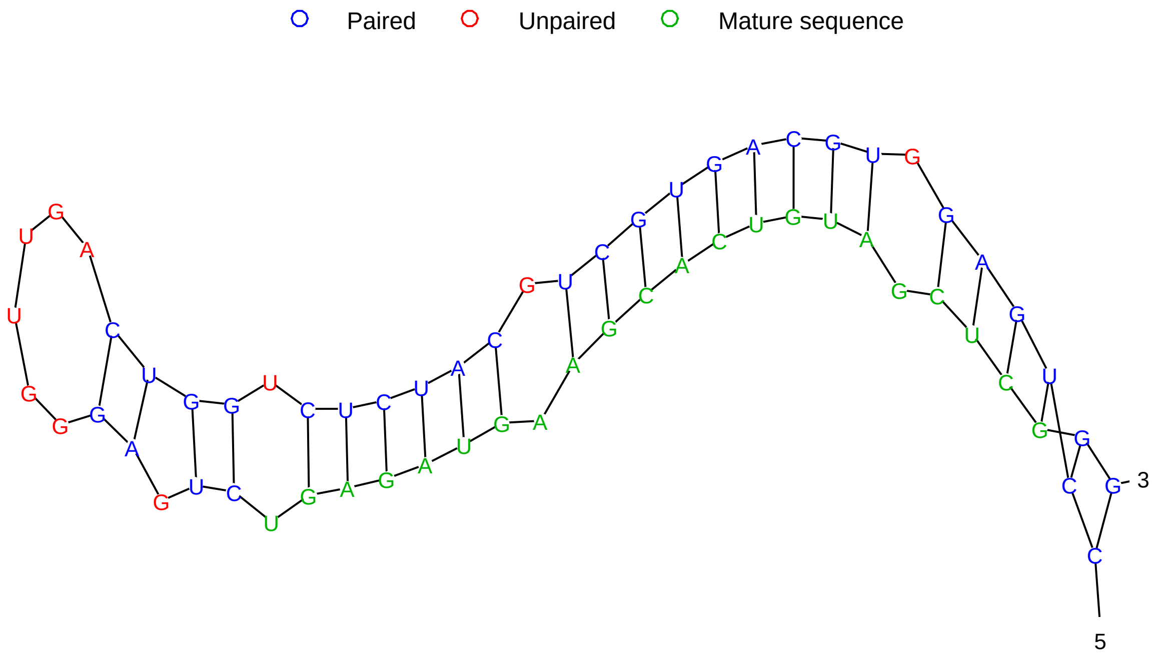

miRNA: bta-miR-143
Stem loop (UMD3.1): chr7:62809320-62809382
Mature (UMD3.1): chr7:62809359-62809380
Mature seq len: 22
Total raw counts (9 samples): 153241
Average raw counts: 17027
Strand: Forward
Orientation: 3p
Minimum free energy: -36.20

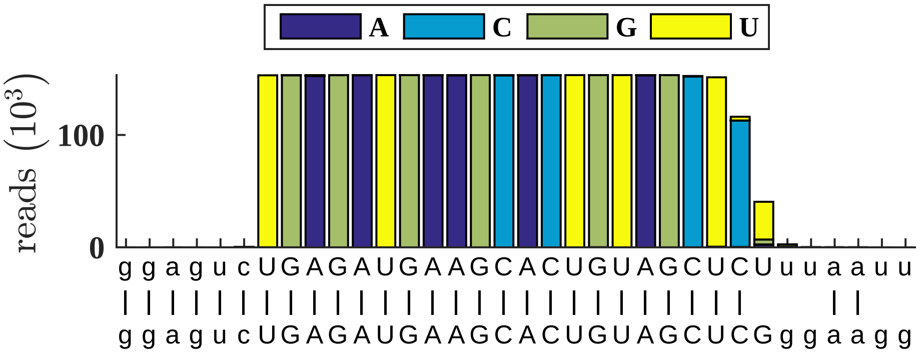

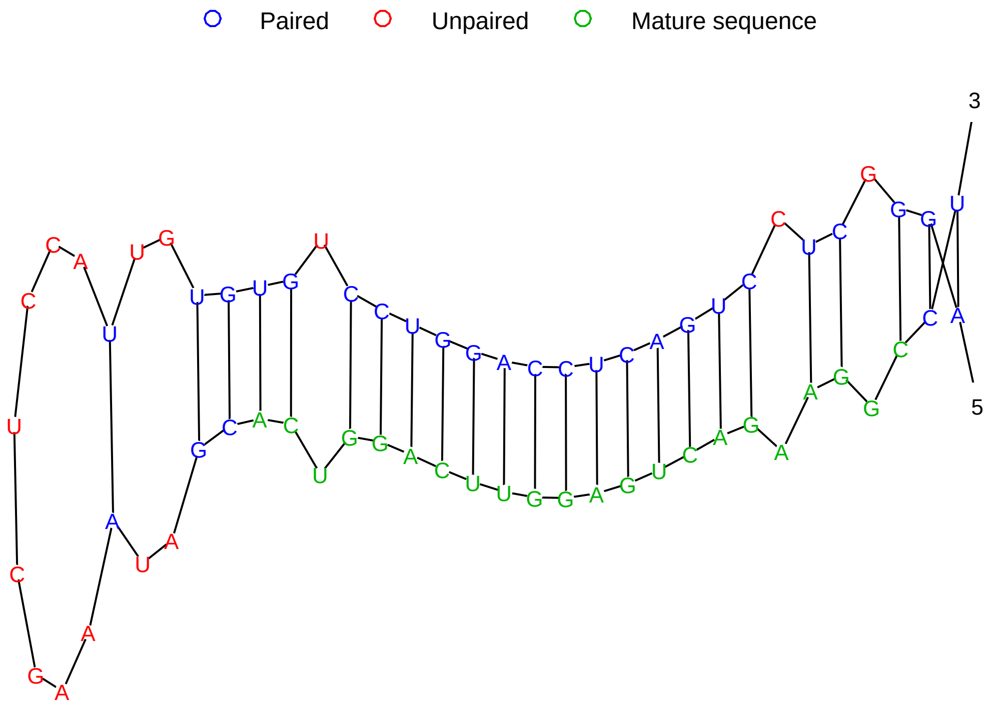

miRNA: bta-miR-378
Stem loop (UMD3.1): chr7:63067301-63067366
Mature (UMD3.1): chr7:63067343-63067364
Mature seq len: 22
Total raw counts (9 samples): 44922
Average raw counts: 4992
Strand: Forward
Orientation: 3p
Minimum free energy: -38.50

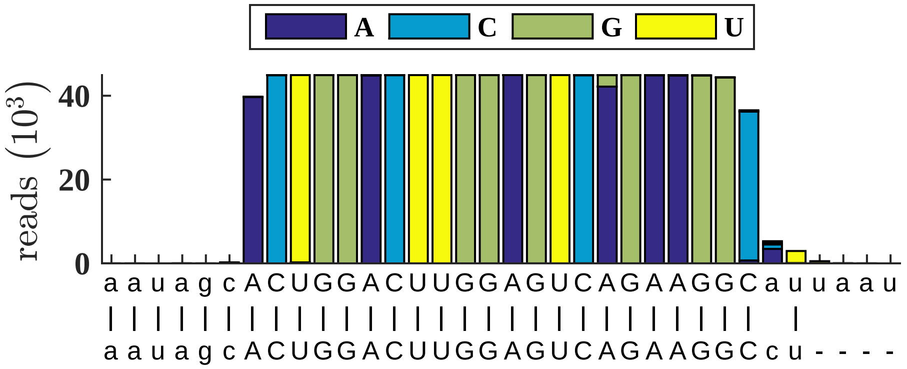

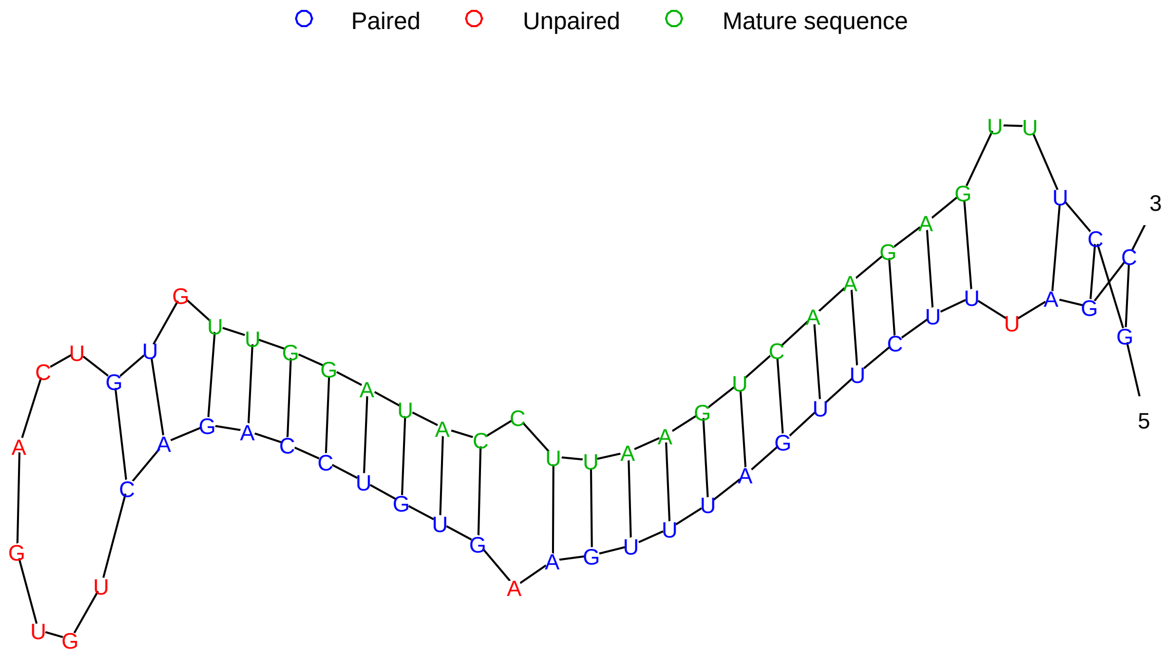

miRNA: bta-miR-146a
Stem loop (UMD3.1): chr7:74185051-74185113
Mature (UMD3.1): chr7:74185054-74185076
Mature seq len: 23
Total raw counts (9 samples): 708
Average raw counts: 79
Strand: Forward
Orientation: 5p
Minimum free energy: -25.40

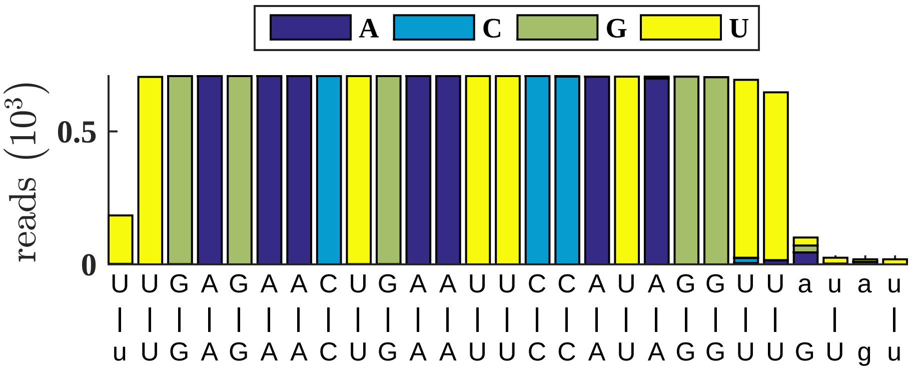

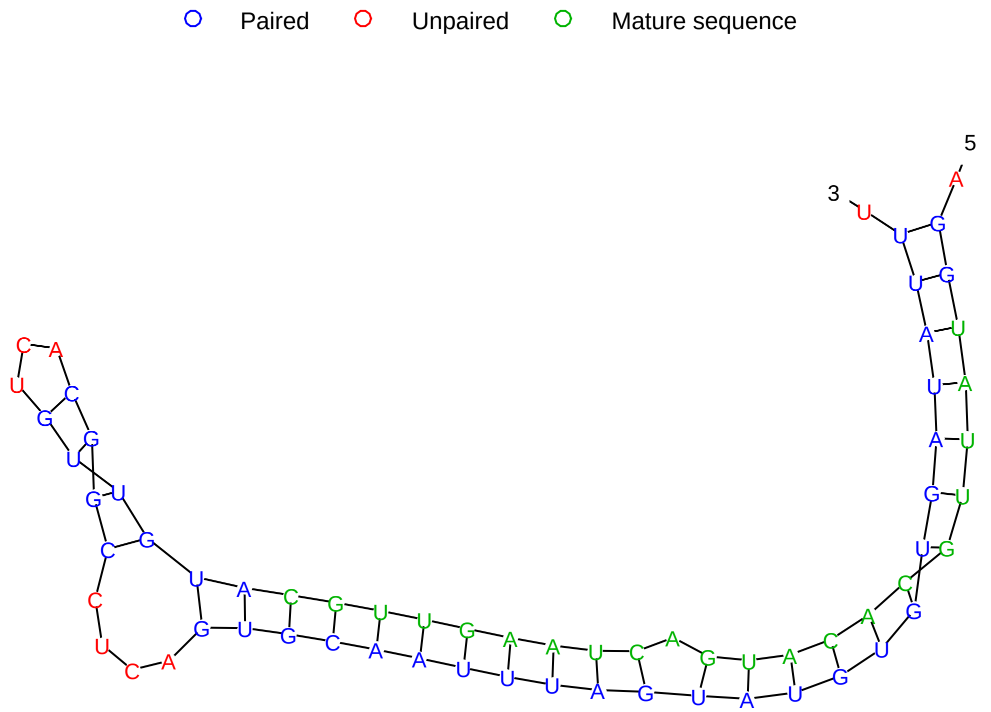

miRNA: bta-miR-32
Stem loop (UMD3.1): chr8:100305162-100305227
Mature (UMD3.1): chr8:100305204-100305224
Mature seq len: 21
Total raw counts (9 samples): 3993
Average raw counts: 444
Strand: Reverse
Orientation: 5p
Minimum free energy: -26.30

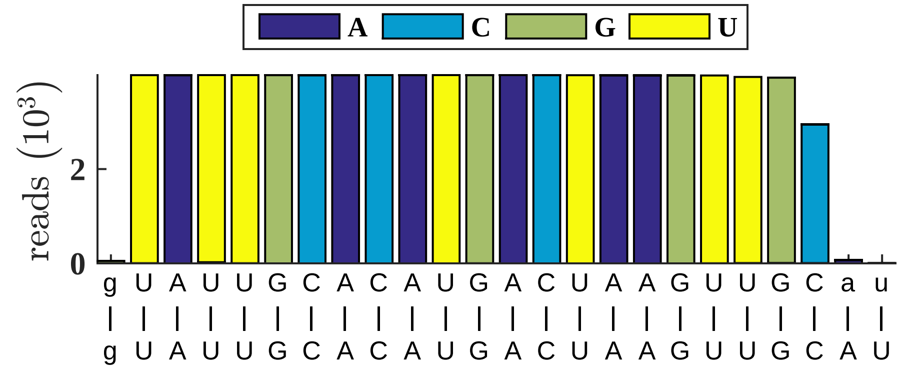

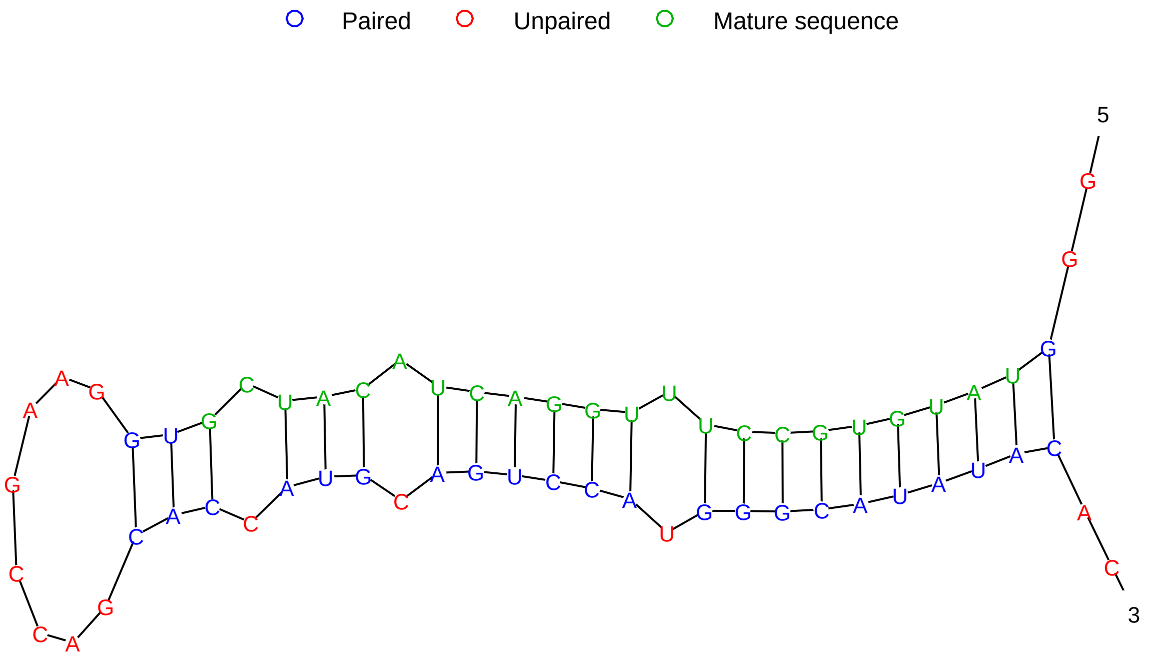

miRNA: bta-miR-455-5p
Stem loop (UMD3.1): chr8:105147059-105147120
Mature (UMD3.1): chr8:105147062-105147083
Mature seq len: 22
Total raw counts (9 samples): 611
Average raw counts: 68
Strand: Forward
Orientation: 5p
Minimum free energy: -31.60

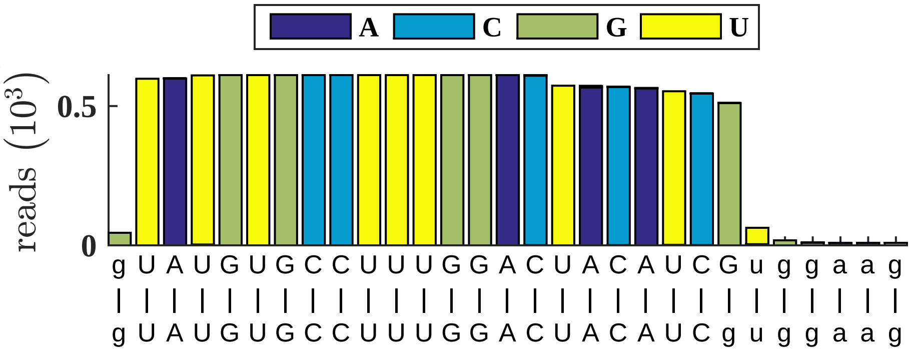

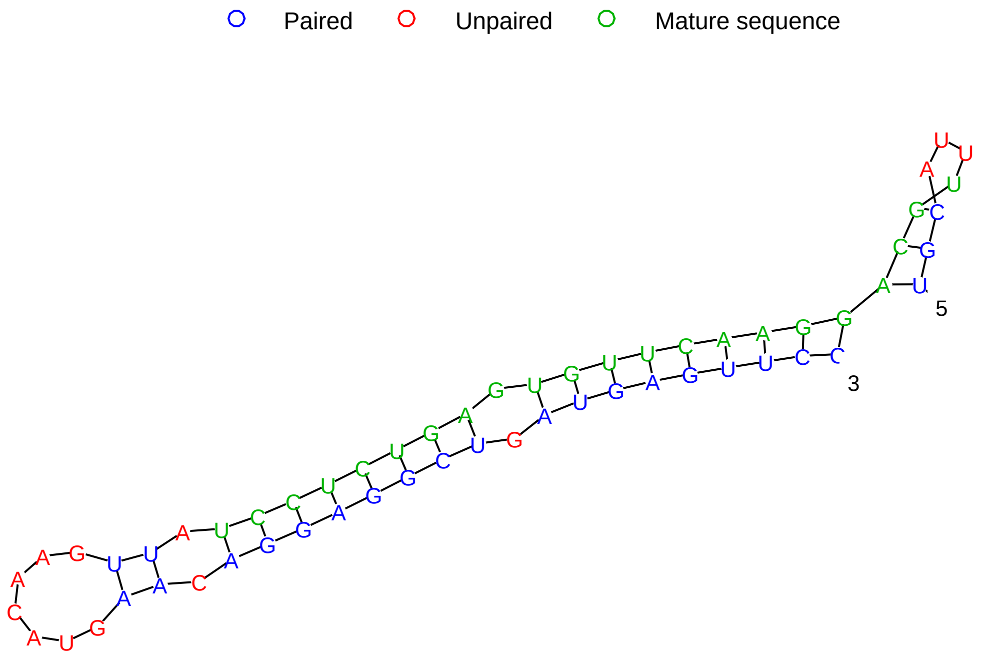

miRNA: bta-miR-873
Stem loop (UMD3.1): chr8:15159563-15159621
Mature (UMD3.1): chr8:15159569-15159590
Mature seq len: 22
Total raw counts (9 samples): 13303
Average raw counts: 1479
Strand: Forward
Orientation: 5p
Minimum free energy: -26.40

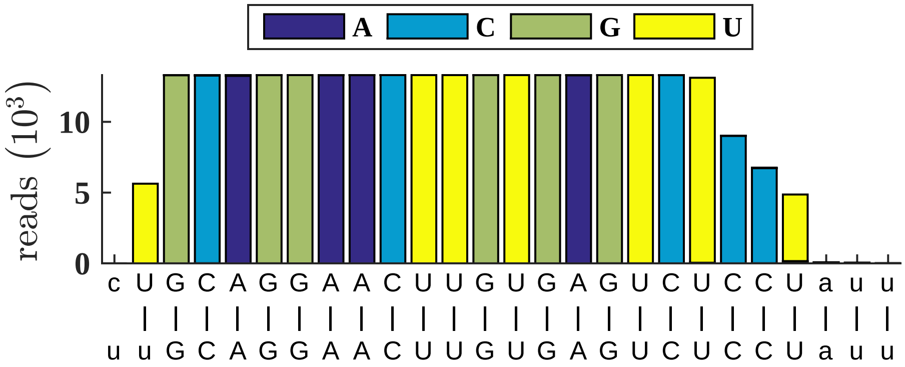

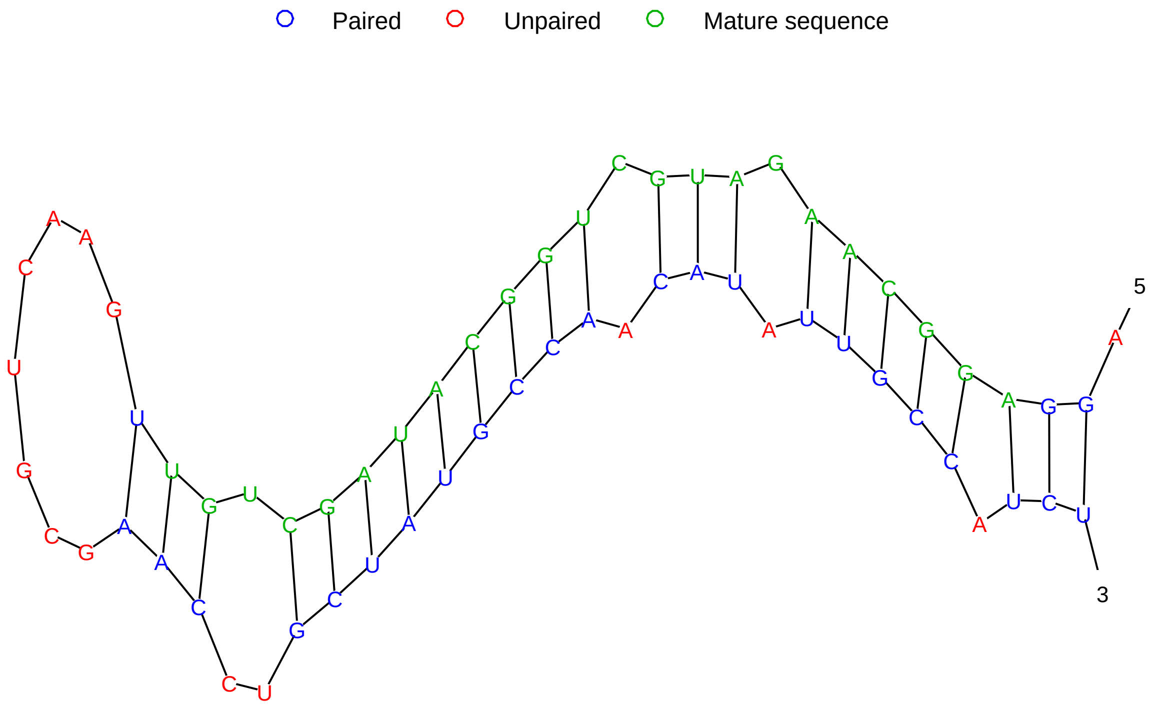

miRNA: bta-miR-31
Stem loop (UMD3.1): chr8:22534818-22534880
Mature (UMD3.1): chr8:22534821-22534843
Mature seq len: 23
Total raw counts (9 samples): 100965
Average raw counts: 11219
Strand: Forward
Orientation: 5p
Minimum free energy: -28.80

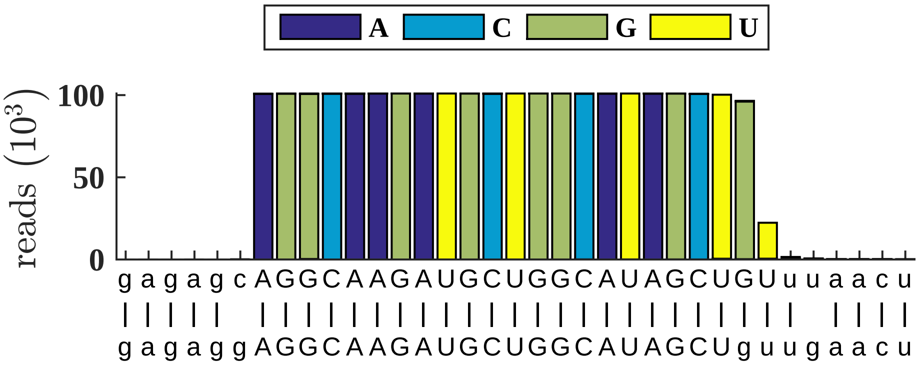

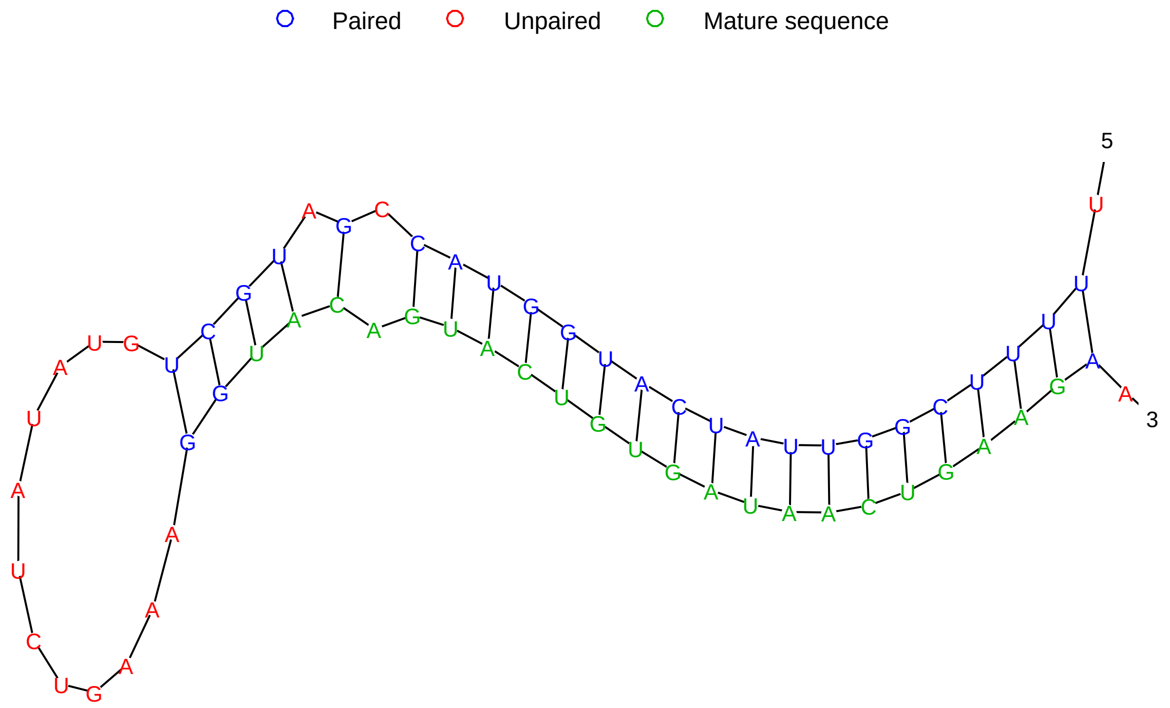

miRNA: bta-miR-101
Stem loop (UMD3.1): chr8:39940839-39940903
Mature (UMD3.1): chr8:39940841-39940862
Mature seq len: 22
Total raw counts (9 samples): 19227
Average raw counts: 2137
Strand: Reverse
Orientation: 3p
Minimum free energy: -25.80

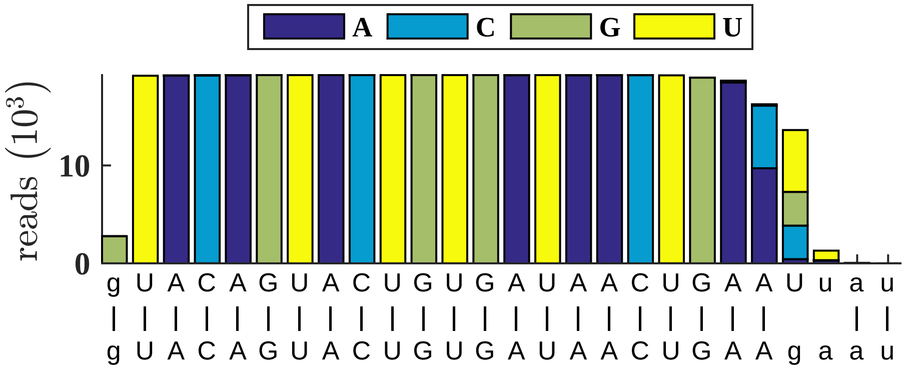

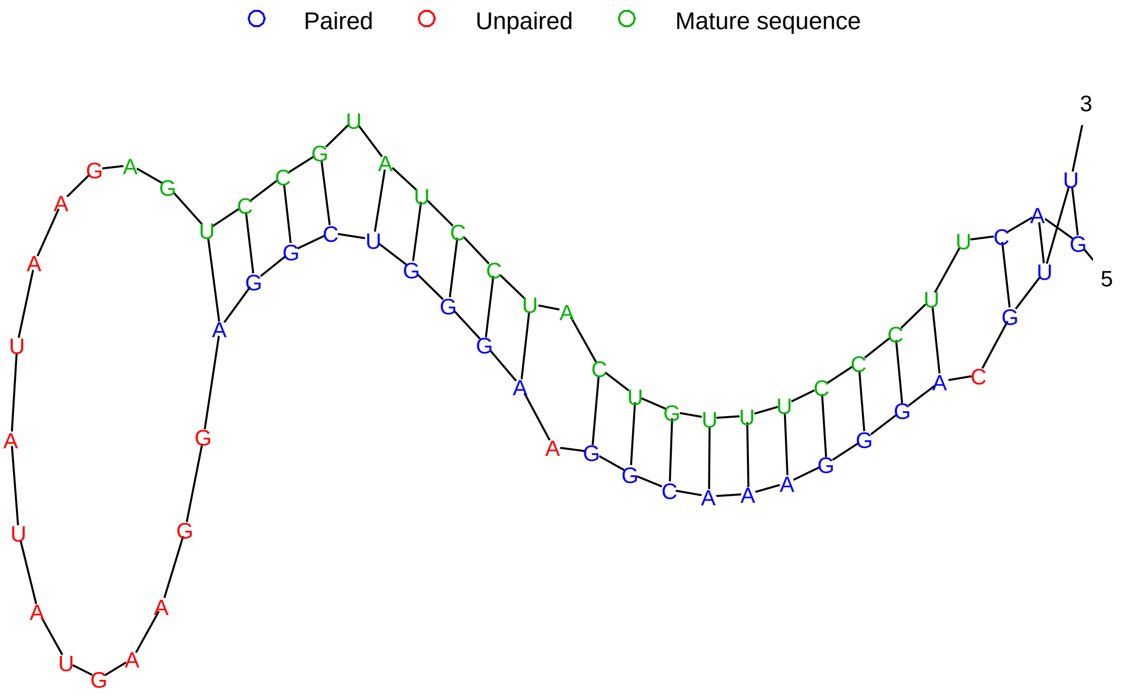

miRNA: bta-miR-204
Stem loop (UMD3.1): chr8:47259552-47259615
Mature (UMD3.1): chr8:47259589-47259612
Mature seq len: 24
Total raw counts (9 samples): 8951
Average raw counts: 995
Strand: Reverse
Orientation: 5p
Minimum free energy: -30.10

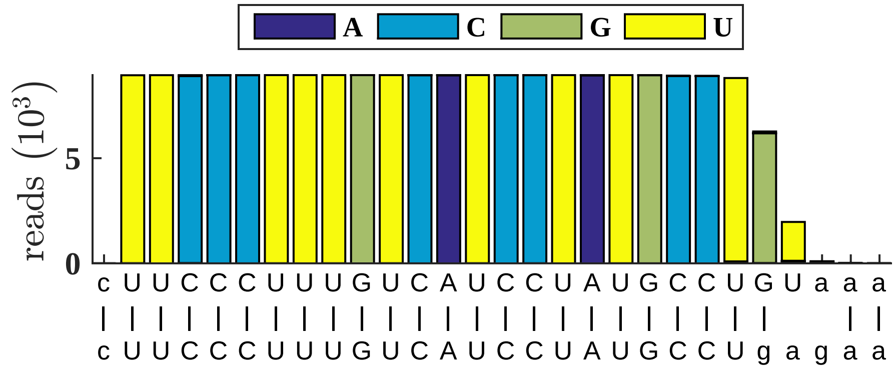

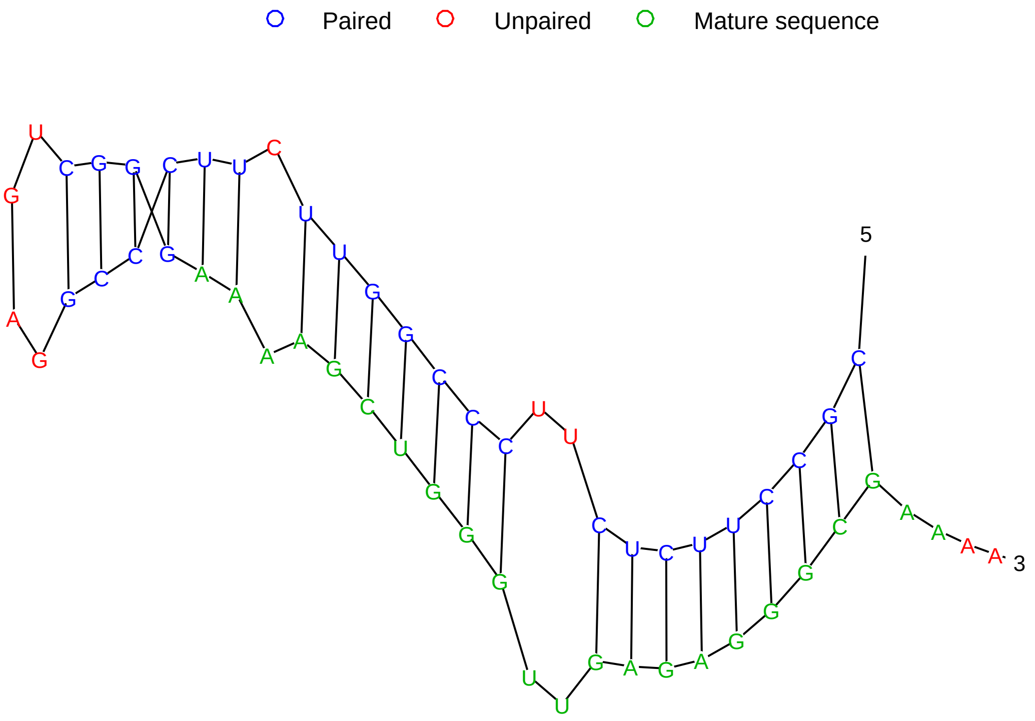

miRNA: bta-miR-320a
Stem loop (UMD3.1): chr8:70060386-70060443
Mature (UMD3.1): chr8:70060388-70060410
Mature seq len: 23
Total raw counts (9 samples): 59285
Average raw counts: 6588
Strand: Reverse
Orientation: 3p
Minimum free energy: -39.90

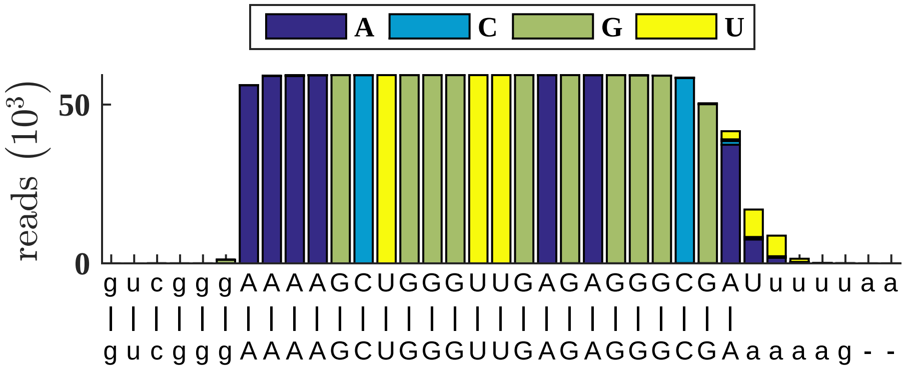

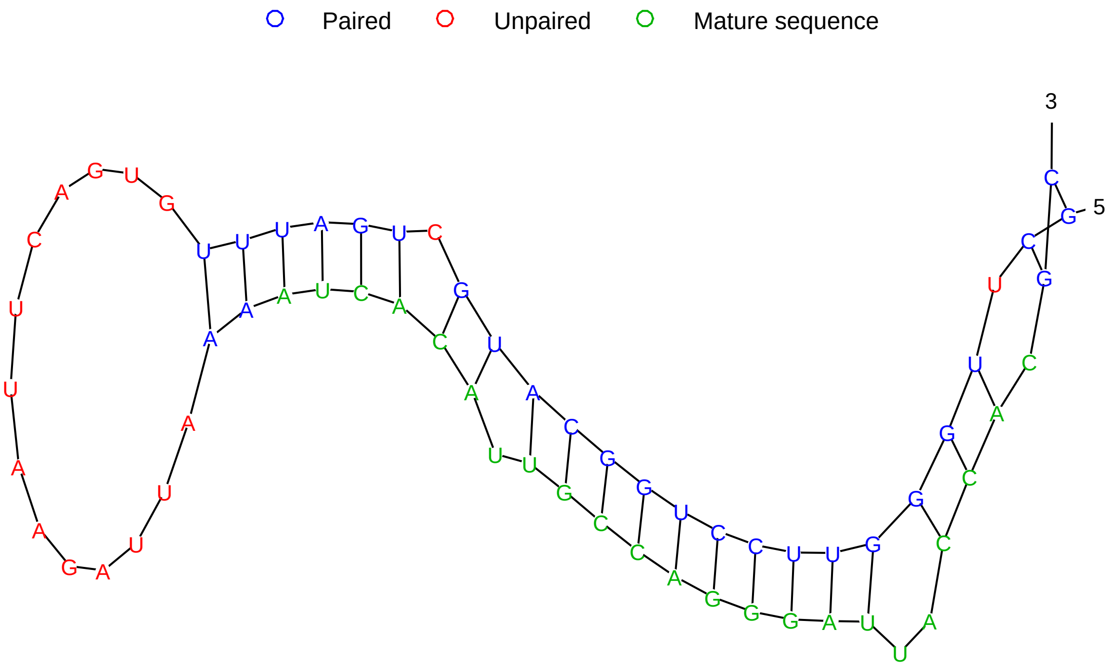

miRNA: bta-miR-23b-3p
Stem loop (UMD3.1): chr8:83009611-83009676
Mature (UMD3.1): chr8:83009652-83009674
Mature seq len: 23
Total raw counts (9 samples): 29350
Average raw counts: 3262
Strand: Forward
Orientation: 3p
Minimum free energy: -24.60

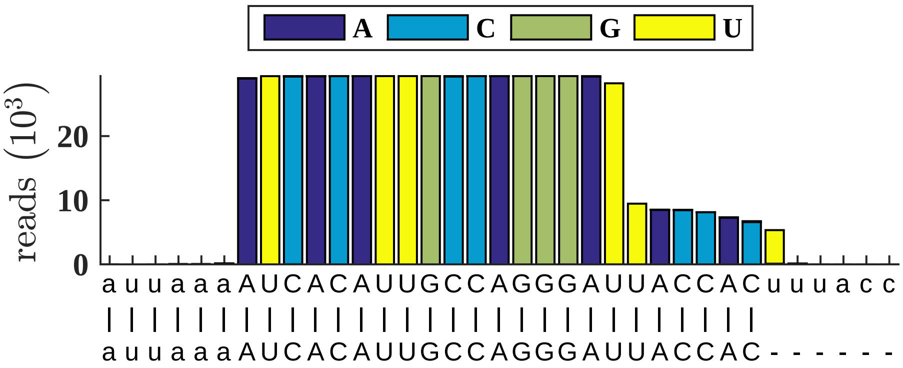

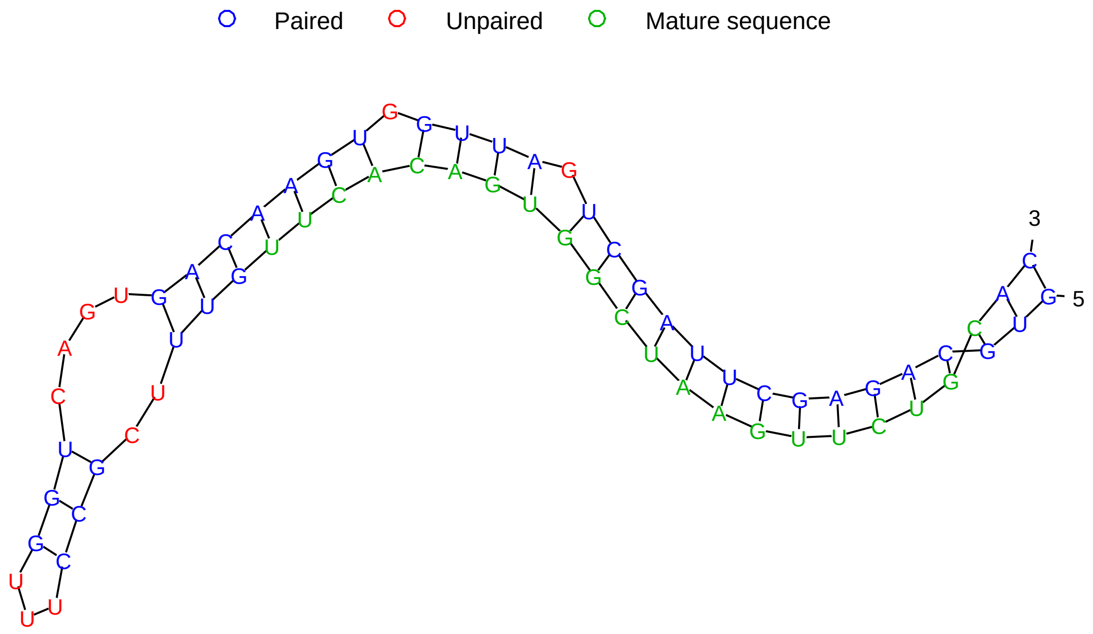

miRNA: bta-miR-27b
Stem loop (UMD3.1): chr8:83009837-83009905
Mature (UMD3.1): chr8:83009883-83009903
Mature seq len: 21
Total raw counts (9 samples): 794347
Average raw counts: 88261
Strand: Forward
Orientation: 3p
Minimum free energy: -34.60

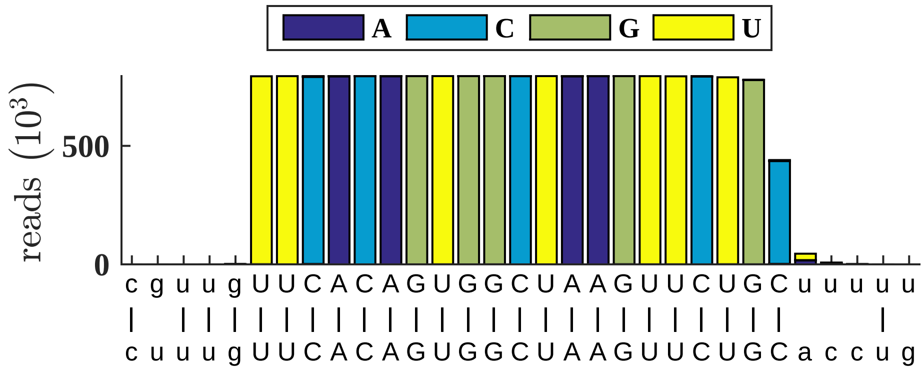

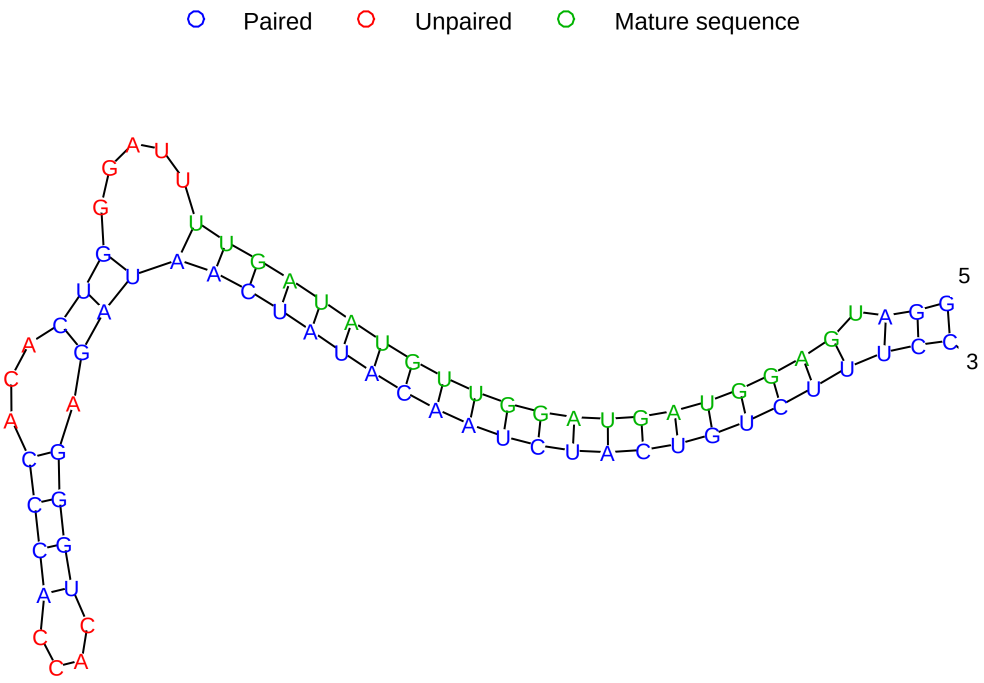

miRNA: bta-let-7a-5p
Stem loop (UMD3.1): chr8:86884874-86884949
Mature (UMD3.1): chr8:86884877-86884898
Mature seq len: 22
Total raw counts (9 samples): 77298
Average raw counts: 8589
Strand: Forward
Orientation: 5p
Minimum free energy: -31.60

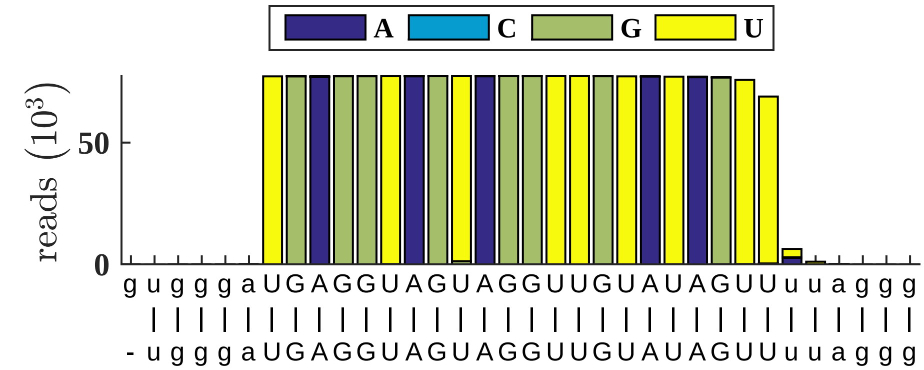

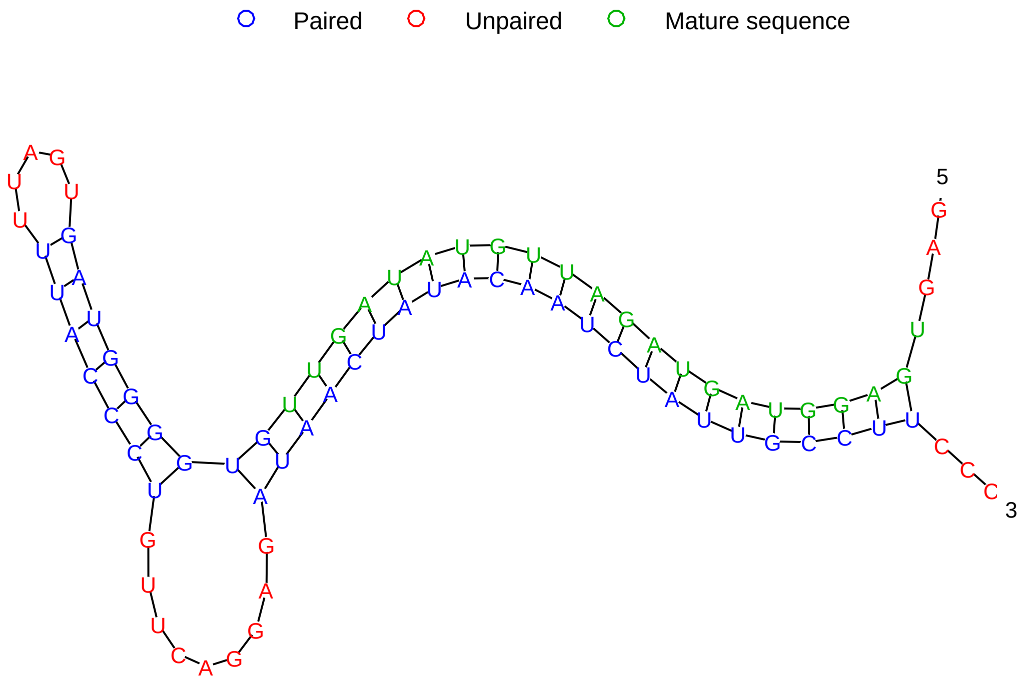

miRNA: bta-let-7f
Stem loop (UMD3.1): chr8:86885228-86885308
Mature (UMD3.1): chr8:86885231-86885252
Mature seq len: 22
Total raw counts (9 samples): 254119
Average raw counts: 28236
Strand: Forward
Orientation: 5p
Minimum free energy: -37.90

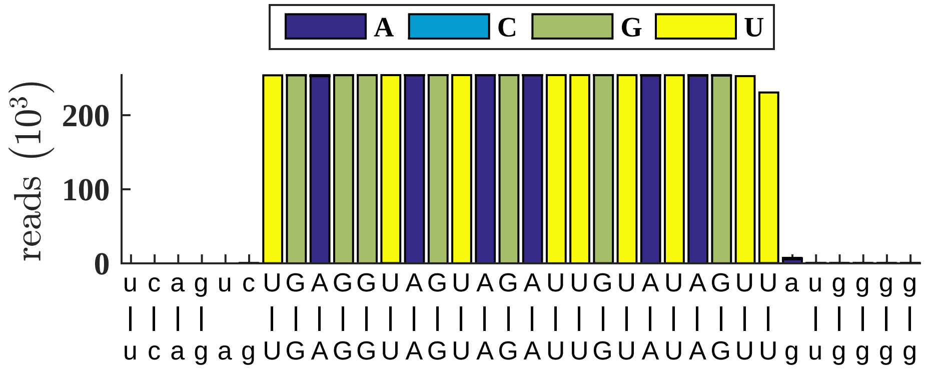

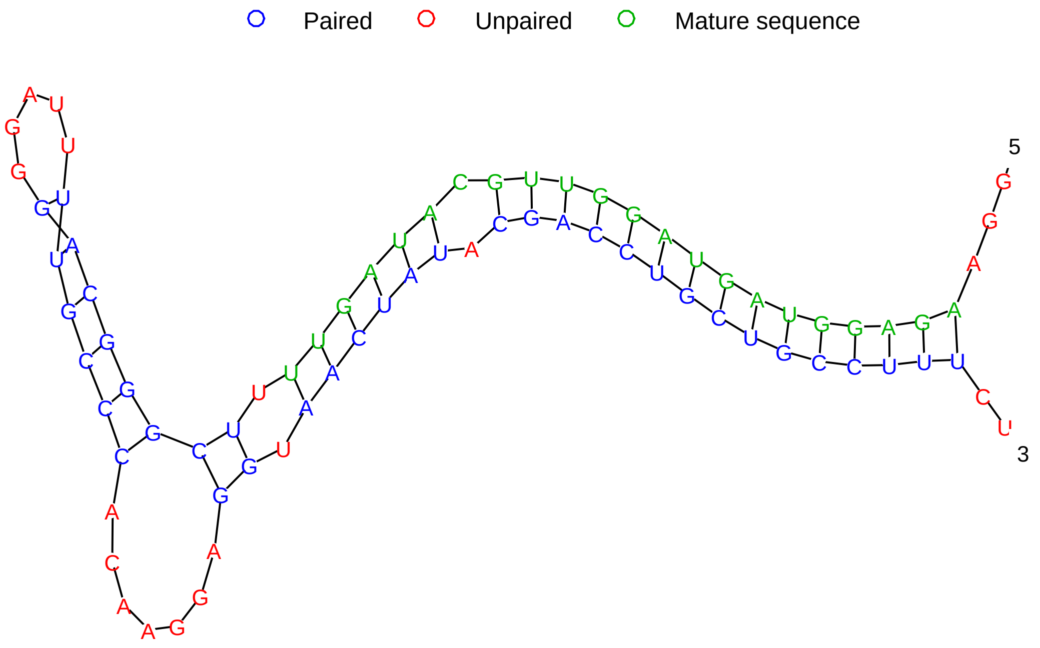

miRNA: bta-let-7d
Stem loop (UMD3.1): chr8:86887435-86887513
Mature (UMD3.1): chr8:86887438-86887459
Mature seq len: 22
Total raw counts (9 samples): 17658
Average raw counts: 1962
Strand: Forward
Orientation: 5p
Minimum free energy: -36.10

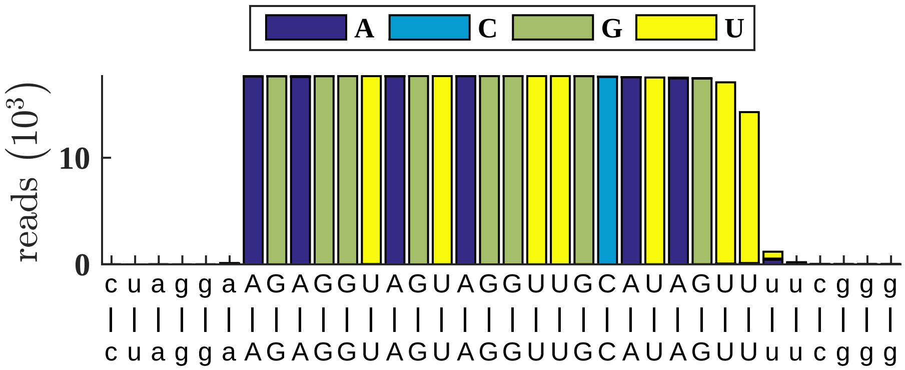

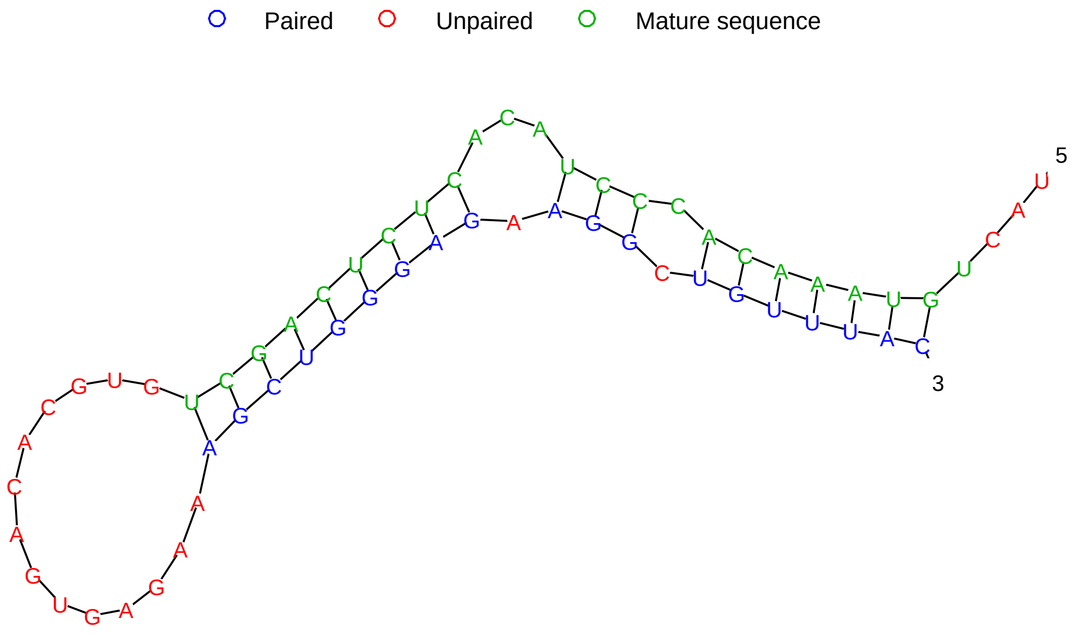

miRNA: bta-miR-30f
Stem loop (UMD3.1): chr9:10730941-10731002
Mature (UMD3.1): chr9:10730976-10730999
Mature seq len: 24
Total raw counts (9 samples): 2859
Average raw counts: 318
Strand: Reverse
Orientation: 5p
Minimum free energy: -23.20

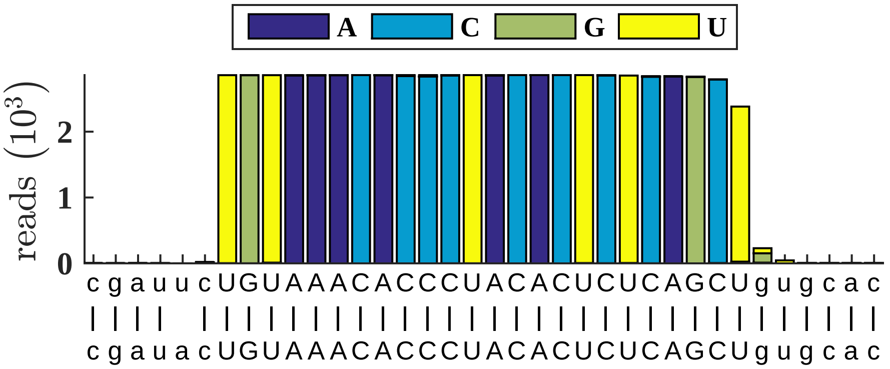

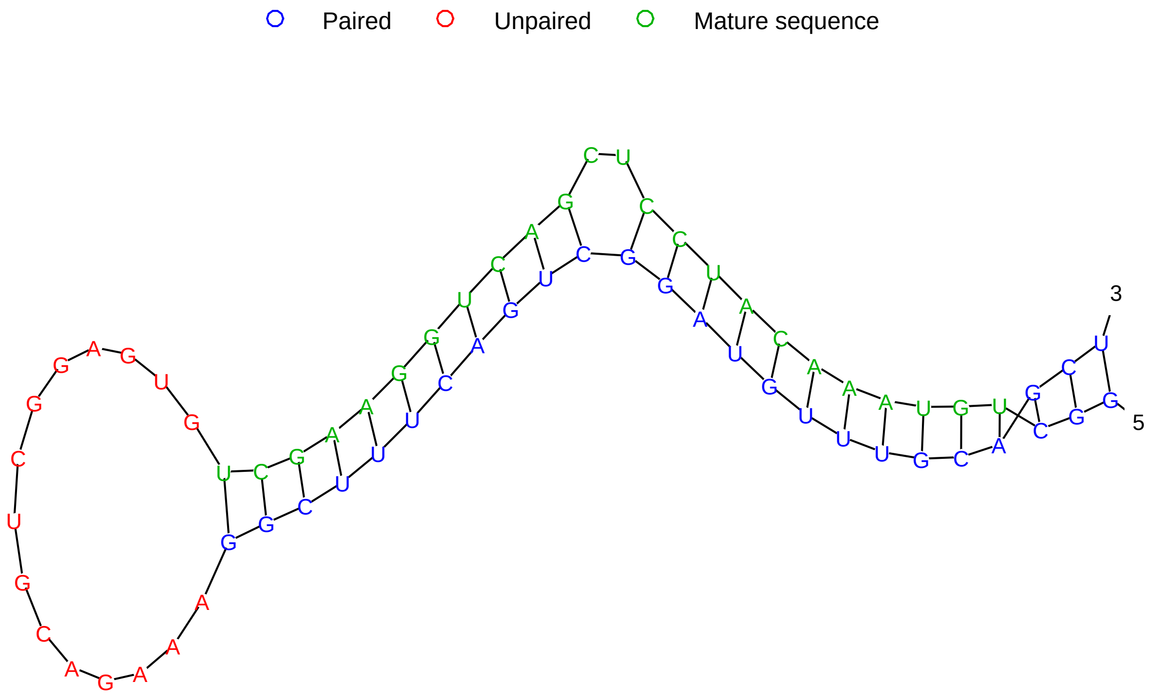

miRNA: bta-miR-30a-5p
Stem loop (UMD3.1): chr9:10768283-10768349
Mature (UMD3.1): chr9:10768323-10768346
Mature seq len: 24
Total raw counts (9 samples): 192160
Average raw counts: 21352
Strand: Reverse
Orientation: 5p
Minimum free energy: -36.90

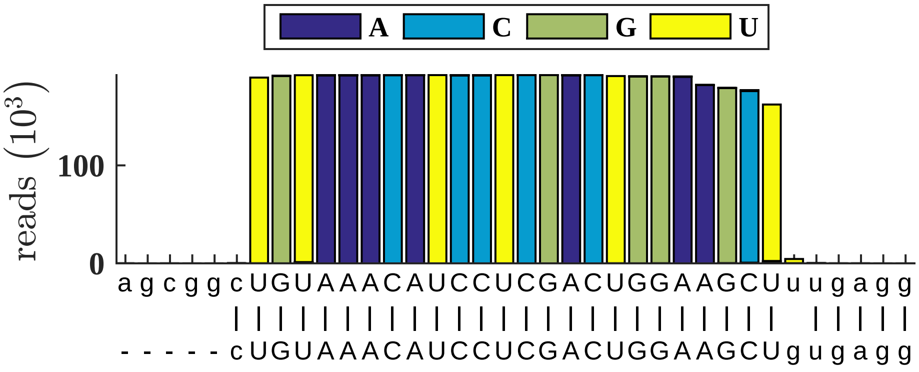

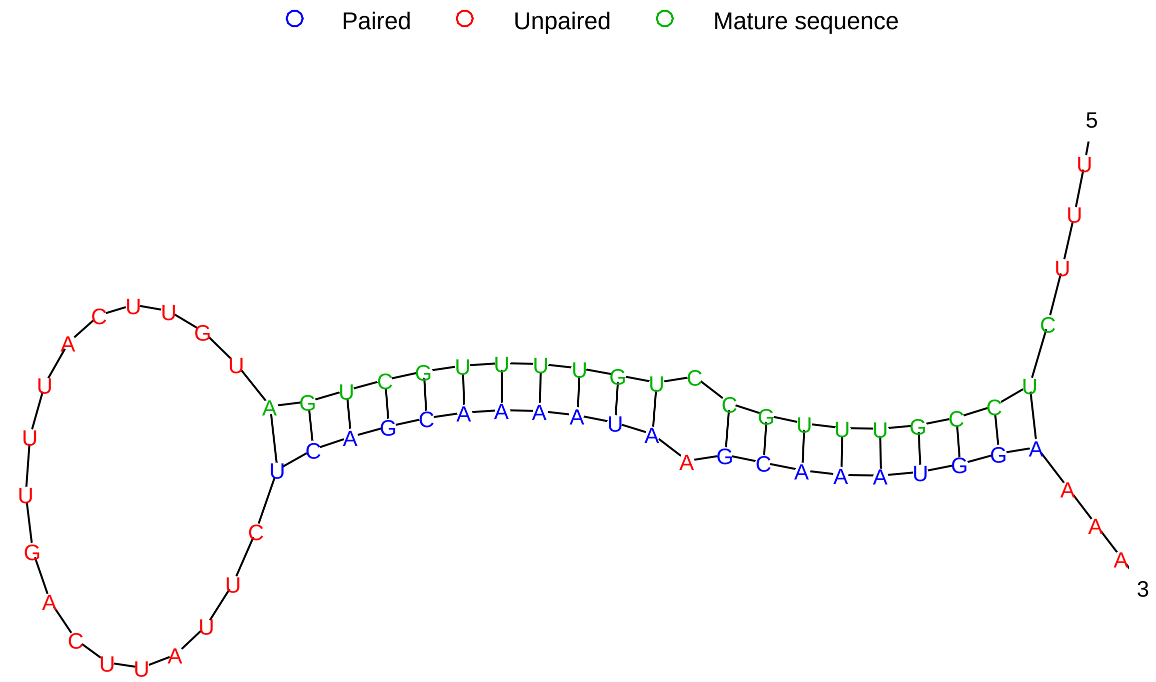

miRNA: bta-miR-1468
Stem loop (UMD3.1): chrX:101844107-101844173
Mature (UMD3.1): chrX:101844110-101844131
Mature seq len: 22
Total raw counts (9 samples): 6020
Average raw counts: 669
Strand: Forward
Orientation: 5p
Minimum free energy: -25.50

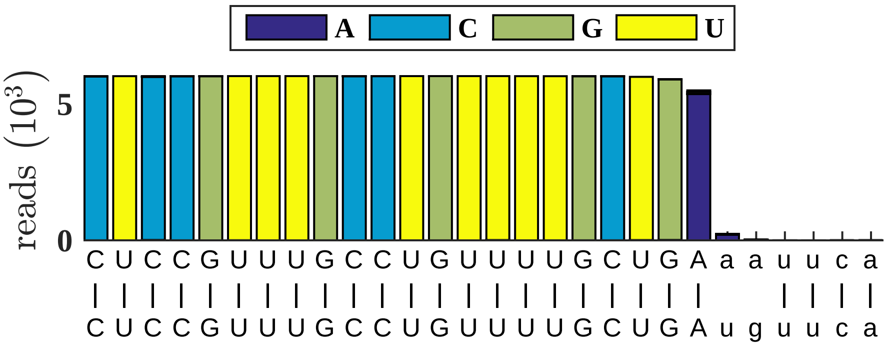

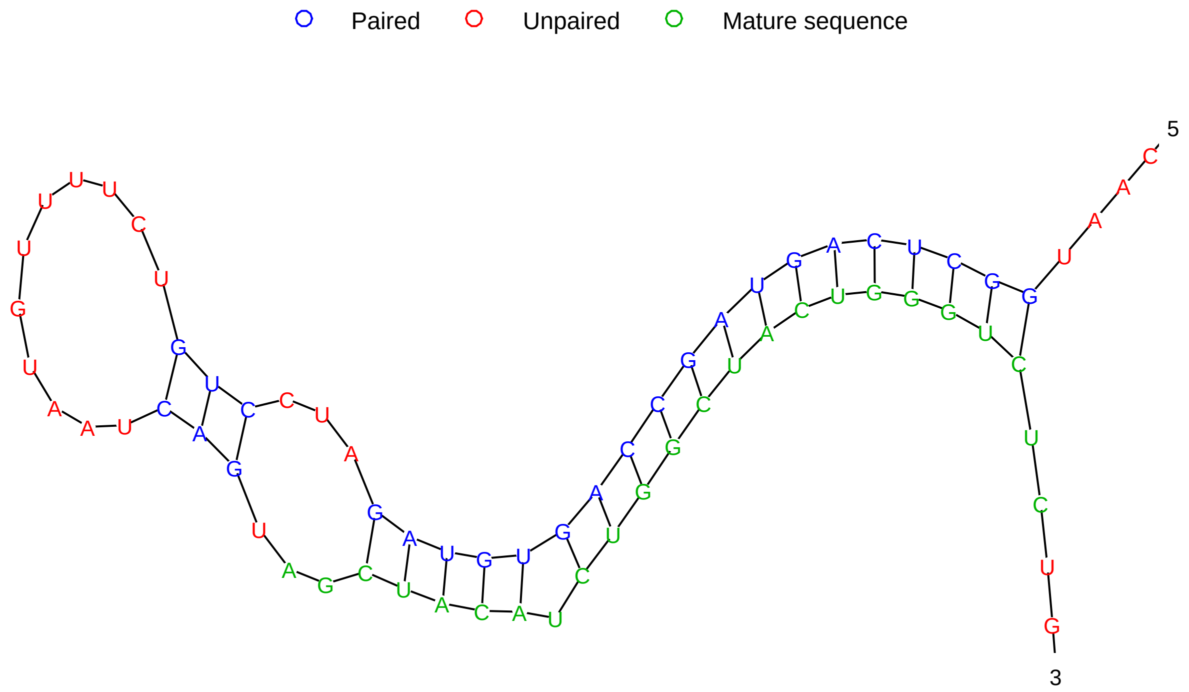

miRNA: bta-miR-222
Stem loop (UMD3.1): chrX:103538165-103538234
Mature (UMD3.1): chrX:103538209-103538232
Mature seq len: 24
Total raw counts (9 samples): 2410
Average raw counts: 268
Strand: Forward
Orientation: 3p
Minimum free energy: -33.60

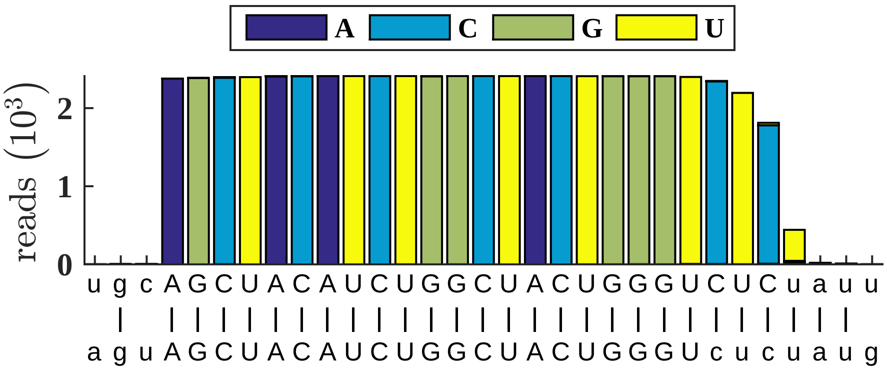

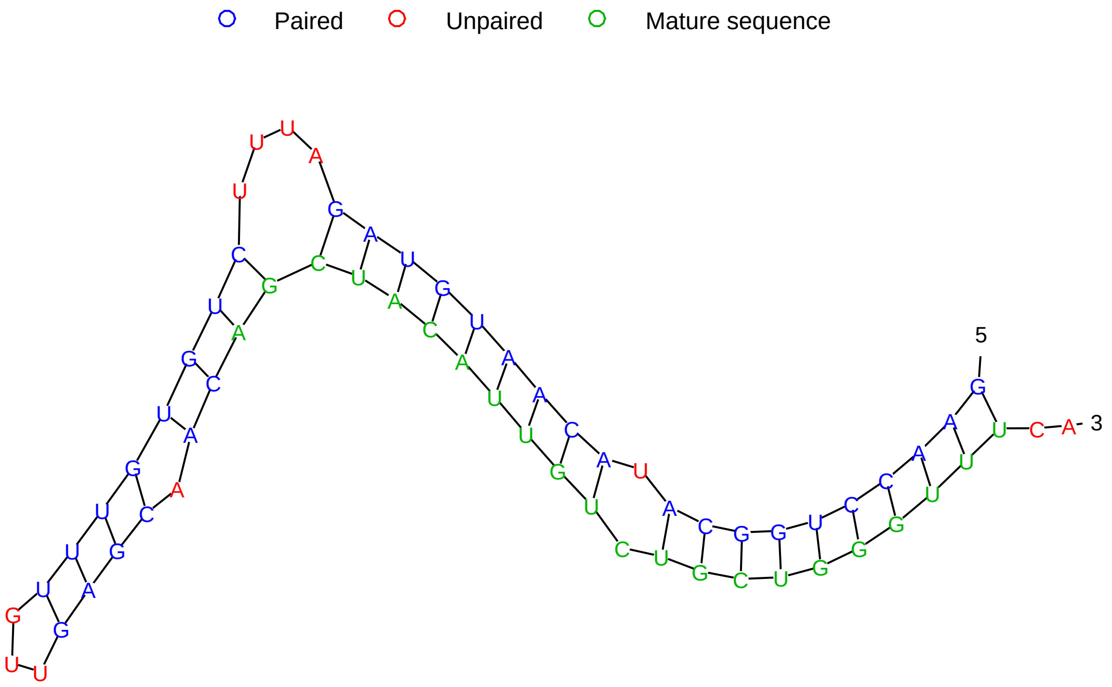

miRNA: bta-miR-221
Stem loop (UMD3.1): chrX:103538857-103538922
Mature (UMD3.1): chrX:103538899-103538920
Mature seq len: 22
Total raw counts (9 samples): 5067
Average raw counts: 563
Strand: Forward
Orientation: 3p
Minimum free energy: -28.40

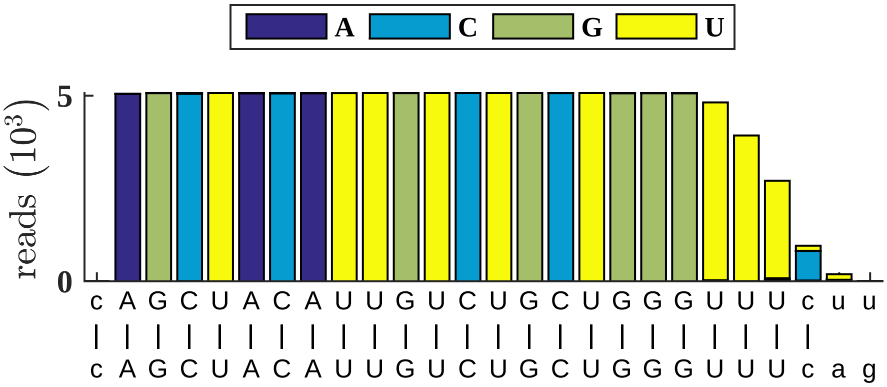

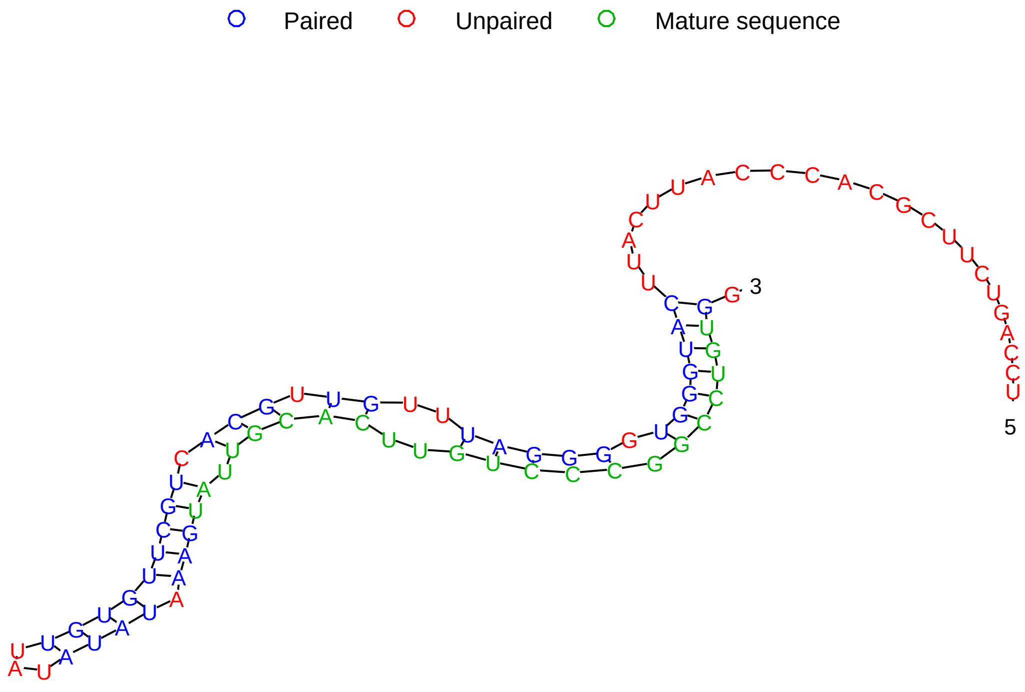

miRNA: bta-miR-92a
Stem loop (UMD3.1): chrX:17917861-17917950
Mature (UMD3.1): chrX:17917863-17917884
Mature seq len: 22
Total raw counts (9 samples): 291632
Average raw counts: 32404
Strand: Reverse
Orientation: 3p
Minimum free energy: -28.90

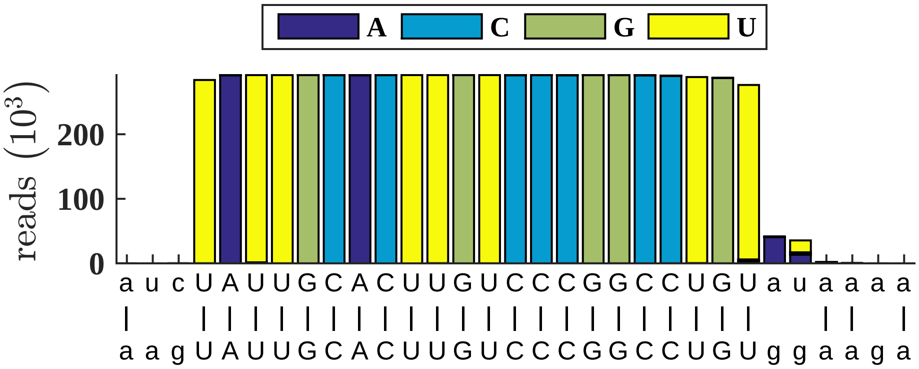

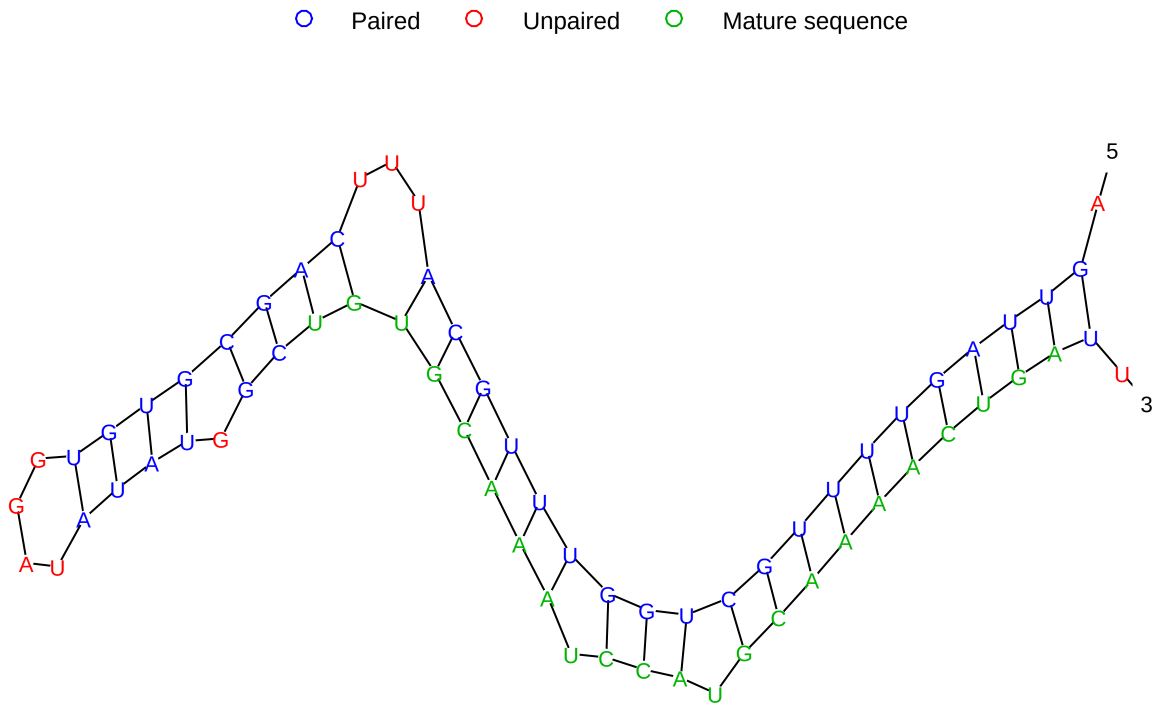

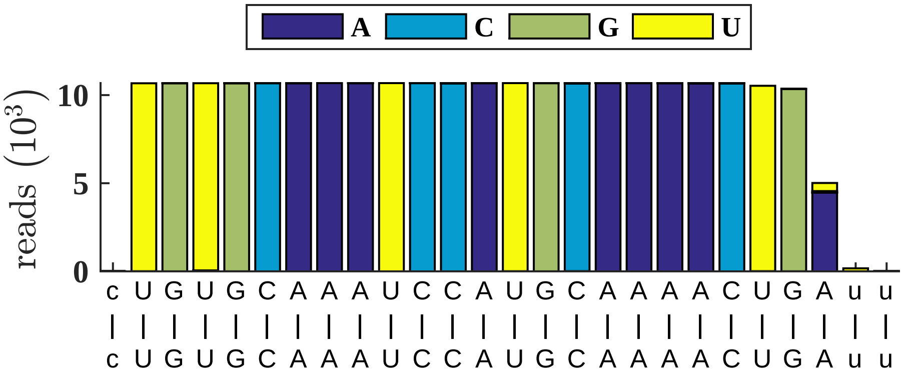

miRNA: bta-miR-19b
Stem loop (UMD3.1): chrX:17918011-17918078
Mature (UMD3.1): chrX:17918013-17918035
Mature seq len: 23
Total raw counts (9 samples): 10680
Average raw counts: 1187
Strand: Reverse
Orientation: 3p
Minimum free energy: -27.90

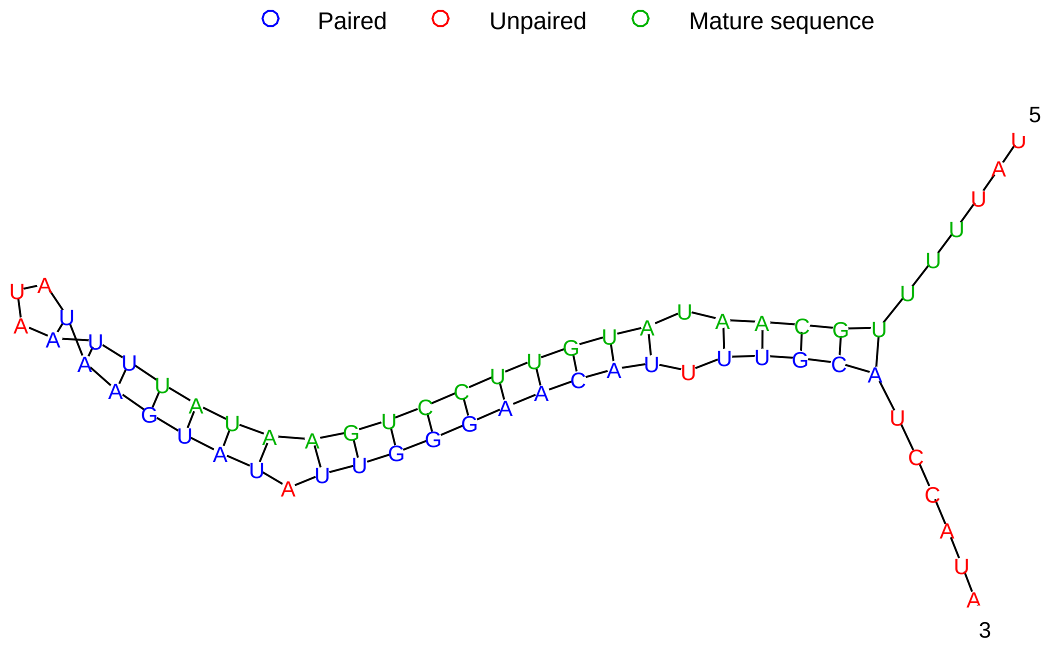

miRNA: bta-miR-450b
Stem loop (UMD3.1): chrX:18178919-18178980
Mature (UMD3.1): chrX:18178955-18178977
Mature seq len: 23
Total raw counts (9 samples): 37441
Average raw counts: 4161
Strand: Reverse
Orientation: 5p
Minimum free energy: -20.20

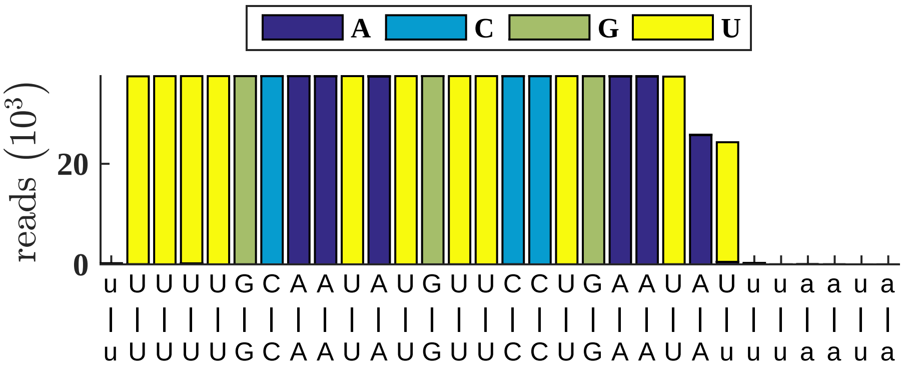

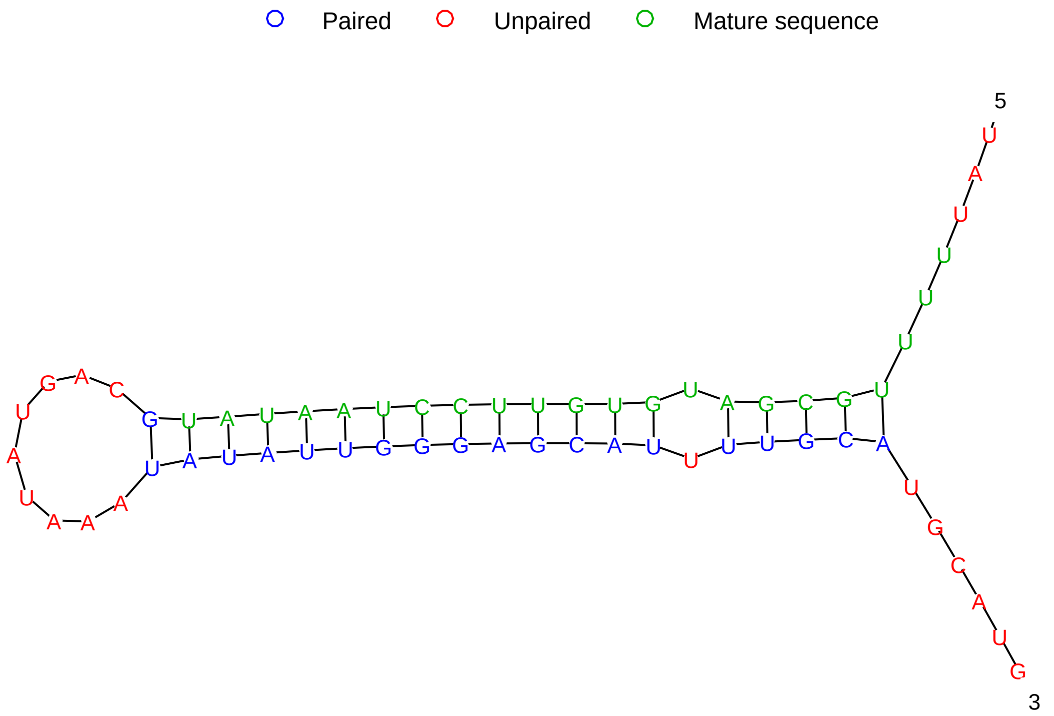

miRNA: bta-miR-450a
Stem loop (UMD3.1): chrX:18179089-18179149
Mature (UMD3.1): chrX:18179125-18179146
Mature seq len: 22
Total raw counts (9 samples): 1264
Average raw counts: 141
Strand: Reverse
Orientation: 5p
Minimum free energy: -21.40

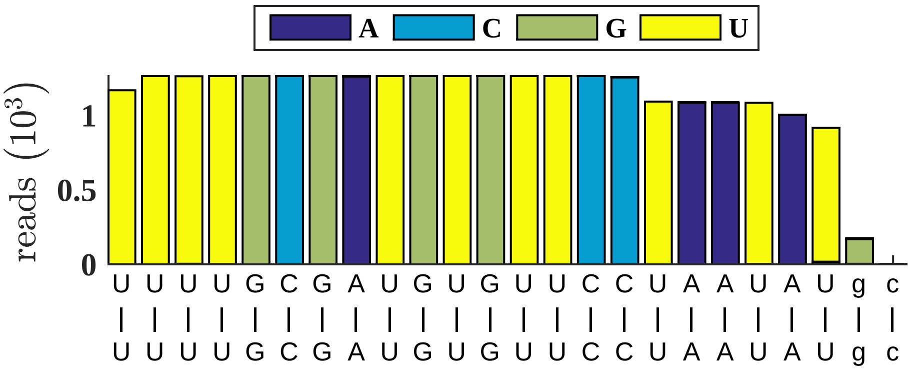

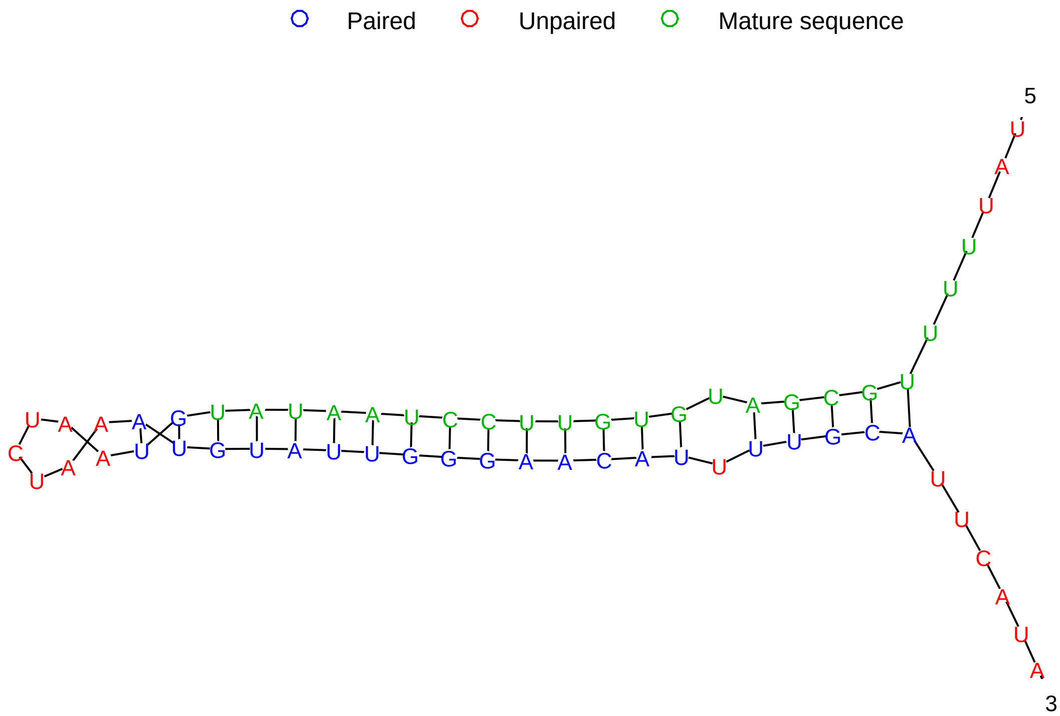

miRNA: bta-miR-450a
Stem loop (UMD3.1): chrX:18179234-18179294
Mature (UMD3.1): chrX:18179270-18179291
Mature seq len: 22
Total raw counts (9 samples): 1310
Average raw counts: 146
Strand: Reverse
Orientation: 5p
Minimum free energy: -22.60

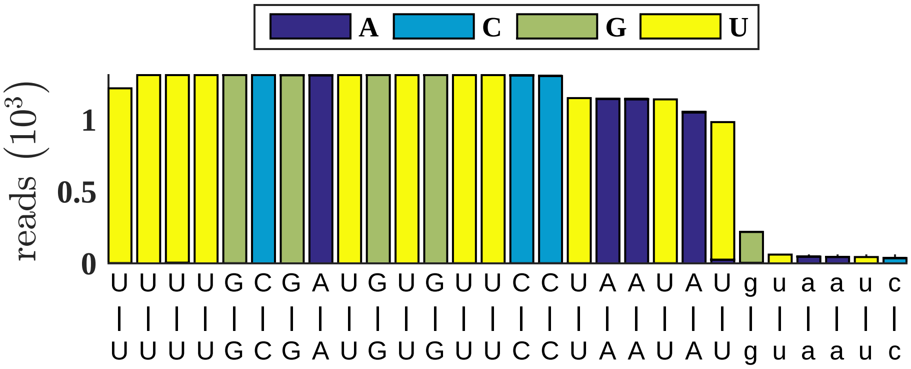

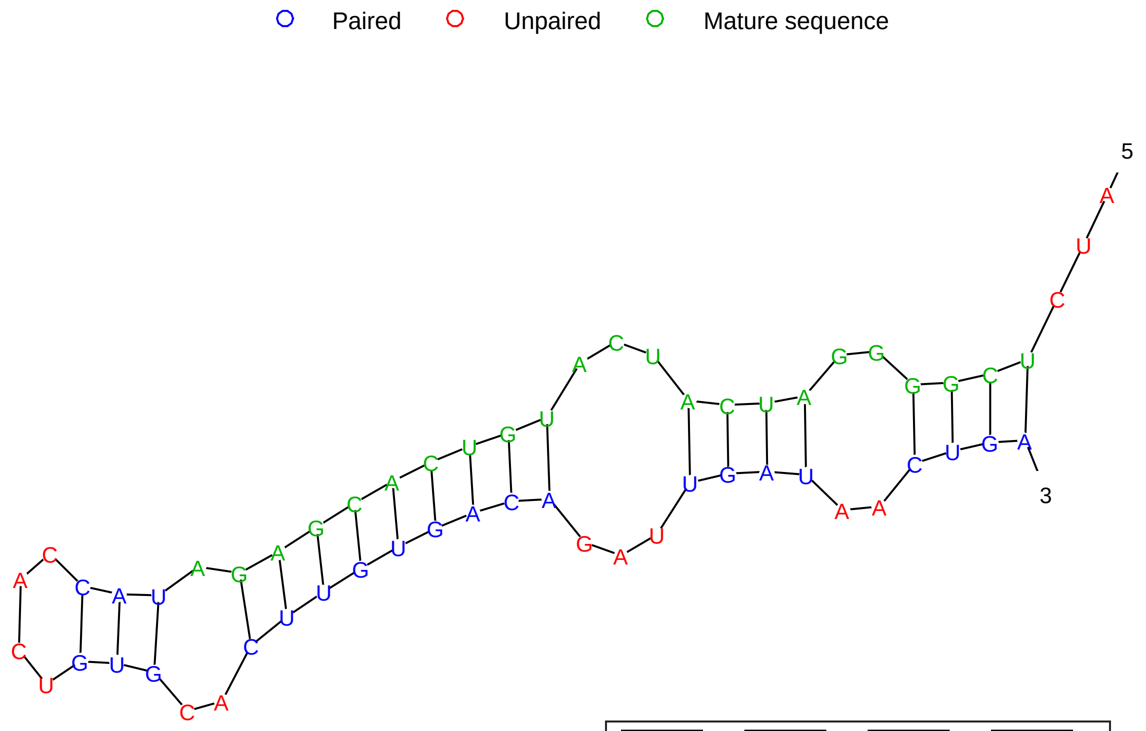

miRNA: bta-miR-542-5p
Stem loop (UMD3.1): chrX:18180153-18180212
Mature (UMD3.1): chrX:18180187-18180209
Mature seq len: 23
Total raw counts (9 samples): 585
Average raw counts: 65
Strand: Reverse
Orientation: 5p
Minimum free energy: -21.30

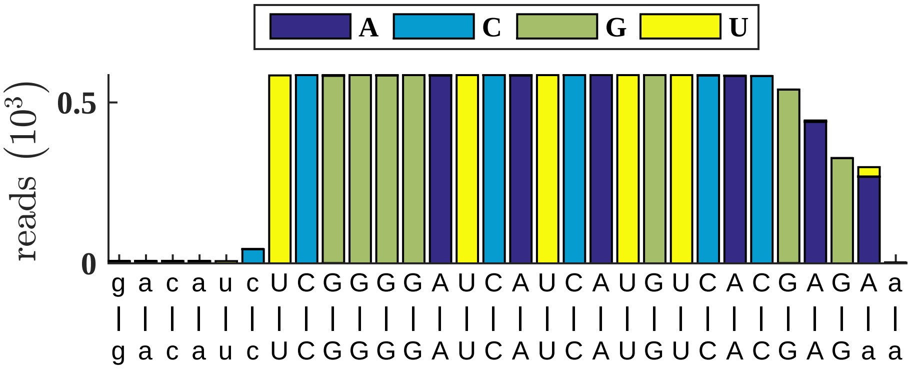

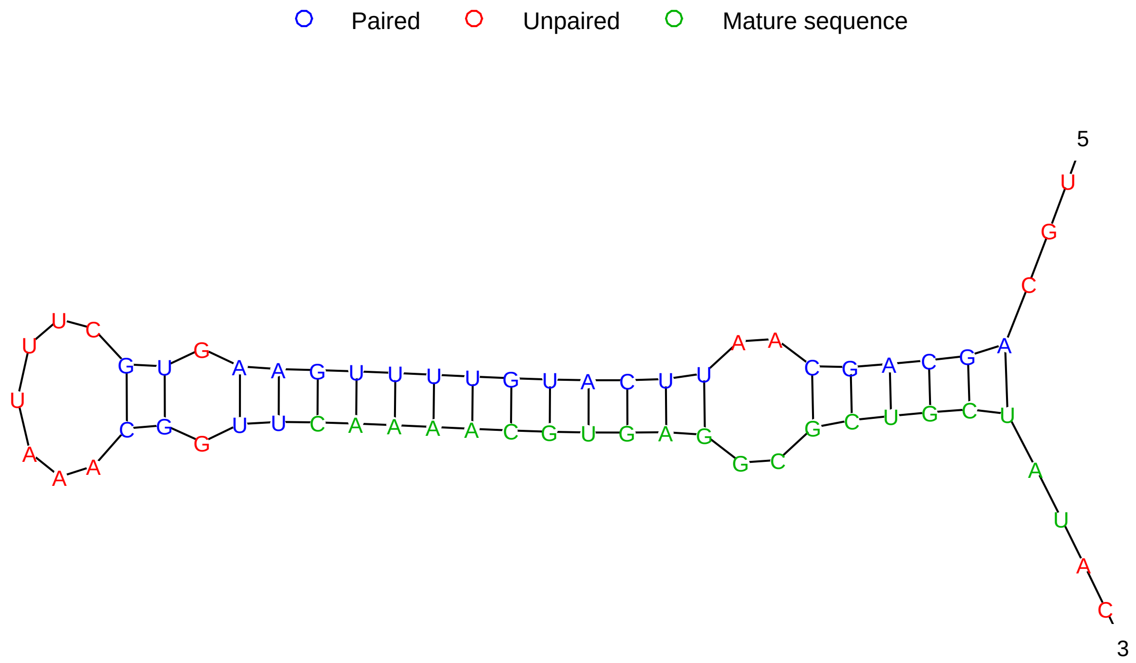

miRNA: bta-miR-424-3p
Stem loop (UMD3.1): chrX:18185455-18185516
Mature (UMD3.1): chrX:18185457-18185477
Mature seq len: 21
Total raw counts (9 samples): 2475
Average raw counts: 275
Strand: Reverse
Orientation: 3p
Minimum free energy: -26.90

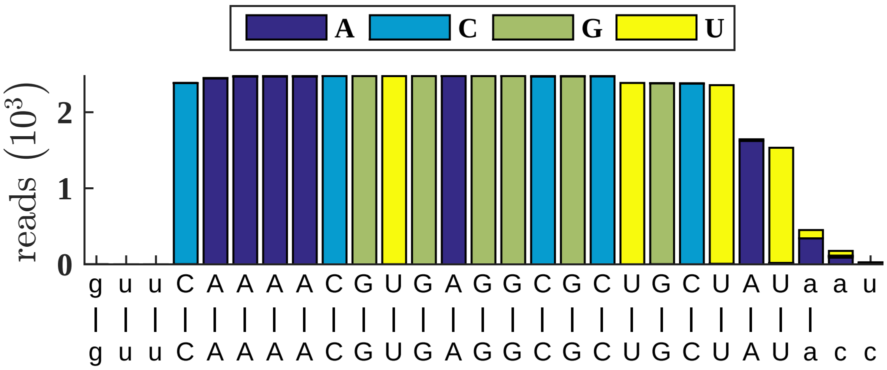

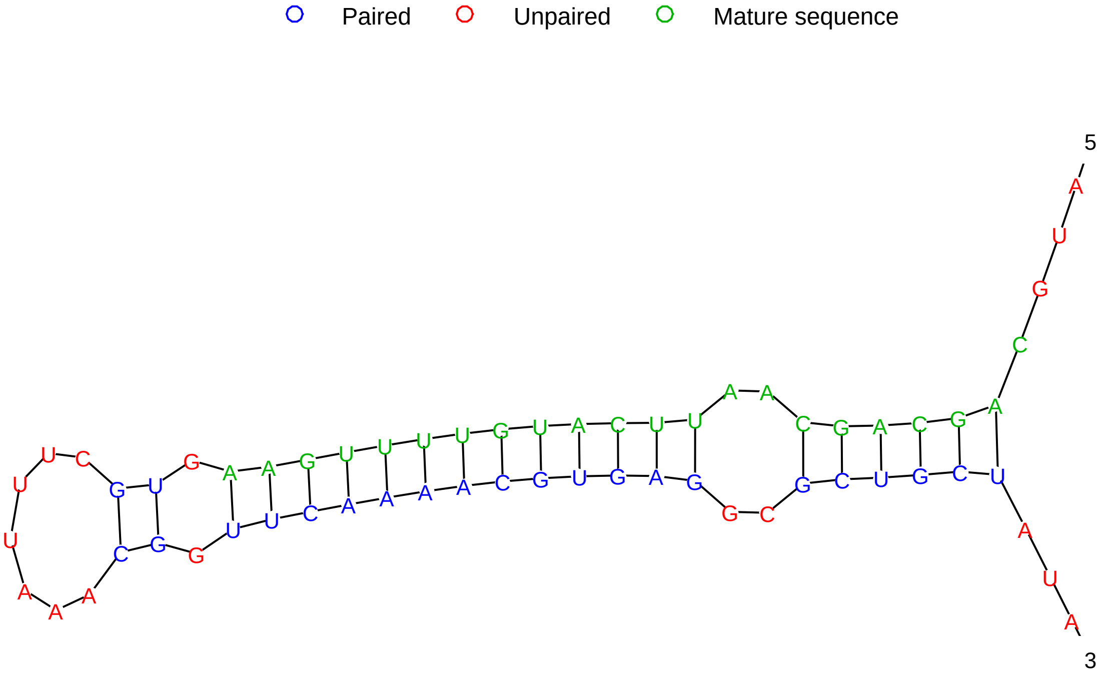

miRNA: bta-miR-424-5p
Stem loop (UMD3.1): chrX:18185456-18185517
Mature (UMD3.1): chrX:18185493-18185514
Mature seq len: 22
Total raw counts (9 samples): 2255
Average raw counts: 251
Strand: Reverse
Orientation: 5p
Minimum free energy: -26.90

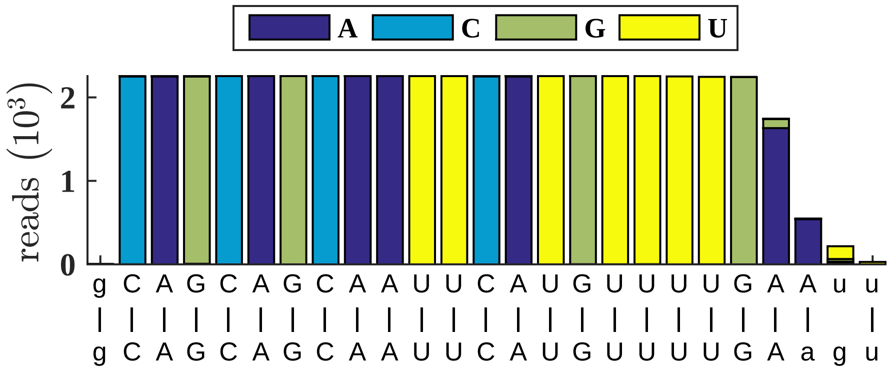

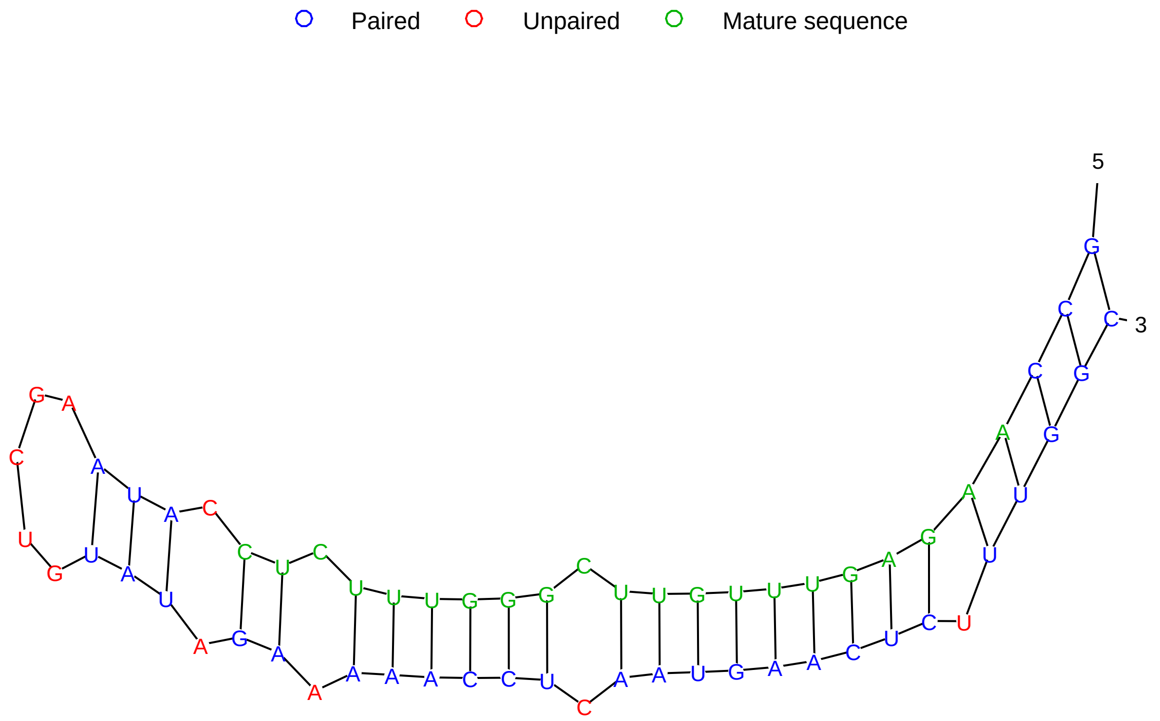

miRNA: bta-miR-2284w
Stem loop (UMD3.1): chrX:2676190-2676251
Mature (UMD3.1): chrX:2676193-2676213
Mature seq len: 21
Total raw counts (9 samples): 565
Average raw counts: 63
Strand: Forward
Orientation: 5p
Minimum free energy: -22.70

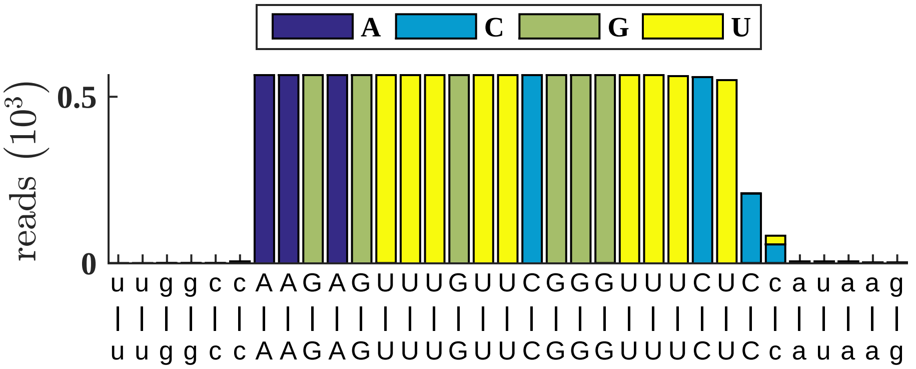

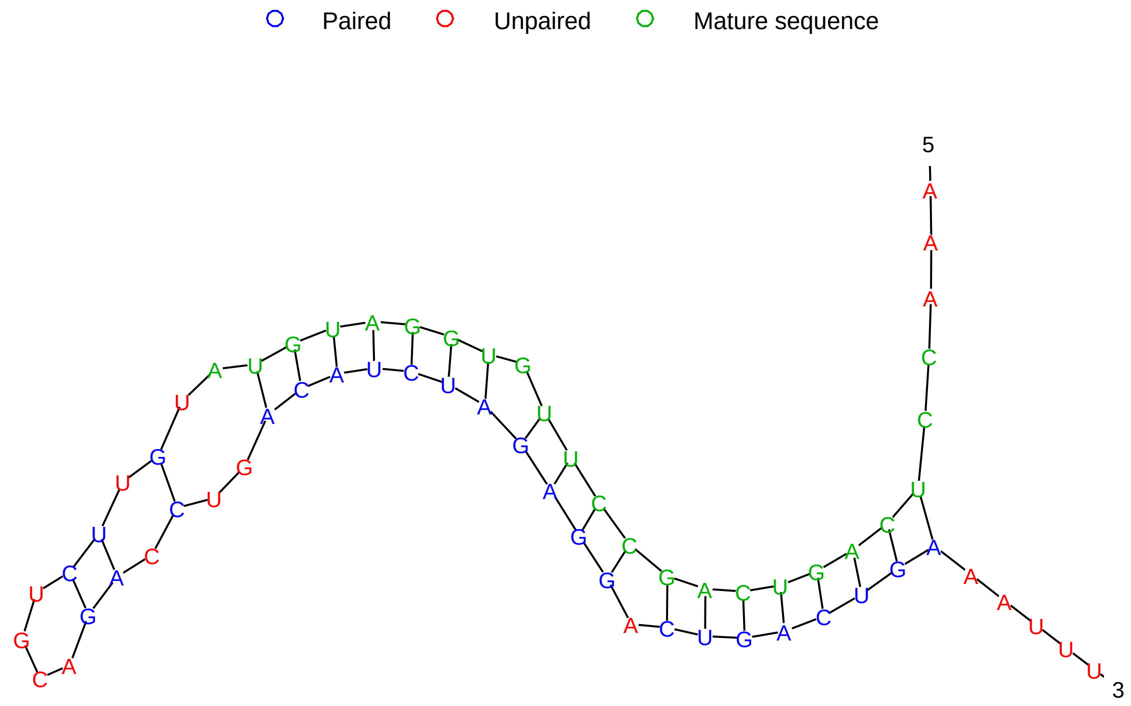

miRNA: bta-miR-3431
Stem loop (UMD3.1): chrX:34662674-34662739
Mature (UMD3.1): chrX:34662714-34662736
Mature seq len: 23
Total raw counts (9 samples): 3365
Average raw counts: 374
Strand: Reverse
Orientation: 5p
Minimum free energy: -26.30

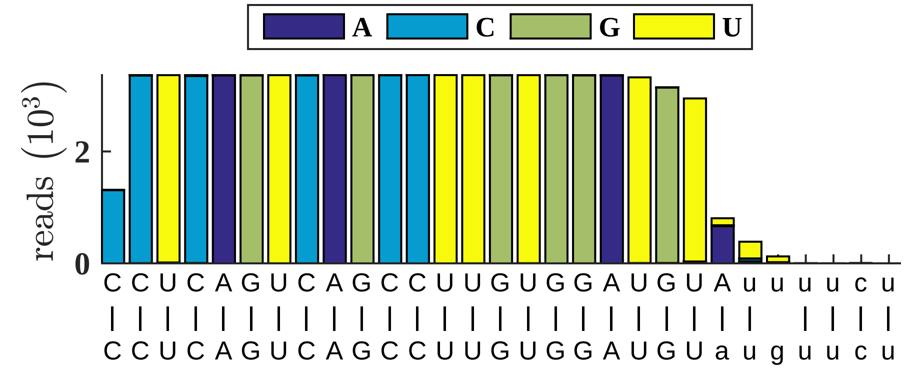

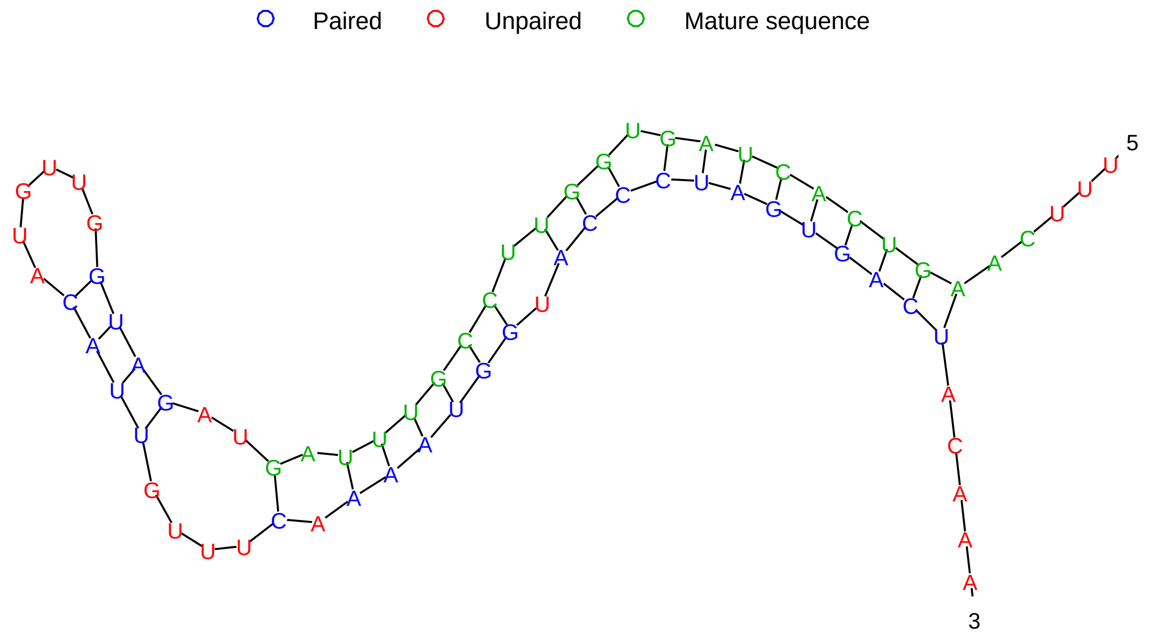

miRNA: bta-miR-224
Stem loop (UMD3.1): chrX:34664594-34664666
Mature (UMD3.1): chrX:34664640-34664663
Mature seq len: 24
Total raw counts (9 samples): 2717
Average raw counts: 302
Strand: Reverse
Orientation: 5p
Minimum free energy: -26.80

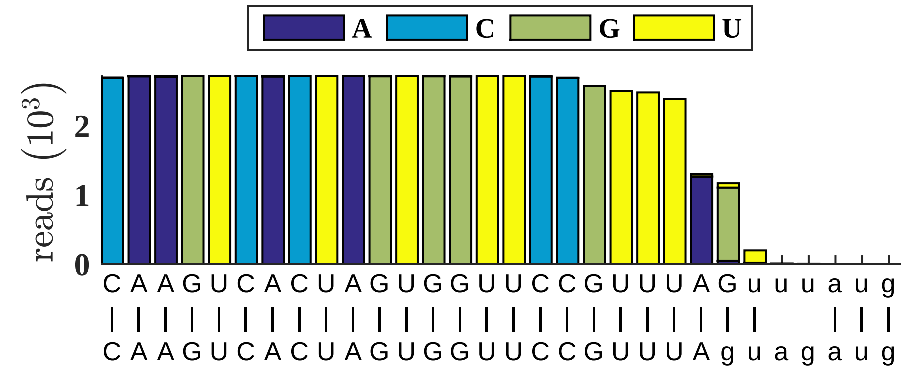

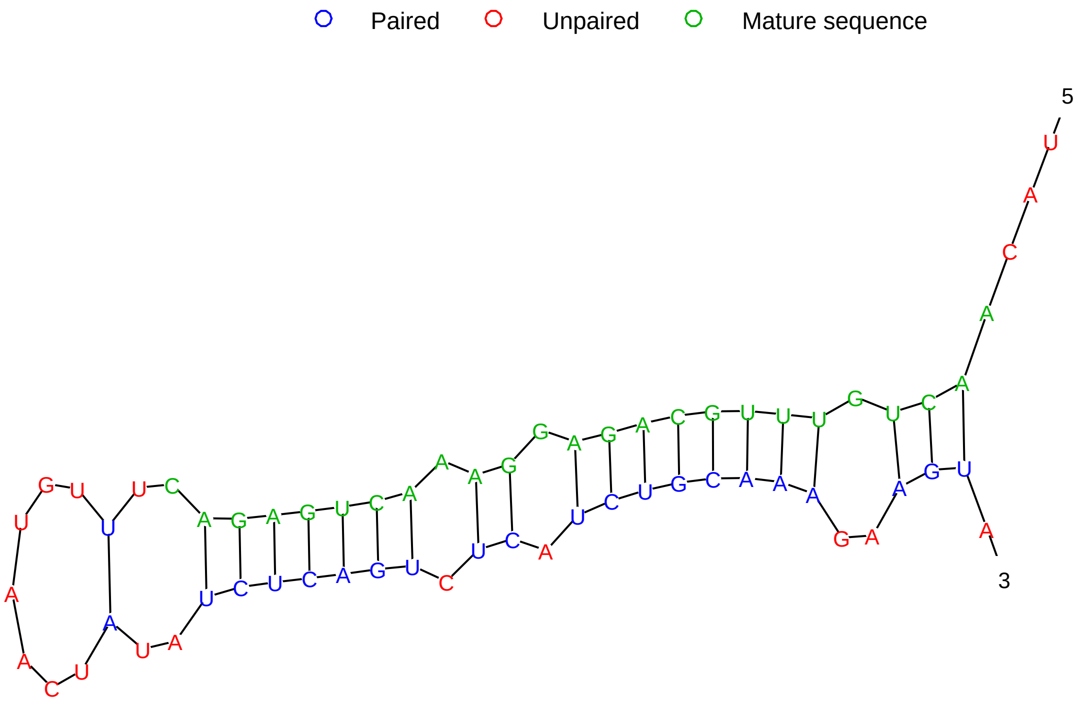

miRNA: bta-miR-452
Stem loop (UMD3.1): chrX:34665634-34665698
Mature (UMD3.1): chrX:34665671-34665695
Mature seq len: 25
Total raw counts (9 samples): 1399
Average raw counts: 156
Strand: Reverse
Orientation: 5p
Minimum free energy: -22.20

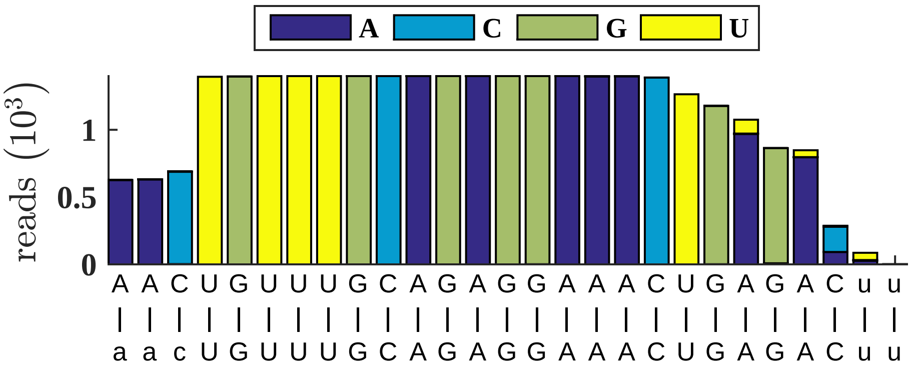

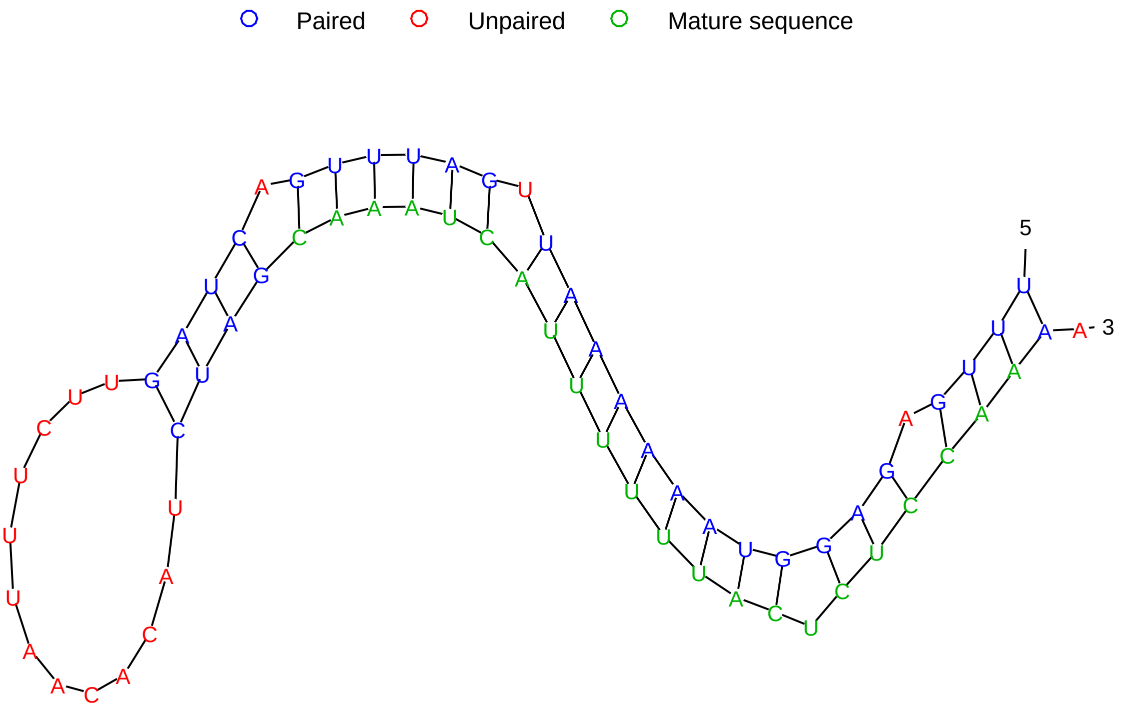

miRNA: bta-miR-6119-3p
Stem loop (UMD3.1): chrX:3998284-3998353
Mature (UMD3.1): chrX:3998286-3998307
Mature seq len: 22
Total raw counts (9 samples): 480
Average raw counts: 54
Strand: Reverse
Orientation: 3p
Minimum free energy: -22.40

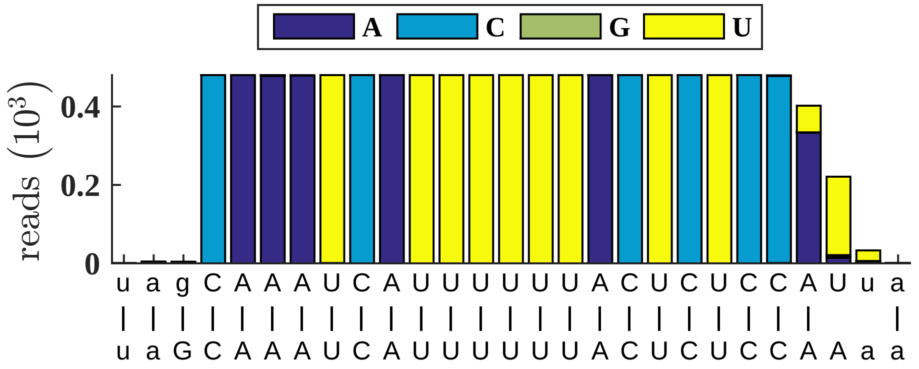

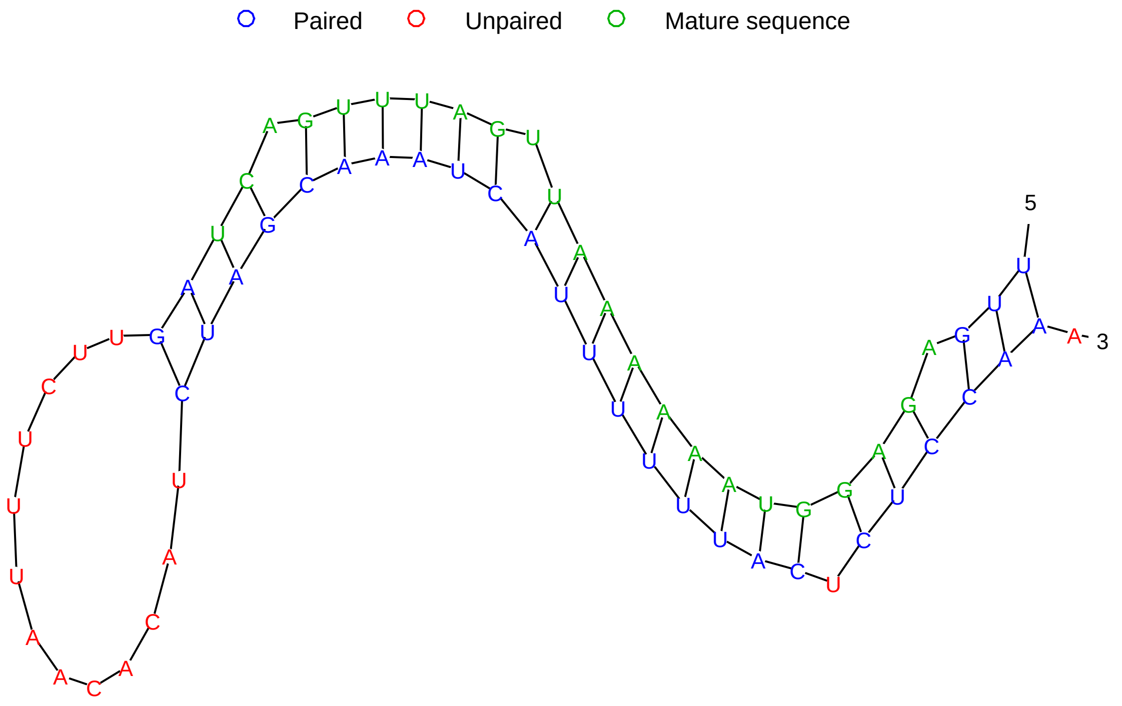

miRNA: bta-miR-6119-5p
Stem loop (UMD3.1): chrX:3998285-3998352
Mature (UMD3.1): chrX:3998327-3998349
Mature seq len: 23
Total raw counts (9 samples): 16832
Average raw counts: 1871
Strand: Reverse
Orientation: 5p
Minimum free energy: -21.50

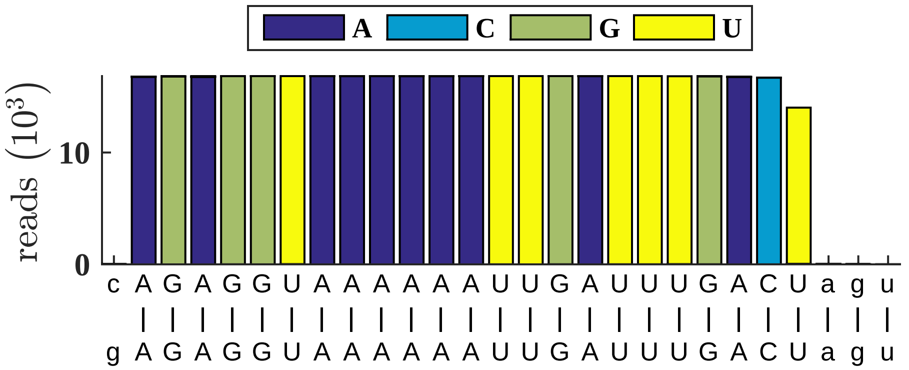

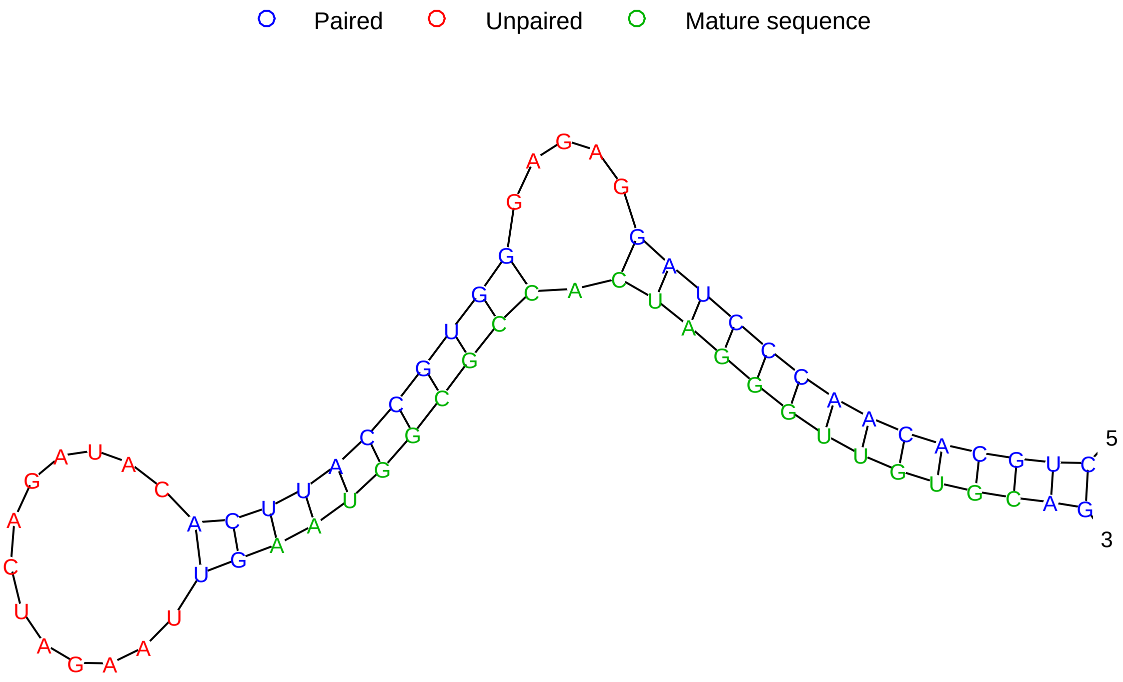

miRNA: bta-miR-652
Stem loop (UMD3.1): chrX:62939298-62939366
Mature (UMD3.1): chrX:62939343-62939364
Mature seq len: 22
Total raw counts (9 samples): 5785
Average raw counts: 643
Strand: Forward
Orientation: 3p
Minimum free energy: -40.70

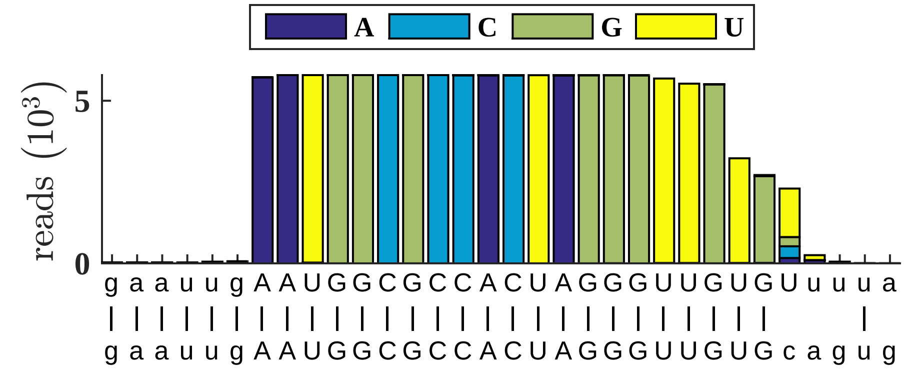

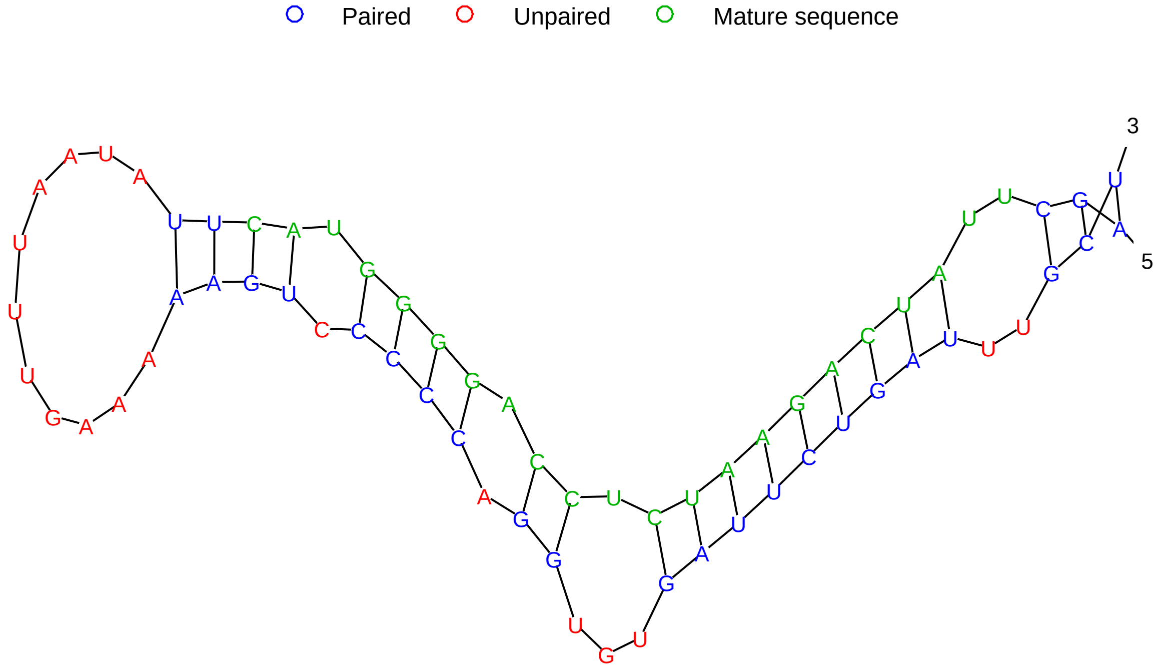

miRNA: bta-miR-361
Stem loop (UMD3.1): chrX:74328424-74328490
Mature (UMD3.1): chrX:74328427-74328448
Mature seq len: 22
Total raw counts (9 samples): 12484
Average raw counts: 1388
Strand: Forward
Orientation: 5p
Minimum free energy: -27.10

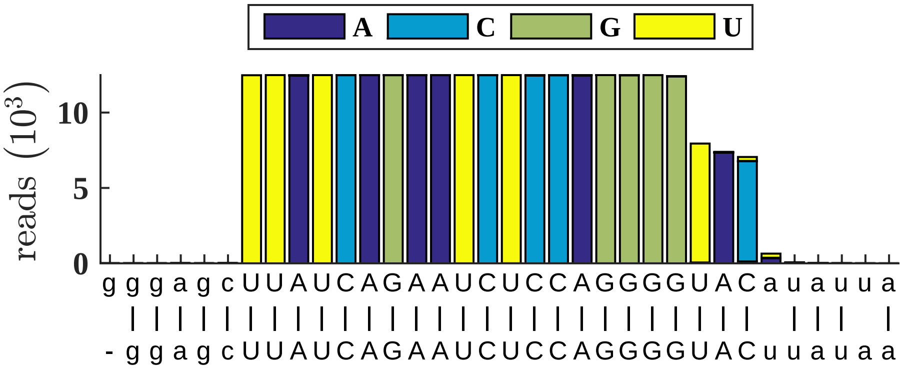

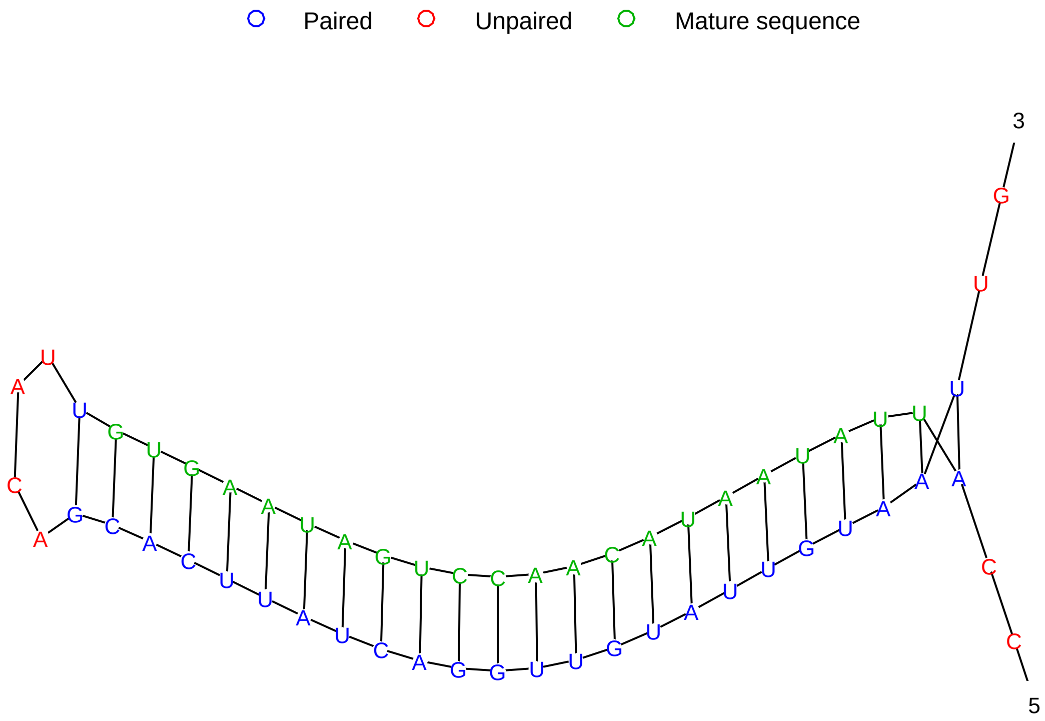

miRNA: bta-miR-374a
Stem loop (UMD3.1): chrX:81951231-81951286
Mature (UMD3.1): chrX:81951234-81951255
Mature seq len: 22
Total raw counts (9 samples): 2005
Average raw counts: 223
Strand: Forward
Orientation: 5p
Minimum free energy: -33.20

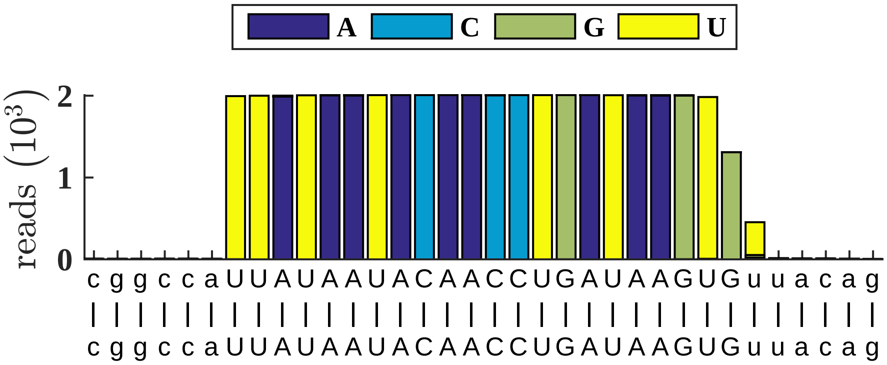

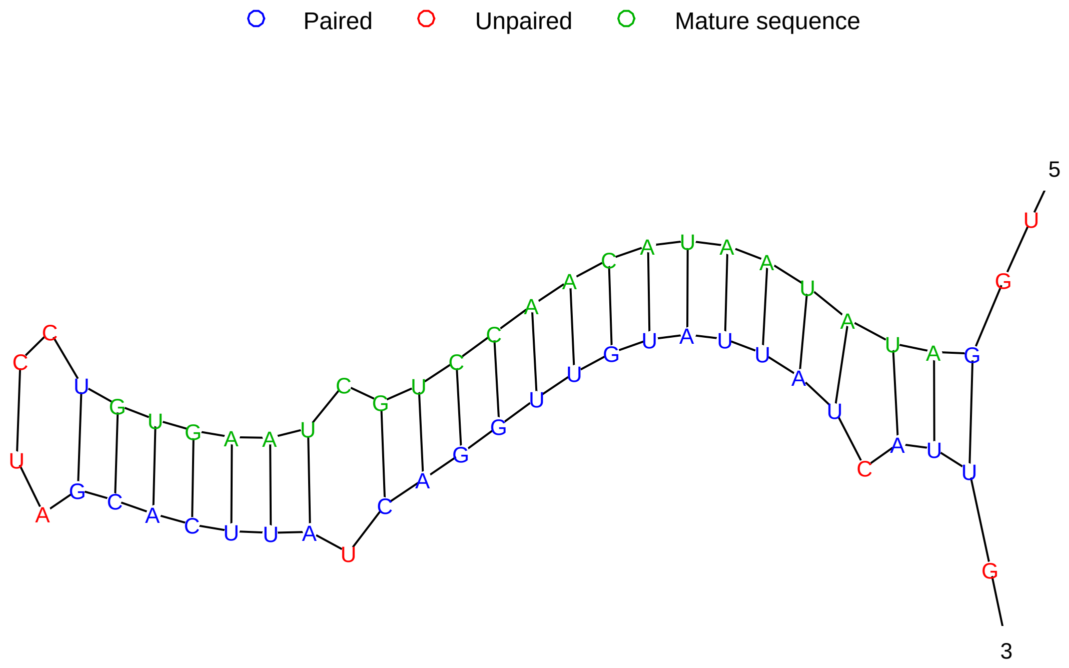

miRNA: bta-miR-374b
Stem loop (UMD3.1): chrX:82023929-82023984
Mature (UMD3.1): chrX:82023932-82023953
Mature seq len: 22
Total raw counts (9 samples): 3797
Average raw counts: 422
Strand: Forward
Orientation: 5p
Minimum free energy: -25.30

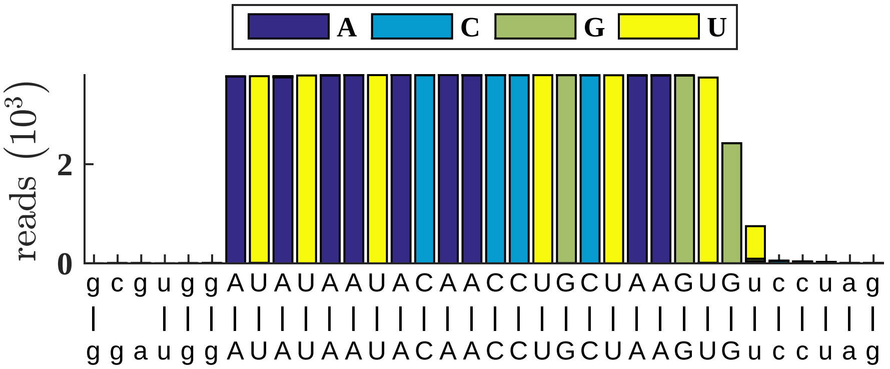

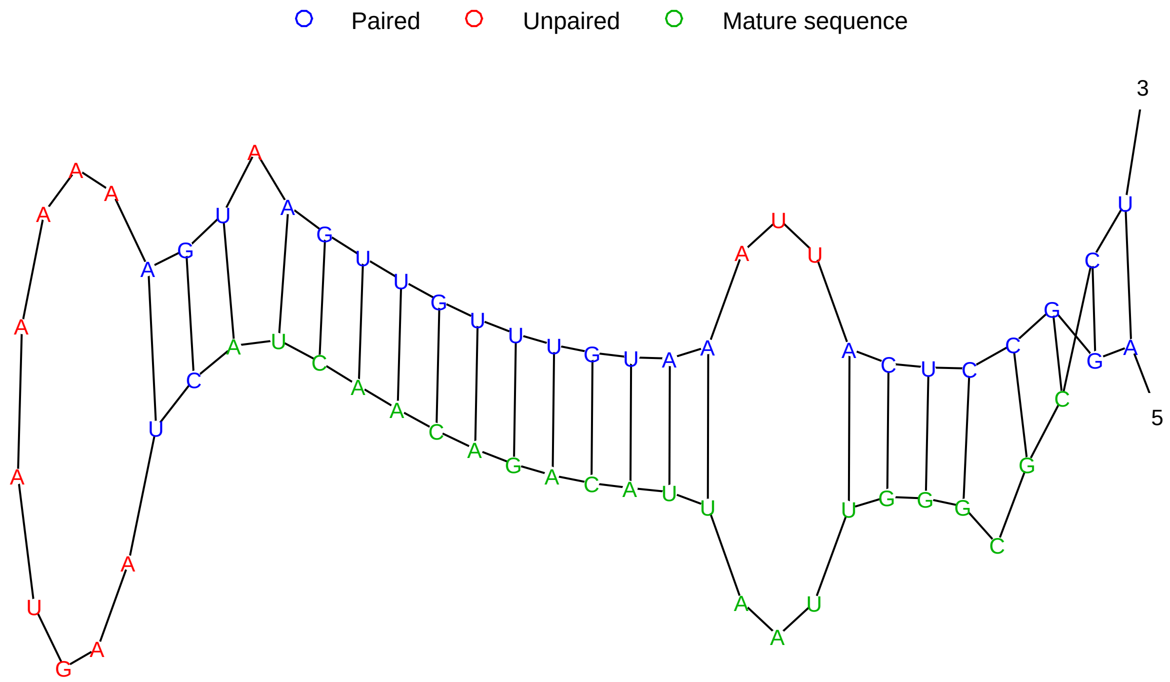

miRNA: bta-miR-421
Stem loop (UMD3.1): chrX:82024088-82024150
Mature (UMD3.1): chrX:82024126-82024148
Mature seq len: 23
Total raw counts (9 samples): 14909
Average raw counts: 1657
Strand: Forward
Orientation: 3p
Minimum free energy: -24.30

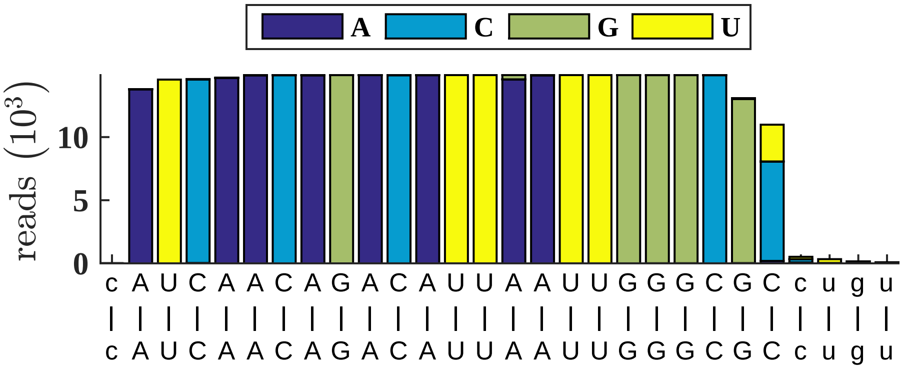

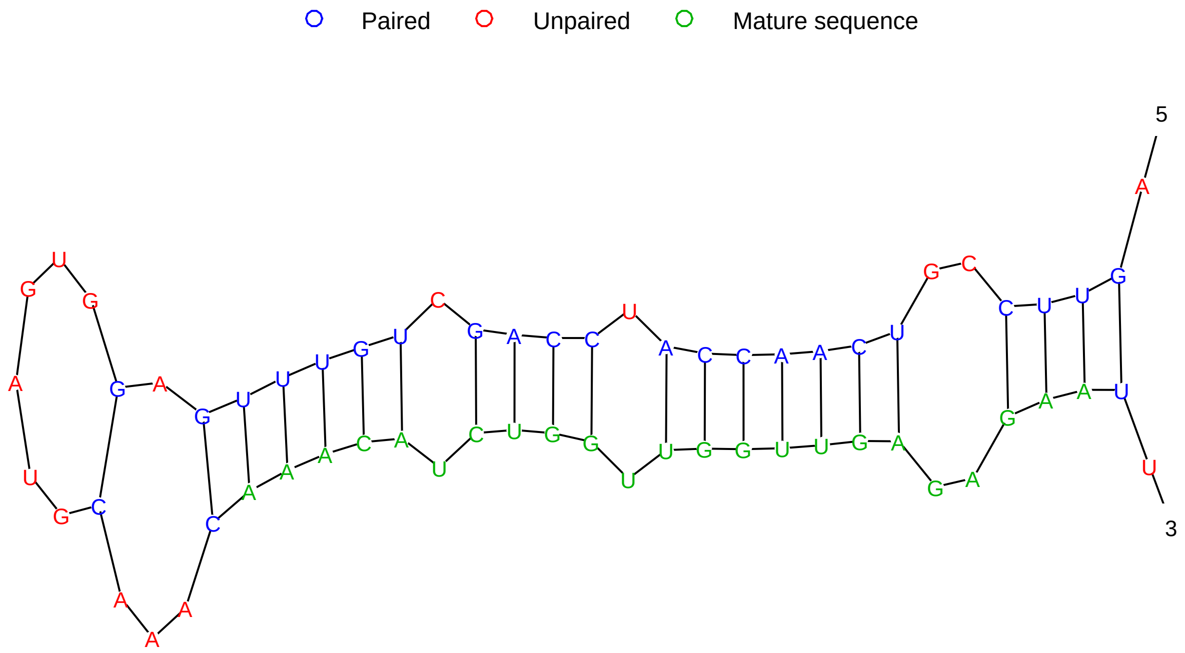

miRNA: bta-miR-2483-3p
Stem loop (UMD3.1): chrX:9189814-9189877
Mature (UMD3.1): chrX:9189816-9189838
Mature seq len: 23
Total raw counts (9 samples): 1196
Average raw counts: 133
Strand: Reverse
Orientation: 3p
Minimum free energy: -25.90

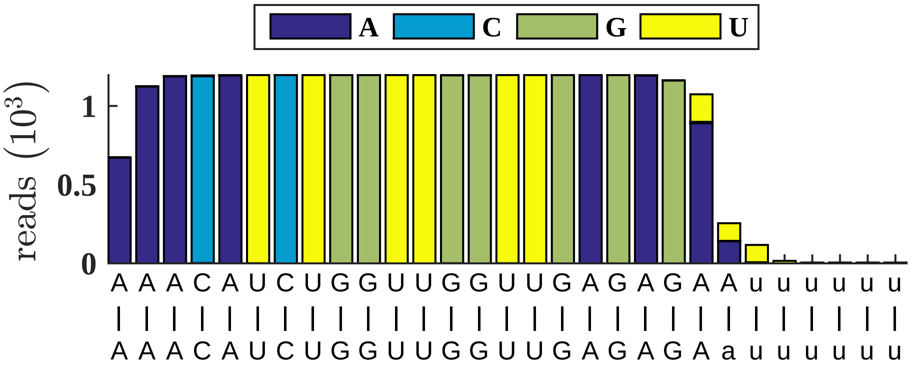

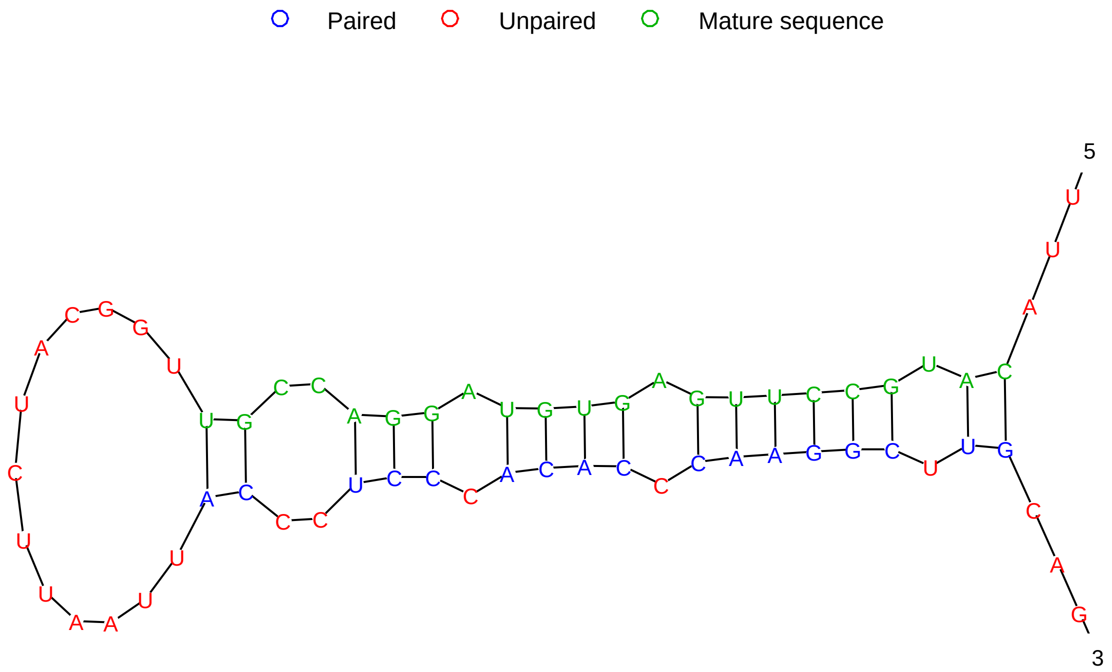

miRNA: bta-miR-532
Stem loop (UMD3.1): chrX:92886516-92886578
Mature (UMD3.1): chrX:92886519-92886540
Mature seq len: 22
Total raw counts (9 samples): 17591
Average raw counts: 1955
Strand: Forward
Orientation: 5p
Minimum free energy: -19.20

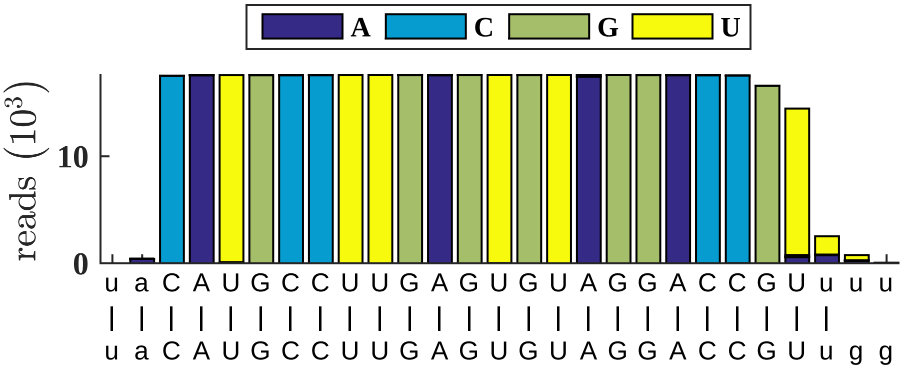

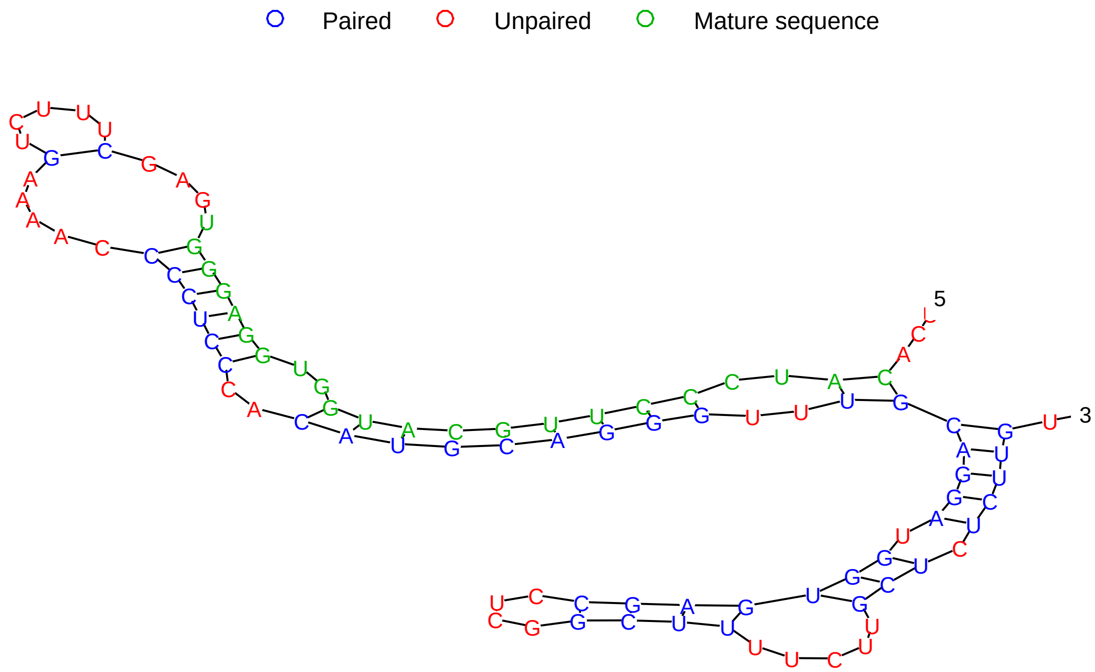

miRNA: bta-miR-188
Stem loop (UMD3.1): chrX:92886838-92886934
Mature (UMD3.1): chrX:92886841-92886862
Mature seq len: 22
Total raw counts (9 samples): 465
Average raw counts: 52
Strand: Forward
Orientation: 5p
Minimum free energy: -38.10

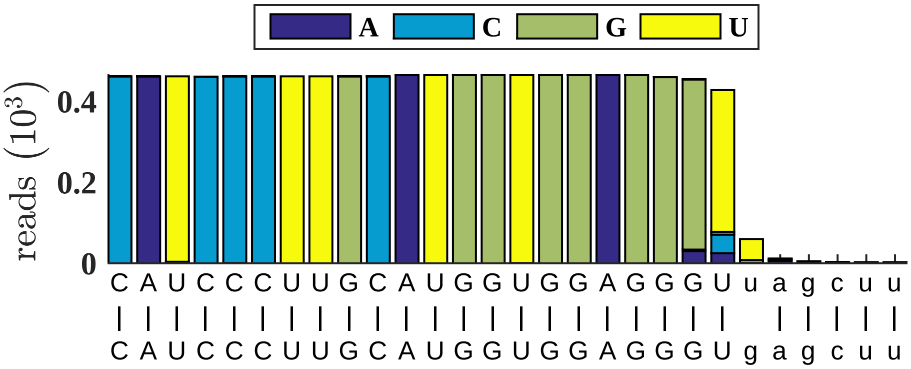

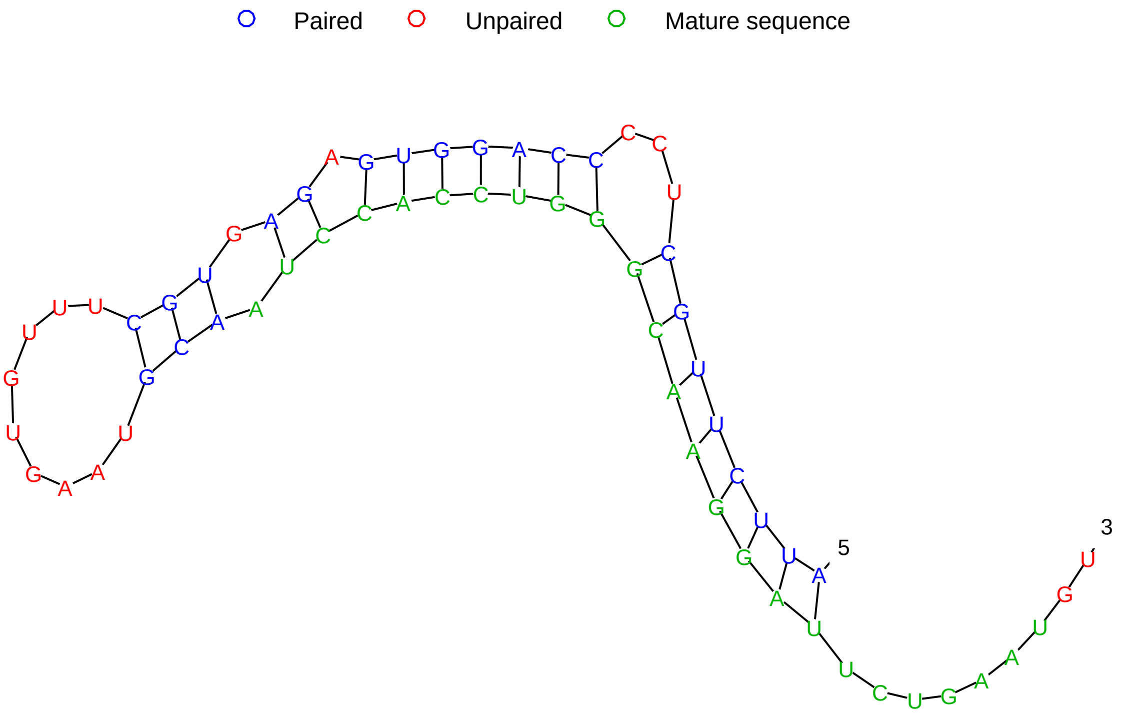

miRNA: bta-miR-502b
Stem loop (UMD3.1): chrX:92897965-92898028
Mature (UMD3.1): chrX:92898002-92898026
Mature seq len: 25
Total raw counts (9 samples): 680
Average raw counts: 76
Strand: Forward
Orientation: 3p
Minimum free energy: -25.10

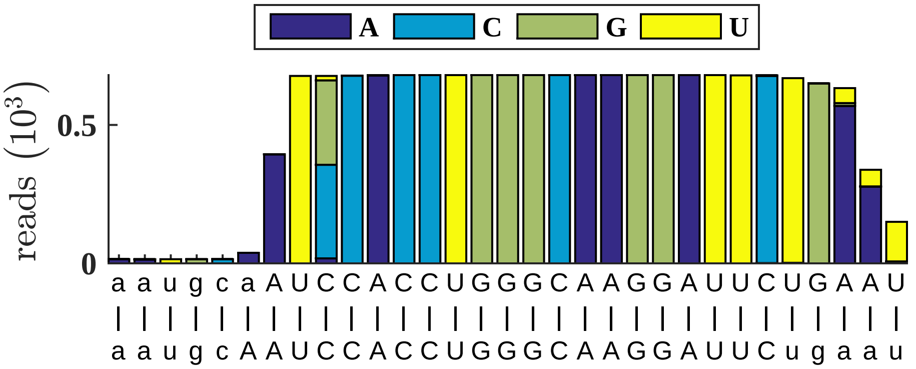

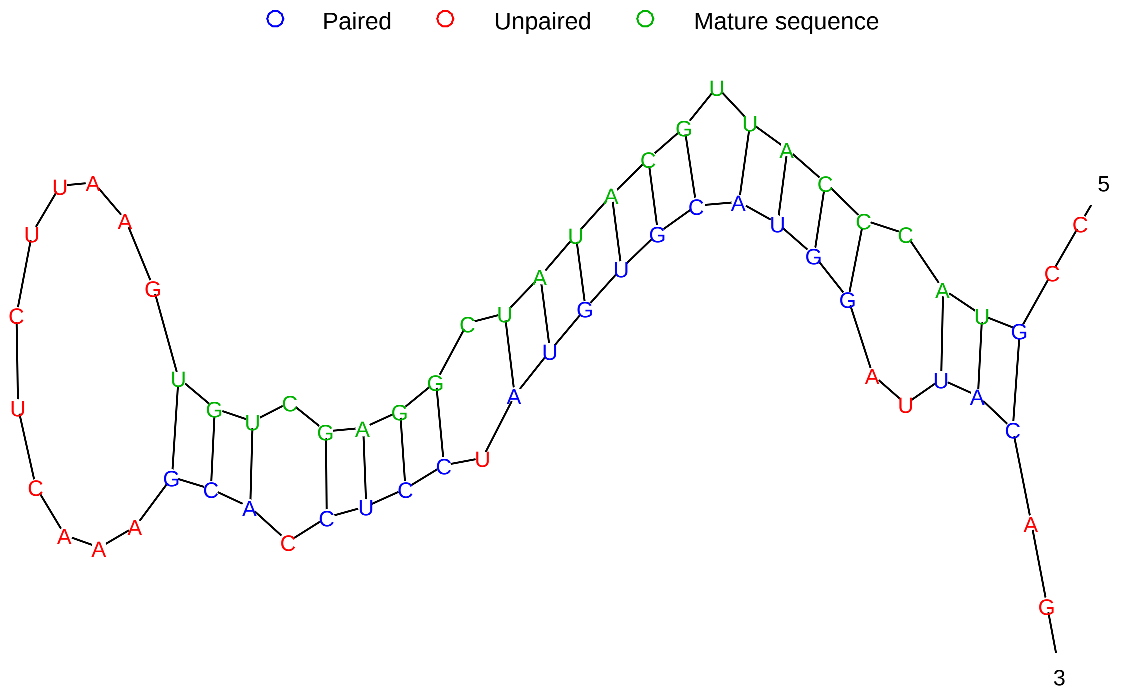

miRNA: bta-miR-660
Stem loop (UMD3.1): chrX:92901862-92901924
Mature (UMD3.1): chrX:92901865-92901887
Mature seq len: 23
Total raw counts (9 samples): 80498
Average raw counts: 8945
Strand: Forward
Orientation: 5p
Minimum free energy: -20.10

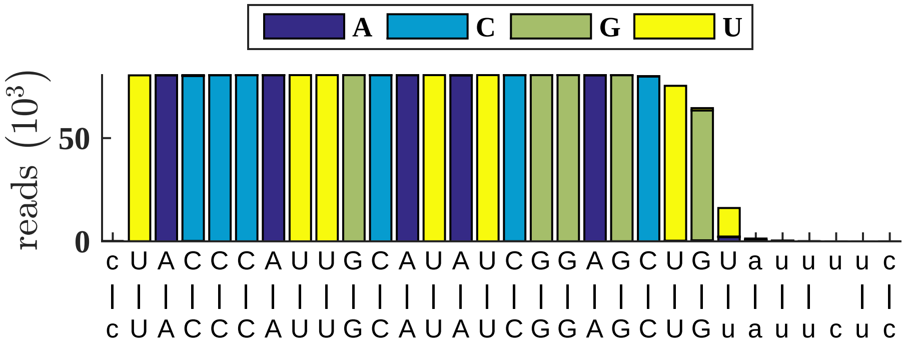

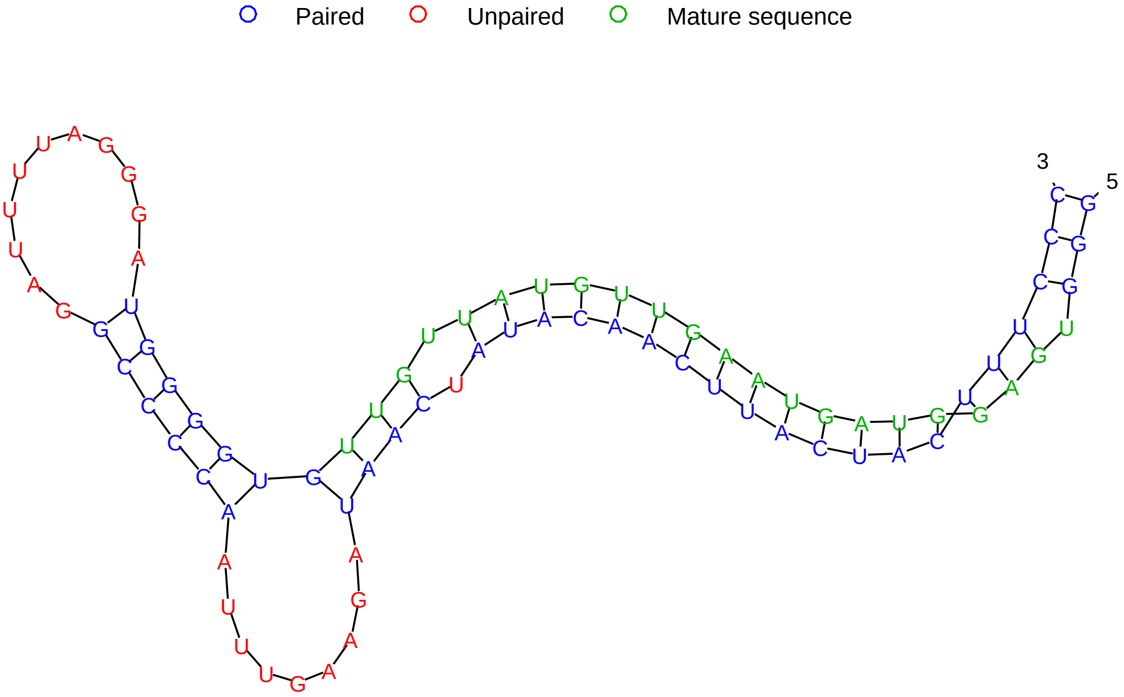

miRNA: bta-miR-98
Stem loop (UMD3.1): chrX:96382646-96382728
Mature (UMD3.1): chrX:96382704-96382725
Mature seq len: 22
Total raw counts (9 samples): 7299
Average raw counts: 811
Strand: Reverse
Orientation: 5p
Minimum free energy: -35.80

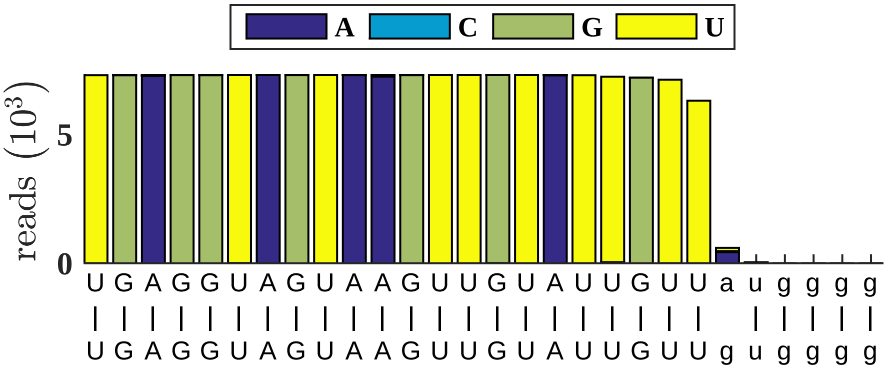

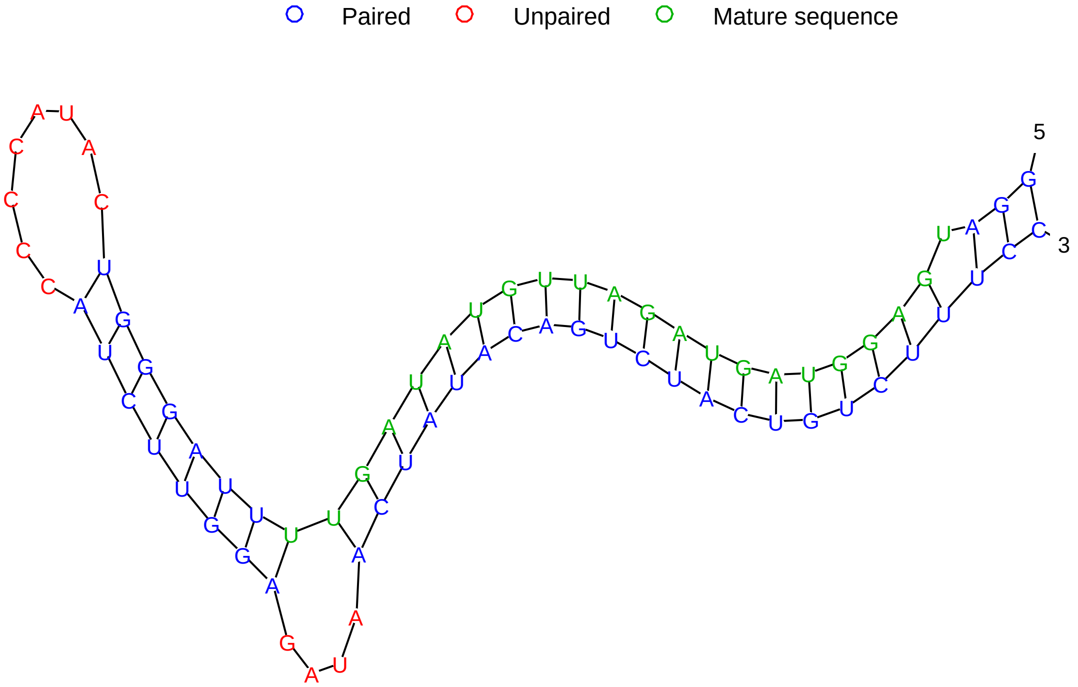

miRNA: bta-let-7f
Stem loop (UMD3.1): chrX:96383536-96383610
Mature (UMD3.1): chrX:96383586-96383607
Mature seq len: 22
Total raw counts (9 samples): 286565
Average raw counts: 31841
Strand: Reverse
Orientation: 5p
Minimum free energy: -29.90

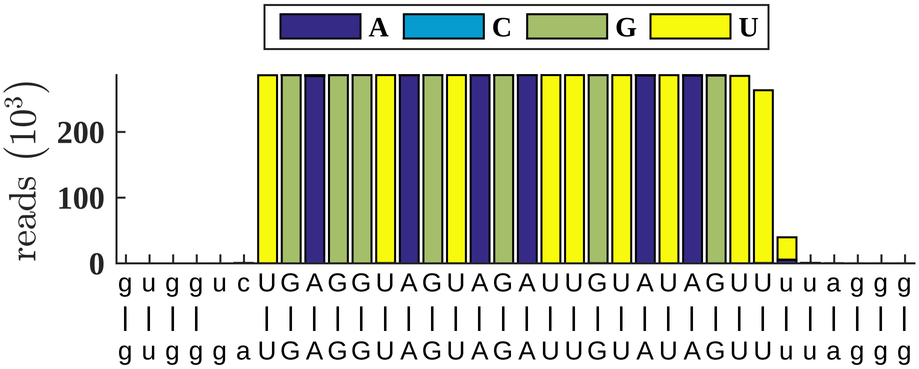

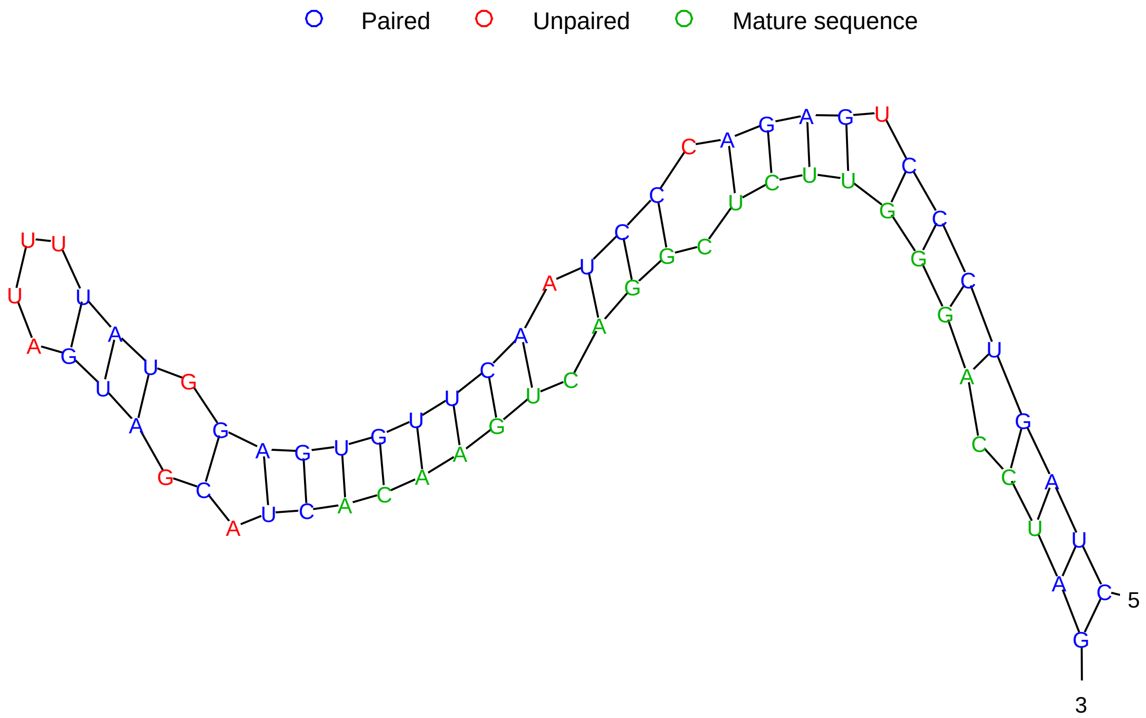

Similar miRNA: chi-miR-125b-3p
Stem loop (UMD3.1): chr1:19881357-19881423
Mature (UMD3.1): chr1:19881359-19881380
Mature seq len: 22
Total raw counts (9 samples): 17831
Average raw counts: 1982
Strand: Reverse
Orientation: 3p
Minimum free energy: -29.60

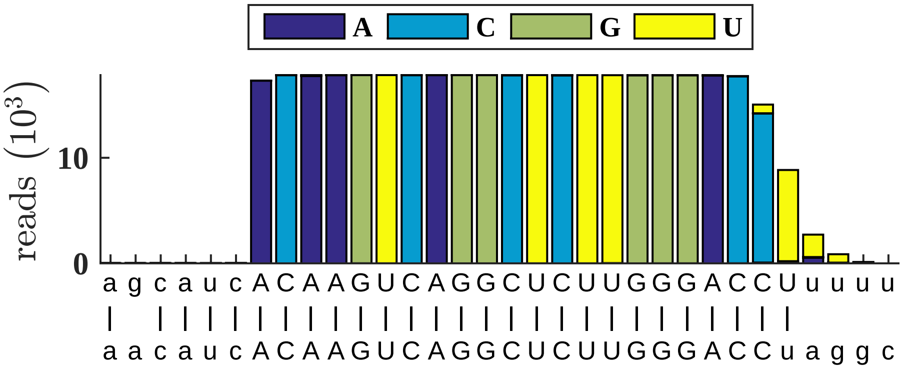

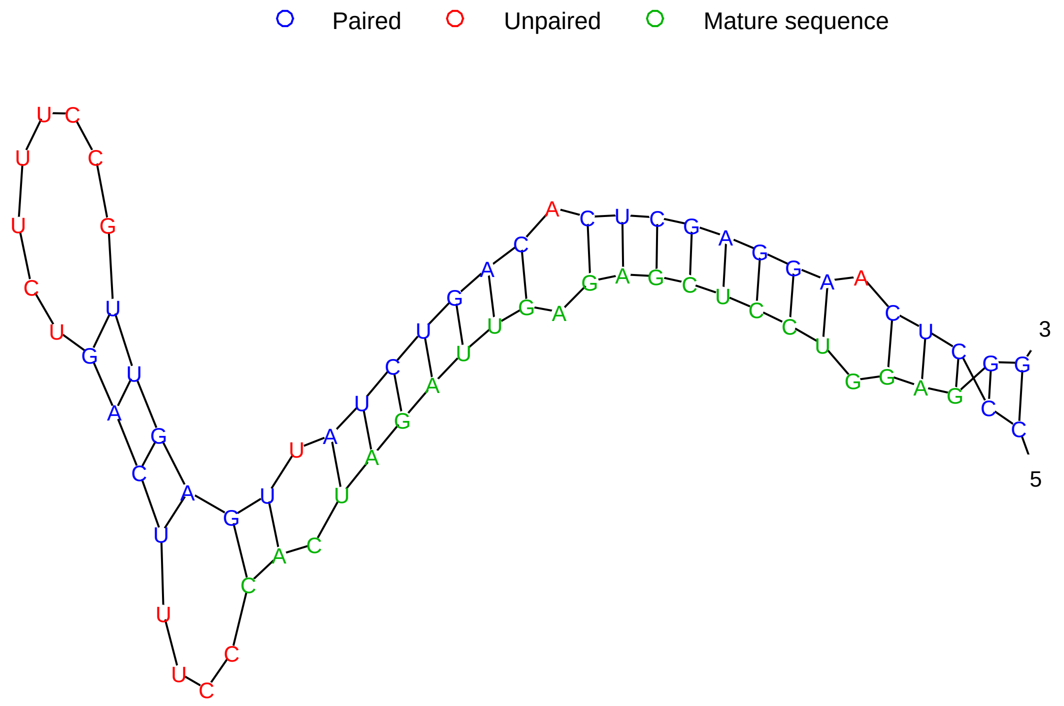

Similar miRNA: chi-miR-28-3p
Stem loop (UMD3.1): chr1:79250539-79250608
Mature (UMD3.1): chr1:79250541-79250563
Mature seq len: 23
Total raw counts (9 samples): 42876
Average raw counts: 4764
Strand: Reverse
Orientation: 3p
Minimum free energy: -33.20

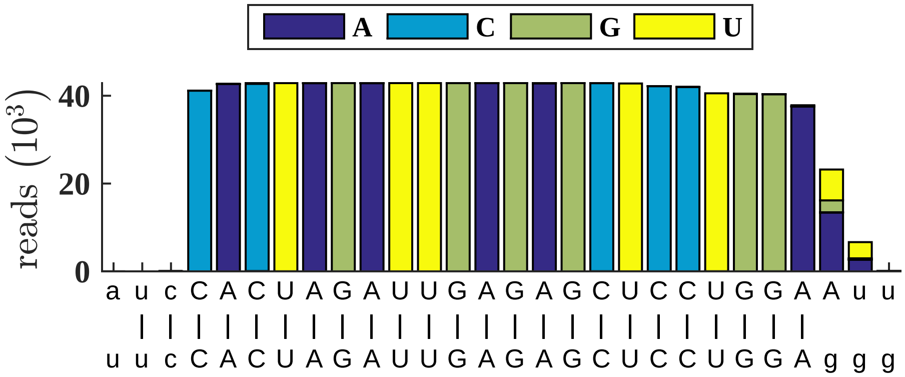

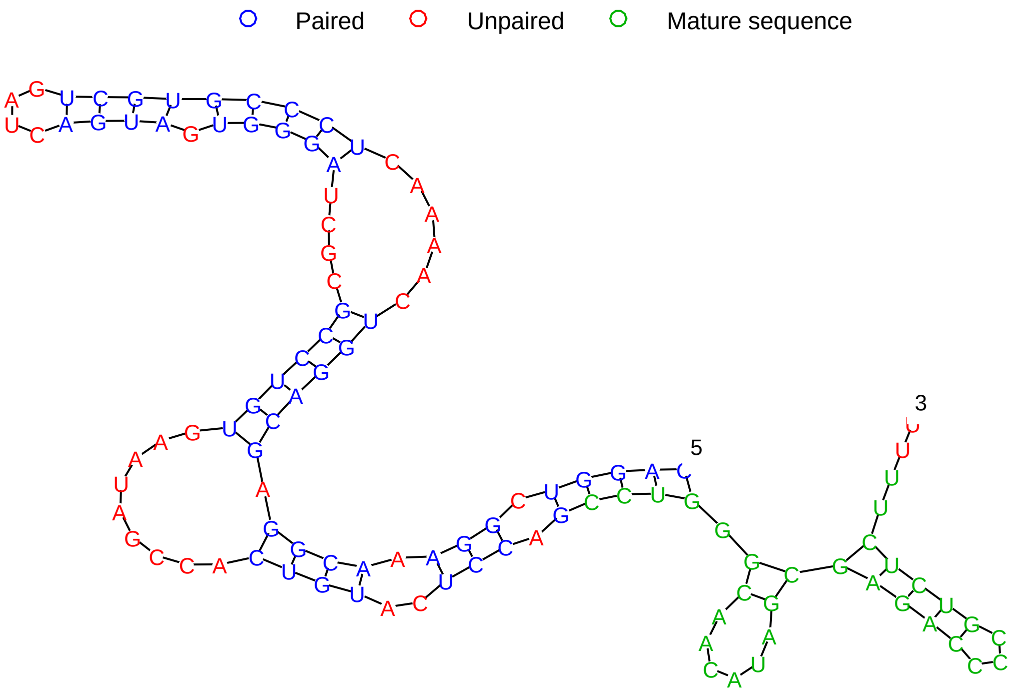

Similar miRNA: ssc-miR-1285
Stem loop (UMD3.1): chr10:42863851-42863962
Mature (UMD3.1): chr10:42863853-42863882
Mature seq len: 30
Total raw counts (9 samples): 847
Average raw counts: 95
Strand: Reverse
Orientation: 3p
Minimum free energy: -33.40

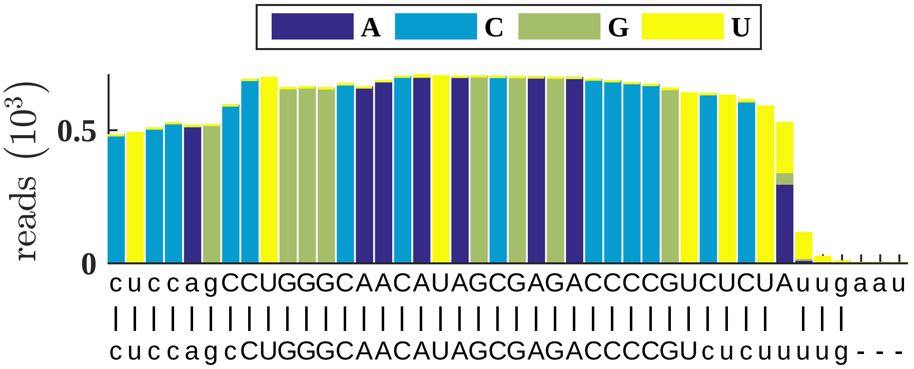

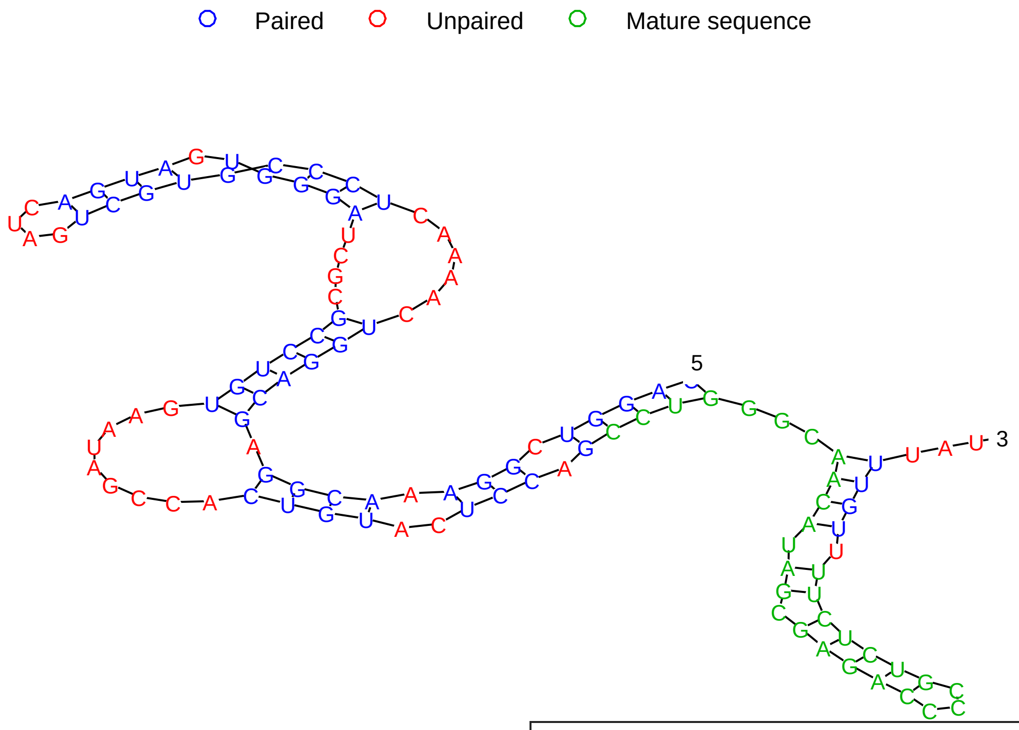

Similar miRNA: ssc-miR-1285
Stem loop (UMD3.1): chr10:42877884-42878001
Mature (UMD3.1): chr10:42877892-42877921
Mature seq len: 30
Total raw counts (9 samples): 816
Average raw counts: 91
Strand: Reverse
Orientation: 3p
Minimum free energy: -33.60

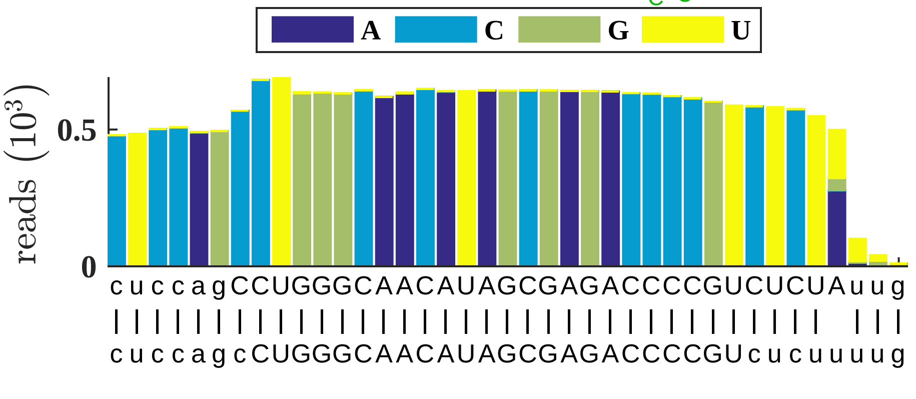

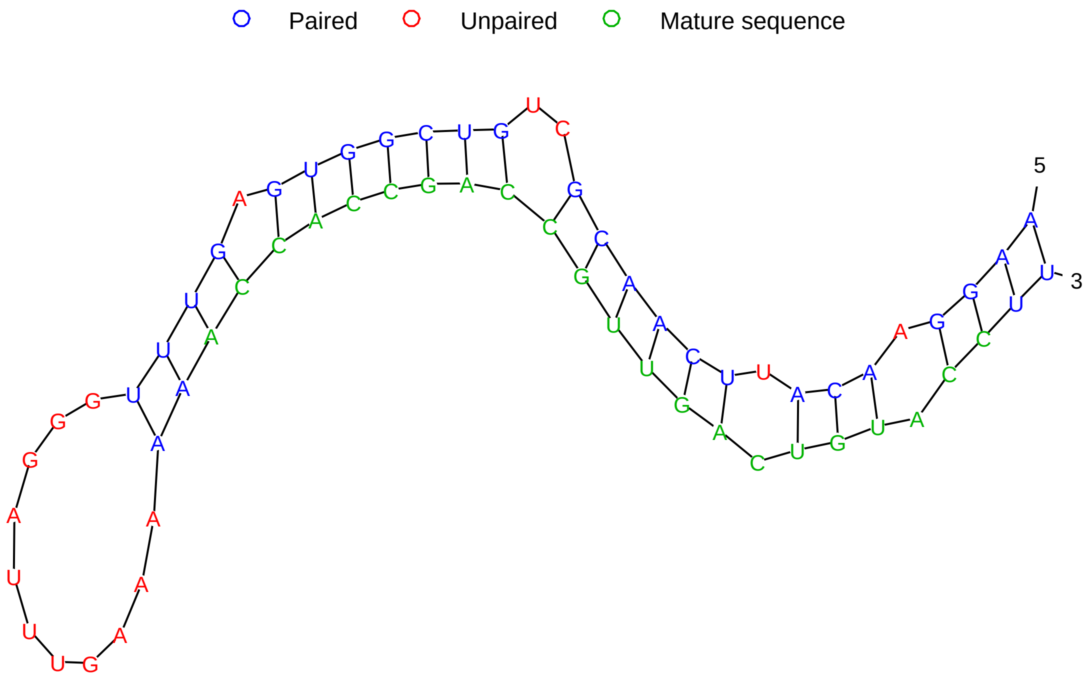

Similar miRNA: efu-miR-181f
Stem loop (UMD3.1): chr11:95709445-95709510
Mature (UMD3.1): chr11:95709487-95709508
Mature seq len: 22
Total raw counts (9 samples): 2757
Average raw counts: 307
Strand: Forward
Orientation: 3p
Minimum free energy: -27.20

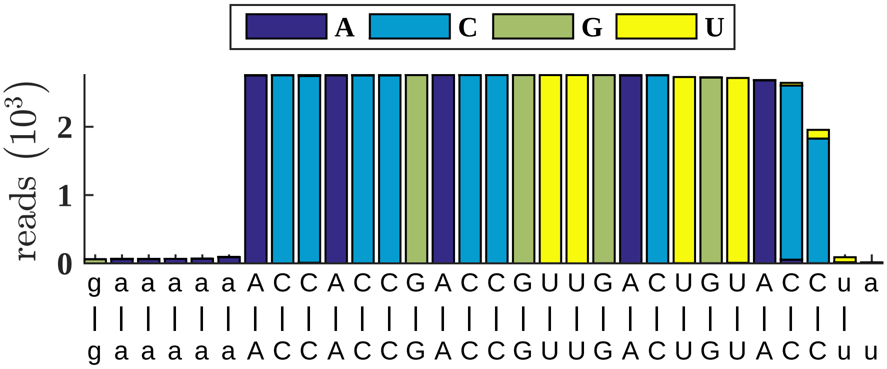

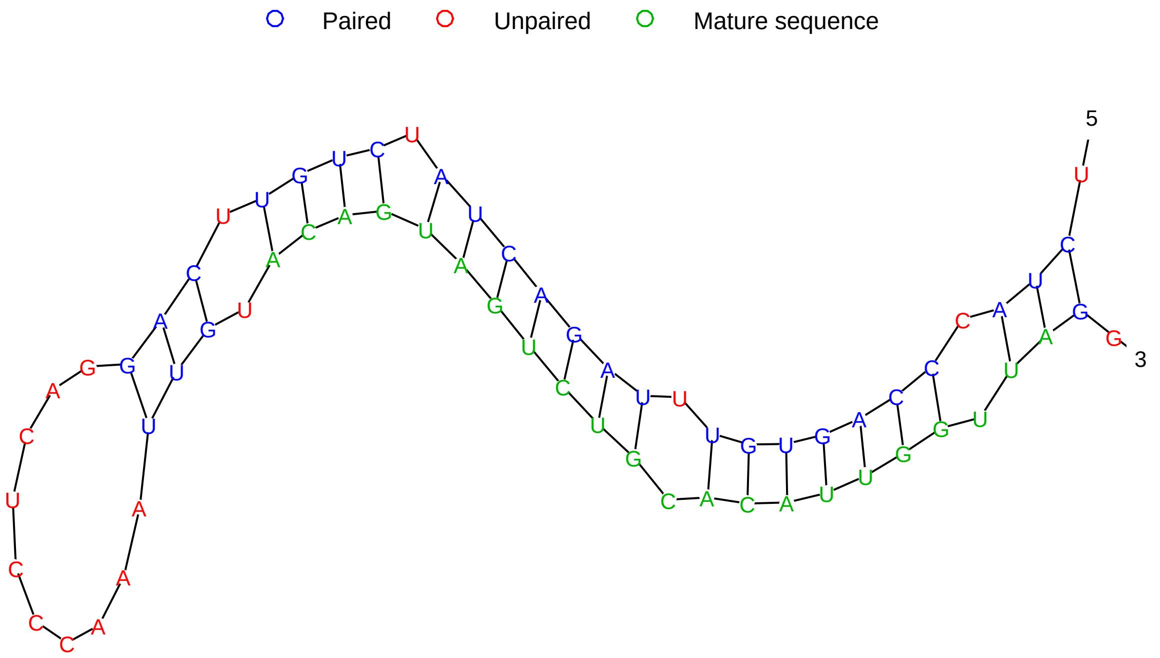

Similar miRNA: eca-miR-199b-3p
Stem loop (UMD3.1): chr11:98867679-98867745
Mature (UMD3.1): chr11:98867681-98867702
Mature seq len: 22
Total raw counts (9 samples): 6254
Average raw counts: 695
Strand: Reverse
Orientation: 3p
Minimum free energy: -25.40

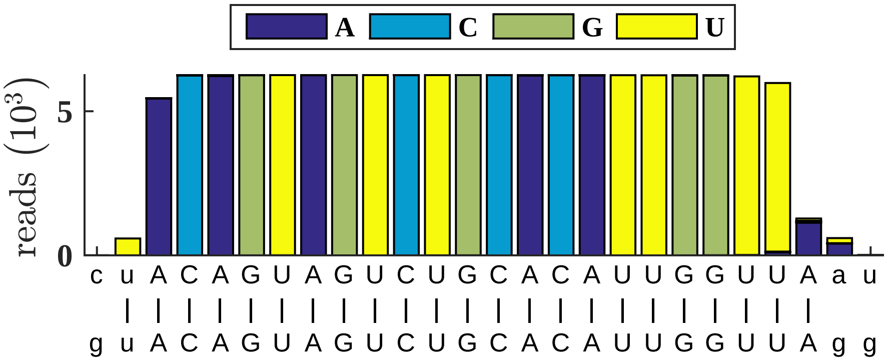

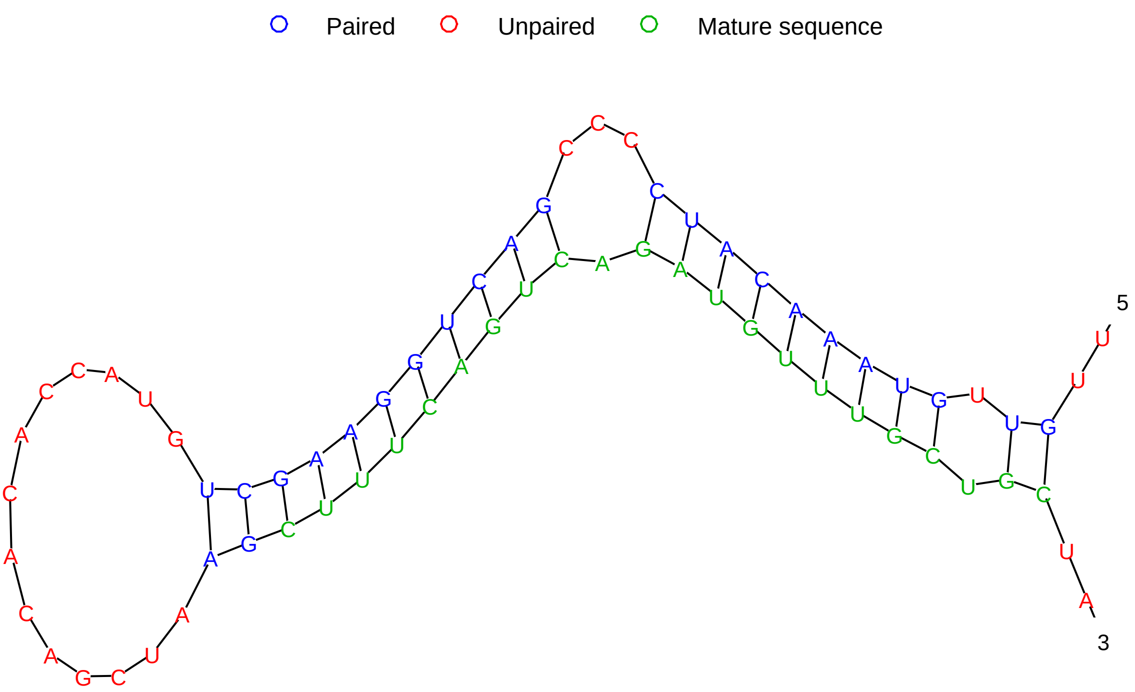

Similar miRNA: chi-miR-30d-3p
Stem loop (UMD3.1): chr14:8080293-8080360
Mature (UMD3.1): chr14:8080337-8080358
Mature seq len: 22
Total raw counts (9 samples): 1374
Average raw counts: 153
Strand: Forward
Orientation: 3p
Minimum free energy: -28.10

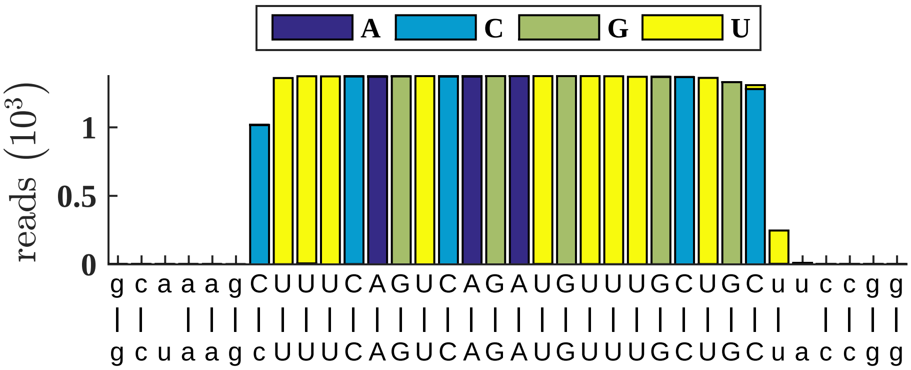

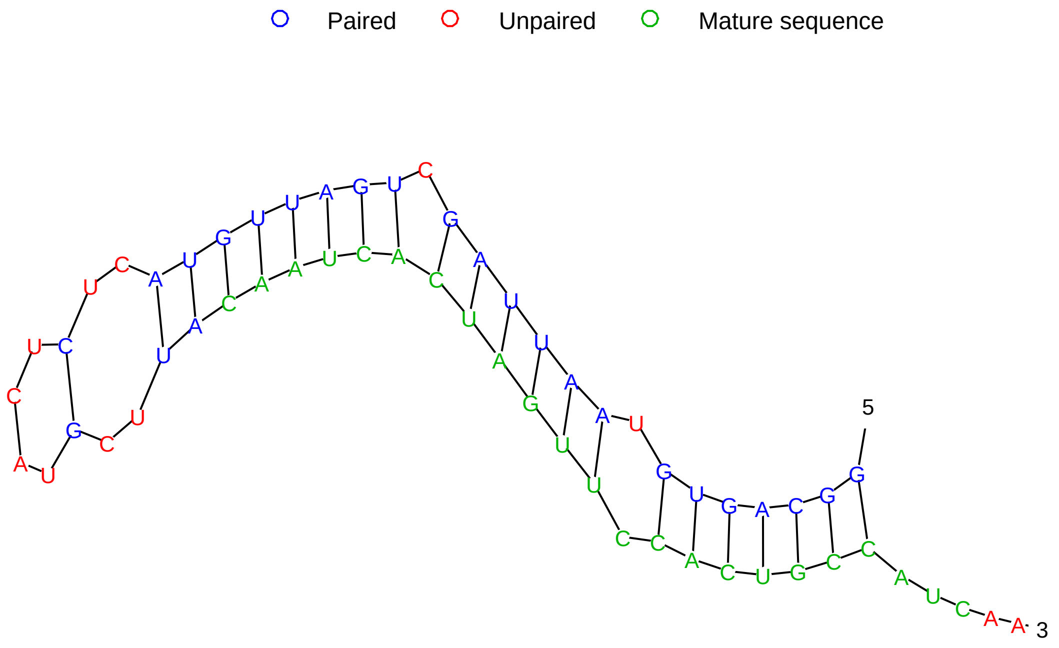

Similar miRNA: chi-miR-34b-3p
Stem loop (UMD3.1): chr15:22134739-22134798
Mature (UMD3.1): chr15:22134774-22134796
Mature seq len: 23
Total raw counts (9 samples): 1308
Average raw counts: 146
Strand: Forward
Orientation: 3p
Minimum free energy: -27.30

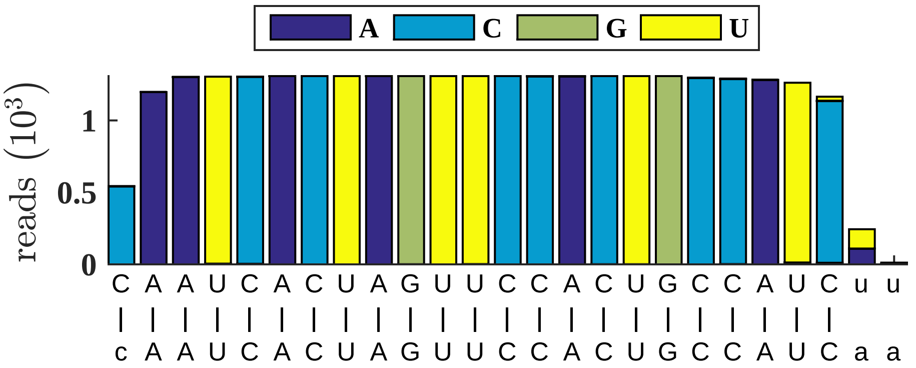

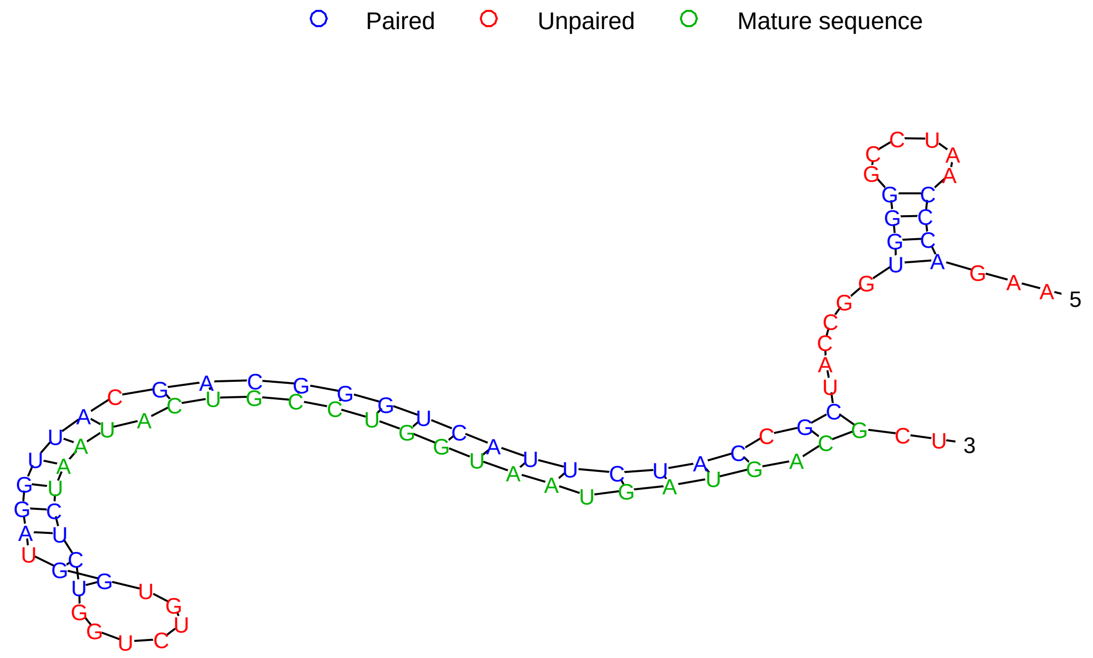

Similar miRNA: tch-miR-200b-3p
Stem loop (UMD3.1): chr16:52521988-52522075
Mature (UMD3.1): chr16:52521990-52522013
Mature seq len: 24
Total raw counts (9 samples): 2264
Average raw counts: 252
Strand: Reverse
Orientation: 3p
Minimum free energy: -31.20

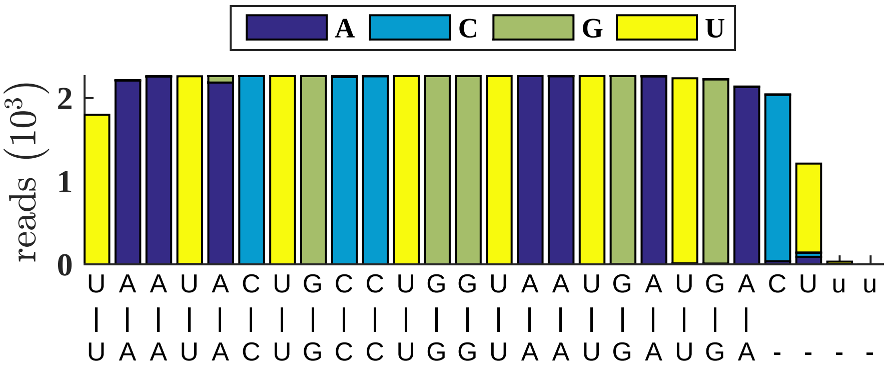

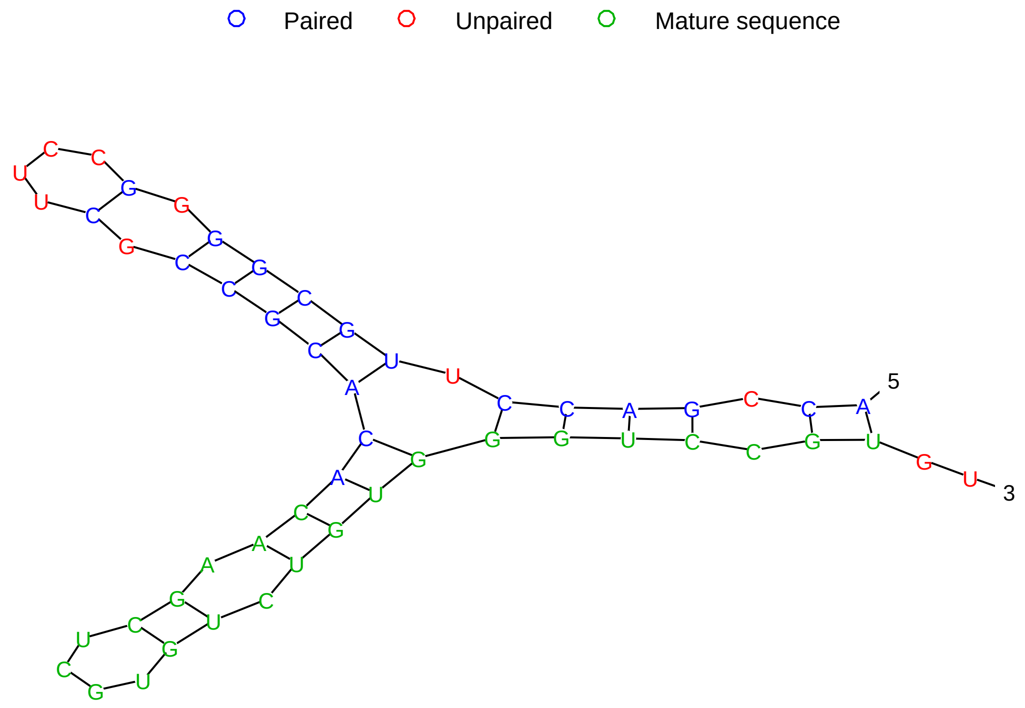

Similar miRNA: rno-miR-99b-3p
Stem loop (UMD3.1): chr18:58014884-58014936
Mature (UMD3.1): chr18:58014912-58014934
Mature seq len: 23
Total raw counts (9 samples): 1217
Average raw counts: 136
Strand: Forward
Orientation: 3p
Minimum free energy: -17.80

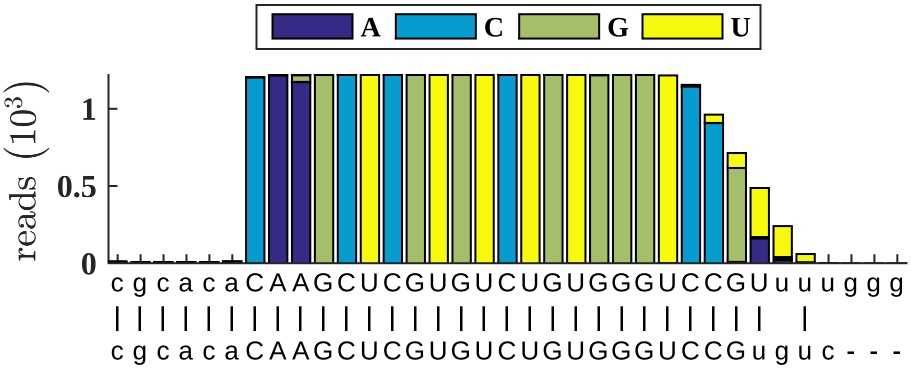

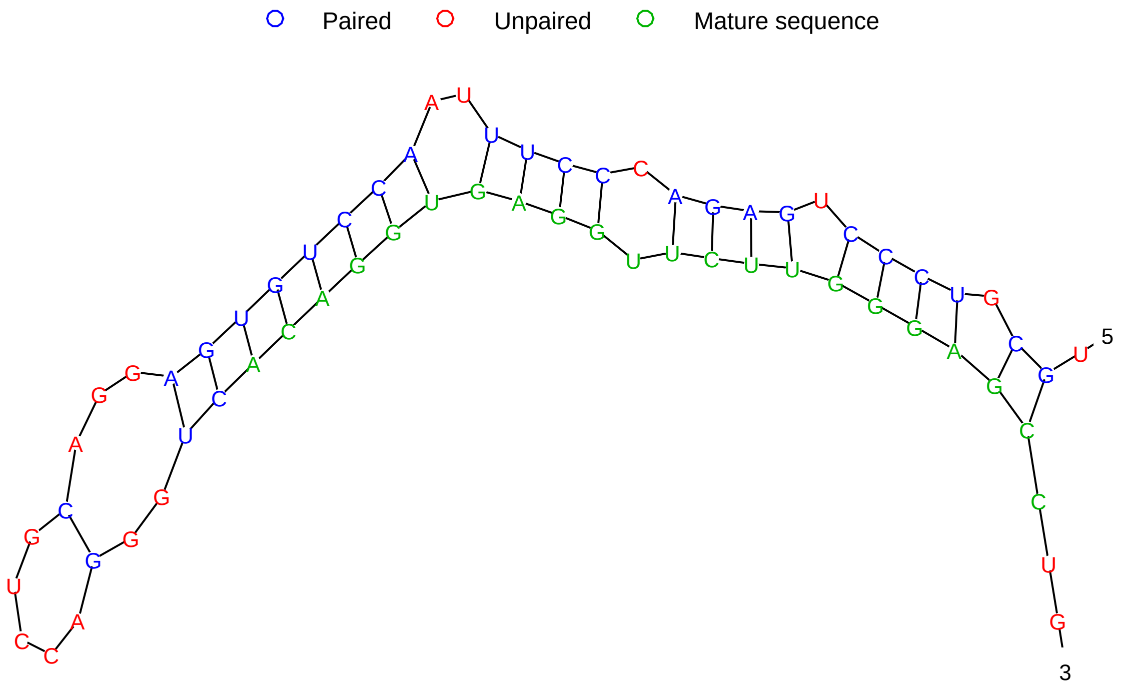

Similar miRNA: chi-miR-125a-3p
Stem loop (UMD3.1): chr18:58015545-58015610
Mature (UMD3.1): chr18:58015587-58015608
Mature seq len: 22
Total raw counts (9 samples): 1882
Average raw counts: 210
Strand: Forward
Orientation: 3p
Minimum free energy: -28.20

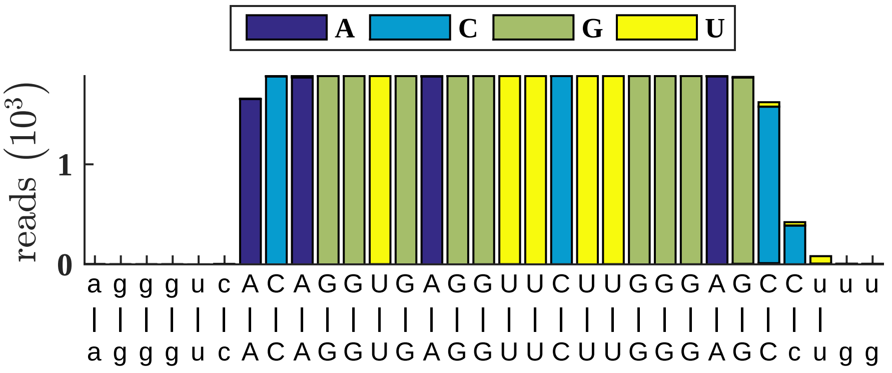

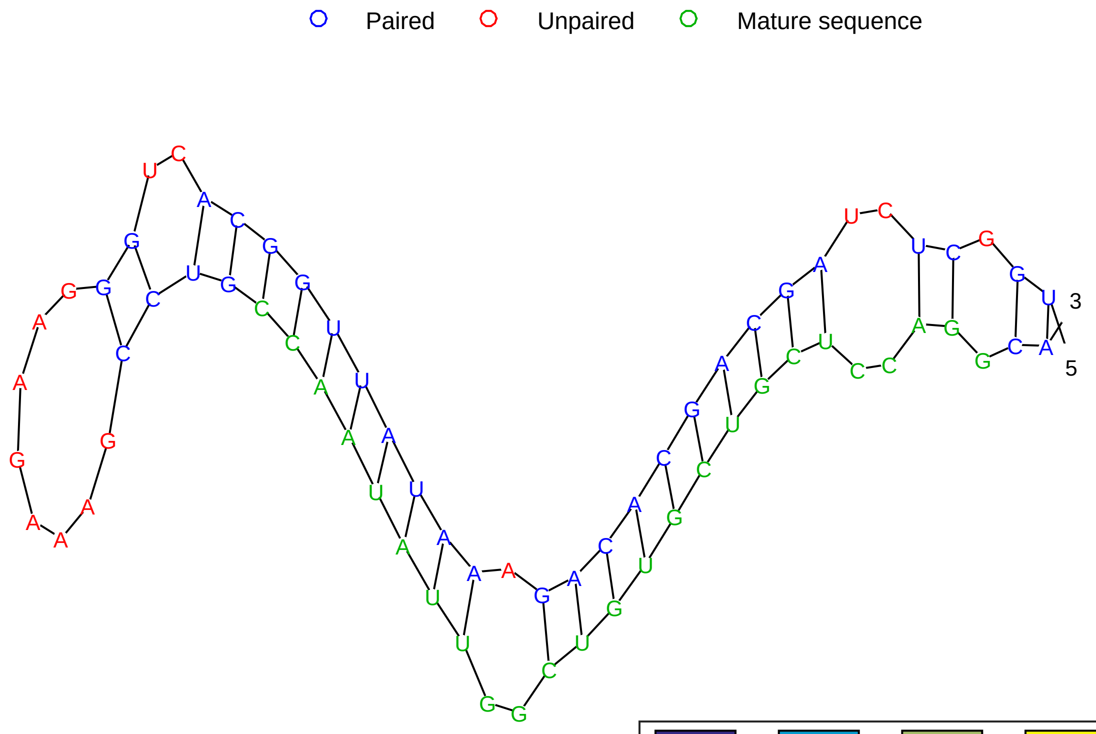

Similar miRNA: chi-miR-195-3p
Stem loop (UMD3.1): chr19:27441351-27441421
Mature (UMD3.1): chr19:27441353-27441377
Mature seq len: 25
Total raw counts (9 samples): 2751
Average raw counts: 306
Strand: Reverse
Orientation: 3p
Minimum free energy: -36.10

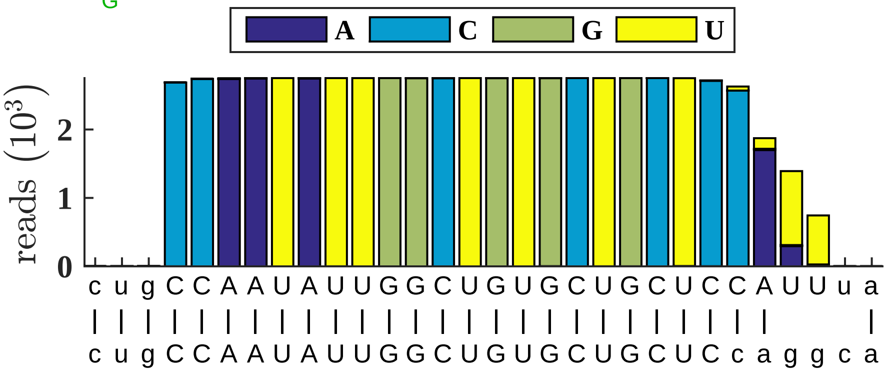

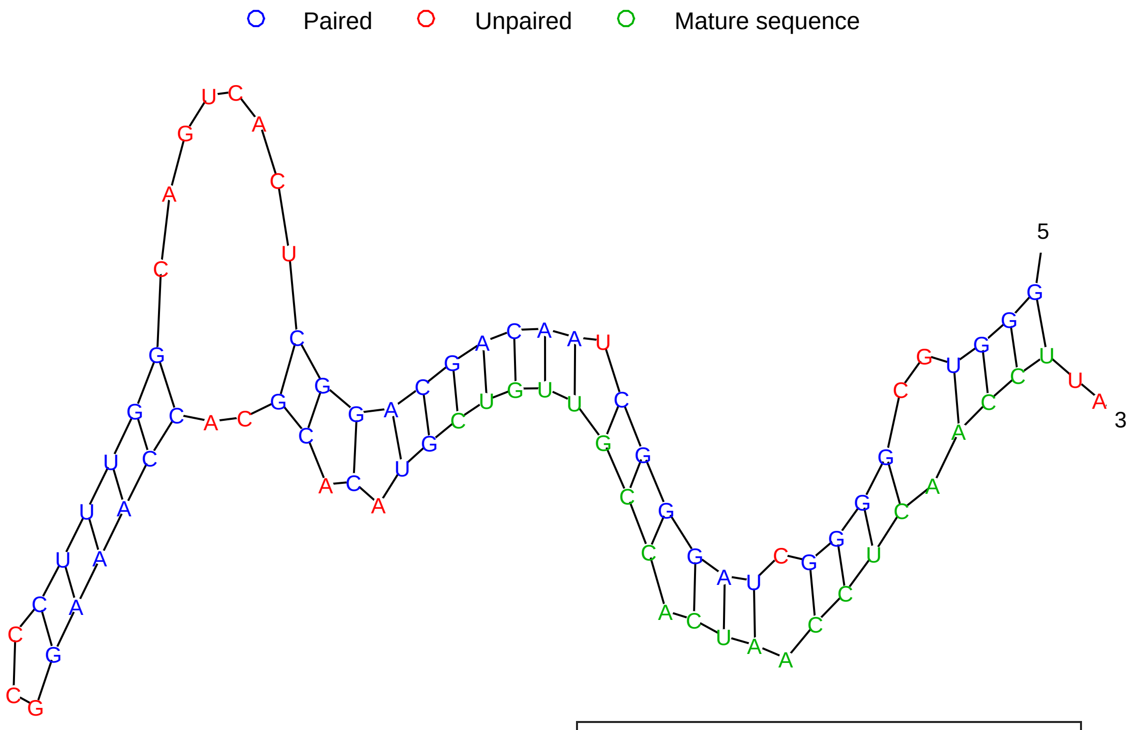

Similar miRNA: hsa-miR-744-3p
Stem loop (UMD3.1): chr19:31333521-31333604
Mature (UMD3.1): chr19:31333581-31333602
Mature seq len: 22
Total raw counts (9 samples): 498
Average raw counts: 56
Strand: Forward
Orientation: 3p
Minimum free energy: -31.70

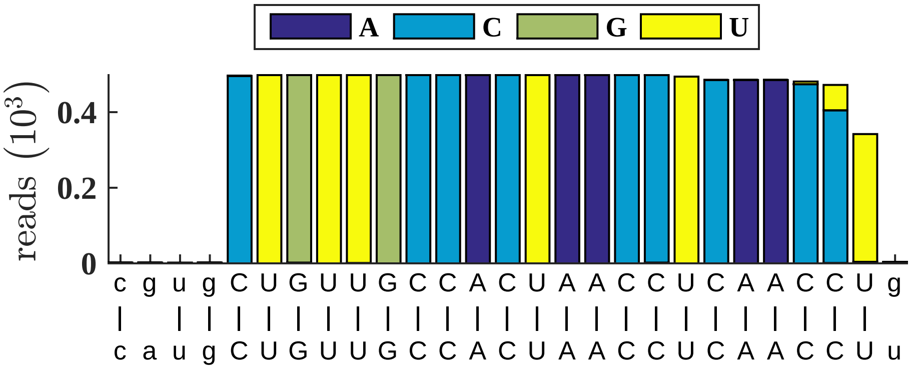

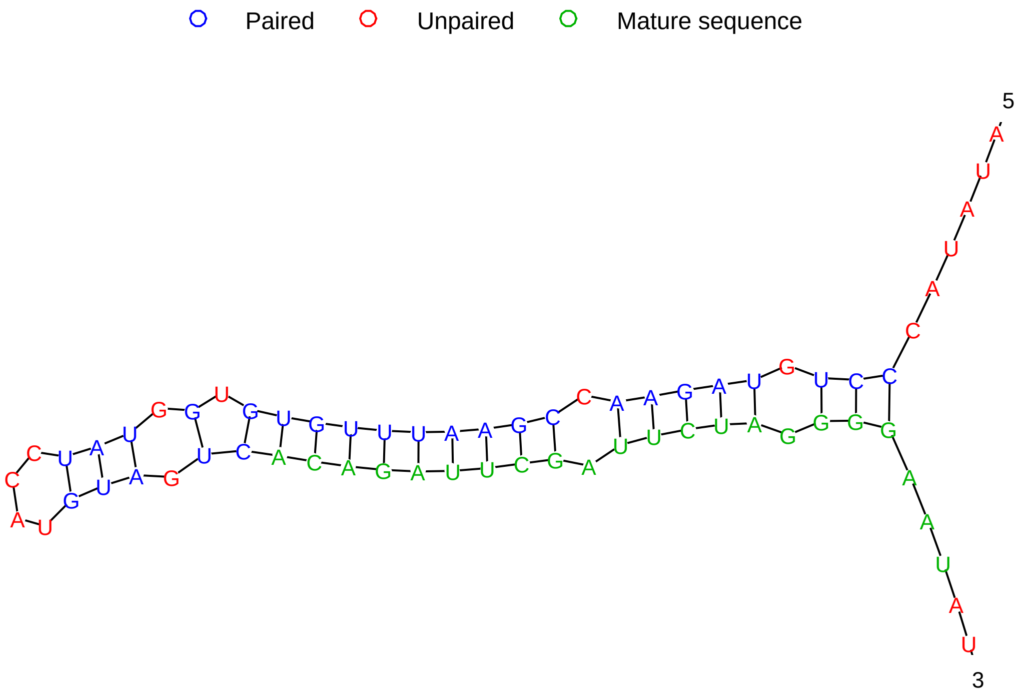

Similar miRNA: chi-miR-10b-3p
Stem loop (UMD3.1): chr2:20797623-20797688
Mature (UMD3.1): chr2:20797625-20797646
Mature seq len: 22
Total raw counts (9 samples): 435
Average raw counts: 49
Strand: Reverse
Orientation: 3p
Minimum free energy: -23.70

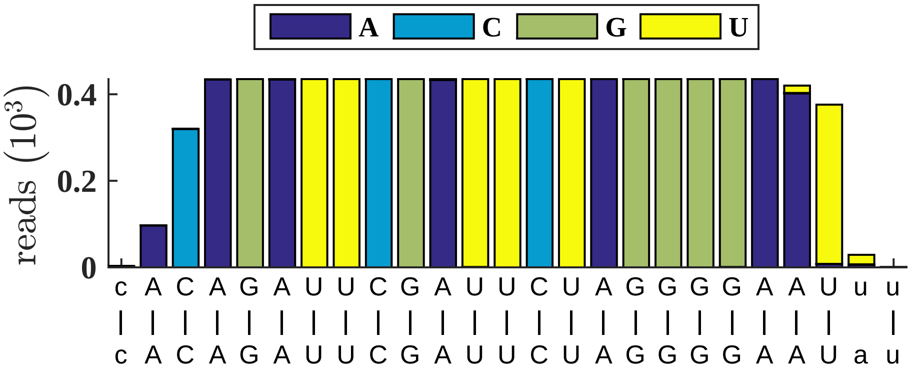

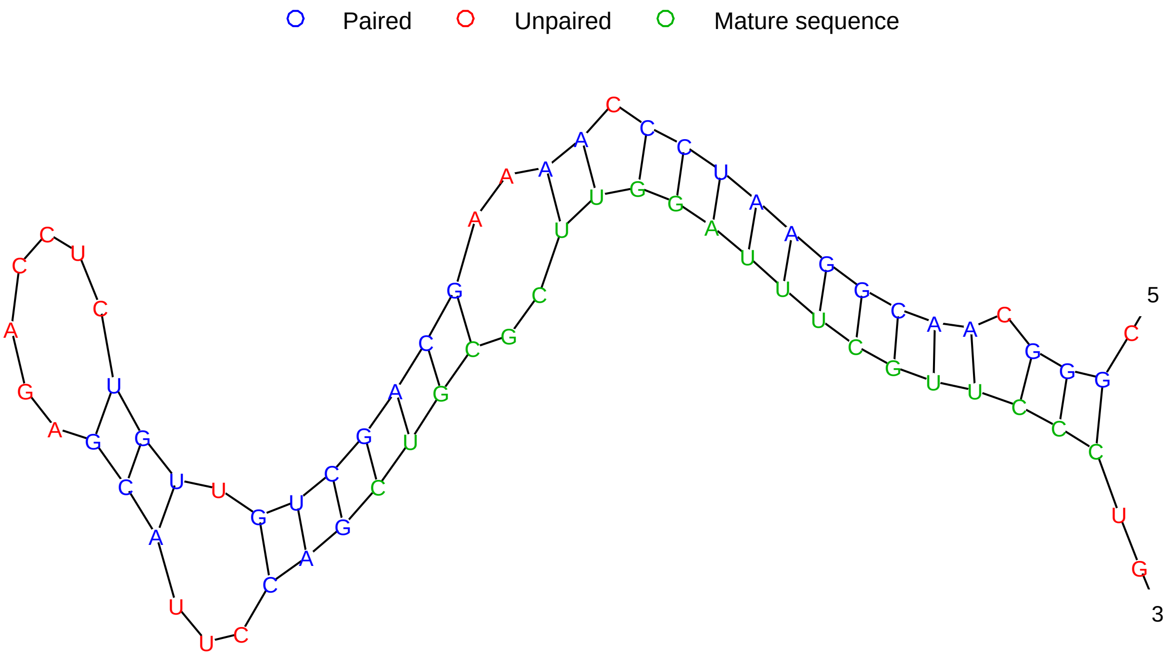

Similar miRNA: chi-miR-191-3p
Stem loop (UMD3.1): chr22:51543480-51543549
Mature (UMD3.1): chr22:51543527-51543547
Mature seq len: 21
Total raw counts (9 samples): 1437
Average raw counts: 160
Strand: Forward
Orientation: 3p
Minimum free energy: -31.90

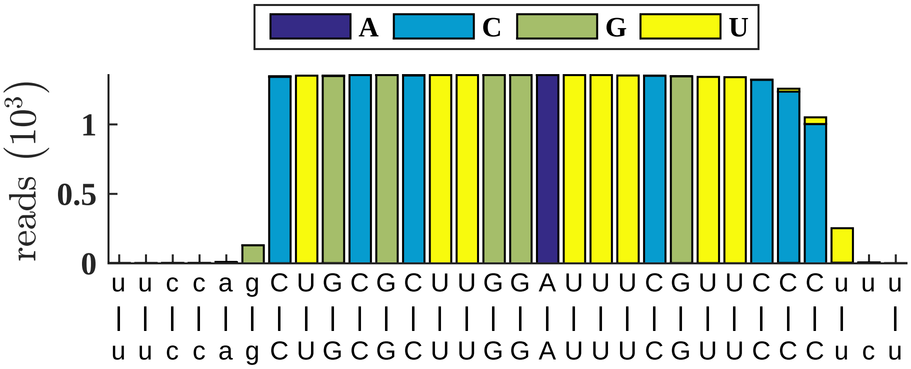

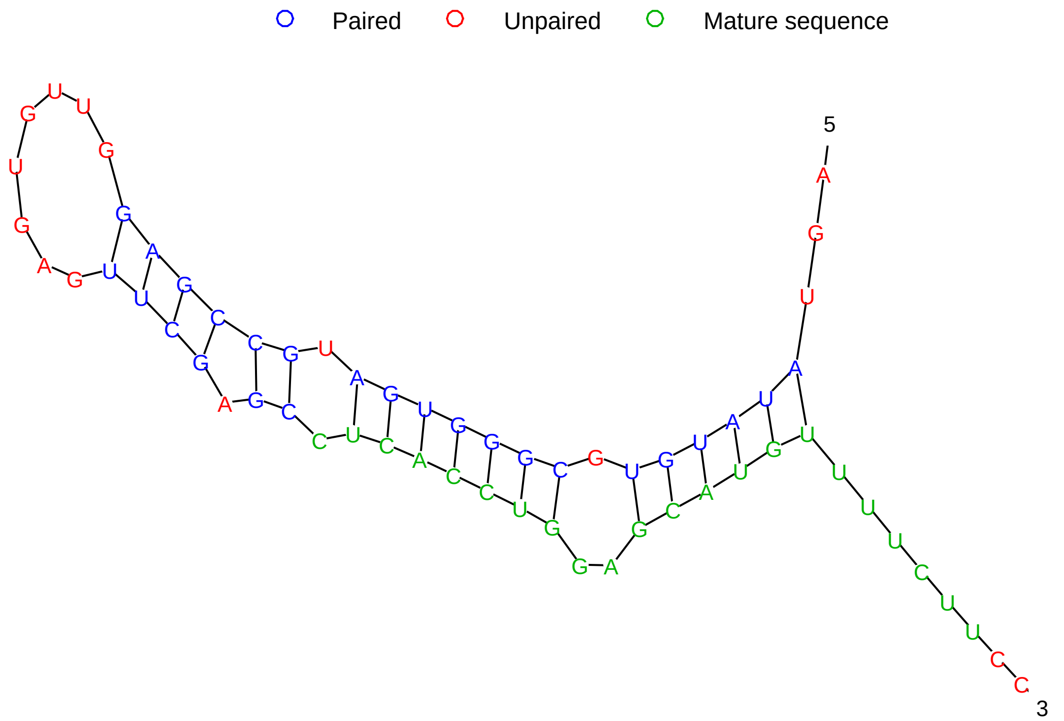

Similar miRNA: mmu-miR-1983
Stem loop (UMD3.1): chr23:30835087-30835149
Mature (UMD3.1): chr23:30835089-30835110
Mature seq len: 22
Total raw counts (9 samples): 465
Average raw counts: 52
Strand: Reverse
Orientation: 3p
Minimum free energy: -22.40

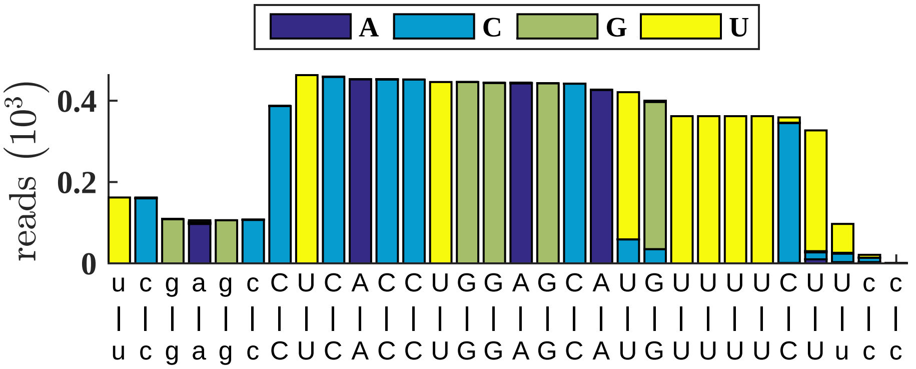

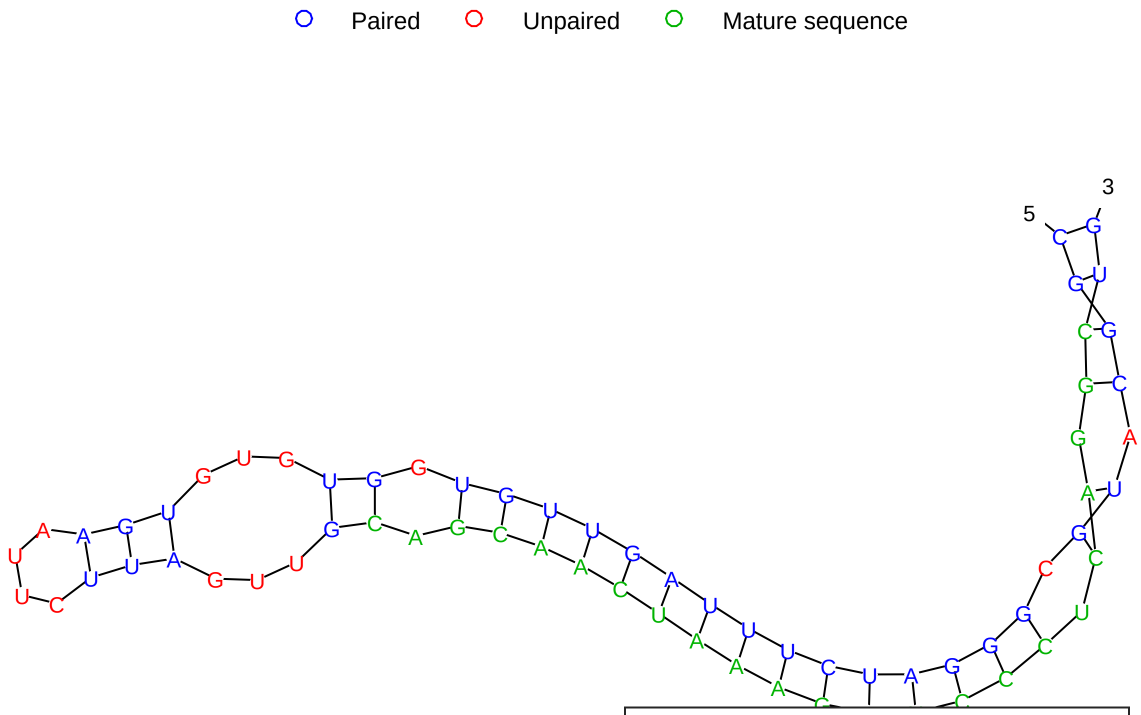

Similar miRNA: chi-miR-3432-3p
Stem loop (UMD3.1): chr25:34409295-34409362
Mature (UMD3.1): chr25:34409297-34409319
Mature seq len: 23
Total raw counts (9 samples): 517
Average raw counts: 58
Strand: Reverse
Orientation: 3p
Minimum free energy: -31.40

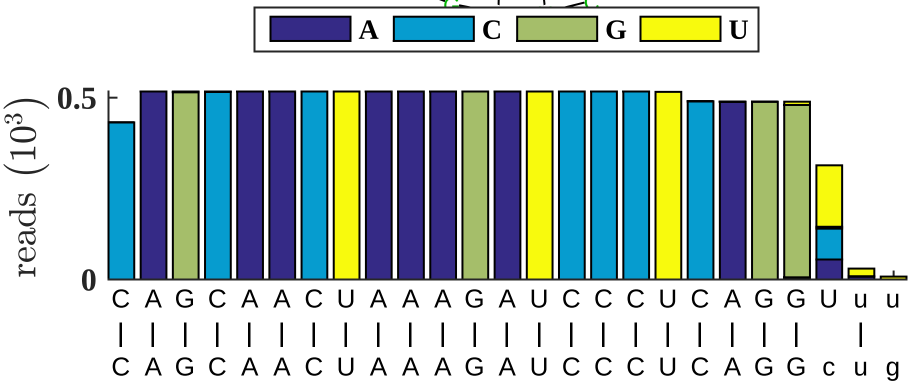

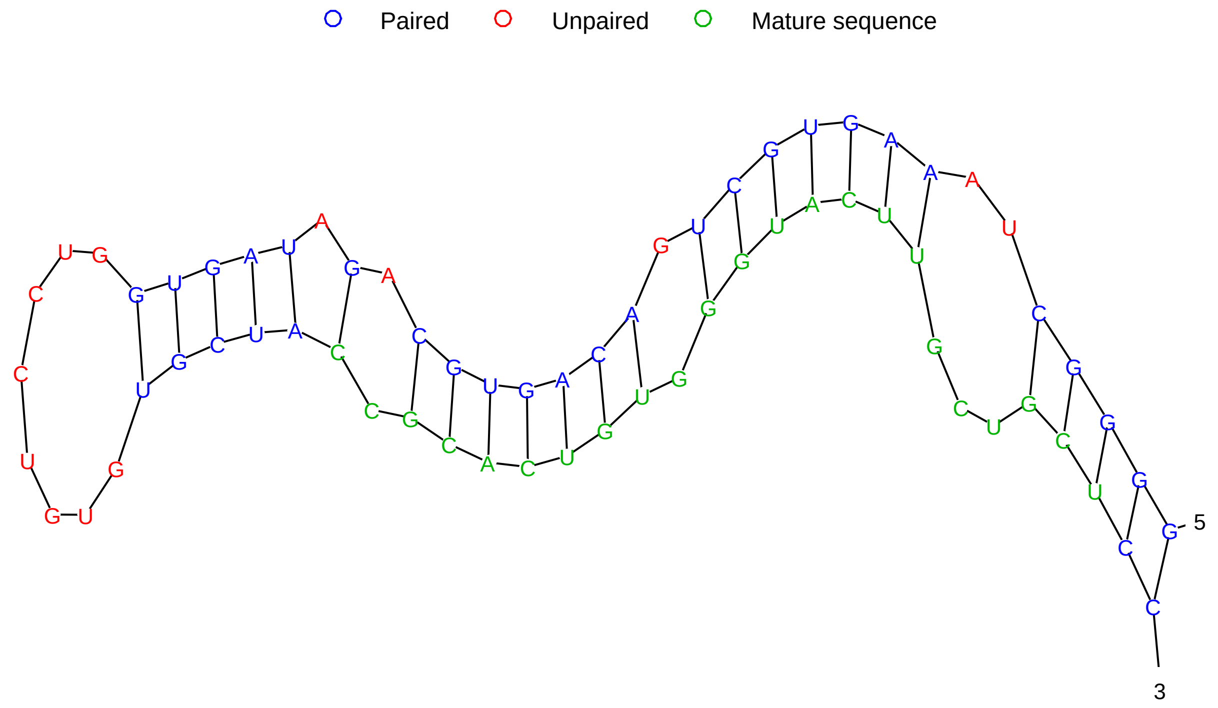

Similar miRNA: chi-miR-106b-3p
Stem loop (UMD3.1): chr25:36892052-36892119
Mature (UMD3.1): chr25:36892095-36892117
Mature seq len: 23
Total raw counts (9 samples): 4725
Average raw counts: 525
Strand: Forward
Orientation: 3p
Minimum free energy: -33.10

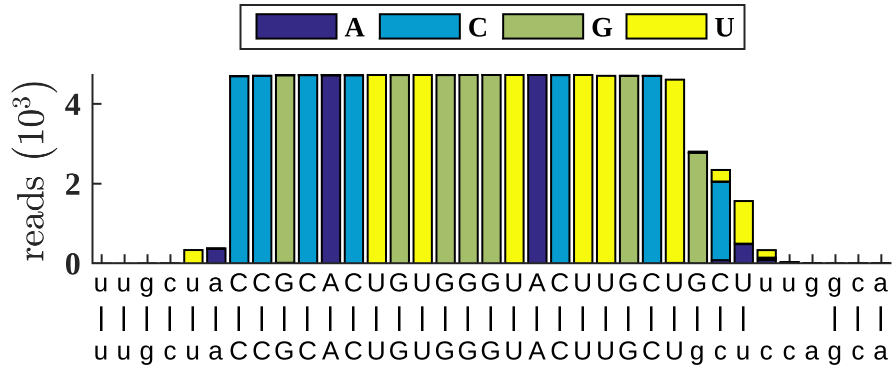

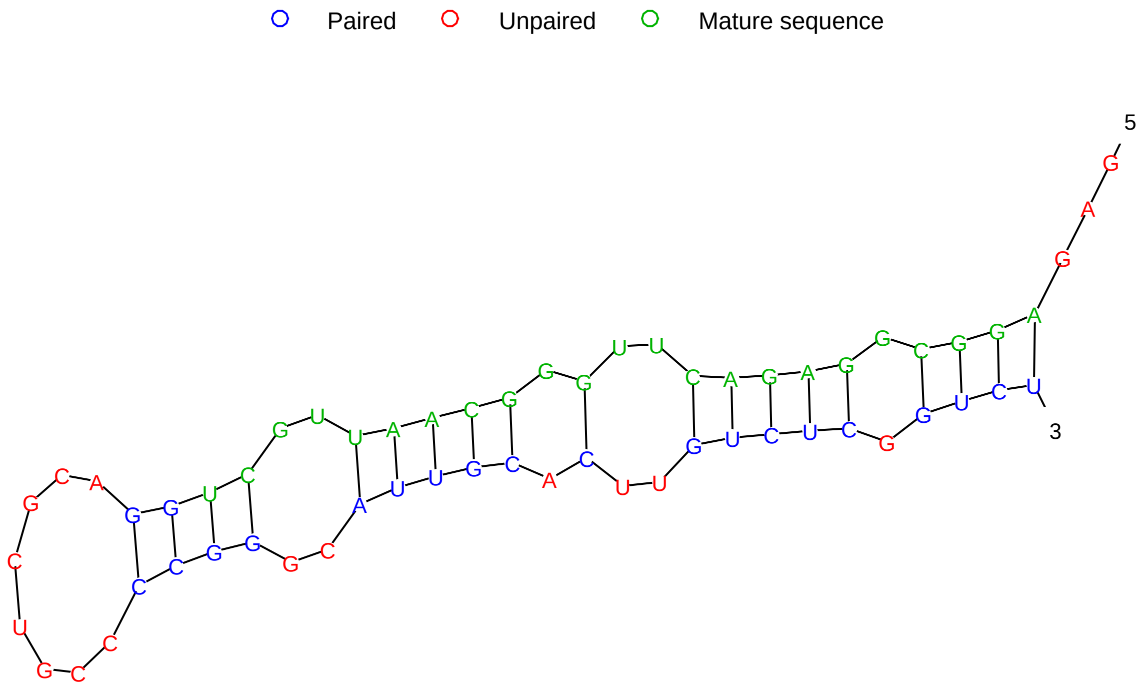

Similar miRNA: chi-miR-25-5p
Stem loop (UMD3.1): chr25:36892459-36892519
Mature (UMD3.1): chr25:36892462-36892484
Mature seq len: 23
Total raw counts (9 samples): 538
Average raw counts: 60
Strand: Forward
Orientation: 5p
Minimum free energy: -26.20

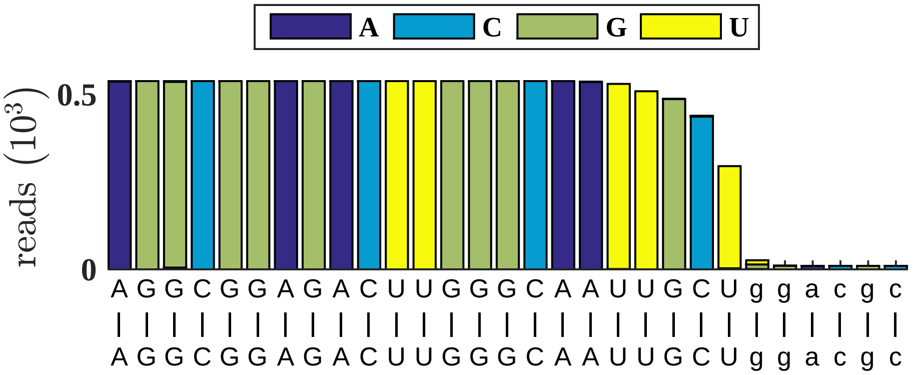

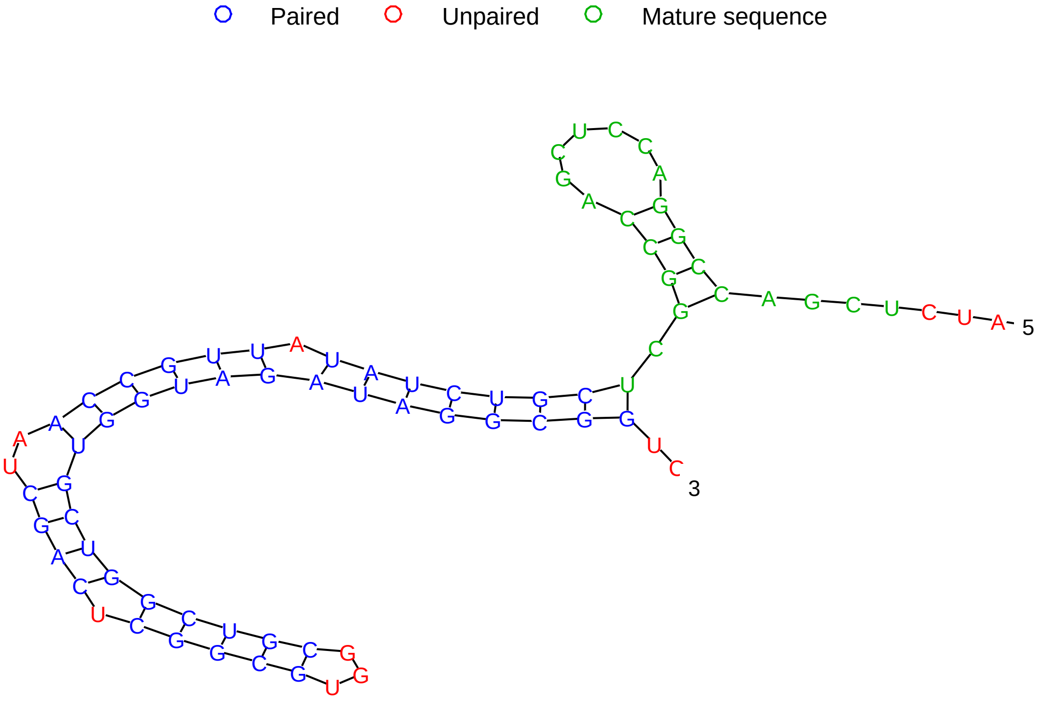

Similar miRNA: chi-miR-1307-5p
Stem loop (UMD3.1): chr26:24230105-24230182
Mature (UMD3.1): chr26:24230159-24230179
Mature seq len: 21
Total raw counts (9 samples): 12127
Average raw counts: 1348
Strand: Reverse
Orientation: 5p
Minimum free energy: -31.50

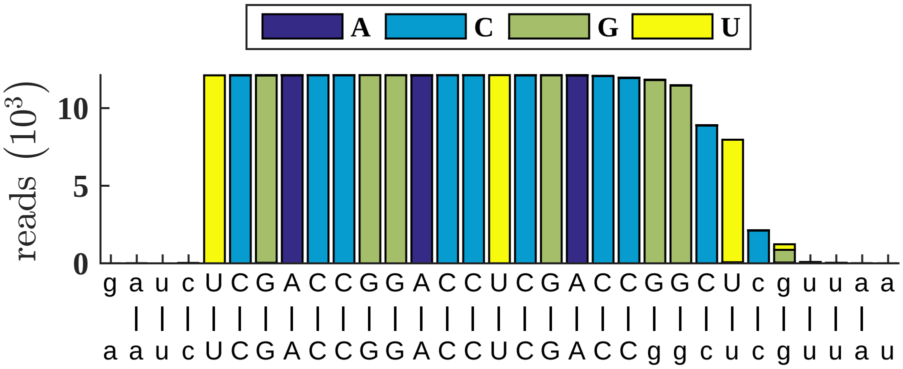

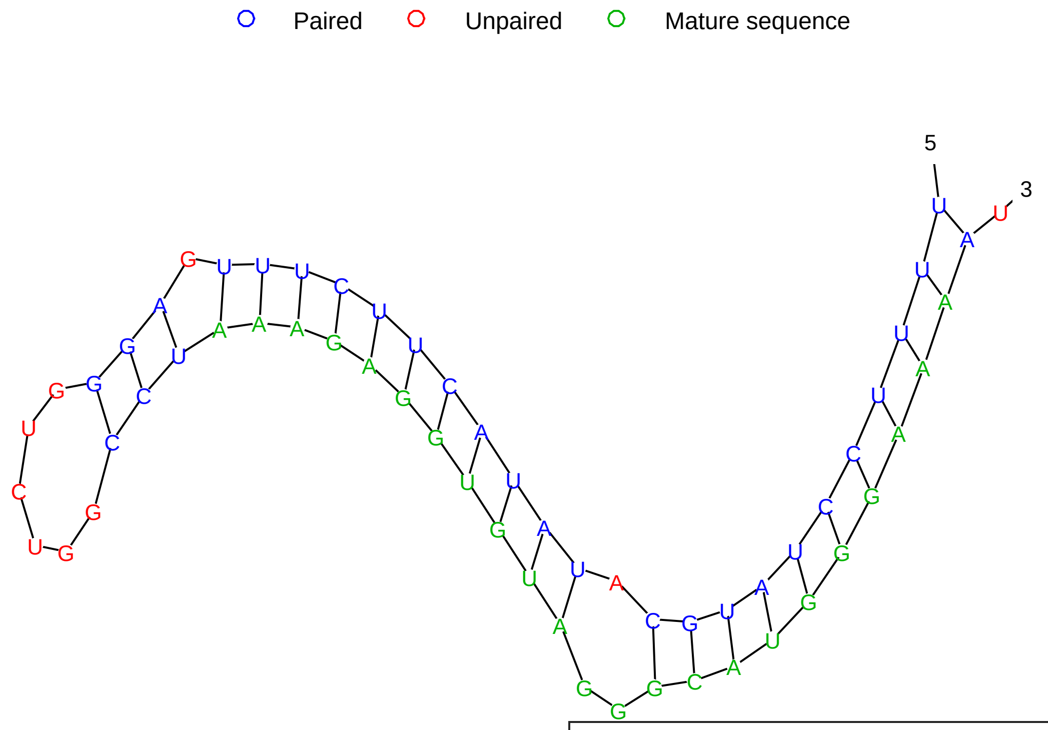

Similar miRNA: chi-miR-202-3p
Stem loop (UMD3.1): chr26:25988256-25988316
Mature (UMD3.1): chr26:25988292-25988314
Mature seq len: 23
Total raw counts (9 samples): 161713
Average raw counts: 17969
Strand: Forward
Orientation: 3p
Minimum free energy: -30.90

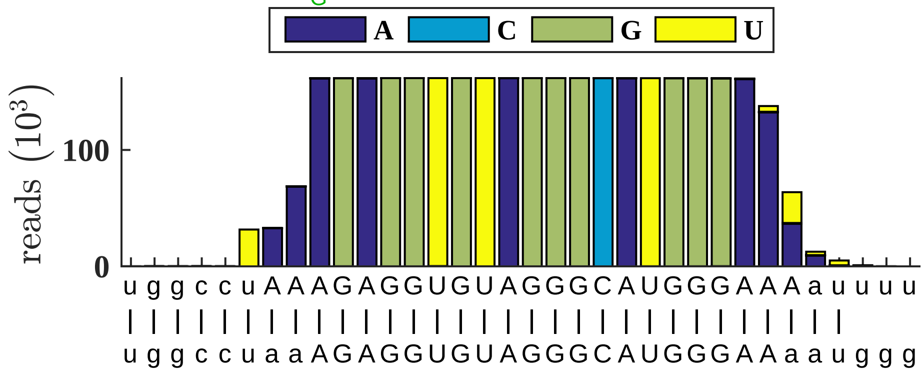

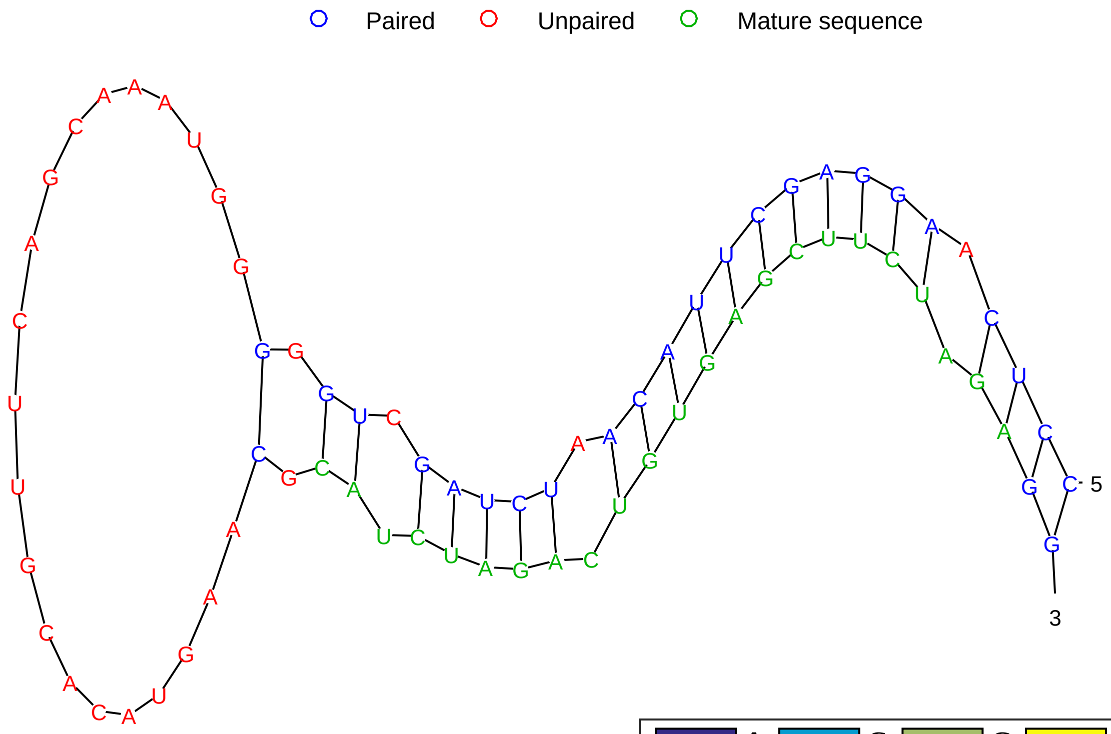

Similar miRNA: chi-miR-708-3p
Stem loop (UMD3.1): chr29:16686246-16686320
Mature (UMD3.1): chr29:16686296-16686318
Mature seq len: 23
Total raw counts (9 samples): 414
Average raw counts: 46
Strand: Forward
Orientation: 3p
Minimum free energy: -30.00

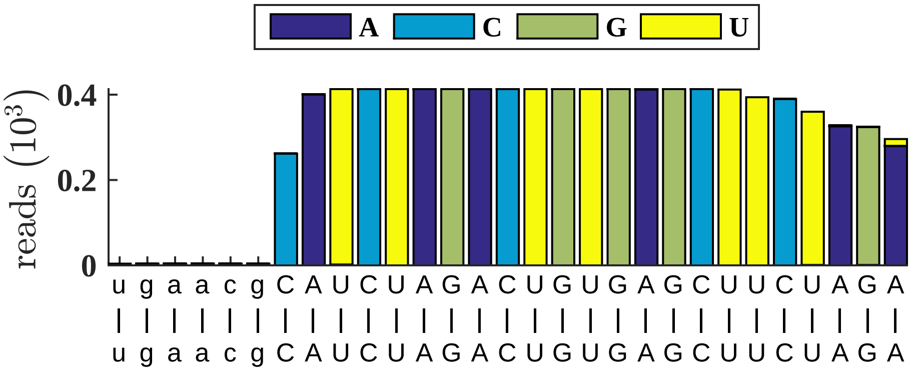

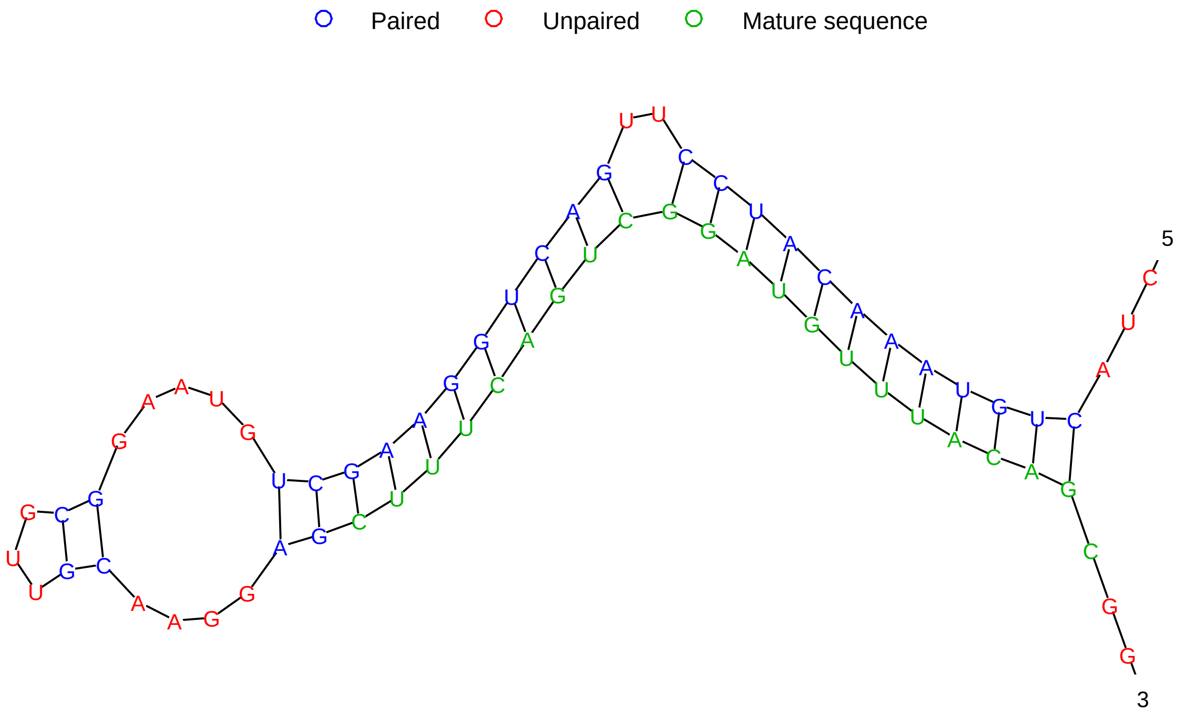

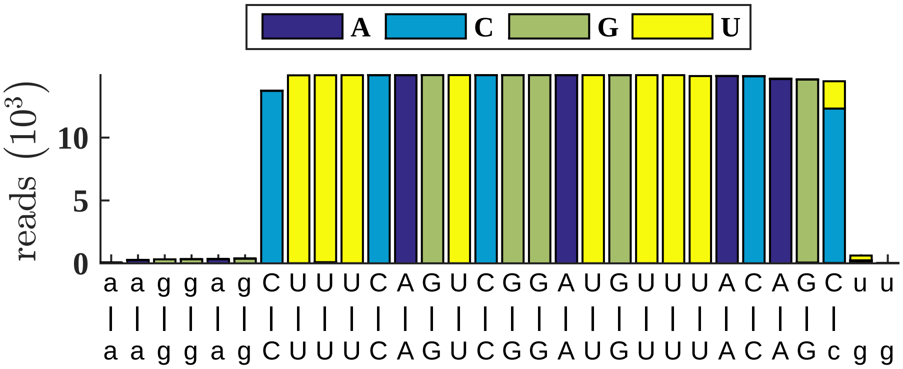

Similar miRNA: chi-miR-30e-3p
Stem loop (UMD3.1): chr3:106062502-106062571
Mature (UMD3.1): chr3:106062504-106062525
Mature seq len: 22
Total raw counts (9 samples): 14977
Average raw counts: 1665
Strand: Reverse
Orientation: 3p
Minimum free energy: -35.10

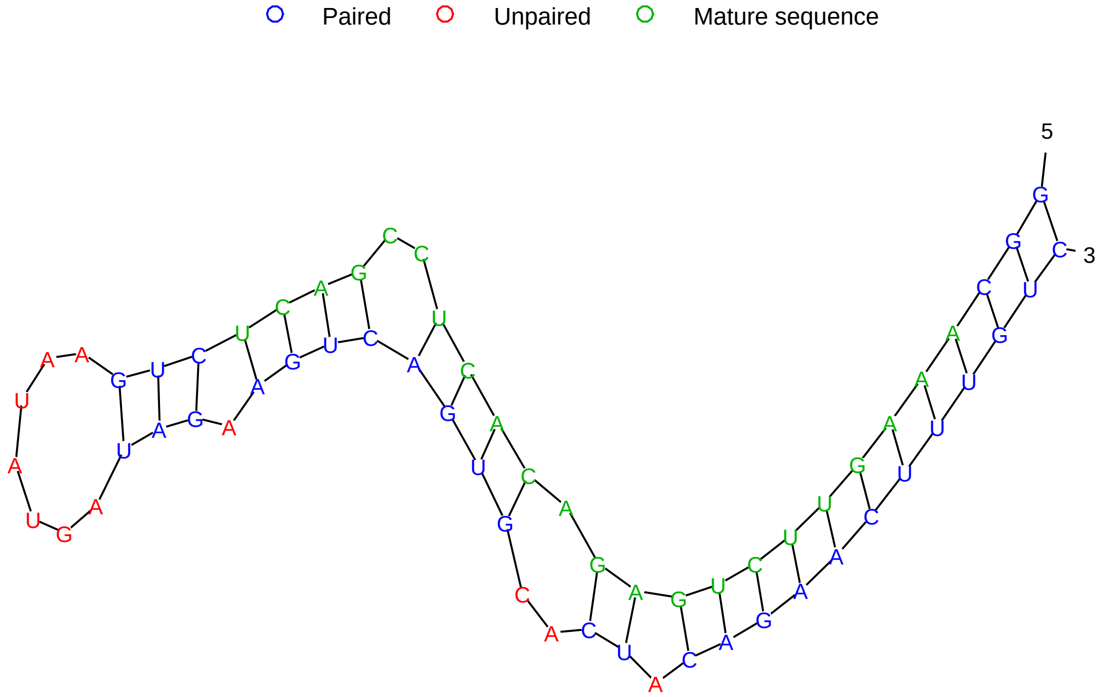

Similar miRNA: chi-miR-148a-5p
Stem loop (UMD3.1): chr4:70414253-70414316
Mature (UMD3.1): chr4:70414256-70414277
Mature seq len: 22
Total raw counts (9 samples): 2685
Average raw counts: 299
Strand: Forward
Orientation: 5p
Minimum free energy: -23.40

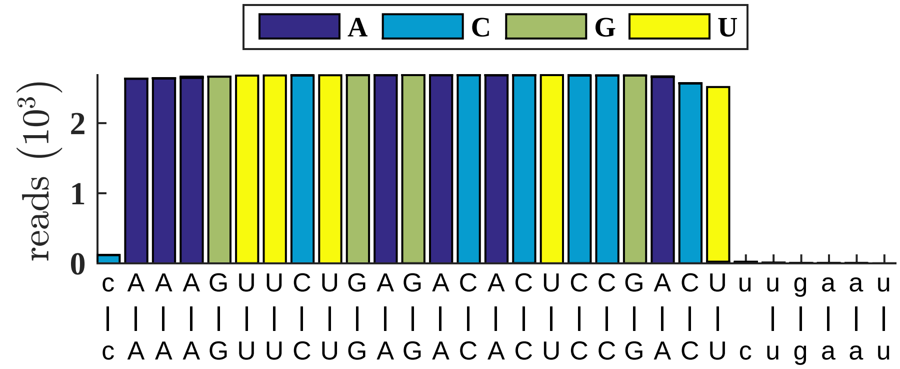

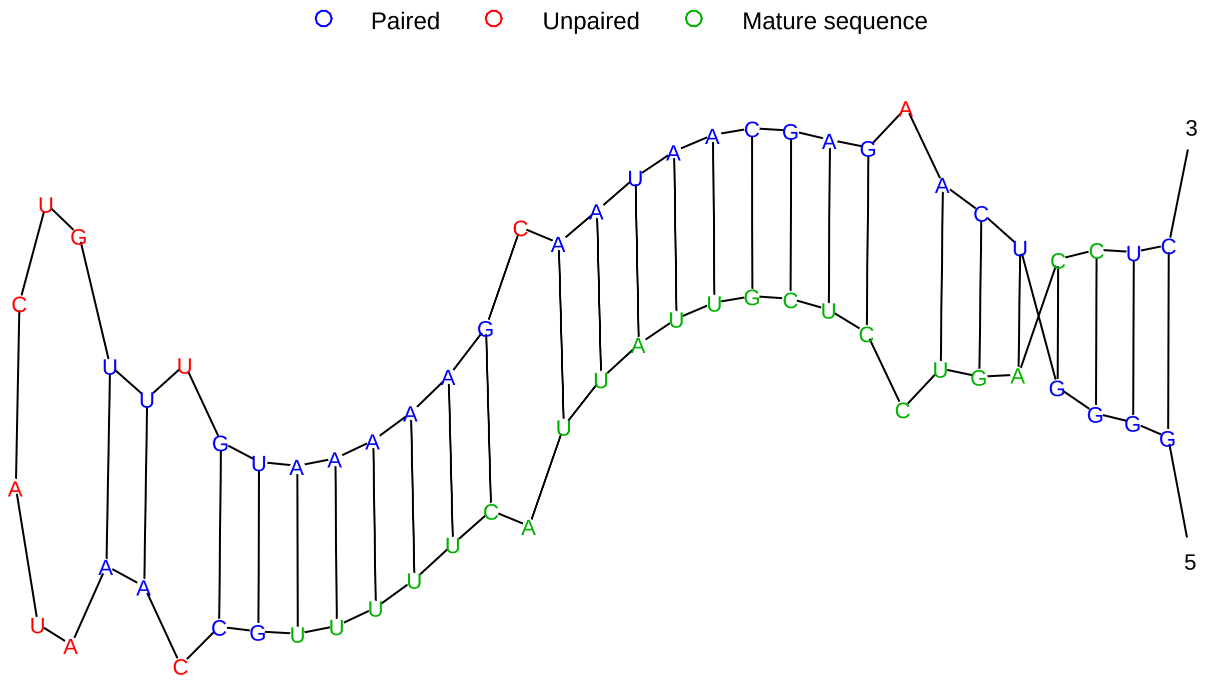

Similar miRNA: chi-miR-335-3p
Stem loop (UMD3.1): chr4:95071002-95071065
Mature (UMD3.1): chr4:95071042-95071063
Mature seq len: 22
Total raw counts (9 samples): 4413
Average raw counts: 491
Strand: Forward
Orientation: 3p
Minimum free energy: -30.00

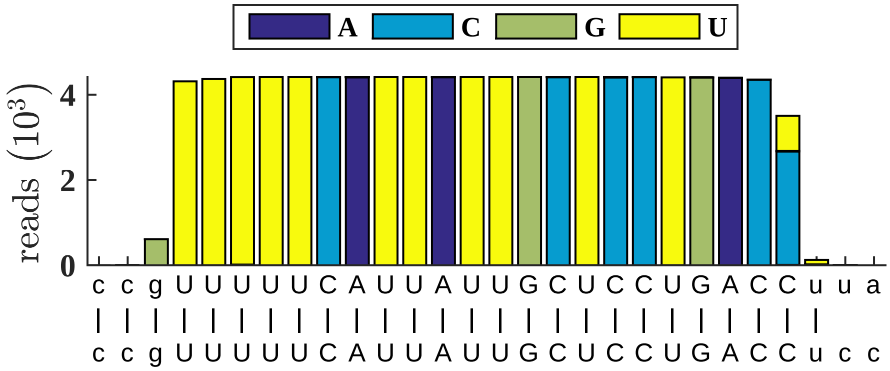

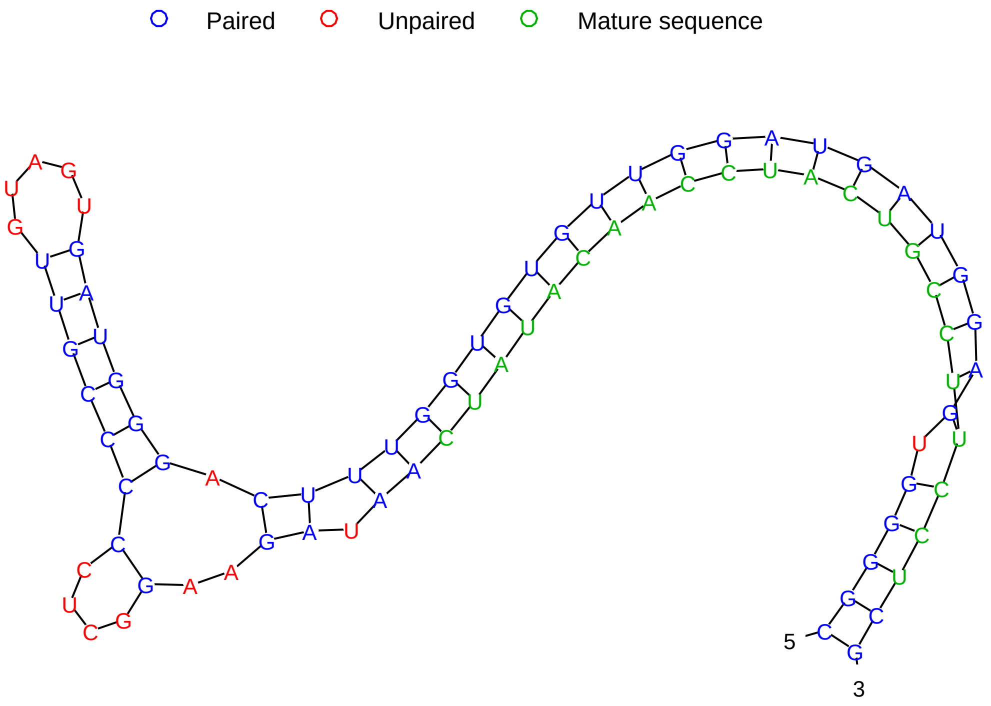

Similar miRNA: chi-let-7b-3p
Stem loop (UMD3.1): chr5:117120185-117120268
Mature (UMD3.1): chr5:117120245-117120266
Mature seq len: 22
Total raw counts (9 samples): 634
Average raw counts: 71
Strand: Forward
Orientation: 3p
Minimum free energy: -43.70

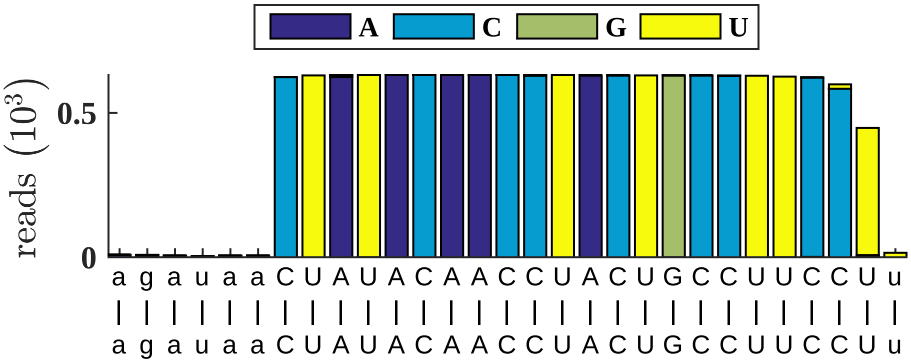

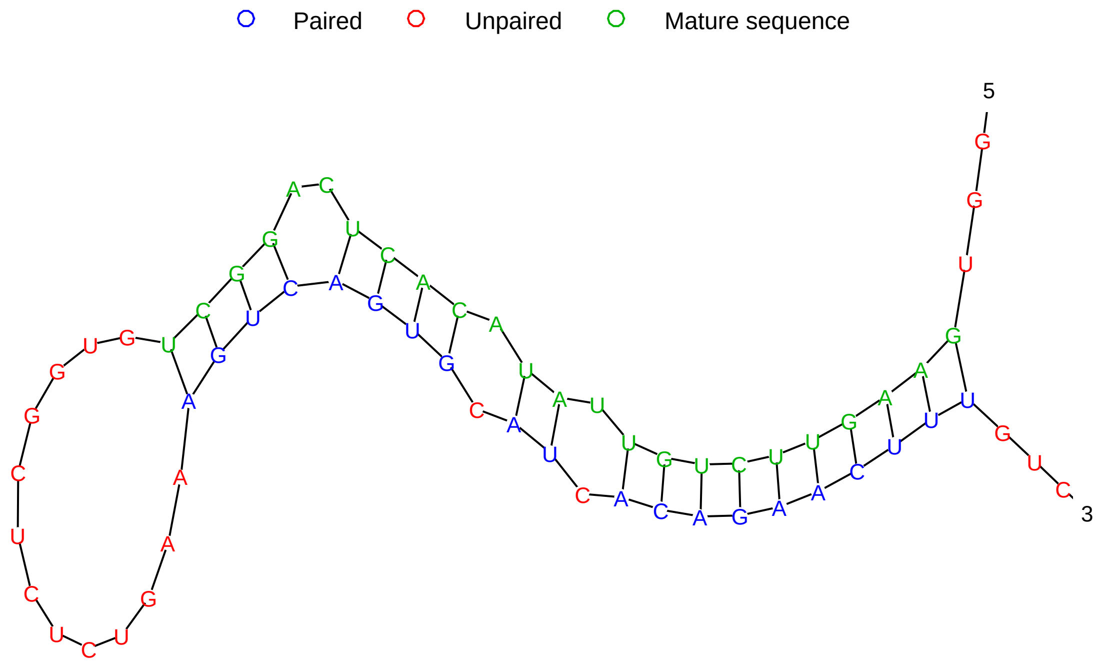

Similar miRNA: ssc-miR-148b-5p
Stem loop (UMD3.1): chr5:25849778-25849842
Mature (UMD3.1): chr5:25849816-25849839
Mature seq len: 24
Total raw counts (9 samples): 2105
Average raw counts: 234
Strand: Reverse
Orientation: 5p
Minimum free energy: -18.40

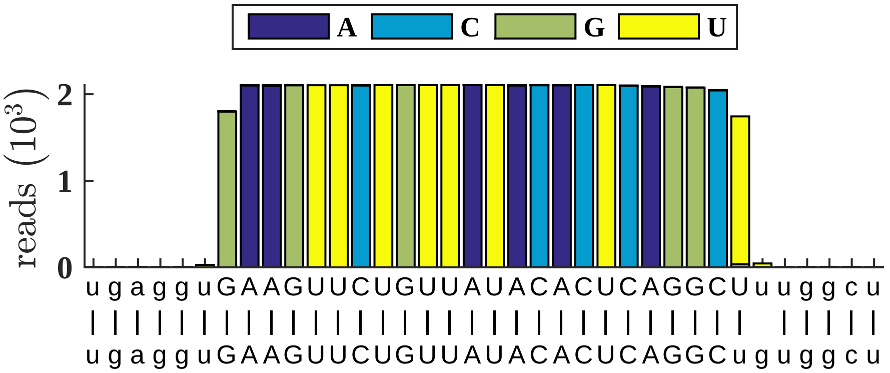

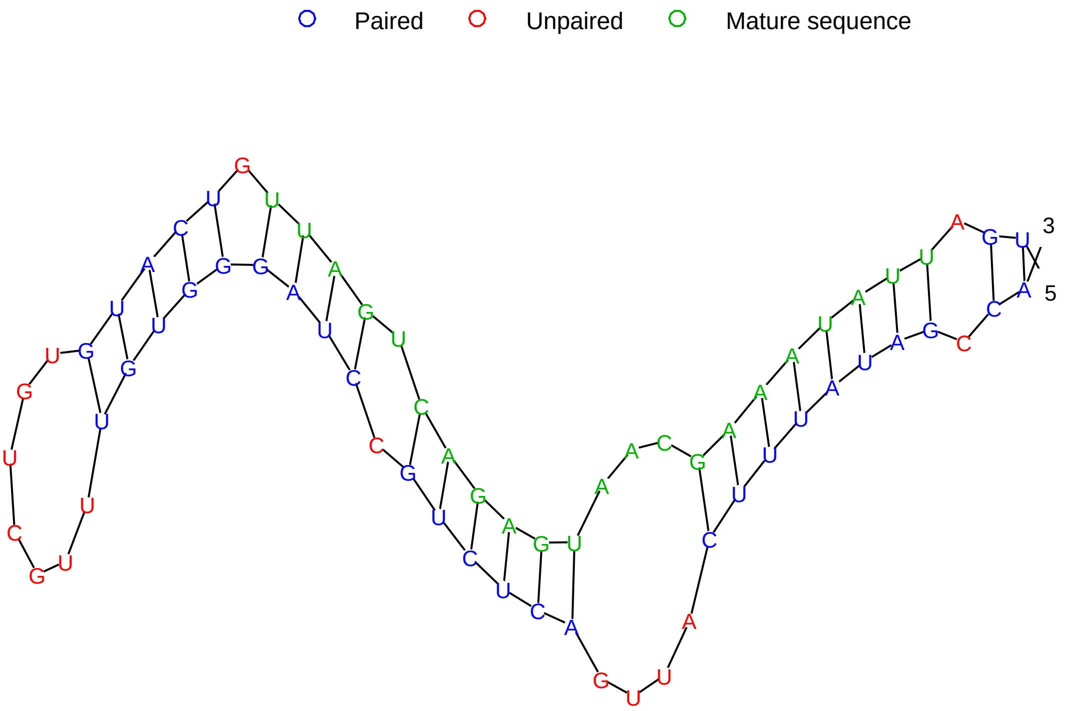

Similar miRNA: chi-miR-340-5p
Stem loop (UMD3.1): chr7:1147000-1147068
Mature (UMD3.1): chr7:1147003-1147024
Mature seq len: 22
Total raw counts (9 samples): 2130
Average raw counts: 237
Strand: Forward
Orientation: 5p
Minimum free energy: -18.10

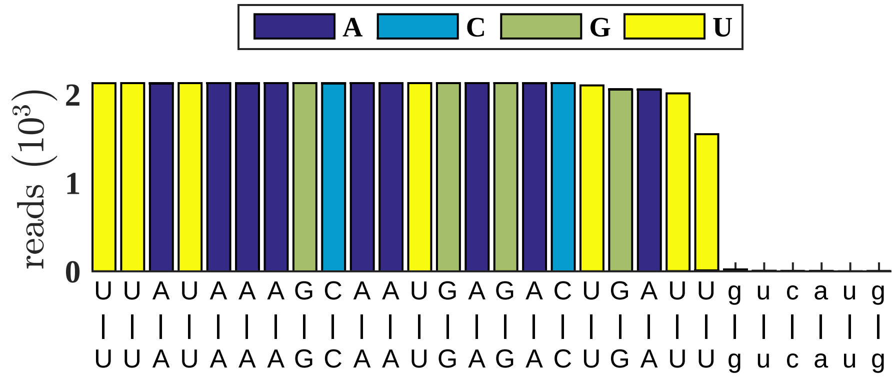

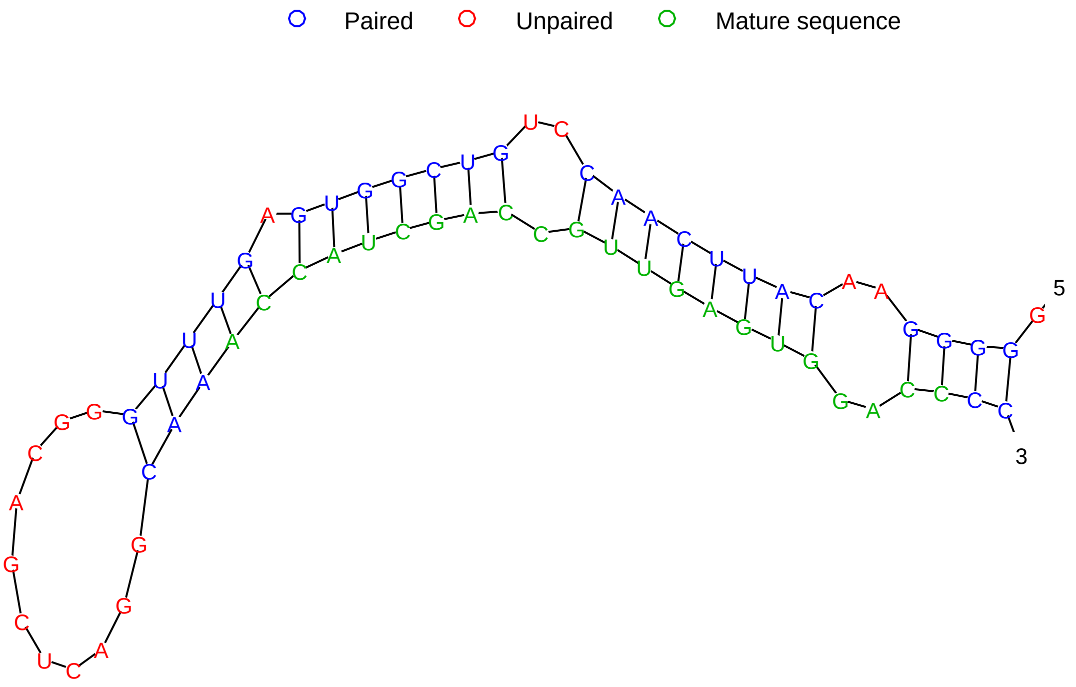

Similar miRNA: chi-miR-181c-3p
Stem loop (UMD3.1): chr7:12915085-12915152
Mature (UMD3.1): chr7:12915129-12915150
Mature seq len: 22
Total raw counts (9 samples): 394
Average raw counts: 44
Strand: Forward
Orientation: 3p
Minimum free energy: -33.00

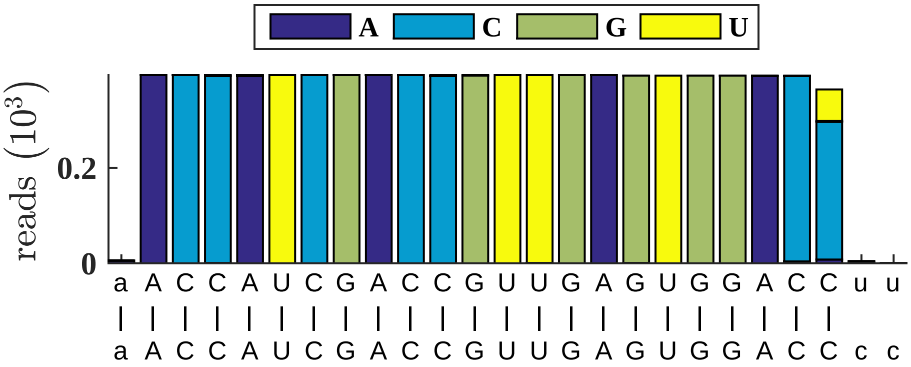

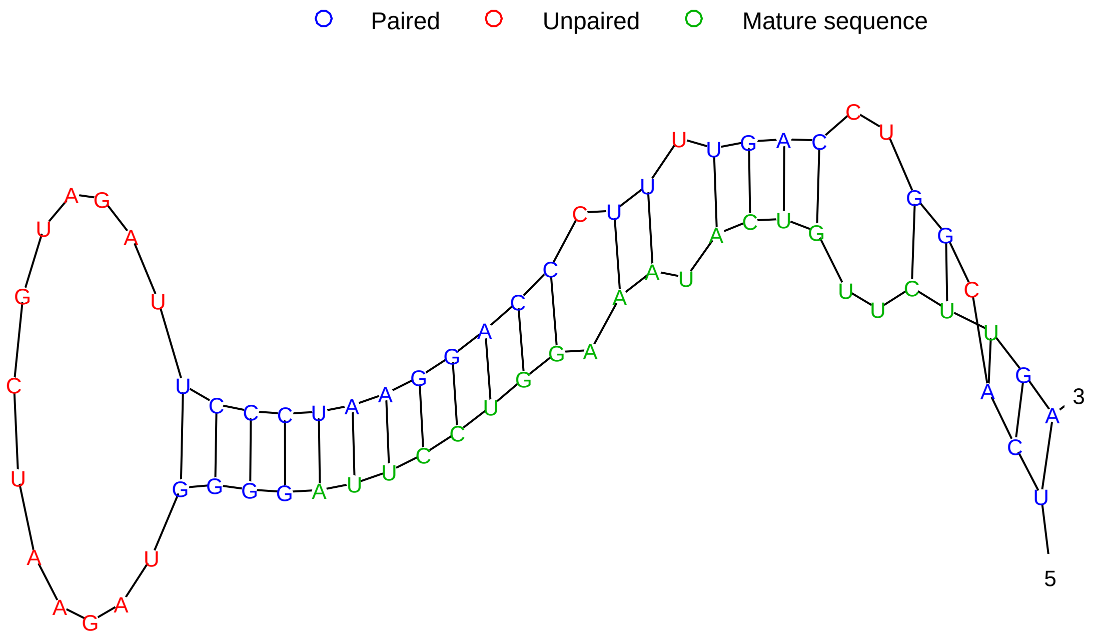

Similar miRNA: chi-miR-145-3p
Stem loop (UMD3.1): chr7:62810743-62810810
Mature (UMD3.1): chr7:62810788-62810808
Mature seq len: 21
Total raw counts (9 samples): 1290
Average raw counts: 144
Strand: Forward
Orientation: 3p
Minimum free energy: -27.70

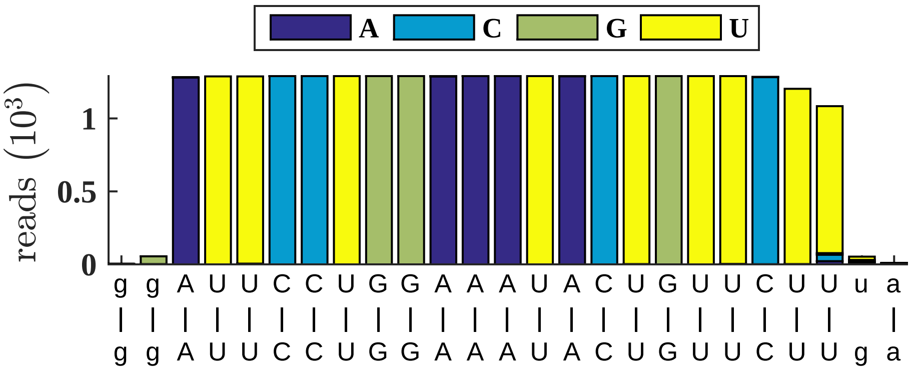

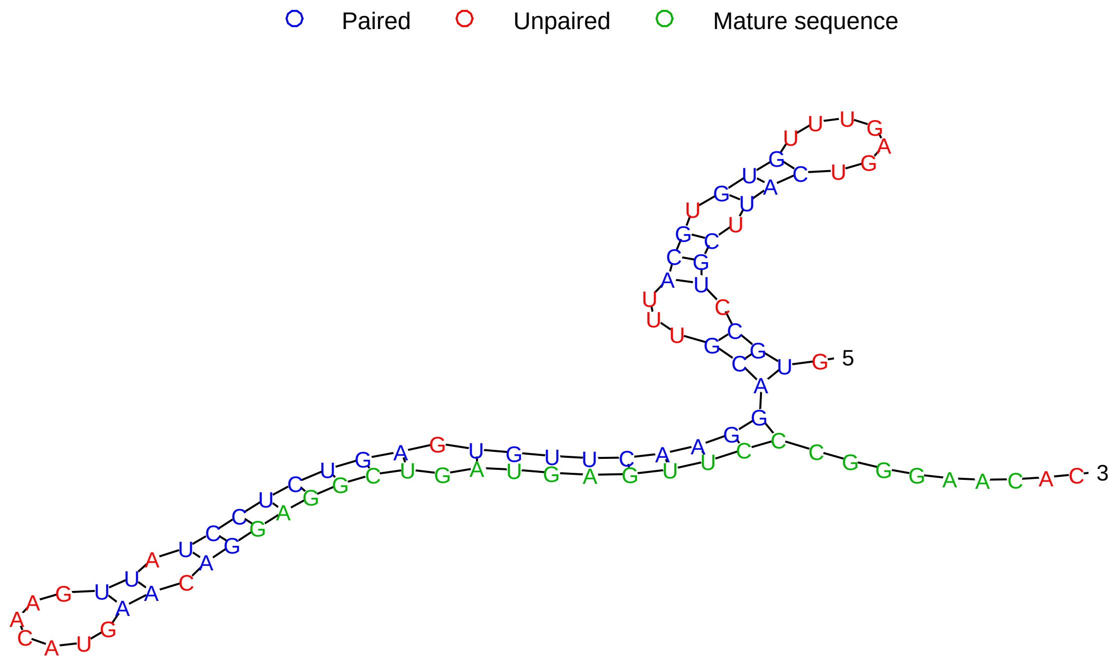

Similar miRNA: chi-miR-873-3p
Stem loop (UMD3.1): chr8:15159541-15159630
Mature (UMD3.1): chr8:15159606-15159628
Mature seq len: 23
Total raw counts (9 samples): 6384
Average raw counts: 710
Strand: Forward
Orientation: 3p
Minimum free energy: -32.00

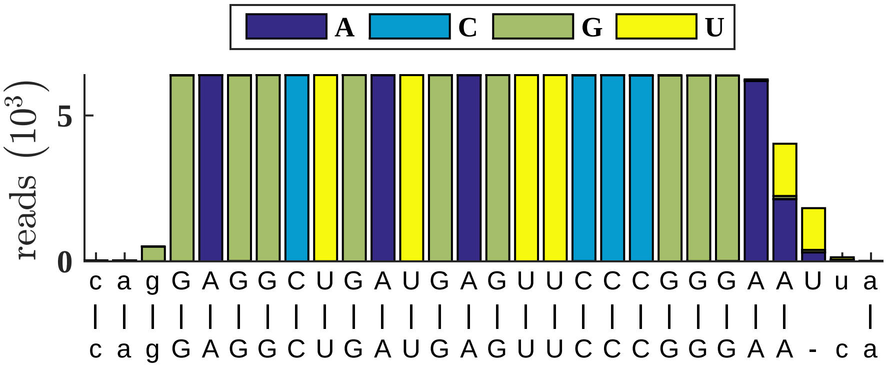

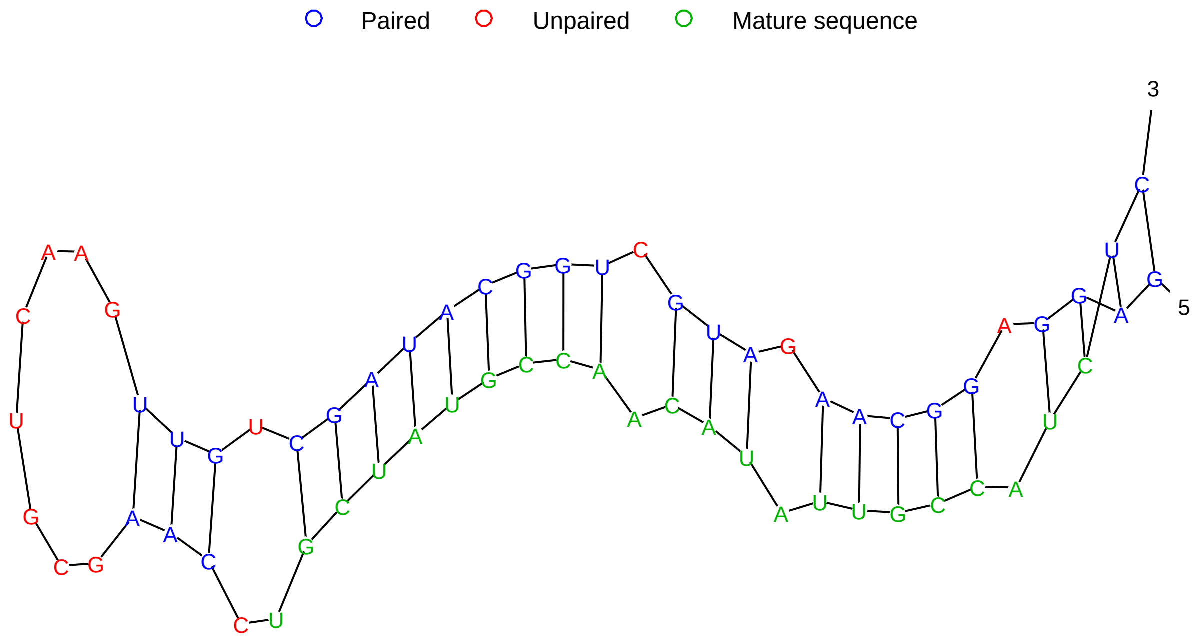

Similar miRNA: mml-miR-31-3p
Stem loop (UMD3.1): chr8:22534817-22534881
Mature (UMD3.1): chr8:22534857-22534879
Mature seq len: 23
Total raw counts (9 samples): 478
Average raw counts: 54
Strand: Forward
Orientation: 3p
Minimum free energy: -31.60

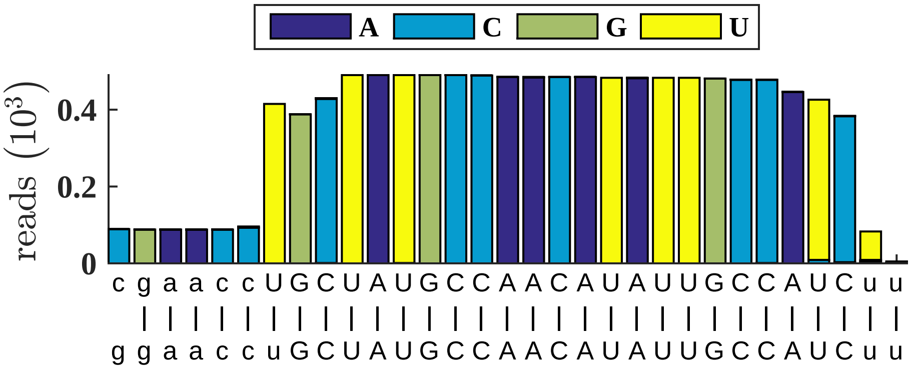

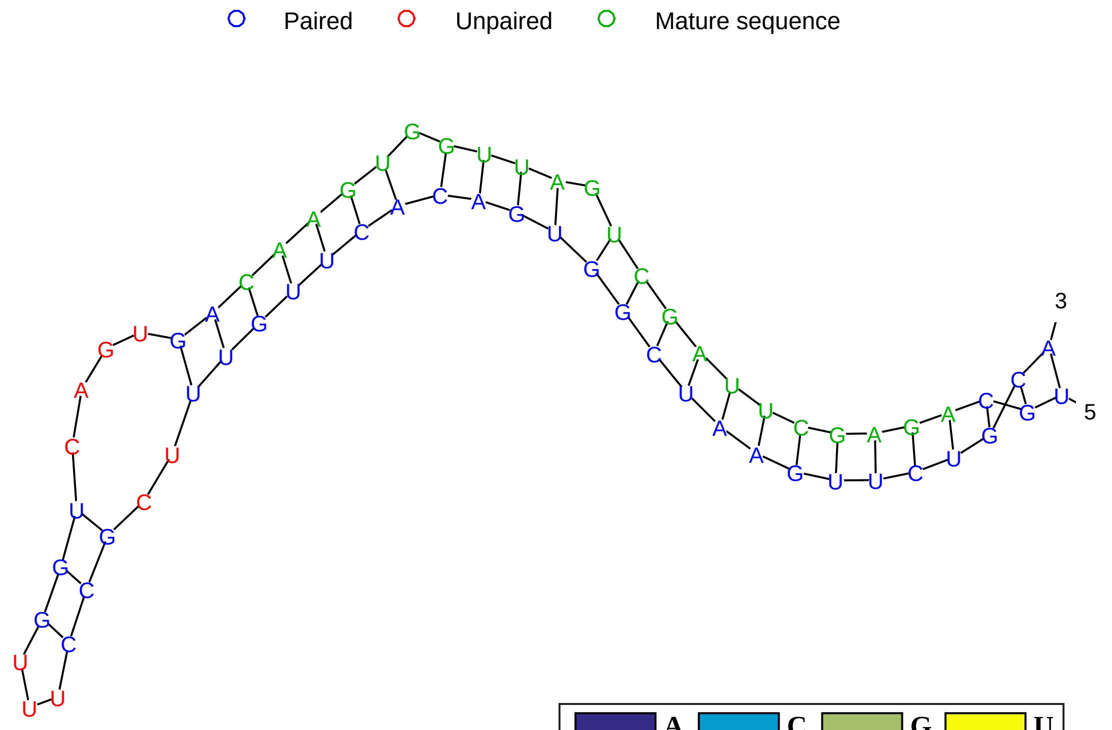

Similar miRNA: chi-miR-27b-5p
Stem loop (UMD3.1): chr8:83009838-83009904
Mature (UMD3.1): chr8:83009841-83009862
Mature seq len: 22
Total raw counts (9 samples): 502
Average raw counts: 56
Strand: Forward
Orientation: 5p
Minimum free energy: -31.90

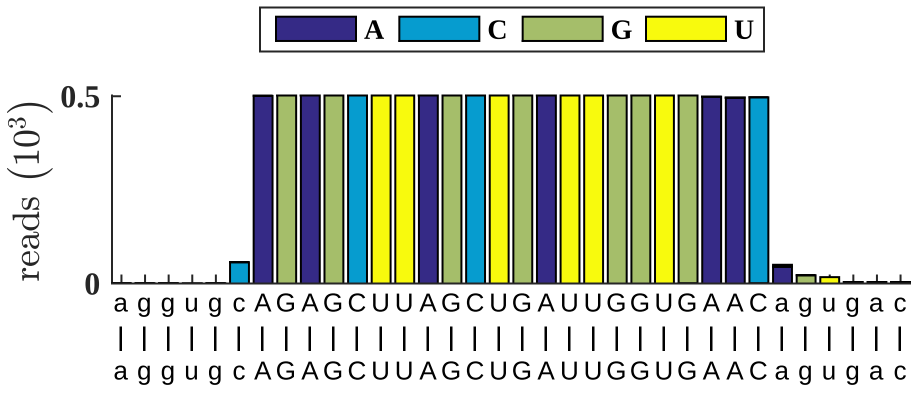

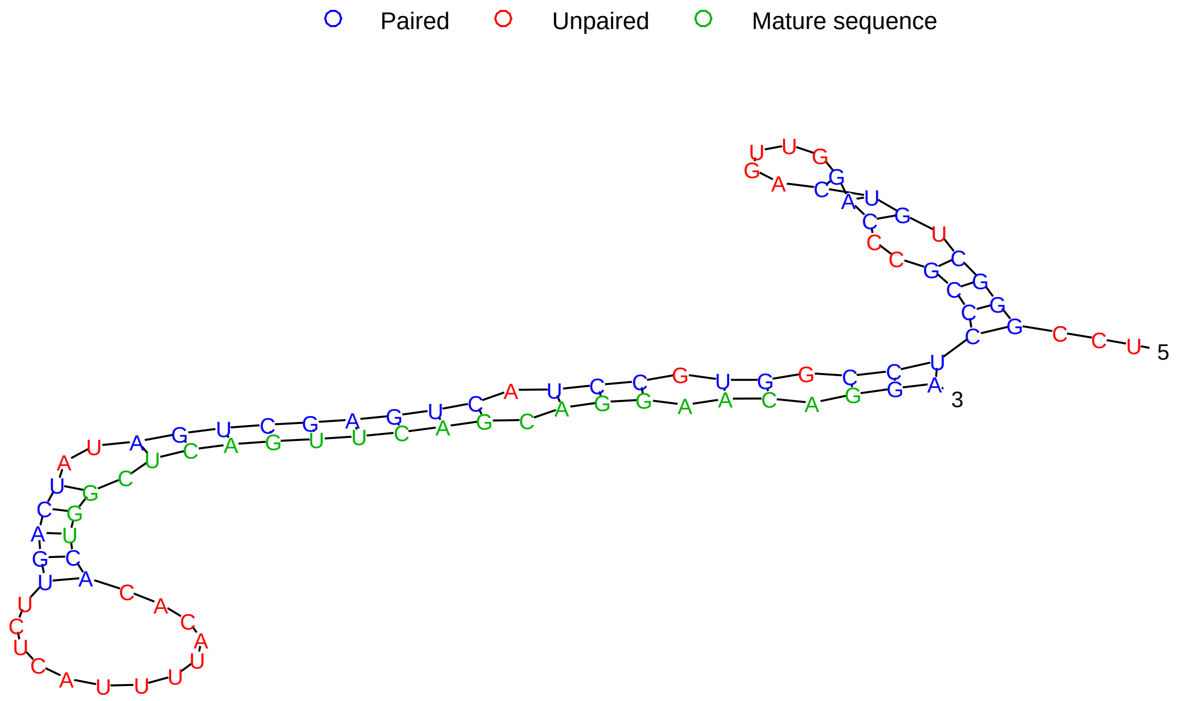

Similar miRNA: eca-miR-24
Stem loop (UMD3.1): chr8:83010353-83010443
Mature (UMD3.1): chr8:83010420-83010441
Mature seq len: 22
Total raw counts (9 samples): 3403
Average raw counts: 379
Strand: Forward
Orientation: 3p
Minimum free energy: -33.20

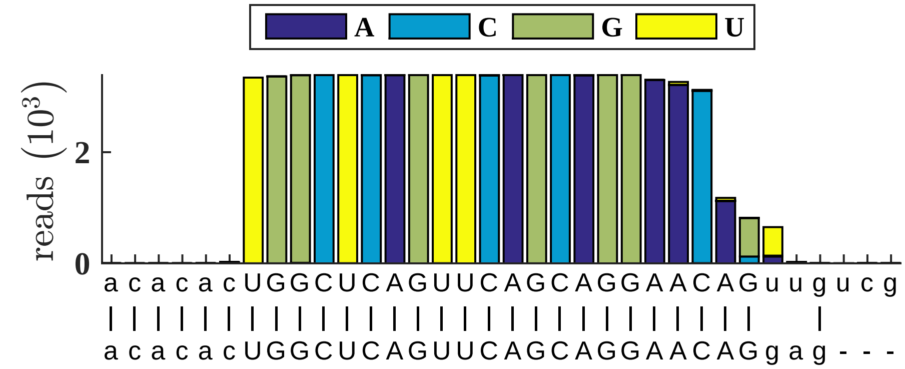

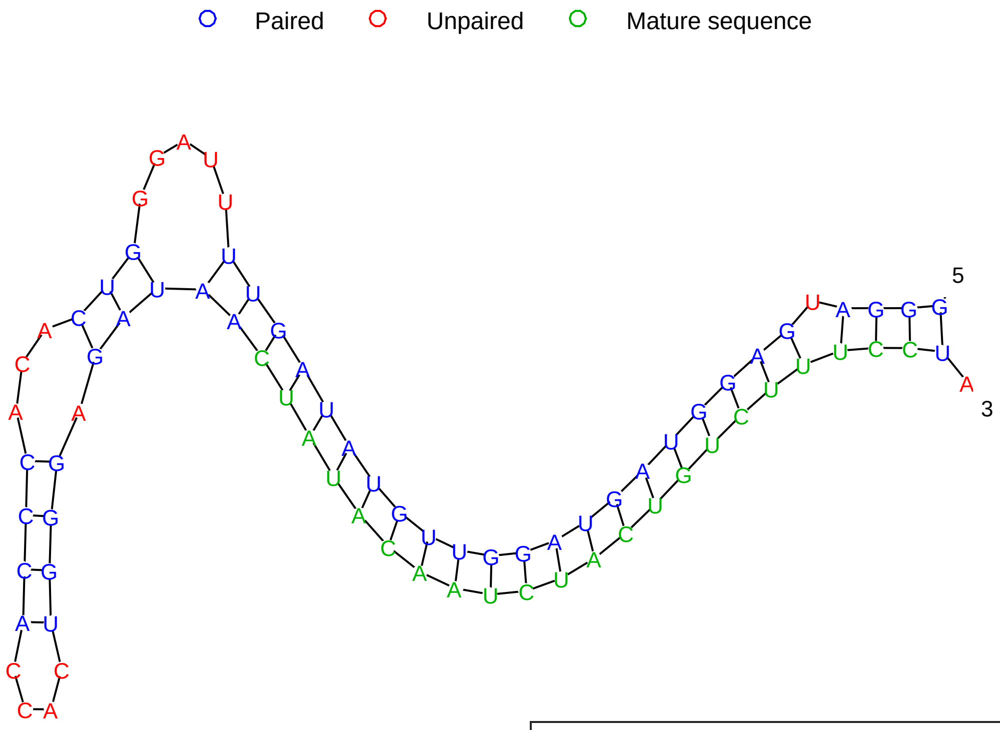

Similar miRNA: rno-let-7a-1-3p
Stem loop (UMD3.1): chr8:86884873-86884951
Mature (UMD3.1): chr8:86884928-86884949
Mature seq len: 22
Total raw counts (9 samples): 1182
Average raw counts: 132
Strand: Forward
Orientation: 3p
Minimum free energy: -33.90

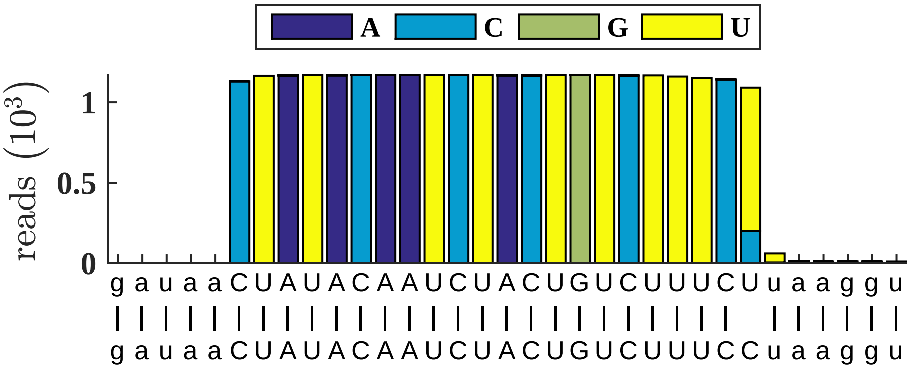

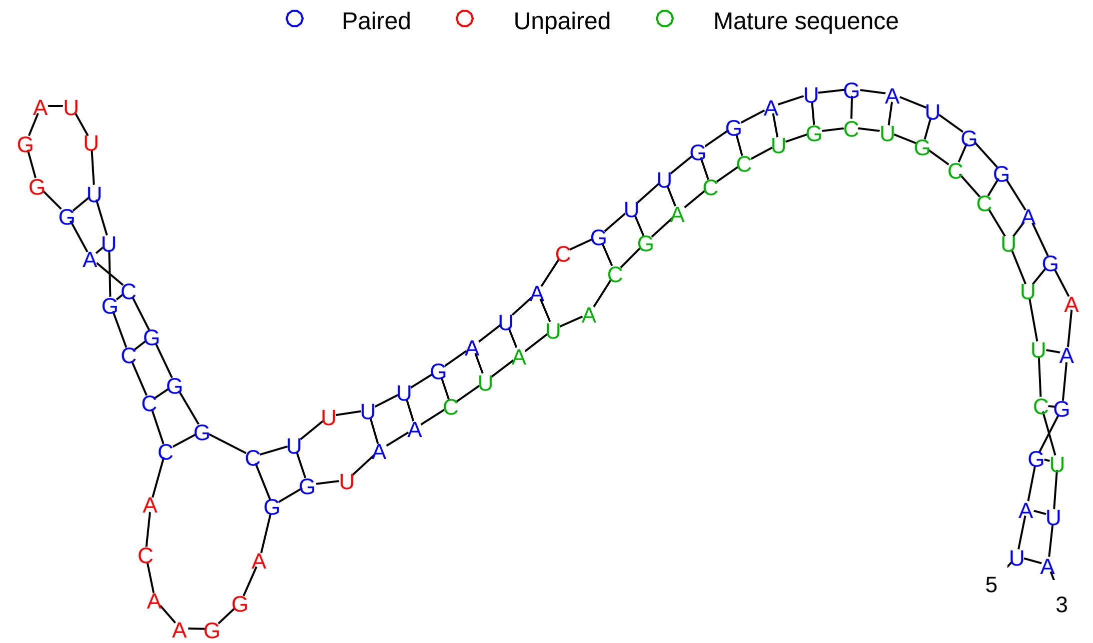

Similar miRNA: chi-let-7d-3p
Stem loop (UMD3.1): chr8:86887433-86887515
Mature (UMD3.1): chr8:86887492-86887513
Mature seq len: 22
Total raw counts (9 samples): 9046
Average raw counts: 1006
Strand: Forward
Orientation: 3p
Minimum free energy: -38.00

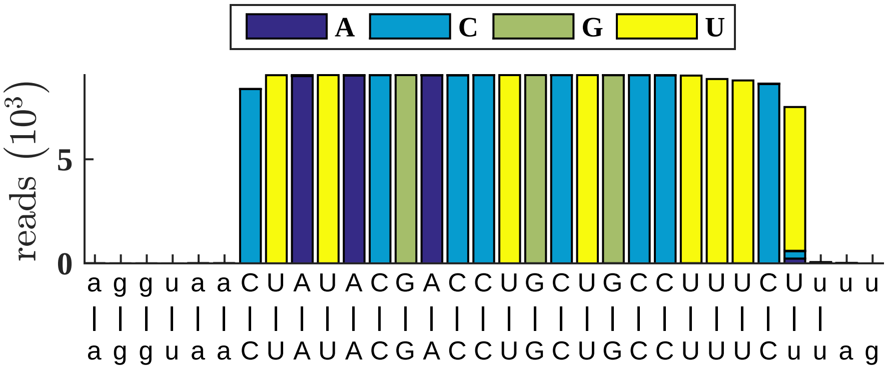

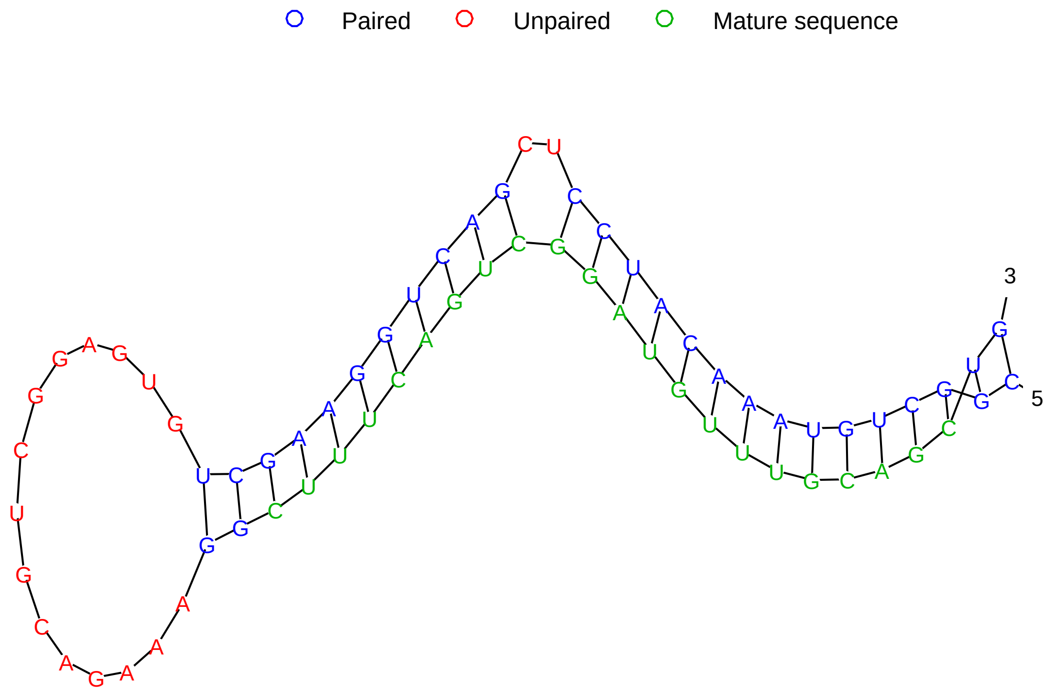

Similar miRNA: oar-miR-30a-3p
Stem loop (UMD3.1): chr9:10768282-10768350
Mature (UMD3.1): chr9:10768284-10768305
Mature seq len: 22
Total raw counts (9 samples): 11669
Average raw counts: 1297
Strand: Reverse
Orientation: 3p
Minimum free energy: -38.80

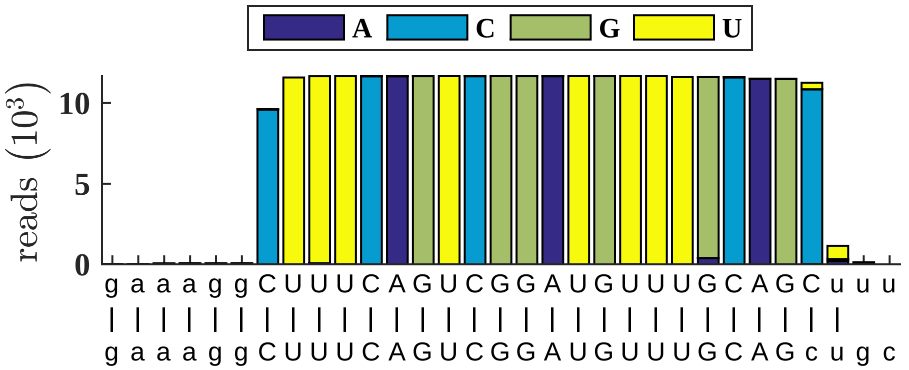

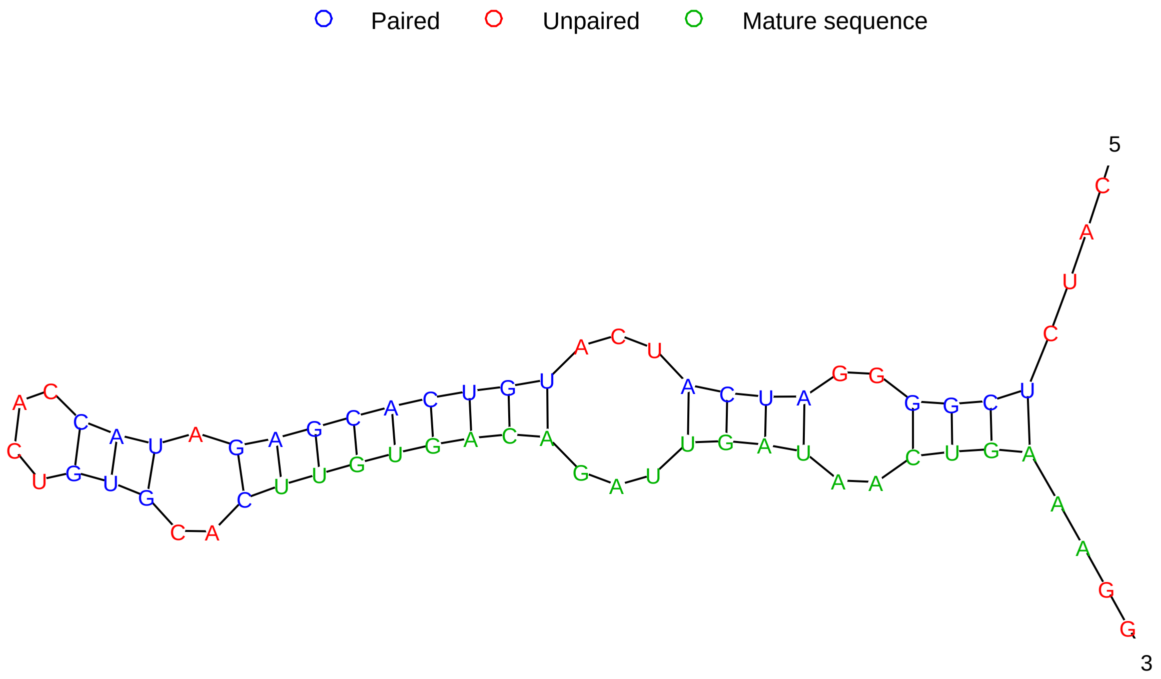

Similar miRNA: cfa-miR-542
Stem loop (UMD3.1): chrX:18180149-18180213
Mature (UMD3.1): chrX:18180151-18180173
Mature seq len: 23
Total raw counts (9 samples): 1932
Average raw counts: 215
Strand: Reverse
Orientation: 3p
Minimum free energy: -22.20

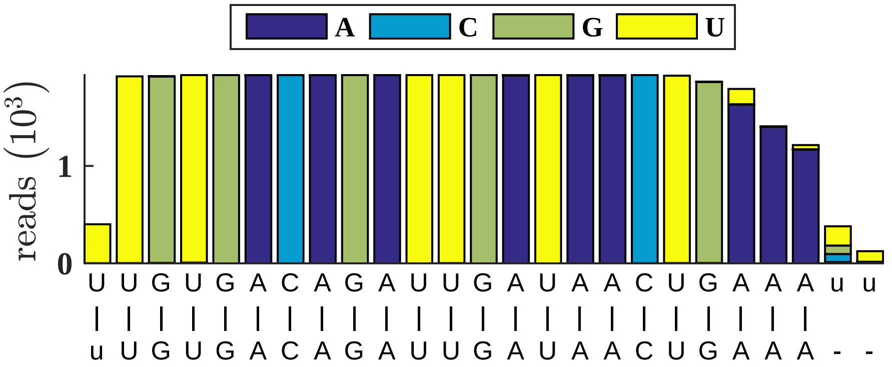

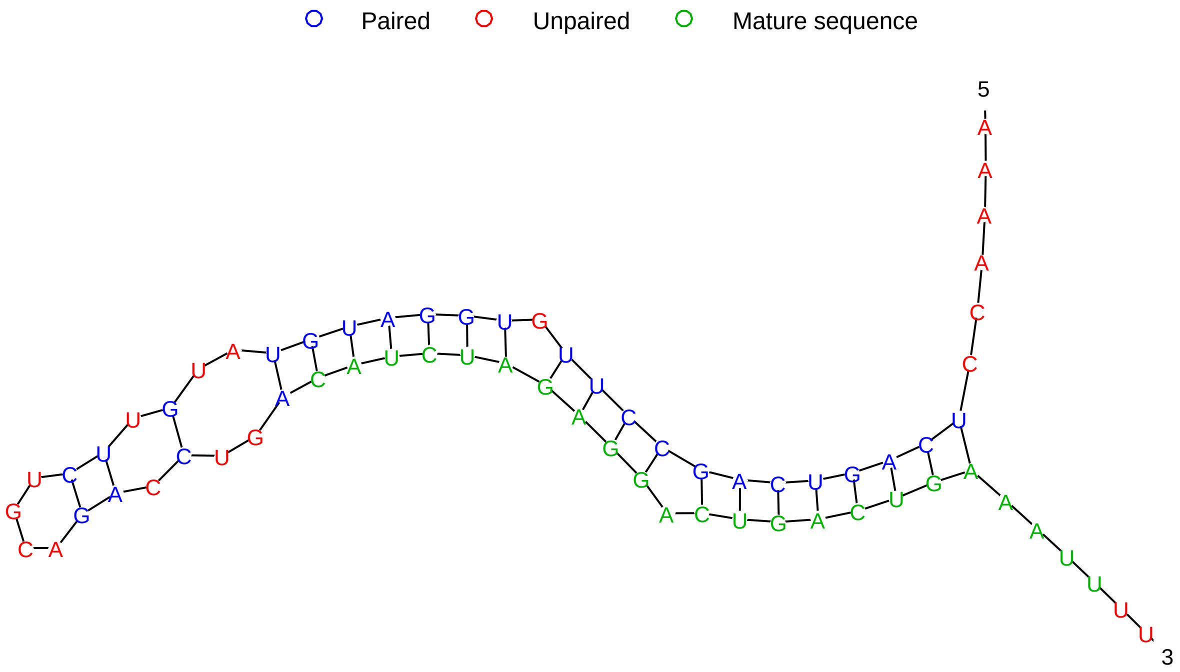

Similar miRNA: chi-miR-3431-3p
Stem loop (UMD3.1): chrX:34662673-34662740
Mature (UMD3.1): chrX:34662675-34662697
Mature seq len: 23
Total raw counts (9 samples): 5441
Average raw counts: 605
Strand: Reverse
Orientation: 3p
Minimum free energy: -26.30

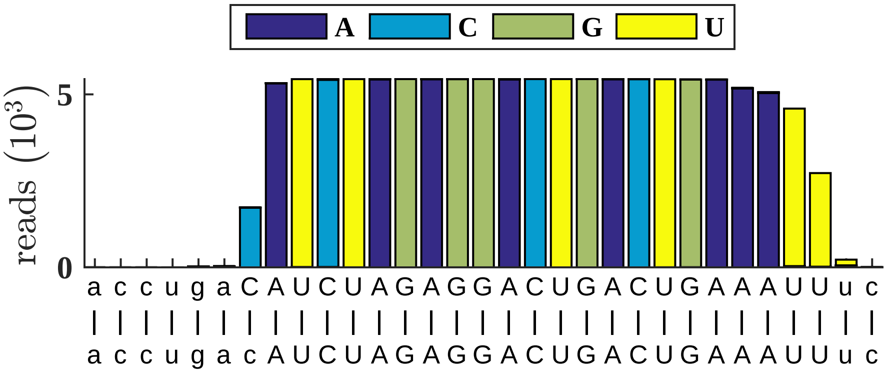

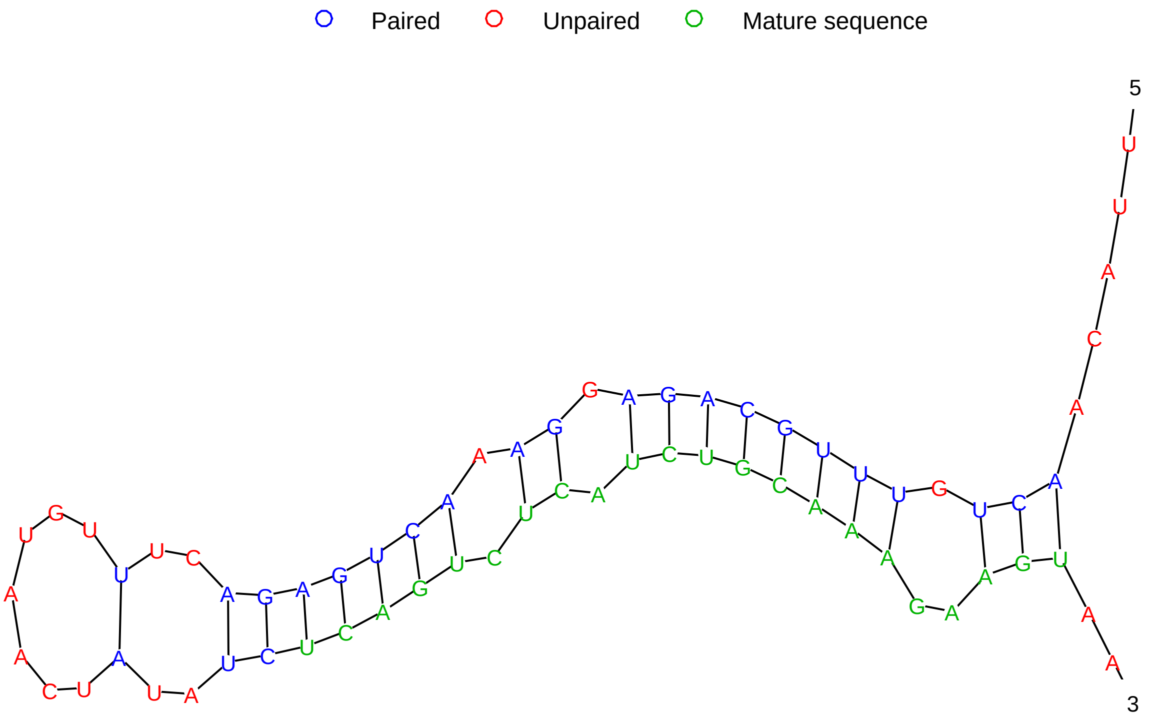

Similar miRNA: mml-miR-452-3p
Stem loop (UMD3.1): chrX:34665633-34665699
Mature (UMD3.1): chrX:34665635-34665656
Mature seq len: 22
Total raw counts (9 samples): 1024
Average raw counts: 114
Strand: Reverse
Orientation: 3p
Minimum free energy: -22.20

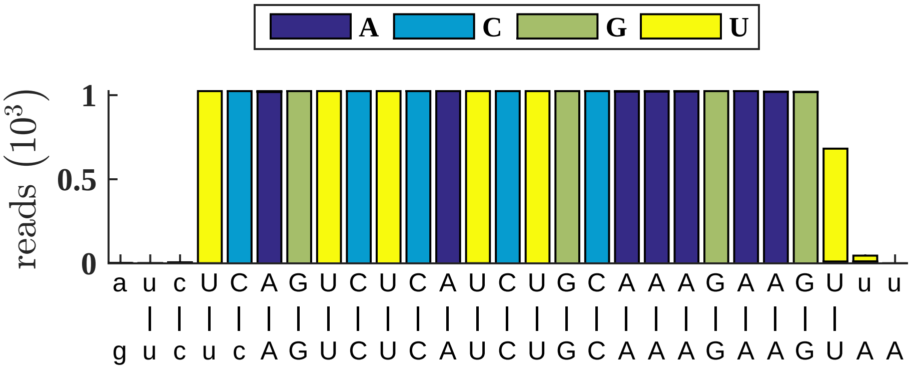

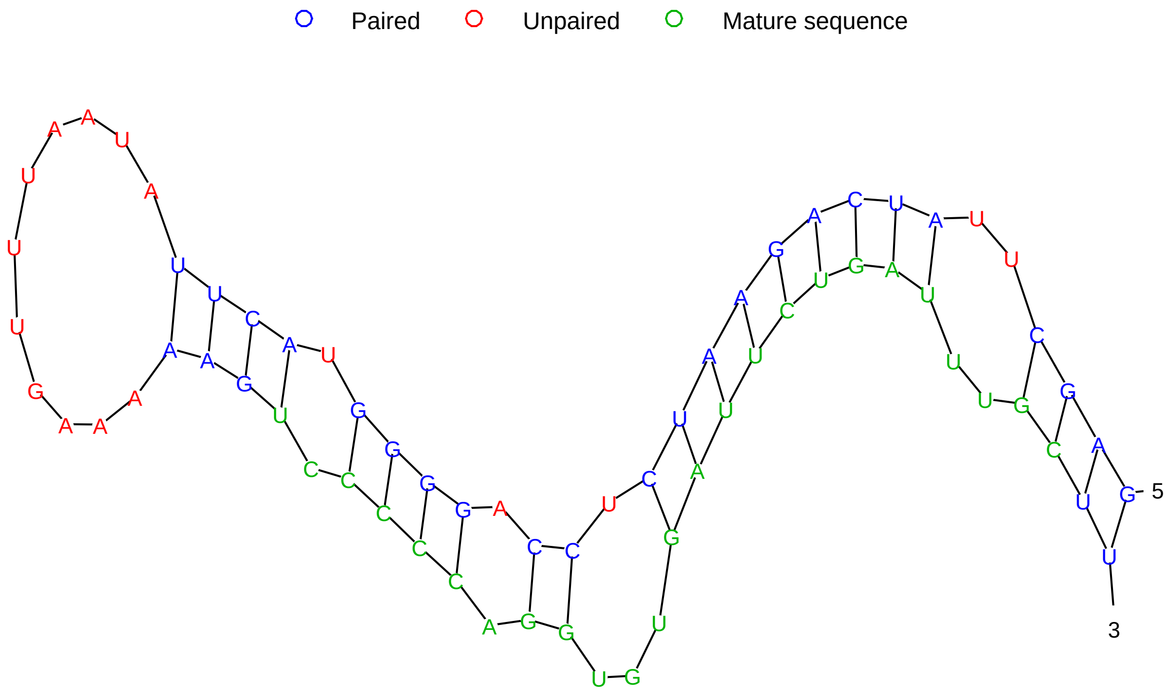

Similar miRNA: chi-miR-361-3p
Stem loop (UMD3.1): chrX:74328423-74328491
Mature (UMD3.1): chrX:74328465-74328489
Mature seq len: 25
Total raw counts (9 samples): 2387
Average raw counts: 266
Strand: Forward
Orientation: 3p
Minimum free energy: -28.40

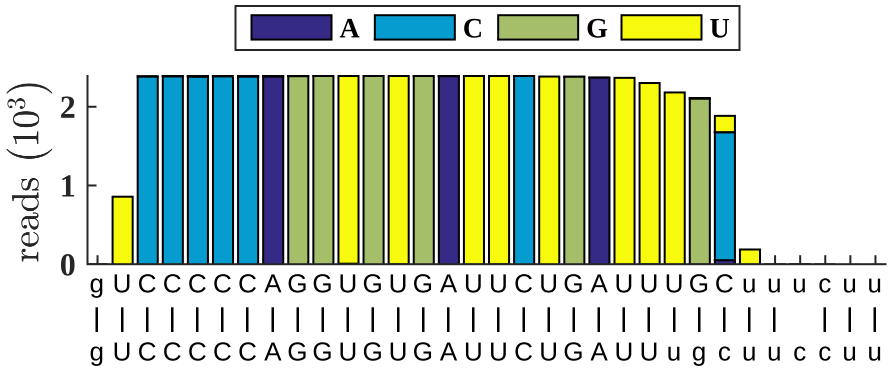

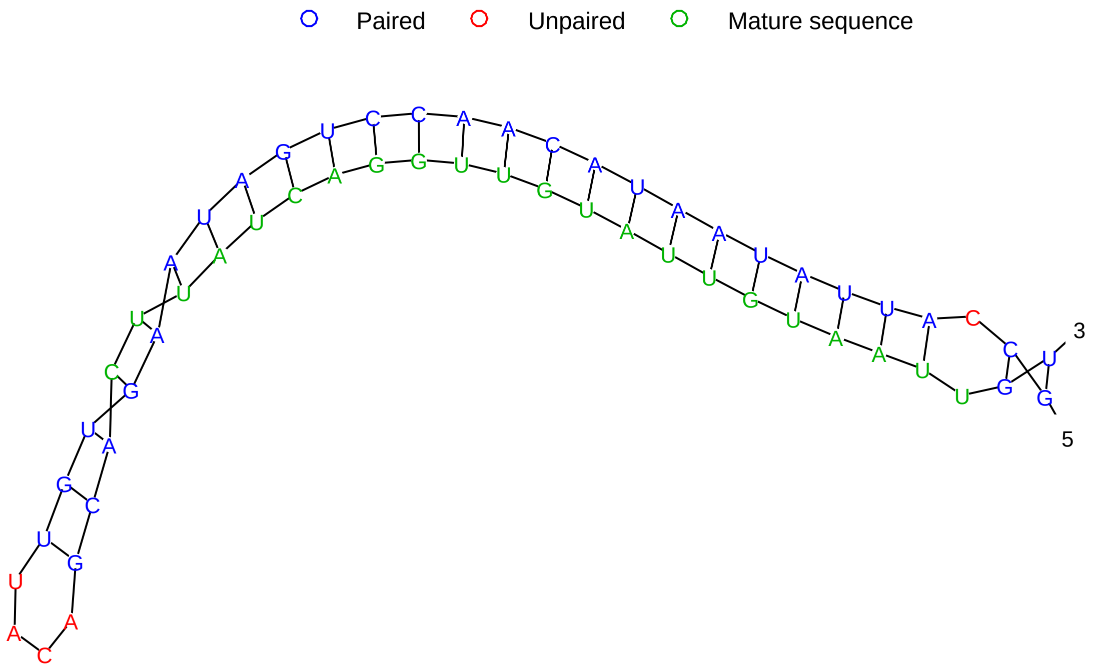

Similar miRNA: chi-miR-374a-3p
Stem loop (UMD3.1): chrX:81951230-81951287
Mature (UMD3.1): chrX:81951264-81951285
Mature seq len: 22
Total raw counts (9 samples): 2142
Average raw counts: 238
Strand: Forward
Orientation: 3p
Minimum free energy: -33.90

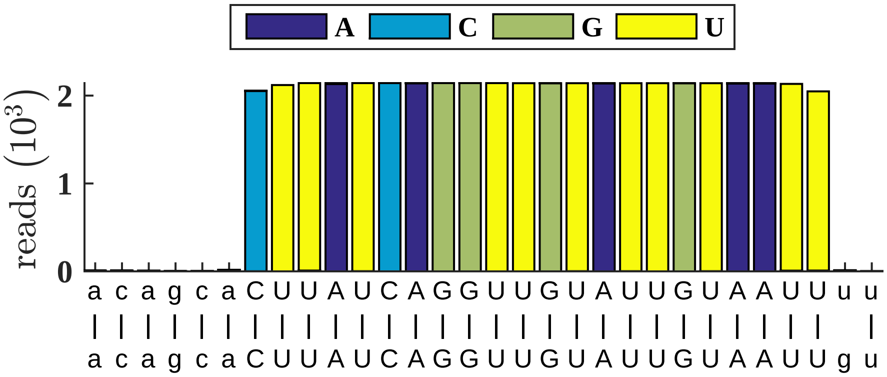

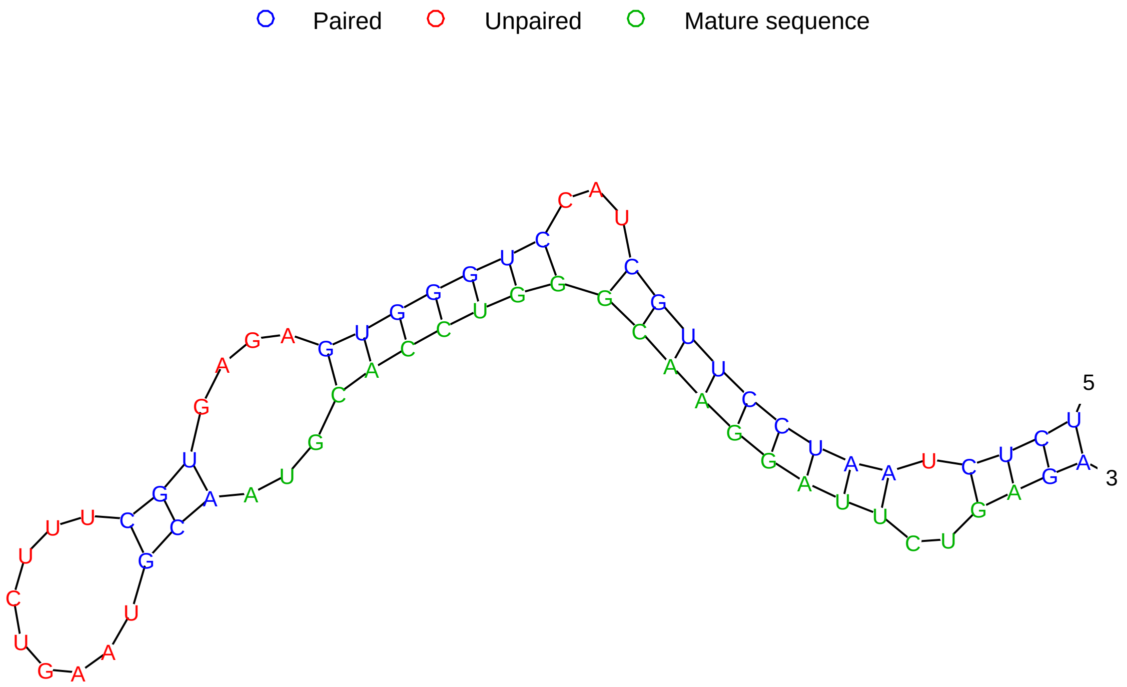

Similar miRNA: chi-miR-500-3p
Stem loop (UMD3.1): chrX:92899706-92899773
Mature (UMD3.1): chrX:92899749-92899771
Mature seq len: 23
Total raw counts (9 samples): 13608
Average raw counts: 1512
Strand: Forward
Orientation: 3p
Minimum free energy: -28.10

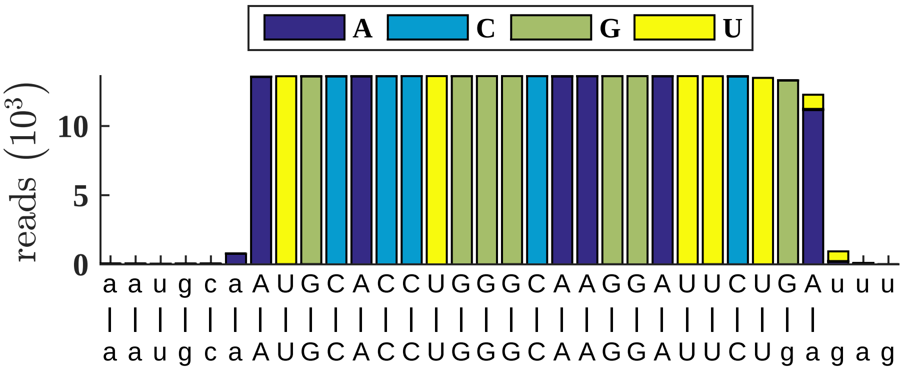

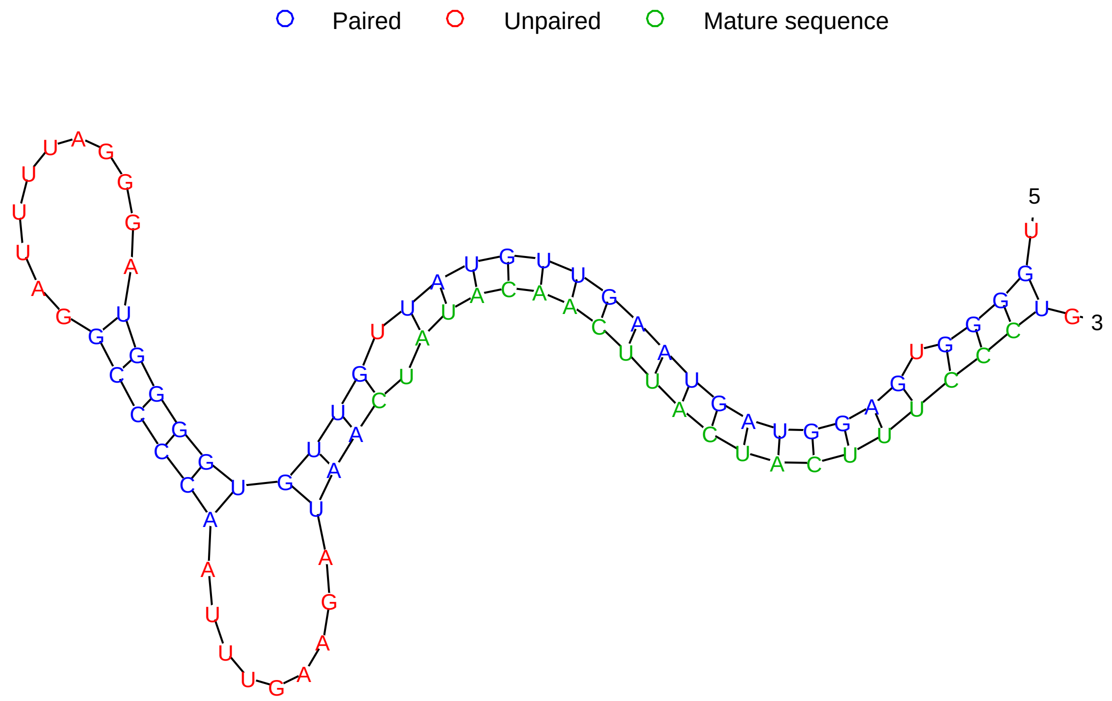

Similar miRNA: chi-miR-98-3p
Stem loop (UMD3.1): chrX:96382644-96382730
Mature (UMD3.1): chrX:96382646-96382667
Mature seq len: 22
Total raw counts (9 samples): 867
Average raw counts: 97
Strand: Reverse
Orientation: 3p
Minimum free energy: -38.20

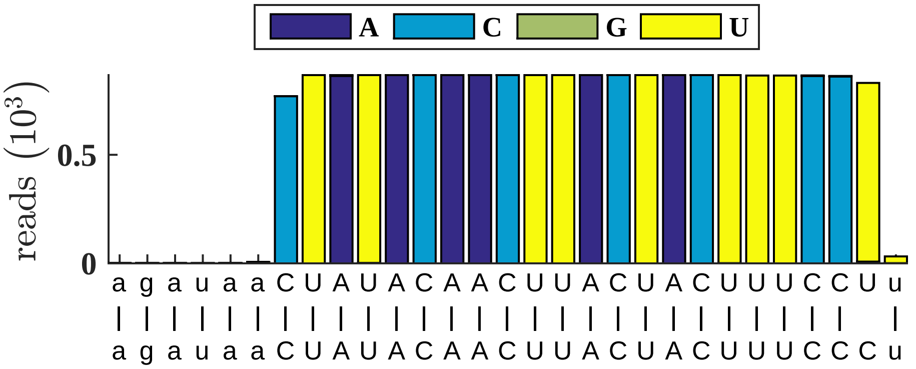

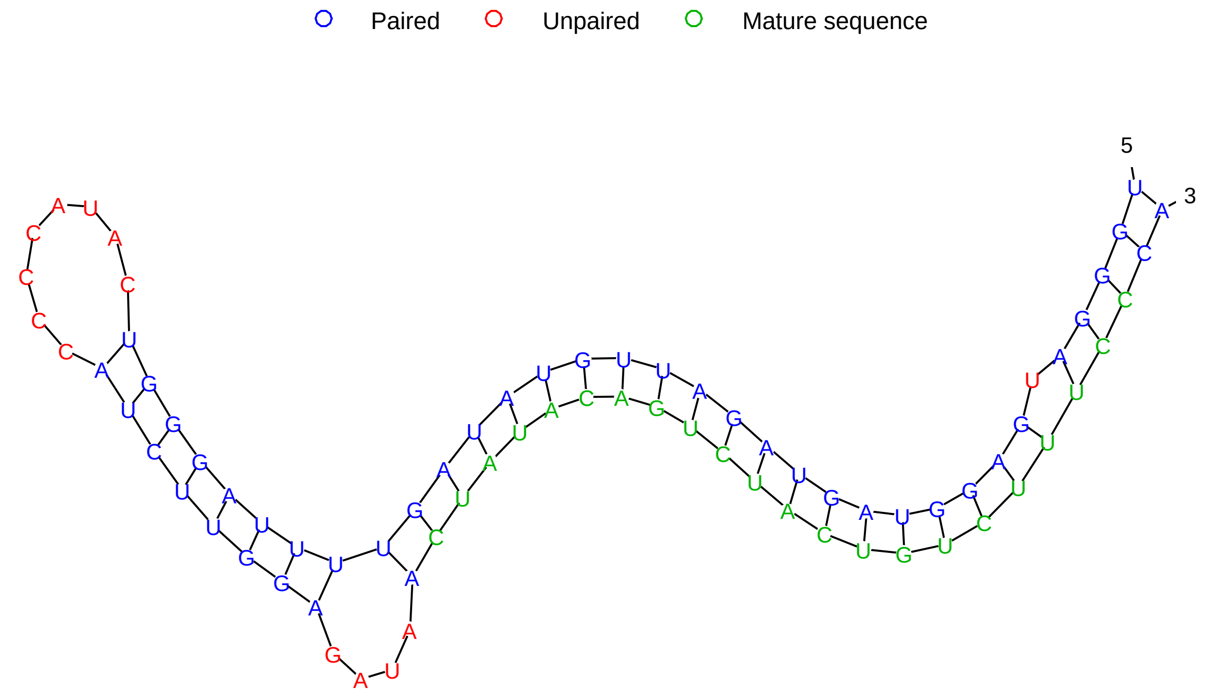

Similar miRNA: hsa-let-7f-2-3p
Stem loop (UMD3.1): chrX:96383534-96383612
Mature (UMD3.1): chrX:96383536-96383557
Mature seq len: 22
Total raw counts (9 samples): 1193
Average raw counts: 133
Strand: Reverse
Orientation: 3p
Minimum free energy: -34.80

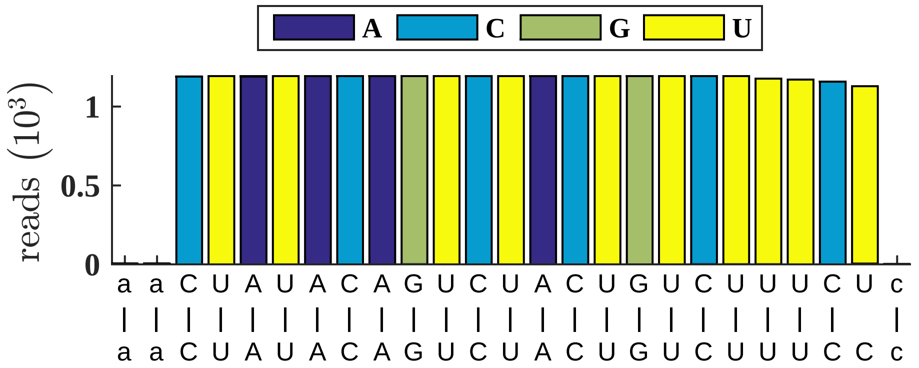

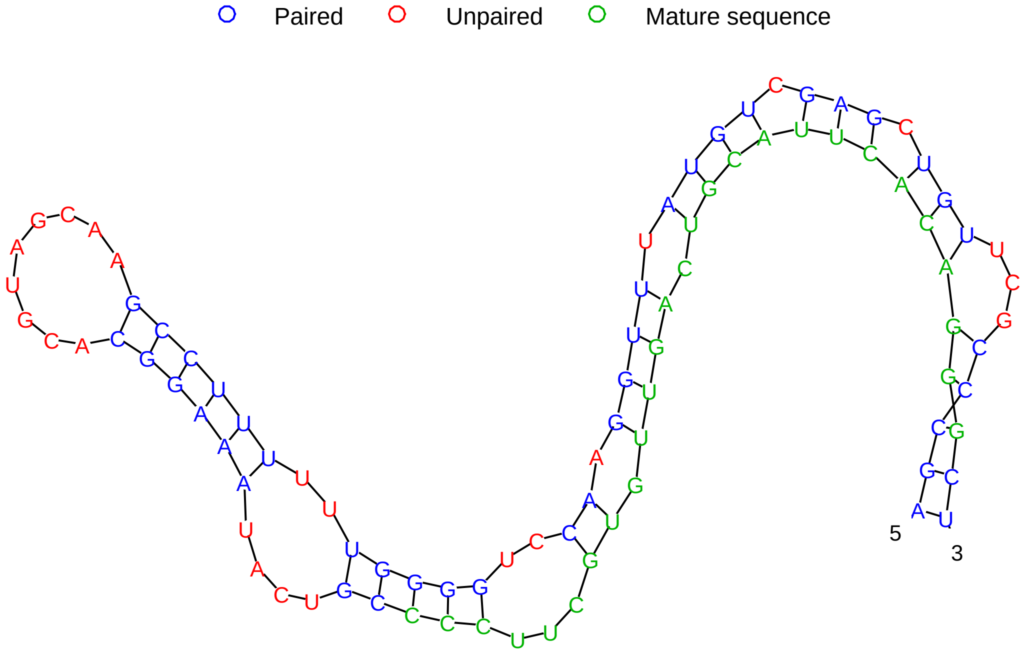

Stem loop (UMD3.1): chr1:116105315-116105407
Mature (UMD3.1): chr1:116105379-116105405
Mature seq len: 27
Total raw counts (9 samples): 1265
Average raw counts: 141
Strand: Forward
Orientation: 3p
Minimum free energy: -27.90

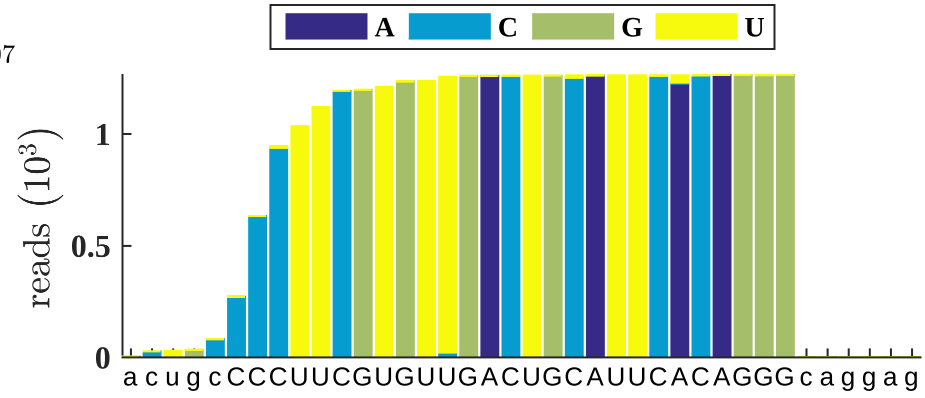

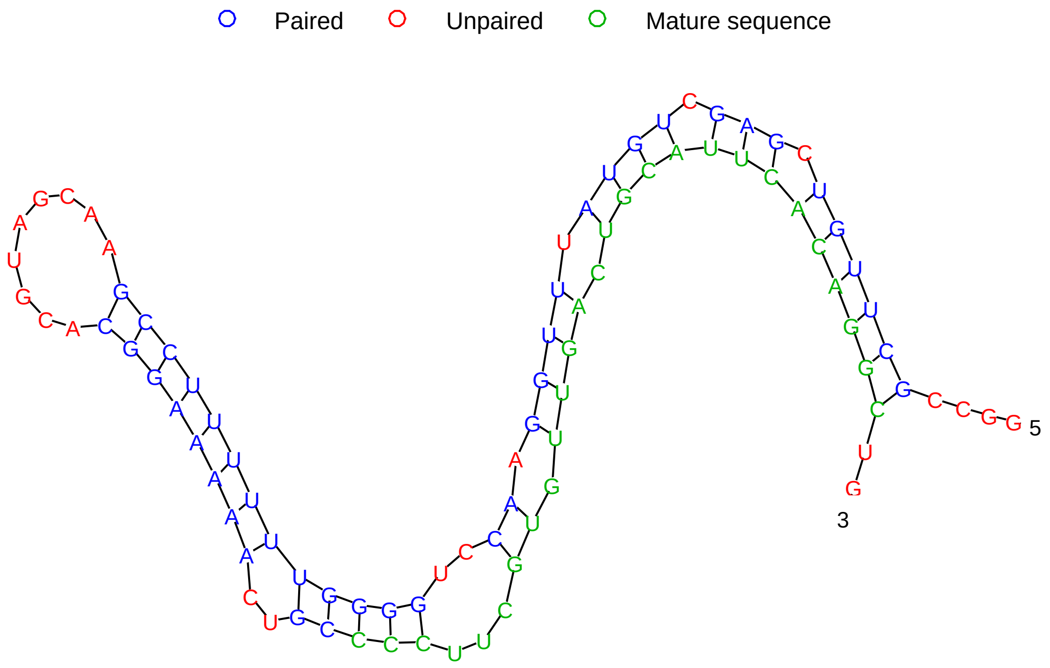

Stem loop (UMD3.1): chr1:116106717-116106808
Mature (UMD3.1): chr1:116106780-116106806
Mature seq len: 27
Total raw counts (9 samples): 1190
Average raw counts: 133
Strand: Forward
Orientation: 3p
Minimum free energy: -28.90

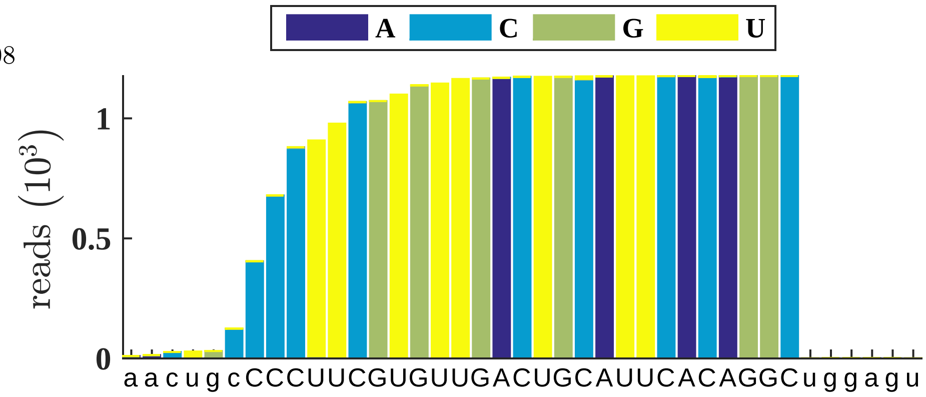

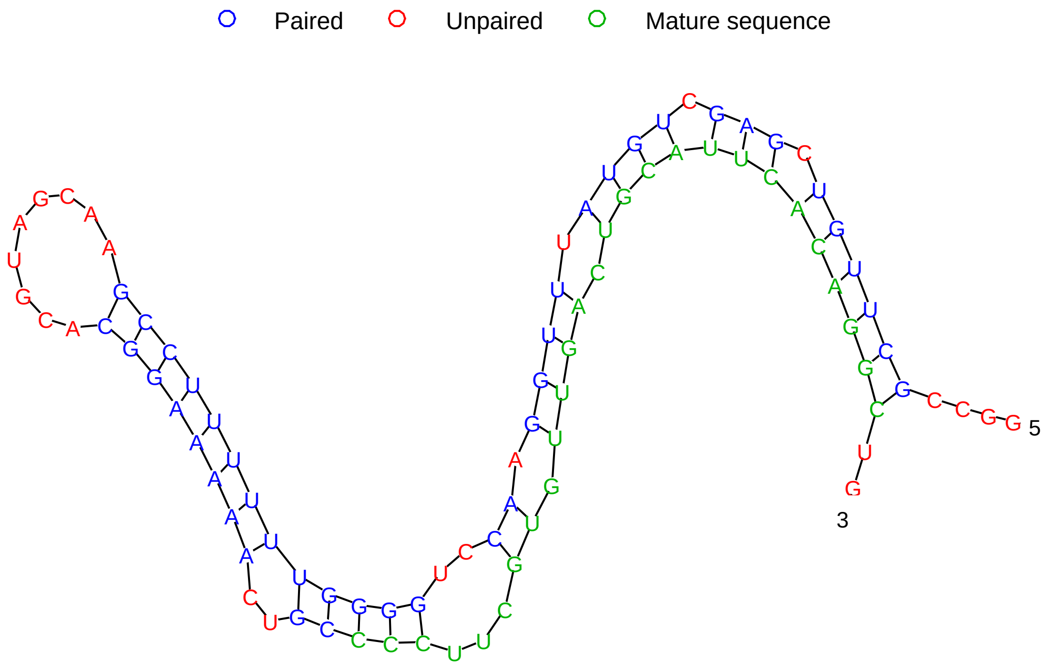

Stem loop (UMD3.1): chr1:124942296-124942387
Mature (UMD3.1): chr1:124942359-124942385
Mature seq len: 27
Total raw counts (9 samples): 1167
Average raw counts: 130
Strand: Forward
Orientation: 3p
Minimum free energy: -28.90

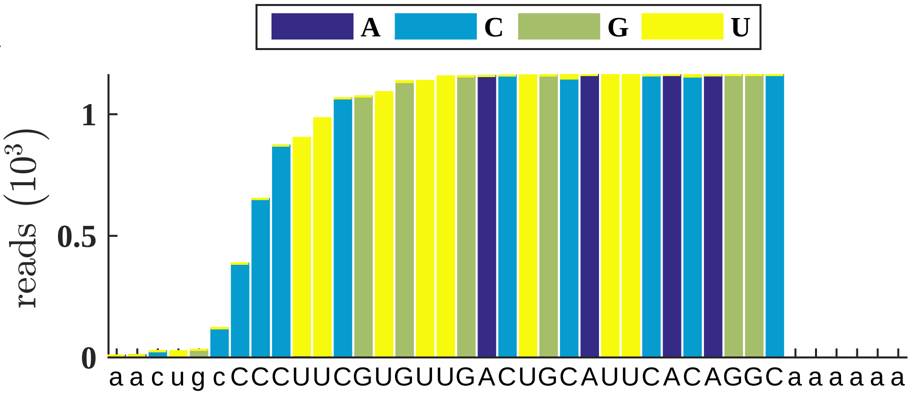

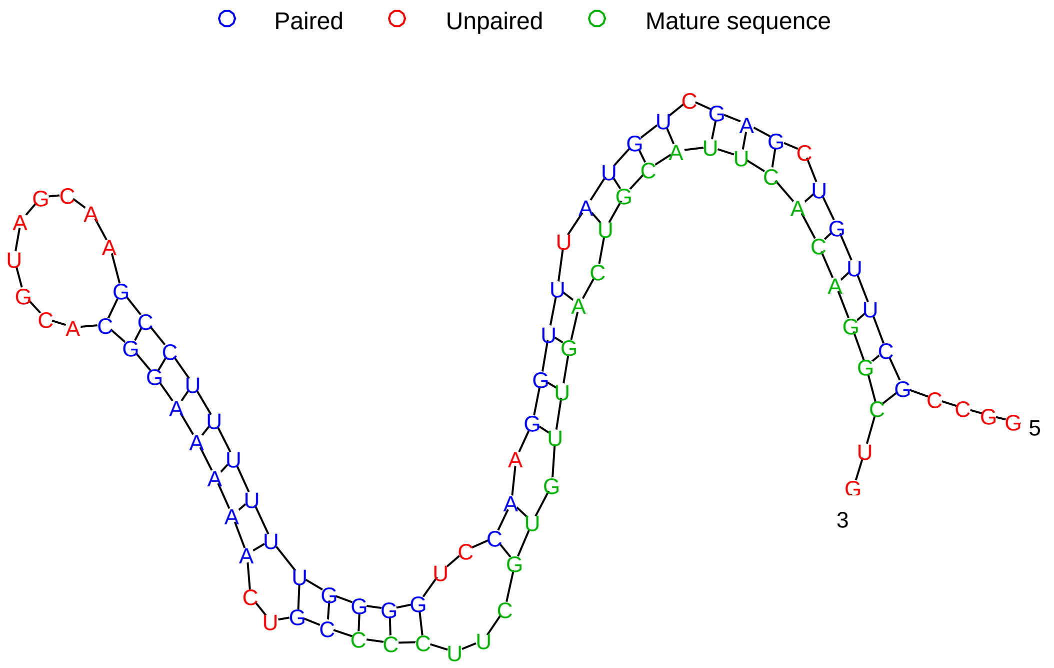

Stem loop (UMD3.1): chr1:124943819-124943910
Mature (UMD3.1): chr1:124943821-124943847
Mature seq len: 27
Total raw counts (9 samples): 1204
Average raw counts: 134
Strand: Reverse
Orientation: 3p
Minimum free energy: -28.90

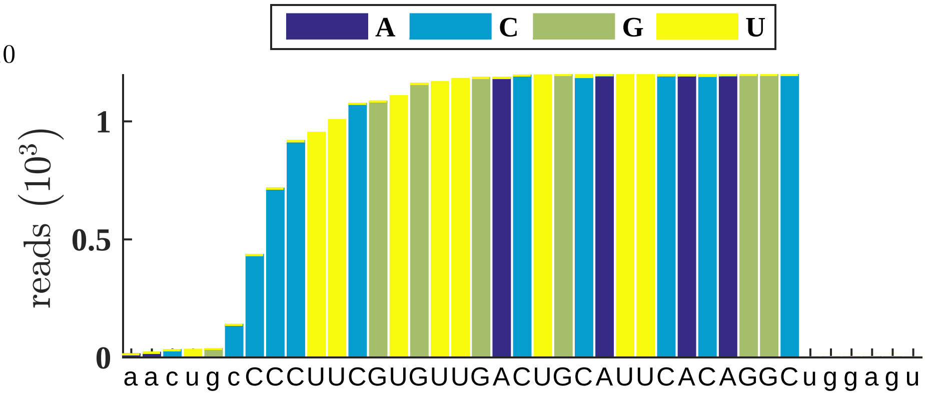

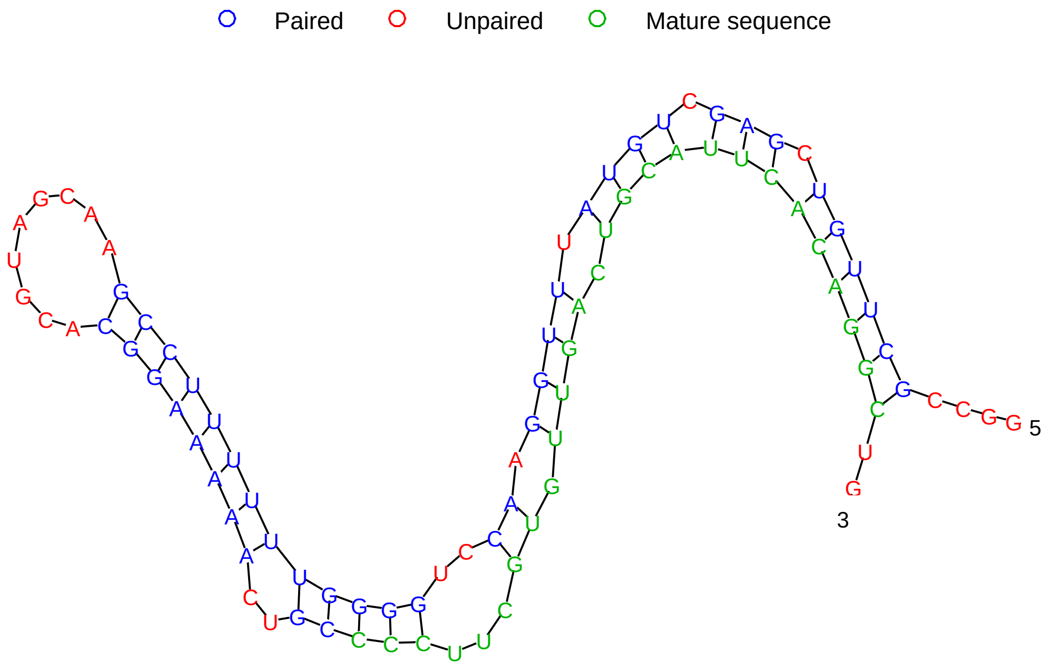

Stem loop (UMD3.1): chr1:124946085-124946176
Mature (UMD3.1): chr1:124946087-124946113
Mature seq len: 27
Total raw counts (9 samples): 1240
Average raw counts: 138
Strand: Reverse
Orientation: 3p
Minimum free energy: -28.90

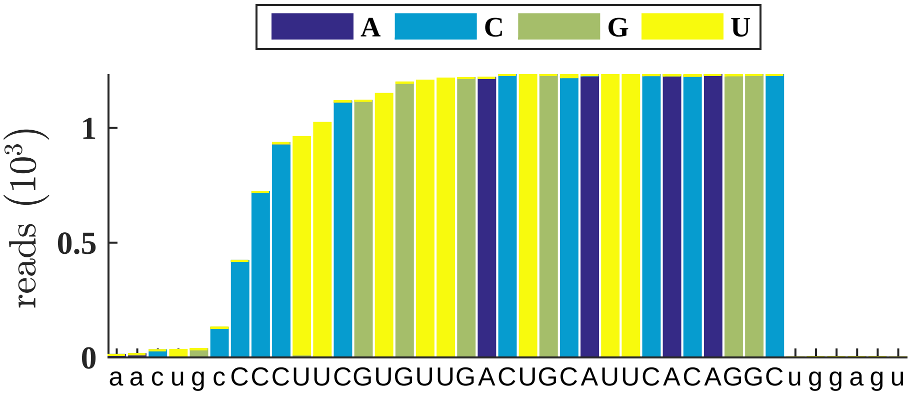

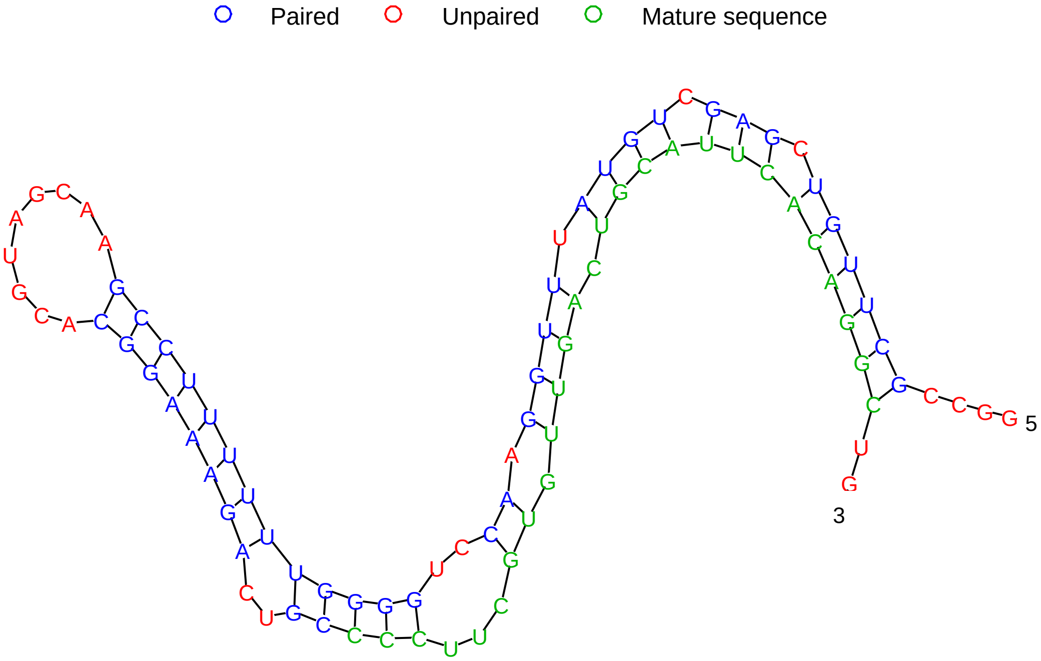

Stem loop (UMD3.1): chr1:124954435-124954526
Mature (UMD3.1): chr1:124954437-124954463
Mature seq len: 27
Total raw counts (9 samples): 1157
Average raw counts: 129
Strand: Reverse
Orientation: 3p
Minimum free energy: -29.00

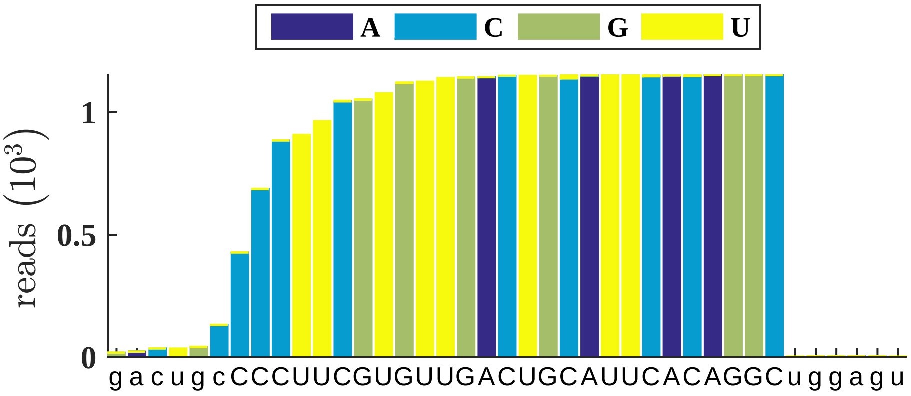

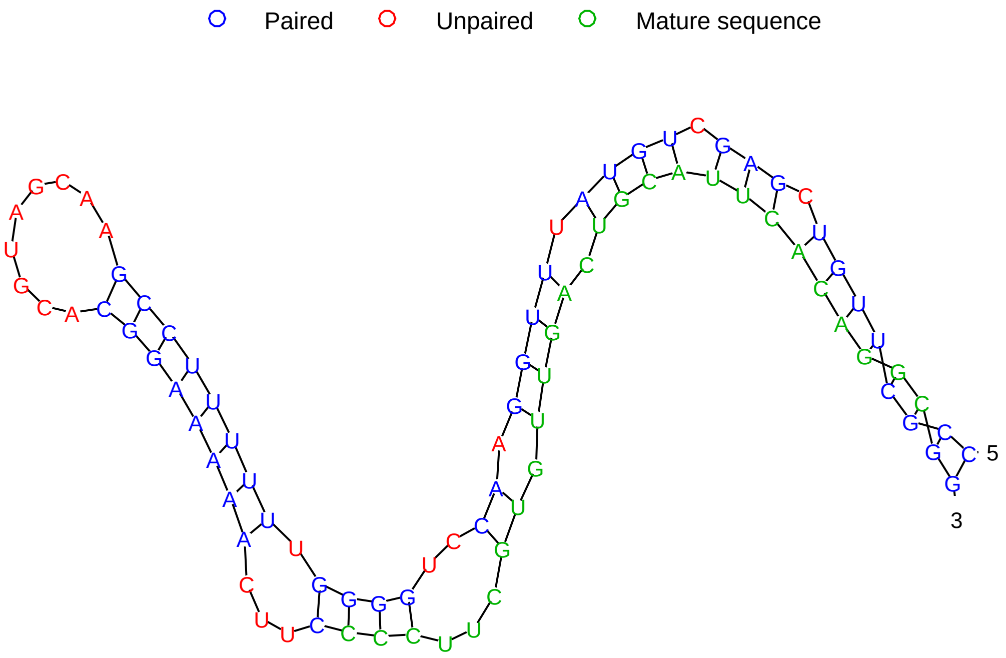

Stem loop (UMD3.1): chr1:13546939-13547028
Mature (UMD3.1): chr1:13546941-13546967
Mature seq len: 27
Total raw counts (9 samples): 1189
Average raw counts: 133
Strand: Reverse
Orientation: 3p
Minimum free energy: -31.50

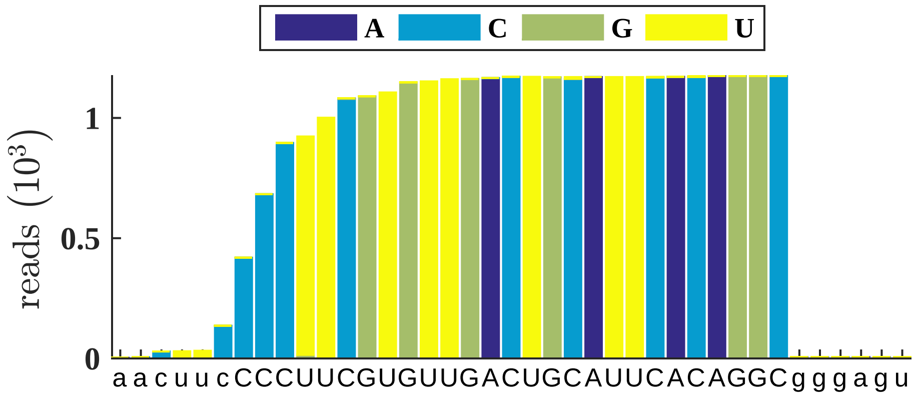

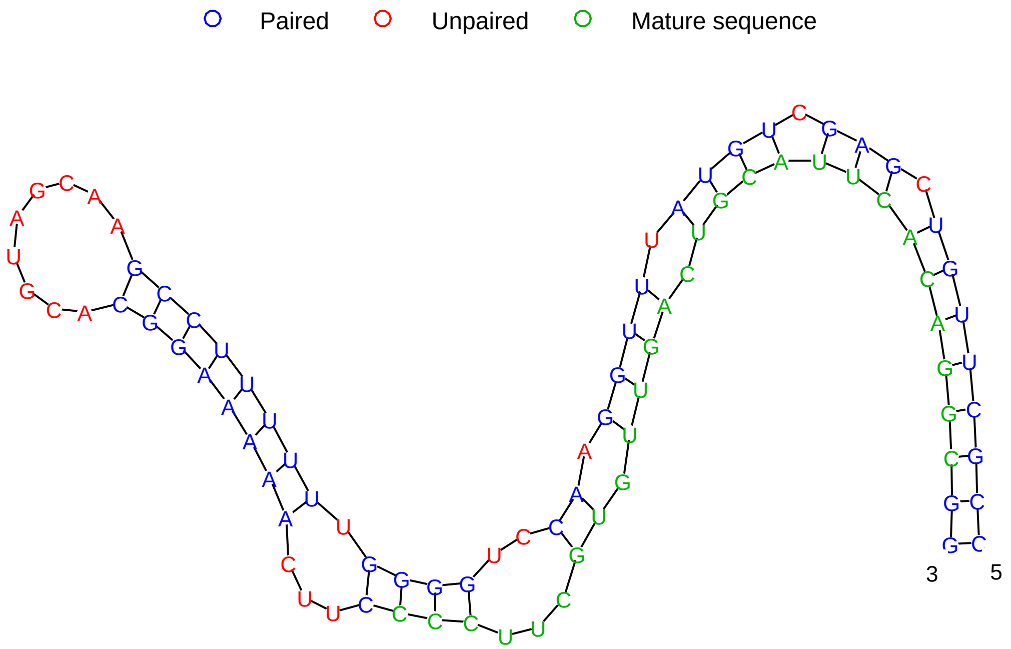

Stem loop (UMD3.1): chr1:13549454-13549543
Mature (UMD3.1): chr1:13549515-13549541
Mature seq len: 27
Total raw counts (9 samples): 1231
Average raw counts: 137
Strand: Forward
Orientation: 3p
Minimum free energy: -31.50

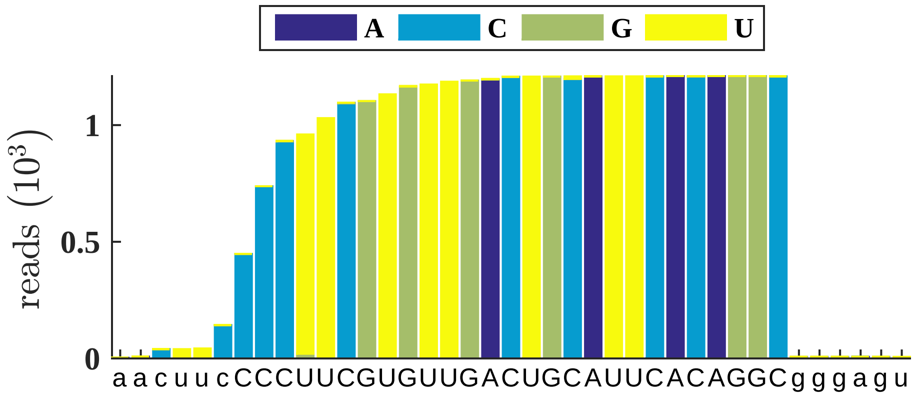

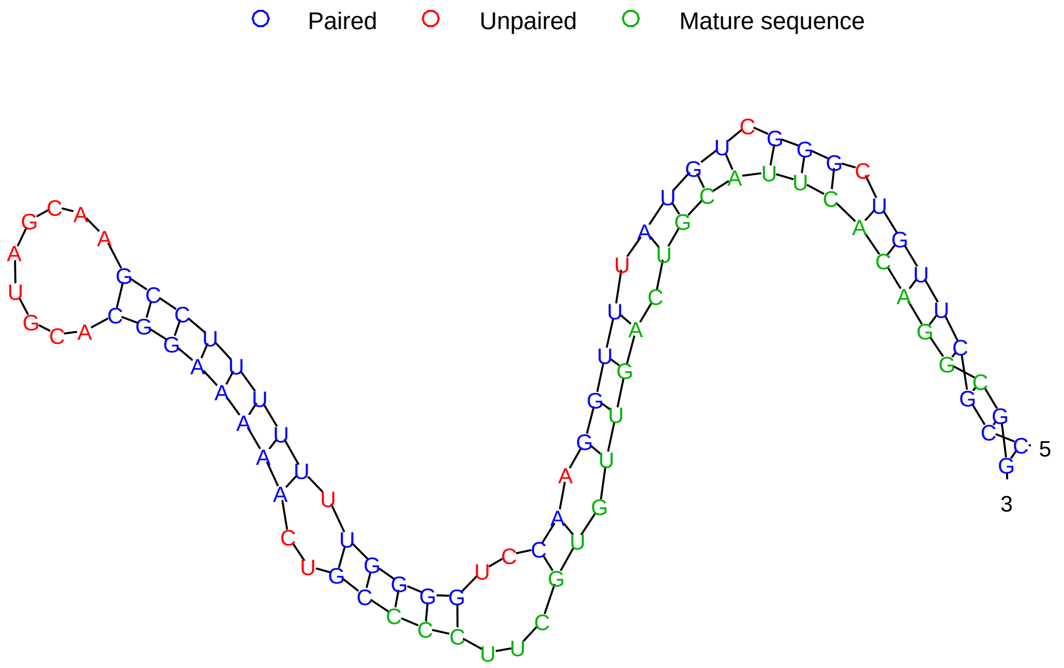

Stem loop (UMD3.1): chr1:135833119-135833209
Mature (UMD3.1): chr1:135833121-135833147
Mature seq len: 27
Total raw counts (9 samples): 1255
Average raw counts: 140
Strand: Reverse
Orientation: 3p
Minimum free energy: -33.00

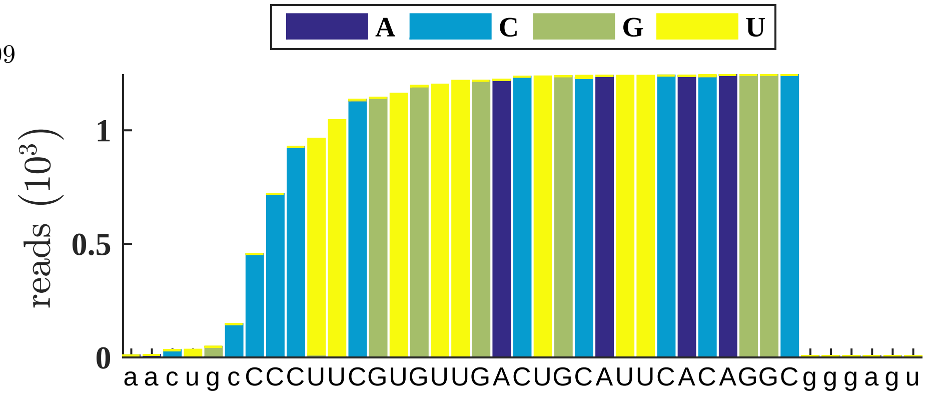

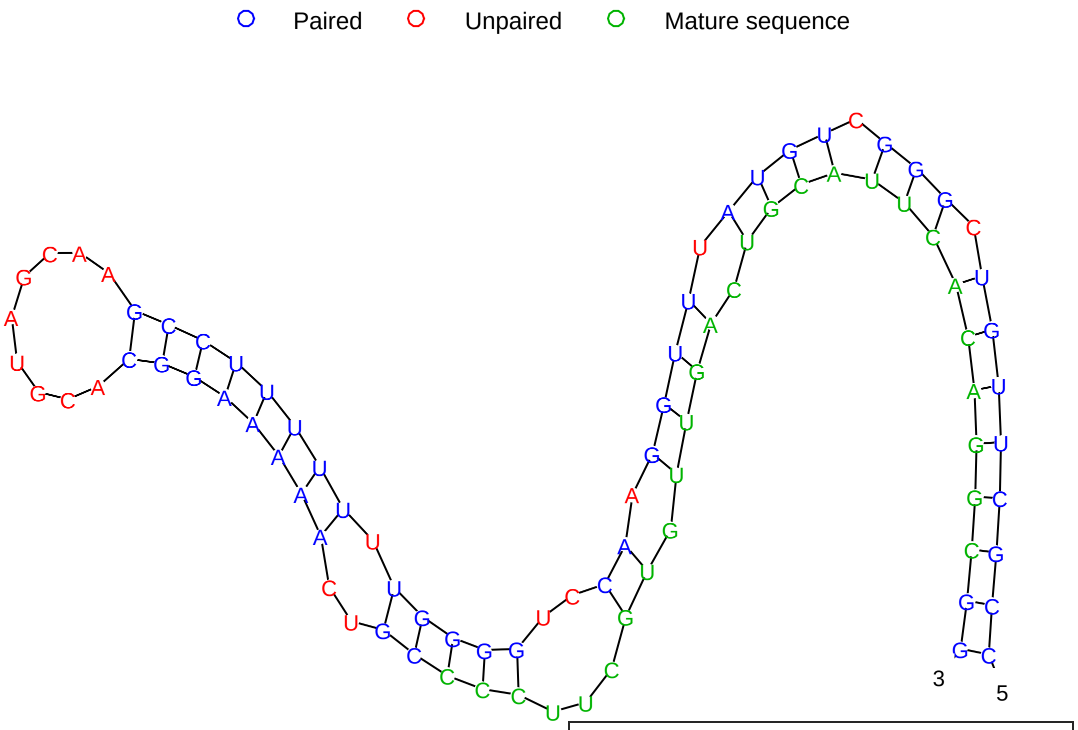

Stem loop (UMD3.1): chr1:139206907-139206997
Mature (UMD3.1): chr1:139206909-139206935
Mature seq len: 27
Total raw counts (9 samples): 1155
Average raw counts: 129
Strand: Reverse
Orientation: 3p
Minimum free energy: -33.00

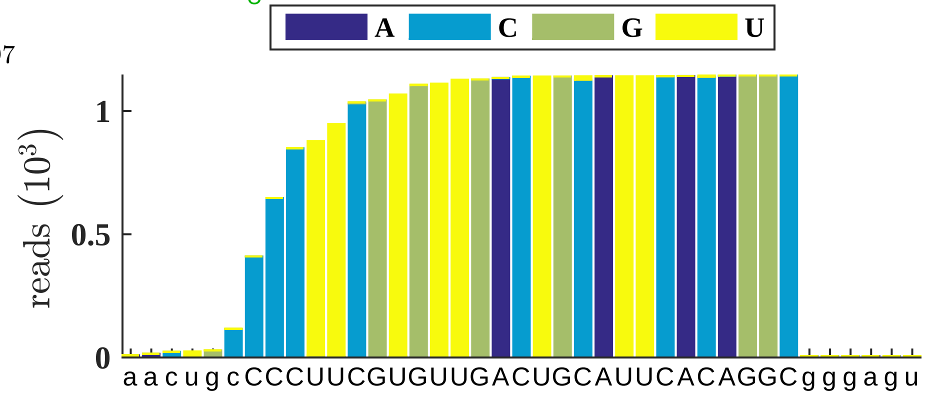

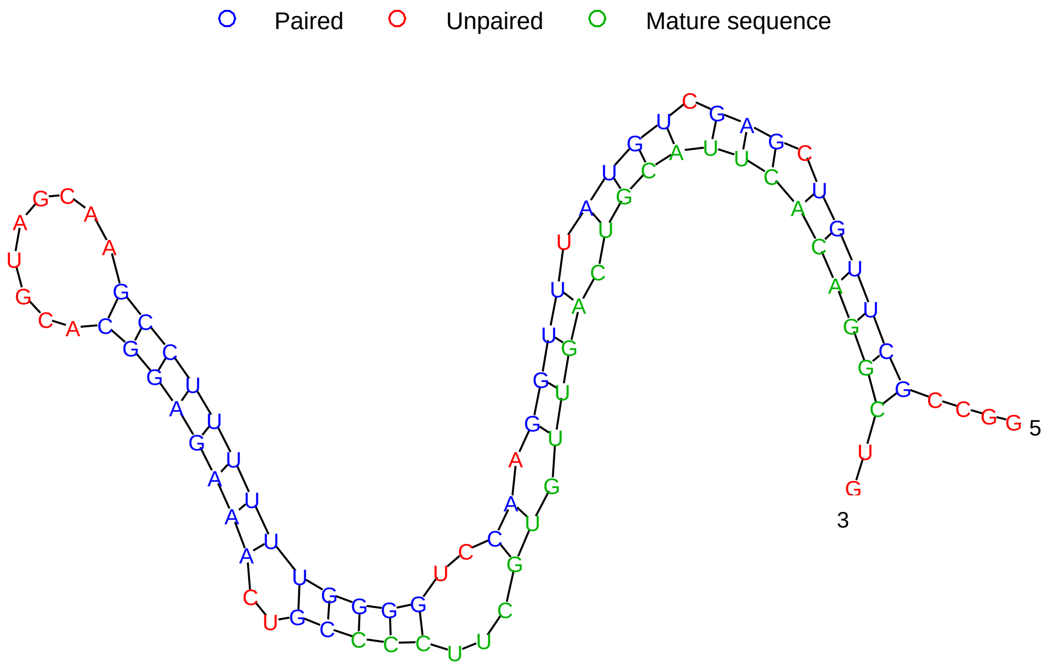

Stem loop (UMD3.1): chr1:144097441-144097532
Mature (UMD3.1): chr1:144097504-144097530
Mature seq len: 27
Total raw counts (9 samples): 1229
Average raw counts: 137
Strand: Forward
Orientation: 3p
Minimum free energy: -29.00

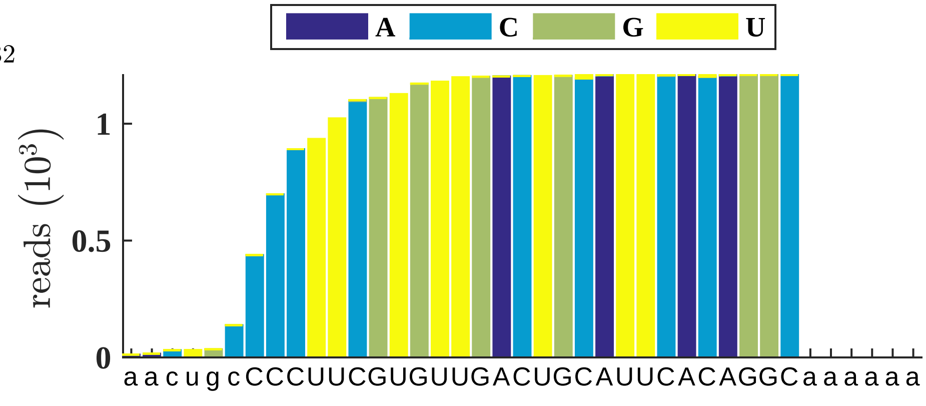

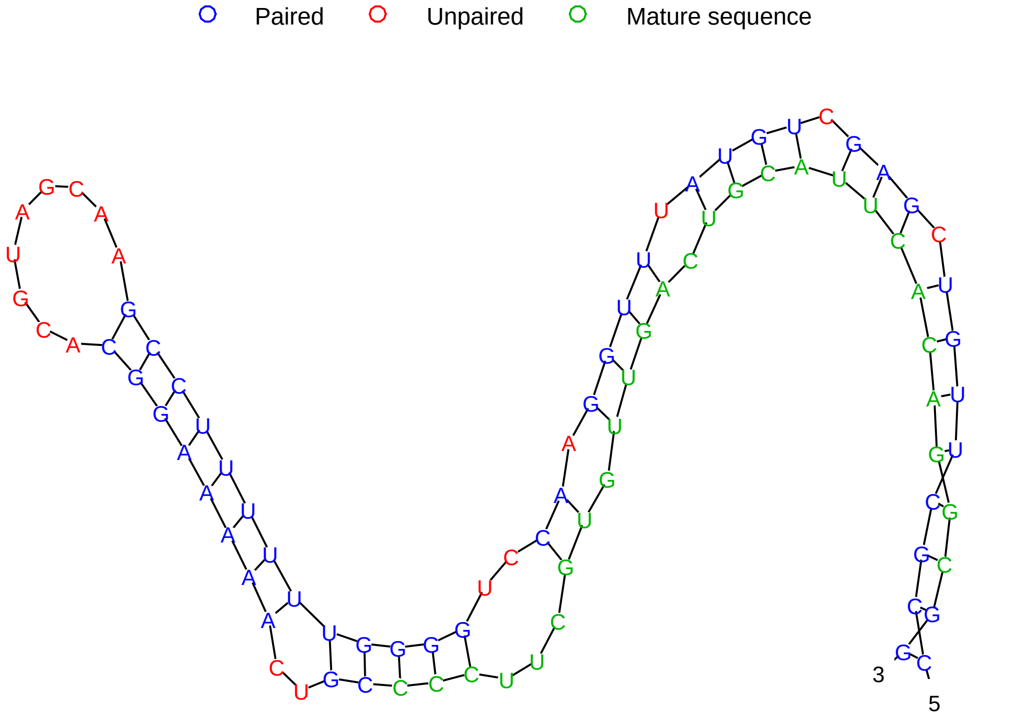

Stem loop (UMD3.1): chr1:153203295-153203384
Mature (UMD3.1): chr1:153203297-153203323
Mature seq len: 27
Total raw counts (9 samples): 1200
Average raw counts: 134
Strand: Reverse
Orientation: 3p
Minimum free energy: -33.20

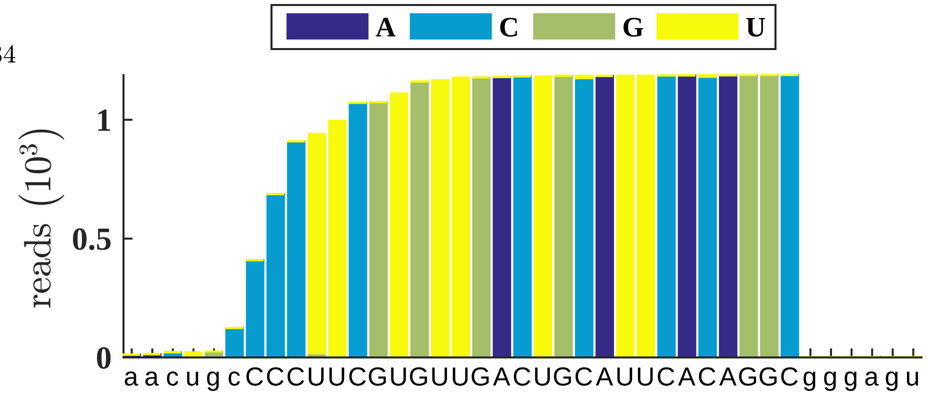

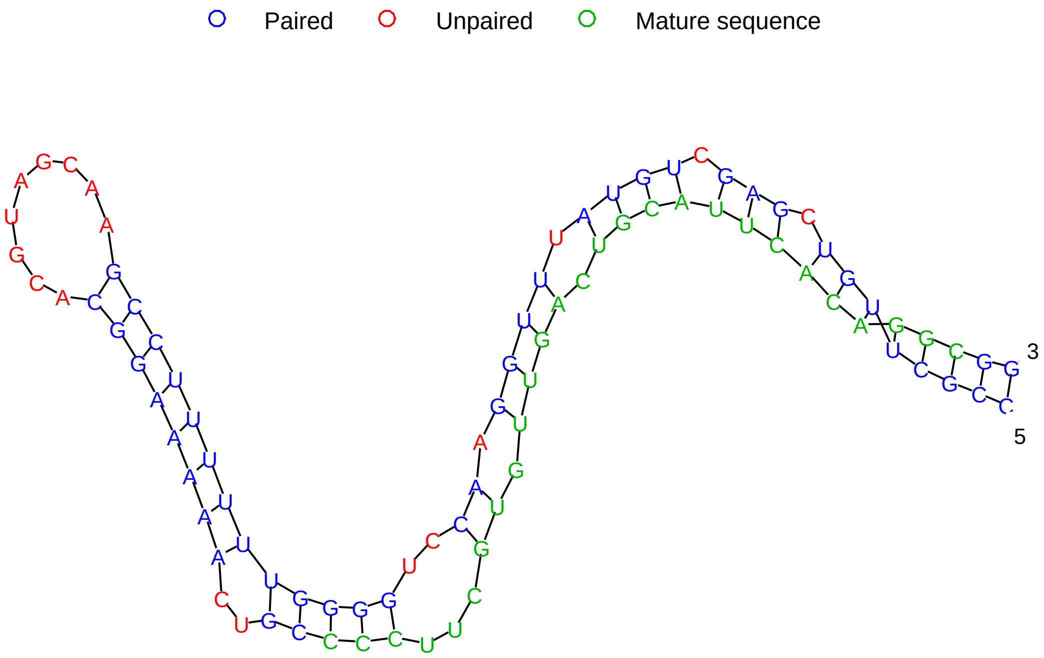

Stem loop (UMD3.1): chr1:153204673-153204762
Mature (UMD3.1): chr1:153204675-153204701
Mature seq len: 27
Total raw counts (9 samples): 1237
Average raw counts: 138
Strand: Reverse
Orientation: 3p
Minimum free energy: -33.20

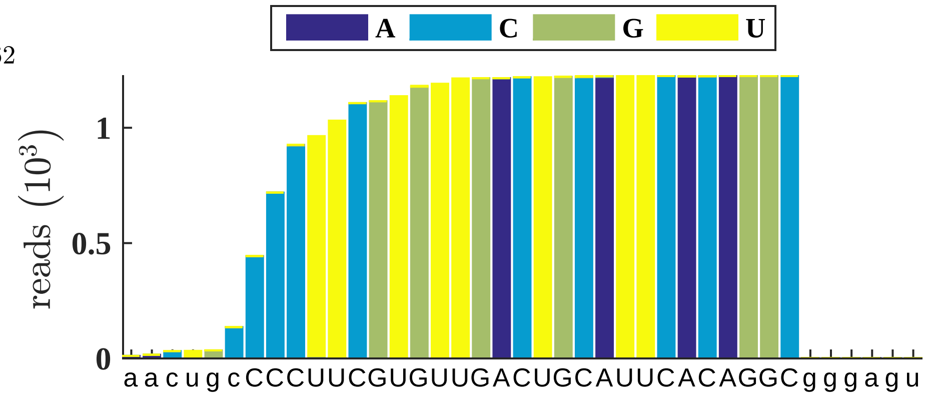

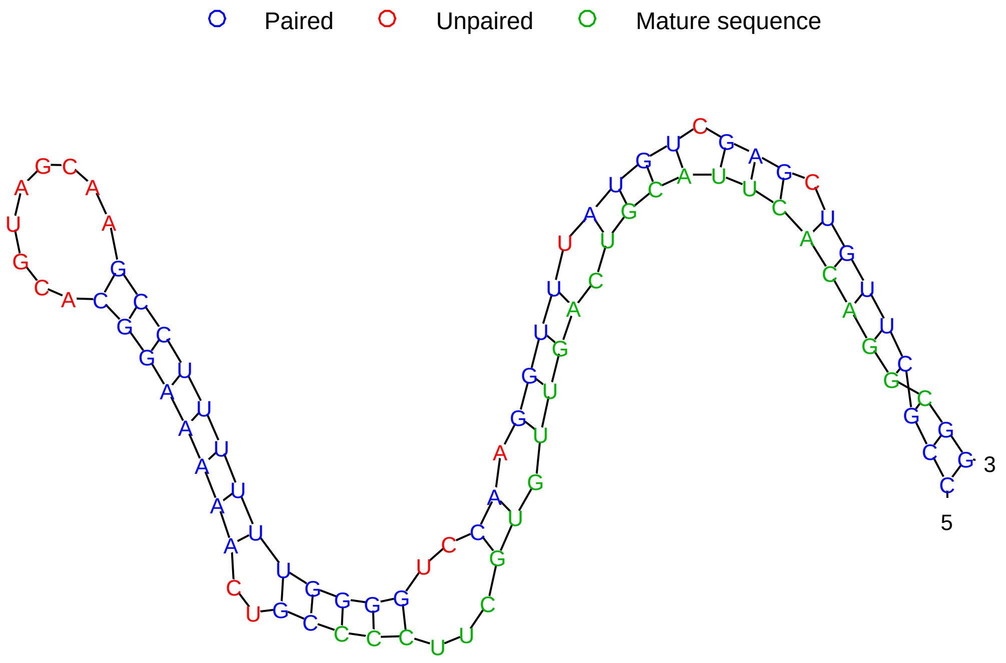

Stem loop (UMD3.1): chr1:153207124-153207213
Mature (UMD3.1): chr1:153207126-153207152
Mature seq len: 27
Total raw counts (9 samples): 1260
Average raw counts: 140
Strand: Reverse
Orientation: 3p
Minimum free energy: -33.20

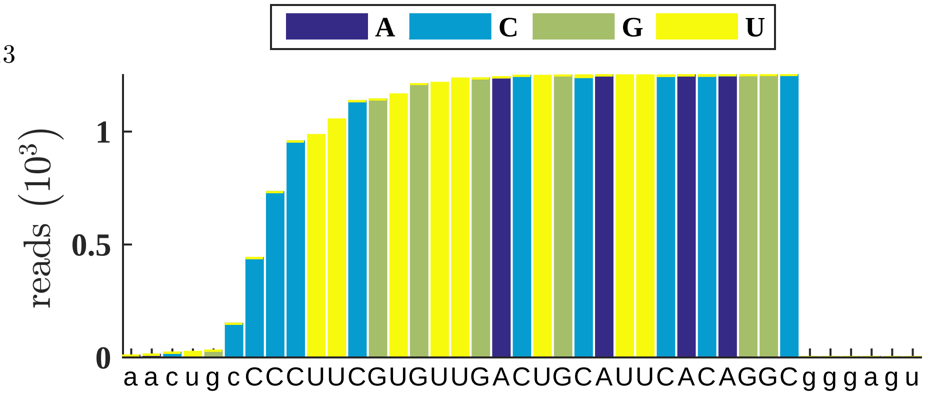

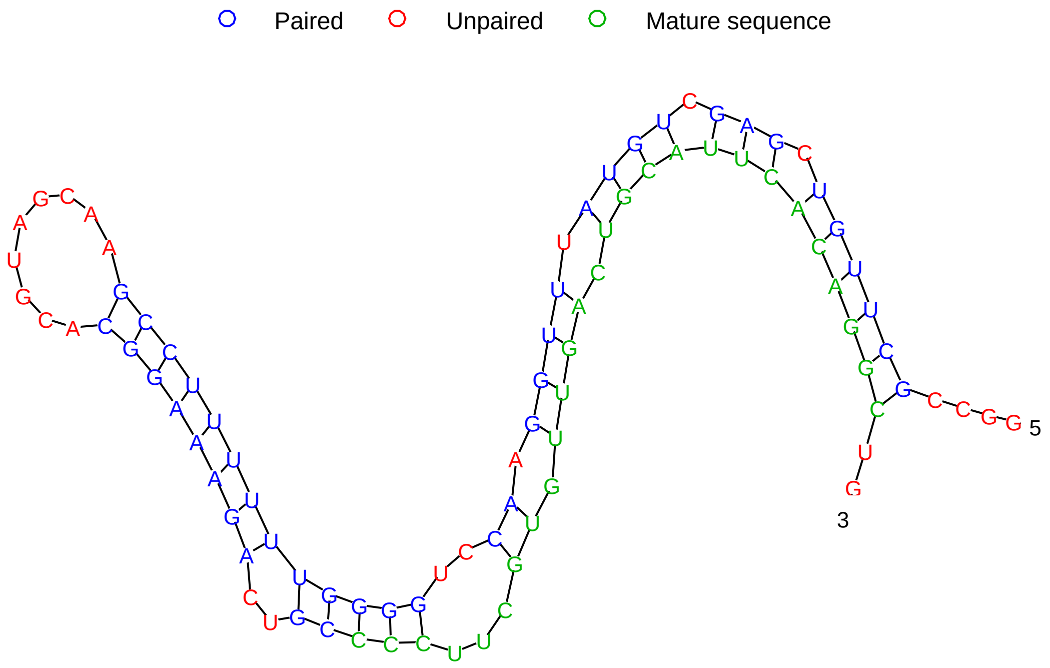

Stem loop (UMD3.1): chr1:153208999-153209090
Mature (UMD3.1): chr1:153209001-153209027
Mature seq len: 27
Total raw counts (9 samples): 1219
Average raw counts: 136
Strand: Reverse
Orientation: 3p
Minimum free energy: -29.00

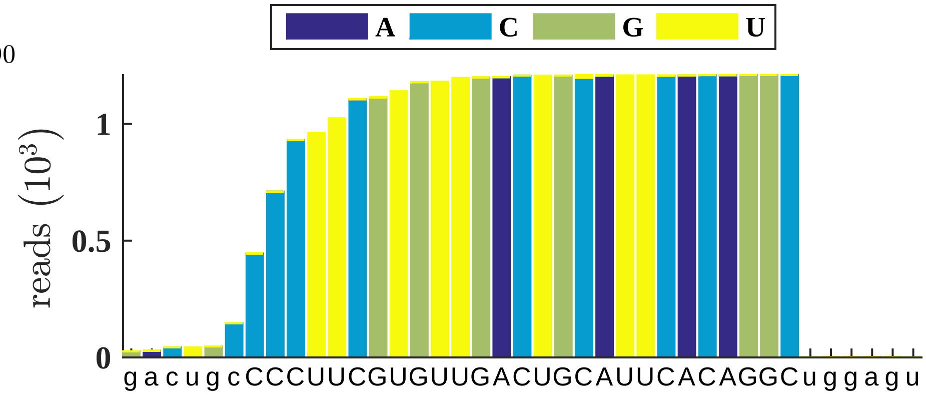

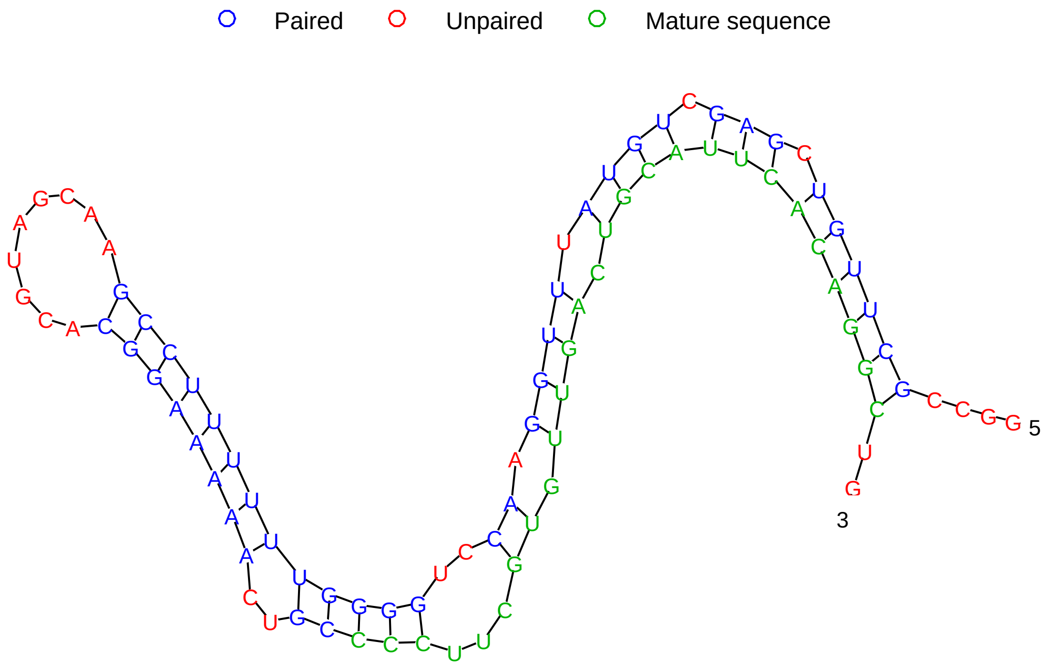

Stem loop (UMD3.1): chr1:153212513-153212604
Mature (UMD3.1): chr1:153212515-153212541
Mature seq len: 27
Total raw counts (9 samples): 1135
Average raw counts: 127
Strand: Reverse
Orientation: 3p
Minimum free energy: -28.90

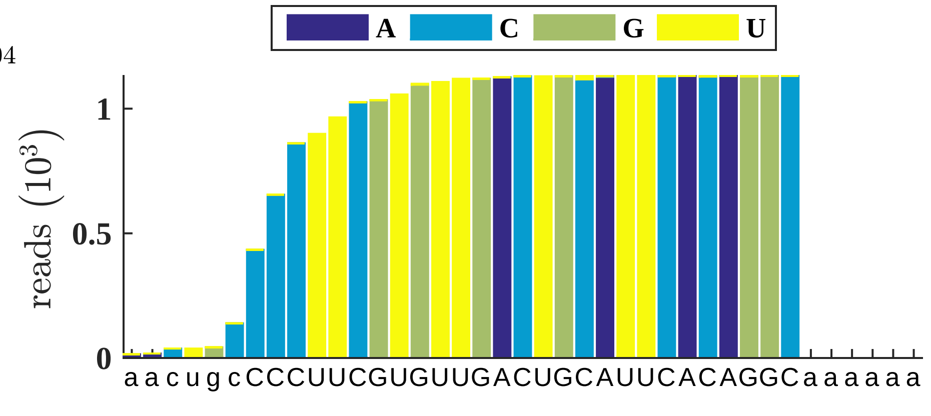

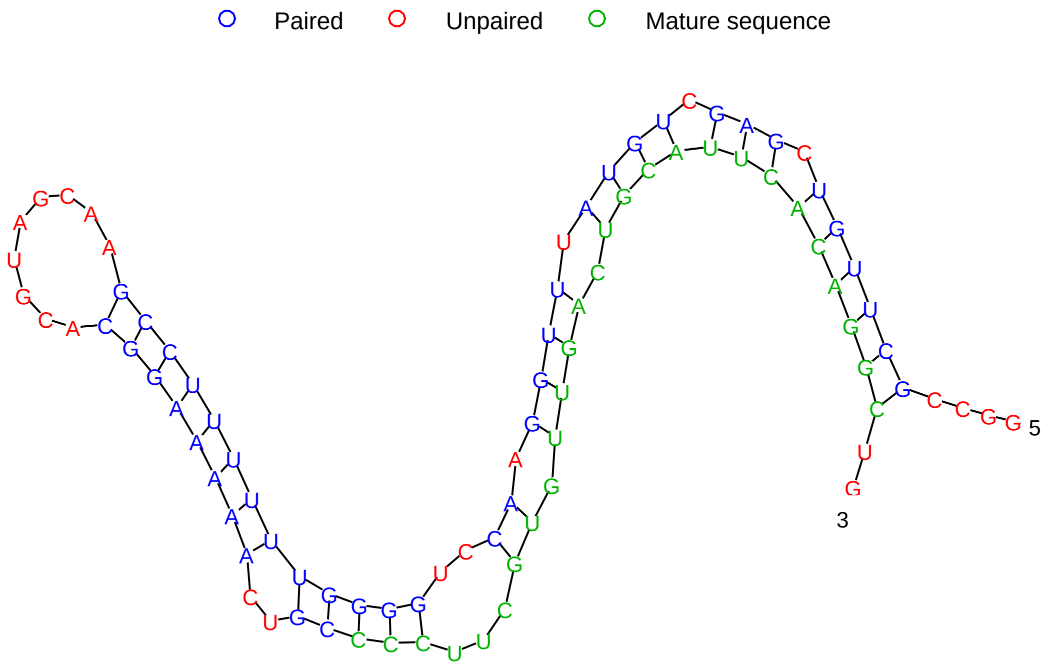

Stem loop (UMD3.1): chr1:153213720-153213811
Mature (UMD3.1): chr1:153213783-153213809
Mature seq len: 27
Total raw counts (9 samples): 1231
Average raw counts: 137
Strand: Forward
Orientation: 3p
Minimum free energy: -28.90

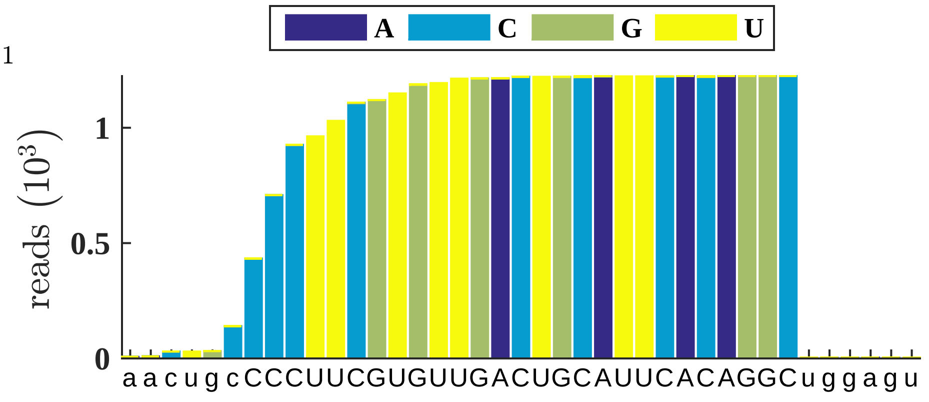

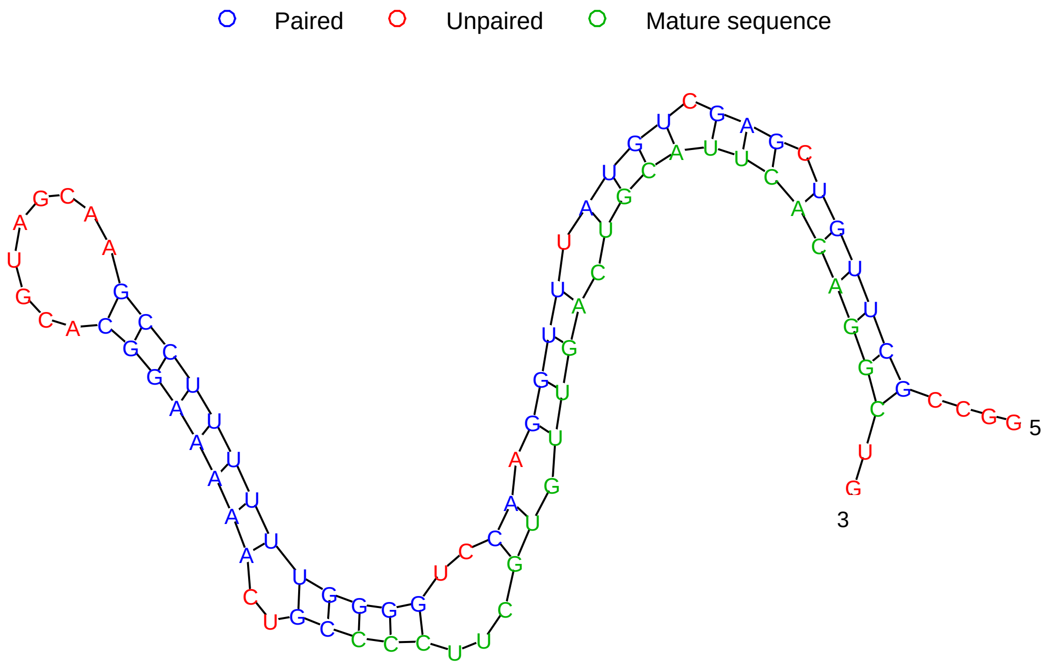

Stem loop (UMD3.1): chr1:153228955-153229046
Mature (UMD3.1): chr1:153229018-153229044
Mature seq len: 27
Total raw counts (9 samples): 1185
Average raw counts: 132
Strand: Forward
Orientation: 3p
Minimum free energy: -28.90

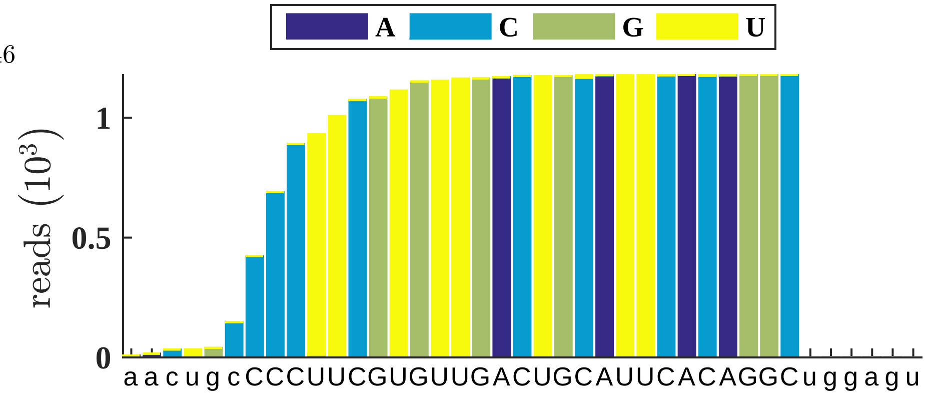

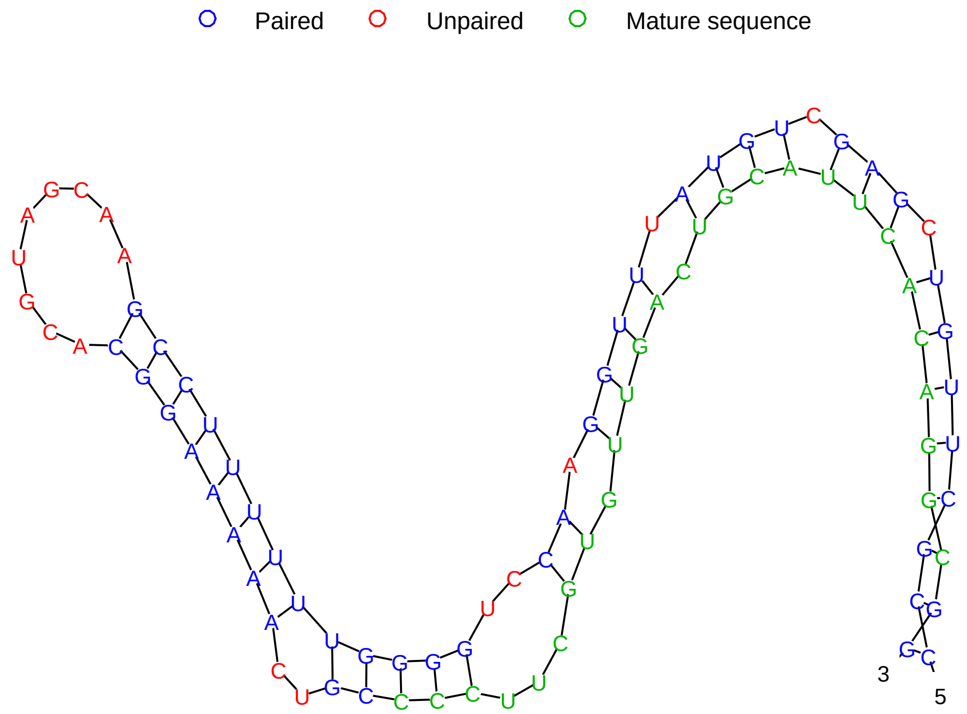

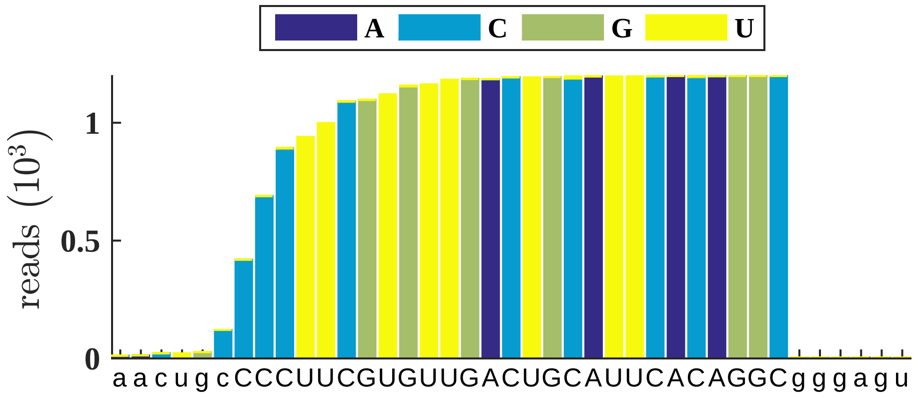

Stem loop (UMD3.1): chr1:34922497-34922586
Mature (UMD3.1): chr1:34922558-34922584
Mature seq len: 27
Total raw counts (9 samples): 1209
Average raw counts: 135
Strand: Forward
Orientation: 3p
Minimum free energy: -33.20

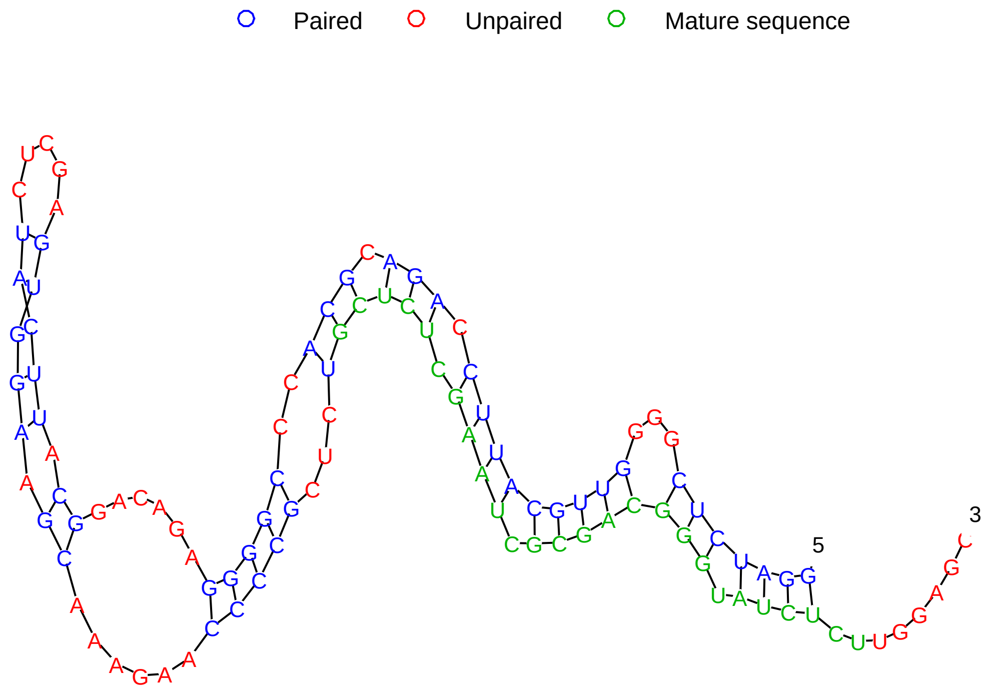

Stem loop (UMD3.1): chr1:5273323-5273430
Mature (UMD3.1): chr1:5273329-5273354
Mature seq len: 26
Total raw counts (9 samples): 3943
Average raw counts: 439
Strand: Reverse
Orientation: 3p
Minimum free energy: -32.40

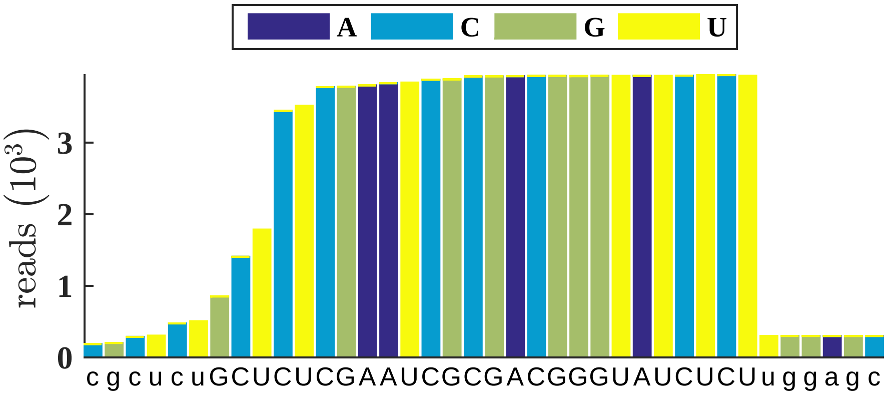

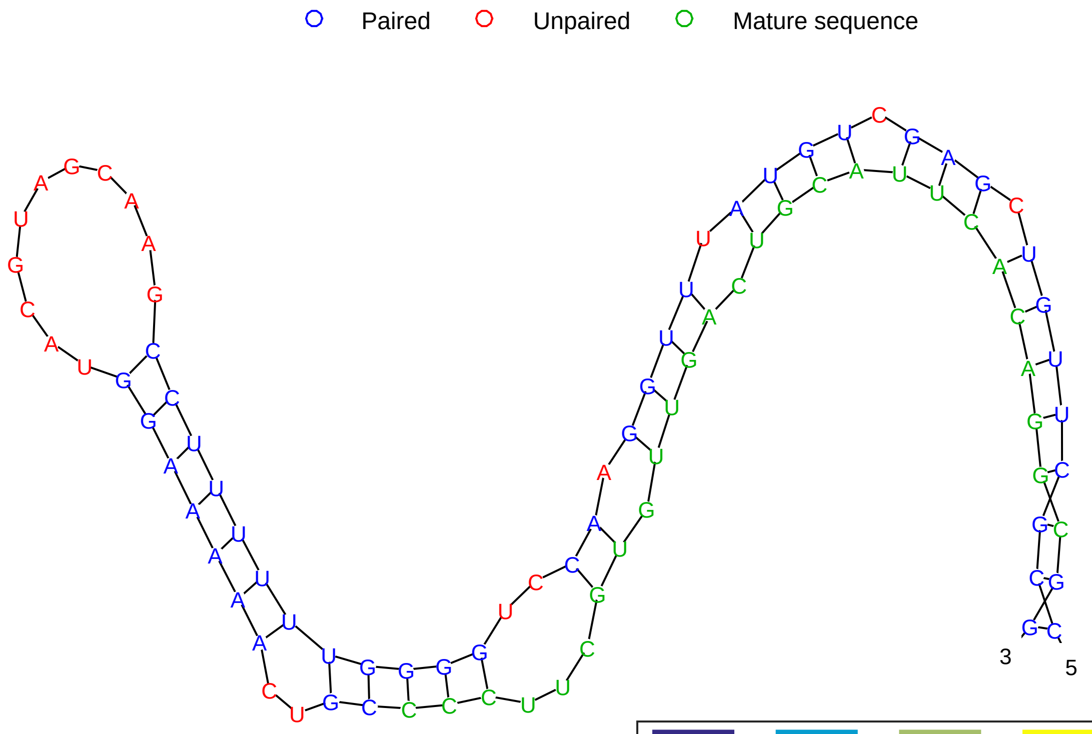

Stem loop (UMD3.1): chr1:57447211-57447300
Mature (UMD3.1): chr1:57447213-57447239
Mature seq len: 27
Total raw counts (9 samples): 1186
Average raw counts: 132
Strand: Reverse
Orientation: 3p
Minimum free energy: -31.00

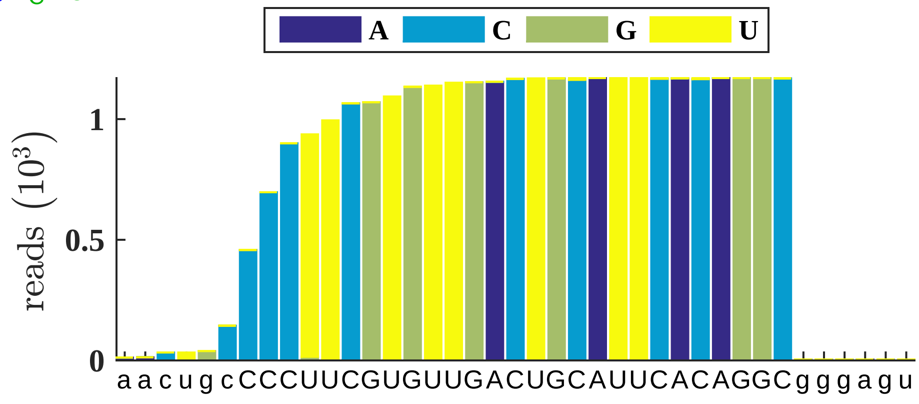

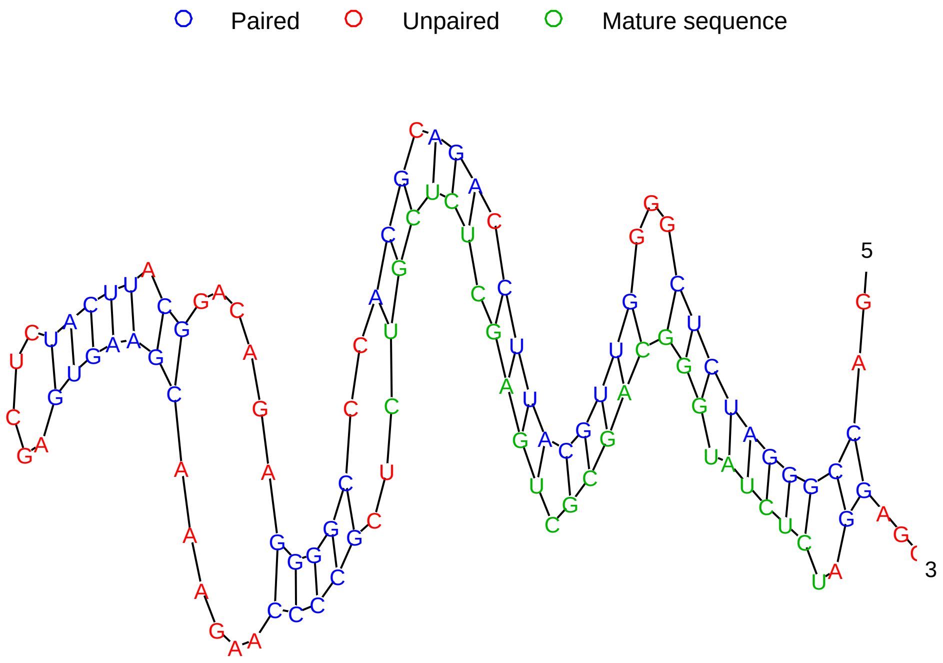

Stem loop (UMD3.1): chr1:57470917-57471028
Mature (UMD3.1): chr1:57470923-57470950
Mature seq len: 28
Total raw counts (9 samples): 13105
Average raw counts: 1457
Strand: Reverse
Orientation: 3p
Minimum free energy: -35.90

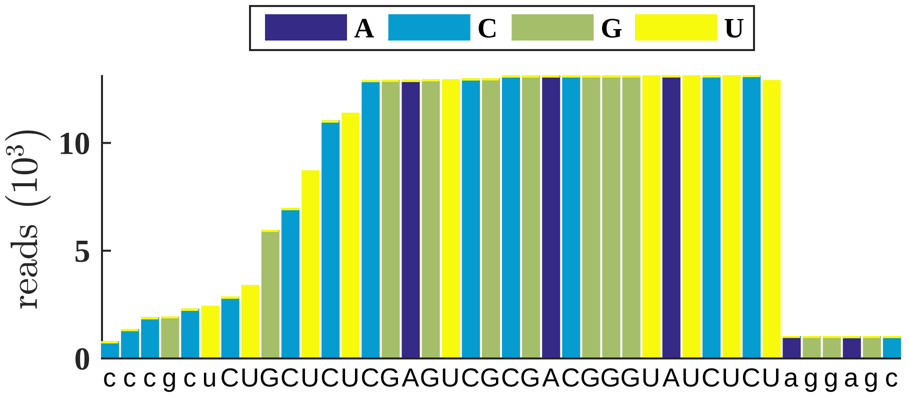

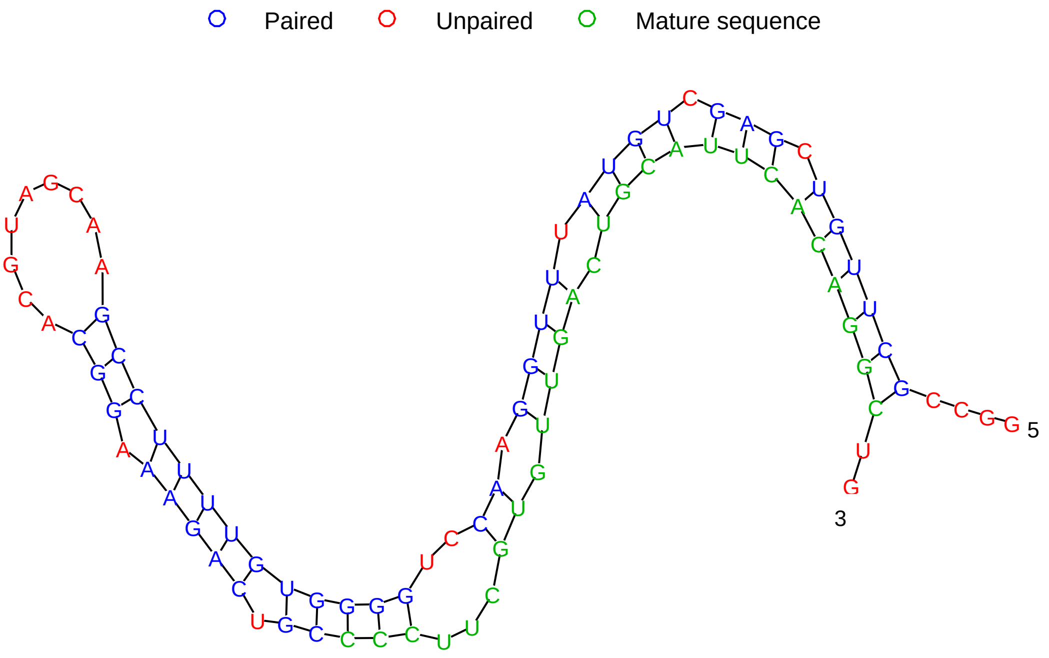

Stem loop (UMD3.1): chr1:57482101-57482192
Mature (UMD3.1): chr1:57482164-57482190
Mature seq len: 27
Total raw counts (9 samples): 1207
Average raw counts: 135
Strand: Forward
Orientation: 3p
Minimum free energy: -27.90

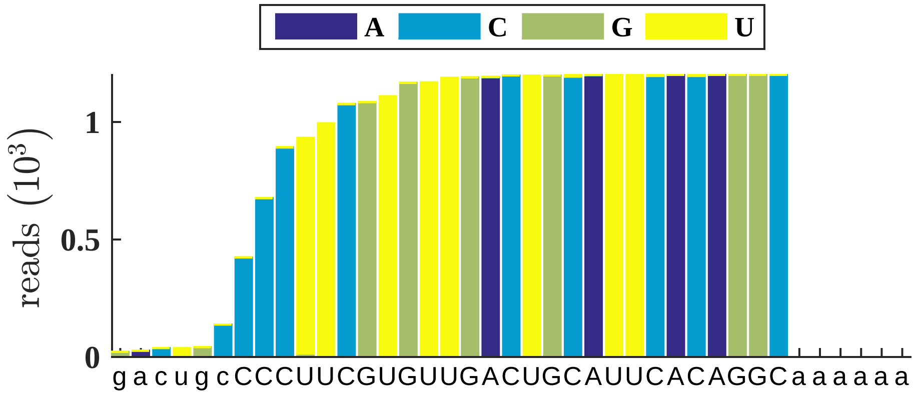

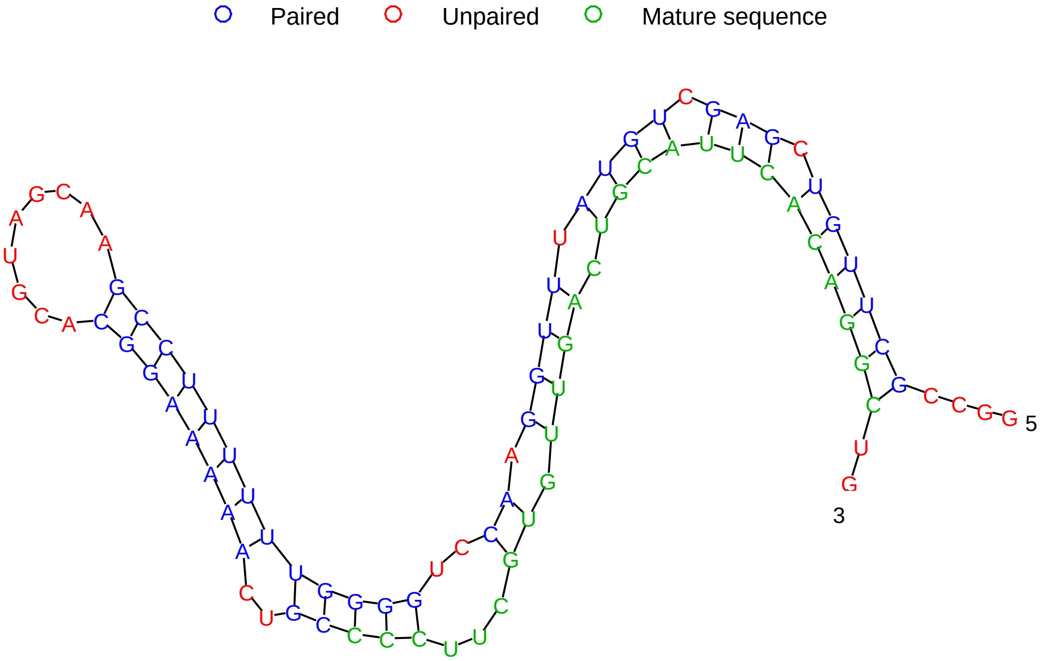

Stem loop (UMD3.1): chr1:65323748-65323839
Mature (UMD3.1): chr1:65323750-65323776
Mature seq len: 27
Total raw counts (9 samples): 1257
Average raw counts: 140
Strand: Reverse
Orientation: 3p
Minimum free energy: -28.90

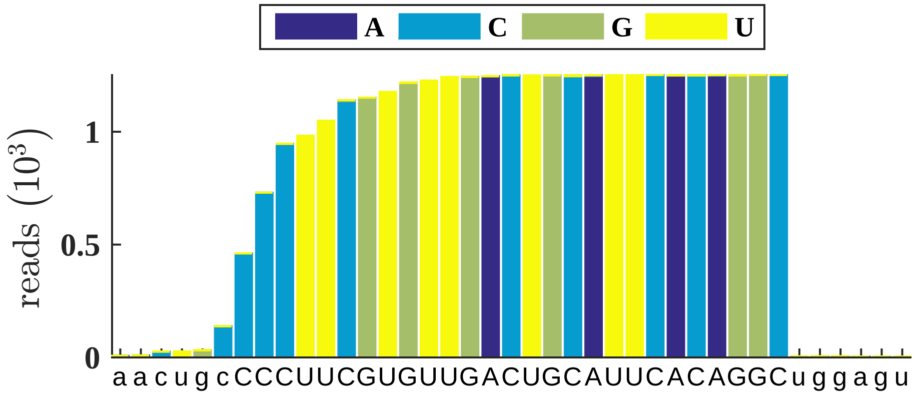

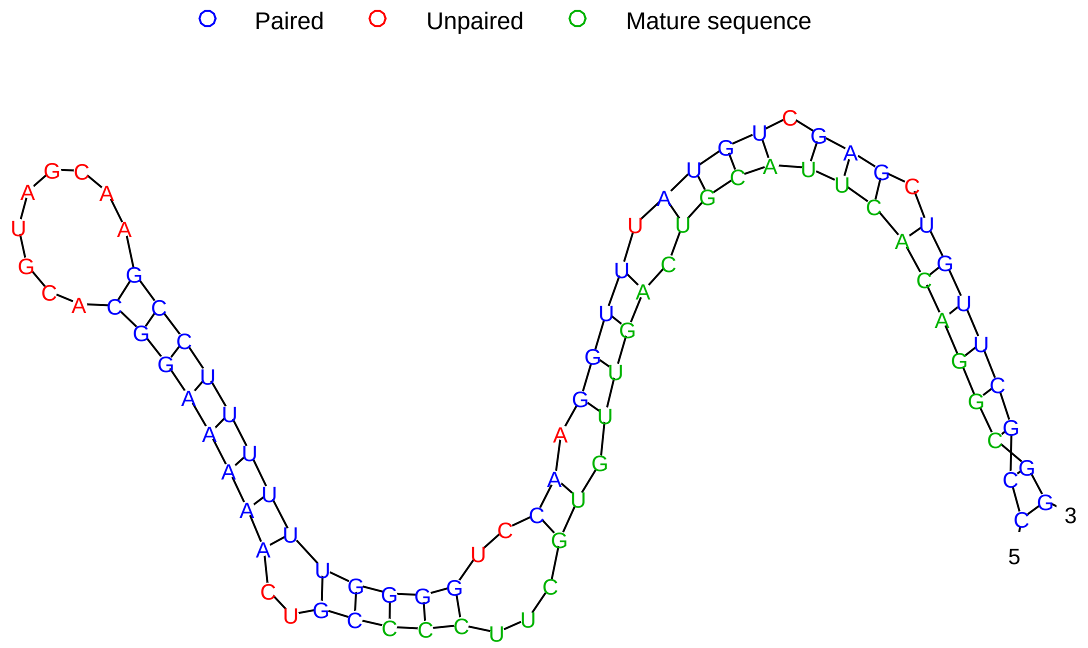

Stem loop (UMD3.1): chr1:84804565-84804654
Mature (UMD3.1): chr1:84804567-84804593
Mature seq len: 27
Total raw counts (9 samples): 1241
Average raw counts: 138
Strand: Reverse
Orientation: 3p
Minimum free energy: -33.20

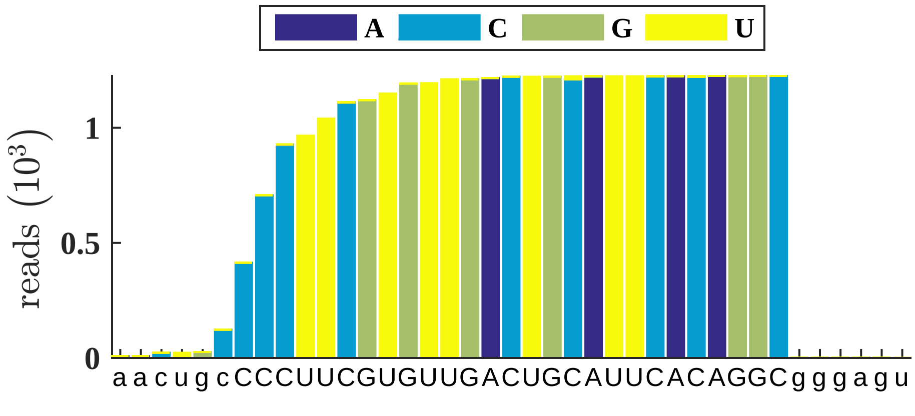

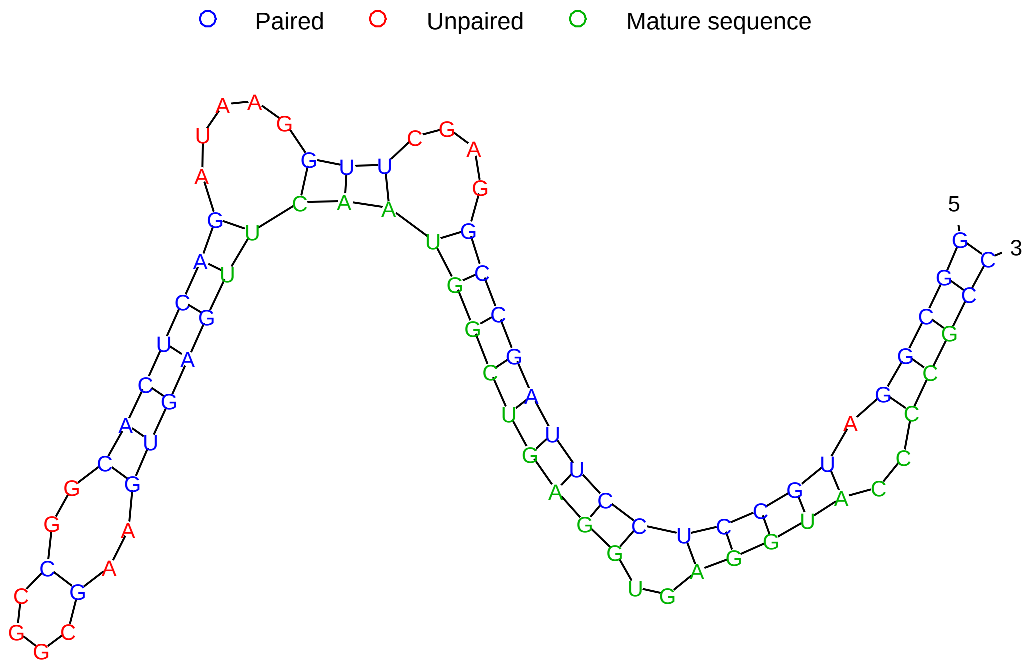

Stem loop (UMD3.1): chr10:26814466-26814547
Mature (UMD3.1): chr10:26814520-26814545
Mature seq len: 26
Total raw counts (9 samples): 6835
Average raw counts: 760
Strand: Forward
Orientation: 3p
Minimum free energy: -35.10

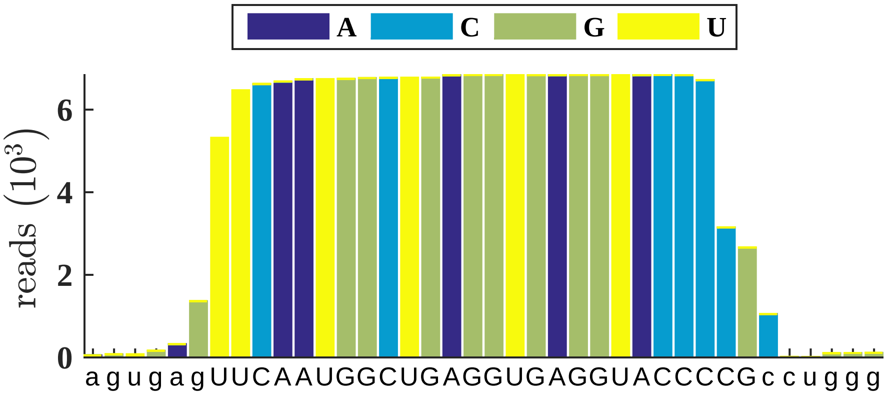

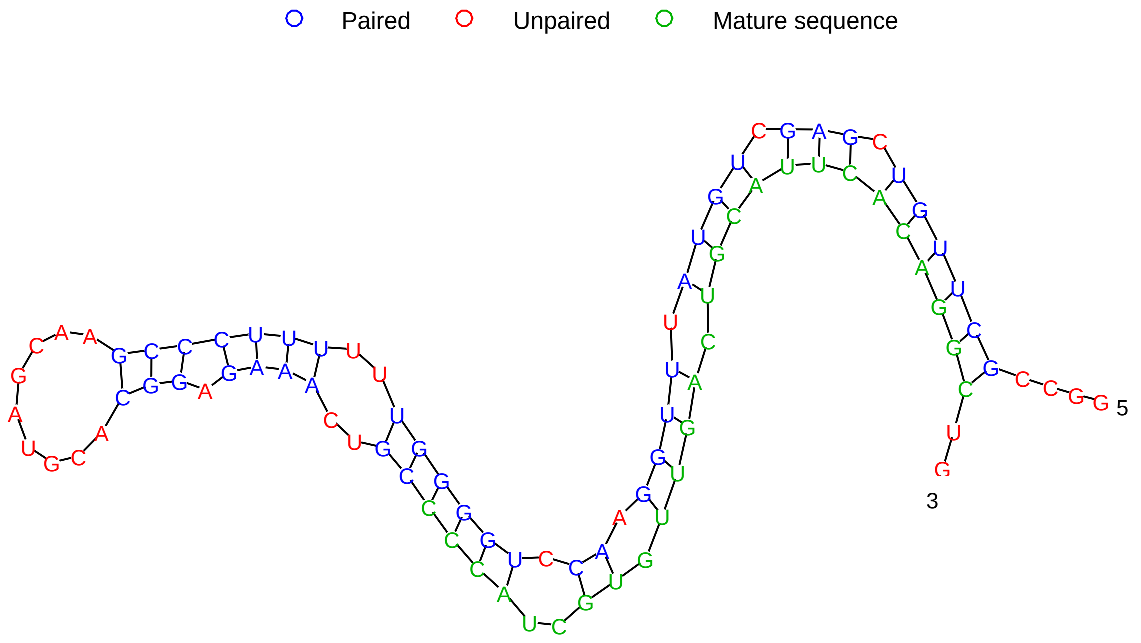

Stem loop (UMD3.1): chr10:287464-287556
Mature (UMD3.1): chr10:287528-287554
Mature seq len: 27
Total raw counts (9 samples): 939
Average raw counts: 105
Strand: Forward
Orientation: 3p
Minimum free energy: -29.20

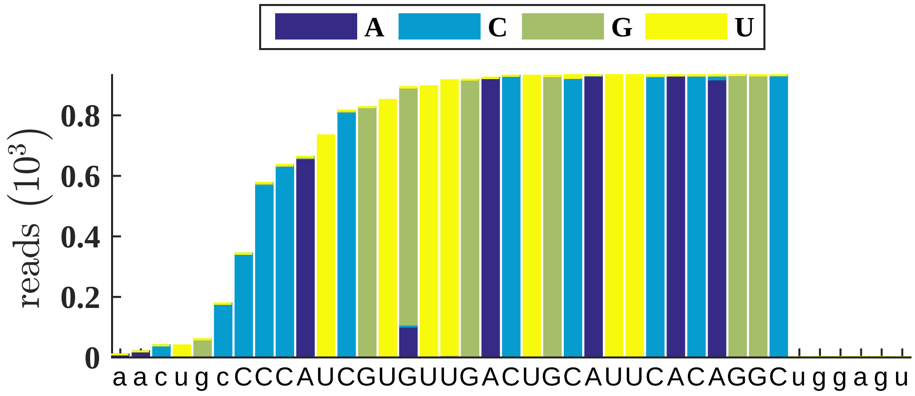

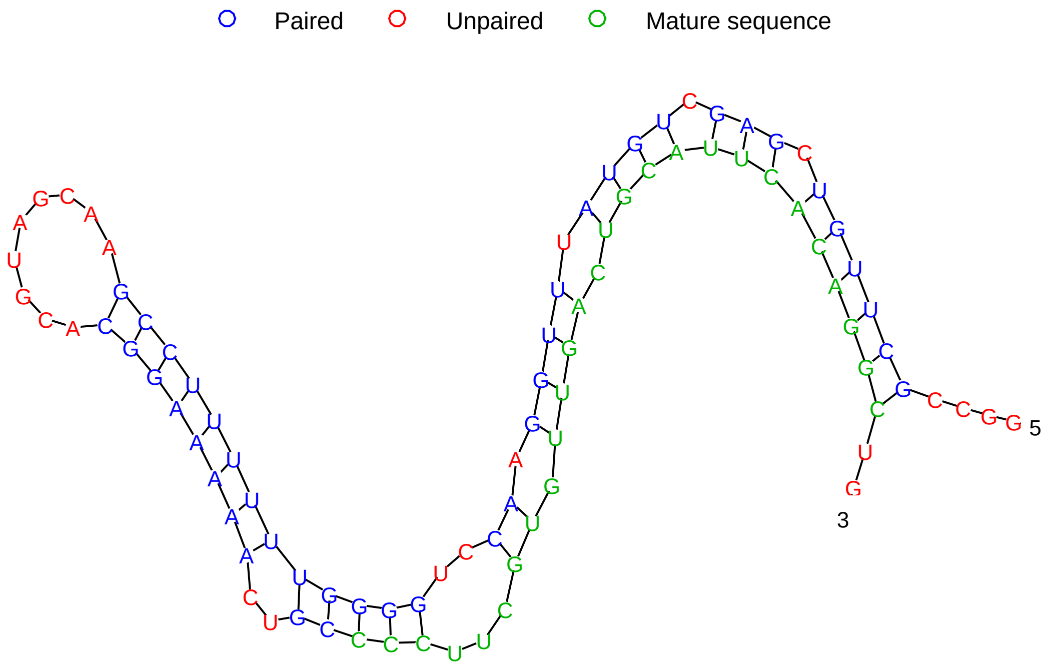

Stem loop (UMD3.1): chr10:39688733-39688824
Mature (UMD3.1): chr10:39688735-39688761
Mature seq len: 27
Total raw counts (9 samples): 1189
Average raw counts: 133
Strand: Reverse
Orientation: 3p
Minimum free energy: -28.90

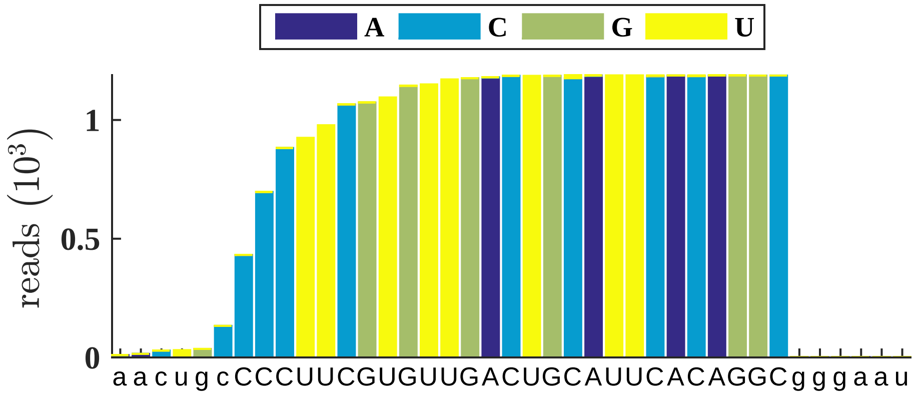

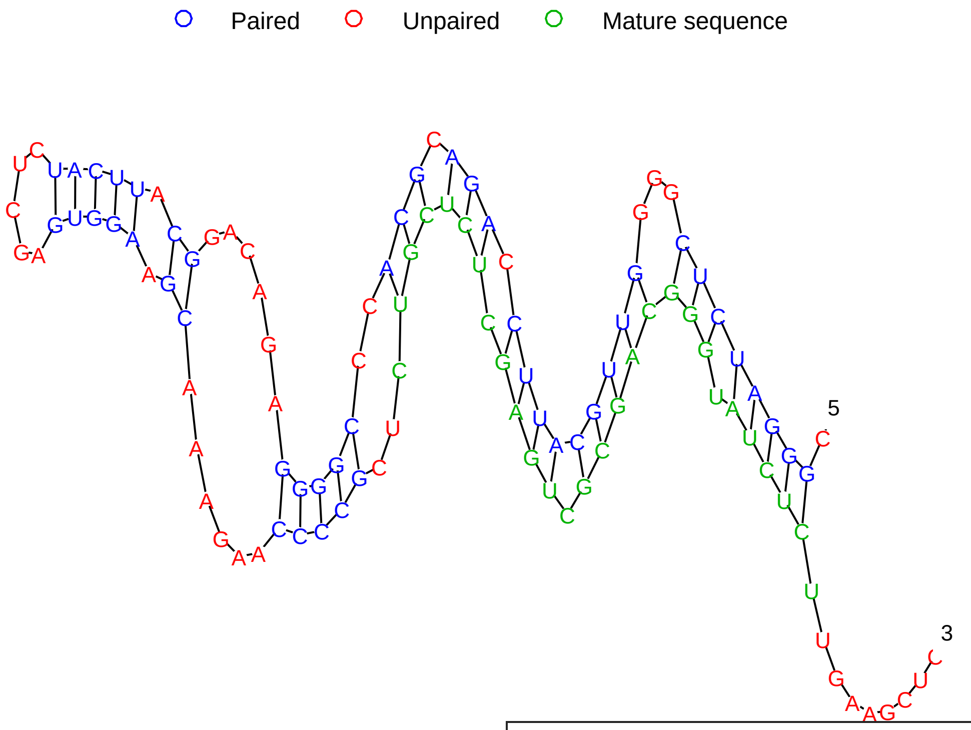

Stem loop (UMD3.1): chr10:59501651-59501762
Mature (UMD3.1): chr10:59501659-59501686
Mature seq len: 28
Total raw counts (9 samples): 12974
Average raw counts: 1442
Strand: Reverse
Orientation: 3p
Minimum free energy: -35.70

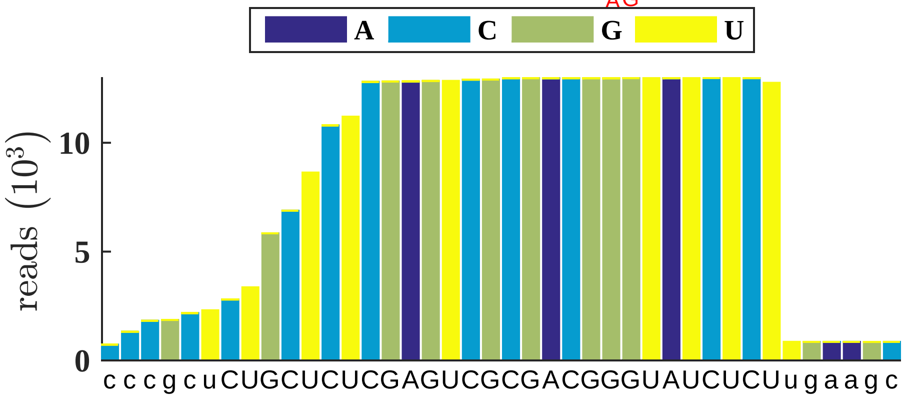

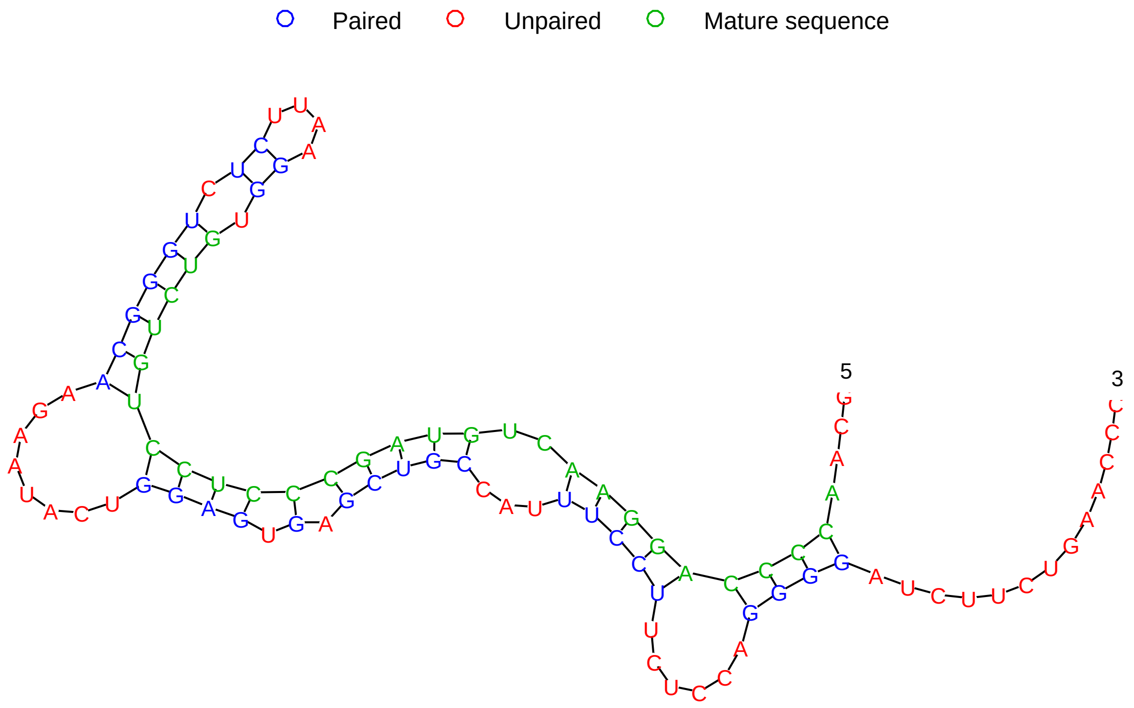

Stem loop (UMD3.1): chr10:61715282-61715379
Mature (UMD3.1): chr10:61715349-61715376
Mature seq len: 28
Total raw counts (9 samples): 7200
Average raw counts: 800
Strand: Reverse
Orientation: 5p
Minimum free energy: -26.10

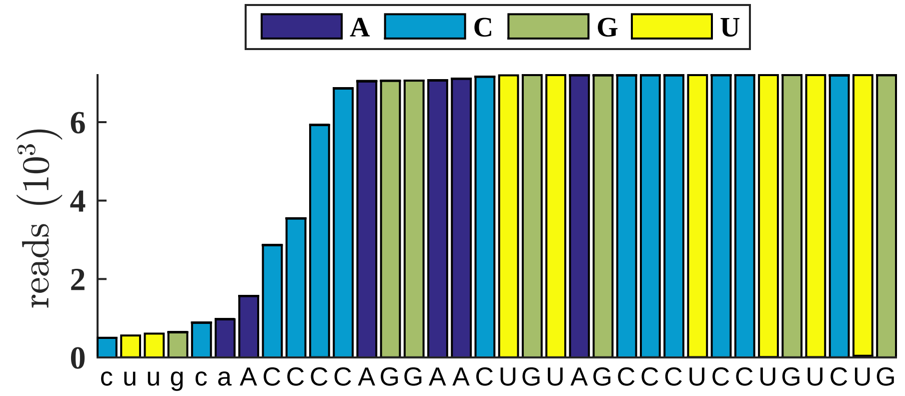

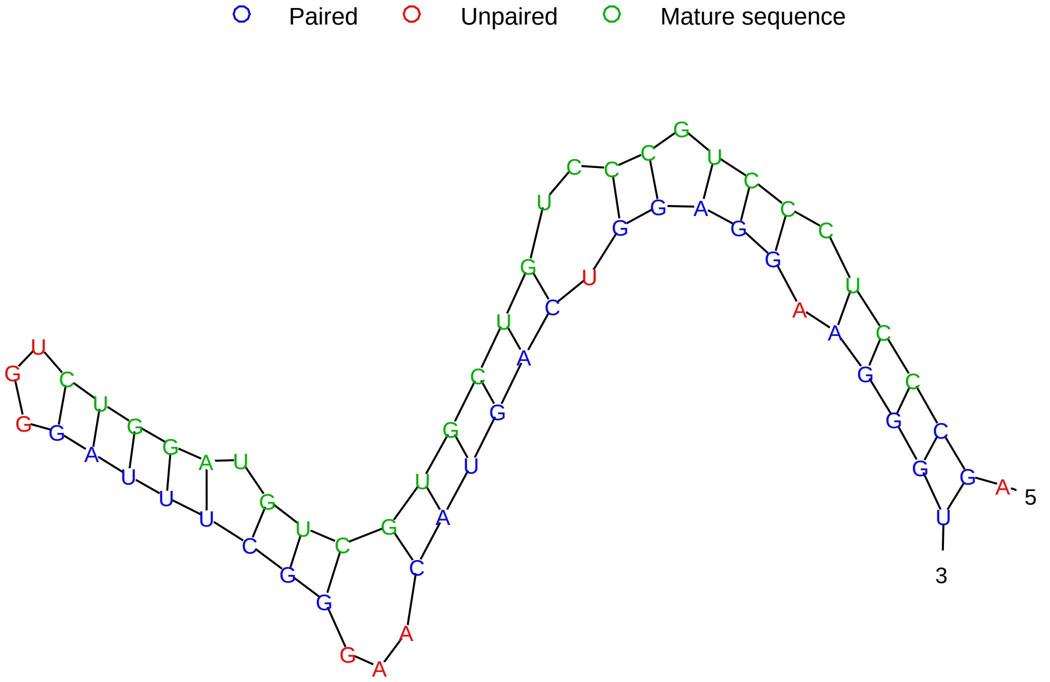

Stem loop (UMD3.1): chr11:100999575-100999636
Mature (UMD3.1): chr11:100999578-100999604
Mature seq len: 27
Total raw counts (9 samples): 612
Average raw counts: 68
Strand: Forward
Orientation: 5p
Minimum free energy: -23.50

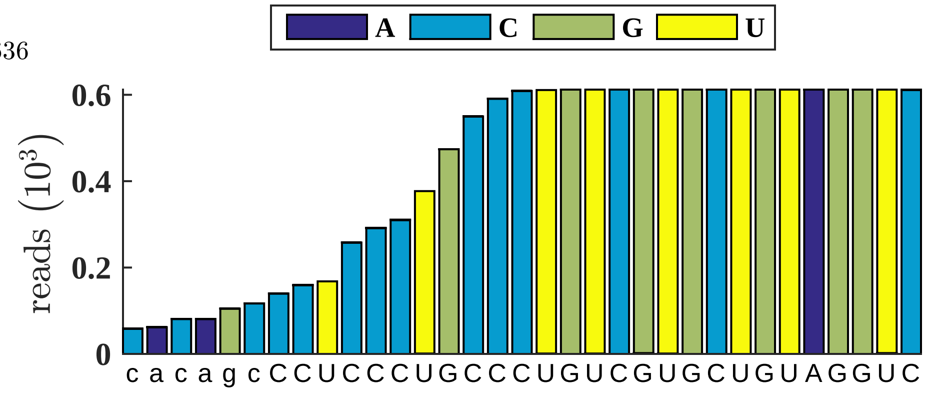

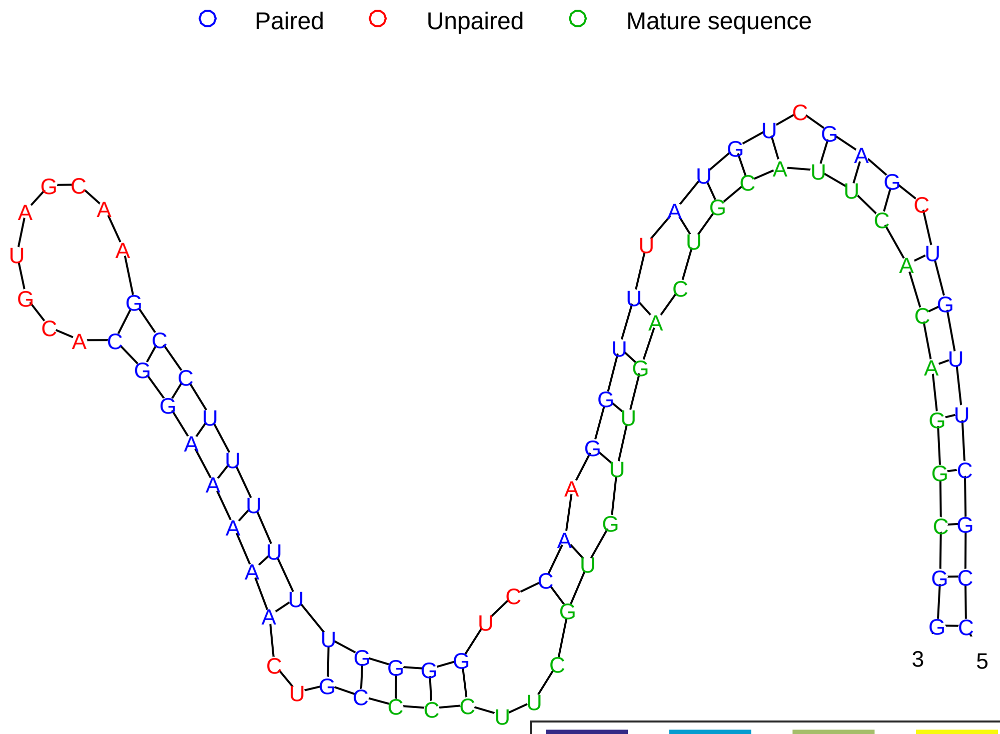

Stem loop (UMD3.1): chr11:70172380-70172469
Mature (UMD3.1): chr11:70172441-70172467
Mature seq len: 27
Total raw counts (9 samples): 1191
Average raw counts: 133
Strand: Forward
Orientation: 3p
Minimum free energy: -33.20

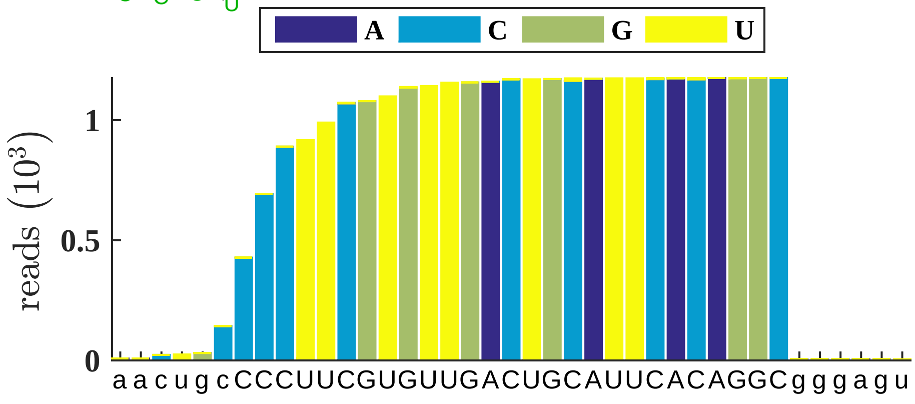

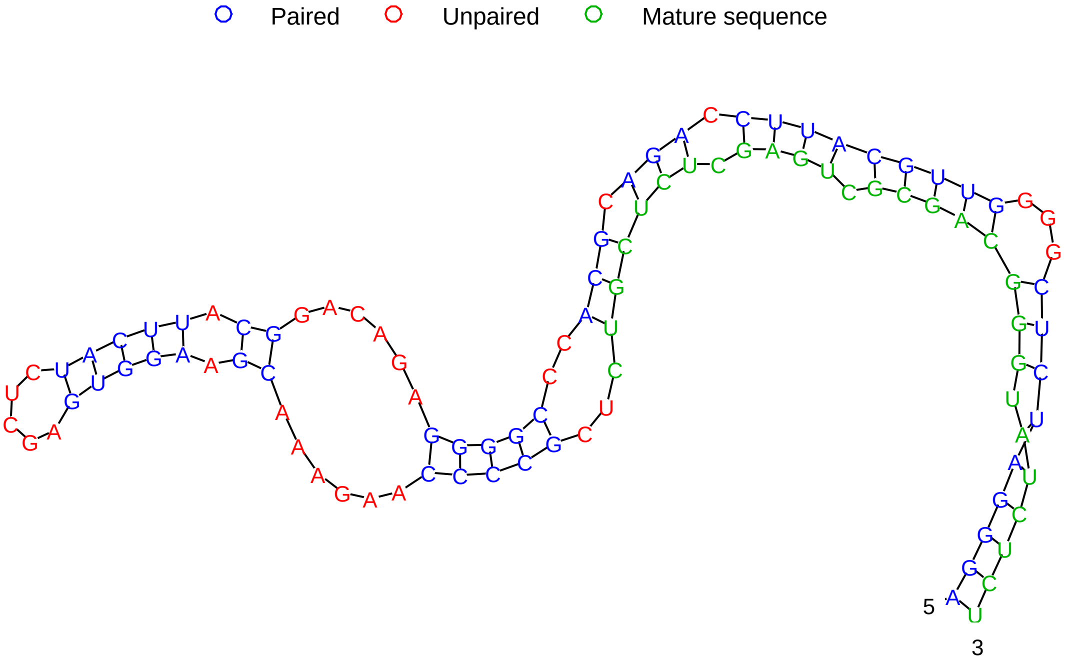

Stem loop (UMD3.1): chr11:70173058-70173161
Mature (UMD3.1): chr11:70173134-70173161
Mature seq len: 28
Total raw counts (9 samples): 12112
Average raw counts: 1346
Strand: Forward
Orientation: 3p
Minimum free energy: -35.90

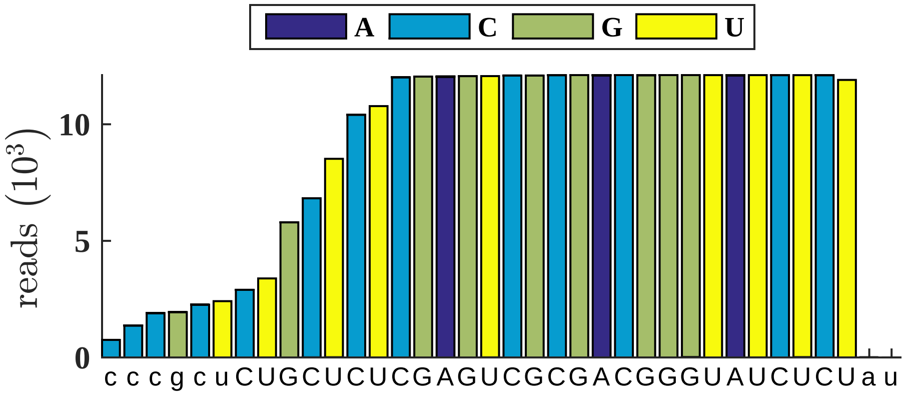

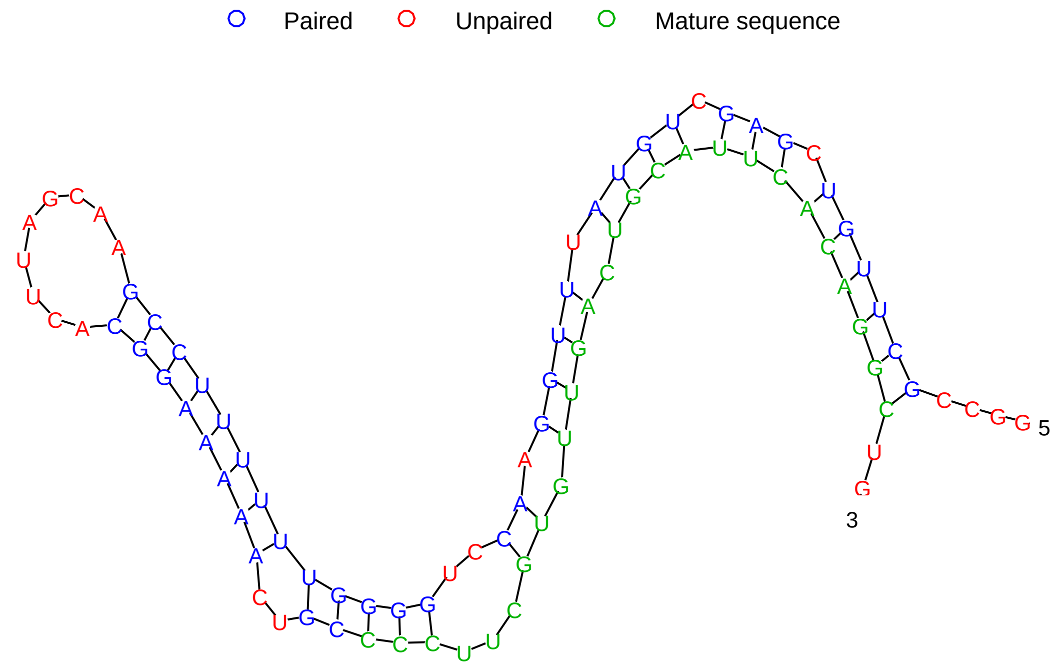

Stem loop (UMD3.1): chr11:70173371-70173462
Mature (UMD3.1): chr11:70173434-70173460
Mature seq len: 27
Total raw counts (9 samples): 1141
Average raw counts: 127
Strand: Forward
Orientation: 3p
Minimum free energy: -28.90

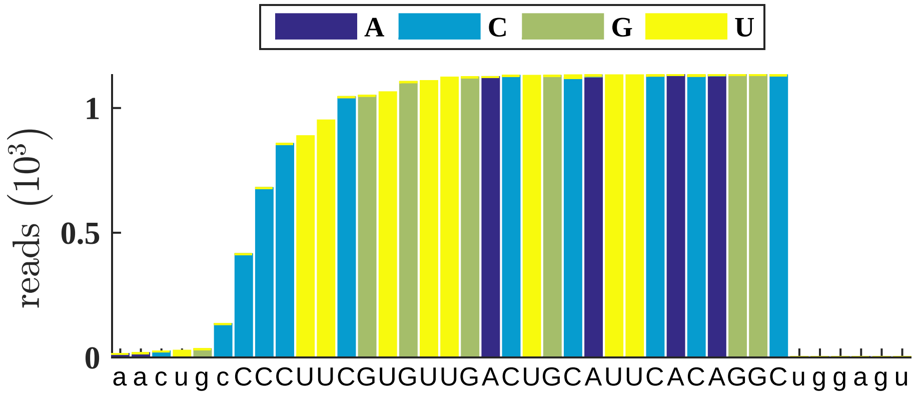

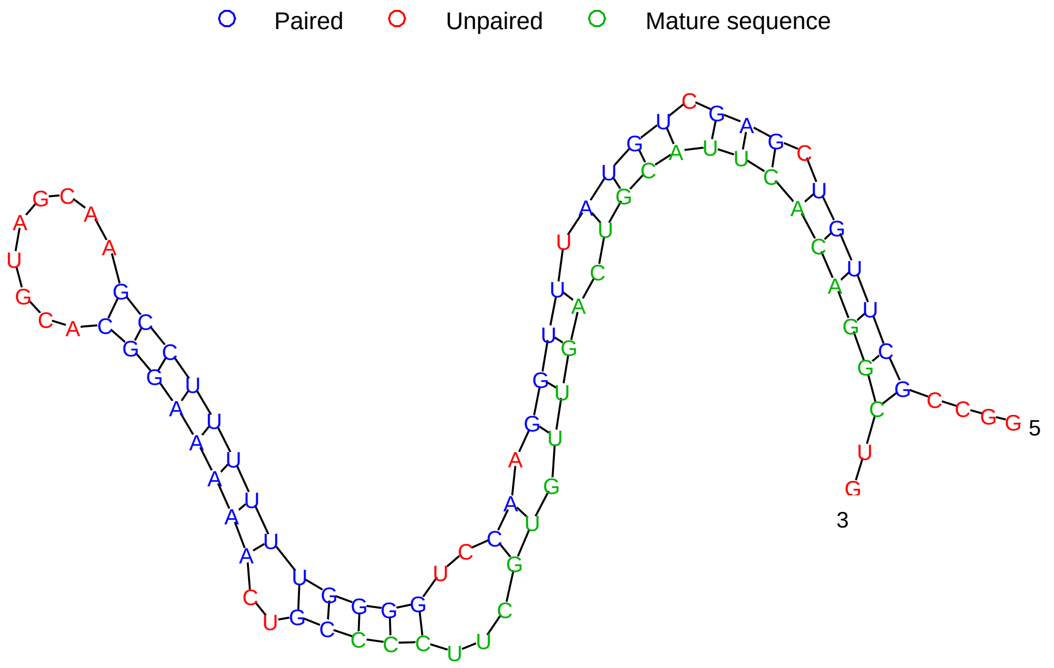

Stem loop (UMD3.1): chr11:70176132-70176223
Mature (UMD3.1): chr11:70176195-70176221
Mature seq len: 27
Total raw counts (9 samples): 1221
Average raw counts: 136
Strand: Forward
Orientation: 3p
Minimum free energy: -28.90

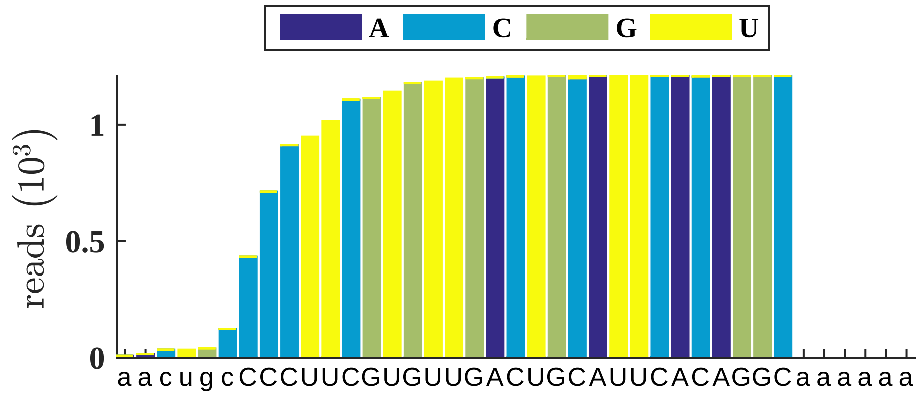

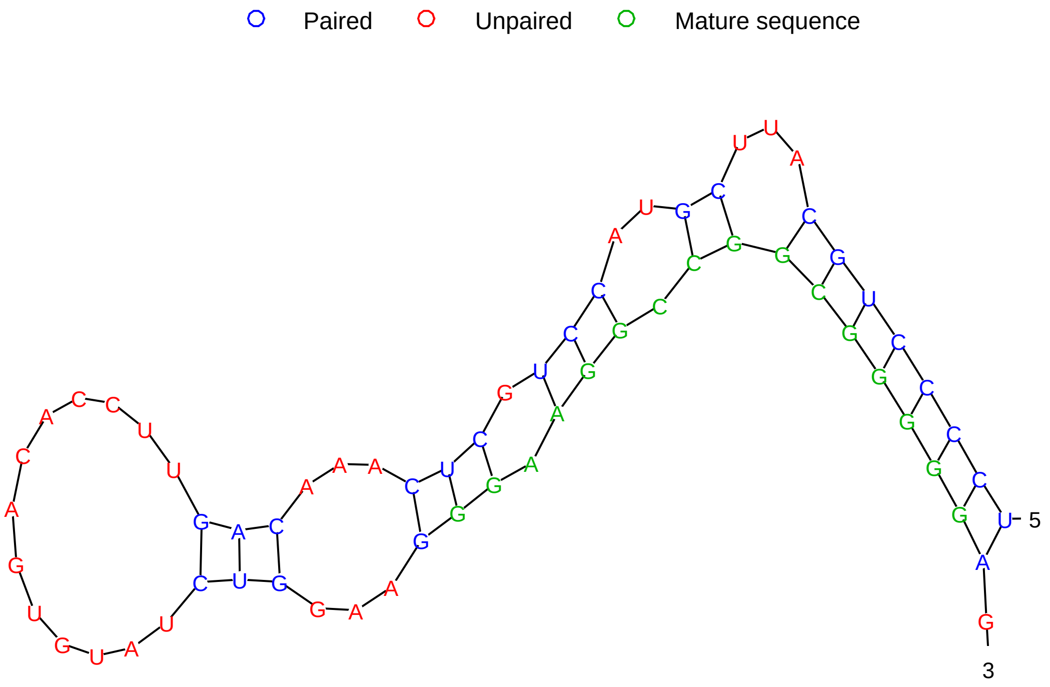

Stem loop (UMD3.1): chr11:72567760-72567825
Mature (UMD3.1): chr11:72567808-72567823
Mature seq len: 16
Total raw counts (9 samples): 1928
Average raw counts: 215
Strand: Forward
Orientation: 3p
Minimum free energy: -22.20

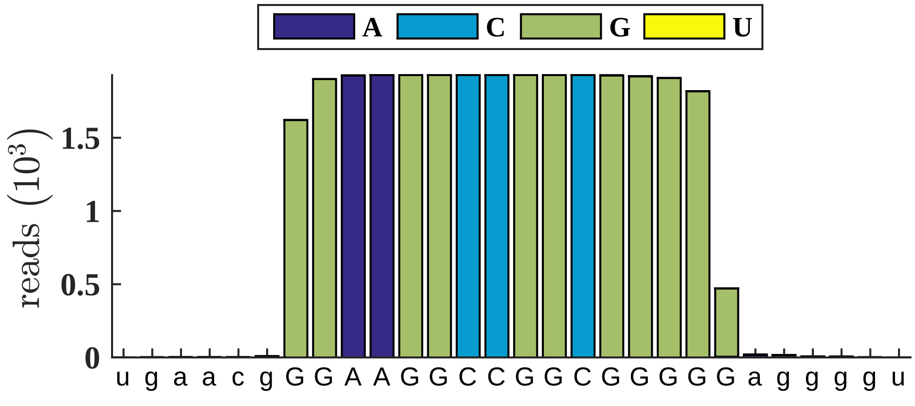

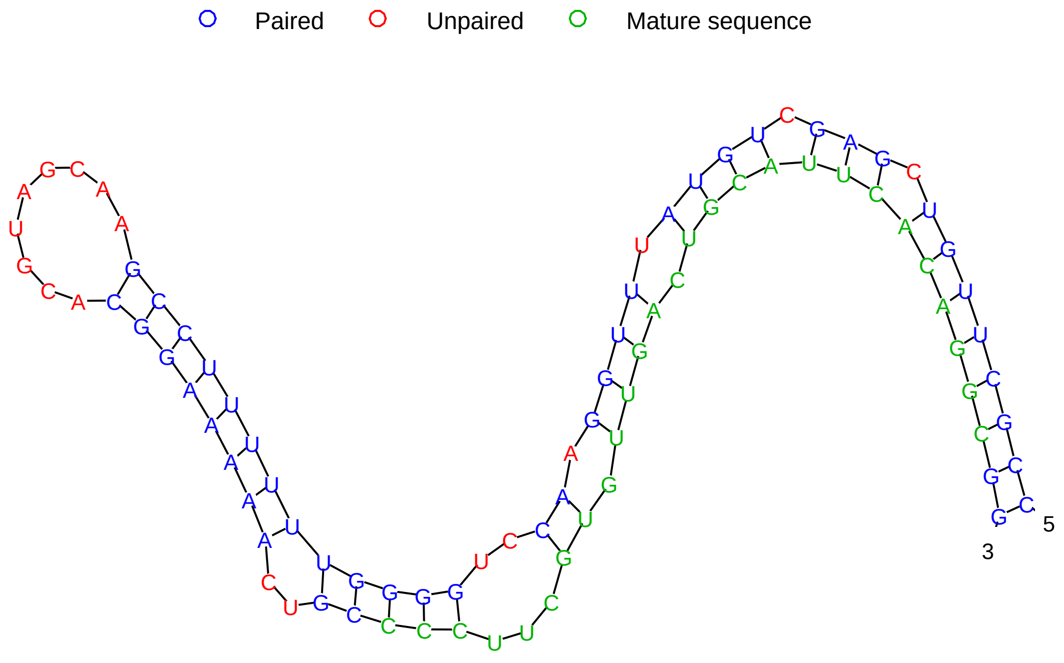

Stem loop (UMD3.1): chr11:79572223-79572312
Mature (UMD3.1): chr11:79572225-79572251
Mature seq len: 27
Total raw counts (9 samples): 1219
Average raw counts: 136
Strand: Reverse
Orientation: 3p
Minimum free energy: -33.20

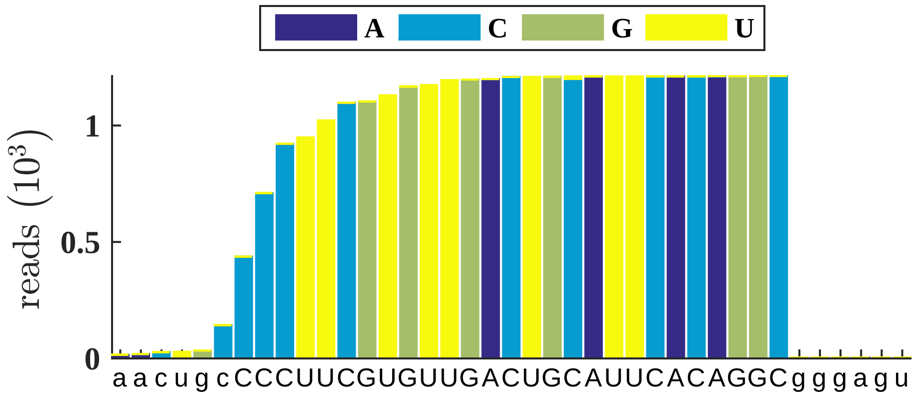

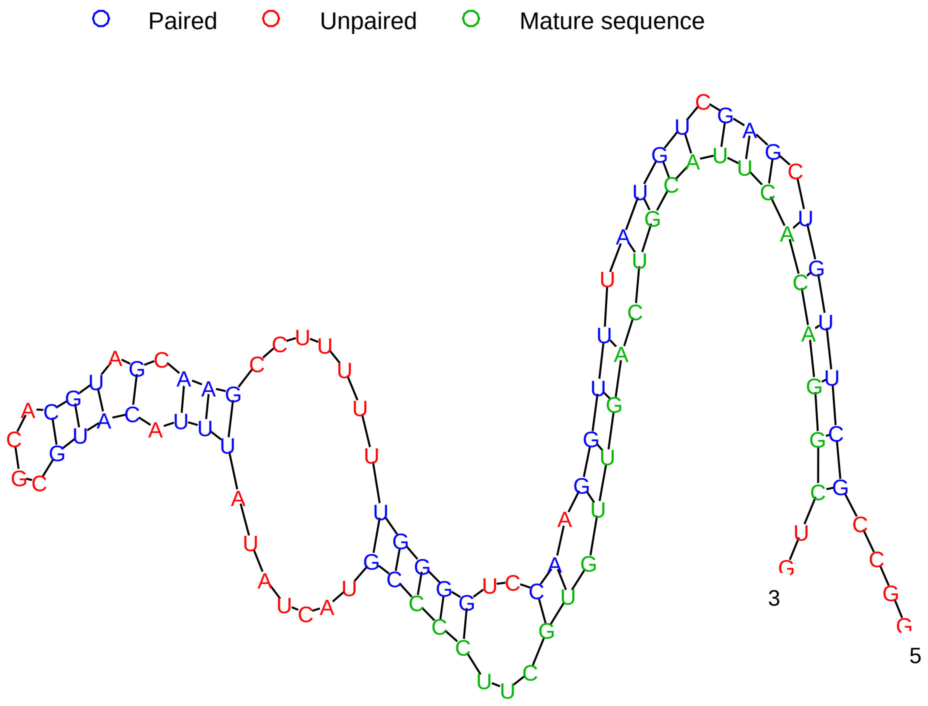

Stem loop (UMD3.1): chr11:79833941-79834040
Mature (UMD3.1): chr11:79834012-79834038
Mature seq len: 27
Total raw counts (9 samples): 1110
Average raw counts: 124
Strand: Forward
Orientation: 3p
Minimum free energy: -21.40

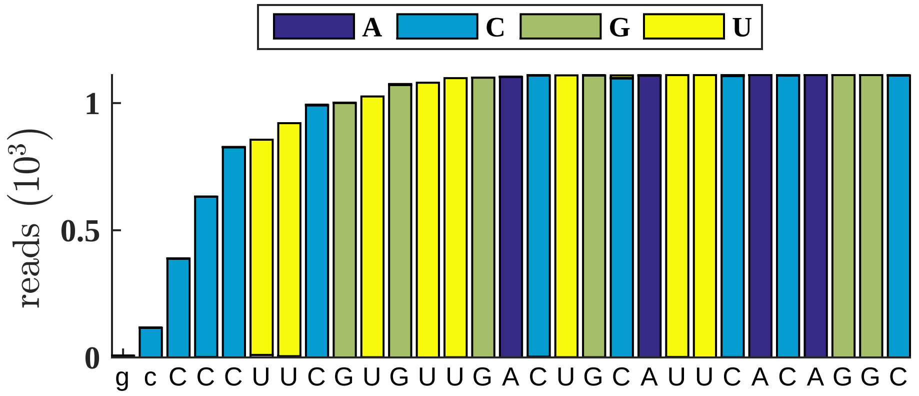

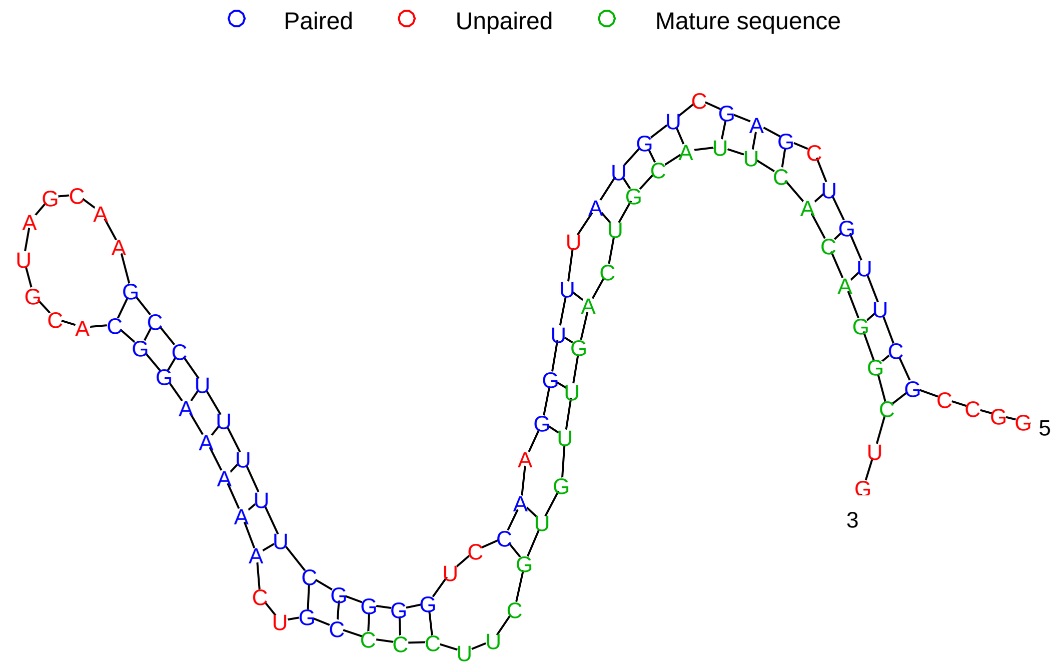

Stem loop (UMD3.1): chr11:79837057-79837148
Mature (UMD3.1): chr11:79837059-79837085
Mature seq len: 27
Total raw counts (9 samples): 1199
Average raw counts: 134
Strand: Reverse
Orientation: 3p
Minimum free energy: -30.30

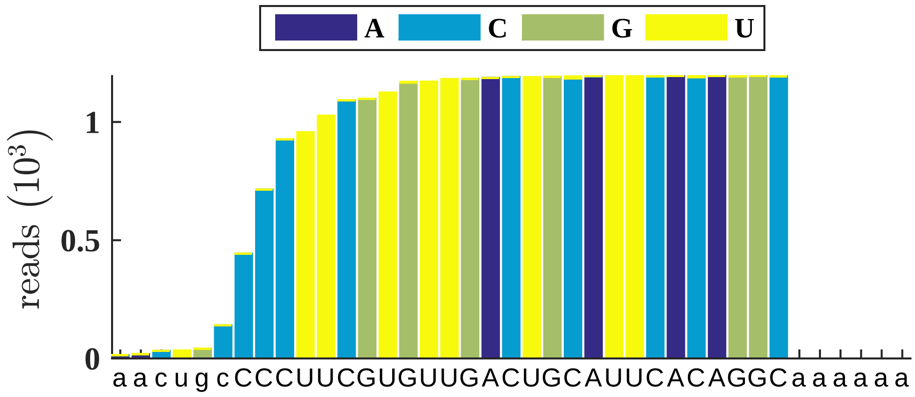

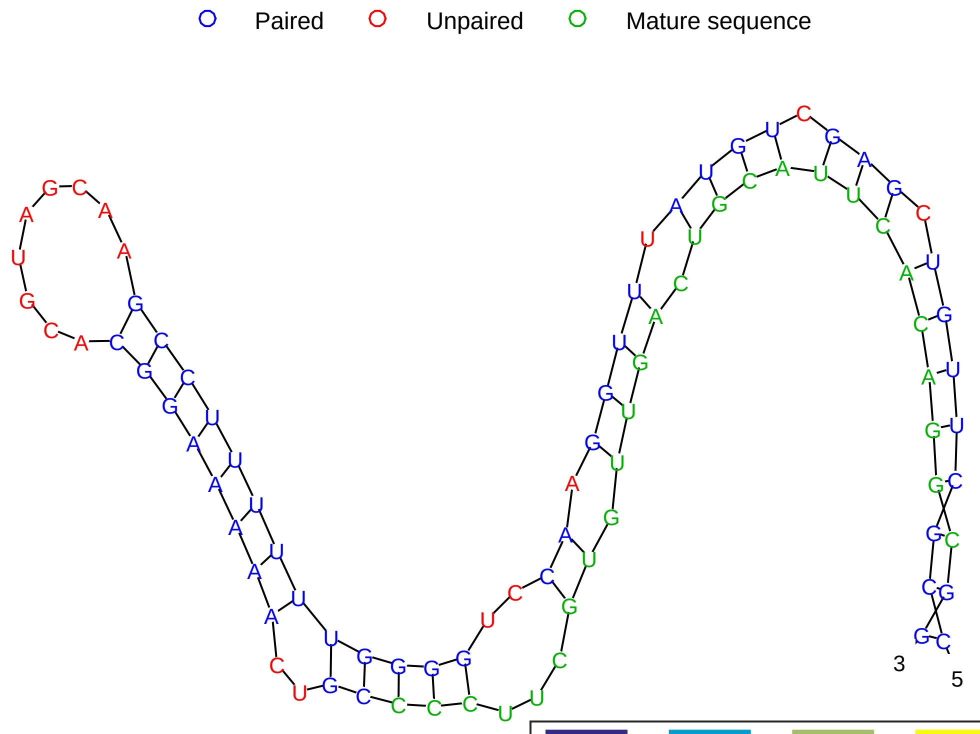

Stem loop (UMD3.1): chr11:79838165-79838254
Mature (UMD3.1): chr11:79838167-79838193
Mature seq len: 27
Total raw counts (9 samples): 1258
Average raw counts: 140
Strand: Reverse
Orientation: 3p
Minimum free energy: -33.20

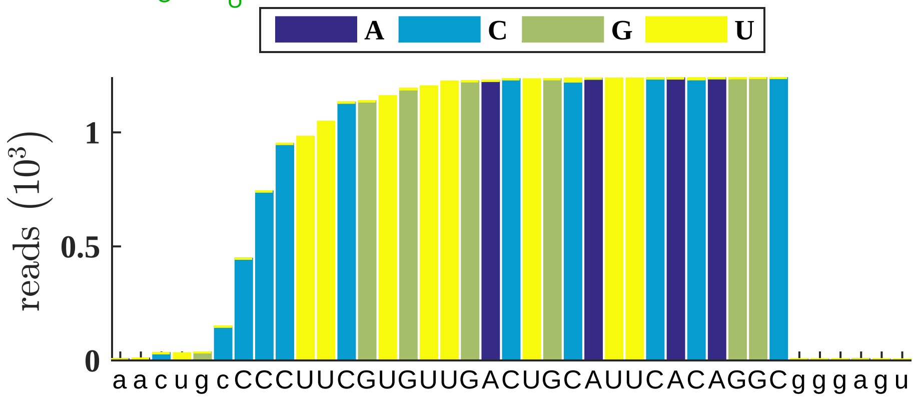

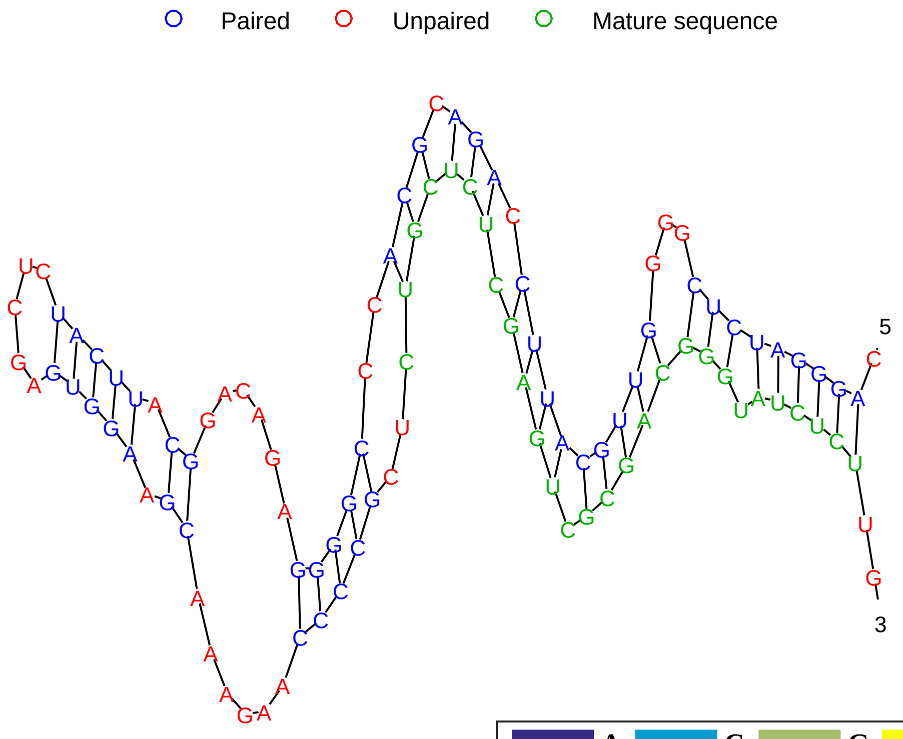

Stem loop (UMD3.1): chr11:80496114-80496220
Mature (UMD3.1): chr11:80496116-80496143
Mature seq len: 28
Total raw counts (9 samples): 12990
Average raw counts: 1444
Strand: Reverse
Orientation: 3p
Minimum free energy: -36.50

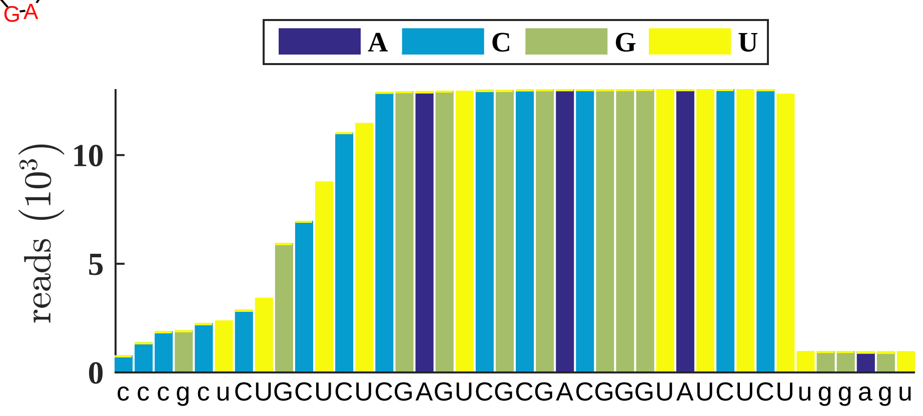

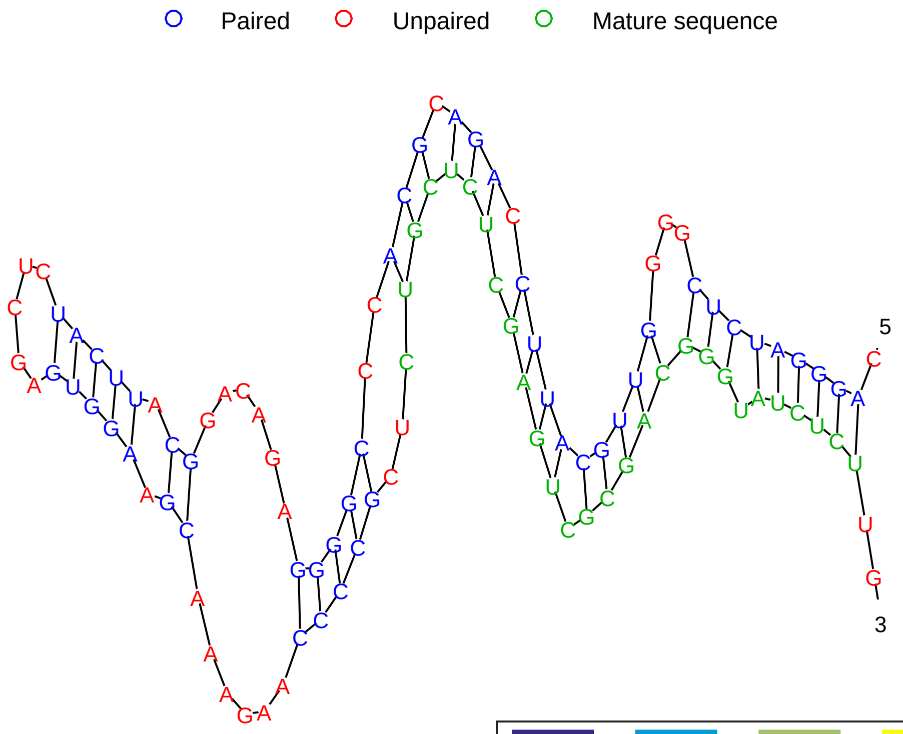

Stem loop (UMD3.1): chr11:80497504-80497610
Mature (UMD3.1): chr11:80497506-80497533
Mature seq len: 28
Total raw counts (9 samples): 12877
Average raw counts: 1431
Strand: Reverse
Orientation: 3p
Minimum free energy: -36.50

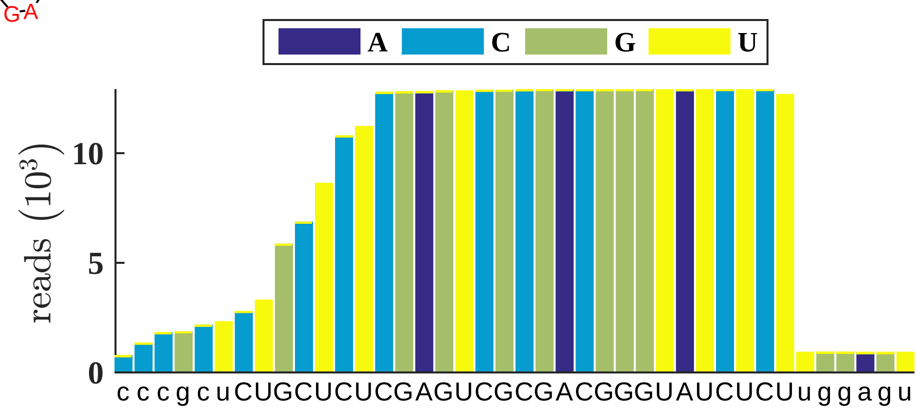

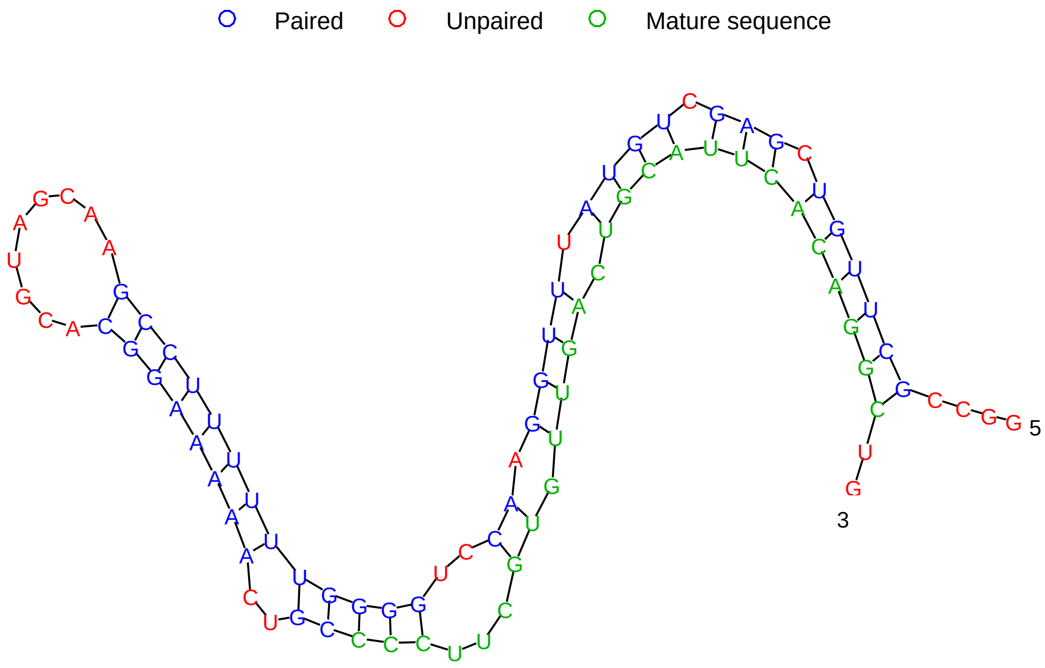

Stem loop (UMD3.1): chr11:80511821-80511912
Mature (UMD3.1): chr11:80511884-80511910
Mature seq len: 27
Total raw counts (9 samples): 1228
Average raw counts: 137
Strand: Forward
Orientation: 3p
Minimum free energy: -28.90

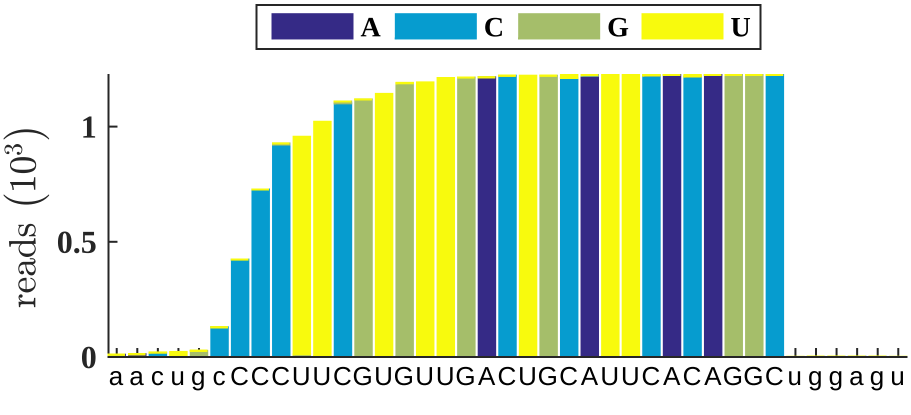

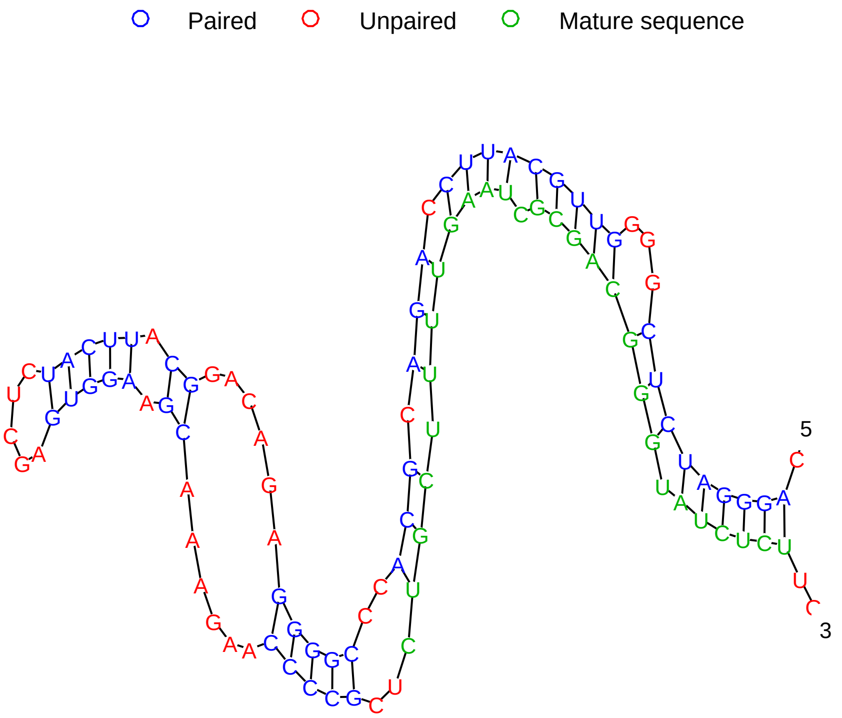

Stem loop (UMD3.1): chr11:80513093-80513199
Mature (UMD3.1): chr11:80513170-80513197
Mature seq len: 28
Total raw counts (9 samples): 2182
Average raw counts: 243
Strand: Forward
Orientation: 3p
Minimum free energy: -33.90

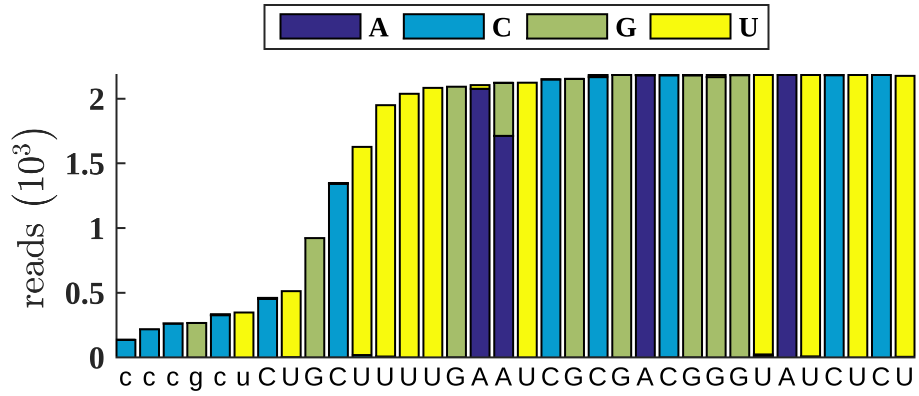

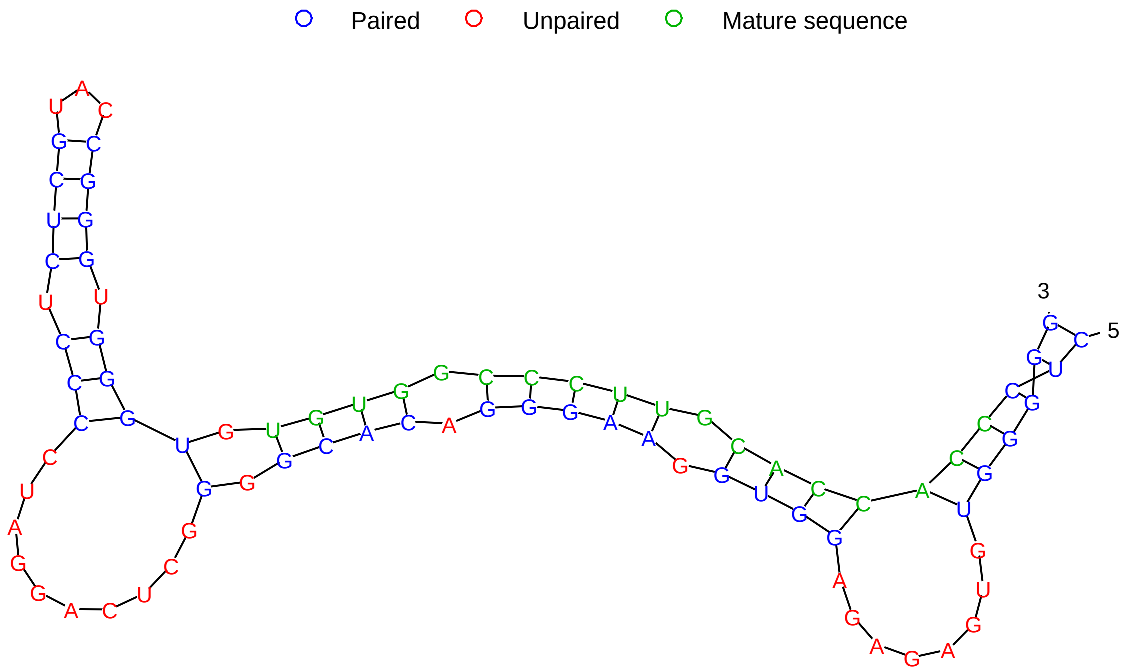

Stem loop (UMD3.1): chr11:87082179-87082261
Mature (UMD3.1): chr11:87082241-87082258
Mature seq len: 18
Total raw counts (9 samples): 1328
Average raw counts: 148
Strand: Reverse
Orientation: 5p
Minimum free energy: -35.90

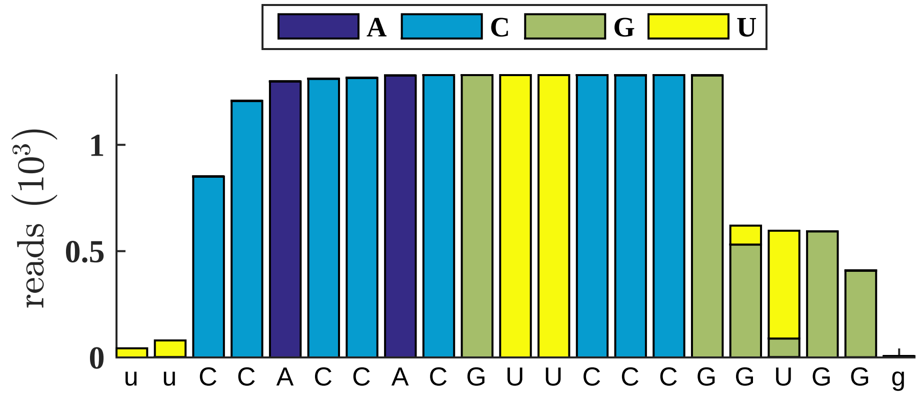

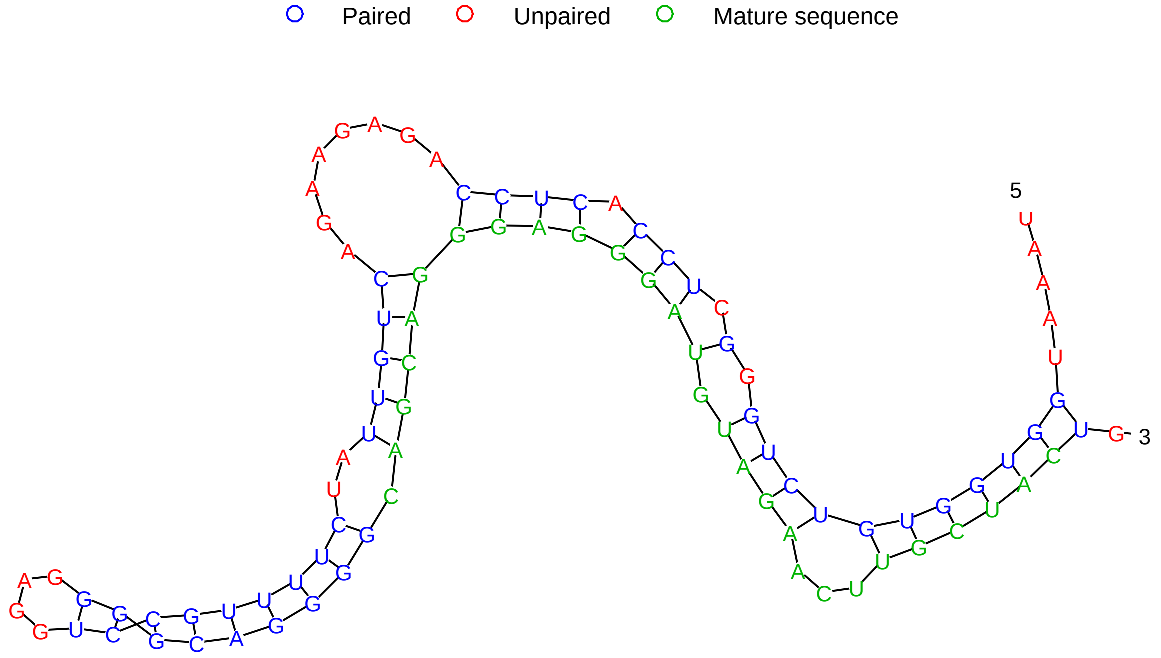

Stem loop (UMD3.1): chr11:87705806-87705899
Mature (UMD3.1): chr11:87705808-87705835
Mature seq len: 28
Total raw counts (9 samples): 2663
Average raw counts: 296
Strand: Reverse
Orientation: 3p
Minimum free energy: -32.20

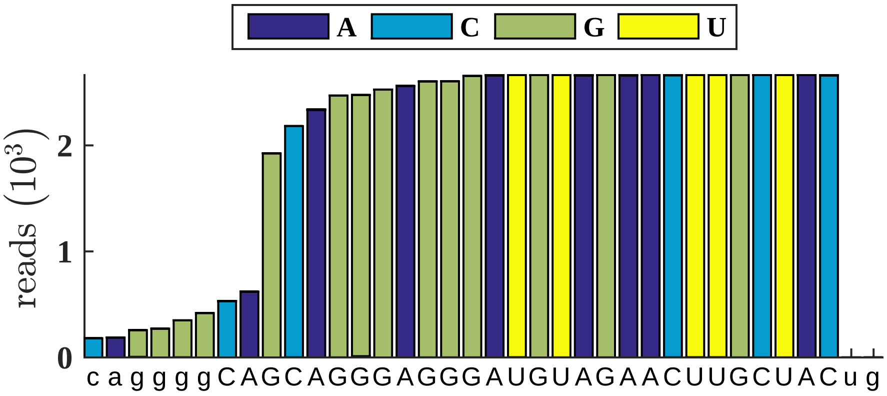

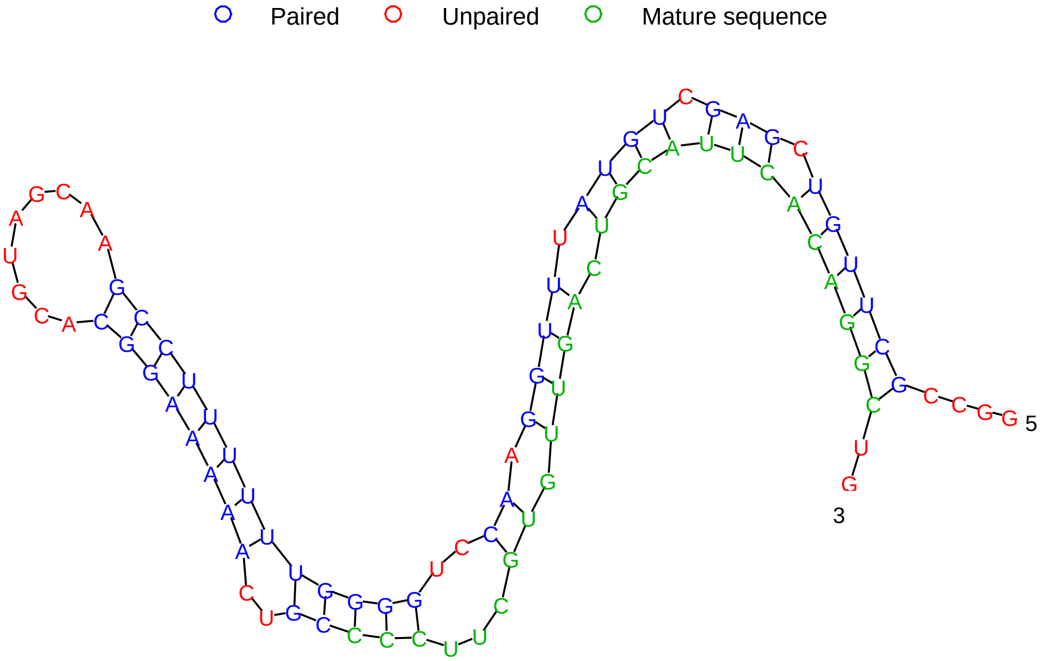

Stem loop (UMD3.1): chr11:97756059-97756150
Mature (UMD3.1): chr11:97756122-97756148
Mature seq len: 27
Total raw counts (9 samples): 1253
Average raw counts: 140
Strand: Forward
Orientation: 3p
Minimum free energy: -28.90

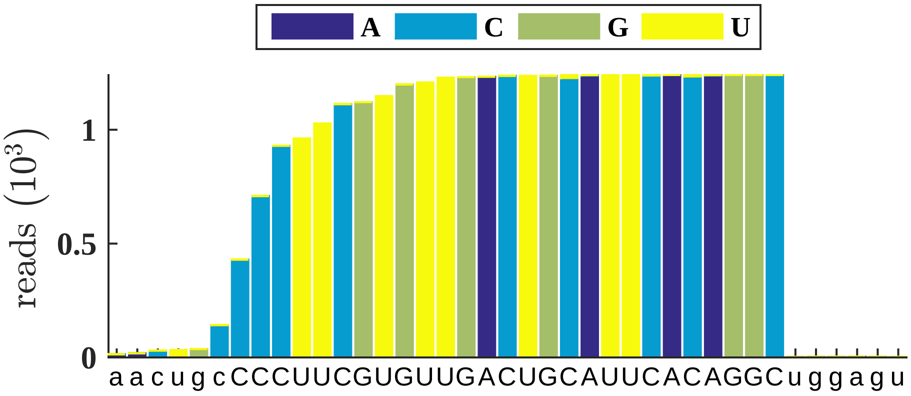

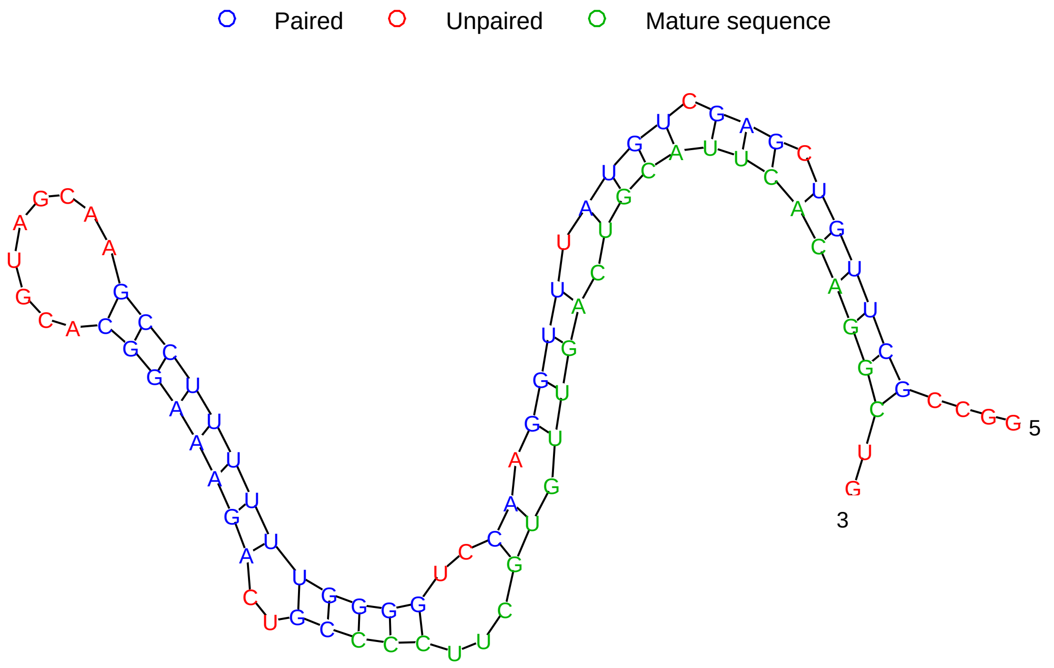

Stem loop (UMD3.1): chr11:97757470-97757561
Mature (UMD3.1): chr11:97757533-97757559
Mature seq len: 27
Total raw counts (9 samples): 1174
Average raw counts: 131
Strand: Forward
Orientation: 3p
Minimum free energy: -29.00

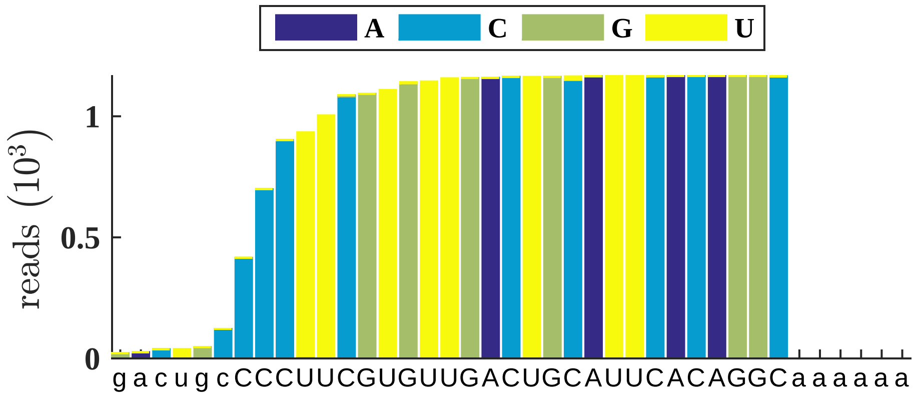

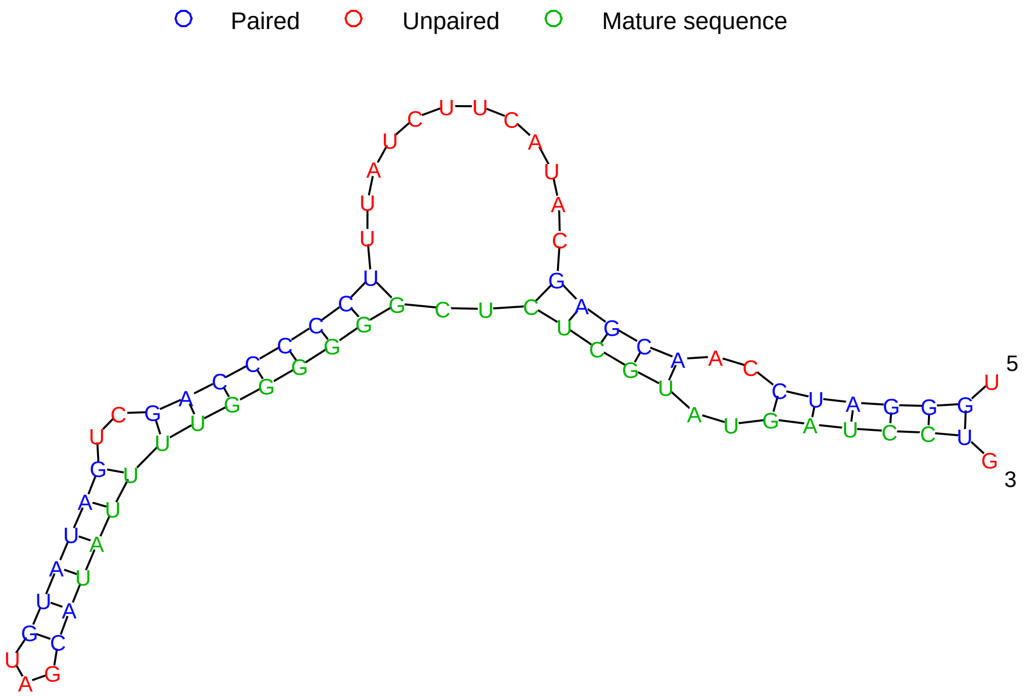

Stem loop (UMD3.1): chr12:37986381-37986455
Mature (UMD3.1): chr12:37986383-37986408
Mature seq len: 26
Total raw counts (9 samples): 691
Average raw counts: 77
Strand: Reverse
Orientation: 3p
Minimum free energy: -27.10

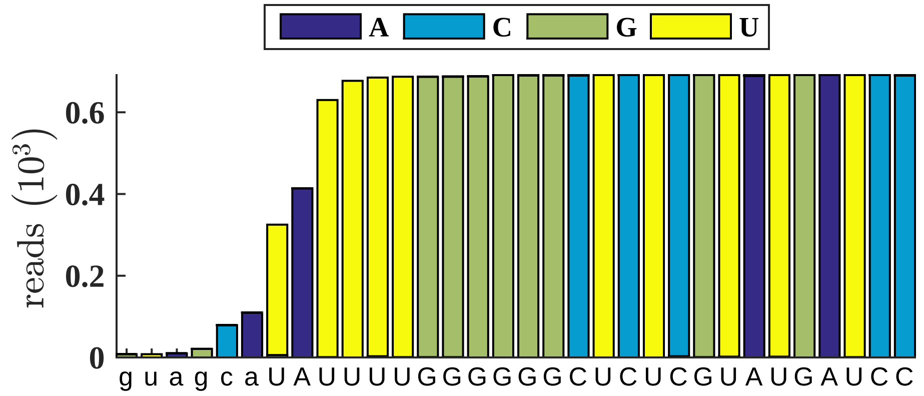

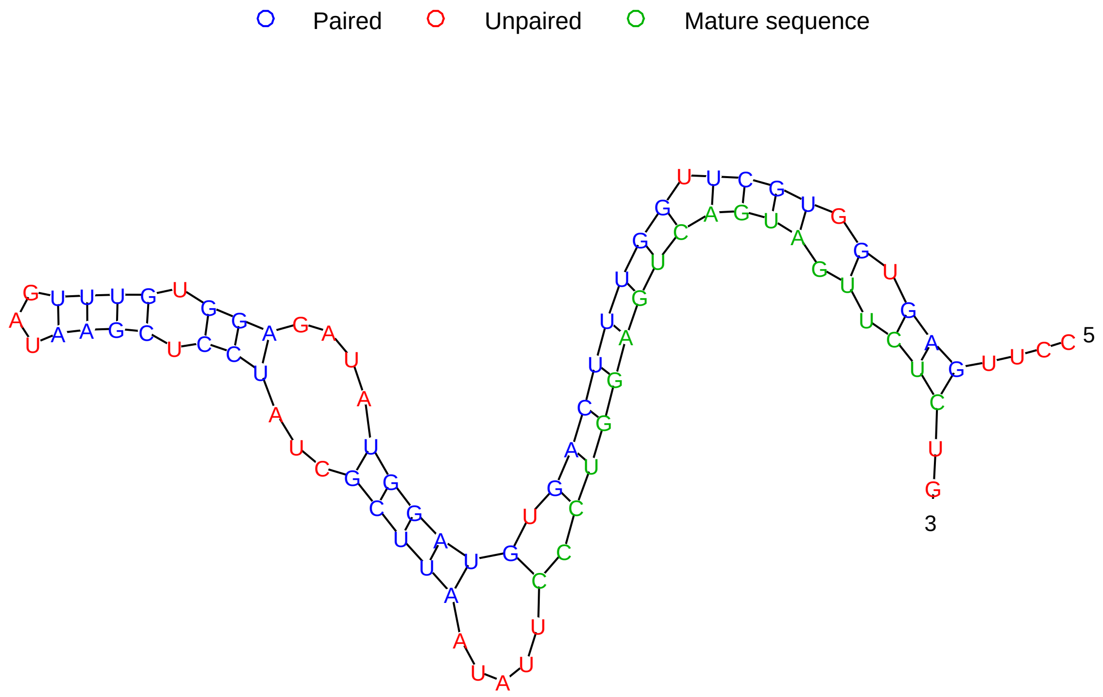

Stem loop (UMD3.1): chr12:38214938-38215025
Mature (UMD3.1): chr12:38214940-38214959
Mature seq len: 20
Total raw counts (9 samples): 1535
Average raw counts: 171
Strand: Reverse
Orientation: 3p
Minimum free energy: -19.10

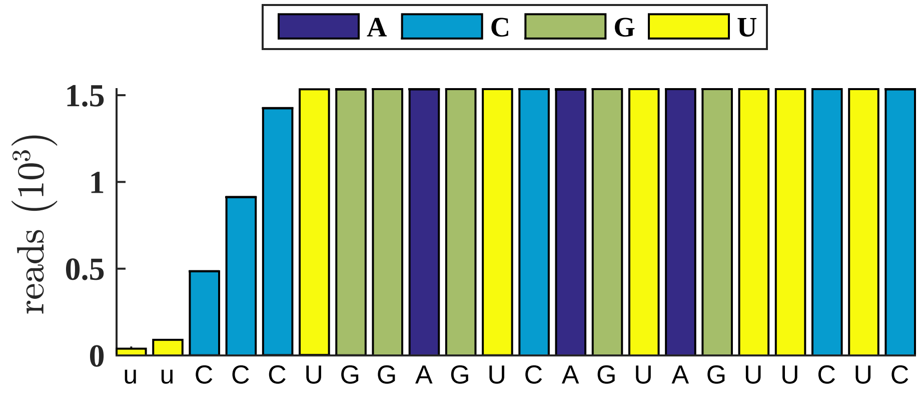

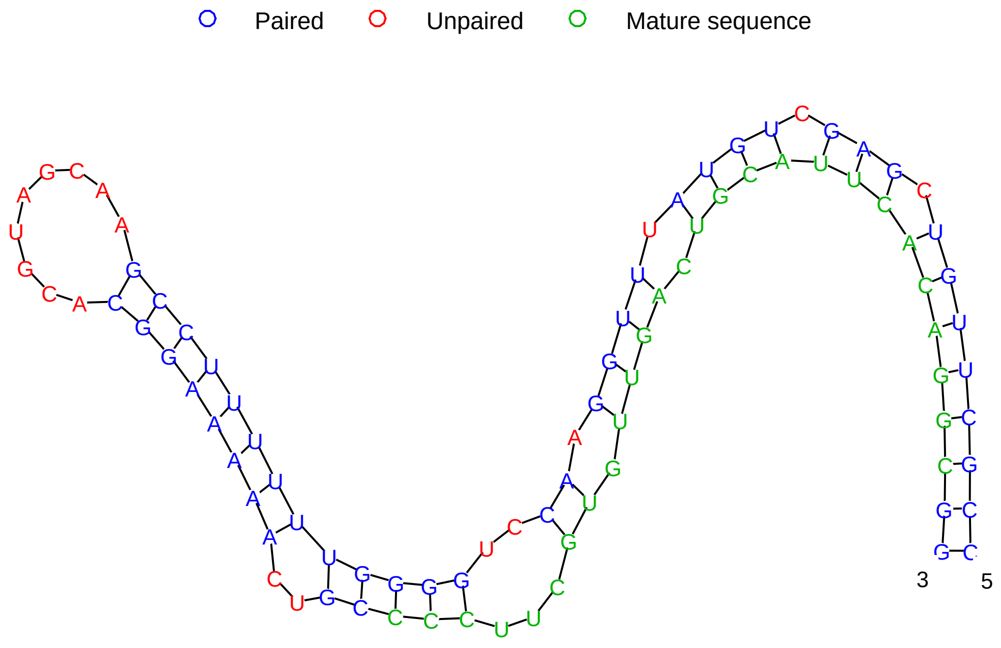

Stem loop (UMD3.1): chr12:64981353-64981442
Mature (UMD3.1): chr12:64981414-64981440
Mature seq len: 27
Total raw counts (9 samples): 1215
Average raw counts: 135
Strand: Forward
Orientation: 3p
Minimum free energy: -33.20

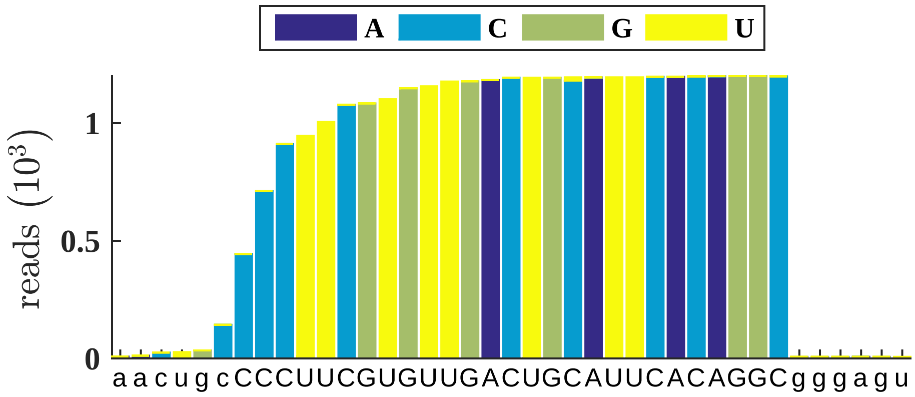

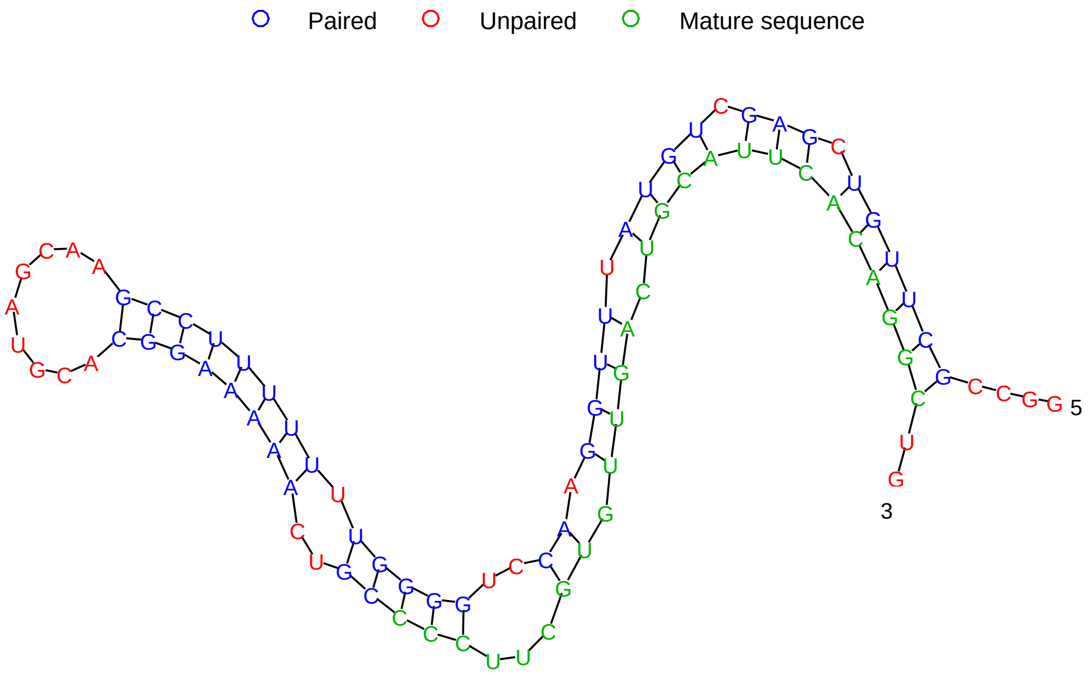

Stem loop (UMD3.1): chr12:70241145-70241237
Mature (UMD3.1): chr12:70241209-70241235
Mature seq len: 27
Total raw counts (9 samples): 1198
Average raw counts: 134
Strand: Forward
Orientation: 3p
Minimum free energy: -29.70

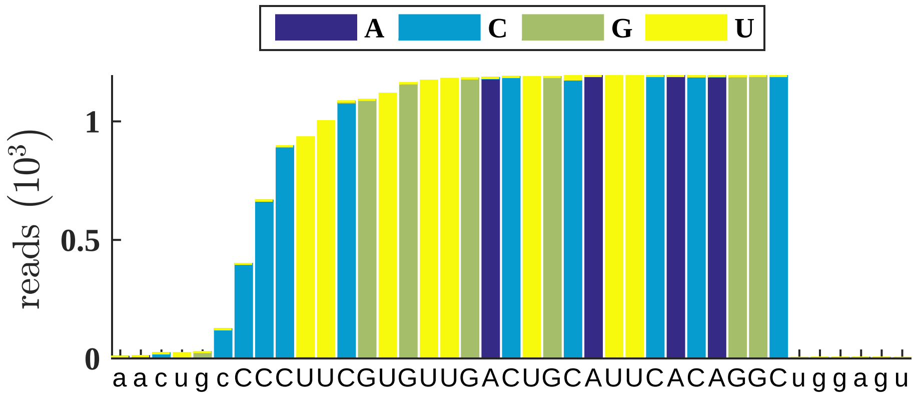

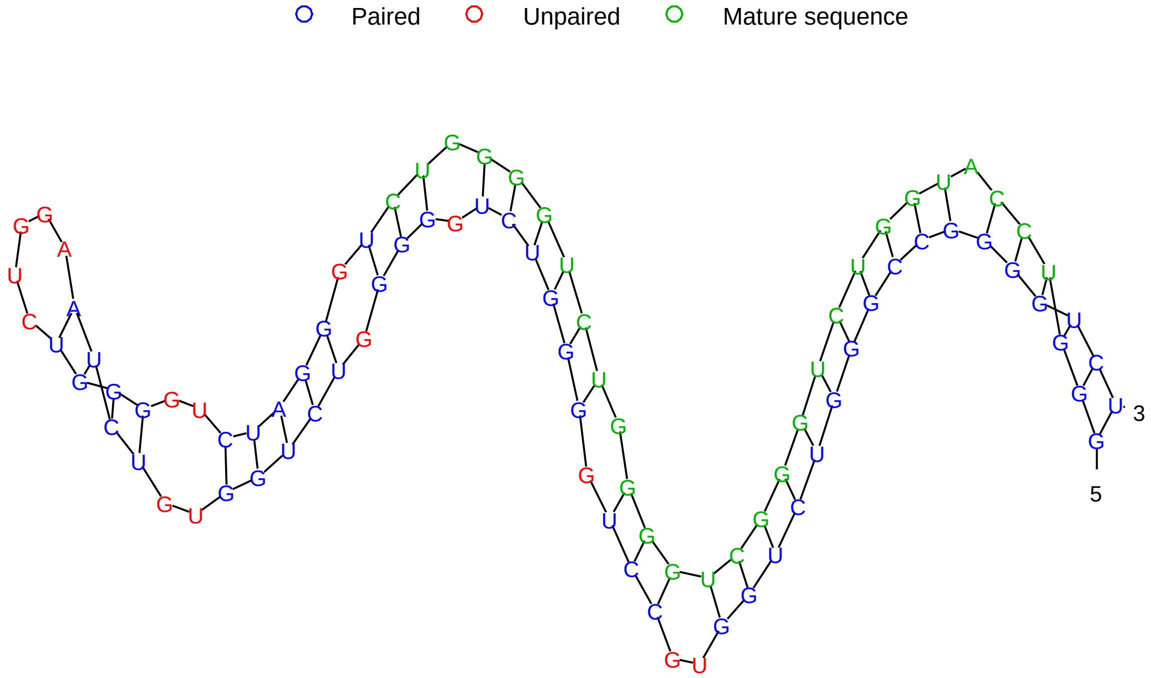

Stem loop (UMD3.1): chr12:89782134-89782227
Mature (UMD3.1): chr12:89782137-89782164
Mature seq len: 28
Total raw counts (9 samples): 1027
Average raw counts: 115
Strand: Forward
Orientation: 5p
Minimum free energy: -43.40

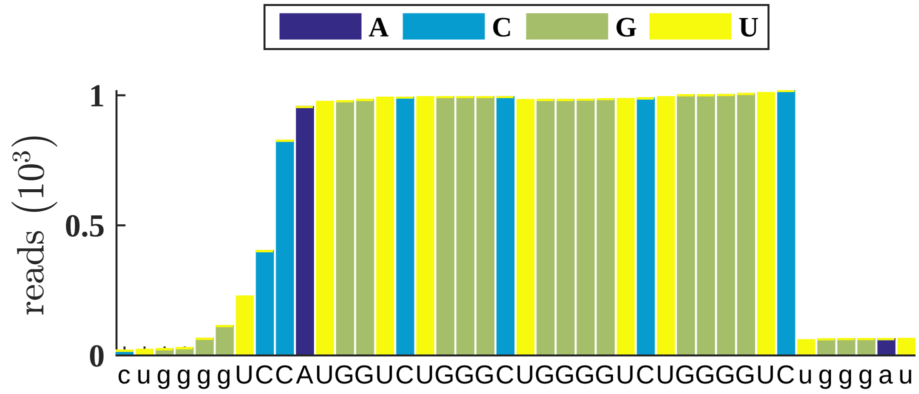

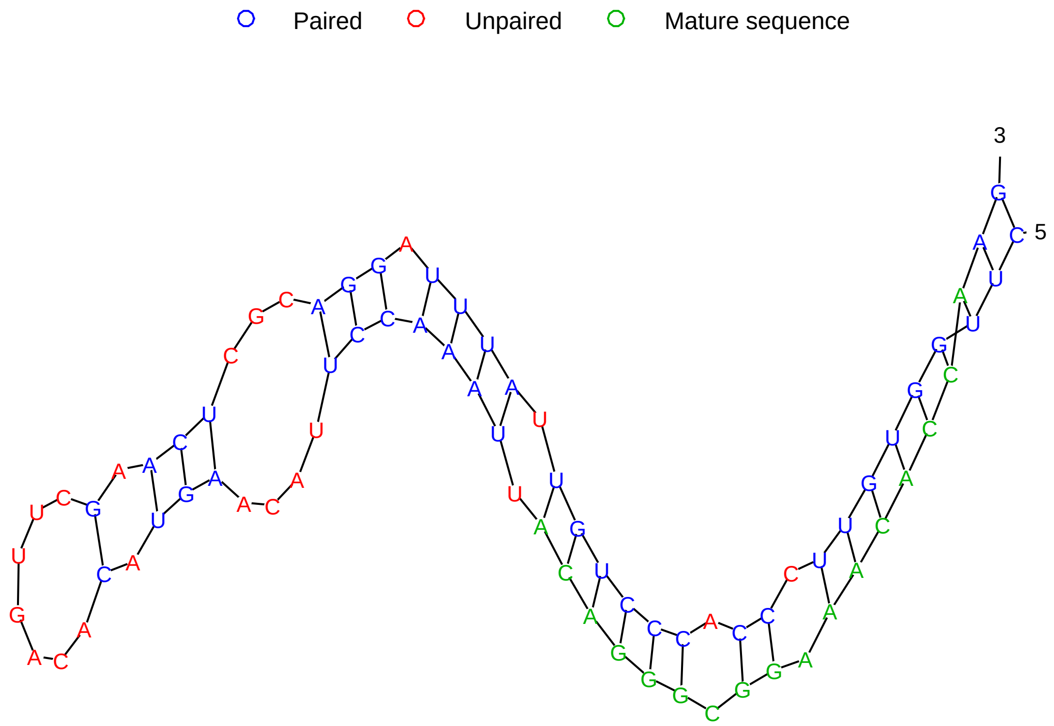

Stem loop (UMD3.1): chr13:13589074-13589152
Mature (UMD3.1): chr13:13589076-13589092
Mature seq len: 17
Total raw counts (9 samples): 3009
Average raw counts: 335
Strand: Reverse
Orientation: 3p
Minimum free energy: -30.80

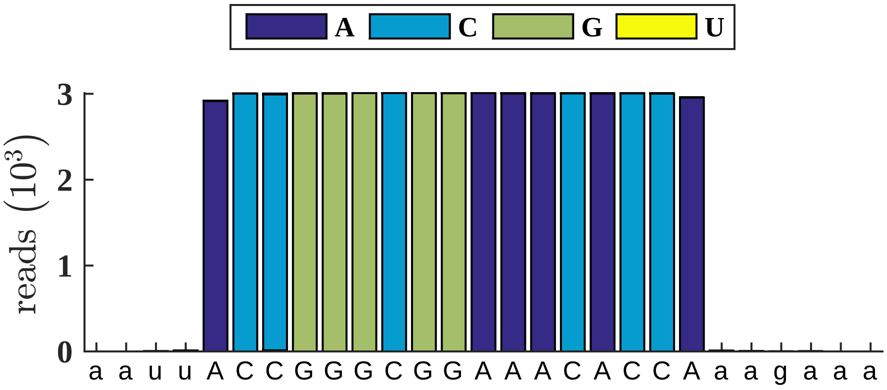

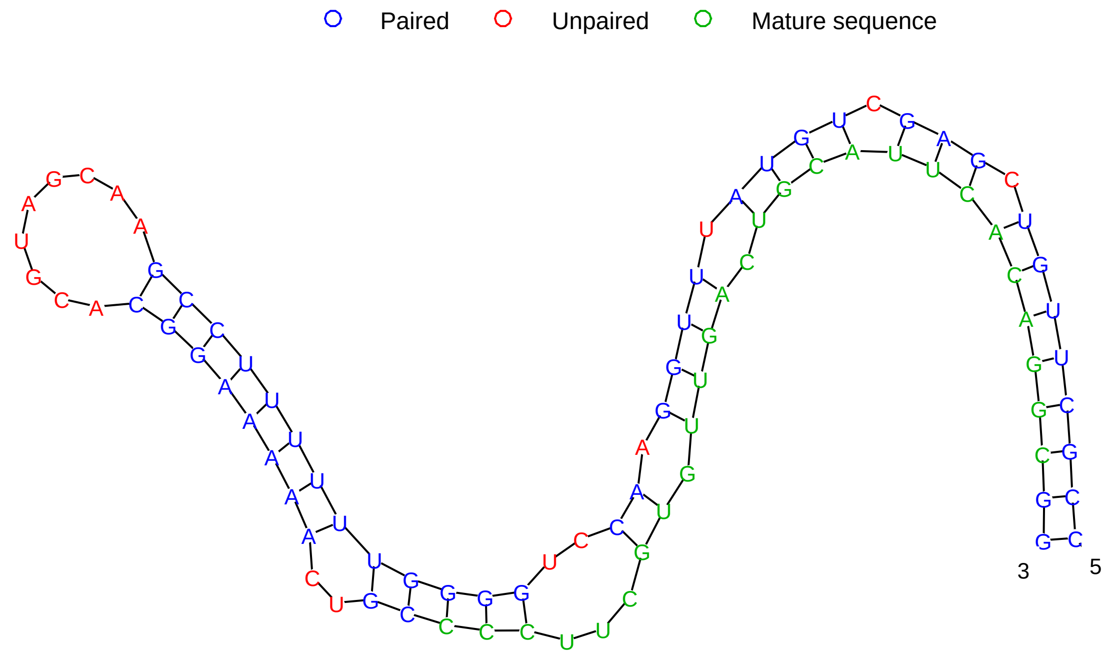

Stem loop (UMD3.1): chr13:31602808-31602897
Mature (UMD3.1): chr13:31602810-31602836
Mature seq len: 27
Total raw counts (9 samples): 1107
Average raw counts: 123
Strand: Reverse
Orientation: 3p
Minimum free energy: -33.20

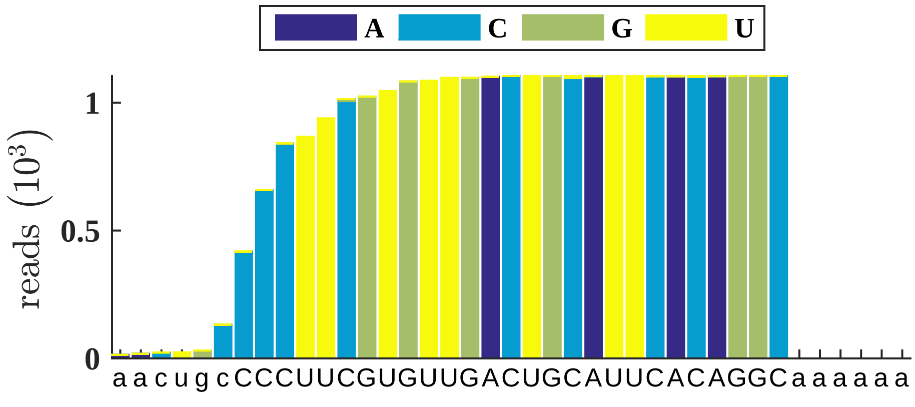

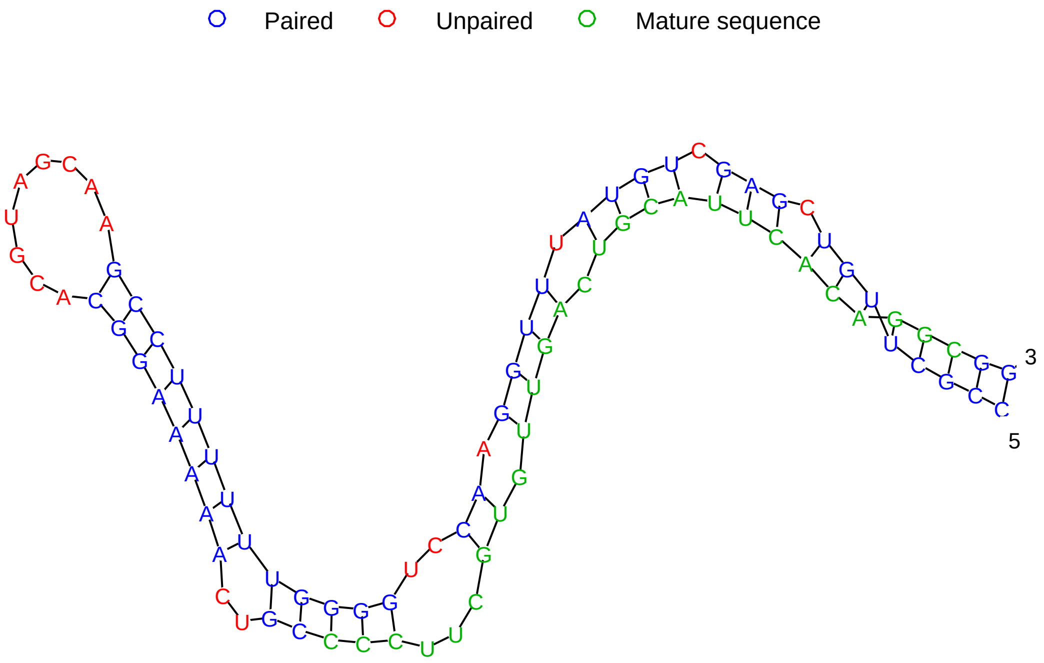

Stem loop (UMD3.1): chr13:31604914-31605003
Mature (UMD3.1): chr13:31604916-31604942
Mature seq len: 27
Total raw counts (9 samples): 1183
Average raw counts: 132
Strand: Reverse
Orientation: 3p
Minimum free energy: -33.20

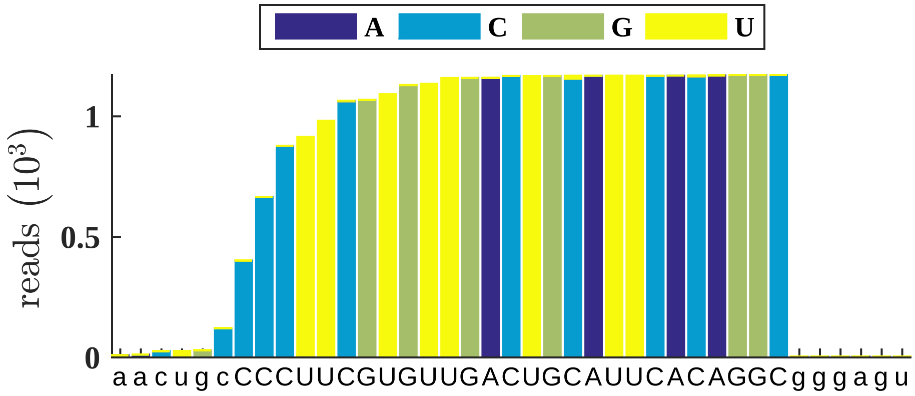

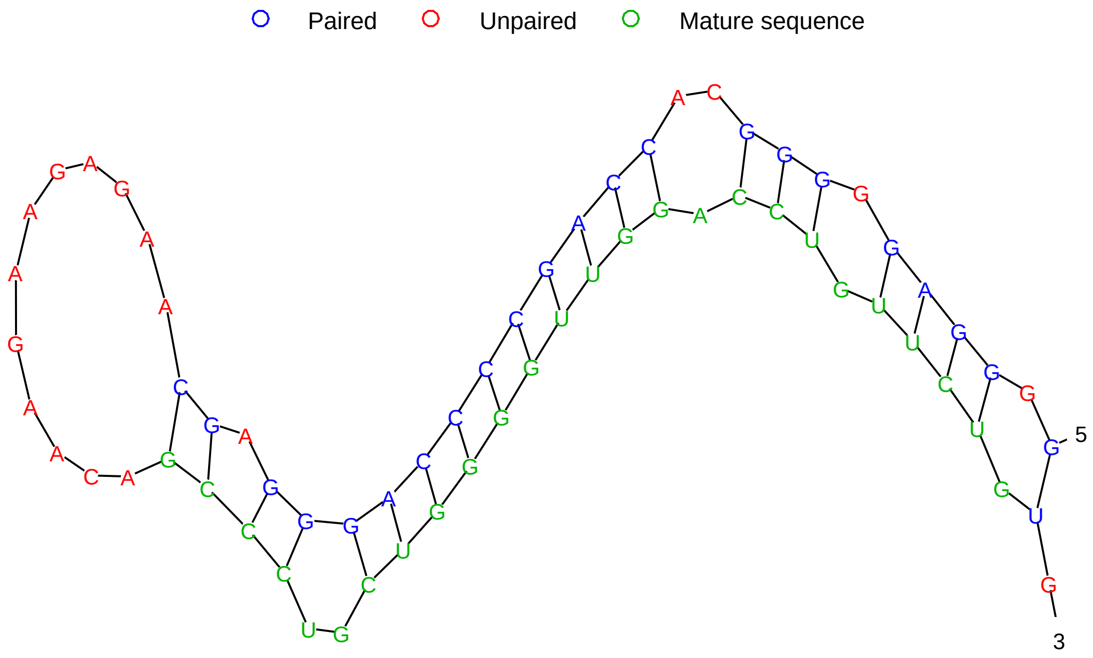

Stem loop (UMD3.1): chr13:40034375-40034441
Mature (UMD3.1): chr13:40034414-40034439
Mature seq len: 26
Total raw counts (9 samples): 811
Average raw counts: 91
Strand: Forward
Orientation: 3p
Minimum free energy: -30.60

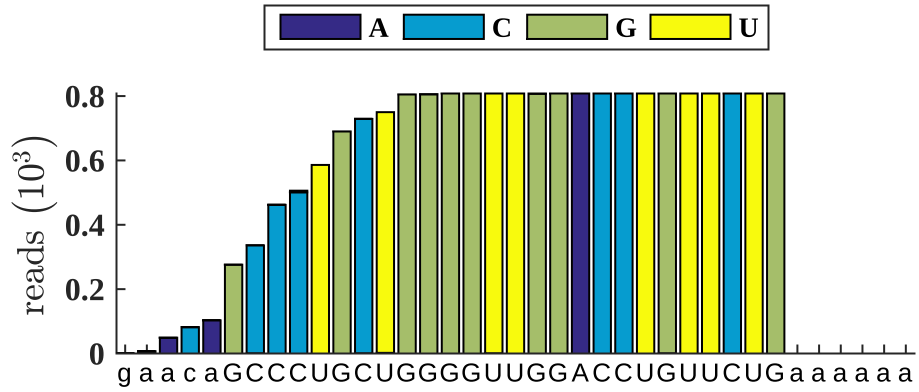

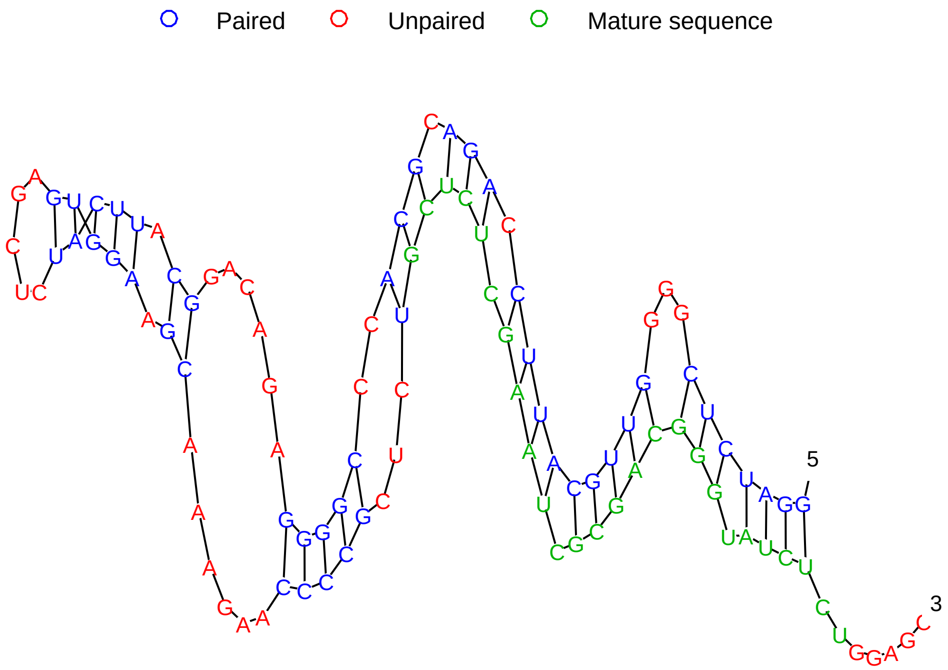

Stem loop (UMD3.1): chr13:7751484-7751590
Mature (UMD3.1): chr13:7751560-7751585
Mature seq len: 26
Total raw counts (9 samples): 3702
Average raw counts: 412
Strand: Forward
Orientation: 3p
Minimum free energy: -32.40

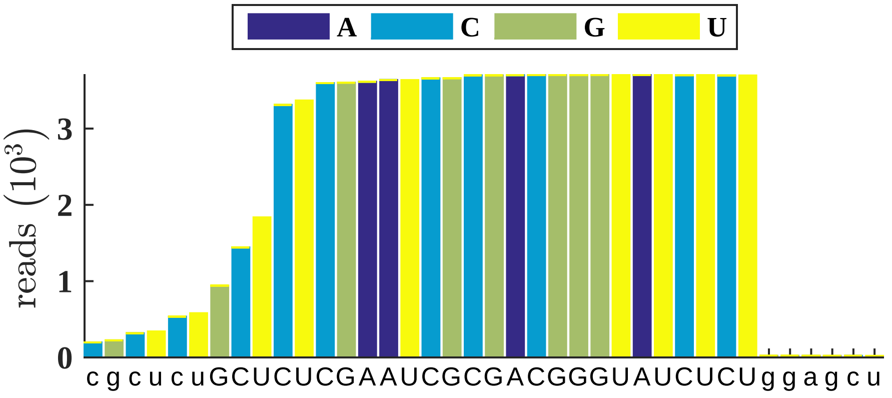

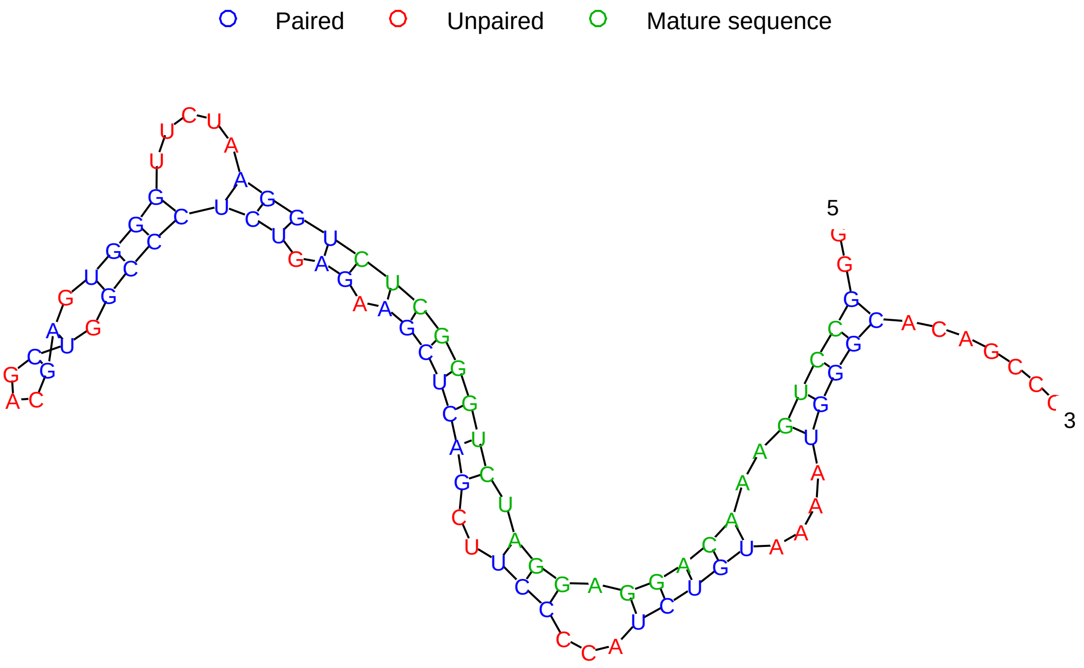

Stem loop (UMD3.1): chr13:78811577-78811672
Mature (UMD3.1): chr13:78811646-78811669
Mature seq len: 24
Total raw counts (9 samples): 2240
Average raw counts: 249
Strand: Reverse
Orientation: 5p
Minimum free energy: -31.40

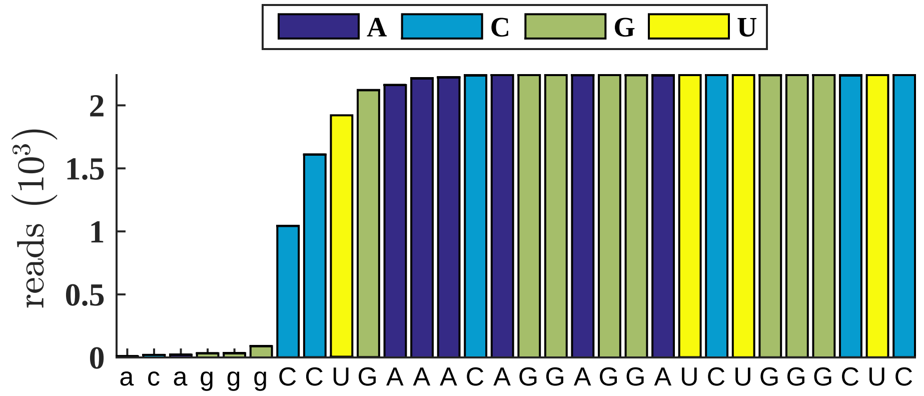

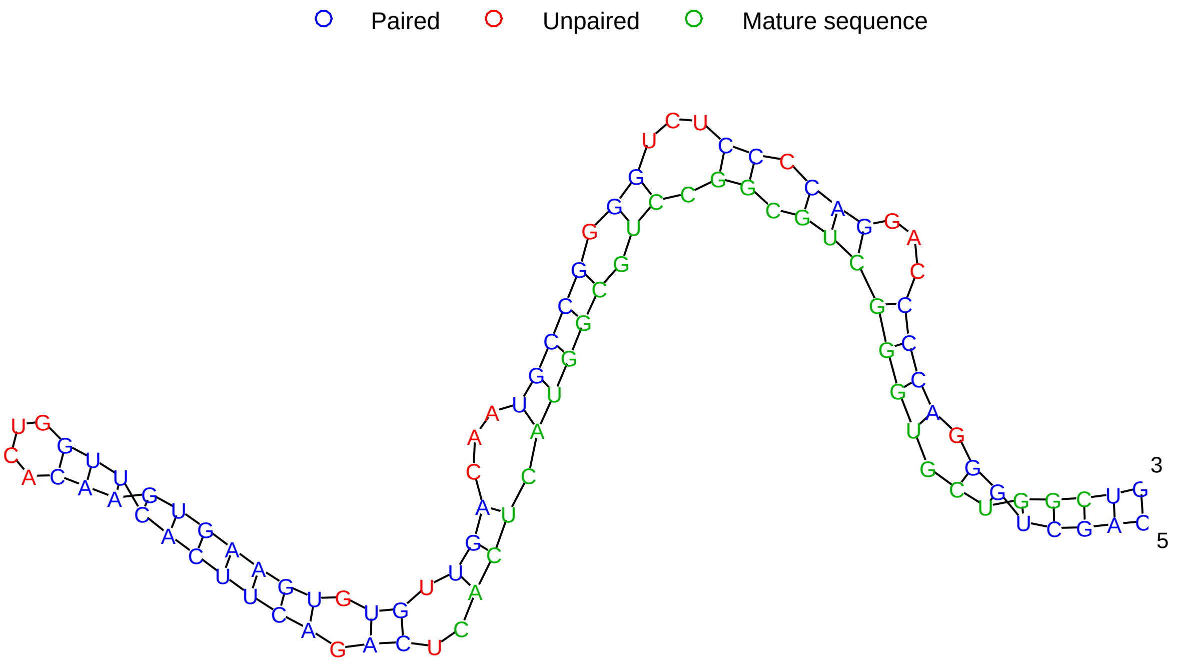

Stem loop (UMD3.1): chr14:4412035-4412136
Mature (UMD3.1): chr14:4412105-4412134
Mature seq len: 30
Total raw counts (9 samples): 2396
Average raw counts: 267
Strand: Forward
Orientation: 3p
Minimum free energy: -50.40

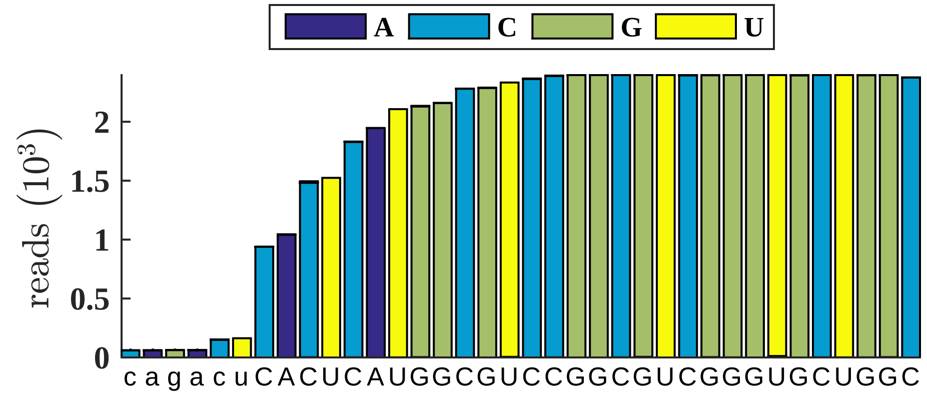

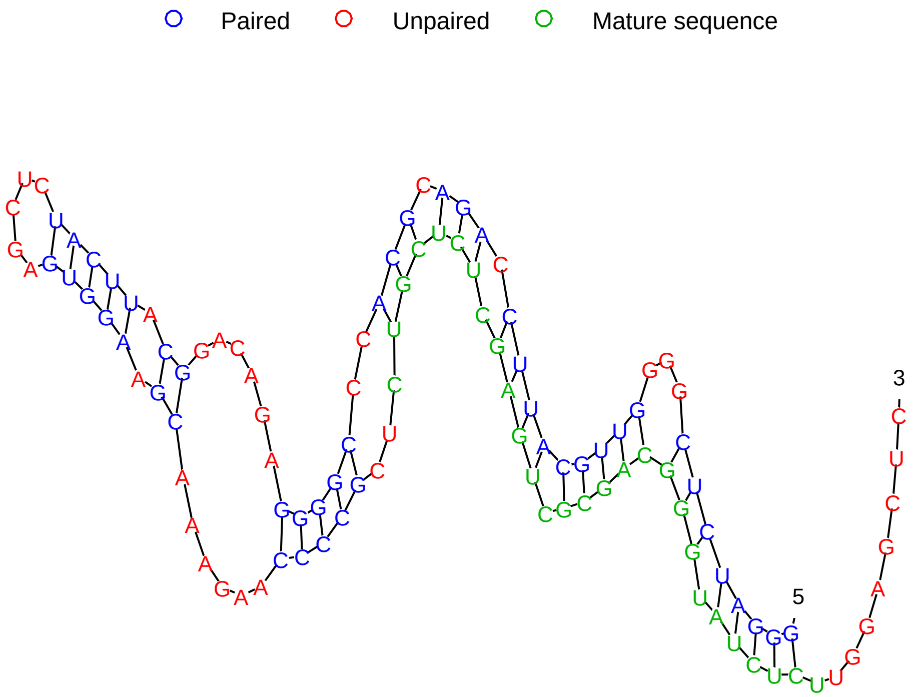

Stem loop (UMD3.1): chr14:4922521-4922631
Mature (UMD3.1): chr14:4922529-4922556
Mature seq len: 28
Total raw counts (9 samples): 13307
Average raw counts: 1479
Strand: Reverse
Orientation: 3p
Minimum free energy: -35.50

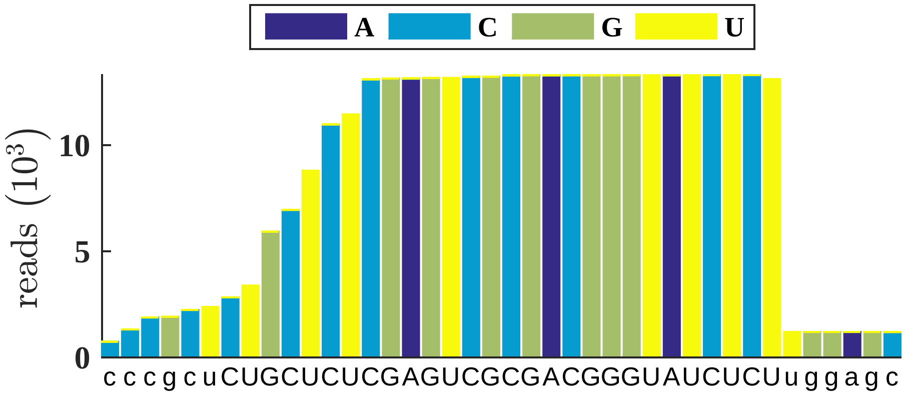

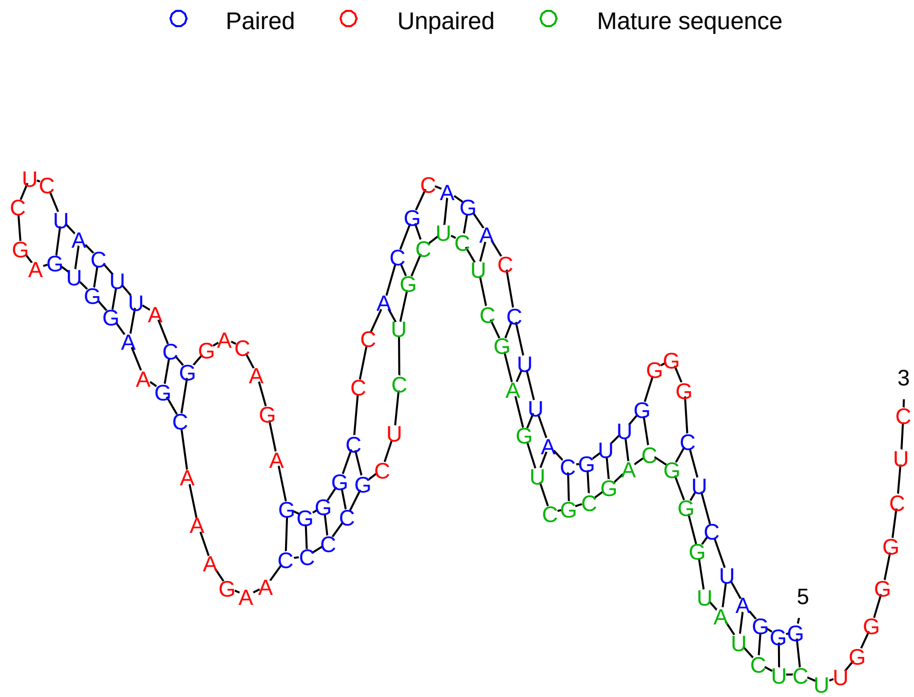

Stem loop (UMD3.1): chr15:26578123-26578233
Mature (UMD3.1): chr15:26578131-26578158
Mature seq len: 28
Total raw counts (9 samples): 12650
Average raw counts: 1406
Strand: Reverse
Orientation: 3p
Minimum free energy: -35.50

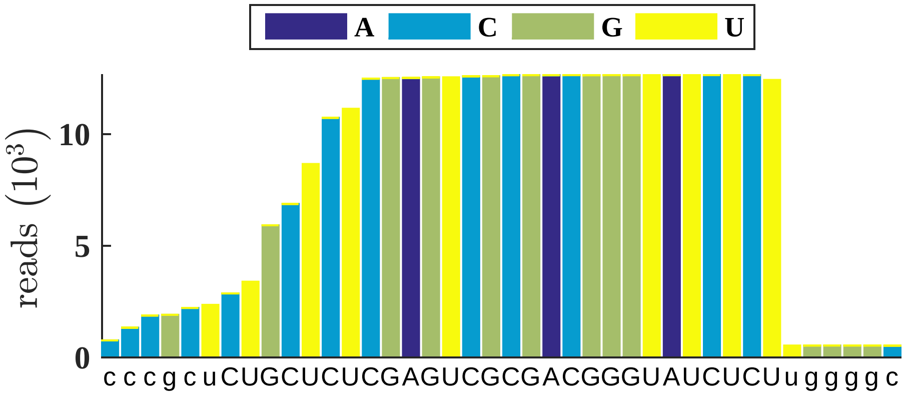

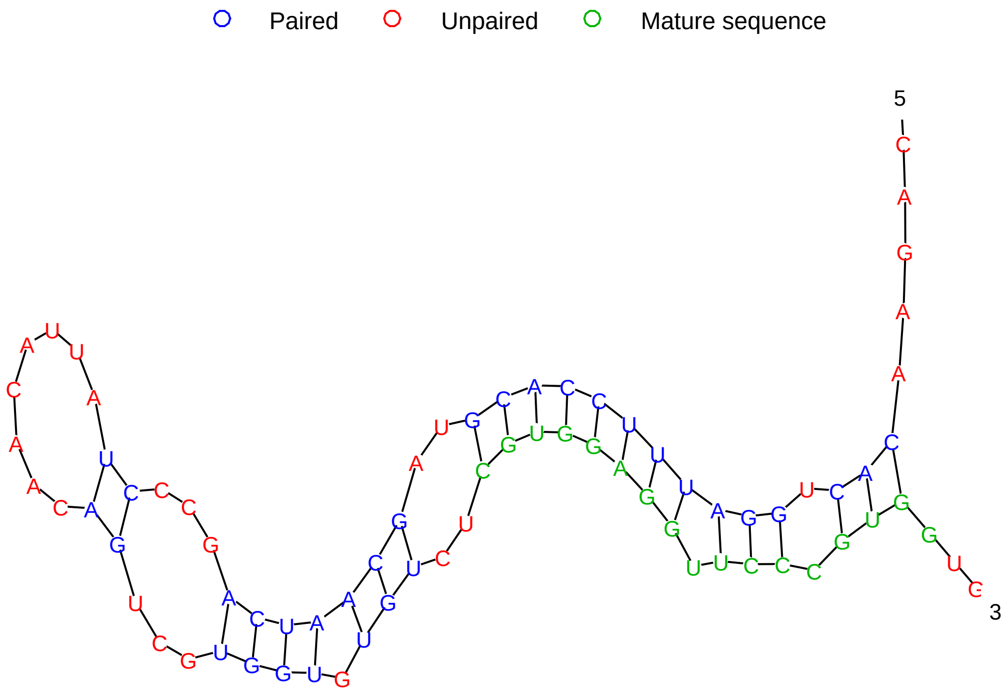

Stem loop (UMD3.1): chr15:65493496-65493571
Mature (UMD3.1): chr15:65493553-65493569
Mature seq len: 17
Total raw counts (9 samples): 1346
Average raw counts: 150
Strand: Forward
Orientation: 3p
Minimum free energy: -23.00

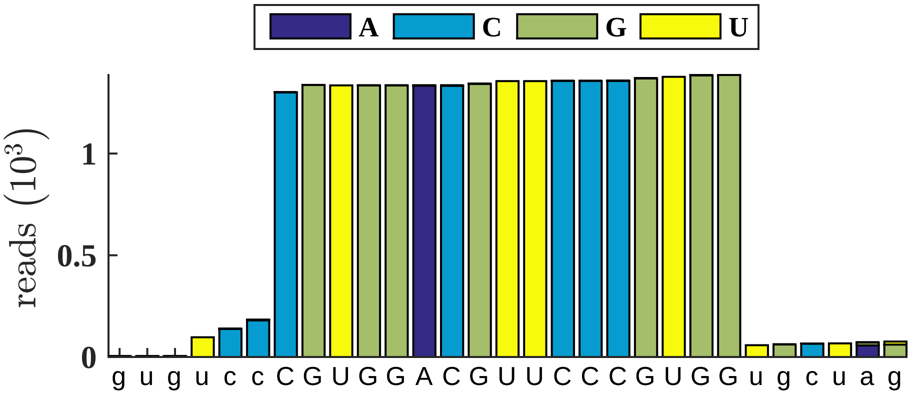

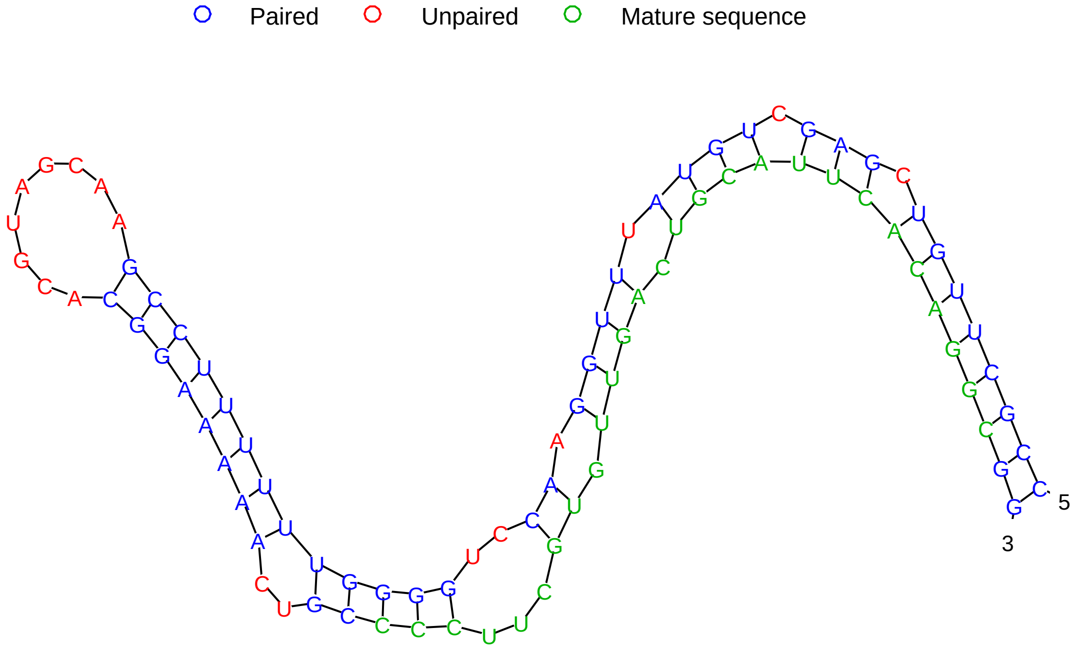

Stem loop (UMD3.1): chr15:65554381-65554470
Mature (UMD3.1): chr15:65554383-65554409
Mature seq len: 27
Total raw counts (9 samples): 1207
Average raw counts: 135
Strand: Reverse
Orientation: 3p
Minimum free energy: -33.20

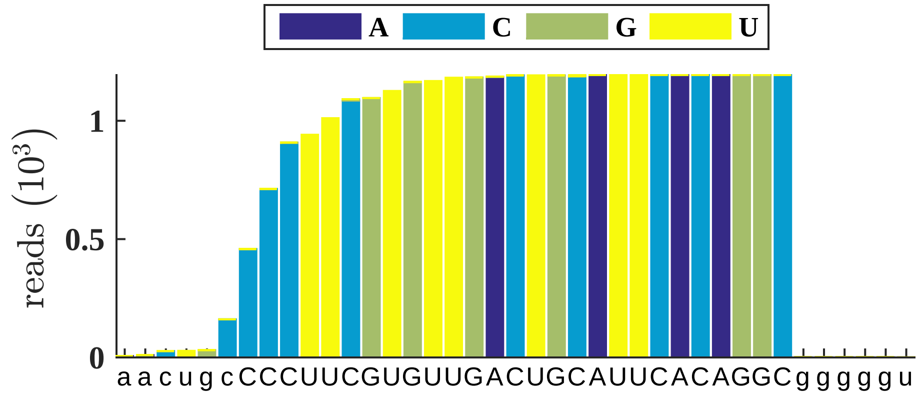

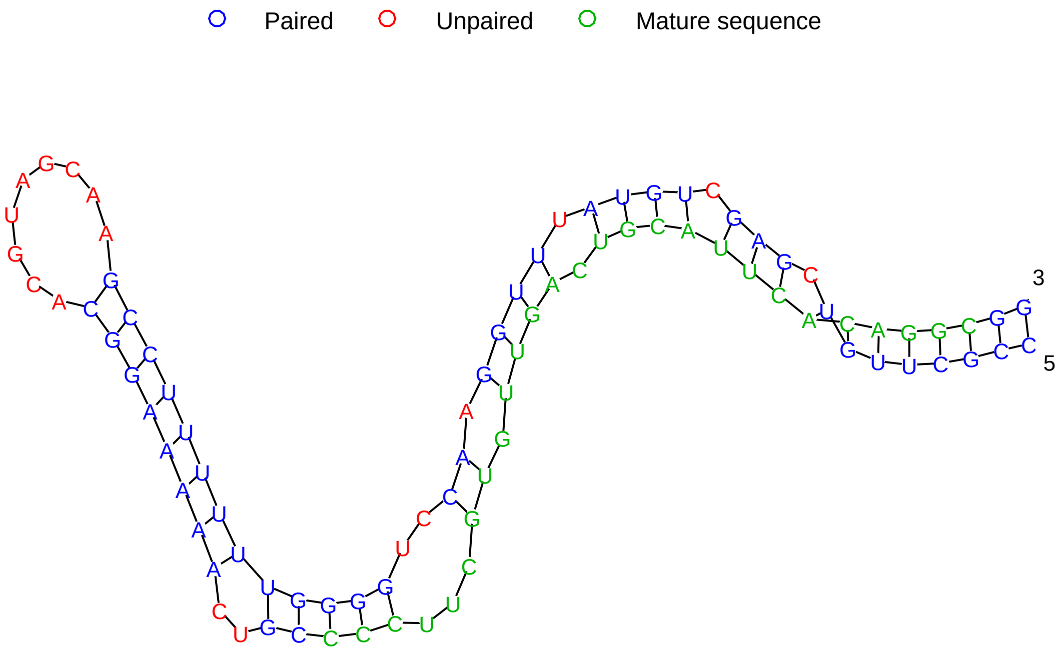

Stem loop (UMD3.1): chr15:82597026-82597115
Mature (UMD3.1): chr15:82597028-82597054
Mature seq len: 27
Total raw counts (9 samples): 1232
Average raw counts: 137
Strand: Reverse
Orientation: 3p
Minimum free energy: -33.20

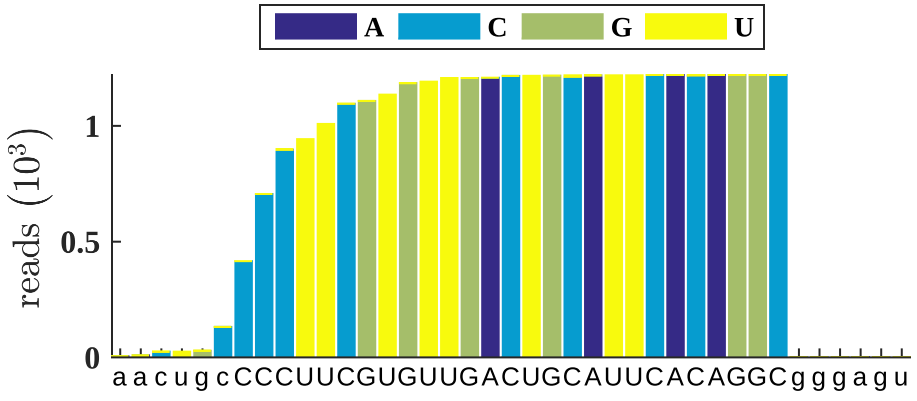

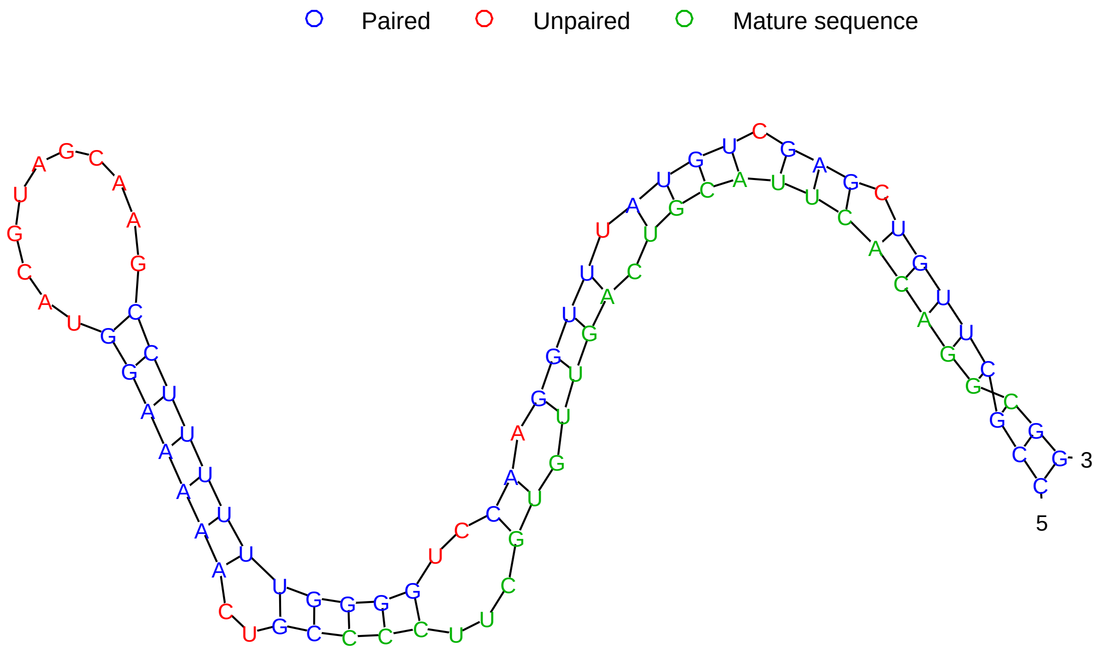

Stem loop (UMD3.1): chr15:82599676-82599765
Mature (UMD3.1): chr15:82599678-82599704
Mature seq len: 27
Total raw counts (9 samples): 1204
Average raw counts: 134
Strand: Reverse
Orientation: 3p
Minimum free energy: -31.00

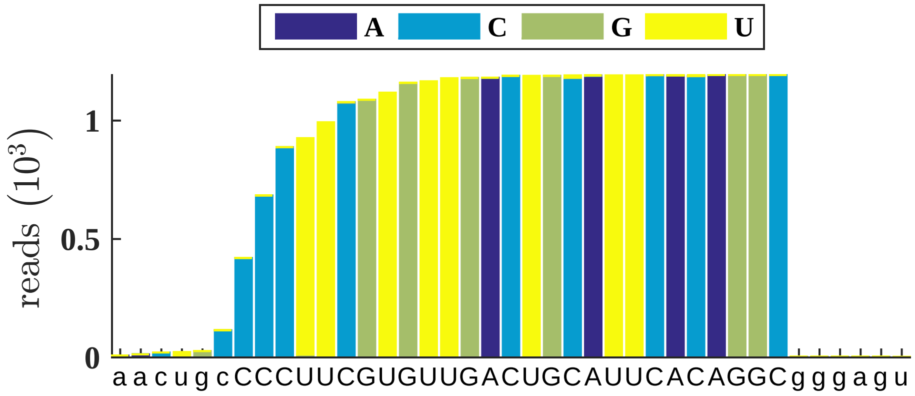

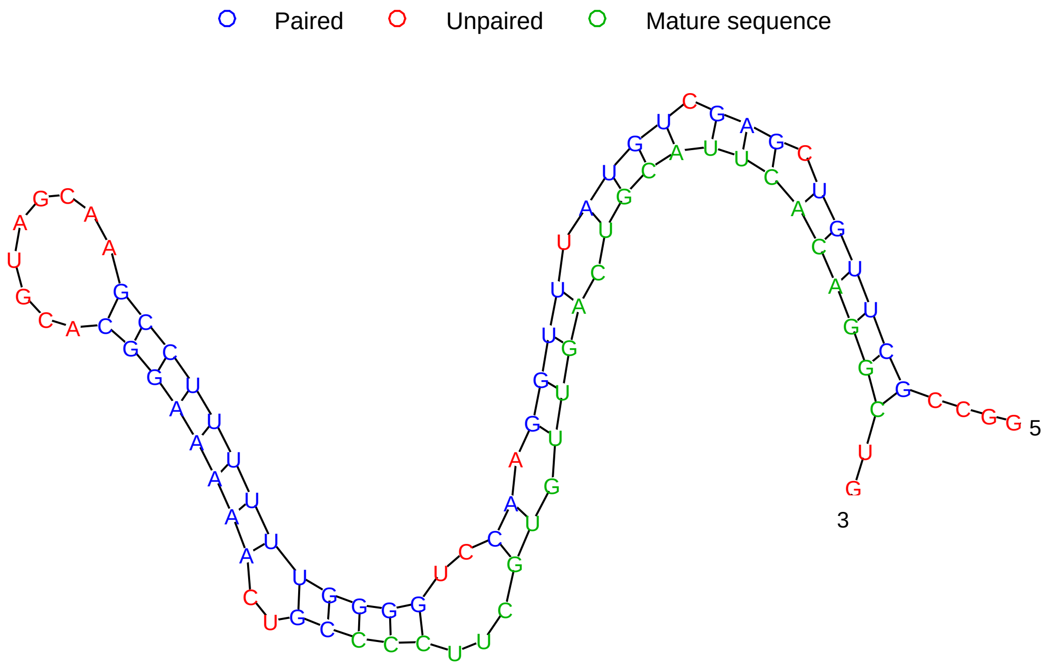

Stem loop (UMD3.1): chr15:82601629-82601720
Mature (UMD3.1): chr15:82601631-82601657
Mature seq len: 27
Total raw counts (9 samples): 1131
Average raw counts: 126
Strand: Reverse
Orientation: 3p
Minimum free energy: -28.90

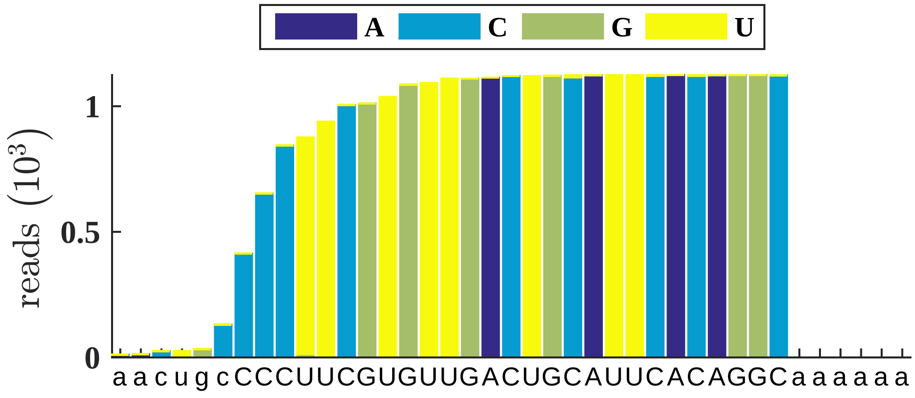

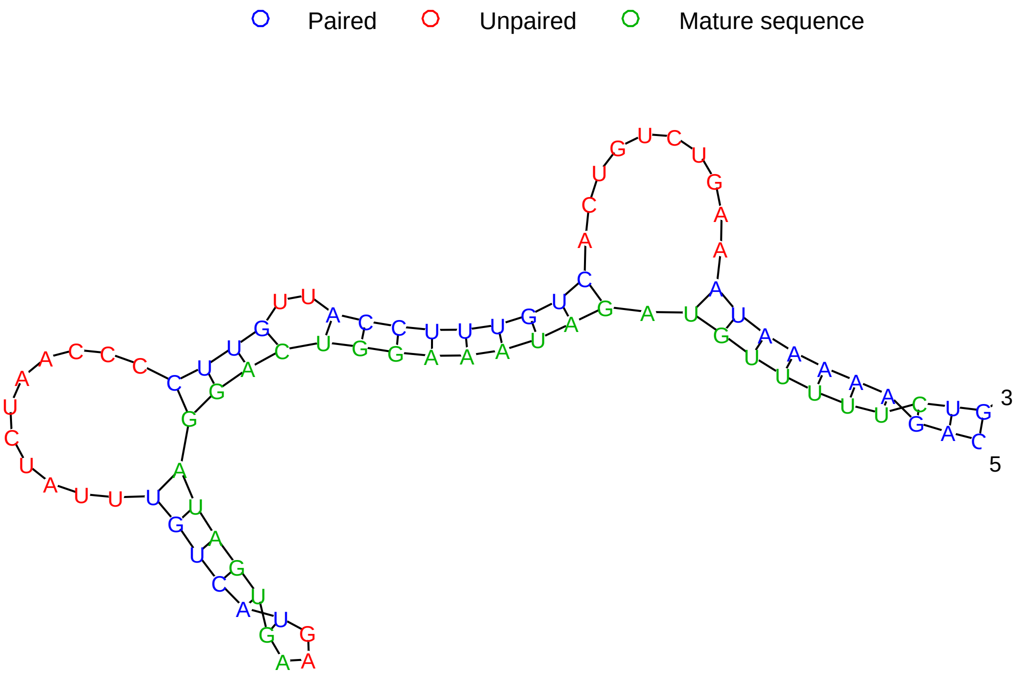

Stem loop (UMD3.1): chr16:10539611-10539695
Mature (UMD3.1): chr16:10539613-10539641
Mature seq len: 29
Total raw counts (9 samples): 1661
Average raw counts: 185
Strand: Reverse
Orientation: 3p
Minimum free energy: -19.00

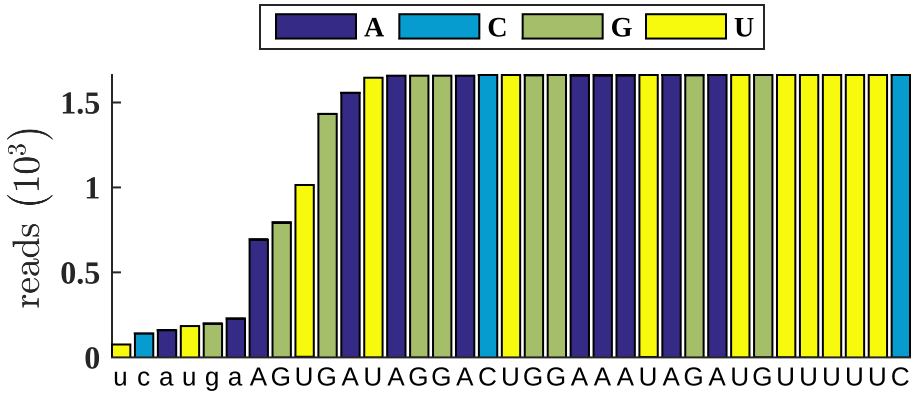

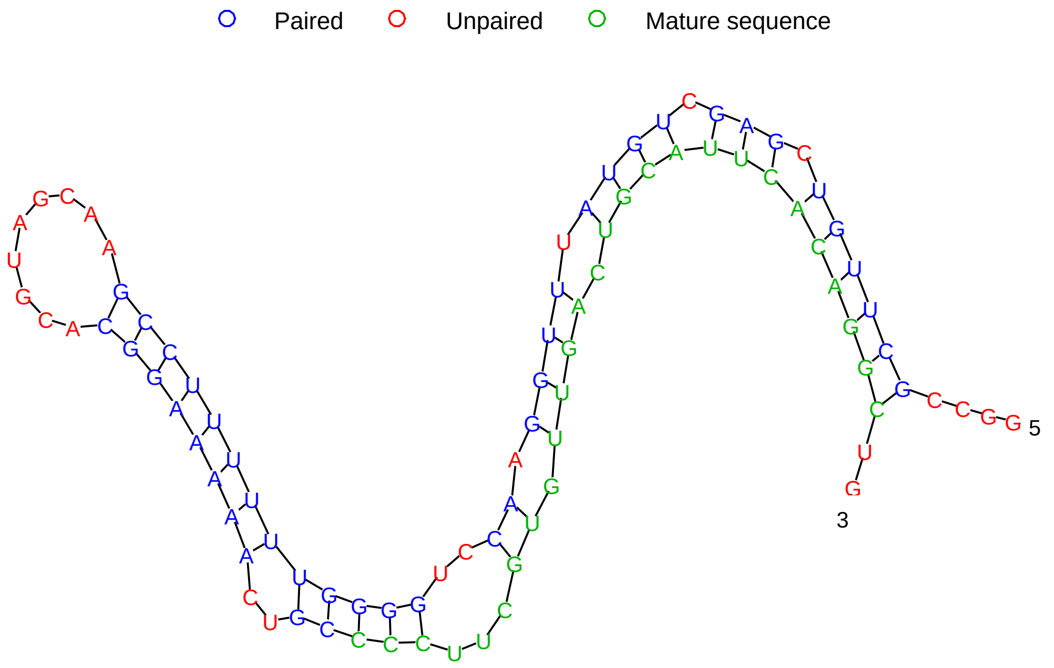

Stem loop (UMD3.1): chr16:23609625-23609716
Mature (UMD3.1): chr16:23609627-23609653
Mature seq len: 27
Total raw counts (9 samples): 1198
Average raw counts: 134
Strand: Reverse
Orientation: 3p
Minimum free energy: -28.90

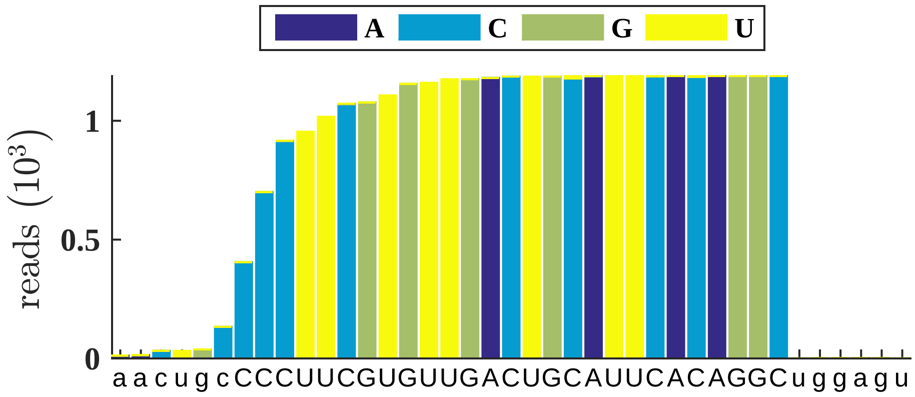

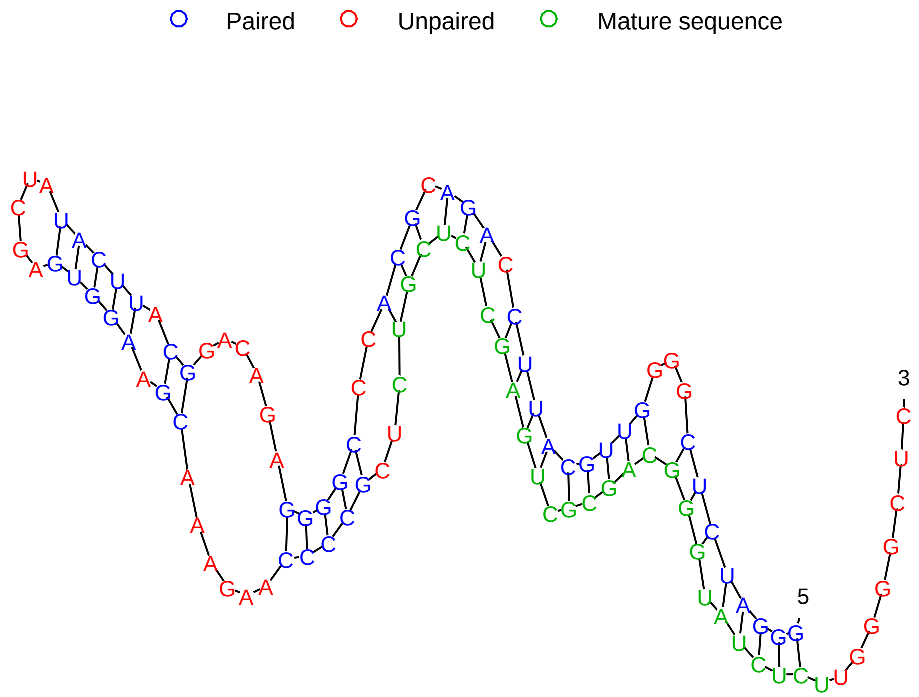

Stem loop (UMD3.1): chr16:36364600-36364710
Mature (UMD3.1): chr16:36364675-36364702
Mature seq len: 28
Total raw counts (9 samples): 12590
Average raw counts: 1399
Strand: Forward
Orientation: 3p
Minimum free energy: -35.80

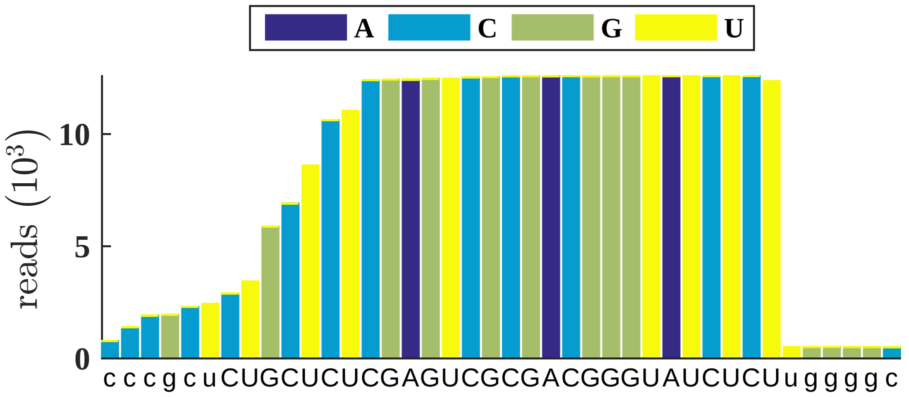

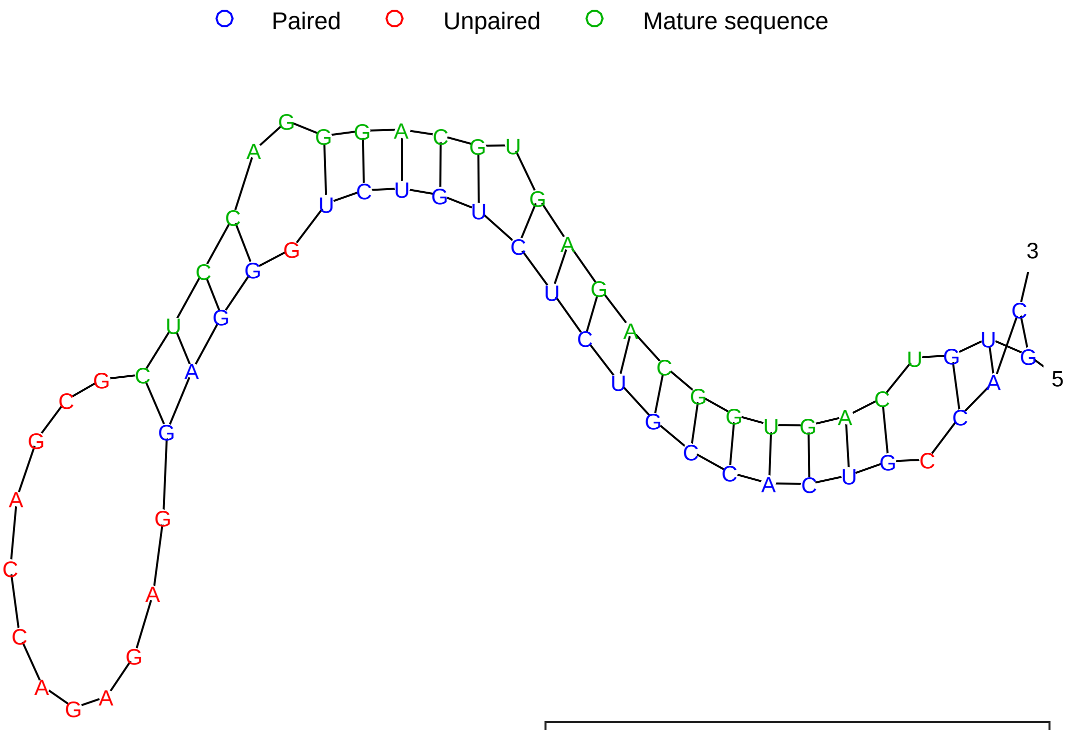

Stem loop (UMD3.1): chr16:71150565-71150628
Mature (UMD3.1): chr16:71150568-71150591
Mature seq len: 24
Total raw counts (9 samples): 1140
Average raw counts: 127
Strand: Forward
Orientation: 5p
Minimum free energy: -35.20

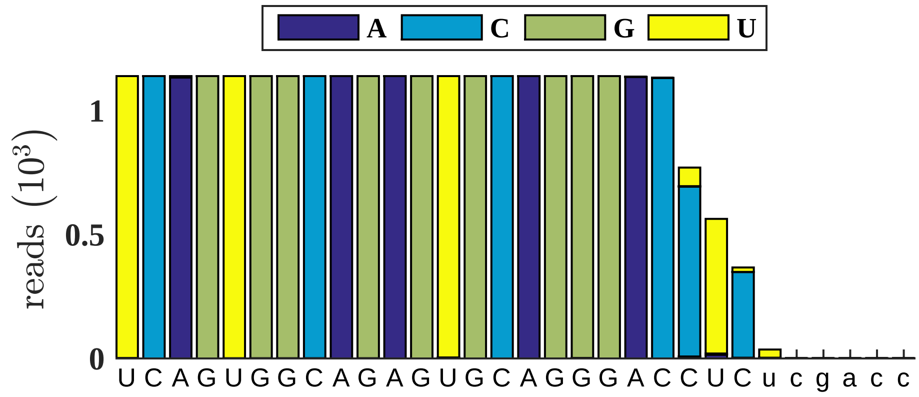

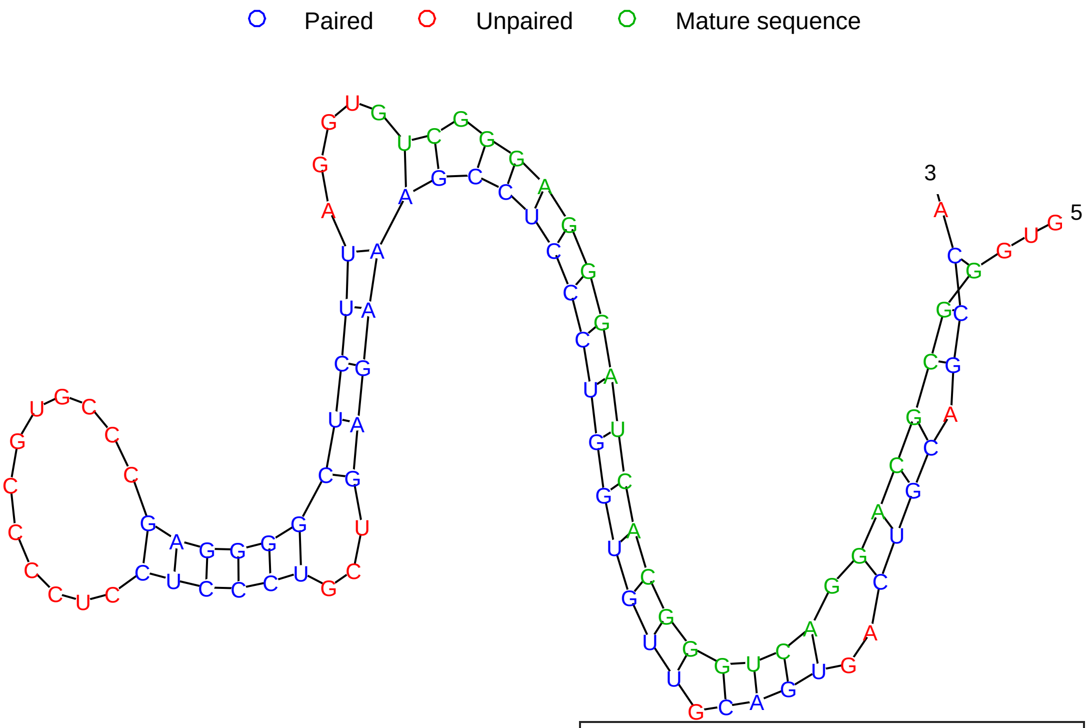

Stem loop (UMD3.1): chr16:75664621-75664724
Mature (UMD3.1): chr16:75664624-75664652
Mature seq len: 29
Total raw counts (9 samples): 699
Average raw counts: 78
Strand: Forward
Orientation: 5p
Minimum free energy: -51.40

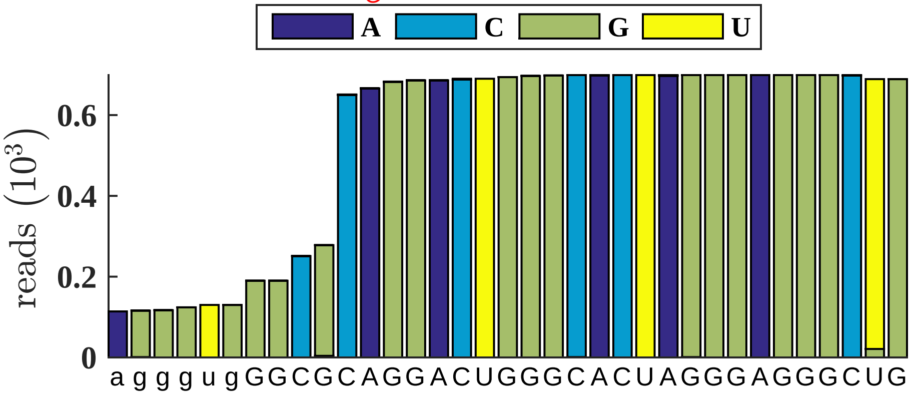

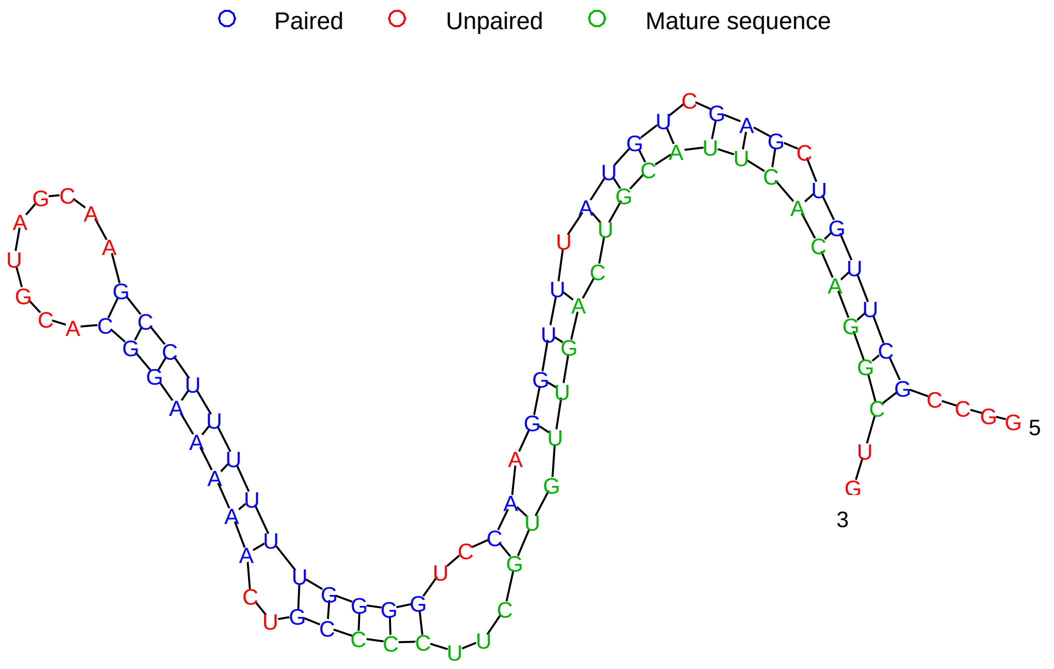

Stem loop (UMD3.1): chr17:36874181-36874272
Mature (UMD3.1): chr17:36874244-36874270
Mature seq len: 27
Total raw counts (9 samples): 1132
Average raw counts: 126
Strand: Forward
Orientation: 3p
Minimum free energy: -28.90

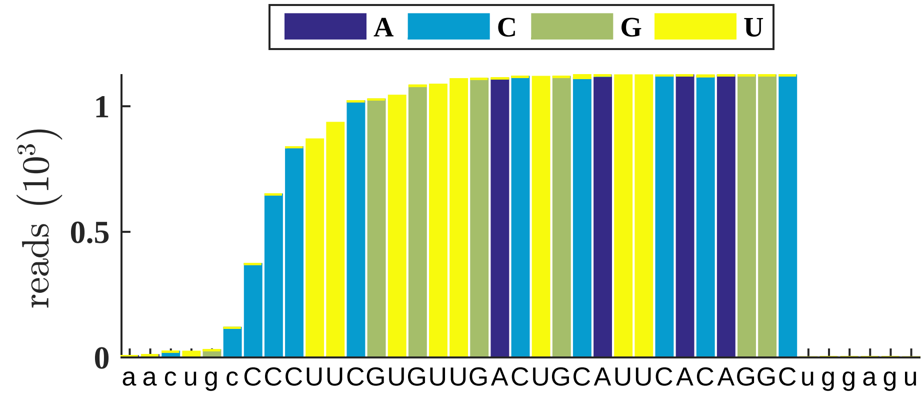

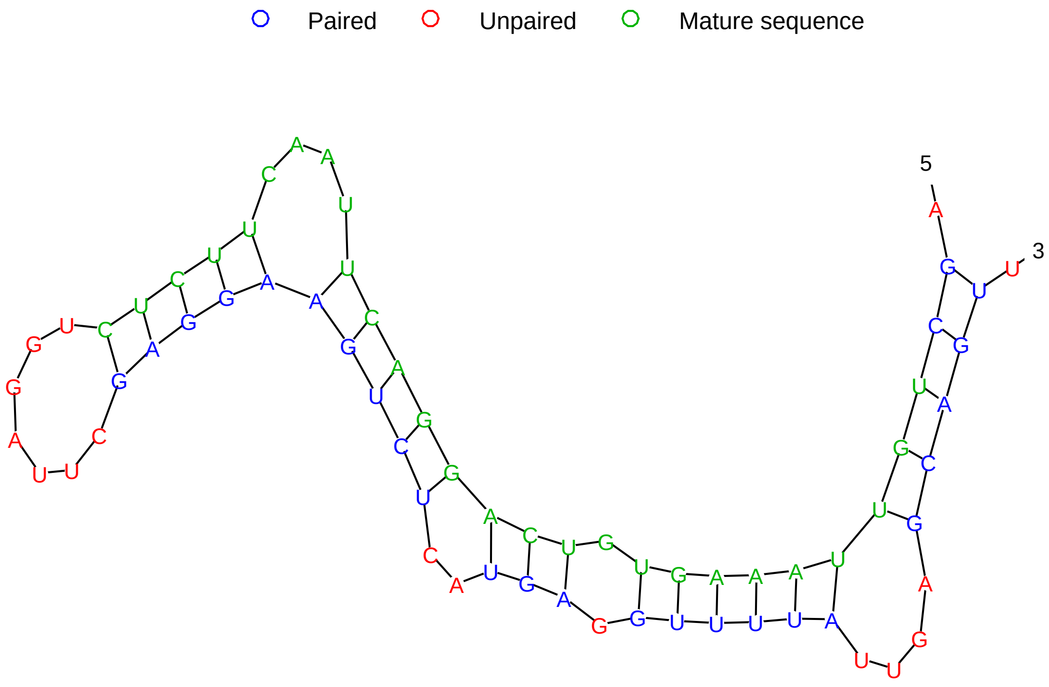

Stem loop (UMD3.1): chr17:62008711-62008779
Mature (UMD3.1): chr17:62008750-62008776
Mature seq len: 27
Total raw counts (9 samples): 675
Average raw counts: 75
Strand: Reverse
Orientation: 5p
Minimum free energy: -15.50

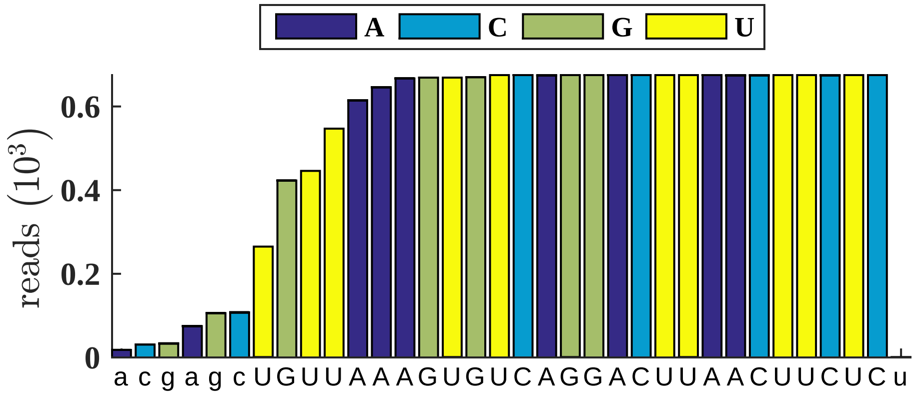

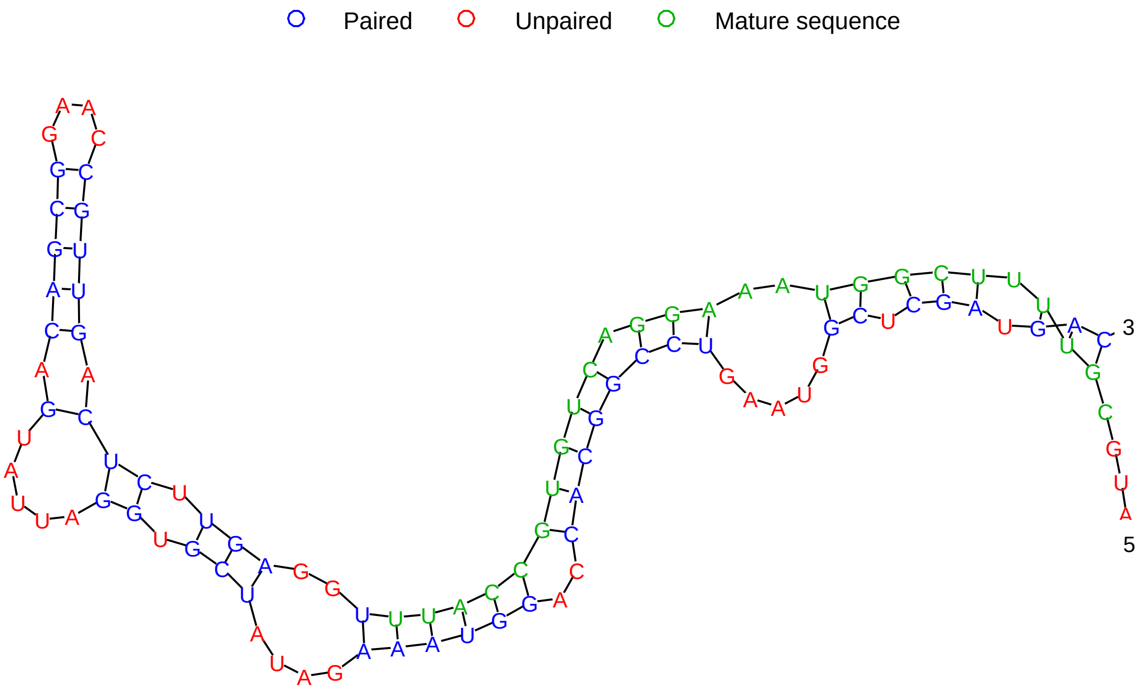

Stem loop (UMD3.1): chr17:66044665-66044766
Mature (UMD3.1): chr17:66044668-66044693
Mature seq len: 26
Total raw counts (9 samples): 835
Average raw counts: 93
Strand: Forward
Orientation: 5p
Minimum free energy: -26.60

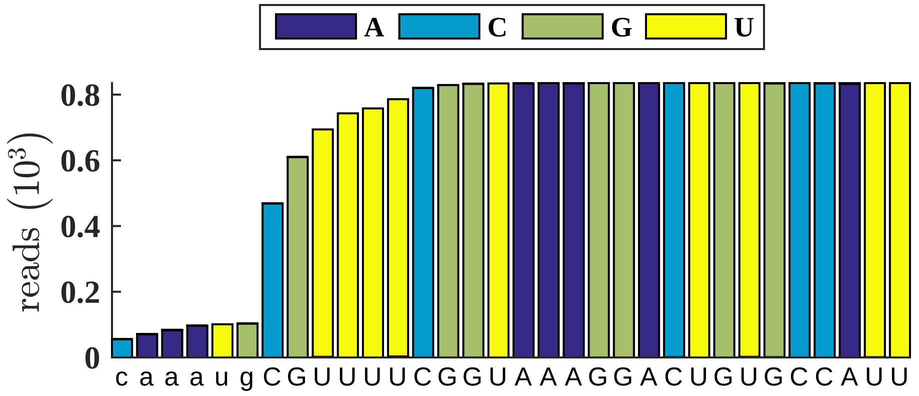

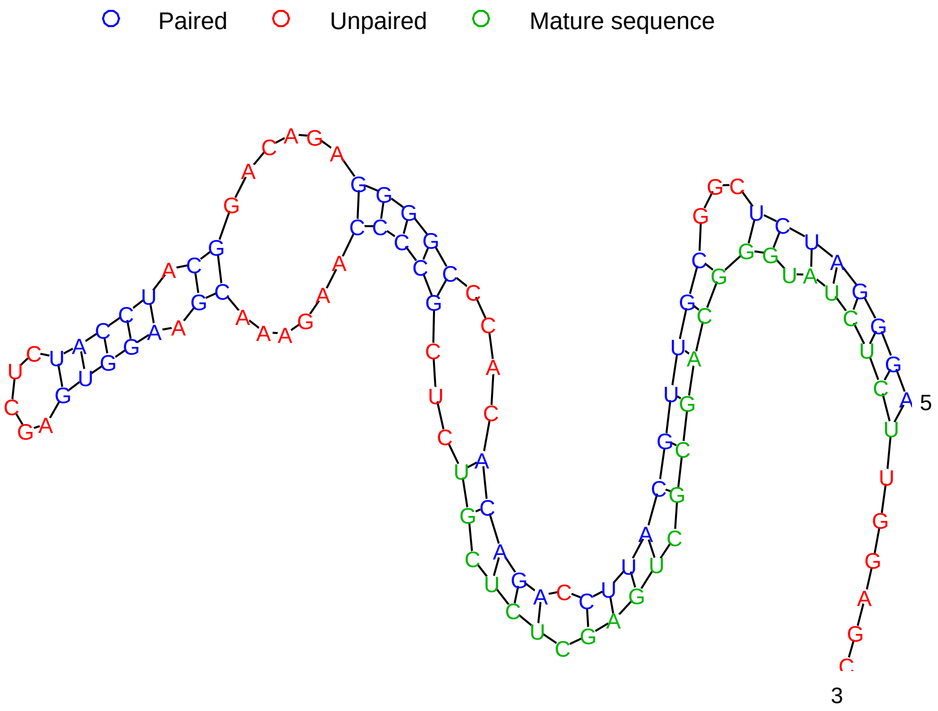

Stem loop (UMD3.1): chr17:72843276-72843385
Mature (UMD3.1): chr17:72843353-72843379
Mature seq len: 27
Total raw counts (9 samples): 14299
Average raw counts: 1589
Strand: Forward
Orientation: 3p
Minimum free energy: -35.90

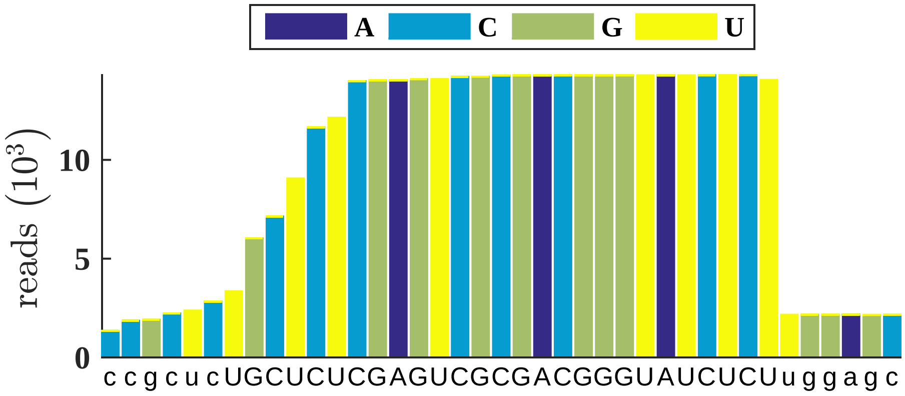

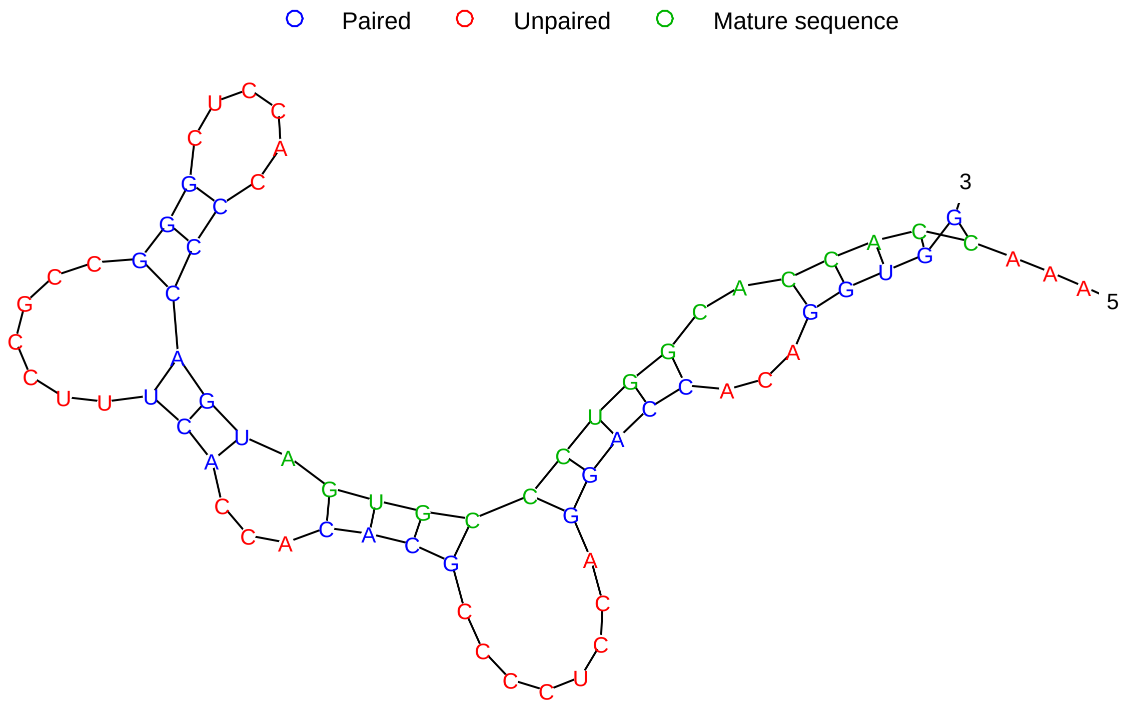

Stem loop (UMD3.1): chr17:74211764-74211836
Mature (UMD3.1): chr17:74211767-74211783
Mature seq len: 17
Total raw counts (9 samples): 798
Average raw counts: 89
Strand: Forward
Orientation: 5p
Minimum free energy: -20.70

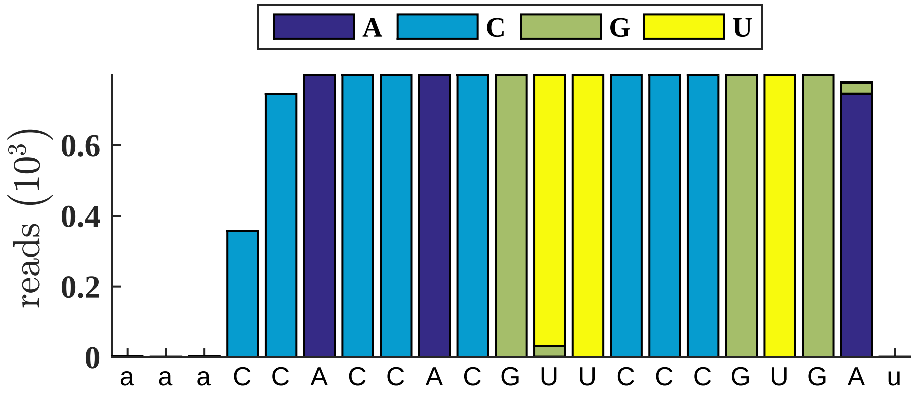

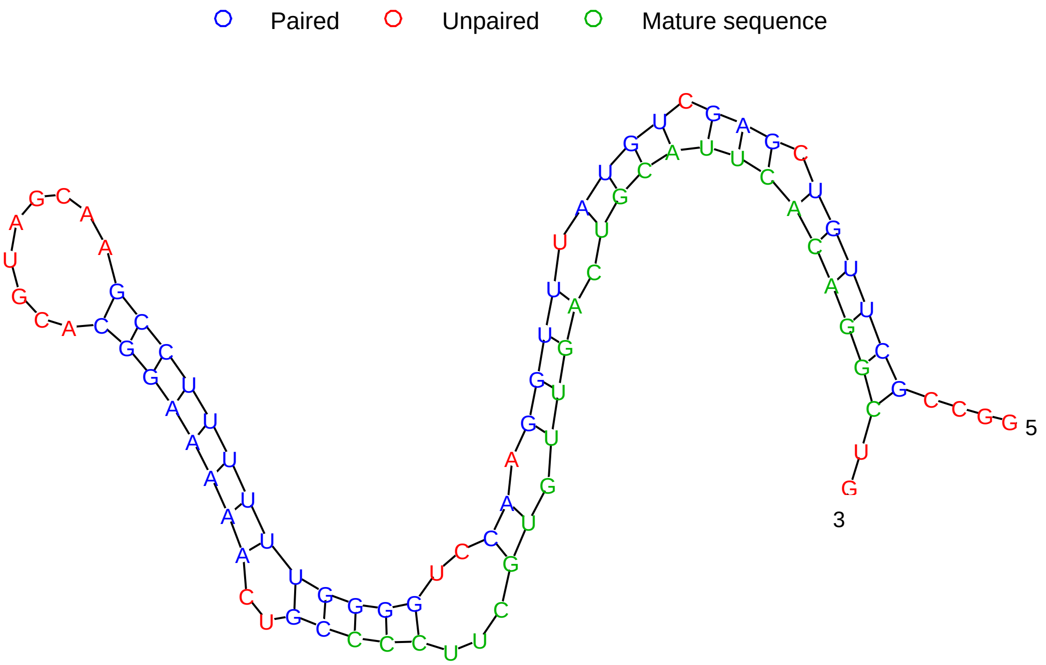

Stem loop (UMD3.1): chr18:11451640-11451731
Mature (UMD3.1): chr18:11451642-11451668
Mature seq len: 27
Total raw counts (9 samples): 1254
Average raw counts: 140
Strand: Reverse
Orientation: 3p
Minimum free energy: -28.90

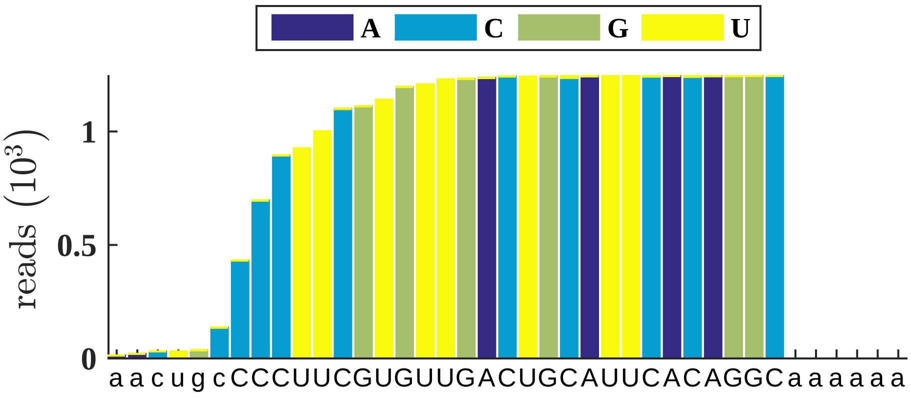

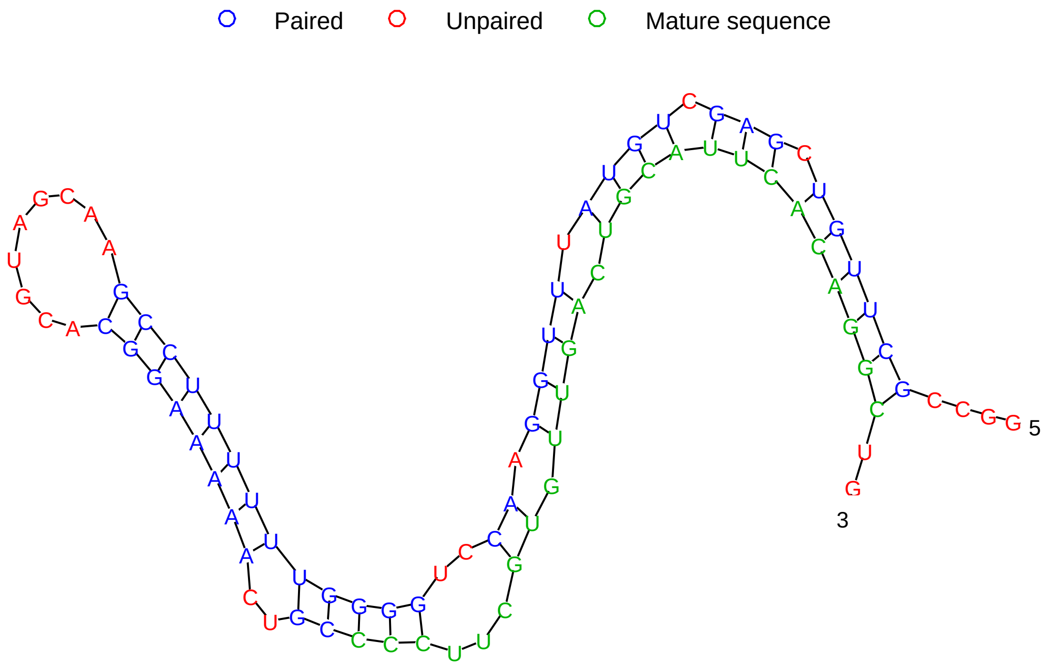

Stem loop (UMD3.1): chr18:11453025-11453116
Mature (UMD3.1): chr18:11453027-11453053
Mature seq len: 27
Total raw counts (9 samples): 1209
Average raw counts: 135
Strand: Reverse
Orientation: 3p
Minimum free energy: -28.90

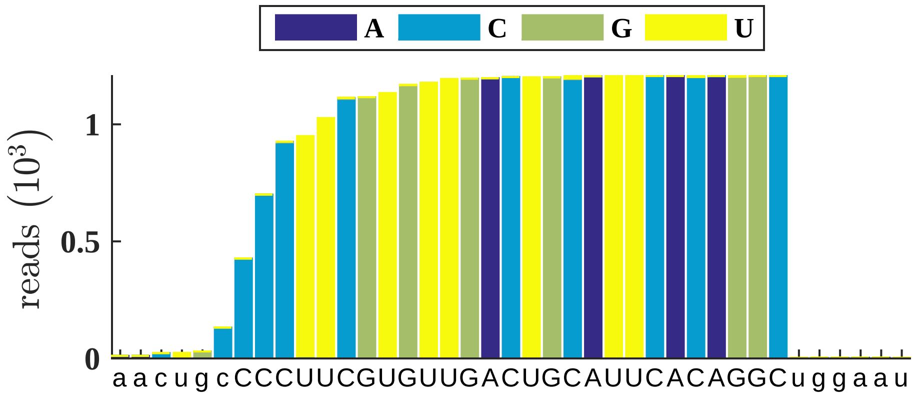

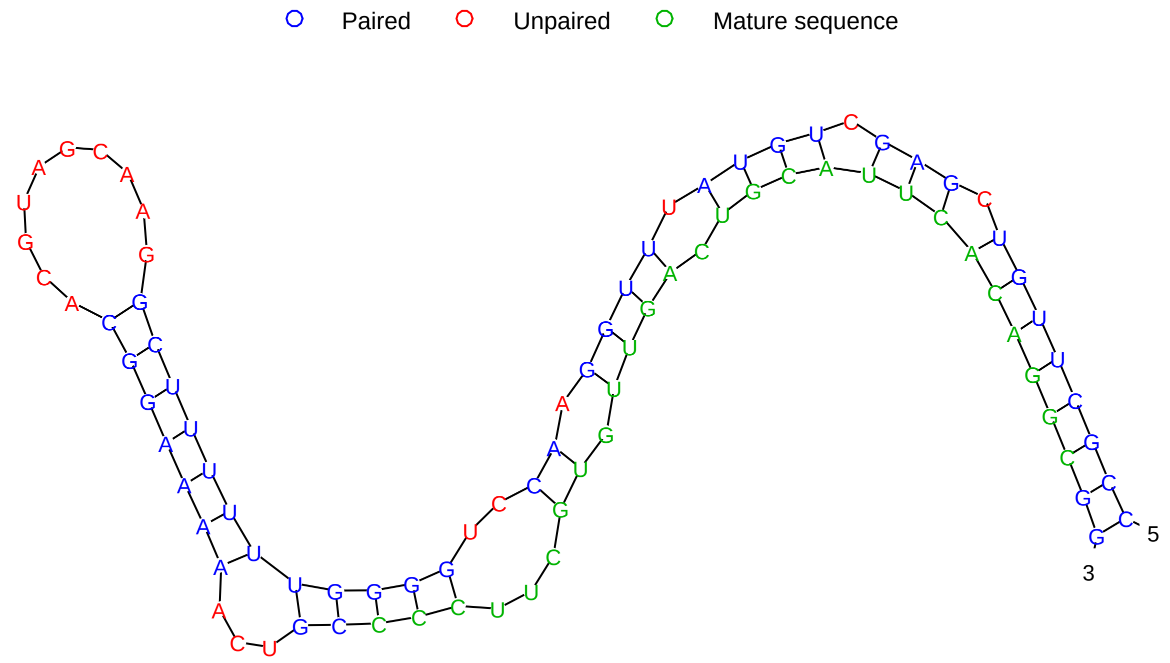

Stem loop (UMD3.1): chr18:11459351-11459440
Mature (UMD3.1): chr18:11459353-11459379
Mature seq len: 27
Total raw counts (9 samples): 1204
Average raw counts: 134
Strand: Reverse
Orientation: 3p
Minimum free energy: -30.30

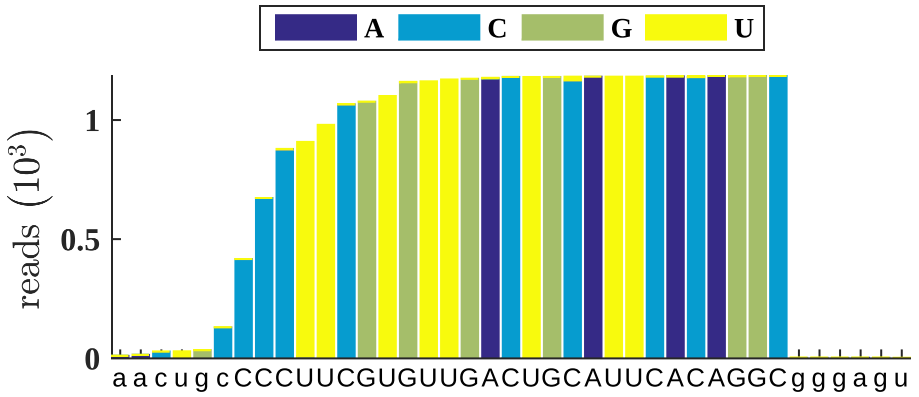

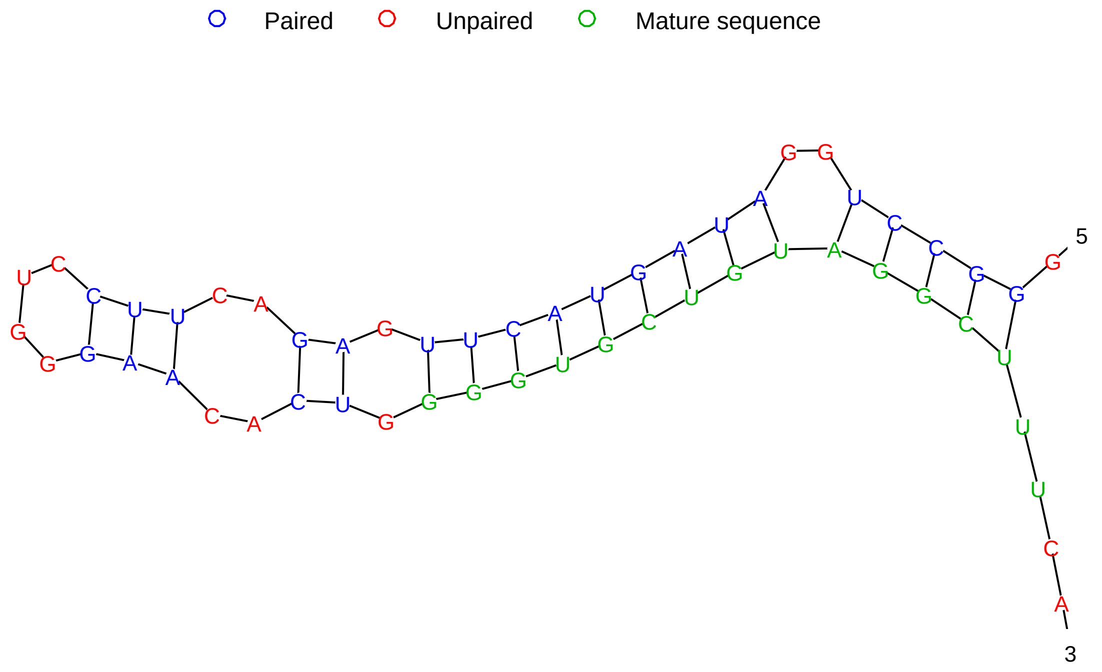

Stem loop (UMD3.1): chr18:22622866-22622920
Mature (UMD3.1): chr18:22622903-22622918
Mature seq len: 16
Total raw counts (9 samples): 22833
Average raw counts: 2537
Strand: Forward
Orientation: 3p
Minimum free energy: -19.70

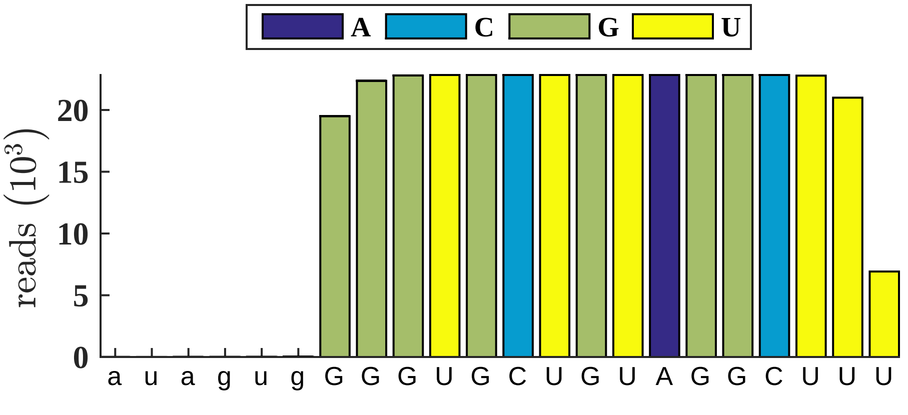

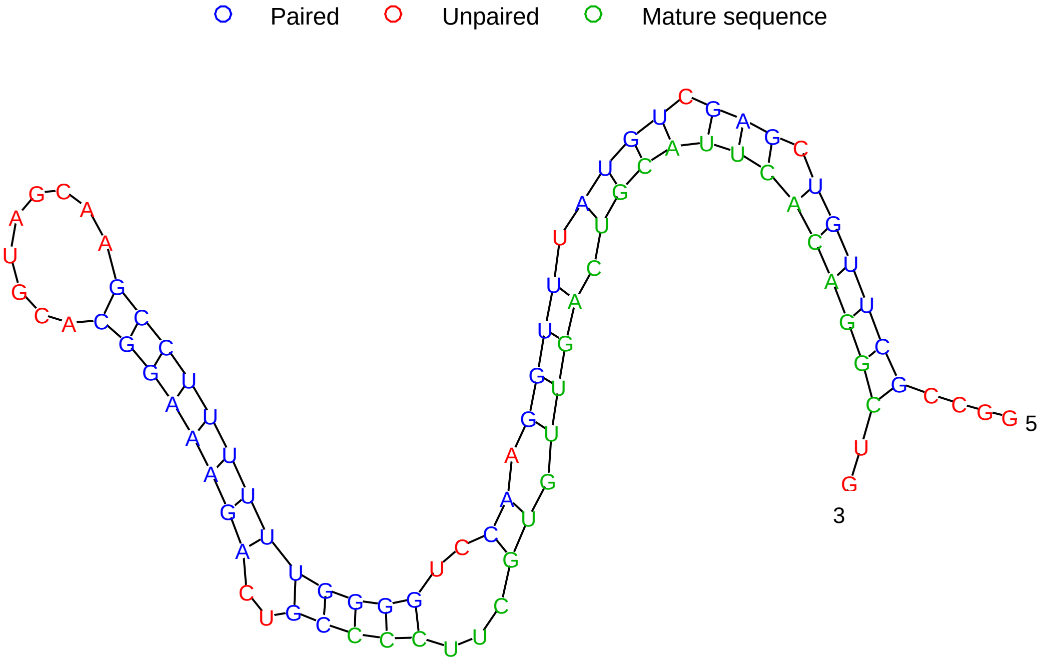

Stem loop (UMD3.1): chr18:30851396-30851487
Mature (UMD3.1): chr18:30851459-30851485
Mature seq len: 27
Total raw counts (9 samples): 1153
Average raw counts: 129
Strand: Forward
Orientation: 3p
Minimum free energy: -29.00

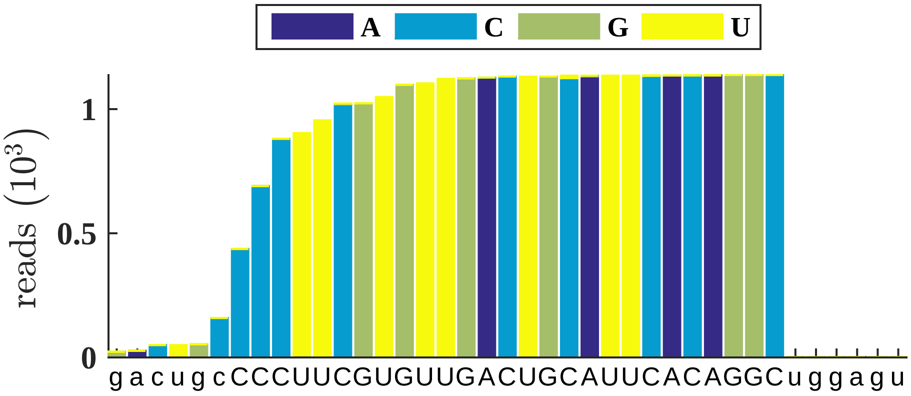

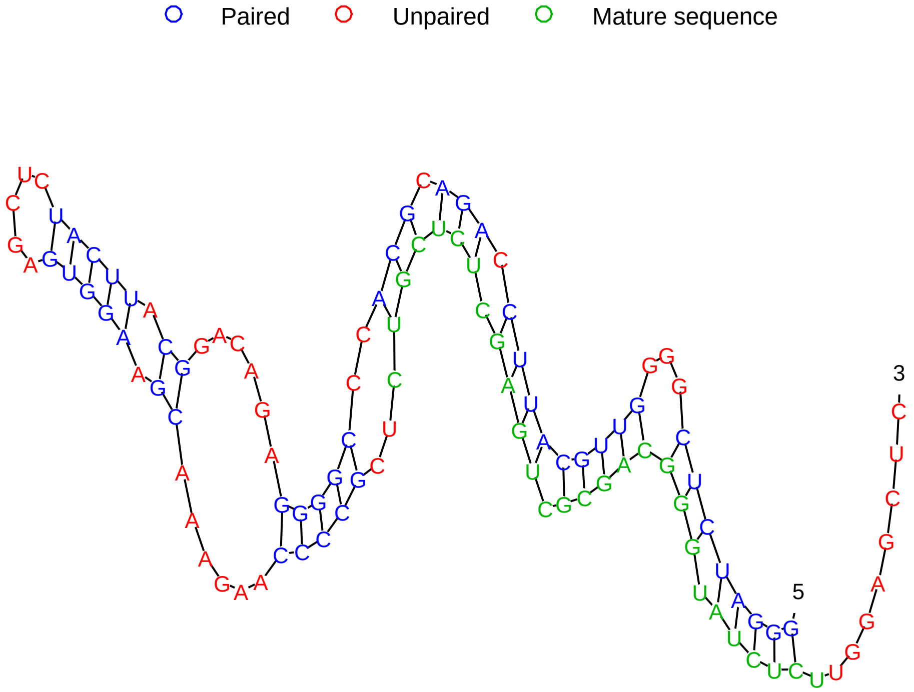

Stem loop (UMD3.1): chr18:30852077-30852187
Mature (UMD3.1): chr18:30852152-30852179
Mature seq len: 28
Total raw counts (9 samples): 13353
Average raw counts: 1484
Strand: Forward
Orientation: 3p
Minimum free energy: -35.50

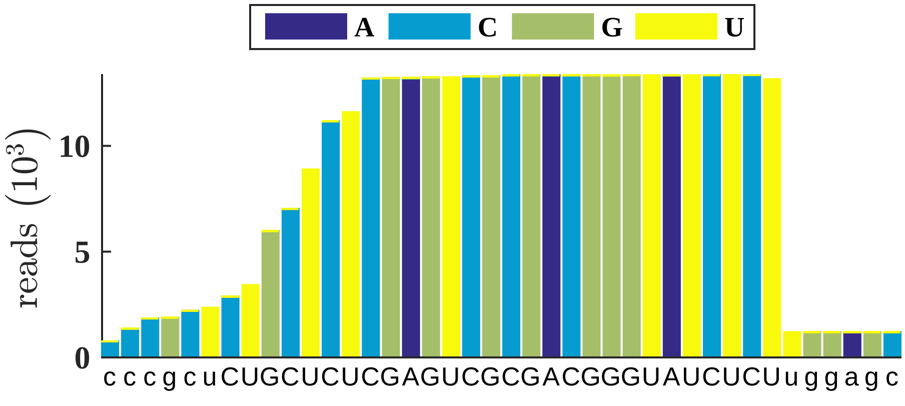

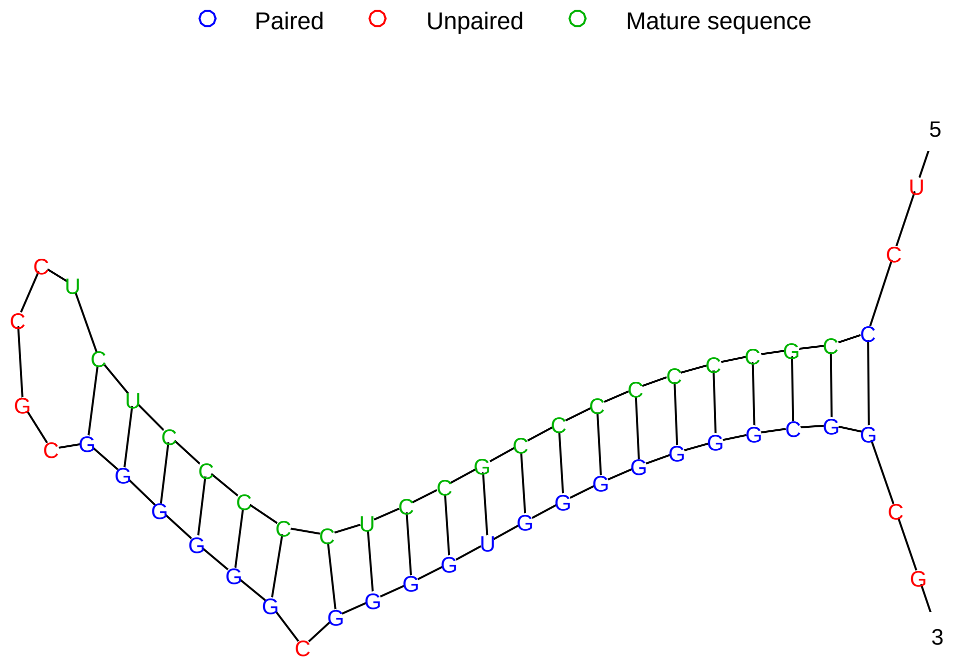

Stem loop (UMD3.1): chr18:58243444-58243495
Mature (UMD3.1): chr18:58243447-58243467
Mature seq len: 21
Total raw counts (9 samples): 867
Average raw counts: 97
Strand: Forward
Orientation: 5p
Minimum free energy: -49.10

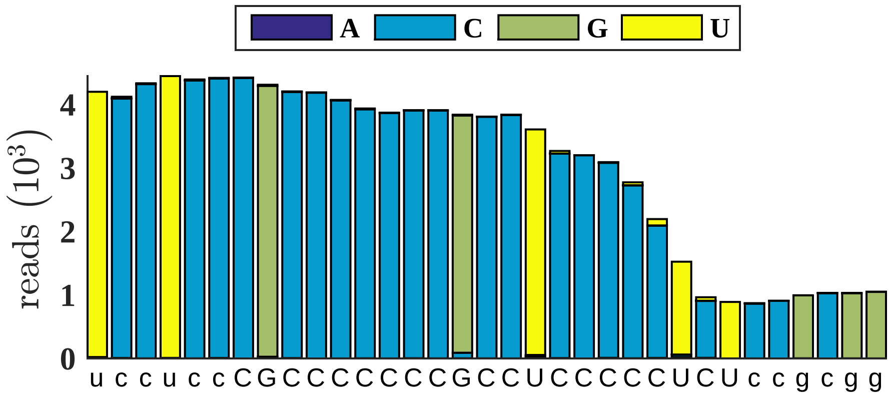

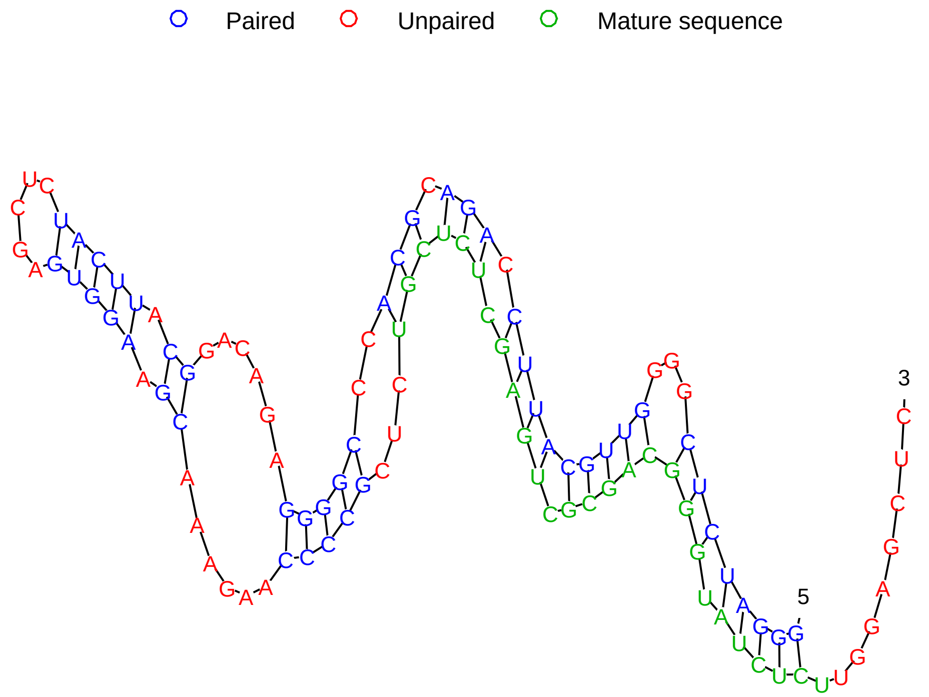

Stem loop (UMD3.1): chr19:38176-38286
Mature (UMD3.1): chr19:38252-38278
Mature seq len: 27
Total raw counts (9 samples): 13947
Average raw counts: 1550
Strand: Forward
Orientation: 3p
Minimum free energy: -35.50

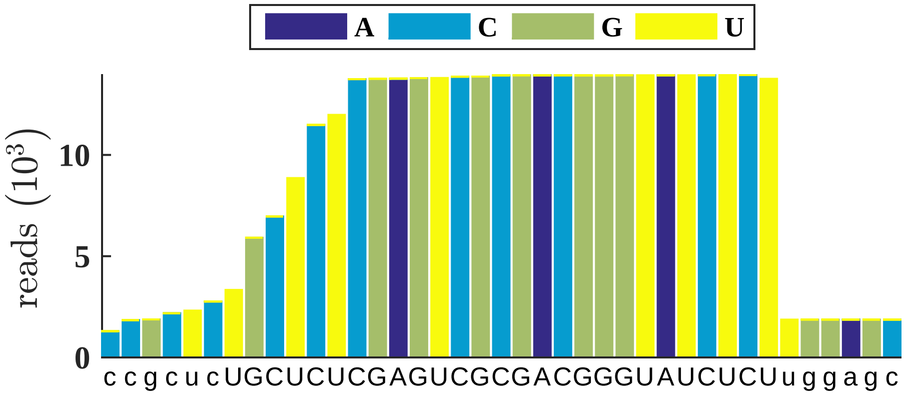

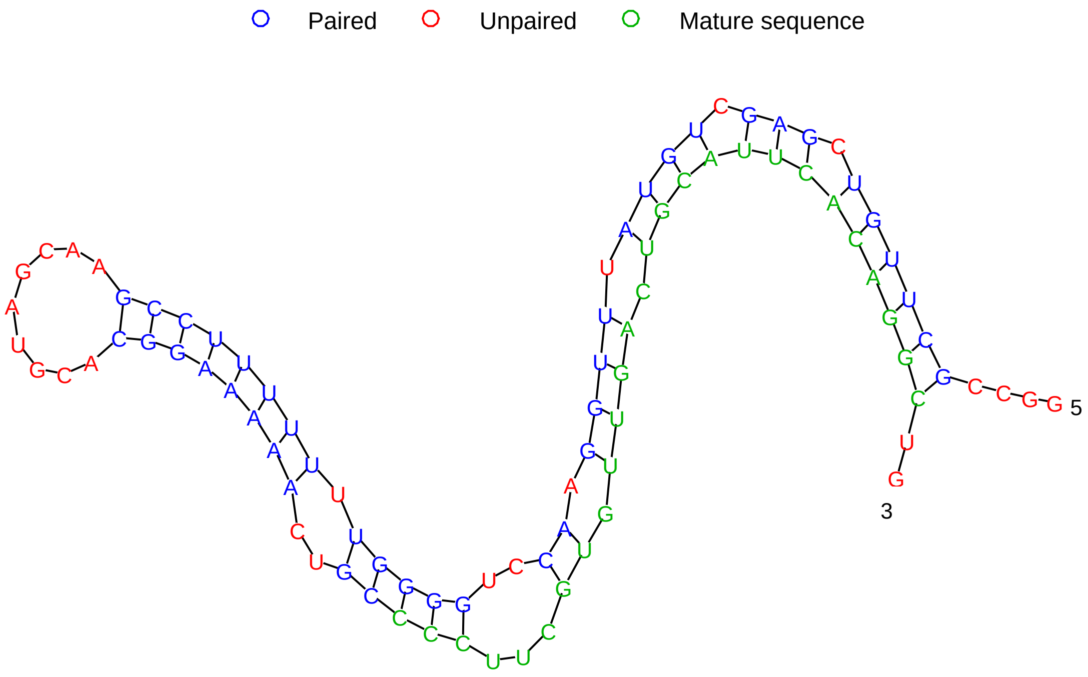

Stem loop (UMD3.1): chr19:40479228-40479320
Mature (UMD3.1): chr19:40479230-40479256
Mature seq len: 27
Total raw counts (9 samples): 1224
Average raw counts: 136
Strand: Reverse
Orientation: 3p
Minimum free energy: -29.70

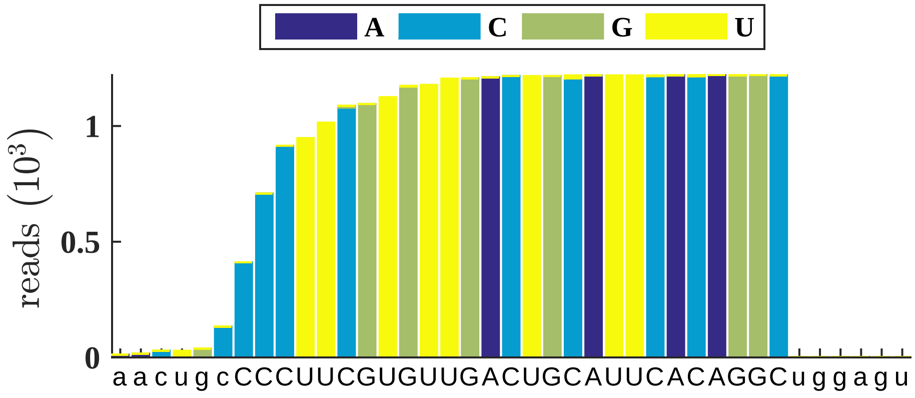

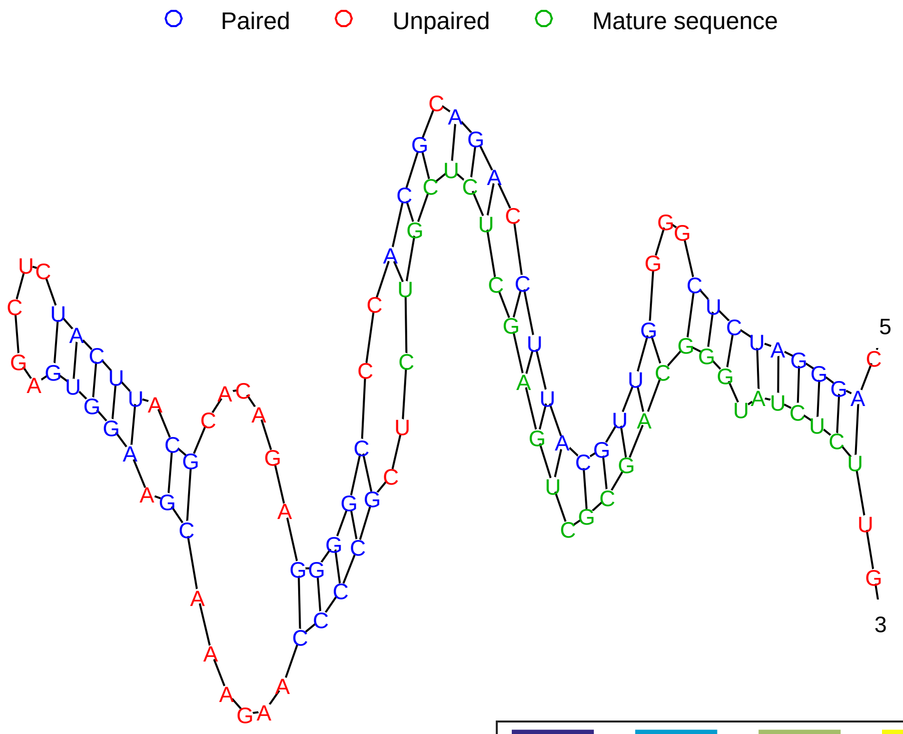

Stem loop (UMD3.1): chr19:57865574-57865680
Mature (UMD3.1): chr19:57865576-57865603
Mature seq len: 28
Total raw counts (9 samples): 14040
Average raw counts: 1560
Strand: Reverse
Orientation: 3p
Minimum free energy: -35.70

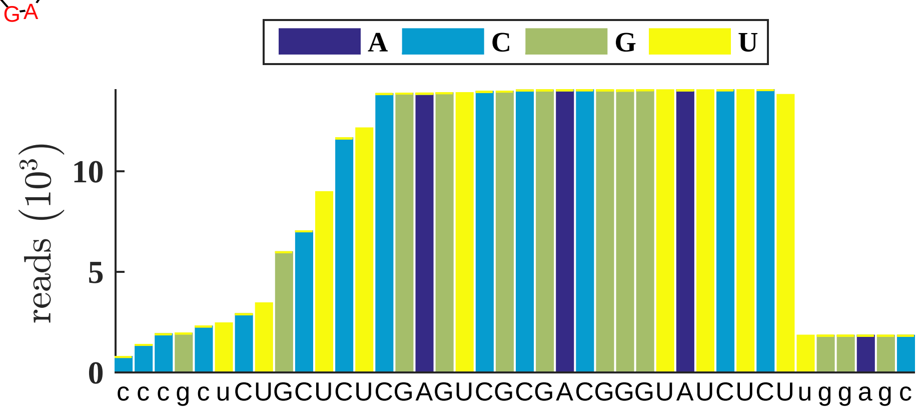

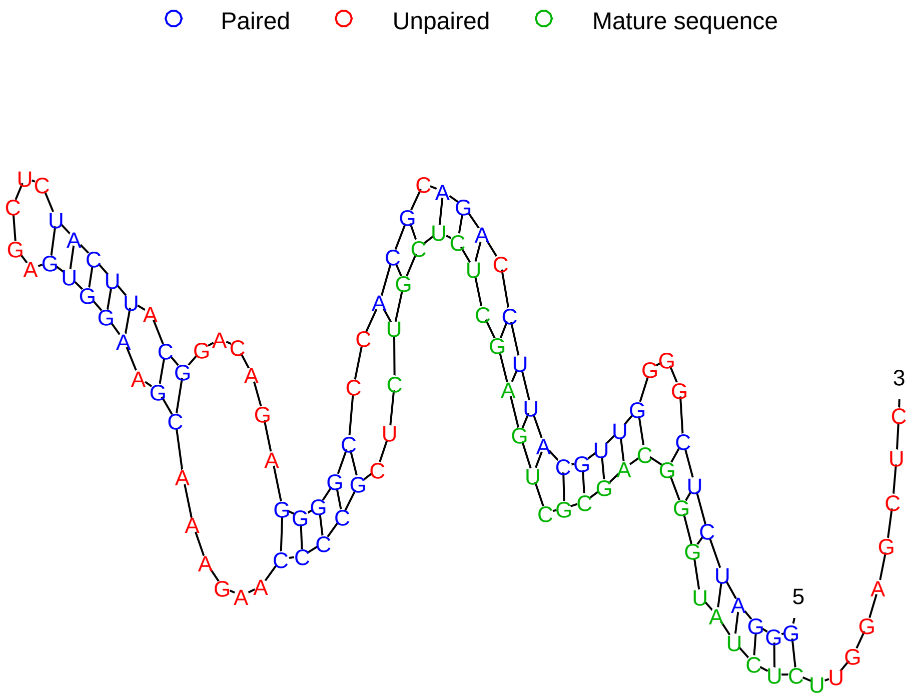

Stem loop (UMD3.1): chr19:61447-61557
Mature (UMD3.1): chr19:61522-61549
Mature seq len: 28
Total raw counts (9 samples): 13591
Average raw counts: 1511
Strand: Forward
Orientation: 3p
Minimum free energy: -35.50

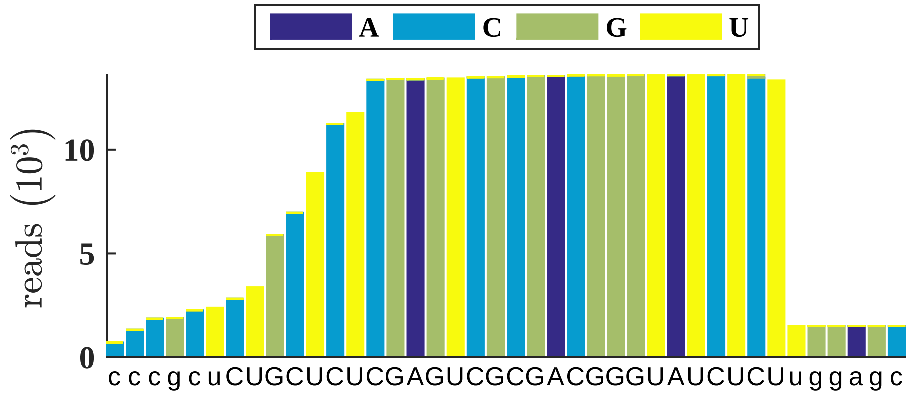

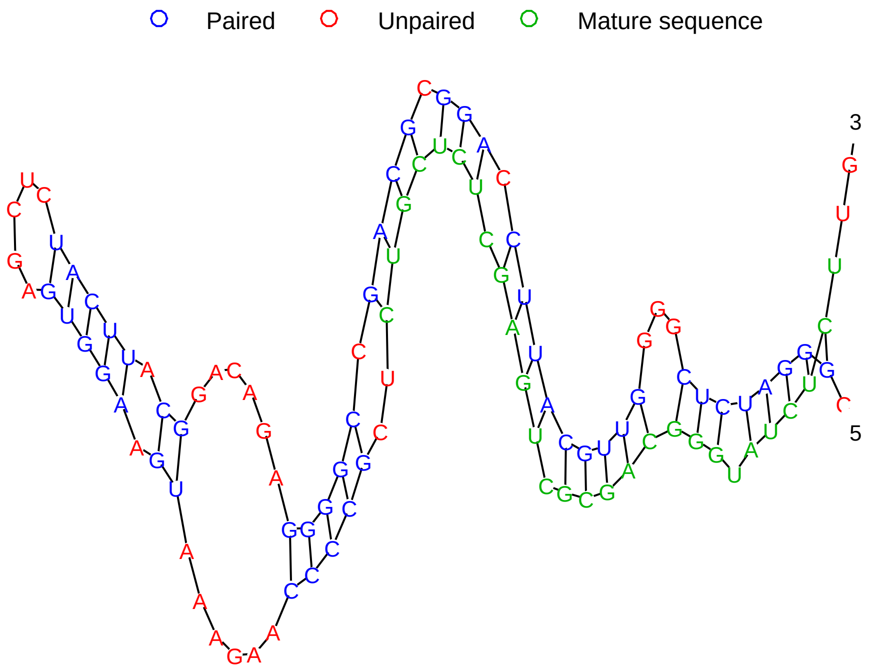

Stem loop (UMD3.1): chr19:61930204-61930309
Mature (UMD3.1): chr19:61930206-61930233
Mature seq len: 28
Total raw counts (9 samples): 13617
Average raw counts: 1513
Strand: Reverse
Orientation: 3p
Minimum free energy: -36.30

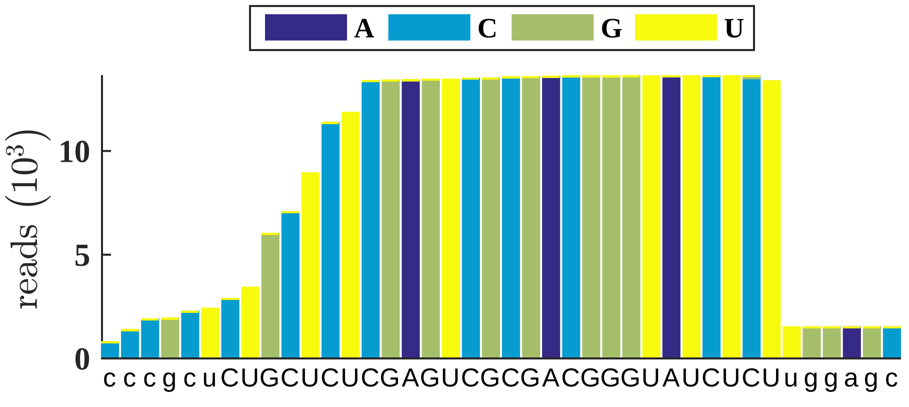

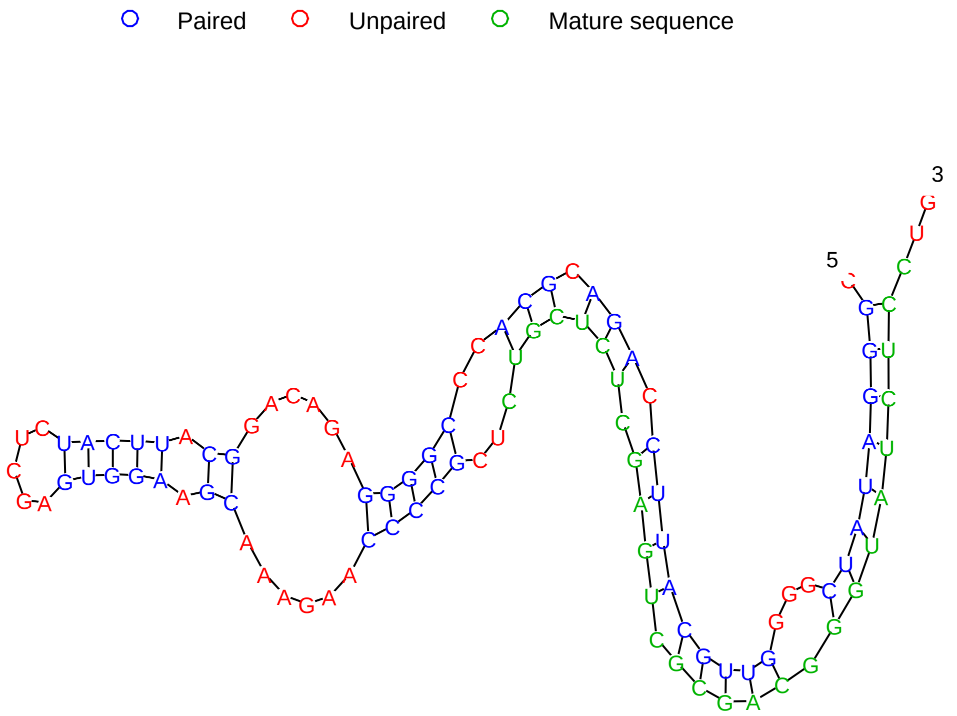

Stem loop (UMD3.1): chr2:100650692-100650797
Mature (UMD3.1): chr2:100650768-100650795
Mature seq len: 28
Total raw counts (9 samples): 1740
Average raw counts: 194
Strand: Forward
Orientation: 3p
Minimum free energy: -38.30

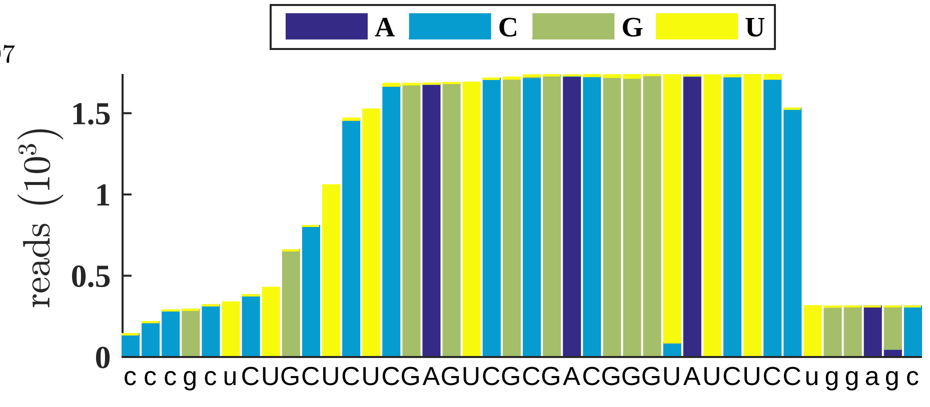

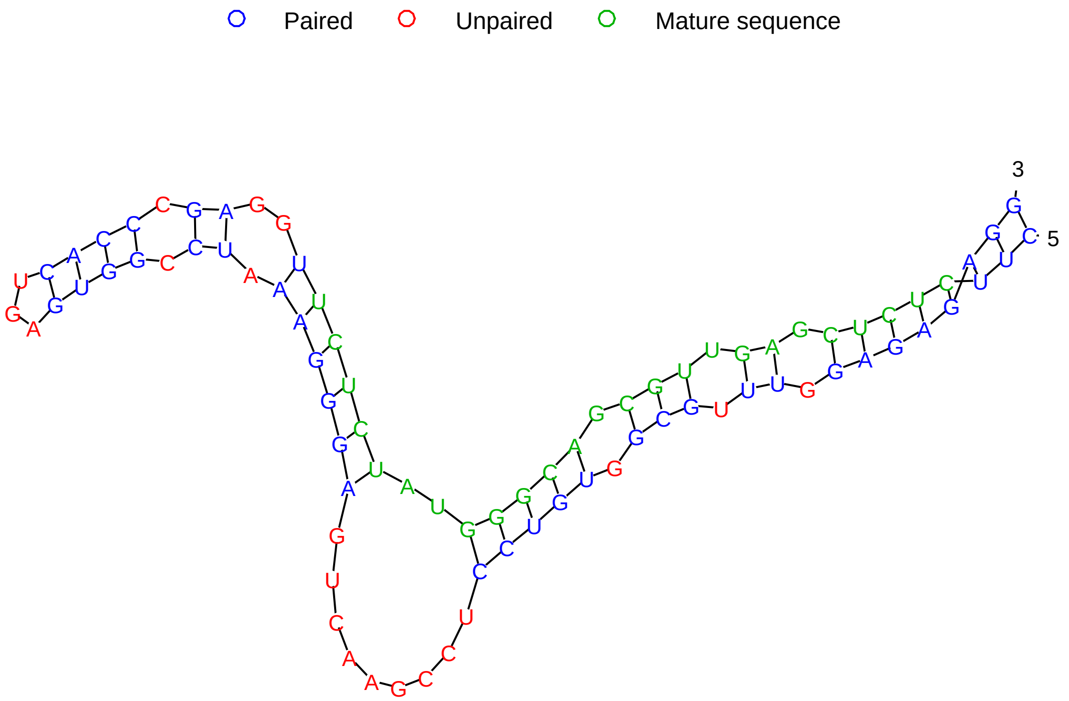

Stem loop (UMD3.1): chr2:162414-162498
Mature (UMD3.1): chr2:162417-162441
Mature seq len: 25
Total raw counts (9 samples): 737
Average raw counts: 82
Strand: Forward
Orientation: 5p
Minimum free energy: -35.60

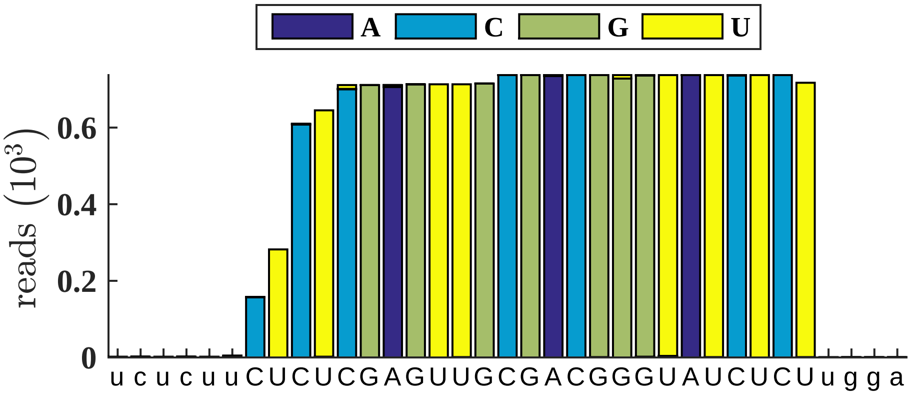

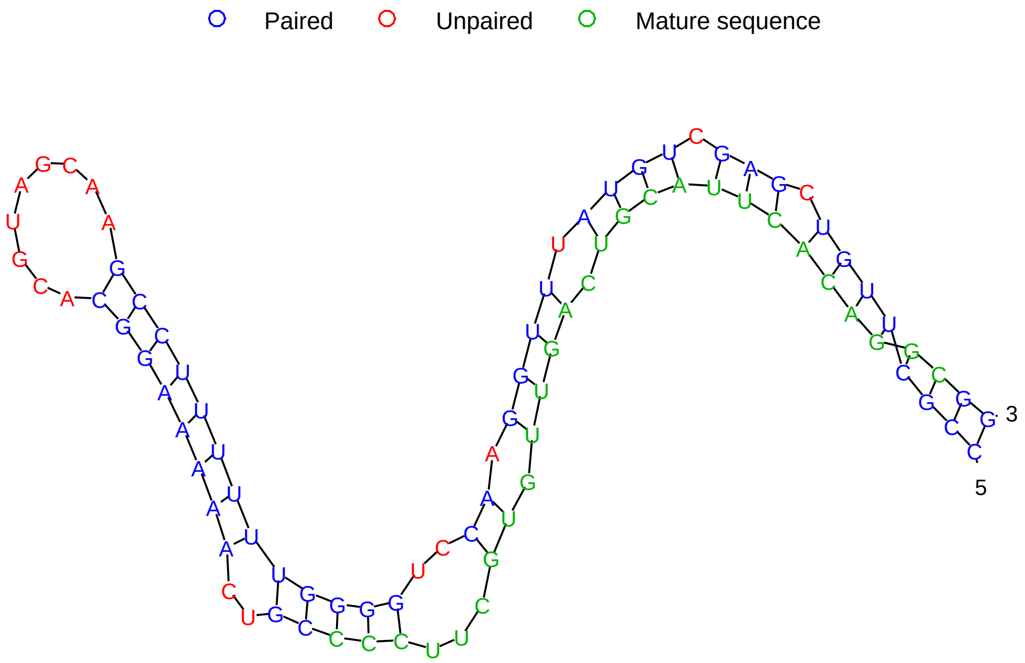

Stem loop (UMD3.1): chr2:8752342-8752431
Mature (UMD3.1): chr2:8752403-8752429
Mature seq len: 27
Total raw counts (9 samples): 1226
Average raw counts: 137
Strand: Forward
Orientation: 3p
Minimum free energy: -33.20

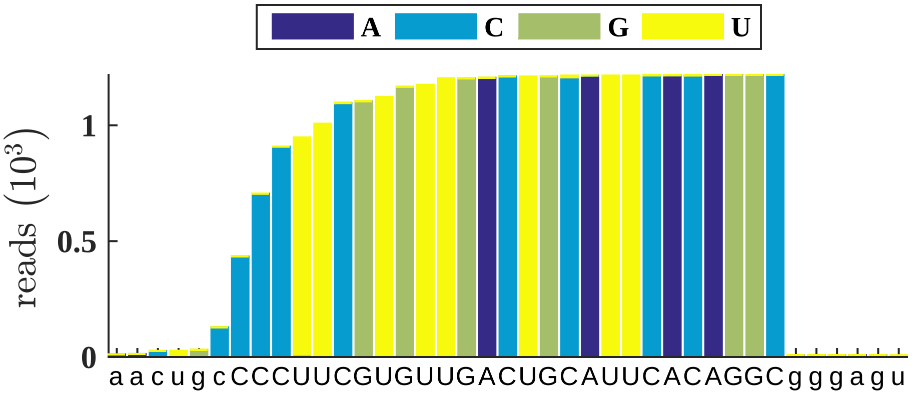

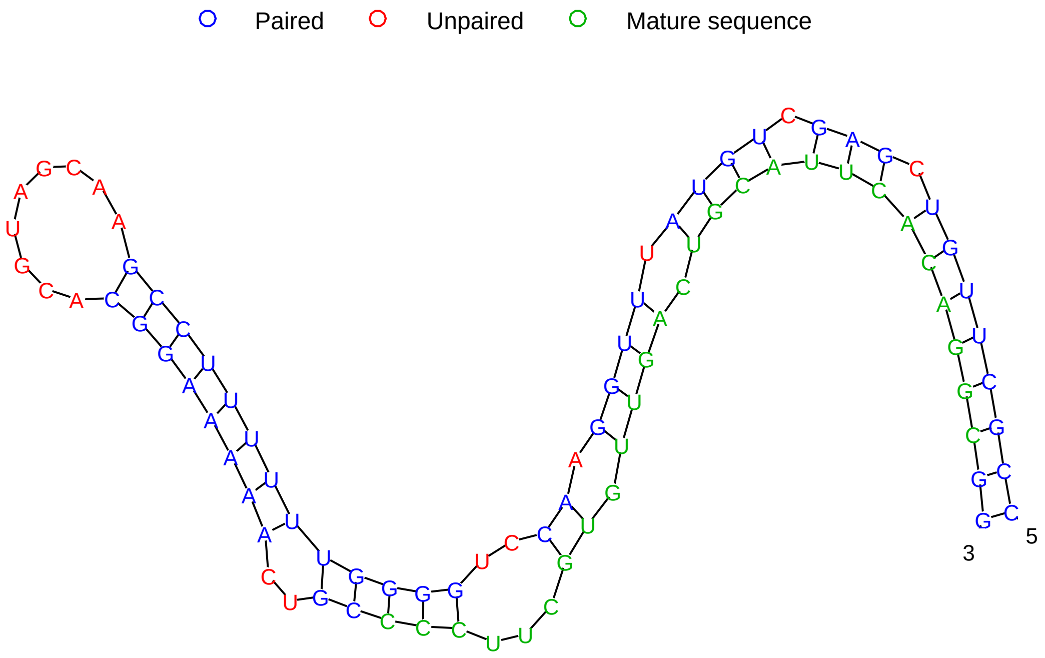

Stem loop (UMD3.1): chr20:23543210-23543299
Mature (UMD3.1): chr20:23543271-23543297
Mature seq len: 27
Total raw counts (9 samples): 1205
Average raw counts: 134
Strand: Forward
Orientation: 3p
Minimum free energy: -33.20

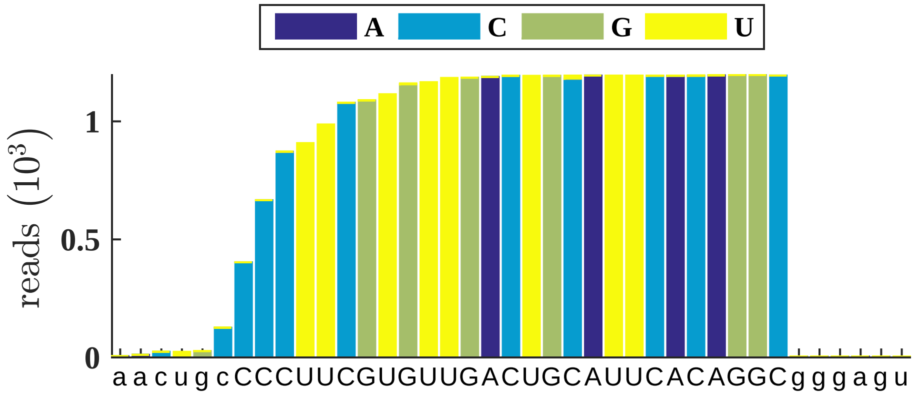

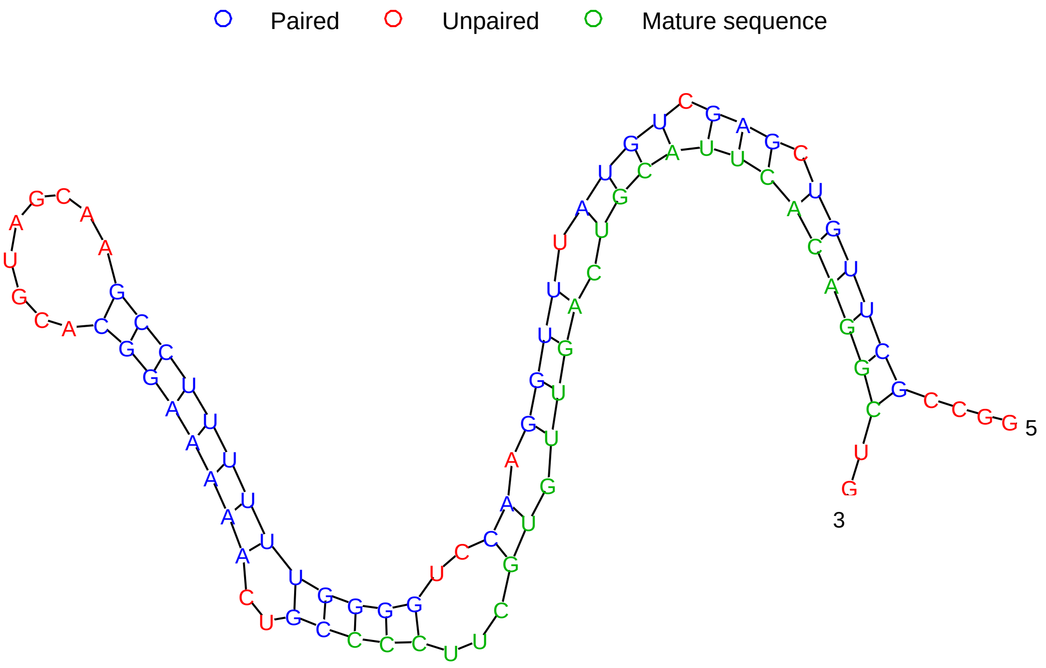

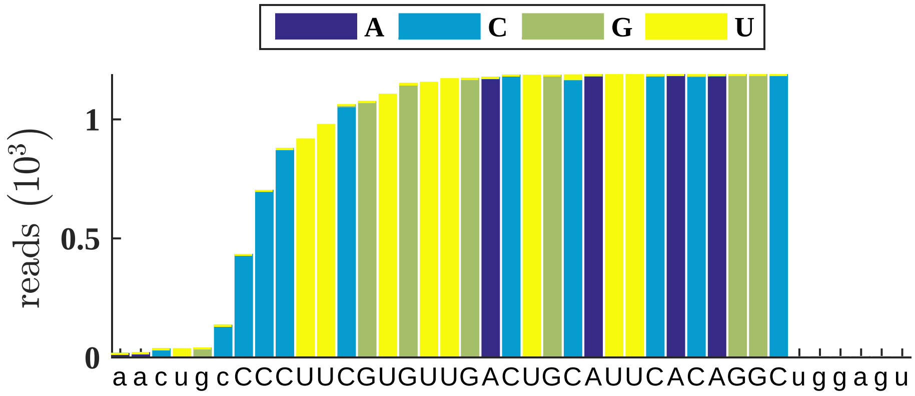

Stem loop (UMD3.1): chr20:2988017-2988108
Mature (UMD3.1): chr20:2988080-2988106
Mature seq len: 27
Total raw counts (9 samples): 1189
Average raw counts: 133
Strand: Forward
Orientation: 3p
Minimum free energy: -28.90

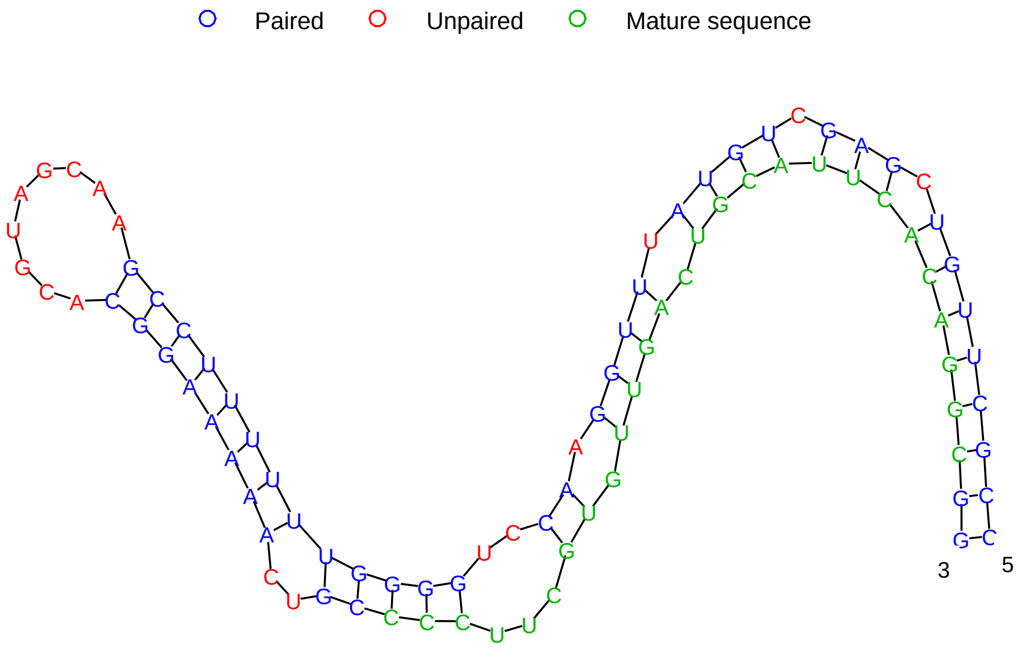

Stem loop (UMD3.1): chr20:2990024-2990113
Mature (UMD3.1): chr20:2990085-2990111
Mature seq len: 27
Total raw counts (9 samples): 1227
Average raw counts: 137
Strand: Forward
Orientation: 3p
Minimum free energy: -33.20

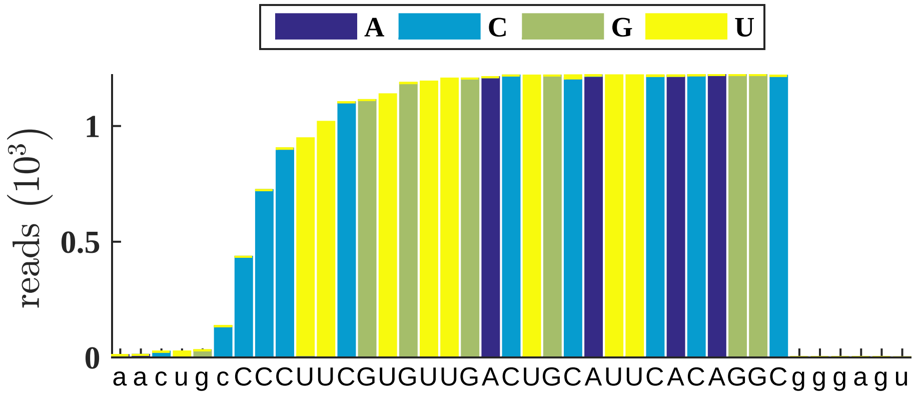

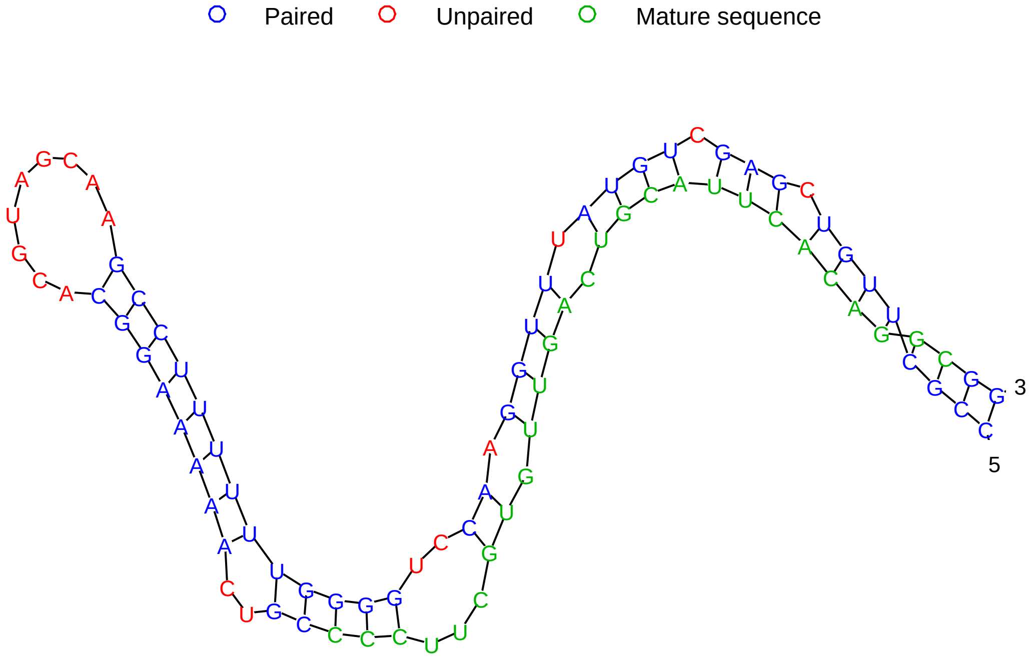

Stem loop (UMD3.1): chr20:2991394-2991483
Mature (UMD3.1): chr20:2991455-2991481
Mature seq len: 27
Total raw counts (9 samples): 1188
Average raw counts: 132
Strand: Forward
Orientation: 3p
Minimum free energy: -33.20

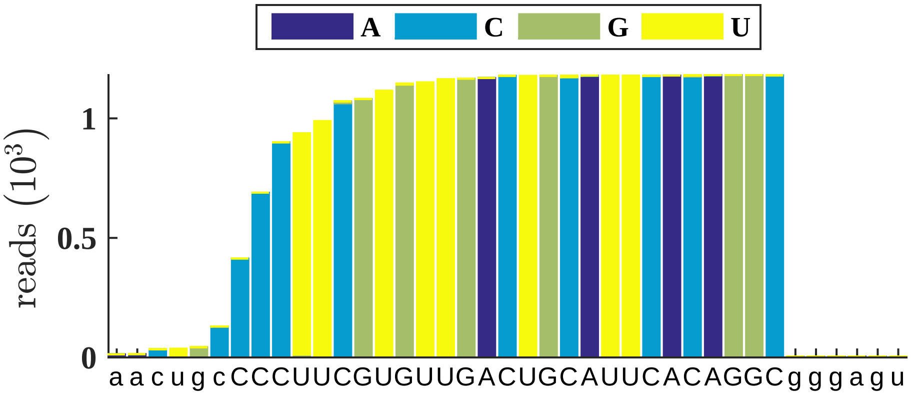

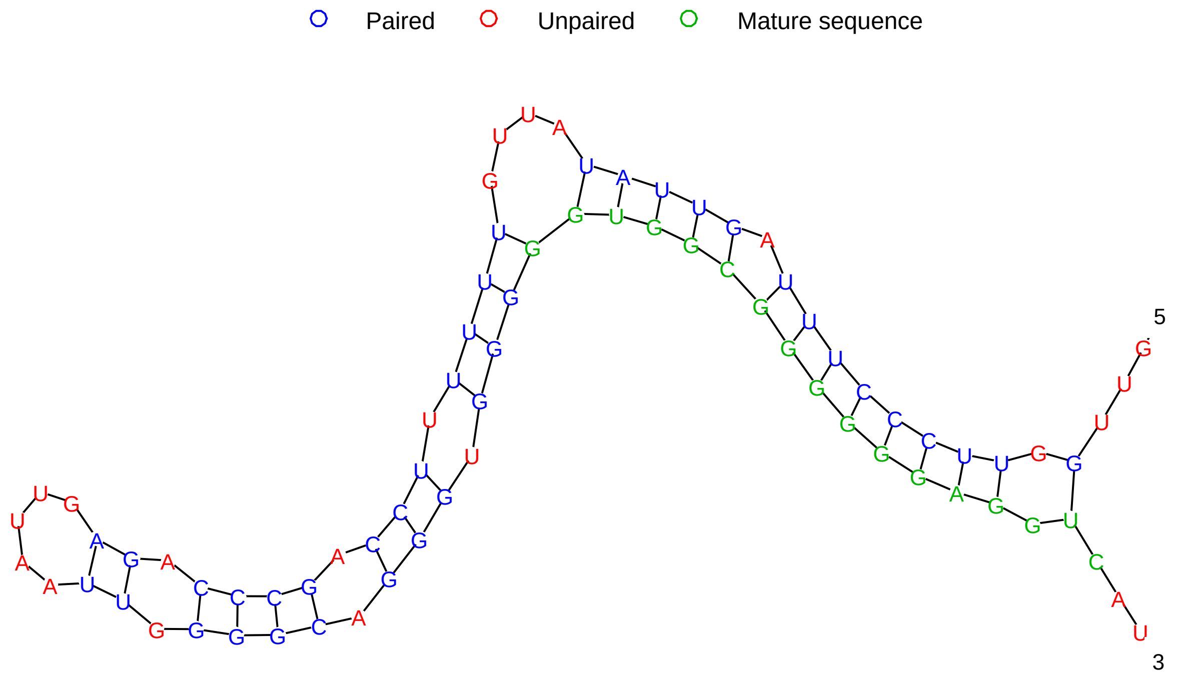

Stem loop (UMD3.1): chr20:55442869-55442946
Mature (UMD3.1): chr20:55442871-55442887
Mature seq len: 17
Total raw counts (9 samples): 1059
Average raw counts: 118
Strand: Reverse
Orientation: 3p
Minimum free energy: -21.20

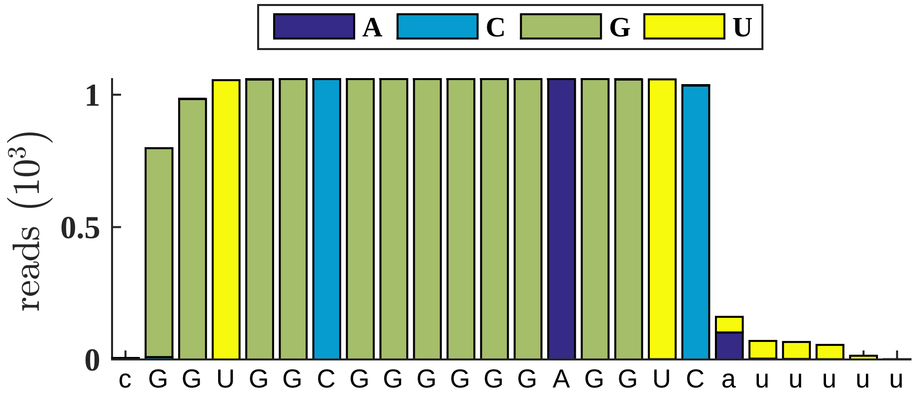

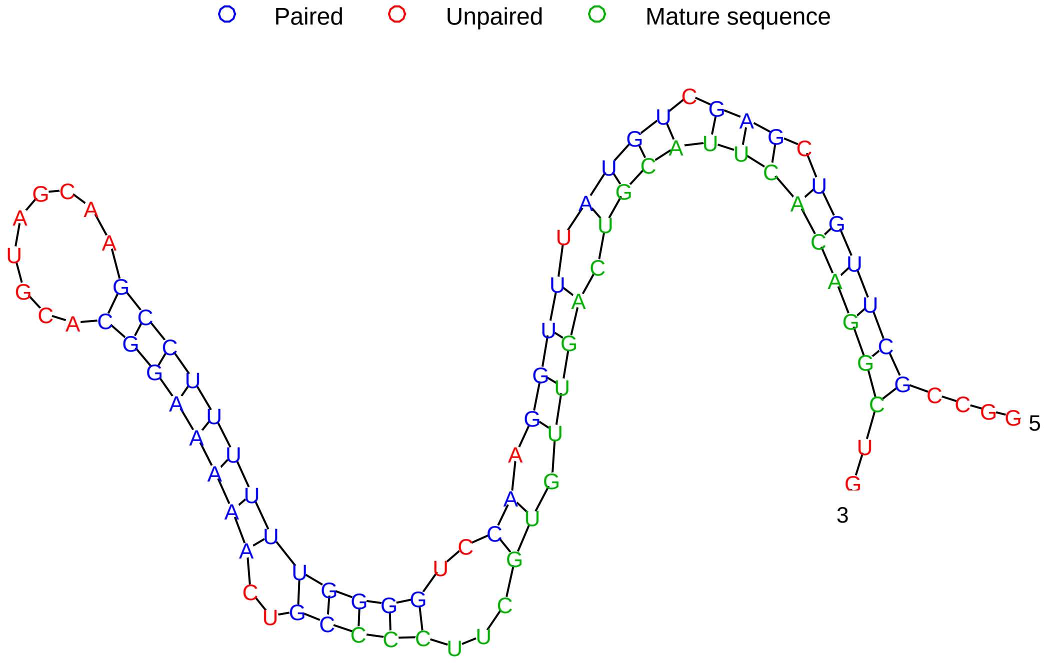

Stem loop (UMD3.1): chr21:19878838-19878929
Mature (UMD3.1): chr21:19878840-19878866
Mature seq len: 27
Total raw counts (9 samples): 1213
Average raw counts: 135
Strand: Reverse
Orientation: 3p
Minimum free energy: -28.90

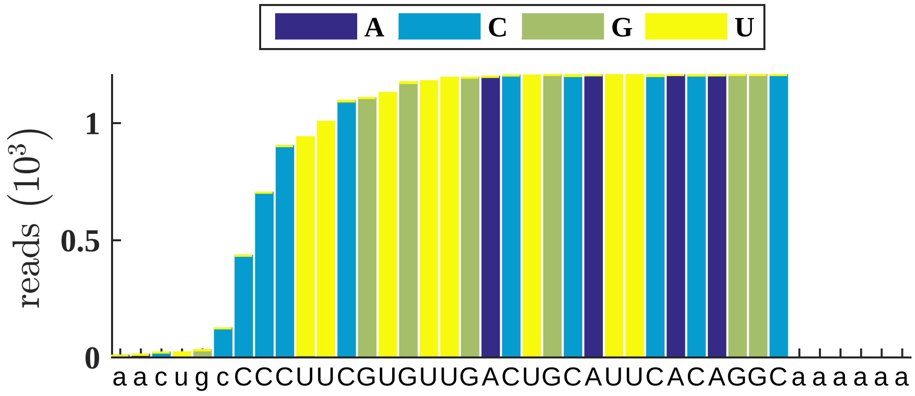

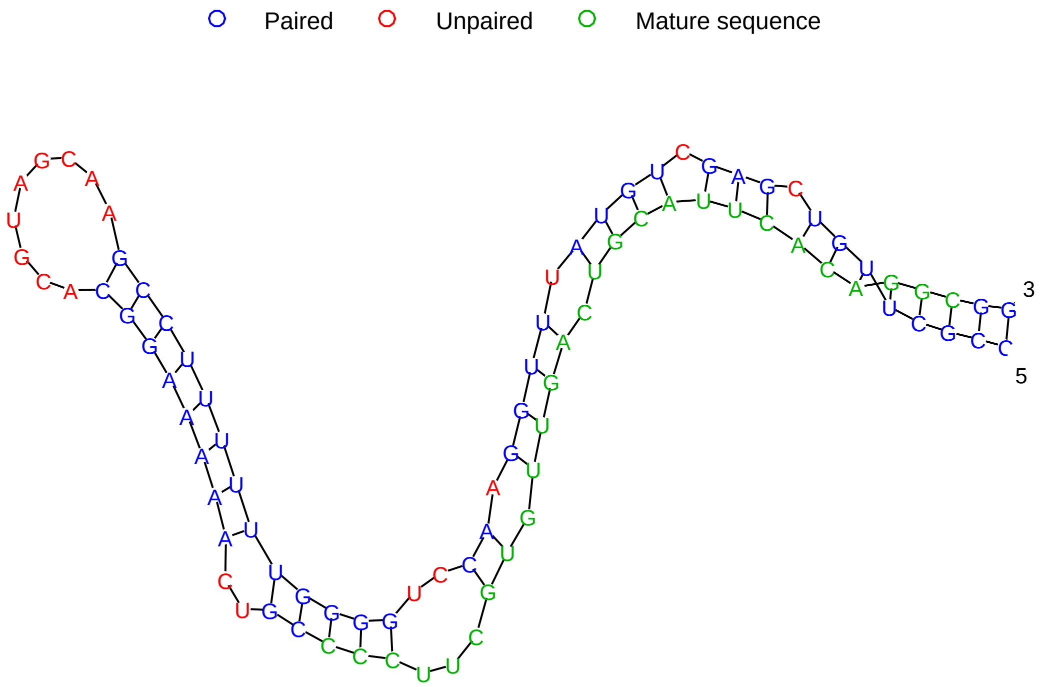

Stem loop (UMD3.1): chr21:19880527-19880616
Mature (UMD3.1): chr21:19880588-19880614
Mature seq len: 27
Total raw counts (9 samples): 1194
Average raw counts: 133
Strand: Forward
Orientation: 3p
Minimum free energy: -33.20

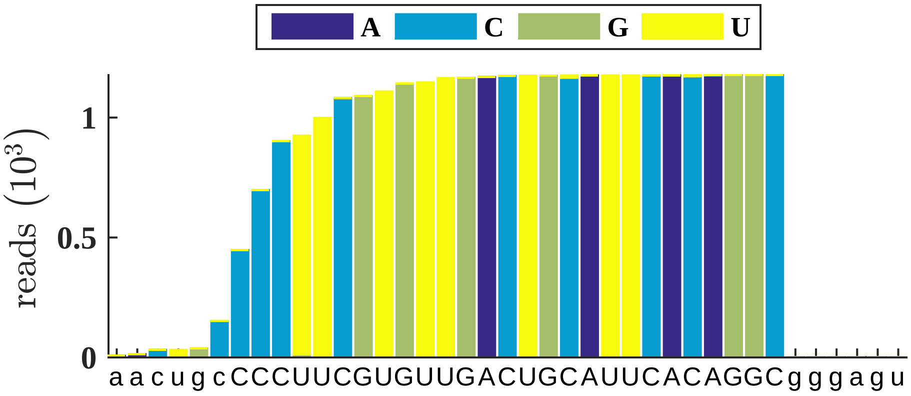

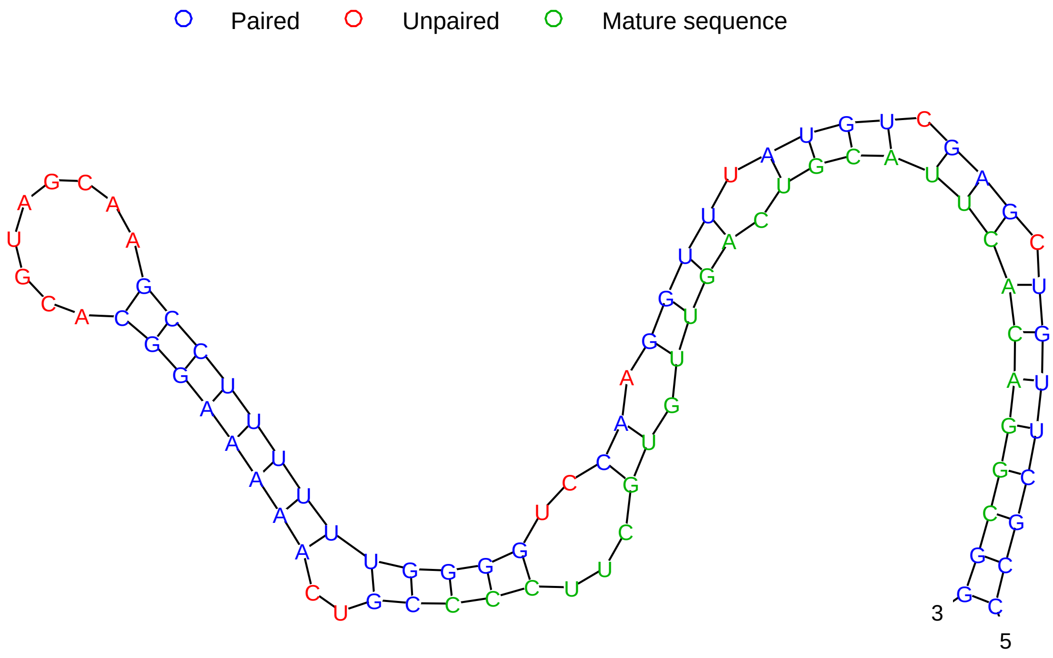

Stem loop (UMD3.1): chr21:19882958-19883047
Mature (UMD3.1): chr21:19883019-19883045
Mature seq len: 27
Total raw counts (9 samples): 1189
Average raw counts: 133
Strand: Forward
Orientation: 3p
Minimum free energy: -33.20

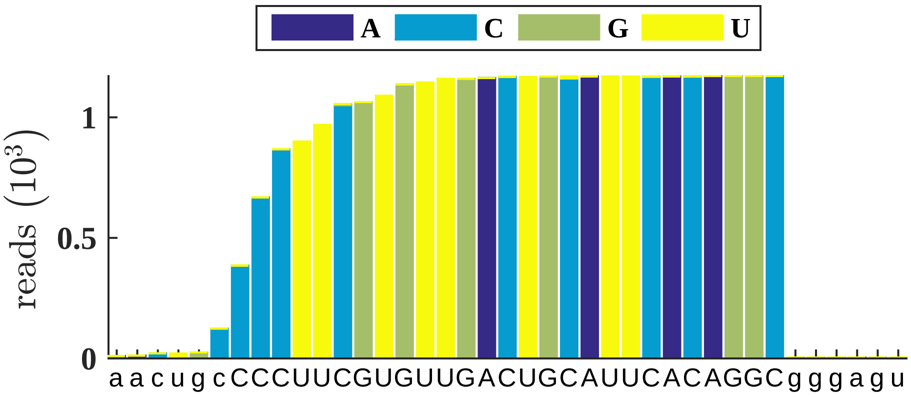

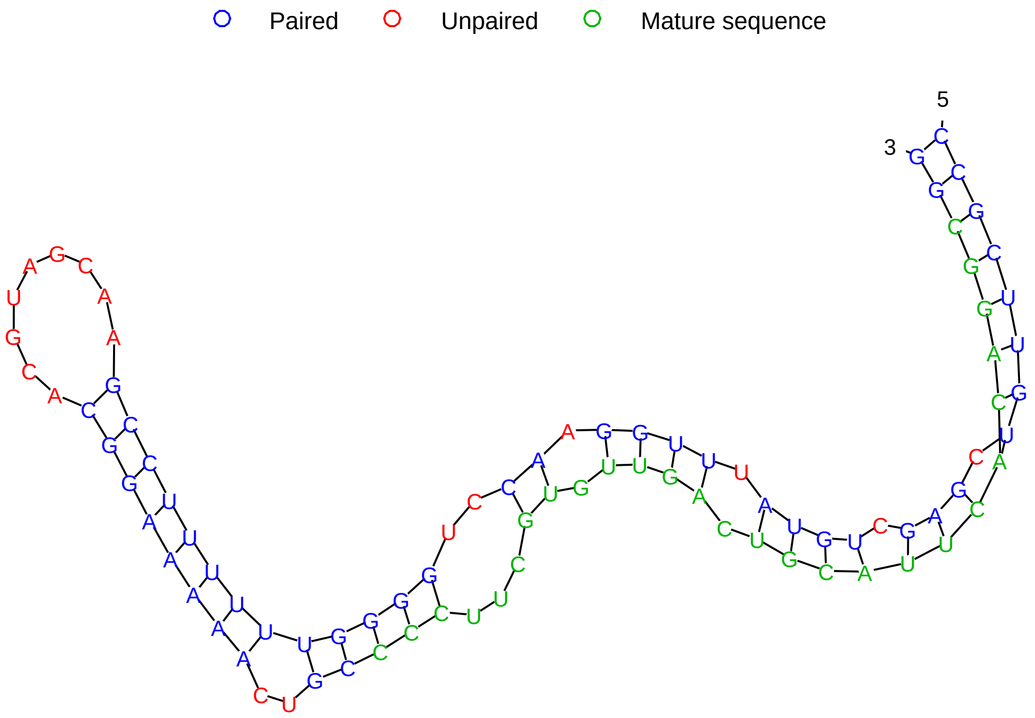

Stem loop (UMD3.1): chr21:19884331-19884420
Mature (UMD3.1): chr21:19884392-19884418
Mature seq len: 27
Total raw counts (9 samples): 1210
Average raw counts: 135
Strand: Forward
Orientation: 3p
Minimum free energy: -33.20

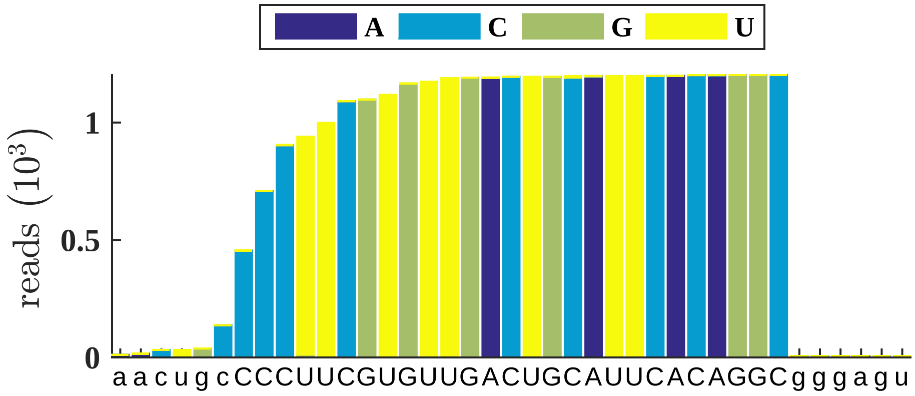

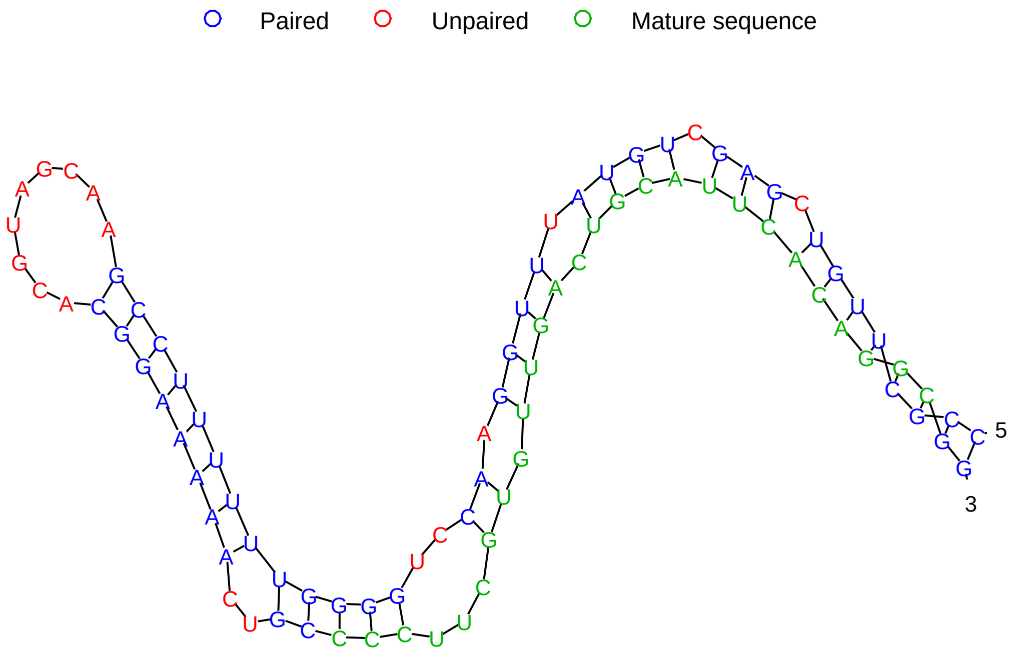

Stem loop (UMD3.1): chr21:19891663-19891752
Mature (UMD3.1): chr21:19891665-19891691
Mature seq len: 27
Total raw counts (9 samples): 1121
Average raw counts: 125
Strand: Reverse
Orientation: 3p
Minimum free energy: -33.20

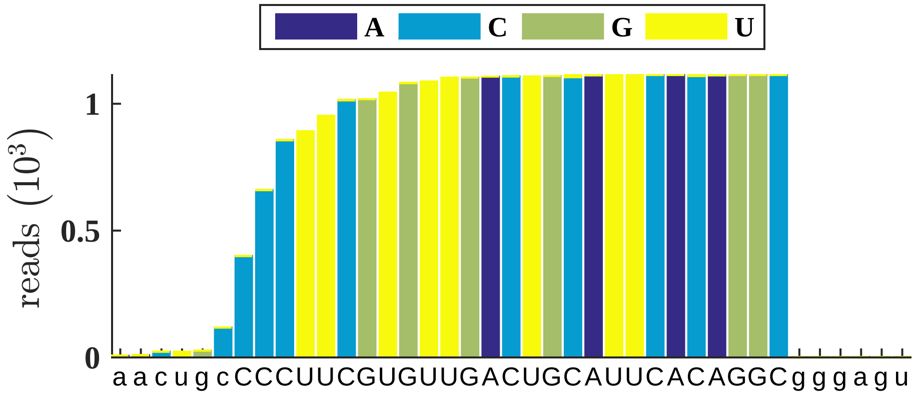

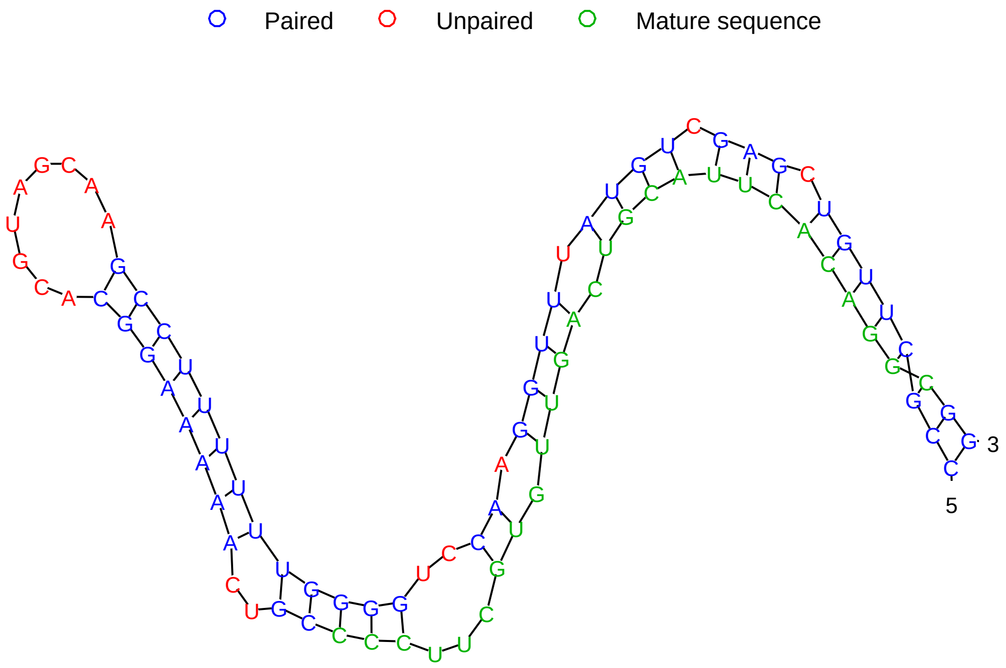

Stem loop (UMD3.1): chr21:19893540-19893629
Mature (UMD3.1): chr21:19893601-19893627
Mature seq len: 27
Total raw counts (9 samples): 1212
Average raw counts: 135
Strand: Forward
Orientation: 3p
Minimum free energy: -33.20

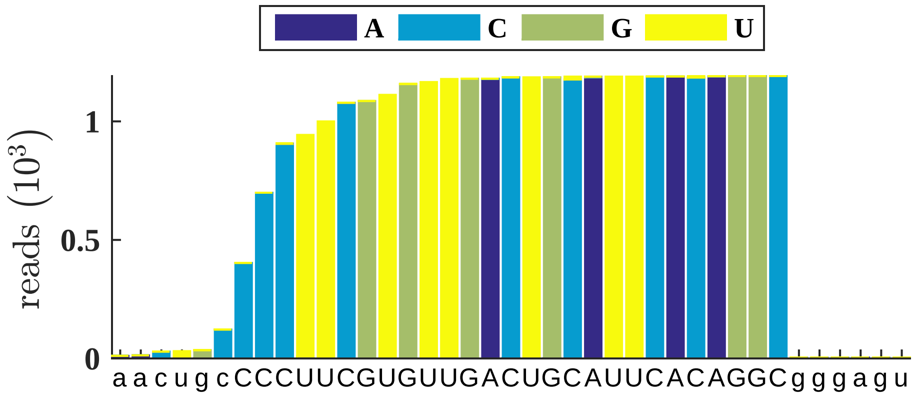

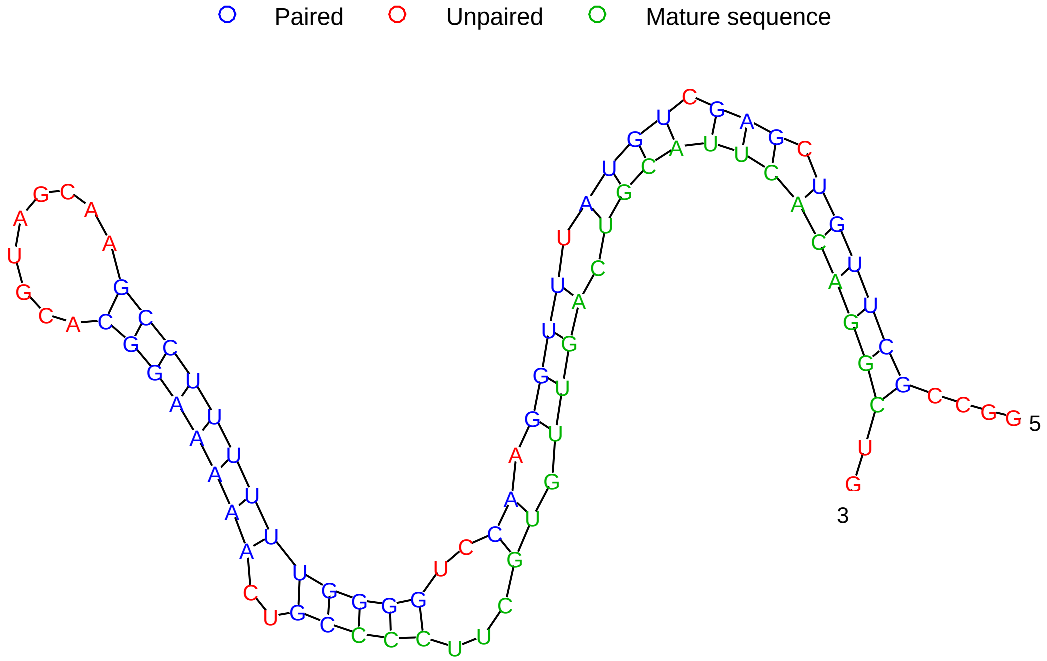

Stem loop (UMD3.1): chr21:24697225-24697316
Mature (UMD3.1): chr21:24697227-24697253
Mature seq len: 27
Total raw counts (9 samples): 1179
Average raw counts: 131
Strand: Reverse
Orientation: 3p
Minimum free energy: -28.90

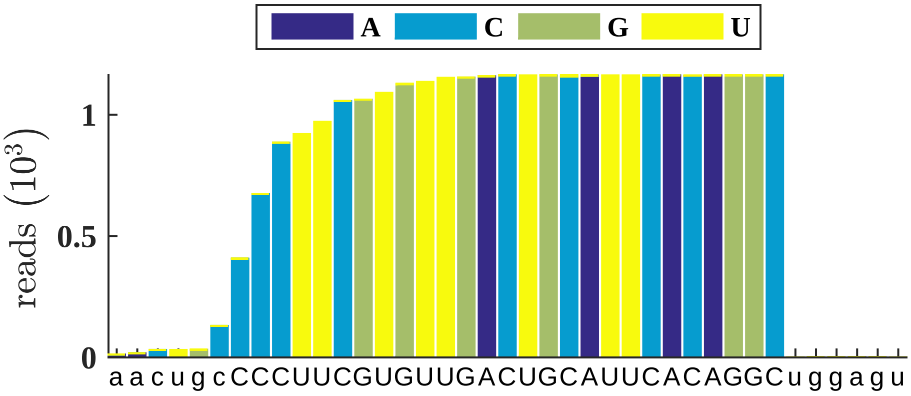

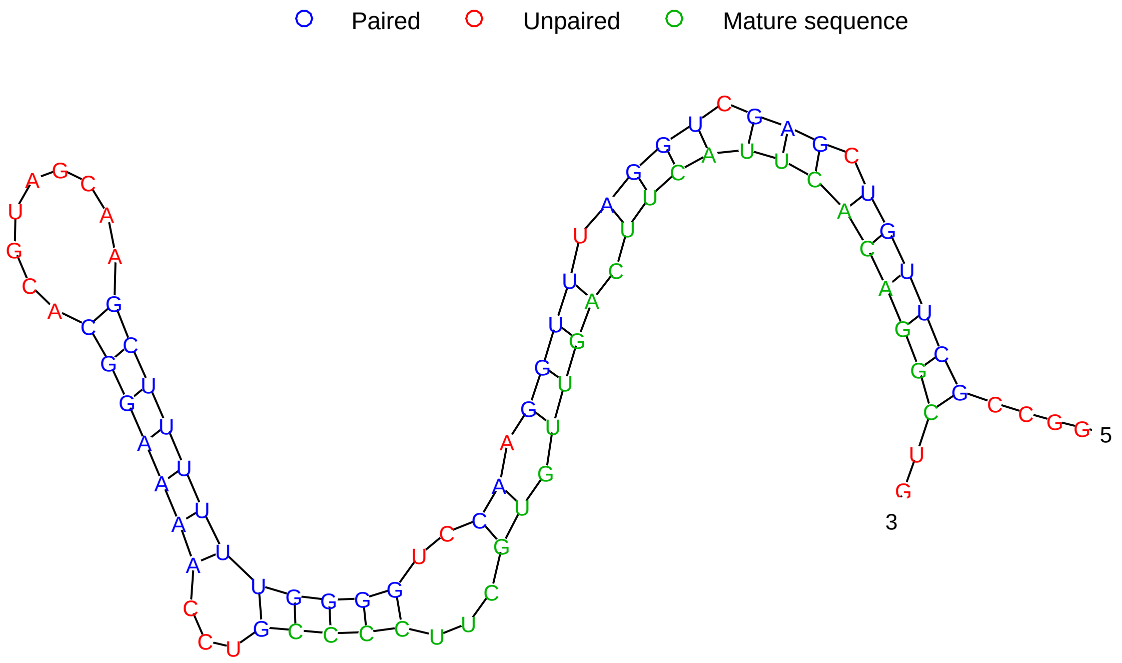

Stem loop (UMD3.1): chr21:26066966-26067056
Mature (UMD3.1): chr21:26067027-26067054
Mature seq len: 28
Total raw counts (9 samples): 1069
Average raw counts: 119
Strand: Forward
Orientation: 3p
Minimum free energy: -24.00

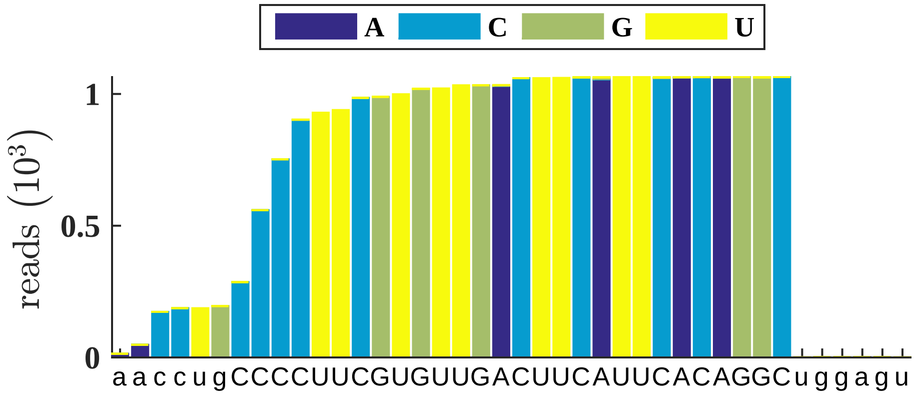

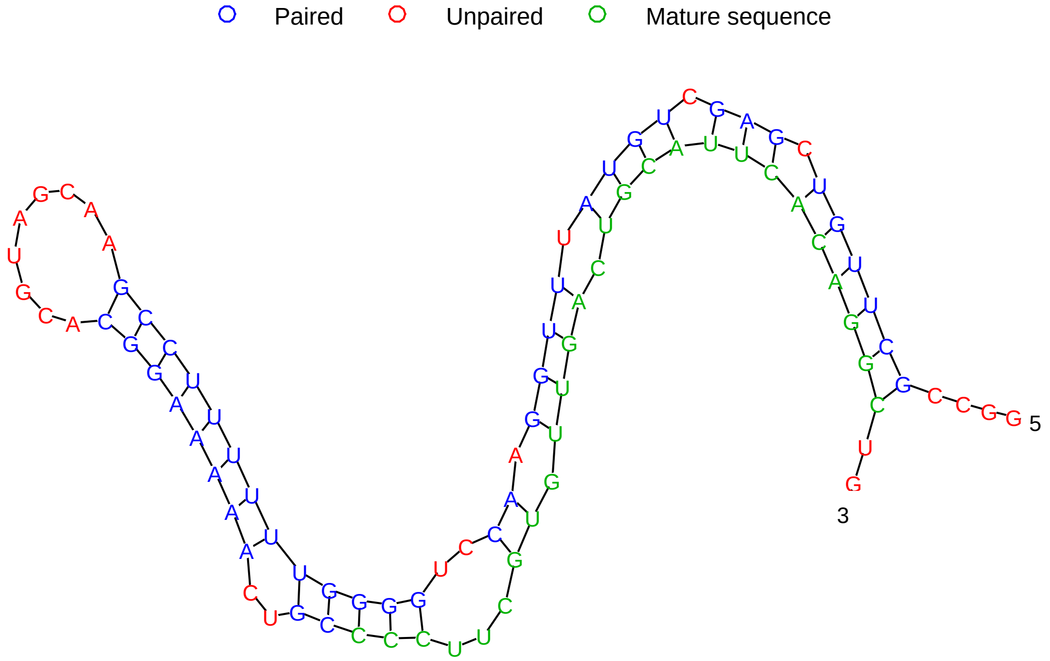

Stem loop (UMD3.1): chr21:35619545-35619636
Mature (UMD3.1): chr21:35619608-35619634
Mature seq len: 27
Total raw counts (9 samples): 1201
Average raw counts: 134
Strand: Forward
Orientation: 3p
Minimum free energy: -28.90

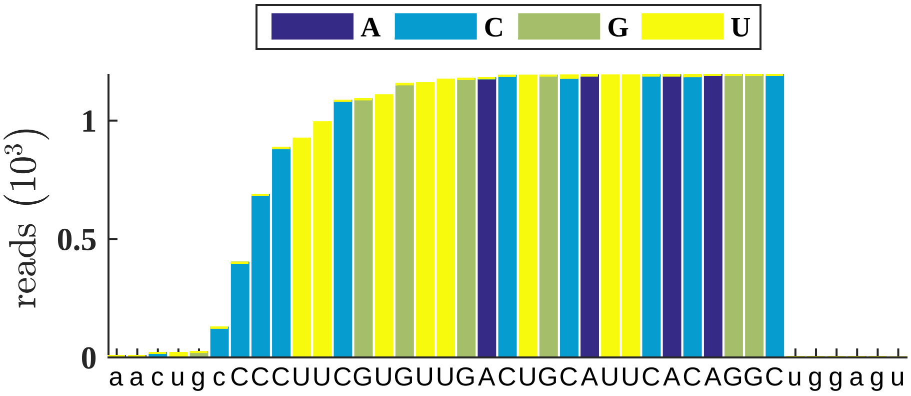

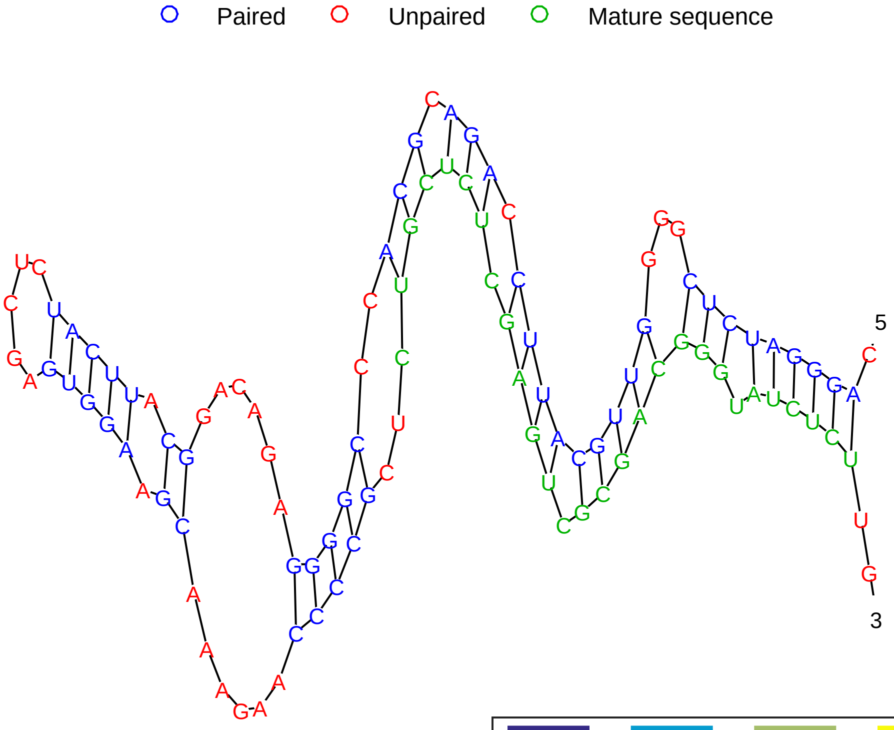

Stem loop (UMD3.1): chr21:35620224-35620330
Mature (UMD3.1): chr21:35620301-35620328
Mature seq len: 28
Total raw counts (9 samples): 12345
Average raw counts: 1372
Strand: Forward
Orientation: 3p
Minimum free energy: -36.50

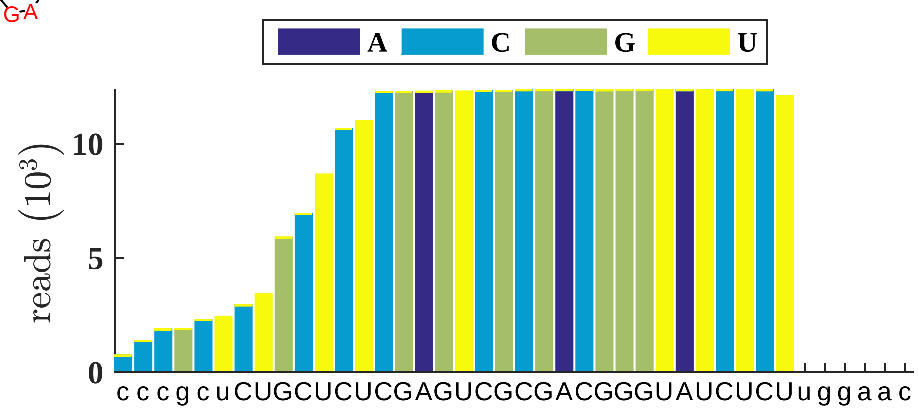

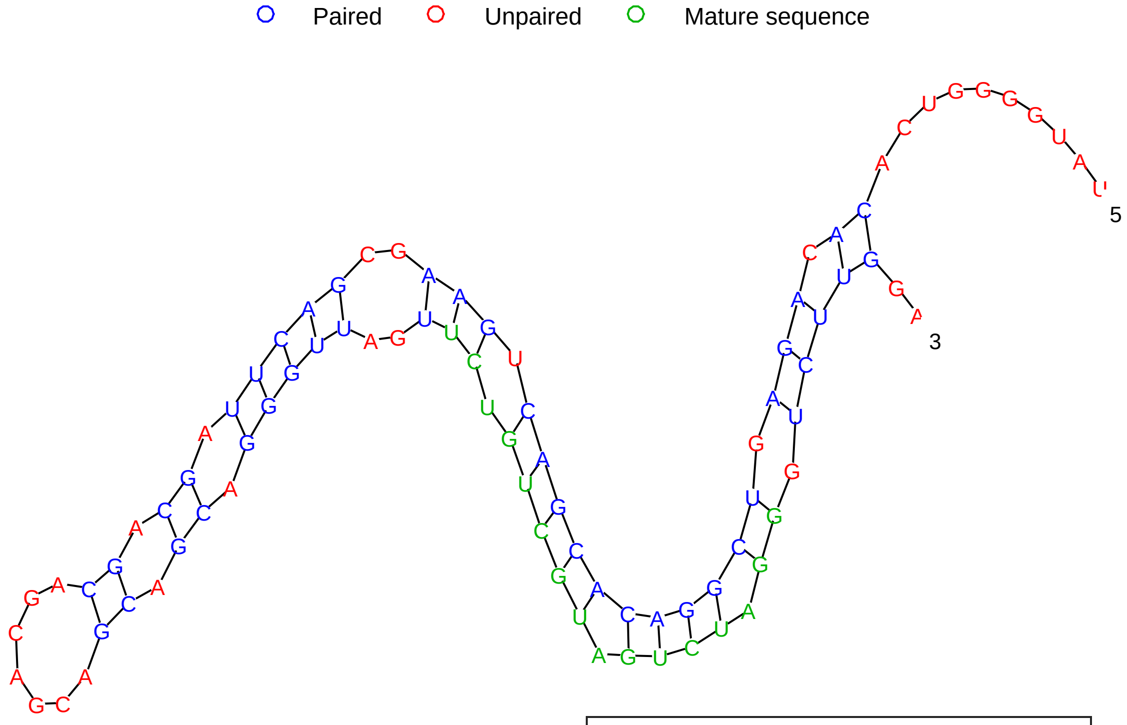

Stem loop (UMD3.1): chr21:42502479-42502568
Mature (UMD3.1): chr21:42502545-42502560
Mature seq len: 16
Total raw counts (9 samples): 644
Average raw counts: 72
Strand: Forward
Orientation: 3p
Minimum free energy: -26.50

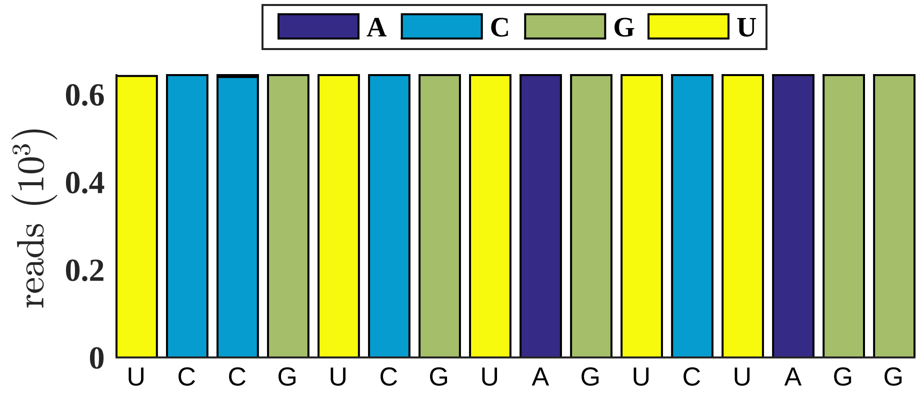

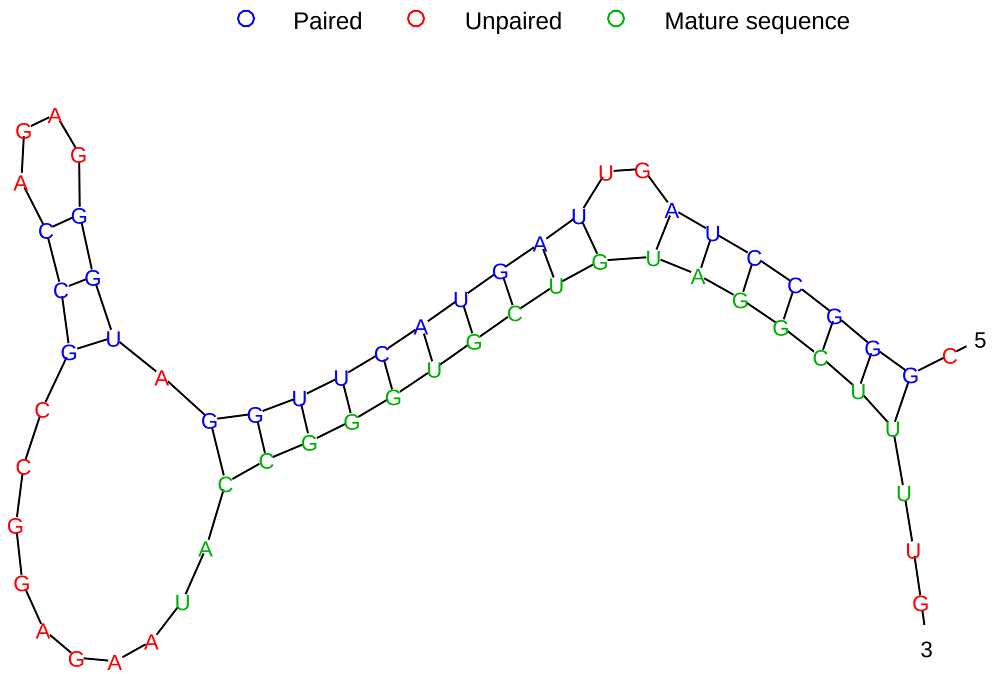

Stem loop (UMD3.1): chr21:60864915-60864975
Mature (UMD3.1): chr21:60864954-60864973
Mature seq len: 20
Total raw counts (9 samples): 51732
Average raw counts: 5748
Strand: Forward
Orientation: 3p
Minimum free energy: -20.40

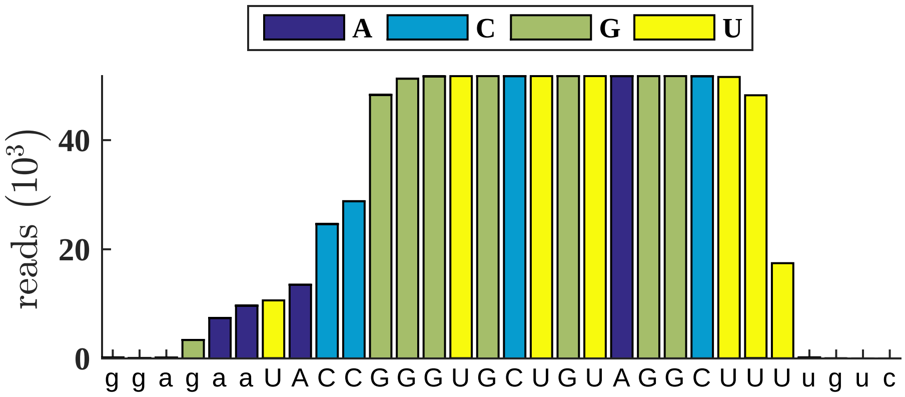

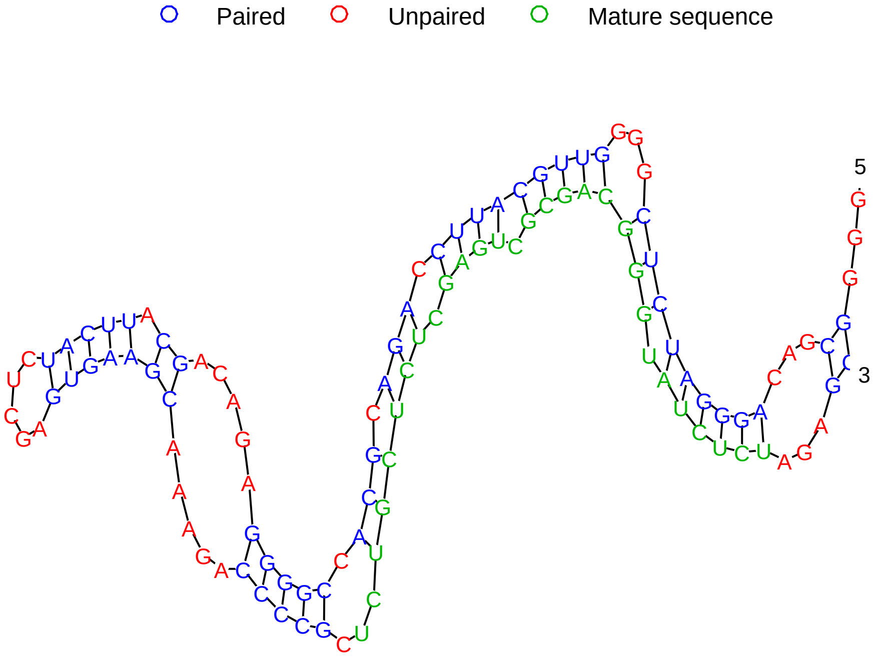

Stem loop (UMD3.1): chr22:2882953-2883065
Mature (UMD3.1): chr22:2883032-2883060
Mature seq len: 29
Total raw counts (9 samples): 12175
Average raw counts: 1353
Strand: Forward
Orientation: 3p
Minimum free energy: -37.80

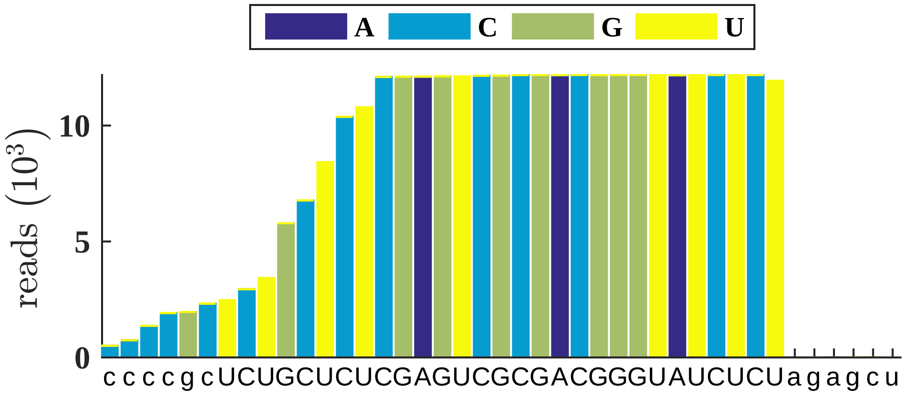

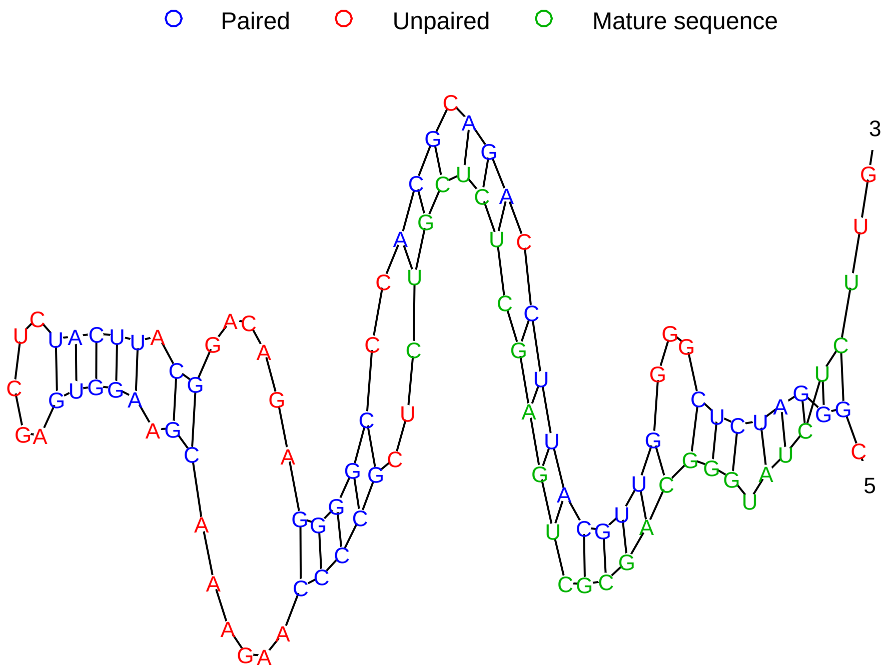

Stem loop (UMD3.1): chr22:2889752-2889857
Mature (UMD3.1): chr22:2889828-2889855
Mature seq len: 28
Total raw counts (9 samples): 14065
Average raw counts: 1563
Strand: Forward
Orientation: 3p
Minimum free energy: -35.70

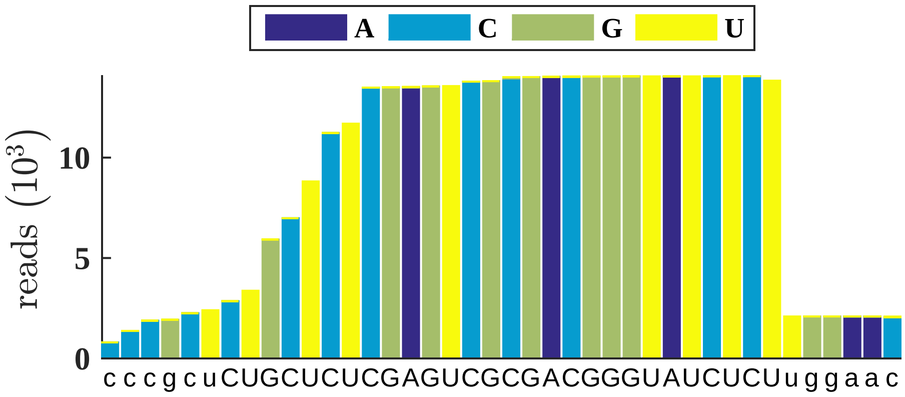

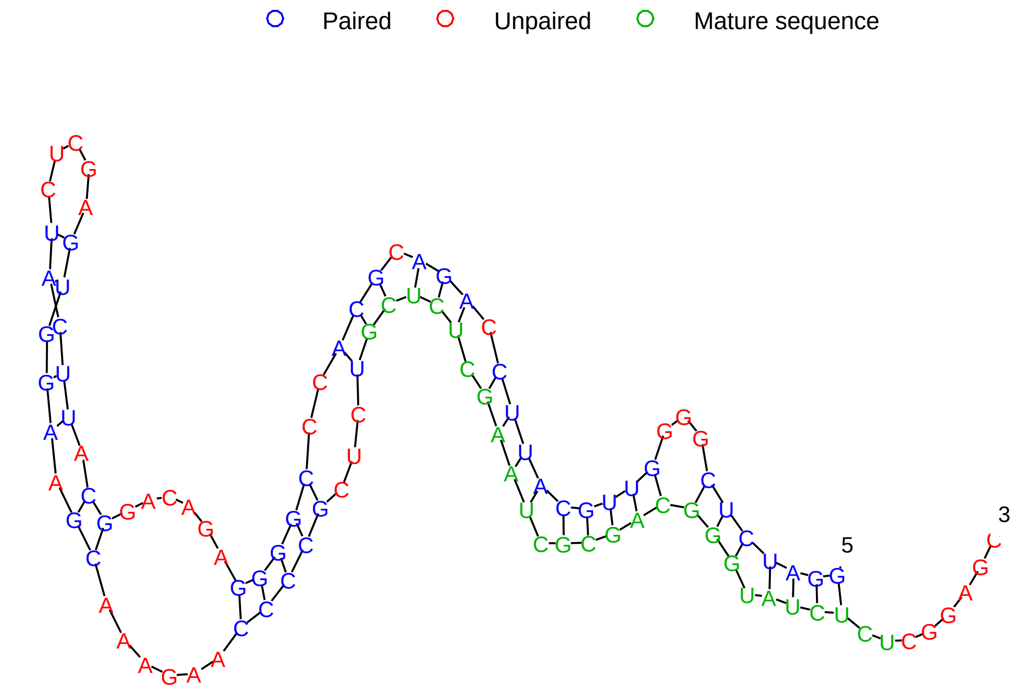

Stem loop (UMD3.1): chr22:37927416-37927523
Mature (UMD3.1): chr22:37927492-37927517
Mature seq len: 26
Total raw counts (9 samples): 3952
Average raw counts: 440
Strand: Forward
Orientation: 3p
Minimum free energy: -32.40

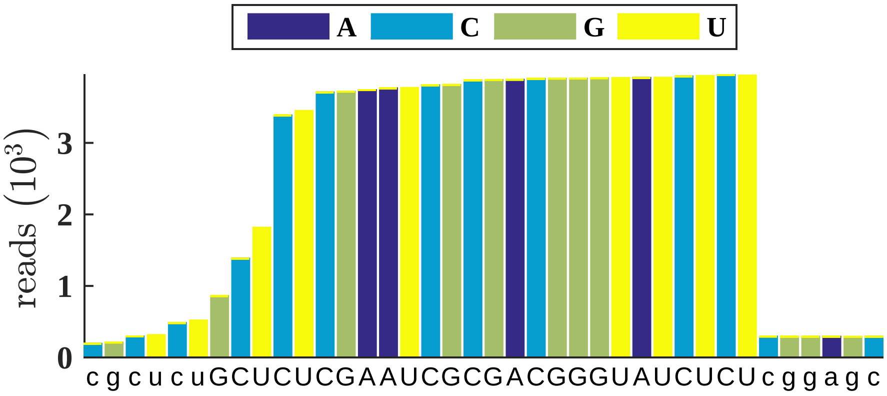

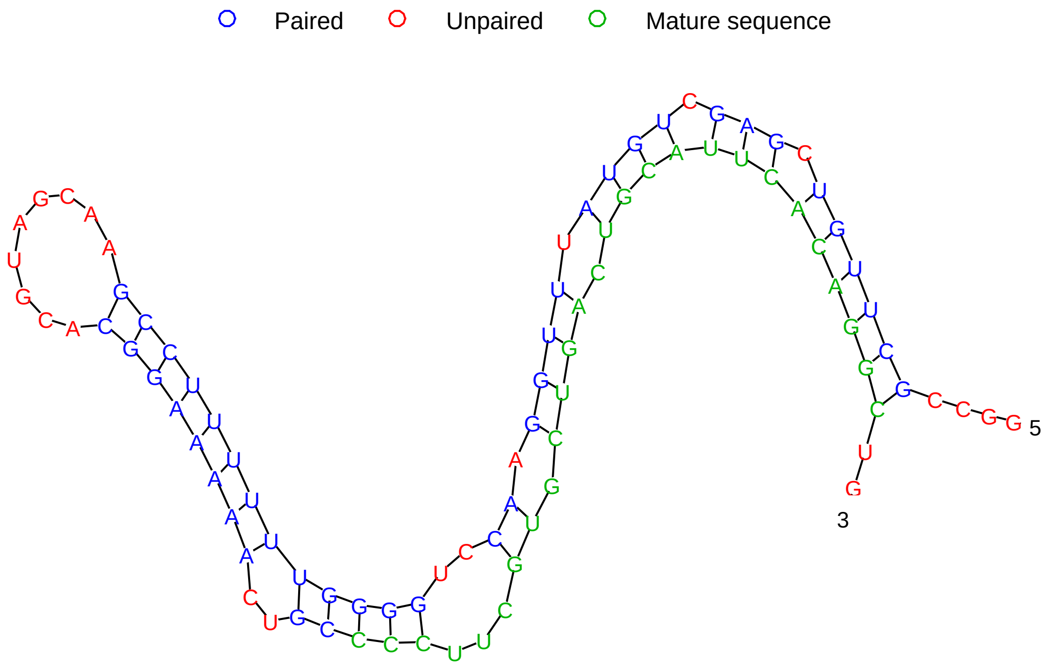

Stem loop (UMD3.1): chr22:37936194-37936285
Mature (UMD3.1): chr22:37936196-37936222
Mature seq len: 27
Total raw counts (9 samples): 1251
Average raw counts: 139
Strand: Reverse
Orientation: 3p
Minimum free energy: -31.20

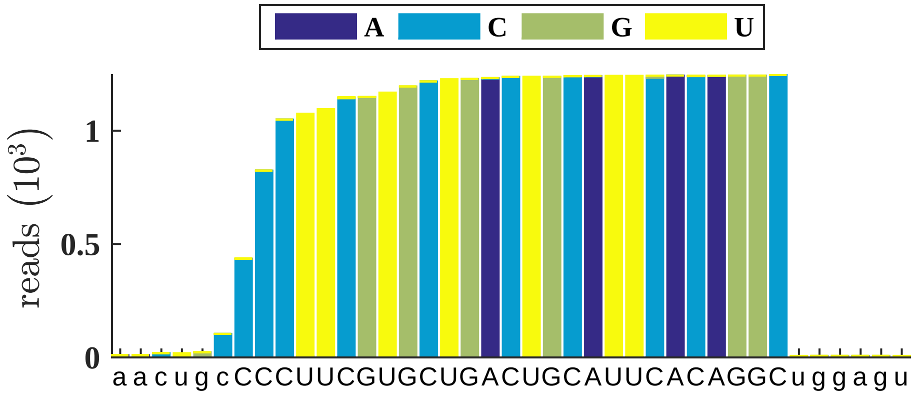

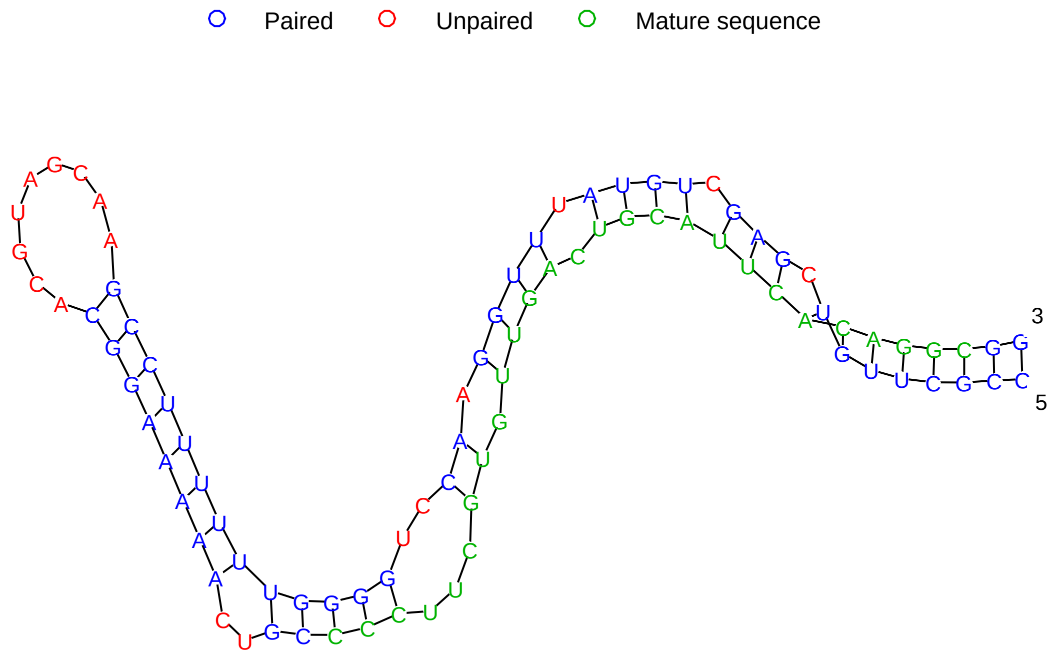

Stem loop (UMD3.1): chr22:53667584-53667673
Mature (UMD3.1): chr22:53667586-53667612
Mature seq len: 27
Total raw counts (9 samples): 1179
Average raw counts: 131
Strand: Reverse
Orientation: 3p
Minimum free energy: -33.20

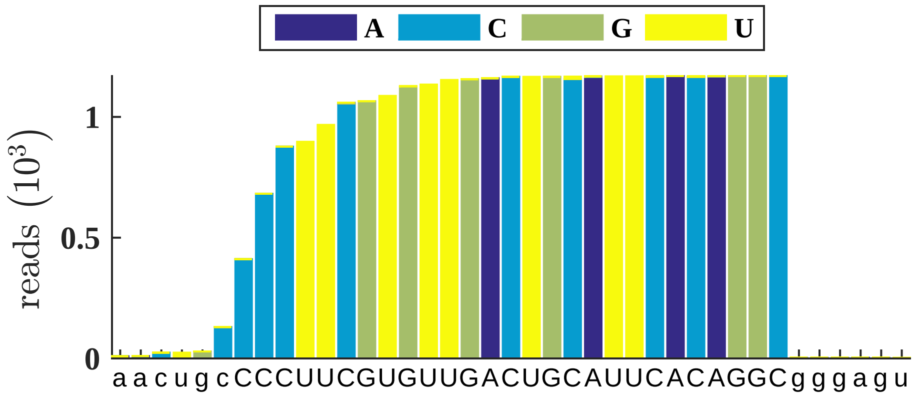

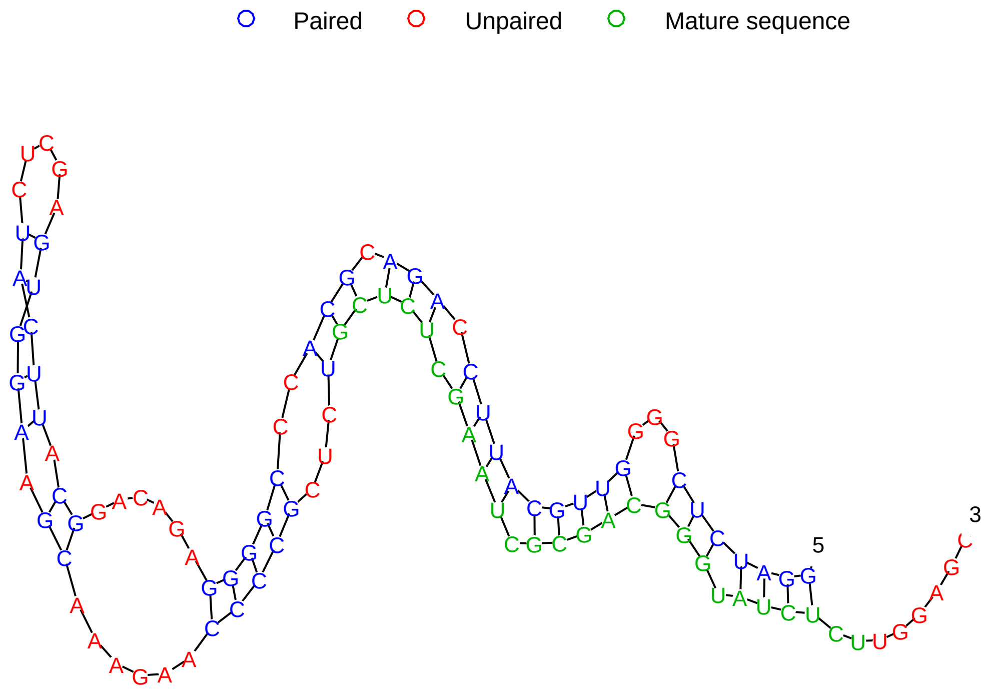

Stem loop (UMD3.1): chr22:53669639-53669746
Mature (UMD3.1): chr22:53669645-53669670
Mature seq len: 26
Total raw counts (9 samples): 4114
Average raw counts: 458
Strand: Reverse
Orientation: 3p
Minimum free energy: -32.40

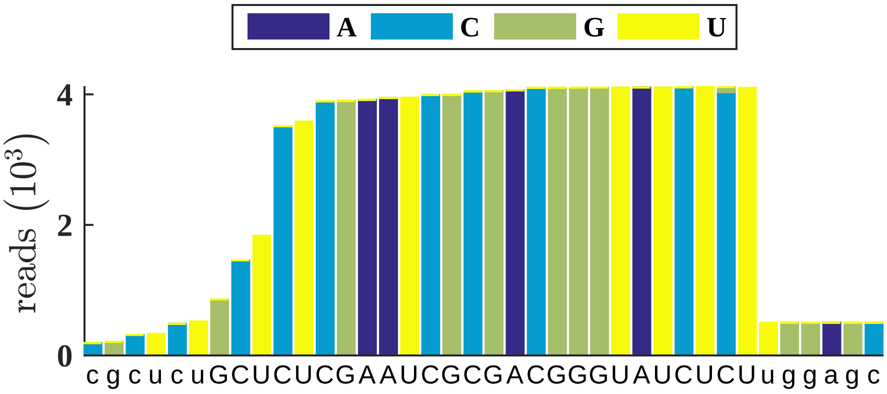

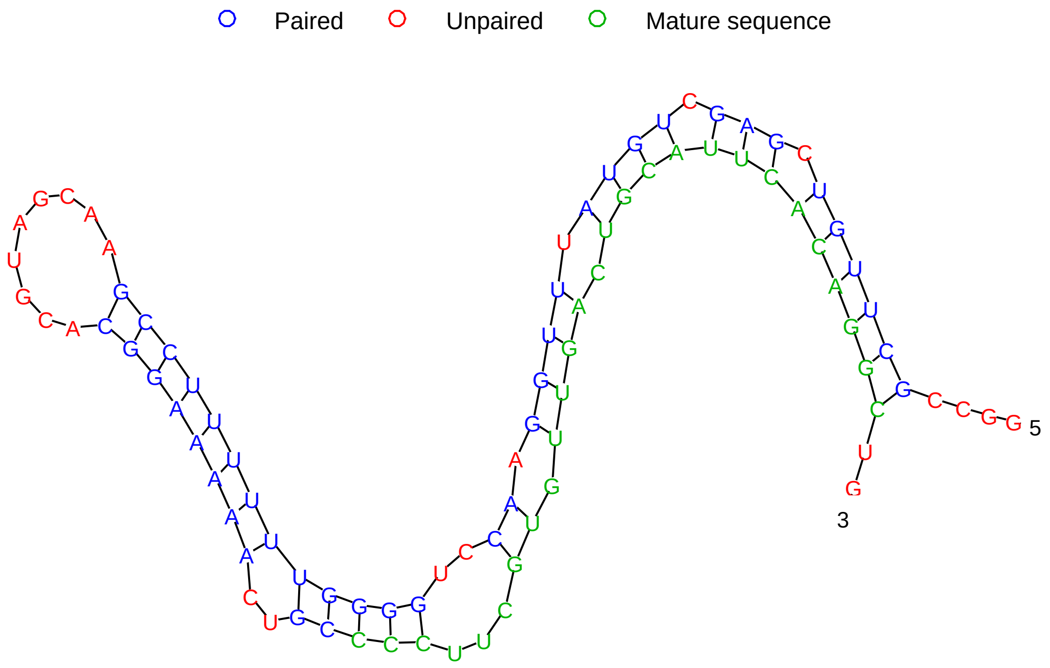

Stem loop (UMD3.1): chr22:53682700-53682791
Mature (UMD3.1): chr22:53682702-53682728
Mature seq len: 27
Total raw counts (9 samples): 1173
Average raw counts: 131
Strand: Reverse
Orientation: 3p
Minimum free energy: -28.90

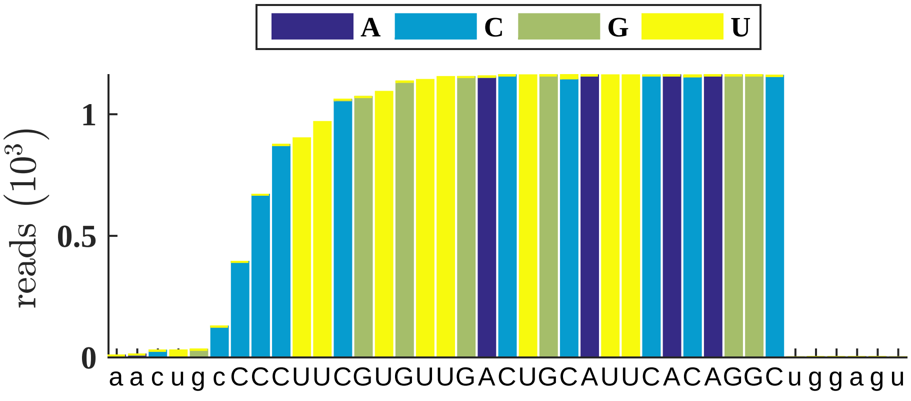

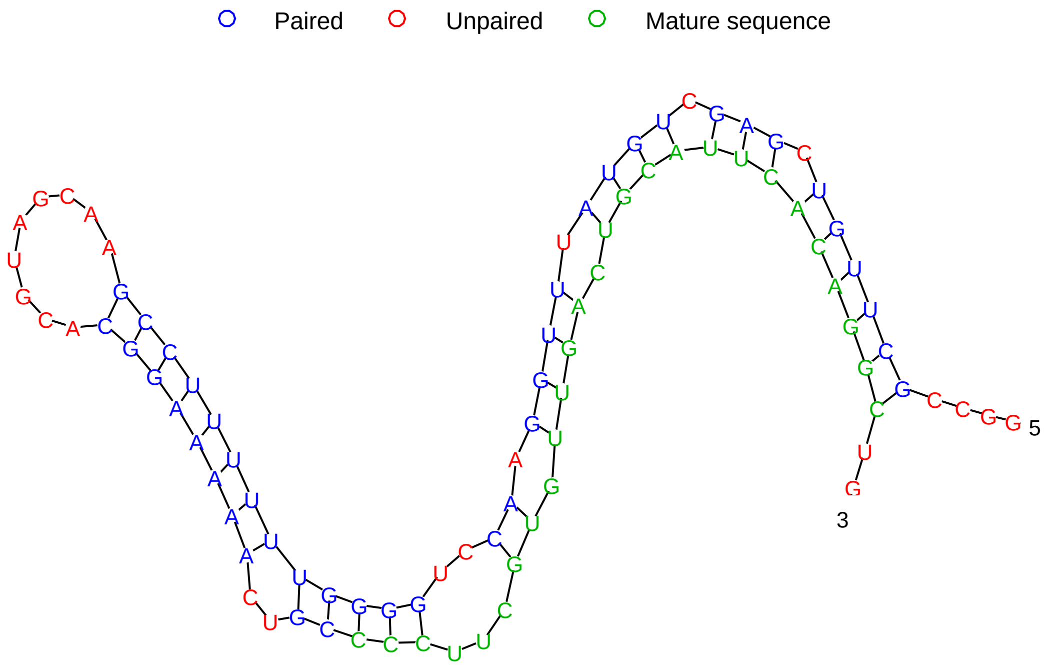

Stem loop (UMD3.1): chr24:35457132-35457223
Mature (UMD3.1): chr24:35457134-35457160
Mature seq len: 27
Total raw counts (9 samples): 1167
Average raw counts: 130
Strand: Reverse
Orientation: 3p
Minimum free energy: -28.90

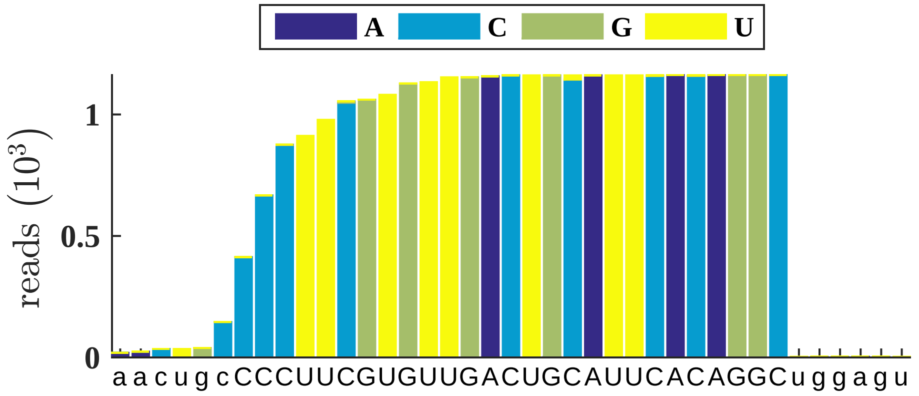

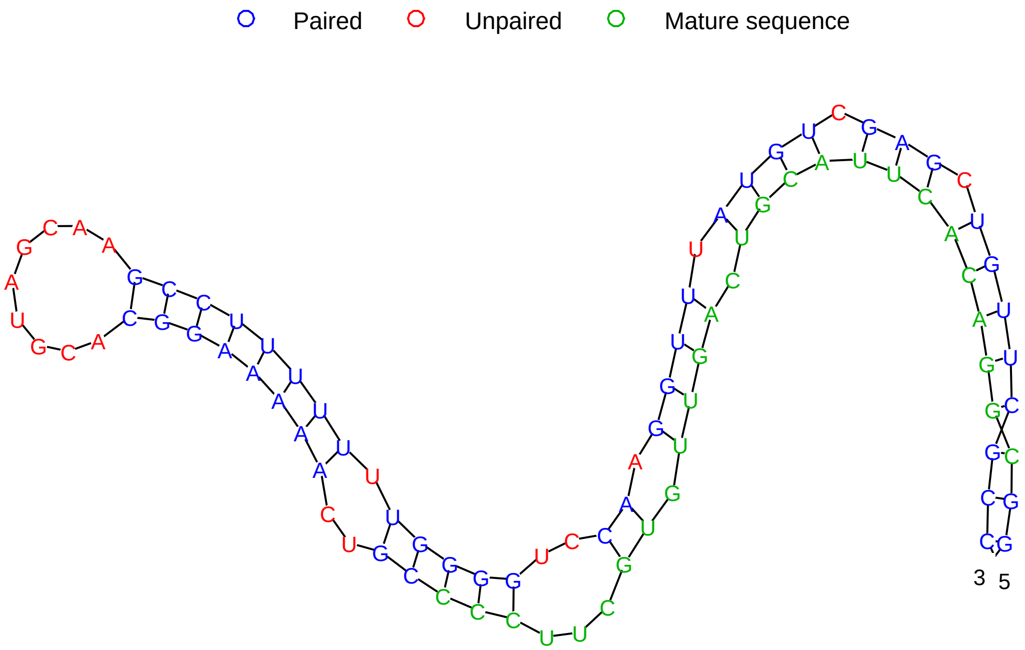

Stem loop (UMD3.1): chr25:18885279-18885369
Mature (UMD3.1): chr25:18885281-18885307
Mature seq len: 27
Total raw counts (9 samples): 1214
Average raw counts: 135
Strand: Reverse
Orientation: 3p
Minimum free energy: -34.00

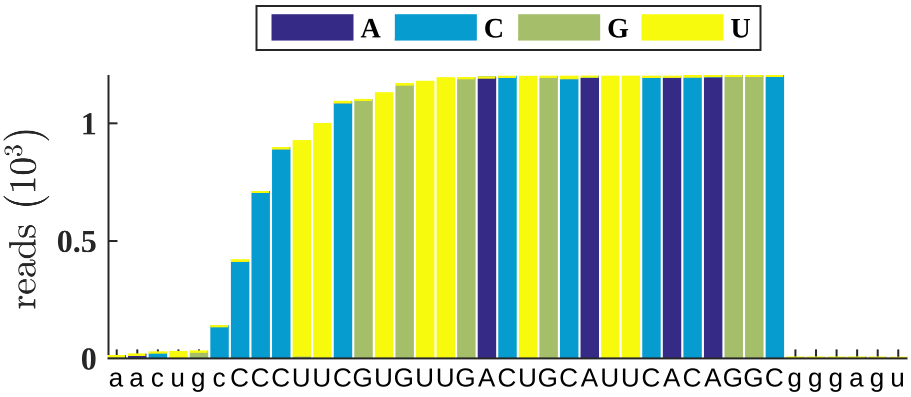

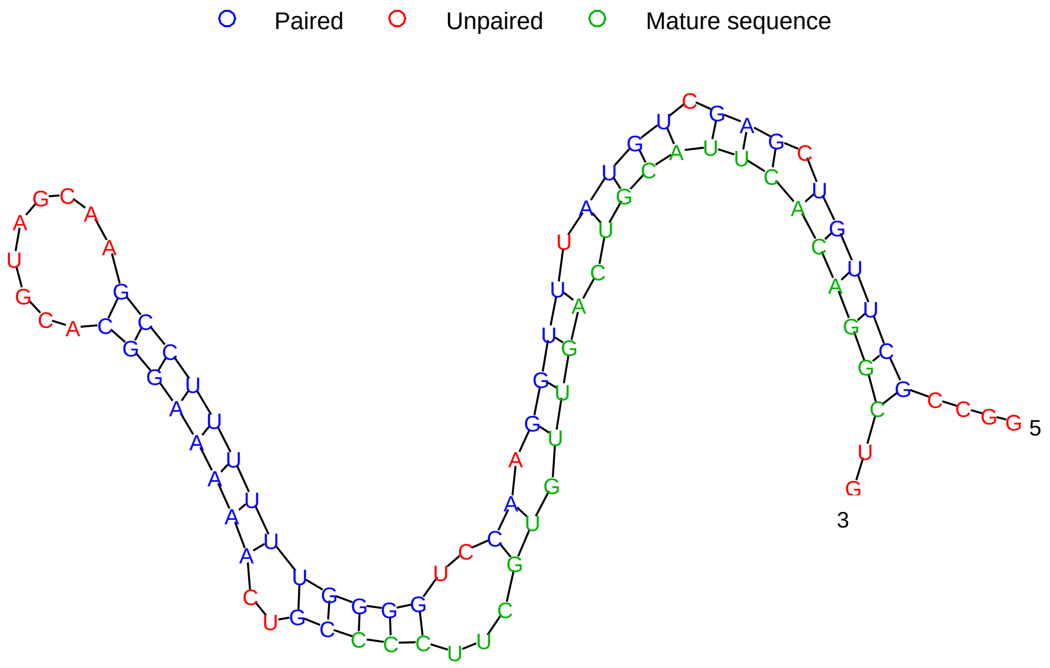

Stem loop (UMD3.1): chr25:18887661-18887752
Mature (UMD3.1): chr25:18887663-18887689
Mature seq len: 27
Total raw counts (9 samples): 1219
Average raw counts: 136
Strand: Reverse
Orientation: 3p
Minimum free energy: -28.90

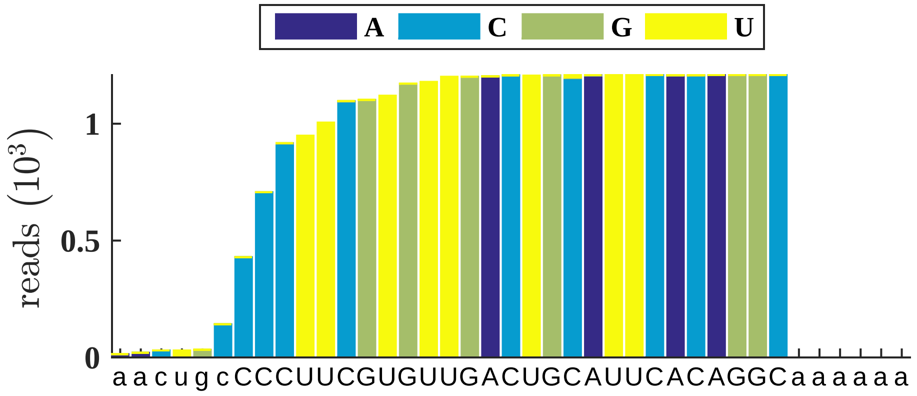

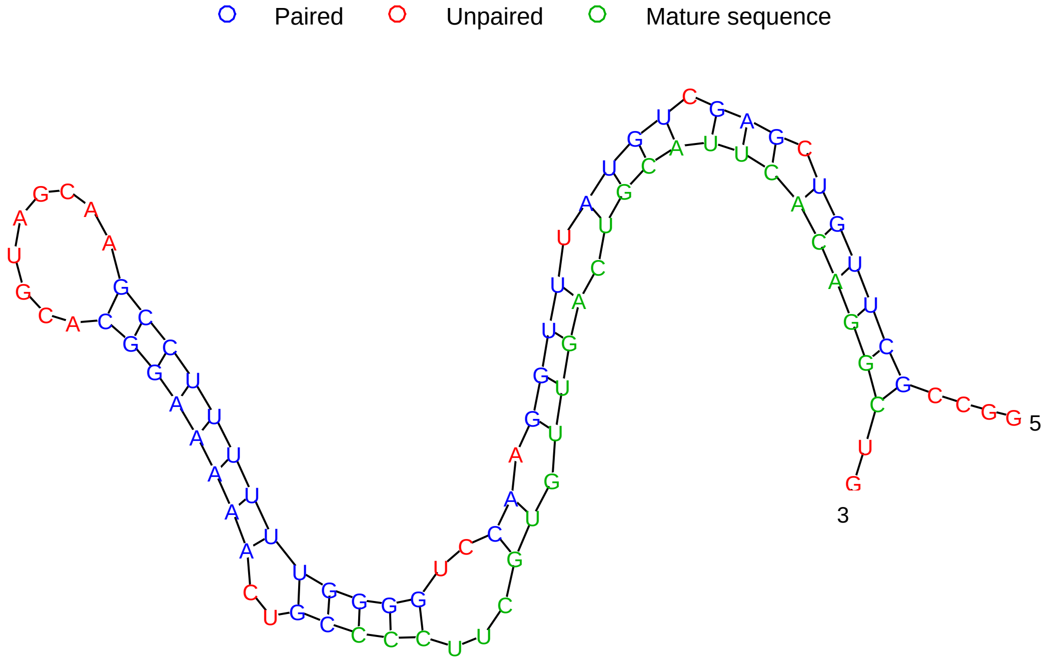

Stem loop (UMD3.1): chr25:18889042-18889133
Mature (UMD3.1): chr25:18889044-18889070
Mature seq len: 27
Total raw counts (9 samples): 1219
Average raw counts: 136
Strand: Reverse
Orientation: 3p
Minimum free energy: -28.90

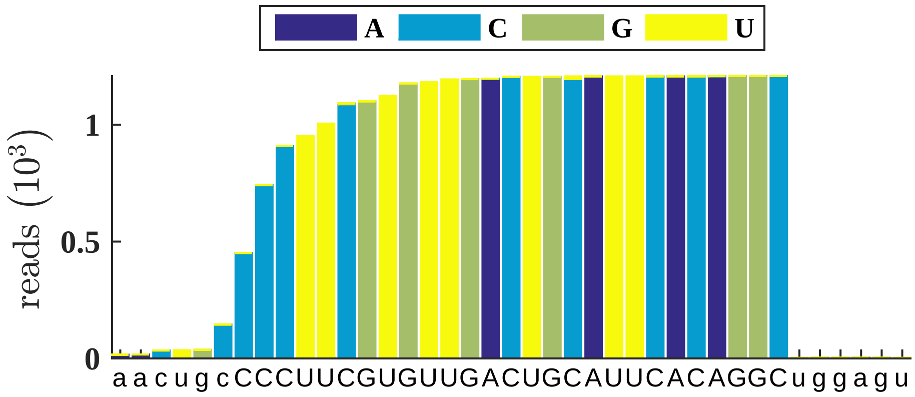

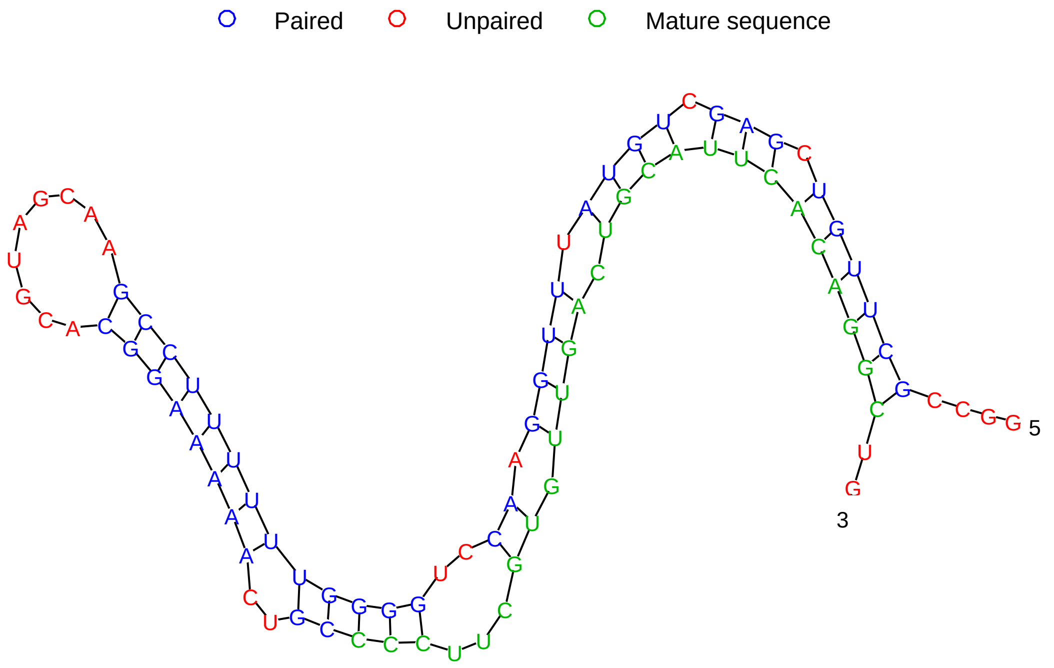

Stem loop (UMD3.1): chr25:18891356-18891447
Mature (UMD3.1): chr25:18891358-18891384
Mature seq len: 27
Total raw counts (9 samples): 1288
Average raw counts: 144
Strand: Reverse
Orientation: 3p
Minimum free energy: -28.90

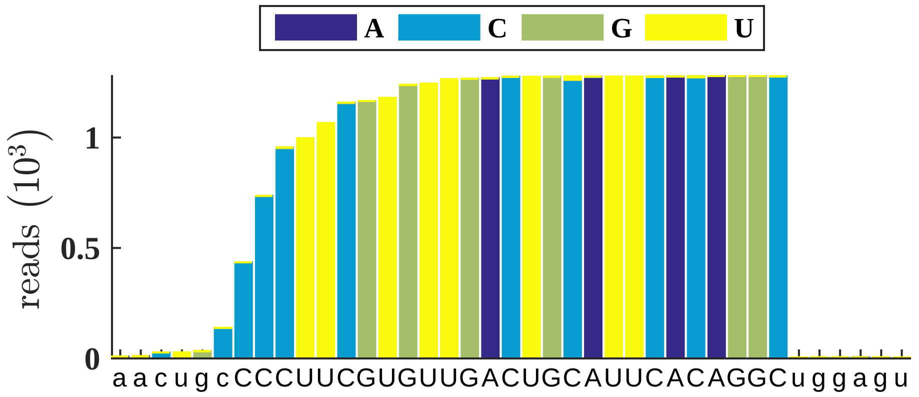

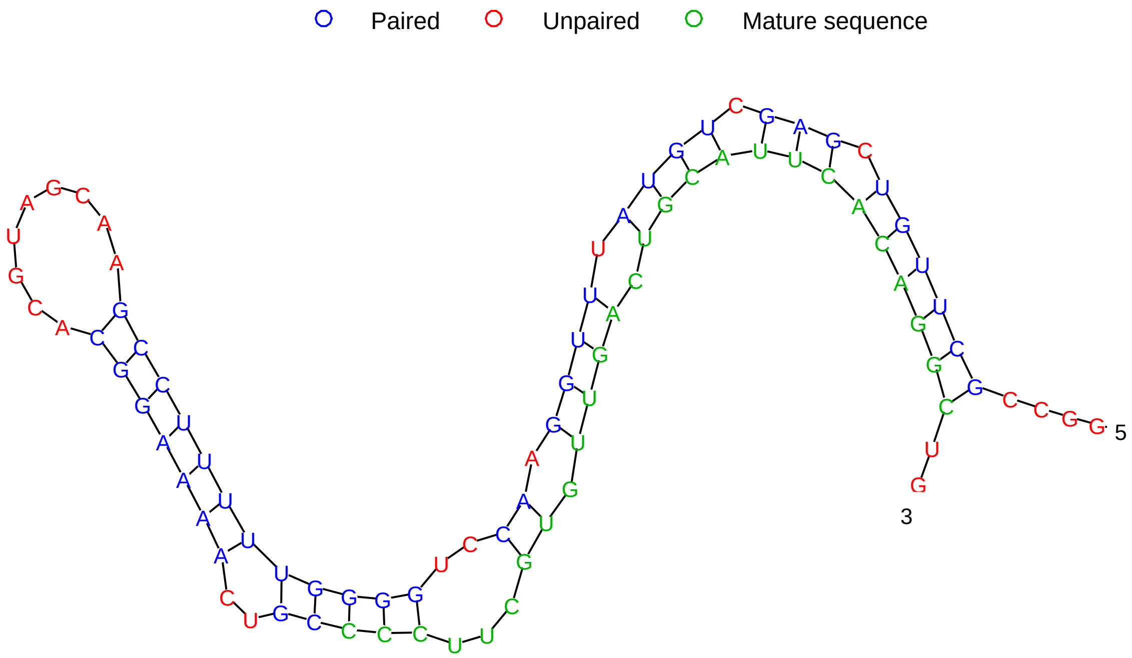

Stem loop (UMD3.1): chr25:18899183-18899272
Mature (UMD3.1): chr25:18899185-18899211
Mature seq len: 27
Total raw counts (9 samples): 1212
Average raw counts: 135
Strand: Reverse
Orientation: 3p
Minimum free energy: -28.00

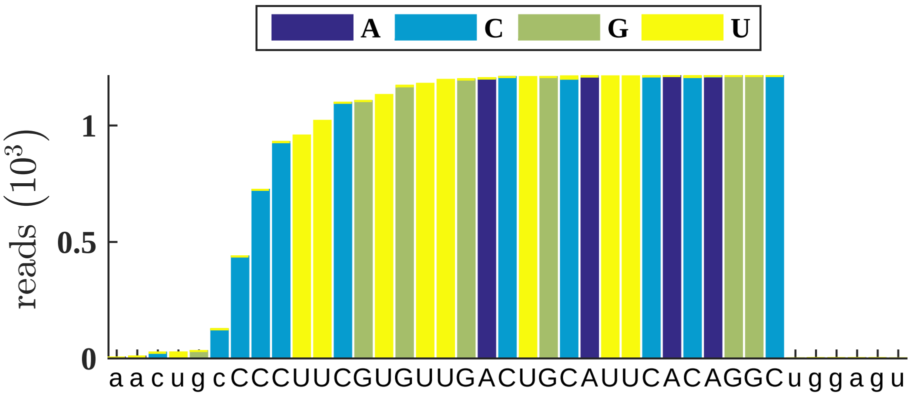

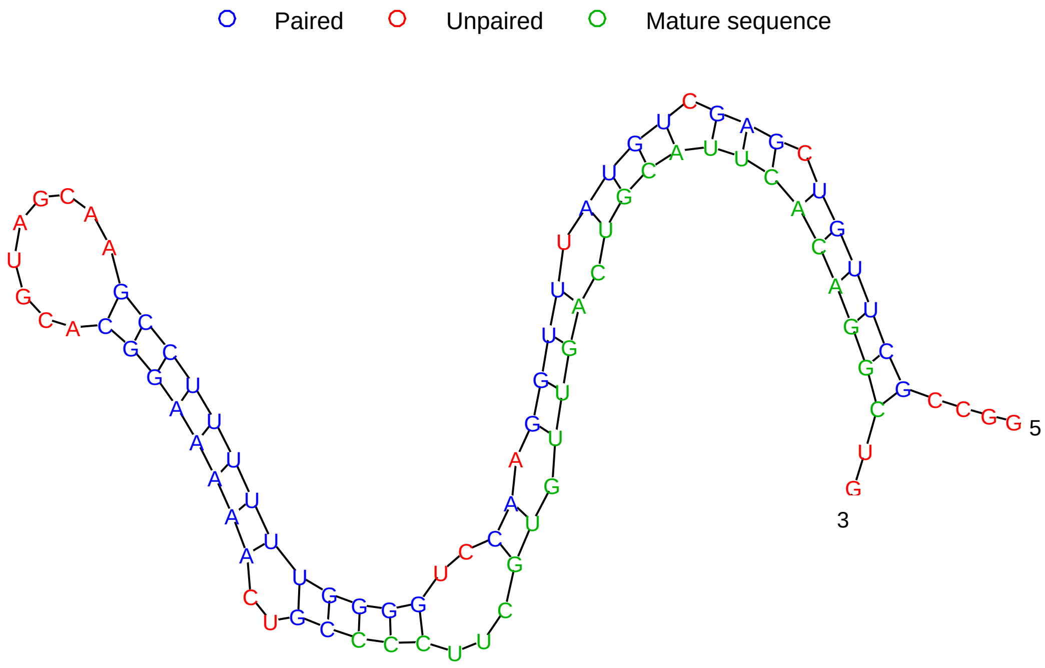

Stem loop (UMD3.1): chr25:18912241-18912332
Mature (UMD3.1): chr25:18912243-18912269
Mature seq len: 27
Total raw counts (9 samples): 1199
Average raw counts: 134
Strand: Reverse
Orientation: 3p
Minimum free energy: -28.90

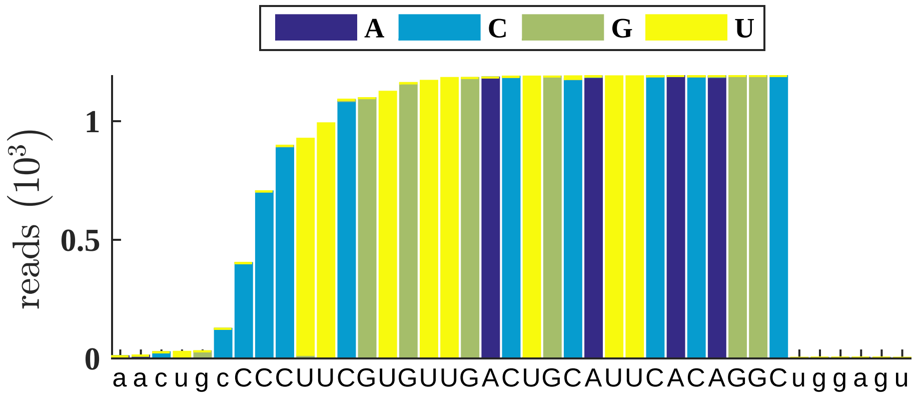

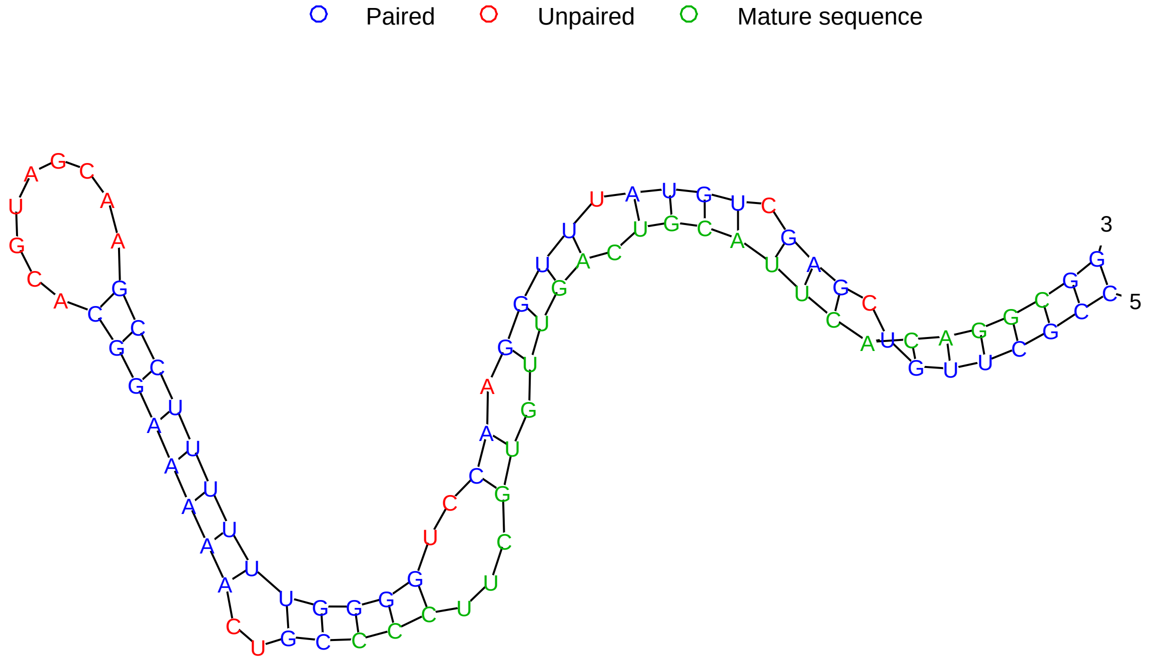

Stem loop (UMD3.1): chr25:18913641-18913730
Mature (UMD3.1): chr25:18913643-18913669
Mature seq len: 27
Total raw counts (9 samples): 1204
Average raw counts: 134
Strand: Reverse
Orientation: 3p
Minimum free energy: -33.20

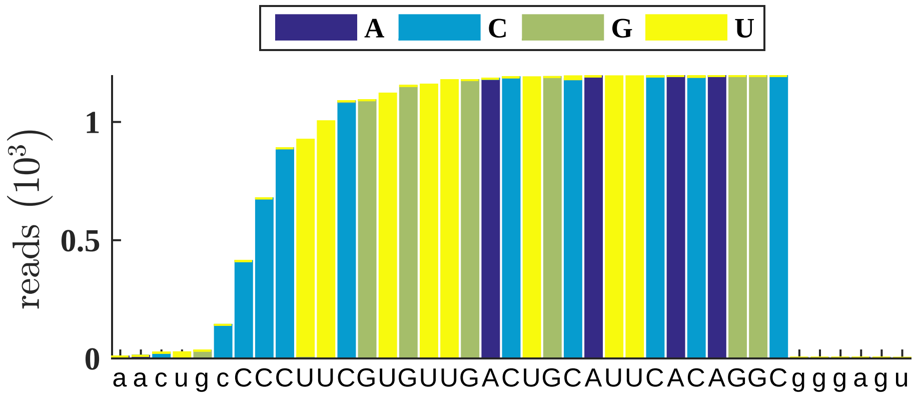

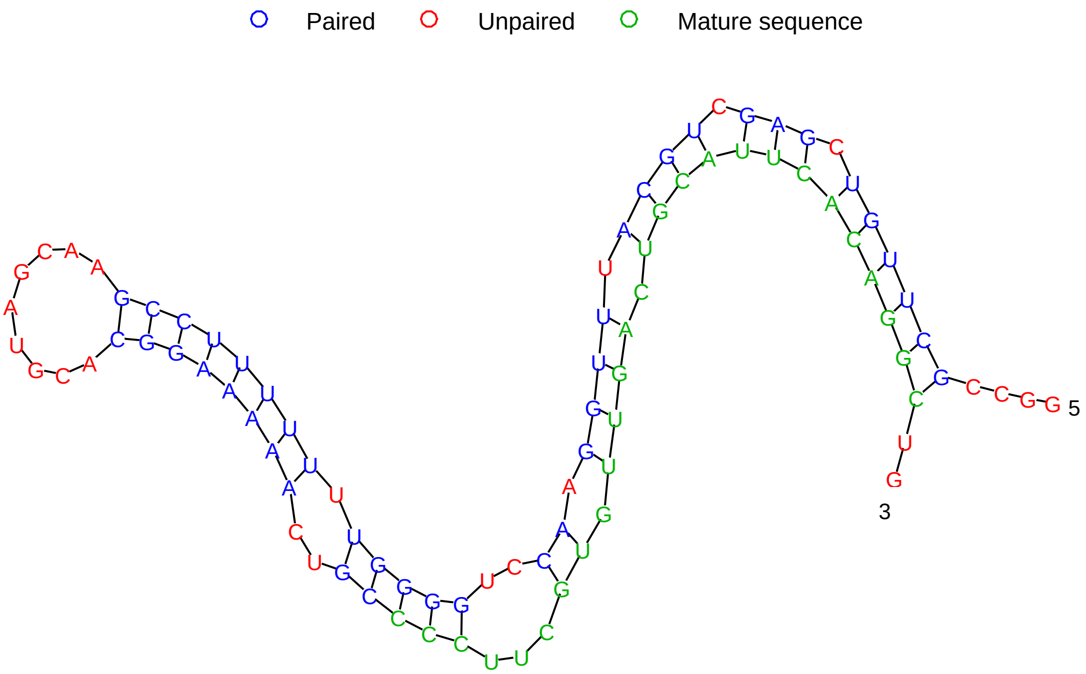

Stem loop (UMD3.1): chr25:18915736-18915828
Mature (UMD3.1): chr25:18915738-18915764
Mature seq len: 27
Total raw counts (9 samples): 1260
Average raw counts: 140
Strand: Reverse
Orientation: 3p
Minimum free energy: -31.70

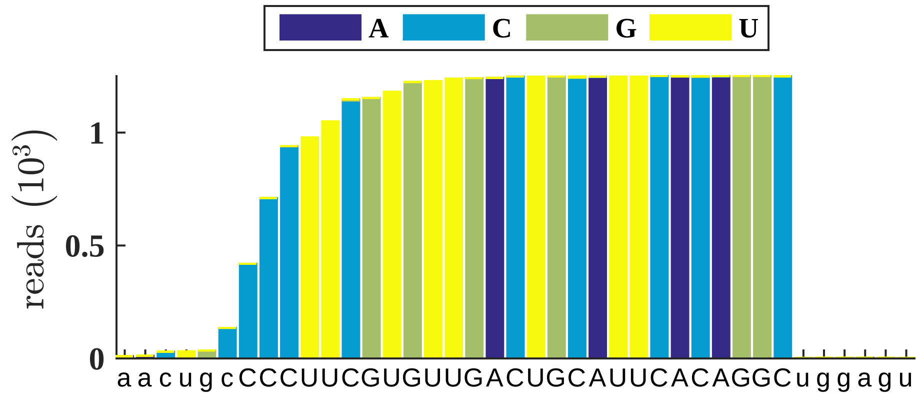

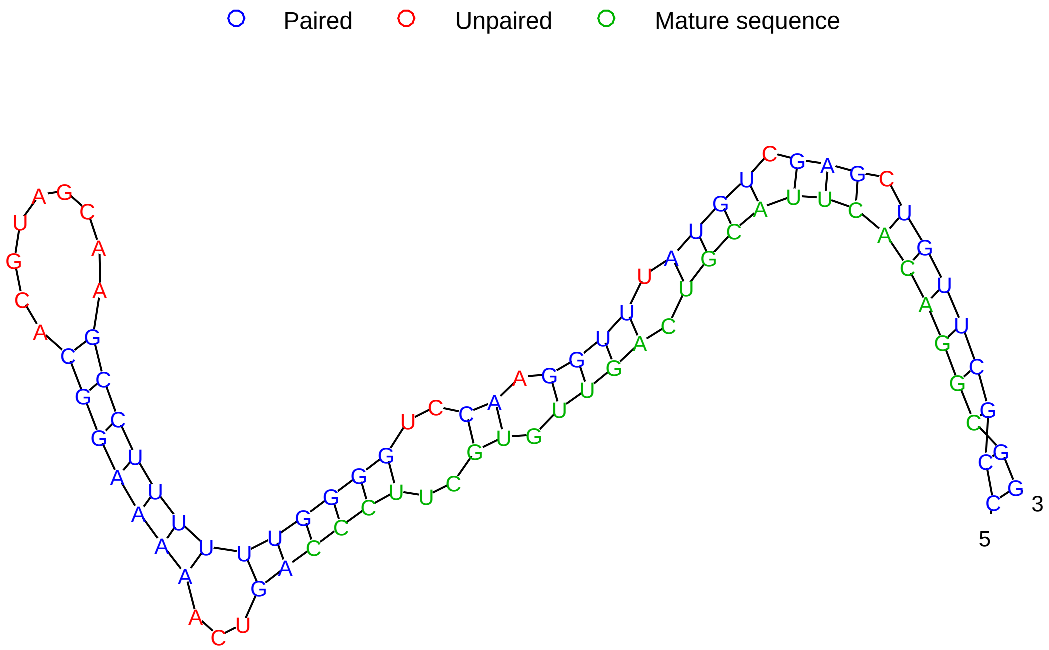

Stem loop (UMD3.1): chr25:18916903-18916992
Mature (UMD3.1): chr25:18916905-18916931
Mature seq len: 27
Total raw counts (9 samples): 1020
Average raw counts: 114
Strand: Reverse
Orientation: 3p
Minimum free energy: -31.80

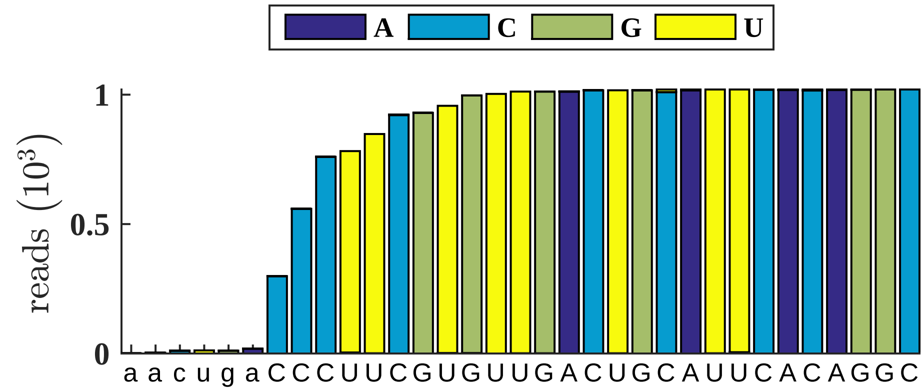

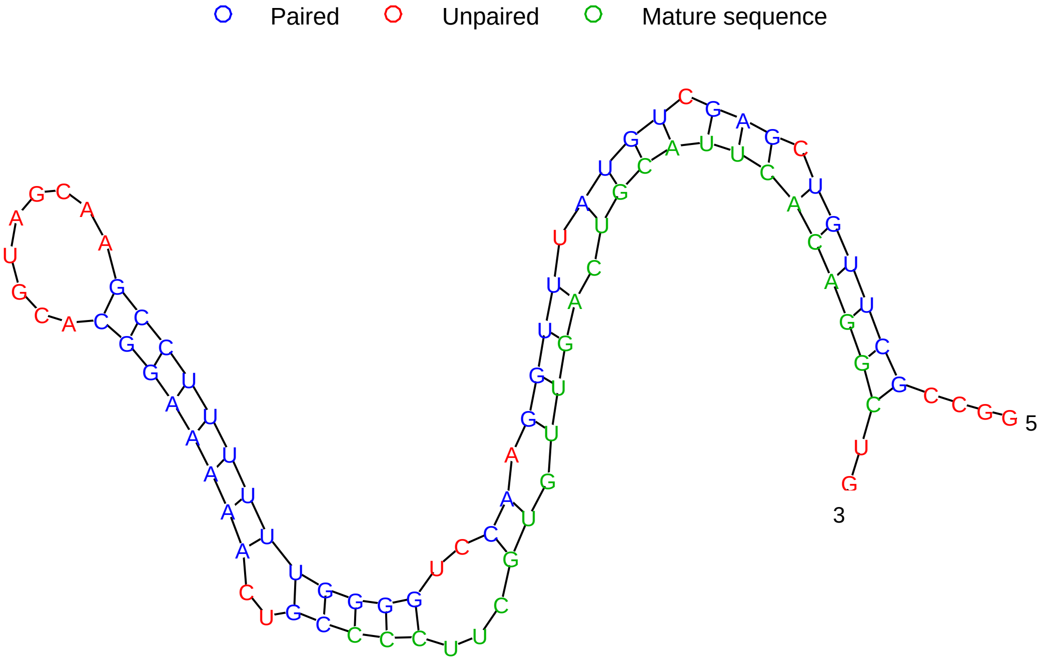

Stem loop (UMD3.1): chr25:19012081-19012172
Mature (UMD3.1): chr25:19012083-19012109
Mature seq len: 27
Total raw counts (9 samples): 1217
Average raw counts: 136
Strand: Reverse
Orientation: 3p
Minimum free energy: -28.90

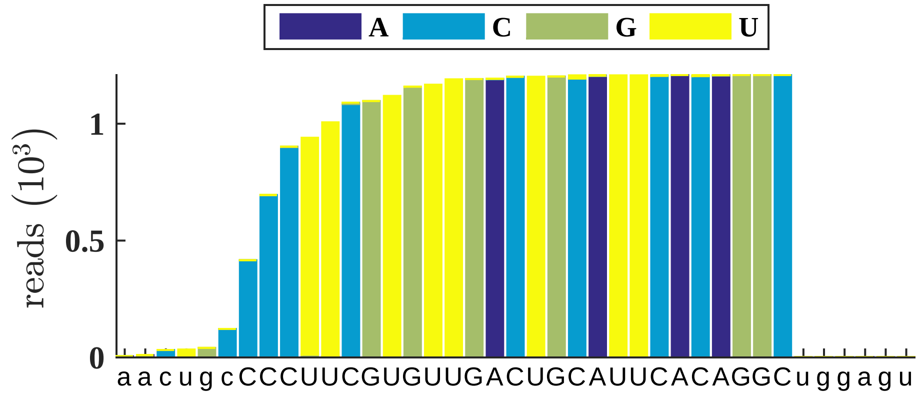

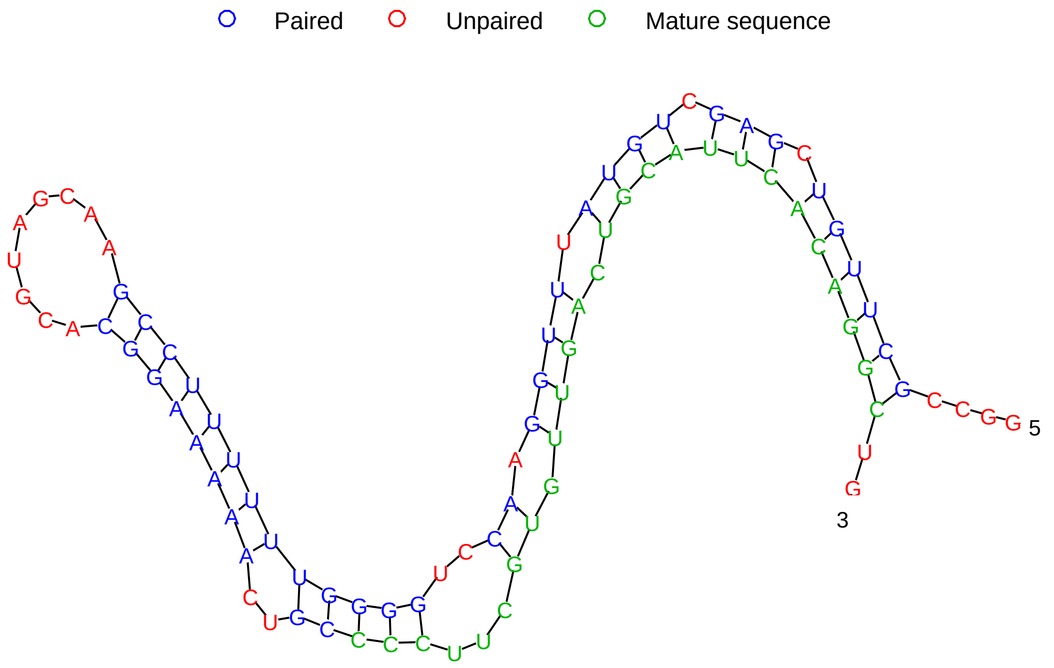

Stem loop (UMD3.1): chr25:19014119-19014210
Mature (UMD3.1): chr25:19014121-19014147
Mature seq len: 27
Total raw counts (9 samples): 1217
Average raw counts: 136
Strand: Reverse
Orientation: 3p
Minimum free energy: -28.90

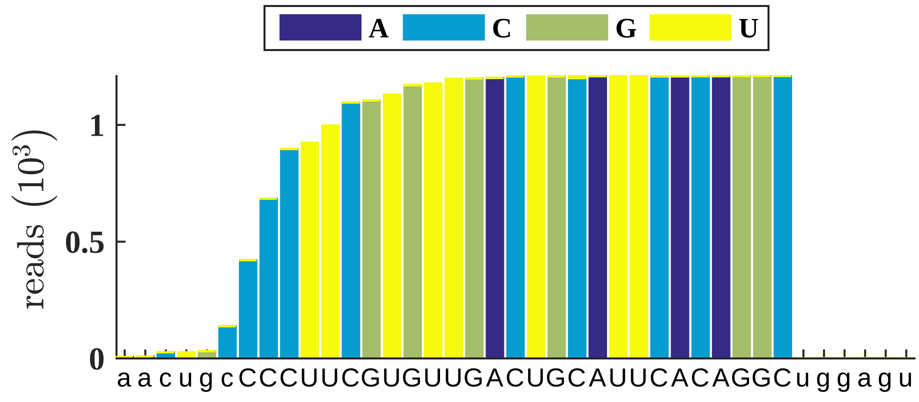

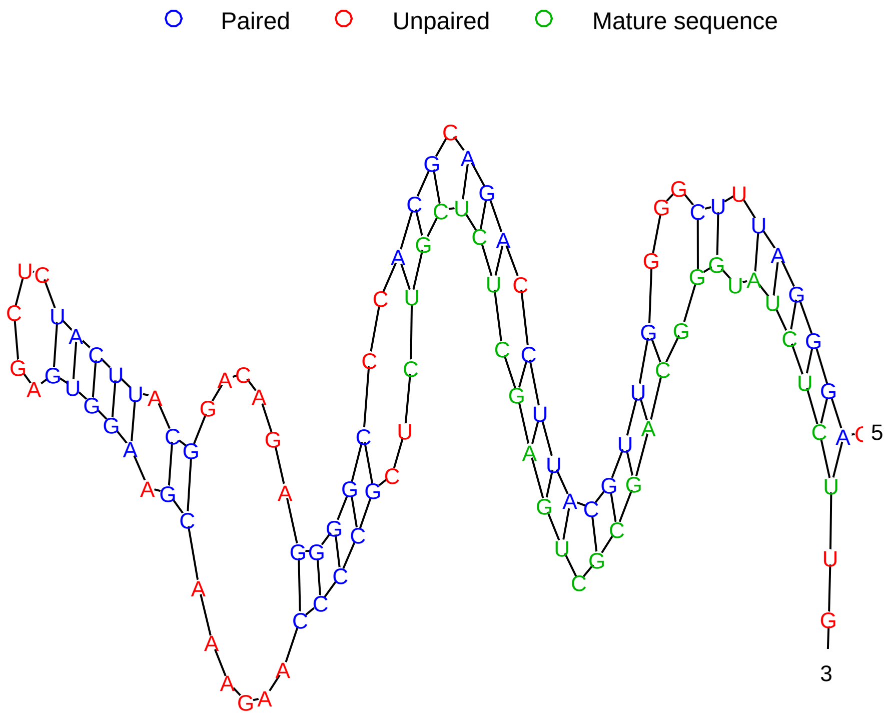

Stem loop (UMD3.1): chr25:19020707-19020813
Mature (UMD3.1): chr25:19020709-19020736
Mature seq len: 28
Total raw counts (9 samples): 13314
Average raw counts: 1480
Strand: Reverse
Orientation: 3p
Minimum free energy: -35.10

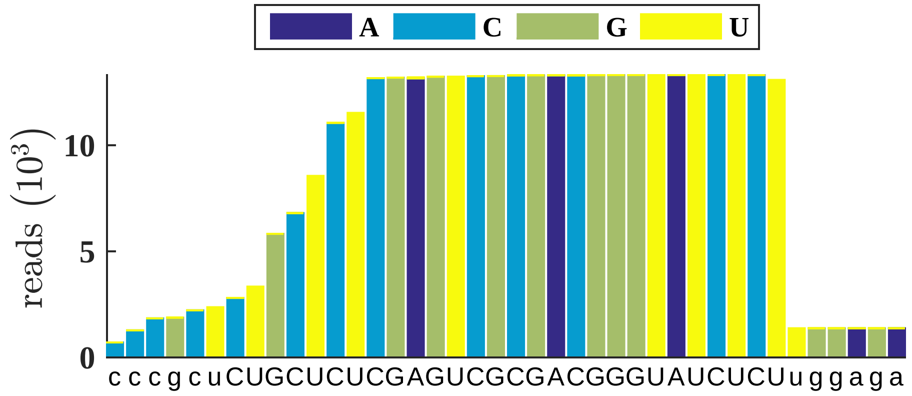

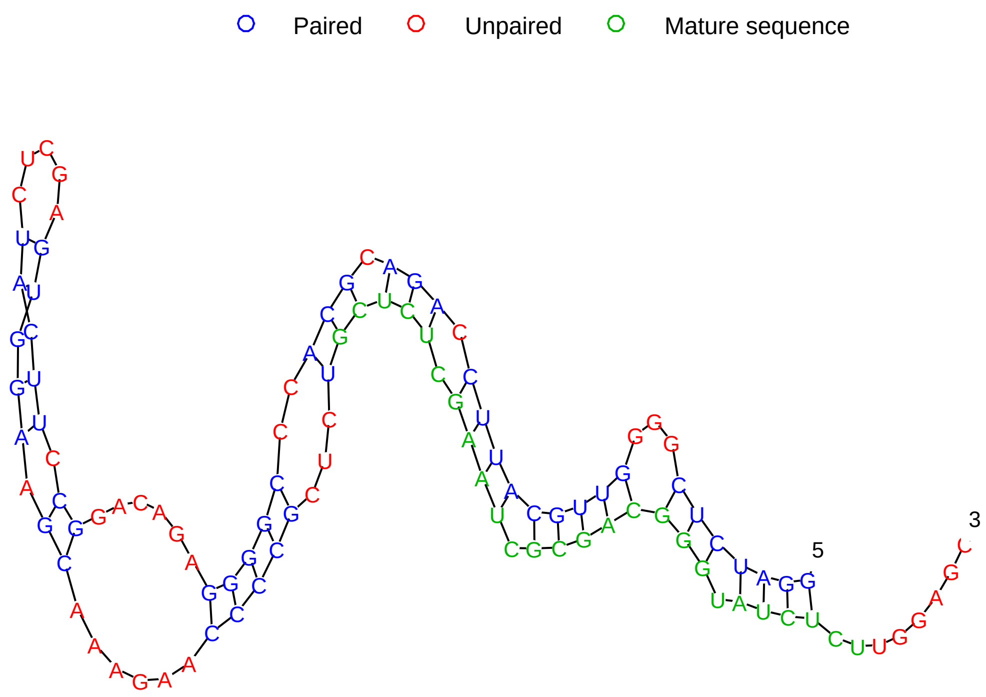

Stem loop (UMD3.1): chr25:19034940-19035047
Mature (UMD3.1): chr25:19034946-19034971
Mature seq len: 26
Total raw counts (9 samples): 4032
Average raw counts: 448
Strand: Reverse
Orientation: 3p
Minimum free energy: -32.40

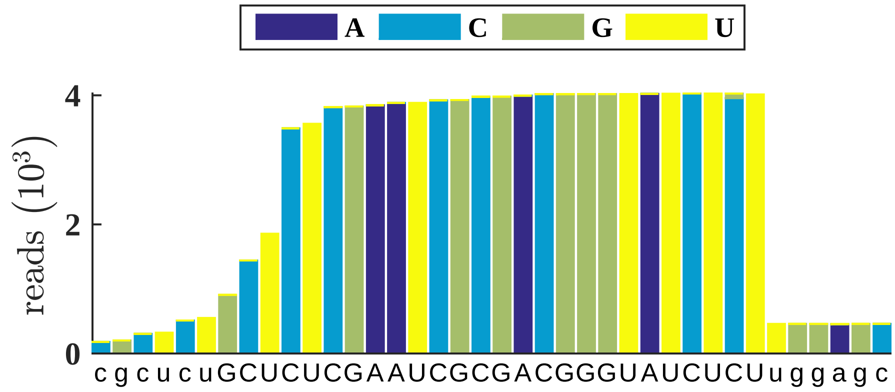

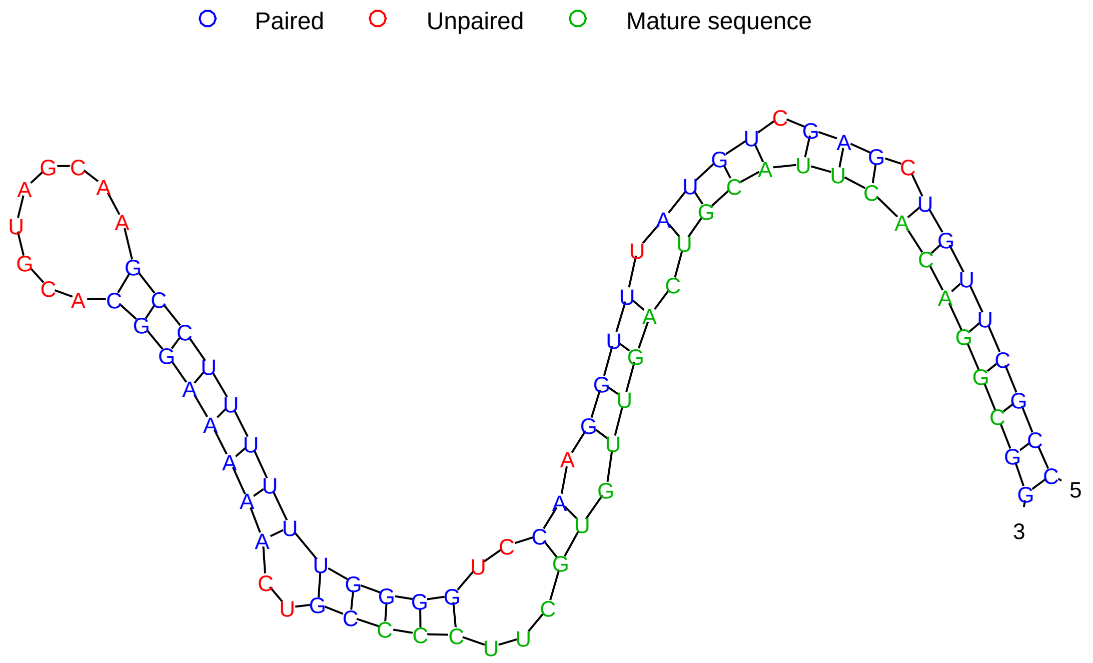

Stem loop (UMD3.1): chr25:19038518-19038607
Mature (UMD3.1): chr25:19038520-19038546
Mature seq len: 27
Total raw counts (9 samples): 1230
Average raw counts: 137
Strand: Reverse
Orientation: 3p
Minimum free energy: -33.20

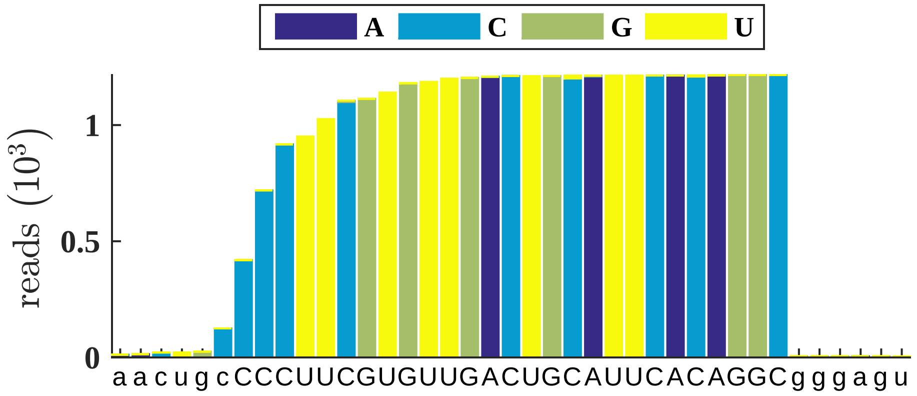

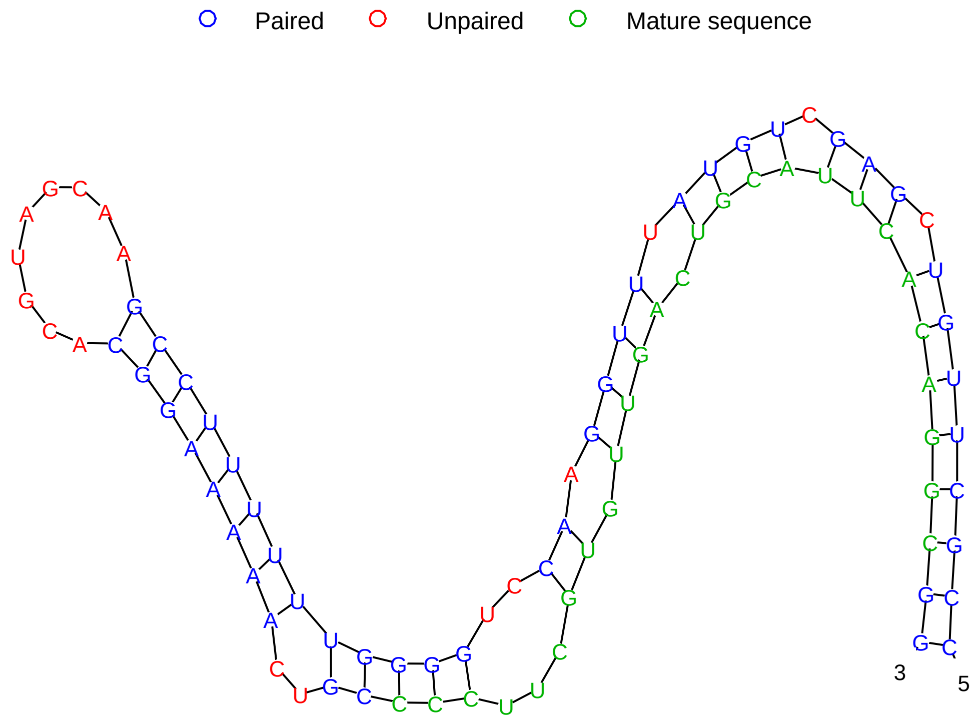

Stem loop (UMD3.1): chr25:19041425-19041514
Mature (UMD3.1): chr25:19041427-19041453
Mature seq len: 27
Total raw counts (9 samples): 1201
Average raw counts: 134
Strand: Reverse
Orientation: 3p
Minimum free energy: -33.20

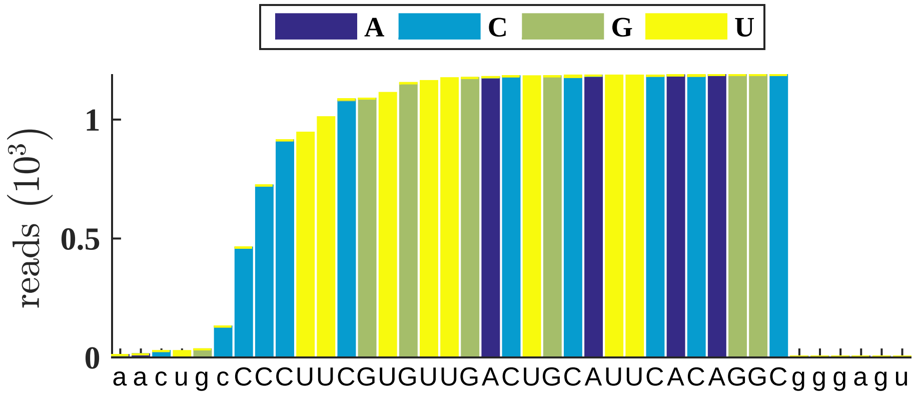

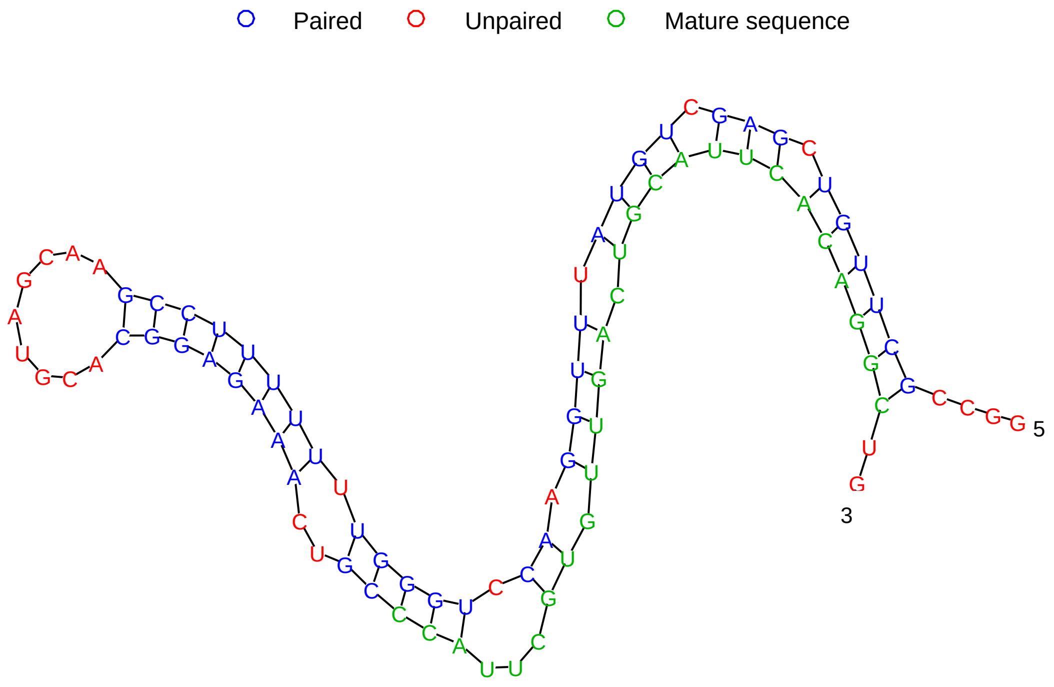

Stem loop (UMD3.1): chr25:19043619-19043710
Mature (UMD3.1): chr25:19043621-19043647
Mature seq len: 27
Total raw counts (9 samples): 708
Average raw counts: 79
Strand: Reverse
Orientation: 3p
Minimum free energy: -27.90

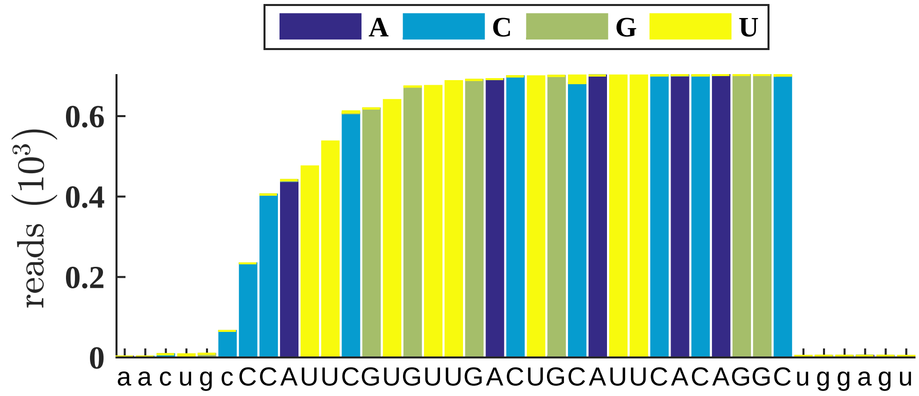

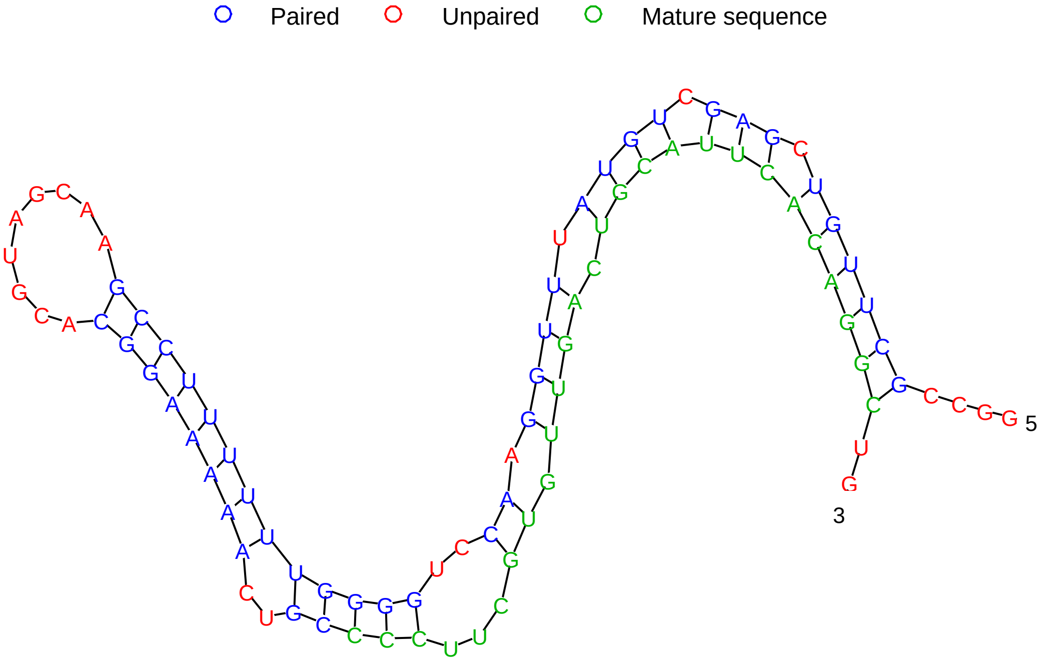

Stem loop (UMD3.1): chr25:30287754-30287845
Mature (UMD3.1): chr25:30287817-30287843
Mature seq len: 27
Total raw counts (9 samples): 1190
Average raw counts: 133
Strand: Forward
Orientation: 3p
Minimum free energy: -28.90

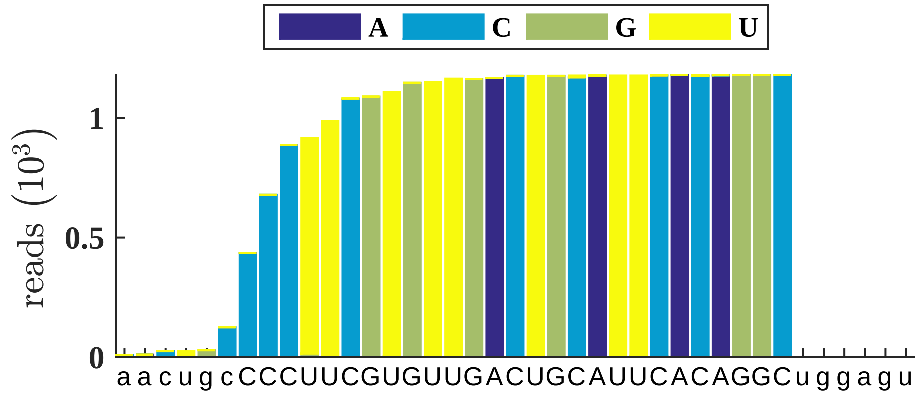

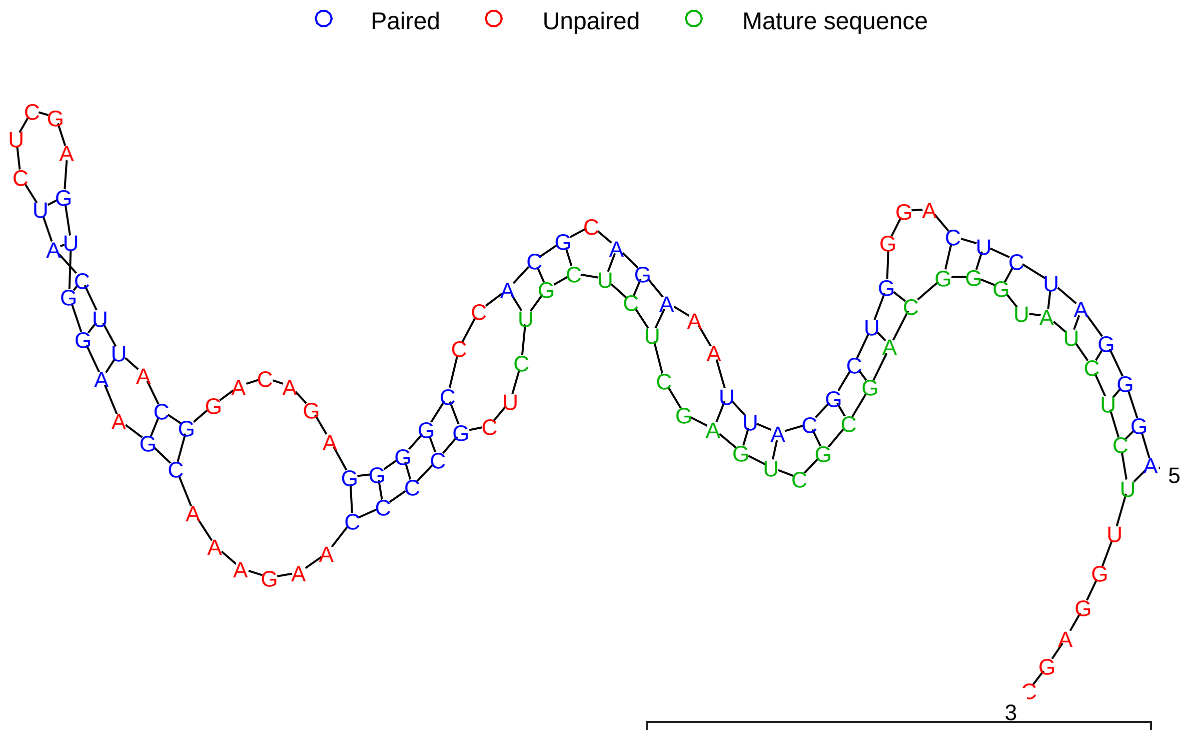

Stem loop (UMD3.1): chr25:30288400-30288509
Mature (UMD3.1): chr25:30288476-30288503
Mature seq len: 28
Total raw counts (9 samples): 13273
Average raw counts: 1475
Strand: Forward
Orientation: 3p
Minimum free energy: -35.50

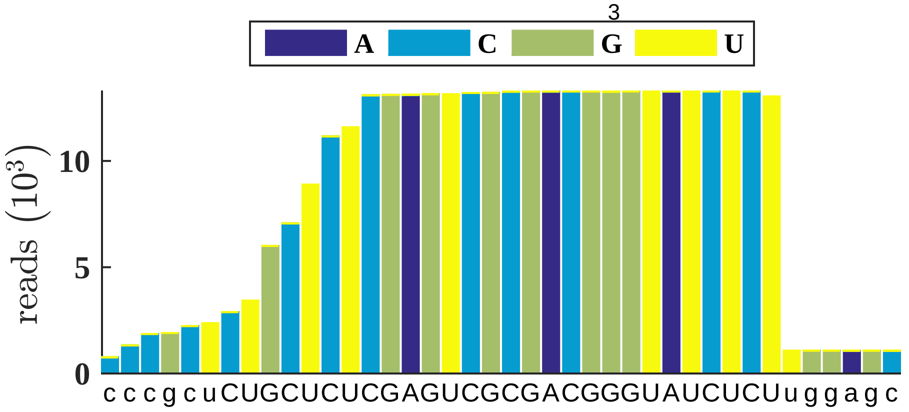

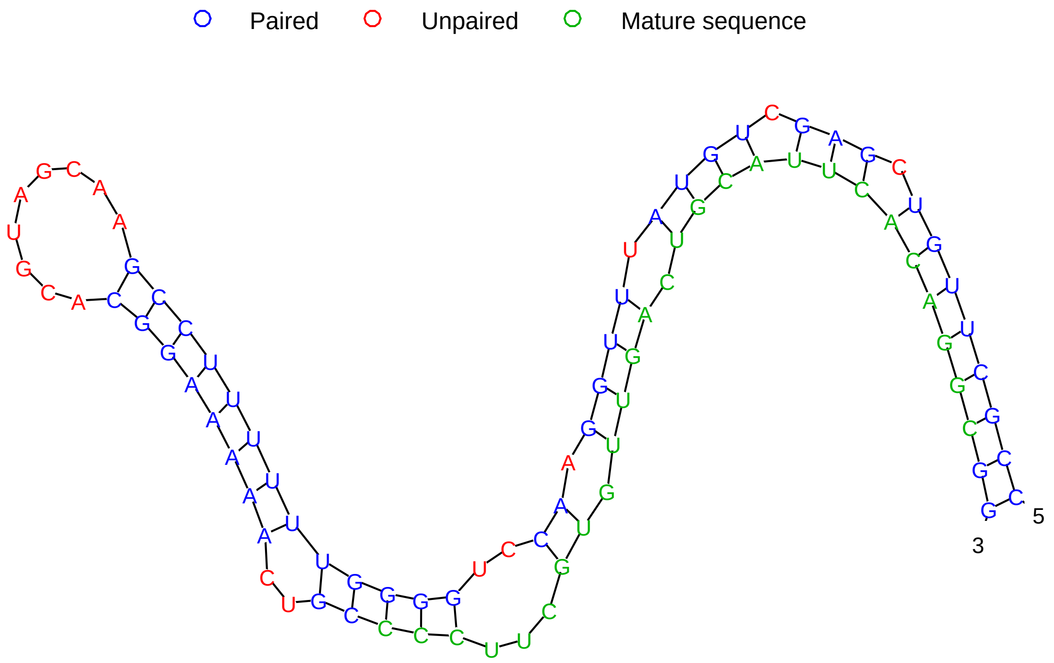

Stem loop (UMD3.1): chr25:30291955-30292044
Mature (UMD3.1): chr25:30292016-30292042
Mature seq len: 27
Total raw counts (9 samples): 1173
Average raw counts: 131
Strand: Forward
Orientation: 3p
Minimum free energy: -33.20

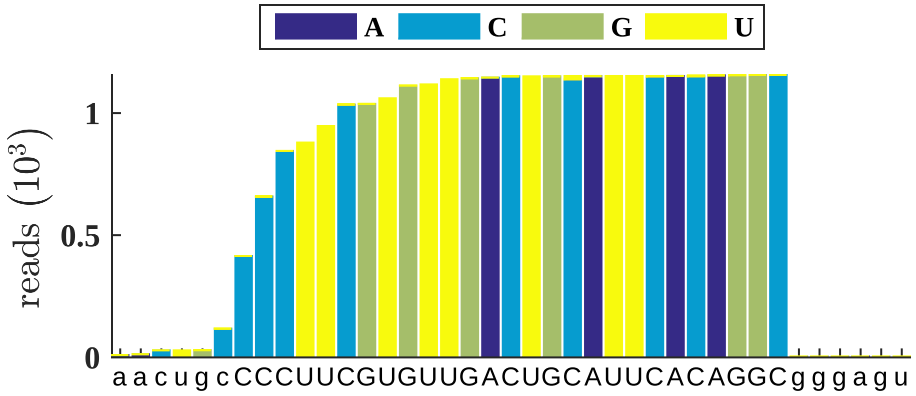

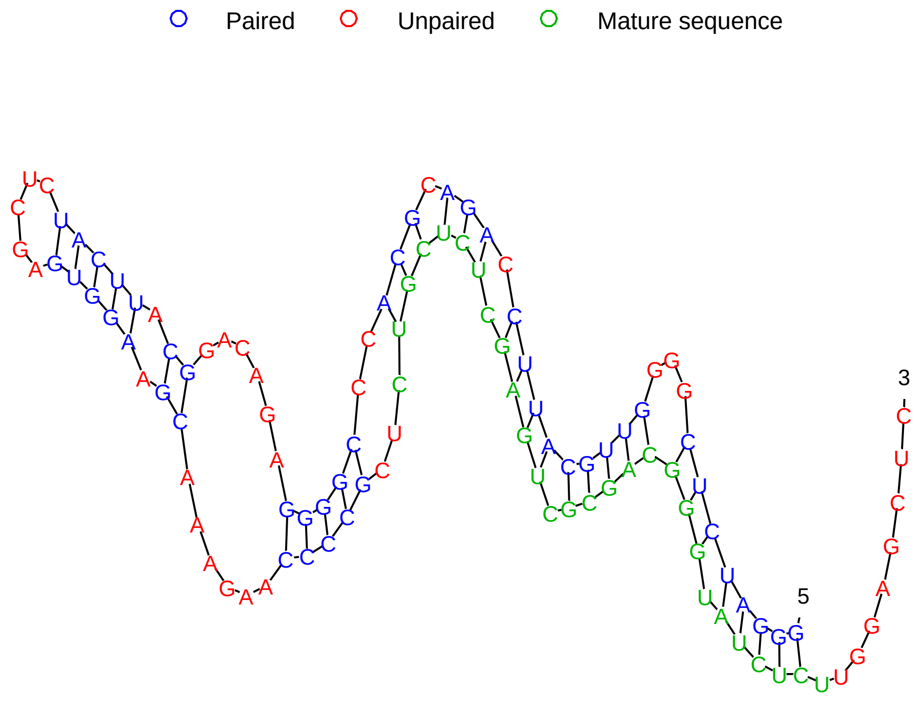

Stem loop (UMD3.1): chr25:30294835-30294945
Mature (UMD3.1): chr25:30294910-30294937
Mature seq len: 28
Total raw counts (9 samples): 13257
Average raw counts: 1473
Strand: Forward
Orientation: 3p
Minimum free energy: -35.50

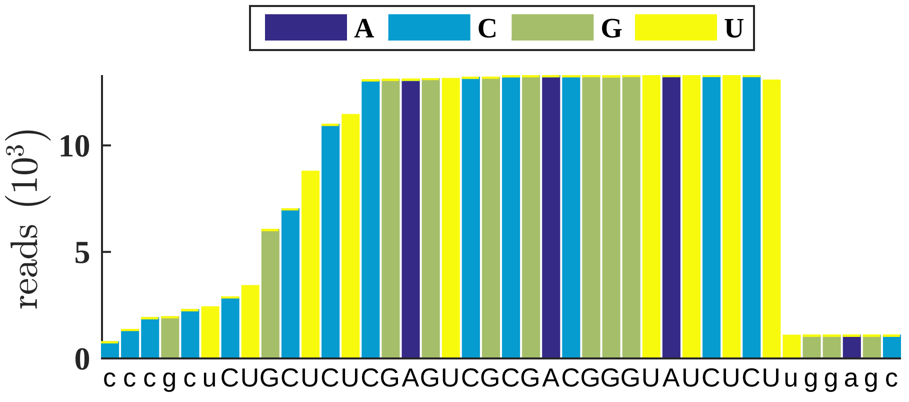

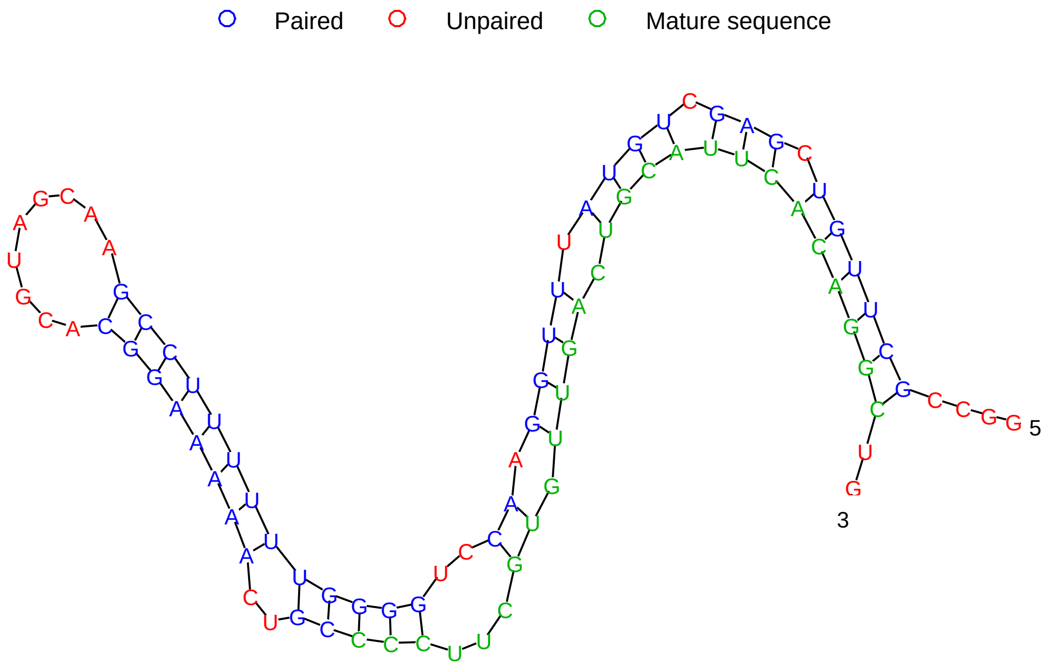

Stem loop (UMD3.1): chr25:30295554-30295645
Mature (UMD3.1): chr25:30295617-30295643
Mature seq len: 27
Total raw counts (9 samples): 1186
Average raw counts: 132
Strand: Forward
Orientation: 3p
Minimum free energy: -28.90

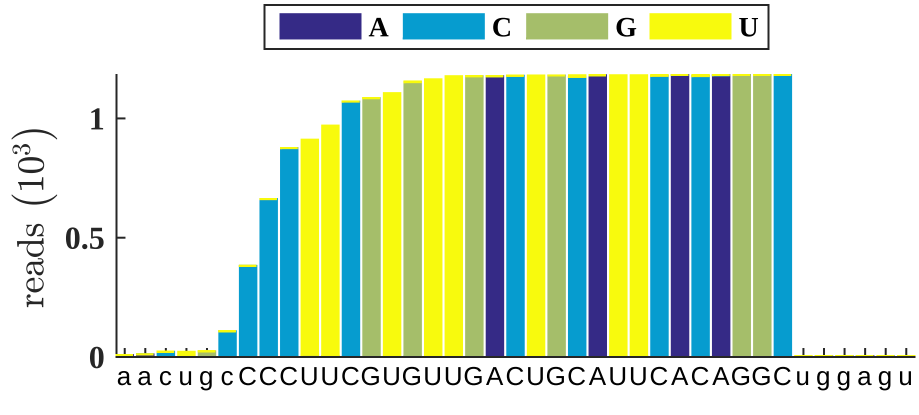

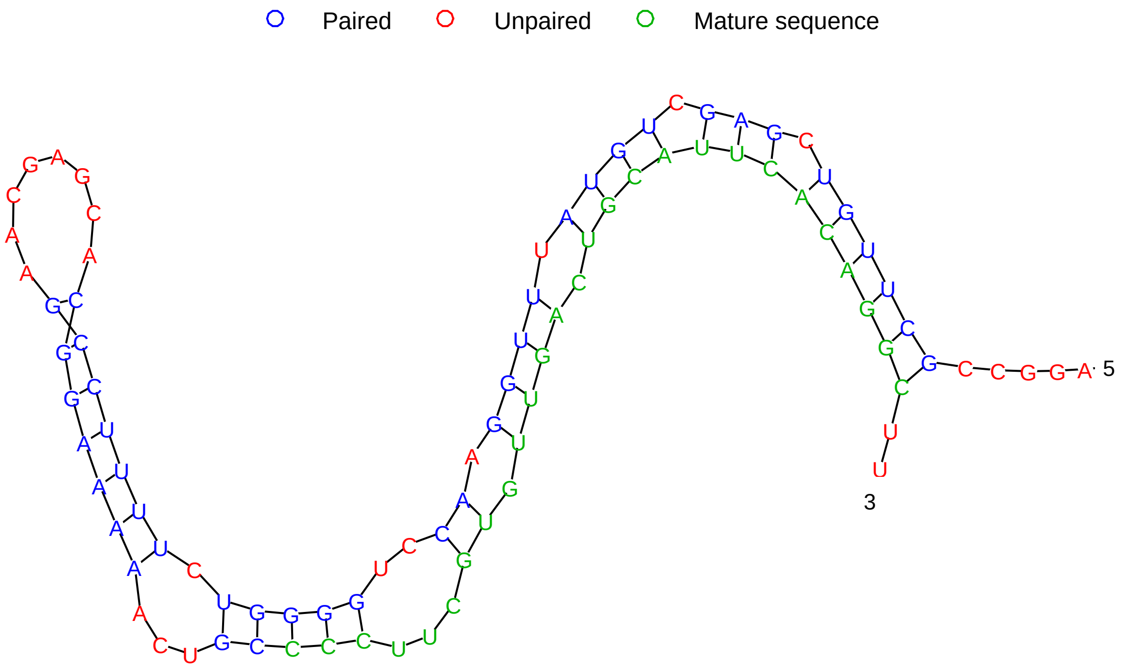

Stem loop (UMD3.1): chr25:30297879-30297970
Mature (UMD3.1): chr25:30297942-30297968
Mature seq len: 27
Total raw counts (9 samples): 1116
Average raw counts: 124
Strand: Forward
Orientation: 3p
Minimum free energy: -29.00

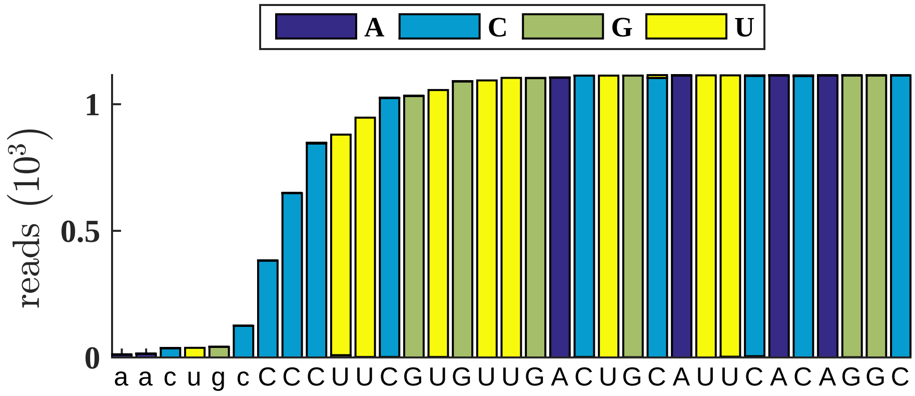

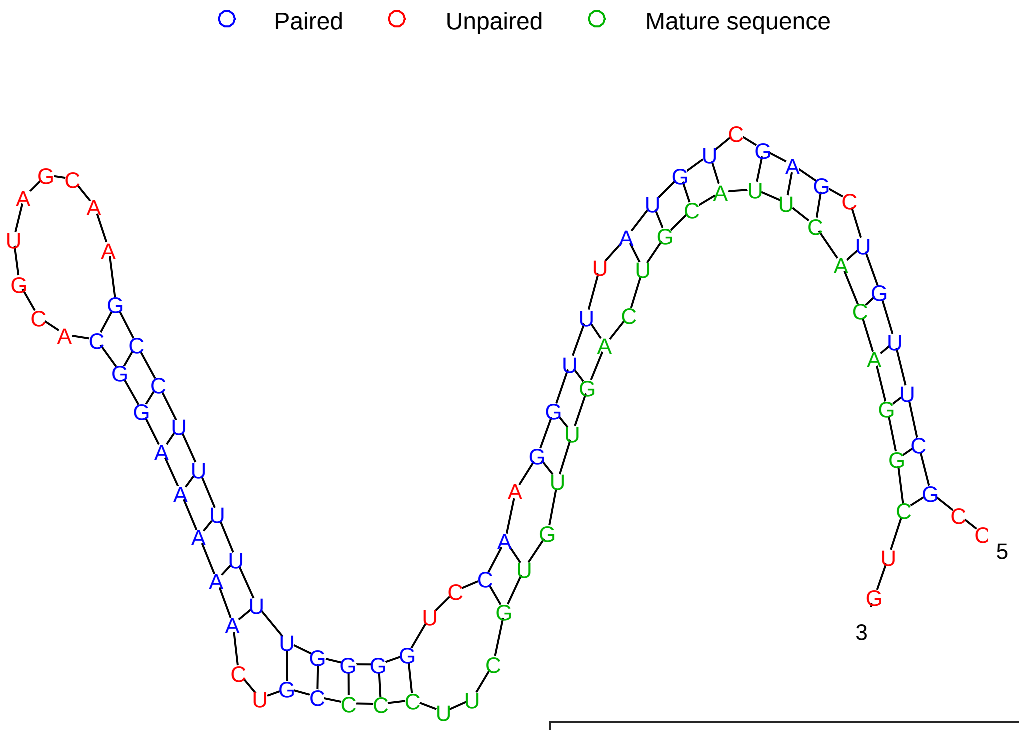

Stem loop (UMD3.1): chr25:30300037-30300126
Mature (UMD3.1): chr25:30300098-30300124
Mature seq len: 27
Total raw counts (9 samples): 1222
Average raw counts: 136
Strand: Forward
Orientation: 3p
Minimum free energy: -28.90

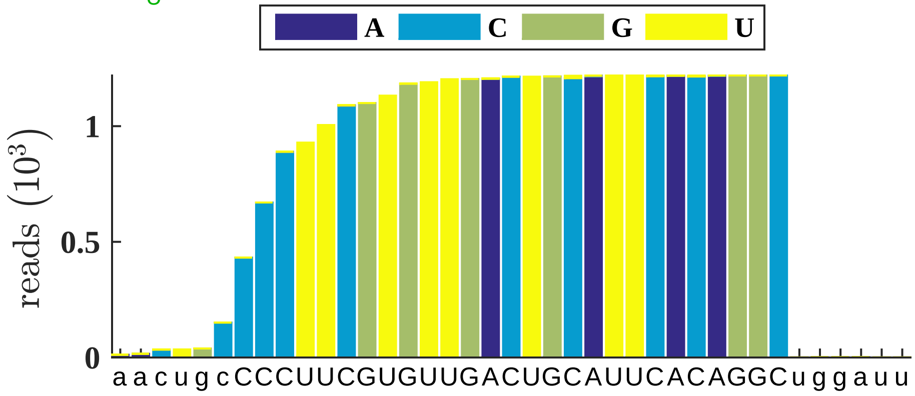

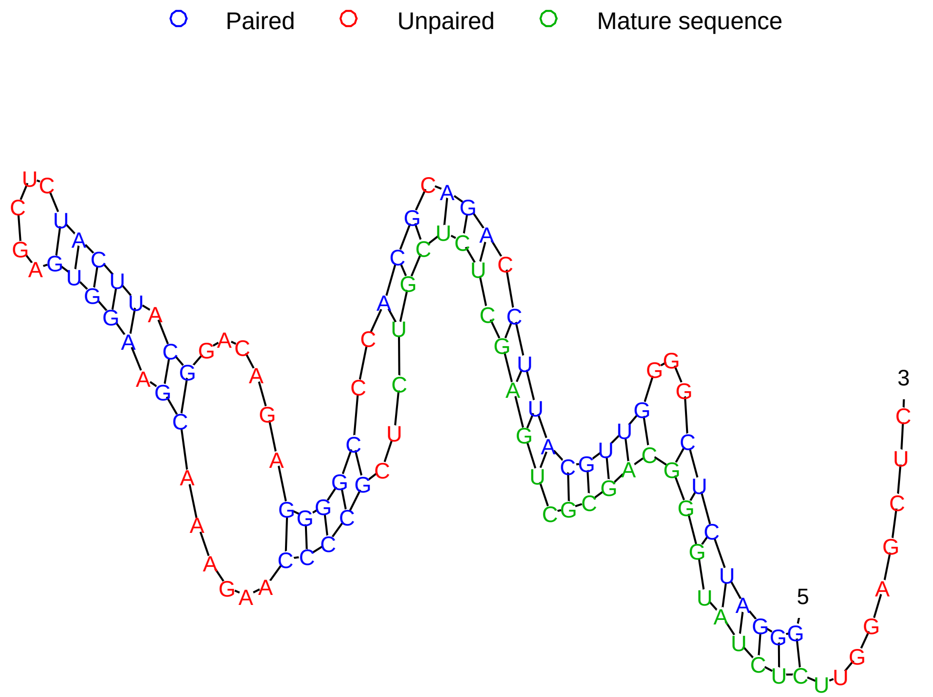

Stem loop (UMD3.1): chr25:30304491-30304601
Mature (UMD3.1): chr25:30304566-30304593
Mature seq len: 28
Total raw counts (9 samples): 13283
Average raw counts: 1476
Strand: Forward
Orientation: 3p
Minimum free energy: -35.50

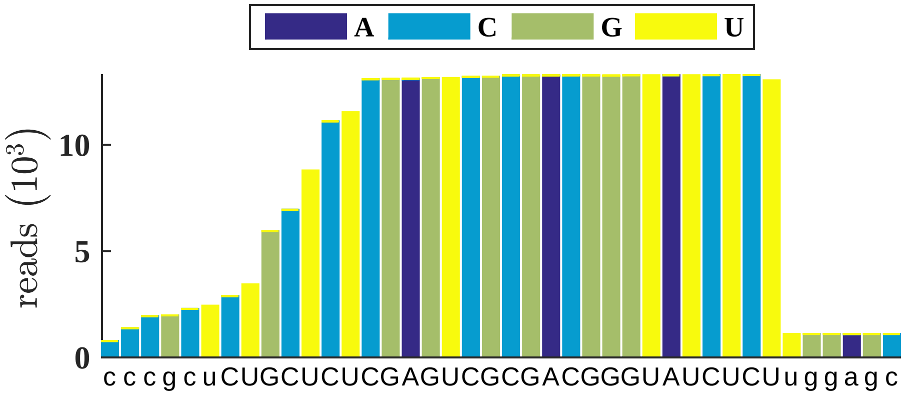

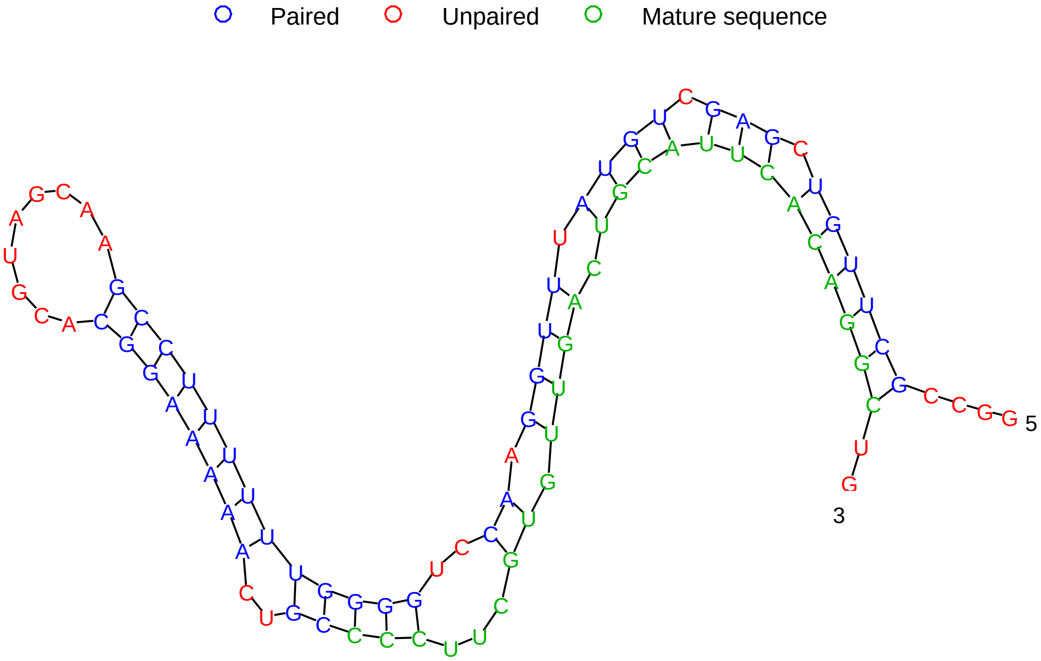

Stem loop (UMD3.1): chr25:30306452-30306543
Mature (UMD3.1): chr25:30306515-30306541
Mature seq len: 27
Total raw counts (9 samples): 1249
Average raw counts: 139
Strand: Forward
Orientation: 3p
Minimum free energy: -28.90

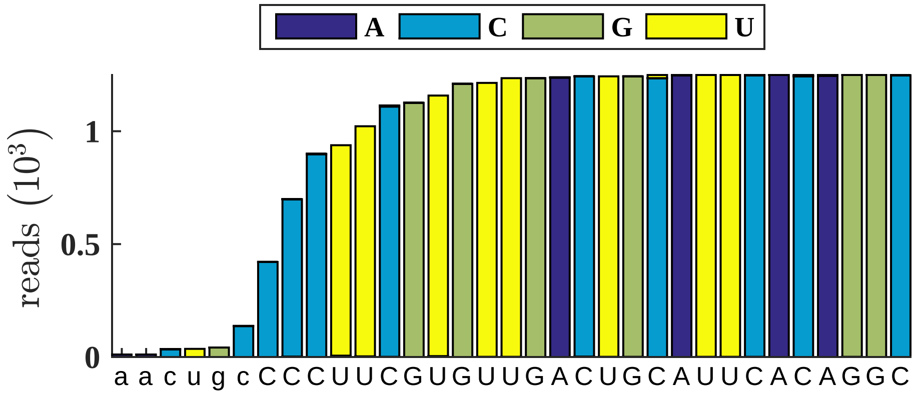

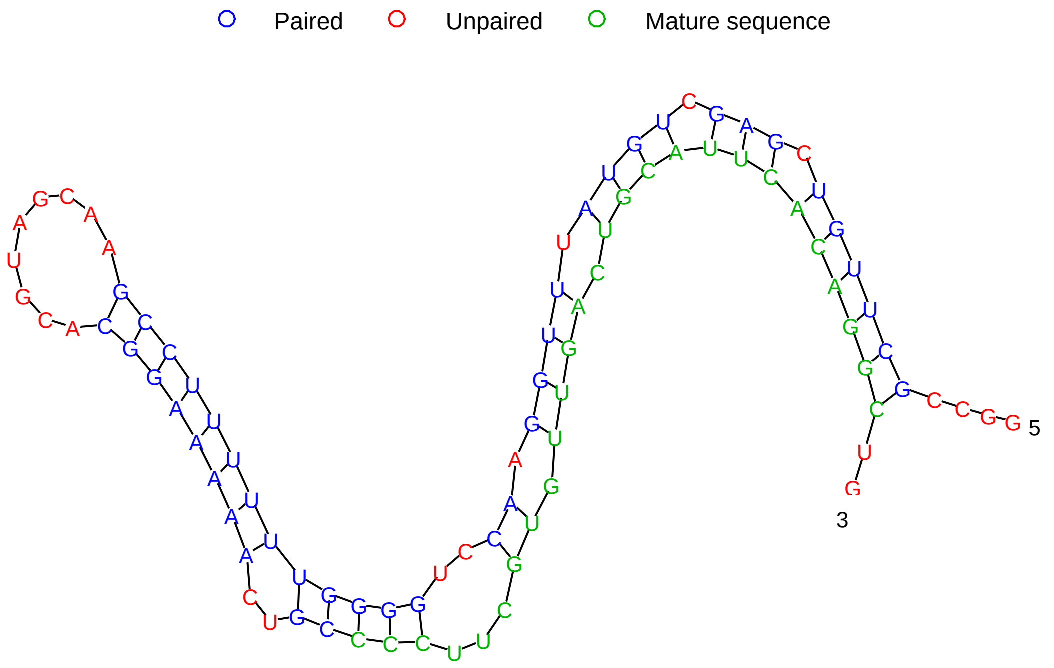

Stem loop (UMD3.1): chr25:30308238-30308329
Mature (UMD3.1): chr25:30308301-30308327
Mature seq len: 27
Total raw counts (9 samples): 1237
Average raw counts: 138
Strand: Forward
Orientation: 3p
Minimum free energy: -28.90

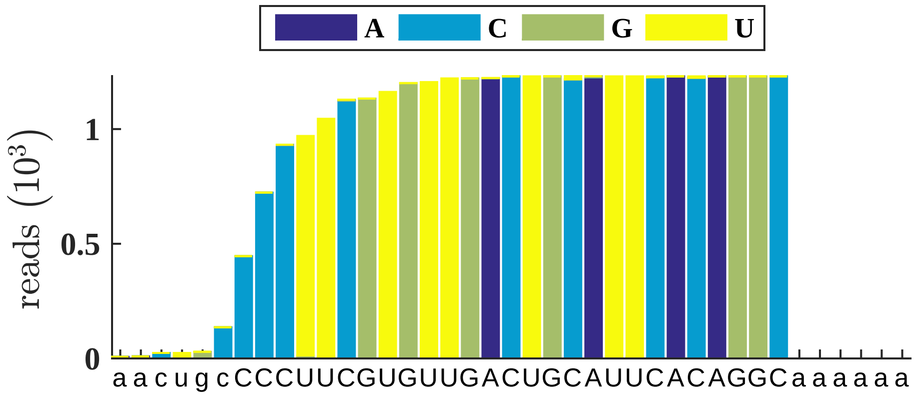

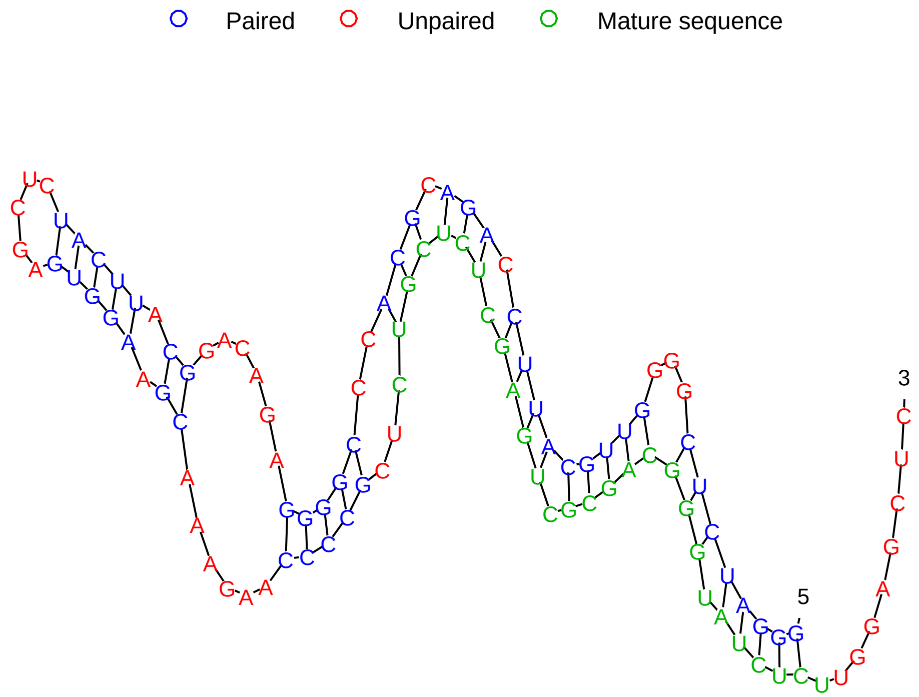

Stem loop (UMD3.1): chr25:30312552-30312662
Mature (UMD3.1): chr25:30312627-30312654
Mature seq len: 28
Total raw counts (9 samples): 13439
Average raw counts: 1494
Strand: Forward
Orientation: 3p
Minimum free energy: -35.50

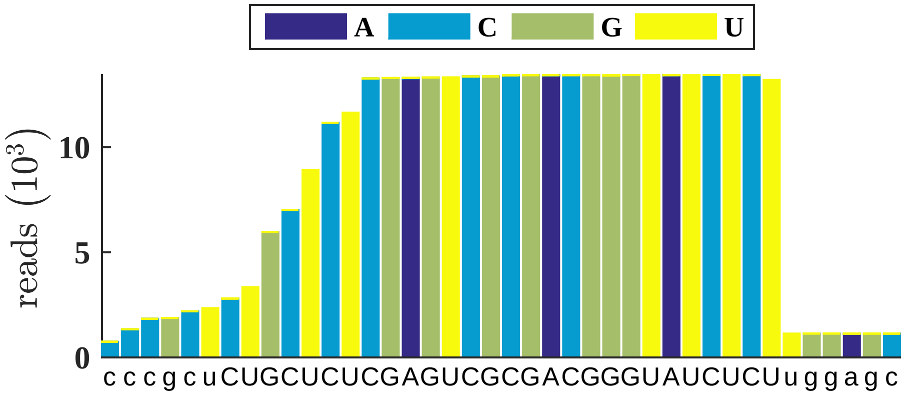

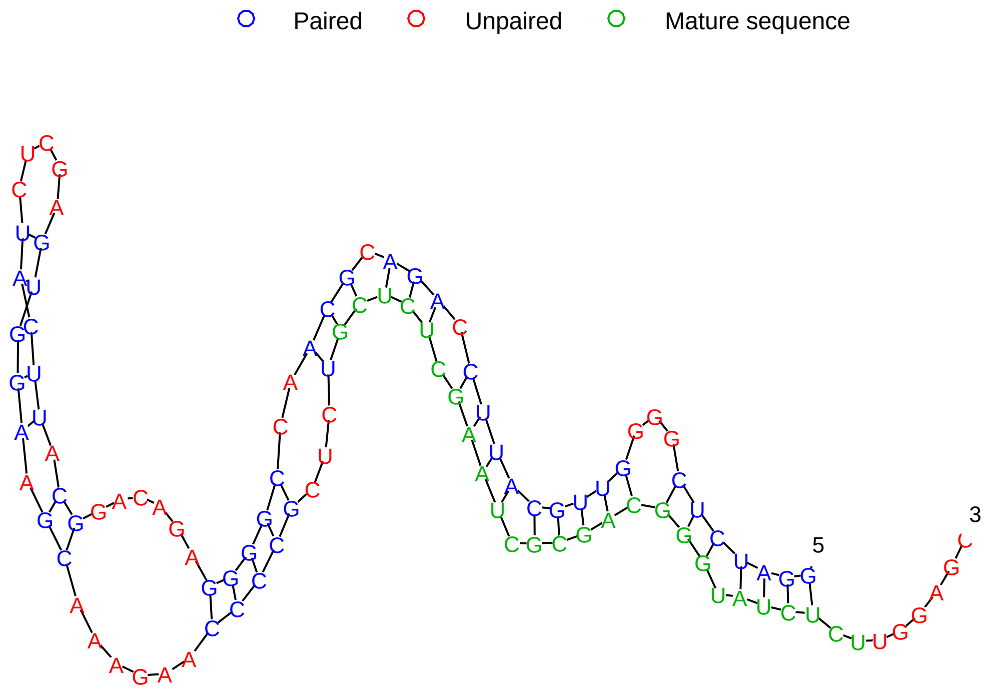

Stem loop (UMD3.1): chr25:30319058-30319165
Mature (UMD3.1): chr25:30319134-30319159
Mature seq len: 26
Total raw counts (9 samples): 3982
Average raw counts: 443
Strand: Forward
Orientation: 3p
Minimum free energy: -32.40

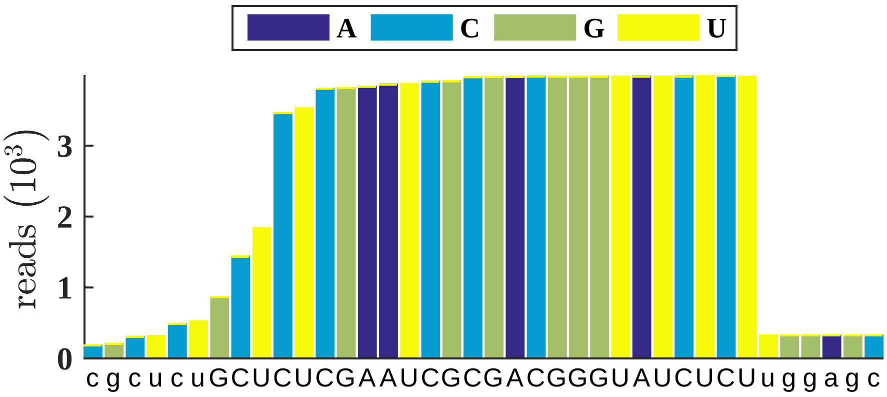

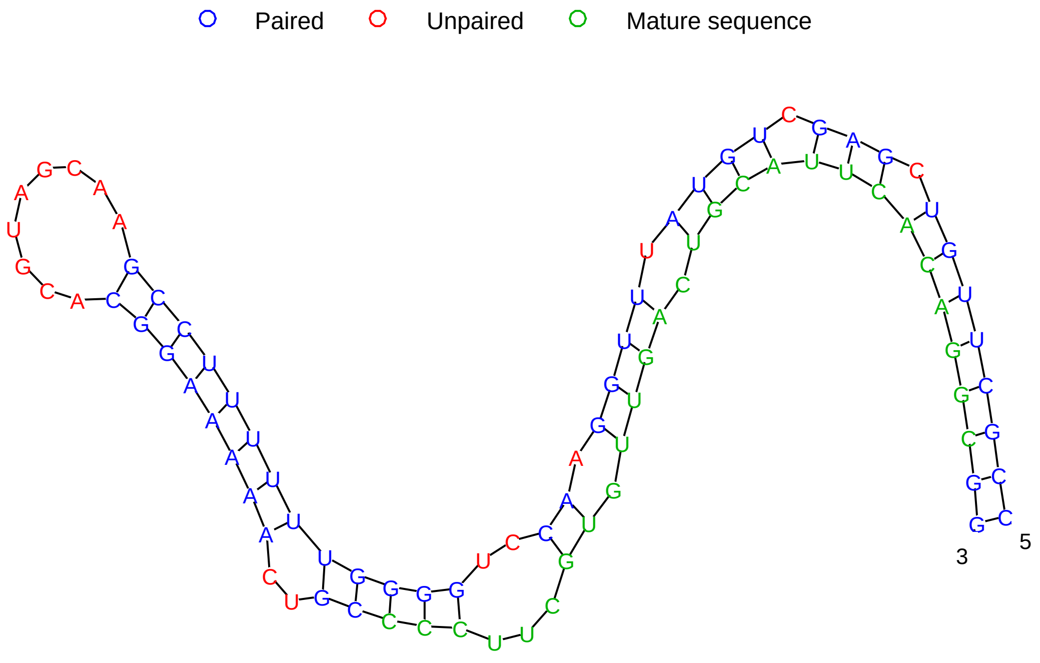

Stem loop (UMD3.1): chr25:30327594-30327683
Mature (UMD3.1): chr25:30327655-30327681
Mature seq len: 27
Total raw counts (9 samples): 1185
Average raw counts: 132
Strand: Forward
Orientation: 3p
Minimum free energy: -33.20

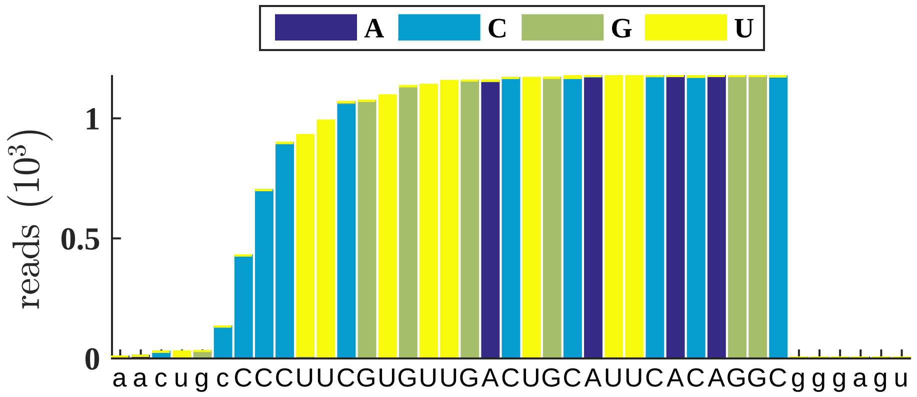

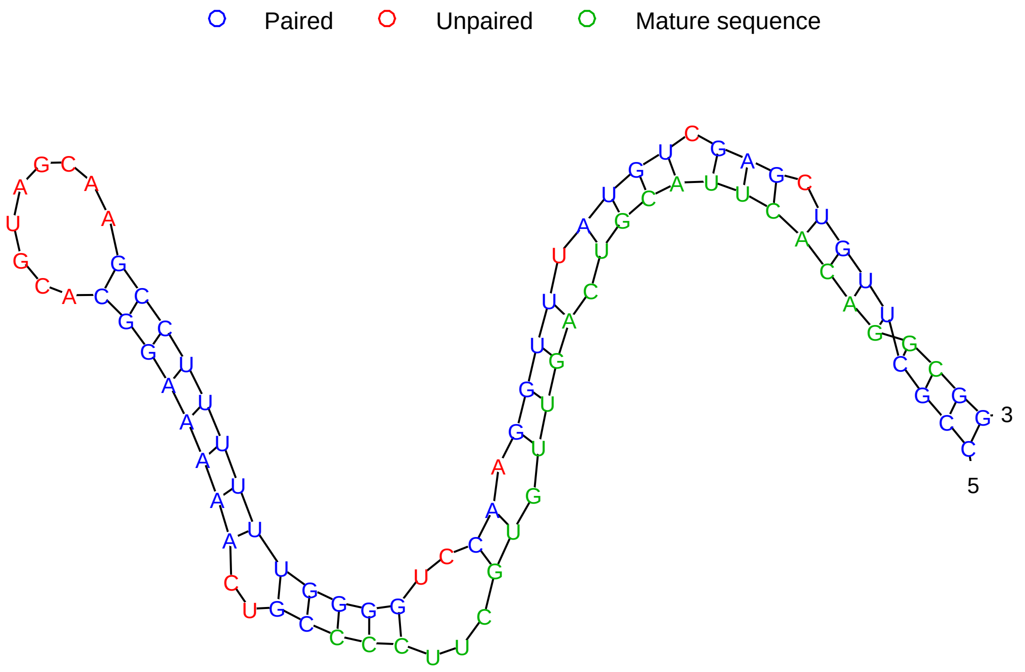

Stem loop (UMD3.1): chr25:30330383-30330472
Mature (UMD3.1): chr25:30330444-30330470
Mature seq len: 27
Total raw counts (9 samples): 1107
Average raw counts: 123
Strand: Forward
Orientation: 3p
Minimum free energy: -33.20

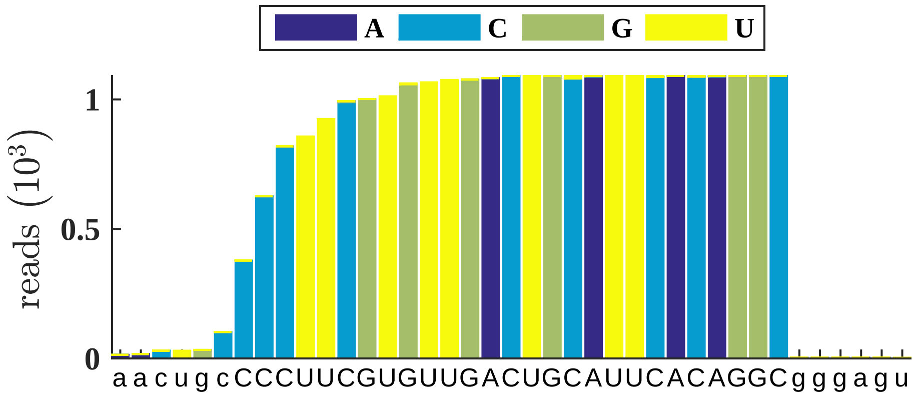

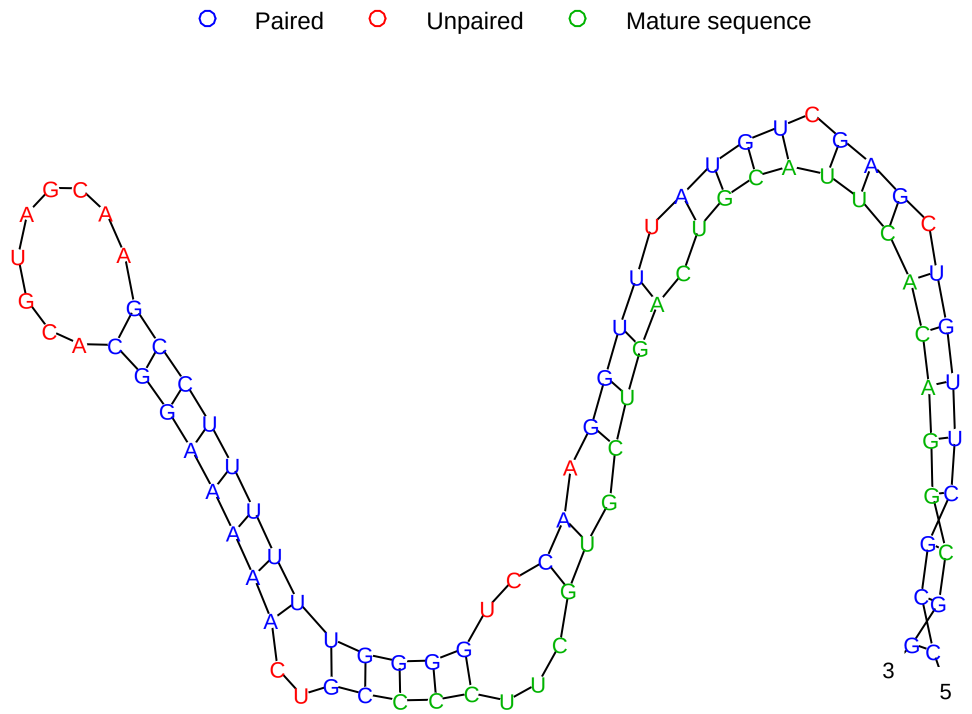

Stem loop (UMD3.1): chr25:30331750-30331839
Mature (UMD3.1): chr25:30331811-30331837
Mature seq len: 27
Total raw counts (9 samples): 1260
Average raw counts: 140
Strand: Forward
Orientation: 3p
Minimum free energy: -35.50

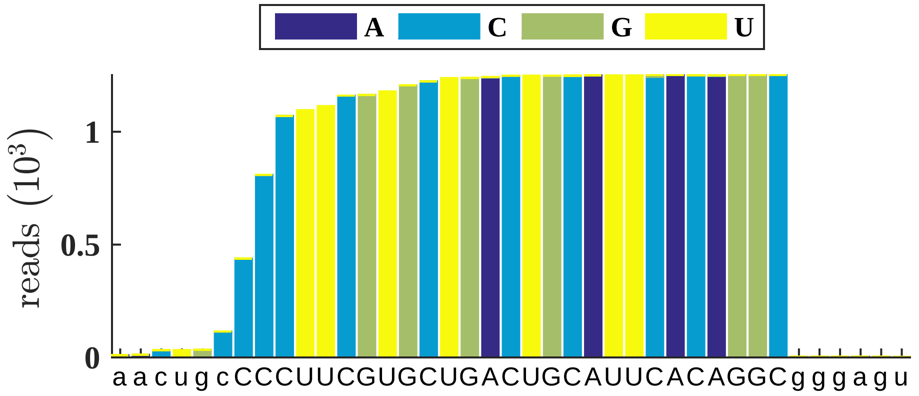

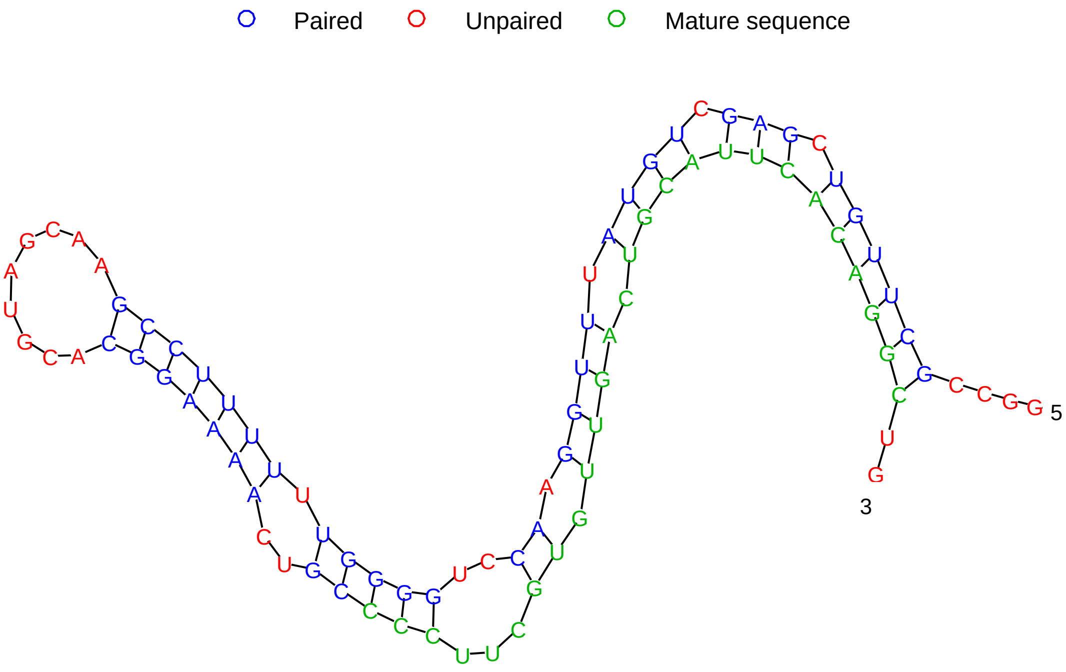

Stem loop (UMD3.1): chr25:30337269-30337359
Mature (UMD3.1): chr25:30337331-30337357
Mature seq len: 27
Total raw counts (9 samples): 1207
Average raw counts: 135
Strand: Forward
Orientation: 3p
Minimum free energy: -28.80

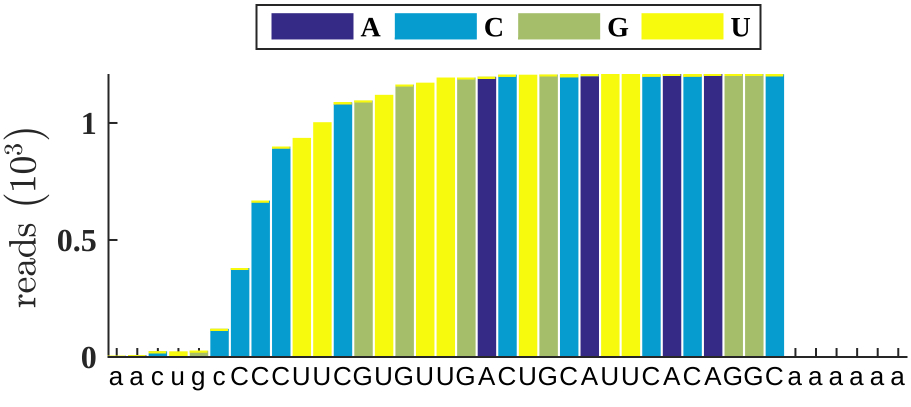

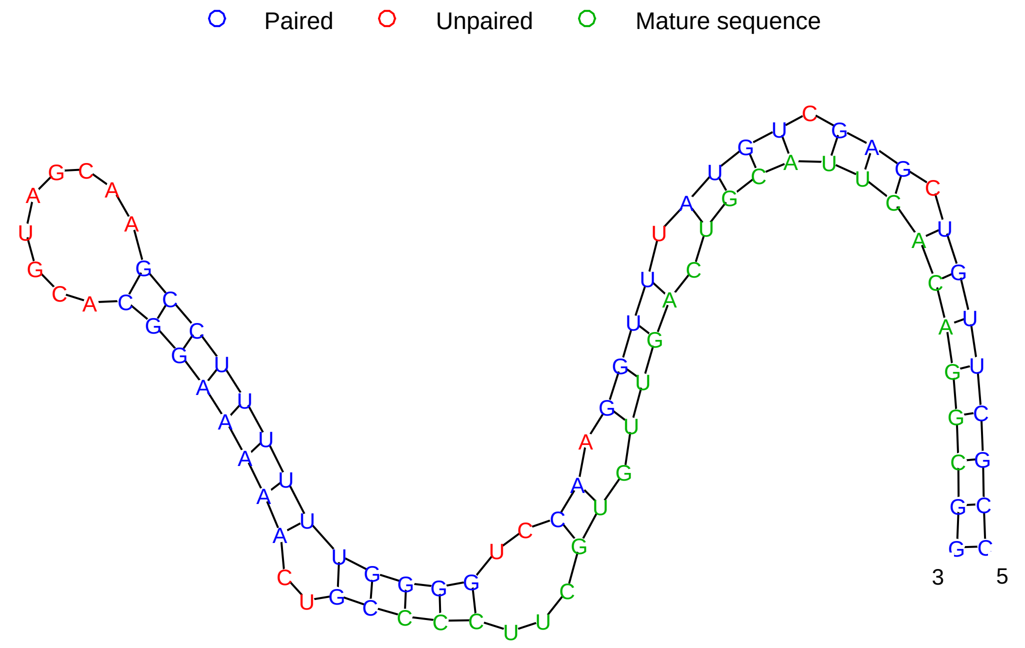

Stem loop (UMD3.1): chr25:30340633-30340722
Mature (UMD3.1): chr25:30340694-30340720
Mature seq len: 27
Total raw counts (9 samples): 1260
Average raw counts: 140
Strand: Forward
Orientation: 3p
Minimum free energy: -33.20

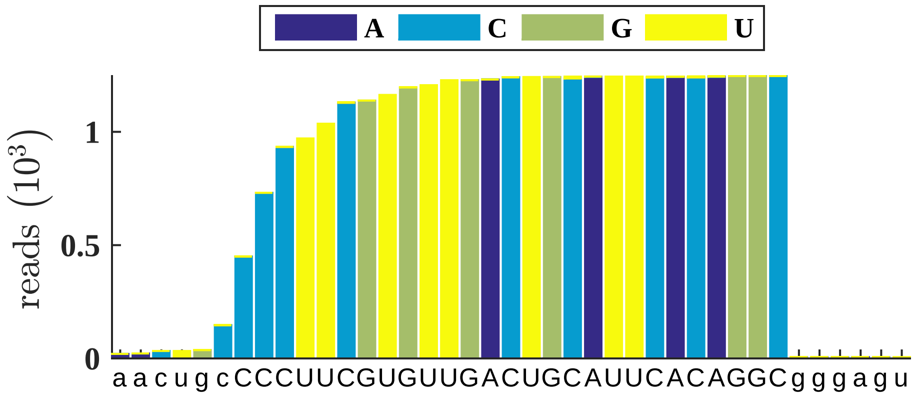

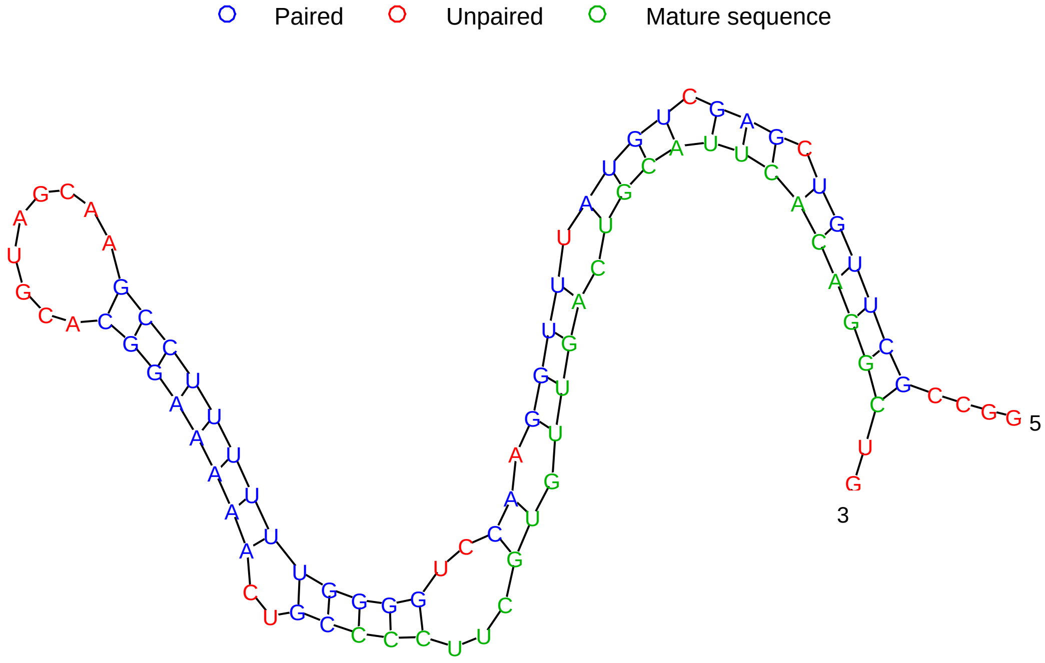

Stem loop (UMD3.1): chr25:30353005-30353096
Mature (UMD3.1): chr25:30353068-30353094
Mature seq len: 27
Total raw counts (9 samples): 1178
Average raw counts: 131
Strand: Forward
Orientation: 3p
Minimum free energy: -28.90

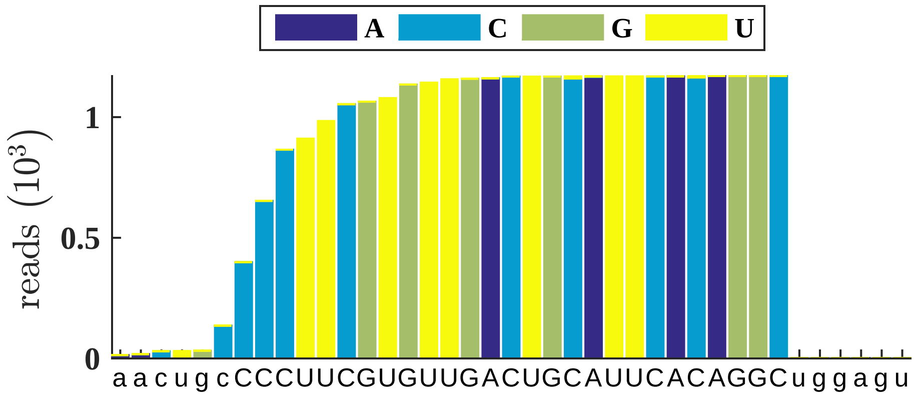

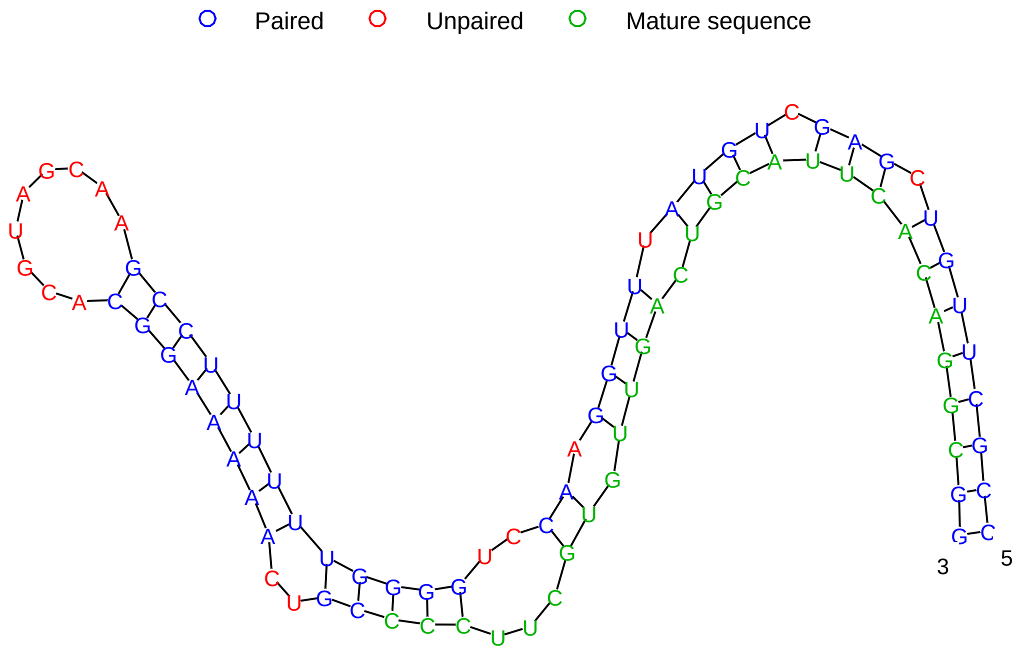

Stem loop (UMD3.1): chr25:30359279-30359368
Mature (UMD3.1): chr25:30359340-30359366
Mature seq len: 27
Total raw counts (9 samples): 1206
Average raw counts: 134
Strand: Forward
Orientation: 3p
Minimum free energy: -33.20

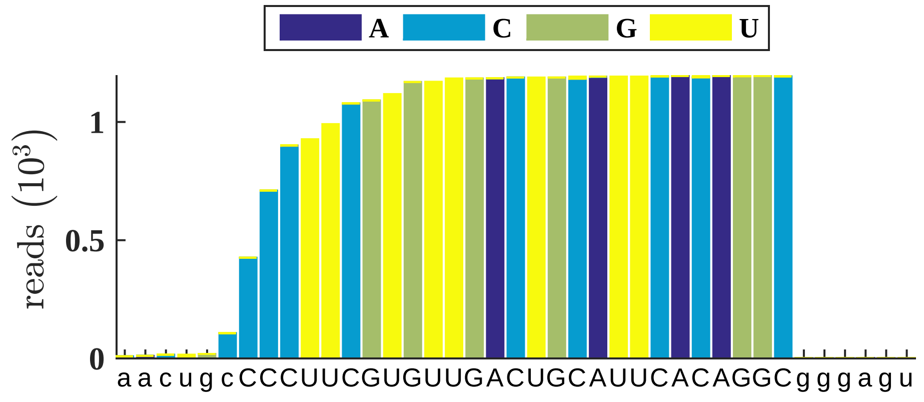

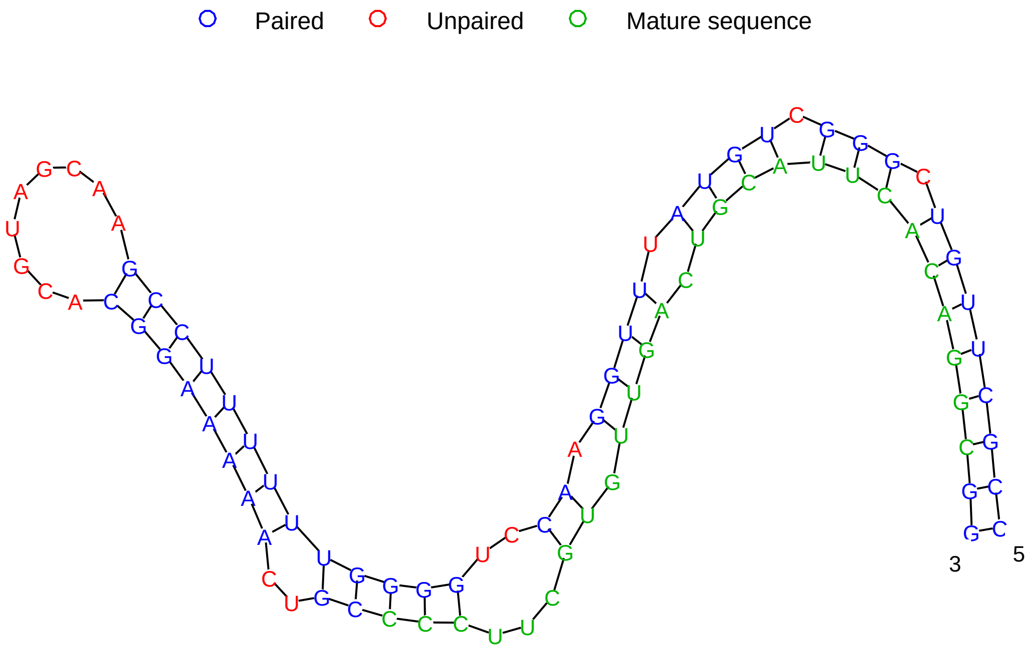

Stem loop (UMD3.1): chr25:30361704-30361793
Mature (UMD3.1): chr25:30361765-30361791
Mature seq len: 27
Total raw counts (9 samples): 1197
Average raw counts: 133
Strand: Forward
Orientation: 3p
Minimum free energy: -32.20

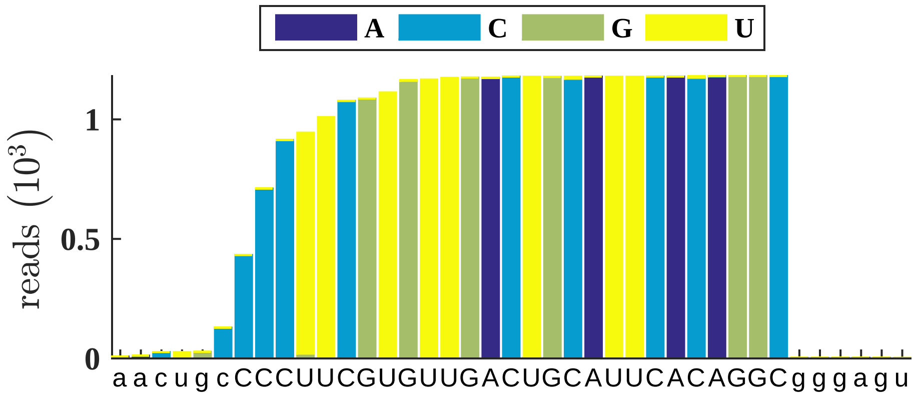

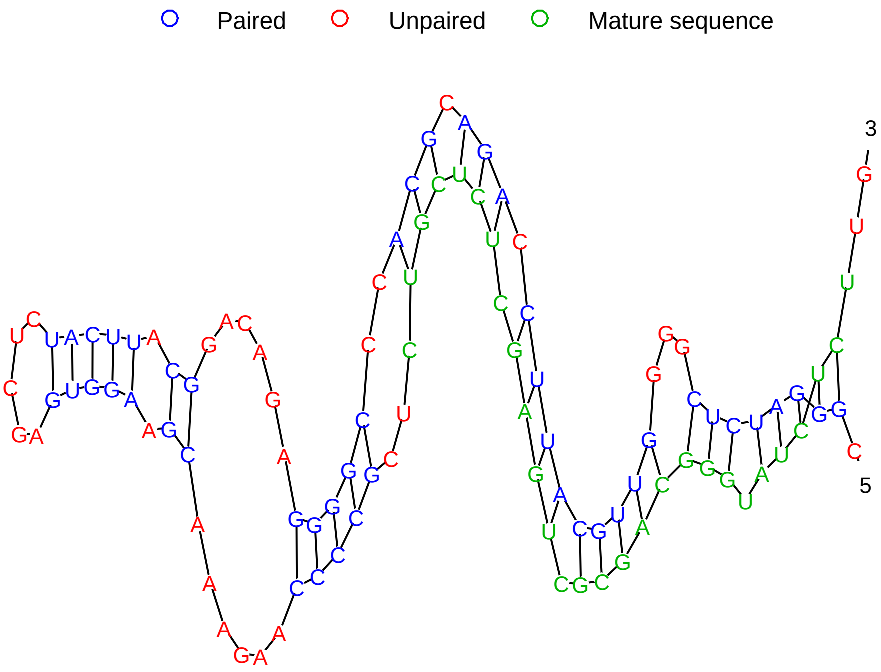

Stem loop (UMD3.1): chr25:30363904-30364009
Mature (UMD3.1): chr25:30363980-30364007
Mature seq len: 28
Total raw counts (9 samples): 12969
Average raw counts: 1441
Strand: Forward
Orientation: 3p
Minimum free energy: -35.70

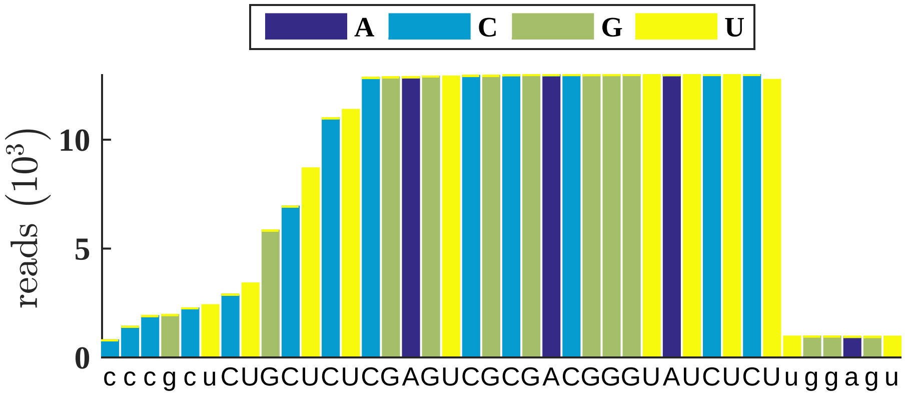

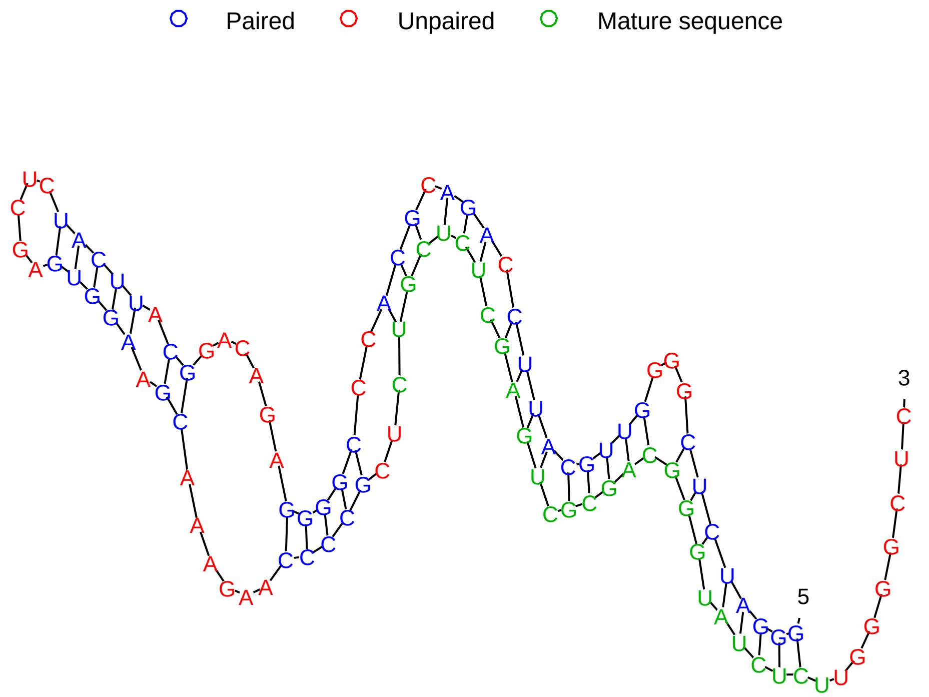

Stem loop (UMD3.1): chr25:35462008-35462118
Mature (UMD3.1): chr25:35462083-35462110
Mature seq len: 28
Total raw counts (9 samples): 12584
Average raw counts: 1399
Strand: Forward
Orientation: 3p
Minimum free energy: -35.50

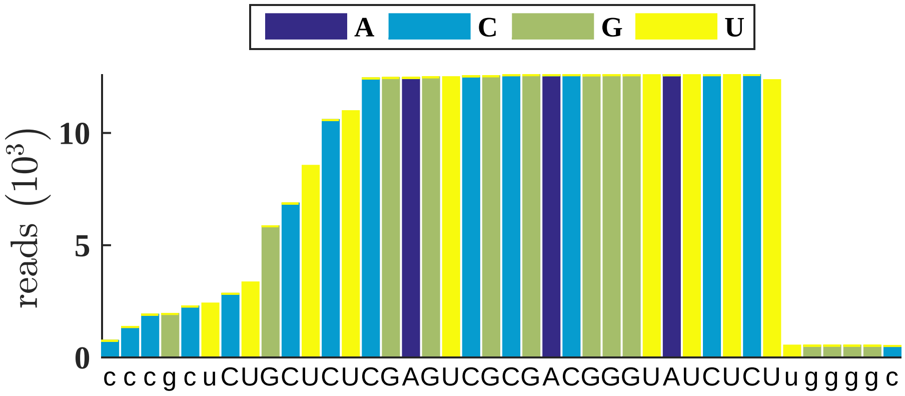

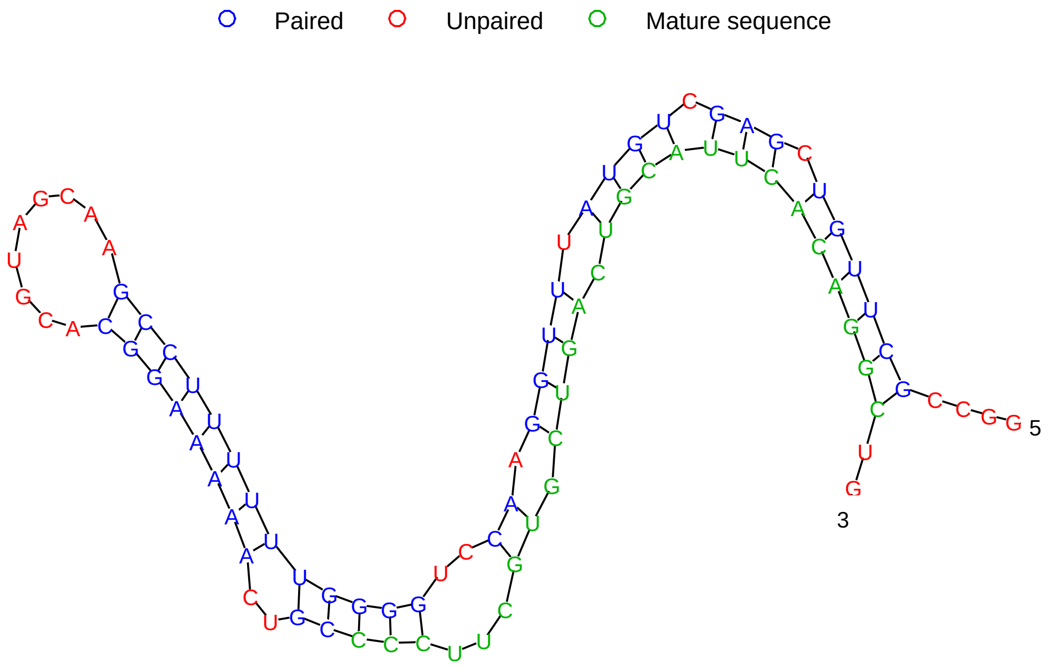

Stem loop (UMD3.1): chr25:4827222-4827313
Mature (UMD3.1): chr25:4827224-4827250
Mature seq len: 27
Total raw counts (9 samples): 1298
Average raw counts: 145
Strand: Reverse
Orientation: 3p
Minimum free energy: -31.20

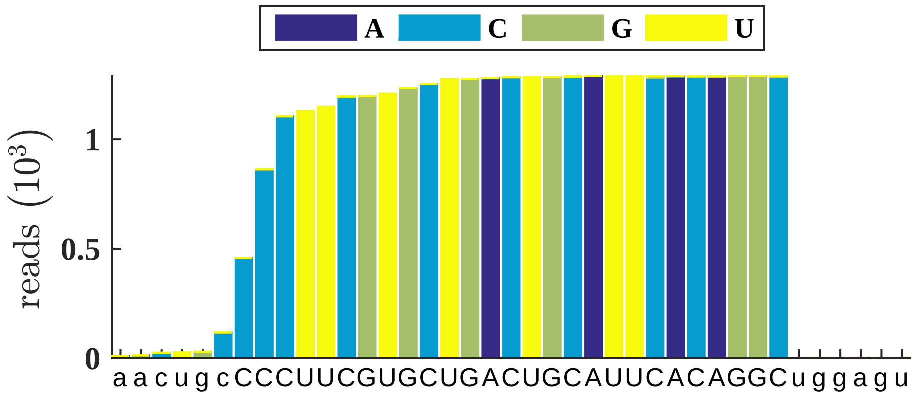

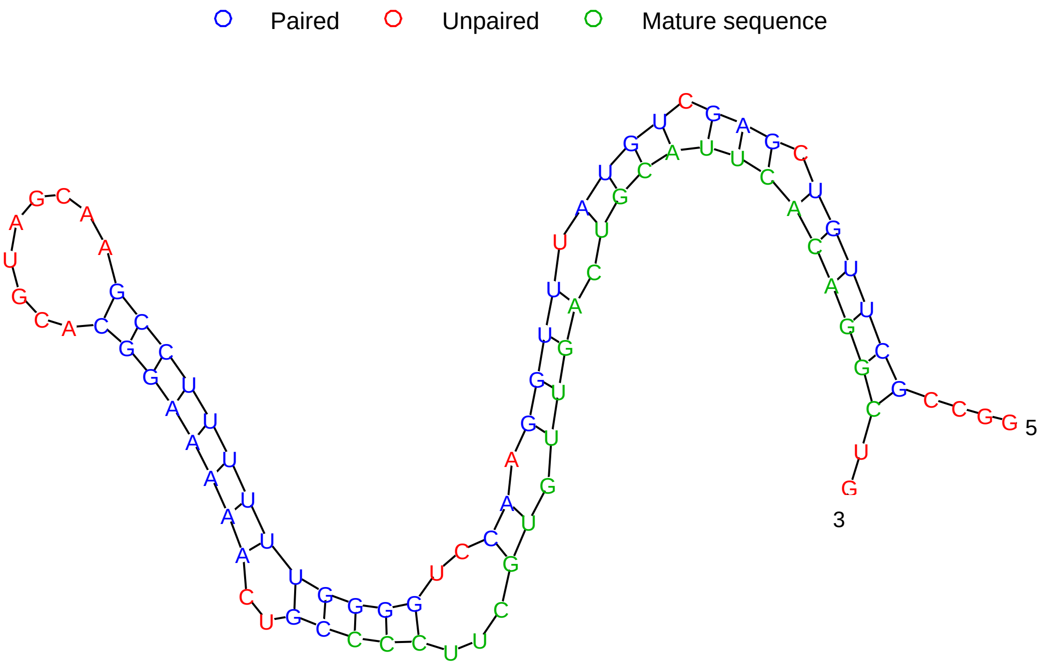

Stem loop (UMD3.1): chr25:4828932-4829023
Mature (UMD3.1): chr25:4828934-4828960
Mature seq len: 27
Total raw counts (9 samples): 1268
Average raw counts: 141
Strand: Reverse
Orientation: 3p
Minimum free energy: -28.90

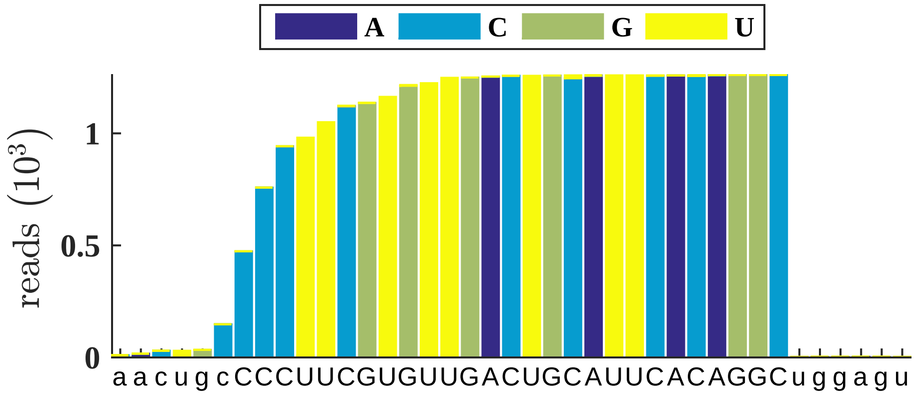

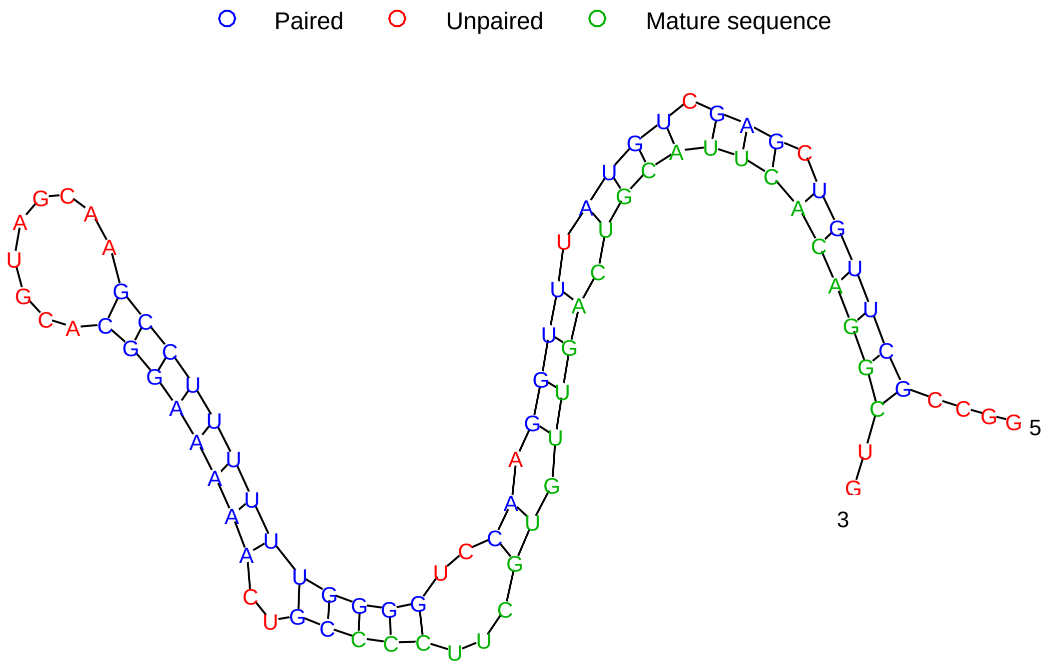

Stem loop (UMD3.1): chr25:4830372-4830463
Mature (UMD3.1): chr25:4830435-4830461
Mature seq len: 27
Total raw counts (9 samples): 1249
Average raw counts: 139
Strand: Forward
Orientation: 3p
Minimum free energy: -28.90

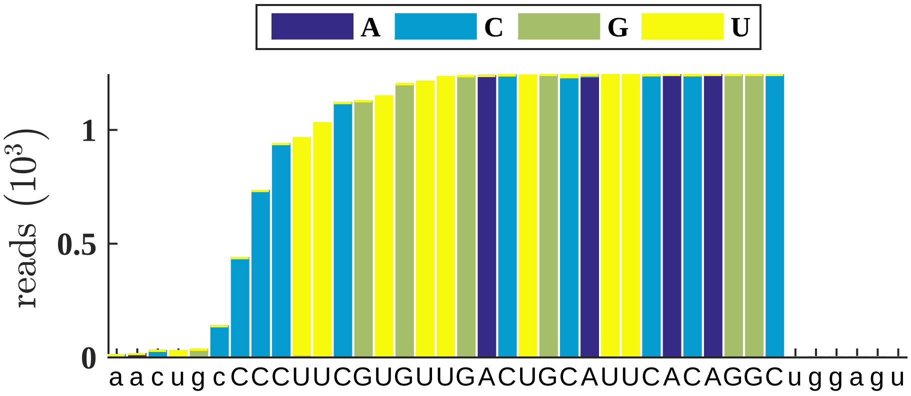

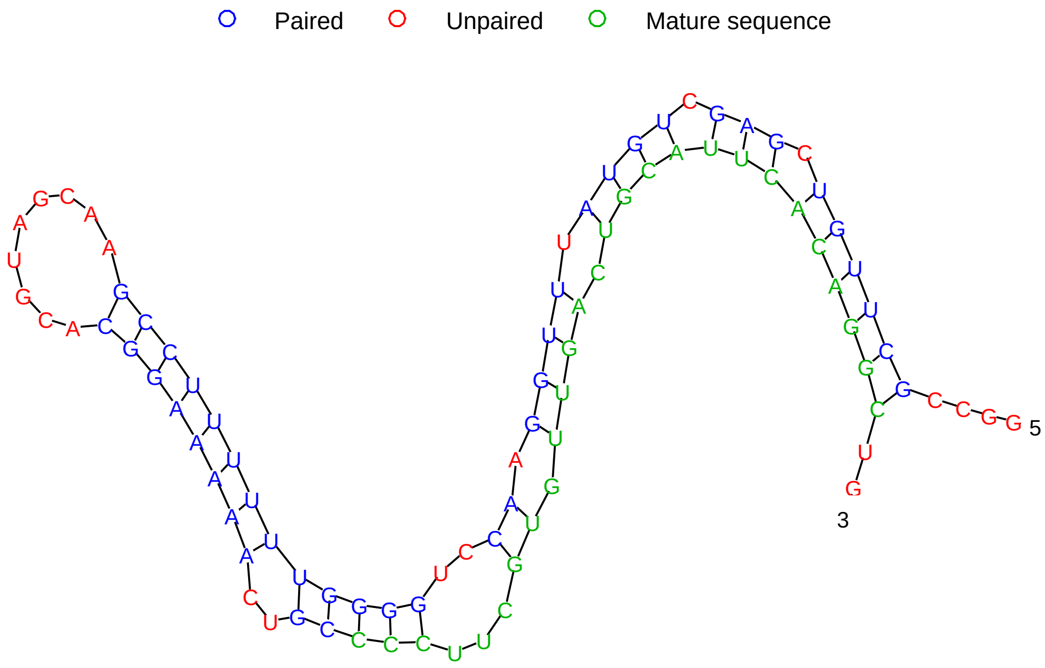

Stem loop (UMD3.1): chr25:4835189-4835280
Mature (UMD3.1): chr25:4835252-4835278
Mature seq len: 27
Total raw counts (9 samples): 1155
Average raw counts: 129
Strand: Forward
Orientation: 3p
Minimum free energy: -28.90

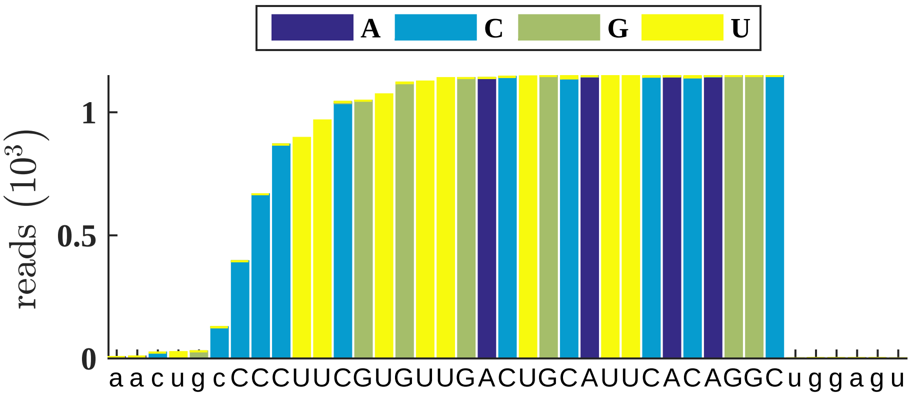

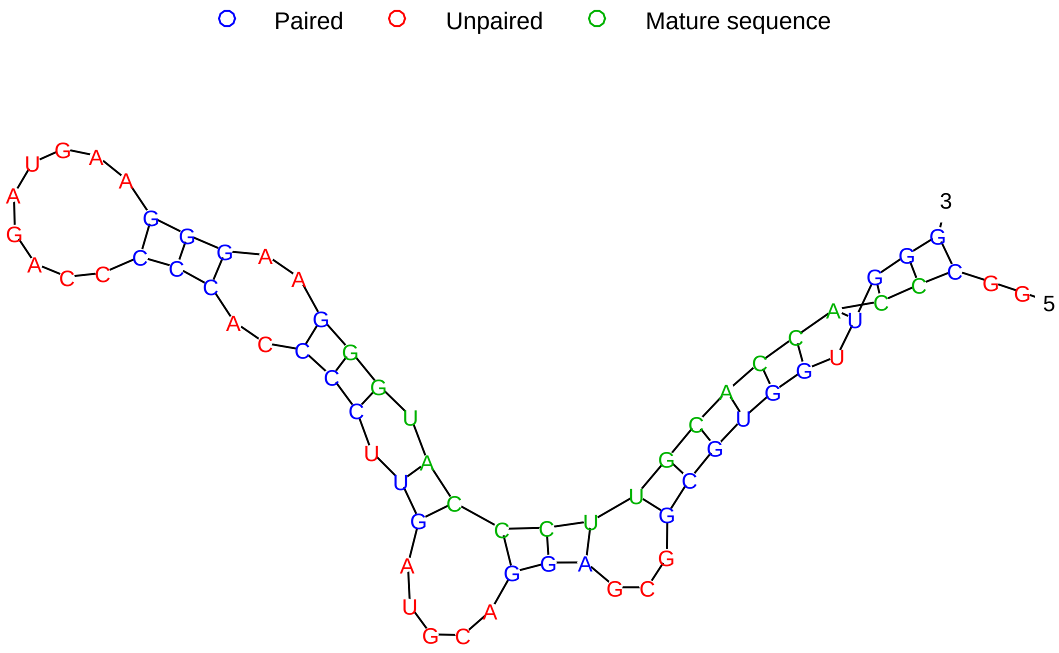

Stem loop (UMD3.1): chr26:33523677-33523744
Mature (UMD3.1): chr26:33523680-33523696
Mature seq len: 17
Total raw counts (9 samples): 91933
Average raw counts: 10215
Strand: Forward
Orientation: 5p
Minimum free energy: -25.90

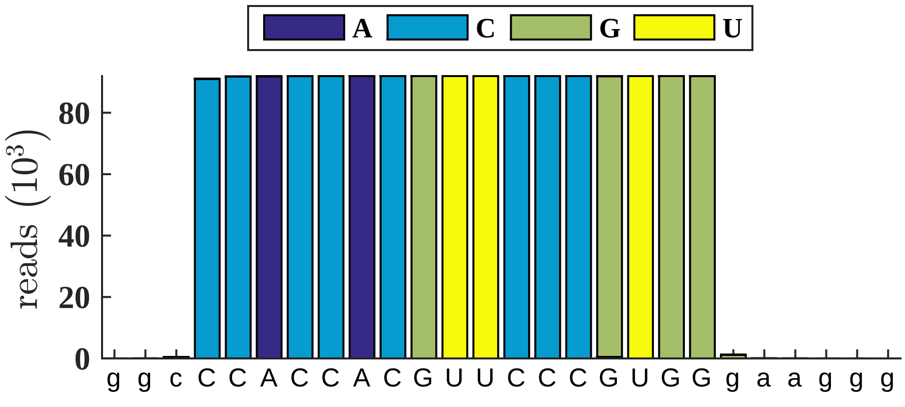

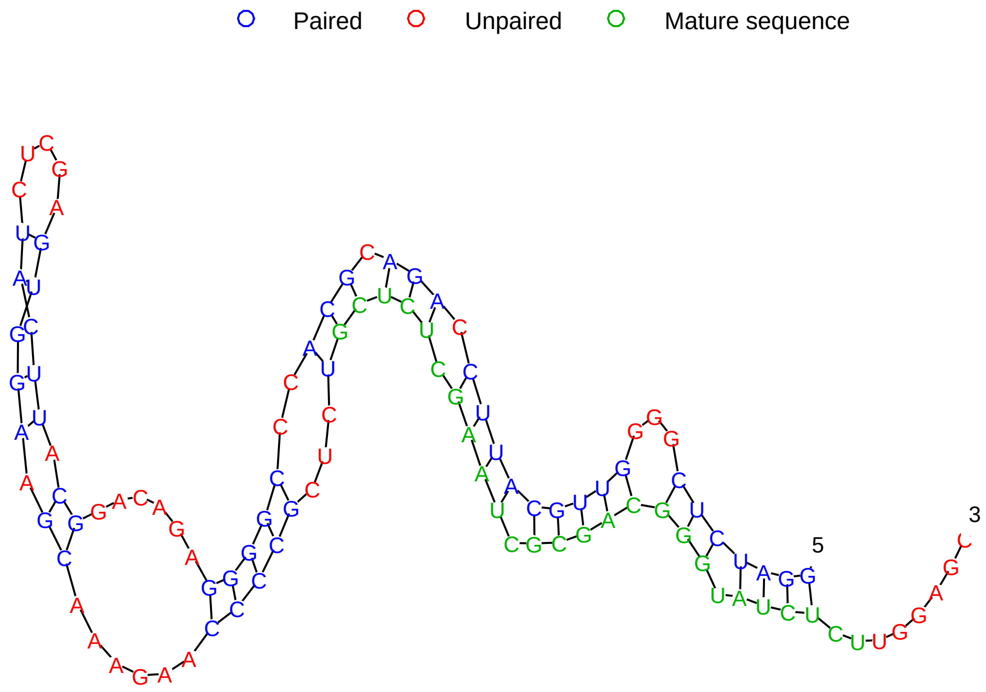

Stem loop (UMD3.1): chr26:40473345-40473452
Mature (UMD3.1): chr26:40473351-40473376
Mature seq len: 26
Total raw counts (9 samples): 3677
Average raw counts: 409
Strand: Reverse
Orientation: 3p
Minimum free energy: -32.40

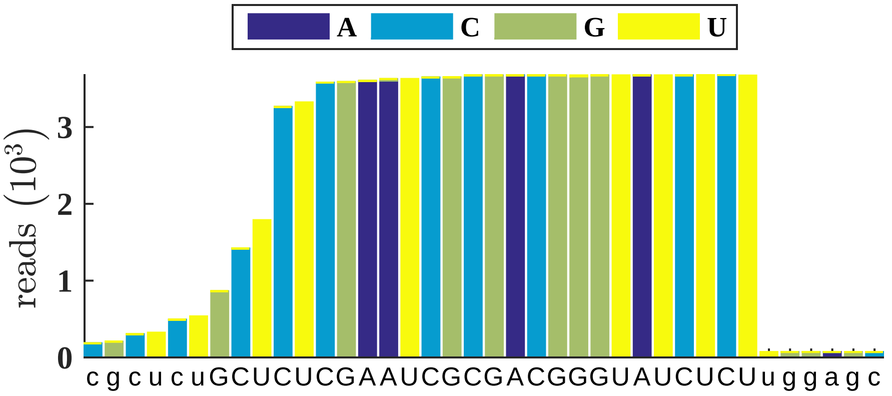

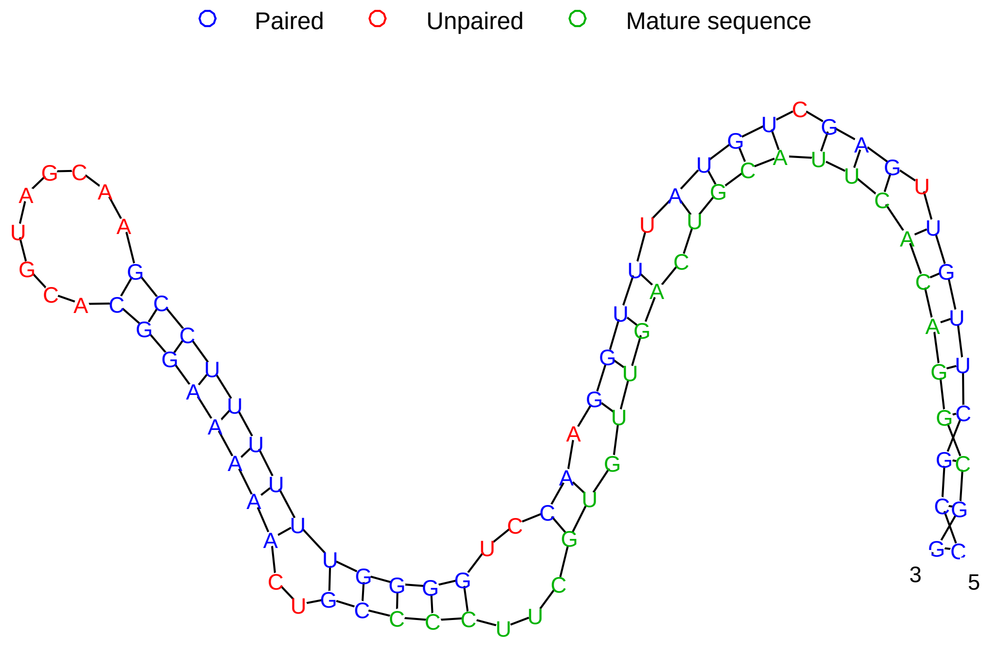

Stem loop (UMD3.1): chr26:40486989-40487078
Mature (UMD3.1): chr26:40486991-40487017
Mature seq len: 27
Total raw counts (9 samples): 1201
Average raw counts: 134
Strand: Reverse
Orientation: 3p
Minimum free energy: -33.20

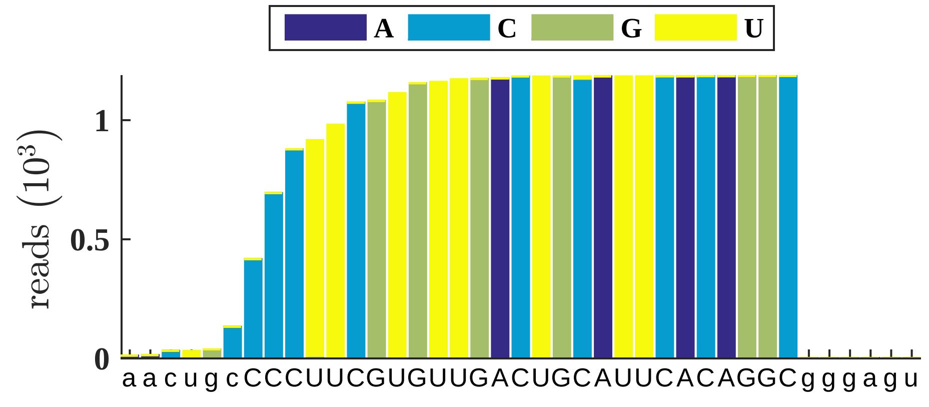

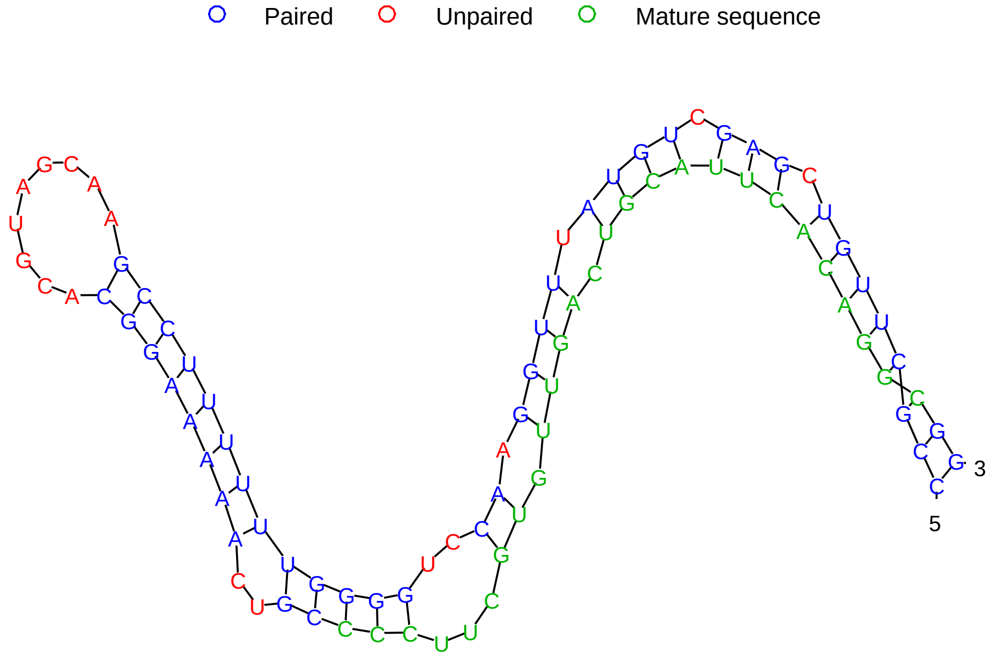

Stem loop (UMD3.1): chr26:40629415-40629504
Mature (UMD3.1): chr26:40629476-40629502
Mature seq len: 27
Total raw counts (9 samples): 1133
Average raw counts: 126
Strand: Forward
Orientation: 3p
Minimum free energy: -33.20

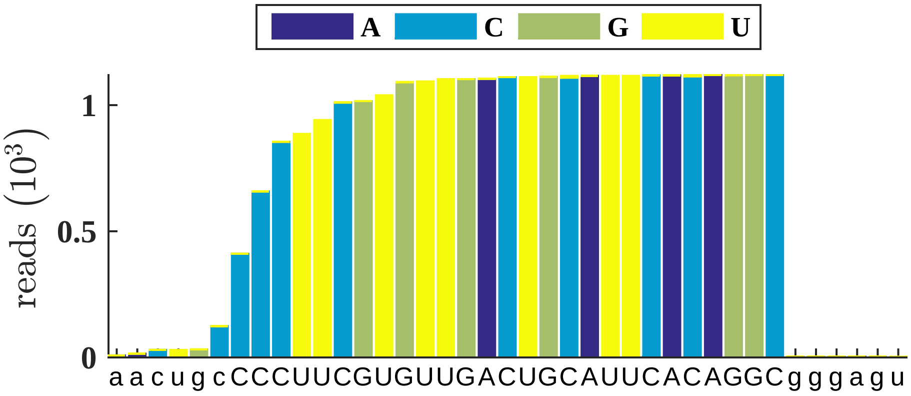

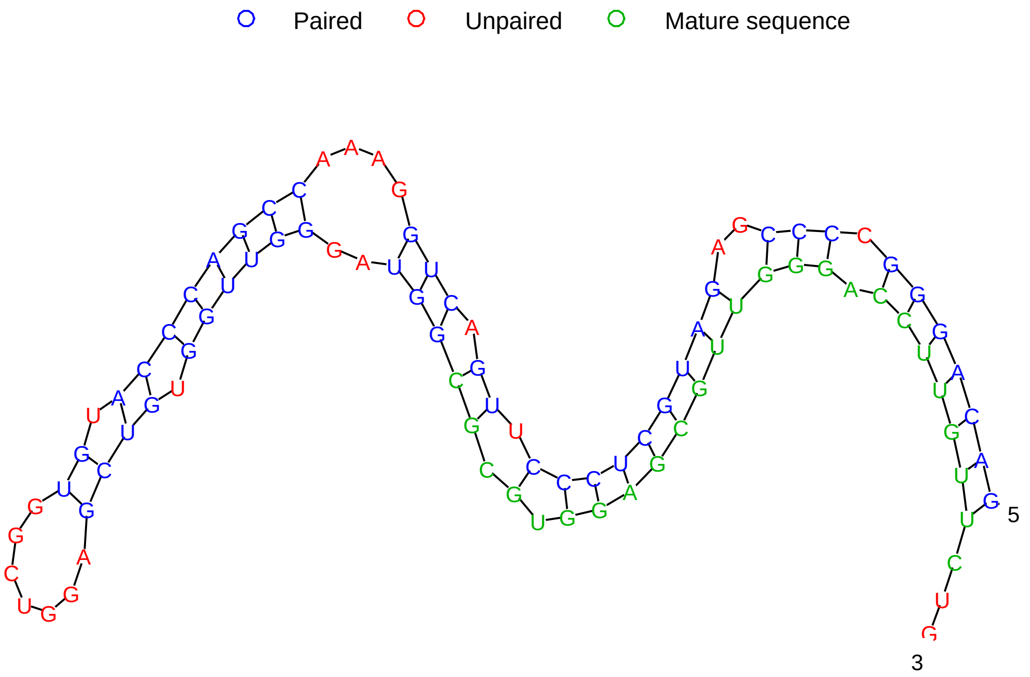

Stem loop (UMD3.1): chr26:44605911-44606004
Mature (UMD3.1): chr26:44605913-44605937
Mature seq len: 25
Total raw counts (9 samples): 736
Average raw counts: 82
Strand: Reverse
Orientation: 3p
Minimum free energy: -37.50

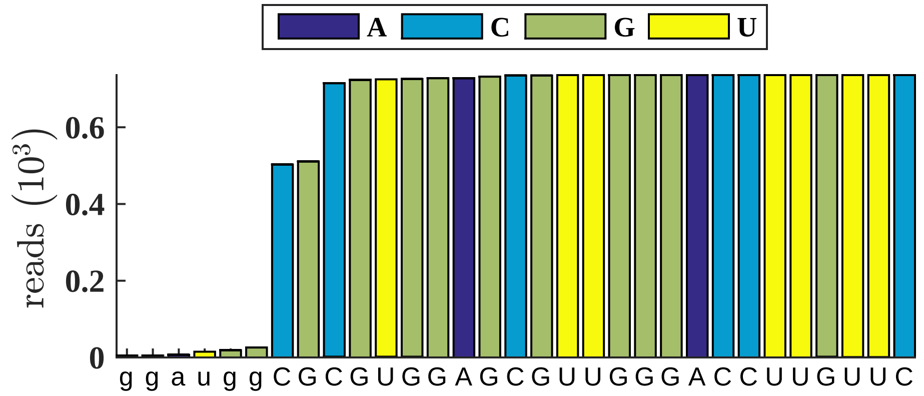

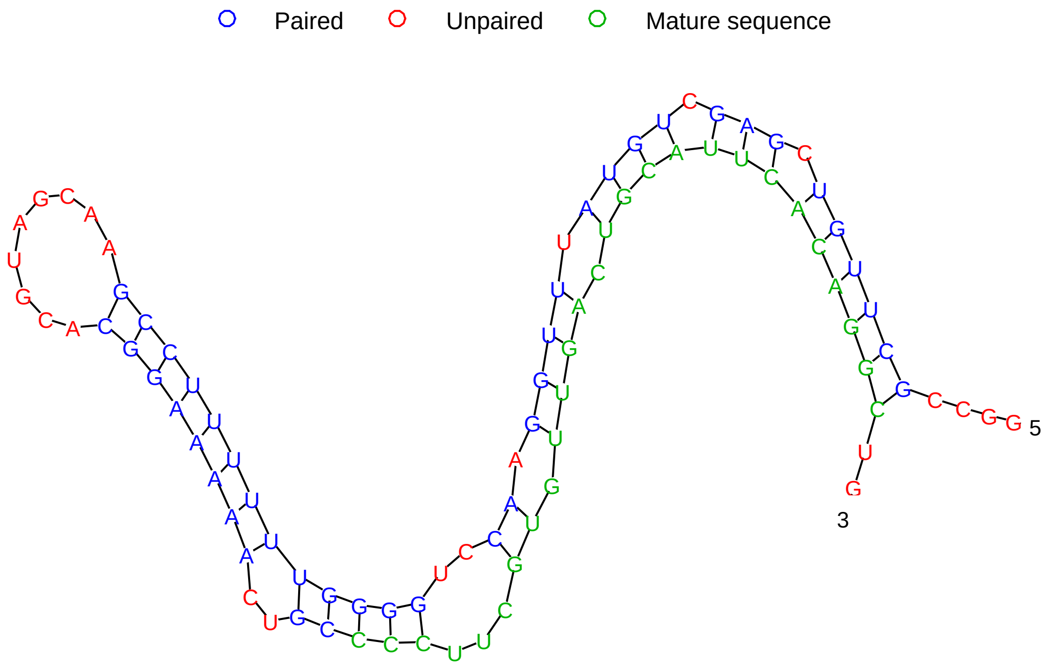

Stem loop (UMD3.1): chr26:51203774-51203865
Mature (UMD3.1): chr26:51203776-51203802
Mature seq len: 27
Total raw counts (9 samples): 1225
Average raw counts: 137
Strand: Reverse
Orientation: 3p
Minimum free energy: -28.90

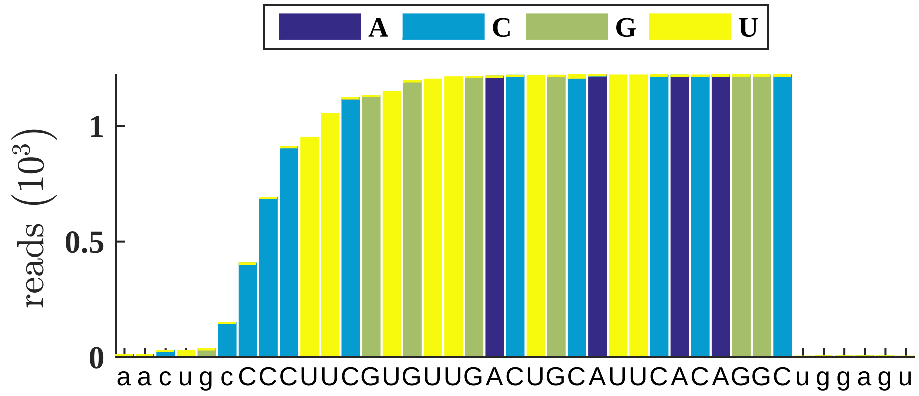

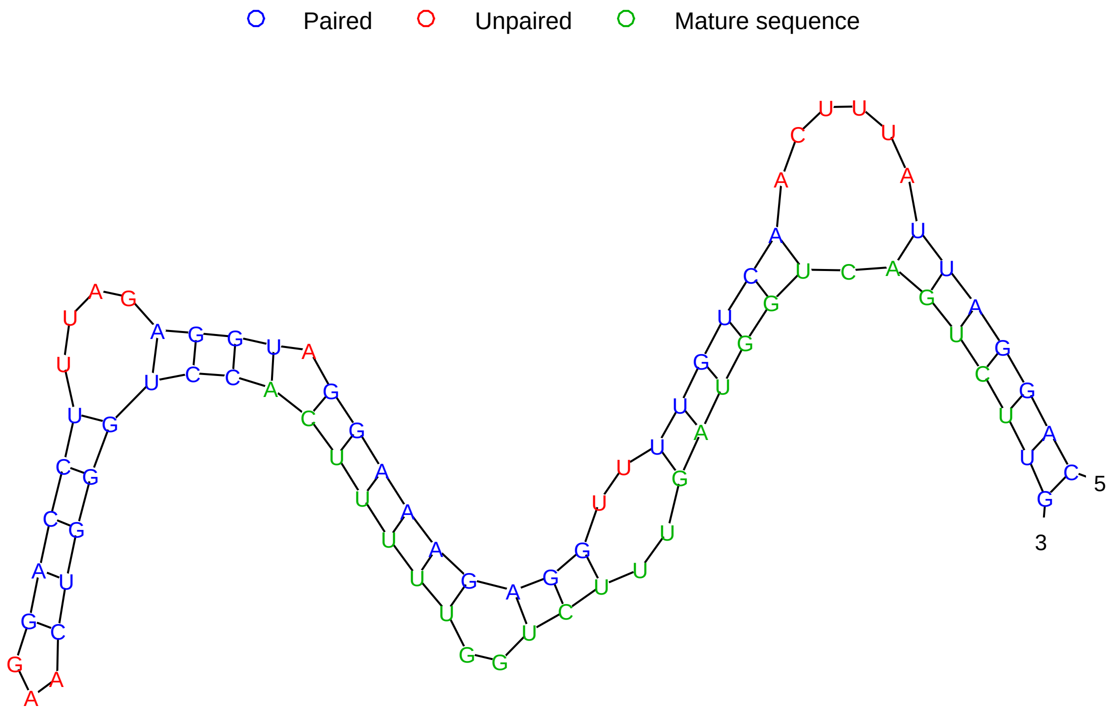

Stem loop (UMD3.1): chr27:17494808-17494890
Mature (UMD3.1): chr27:17494810-17494835
Mature seq len: 26
Total raw counts (9 samples): 5620
Average raw counts: 625
Strand: Reverse
Orientation: 3p
Minimum free energy: -18.30

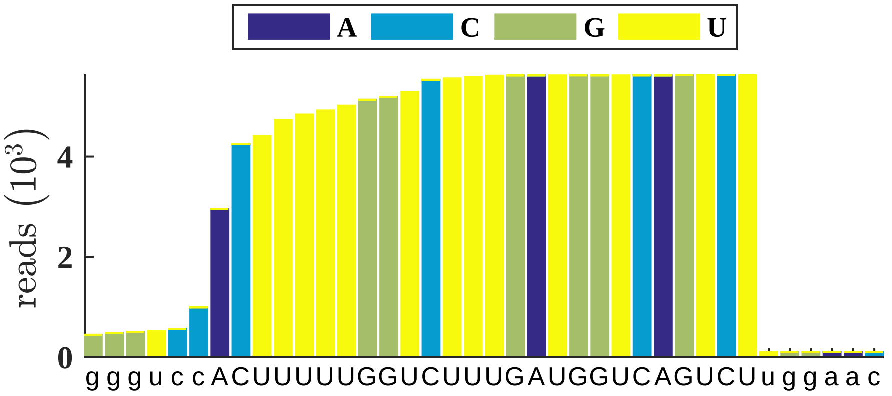

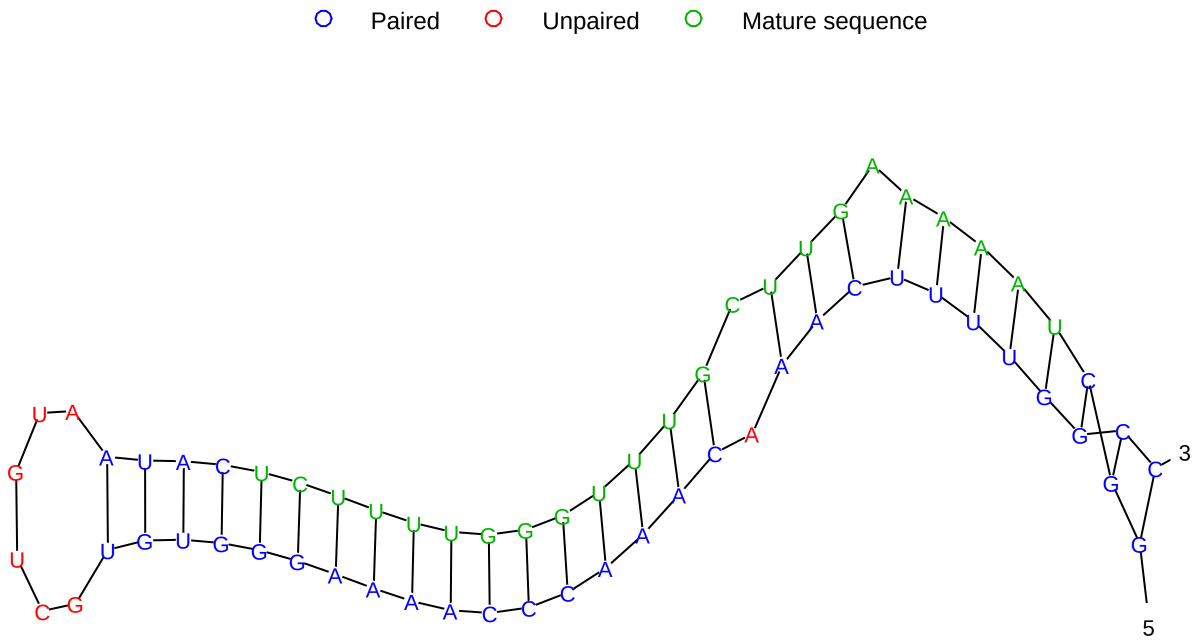

Stem loop (UMD3.1): chr27:27255986-27256050
Mature (UMD3.1): chr27:27255989-27256011
Mature seq len: 23
Total raw counts (9 samples): 790
Average raw counts: 88
Strand: Forward
Orientation: 5p
Minimum free energy: -35.70

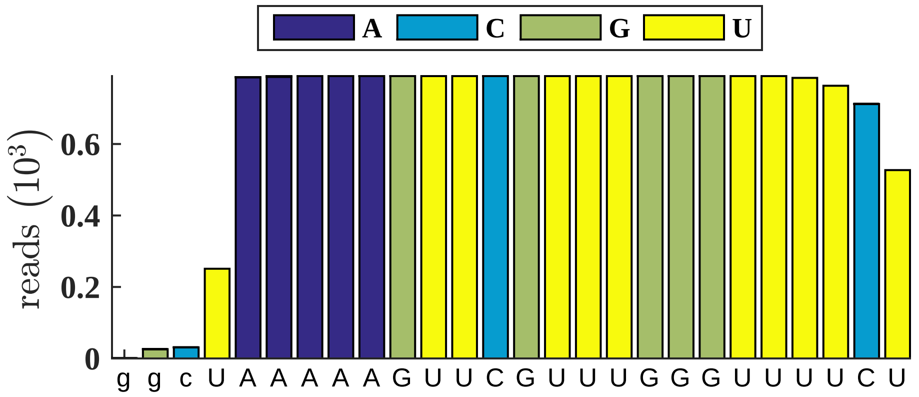

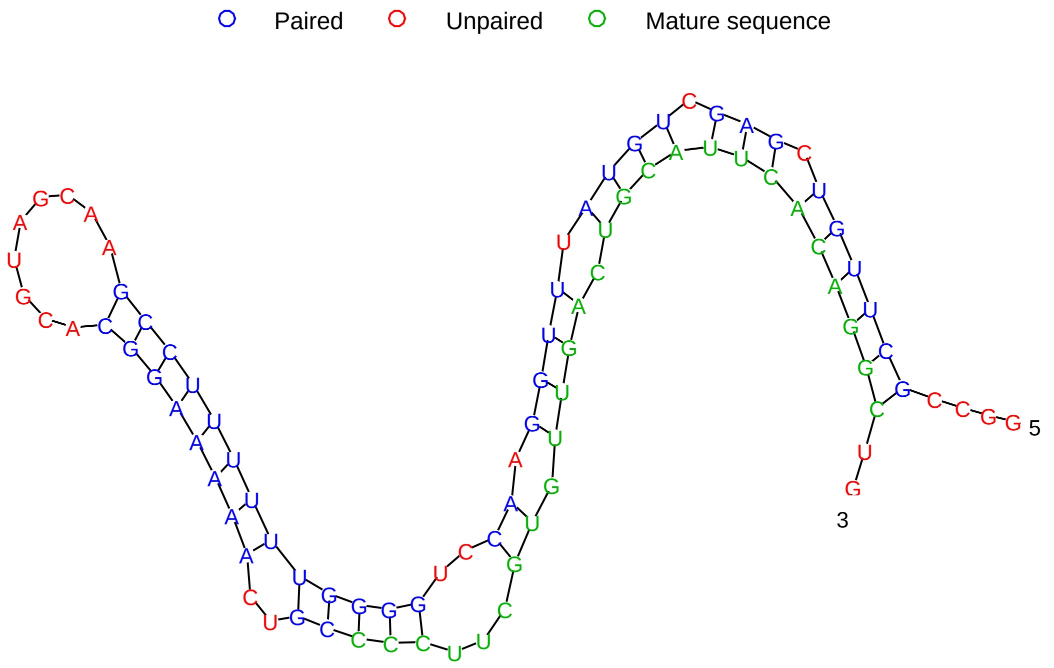

Stem loop (UMD3.1): chr27:35839097-35839188
Mature (UMD3.1): chr27:35839099-35839125
Mature seq len: 27
Total raw counts (9 samples): 1228
Average raw counts: 137
Strand: Reverse
Orientation: 3p
Minimum free energy: -28.90

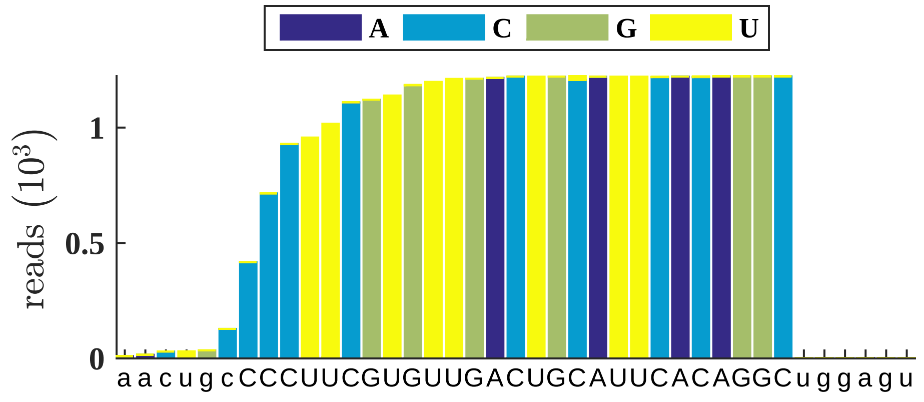

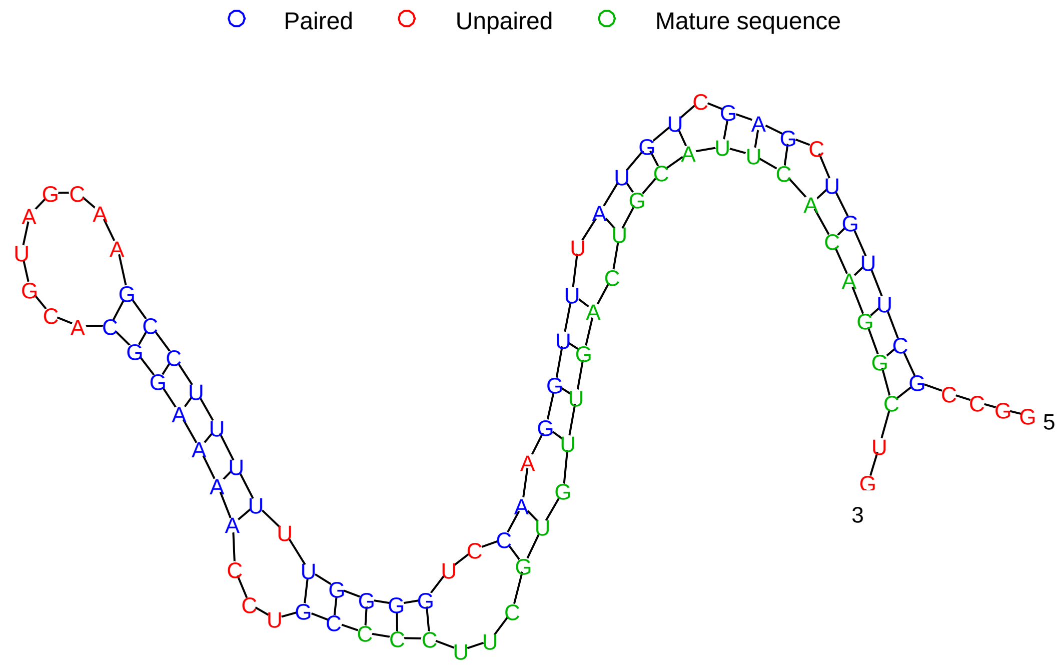

Stem loop (UMD3.1): chr27:35842144-35842235
Mature (UMD3.1): chr27:35842146-35842172
Mature seq len: 27
Total raw counts (9 samples): 1239
Average raw counts: 138
Strand: Reverse
Orientation: 3p
Minimum free energy: -28.10

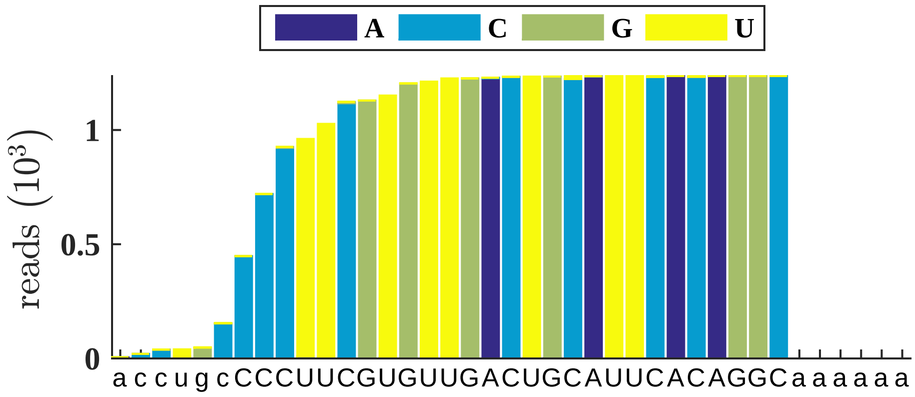

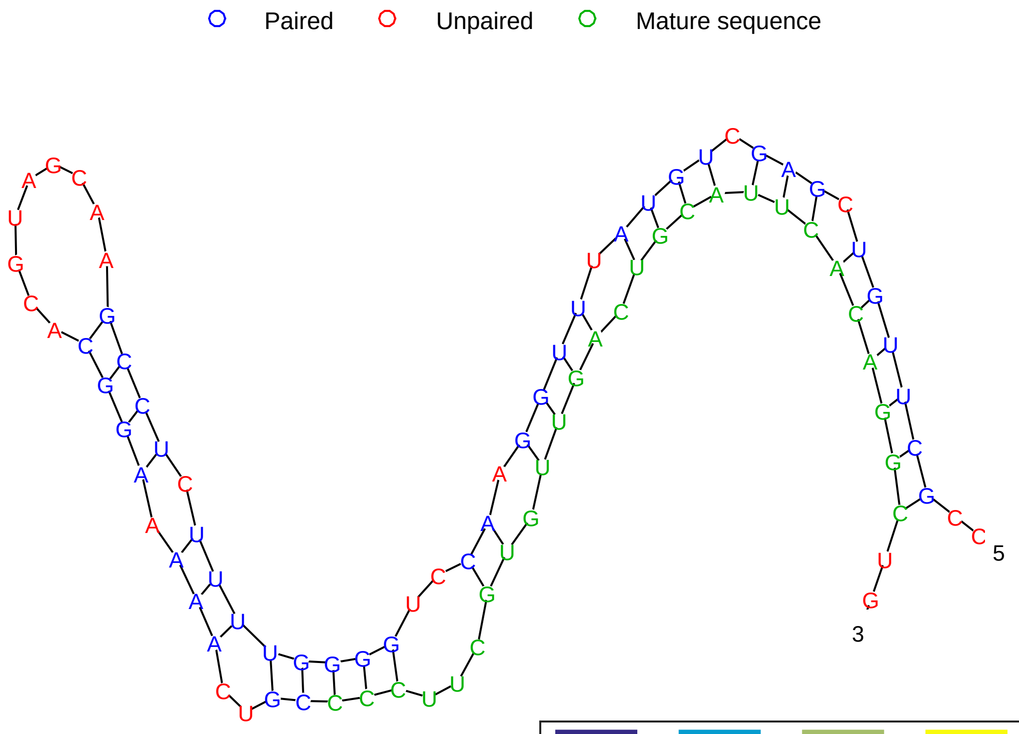

Stem loop (UMD3.1): chr27:35847719-35847808
Mature (UMD3.1): chr27:35847780-35847806
Mature seq len: 27
Total raw counts (9 samples): 1200
Average raw counts: 134
Strand: Forward
Orientation: 3p
Minimum free energy: -25.20

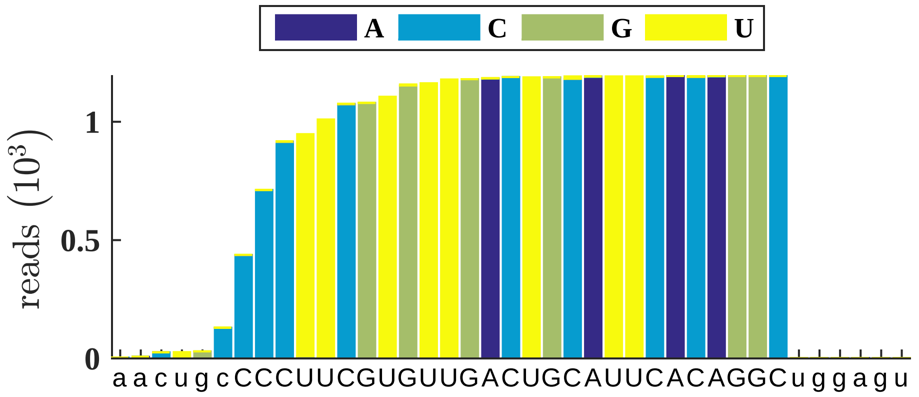

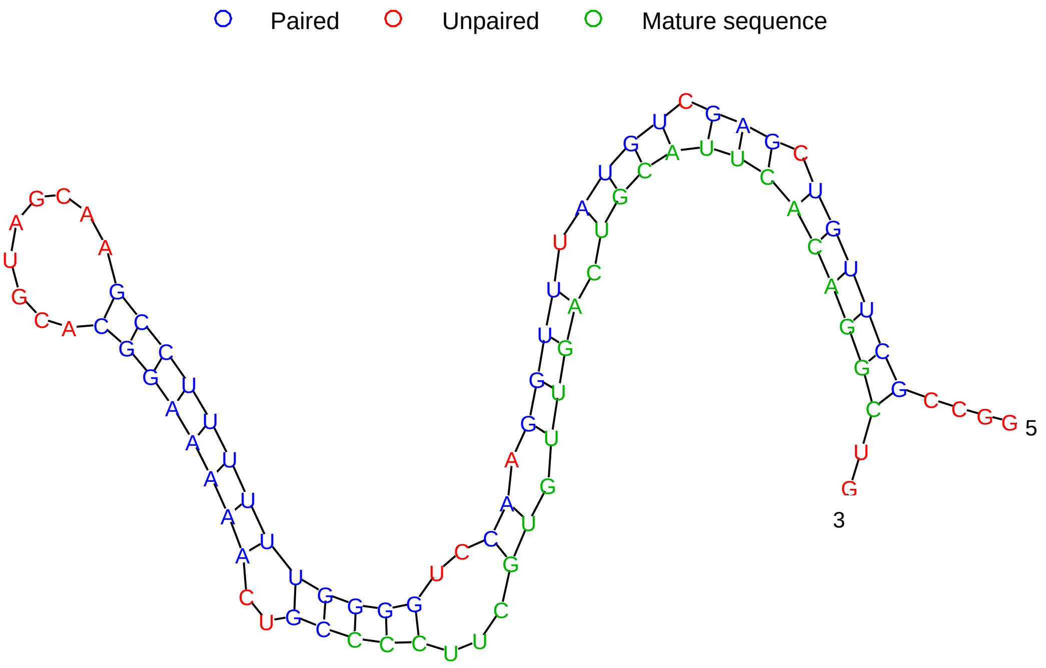

Stem loop (UMD3.1): chr27:37445497-37445588
Mature (UMD3.1): chr27:37445560-37445586
Mature seq len: 27
Total raw counts (9 samples): 1210
Average raw counts: 135
Strand: Forward
Orientation: 3p
Minimum free energy: -28.90

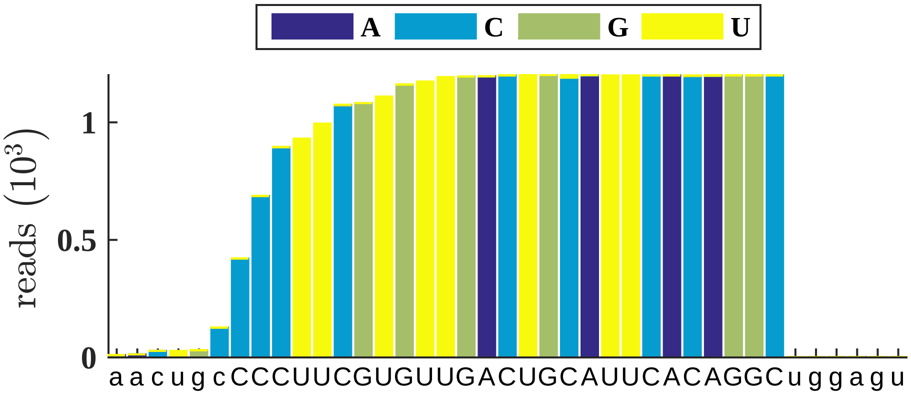

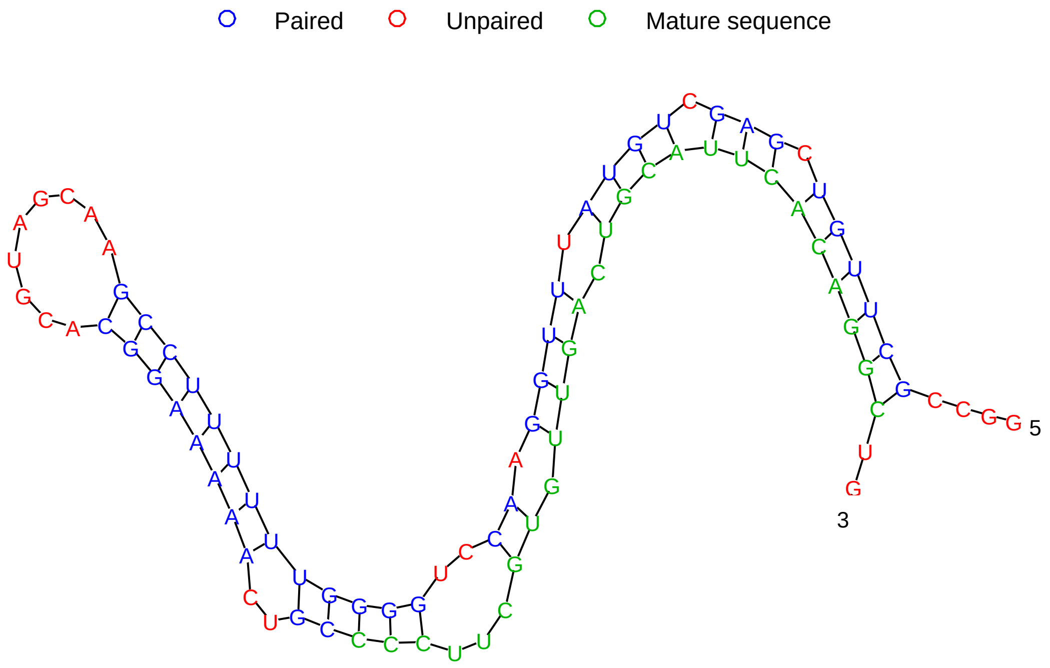

Stem loop (UMD3.1): chr27:37558888-37558979
Mature (UMD3.1): chr27:37558890-37558916
Mature seq len: 27
Total raw counts (9 samples): 1180
Average raw counts: 132
Strand: Reverse
Orientation: 3p
Minimum free energy: -28.90

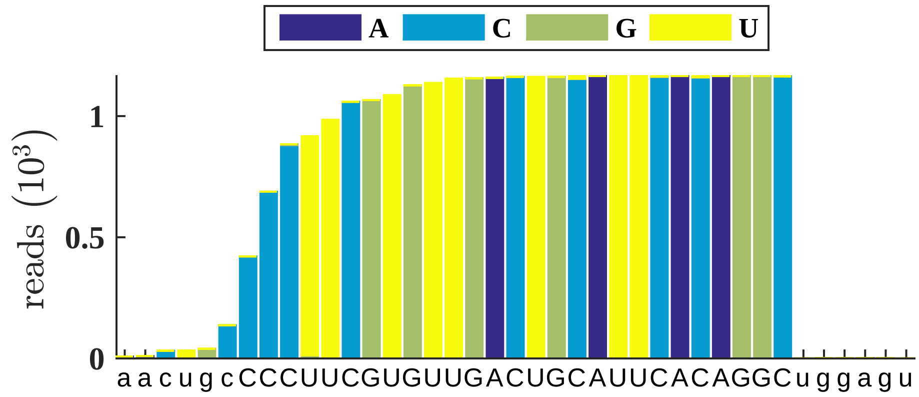

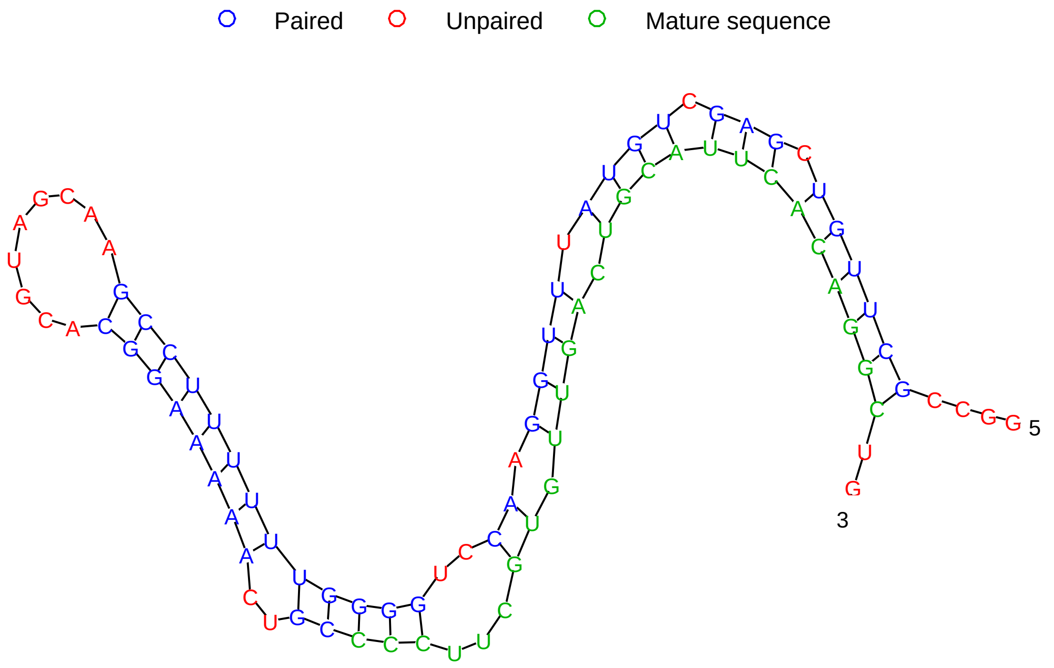

Stem loop (UMD3.1): chr27:37574421-37574512
Mature (UMD3.1): chr27:37574484-37574510
Mature seq len: 27
Total raw counts (9 samples): 1204
Average raw counts: 134
Strand: Forward
Orientation: 3p
Minimum free energy: -28.90

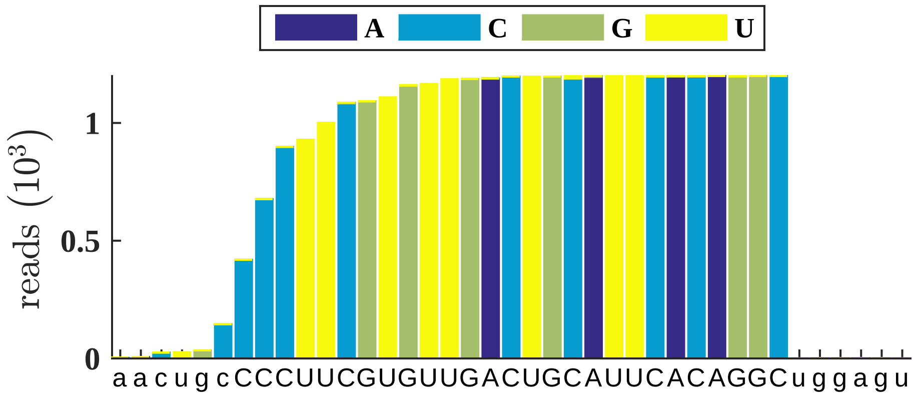

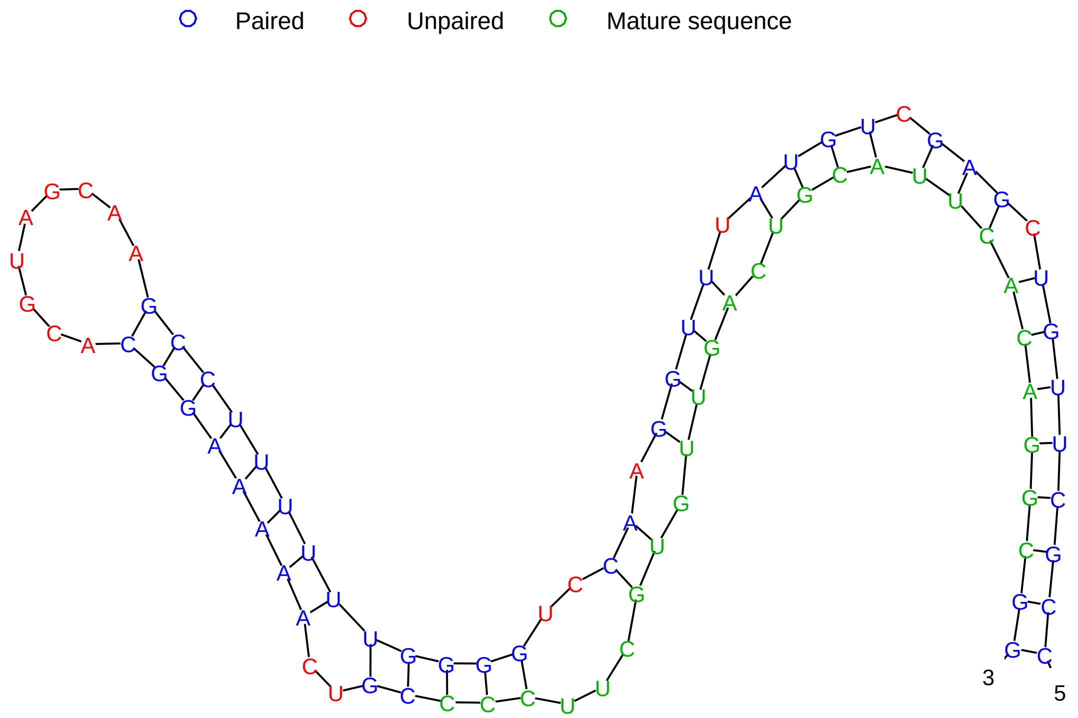

Stem loop (UMD3.1): chr27:37581483-37581572
Mature (UMD3.1): chr27:37581485-37581511
Mature seq len: 27
Total raw counts (9 samples): 1212
Average raw counts: 135
Strand: Reverse
Orientation: 3p
Minimum free energy: -33.20

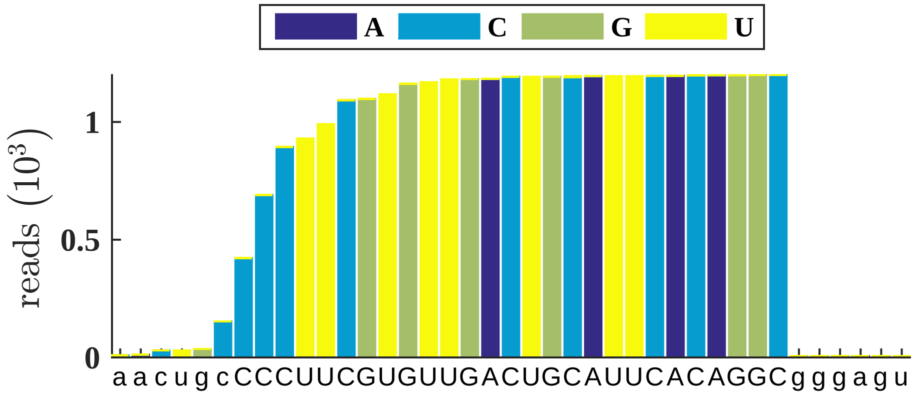

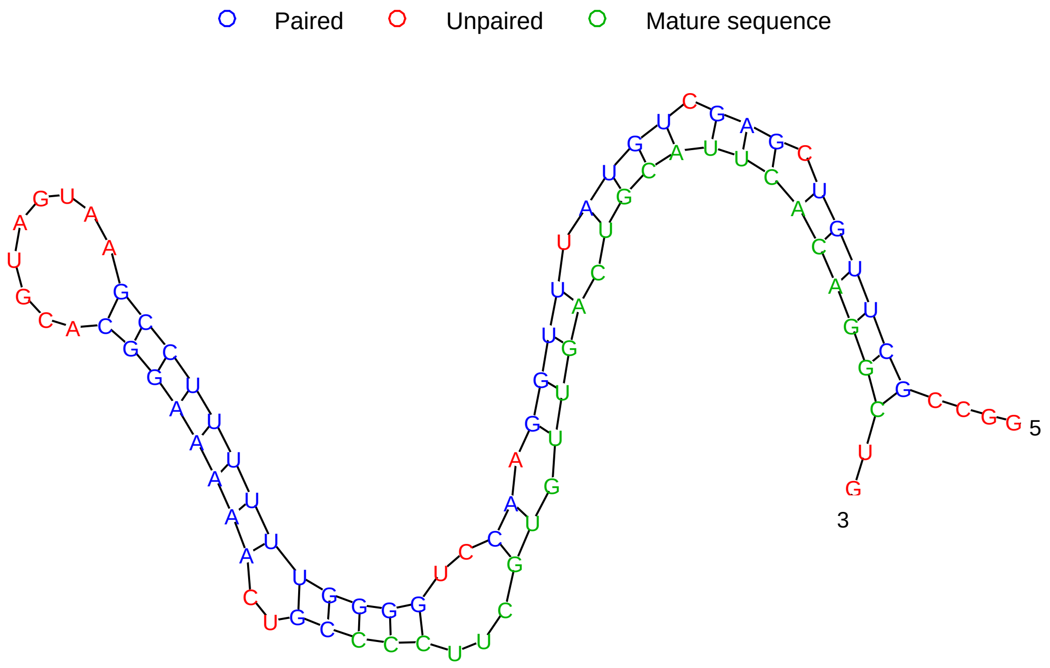

Stem loop (UMD3.1): chr27:37583493-37583584
Mature (UMD3.1): chr27:37583556-37583582
Mature seq len: 27
Total raw counts (9 samples): 1202
Average raw counts: 134
Strand: Forward
Orientation: 3p
Minimum free energy: -28.90

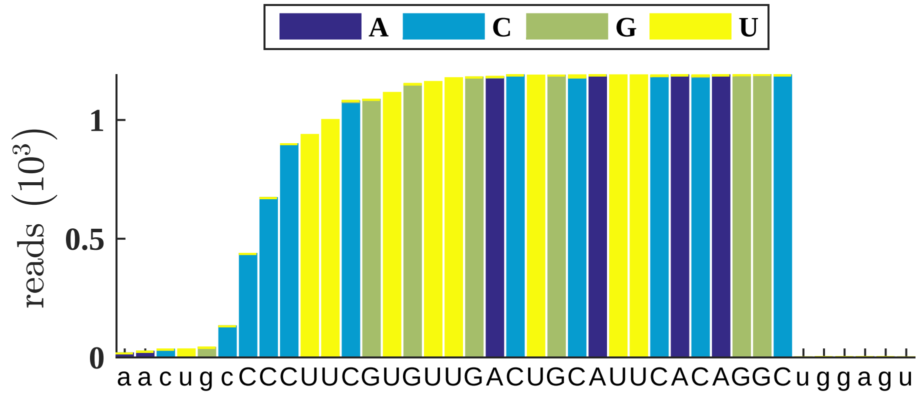

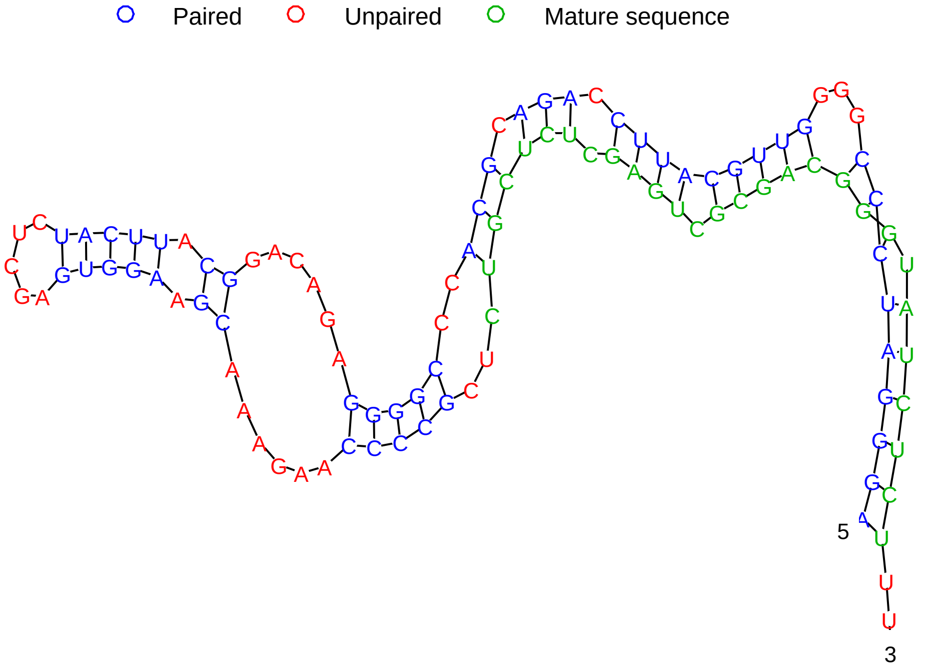

Stem loop (UMD3.1): chr27:37585459-37585564
Mature (UMD3.1): chr27:37585461-37585488
Mature seq len: 28
Total raw counts (9 samples): 12456
Average raw counts: 1384
Strand: Reverse
Orientation: 3p
Minimum free energy: -39.00

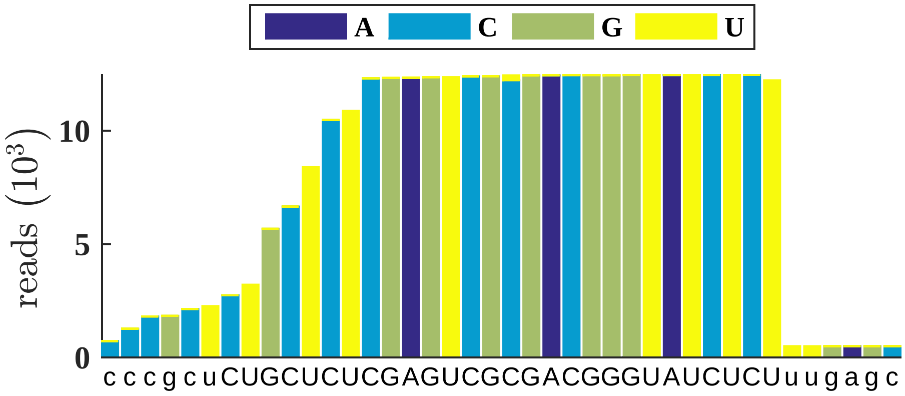

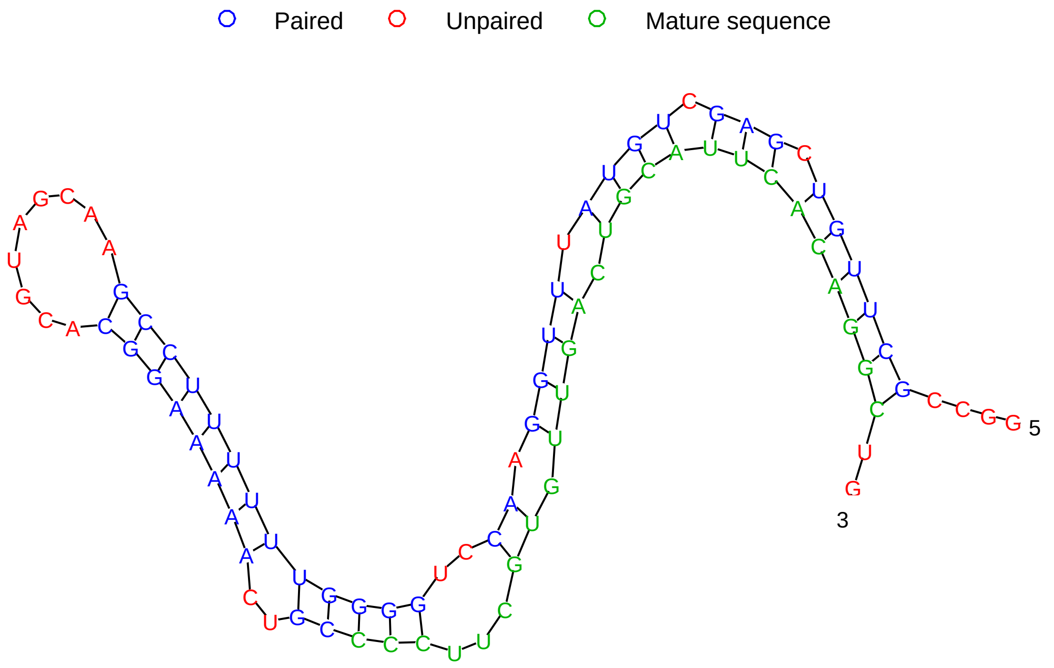

Stem loop (UMD3.1): chr27:37589999-37590090
Mature (UMD3.1): chr27:37590001-37590027
Mature seq len: 27
Total raw counts (9 samples): 1188
Average raw counts: 132
Strand: Reverse
Orientation: 3p
Minimum free energy: -28.90

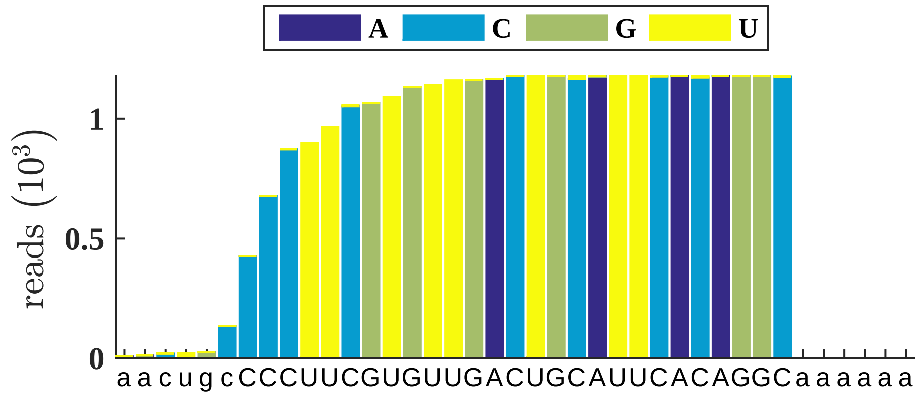

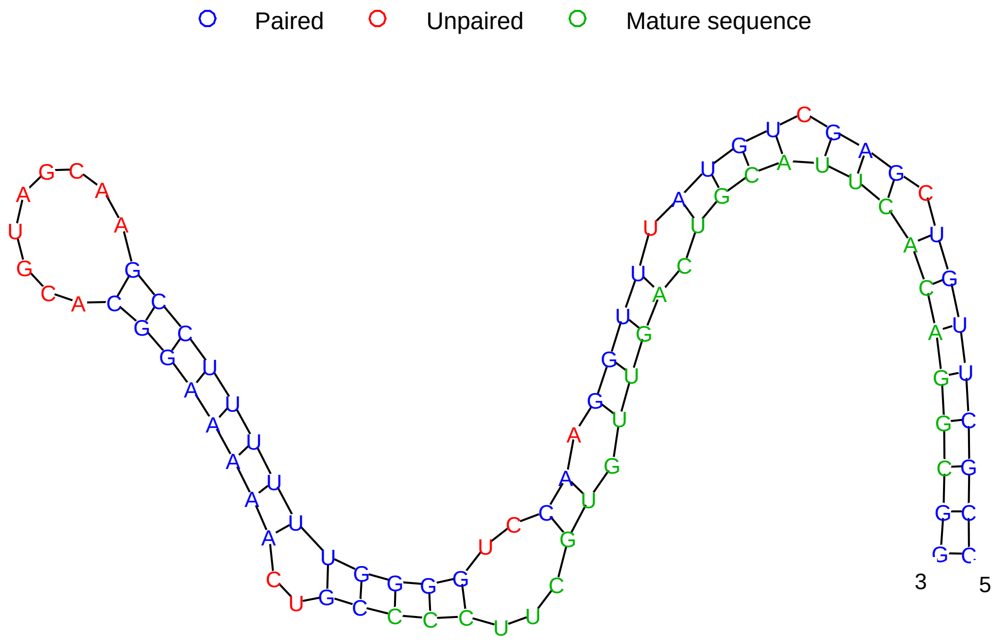

Stem loop (UMD3.1): chr27:37593784-37593873
Mature (UMD3.1): chr27:37593786-37593812
Mature seq len: 27
Total raw counts (9 samples): 1200
Average raw counts: 134
Strand: Reverse
Orientation: 3p
Minimum free energy: -33.20

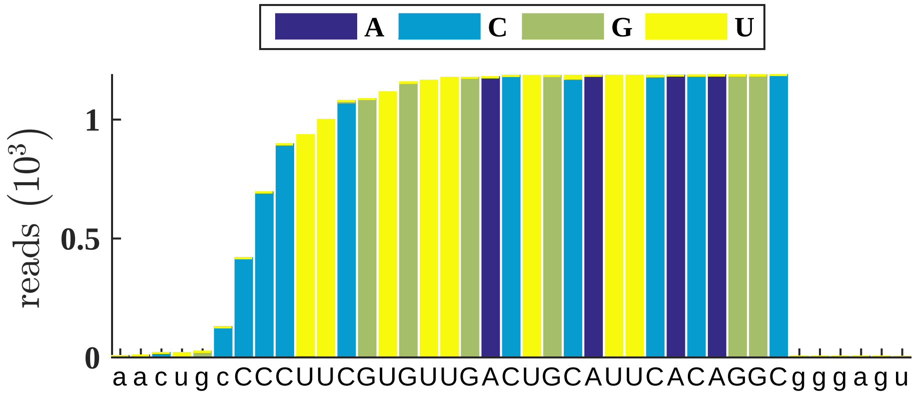

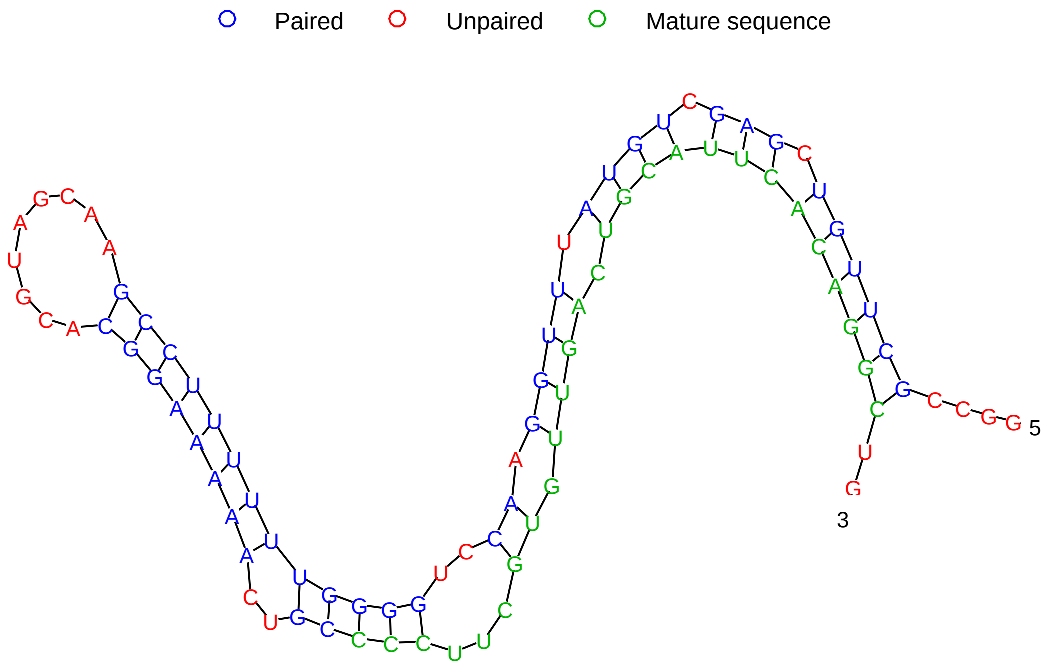

Stem loop (UMD3.1): chr27:37595632-37595723
Mature (UMD3.1): chr27:37595634-37595660
Mature seq len: 27
Total raw counts (9 samples): 1173
Average raw counts: 131
Strand: Reverse
Orientation: 3p
Minimum free energy: -28.90

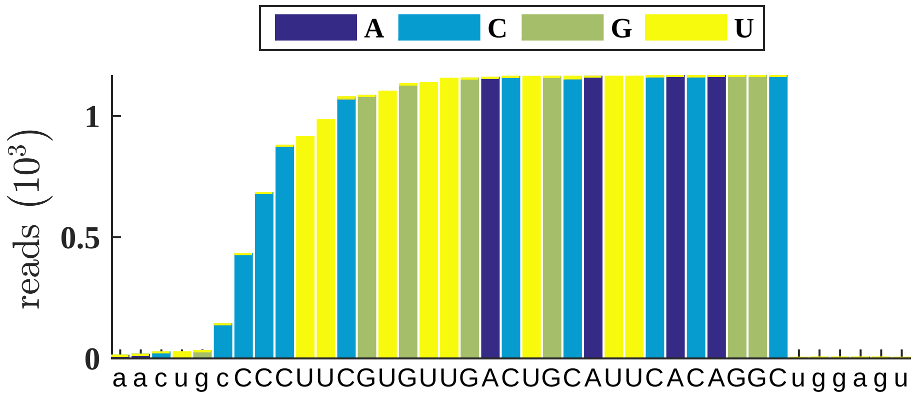

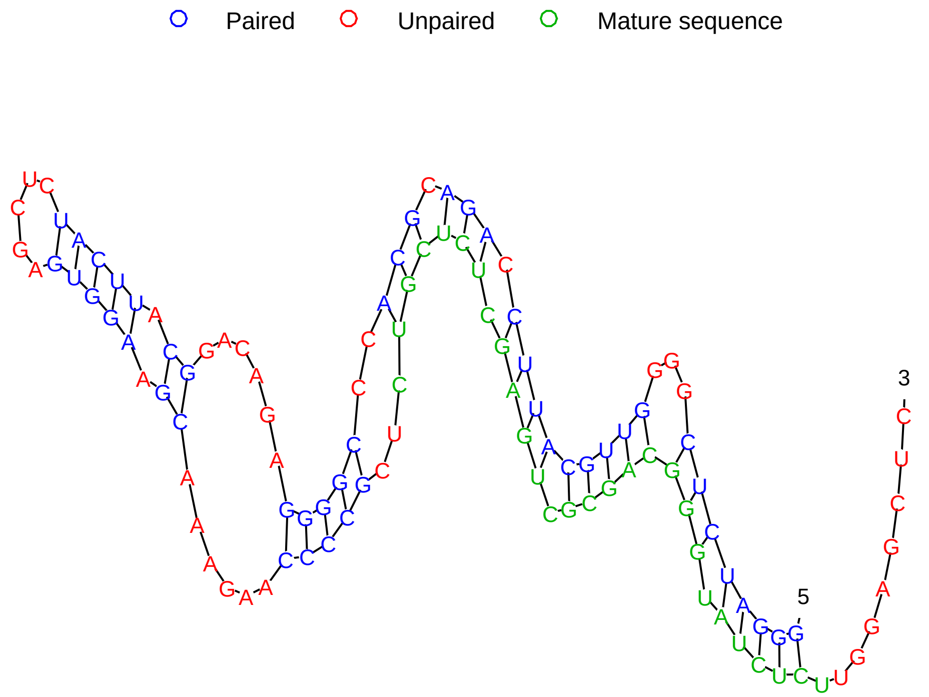

Stem loop (UMD3.1): chr27:37597550-37597660
Mature (UMD3.1): chr27:37597625-37597652
Mature seq len: 28
Total raw counts (9 samples): 13123
Average raw counts: 1459
Strand: Forward
Orientation: 3p
Minimum free energy: -35.50

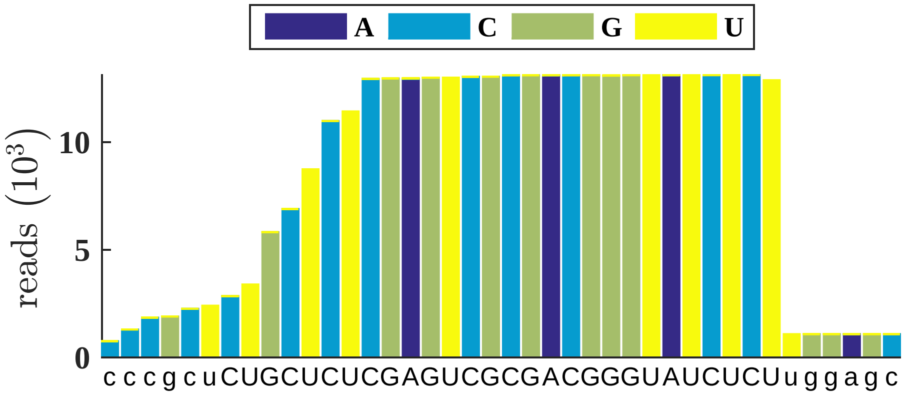

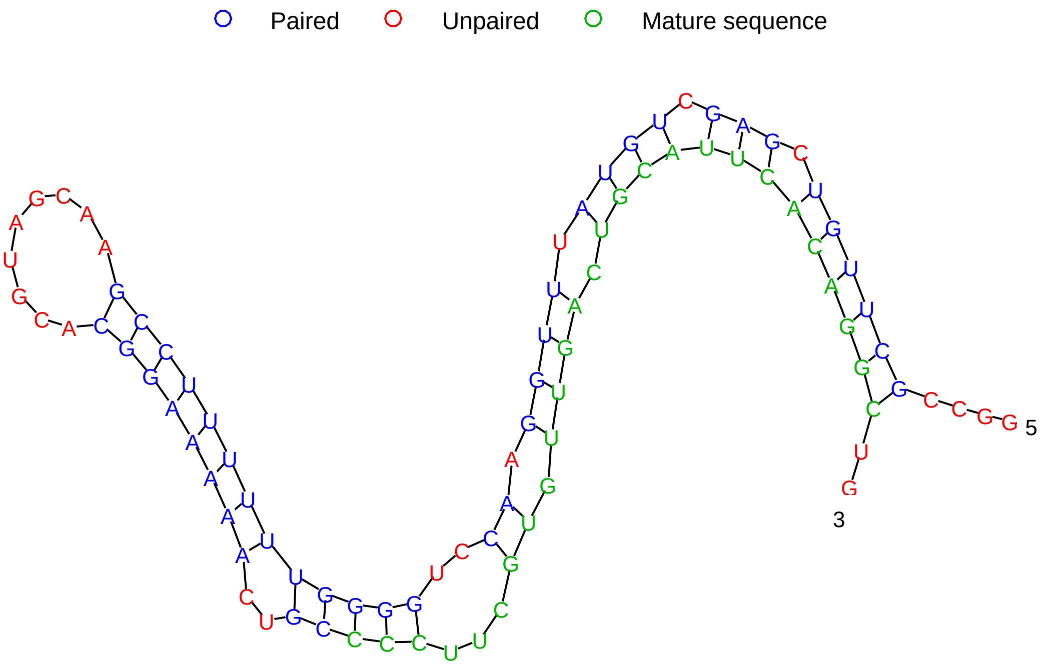

Stem loop (UMD3.1): chr27:37605995-37606086
Mature (UMD3.1): chr27:37606058-37606084
Mature seq len: 27
Total raw counts (9 samples): 1235
Average raw counts: 138
Strand: Forward
Orientation: 3p
Minimum free energy: -28.90

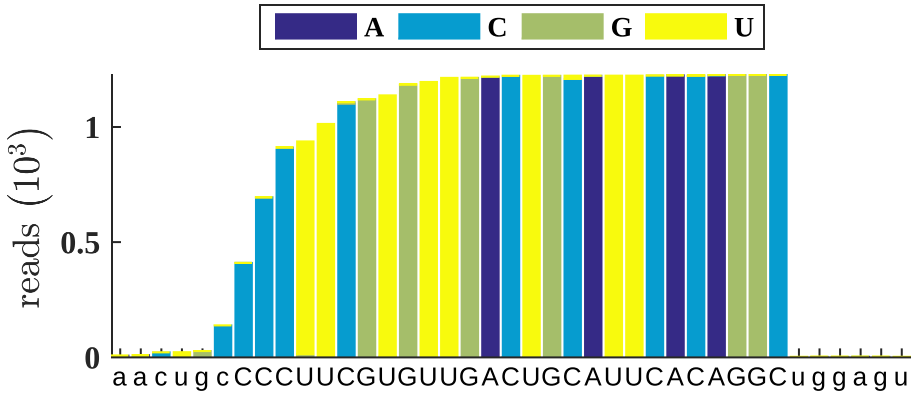

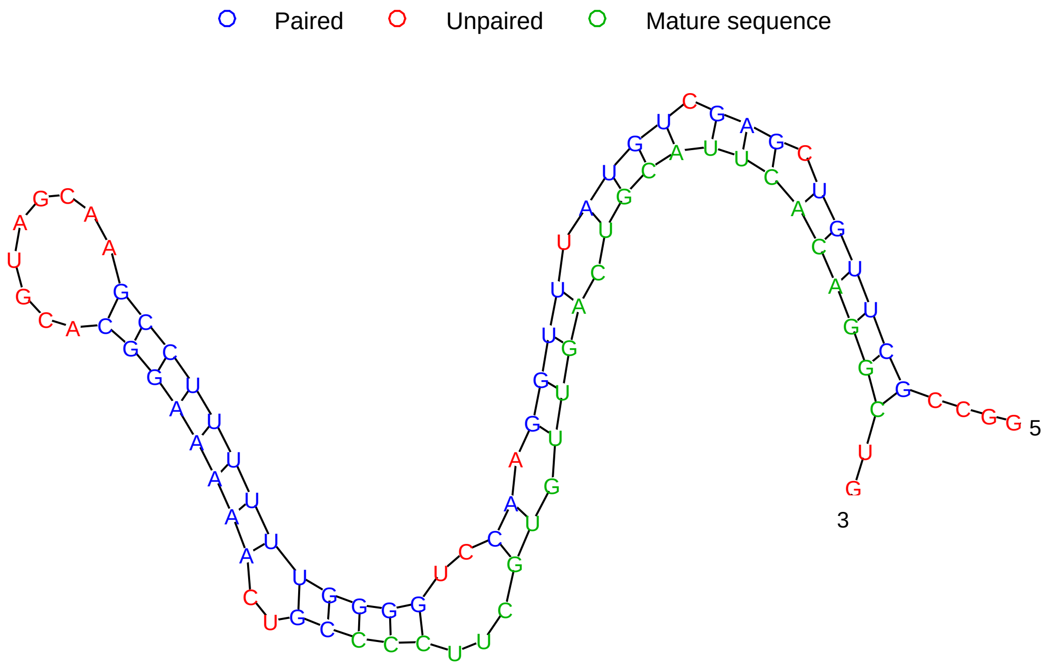

Stem loop (UMD3.1): chr27:37606962-37607053
Mature (UMD3.1): chr27:37606964-37606990
Mature seq len: 27
Total raw counts (9 samples): 1225
Average raw counts: 137
Strand: Reverse
Orientation: 3p
Minimum free energy: -28.90

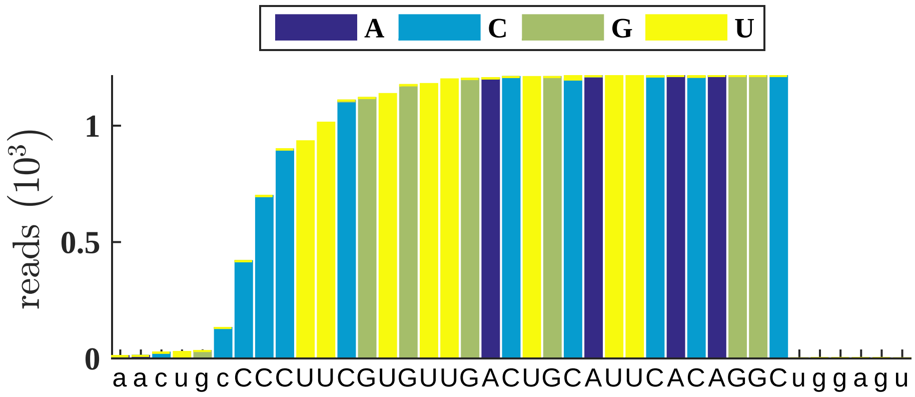

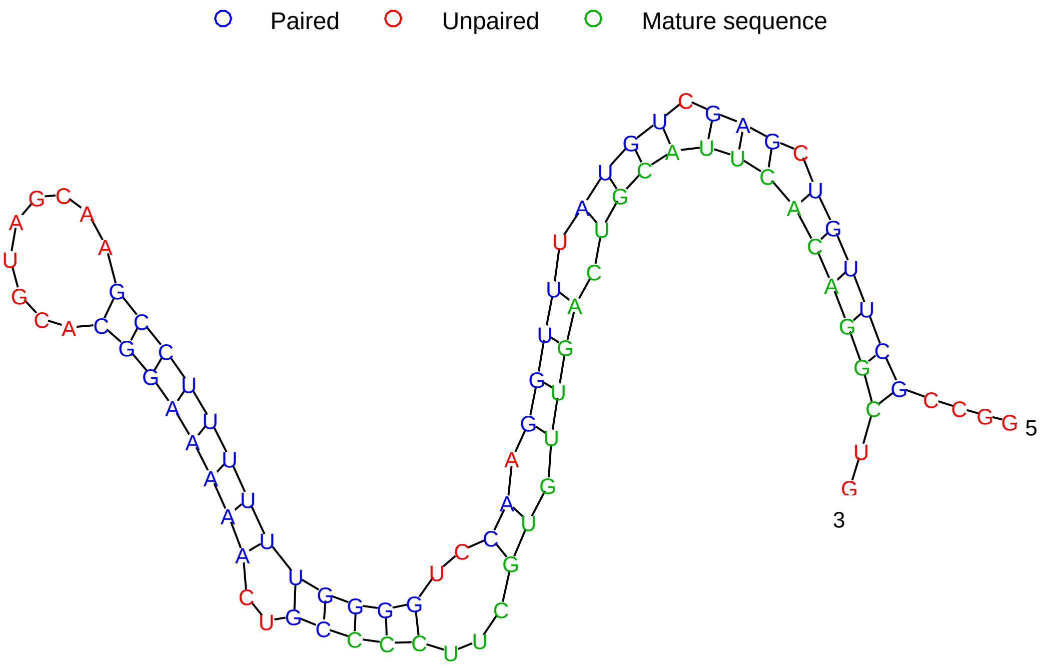

Stem loop (UMD3.1): chr27:37608355-37608446
Mature (UMD3.1): chr27:37608357-37608383
Mature seq len: 27
Total raw counts (9 samples): 1202
Average raw counts: 134
Strand: Reverse
Orientation: 3p
Minimum free energy: -28.90

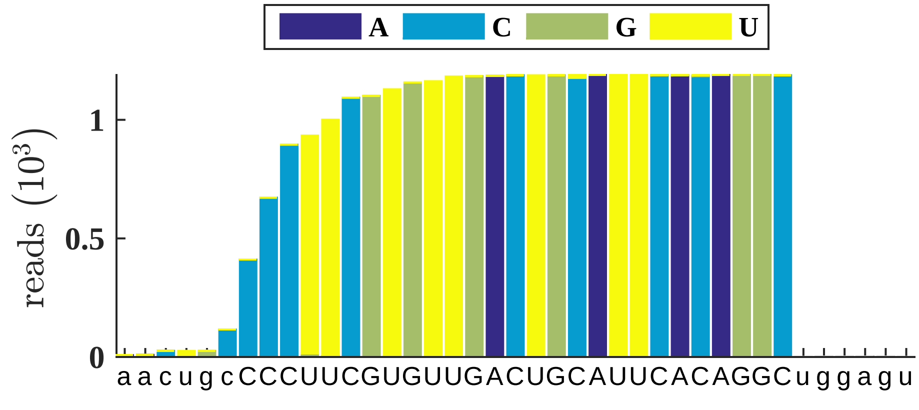

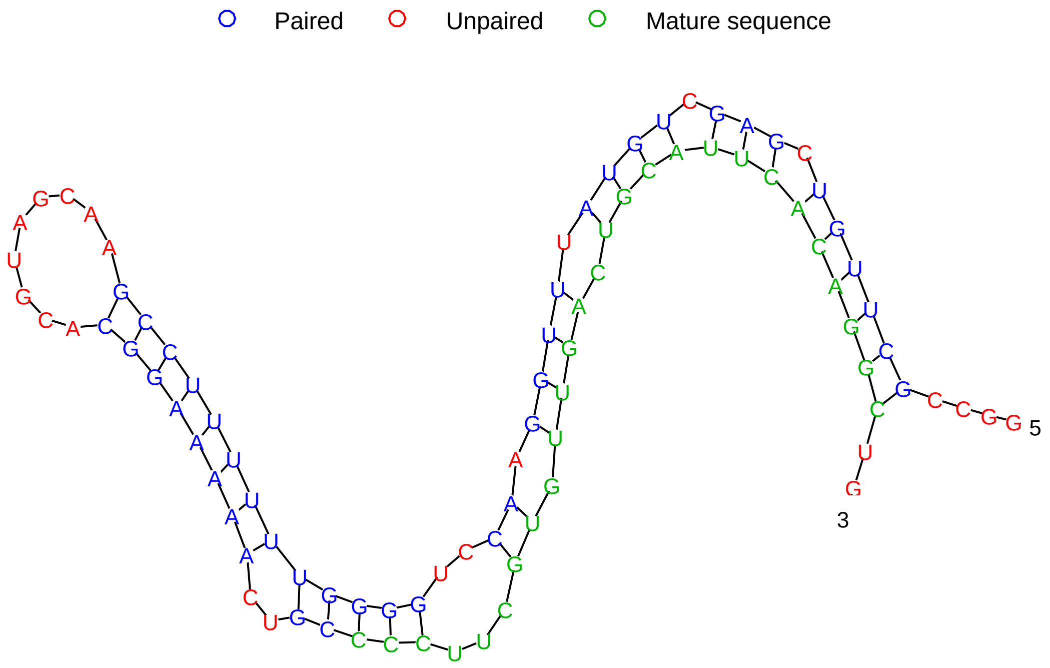

Stem loop (UMD3.1): chr27:37619166-37619257
Mature (UMD3.1): chr27:37619168-37619194
Mature seq len: 27
Total raw counts (9 samples): 1179
Average raw counts: 131
Strand: Reverse
Orientation: 3p
Minimum free energy: -28.90

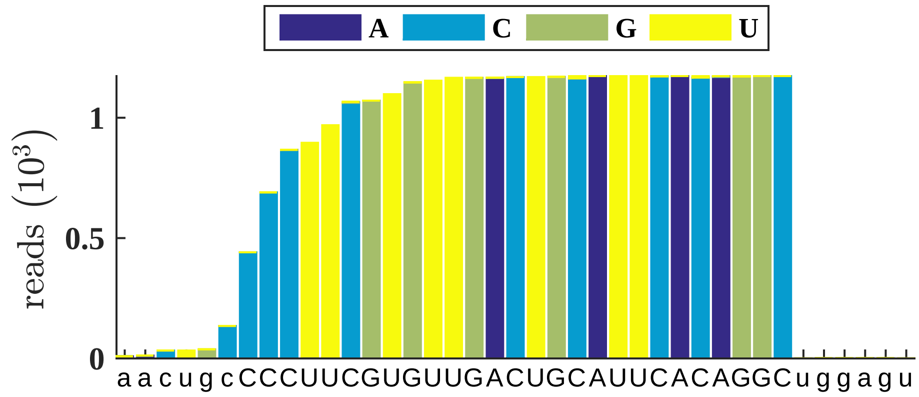

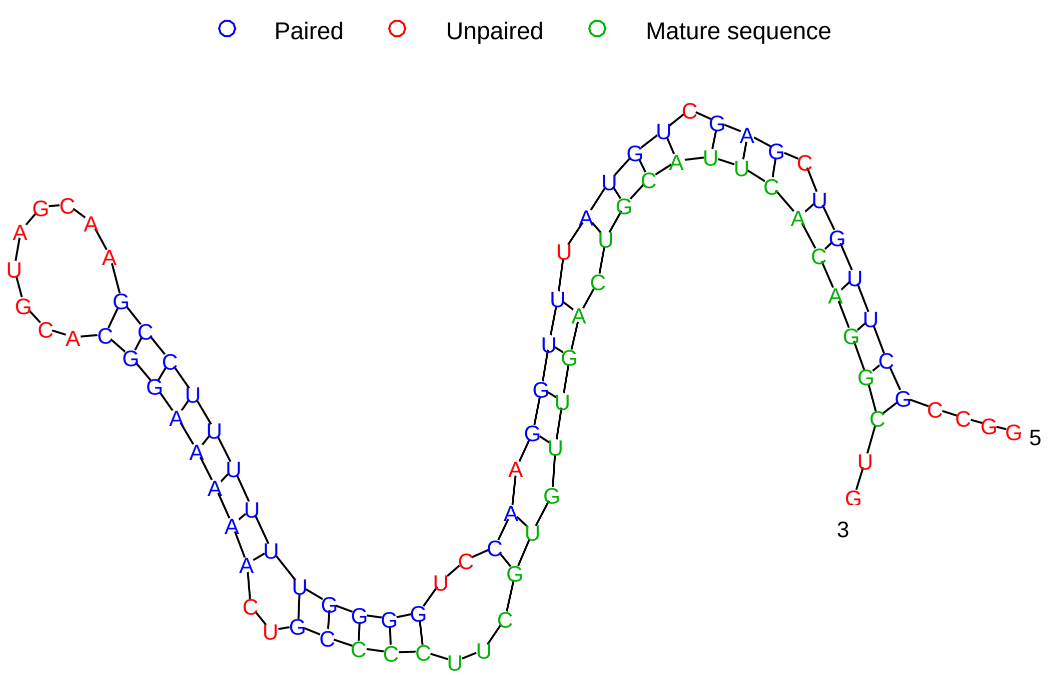

Stem loop (UMD3.1): chr27:37625841-37625932
Mature (UMD3.1): chr27:37625843-37625869
Mature seq len: 27
Total raw counts (9 samples): 1149
Average raw counts: 128
Strand: Reverse
Orientation: 3p
Minimum free energy: -28.90

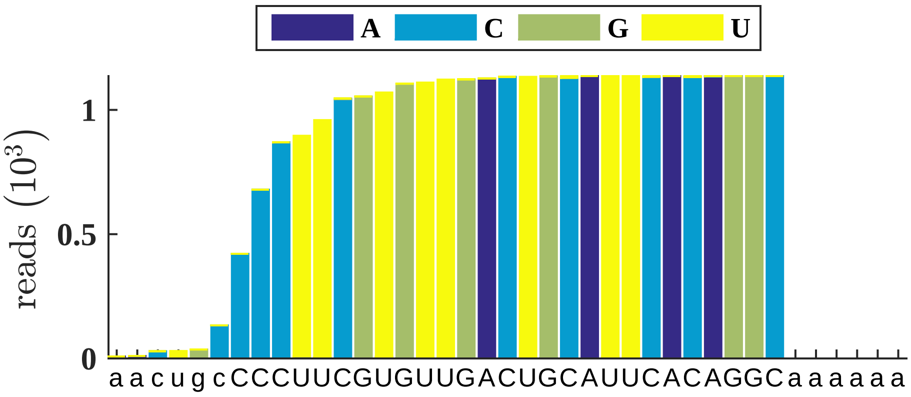

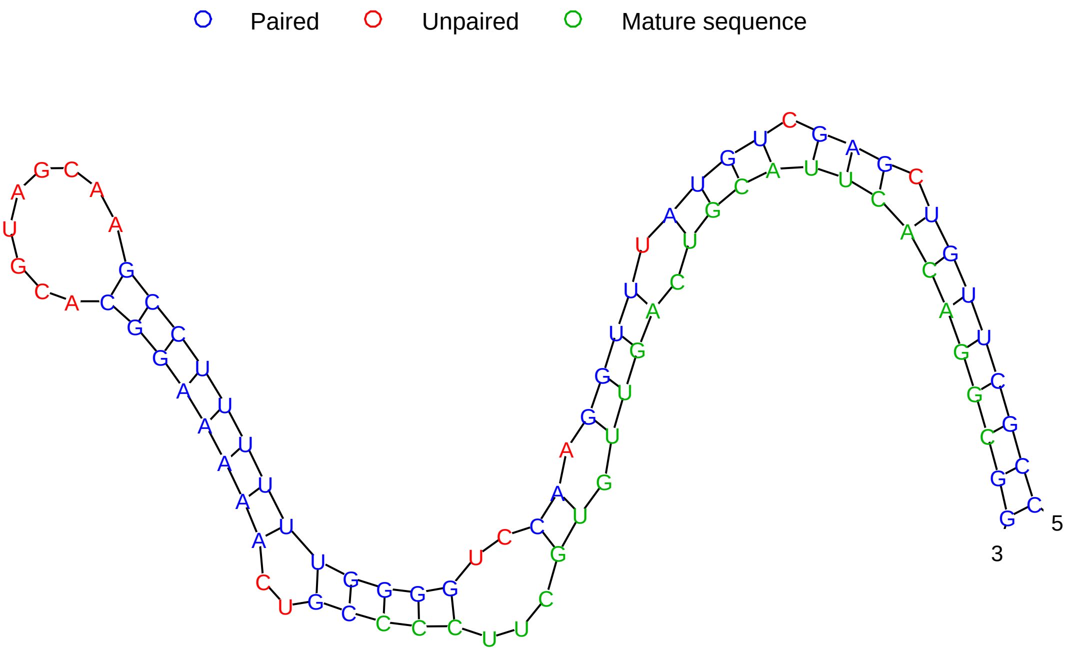

Stem loop (UMD3.1): chr27:37628014-37628103
Mature (UMD3.1): chr27:37628016-37628042
Mature seq len: 27
Total raw counts (9 samples): 1265
Average raw counts: 141
Strand: Reverse
Orientation: 3p
Minimum free energy: -33.20

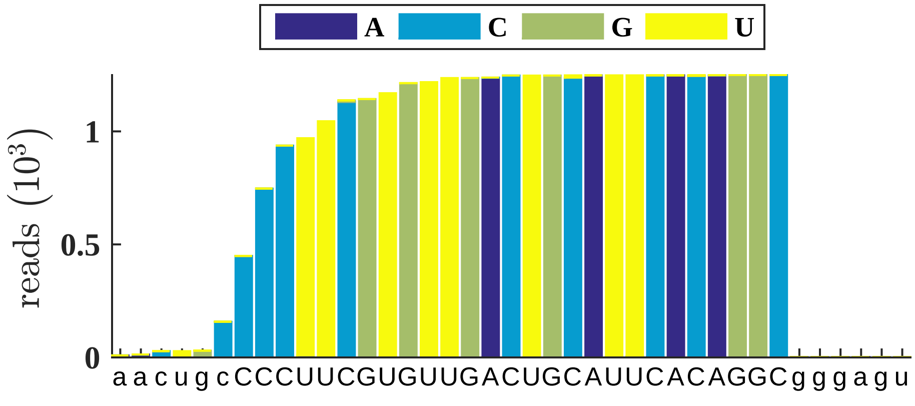

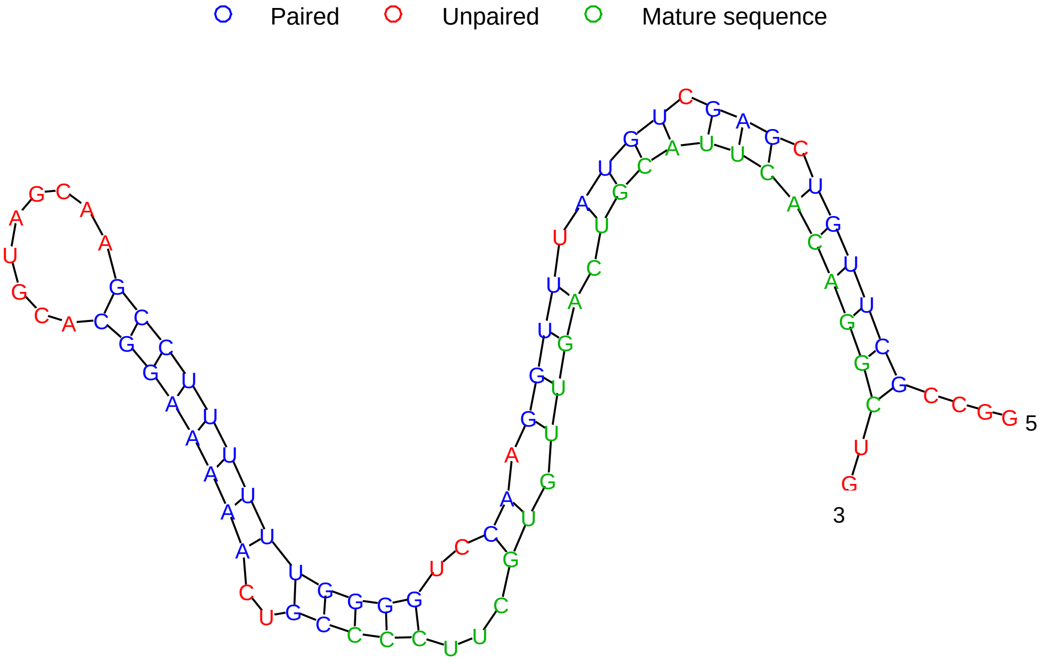

Stem loop (UMD3.1): chr27:37629443-37629534
Mature (UMD3.1): chr27:37629445-37629471
Mature seq len: 27
Total raw counts (9 samples): 1169
Average raw counts: 130
Strand: Reverse
Orientation: 3p
Minimum free energy: -28.90

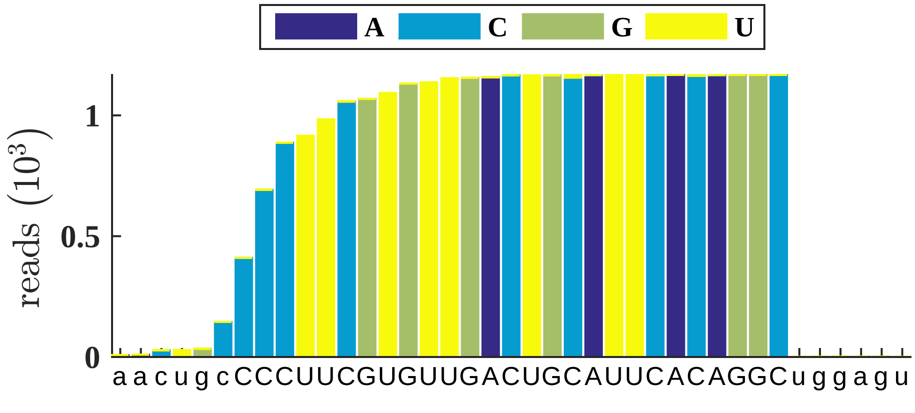

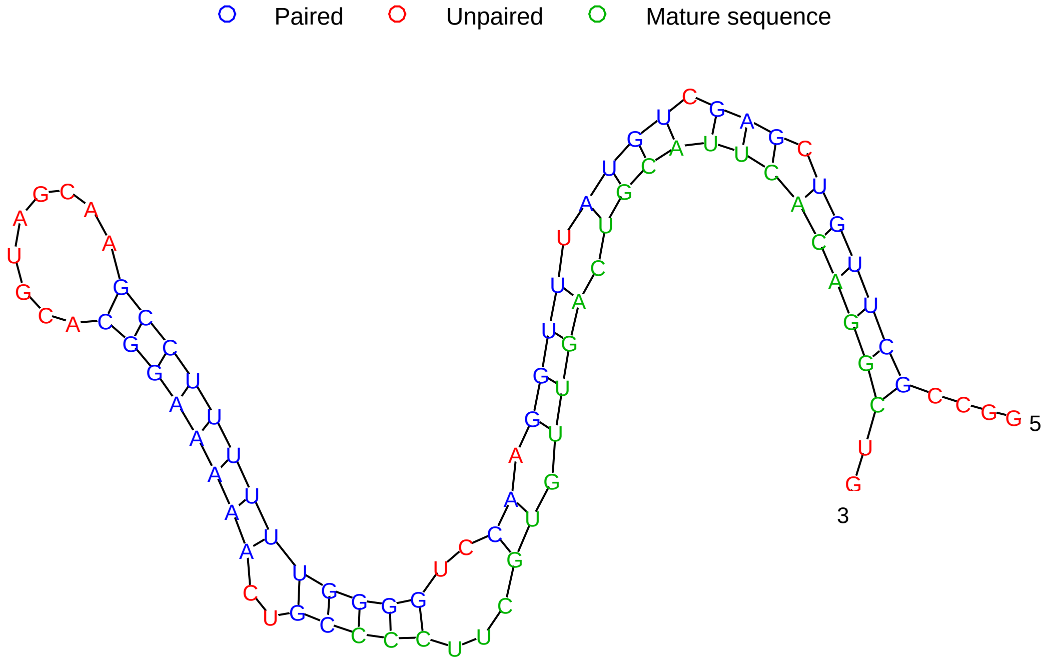

Stem loop (UMD3.1): chr27:37630750-37630841
Mature (UMD3.1): chr27:37630813-37630839
Mature seq len: 27
Total raw counts (9 samples): 1180
Average raw counts: 132
Strand: Forward
Orientation: 3p
Minimum free energy: -28.90

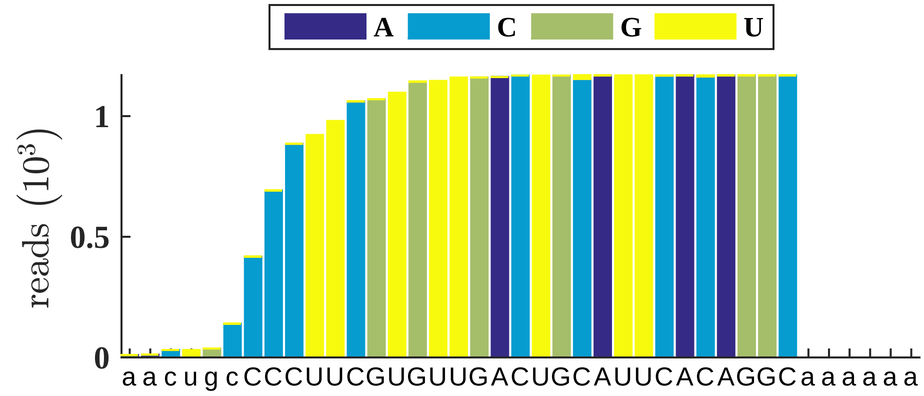

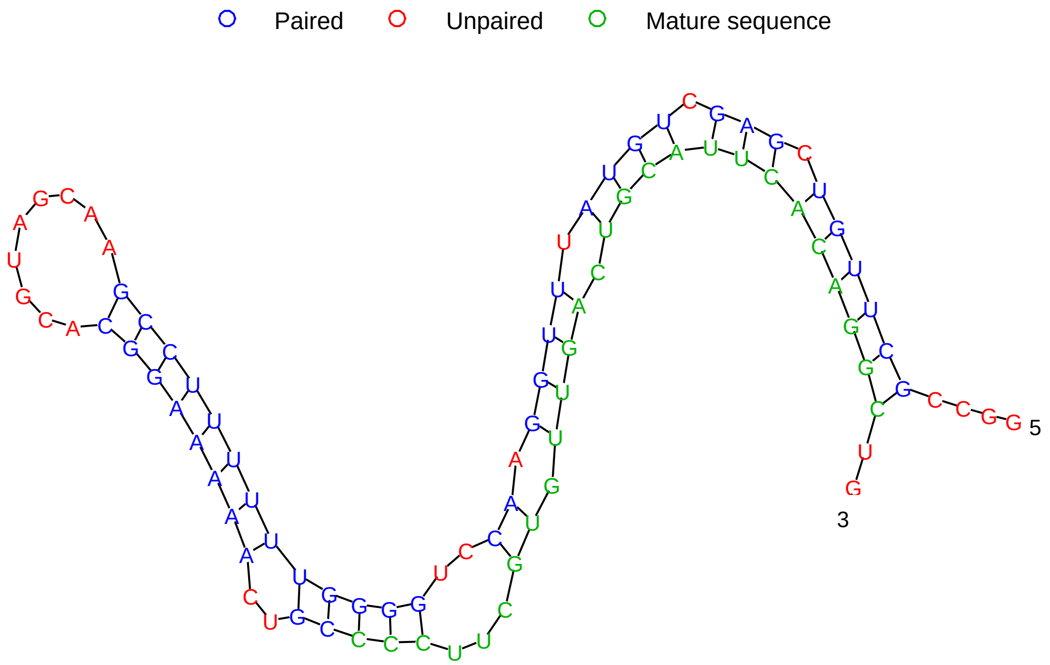

Stem loop (UMD3.1): chr27:37634093-37634184
Mature (UMD3.1): chr27:37634095-37634121
Mature seq len: 27
Total raw counts (9 samples): 1201
Average raw counts: 134
Strand: Reverse
Orientation: 3p
Minimum free energy: -28.90

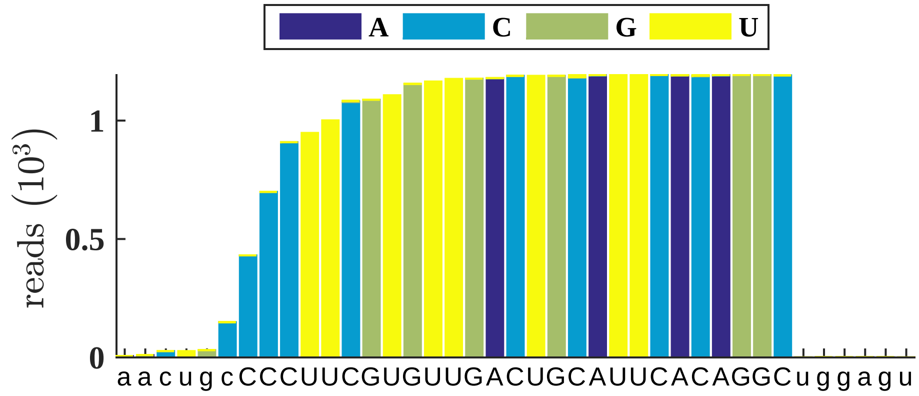

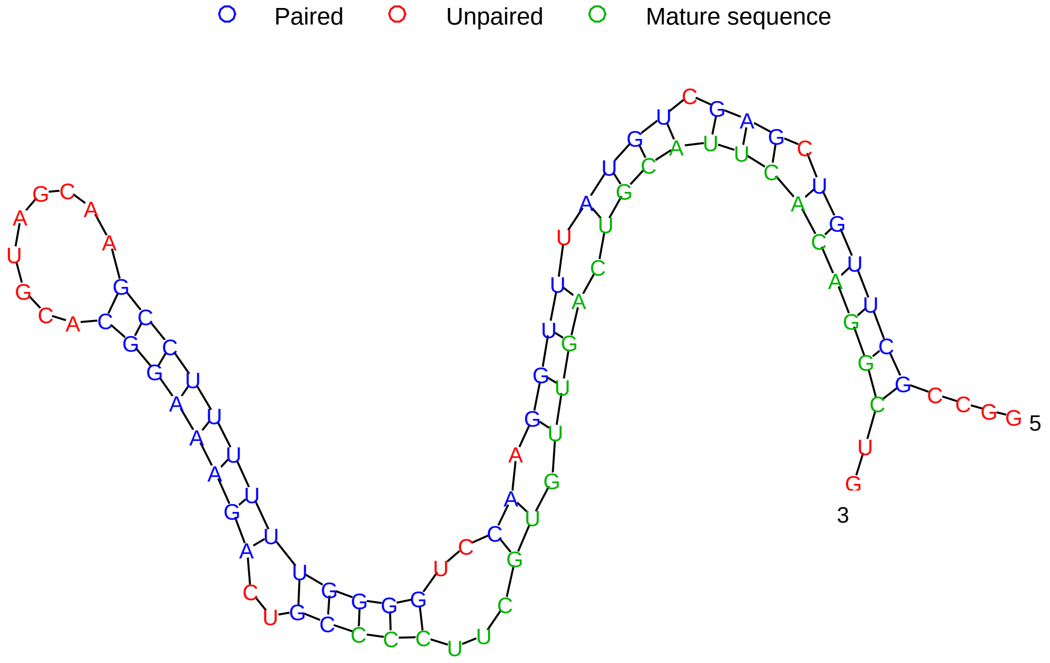

Stem loop (UMD3.1): chr27:37635274-37635365
Mature (UMD3.1): chr27:37635337-37635363
Mature seq len: 27
Total raw counts (9 samples): 1239
Average raw counts: 138
Strand: Forward
Orientation: 3p
Minimum free energy: -29.00

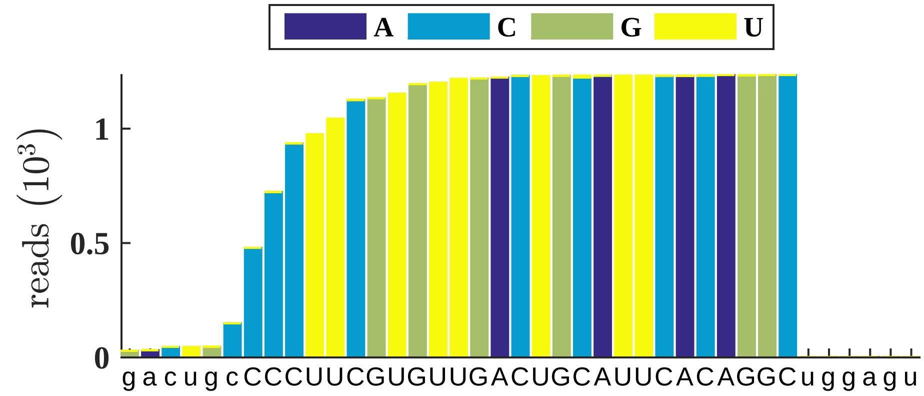

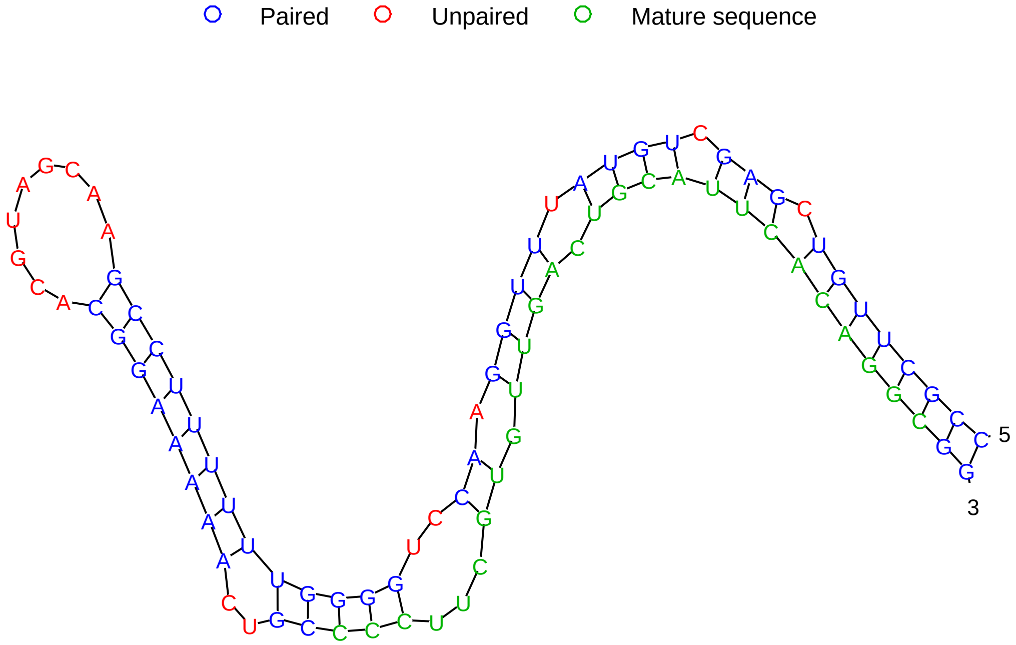

Stem loop (UMD3.1): chr27:37637347-37637436
Mature (UMD3.1): chr27:37637408-37637434
Mature seq len: 27
Total raw counts (9 samples): 1187
Average raw counts: 132
Strand: Forward
Orientation: 3p
Minimum free energy: -33.20

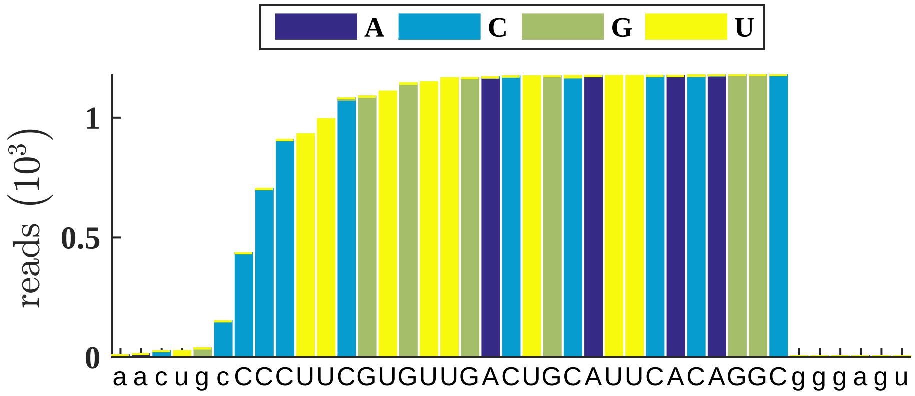

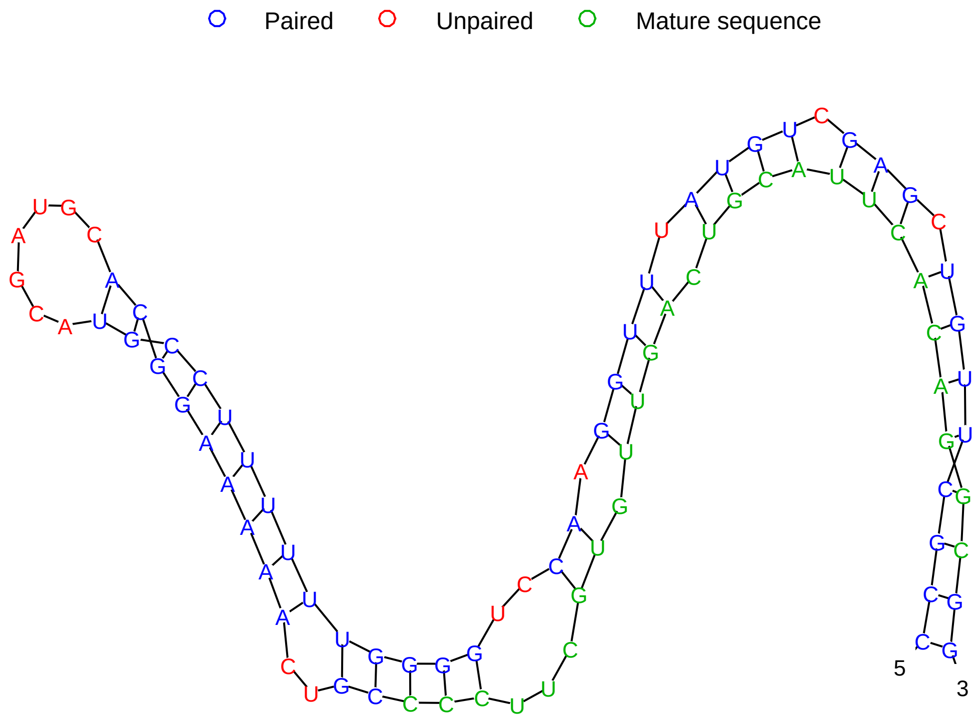

Stem loop (UMD3.1): chr27:37638726-37638815
Mature (UMD3.1): chr27:37638787-37638813
Mature seq len: 27
Total raw counts (9 samples): 1254
Average raw counts: 140
Strand: Forward
Orientation: 3p
Minimum free energy: -35.00

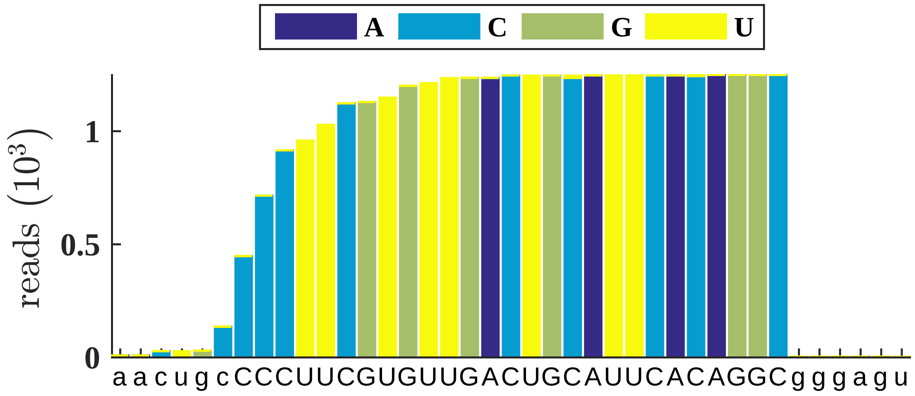

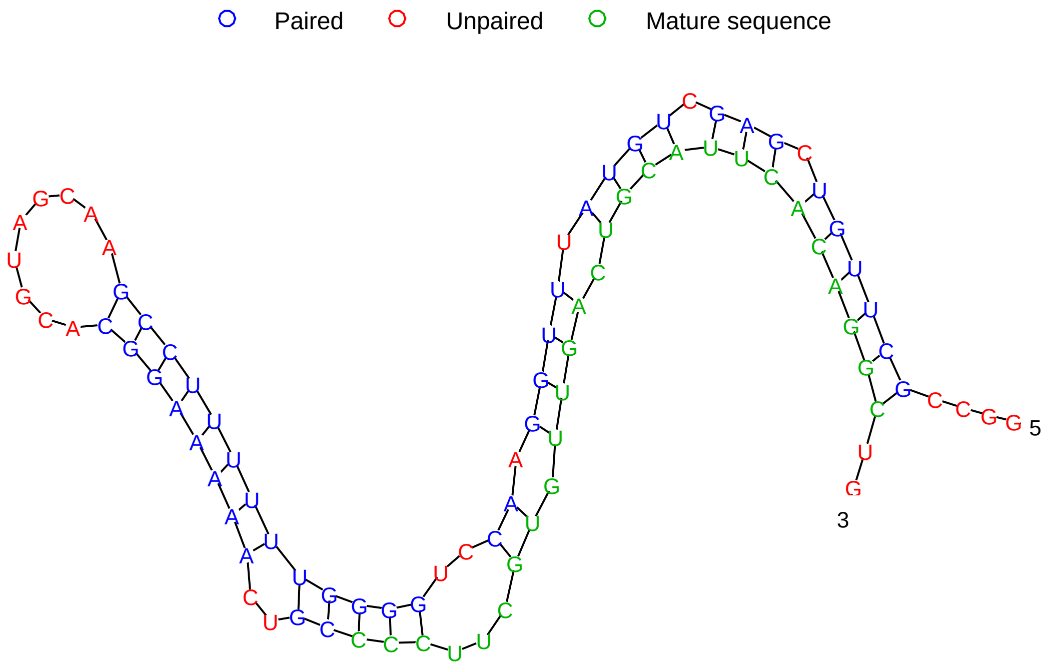

Stem loop (UMD3.1): chr27:37639497-37639588
Mature (UMD3.1): chr27:37639560-37639586
Mature seq len: 27
Total raw counts (9 samples): 1181
Average raw counts: 132
Strand: Forward
Orientation: 3p
Minimum free energy: -28.90

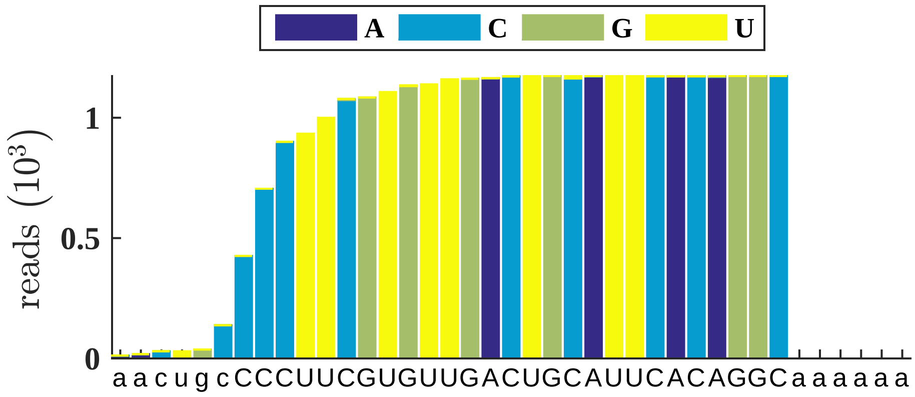

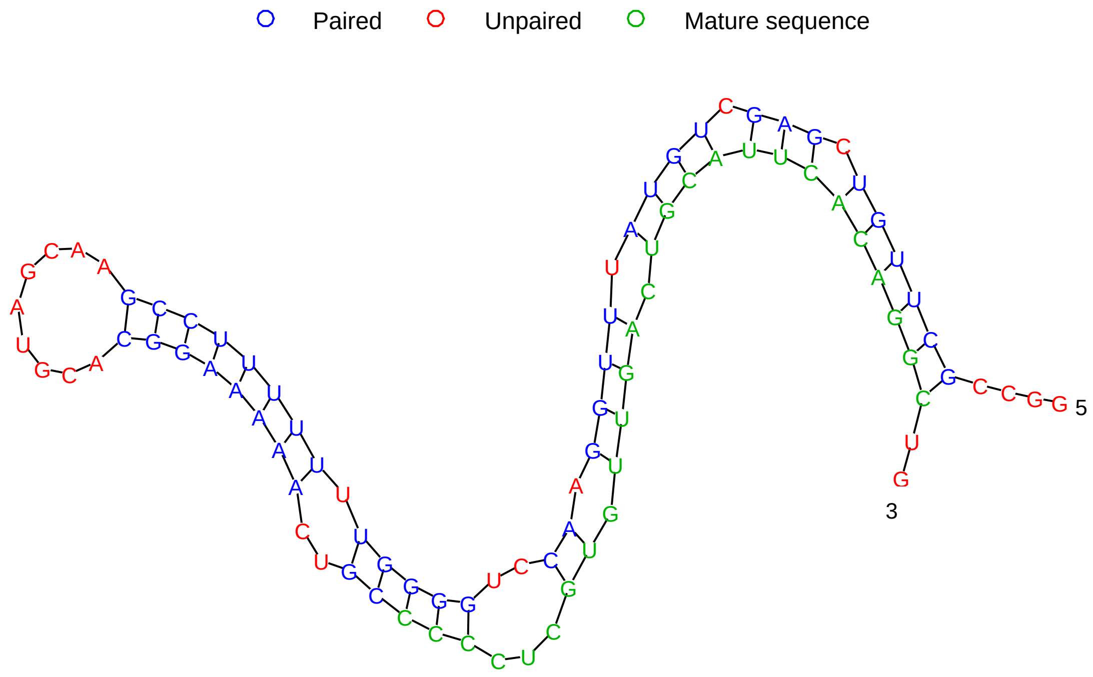

Stem loop (UMD3.1): chr27:37645872-37645964
Mature (UMD3.1): chr27:37645936-37645962
Mature seq len: 27
Total raw counts (9 samples): 664
Average raw counts: 74
Strand: Forward
Orientation: 3p
Minimum free energy: -29.40

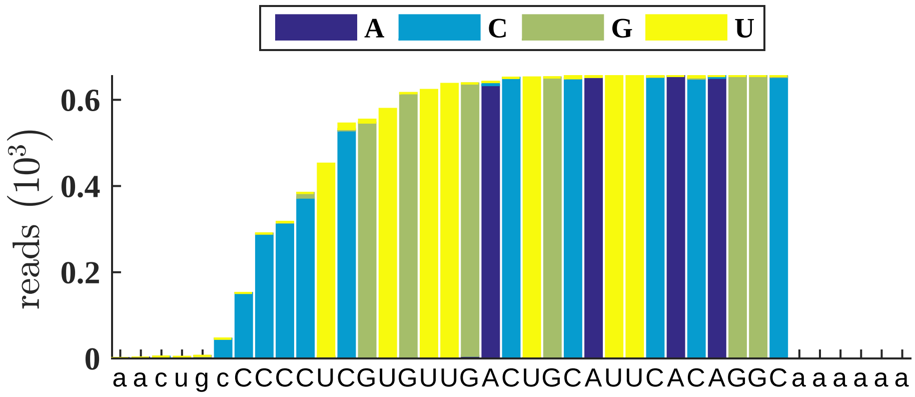

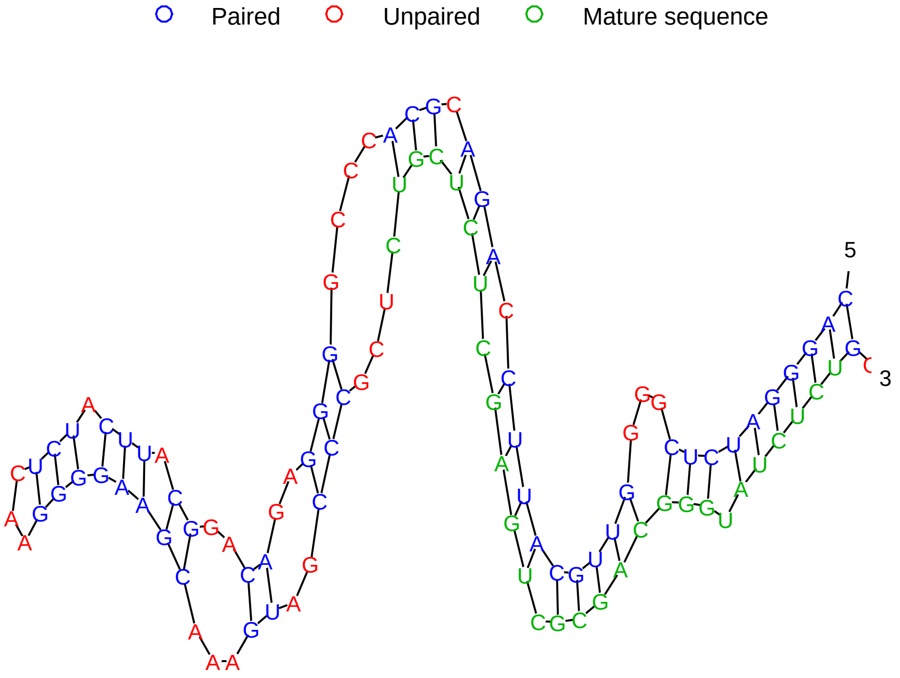

Stem loop (UMD3.1): chr27:37649607-37649713
Mature (UMD3.1): chr27:37649684-37649711
Mature seq len: 28
Total raw counts (9 samples): 11685
Average raw counts: 1299
Strand: Forward
Orientation: 3p
Minimum free energy: -35.60

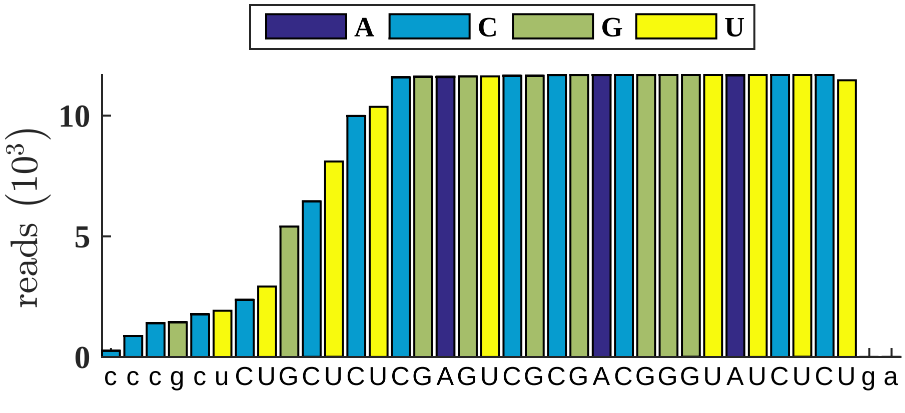

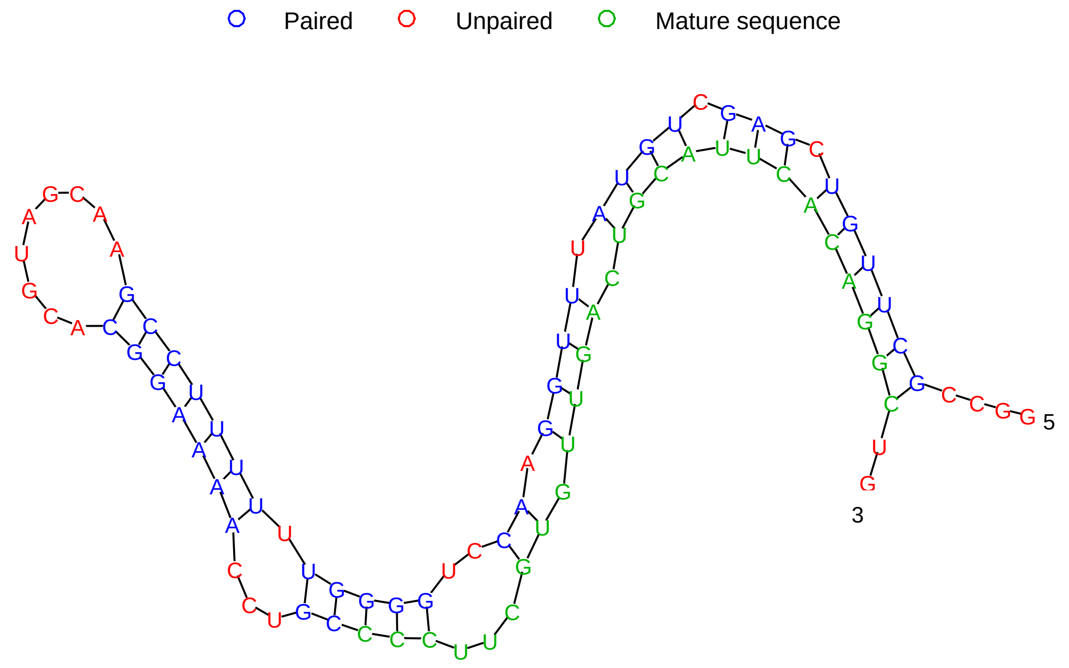

Stem loop (UMD3.1): chr27:37649949-37650040
Mature (UMD3.1): chr27:37650012-37650038
Mature seq len: 27
Total raw counts (9 samples): 1206
Average raw counts: 134
Strand: Forward
Orientation: 3p
Minimum free energy: -28.10

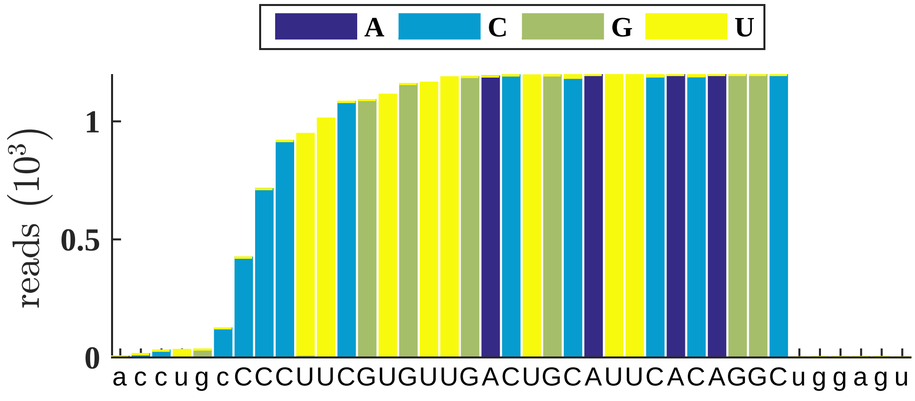

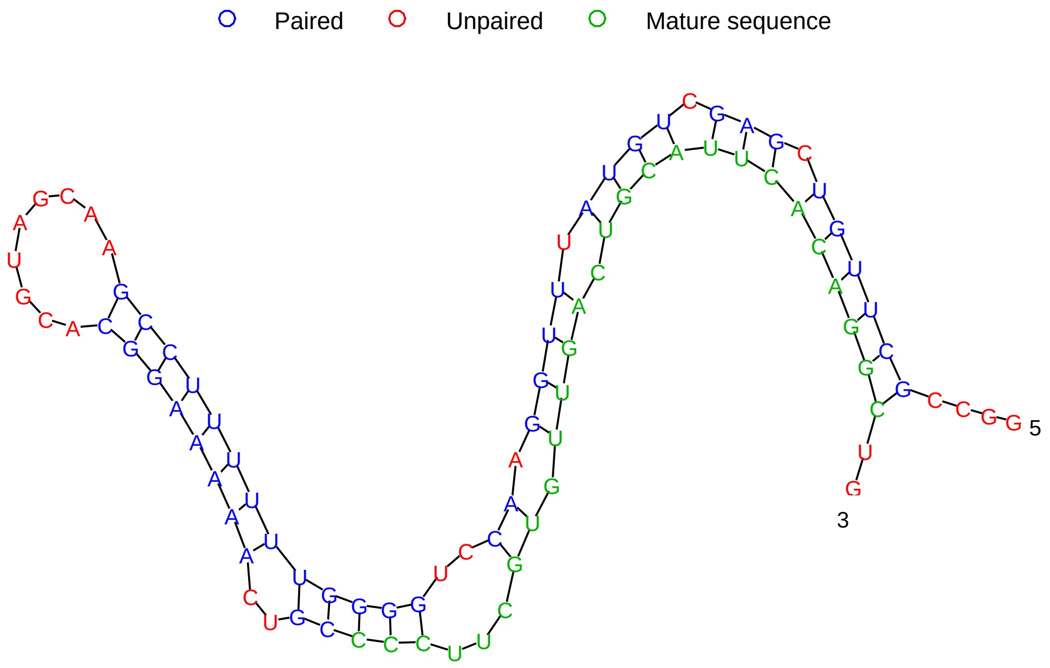

Stem loop (UMD3.1): chr27:5115325-5115416
Mature (UMD3.1): chr27:5115388-5115414
Mature seq len: 27
Total raw counts (9 samples): 1173
Average raw counts: 131
Strand: Forward
Orientation: 3p
Minimum free energy: -28.90

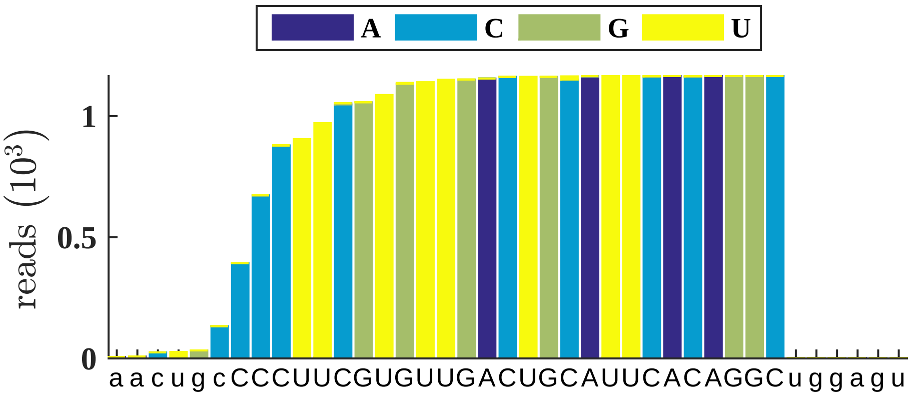

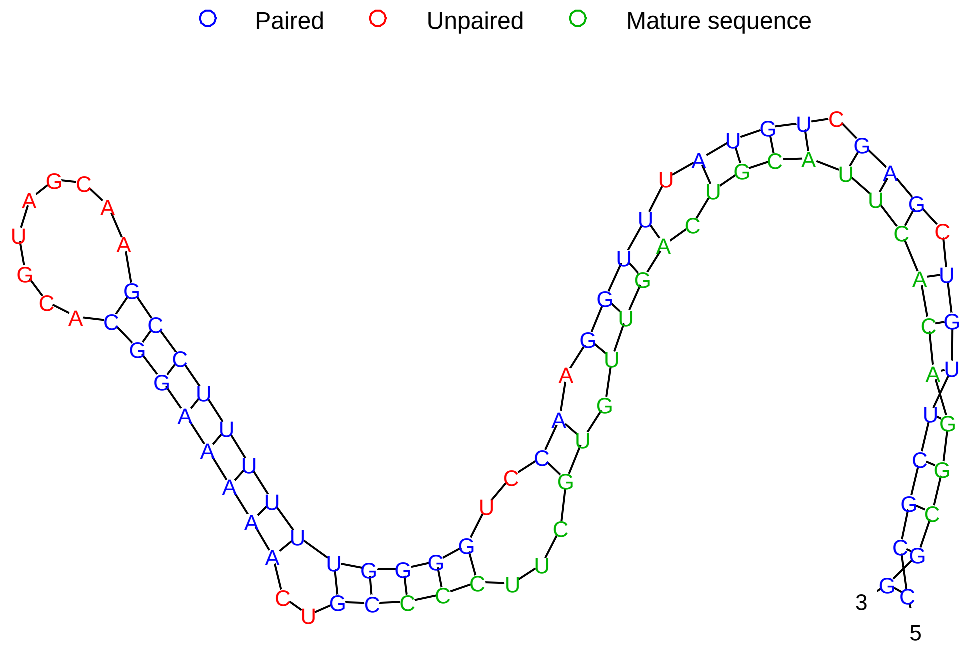

Stem loop (UMD3.1): chr27:6211445-6211534
Mature (UMD3.1): chr27:6211506-6211532
Mature seq len: 27
Total raw counts (9 samples): 1192
Average raw counts: 133
Strand: Forward
Orientation: 3p
Minimum free energy: -33.20

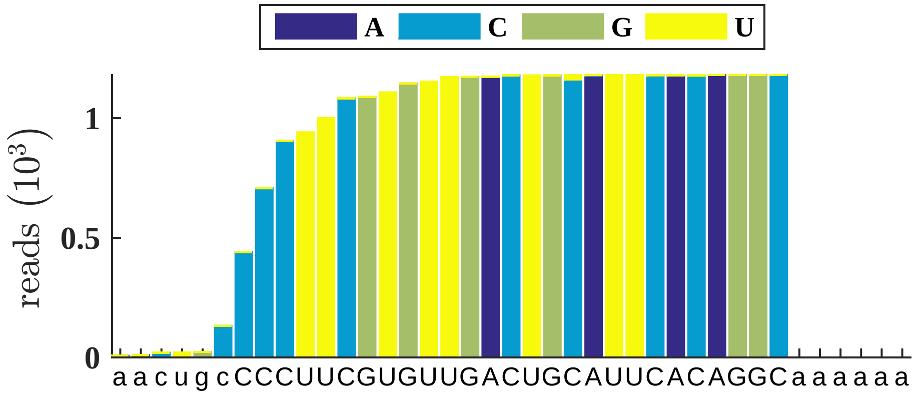

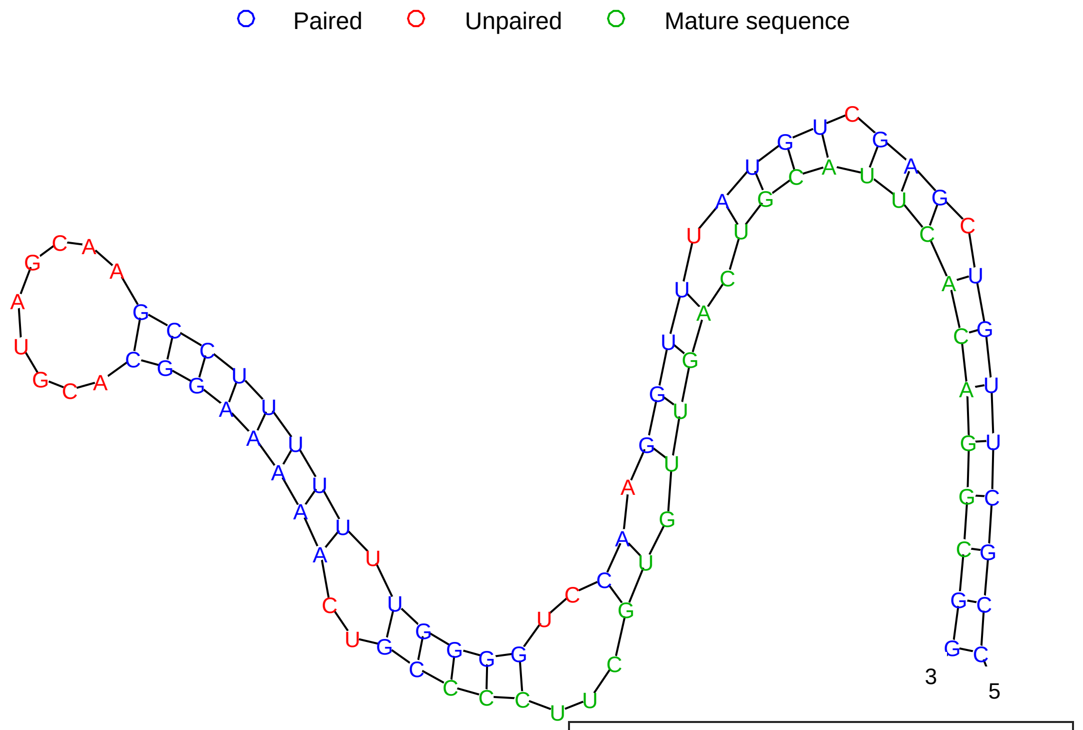

Stem loop (UMD3.1): chr27:6858544-6858634
Mature (UMD3.1): chr27:6858606-6858632
Mature seq len: 27
Total raw counts (9 samples): 1251
Average raw counts: 139
Strand: Forward
Orientation: 3p
Minimum free energy: -34.00

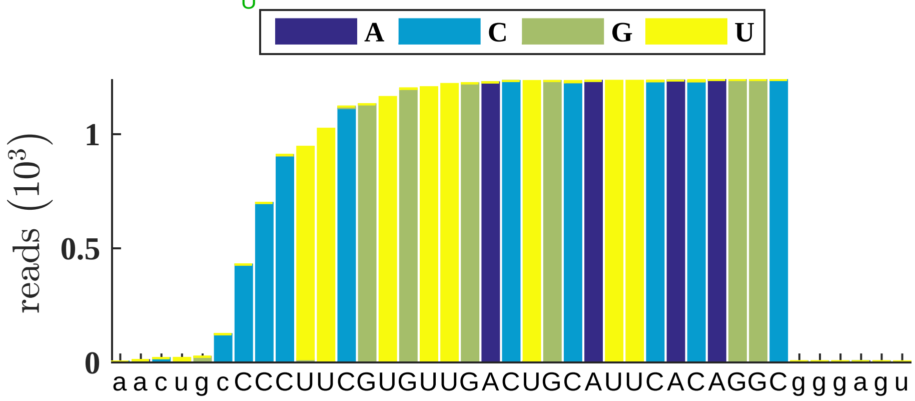

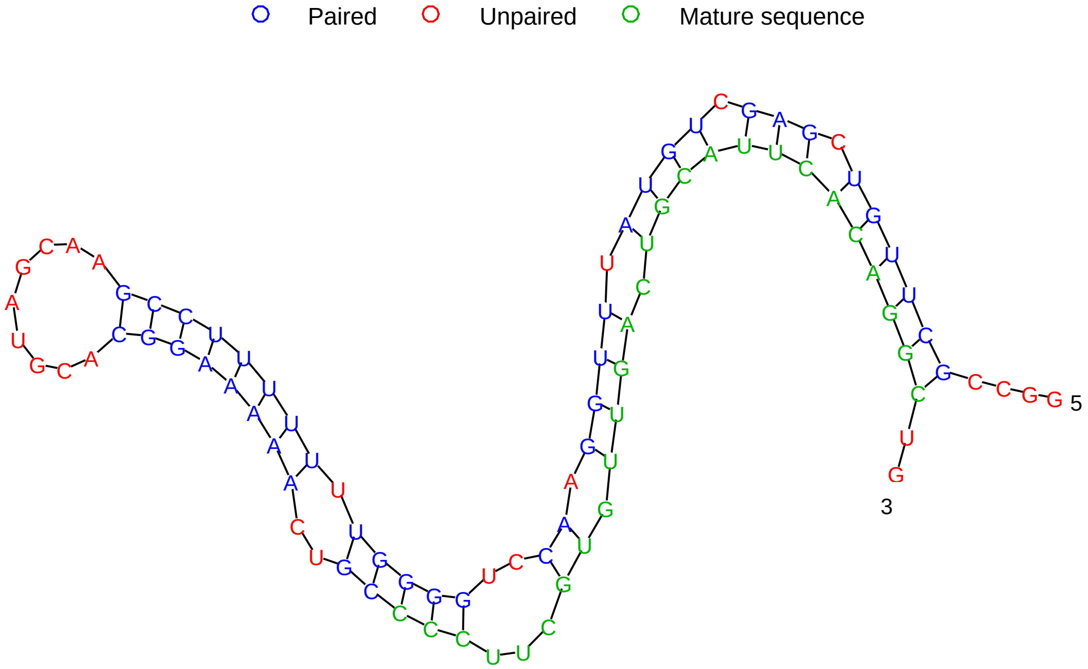

Stem loop (UMD3.1): chr27:6859944-6860036
Mature (UMD3.1): chr27:6860008-6860034
Mature seq len: 27
Total raw counts (9 samples): 1262
Average raw counts: 141
Strand: Forward
Orientation: 3p
Minimum free energy: -29.70

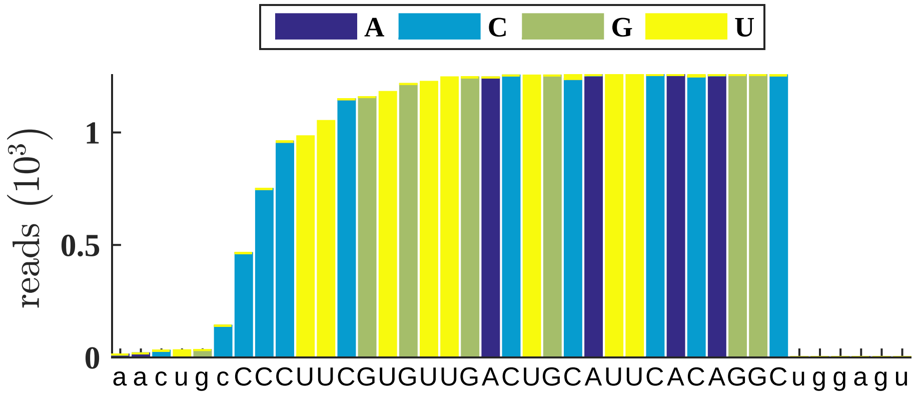

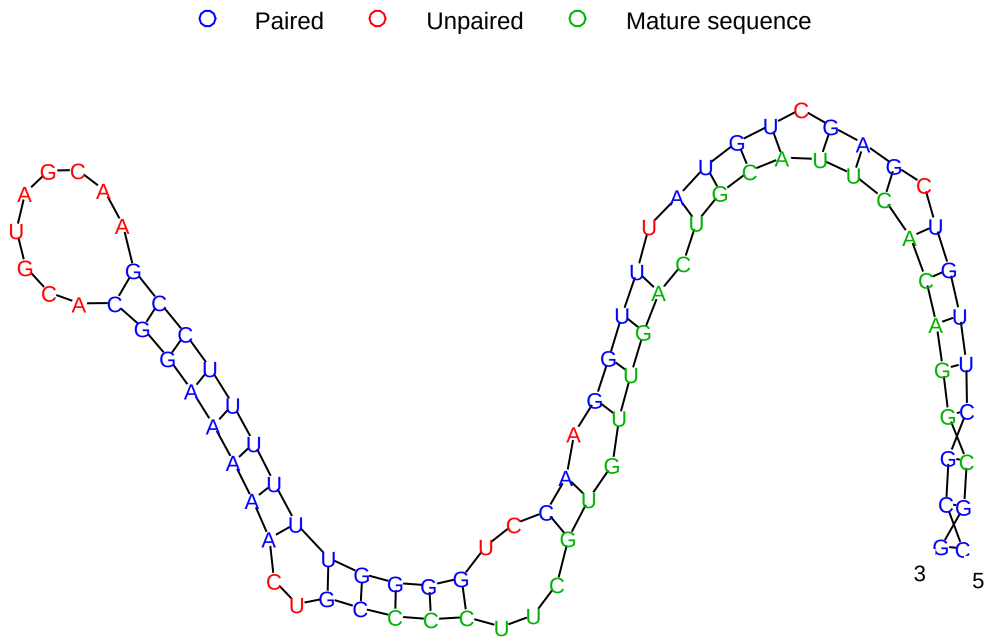

Stem loop (UMD3.1): chr28:11063121-11063210
Mature (UMD3.1): chr28:11063182-11063208
Mature seq len: 27
Total raw counts (9 samples): 1207
Average raw counts: 135
Strand: Forward
Orientation: 3p
Minimum free energy: -33.20

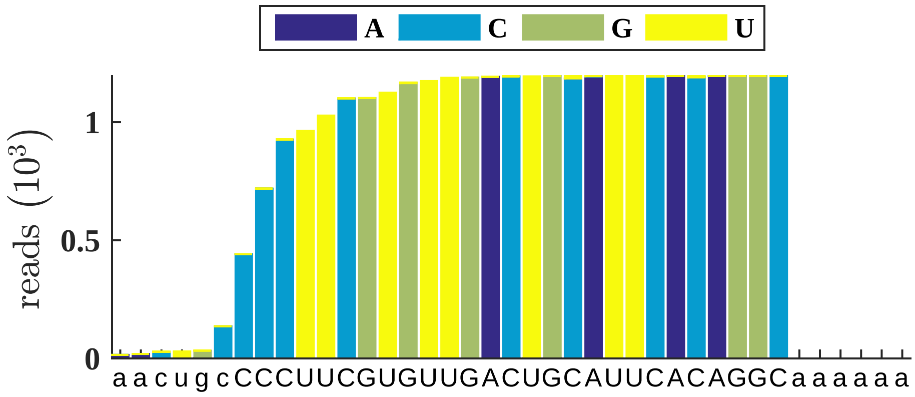

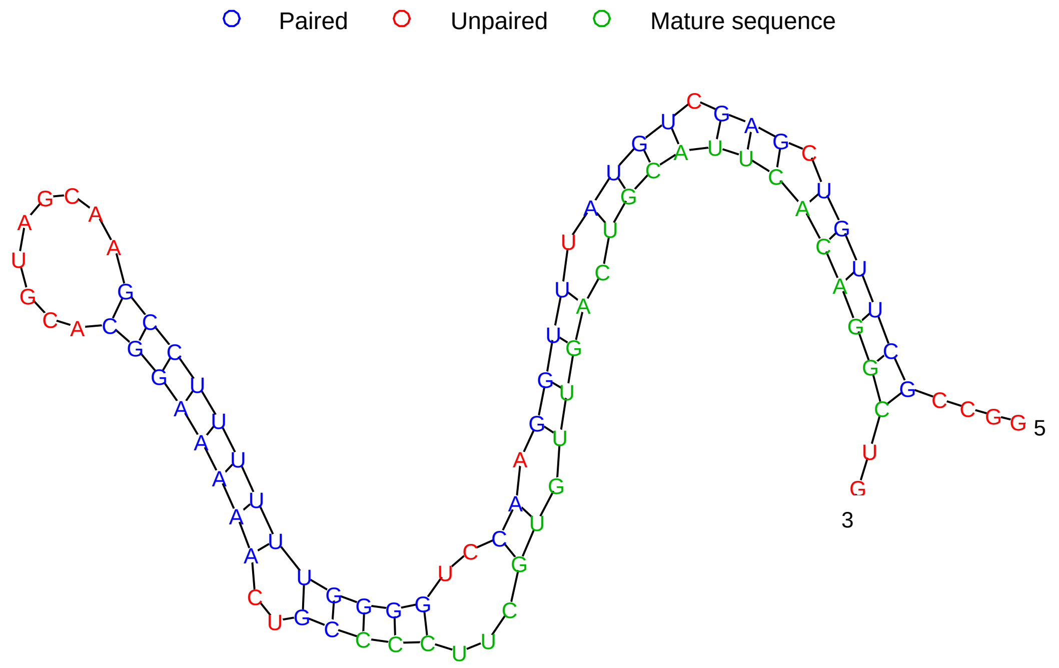

Stem loop (UMD3.1): chr28:11065312-11065403
Mature (UMD3.1): chr28:11065375-11065401
Mature seq len: 27
Total raw counts (9 samples): 1261
Average raw counts: 141
Strand: Forward
Orientation: 3p
Minimum free energy: -28.90

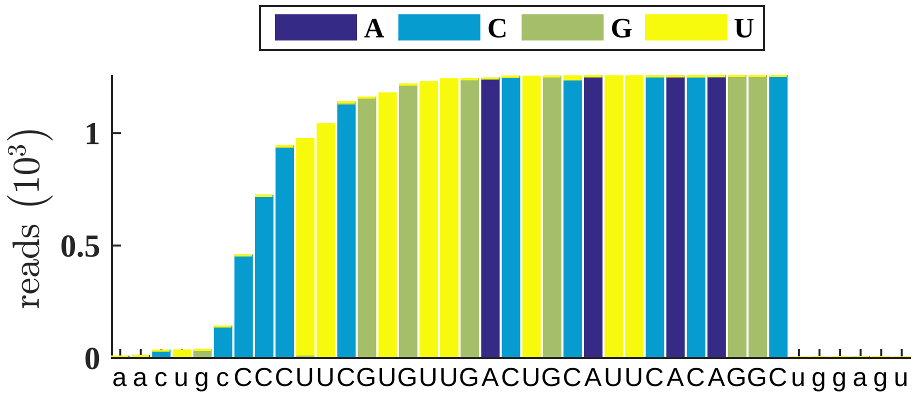

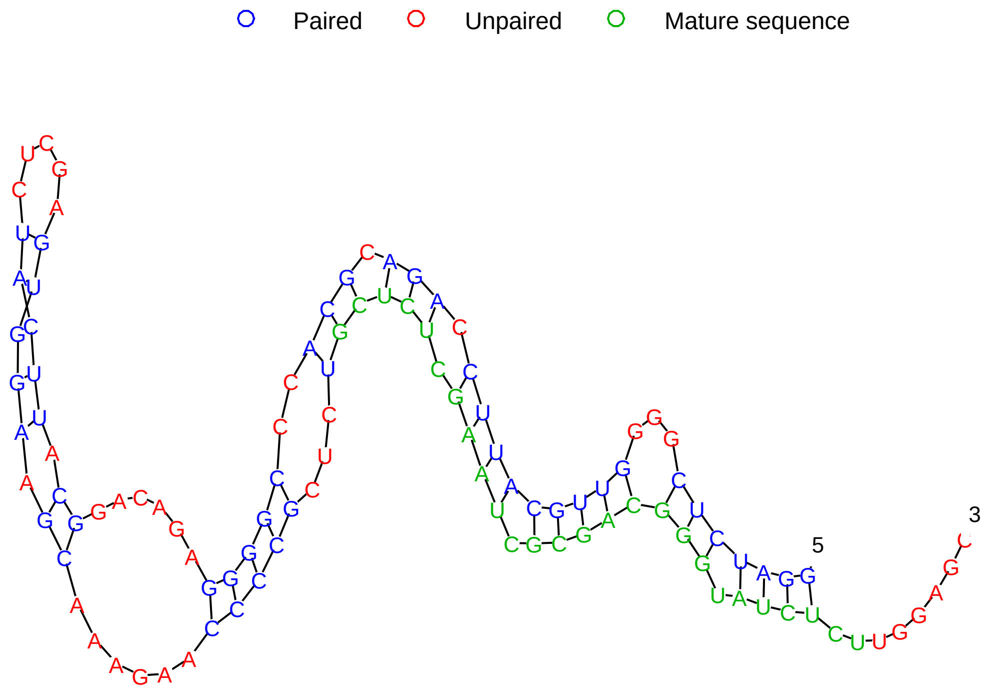

Stem loop (UMD3.1): chr28:11066003-11066110
Mature (UMD3.1): chr28:11066079-11066104
Mature seq len: 26
Total raw counts (9 samples): 4506
Average raw counts: 501
Strand: Forward
Orientation: 3p
Minimum free energy: -32.40

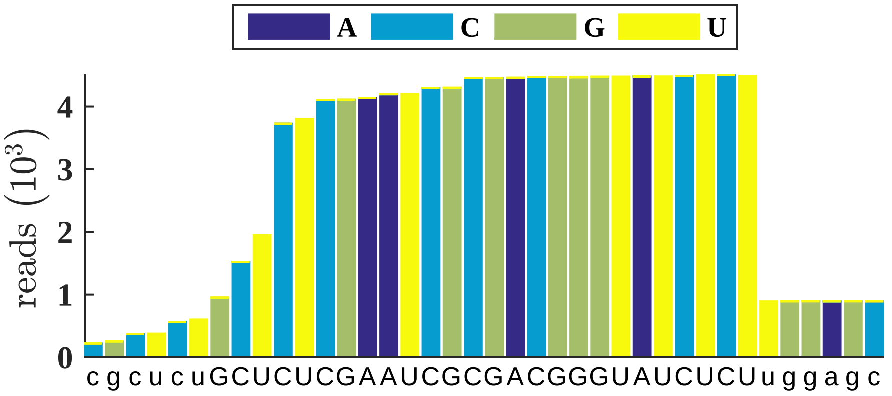

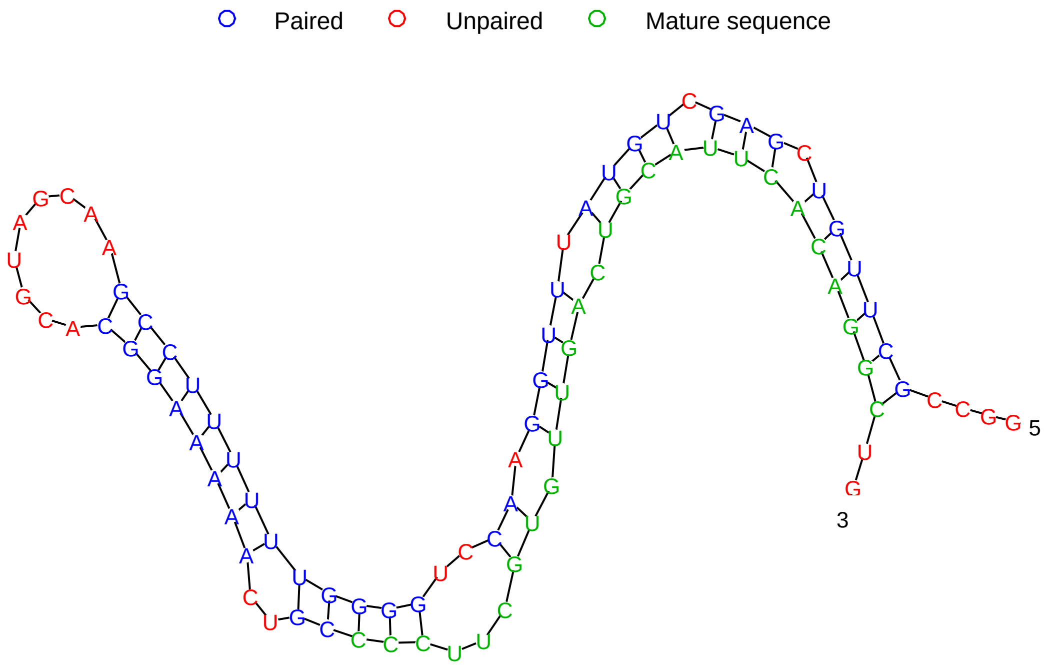

Stem loop (UMD3.1): chr28:11075292-11075383
Mature (UMD3.1): chr28:11075294-11075320
Mature seq len: 27
Total raw counts (9 samples): 1213
Average raw counts: 135
Strand: Reverse
Orientation: 3p
Minimum free energy: -28.90

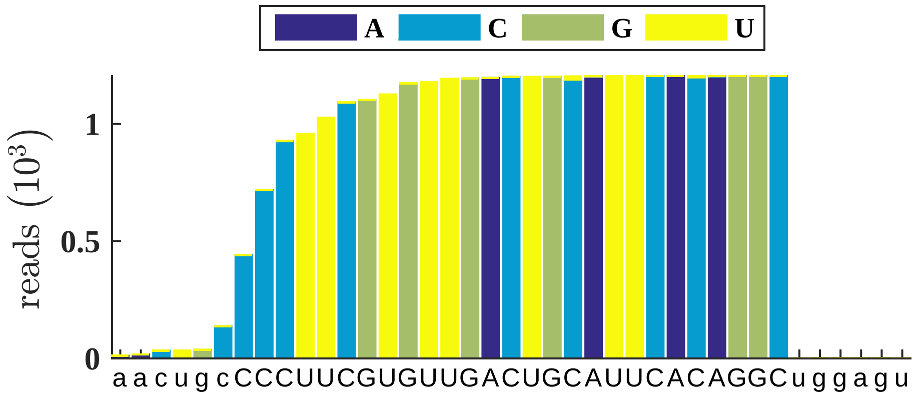

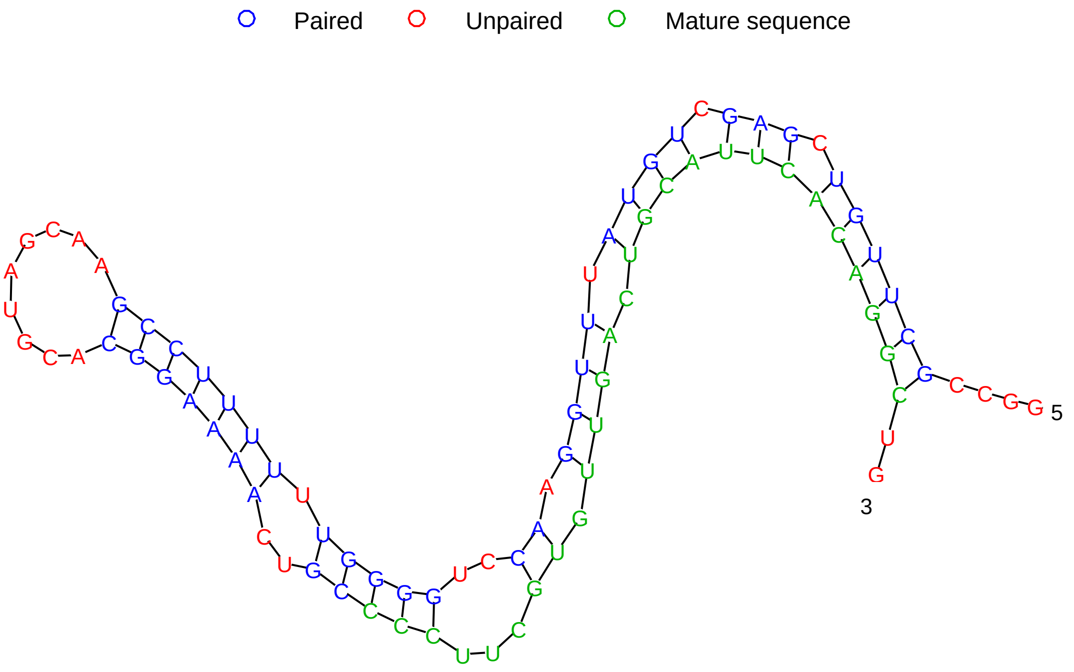

Stem loop (UMD3.1): chr28:11076683-11076773
Mature (UMD3.1): chr28:11076685-11076711
Mature seq len: 27
Total raw counts (9 samples): 1166
Average raw counts: 130
Strand: Reverse
Orientation: 3p
Minimum free energy: -28.80

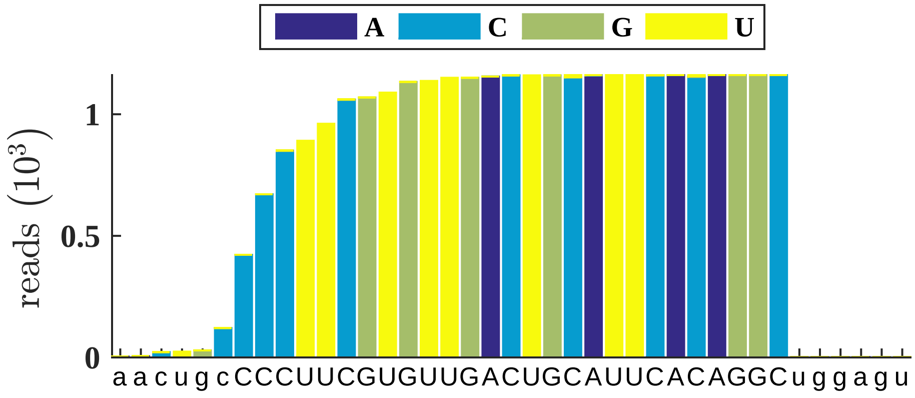

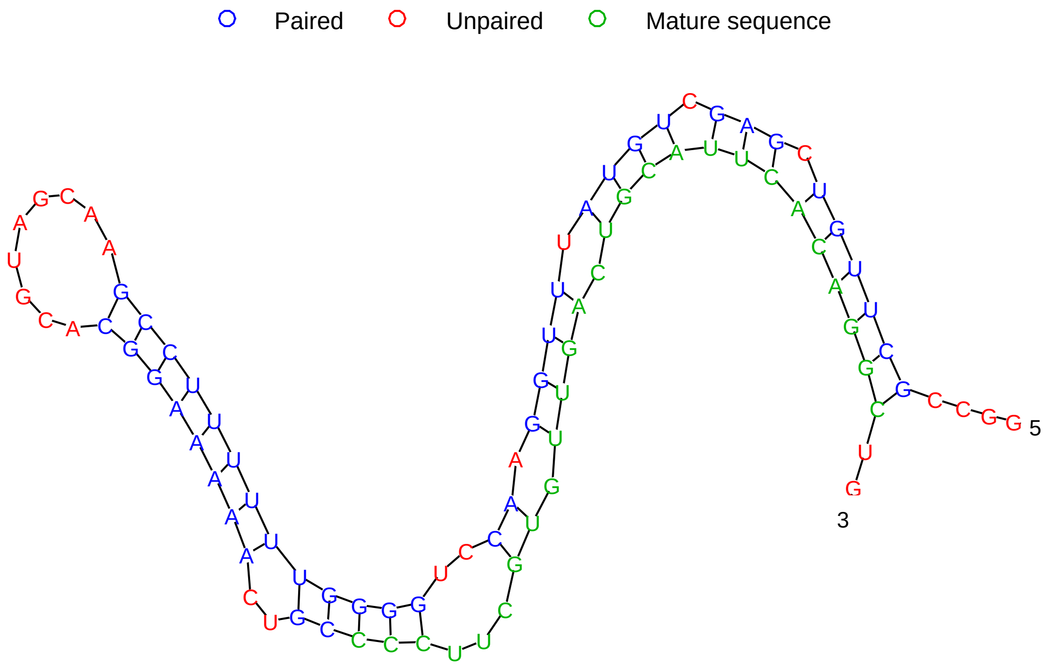

Stem loop (UMD3.1): chr28:11165150-11165241
Mature (UMD3.1): chr28:11165152-11165178
Mature seq len: 27
Total raw counts (9 samples): 1179
Average raw counts: 131
Strand: Reverse
Orientation: 3p
Minimum free energy: -28.90

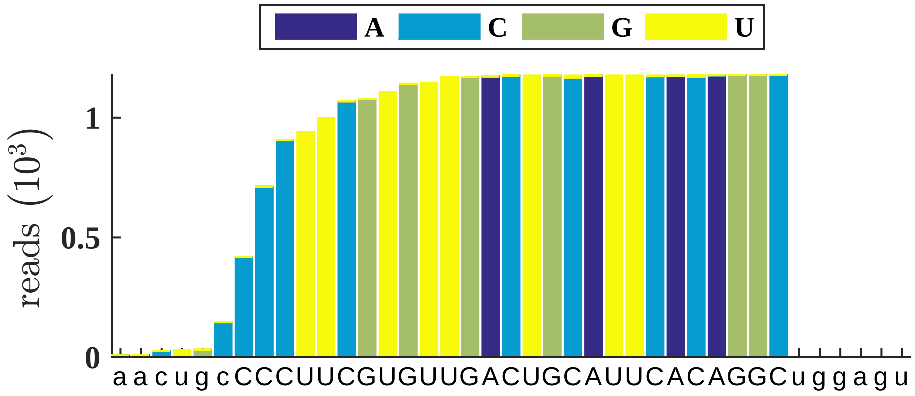

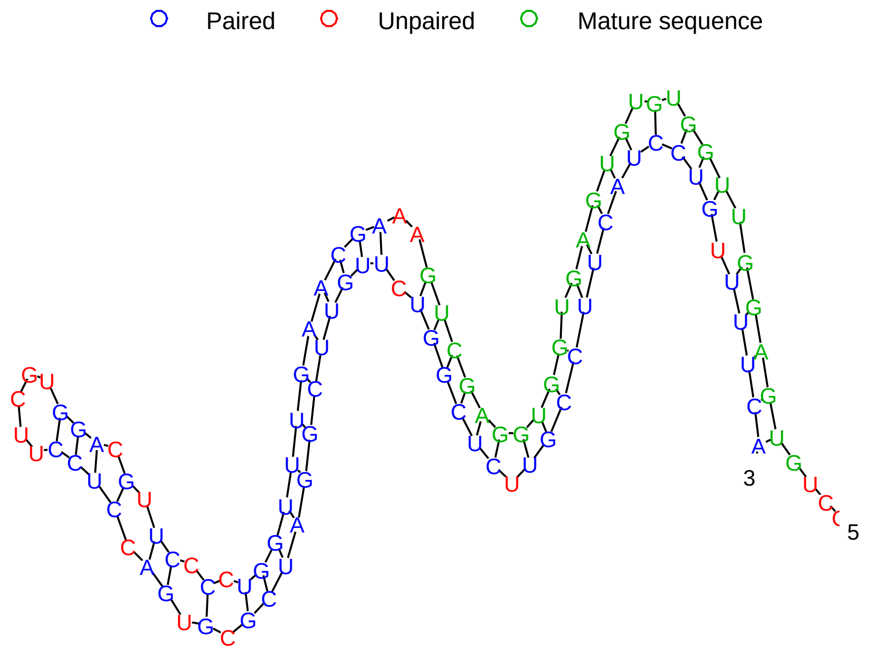

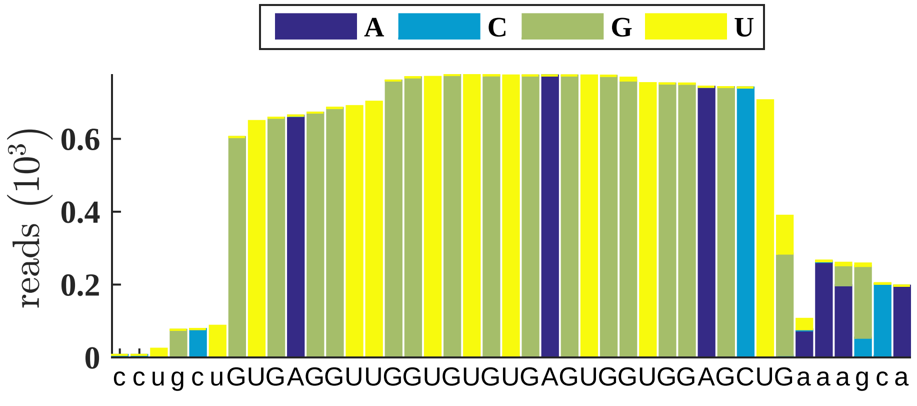

Stem loop (UMD3.1): chr28:25631925-25632035
Mature (UMD3.1): chr28:25631928-25631956
Mature seq len: 29
Total raw counts (9 samples): 775
Average raw counts: 87
Strand: Forward
Orientation: 5p
Minimum free energy: -34.10

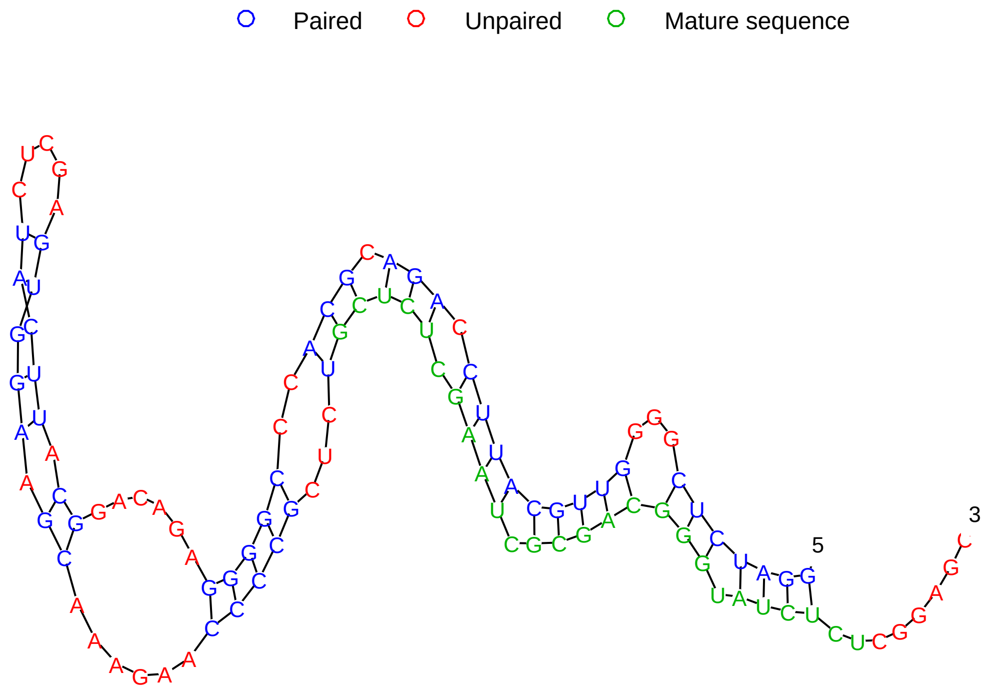

Stem loop (UMD3.1): chr28:31993178-31993285
Mature (UMD3.1): chr28:31993184-31993209
Mature seq len: 26
Total raw counts (9 samples): 3912
Average raw counts: 435
Strand: Reverse
Orientation: 3p
Minimum free energy: -32.40

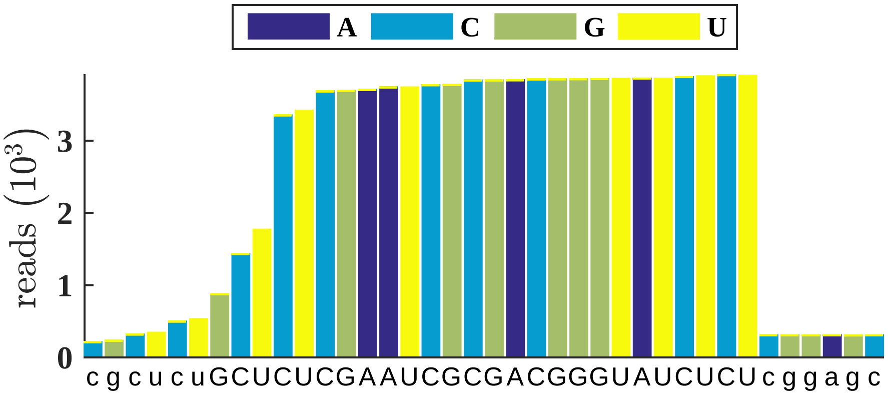

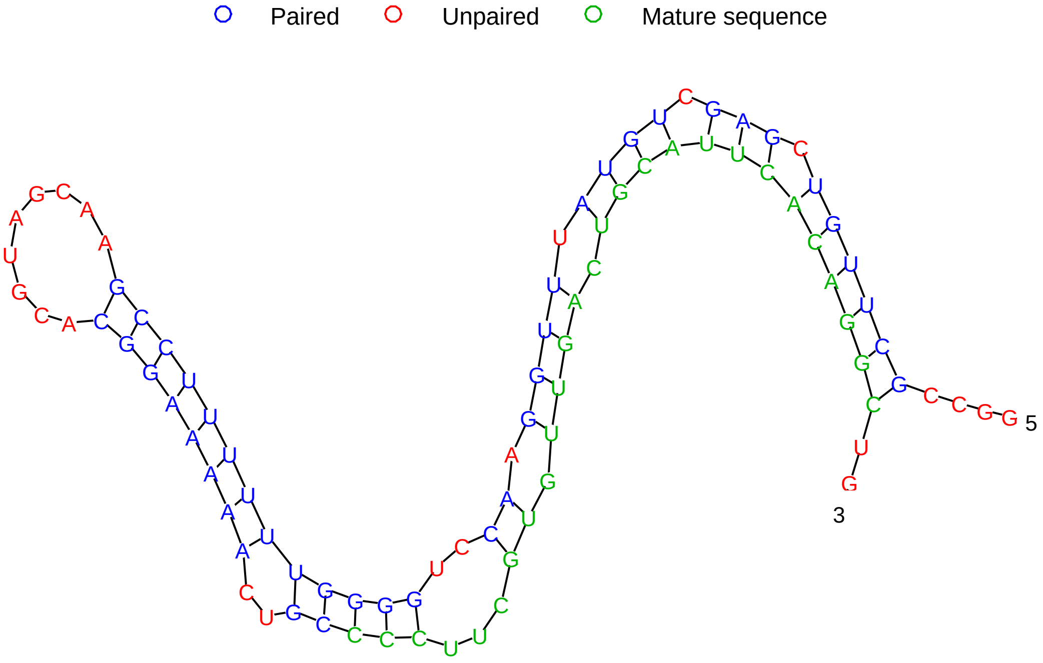

Stem loop (UMD3.1): chr28:31998418-31998509
Mature (UMD3.1): chr28:31998420-31998446
Mature seq len: 27
Total raw counts (9 samples): 1230
Average raw counts: 137
Strand: Reverse
Orientation: 3p
Minimum free energy: -28.90

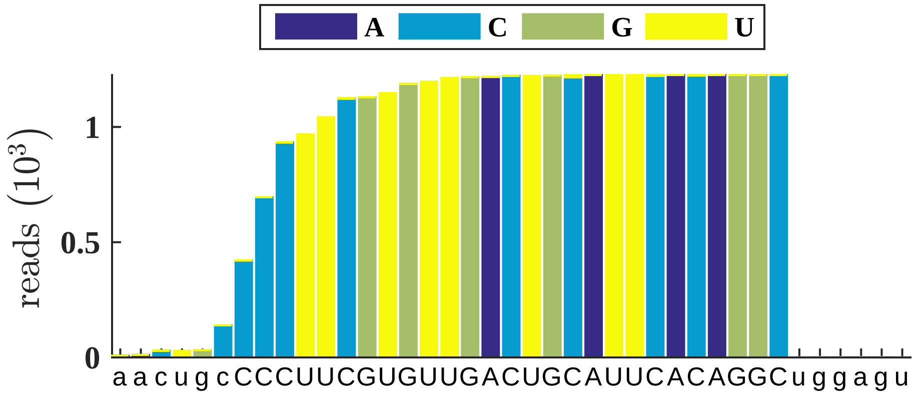

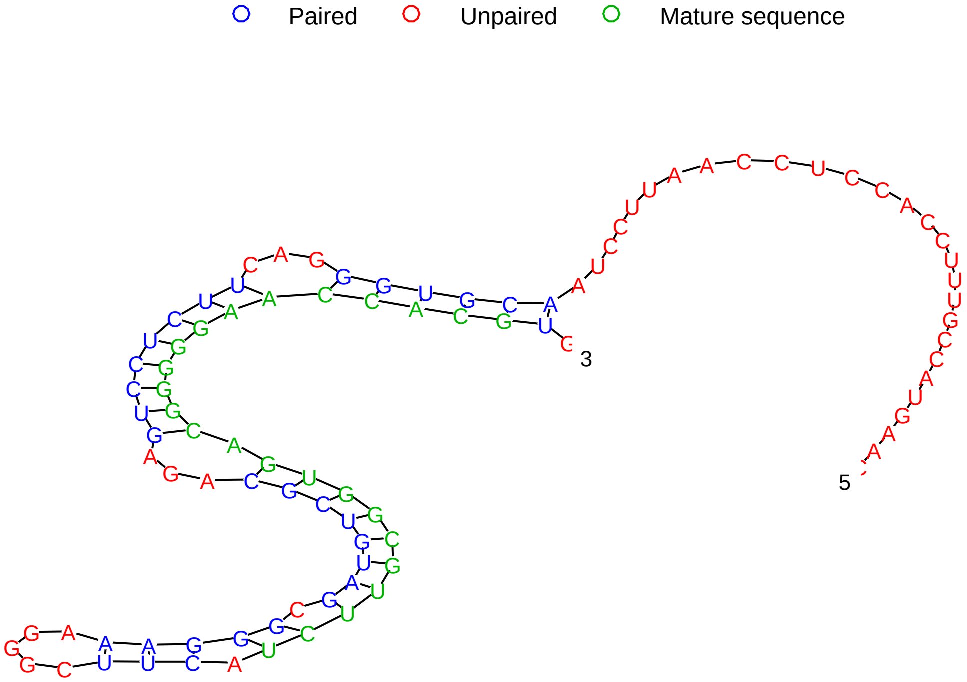

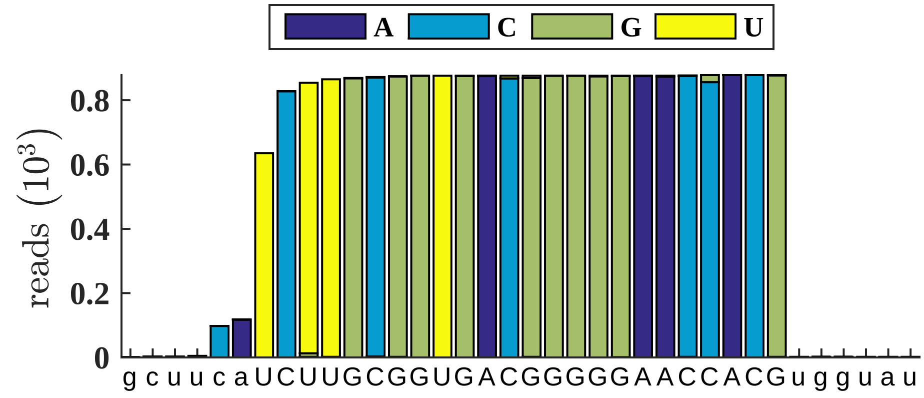

Stem loop (UMD3.1): chr28:39357756-39357852
Mature (UMD3.1): chr28:39357758-39357781
Mature seq len: 24
Total raw counts (9 samples): 895
Average raw counts: 100
Strand: Reverse
Orientation: 3p
Minimum free energy: -28.20

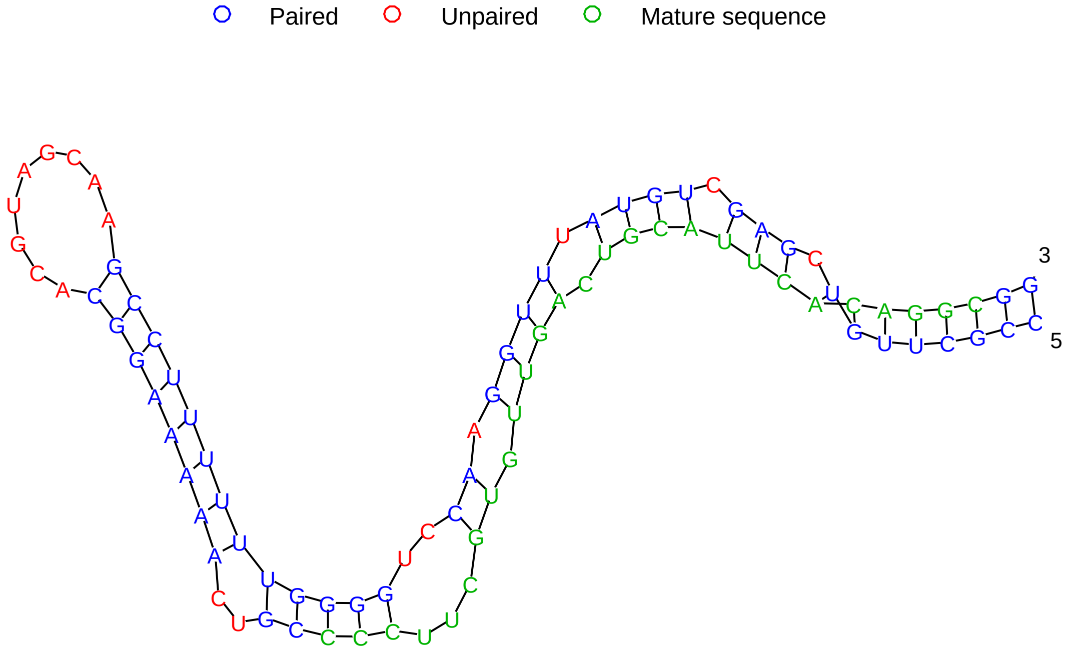

Stem loop (UMD3.1): chr28:43054426-43054515
Mature (UMD3.1): chr28:43054428-43054454
Mature seq len: 27
Total raw counts (9 samples): 1234
Average raw counts: 138
Strand: Reverse
Orientation: 3p
Minimum free energy: -33.20

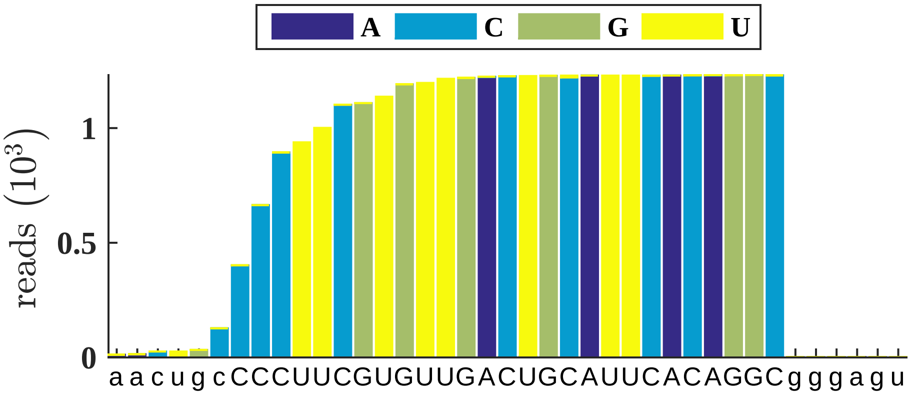

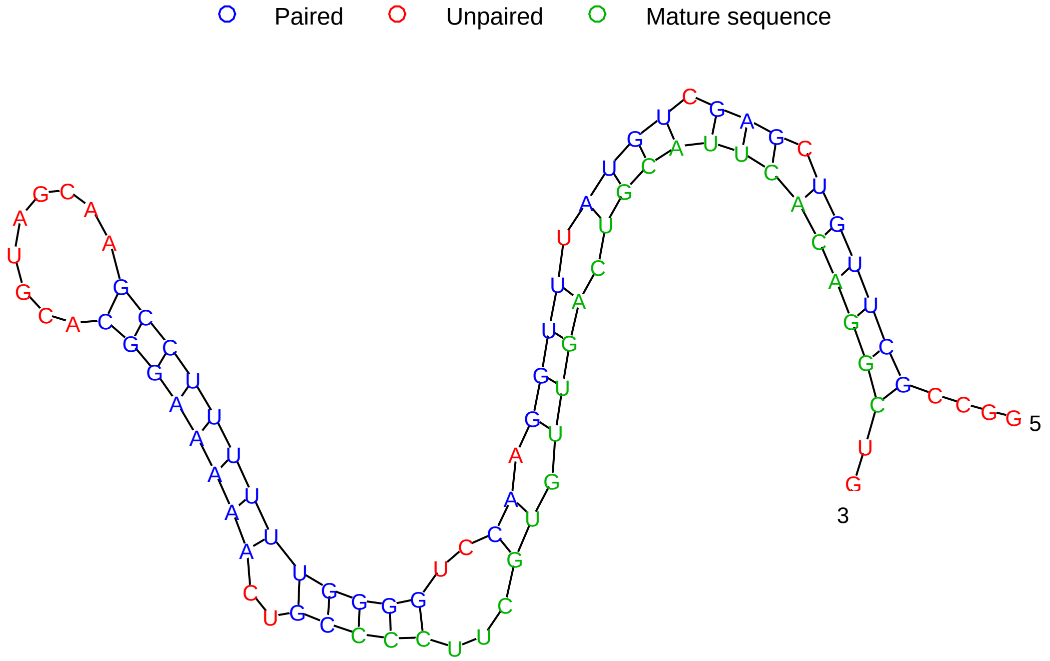

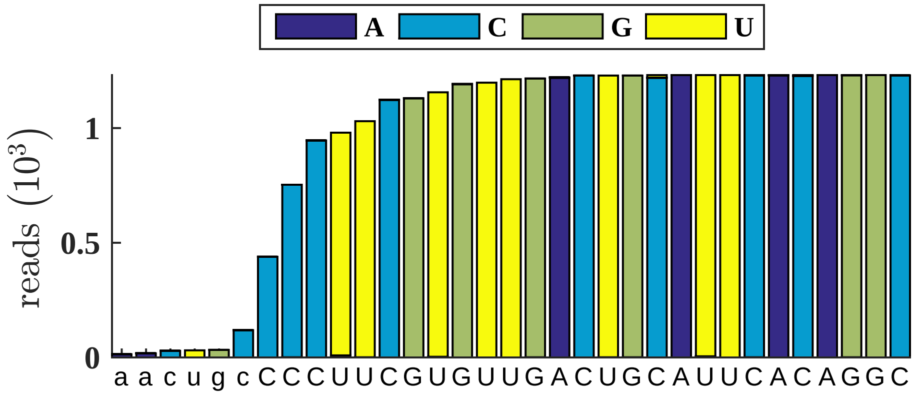

Stem loop (UMD3.1): chr28:43062161-43062252
Mature (UMD3.1): chr28:43062163-43062189
Mature seq len: 27
Total raw counts (9 samples): 1232
Average raw counts: 137
Strand: Reverse
Orientation: 3p
Minimum free energy: -28.90

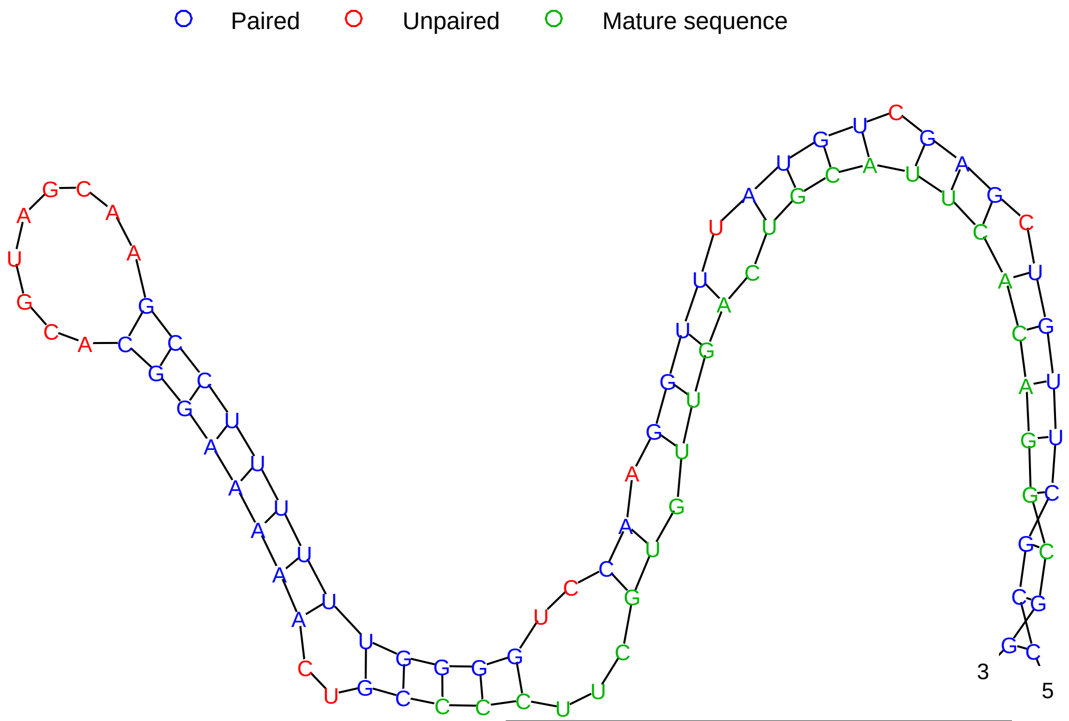

Stem loop (UMD3.1): chr28:43070489-43070578
Mature (UMD3.1): chr28:43070491-43070517
Mature seq len: 27
Total raw counts (9 samples): 1181
Average raw counts: 132
Strand: Reverse
Orientation: 3p
Minimum free energy: -33.20

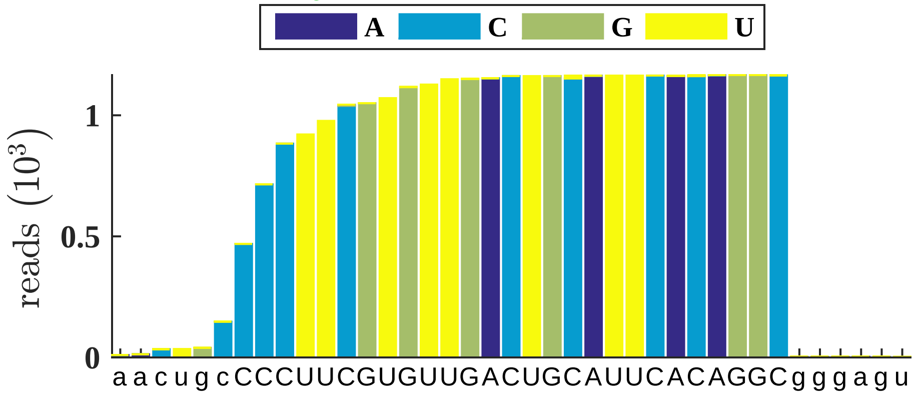

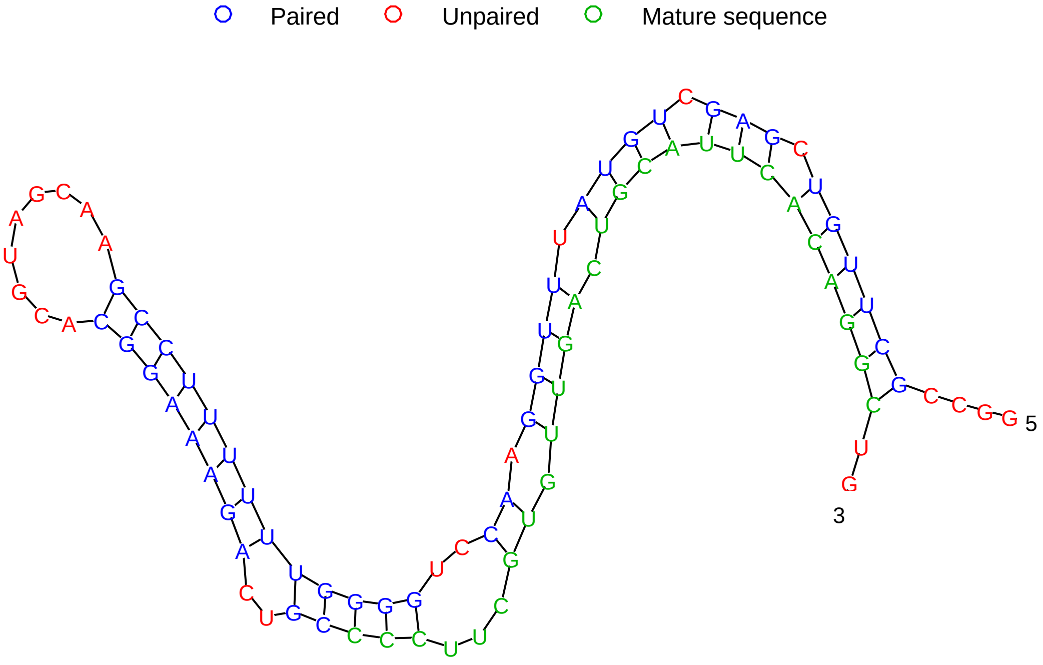

Stem loop (UMD3.1): chr28:43081839-43081930
Mature (UMD3.1): chr28:43081841-43081867
Mature seq len: 27
Total raw counts (9 samples): 1221
Average raw counts: 136
Strand: Reverse
Orientation: 3p
Minimum free energy: -29.00

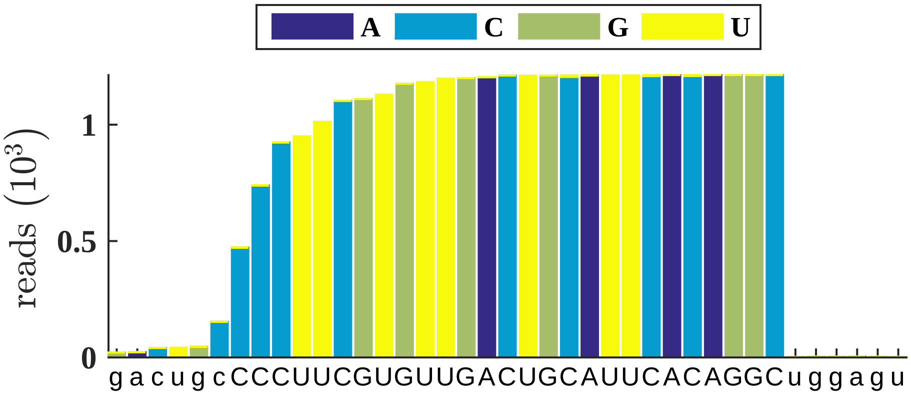

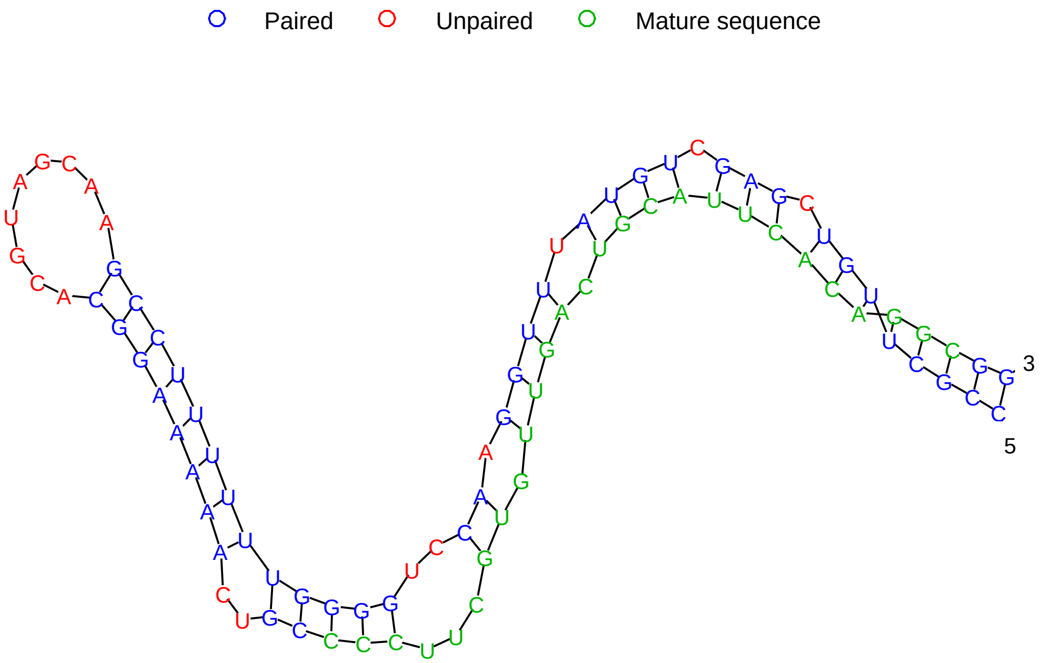

Stem loop (UMD3.1): chr28:43342743-43342832
Mature (UMD3.1): chr28:43342804-43342830
Mature seq len: 27
Total raw counts (9 samples): 1138
Average raw counts: 127
Strand: Forward
Orientation: 3p
Minimum free energy: -33.20

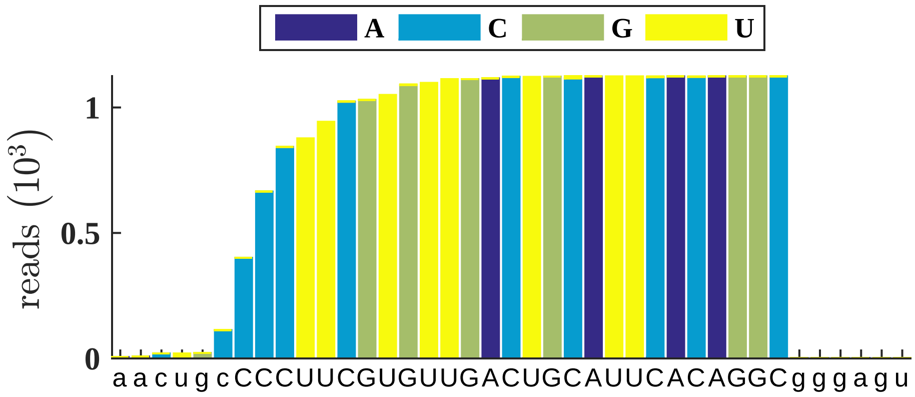

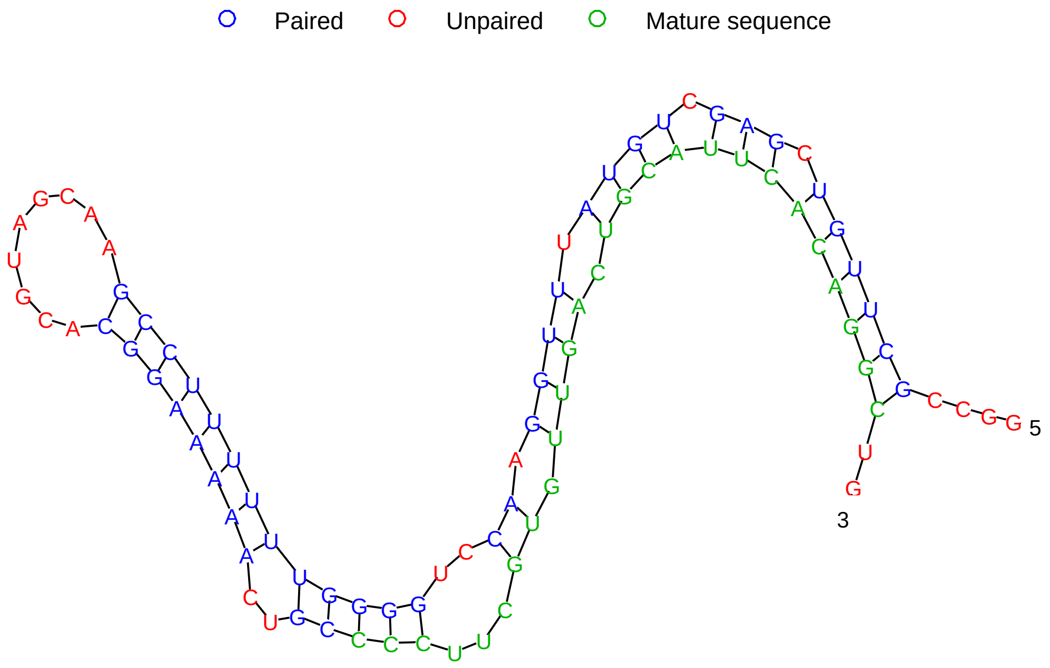

Stem loop (UMD3.1): chr29:31047048-31047139
Mature (UMD3.1): chr29:31047050-31047076
Mature seq len: 27
Total raw counts (9 samples): 1147
Average raw counts: 128
Strand: Reverse
Orientation: 3p
Minimum free energy: -28.90

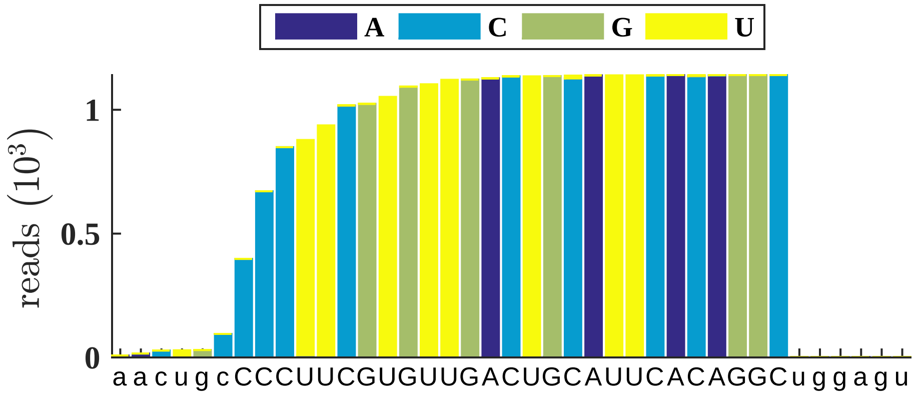

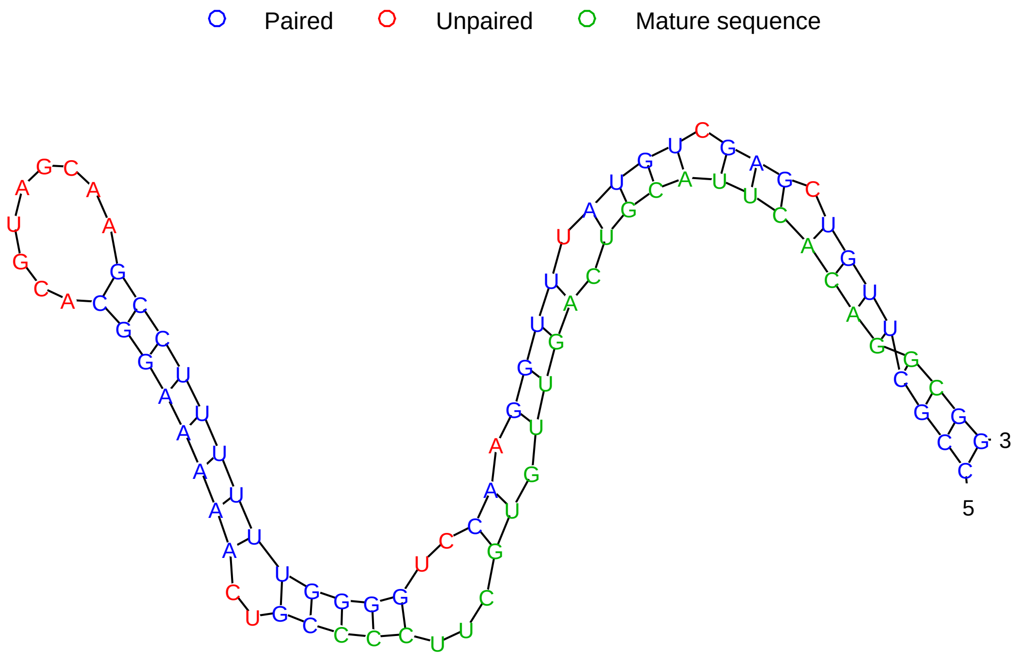

Stem loop (UMD3.1): chr3:13911512-13911601
Mature (UMD3.1): chr3:13911514-13911540
Mature seq len: 27
Total raw counts (9 samples): 1230
Average raw counts: 137
Strand: Reverse
Orientation: 3p
Minimum free energy: -33.20

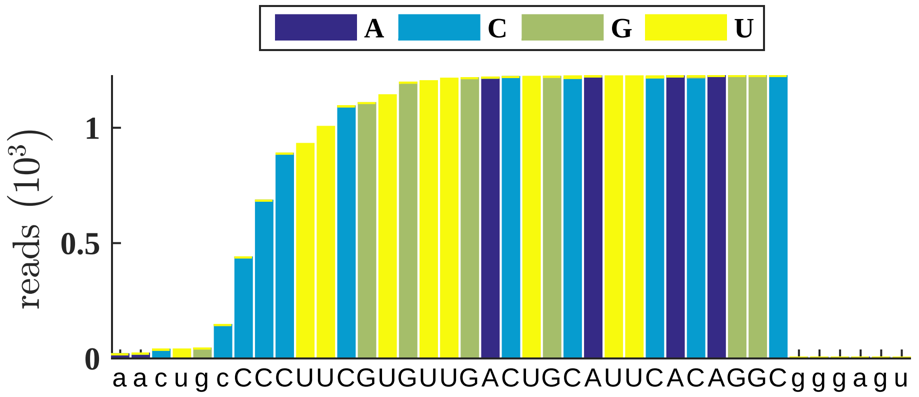

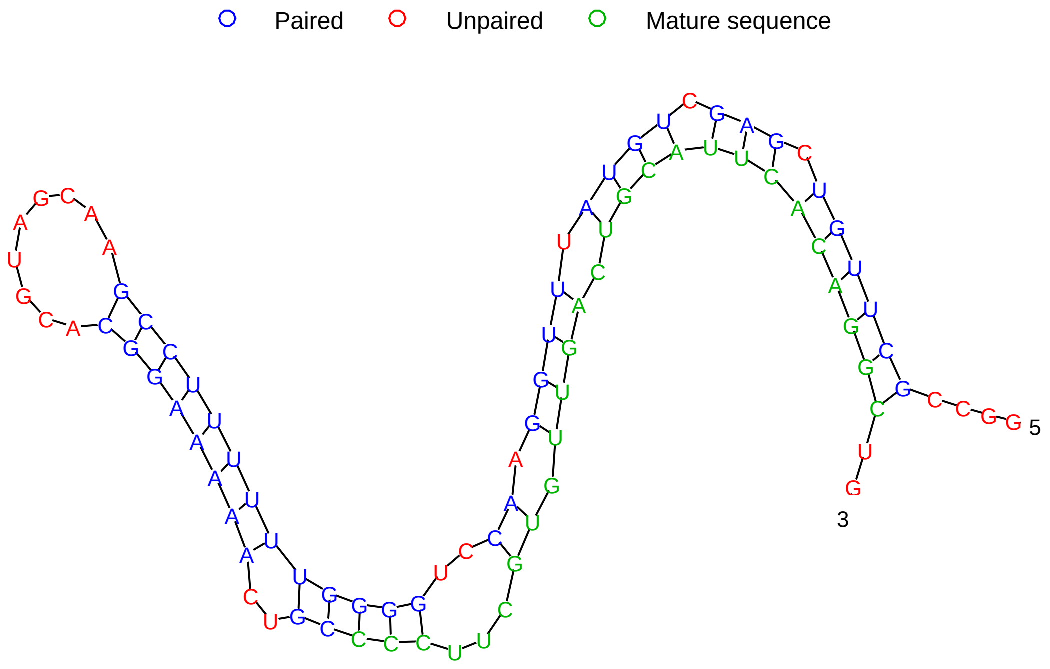

Stem loop (UMD3.1): chr3:13916307-13916398
Mature (UMD3.1): chr3:13916309-13916335
Mature seq len: 27
Total raw counts (9 samples): 1192
Average raw counts: 133
Strand: Reverse
Orientation: 3p
Minimum free energy: -28.90

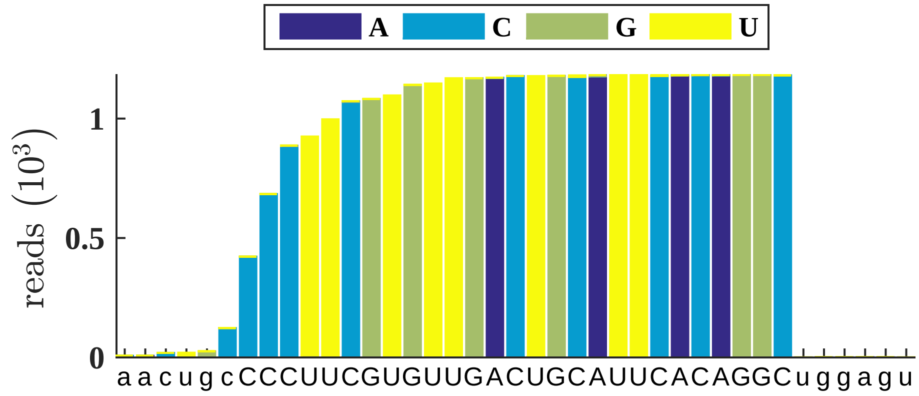

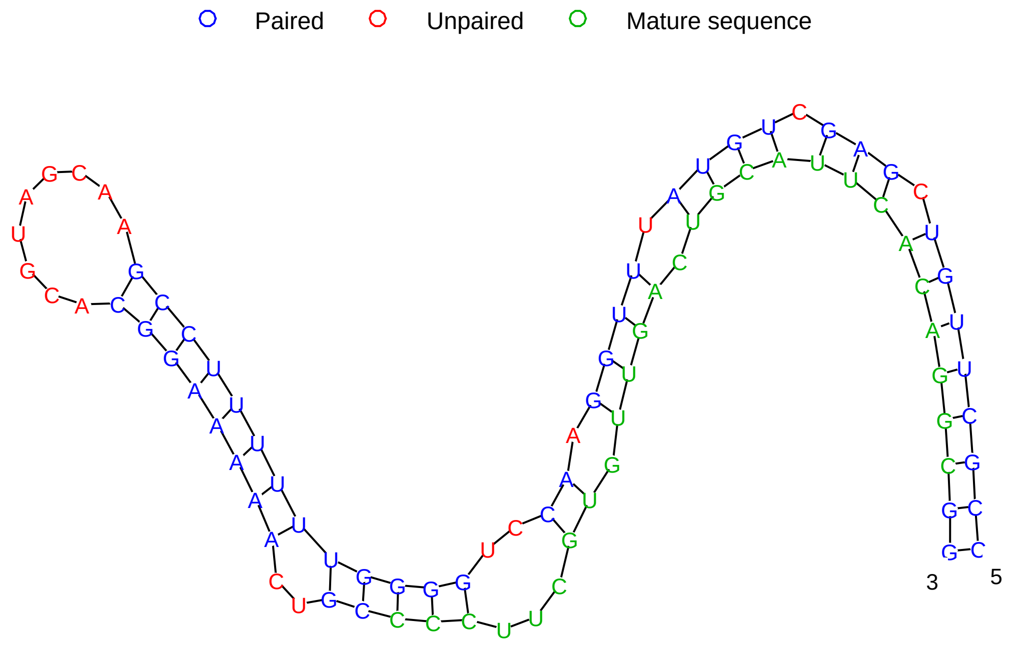

Stem loop (UMD3.1): chr3:13940206-13940295
Mature (UMD3.1): chr3:13940208-13940234
Mature seq len: 27
Total raw counts (9 samples): 1180
Average raw counts: 132
Strand: Reverse
Orientation: 3p
Minimum free energy: -33.20

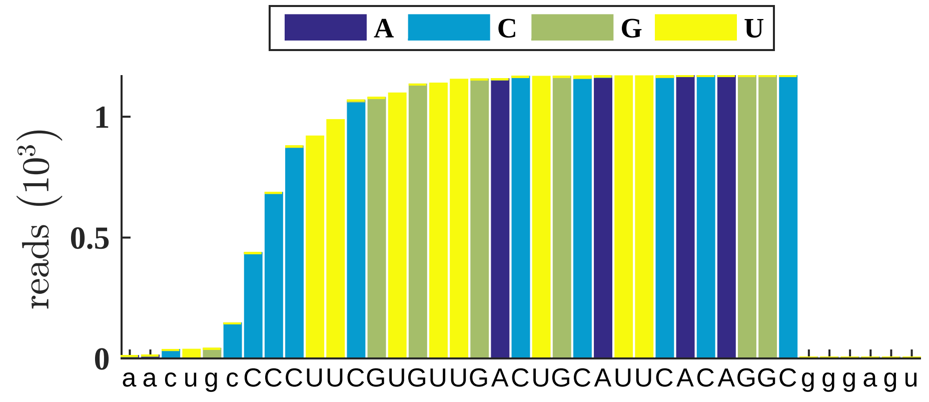

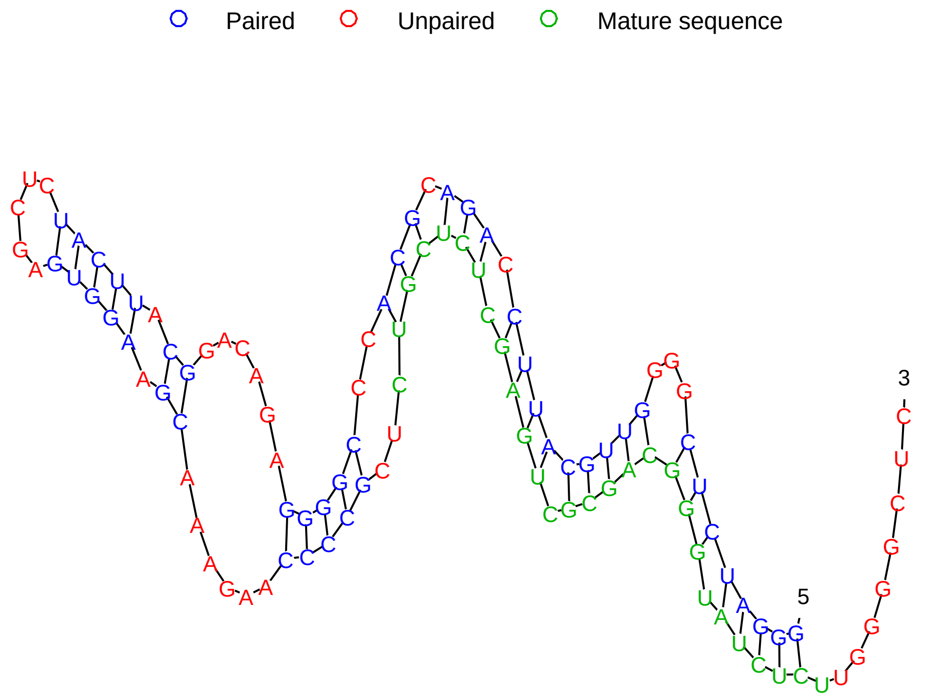

Stem loop (UMD3.1): chr3:29608508-29608618
Mature (UMD3.1): chr3:29608583-29608610
Mature seq len: 28
Total raw counts (9 samples): 12371
Average raw counts: 1375
Strand: Forward
Orientation: 3p
Minimum free energy: -35.50

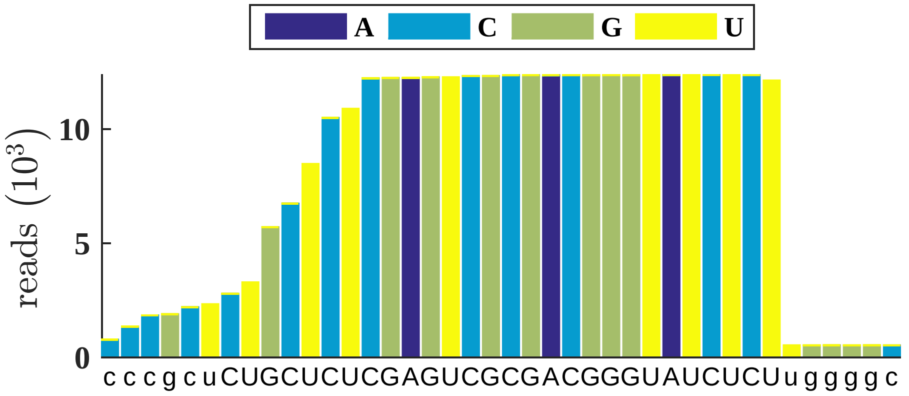

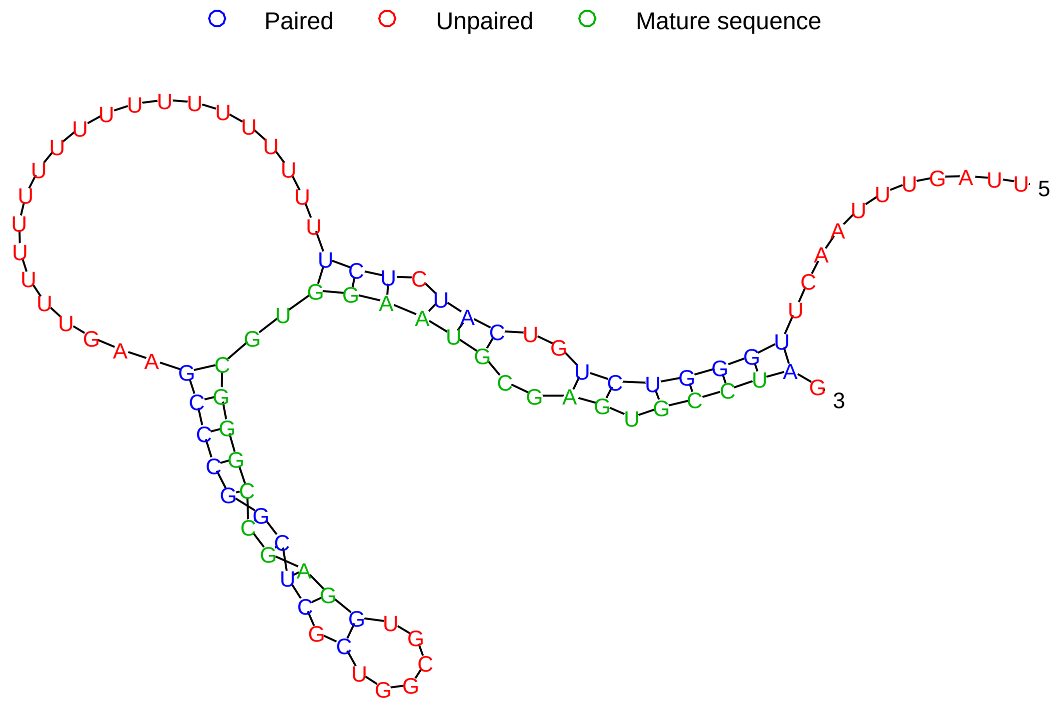

Stem loop (UMD3.1): chr3:63639299-63639393
Mature (UMD3.1): chr3:63639301-63639326
Mature seq len: 26
Total raw counts (9 samples): 3091
Average raw counts: 344
Strand: Reverse
Orientation: 3p
Minimum free energy: -27.80

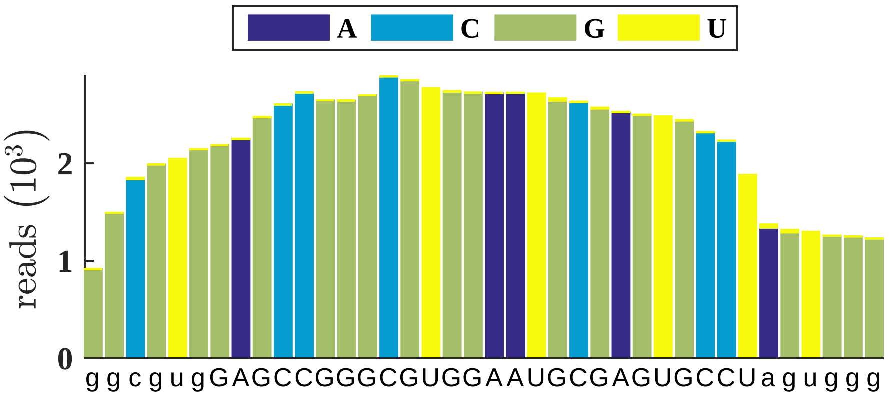

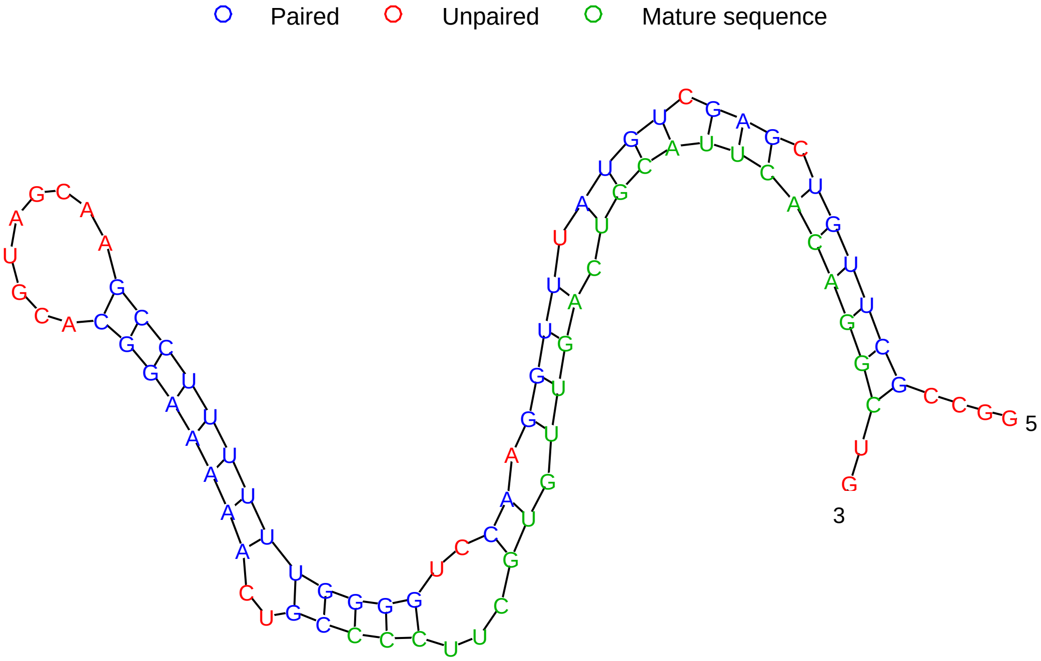

Stem loop (UMD3.1): chr3:64399493-64399584
Mature (UMD3.1): chr3:64399556-64399582
Mature seq len: 27
Total raw counts (9 samples): 1221
Average raw counts: 136
Strand: Forward
Orientation: 3p
Minimum free energy: -28.90

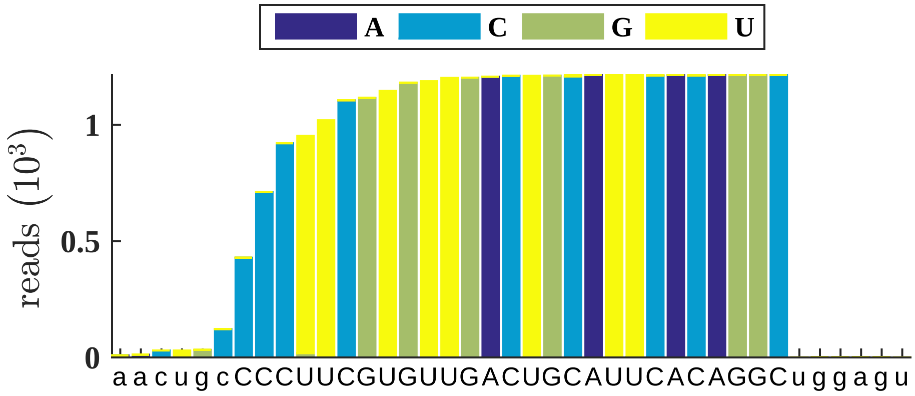

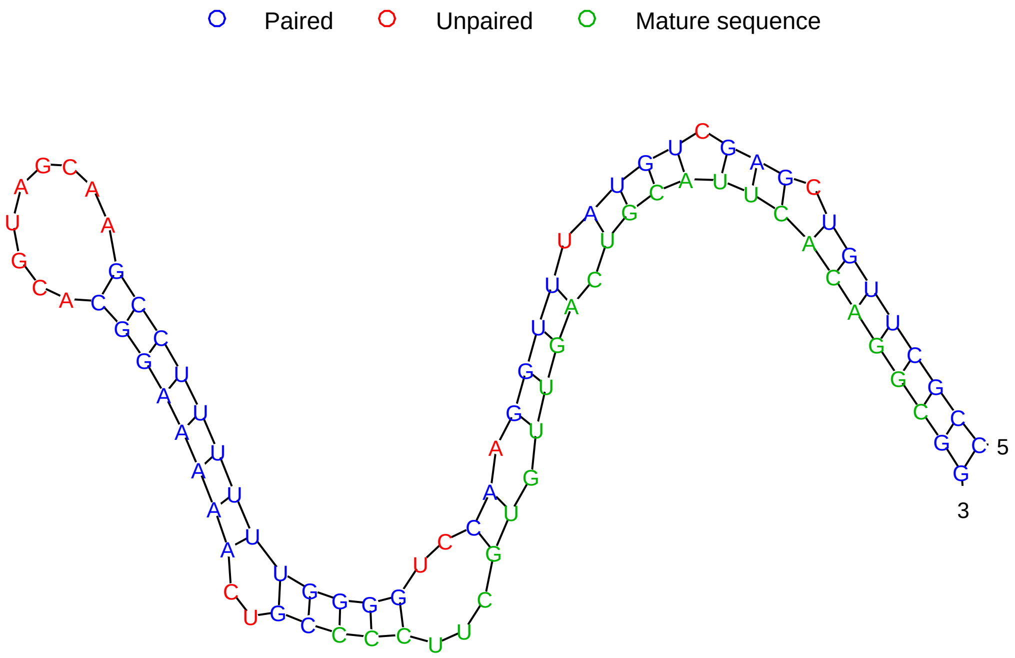

Stem loop (UMD3.1): chr3:74981309-74981398
Mature (UMD3.1): chr3:74981311-74981337
Mature seq len: 27
Total raw counts (9 samples): 1240
Average raw counts: 138
Strand: Reverse
Orientation: 3p
Minimum free energy: -33.20

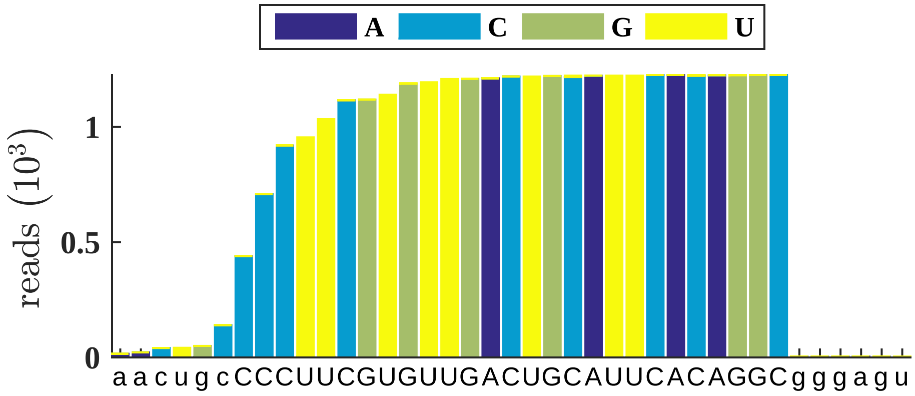

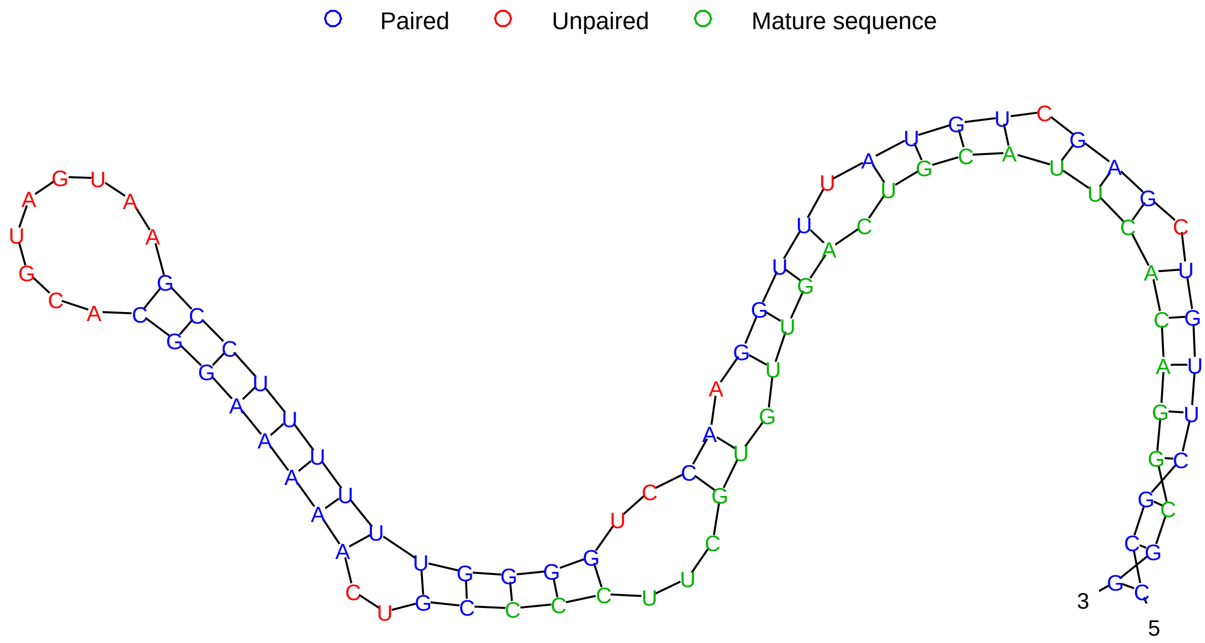

Stem loop (UMD3.1): chr3:75990457-75990546
Mature (UMD3.1): chr3:75990459-75990485
Mature seq len: 27
Total raw counts (9 samples): 1159
Average raw counts: 129
Strand: Reverse
Orientation: 3p
Minimum free energy: -33.20

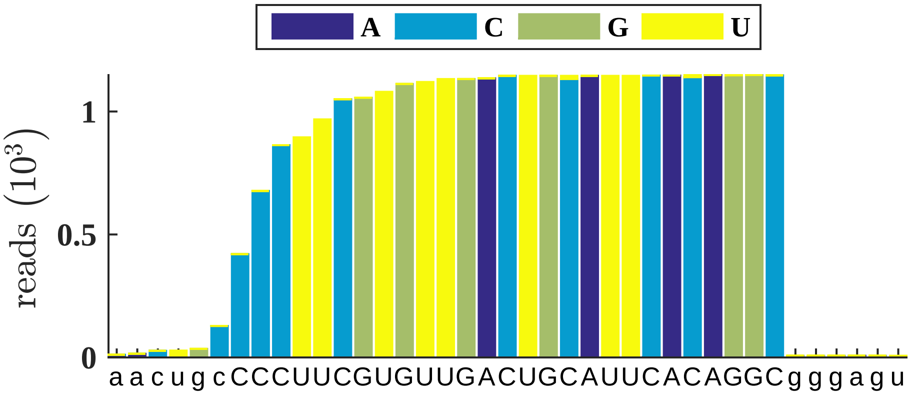

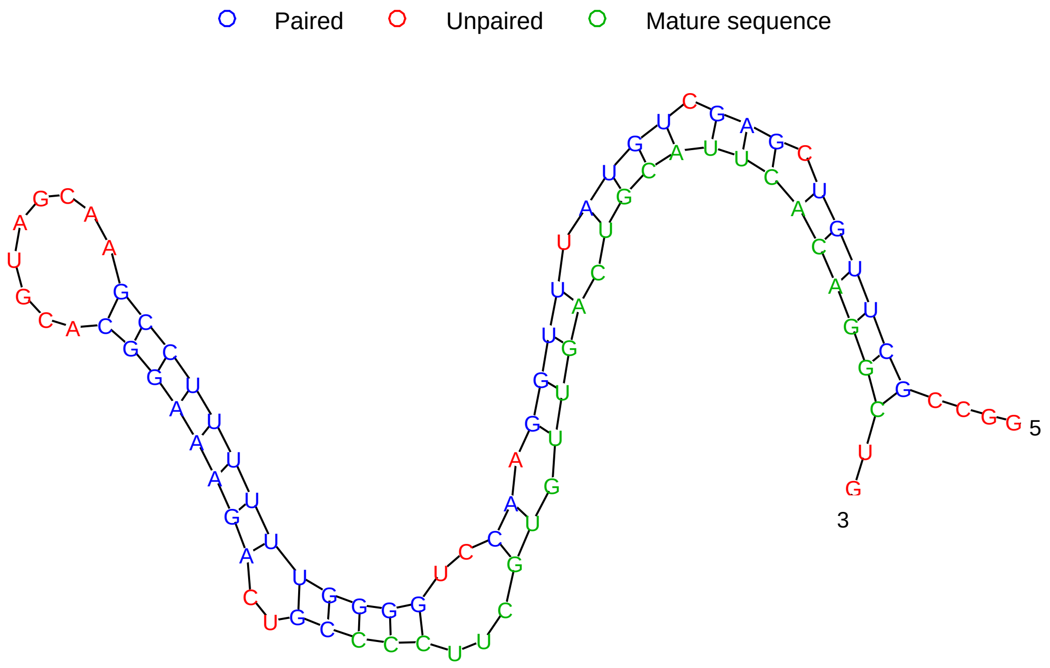

Stem loop (UMD3.1): chr3:82757389-82757480
Mature (UMD3.1): chr3:82757452-82757478
Mature seq len: 27
Total raw counts (9 samples): 1196
Average raw counts: 133
Strand: Forward
Orientation: 3p
Minimum free energy: -29.00

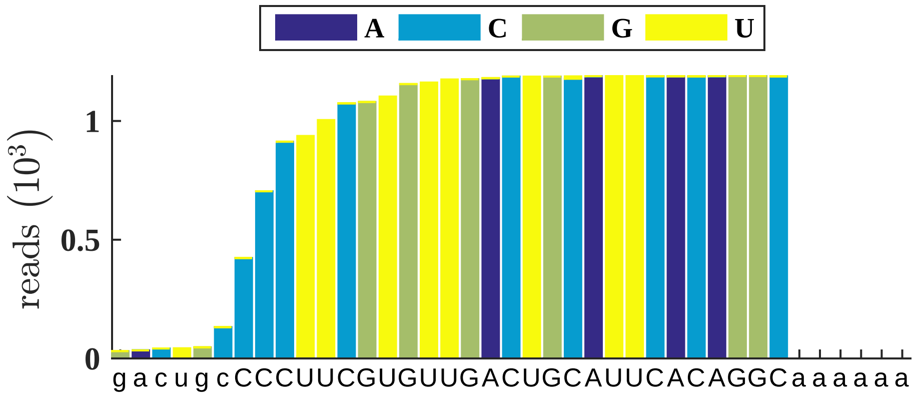

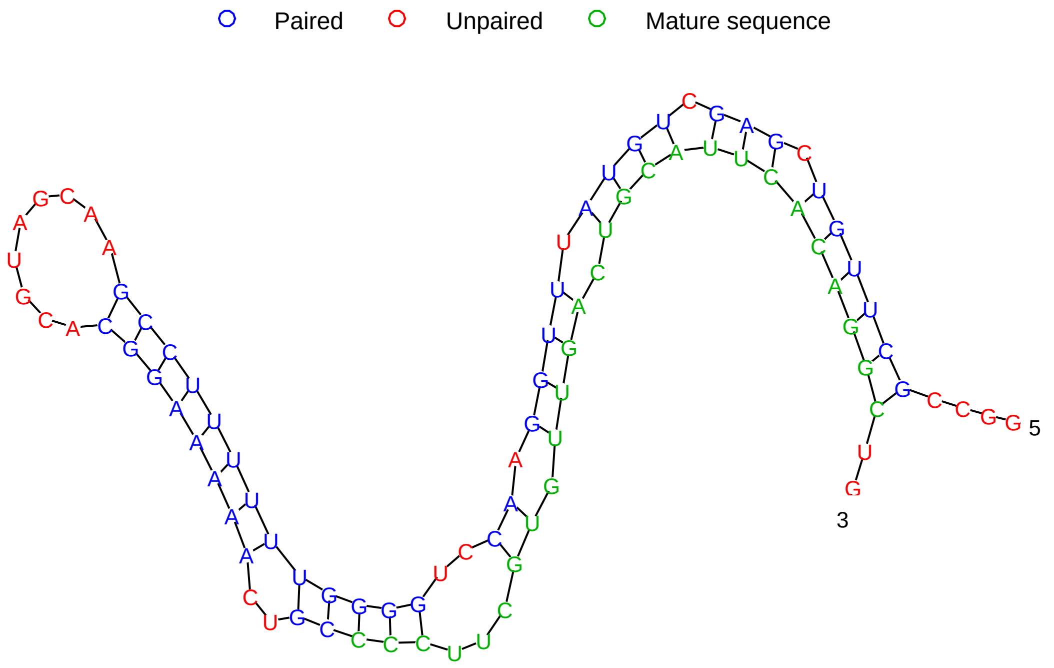

Stem loop (UMD3.1): chr3:82784298-82784389
Mature (UMD3.1): chr3:82784361-82784387
Mature seq len: 27
Total raw counts (9 samples): 1159
Average raw counts: 129
Strand: Forward
Orientation: 3p
Minimum free energy: -28.90

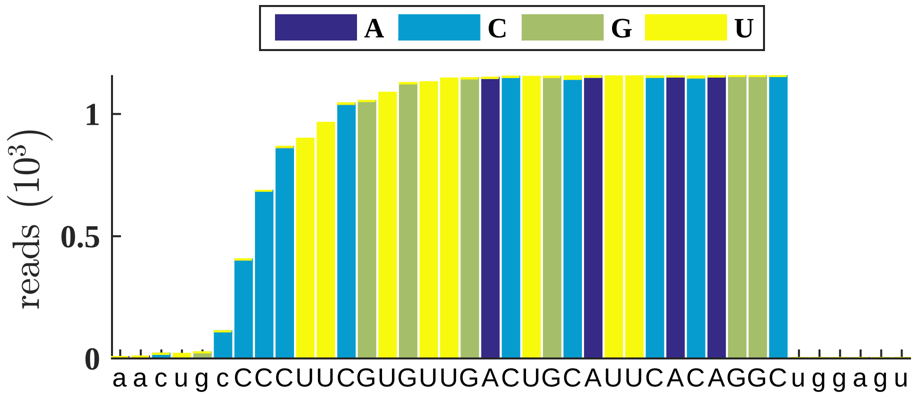

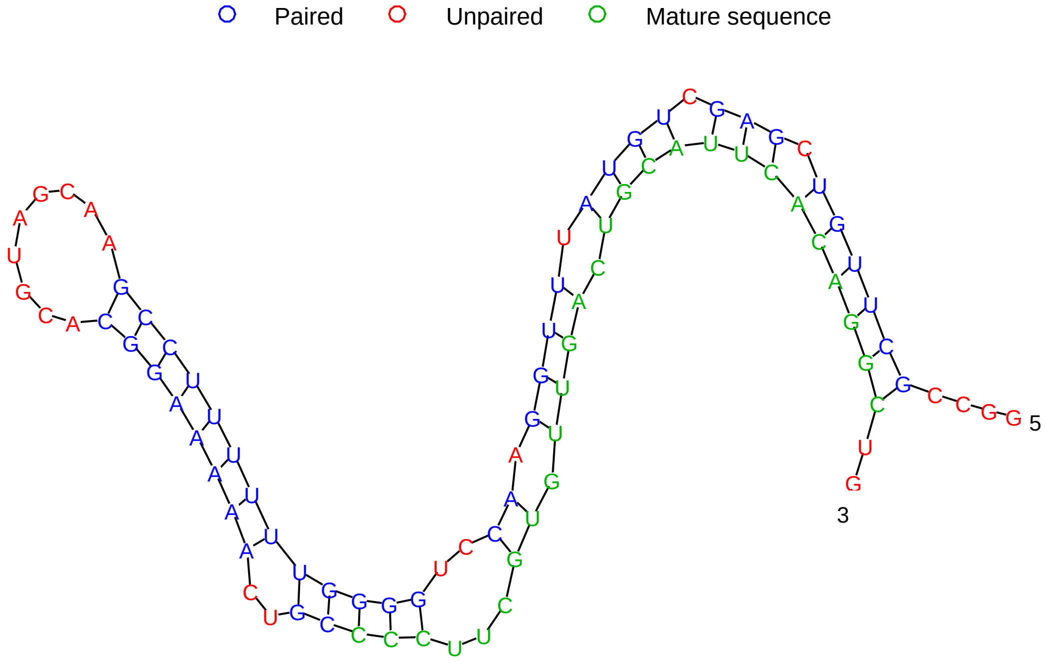

Stem loop (UMD3.1): chr3:82787056-82787147
Mature (UMD3.1): chr3:82787119-82787145
Mature seq len: 27
Total raw counts (9 samples): 1214
Average raw counts: 135
Strand: Forward
Orientation: 3p
Minimum free energy: -28.90

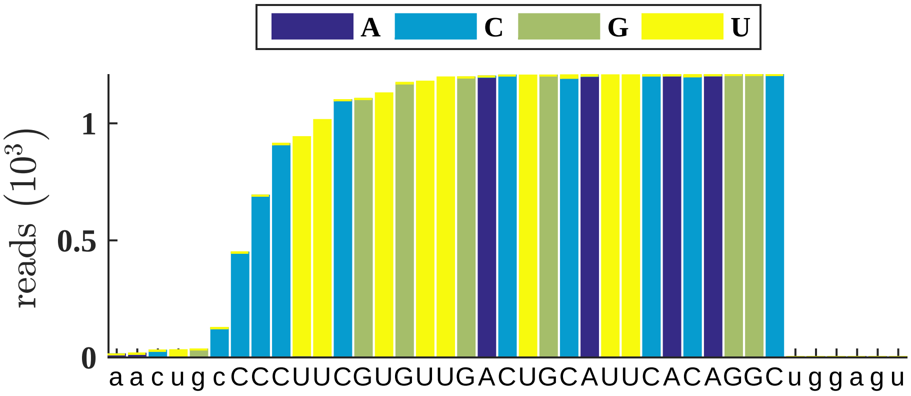

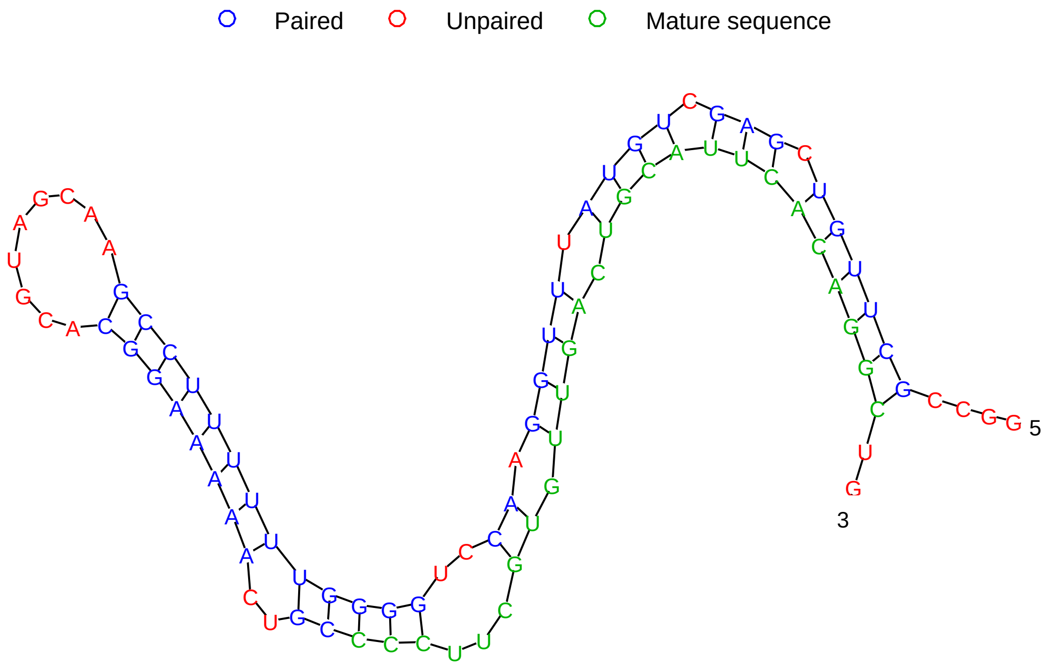

Stem loop (UMD3.1): chr3:82789838-82789929
Mature (UMD3.1): chr3:82789901-82789927
Mature seq len: 27
Total raw counts (9 samples): 1192
Average raw counts: 133
Strand: Forward
Orientation: 3p
Minimum free energy: -28.90

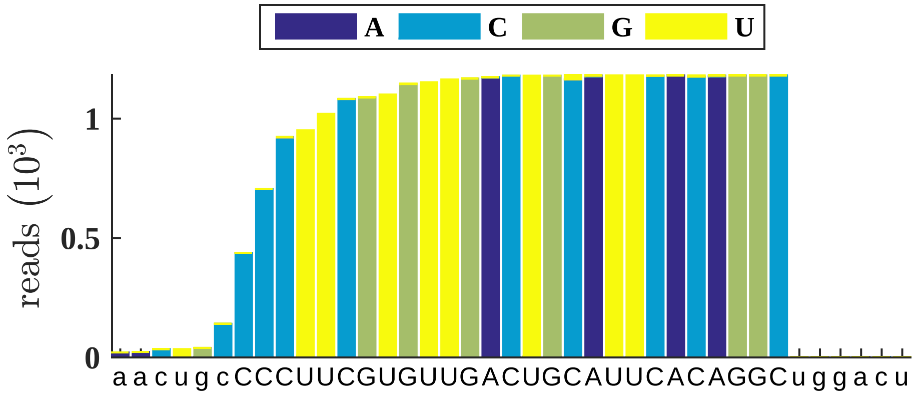

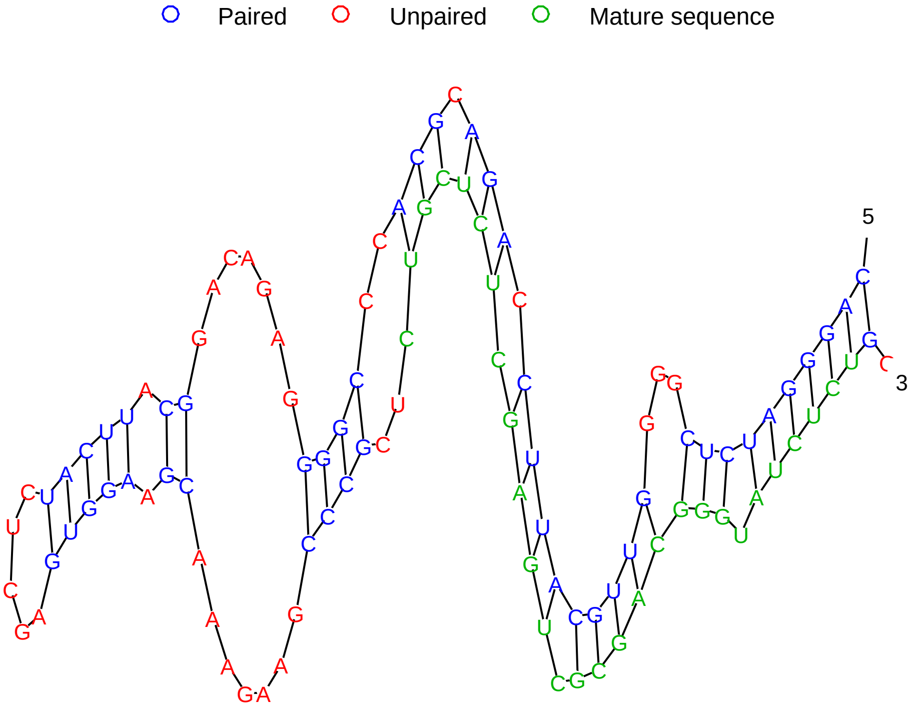

Stem loop (UMD3.1): chr3:82790517-82790623
Mature (UMD3.1): chr3:82790594-82790621
Mature seq len: 28
Total raw counts (9 samples): 11573
Average raw counts: 1286
Strand: Forward
Orientation: 3p
Minimum free energy: -37.30

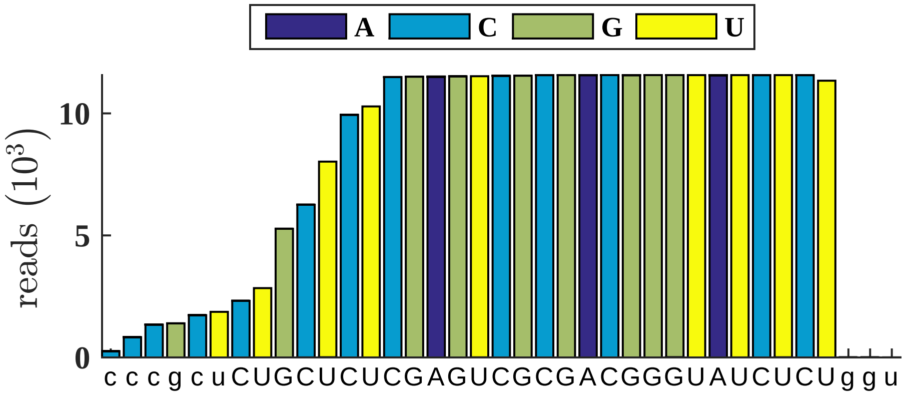

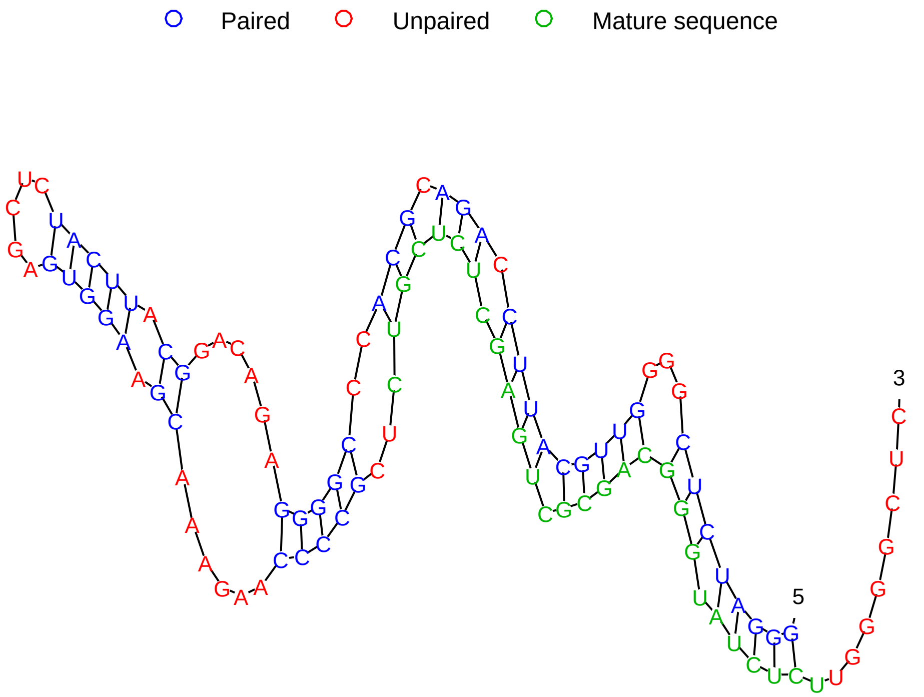

Stem loop (UMD3.1): chr3:82846495-82846605
Mature (UMD3.1): chr3:82846503-82846530
Mature seq len: 28
Total raw counts (9 samples): 12883
Average raw counts: 1432
Strand: Reverse
Orientation: 3p
Minimum free energy: -35.50

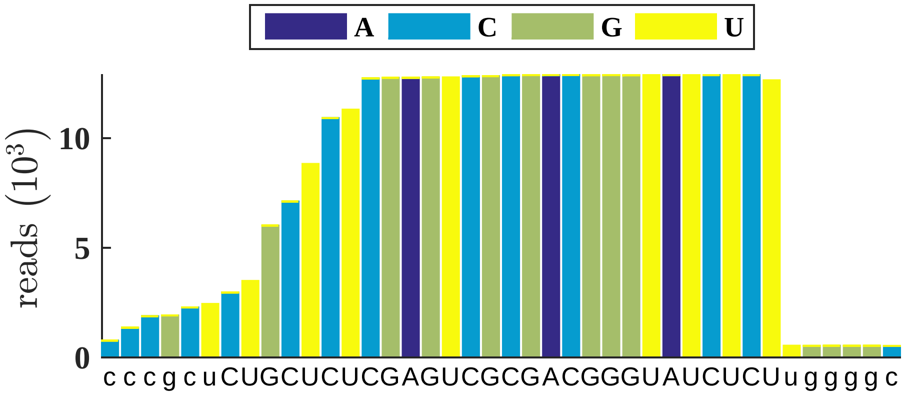

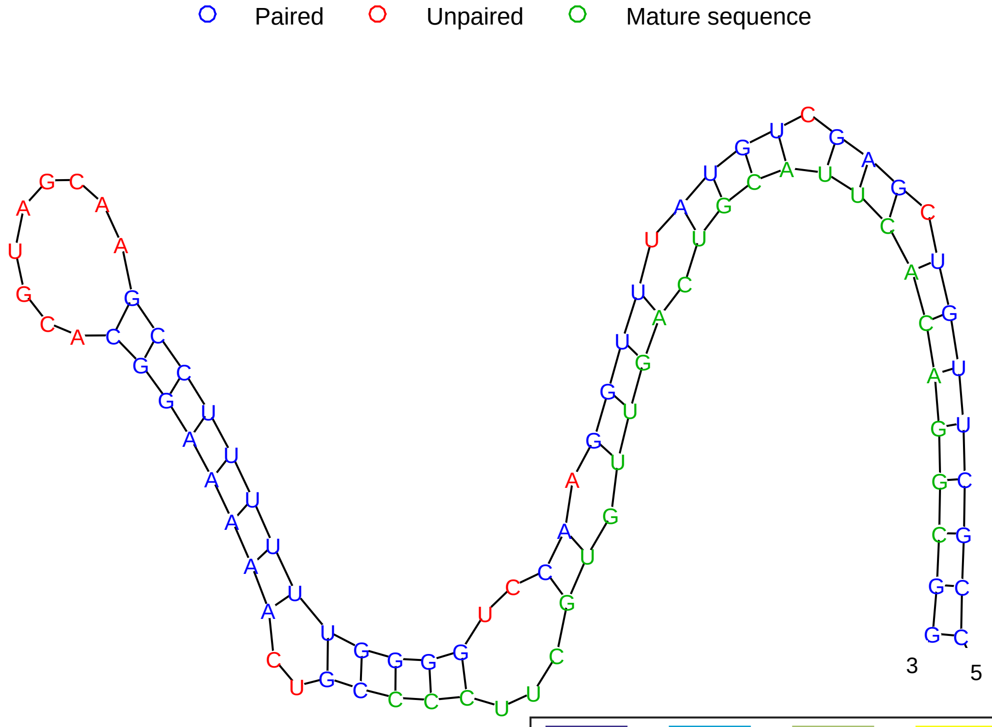

Stem loop (UMD3.1): chr3:82847808-82847897
Mature (UMD3.1): chr3:82847869-82847895
Mature seq len: 27
Total raw counts (9 samples): 1132
Average raw counts: 126
Strand: Forward
Orientation: 3p
Minimum free energy: -33.20

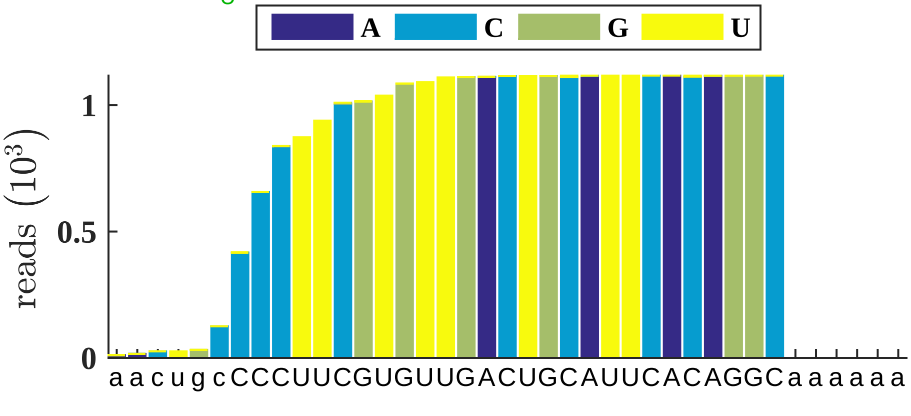

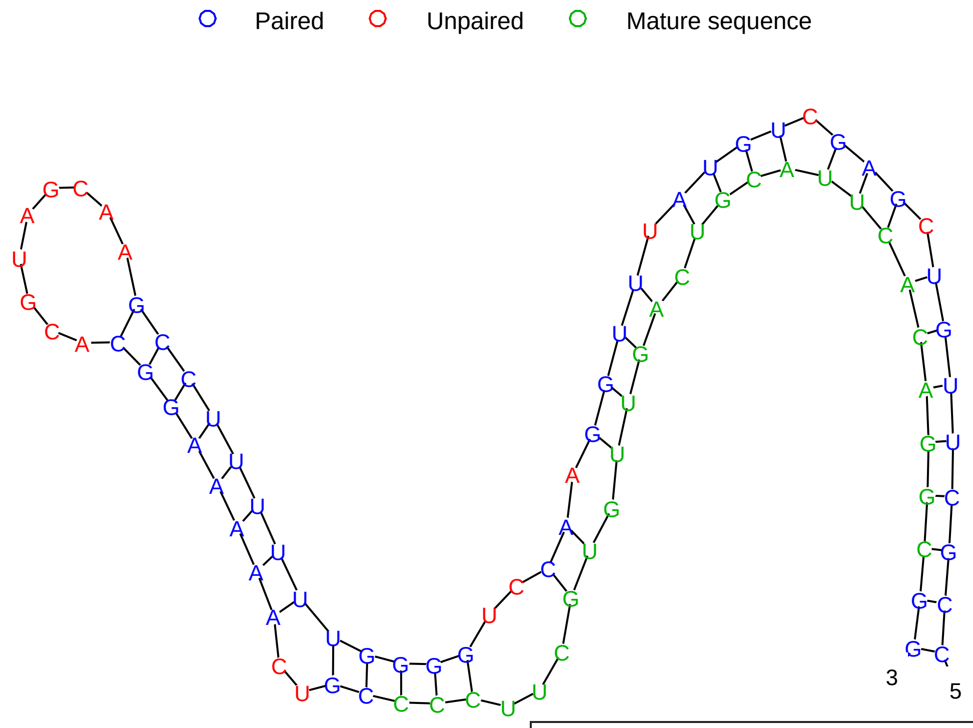

Stem loop (UMD3.1): chr3:82851165-82851254
Mature (UMD3.1): chr3:82851226-82851252
Mature seq len: 27
Total raw counts (9 samples): 1237
Average raw counts: 138
Strand: Forward
Orientation: 3p
Minimum free energy: -33.20

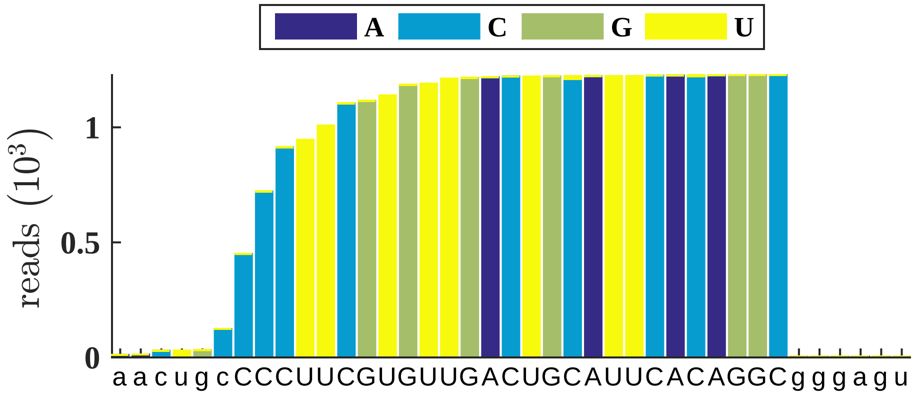

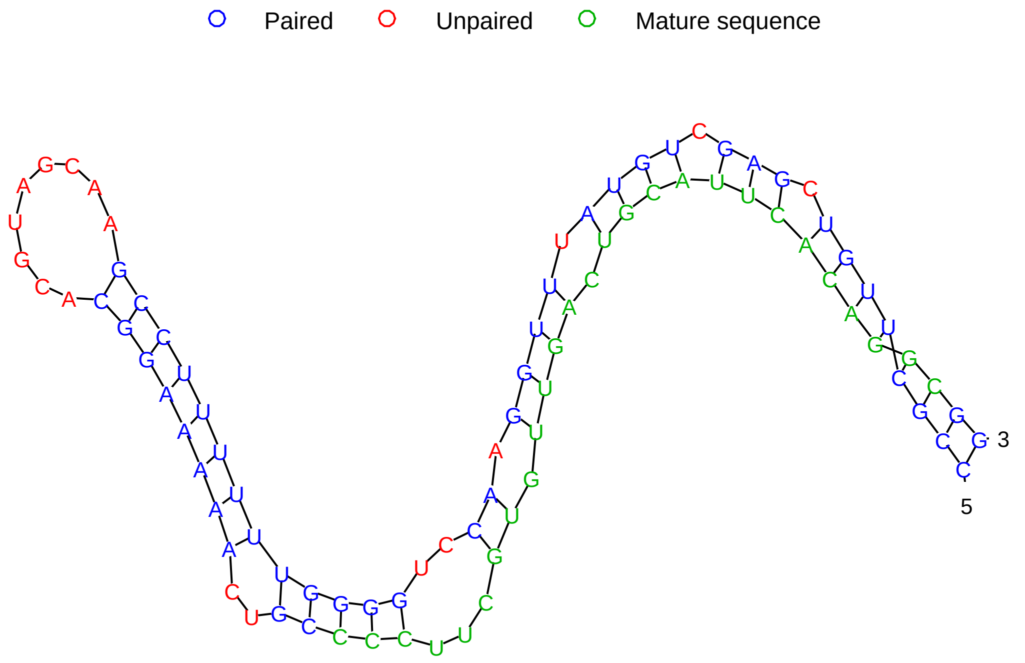

Stem loop (UMD3.1): chr3:82861954-82862043
Mature (UMD3.1): chr3:82862015-82862041
Mature seq len: 27
Total raw counts (9 samples): 1190
Average raw counts: 133
Strand: Forward
Orientation: 3p
Minimum free energy: -33.20

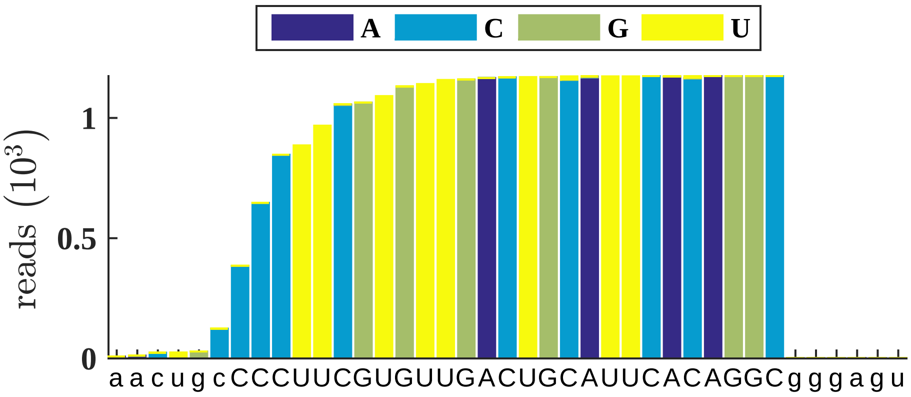

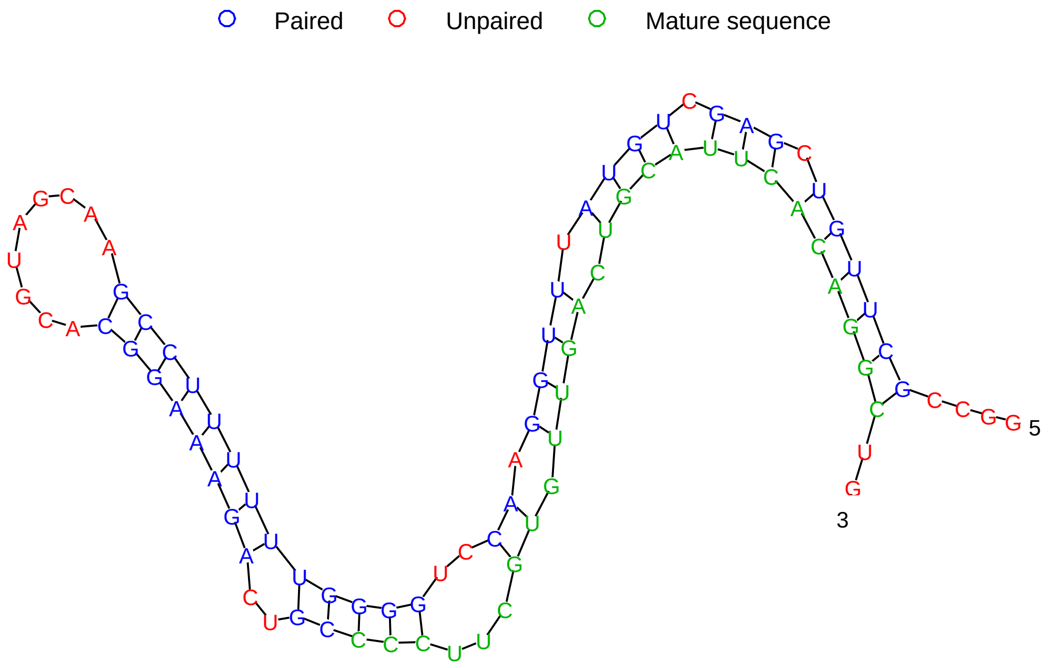

Stem loop (UMD3.1): chr3:82887297-82887388
Mature (UMD3.1): chr3:82887360-82887386
Mature seq len: 27
Total raw counts (9 samples): 1170
Average raw counts: 130
Strand: Forward
Orientation: 3p
Minimum free energy: -29.00

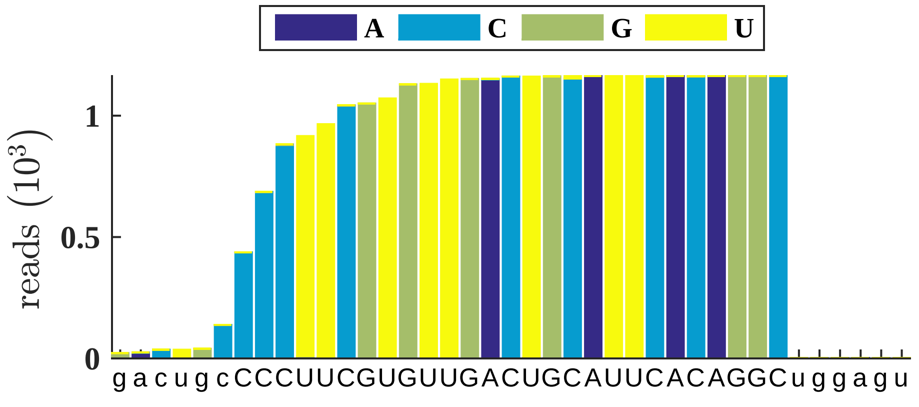

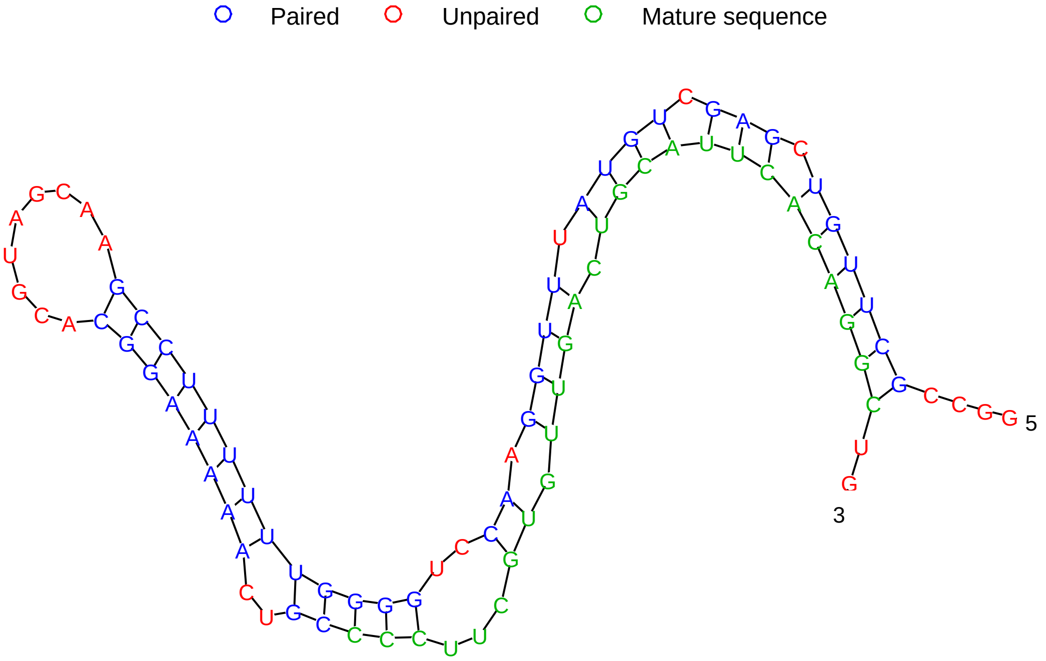

Stem loop (UMD3.1): chr3:82891861-82891952
Mature (UMD3.1): chr3:82891924-82891950
Mature seq len: 27
Total raw counts (9 samples): 1188
Average raw counts: 132
Strand: Forward
Orientation: 3p
Minimum free energy: -28.90

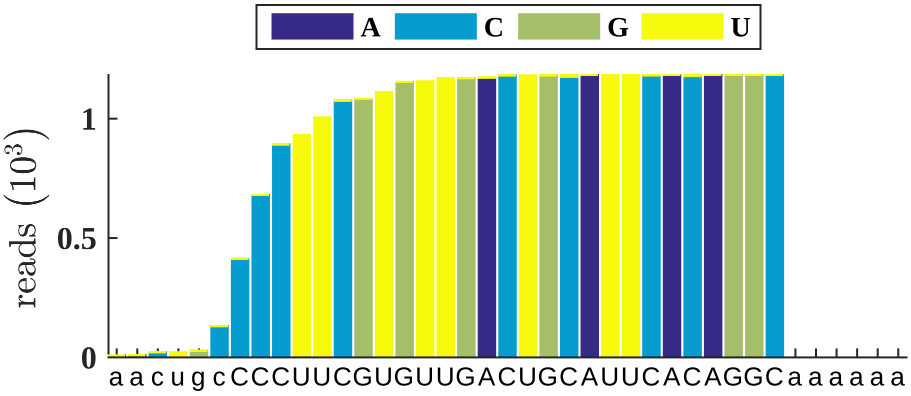

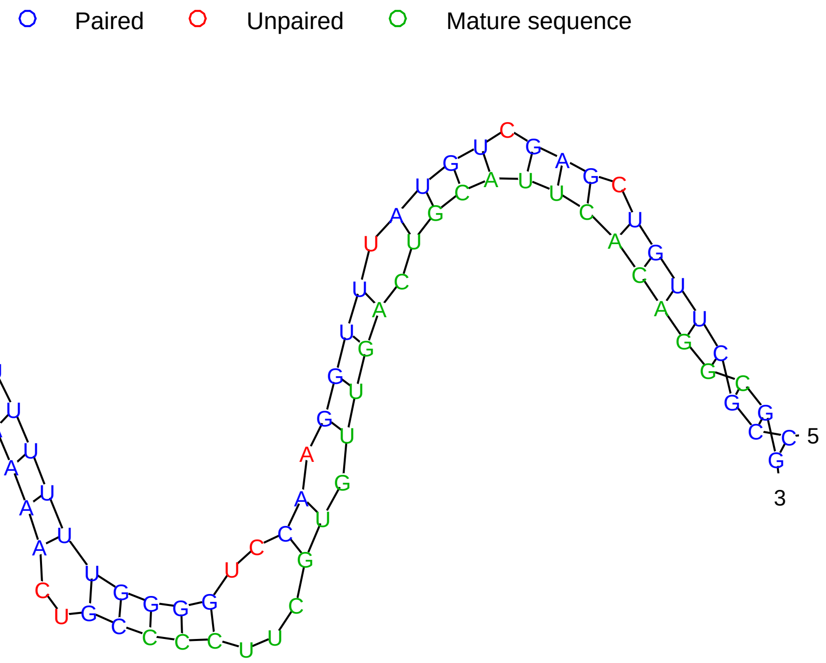

Stem loop (UMD3.1): chr3:86723310-86723399
Mature (UMD3.1): chr3:86723312-86723338
Mature seq len: 27
Total raw counts (9 samples): 1162
Average raw counts: 130
Strand: Reverse
Orientation: 3p
Minimum free energy: -33.20

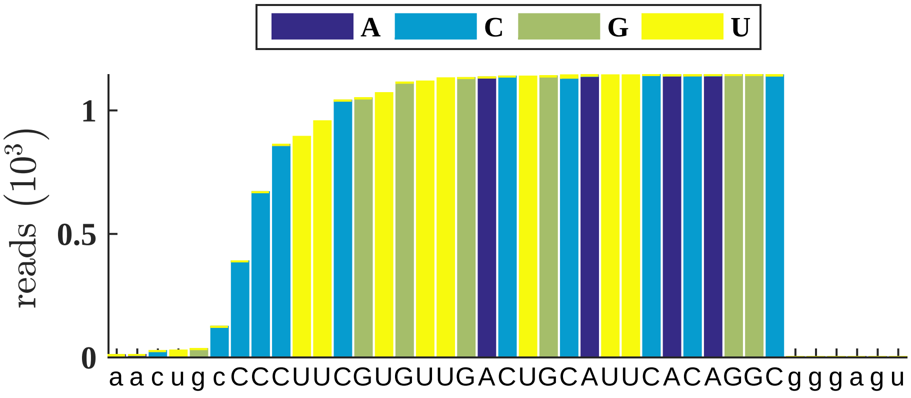

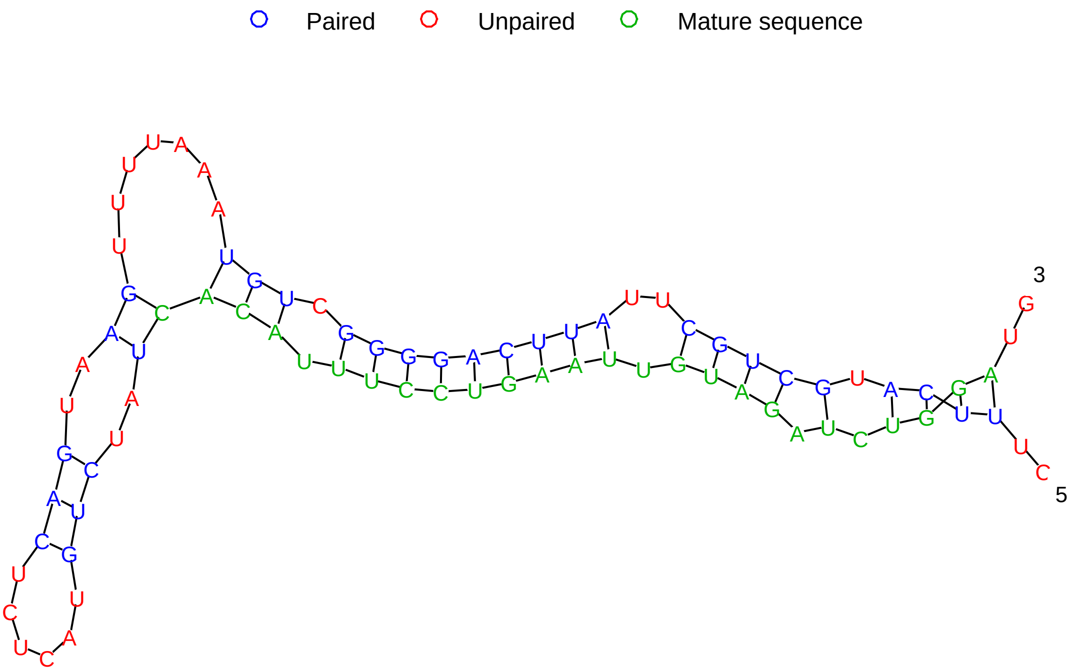

Stem loop (UMD3.1): chr4:110272023-110272103
Mature (UMD3.1): chr4:110272076-110272101
Mature seq len: 26
Total raw counts (9 samples): 638
Average raw counts: 71
Strand: Forward
Orientation: 3p
Minimum free energy: -18.50

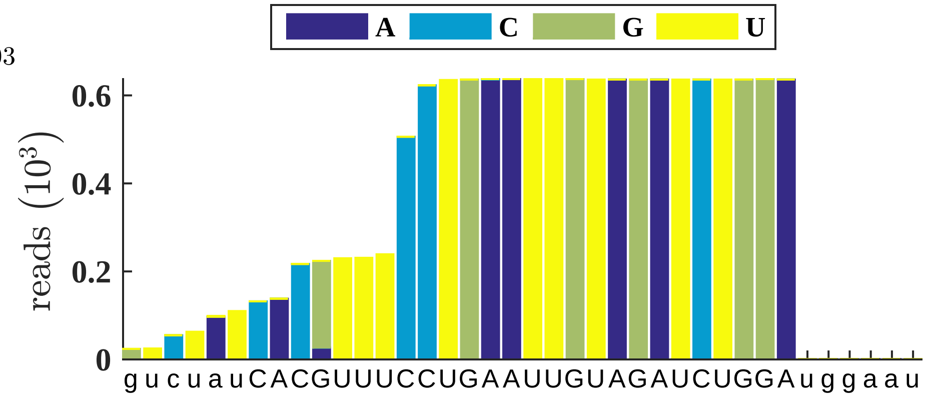

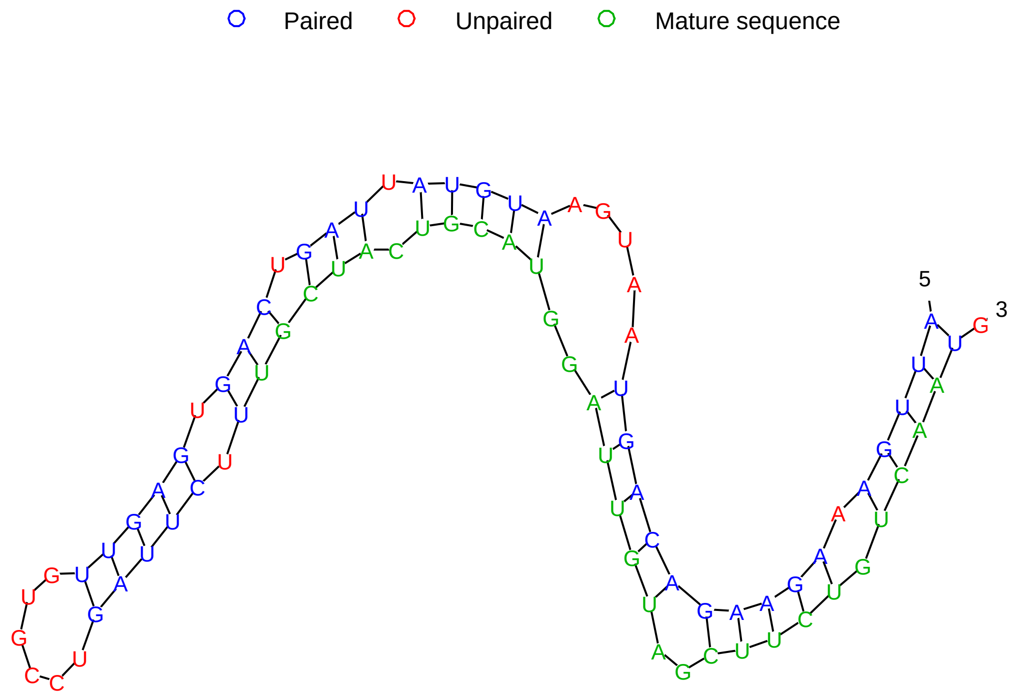

Stem loop (UMD3.1): chr4:110692084-110692168
Mature (UMD3.1): chr4:110692086-110692115
Mature seq len: 30
Total raw counts (9 samples): 646
Average raw counts: 72
Strand: Reverse
Orientation: 3p
Minimum free energy: -21.80

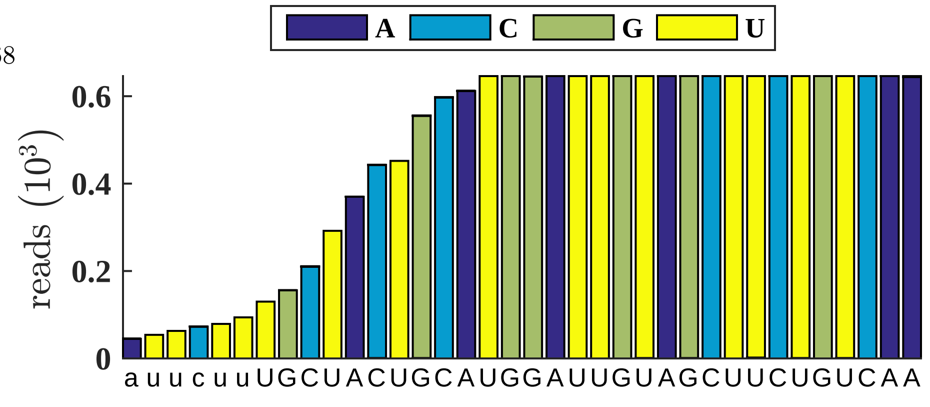

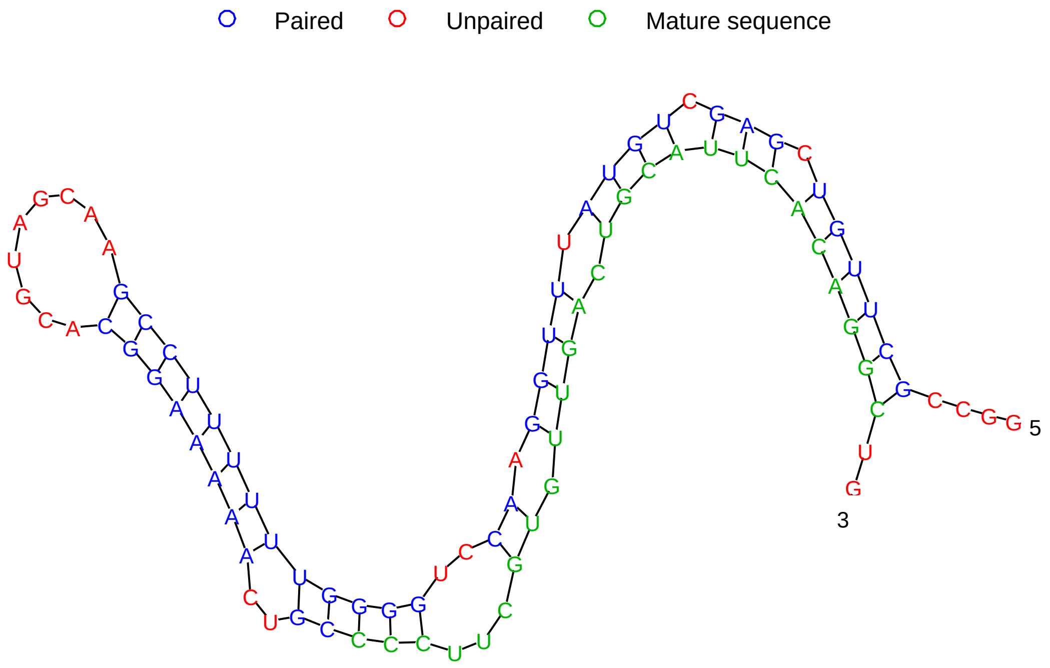

Stem loop (UMD3.1): chr4:11303425-11303516
Mature (UMD3.1): chr4:11303488-11303514
Mature seq len: 27
Total raw counts (9 samples): 1173
Average raw counts: 131
Strand: Forward
Orientation: 3p
Minimum free energy: -28.90

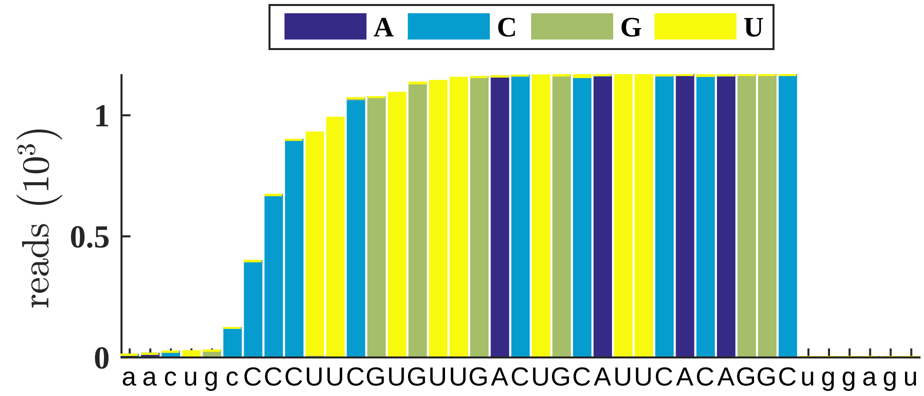

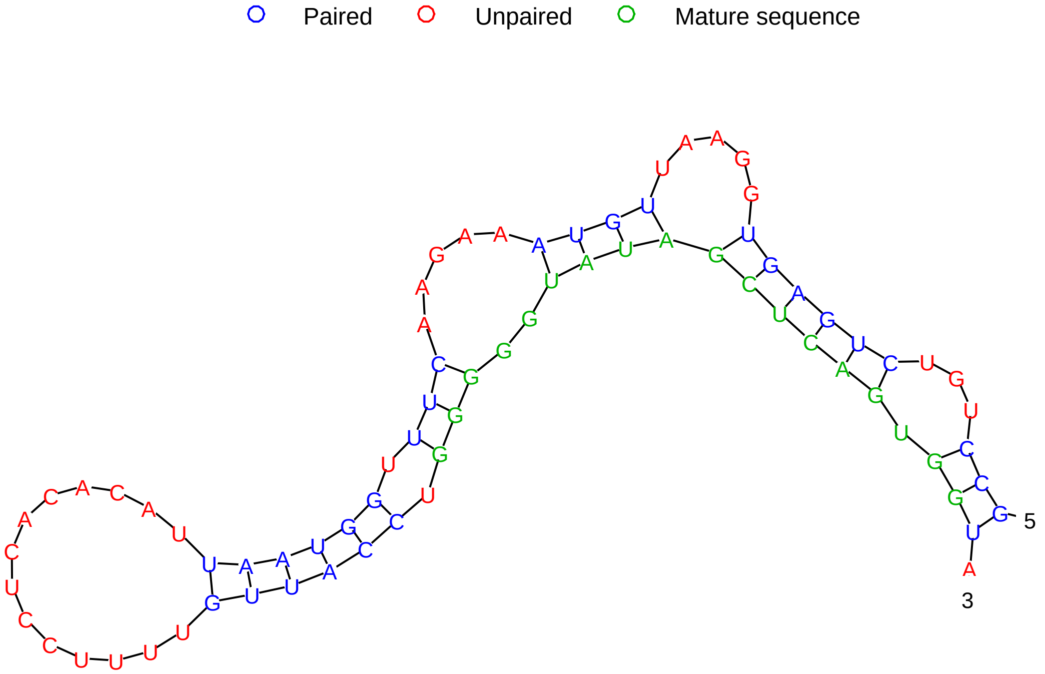

Stem loop (UMD3.1): chr4:113198365-113198441
Mature (UMD3.1): chr4:113198367-113198384
Mature seq len: 18
Total raw counts (9 samples): 695
Average raw counts: 78
Strand: Reverse
Orientation: 3p
Minimum free energy: -15.40

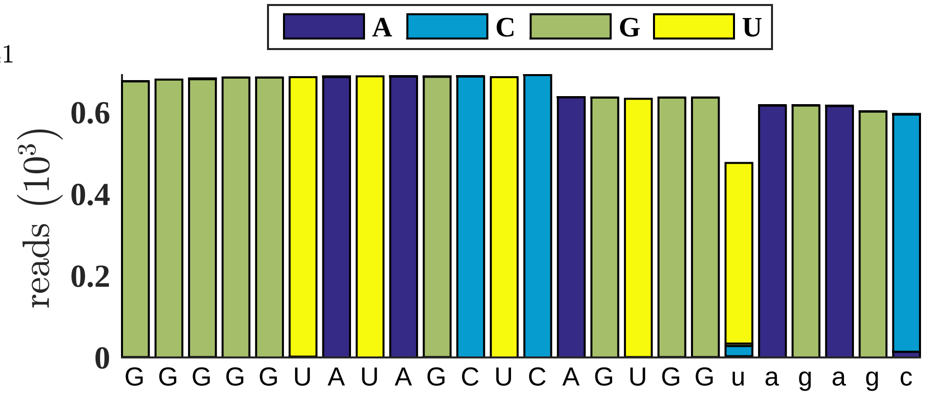

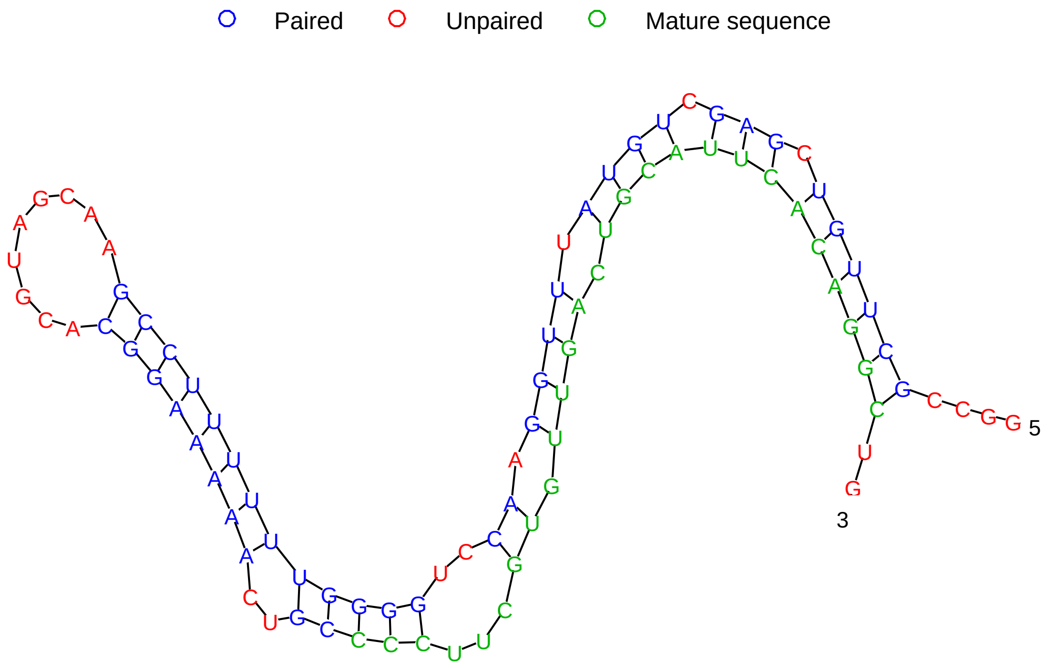

Stem loop (UMD3.1): chr4:119795235-119795326
Mature (UMD3.1): chr4:119795237-119795263
Mature seq len: 27
Total raw counts (9 samples): 1262
Average raw counts: 141
Strand: Reverse
Orientation: 3p
Minimum free energy: -28.90

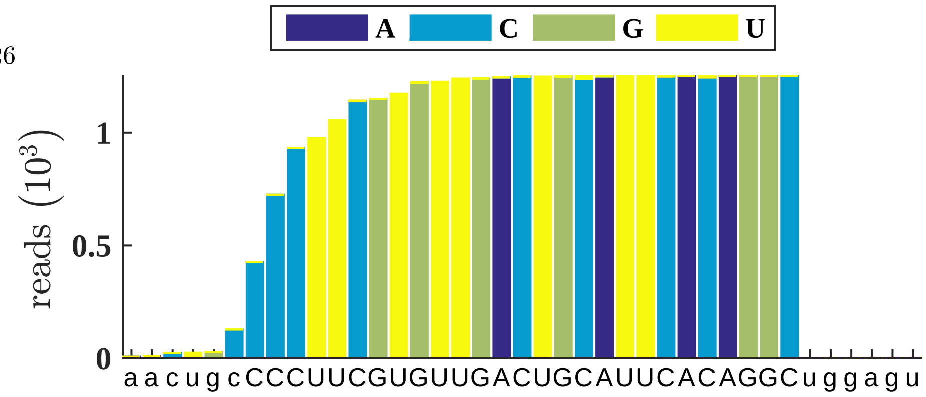

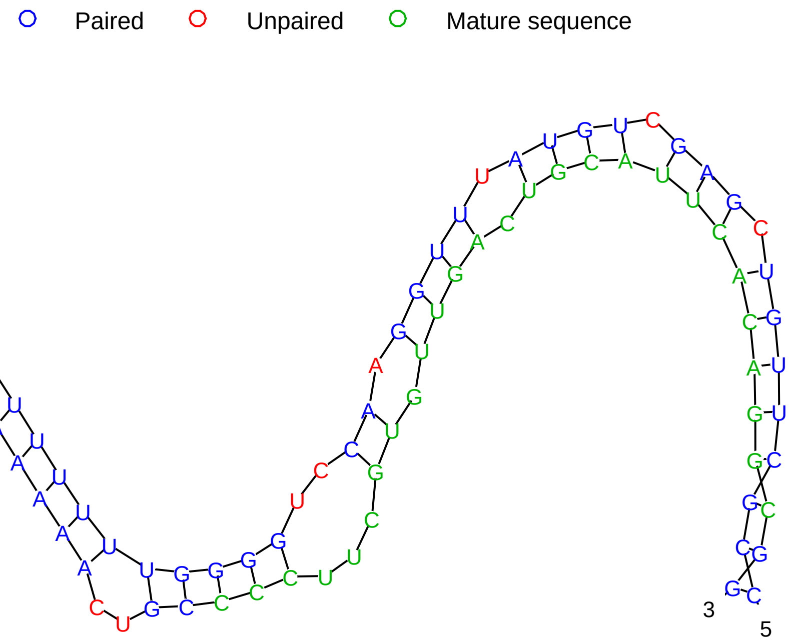

Stem loop (UMD3.1): chr4:27745436-27745525
Mature (UMD3.1): chr4:27745497-27745523
Mature seq len: 27
Total raw counts (9 samples): 1218
Average raw counts: 136
Strand: Forward
Orientation: 3p
Minimum free energy: -33.20

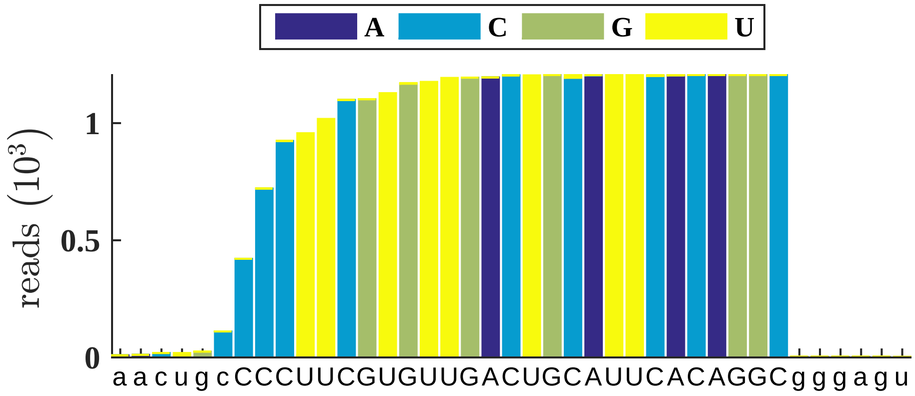

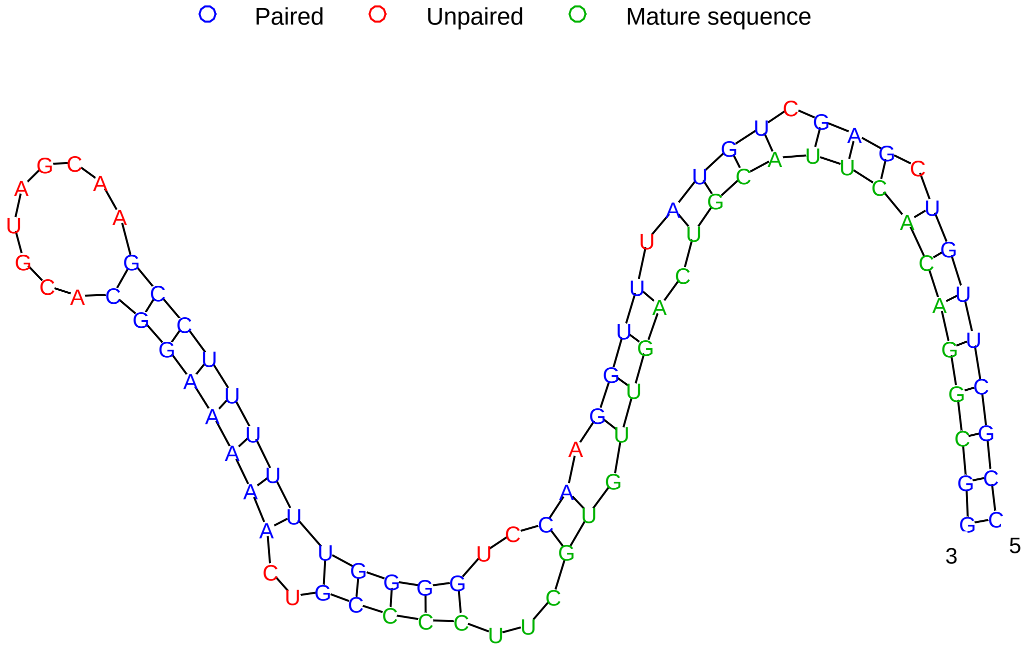

Stem loop (UMD3.1): chr4:27746827-27746916
Mature (UMD3.1): chr4:27746888-27746914
Mature seq len: 27
Total raw counts (9 samples): 1186
Average raw counts: 132
Strand: Forward
Orientation: 3p
Minimum free energy: -33.20

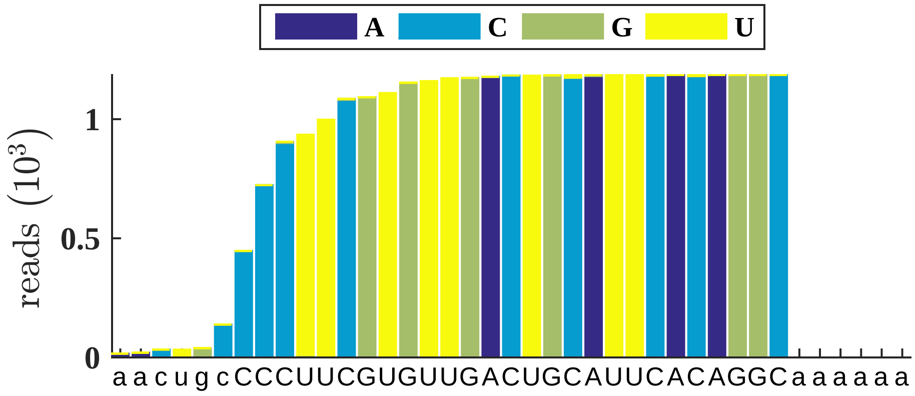

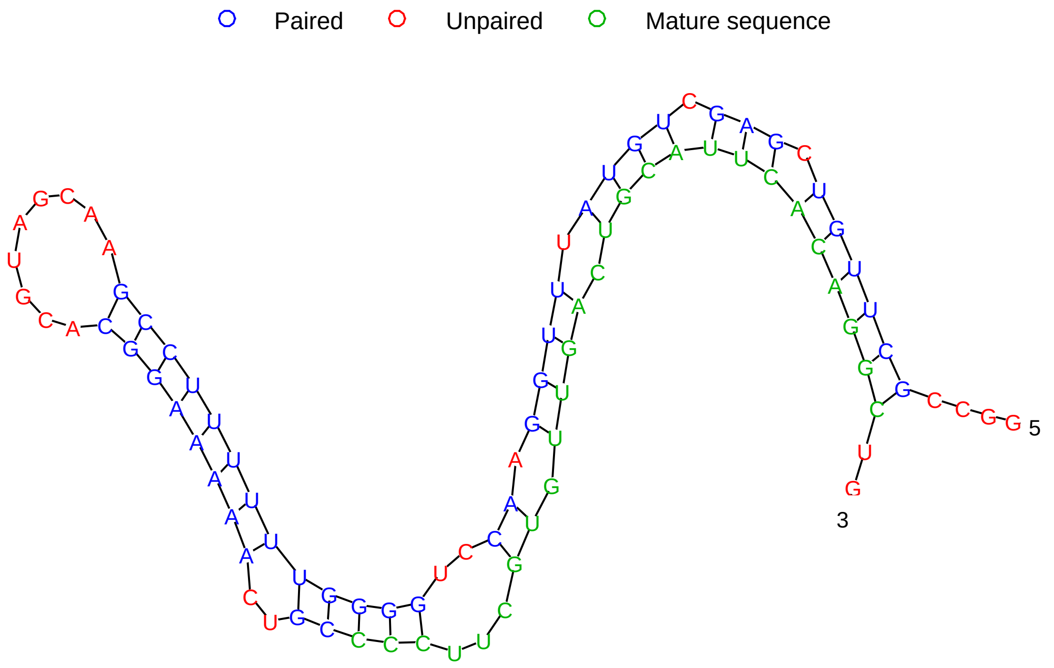

Stem loop (UMD3.1): chr4:28956582-28956673
Mature (UMD3.1): chr4:28956584-28956610
Mature seq len: 27
Total raw counts (9 samples): 1203
Average raw counts: 134
Strand: Reverse
Orientation: 3p
Minimum free energy: -28.90

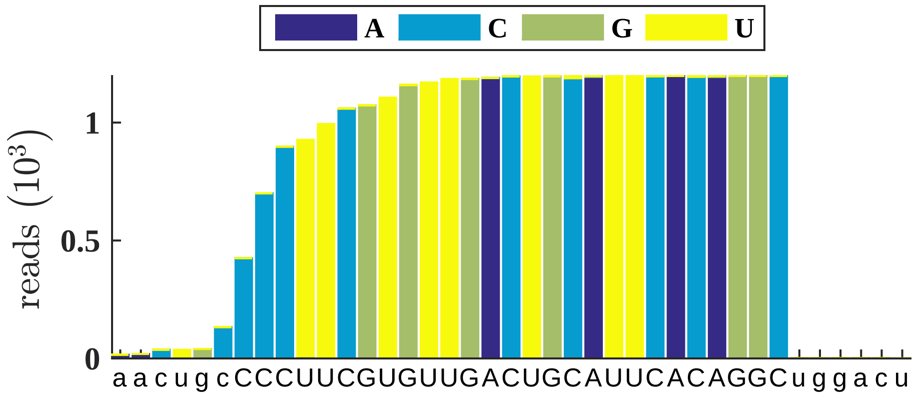

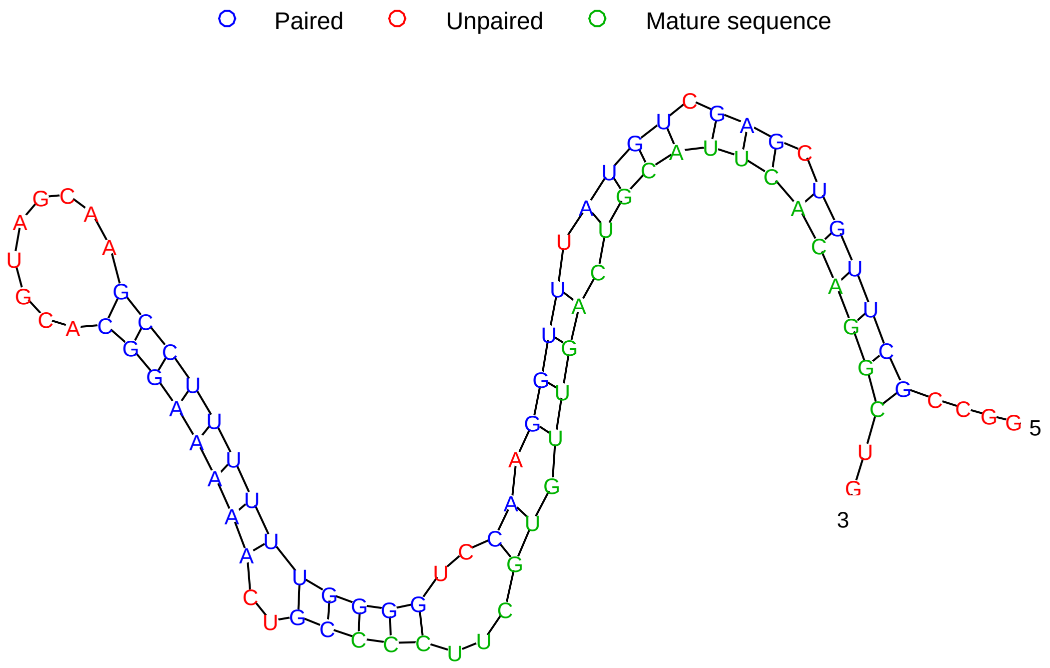

Stem loop (UMD3.1): chr4:28958281-28958372
Mature (UMD3.1): chr4:28958344-28958370
Mature seq len: 27
Total raw counts (9 samples): 1189
Average raw counts: 133
Strand: Forward
Orientation: 3p
Minimum free energy: -28.90

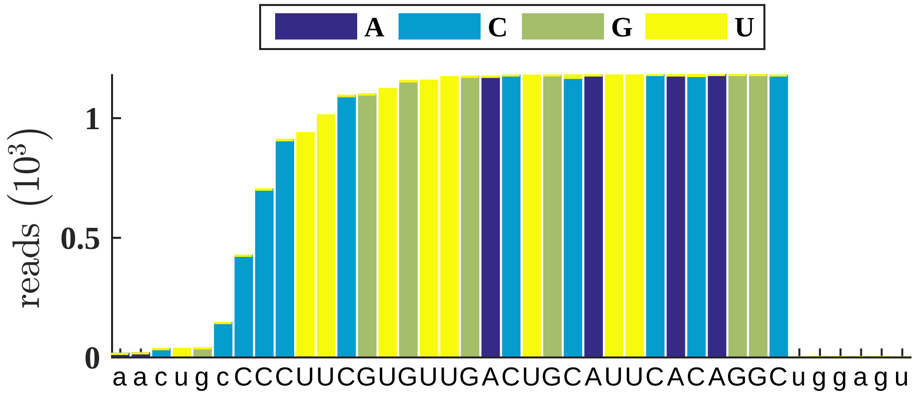

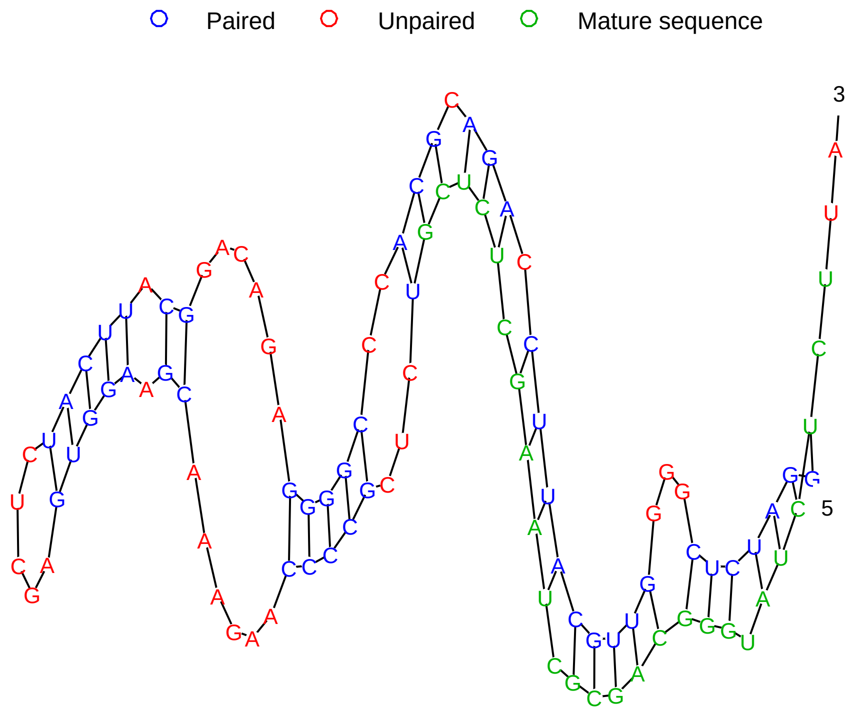

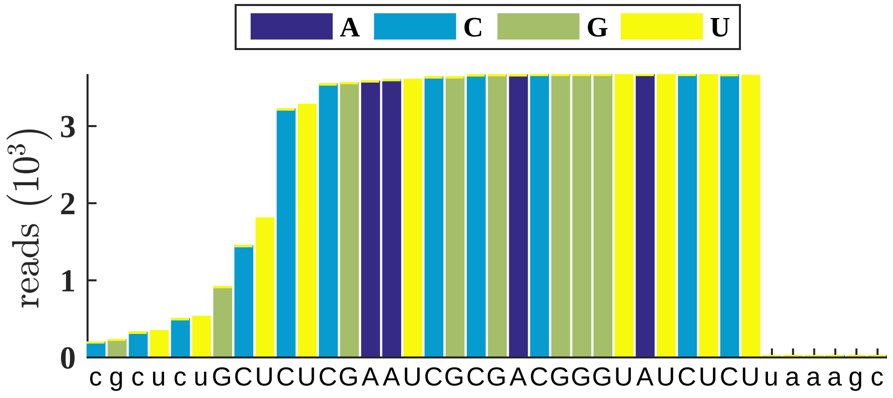

Stem loop (UMD3.1): chr4:28960362-28960465
Mature (UMD3.1): chr4:28960438-28960463
Mature seq len: 26
Total raw counts (9 samples): 3663
Average raw counts: 407
Strand: Forward
Orientation: 3p
Minimum free energy: -32.40

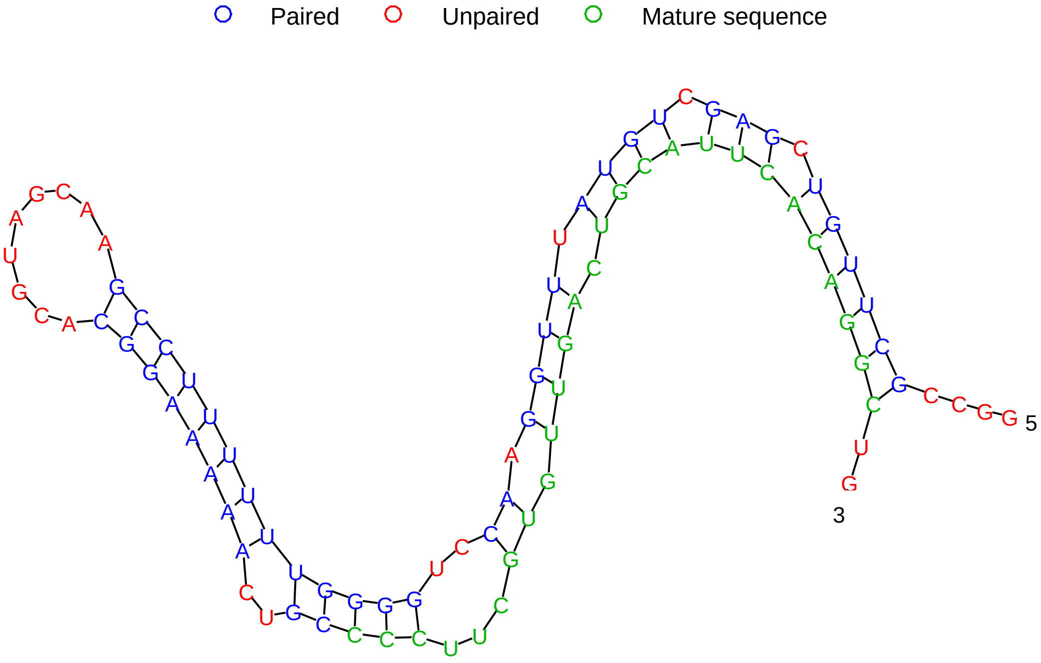

Stem loop (UMD3.1): chr4:28968940-28969031
Mature (UMD3.1): chr4:28968942-28968968
Mature seq len: 27
Total raw counts (9 samples): 1220
Average raw counts: 136
Strand: Reverse
Orientation: 3p
Minimum free energy: -28.90

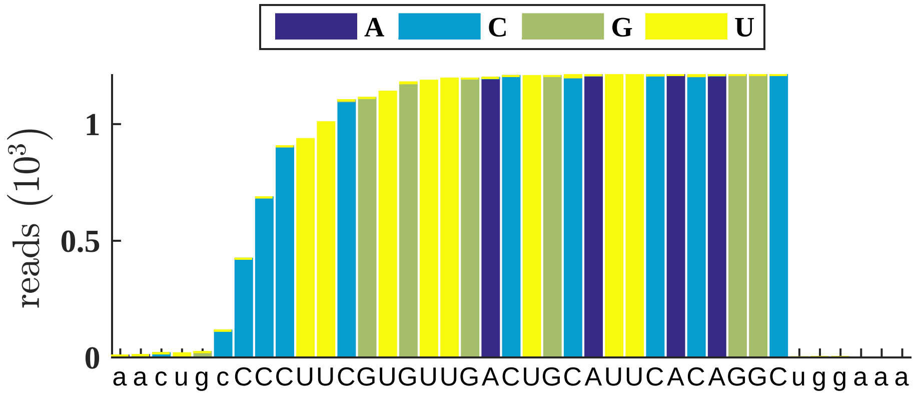

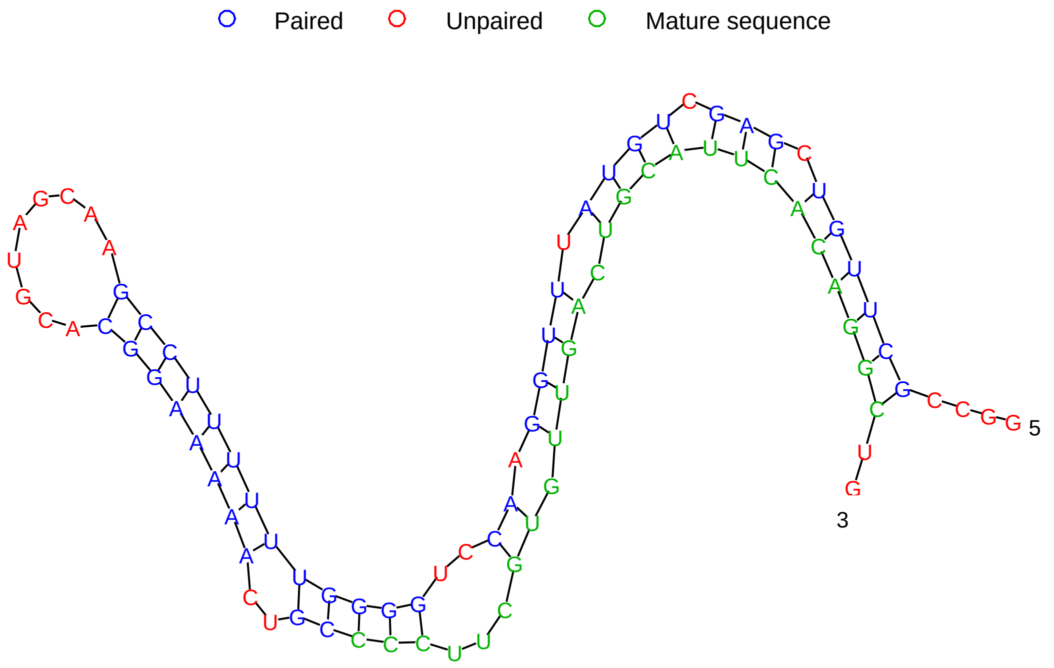

Stem loop (UMD3.1): chr4:28970311-28970402
Mature (UMD3.1): chr4:28970313-28970339
Mature seq len: 27
Total raw counts (9 samples): 1213
Average raw counts: 135
Strand: Reverse
Orientation: 3p
Minimum free energy: -28.90

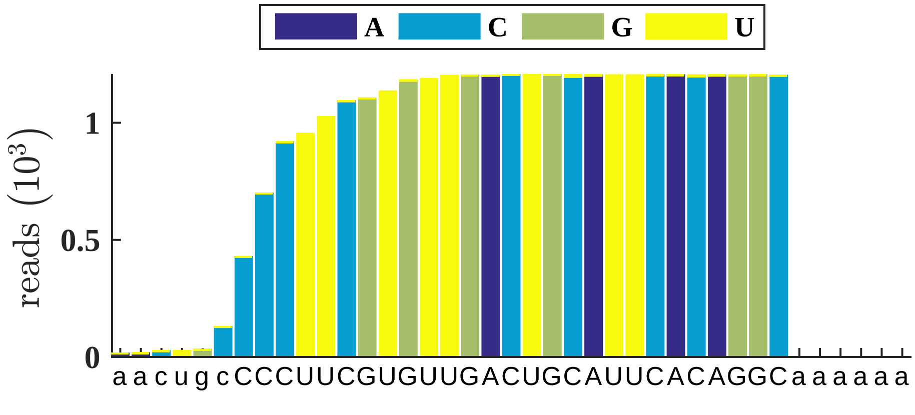

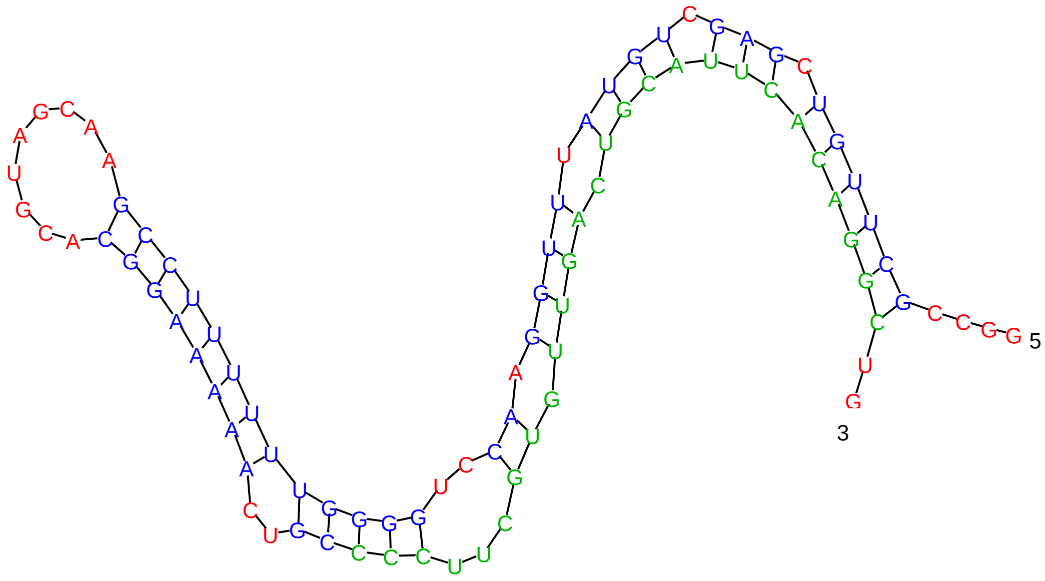

Stem loop (UMD3.1): chr4:28979835-28979926
Mature (UMD3.1): chr4:28979837-28979863
Mature seq len: 27
Total raw counts (9 samples): 1243
Average raw counts: 139
Strand: Reverse
Orientation: 3p
Minimum free energy: -28.90

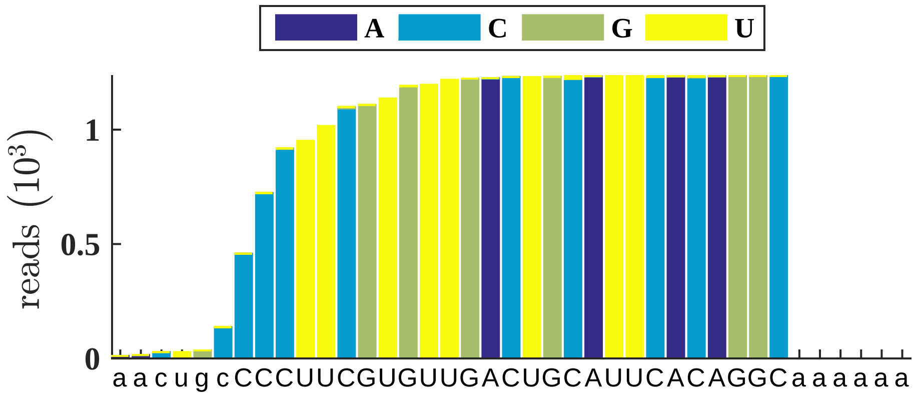

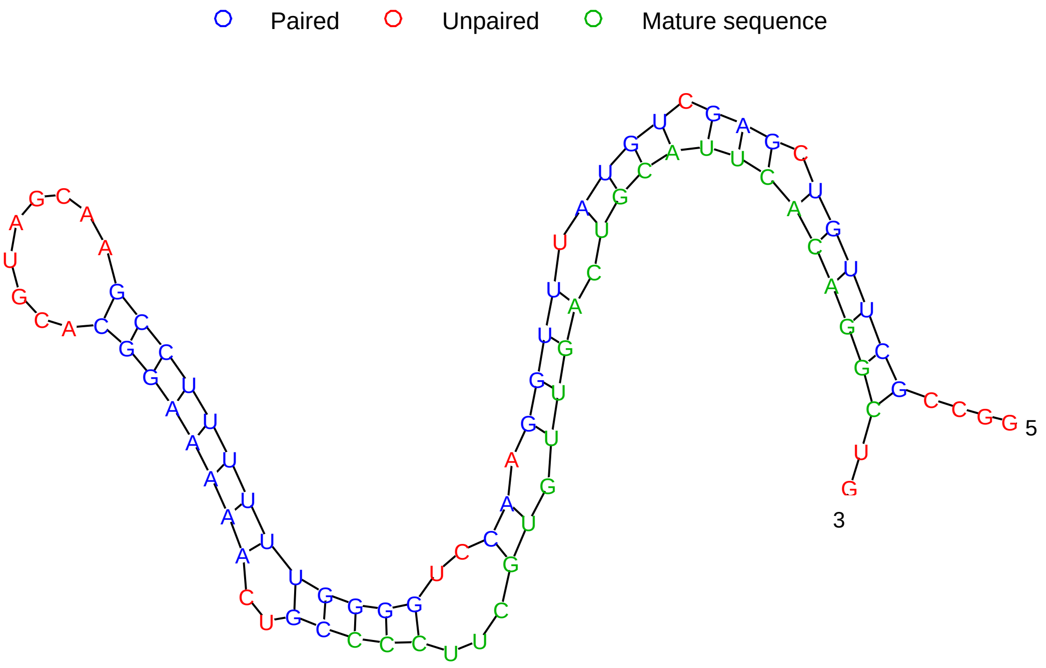

Stem loop (UMD3.1): chr4:28981212-28981303
Mature (UMD3.1): chr4:28981214-28981240
Mature seq len: 27
Total raw counts (9 samples): 1291
Average raw counts: 144
Strand: Reverse
Orientation: 3p
Minimum free energy: -28.90

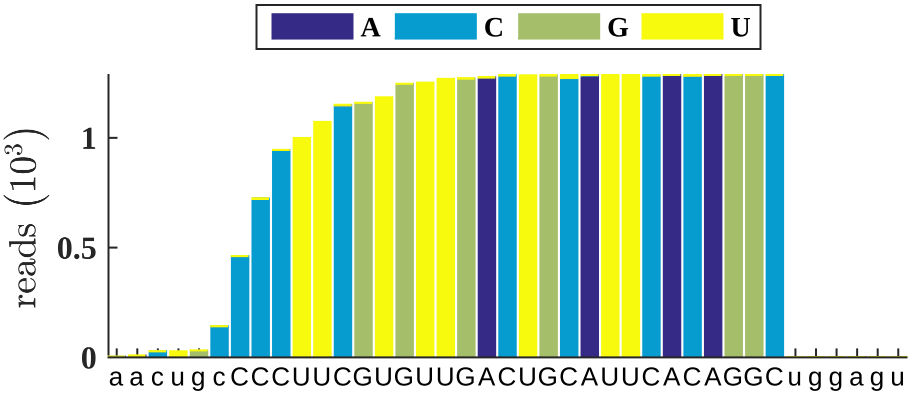

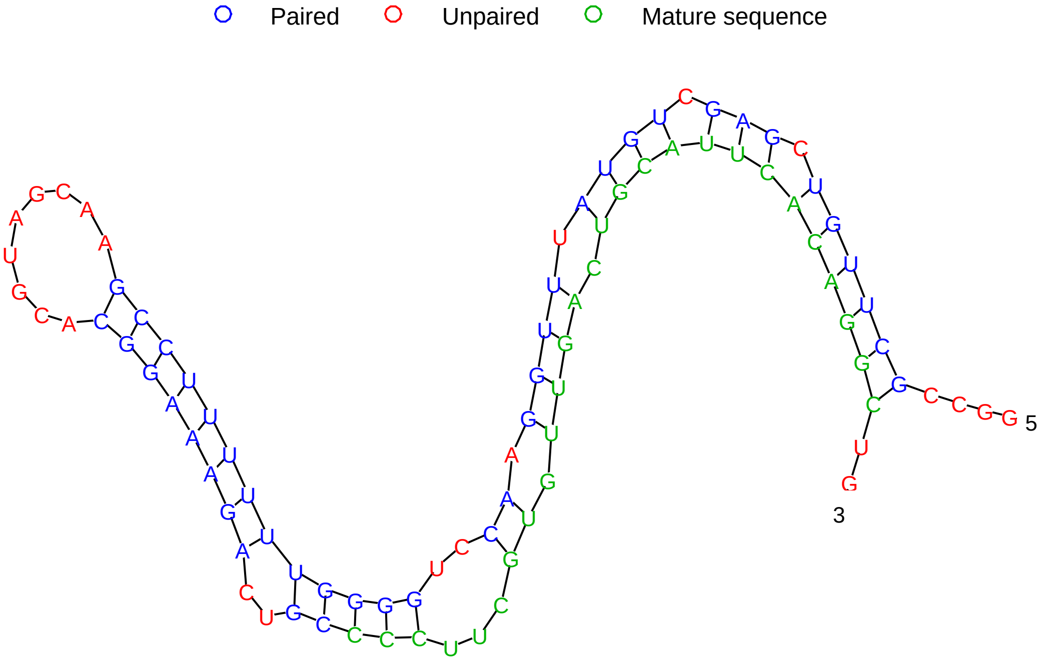

Stem loop (UMD3.1): chr4:28983257-28983348
Mature (UMD3.1): chr4:28983259-28983285
Mature seq len: 27
Total raw counts (9 samples): 1216
Average raw counts: 136
Strand: Reverse
Orientation: 3p
Minimum free energy: -29.00

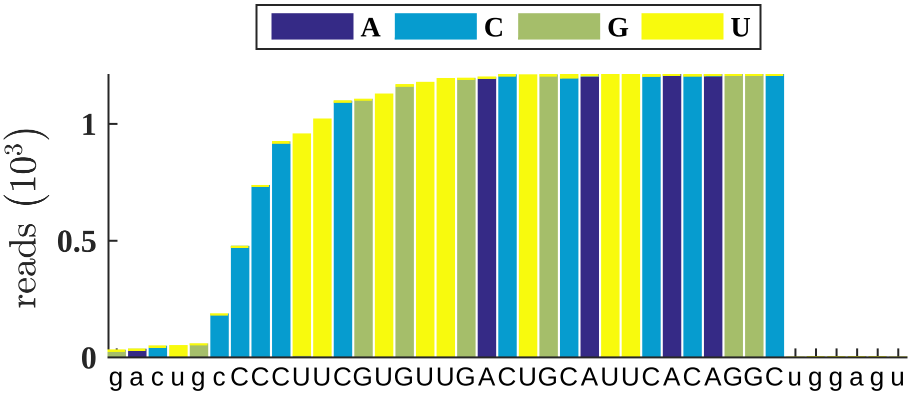

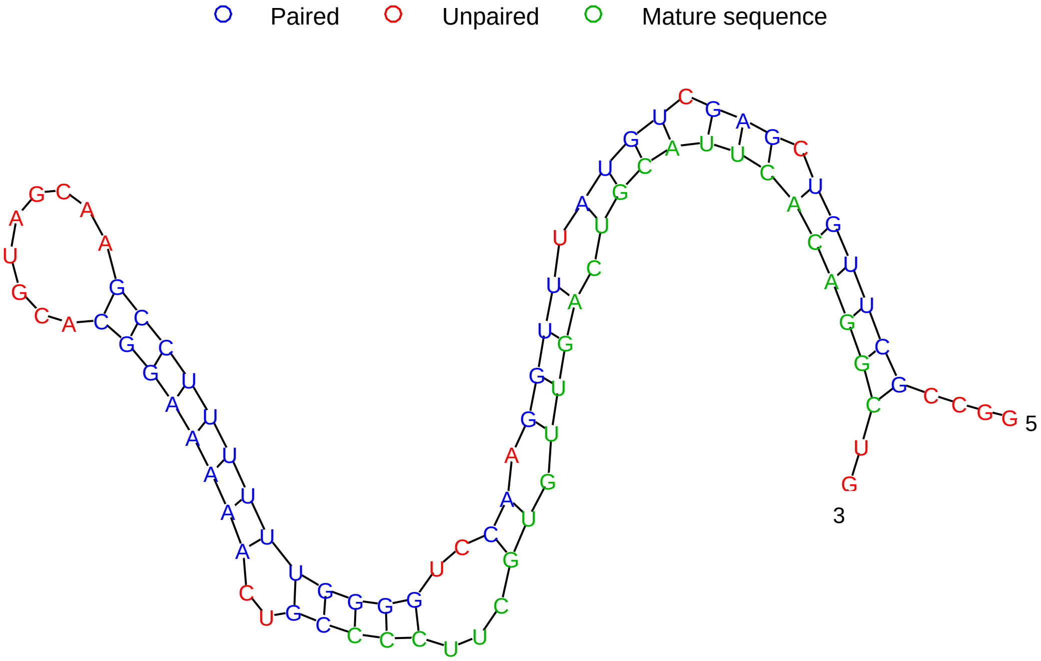

Stem loop (UMD3.1): chr4:28985550-28985641
Mature (UMD3.1): chr4:28985613-28985639
Mature seq len: 27
Total raw counts (9 samples): 1161
Average raw counts: 129
Strand: Forward
Orientation: 3p
Minimum free energy: -28.90

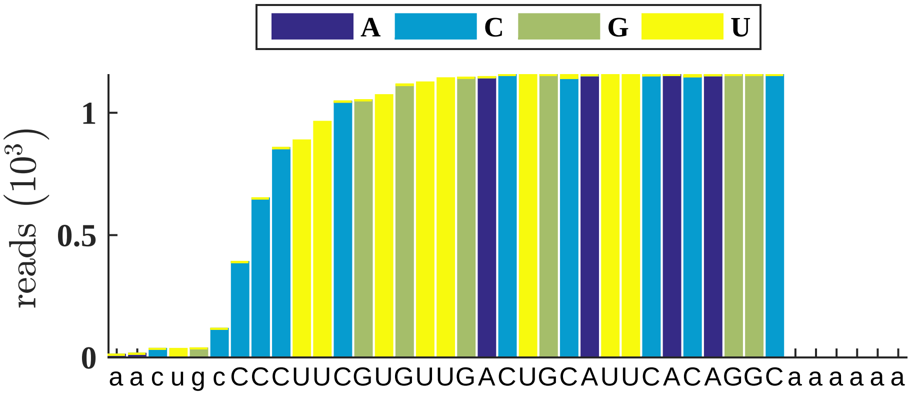

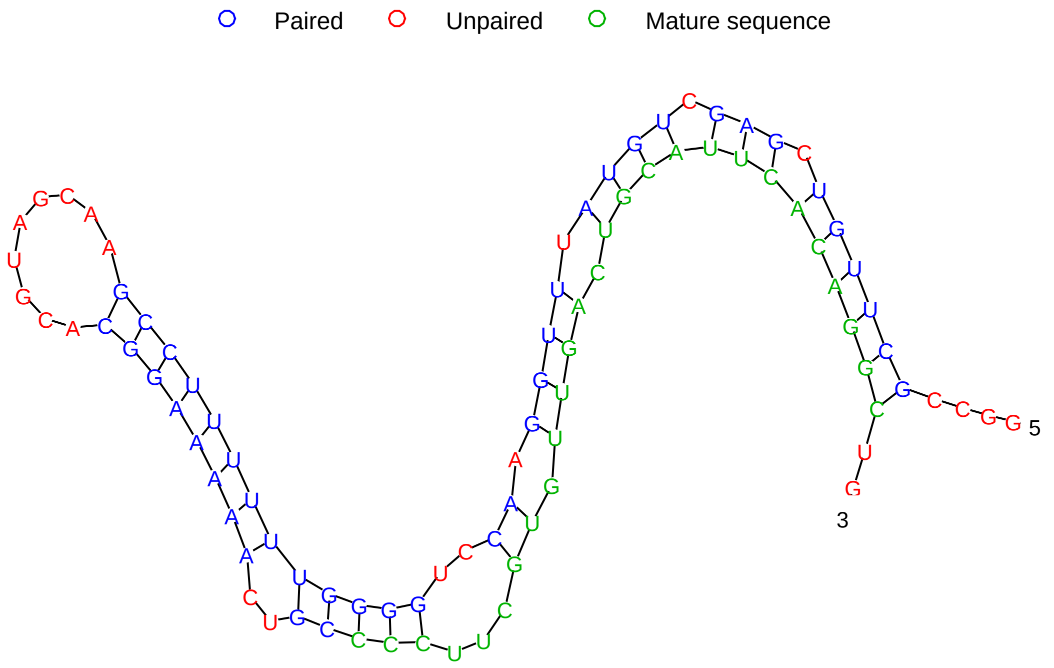

Stem loop (UMD3.1): chr4:28995077-28995168
Mature (UMD3.1): chr4:28995140-28995166
Mature seq len: 27
Total raw counts (9 samples): 1232
Average raw counts: 137
Strand: Forward
Orientation: 3p
Minimum free energy: -28.90

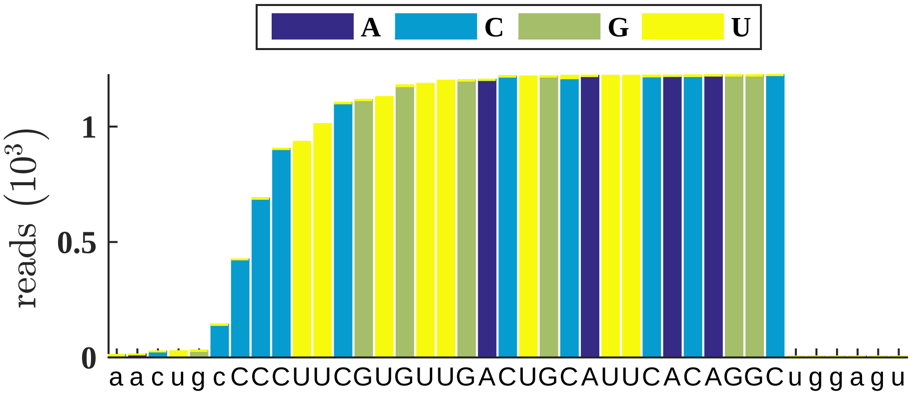

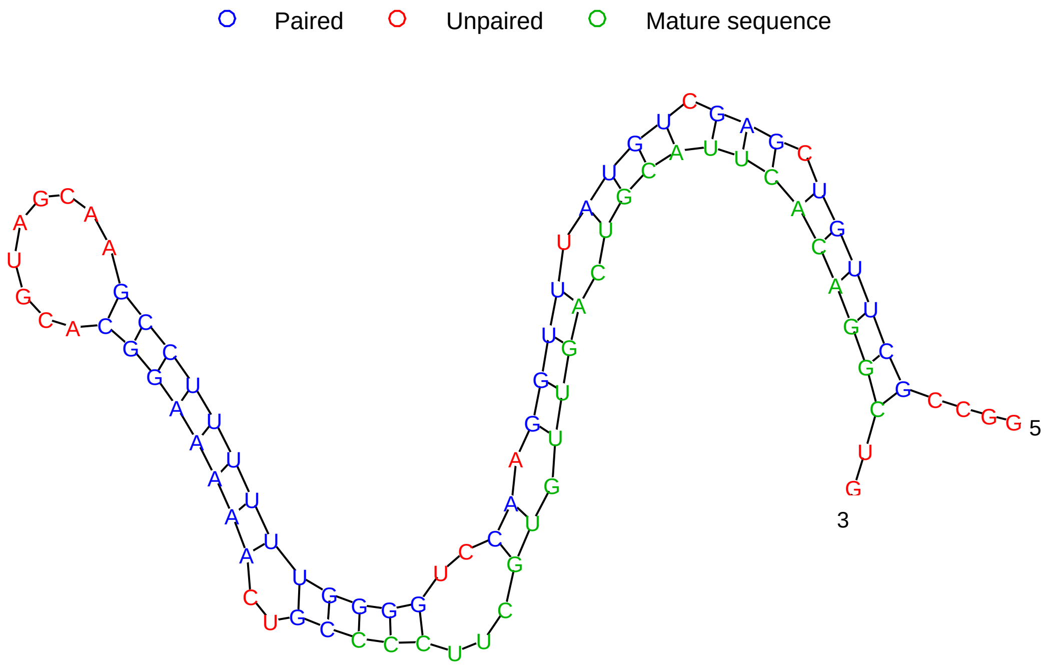

Stem loop (UMD3.1): chr4:29000112-29000203
Mature (UMD3.1): chr4:29000114-29000140
Mature seq len: 27
Total raw counts (9 samples): 1205
Average raw counts: 134
Strand: Reverse
Orientation: 3p
Minimum free energy: -28.90

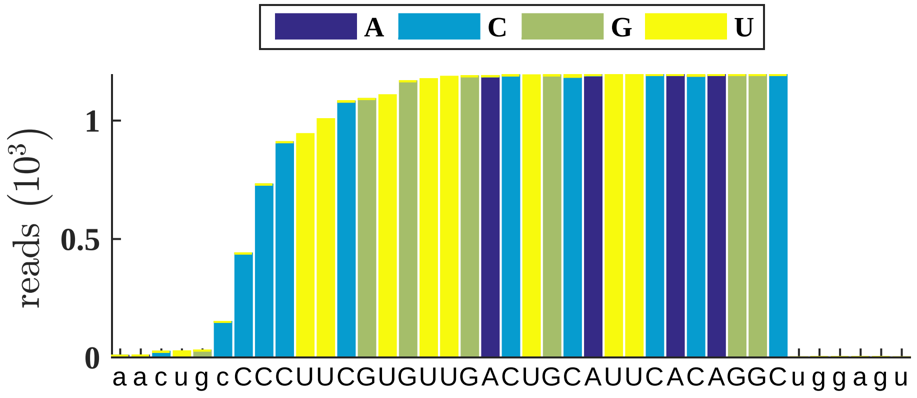

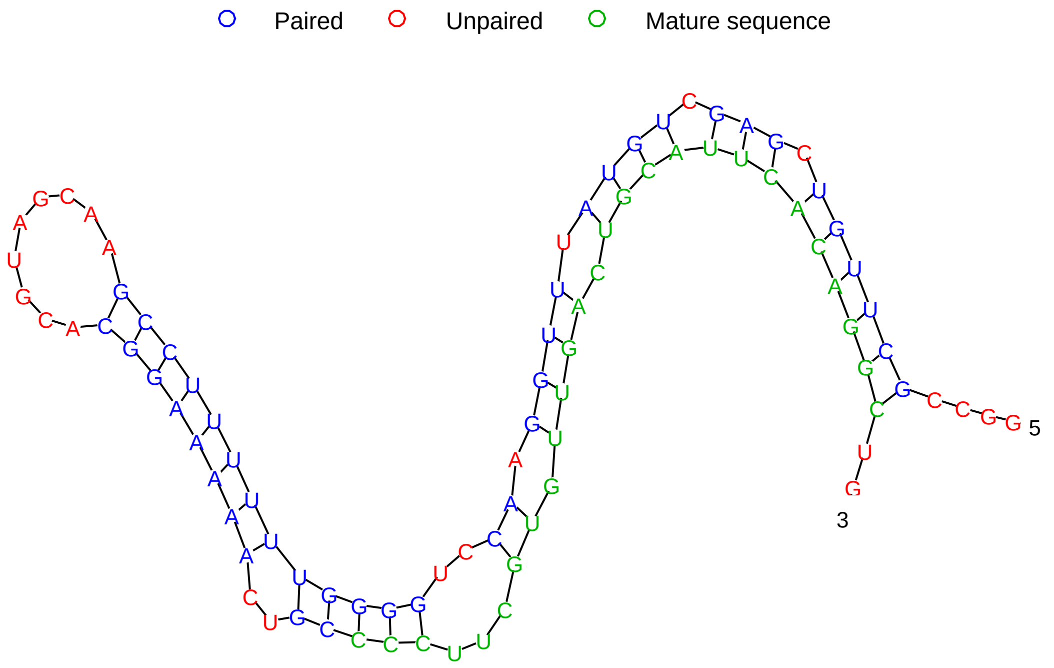

Stem loop (UMD3.1): chr4:29002215-29002306
Mature (UMD3.1): chr4:29002217-29002243
Mature seq len: 27
Total raw counts (9 samples): 1180
Average raw counts: 132
Strand: Reverse
Orientation: 3p
Minimum free energy: -28.90

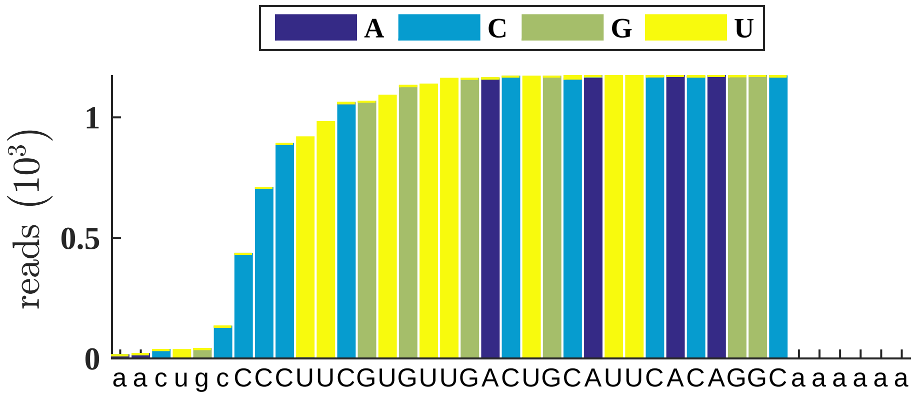

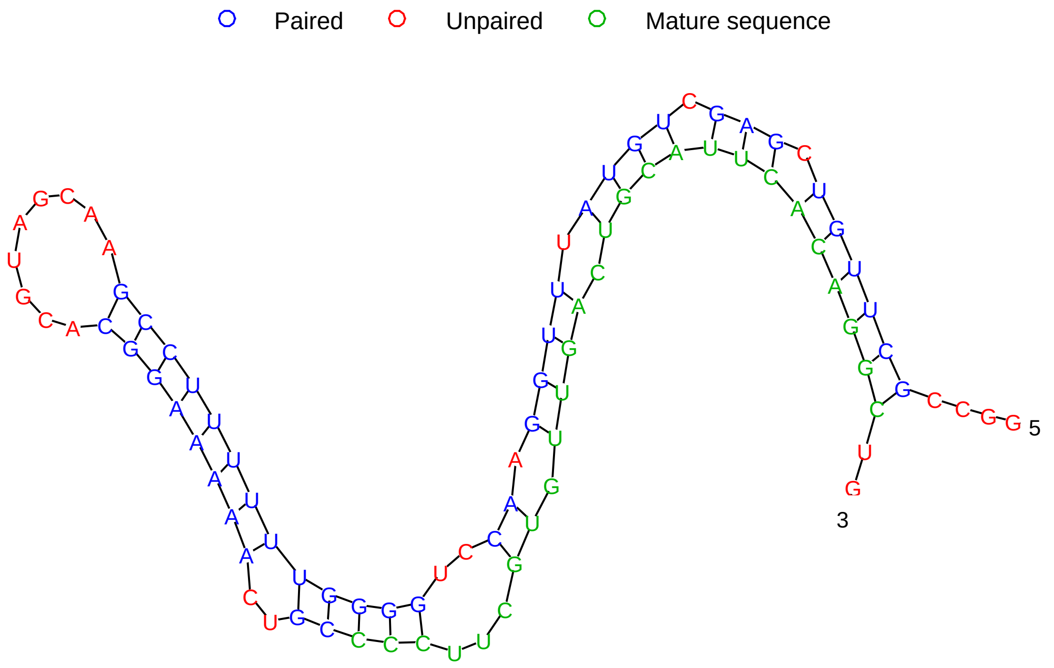

Stem loop (UMD3.1): chr4:29003566-29003657
Mature (UMD3.1): chr4:29003629-29003655
Mature seq len: 27
Total raw counts (9 samples): 1163
Average raw counts: 130
Strand: Forward
Orientation: 3p
Minimum free energy: -28.90

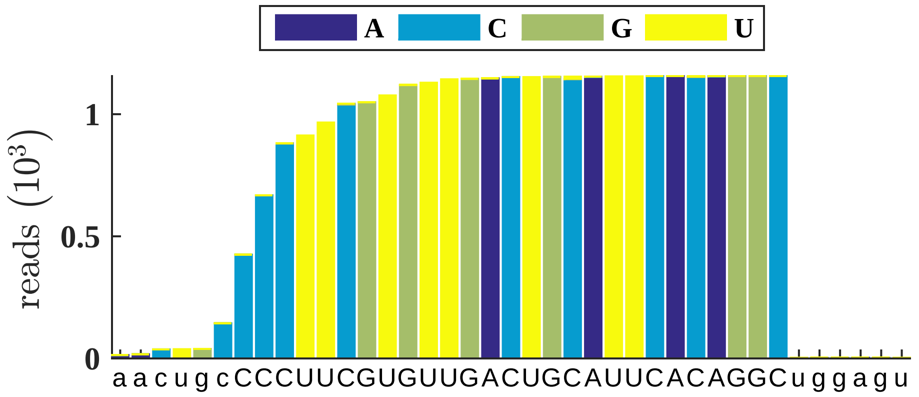

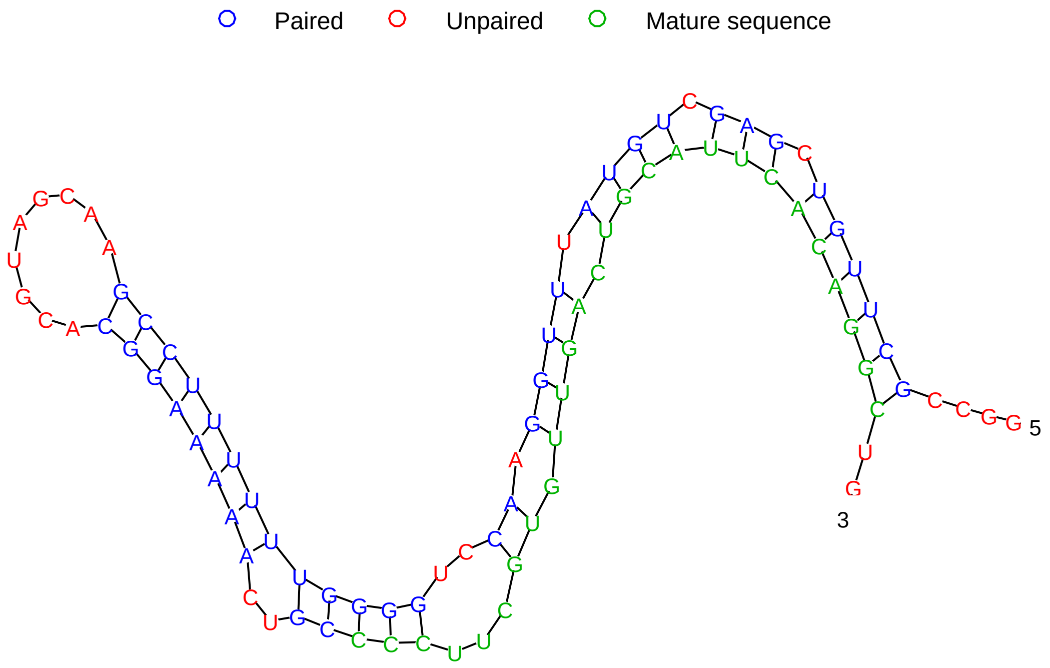

Stem loop (UMD3.1): chr4:29004940-29005031
Mature (UMD3.1): chr4:29005003-29005029
Mature seq len: 27
Total raw counts (9 samples): 1178
Average raw counts: 131
Strand: Forward
Orientation: 3p
Minimum free energy: -28.90

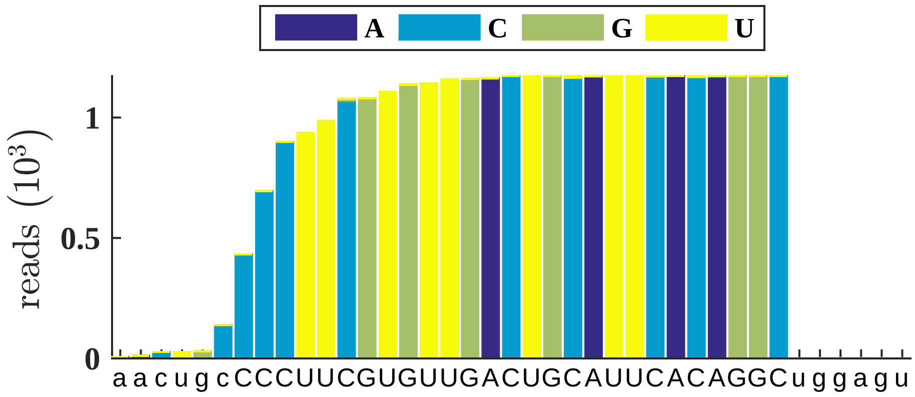

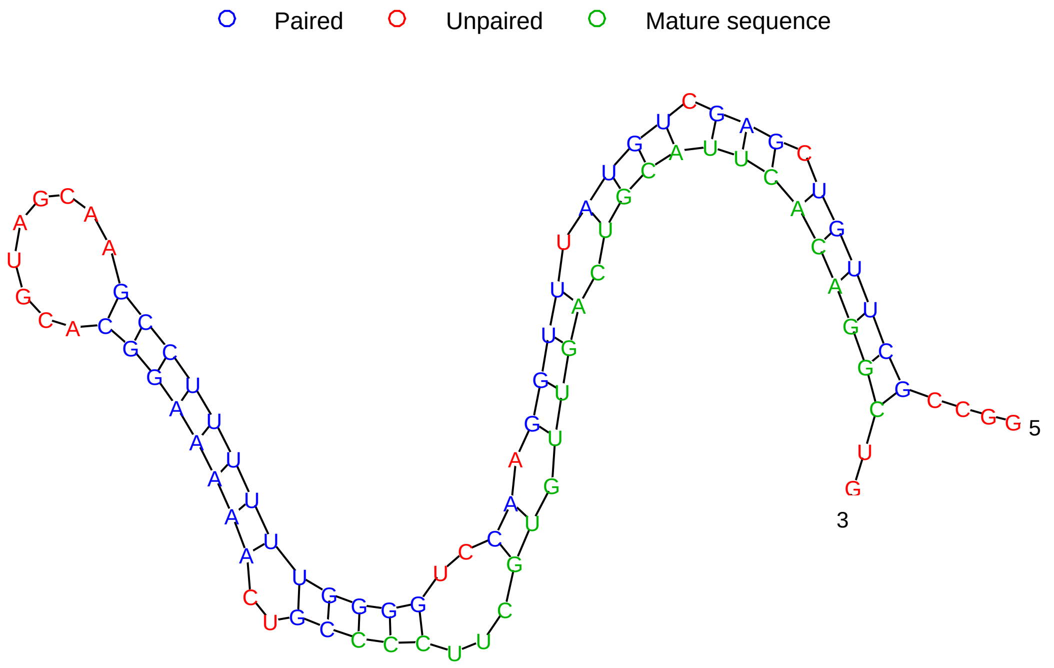

Stem loop (UMD3.1): chr4:29006212-29006303
Mature (UMD3.1): chr4:29006275-29006301
Mature seq len: 27
Total raw counts (9 samples): 1240
Average raw counts: 138
Strand: Forward
Orientation: 3p
Minimum free energy: -28.90

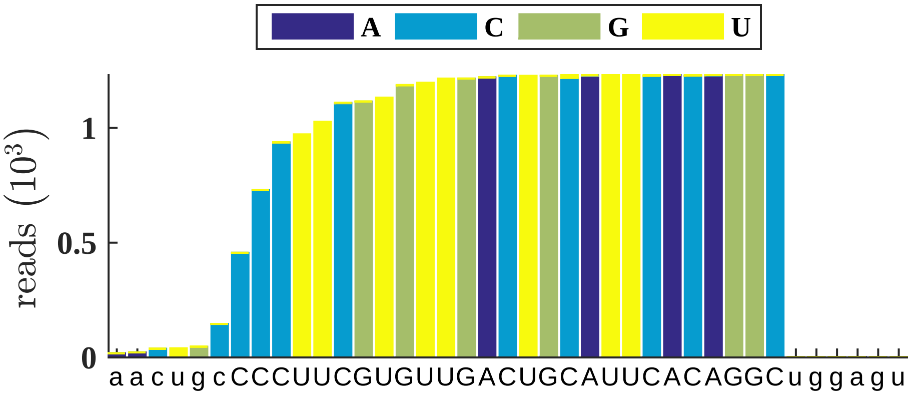

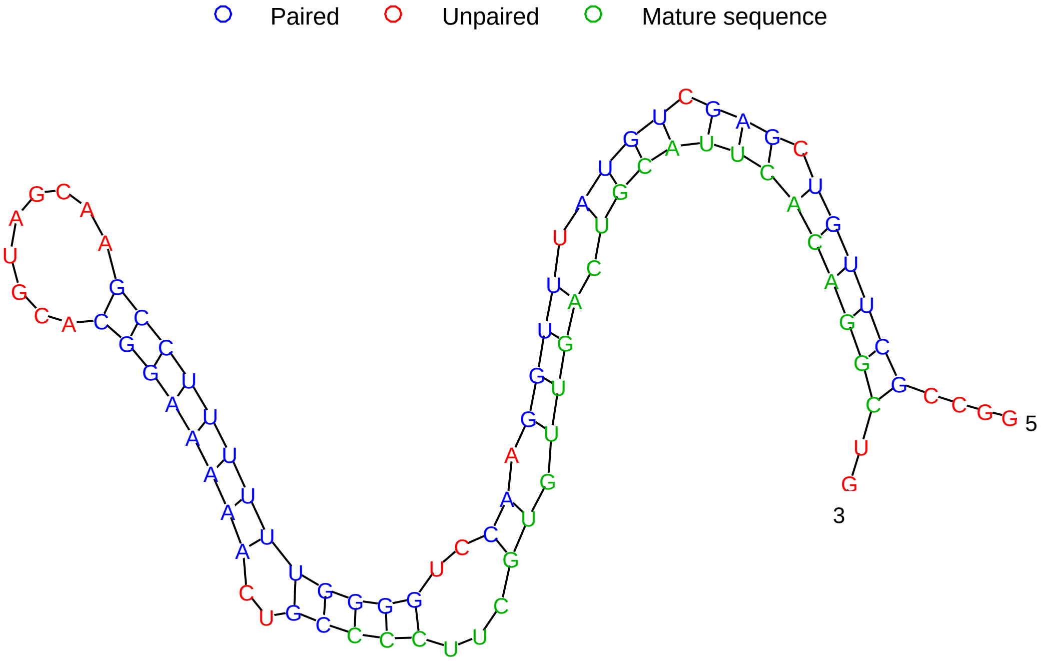

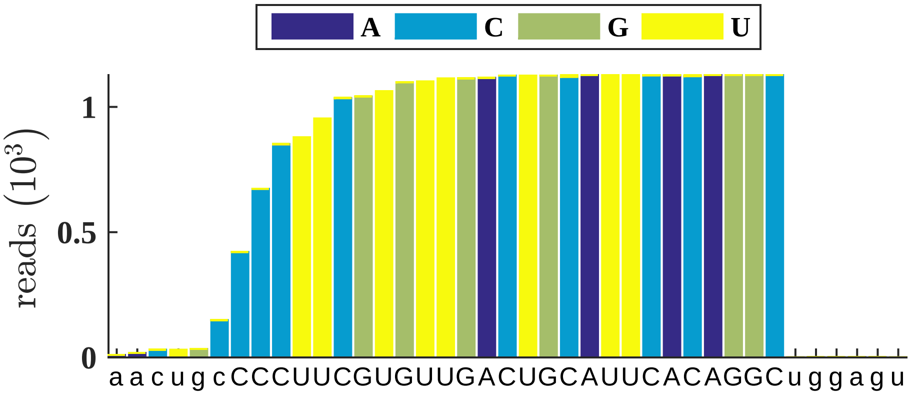

Stem loop (UMD3.1): chr4:29007599-29007690
Mature (UMD3.1): chr4:29007662-29007688
Mature seq len: 27
Total raw counts (9 samples): 1127
Average raw counts: 126
Strand: Forward
Orientation: 3p
Minimum free energy: -28.90

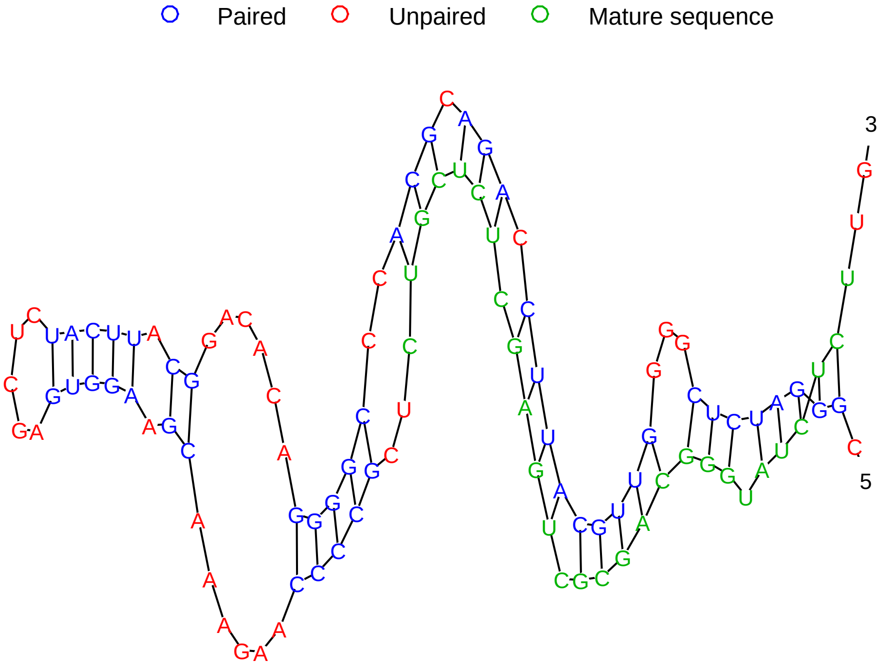

Stem loop (UMD3.1): chr4:29009814-29009919
Mature (UMD3.1): chr4:29009816-29009843
Mature seq len: 28
Total raw counts (9 samples): 13656
Average raw counts: 1518
Strand: Reverse
Orientation: 3p
Minimum free energy: -35.70

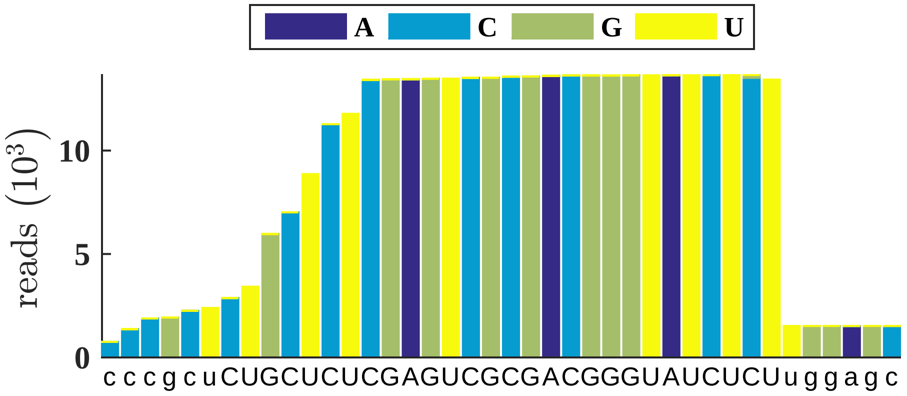

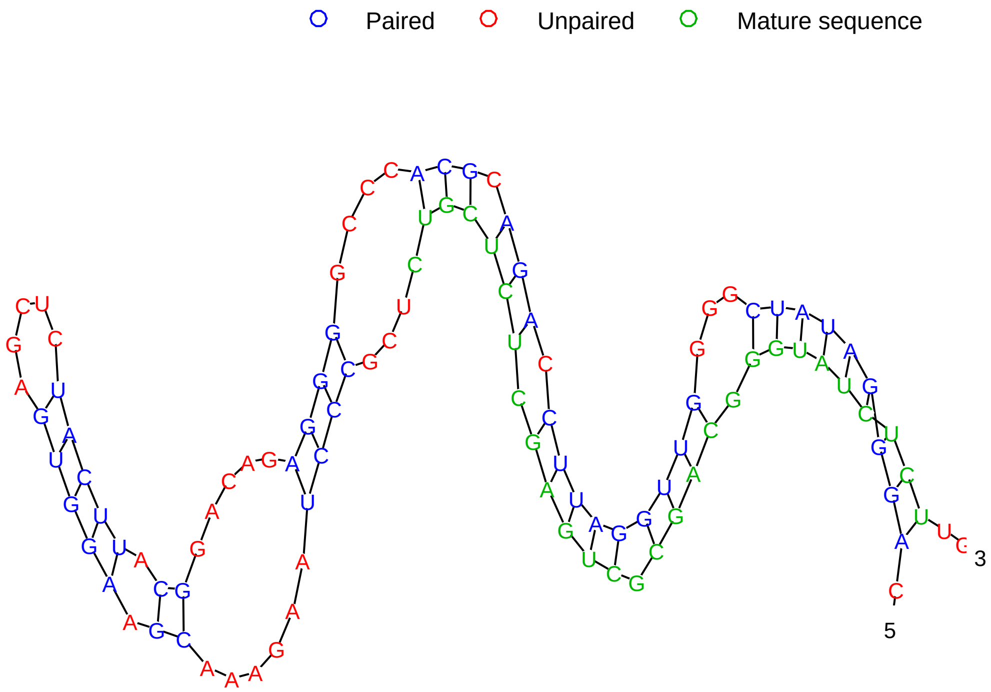

Stem loop (UMD3.1): chr4:30959-31065
Mature (UMD3.1): chr4:31036-31063
Mature seq len: 28
Total raw counts (9 samples): 11594
Average raw counts: 1289
Strand: Forward
Orientation: 3p
Minimum free energy: -37.40

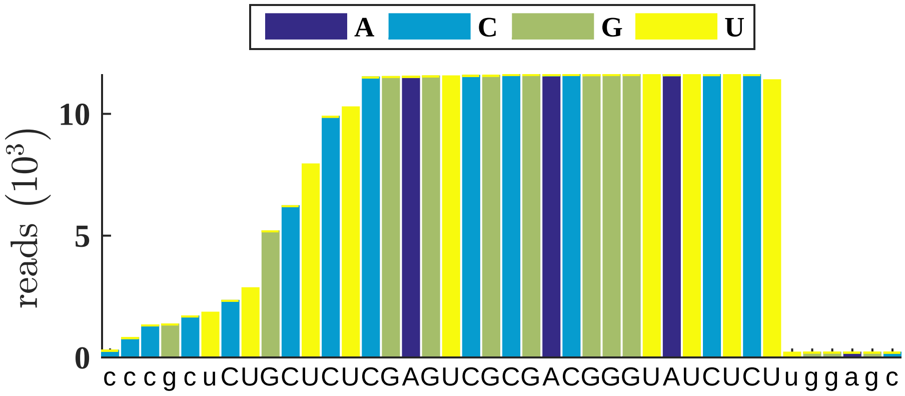

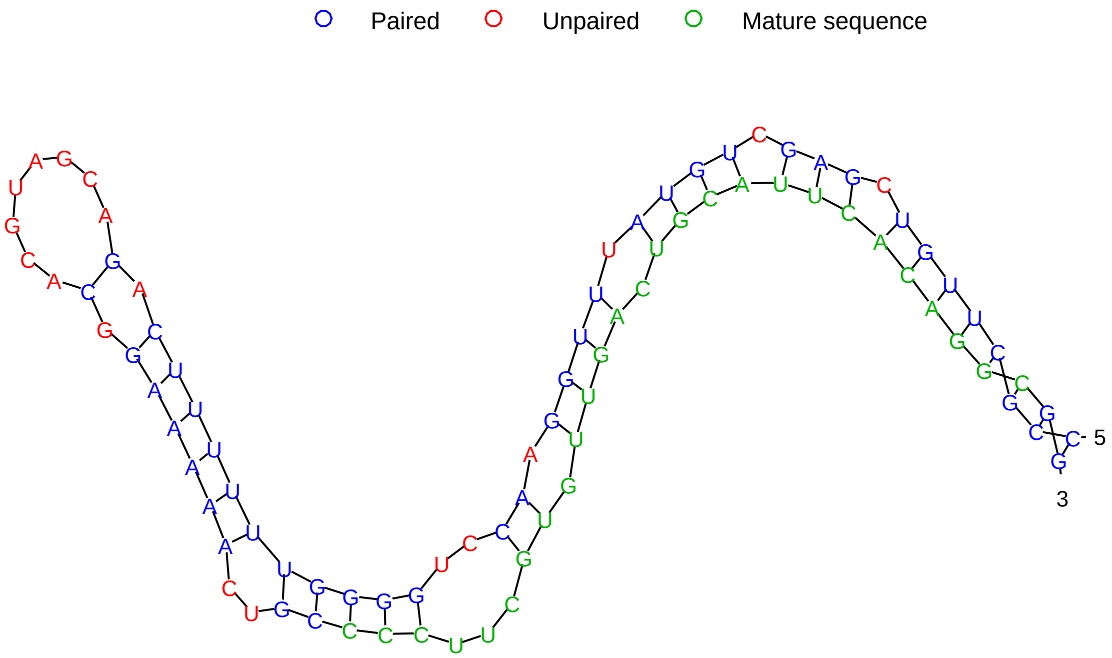

Stem loop (UMD3.1): chr4:33563979-33564067
Mature (UMD3.1): chr4:33563981-33564007
Mature seq len: 27
Total raw counts (9 samples): 1231
Average raw counts: 137
Strand: Reverse
Orientation: 3p
Minimum free energy: -27.90

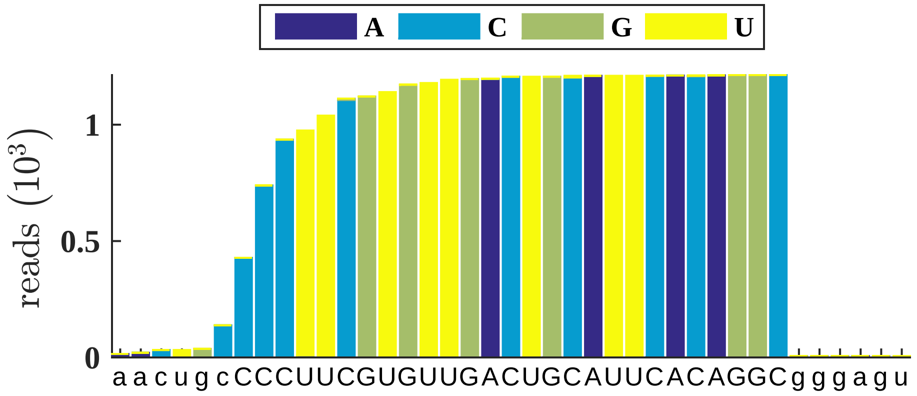

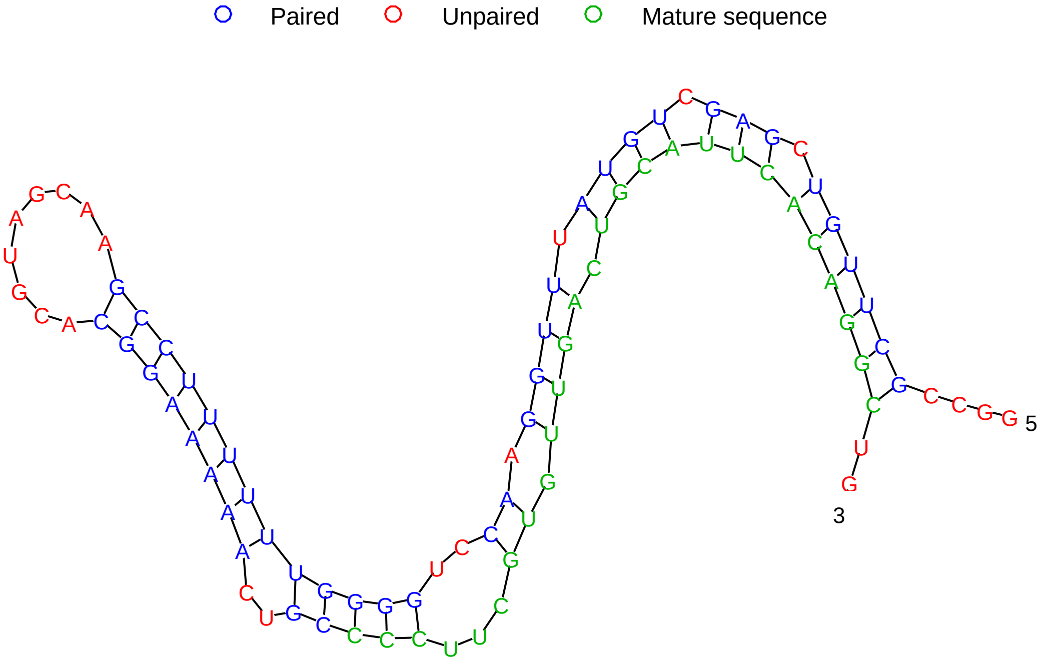

Stem loop (UMD3.1): chr4:33573068-33573159
Mature (UMD3.1): chr4:33573131-33573157
Mature seq len: 27
Total raw counts (9 samples): 1141
Average raw counts: 127
Strand: Forward
Orientation: 3p
Minimum free energy: -28.90

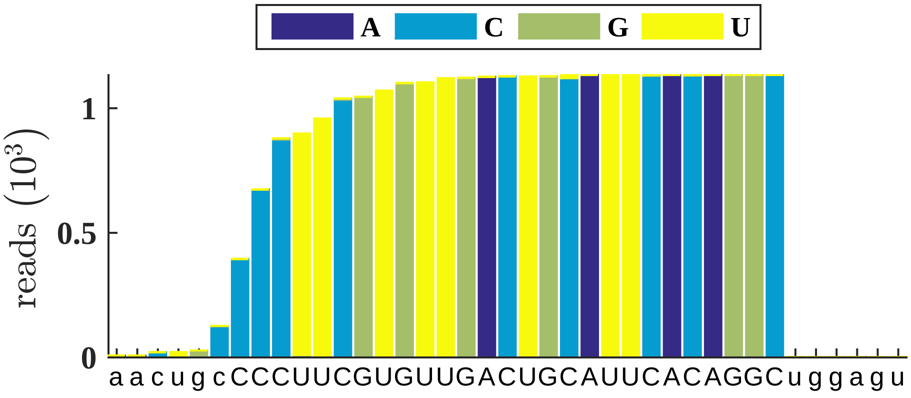

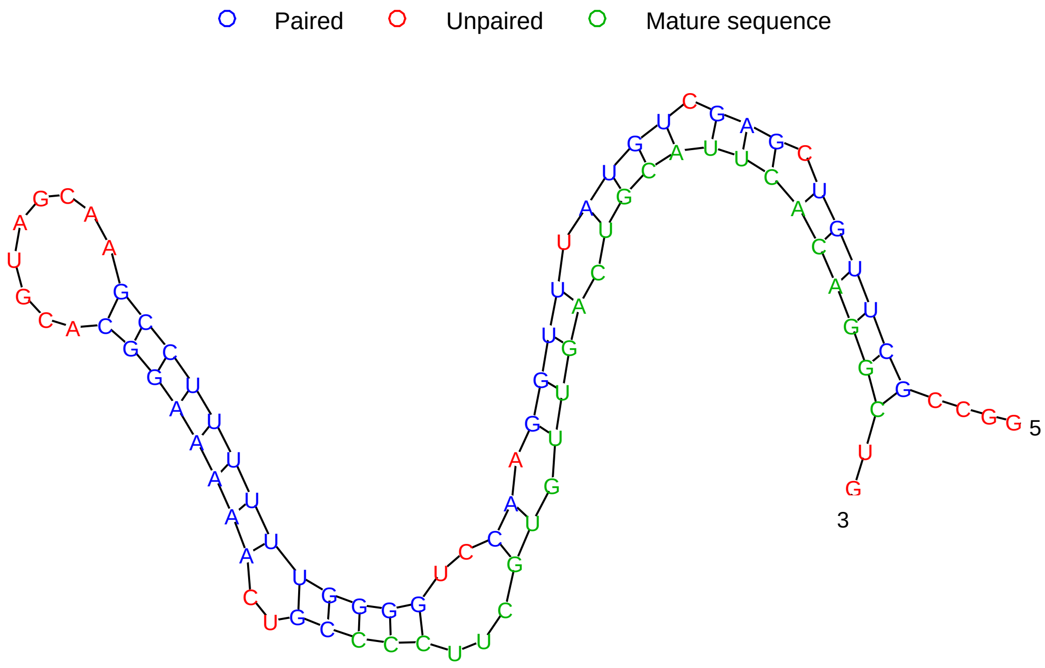

Stem loop (UMD3.1): chr4:33579004-33579095
Mature (UMD3.1): chr4:33579006-33579032
Mature seq len: 27
Total raw counts (9 samples): 1174
Average raw counts: 131
Strand: Reverse
Orientation: 3p
Minimum free energy: -28.90

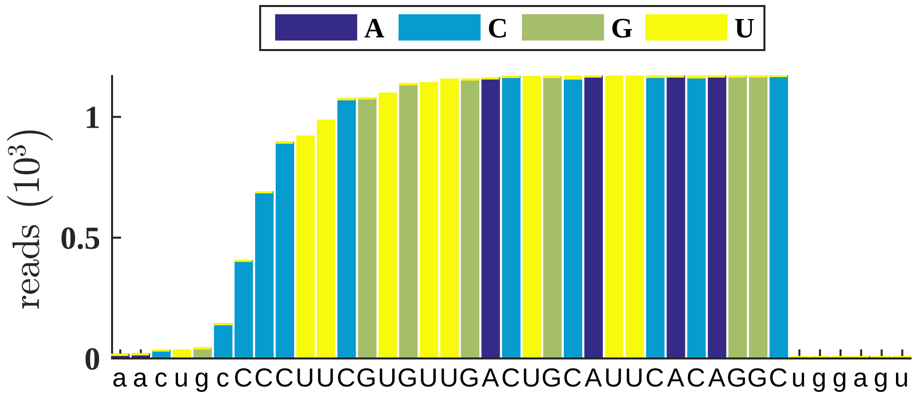

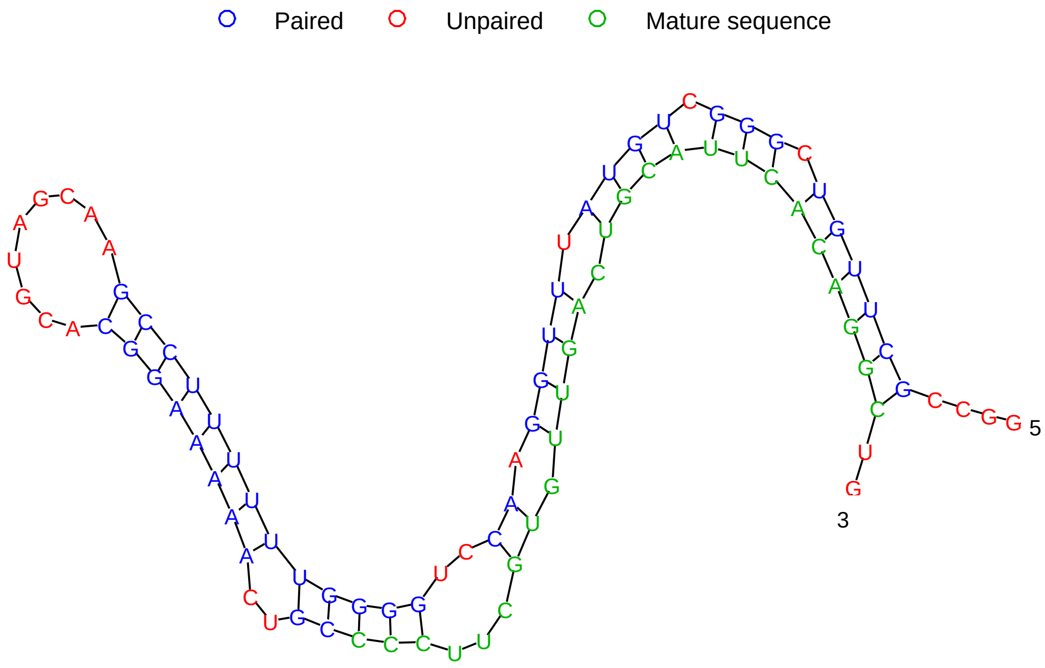

Stem loop (UMD3.1): chr4:33581001-33581092
Mature (UMD3.1): chr4:33581064-33581090
Mature seq len: 27
Total raw counts (9 samples): 1174
Average raw counts: 131
Strand: Forward
Orientation: 3p
Minimum free energy: -27.90

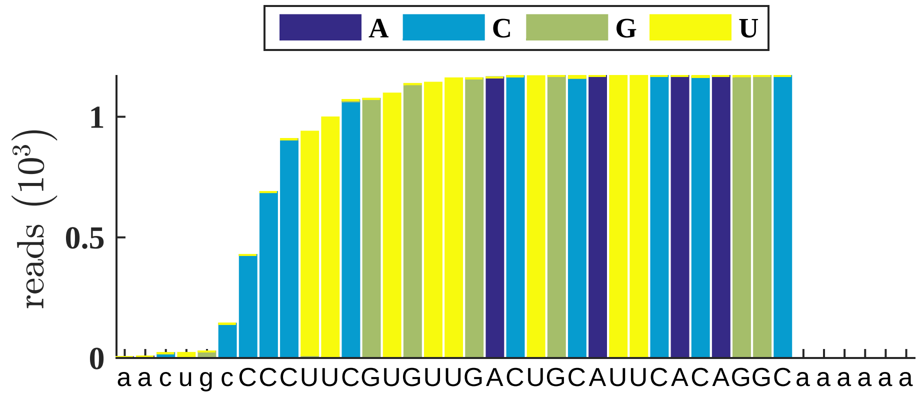

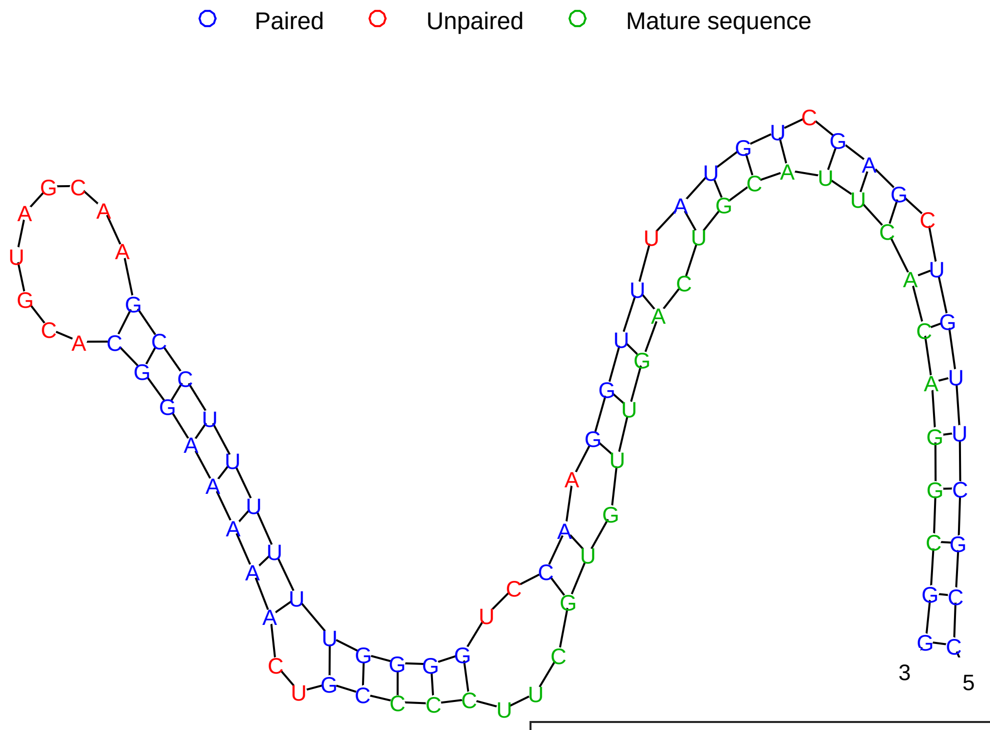

Stem loop (UMD3.1): chr4:35242250-35242339
Mature (UMD3.1): chr4:35242311-35242337
Mature seq len: 27
Total raw counts (9 samples): 1163
Average raw counts: 130
Strand: Forward
Orientation: 3p
Minimum free energy: -33.20

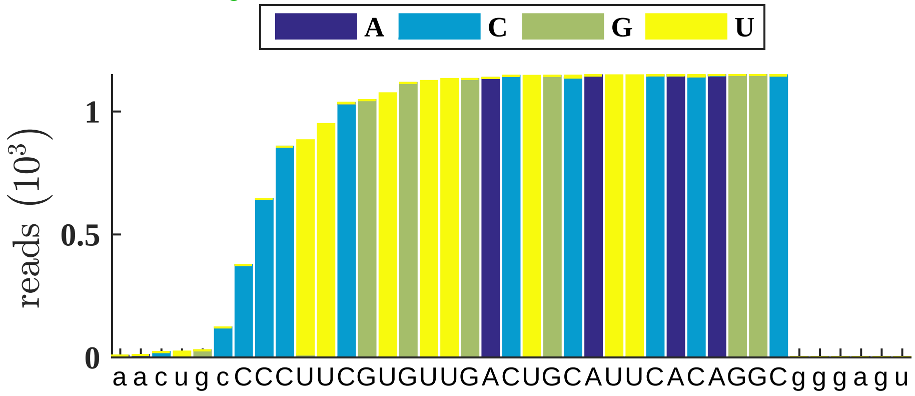

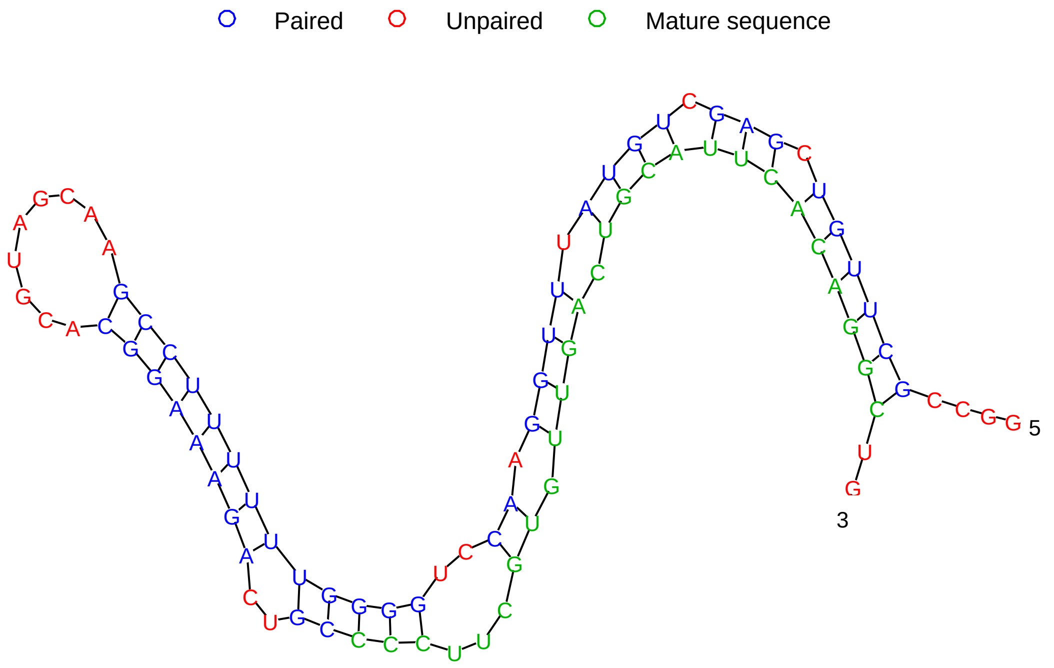

Stem loop (UMD3.1): chr4:35244933-35245024
Mature (UMD3.1): chr4:35244996-35245022
Mature seq len: 27
Total raw counts (9 samples): 1270
Average raw counts: 142
Strand: Forward
Orientation: 3p
Minimum free energy: -29.00

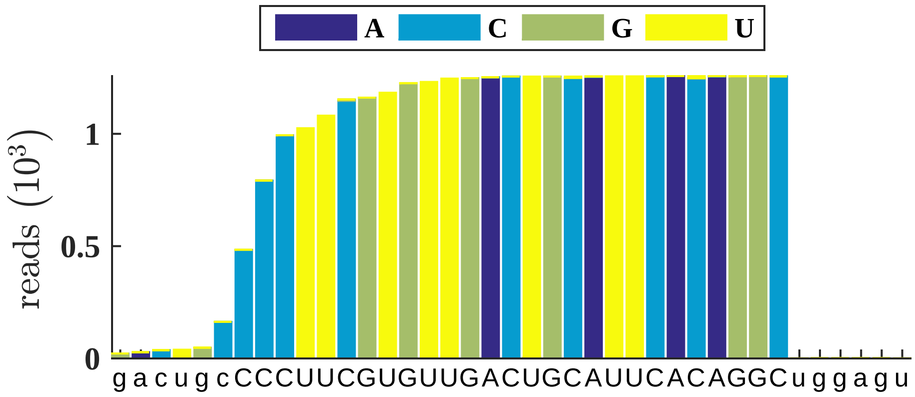

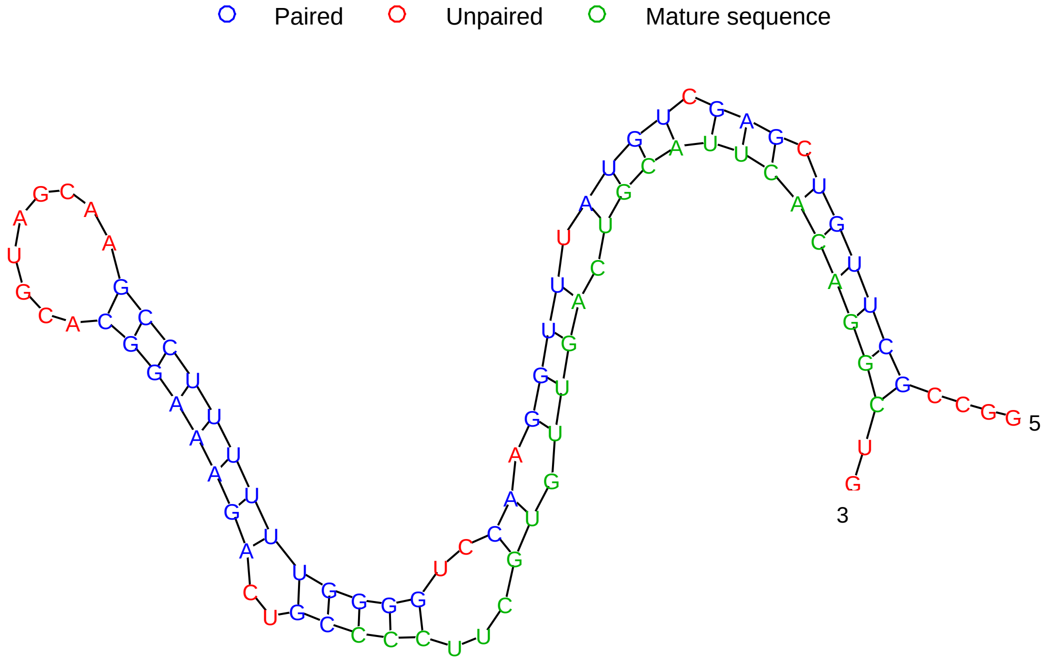

Stem loop (UMD3.1): chr4:35246317-35246408
Mature (UMD3.1): chr4:35246380-35246406
Mature seq len: 27
Total raw counts (9 samples): 1181
Average raw counts: 132
Strand: Forward
Orientation: 3p
Minimum free energy: -29.00

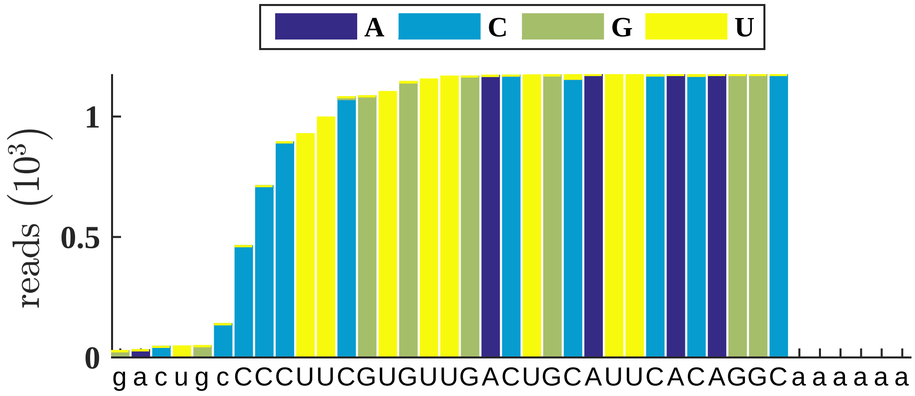

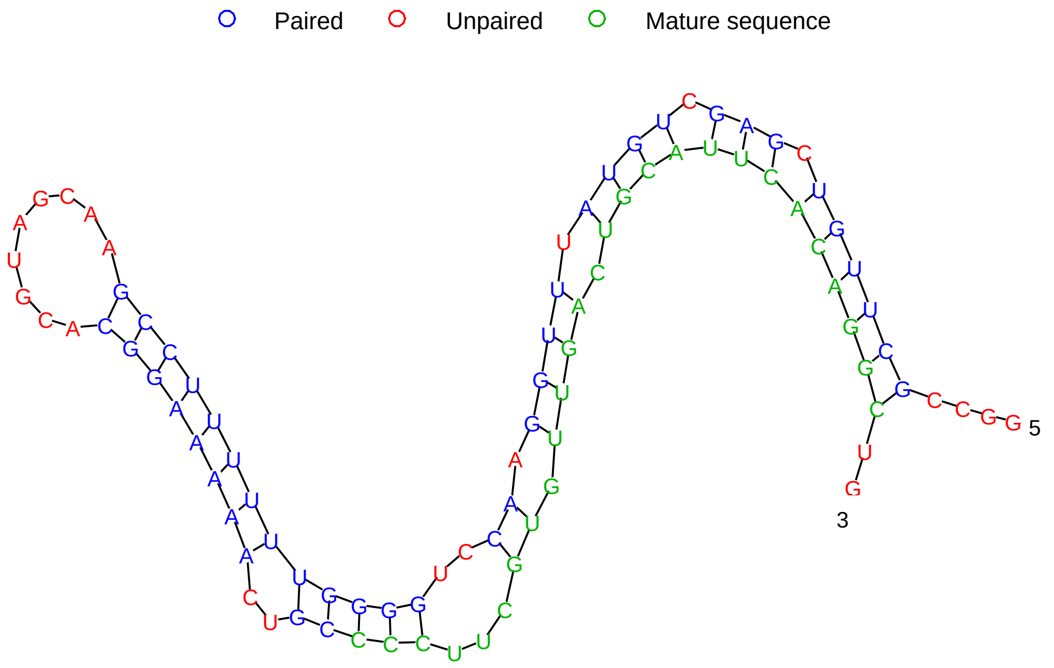

Stem loop (UMD3.1): chr4:35247822-35247913
Mature (UMD3.1): chr4:35247885-35247911
Mature seq len: 27
Total raw counts (9 samples): 1208
Average raw counts: 135
Strand: Forward
Orientation: 3p
Minimum free energy: -28.90

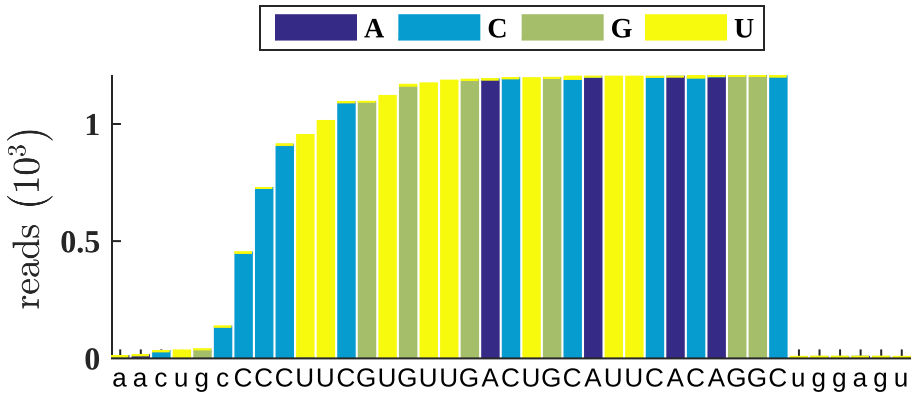

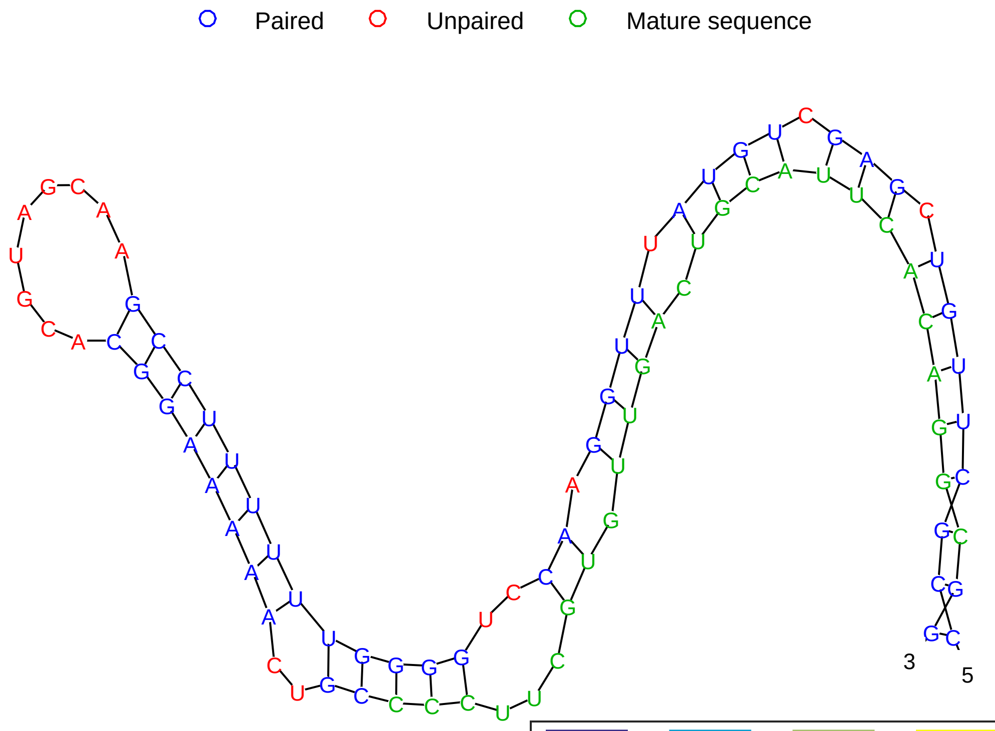

Stem loop (UMD3.1): chr4:35250254-35250343
Mature (UMD3.1): chr4:35250256-35250282
Mature seq len: 27
Total raw counts (9 samples): 1284
Average raw counts: 143
Strand: Reverse
Orientation: 3p
Minimum free energy: -33.20

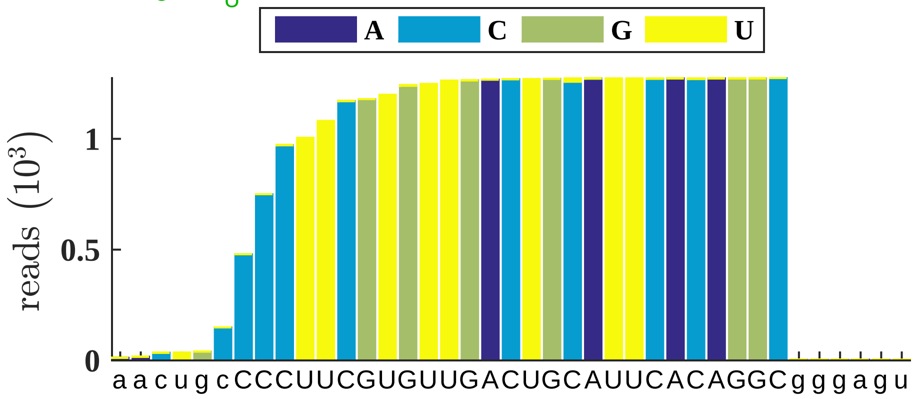

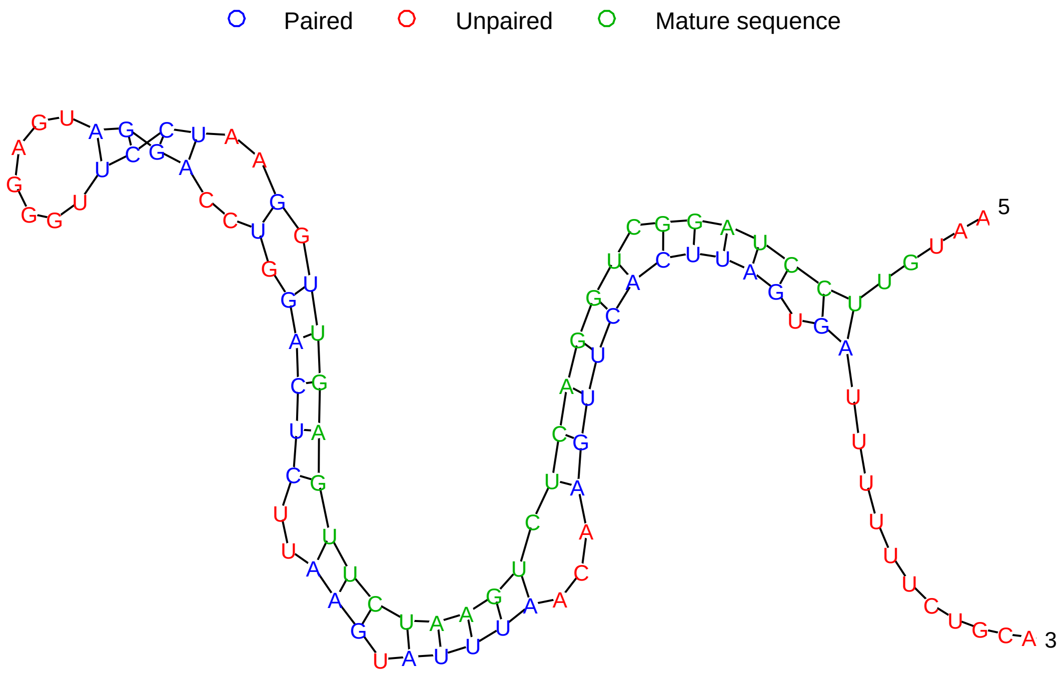

Stem loop (UMD3.1): chr4:46639499-46639598
Mature (UMD3.1): chr4:46639567-46639595
Mature seq len: 29
Total raw counts (9 samples): 1841
Average raw counts: 205
Strand: Reverse
Orientation: 5p
Minimum free energy: -23.40

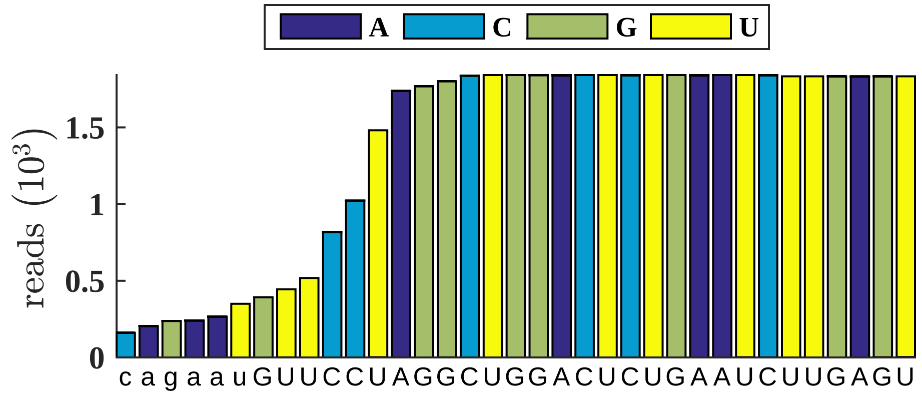

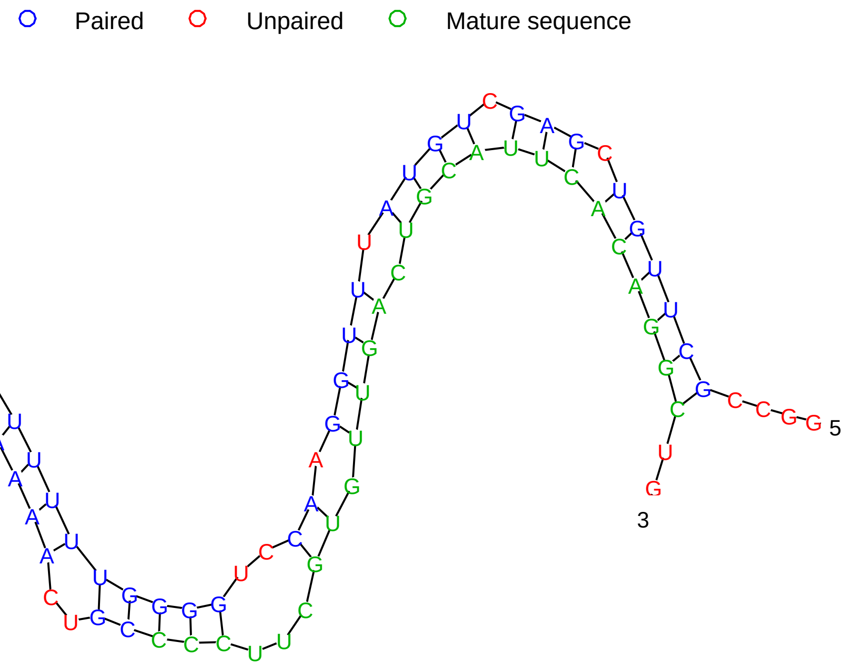

Stem loop (UMD3.1): chr4:48250368-48250459
Mature (UMD3.1): chr4:48250431-48250457
Mature seq len: 27
Total raw counts (9 samples): 1198
Average raw counts: 134
Strand: Forward
Orientation: 3p
Minimum free energy: -28.90

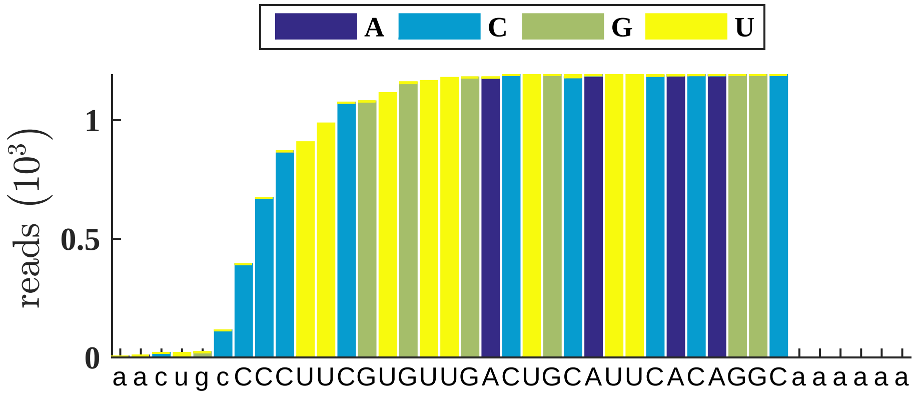

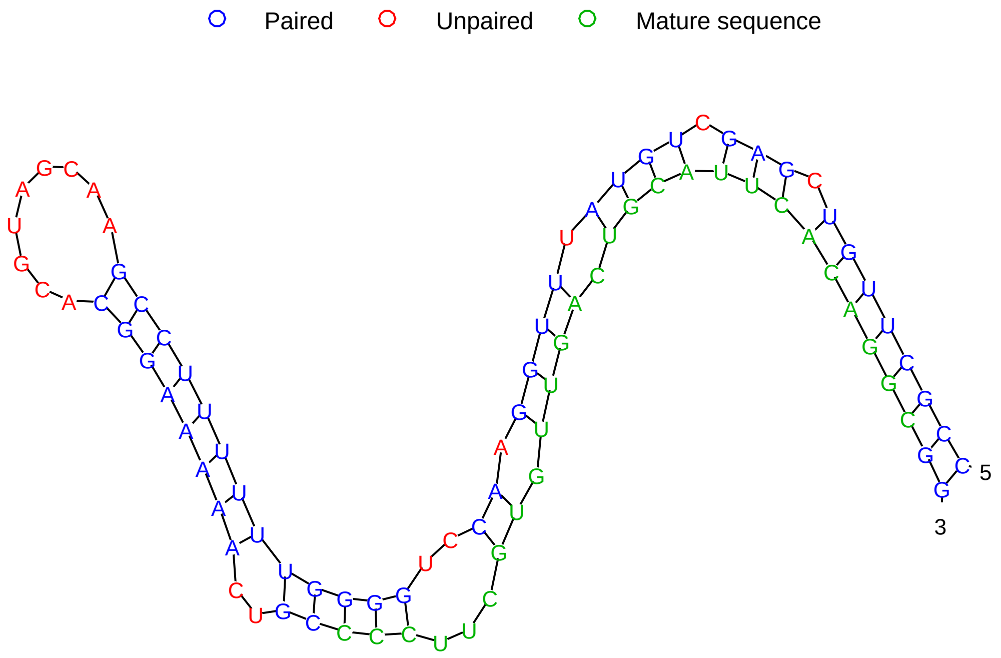

Stem loop (UMD3.1): chr4:48284629-48284718
Mature (UMD3.1): chr4:48284690-48284716
Mature seq len: 27
Total raw counts (9 samples): 1222
Average raw counts: 136
Strand: Forward
Orientation: 3p
Minimum free energy: -33.20

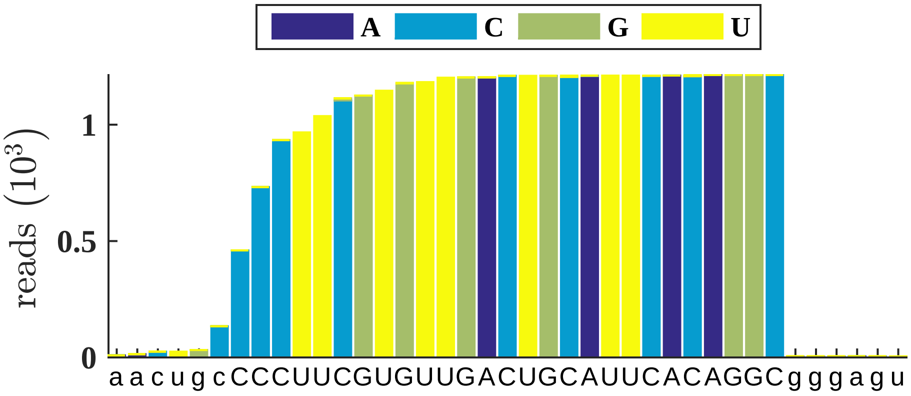

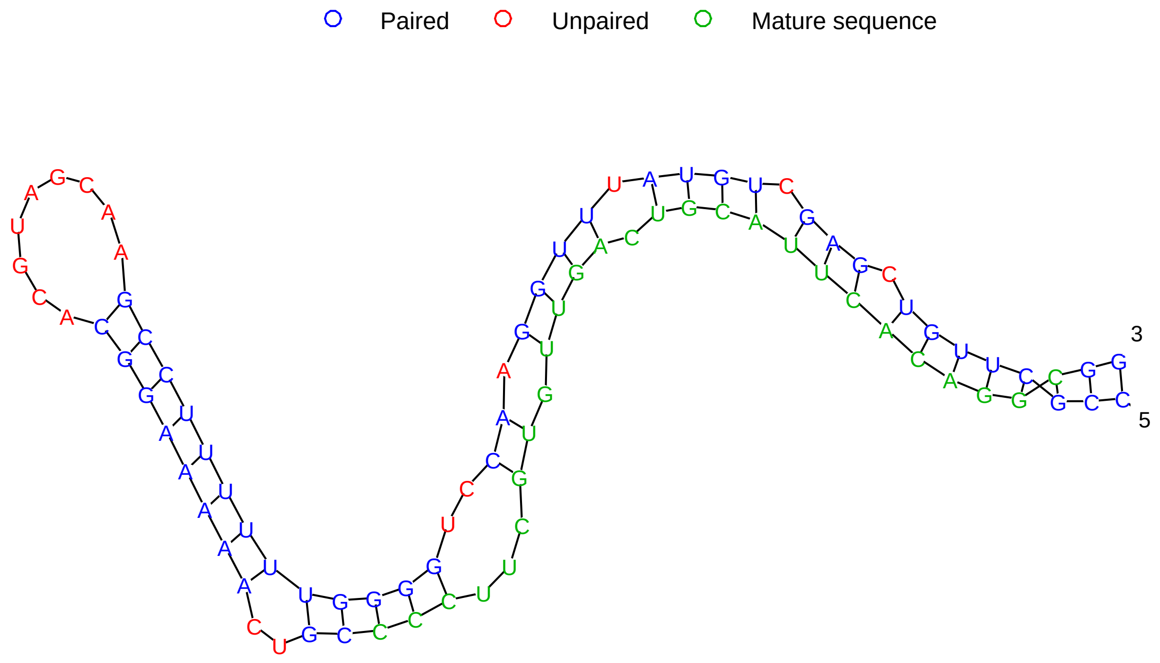

Stem loop (UMD3.1): chr4:66534507-66534596
Mature (UMD3.1): chr4:66534568-66534594
Mature seq len: 27
Total raw counts (9 samples): 1176
Average raw counts: 131
Strand: Forward
Orientation: 3p
Minimum free energy: -33.20

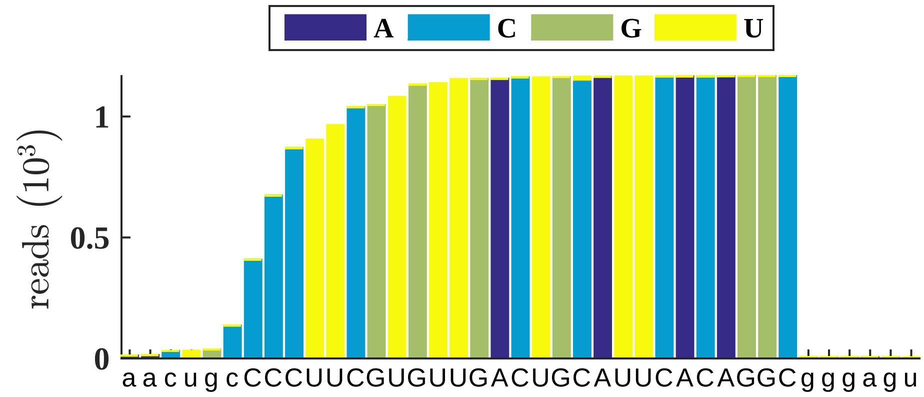

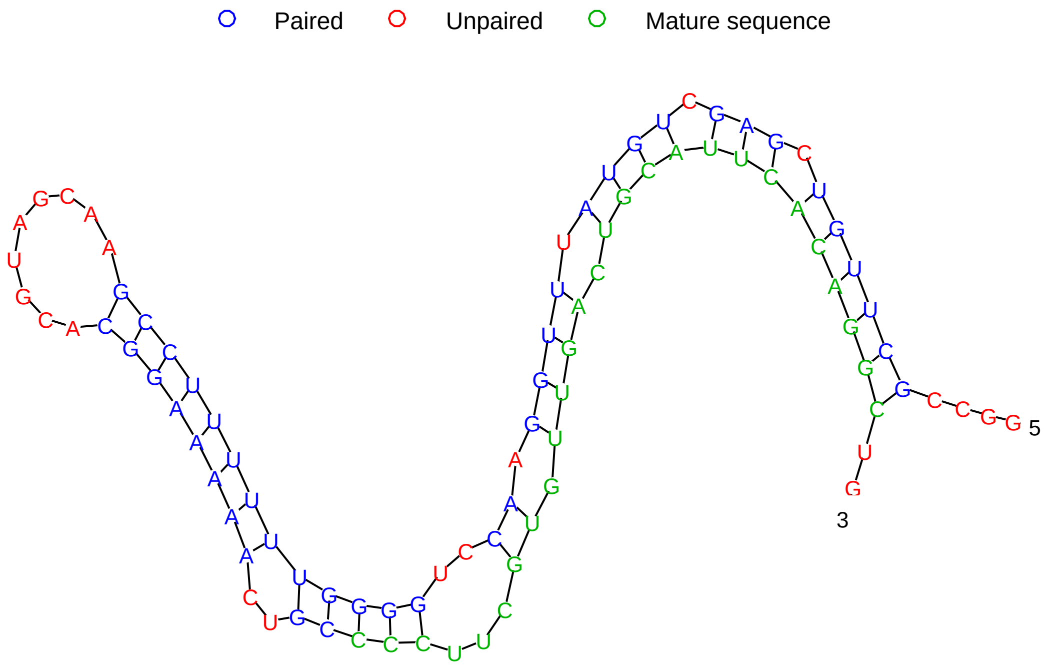

Stem loop (UMD3.1): chr4:66535890-66535981
Mature (UMD3.1): chr4:66535953-66535979
Mature seq len: 27
Total raw counts (9 samples): 1181
Average raw counts: 132
Strand: Forward
Orientation: 3p
Minimum free energy: -28.90

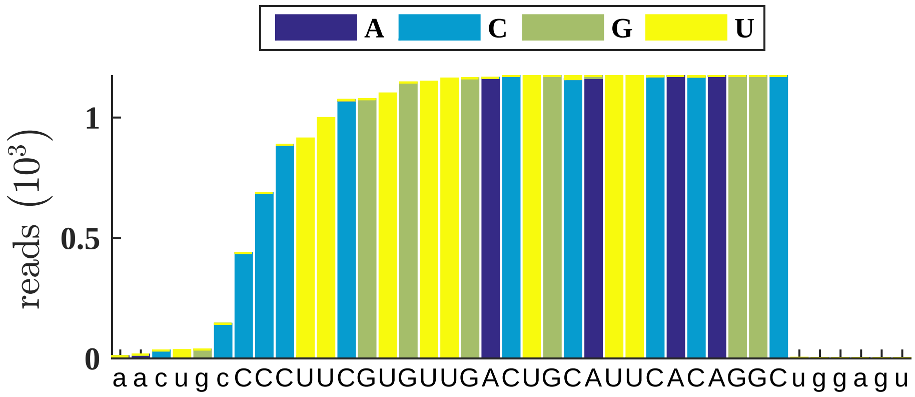

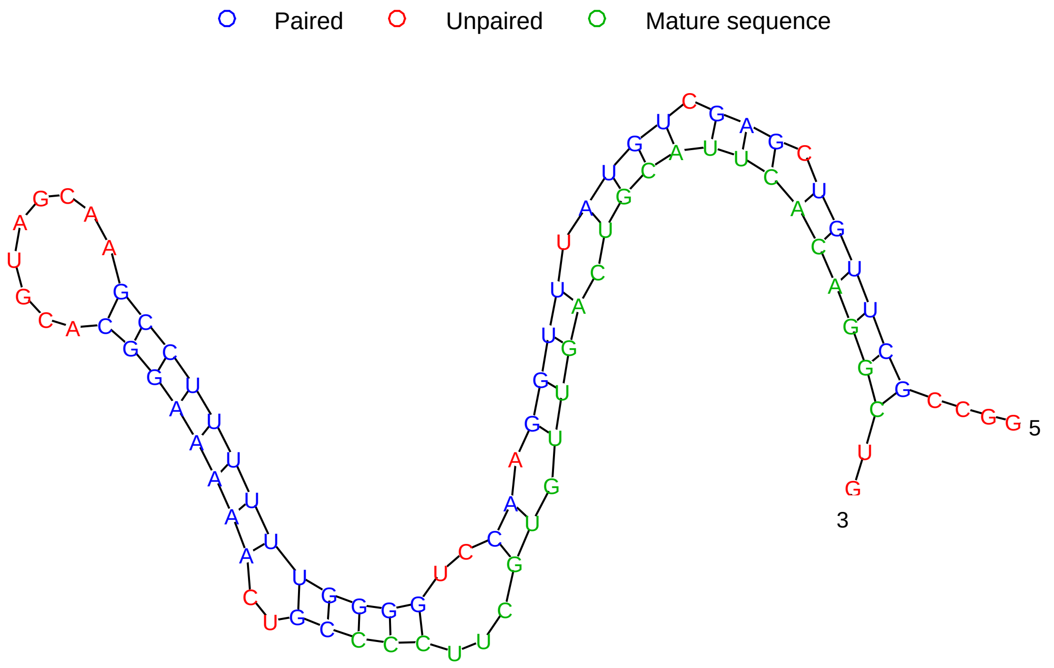

Stem loop (UMD3.1): chr4:66537632-66537723
Mature (UMD3.1): chr4:66537695-66537721
Mature seq len: 27
Total raw counts (9 samples): 1192
Average raw counts: 133
Strand: Forward
Orientation: 3p
Minimum free energy: -28.90

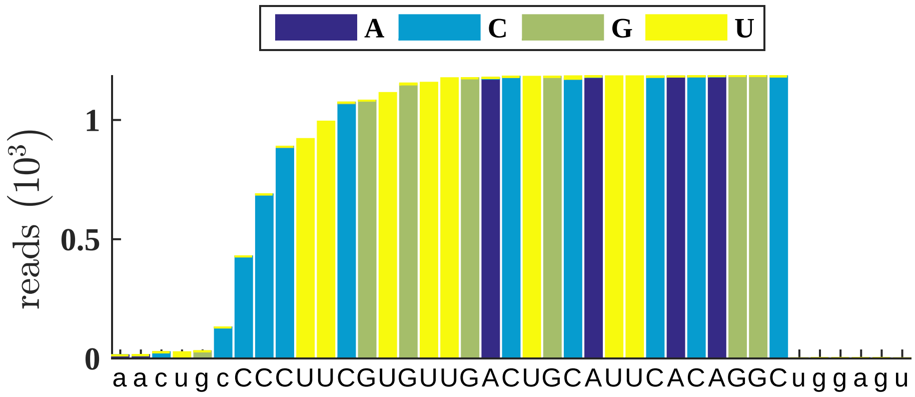

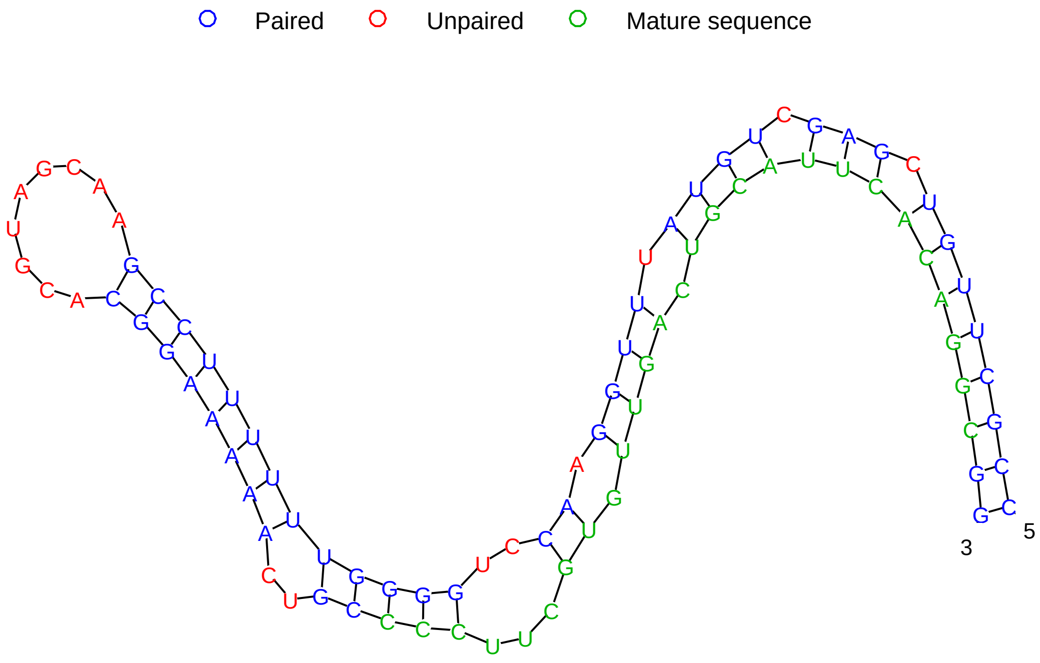

Stem loop (UMD3.1): chr4:66542038-66542127
Mature (UMD3.1): chr4:66542099-66542125
Mature seq len: 27
Total raw counts (9 samples): 1167
Average raw counts: 130
Strand: Forward
Orientation: 3p
Minimum free energy: -33.20

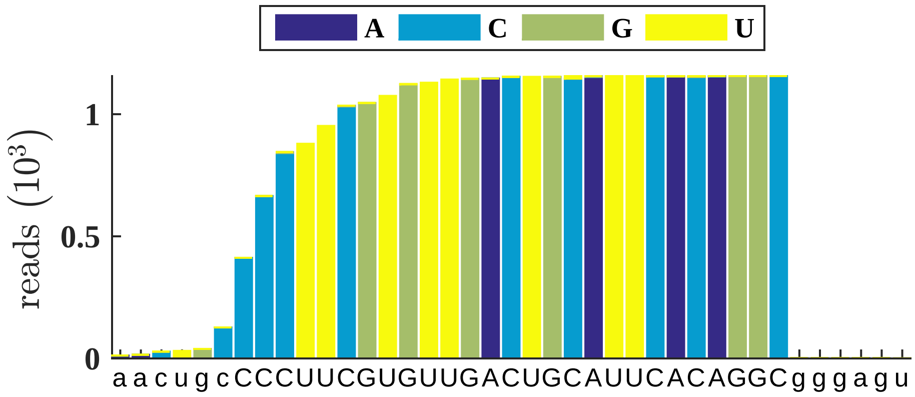

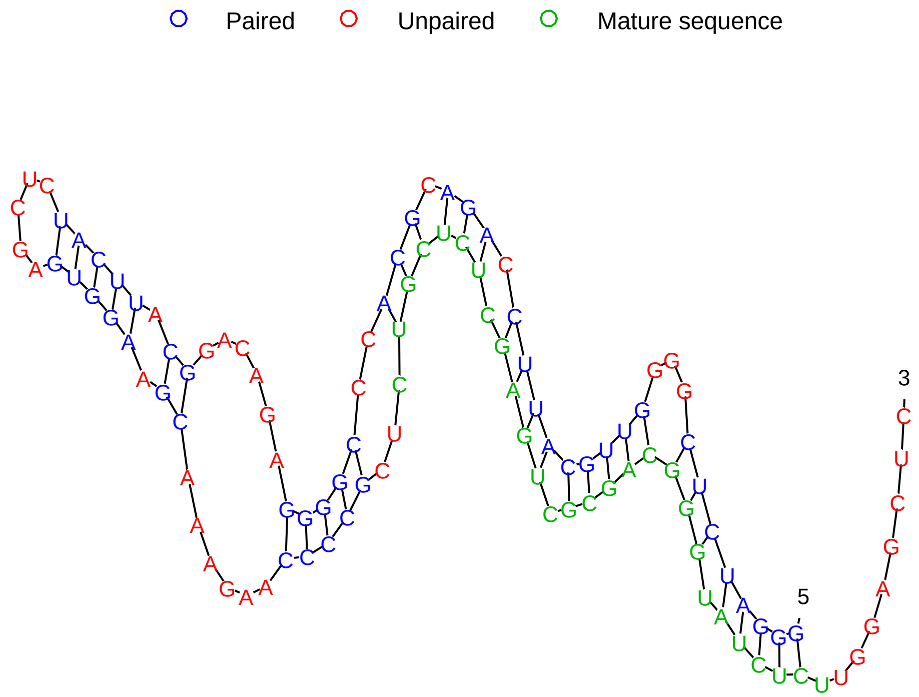

Stem loop (UMD3.1): chr4:66549053-66549163
Mature (UMD3.1): chr4:66549128-66549155
Mature seq len: 28
Total raw counts (9 samples): 13075
Average raw counts: 1453
Strand: Forward
Orientation: 3p
Minimum free energy: -35.50

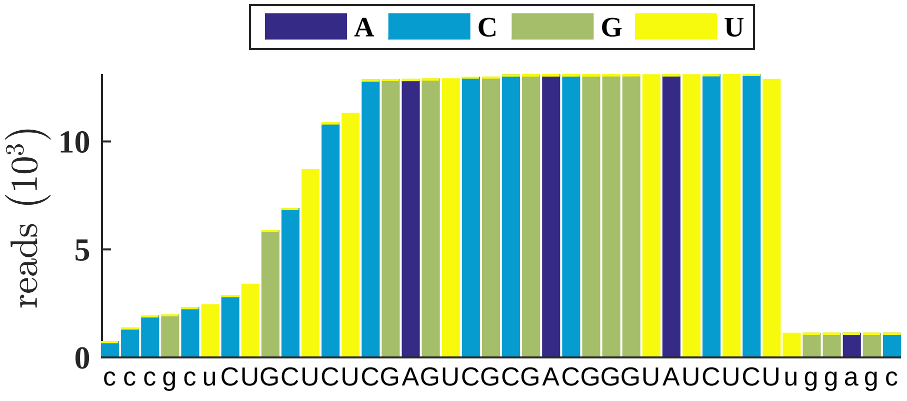

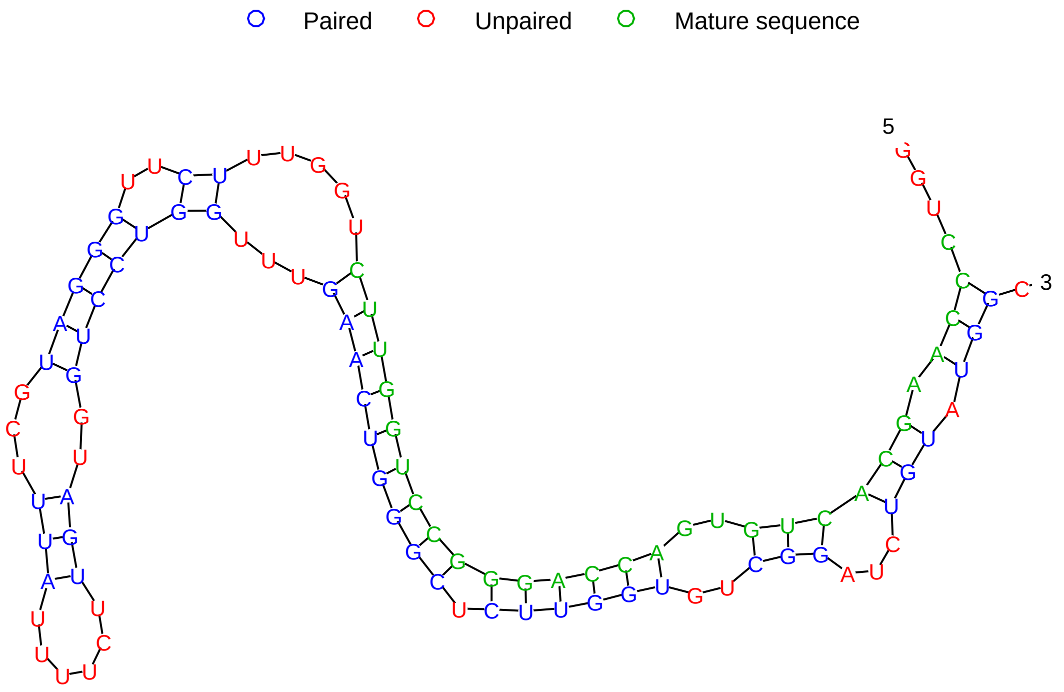

Stem loop (UMD3.1): chr4:79636295-79636398
Mature (UMD3.1): chr4:79636298-79636325
Mature seq len: 28
Total raw counts (9 samples): 888
Average raw counts: 99
Strand: Forward
Orientation: 5p
Minimum free energy: -33.10

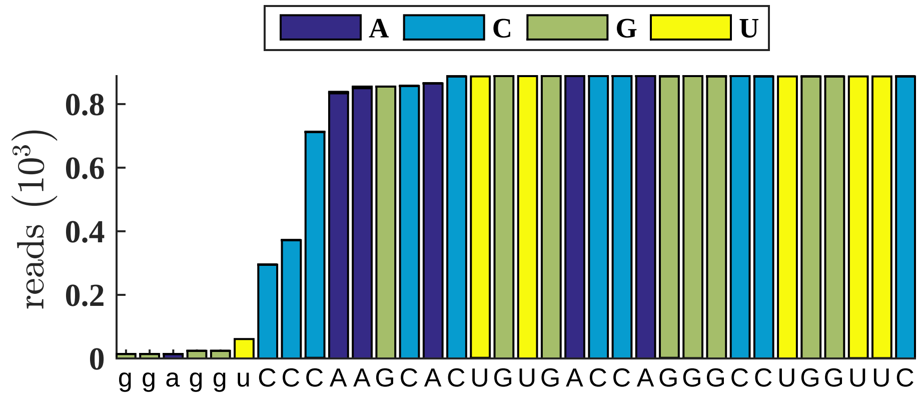

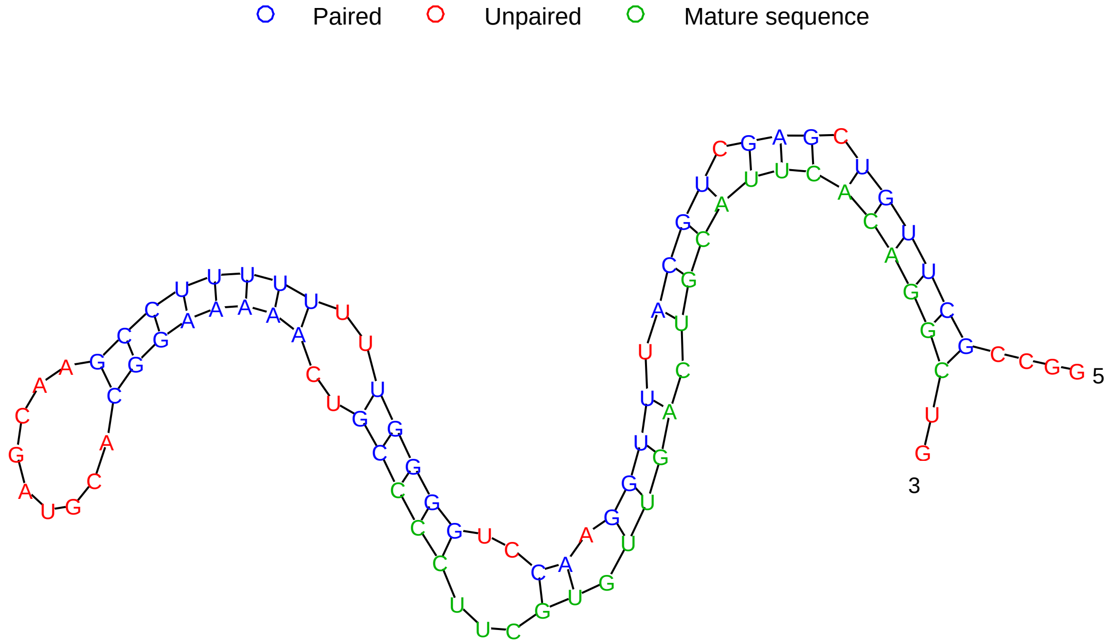

Stem loop (UMD3.1): chr4:9118591-9118684
Mature (UMD3.1): chr4:9118656-9118682
Mature seq len: 27
Total raw counts (9 samples): 1180
Average raw counts: 132
Strand: Forward
Orientation: 3p
Minimum free energy: -32.10

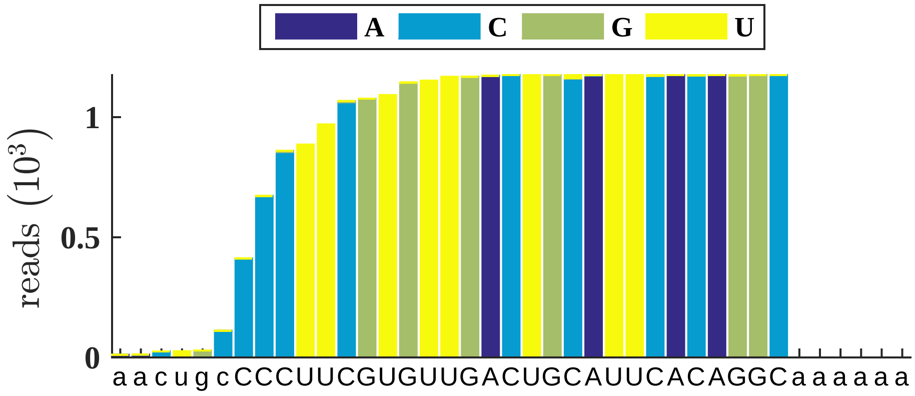

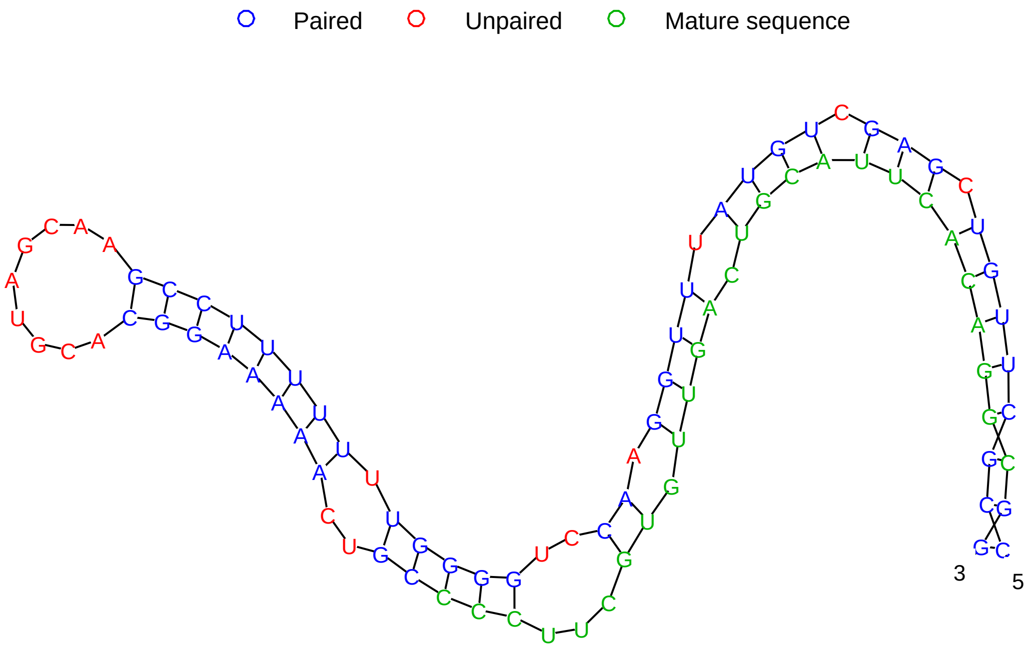

Stem loop (UMD3.1): chr4:9121259-9121349
Mature (UMD3.1): chr4:9121321-9121347
Mature seq len: 27
Total raw counts (9 samples): 1153
Average raw counts: 129
Strand: Forward
Orientation: 3p
Minimum free energy: -34.00

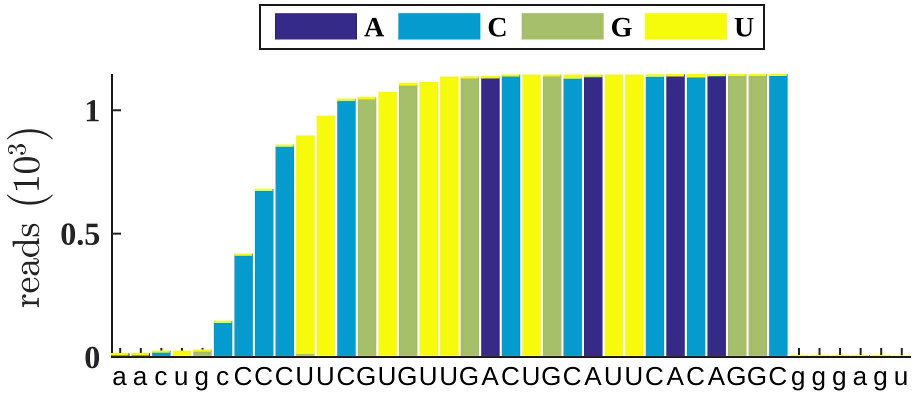

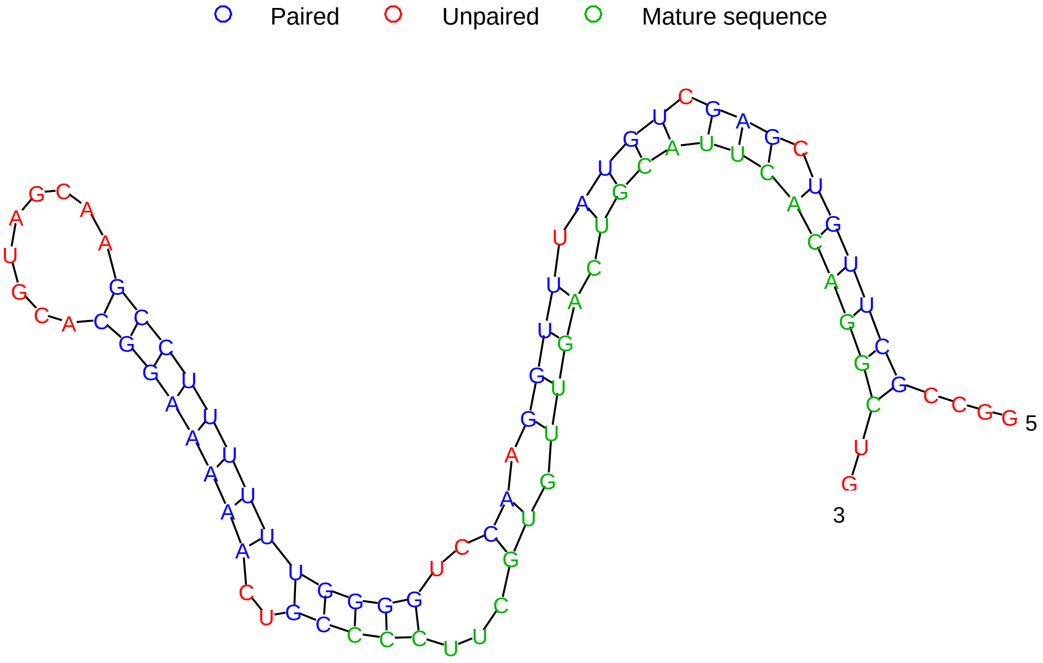

Stem loop (UMD3.1): chr4:95732843-95732934
Mature (UMD3.1): chr4:95732906-95732932
Mature seq len: 27
Total raw counts (9 samples): 1248
Average raw counts: 139
Strand: Forward
Orientation: 3p
Minimum free energy: -28.90

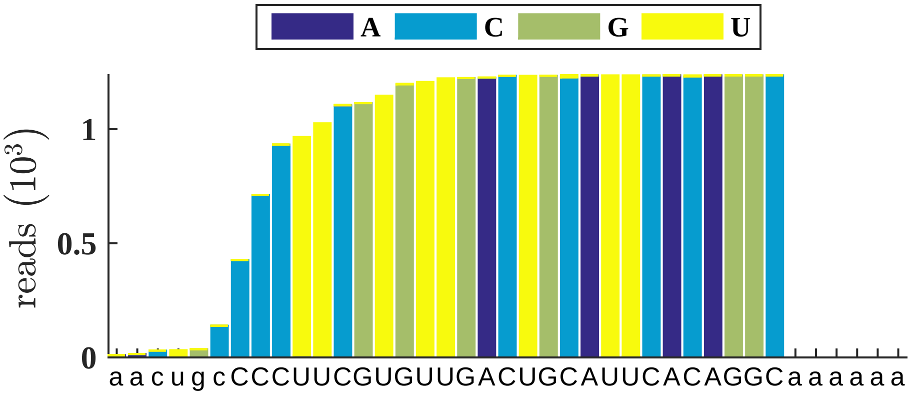

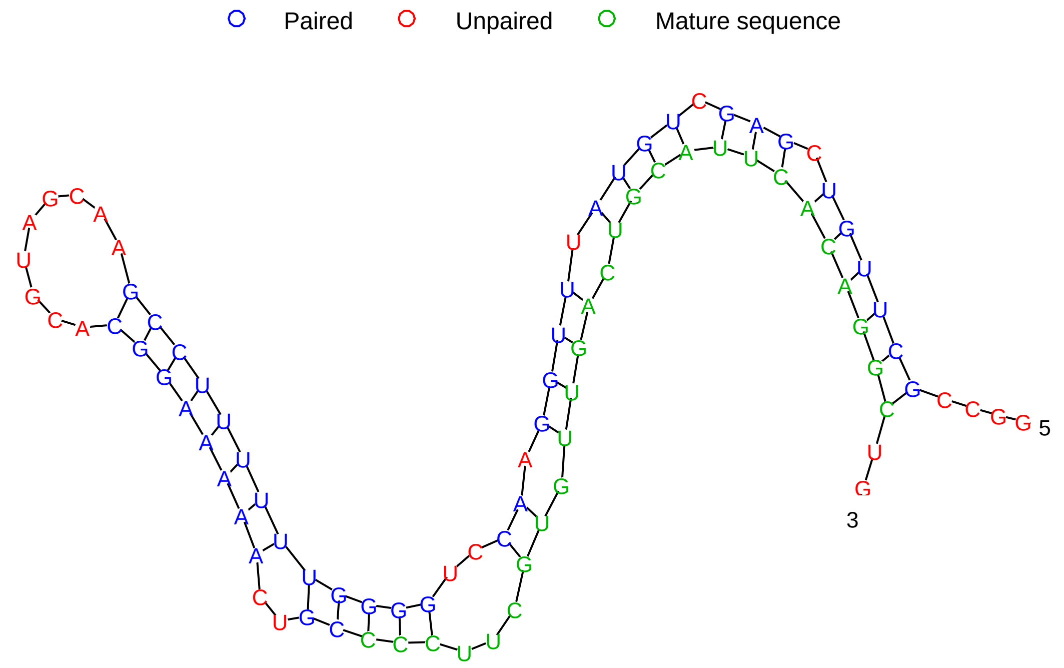

Stem loop (UMD3.1): chr4:95738005-95738096
Mature (UMD3.1): chr4:95738007-95738033
Mature seq len: 27
Total raw counts (9 samples): 1160
Average raw counts: 129
Strand: Reverse
Orientation: 3p
Minimum free energy: -28.90

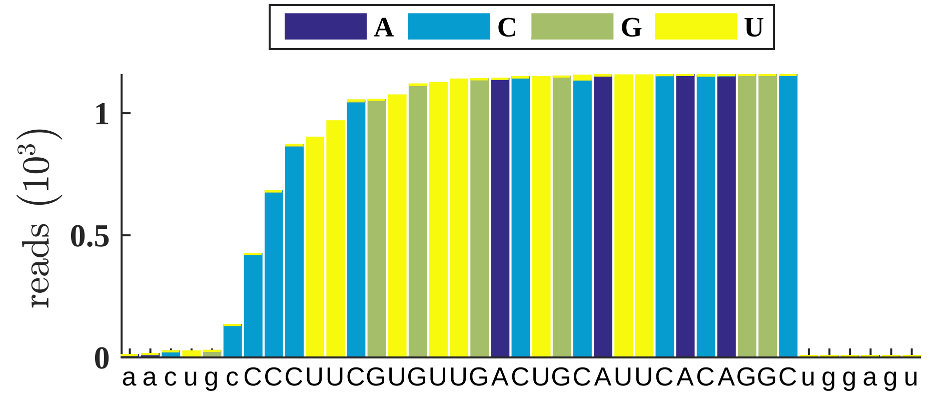

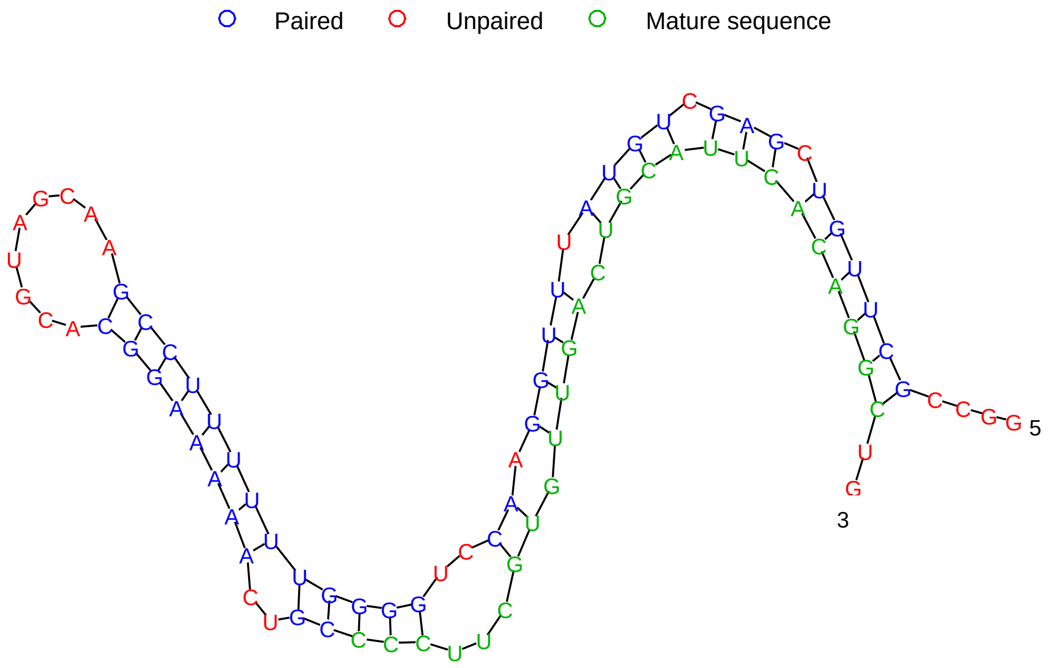

Stem loop (UMD3.1): chr4:95739687-95739778
Mature (UMD3.1): chr4:95739689-95739715
Mature seq len: 27
Total raw counts (9 samples): 1241
Average raw counts: 138
Strand: Reverse
Orientation: 3p
Minimum free energy: -28.90

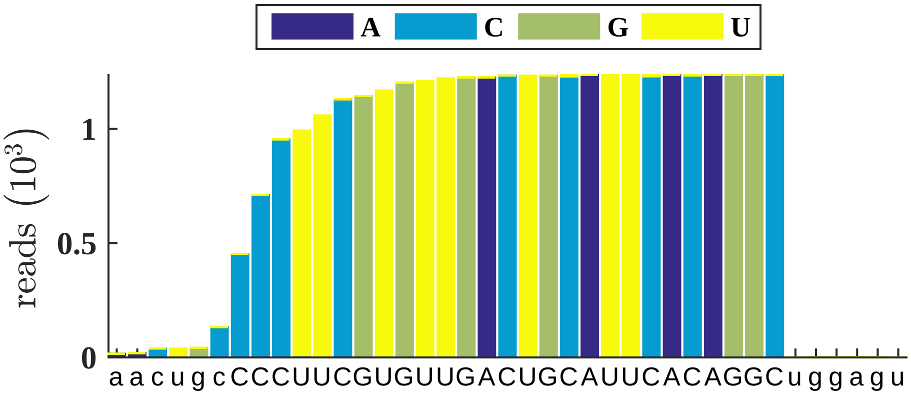

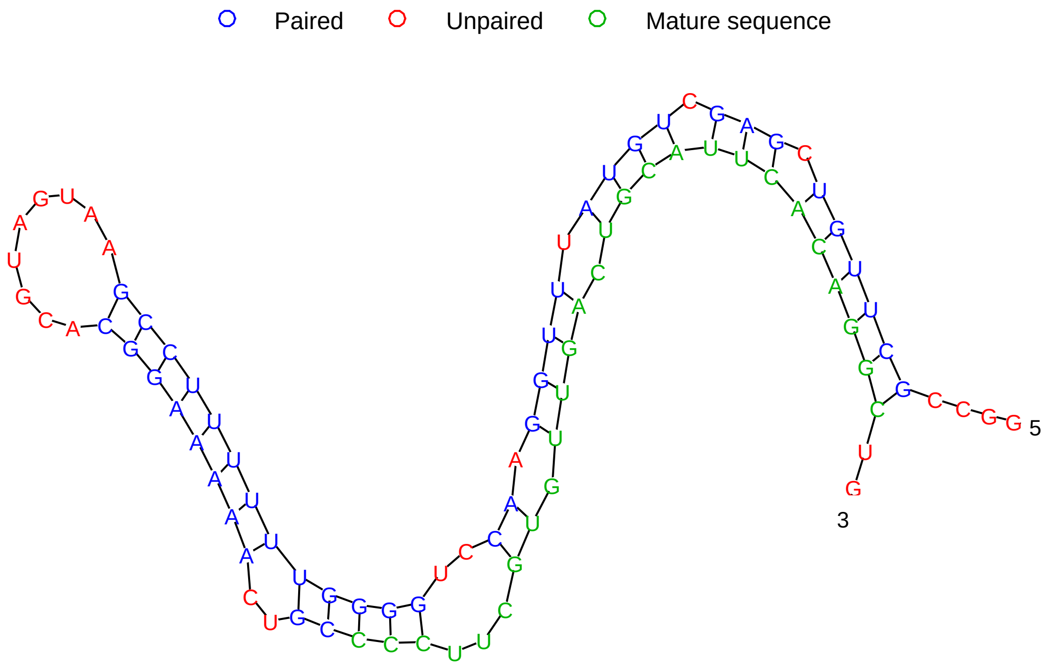

Stem loop (UMD3.1): chr4:95741530-95741621
Mature (UMD3.1): chr4:95741532-95741558
Mature seq len: 27
Total raw counts (9 samples): 1152
Average raw counts: 128
Strand: Reverse
Orientation: 3p
Minimum free energy: -28.90

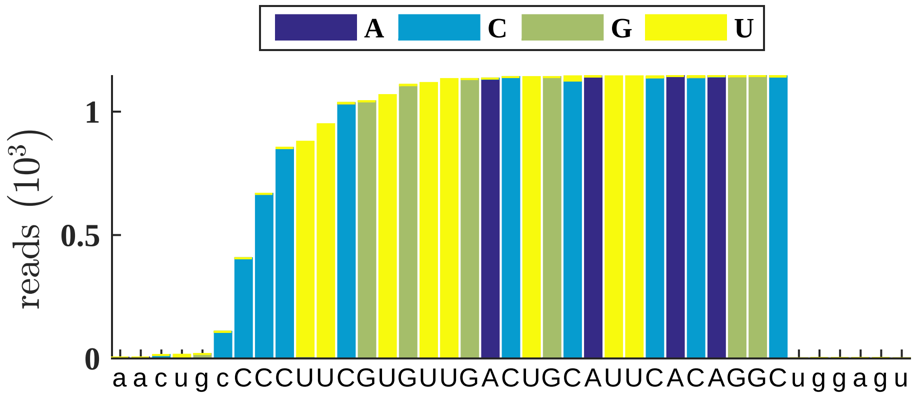

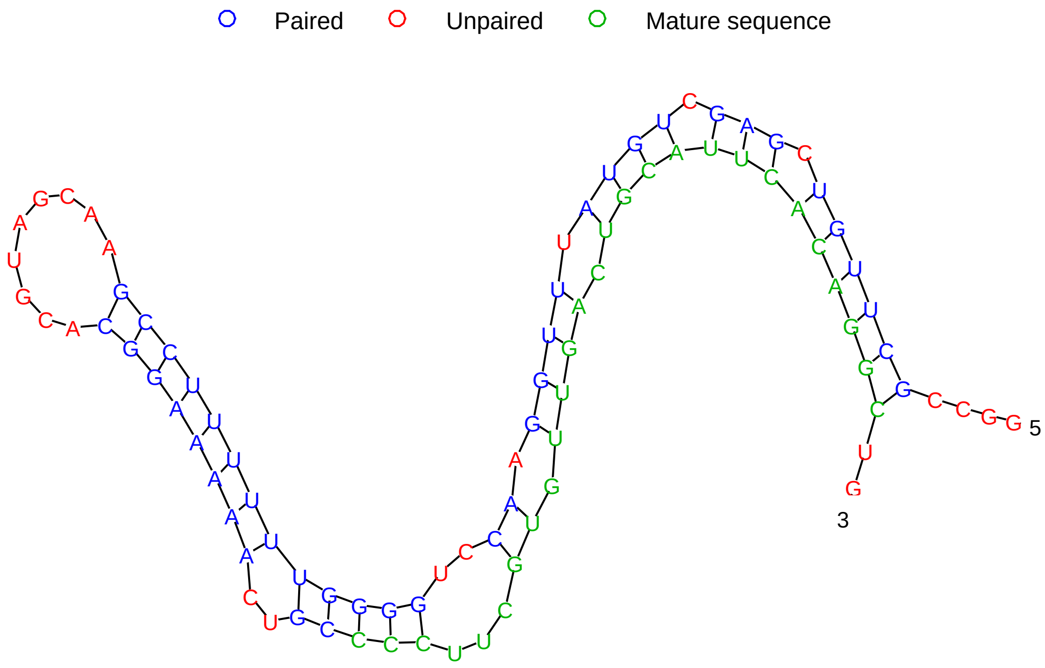

Stem loop (UMD3.1): chr4:95742932-95743023
Mature (UMD3.1): chr4:95742934-95742960
Mature seq len: 27
Total raw counts (9 samples): 1241
Average raw counts: 138
Strand: Reverse
Orientation: 3p
Minimum free energy: -28.90

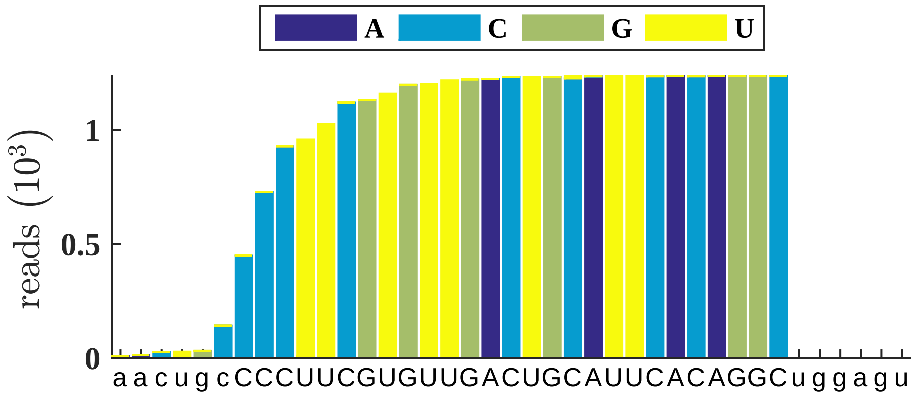

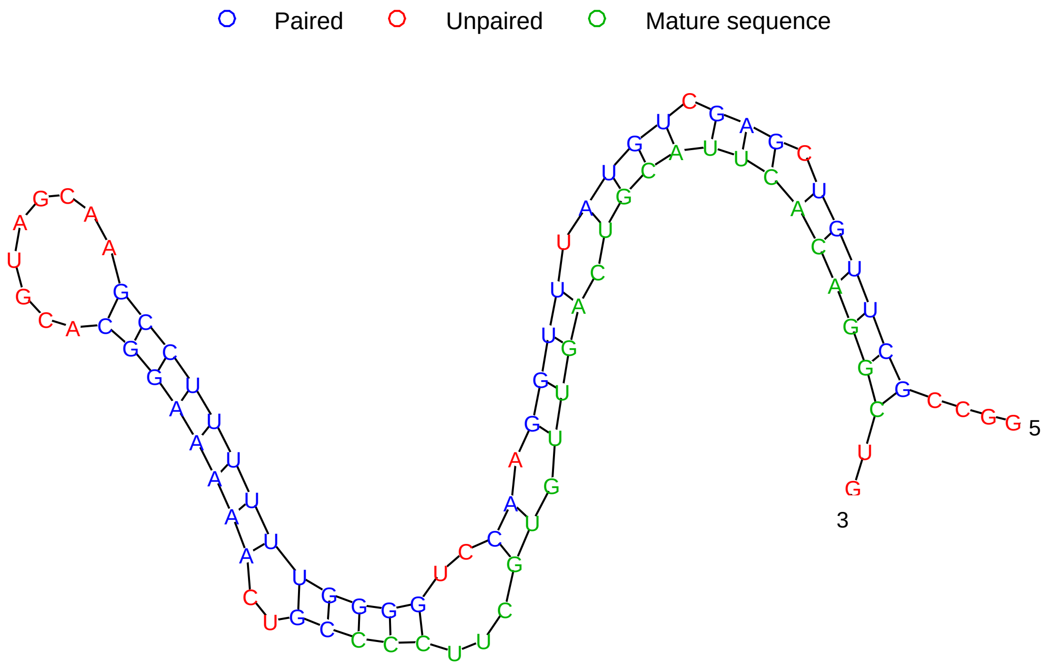

Stem loop (UMD3.1): chr4:95744794-95744885
Mature (UMD3.1): chr4:95744796-95744822
Mature seq len: 27
Total raw counts (9 samples): 1222
Average raw counts: 136
Strand: Reverse
Orientation: 3p
Minimum free energy: -28.90

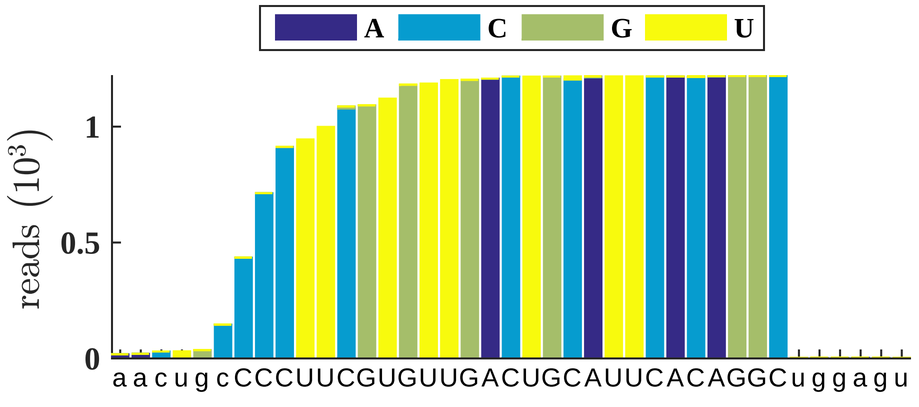

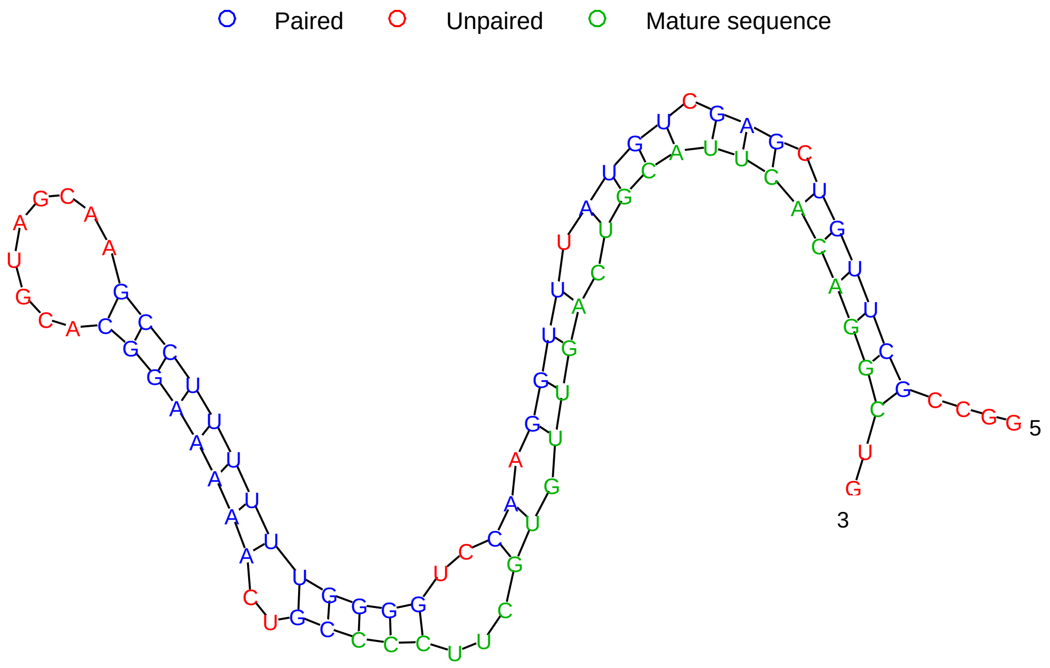

Stem loop (UMD3.1): chr5:103557340-103557431
Mature (UMD3.1): chr5:103557403-103557429
Mature seq len: 27
Total raw counts (9 samples): 1225
Average raw counts: 137
Strand: Forward
Orientation: 3p
Minimum free energy: -28.90

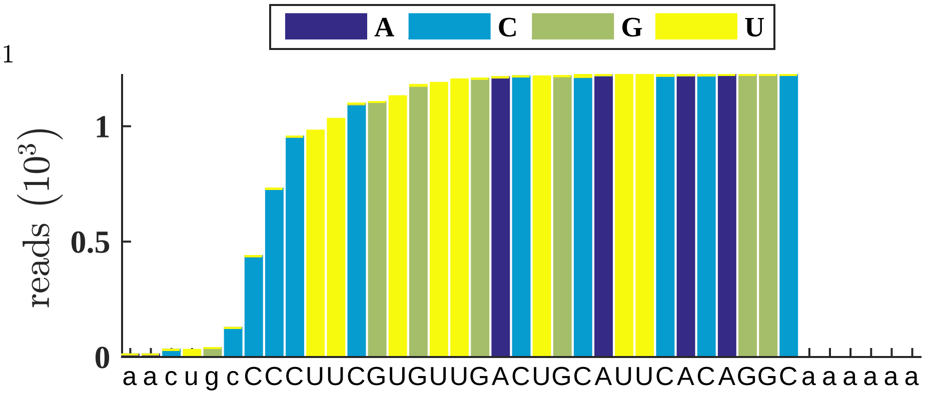

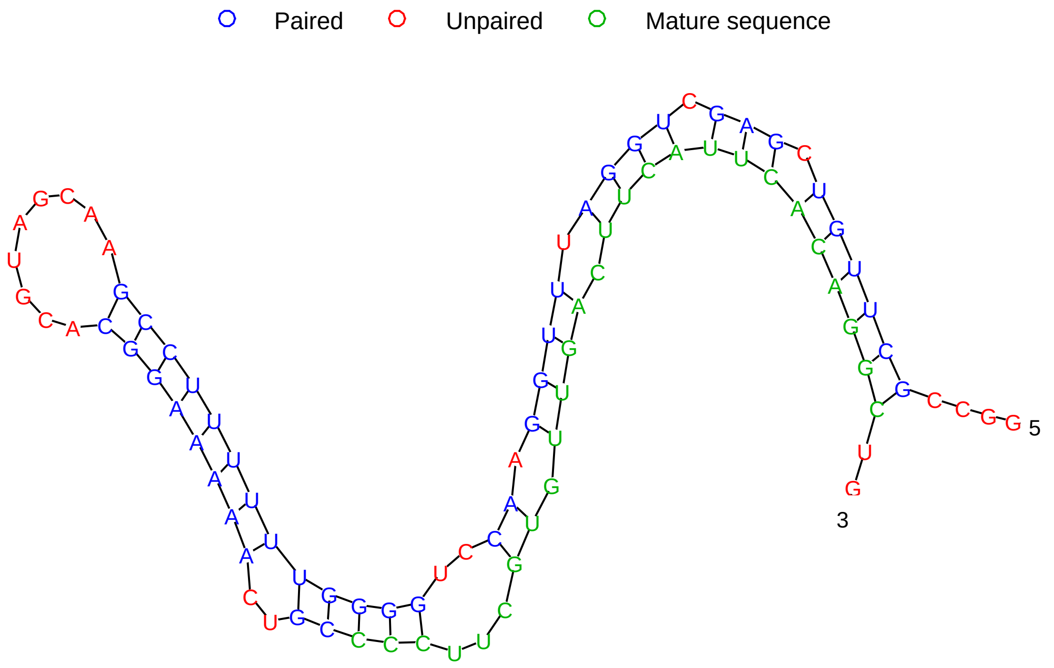

Stem loop (UMD3.1): chr5:103560527-103560618
Mature (UMD3.1): chr5:103560529-103560555
Mature seq len: 27
Total raw counts (9 samples): 845
Average raw counts: 94
Strand: Reverse
Orientation: 3p
Minimum free energy: -28.20

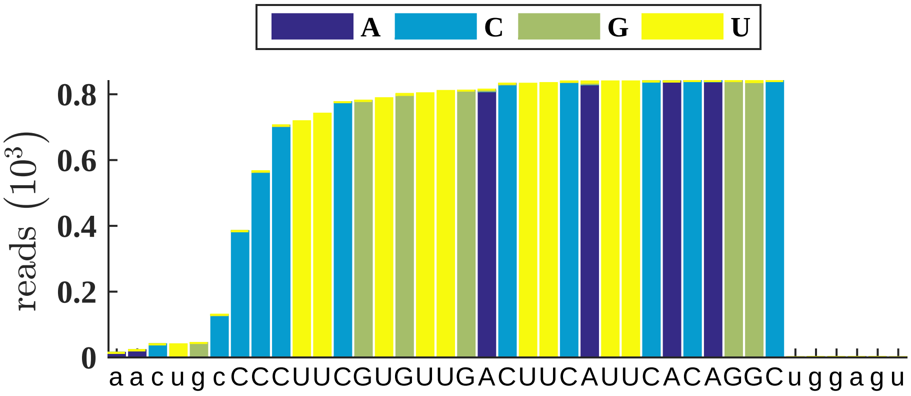

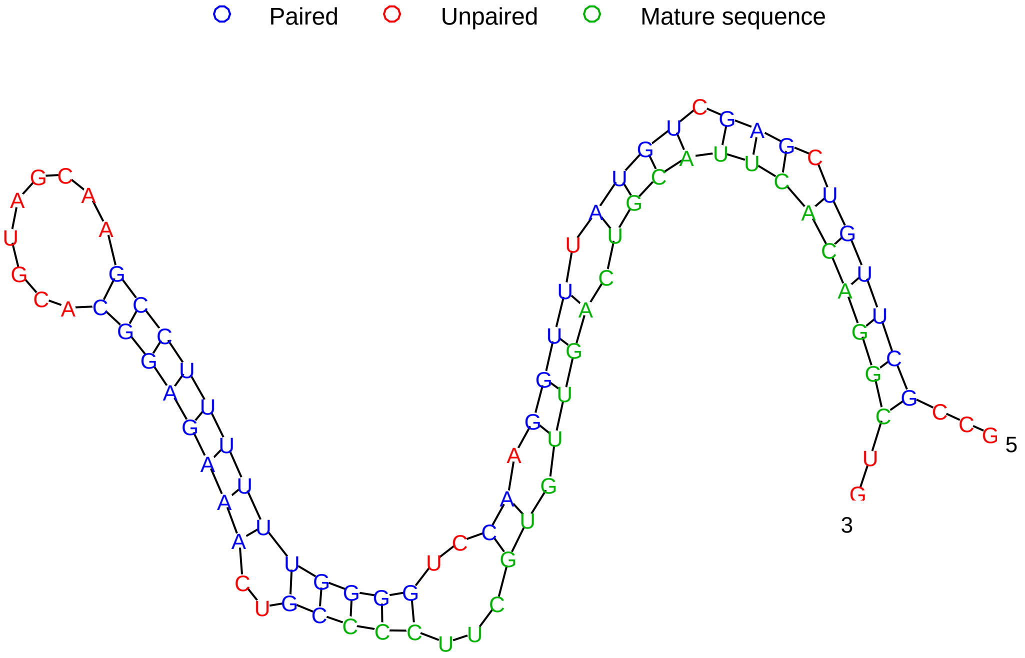

Stem loop (UMD3.1): chr5:115976045-115976135
Mature (UMD3.1): chr5:115976107-115976133
Mature seq len: 27
Total raw counts (9 samples): 1204
Average raw counts: 134
Strand: Forward
Orientation: 3p
Minimum free energy: -29.00

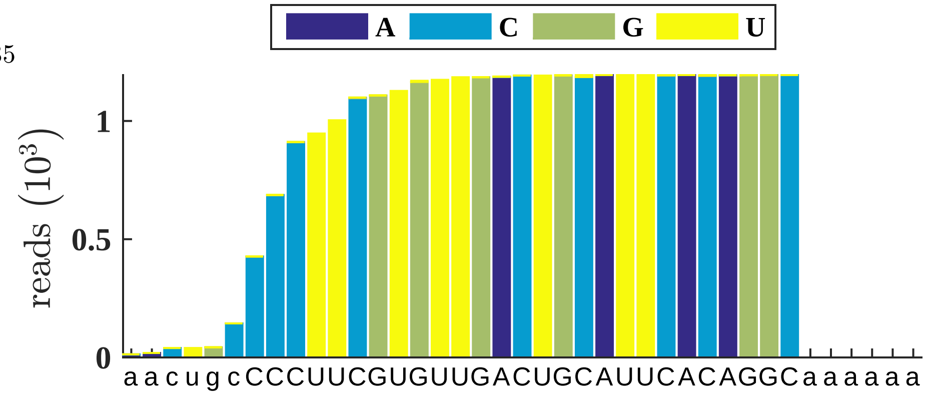

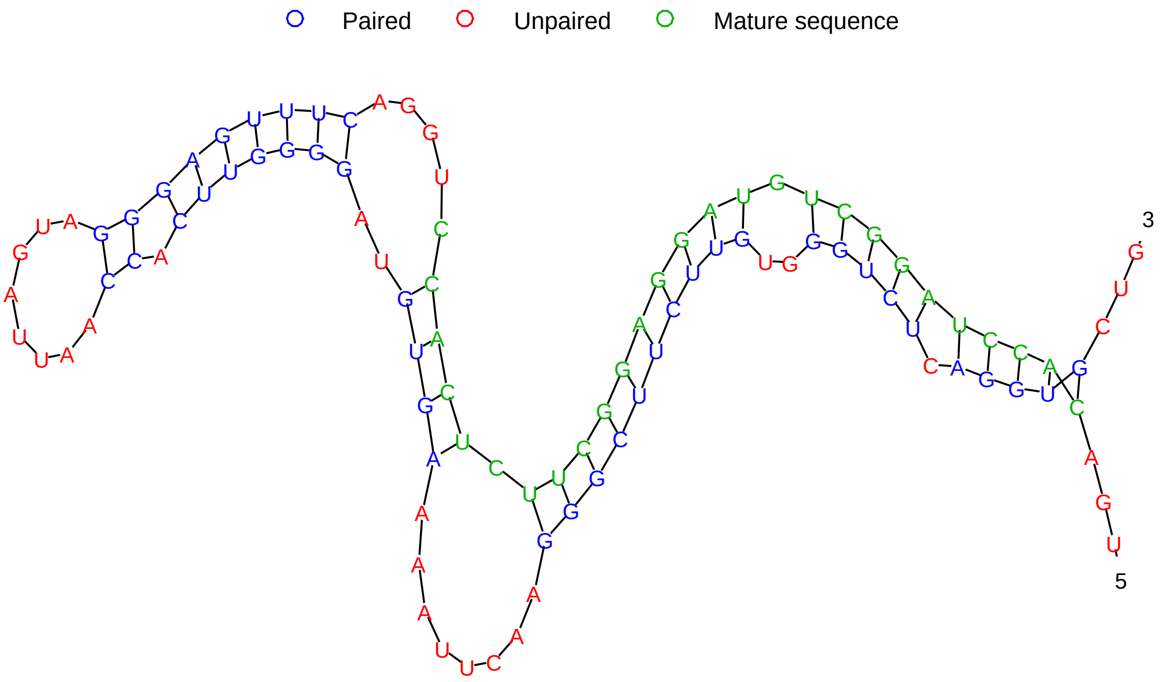

Stem loop (UMD3.1): chr5:37109470-37109570
Mature (UMD3.1): chr5:37109473-37109499
Mature seq len: 27
Total raw counts (9 samples): 1316
Average raw counts: 147
Strand: Forward
Orientation: 5p
Minimum free energy: -29.30

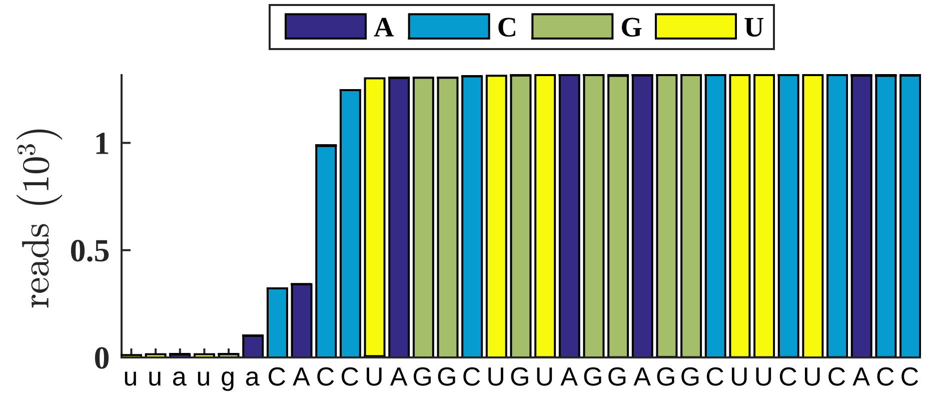

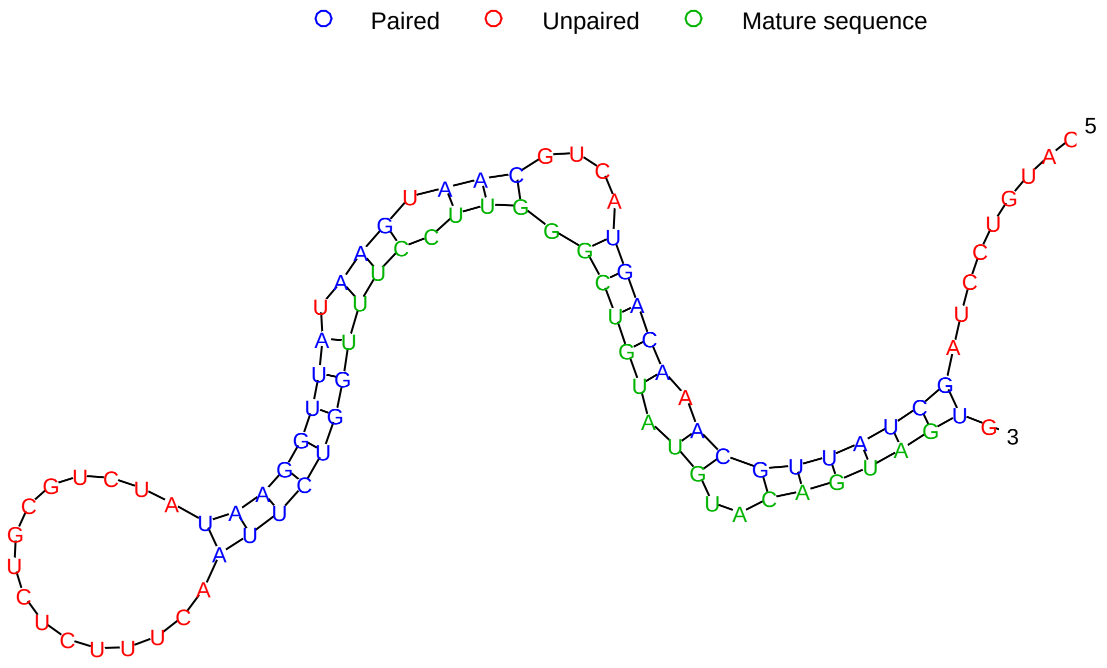

Stem loop (UMD3.1): chr5:38615147-38615240
Mature (UMD3.1): chr5:38615214-38615238
Mature seq len: 25
Total raw counts (9 samples): 1008
Average raw counts: 112
Strand: Forward
Orientation: 3p
Minimum free energy: -15.60

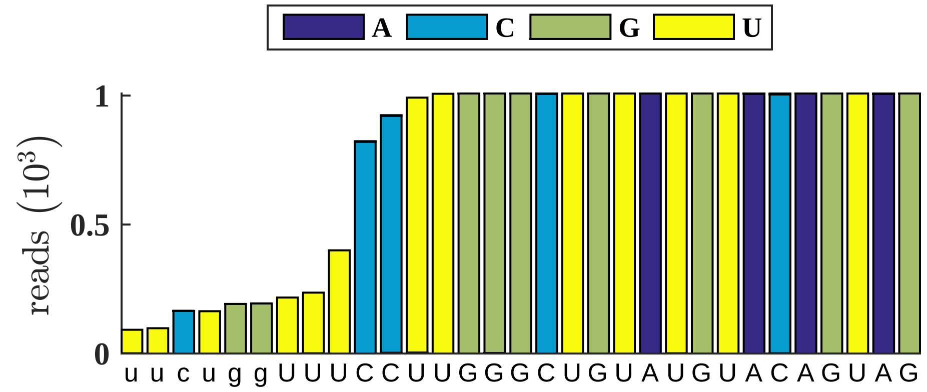

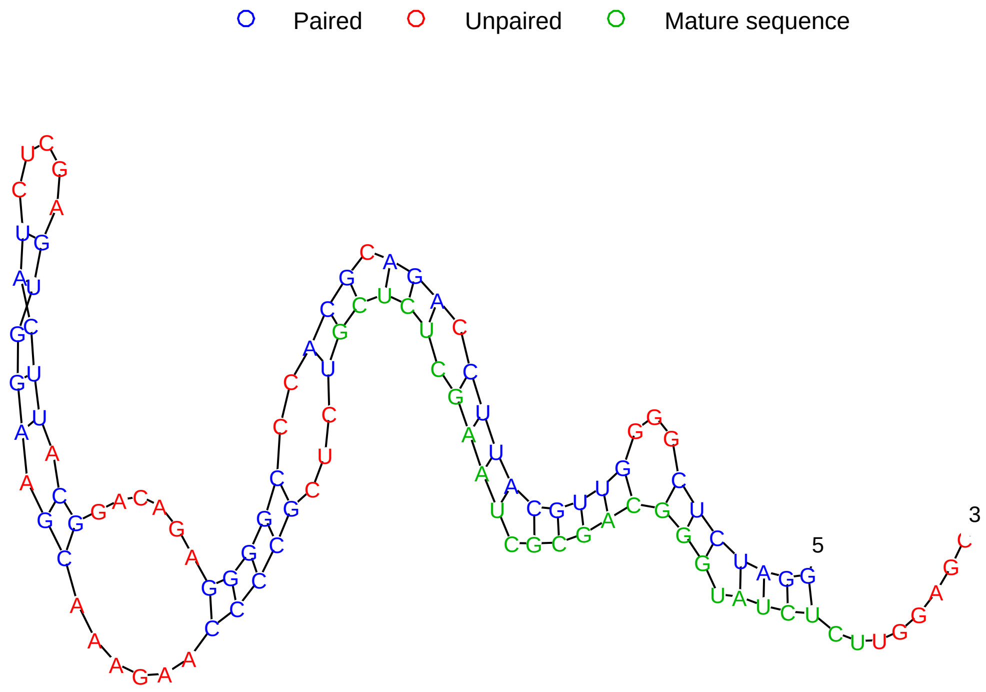

Stem loop (UMD3.1): chr5:53253644-53253751
Mature (UMD3.1): chr5:53253650-53253675
Mature seq len: 26
Total raw counts (9 samples): 3622
Average raw counts: 403
Strand: Reverse
Orientation: 3p
Minimum free energy: -32.40

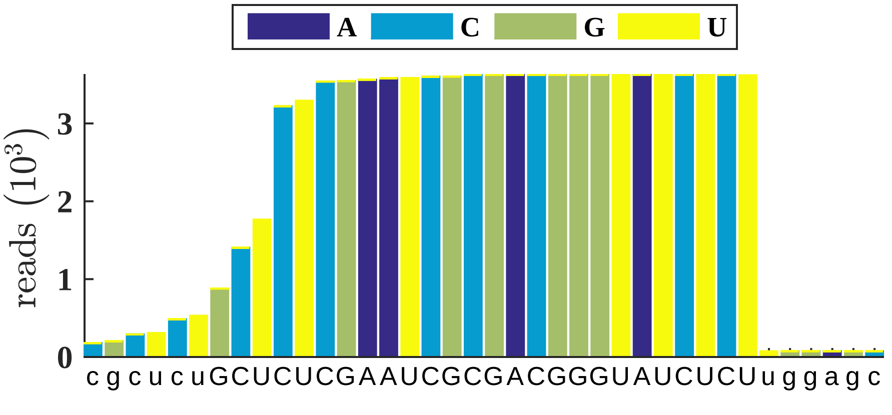

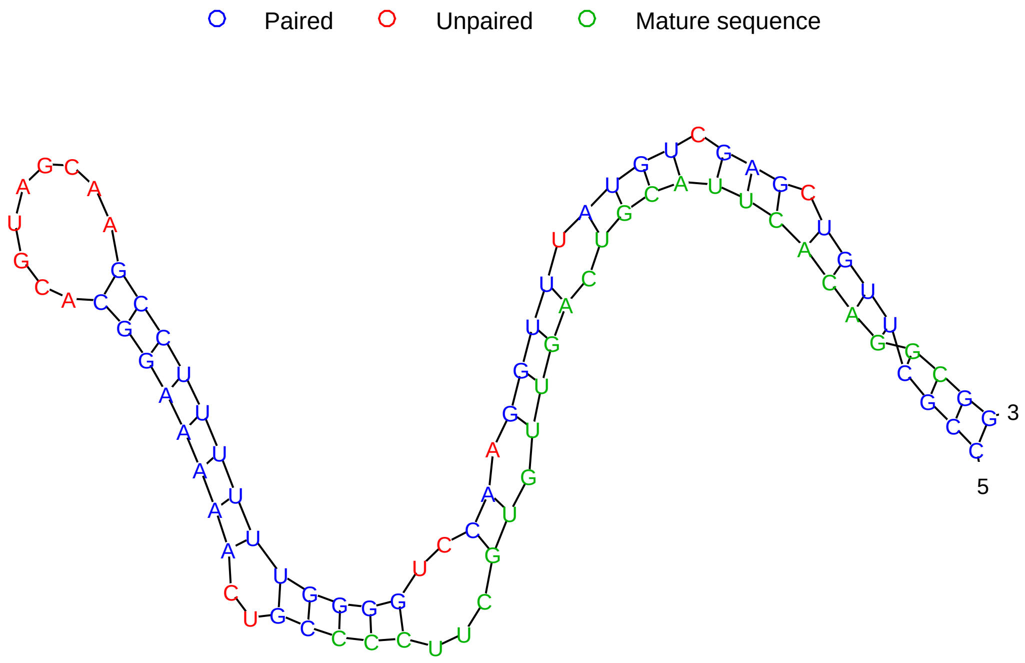

Stem loop (UMD3.1): chr5:9273210-9273299
Mature (UMD3.1): chr5:9273271-9273297
Mature seq len: 27
Total raw counts (9 samples): 1200
Average raw counts: 134
Strand: Forward
Orientation: 3p
Minimum free energy: -33.20

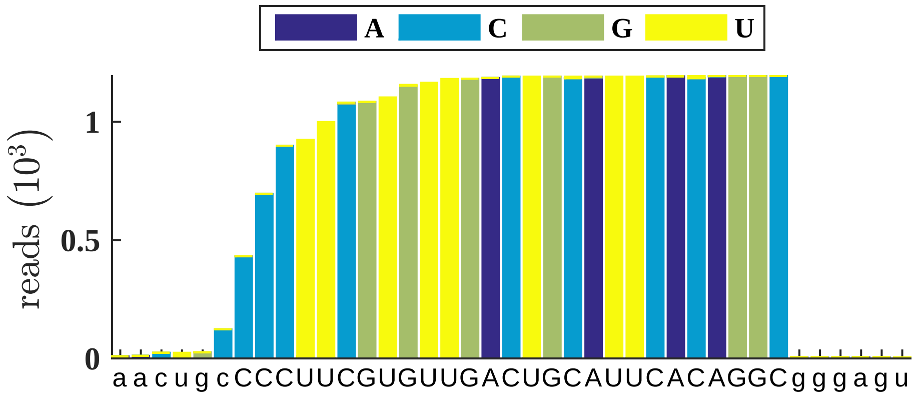

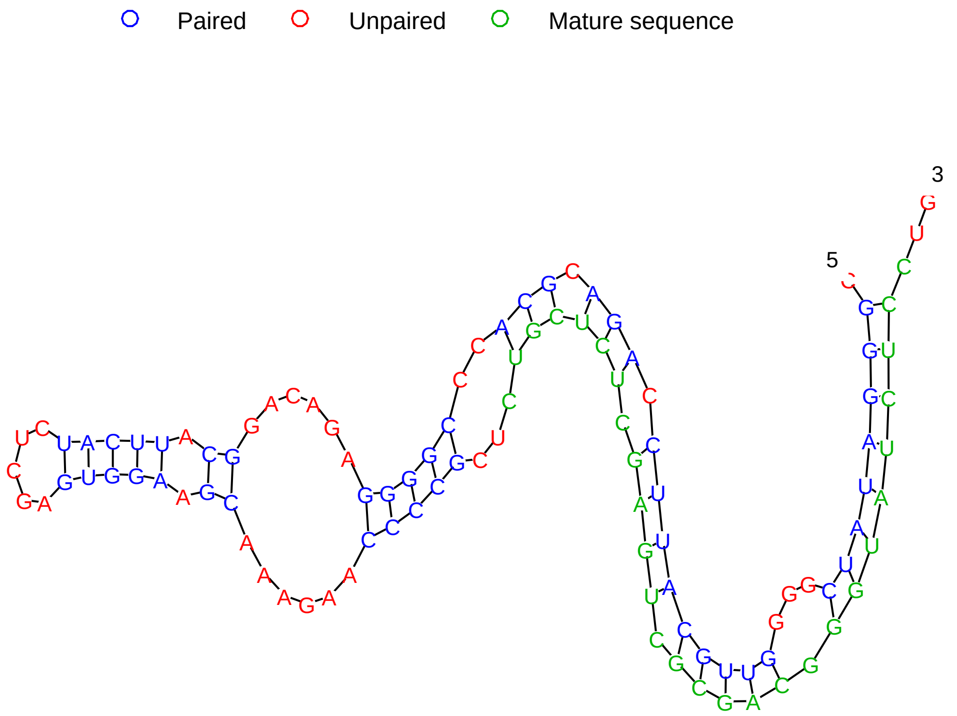

Stem loop (UMD3.1): chr5:97149768-97149873
Mature (UMD3.1): chr5:97149770-97149797
Mature seq len: 28
Total raw counts (9 samples): 1742
Average raw counts: 194
Strand: Reverse
Orientation: 3p
Minimum free energy: -38.30

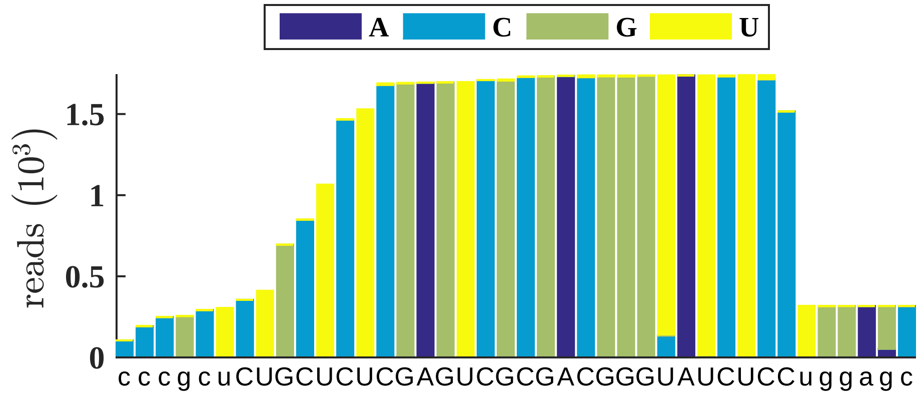

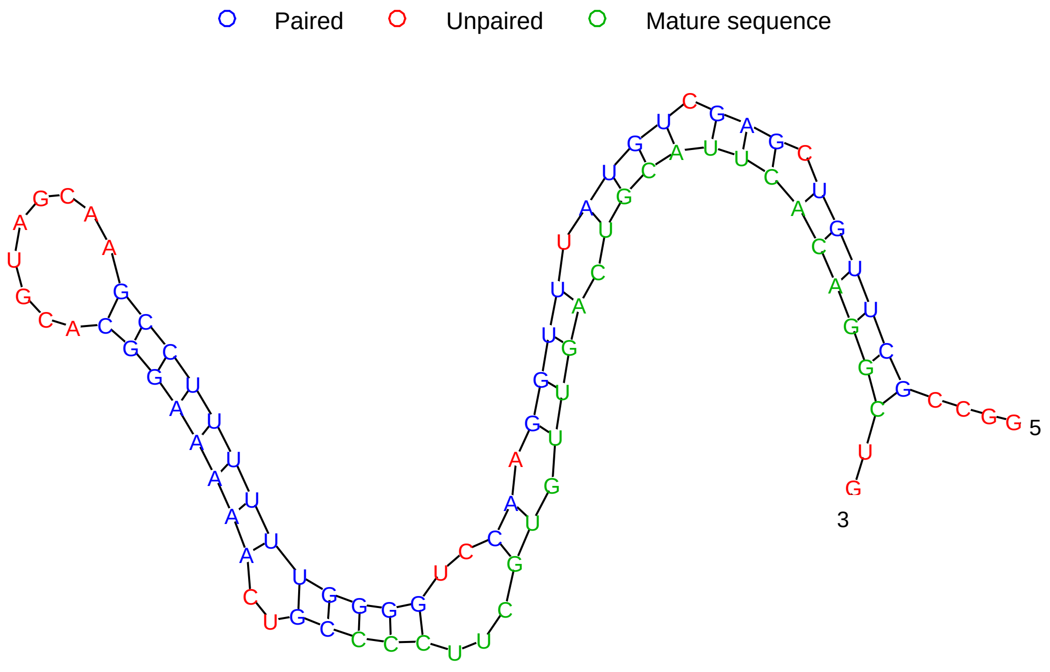

Stem loop (UMD3.1): chr6:10356939-10357030
Mature (UMD3.1): chr6:10356941-10356967
Mature seq len: 27
Total raw counts (9 samples): 1273
Average raw counts: 142
Strand: Reverse
Orientation: 3p
Minimum free energy: -28.90

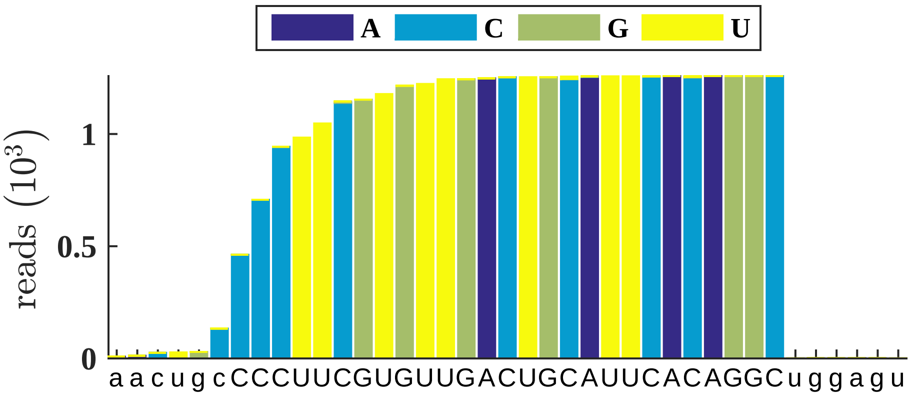

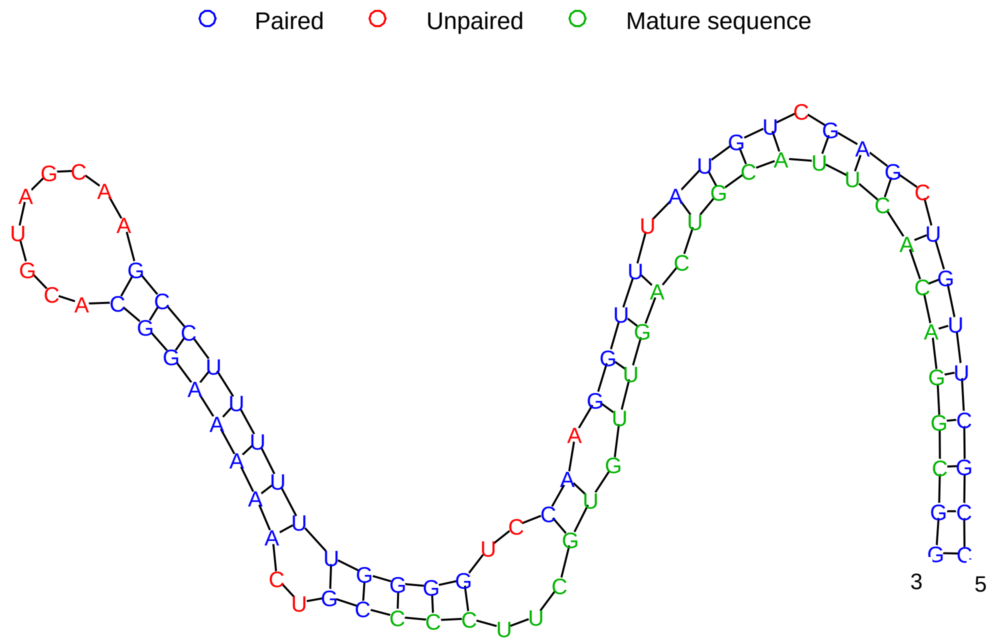

Stem loop (UMD3.1): chr6:112646544-112646633
Mature (UMD3.1): chr6:112646605-112646631
Mature seq len: 27
Total raw counts (9 samples): 1215
Average raw counts: 135
Strand: Forward
Orientation: 3p
Minimum free energy: -33.20

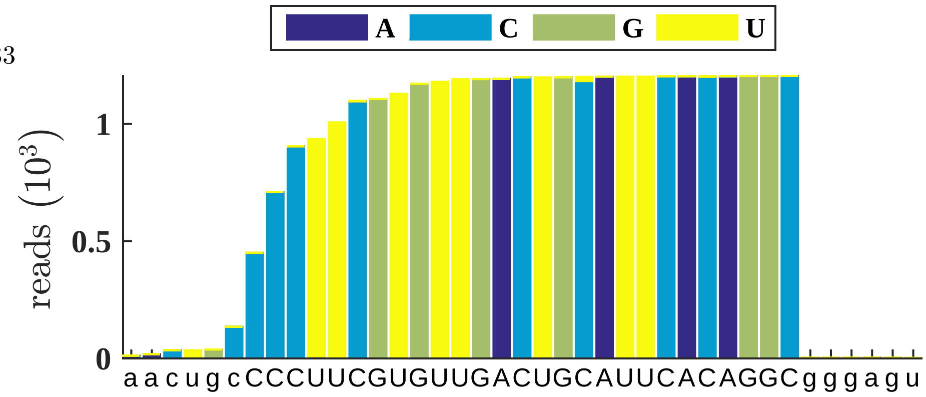

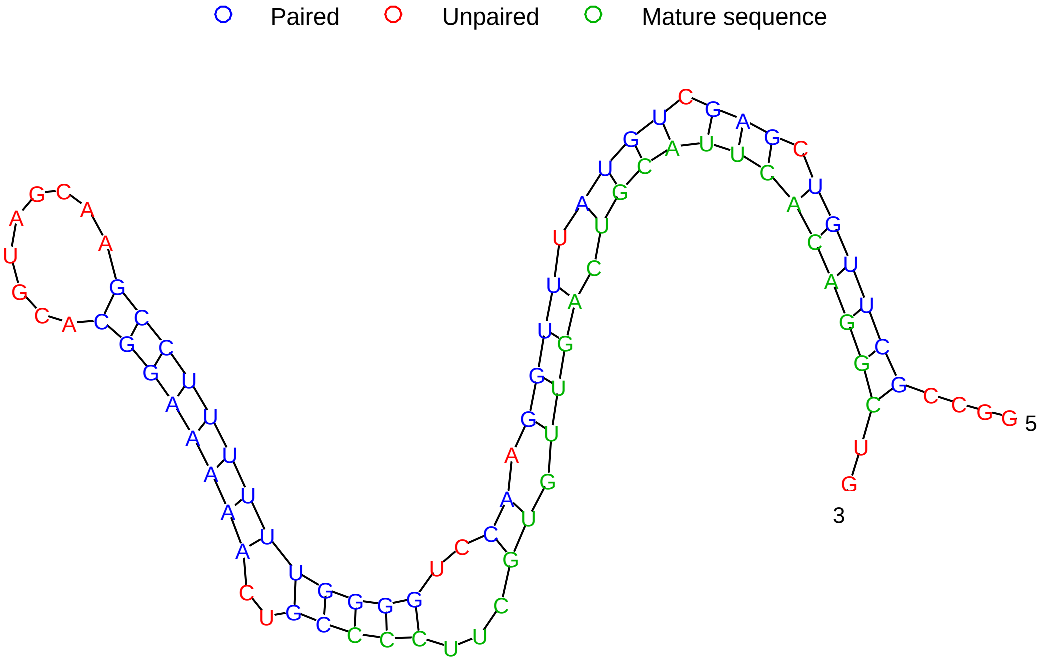

Stem loop (UMD3.1): chr6:17924410-17924501
Mature (UMD3.1): chr6:17924473-17924499
Mature seq len: 27
Total raw counts (9 samples): 1143
Average raw counts: 127
Strand: Forward
Orientation: 3p
Minimum free energy: -28.90

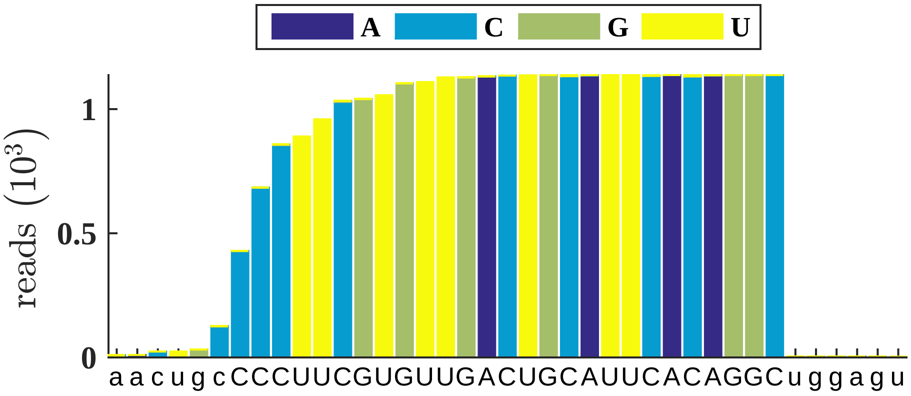

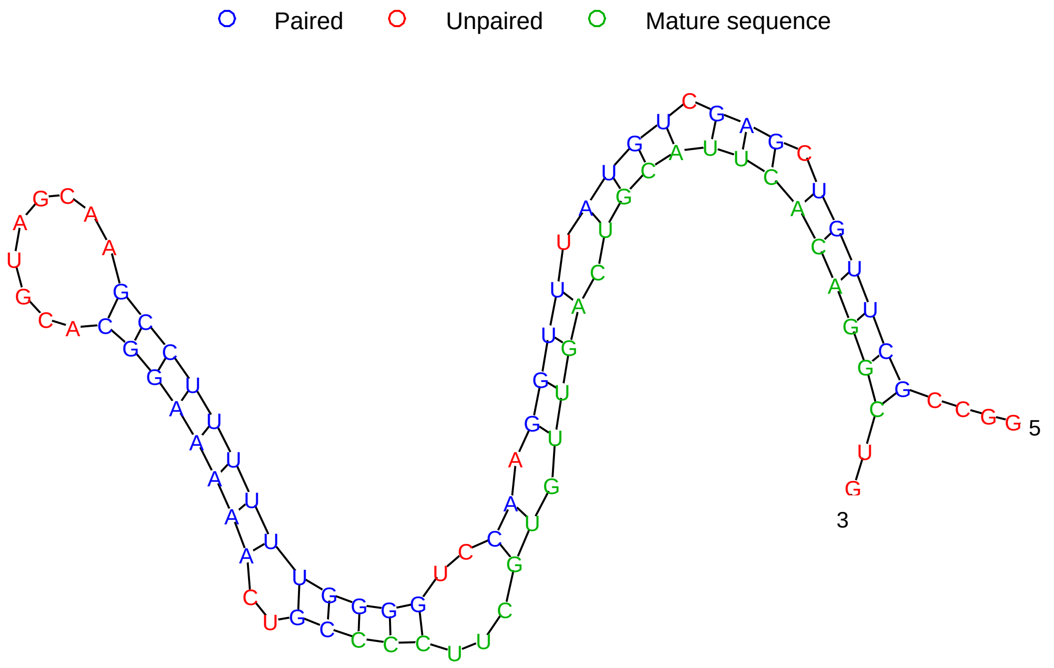

Stem loop (UMD3.1): chr6:17935208-17935299
Mature (UMD3.1): chr6:17935271-17935297
Mature seq len: 27
Total raw counts (9 samples): 1266
Average raw counts: 141
Strand: Forward
Orientation: 3p
Minimum free energy: -28.90

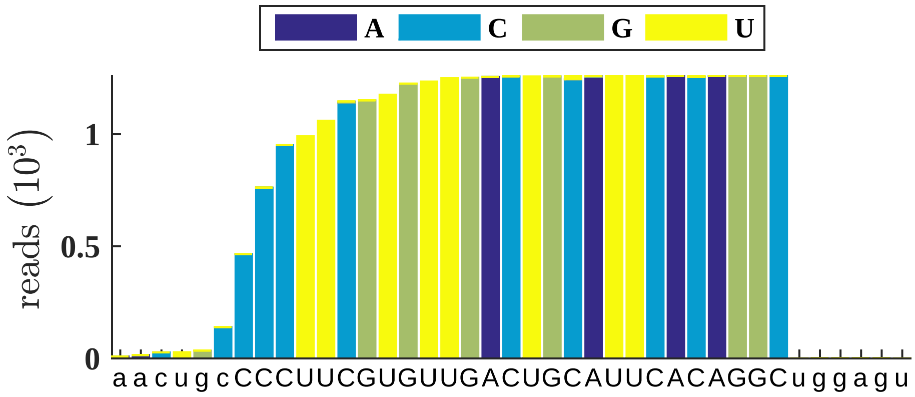

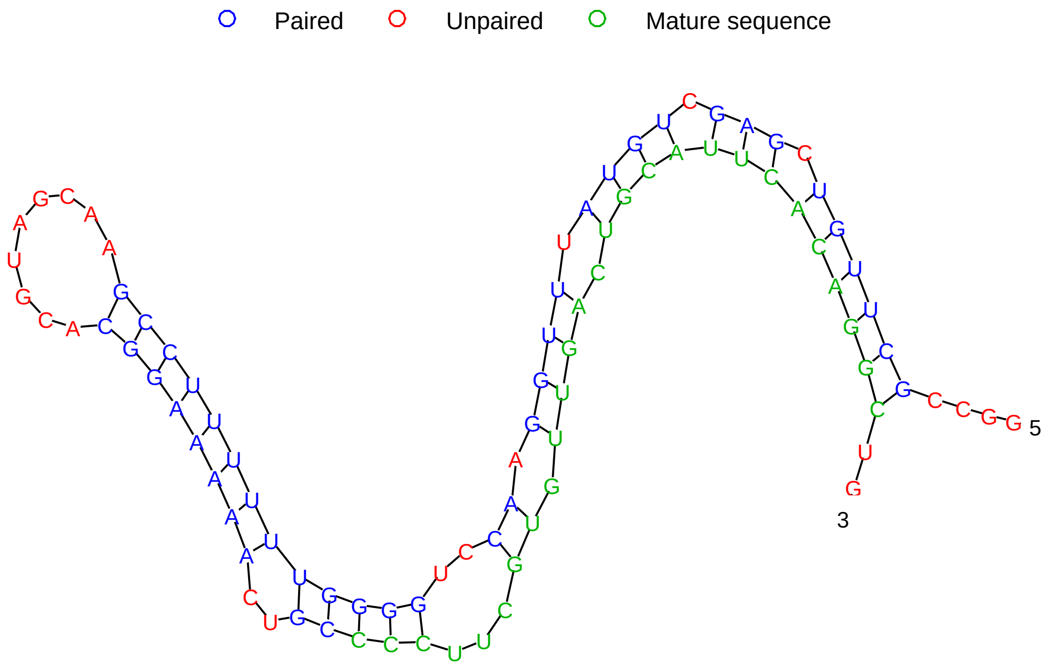

Stem loop (UMD3.1): chr6:17941383-17941474
Mature (UMD3.1): chr6:17941446-17941472
Mature seq len: 27
Total raw counts (9 samples): 1246
Average raw counts: 139
Strand: Forward
Orientation: 3p
Minimum free energy: -28.90

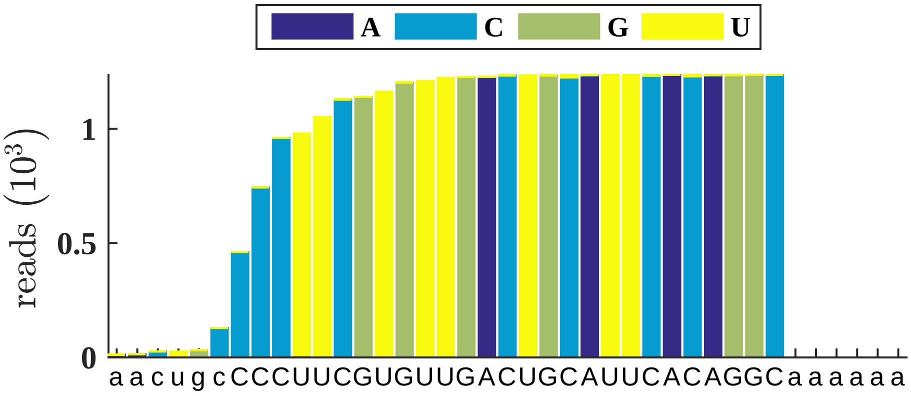

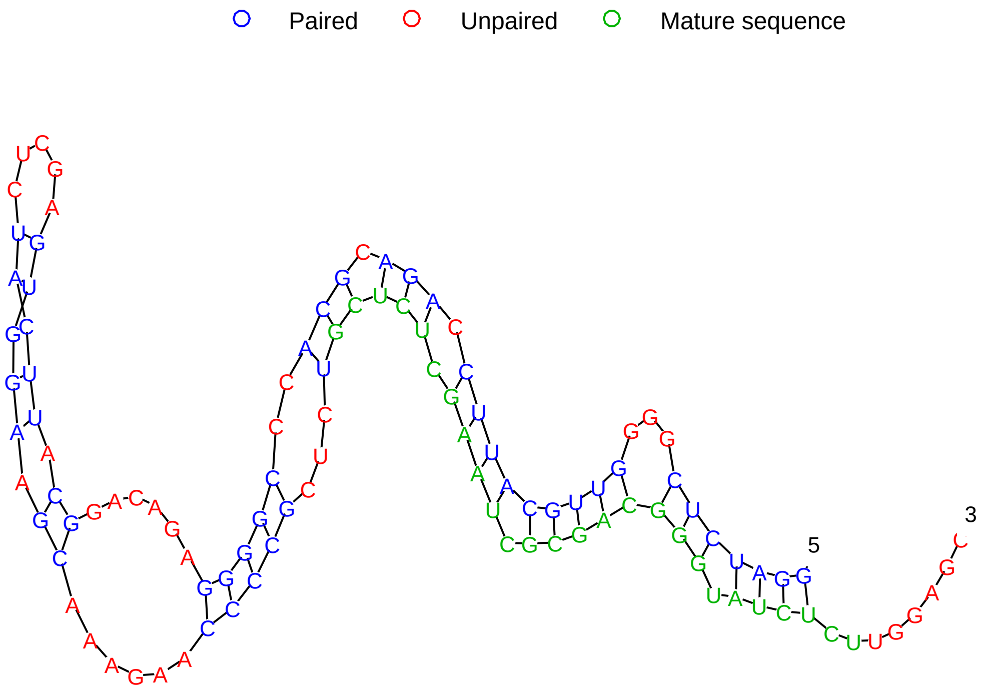

Stem loop (UMD3.1): chr6:17951016-17951123
Mature (UMD3.1): chr6:17951092-17951117
Mature seq len: 26
Total raw counts (9 samples): 4133
Average raw counts: 460
Strand: Forward
Orientation: 3p
Minimum free energy: -32.40

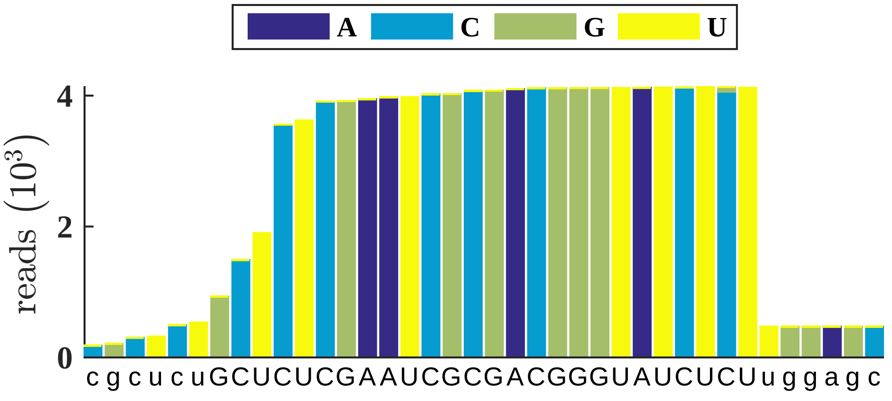

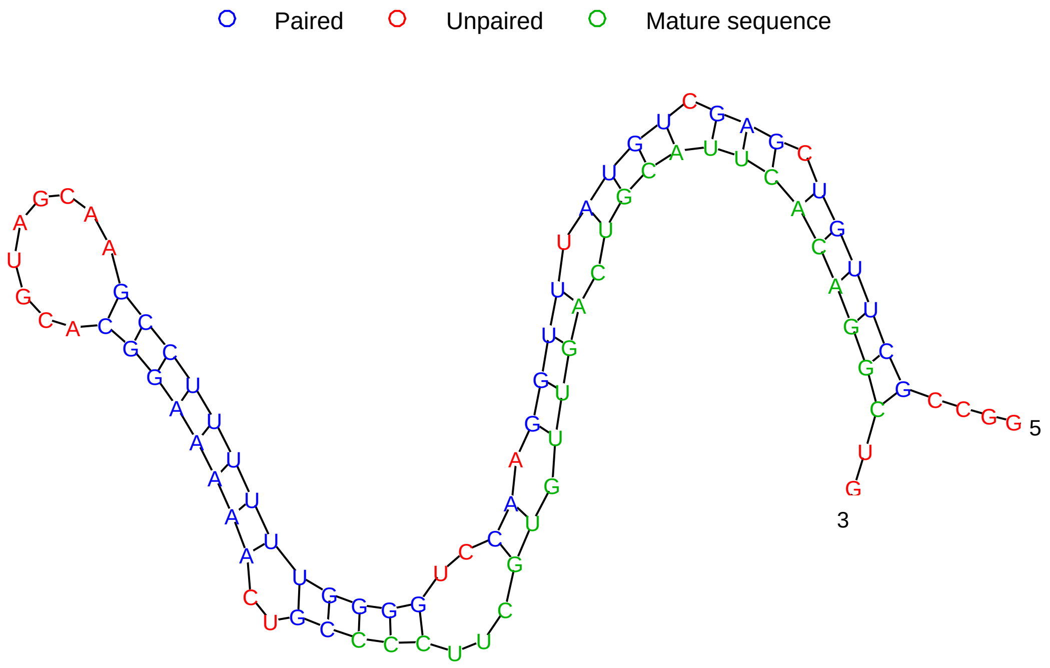

Stem loop (UMD3.1): chr6:17951735-17951826
Mature (UMD3.1): chr6:17951798-17951824
Mature seq len: 27
Total raw counts (9 samples): 1224
Average raw counts: 136
Strand: Forward
Orientation: 3p
Minimum free energy: -28.90

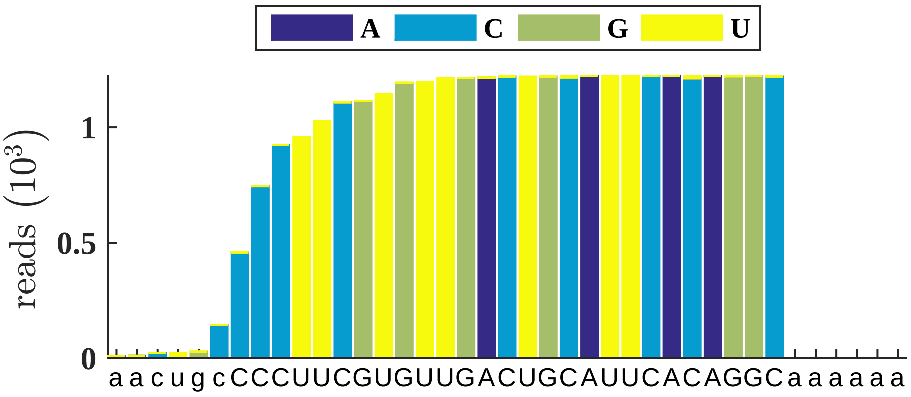

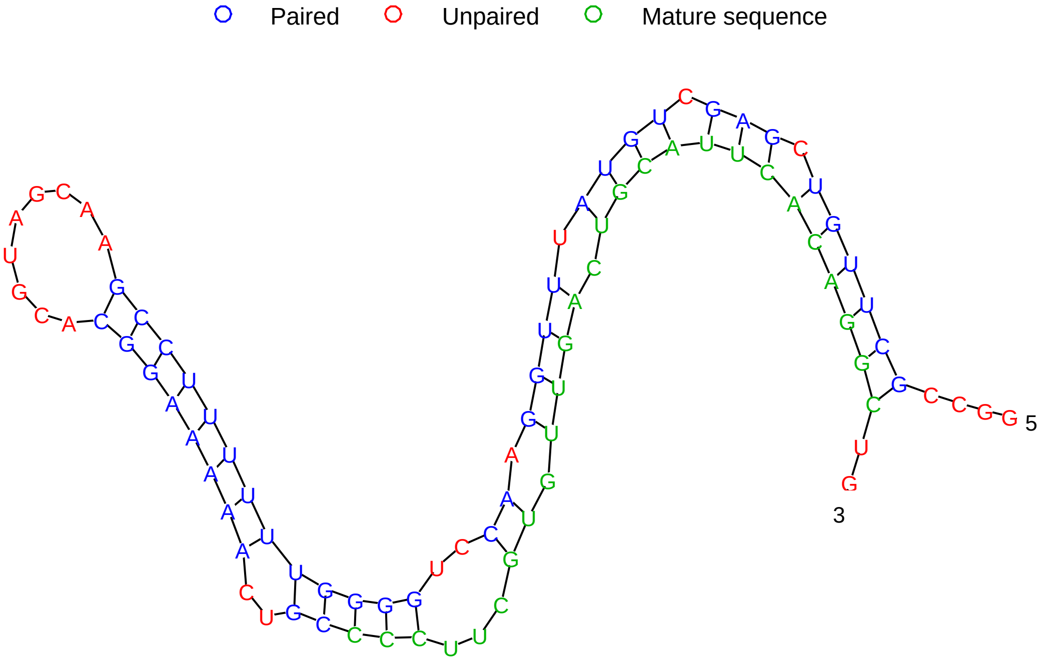

Stem loop (UMD3.1): chr6:17958274-17958365
Mature (UMD3.1): chr6:17958337-17958363
Mature seq len: 27
Total raw counts (9 samples): 1195
Average raw counts: 133
Strand: Forward
Orientation: 3p
Minimum free energy: -28.90

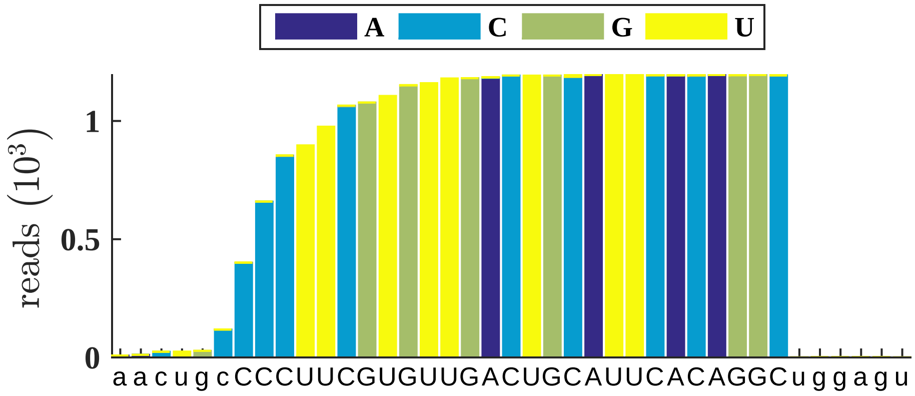

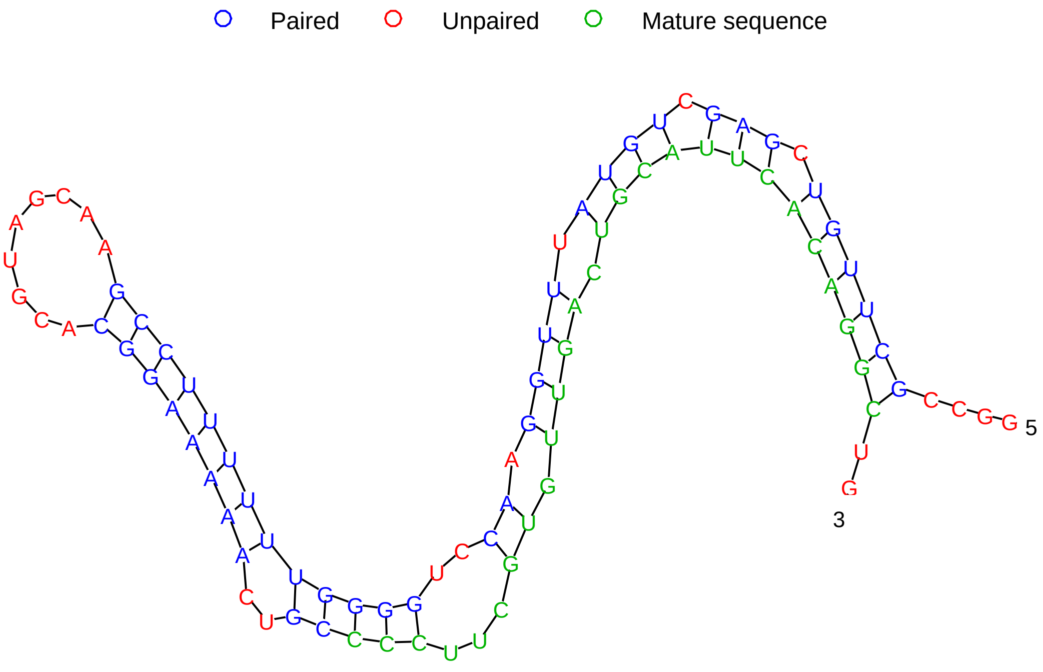

Stem loop (UMD3.1): chr6:17962921-17963012
Mature (UMD3.1): chr6:17962984-17963010
Mature seq len: 27
Total raw counts (9 samples): 1163
Average raw counts: 130
Strand: Forward
Orientation: 3p
Minimum free energy: -28.90

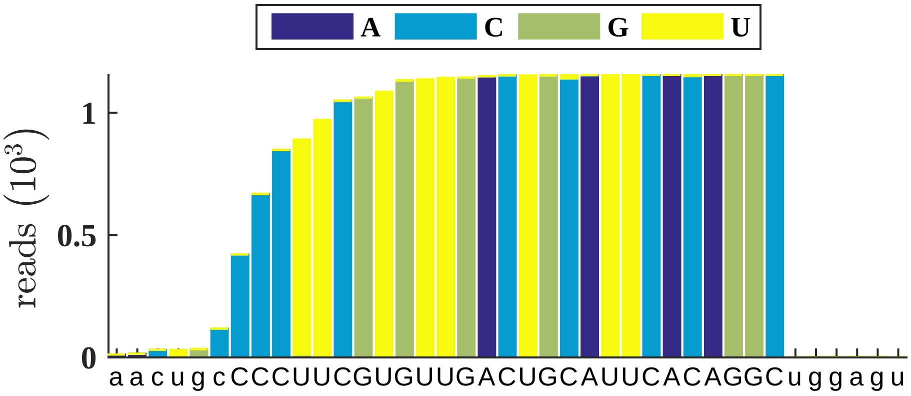

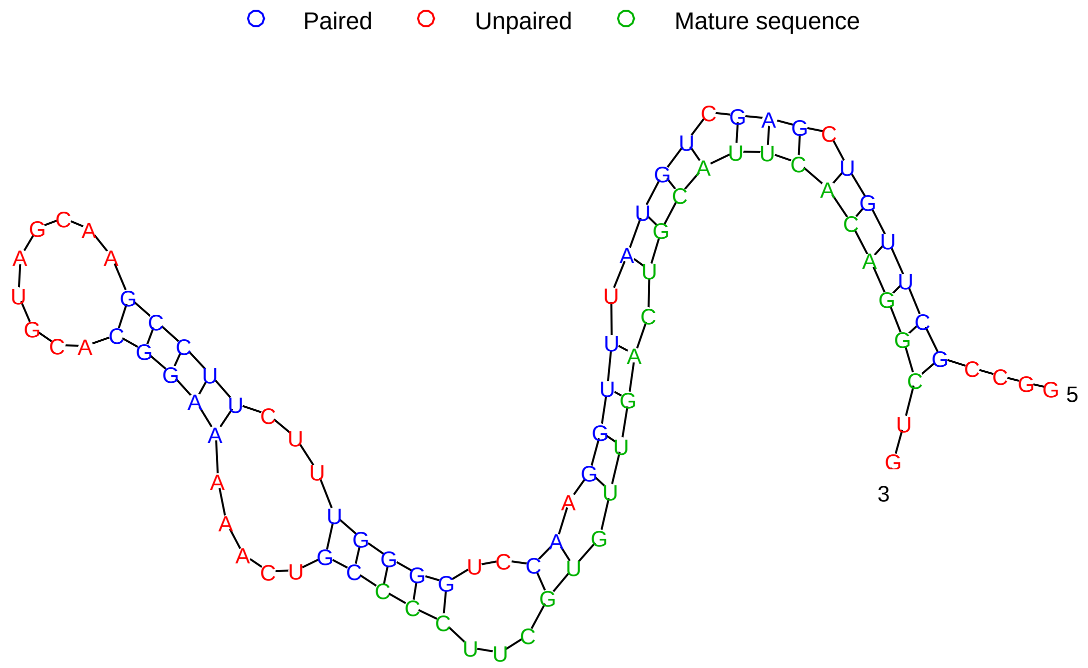

Stem loop (UMD3.1): chr6:17966044-17966135
Mature (UMD3.1): chr6:17966107-17966133
Mature seq len: 27
Total raw counts (9 samples): 1156
Average raw counts: 129
Strand: Forward
Orientation: 3p
Minimum free energy: -25.70

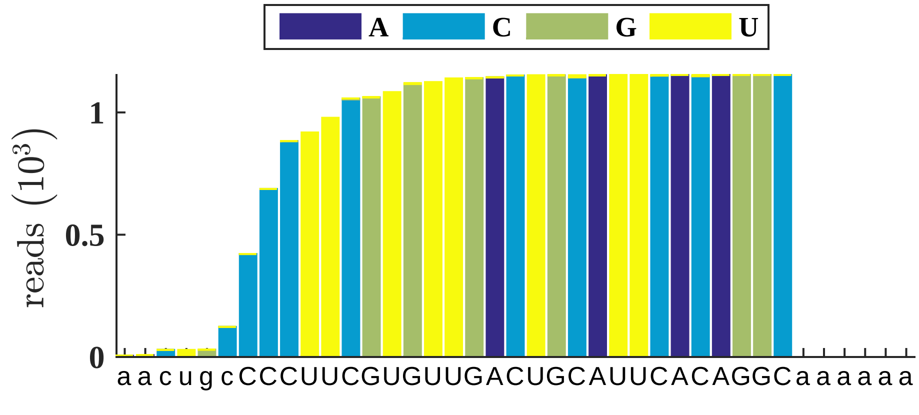

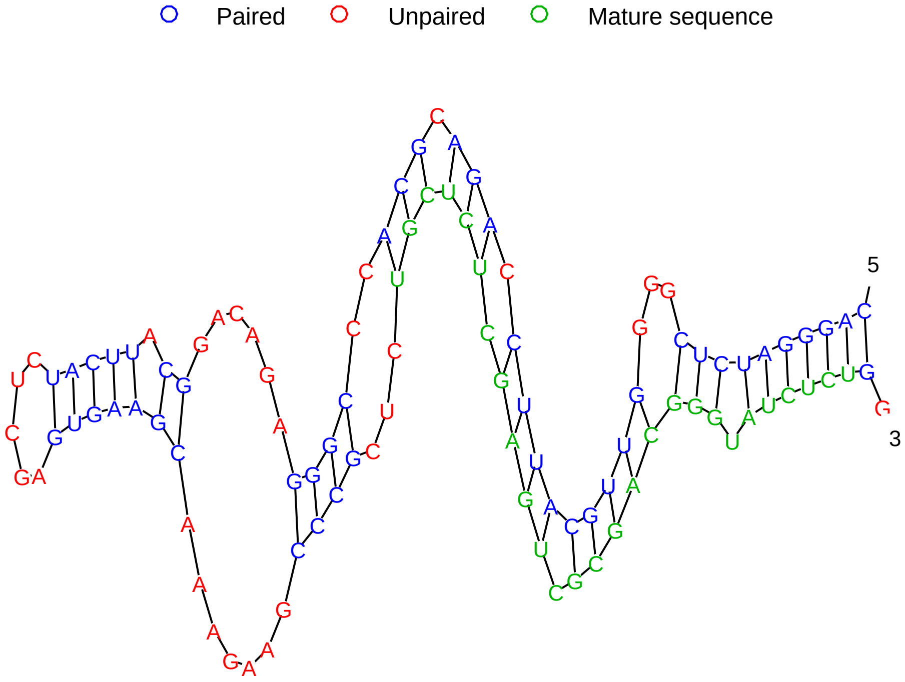

Stem loop (UMD3.1): chr6:17966770-17966874
Mature (UMD3.1): chr6:17966846-17966872
Mature seq len: 27
Total raw counts (9 samples): 11677
Average raw counts: 1298
Strand: Forward
Orientation: 3p
Minimum free energy: -36.60

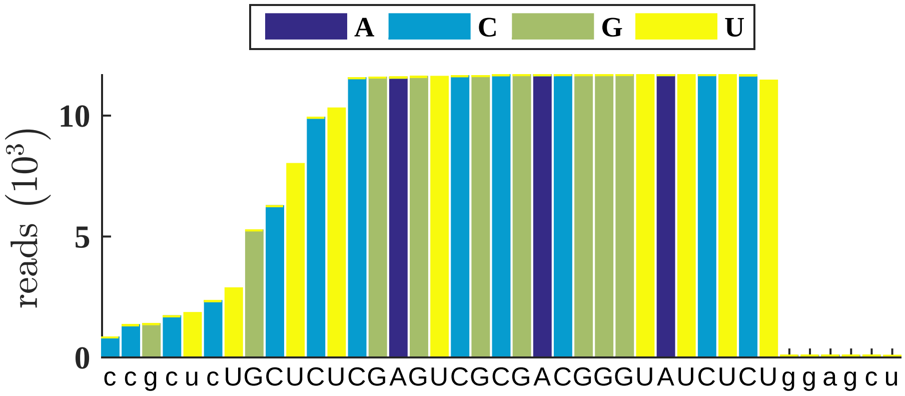

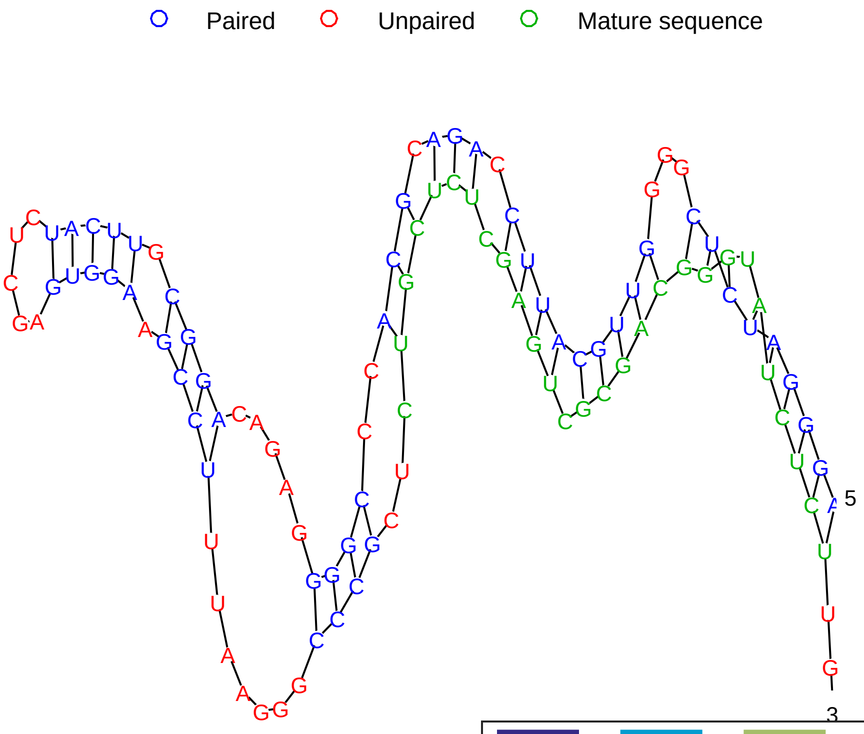

Stem loop (UMD3.1): chr6:17973699-17973806
Mature (UMD3.1): chr6:17973777-17973804
Mature seq len: 28
Total raw counts (9 samples): 11623
Average raw counts: 1292
Strand: Forward
Orientation: 3p
Minimum free energy: -36.40

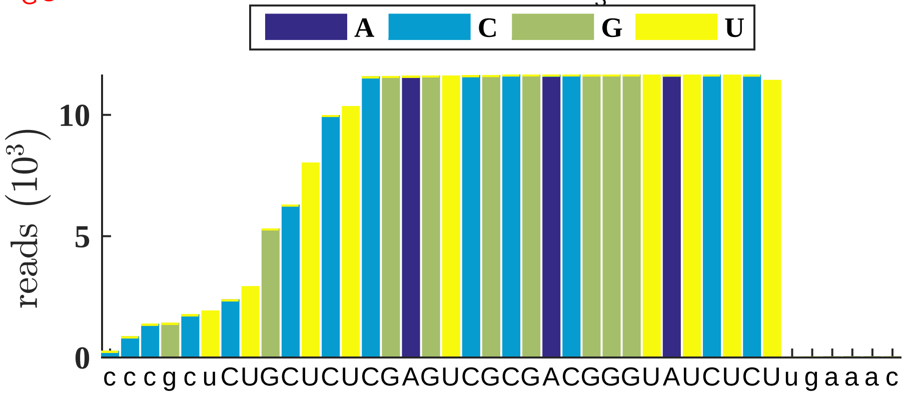

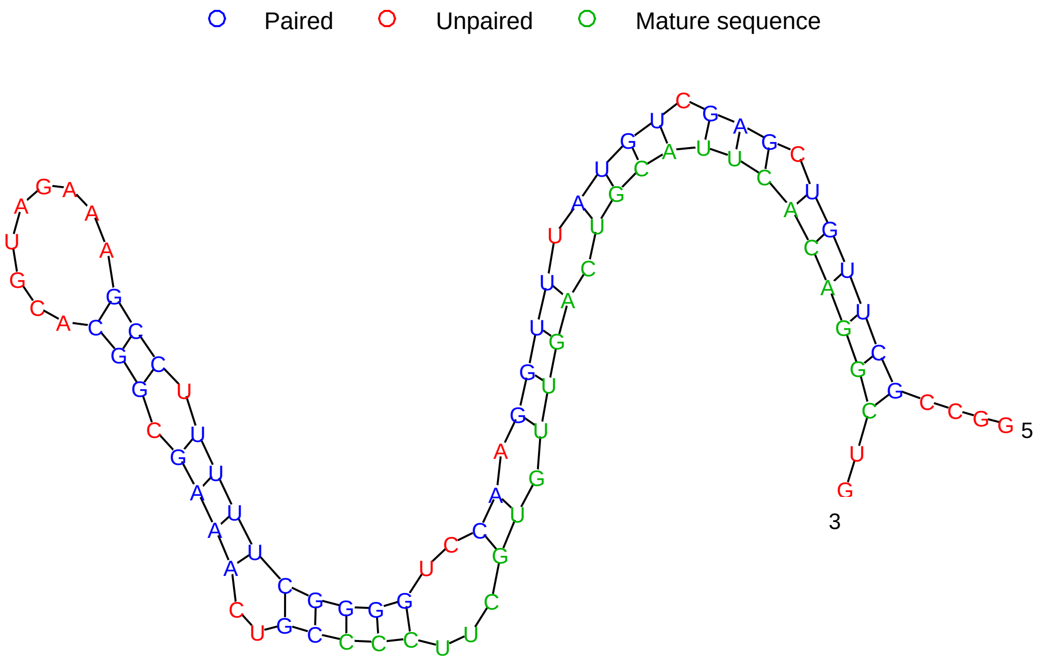

Stem loop (UMD3.1): chr6:17976465-17976556
Mature (UMD3.1): chr6:17976528-17976554
Mature seq len: 27
Total raw counts (9 samples): 1177
Average raw counts: 131
Strand: Forward
Orientation: 3p
Minimum free energy: -26.20

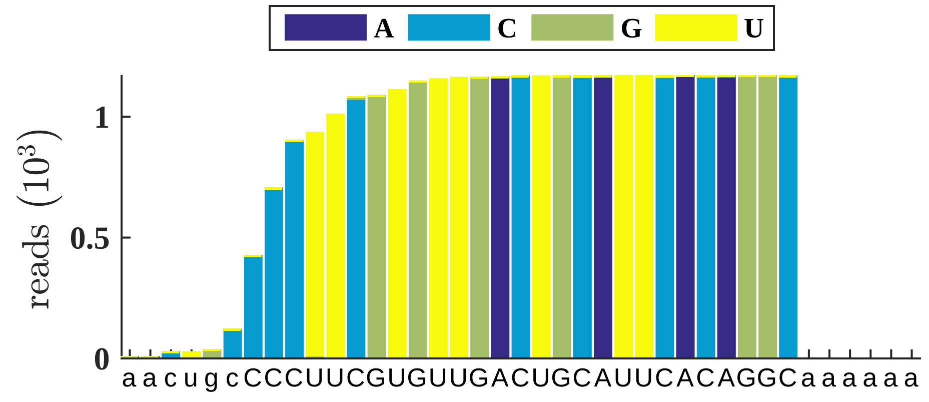

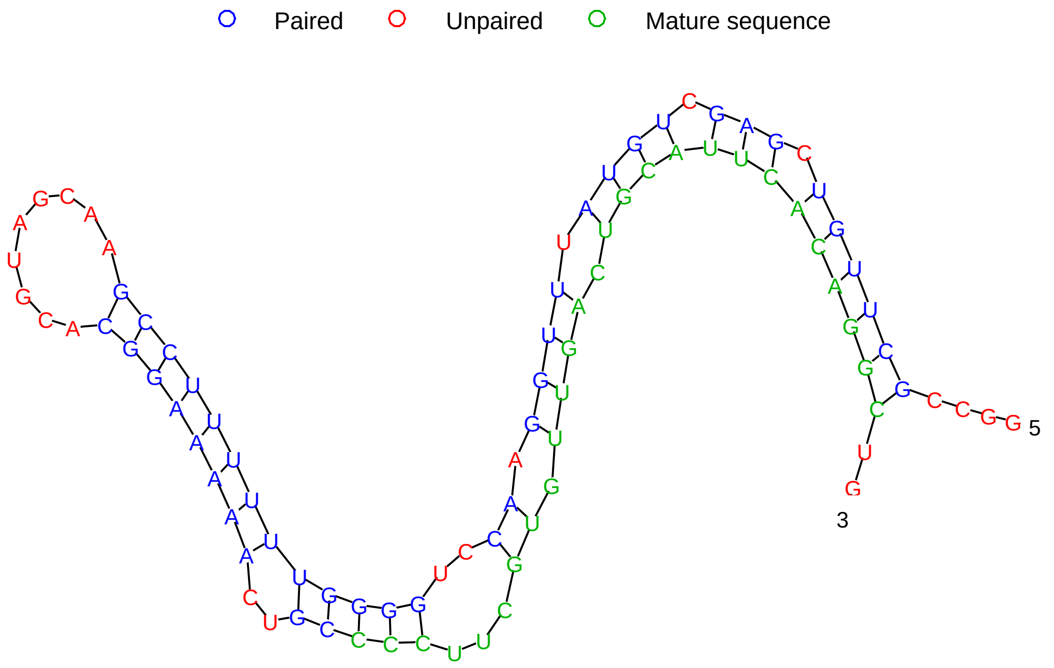

Stem loop (UMD3.1): chr6:17979541-17979632
Mature (UMD3.1): chr6:17979604-17979630
Mature seq len: 27
Total raw counts (9 samples): 1206
Average raw counts: 134
Strand: Forward
Orientation: 3p
Minimum free energy: -28.90

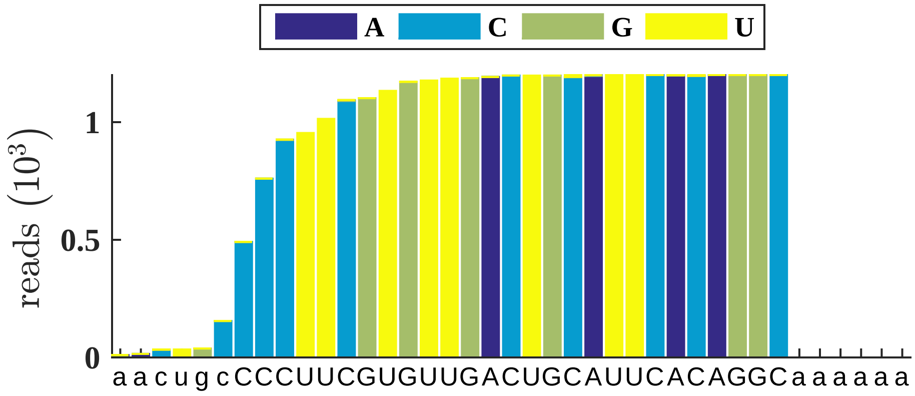

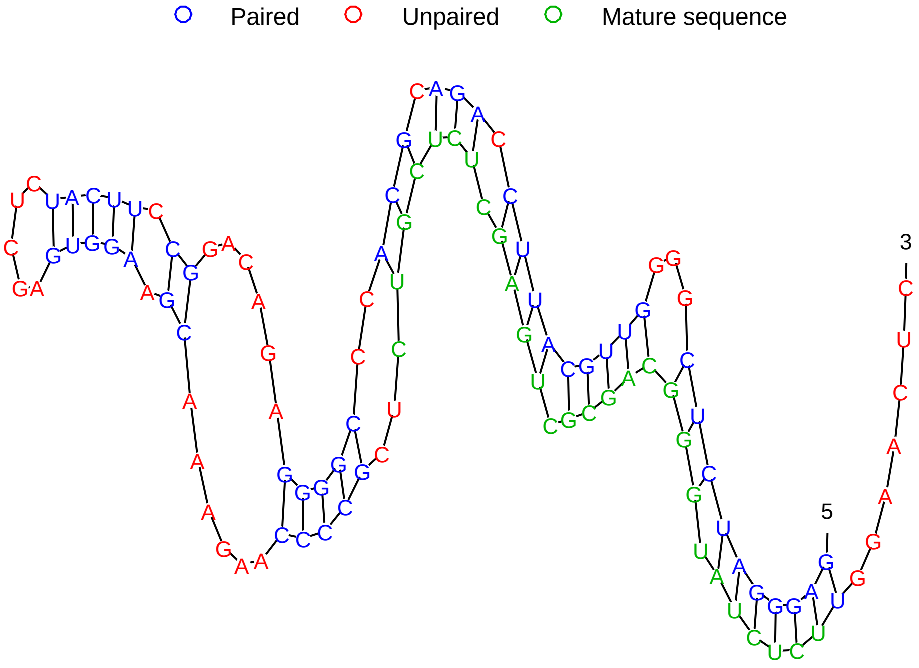

Stem loop (UMD3.1): chr6:17983214-17983326
Mature (UMD3.1): chr6:17983291-17983318
Mature seq len: 28
Total raw counts (9 samples): 14366
Average raw counts: 1597
Strand: Forward
Orientation: 3p
Minimum free energy: -37.90

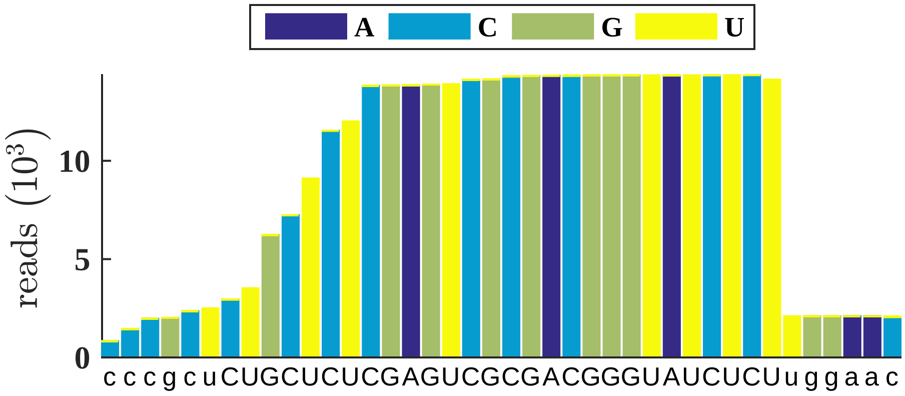

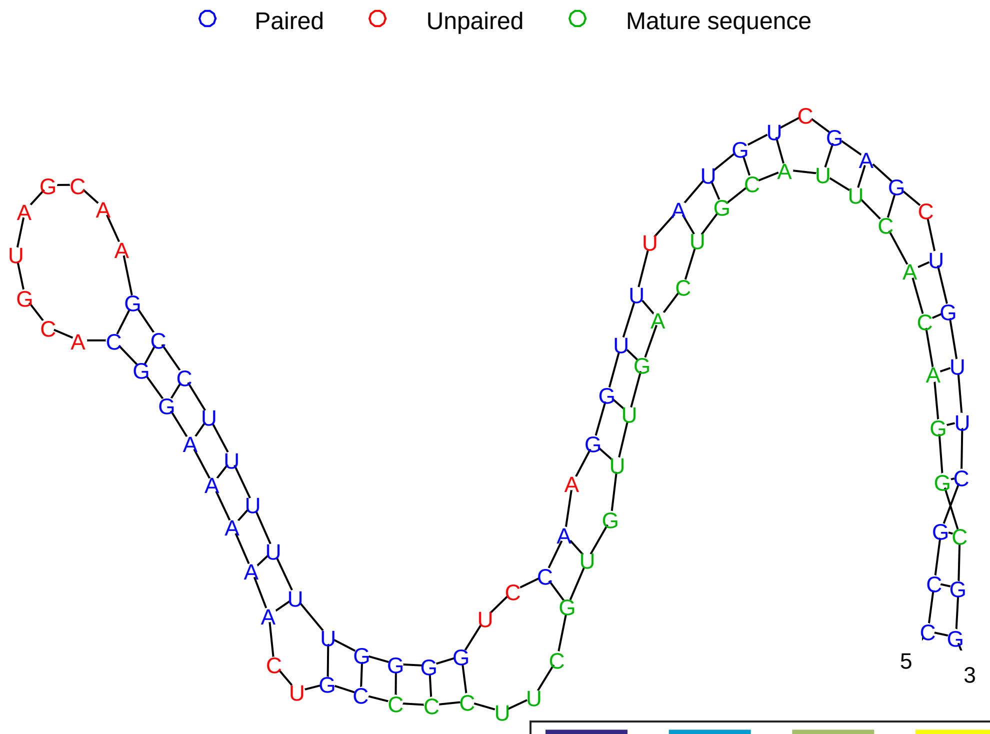

Stem loop (UMD3.1): chr6:17985488-17985577
Mature (UMD3.1): chr6:17985549-17985575
Mature seq len: 27
Total raw counts (9 samples): 1150
Average raw counts: 128
Strand: Forward
Orientation: 3p
Minimum free energy: -33.20

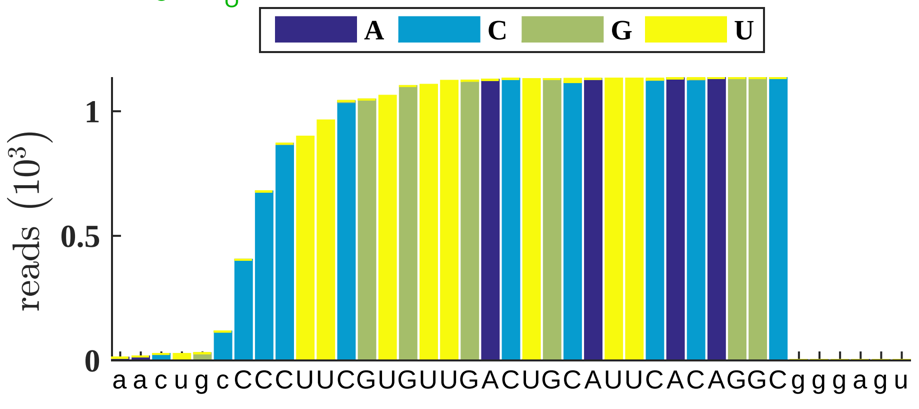

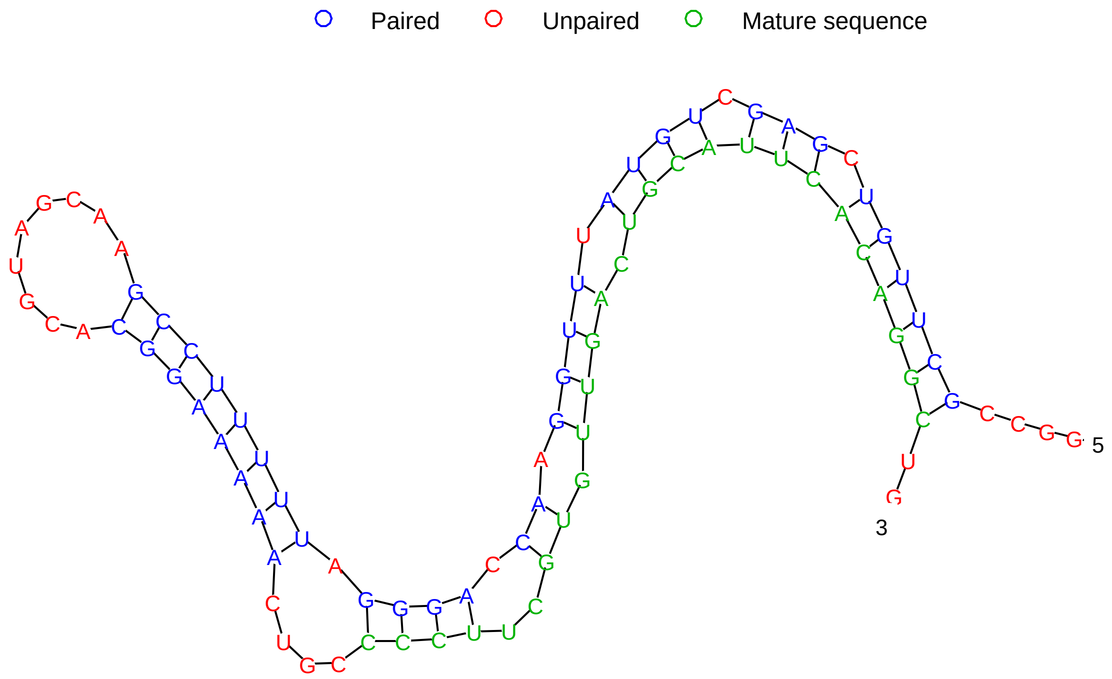

Stem loop (UMD3.1): chr6:17986726-17986816
Mature (UMD3.1): chr6:17986788-17986814
Mature seq len: 27
Total raw counts (9 samples): 1246
Average raw counts: 139
Strand: Forward
Orientation: 3p
Minimum free energy: -23.80

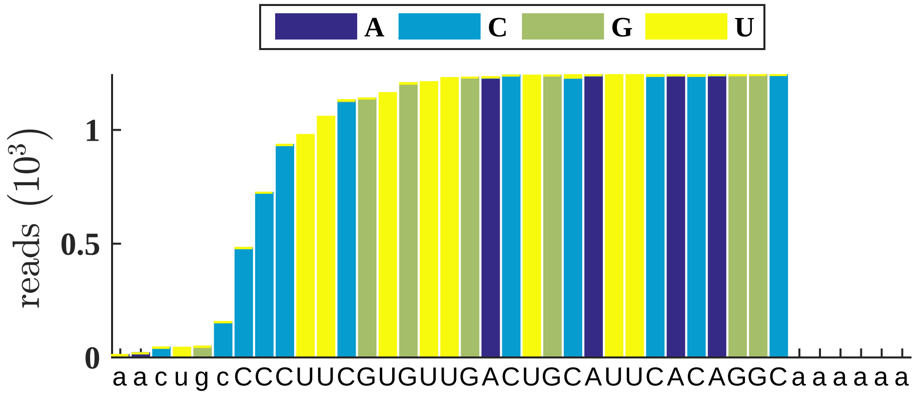

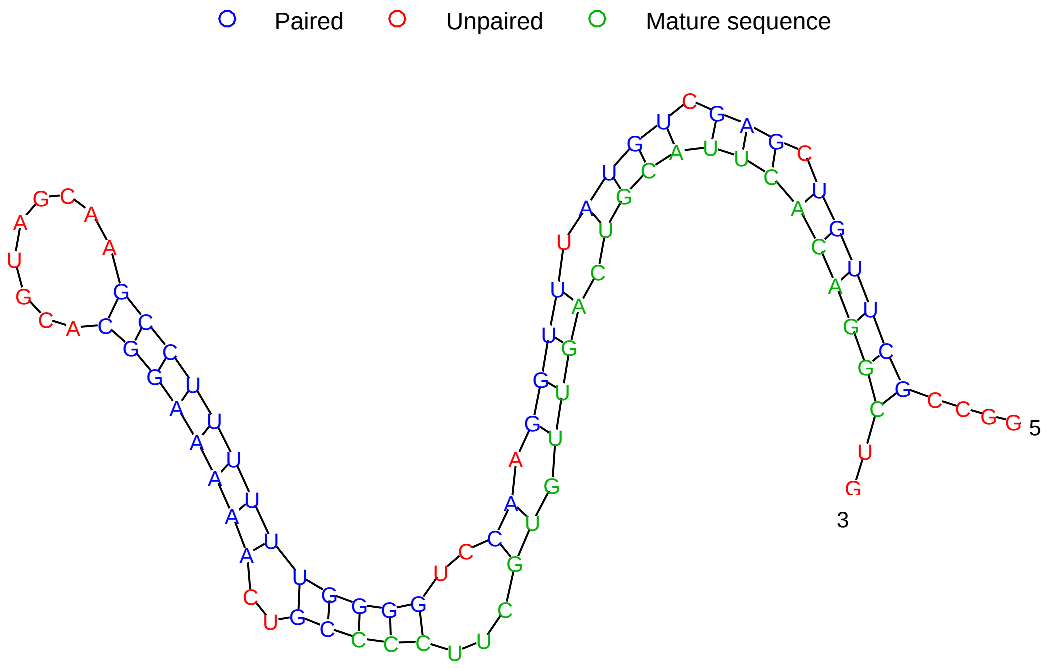

Stem loop (UMD3.1): chr6:17988099-17988190
Mature (UMD3.1): chr6:17988162-17988188
Mature seq len: 27
Total raw counts (9 samples): 1215
Average raw counts: 135
Strand: Forward
Orientation: 3p
Minimum free energy: -28.90

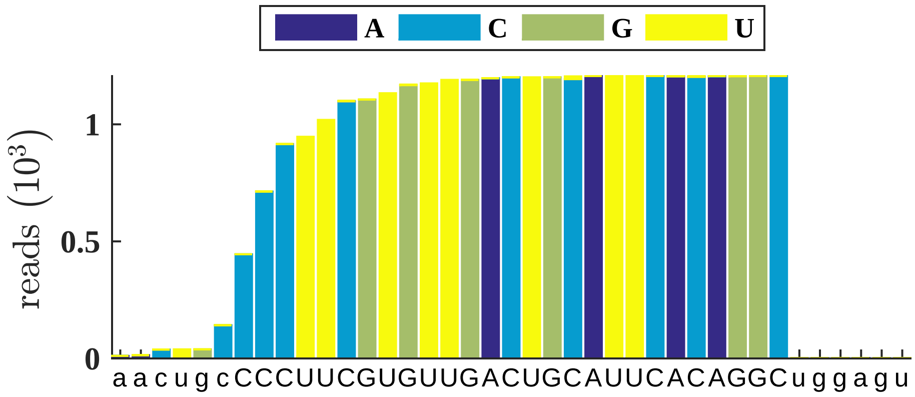

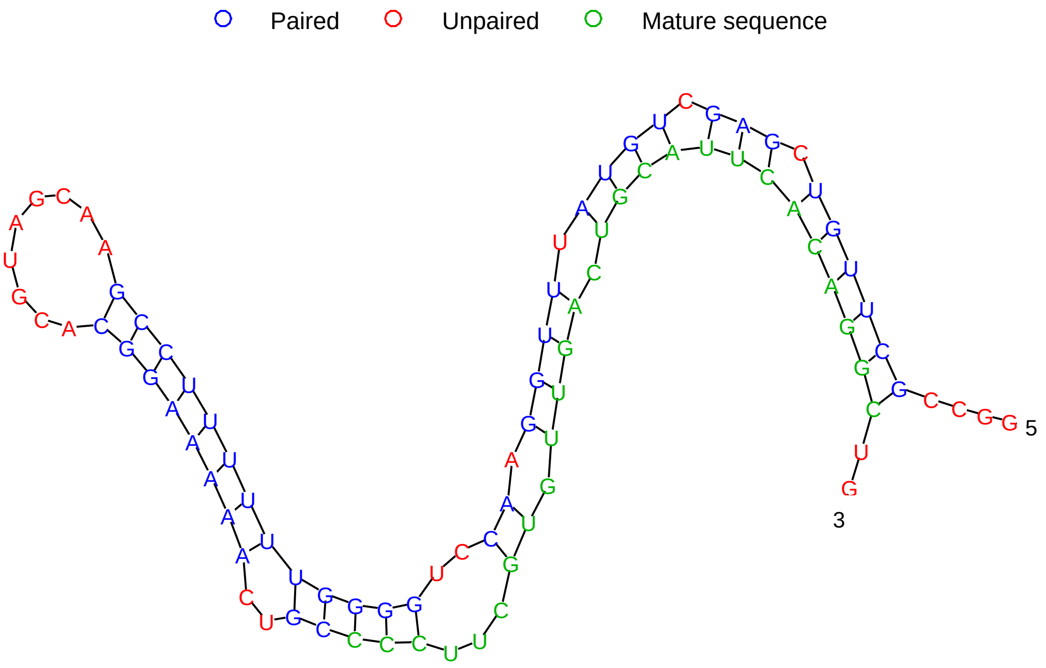

Stem loop (UMD3.1): chr6:17988923-17989014
Mature (UMD3.1): chr6:17988986-17989012
Mature seq len: 27
Total raw counts (9 samples): 1188
Average raw counts: 132
Strand: Forward
Orientation: 3p
Minimum free energy: -28.90

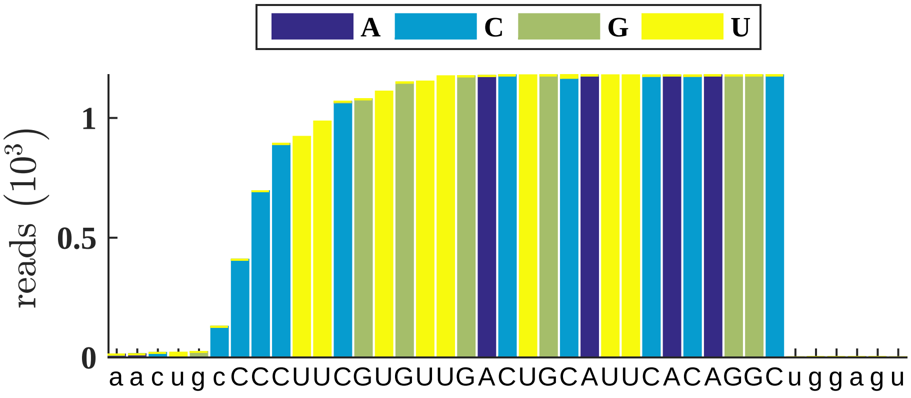

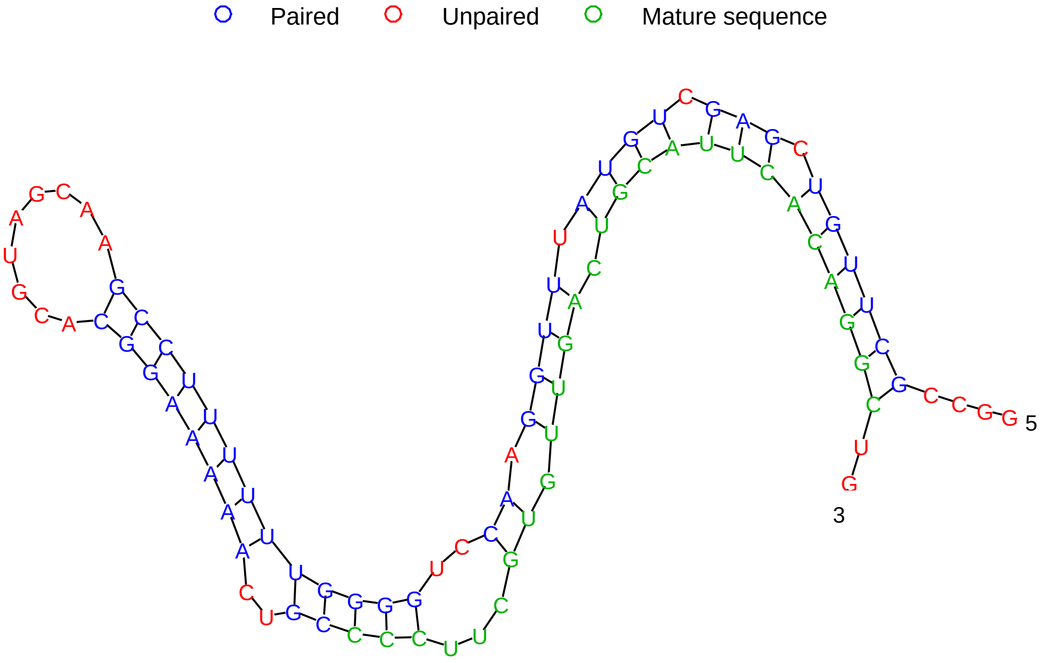

Stem loop (UMD3.1): chr6:17990263-17990354
Mature (UMD3.1): chr6:17990326-17990352
Mature seq len: 27
Total raw counts (9 samples): 1173
Average raw counts: 131
Strand: Forward
Orientation: 3p
Minimum free energy: -28.90

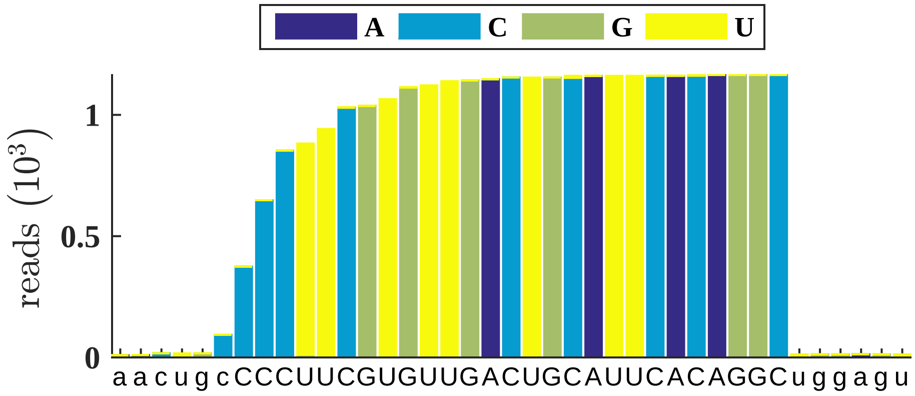

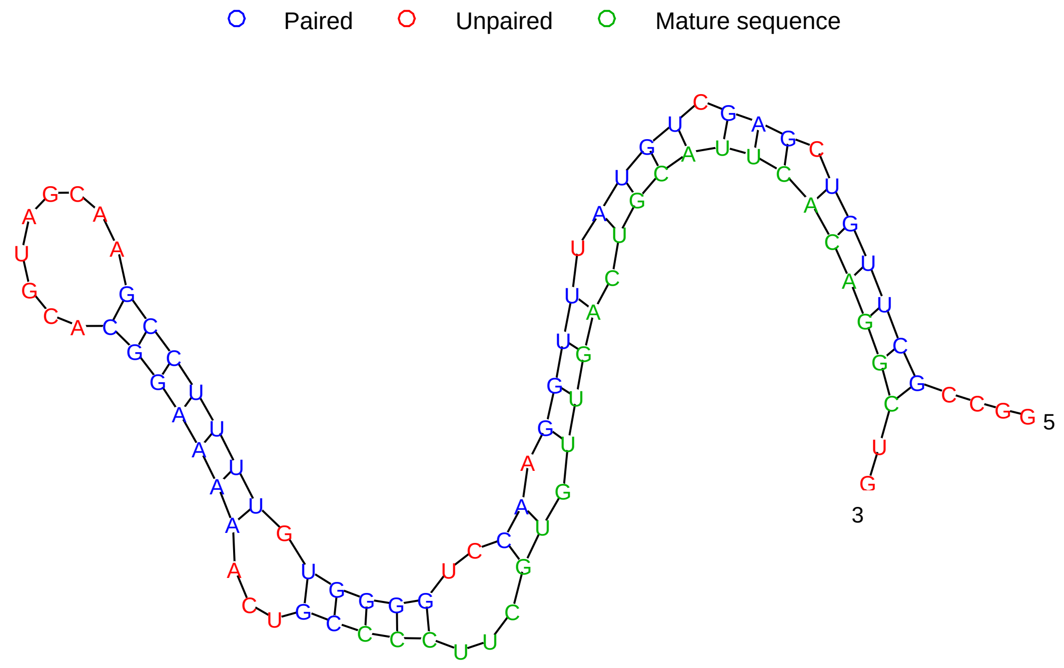

Stem loop (UMD3.1): chr6:17995420-17995511
Mature (UMD3.1): chr6:17995483-17995509
Mature seq len: 27
Total raw counts (9 samples): 1211
Average raw counts: 135
Strand: Forward
Orientation: 3p
Minimum free energy: -28.10

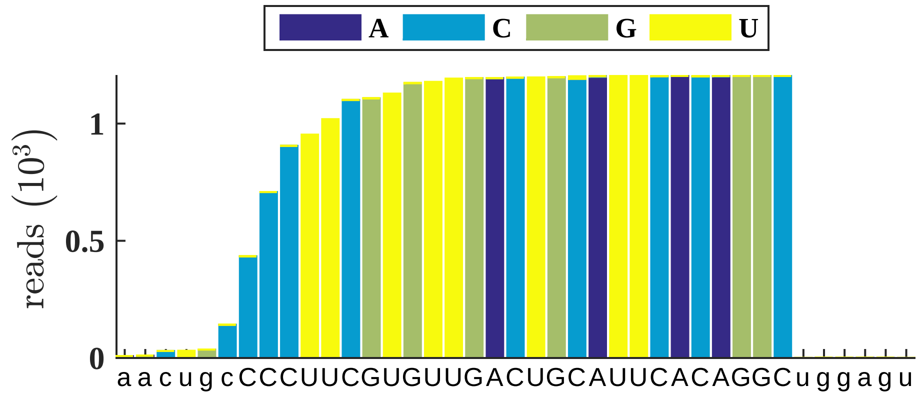

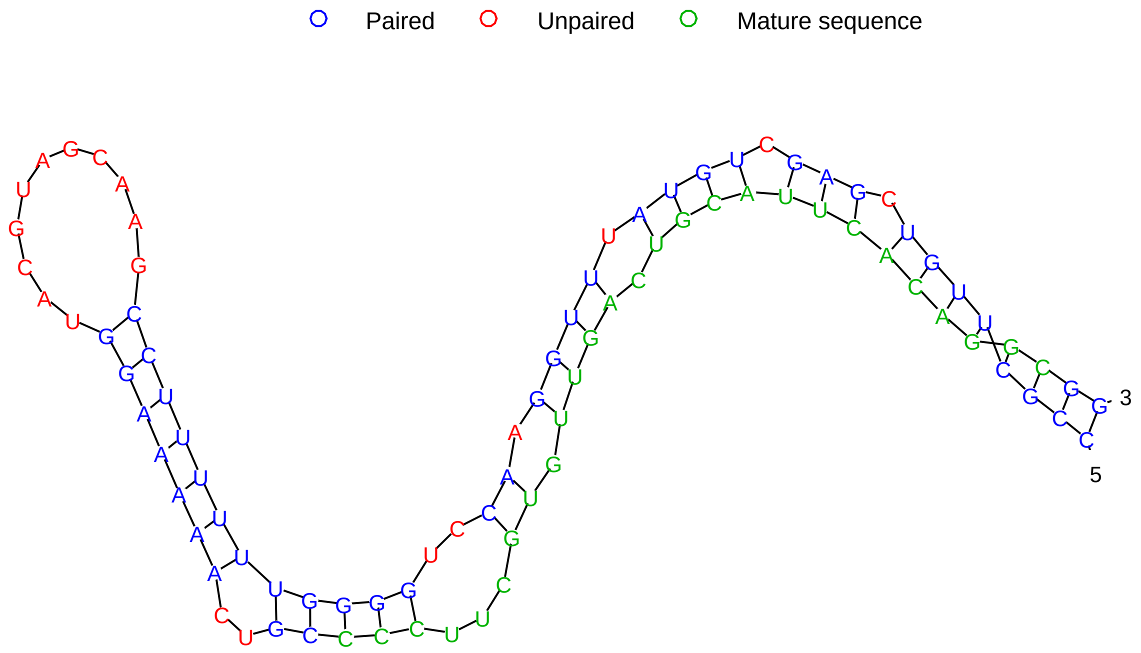

Stem loop (UMD3.1): chr6:18000401-18000490
Mature (UMD3.1): chr6:18000462-18000488
Mature seq len: 27
Total raw counts (9 samples): 1237
Average raw counts: 138
Strand: Forward
Orientation: 3p
Minimum free energy: -31.00

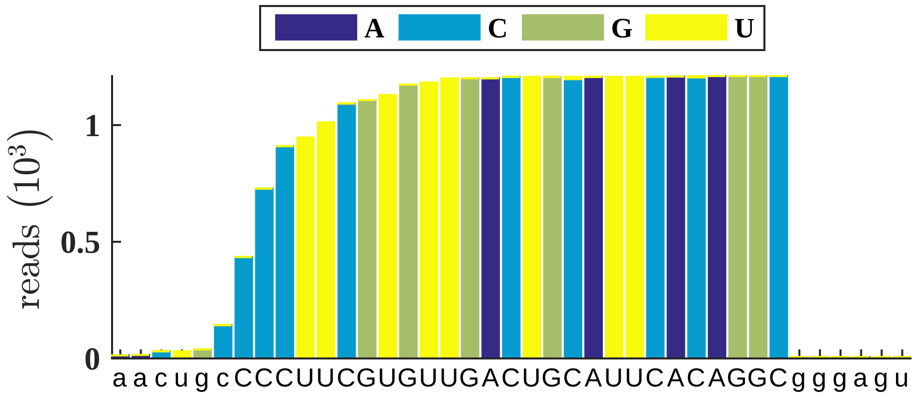

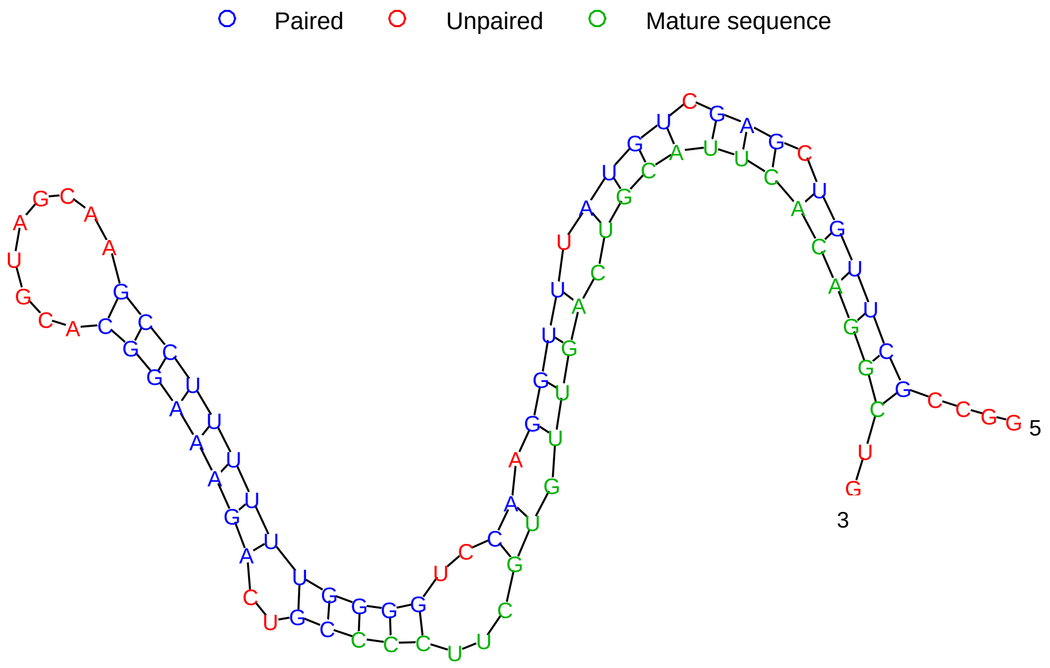

Stem loop (UMD3.1): chr6:18007616-18007707
Mature (UMD3.1): chr6:18007618-18007644
Mature seq len: 27
Total raw counts (9 samples): 1228
Average raw counts: 137
Strand: Reverse
Orientation: 3p
Minimum free energy: -29.00

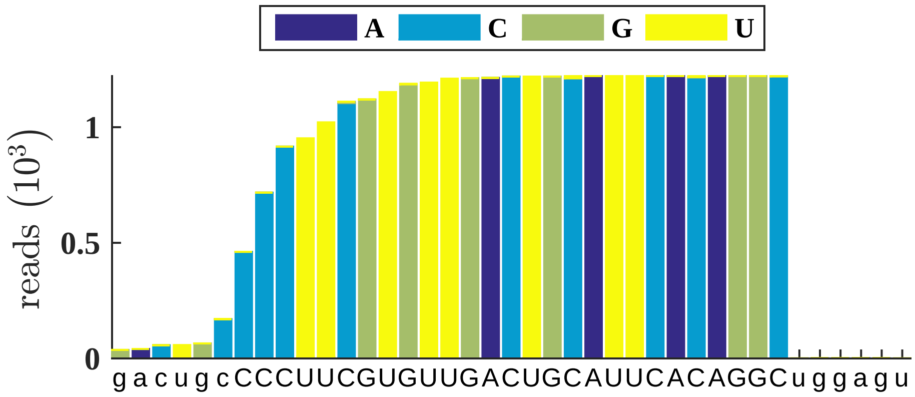

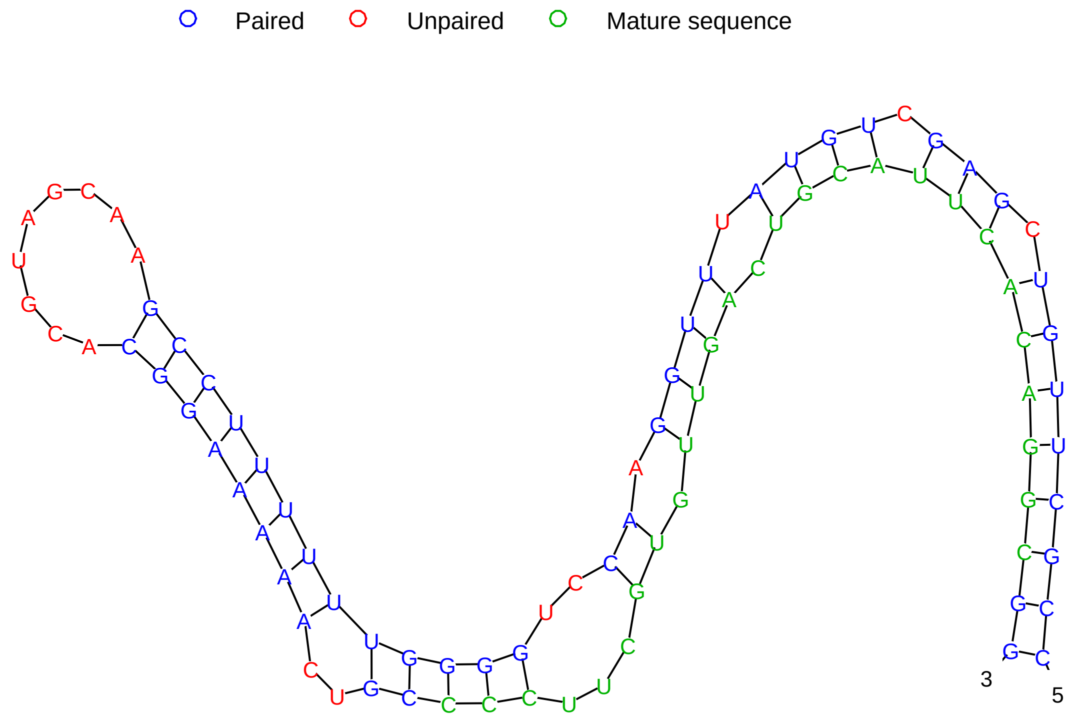

Stem loop (UMD3.1): chr6:23792682-23792771
Mature (UMD3.1): chr6:23792743-23792769
Mature seq len: 27
Total raw counts (9 samples): 1228
Average raw counts: 137
Strand: Forward
Orientation: 3p
Minimum free energy: -33.20

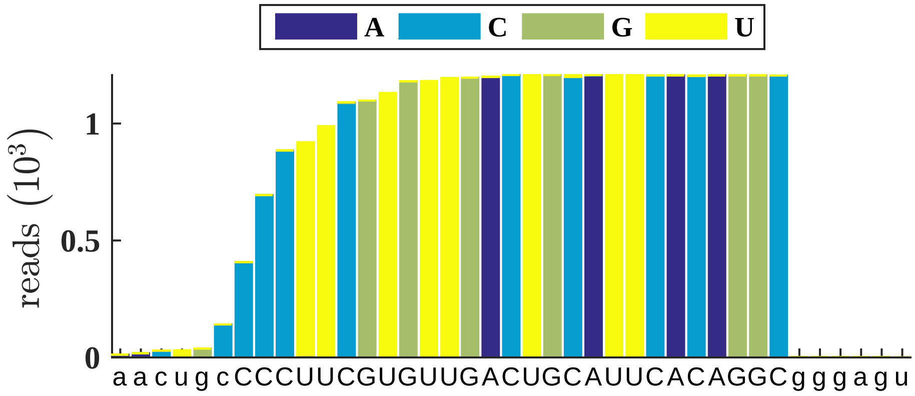

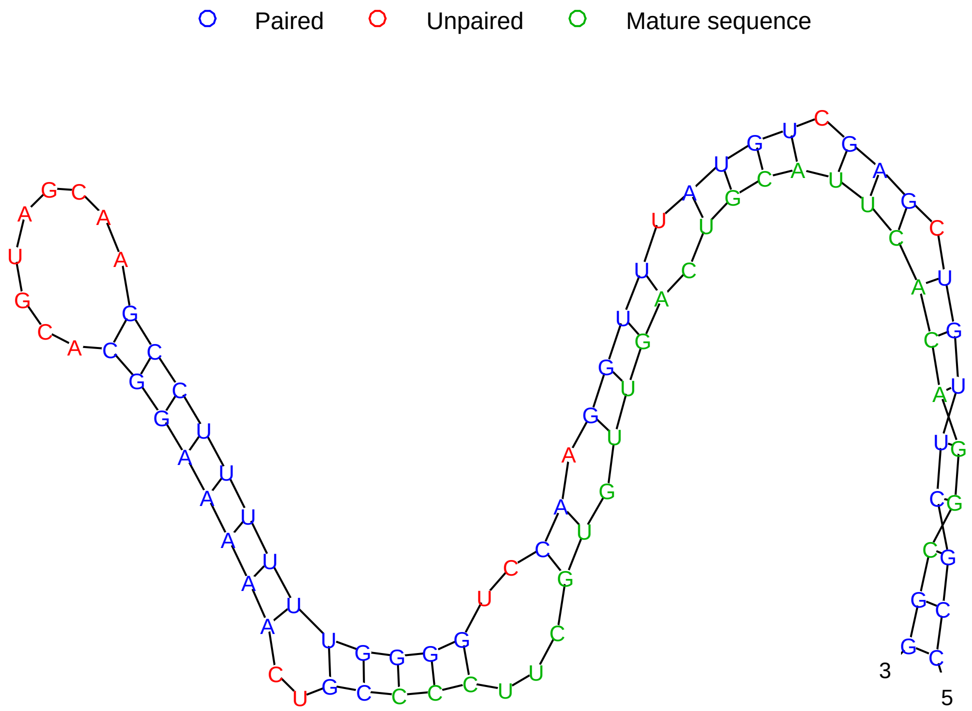

Stem loop (UMD3.1): chr6:23794083-23794172
Mature (UMD3.1): chr6:23794144-23794170
Mature seq len: 27
Total raw counts (9 samples): 1169
Average raw counts: 130
Strand: Forward
Orientation: 3p
Minimum free energy: -33.20

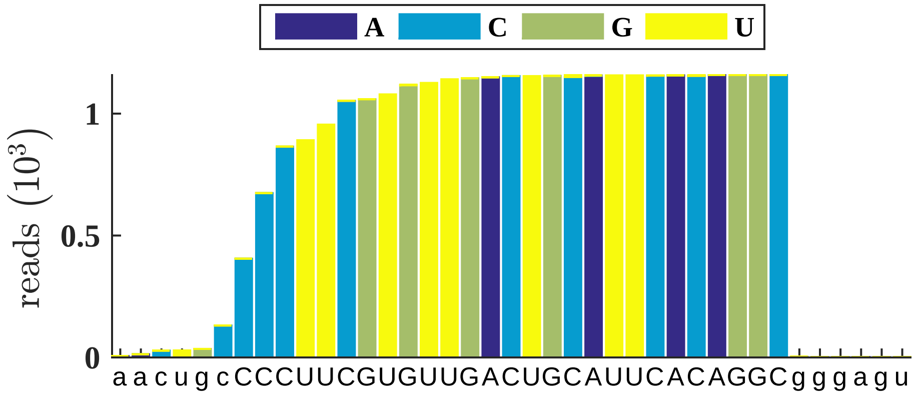

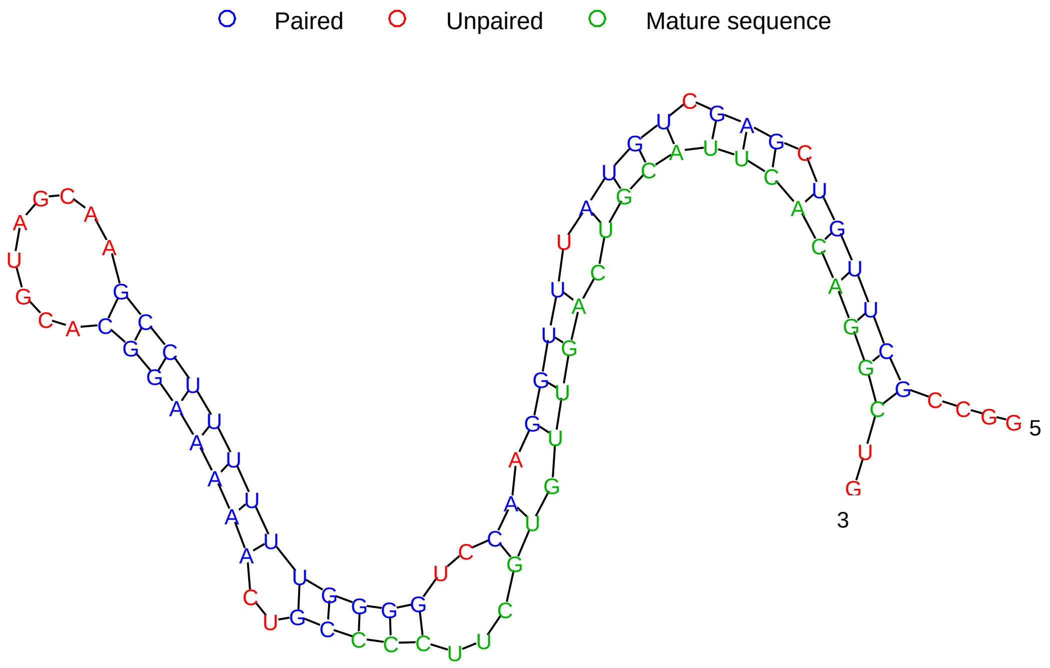

Stem loop (UMD3.1): chr6:26972136-26972227
Mature (UMD3.1): chr6:26972199-26972225
Mature seq len: 27
Total raw counts (9 samples): 1157
Average raw counts: 129
Strand: Forward
Orientation: 3p
Minimum free energy: -28.90

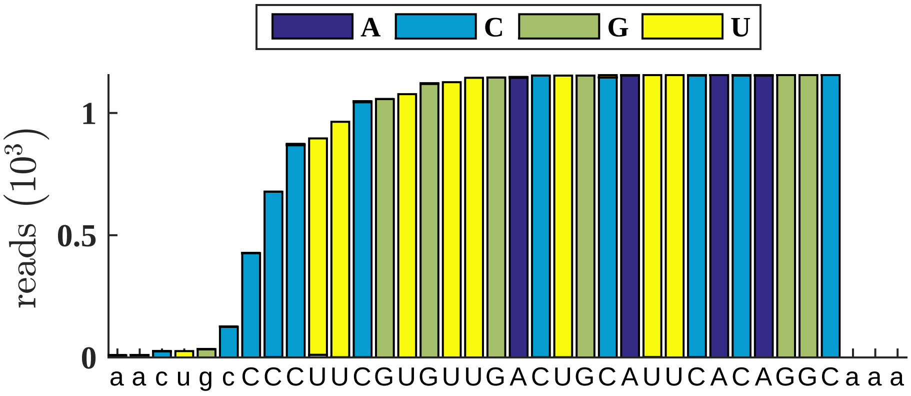

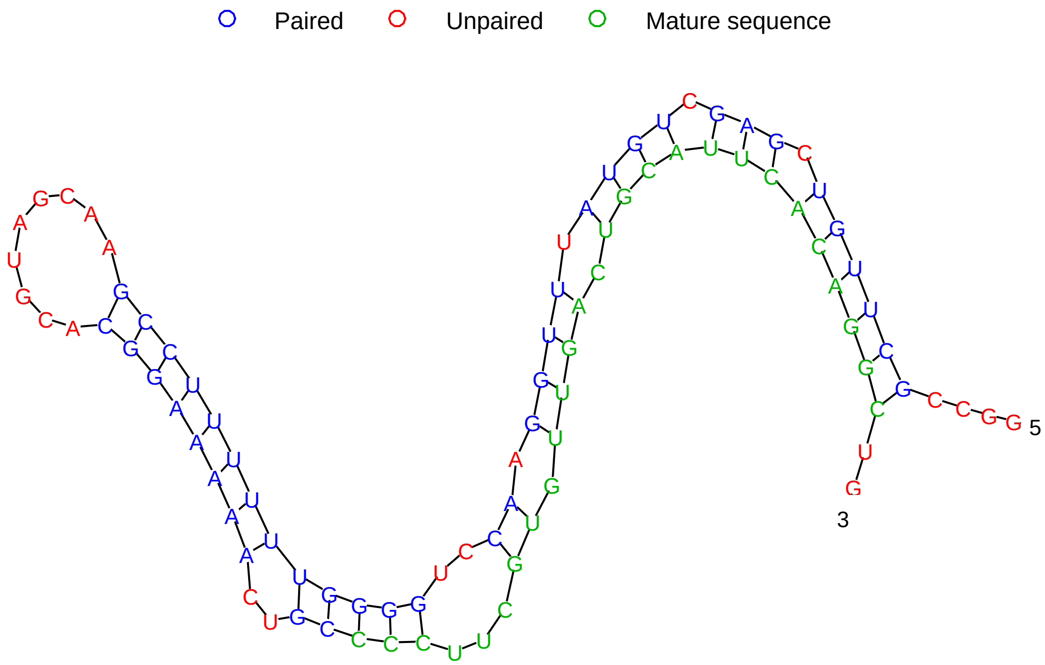

Stem loop (UMD3.1): chr6:27298569-27298660
Mature (UMD3.1): chr6:27298632-27298658
Mature seq len: 27
Total raw counts (9 samples): 1215
Average raw counts: 135
Strand: Forward
Orientation: 3p
Minimum free energy: -28.90

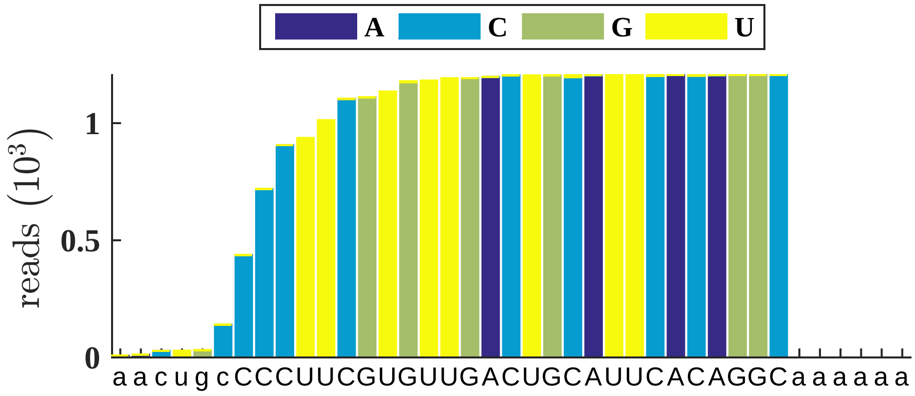

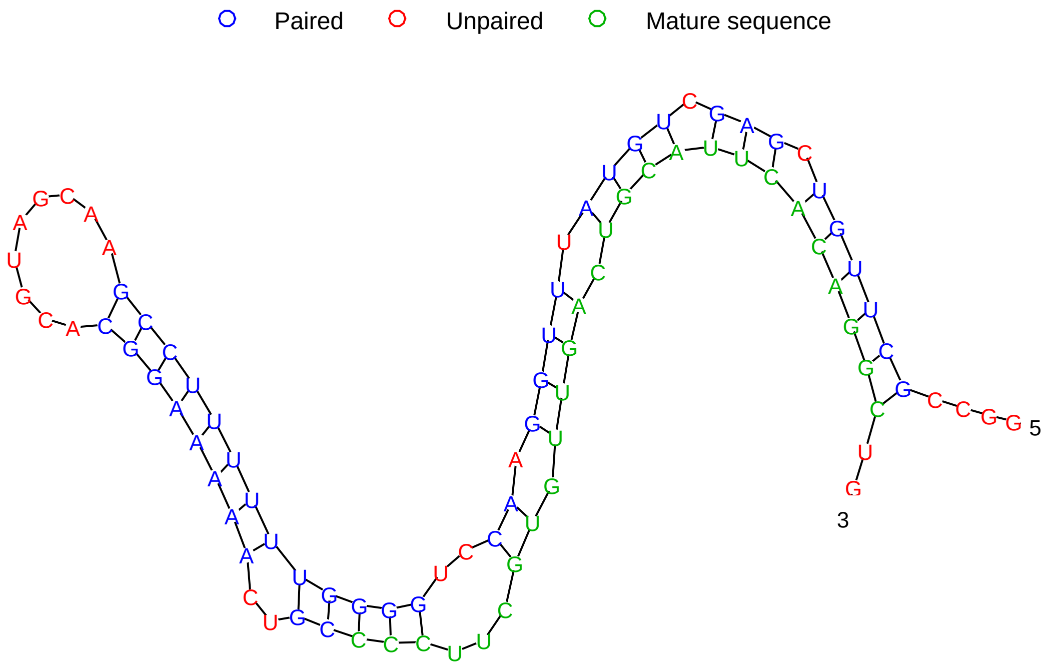

Stem loop (UMD3.1): chr6:27304961-27305052
Mature (UMD3.1): chr6:27305024-27305050
Mature seq len: 27
Total raw counts (9 samples): 1116
Average raw counts: 124
Strand: Forward
Orientation: 3p
Minimum free energy: -28.90

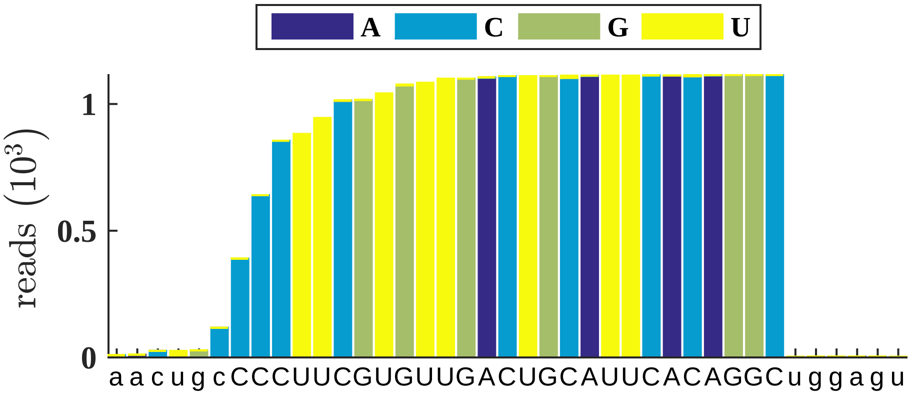

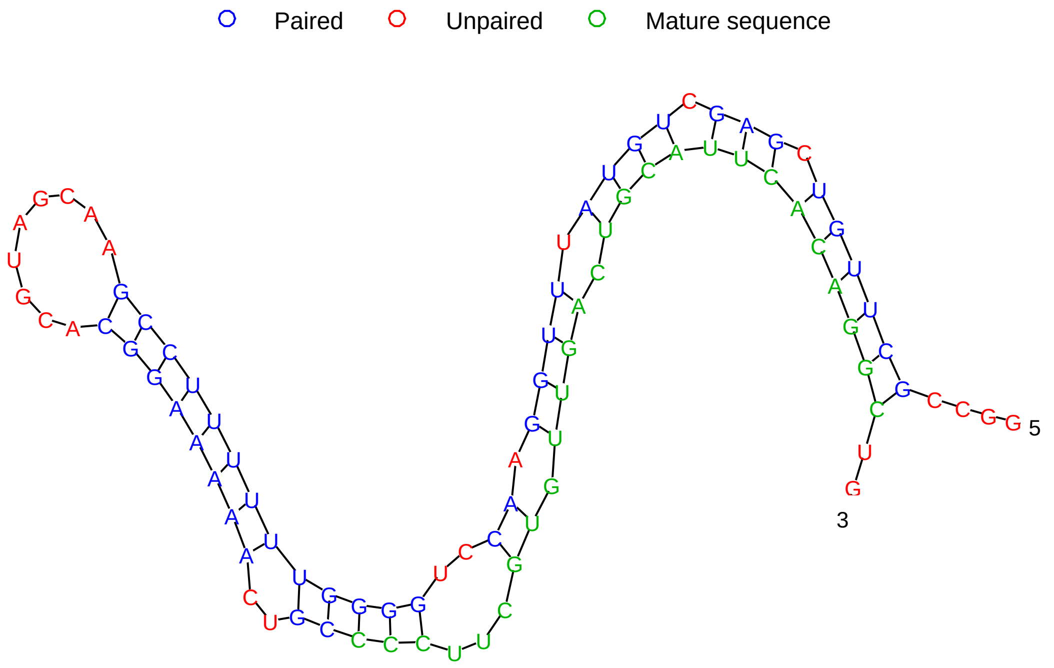

Stem loop (UMD3.1): chr6:27306340-27306431
Mature (UMD3.1): chr6:27306403-27306429
Mature seq len: 27
Total raw counts (9 samples): 1218
Average raw counts: 136
Strand: Forward
Orientation: 3p
Minimum free energy: -28.90

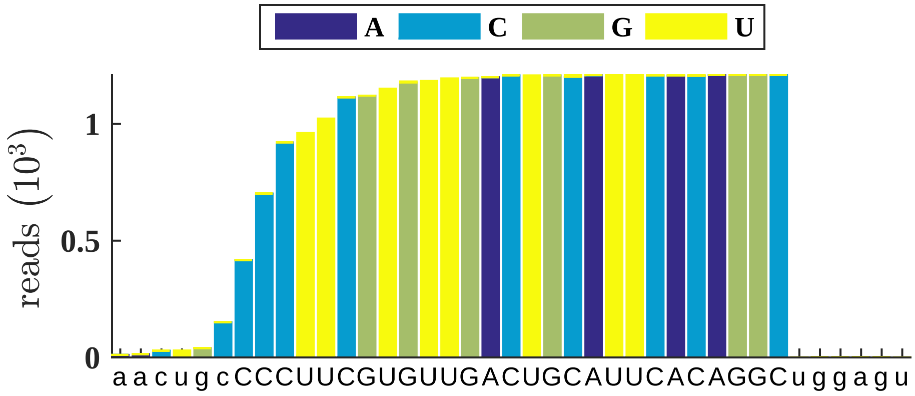

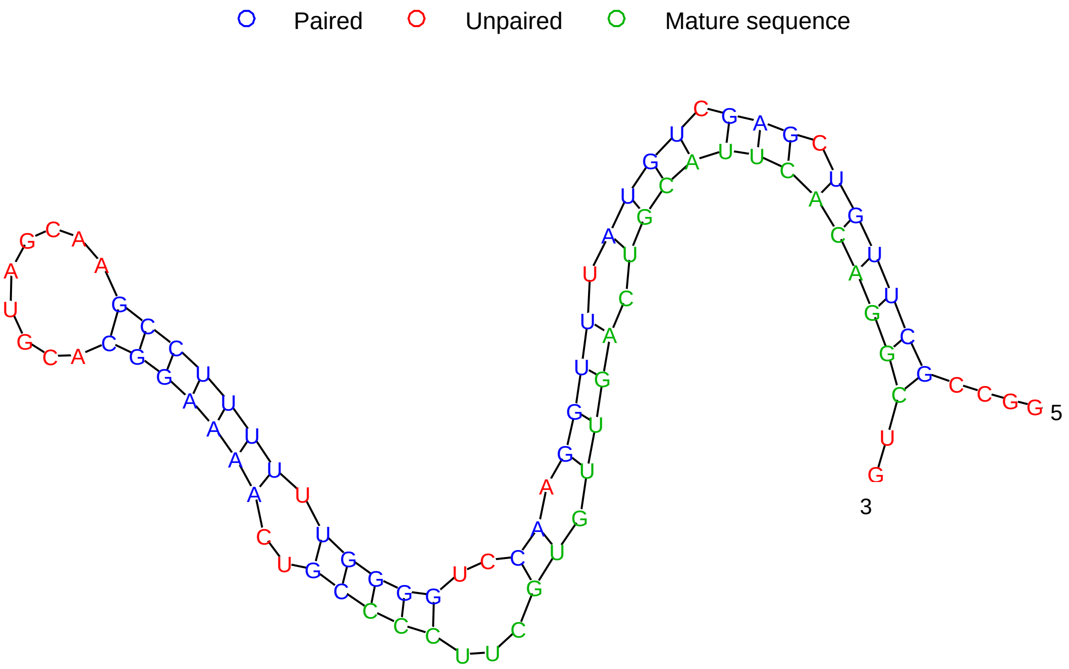

Stem loop (UMD3.1): chr6:27307214-27307304
Mature (UMD3.1): chr6:27307276-27307302
Mature seq len: 27
Total raw counts (9 samples): 1218
Average raw counts: 136
Strand: Forward
Orientation: 3p
Minimum free energy: -28.80

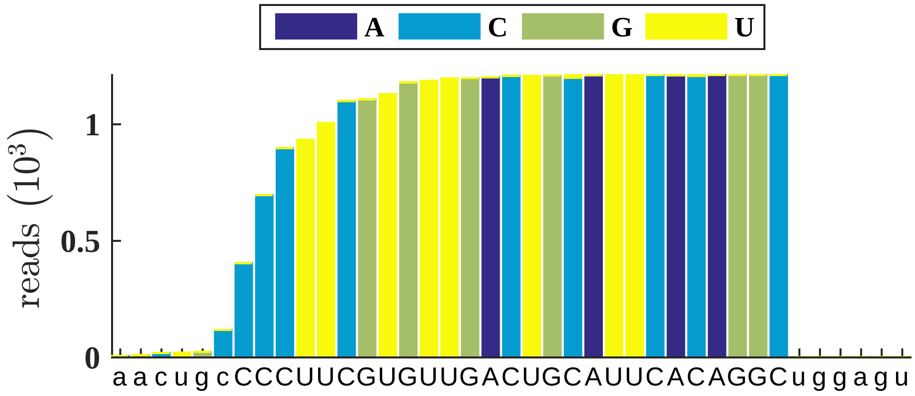

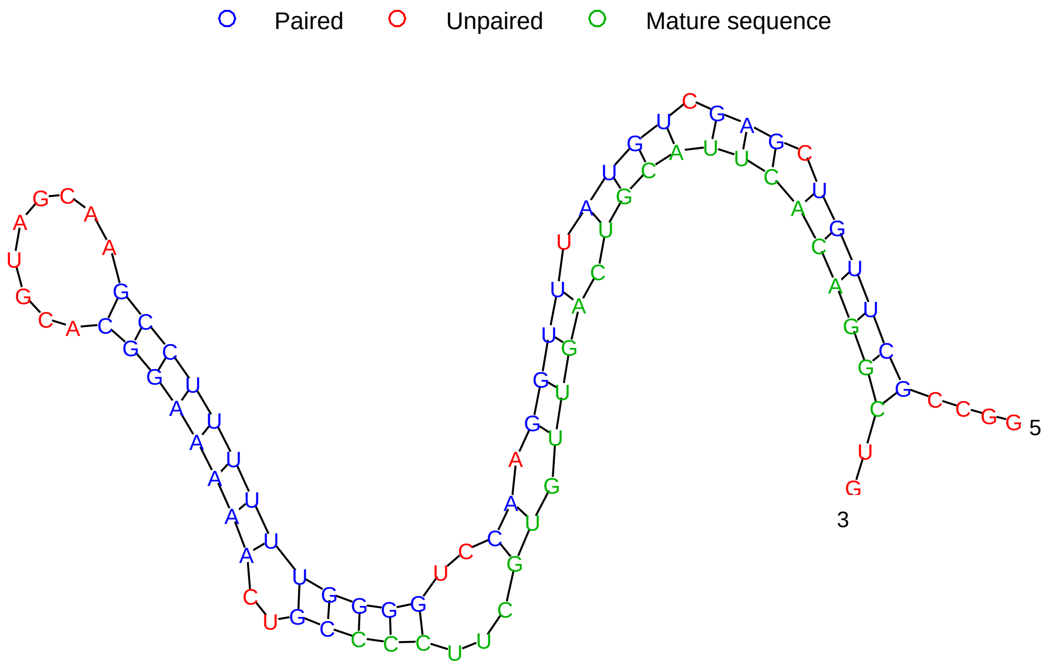

Stem loop (UMD3.1): chr6:27309817-27309908
Mature (UMD3.1): chr6:27309880-27309906
Mature seq len: 27
Total raw counts (9 samples): 1176
Average raw counts: 131
Strand: Forward
Orientation: 3p
Minimum free energy: -28.90

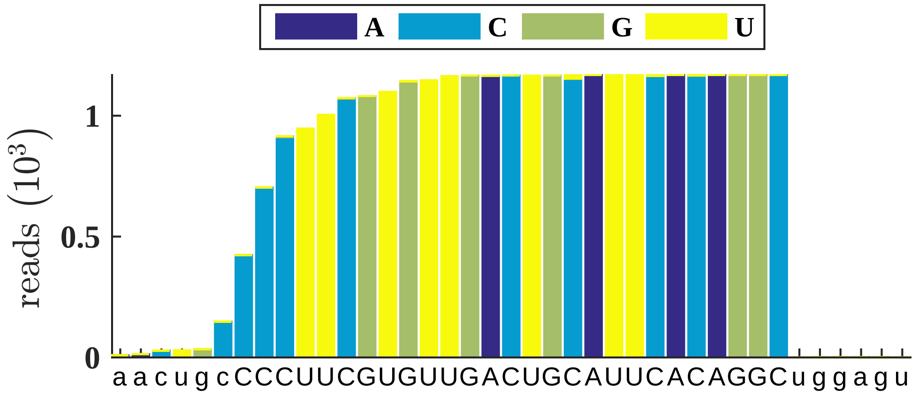

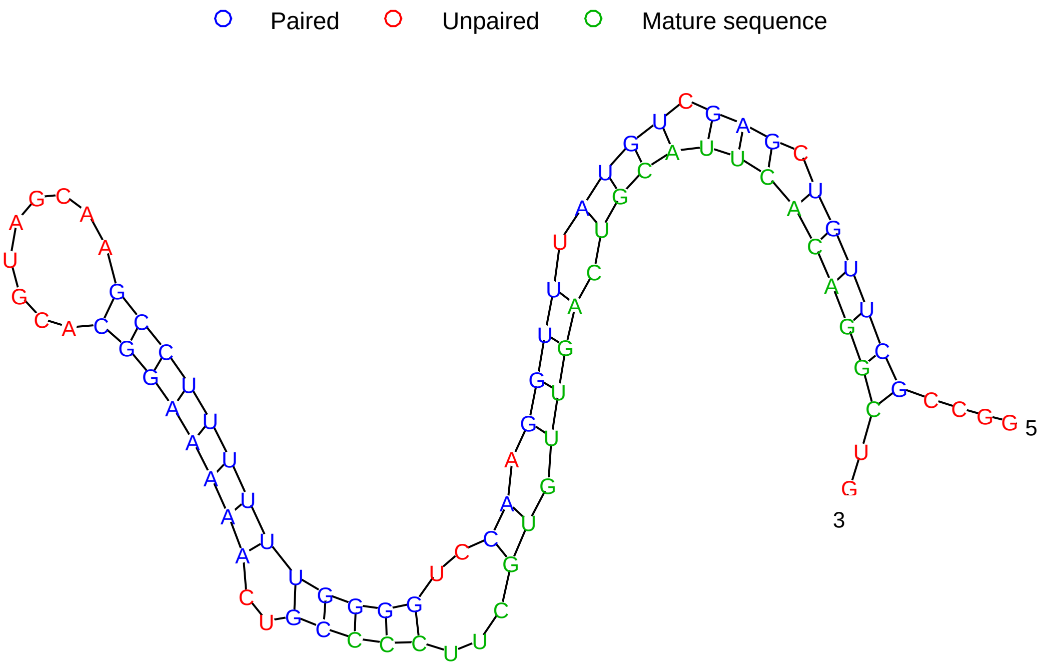

Stem loop (UMD3.1): chr6:27311769-27311860
Mature (UMD3.1): chr6:27311832-27311858
Mature seq len: 27
Total raw counts (9 samples): 1185
Average raw counts: 132
Strand: Forward
Orientation: 3p
Minimum free energy: -28.90

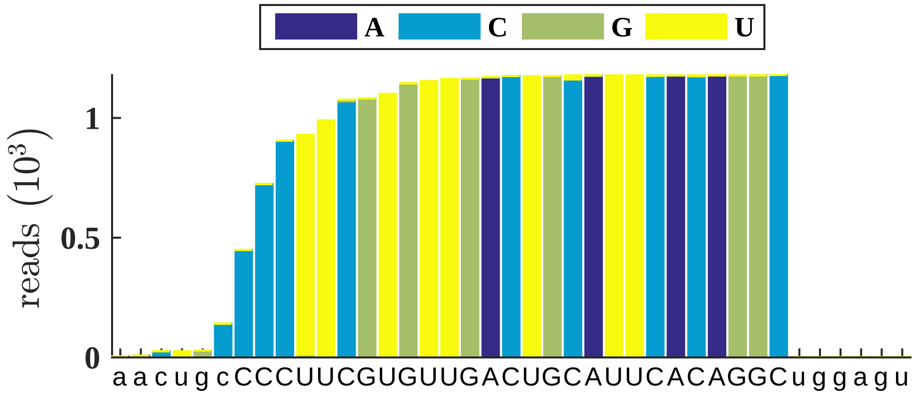

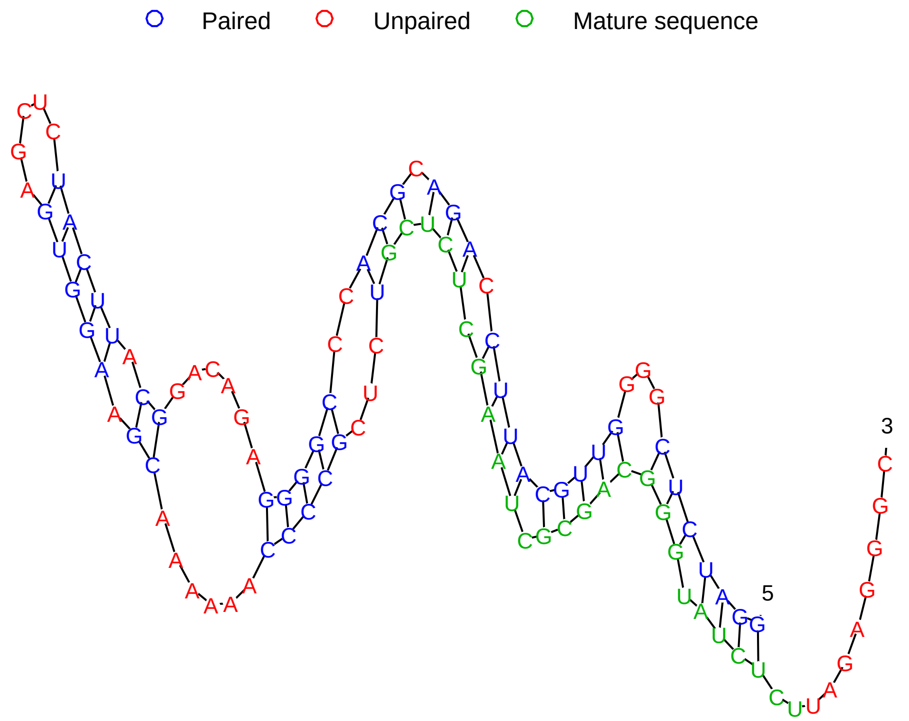

Stem loop (UMD3.1): chr6:27320983-27321092
Mature (UMD3.1): chr6:27321059-27321084
Mature seq len: 26
Total raw counts (9 samples): 3622
Average raw counts: 403
Strand: Forward
Orientation: 3p
Minimum free energy: -32.40

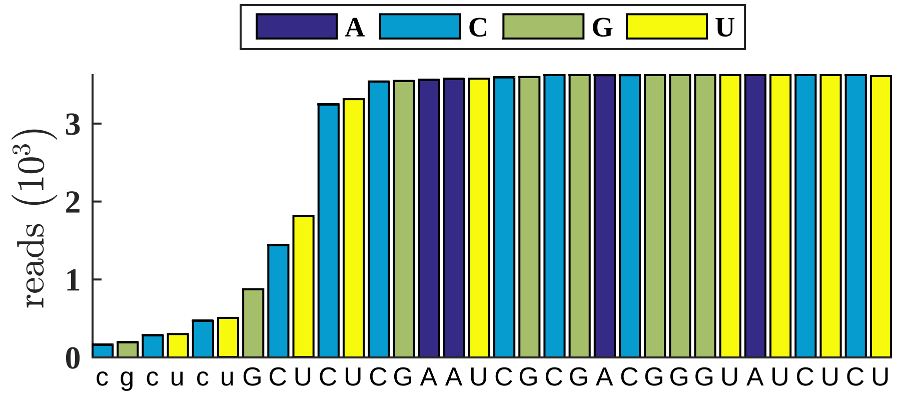

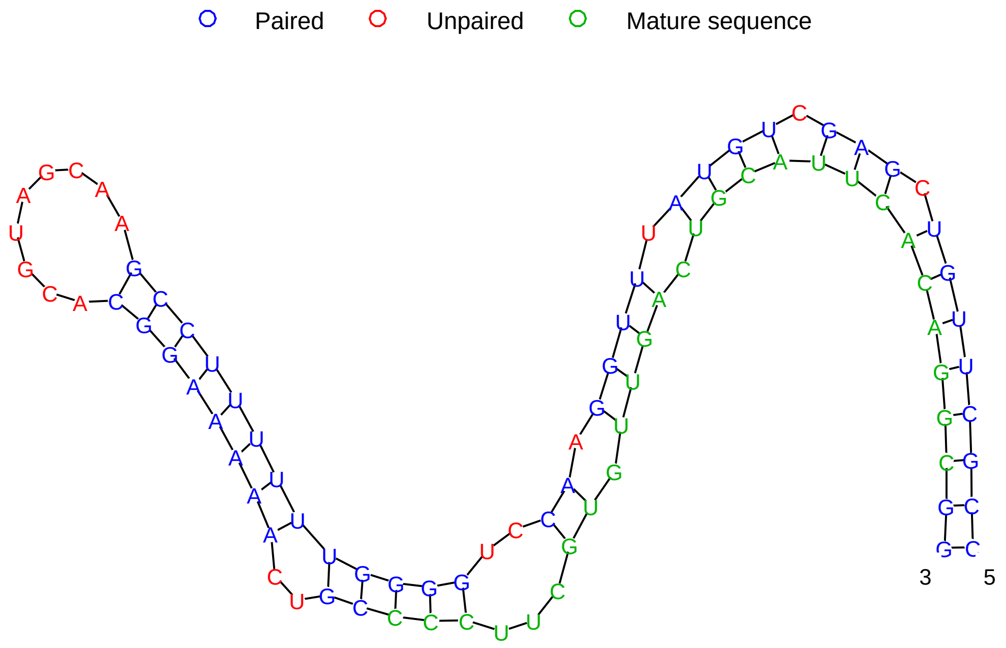

Stem loop (UMD3.1): chr6:33082961-33083050
Mature (UMD3.1): chr6:33082963-33082989
Mature seq len: 27
Total raw counts (9 samples): 1238
Average raw counts: 138
Strand: Reverse
Orientation: 3p
Minimum free energy: -33.20

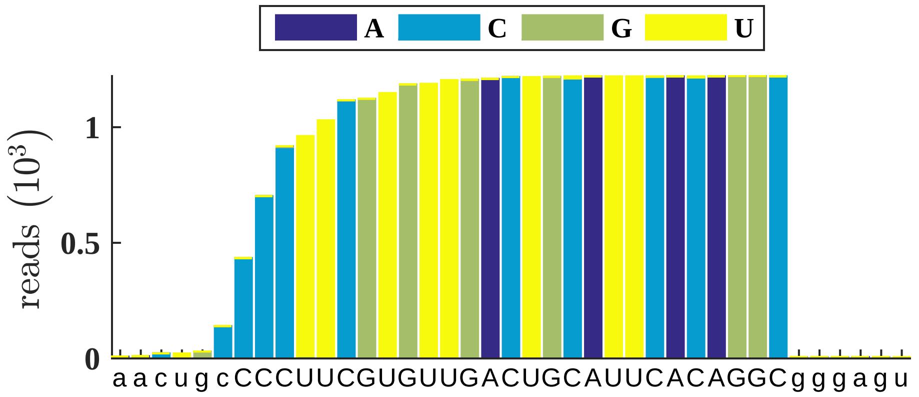

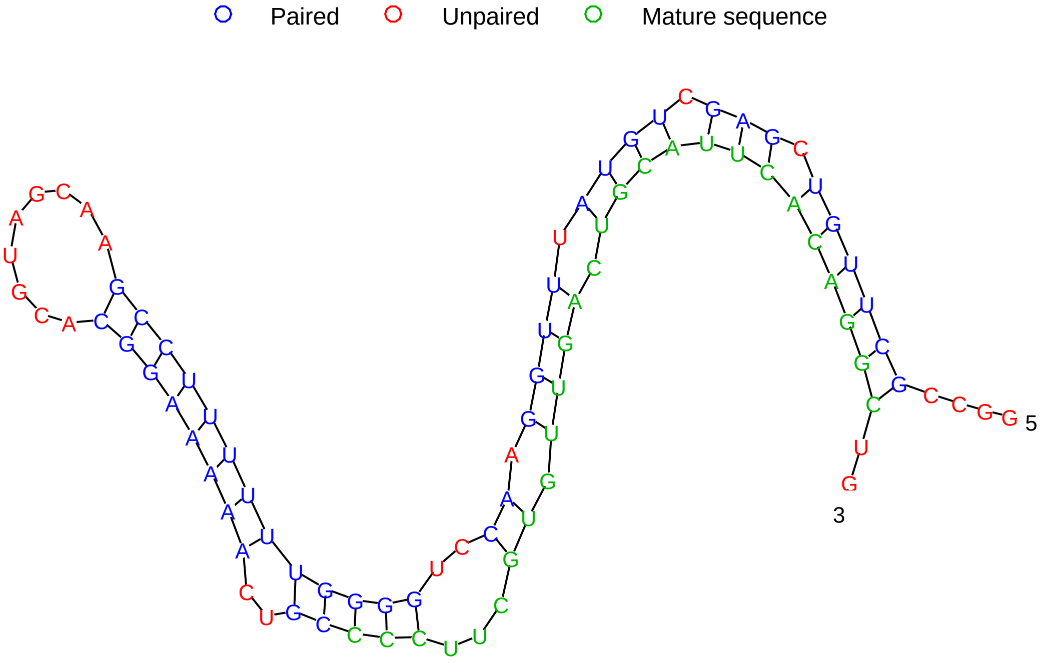

Stem loop (UMD3.1): chr6:45735609-45735700
Mature (UMD3.1): chr6:45735672-45735698
Mature seq len: 27
Total raw counts (9 samples): 1209
Average raw counts: 135
Strand: Forward
Orientation: 3p
Minimum free energy: -28.90

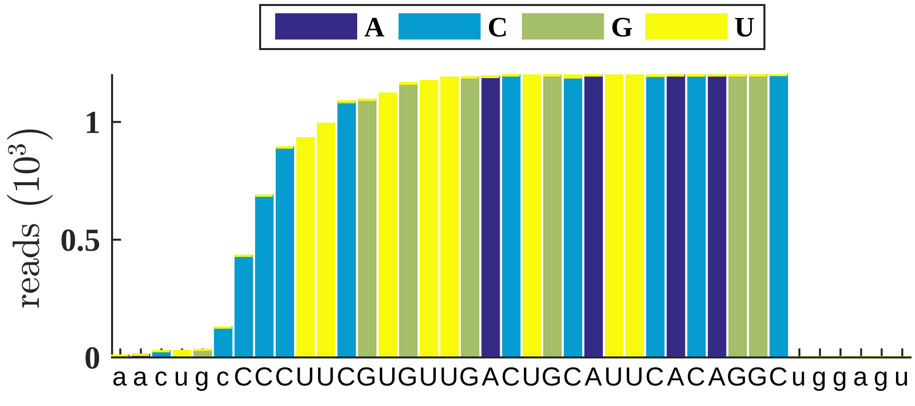

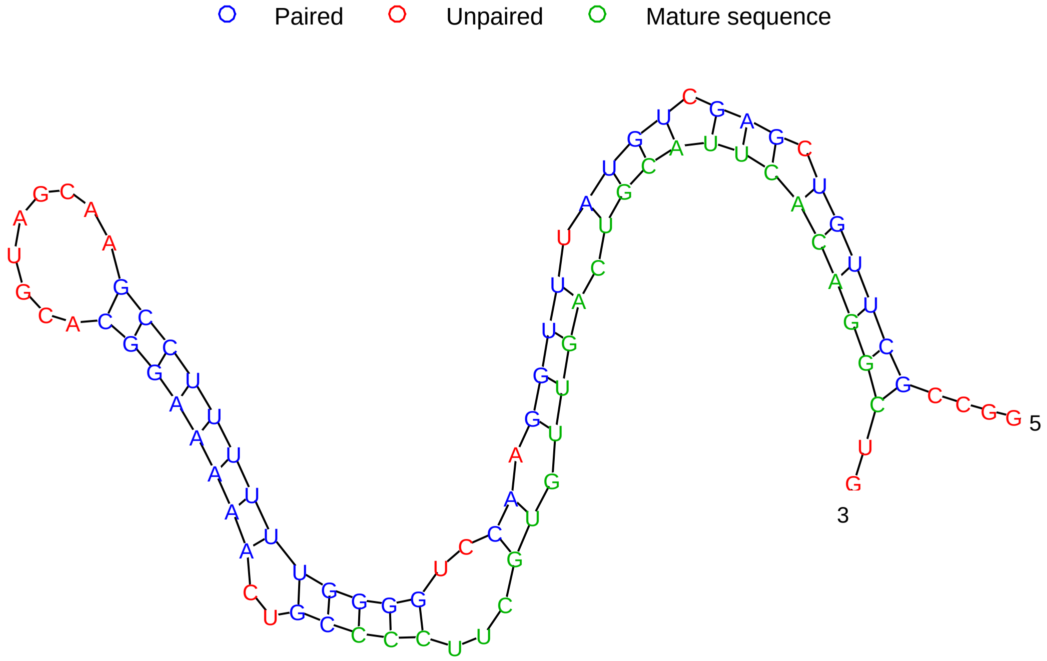

Stem loop (UMD3.1): chr6:45740857-45740948
Mature (UMD3.1): chr6:45740859-45740885
Mature seq len: 27
Total raw counts (9 samples): 1180
Average raw counts: 132
Strand: Reverse
Orientation: 3p
Minimum free energy: -28.90

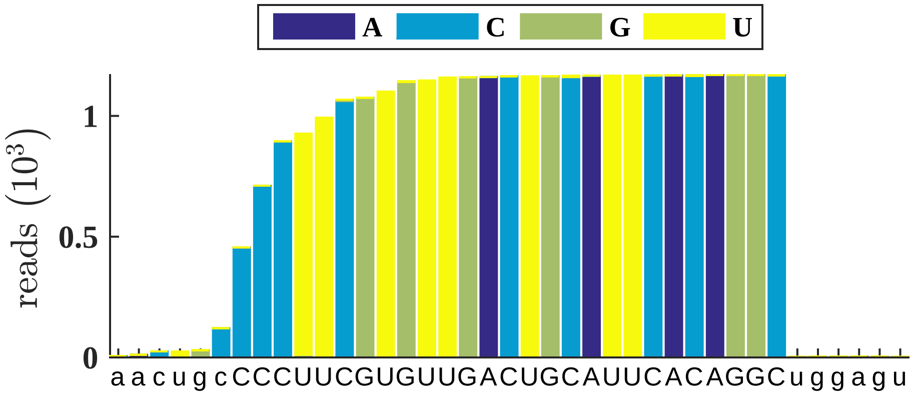

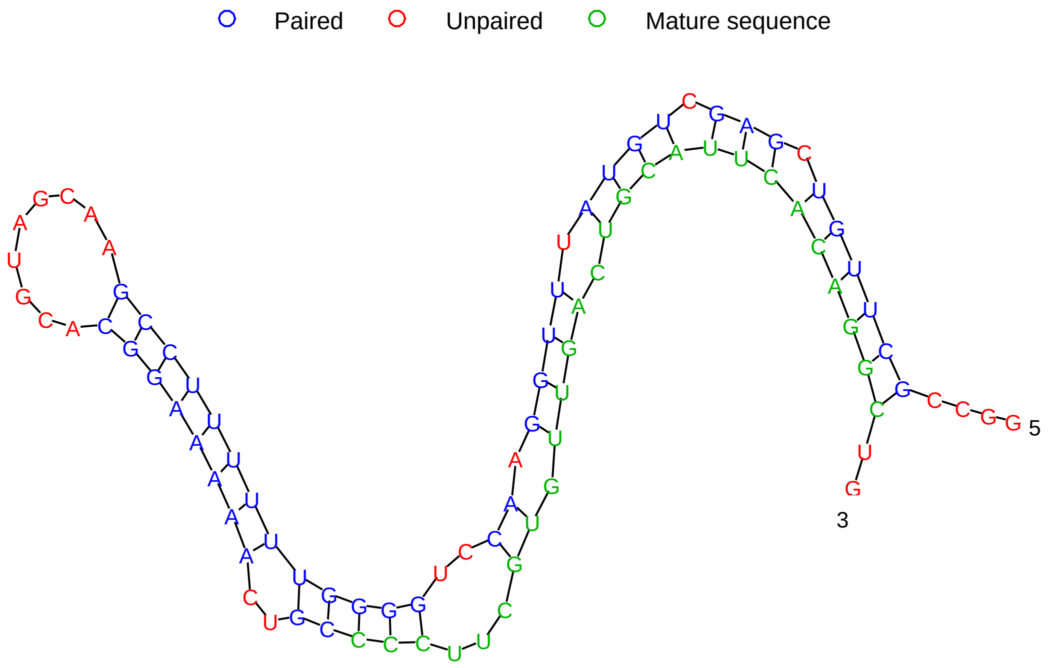

Stem loop (UMD3.1): chr6:45742261-45742352
Mature (UMD3.1): chr6:45742263-45742289
Mature seq len: 27
Total raw counts (9 samples): 1243
Average raw counts: 139
Strand: Reverse
Orientation: 3p
Minimum free energy: -28.90

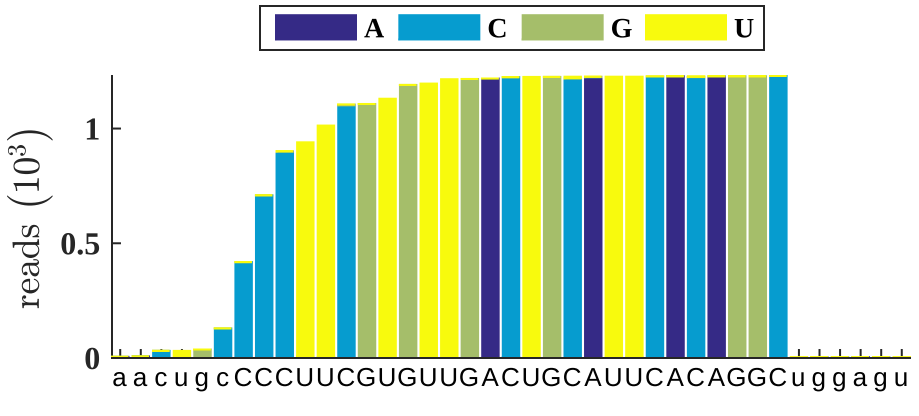

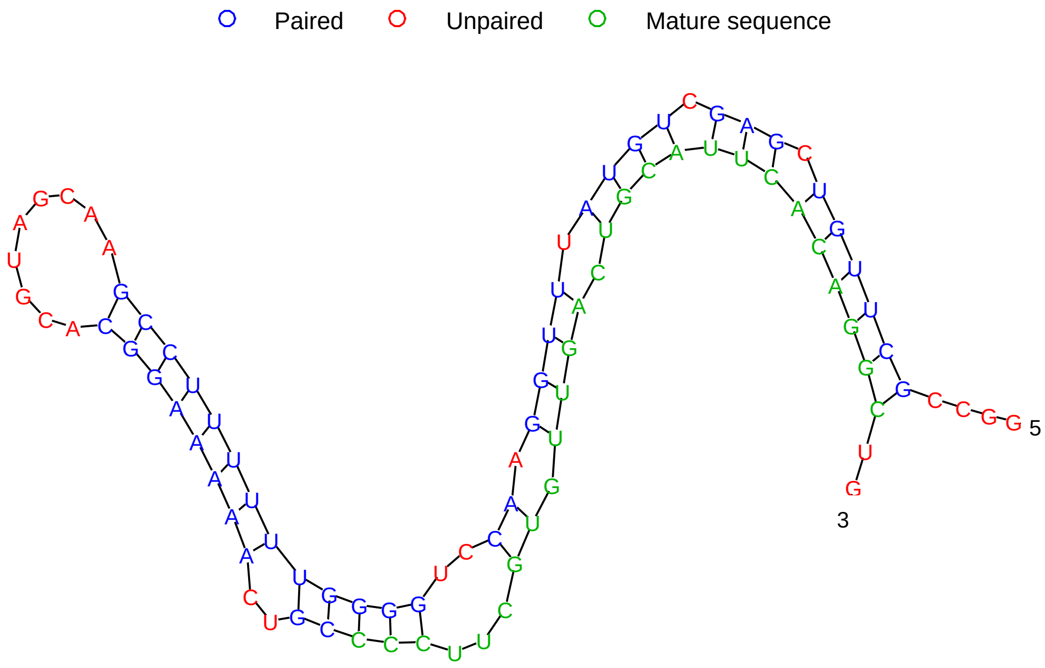

Stem loop (UMD3.1): chr6:45744516-45744607
Mature (UMD3.1): chr6:45744518-45744544
Mature seq len: 27
Total raw counts (9 samples): 1224
Average raw counts: 136
Strand: Reverse
Orientation: 3p
Minimum free energy: -28.90

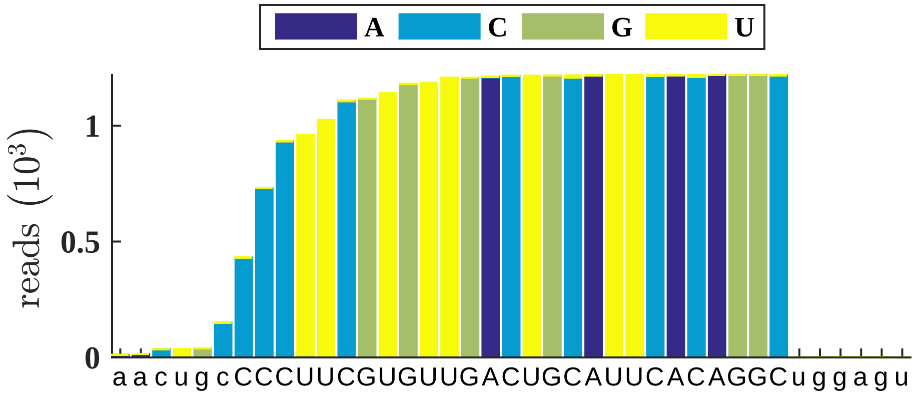

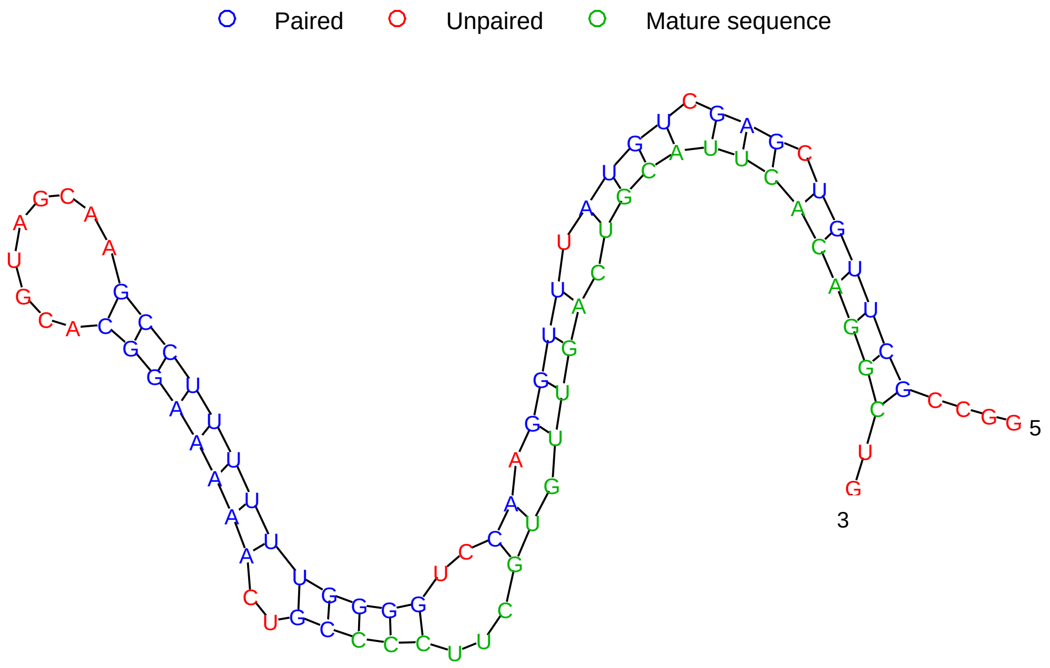

Stem loop (UMD3.1): chr6:45748678-45748769
Mature (UMD3.1): chr6:45748680-45748706
Mature seq len: 27
Total raw counts (9 samples): 1156
Average raw counts: 129
Strand: Reverse
Orientation: 3p
Minimum free energy: -28.90

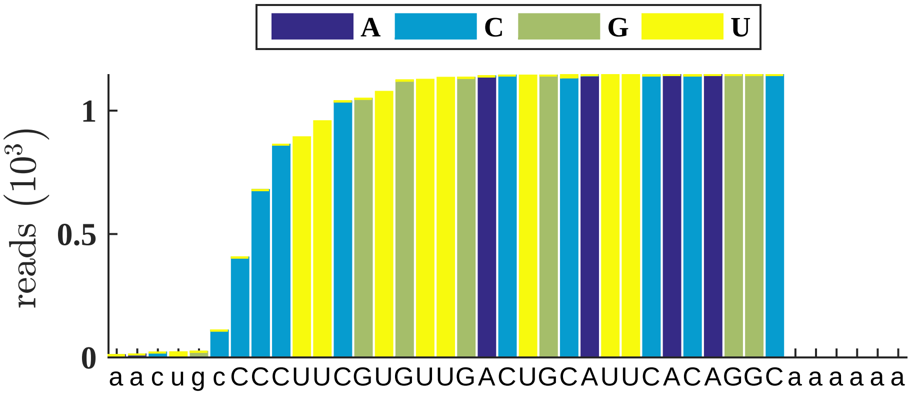

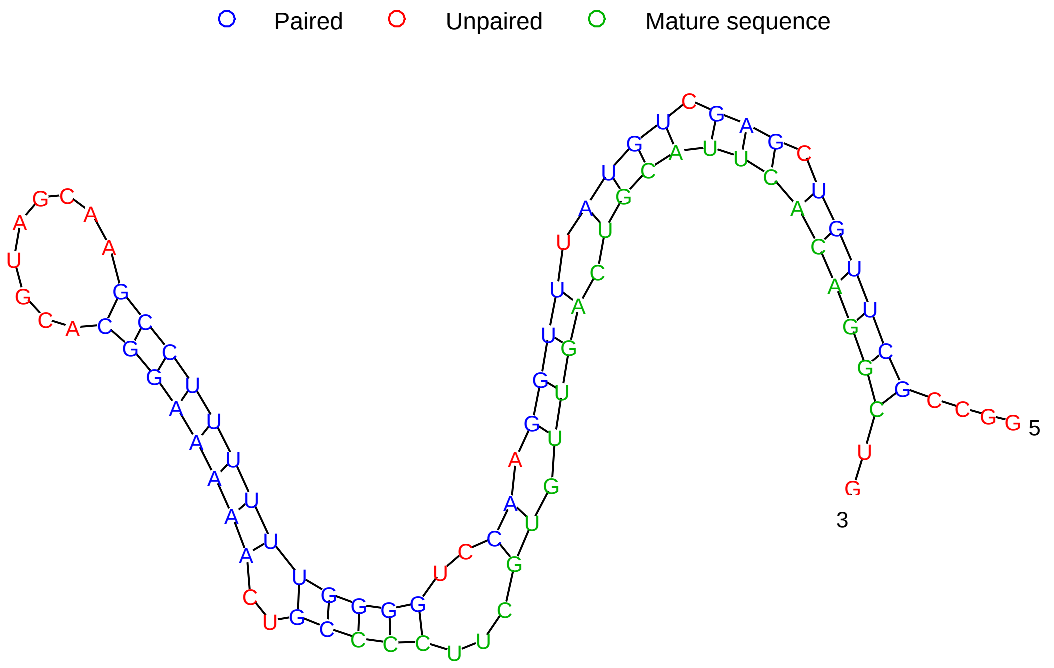

Stem loop (UMD3.1): chr6:45750638-45750729
Mature (UMD3.1): chr6:45750640-45750666
Mature seq len: 27
Total raw counts (9 samples): 1265
Average raw counts: 141
Strand: Reverse
Orientation: 3p
Minimum free energy: -28.90

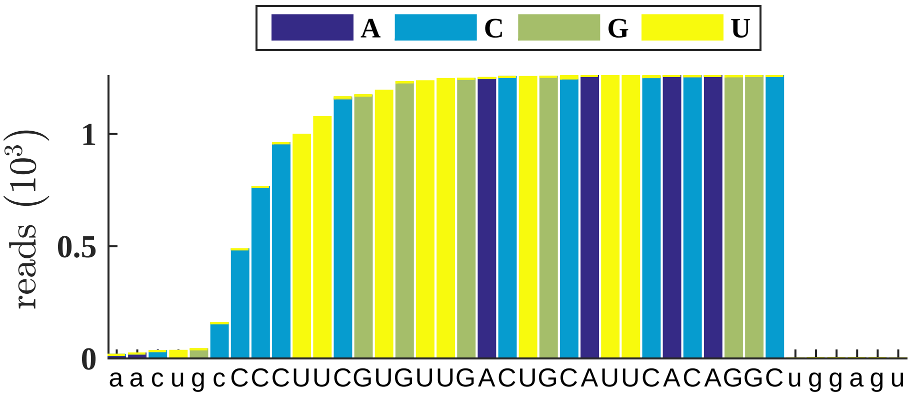

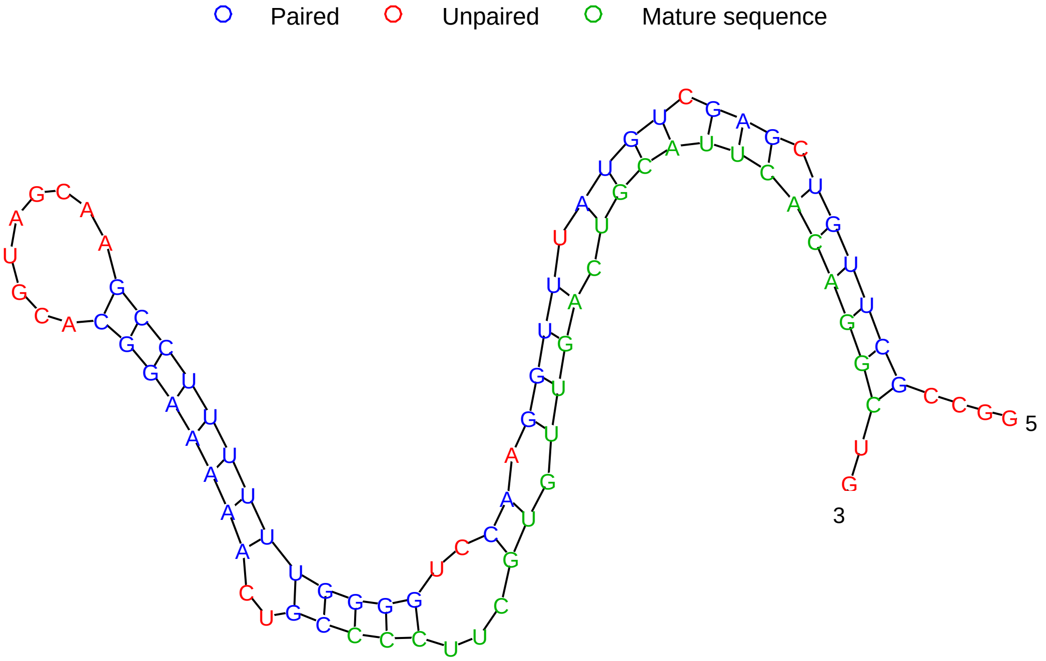

Stem loop (UMD3.1): chr6:45759354-45759445
Mature (UMD3.1): chr6:45759356-45759382
Mature seq len: 27
Total raw counts (9 samples): 1191
Average raw counts: 133
Strand: Reverse
Orientation: 3p
Minimum free energy: -28.90

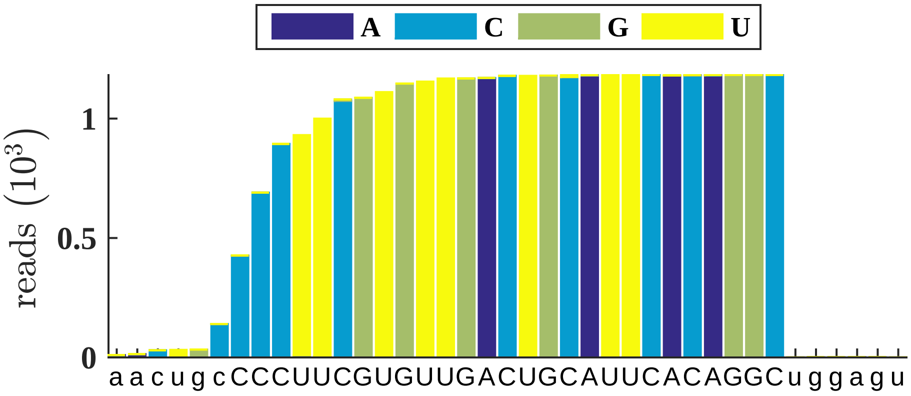

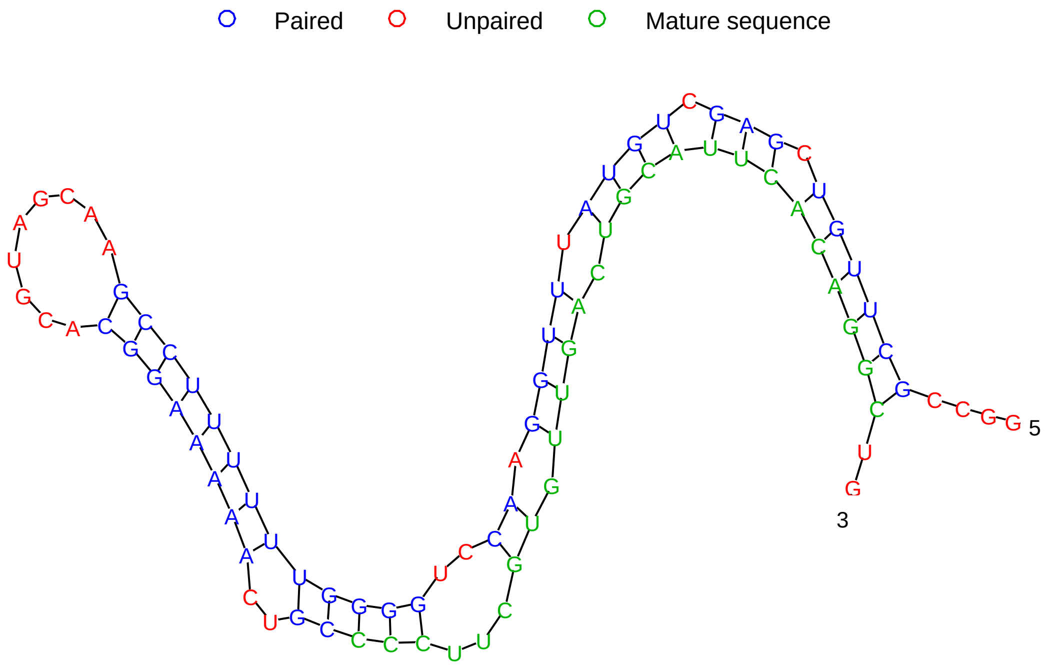

Stem loop (UMD3.1): chr6:45760636-45760727
Mature (UMD3.1): chr6:45760638-45760664
Mature seq len: 27
Total raw counts (9 samples): 1174
Average raw counts: 131
Strand: Reverse
Orientation: 3p
Minimum free energy: -28.90

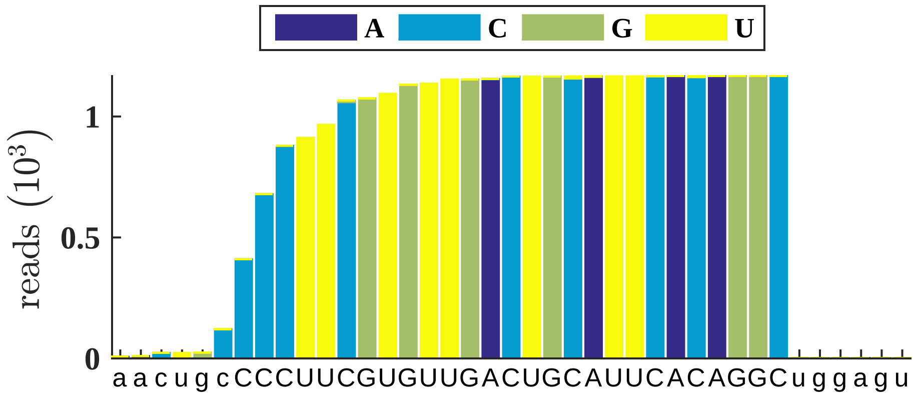

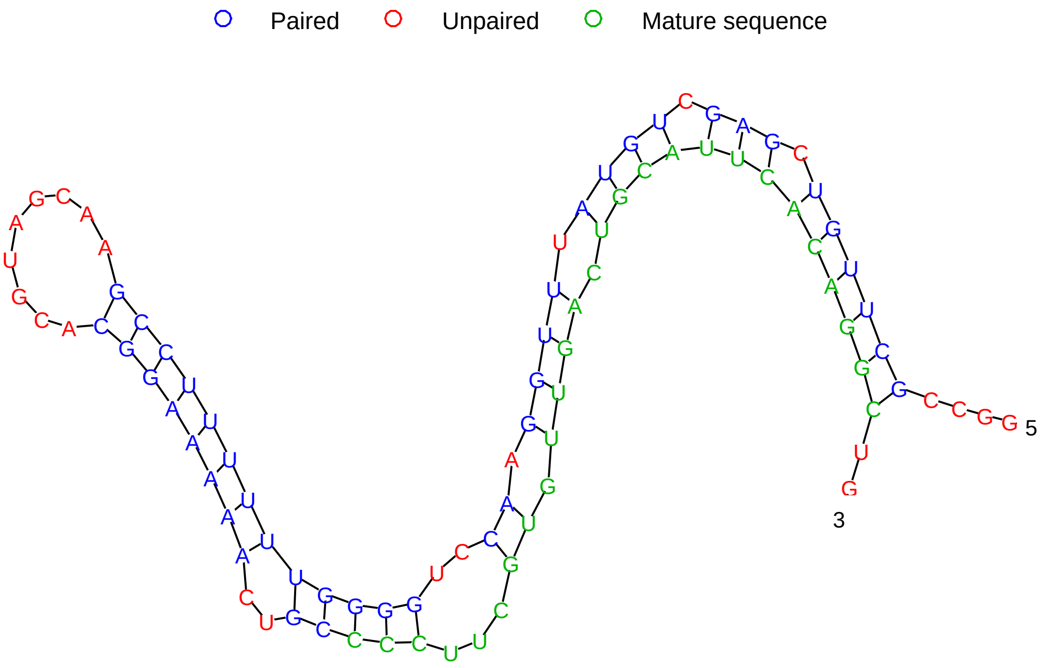

Stem loop (UMD3.1): chr6:45761972-45762063
Mature (UMD3.1): chr6:45761974-45762000
Mature seq len: 27
Total raw counts (9 samples): 1225
Average raw counts: 137
Strand: Reverse
Orientation: 3p
Minimum free energy: -28.90

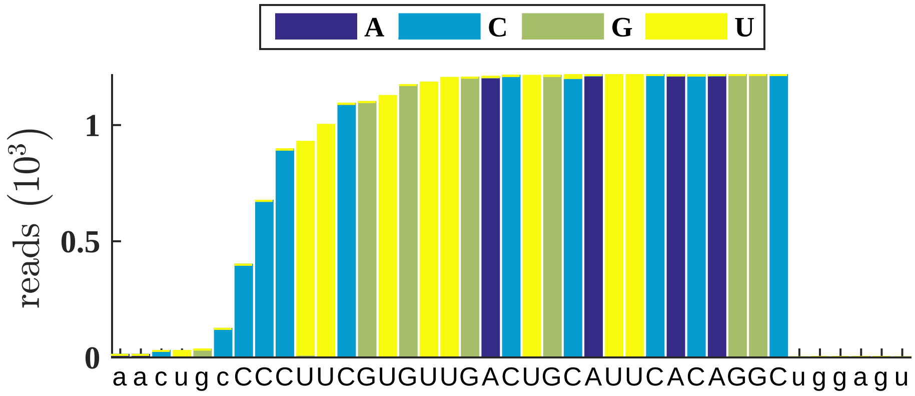

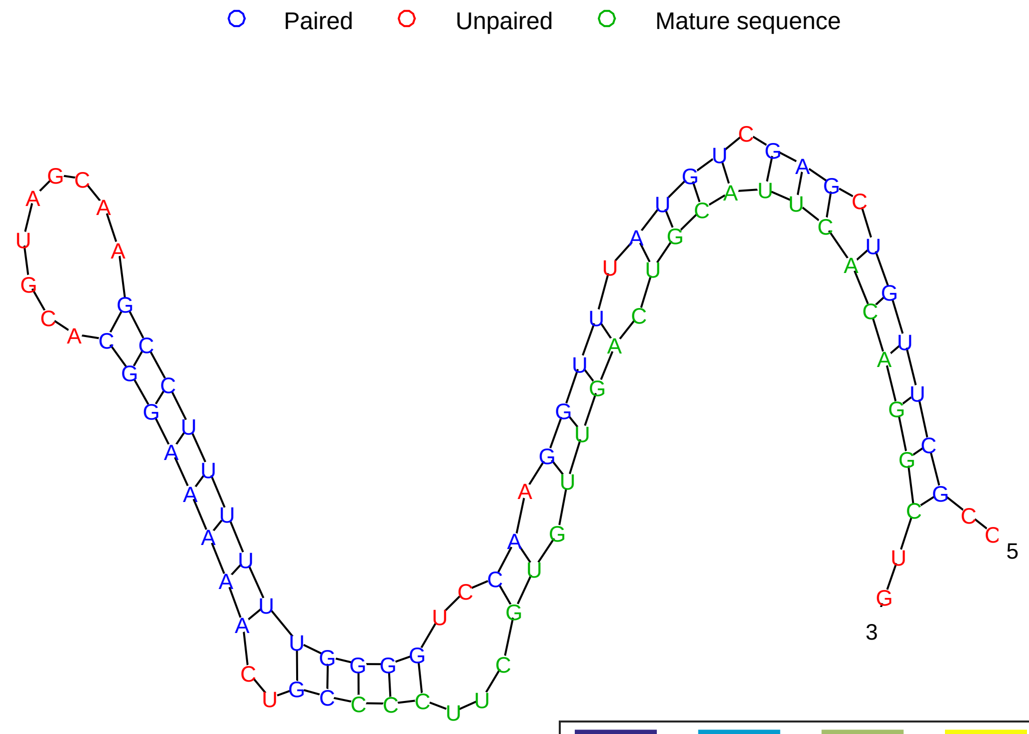

Stem loop (UMD3.1): chr6:45763322-45763411
Mature (UMD3.1): chr6:45763324-45763350
Mature seq len: 27
Total raw counts (9 samples): 1188
Average raw counts: 132
Strand: Reverse
Orientation: 3p
Minimum free energy: -28.90

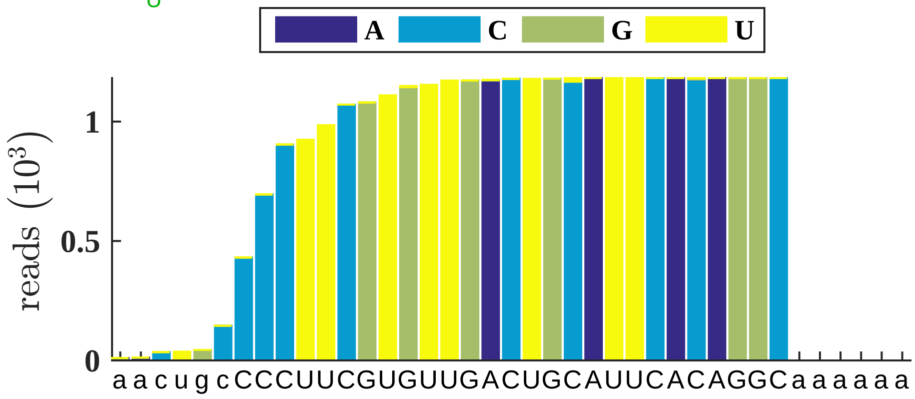

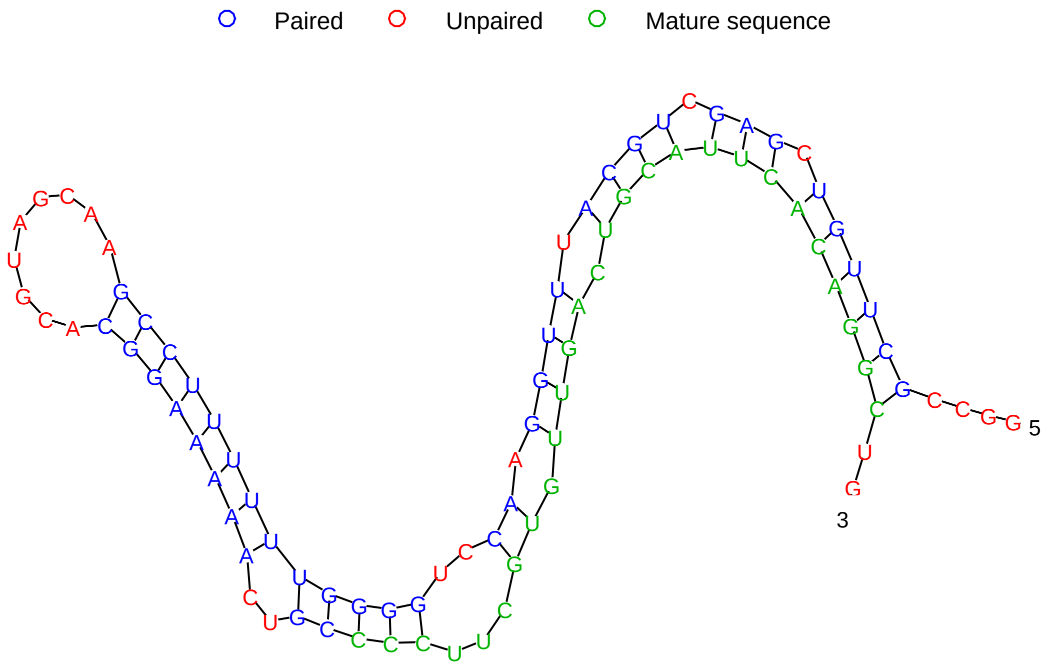

Stem loop (UMD3.1): chr6:45766331-45766422
Mature (UMD3.1): chr6:45766333-45766359
Mature seq len: 27
Total raw counts (9 samples): 1183
Average raw counts: 132
Strand: Reverse
Orientation: 3p
Minimum free energy: -30.90

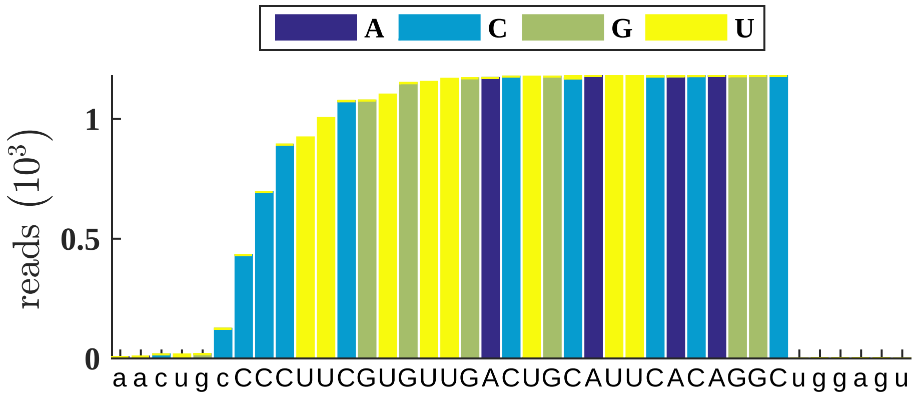

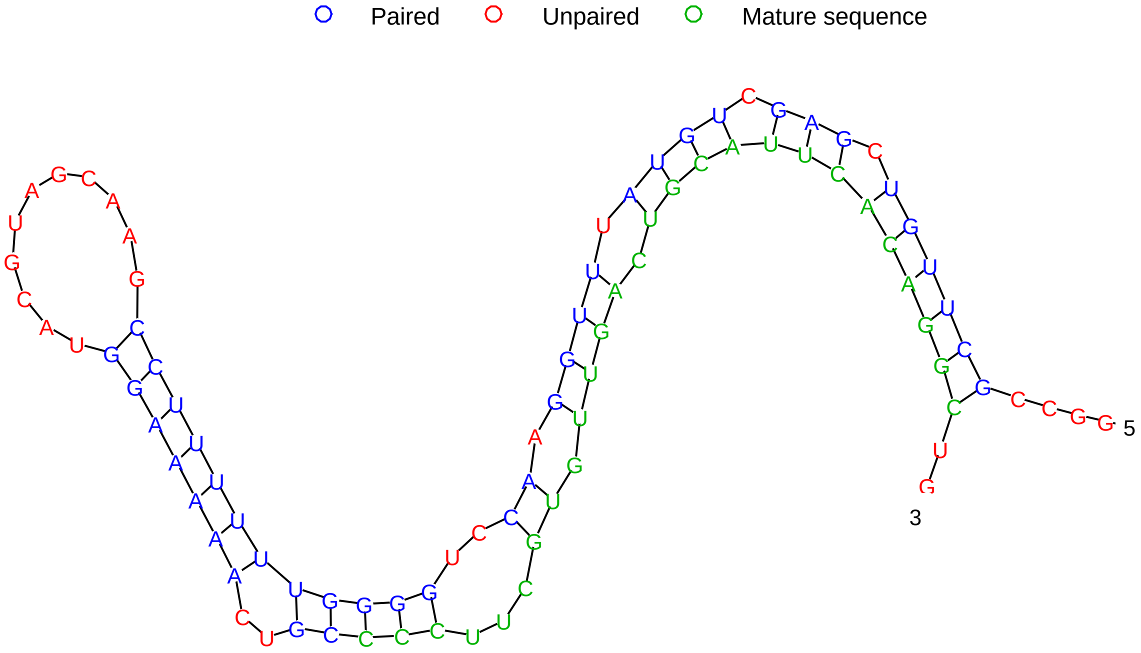

Stem loop (UMD3.1): chr6:45766740-45766831
Mature (UMD3.1): chr6:45766742-45766768
Mature seq len: 27
Total raw counts (9 samples): 1179
Average raw counts: 131
Strand: Reverse
Orientation: 3p
Minimum free energy: -26.70

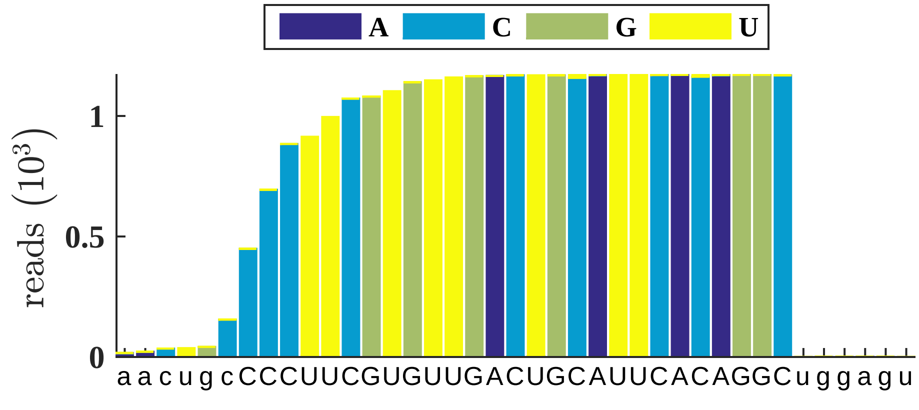

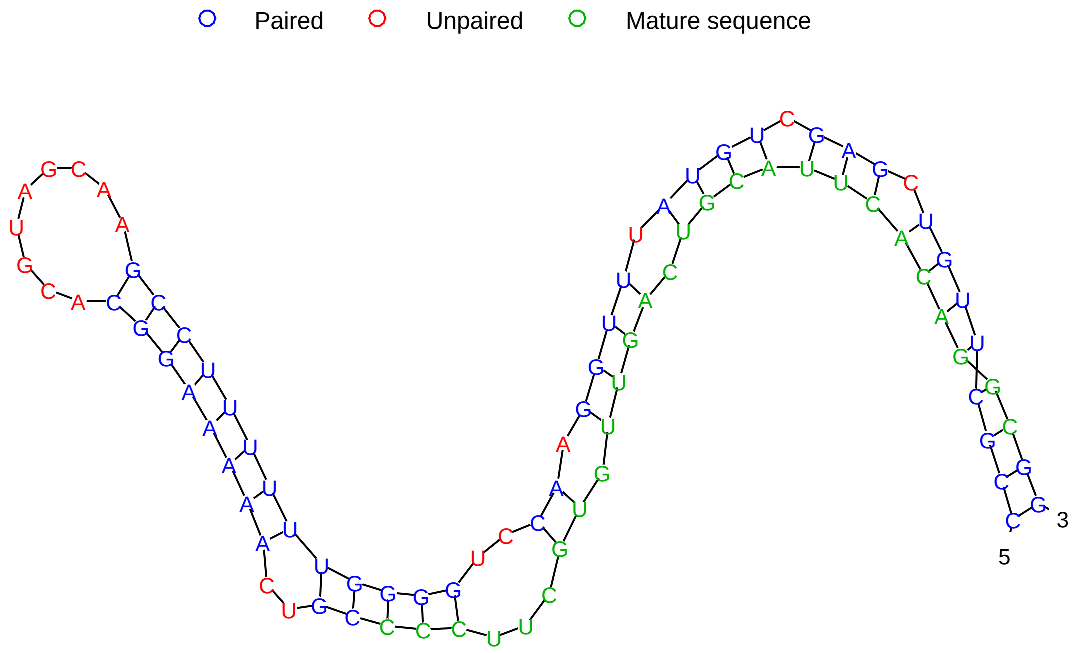

Stem loop (UMD3.1): chr6:7001731-7001820
Mature (UMD3.1): chr6:7001792-7001818
Mature seq len: 27
Total raw counts (9 samples): 1190
Average raw counts: 133
Strand: Forward
Orientation: 3p
Minimum free energy: -33.20

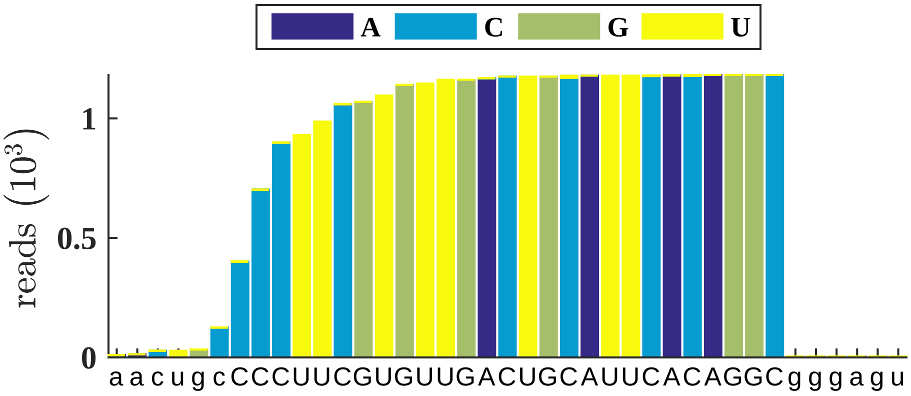

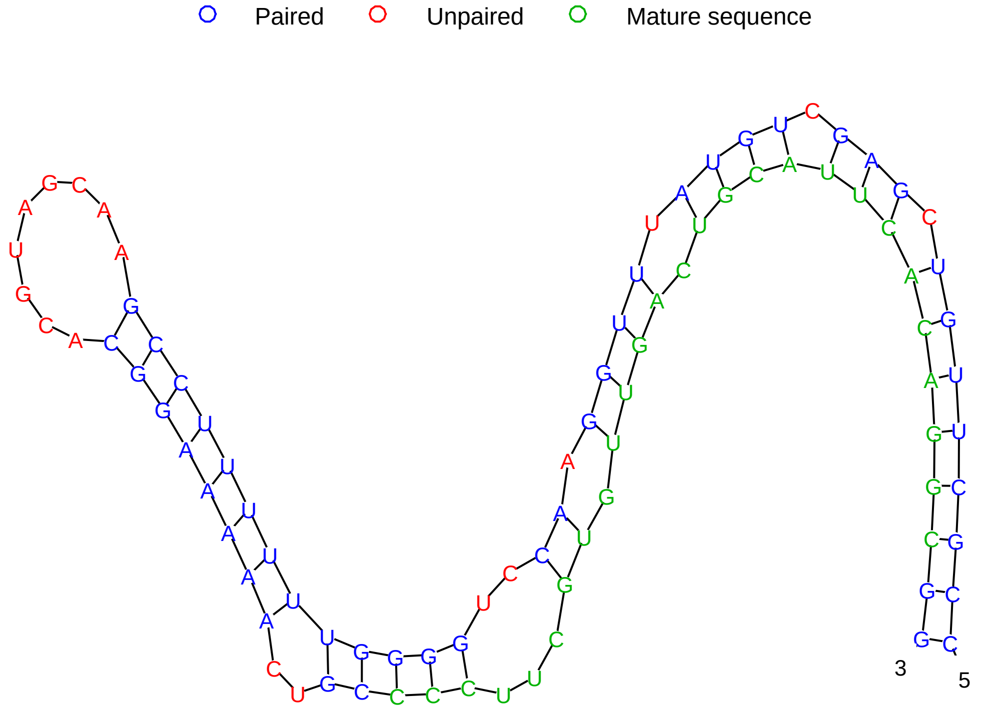

Stem loop (UMD3.1): chr6:7003109-7003198
Mature (UMD3.1): chr6:7003170-7003196
Mature seq len: 27
Total raw counts (9 samples): 1171
Average raw counts: 131
Strand: Forward
Orientation: 3p
Minimum free energy: -33.20

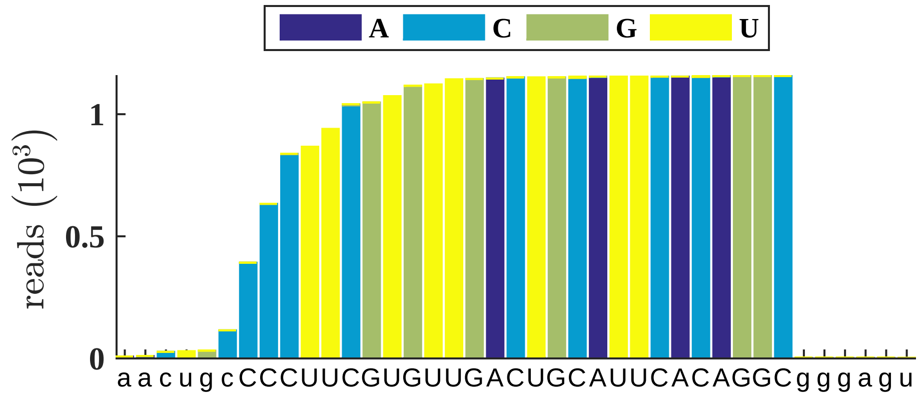

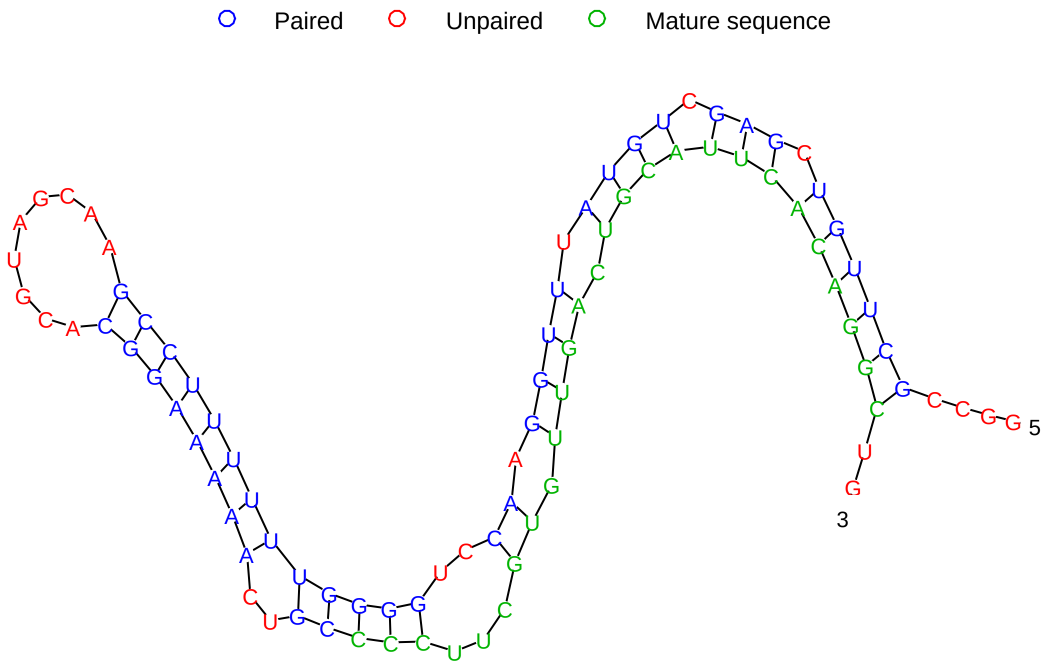

Stem loop (UMD3.1): chr6:7029065-7029156
Mature (UMD3.1): chr6:7029128-7029154
Mature seq len: 27
Total raw counts (9 samples): 1203
Average raw counts: 134
Strand: Forward
Orientation: 3p
Minimum free energy: -28.90

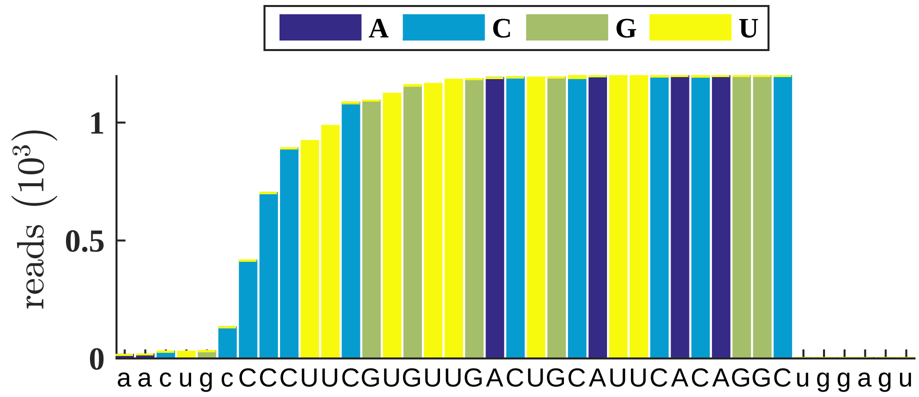

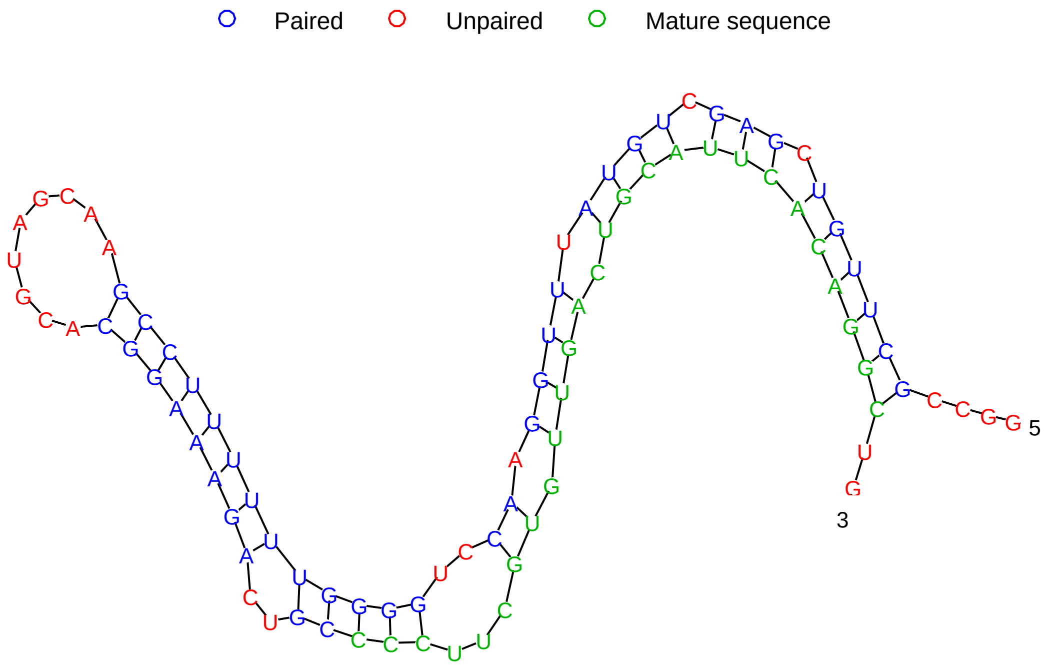

Stem loop (UMD3.1): chr6:7034782-7034873
Mature (UMD3.1): chr6:7034845-7034871
Mature seq len: 27
Total raw counts (9 samples): 1256
Average raw counts: 140
Strand: Forward
Orientation: 3p
Minimum free energy: -29.00

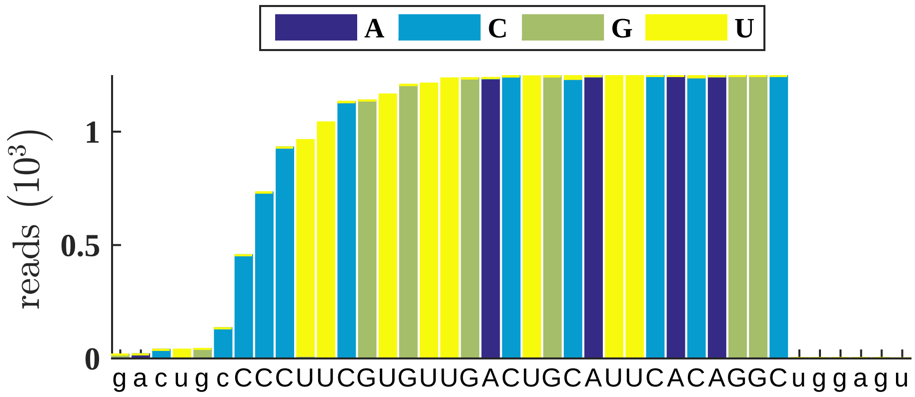

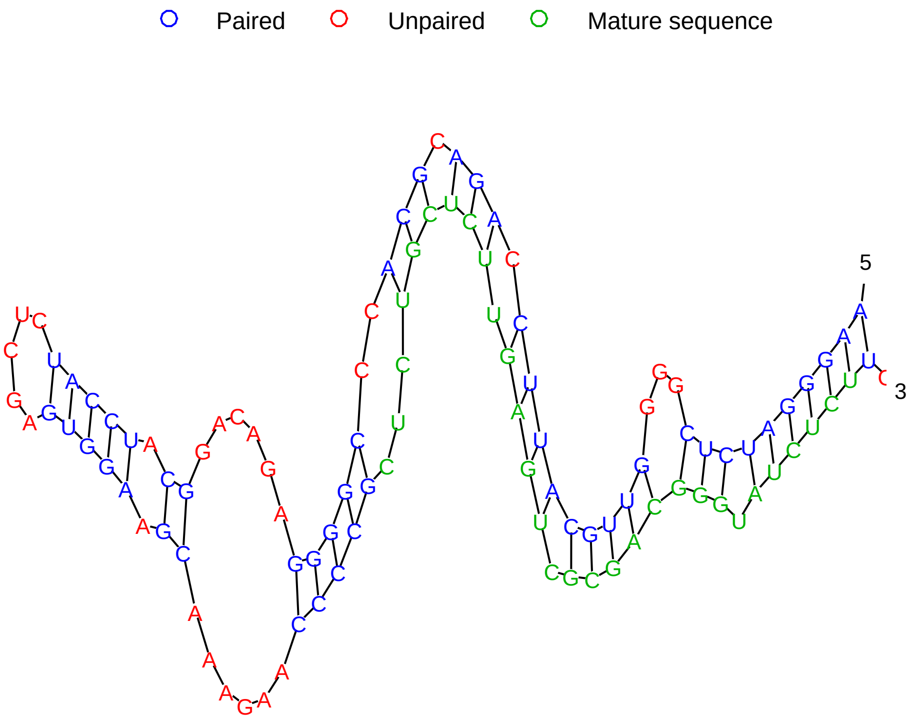

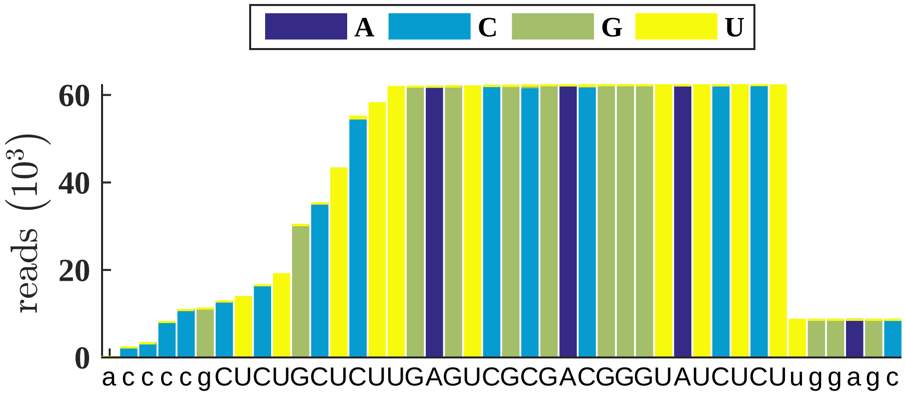

Stem loop (UMD3.1): chr6:89208035-89208141
Mature (UMD3.1): chr6:89208037-89208066
Mature seq len: 30
Total raw counts (9 samples): 62266
Average raw counts: 6919
Strand: Reverse
Orientation: 3p
Minimum free energy: -40.40

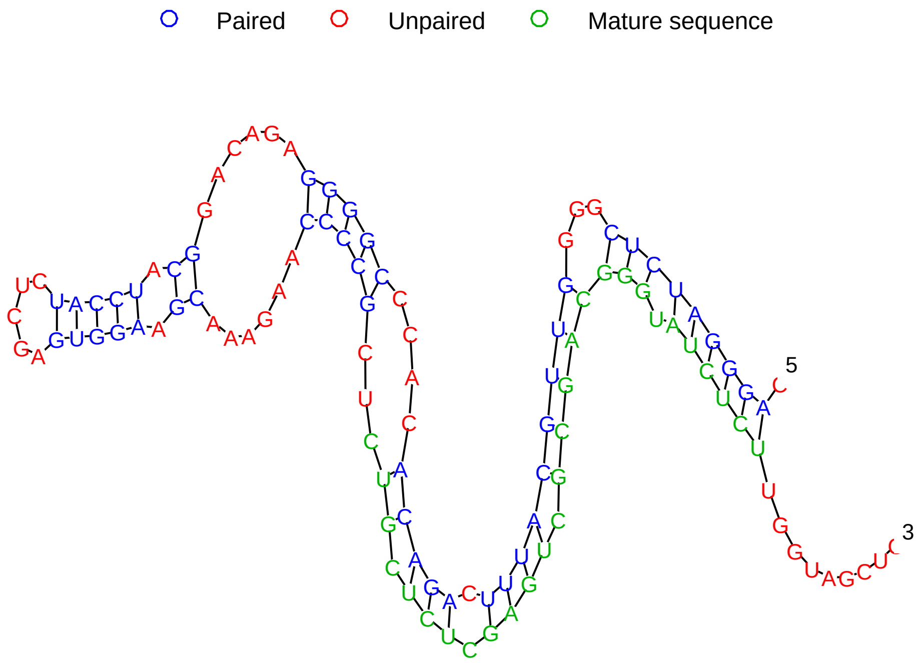

Stem loop (UMD3.1): chr6:89213601-89213714
Mature (UMD3.1): chr6:89213610-89213637
Mature seq len: 28
Total raw counts (9 samples): 12217
Average raw counts: 1358
Strand: Reverse
Orientation: 3p
Minimum free energy: -34.20

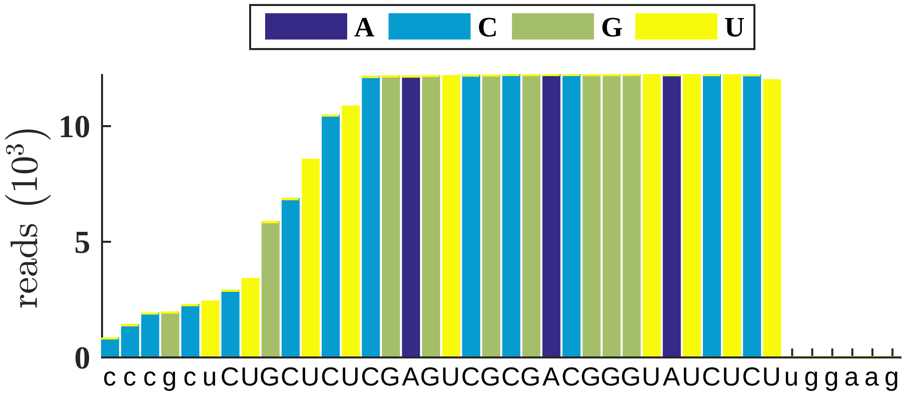

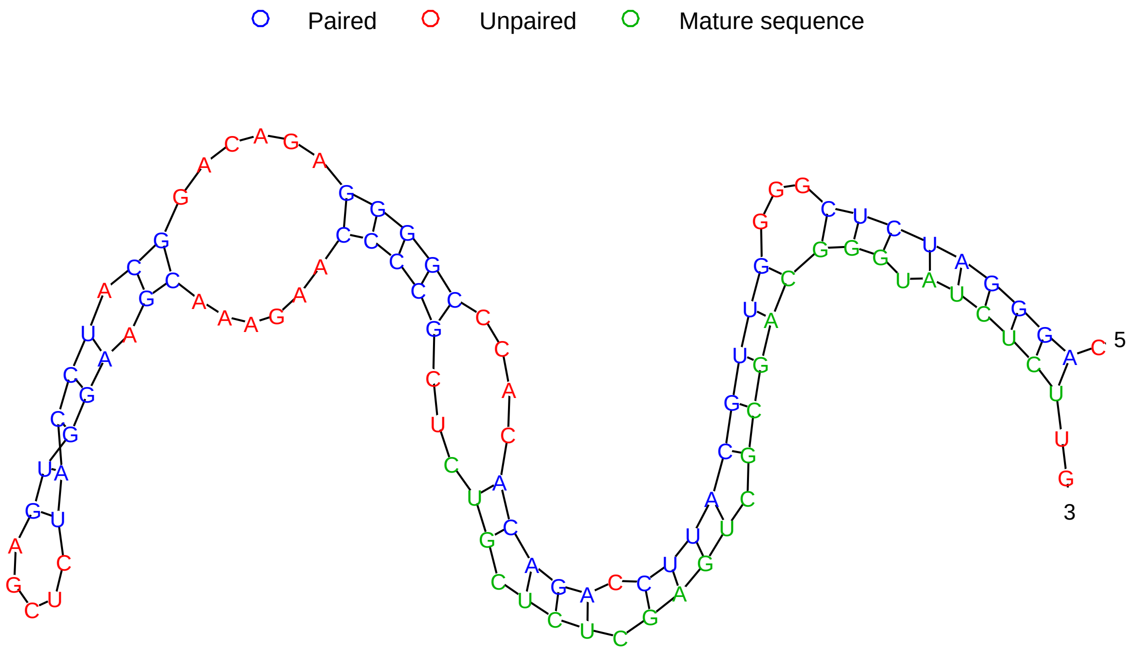

Stem loop (UMD3.1): chr6:89214660-89214766
Mature (UMD3.1): chr6:89214662-89214689
Mature seq len: 28
Total raw counts (9 samples): 12008
Average raw counts: 1335
Strand: Reverse
Orientation: 3p
Minimum free energy: -36.00

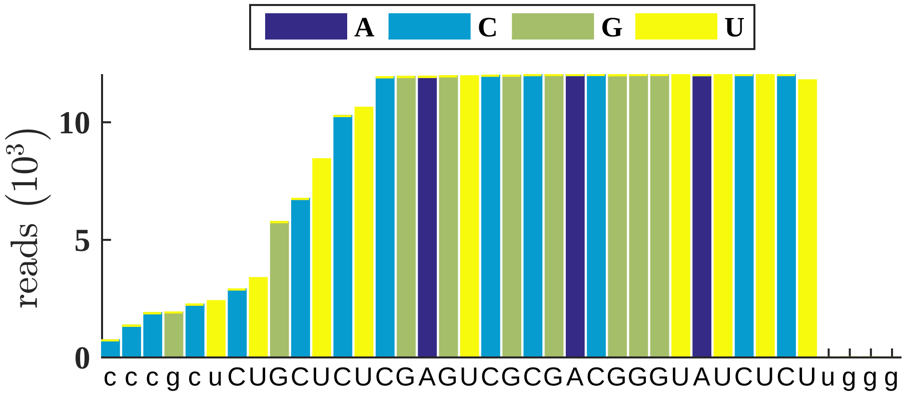

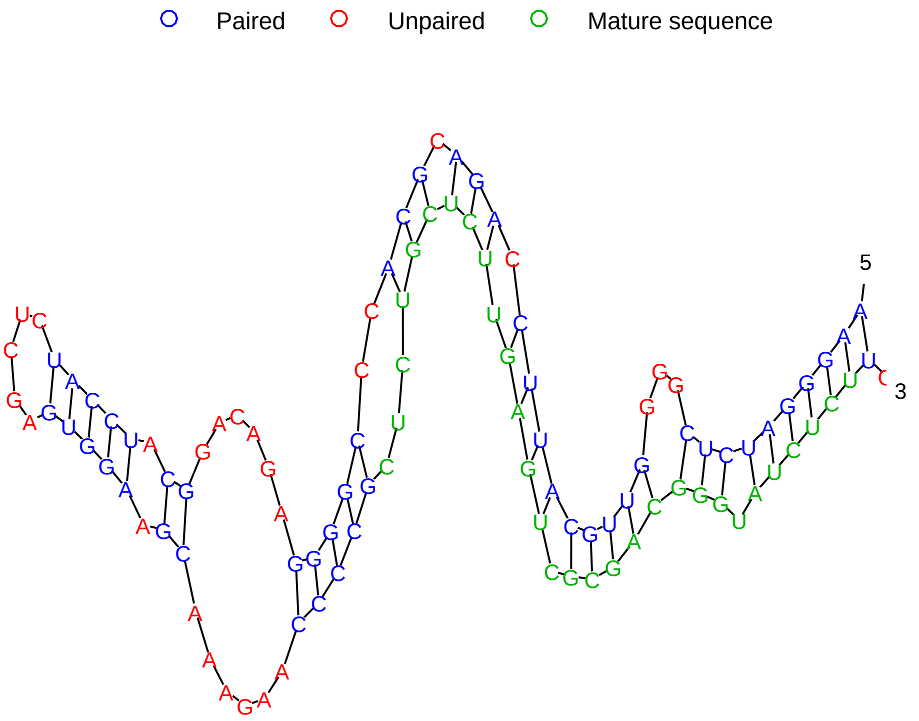

Stem loop (UMD3.1): chr6:89217310-89217416
Mature (UMD3.1): chr6:89217312-89217341
Mature seq len: 30
Total raw counts (9 samples): 62718
Average raw counts: 6969
Strand: Reverse
Orientation: 3p
Minimum free energy: -40.40

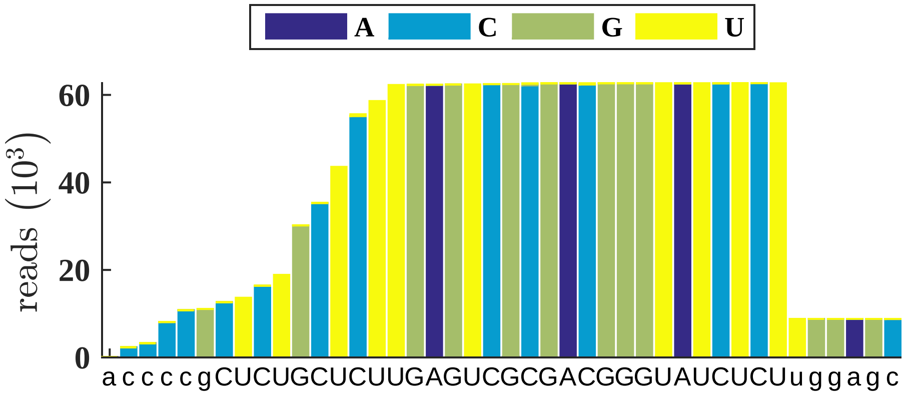

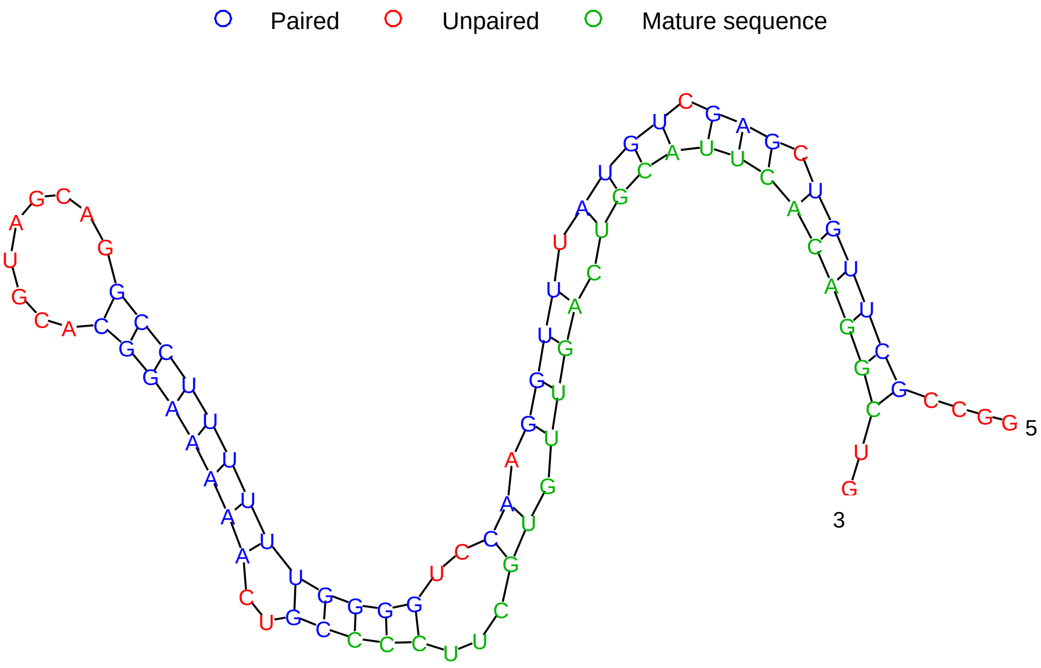

Stem loop (UMD3.1): chr7:110027422-110027513
Mature (UMD3.1): chr7:110027424-110027450
Mature seq len: 27
Total raw counts (9 samples): 1228
Average raw counts: 137
Strand: Reverse
Orientation: 3p
Minimum free energy: -30.30

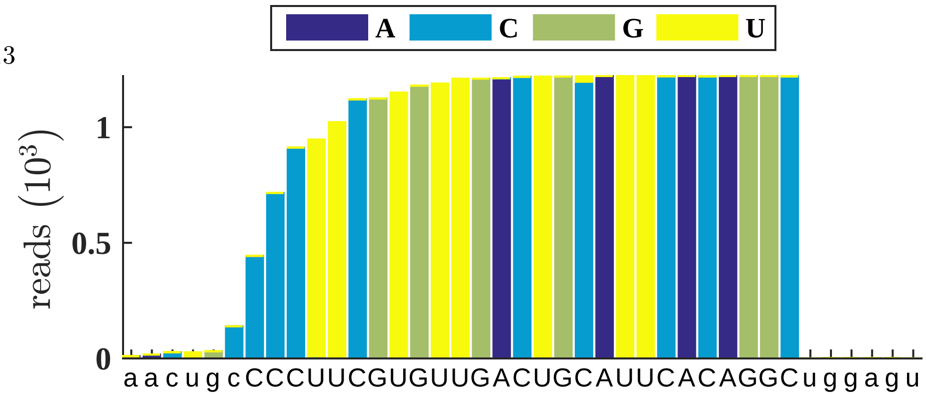

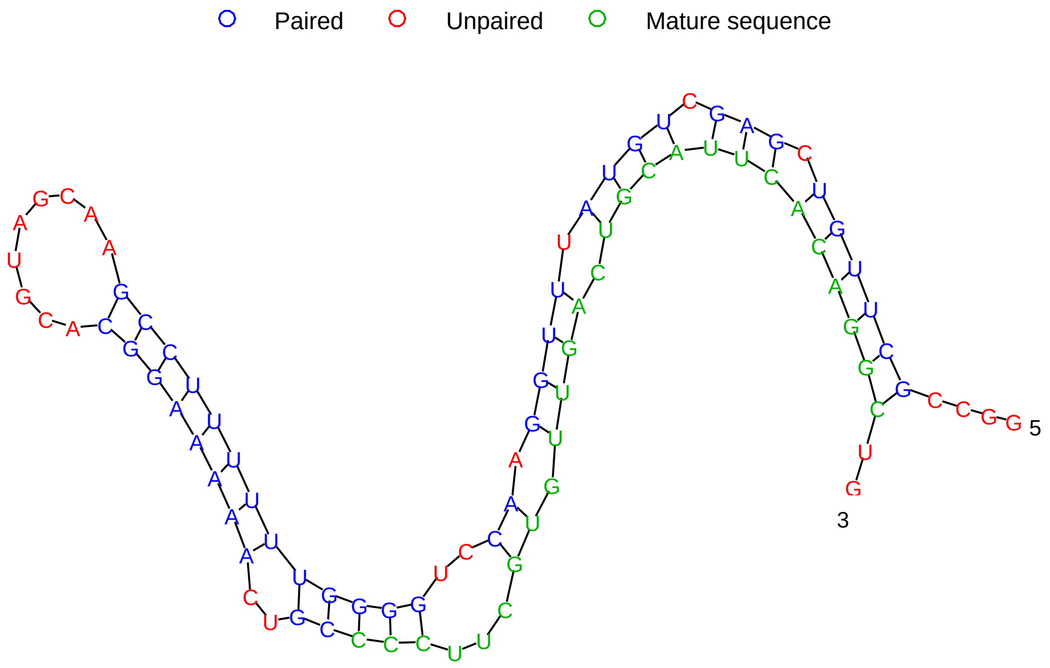

Stem loop (UMD3.1): chr7:110032744-110032835
Mature (UMD3.1): chr7:110032807-110032833
Mature seq len: 27
Total raw counts (9 samples): 1196
Average raw counts: 133
Strand: Forward
Orientation: 3p
Minimum free energy: -28.90

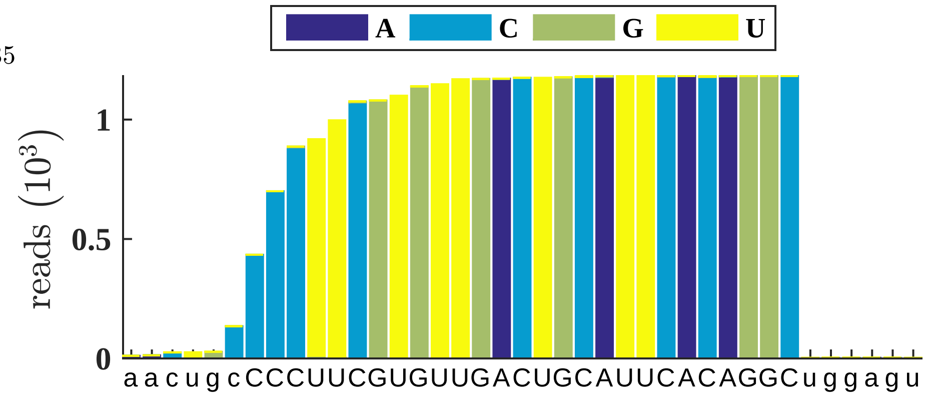

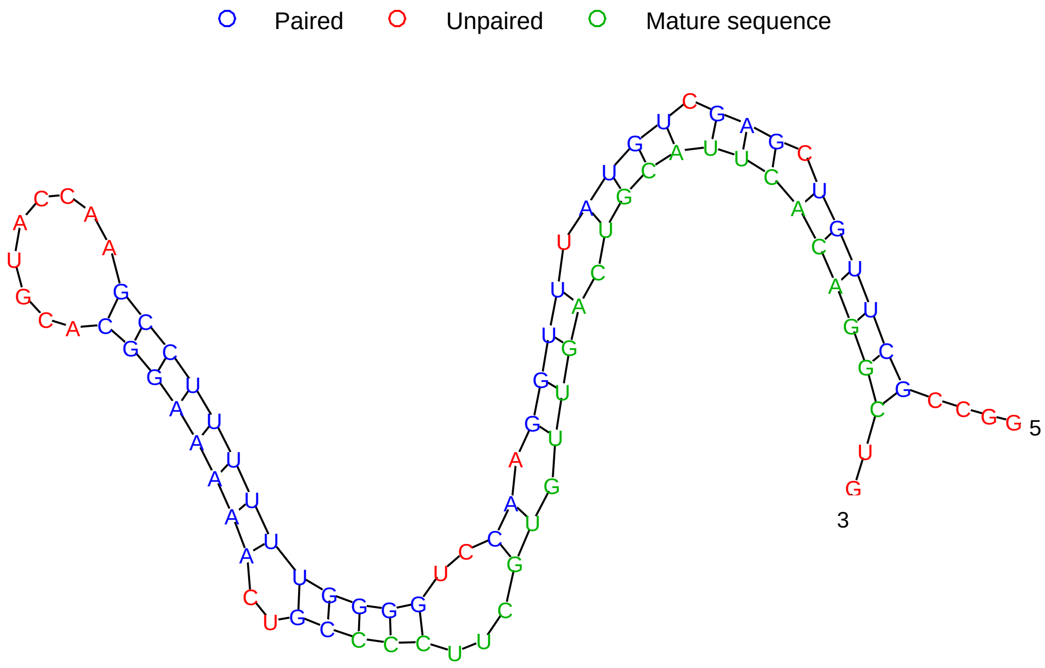

Stem loop (UMD3.1): chr7:12096551-12096642
Mature (UMD3.1): chr7:12096614-12096640
Mature seq len: 27
Total raw counts (9 samples): 1171
Average raw counts: 131
Strand: Forward
Orientation: 3p
Minimum free energy: -28.90

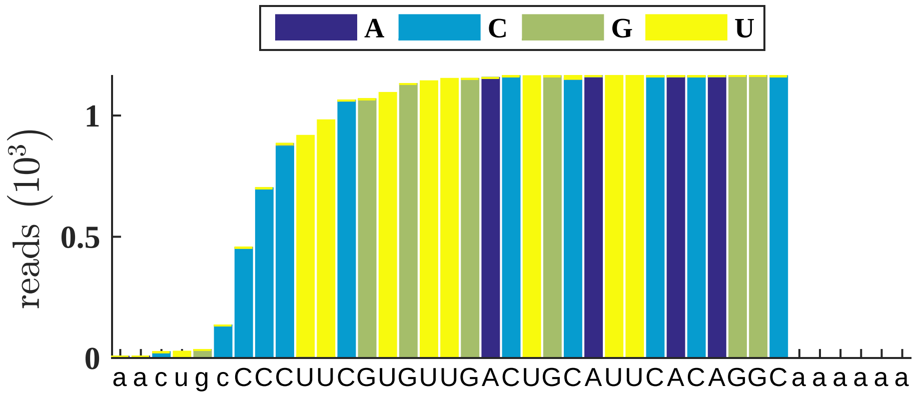

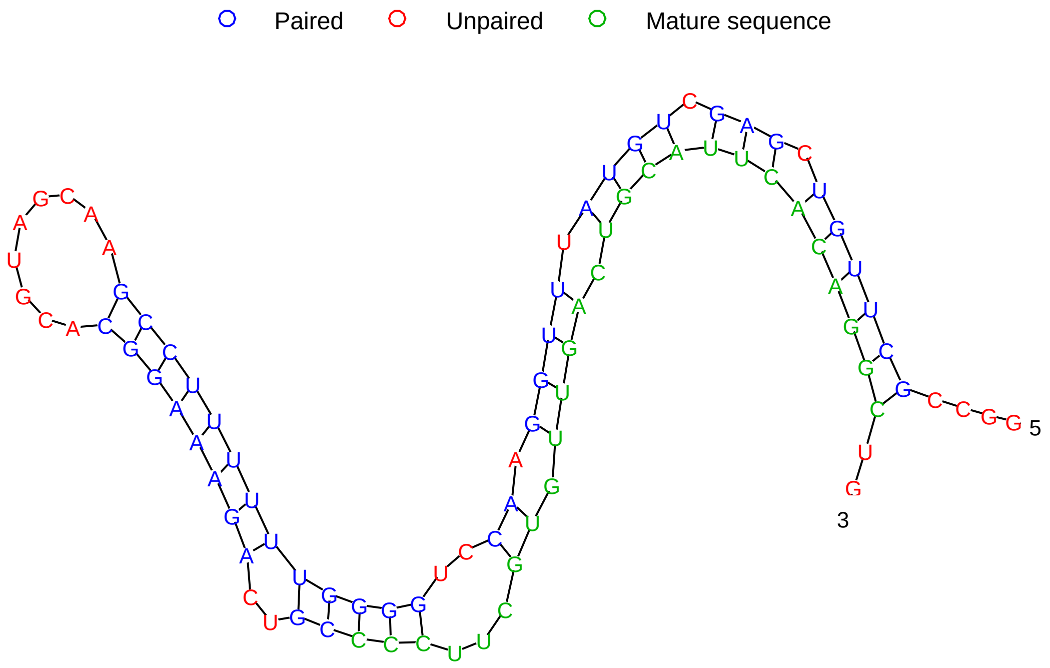

Stem loop (UMD3.1): chr7:12102970-12103061
Mature (UMD3.1): chr7:12103033-12103059
Mature seq len: 27
Total raw counts (9 samples): 1282
Average raw counts: 143
Strand: Forward
Orientation: 3p
Minimum free energy: -29.00

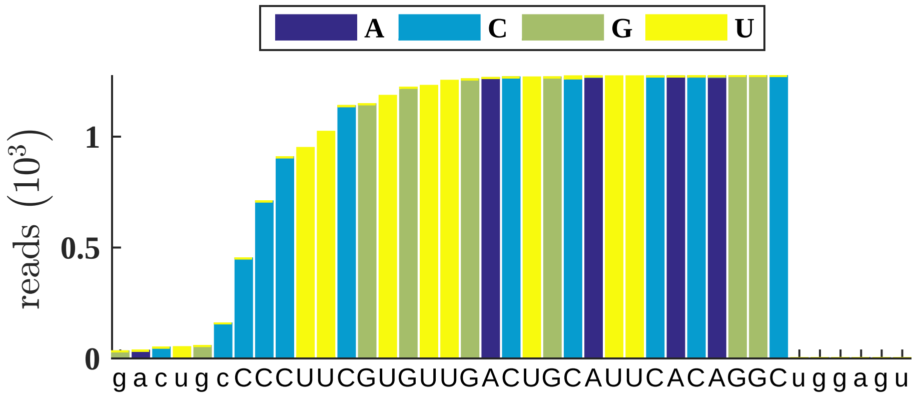

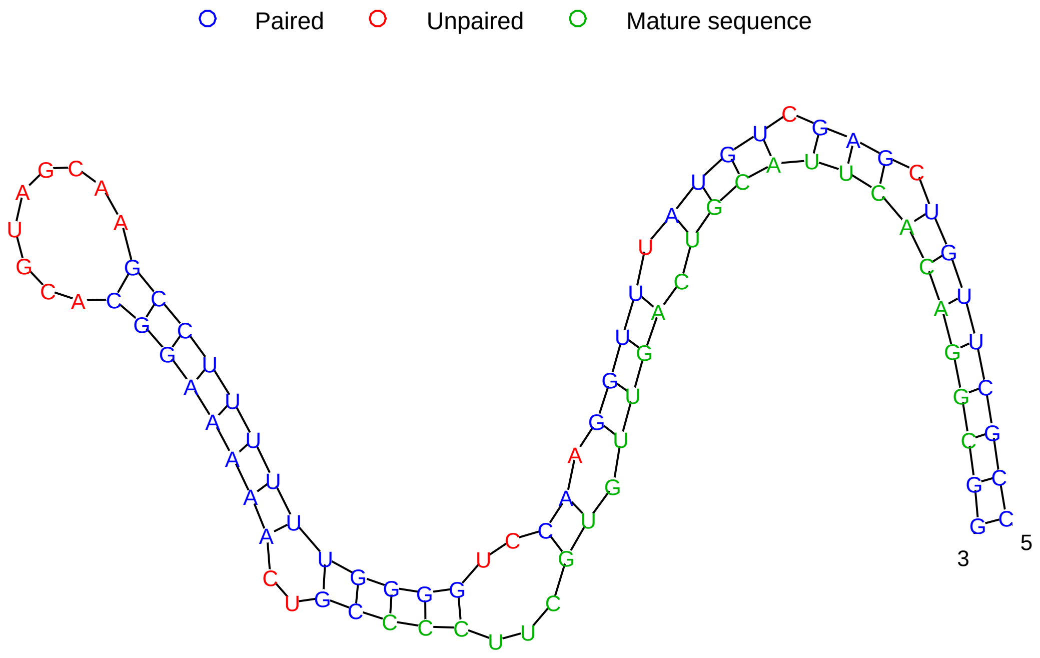

Stem loop (UMD3.1): chr7:12121494-12121583
Mature (UMD3.1): chr7:12121496-12121522
Mature seq len: 27
Total raw counts (9 samples): 1169
Average raw counts: 130
Strand: Reverse
Orientation: 3p
Minimum free energy: -33.20

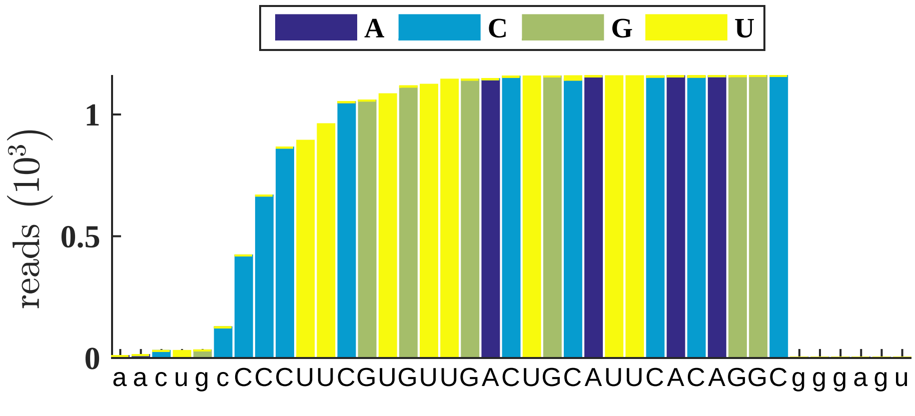

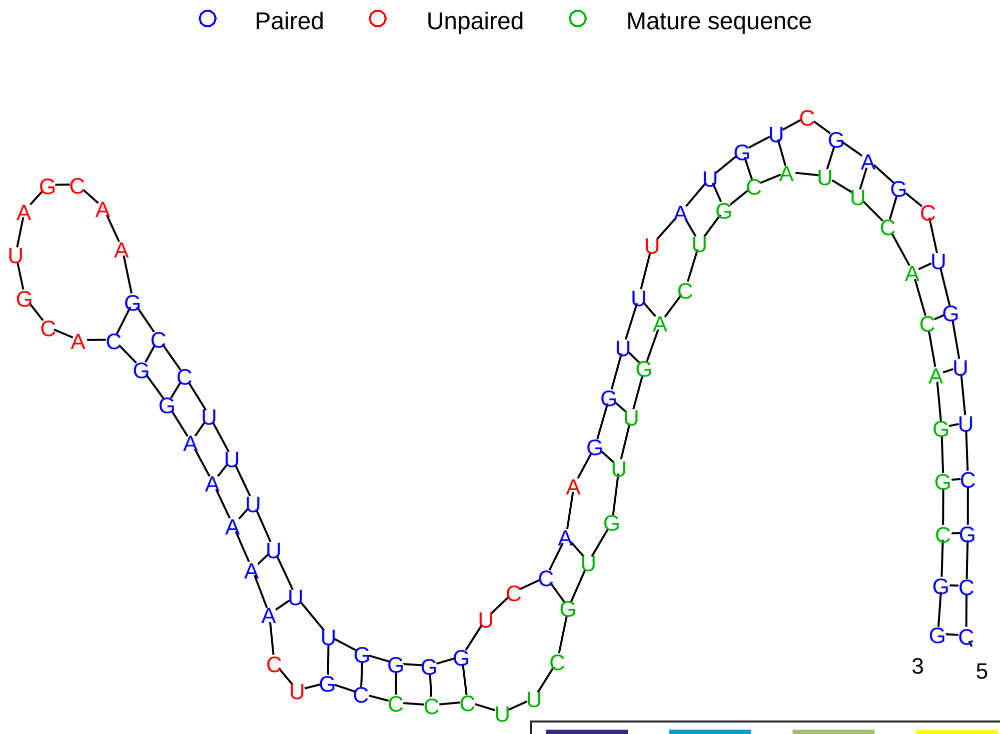

Stem loop (UMD3.1): chr7:12140867-12140956
Mature (UMD3.1): chr7:12140928-12140954
Mature seq len: 27
Total raw counts (9 samples): 1187
Average raw counts: 132
Strand: Forward
Orientation: 3p
Minimum free energy: -33.20

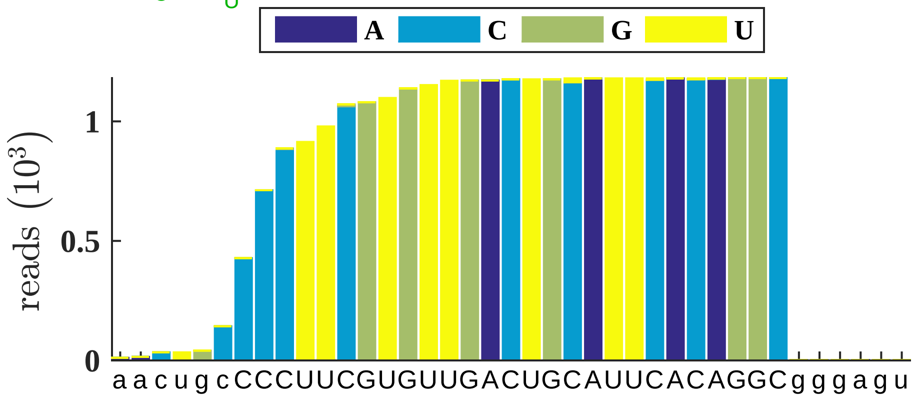

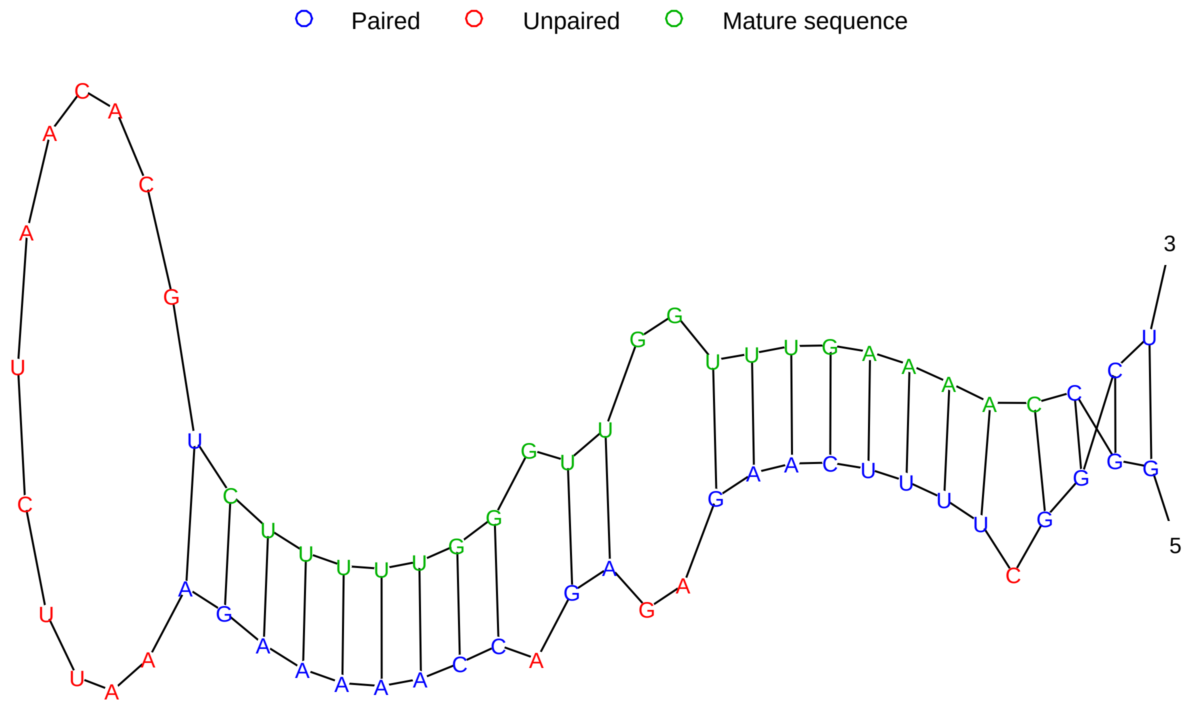

Stem loop (UMD3.1): chr7:20756728-20756792
Mature (UMD3.1): chr7:20756731-20756752
Mature seq len: 22
Total raw counts (9 samples): 624
Average raw counts: 70
Strand: Forward
Orientation: 5p
Minimum free energy: -23.30

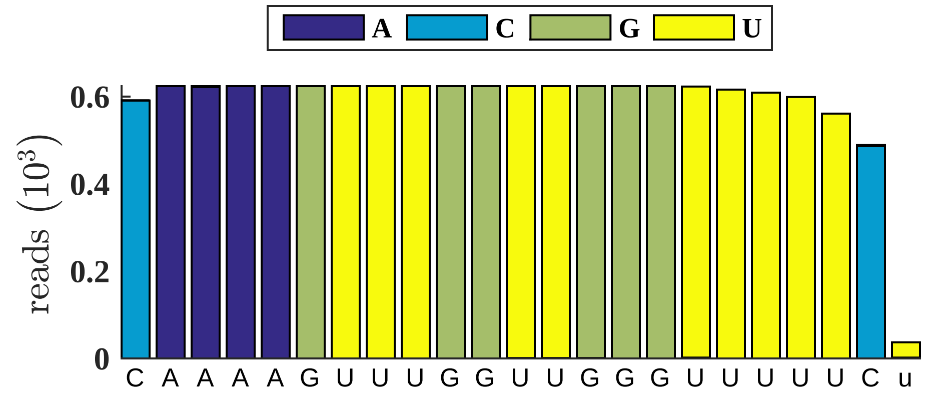

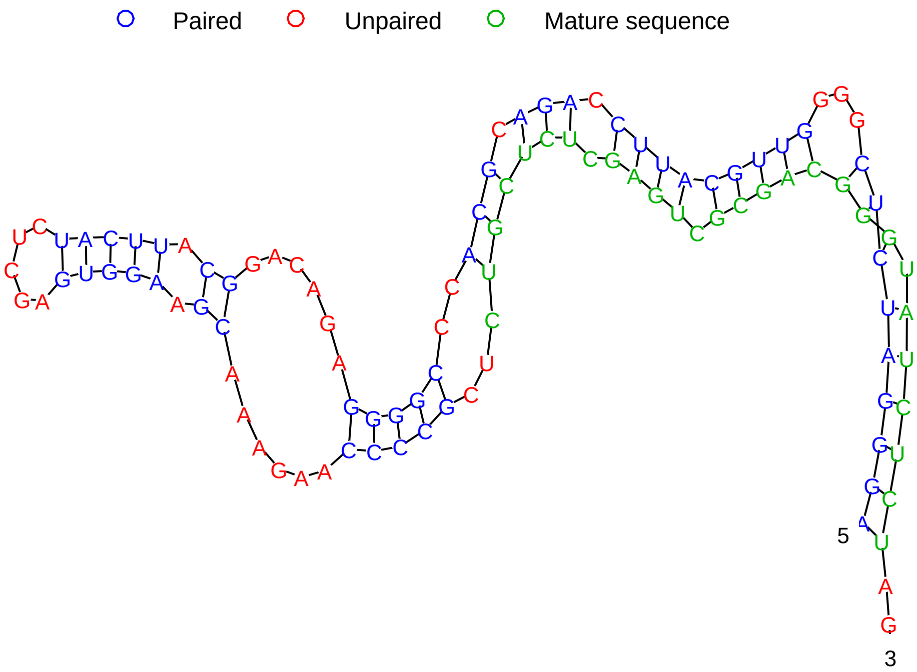

Stem loop (UMD3.1): chr7:41617338-41617443
Mature (UMD3.1): chr7:41617414-41617441
Mature seq len: 28
Total raw counts (9 samples): 13320
Average raw counts: 1480
Strand: Forward
Orientation: 3p
Minimum free energy: -36.60

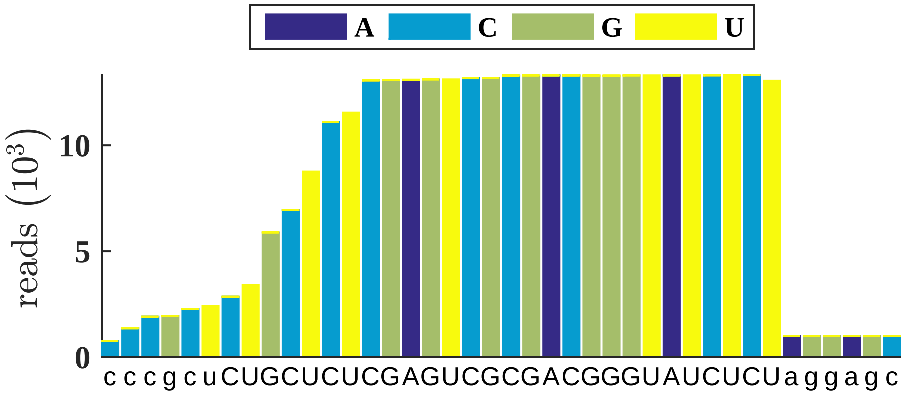

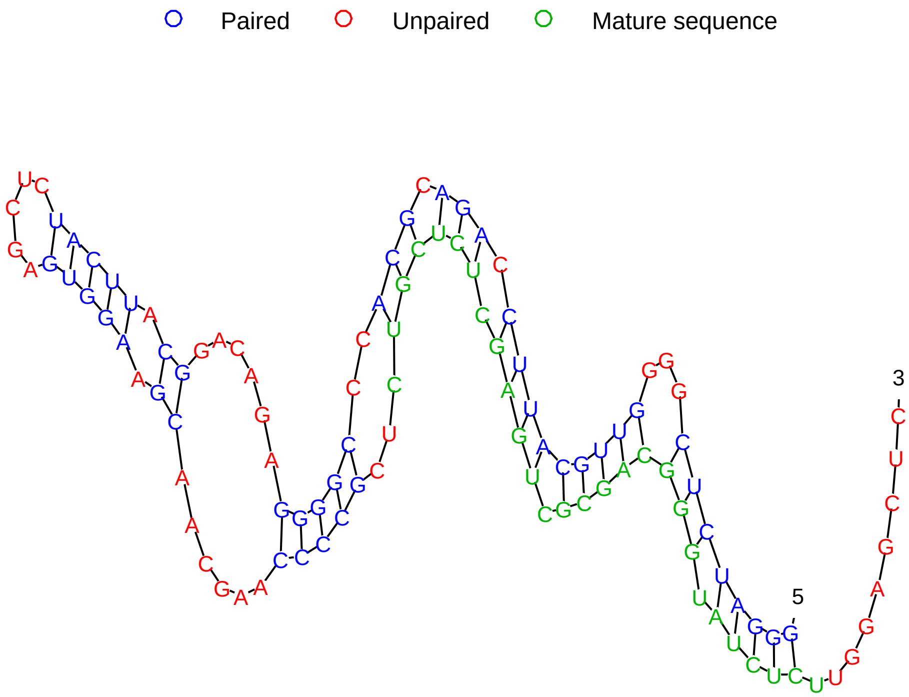

Stem loop (UMD3.1): chr7:41620714-41620824
Mature (UMD3.1): chr7:41620789-41620816
Mature seq len: 28
Total raw counts (9 samples): 12986
Average raw counts: 1443
Strand: Forward
Orientation: 3p
Minimum free energy: -35.50

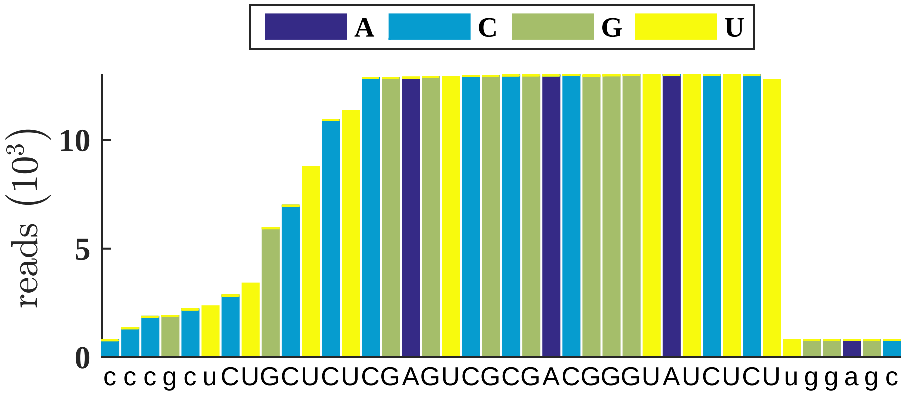

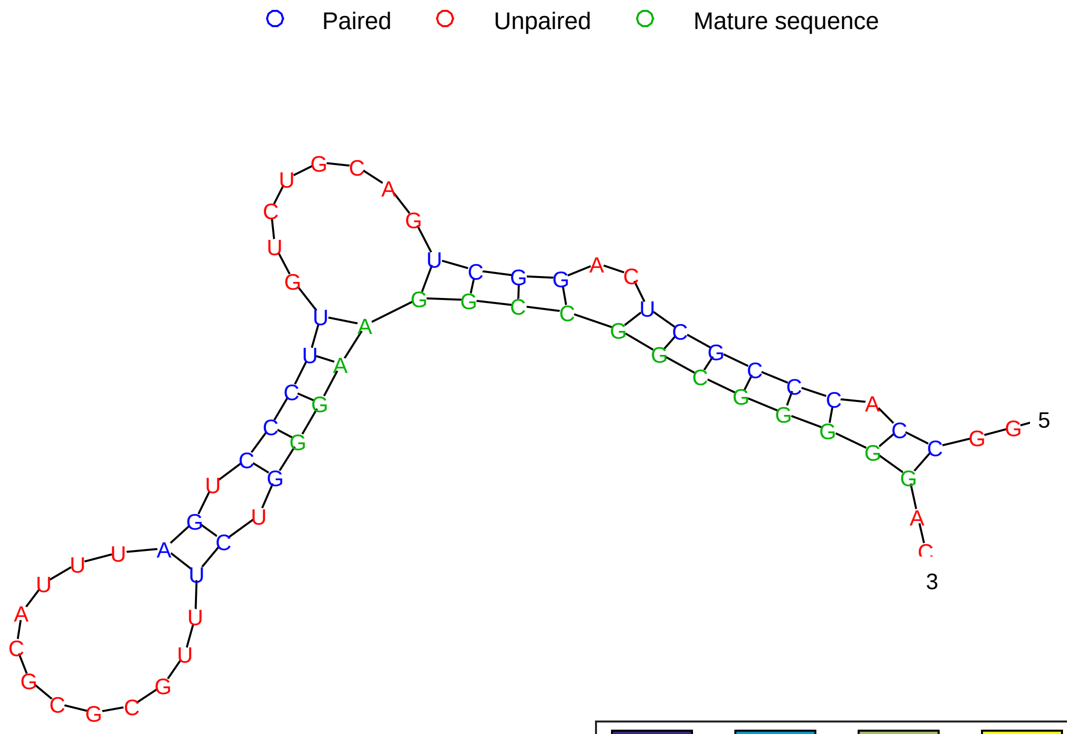

Stem loop (UMD3.1): chr7:44900584-44900650
Mature (UMD3.1): chr7:44900586-44900601
Mature seq len: 16
Total raw counts (9 samples): 1860
Average raw counts: 207
Strand: Reverse
Orientation: 3p
Minimum free energy: -24.80

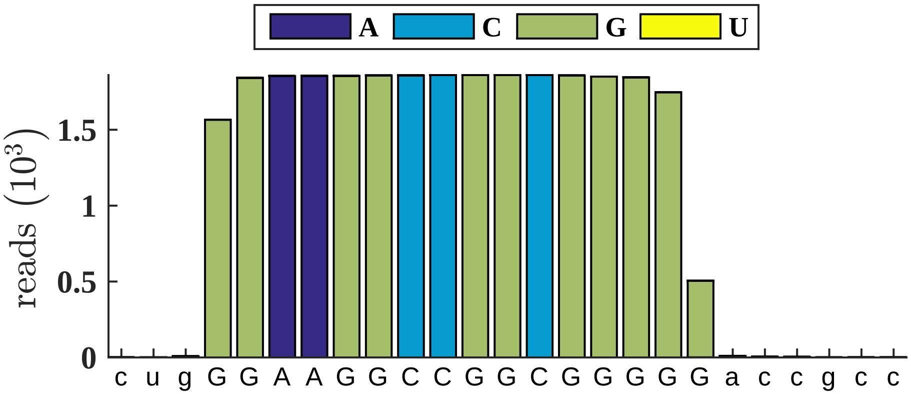

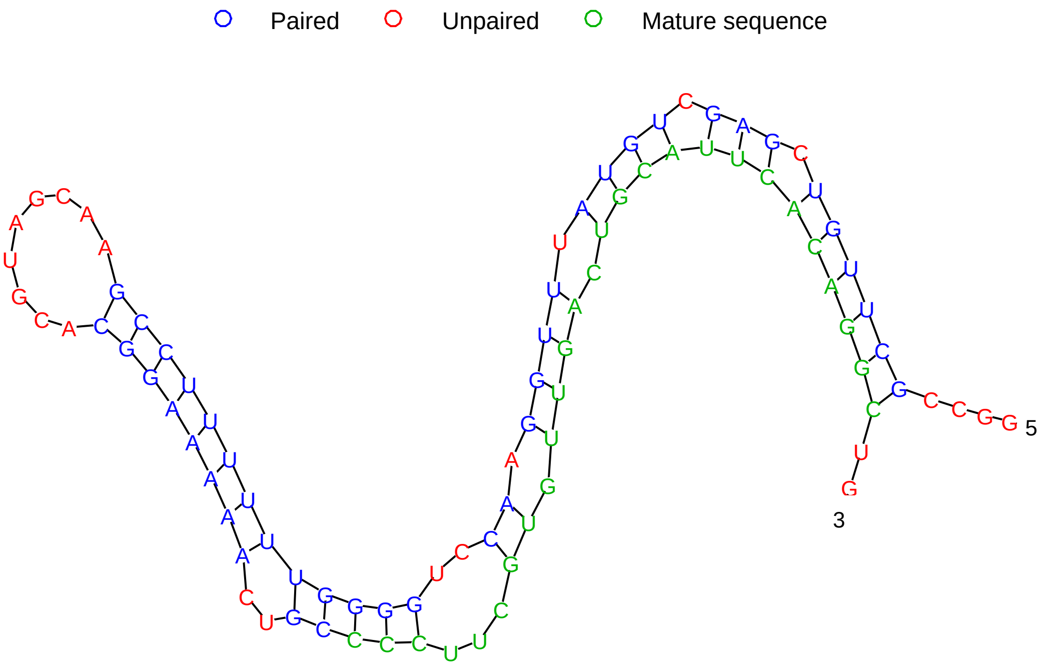

Stem loop (UMD3.1): chr7:50891266-50891357
Mature (UMD3.1): chr7:50891268-50891294
Mature seq len: 27
Total raw counts (9 samples): 1189
Average raw counts: 133
Strand: Reverse
Orientation: 3p
Minimum free energy: -28.90

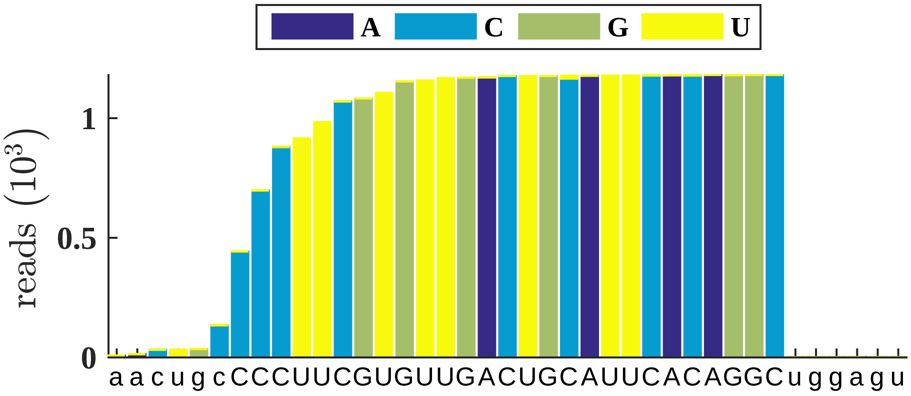

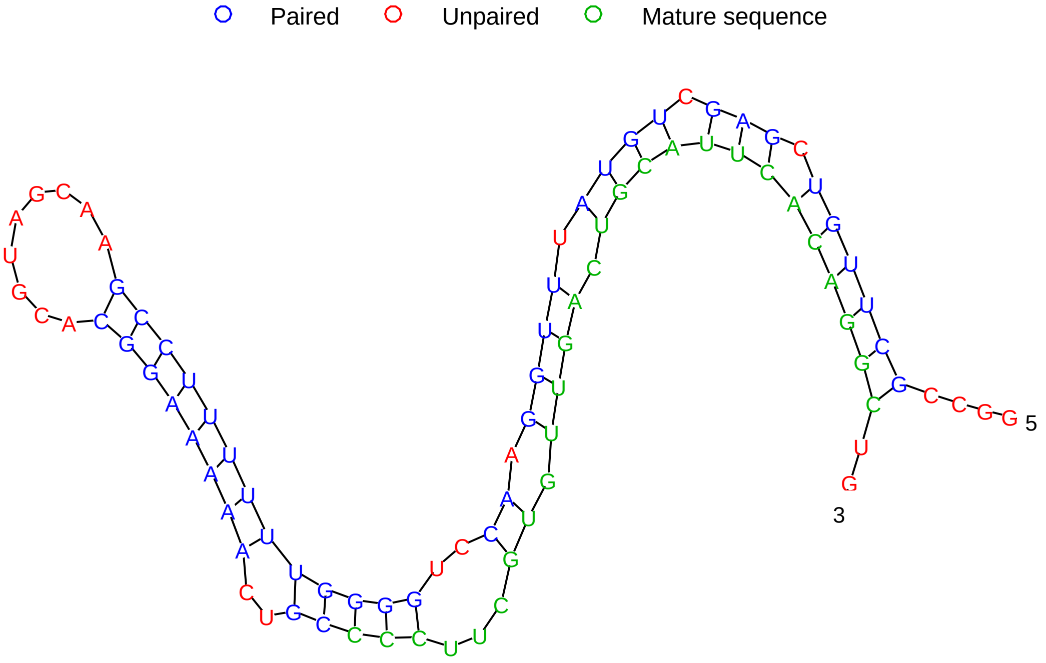

Stem loop (UMD3.1): chr7:50892659-50892750
Mature (UMD3.1): chr7:50892661-50892687
Mature seq len: 27
Total raw counts (9 samples): 1199
Average raw counts: 134
Strand: Reverse
Orientation: 3p
Minimum free energy: -28.90

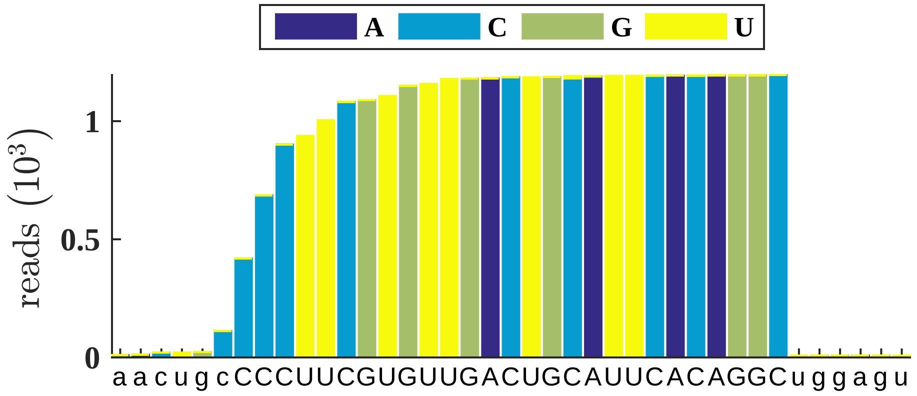

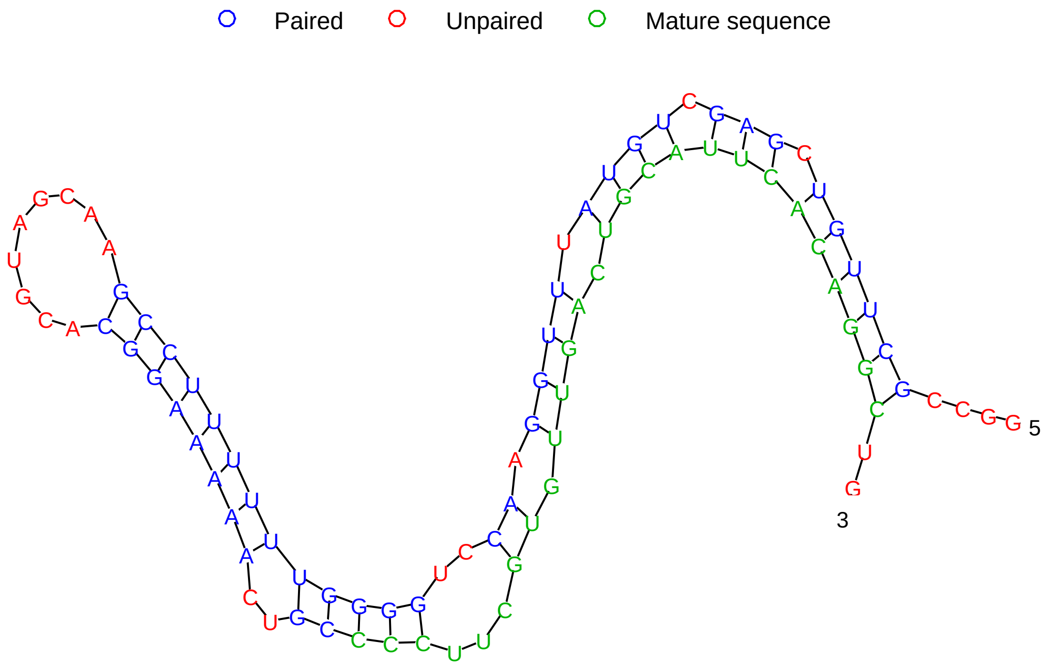

Stem loop (UMD3.1): chr7:51285425-51285516
Mature (UMD3.1): chr7:51285427-51285453
Mature seq len: 27
Total raw counts (9 samples): 1190
Average raw counts: 133
Strand: Reverse
Orientation: 3p
Minimum free energy: -28.90

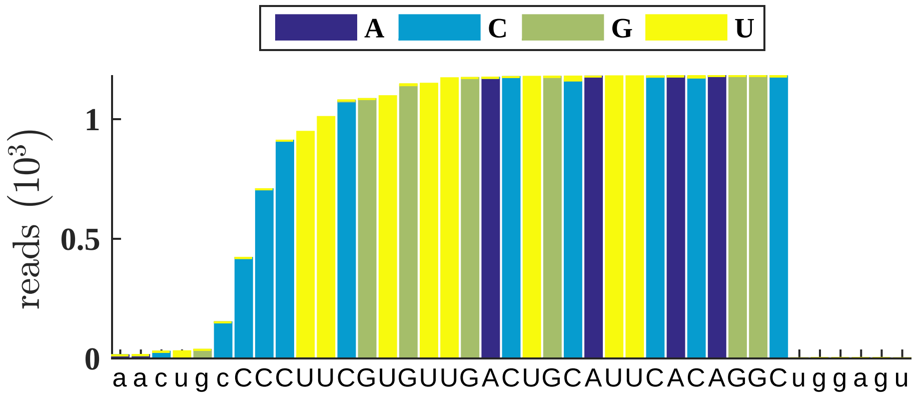

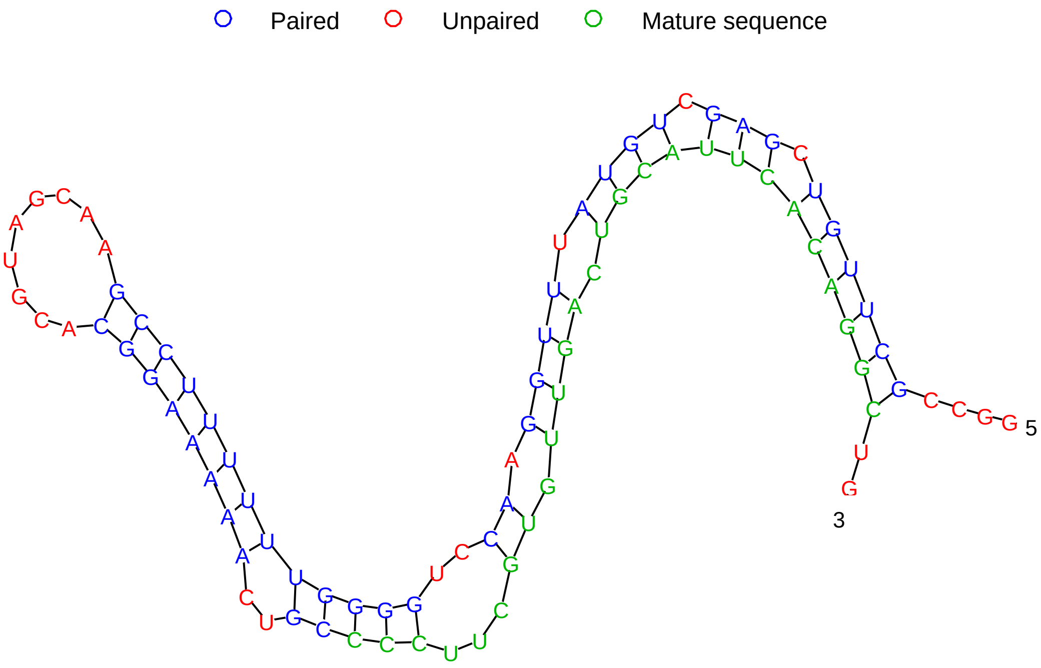

Stem loop (UMD3.1): chr7:51286824-51286915
Mature (UMD3.1): chr7:51286826-51286852
Mature seq len: 27
Total raw counts (9 samples): 1207
Average raw counts: 135
Strand: Reverse
Orientation: 3p
Minimum free energy: -28.90

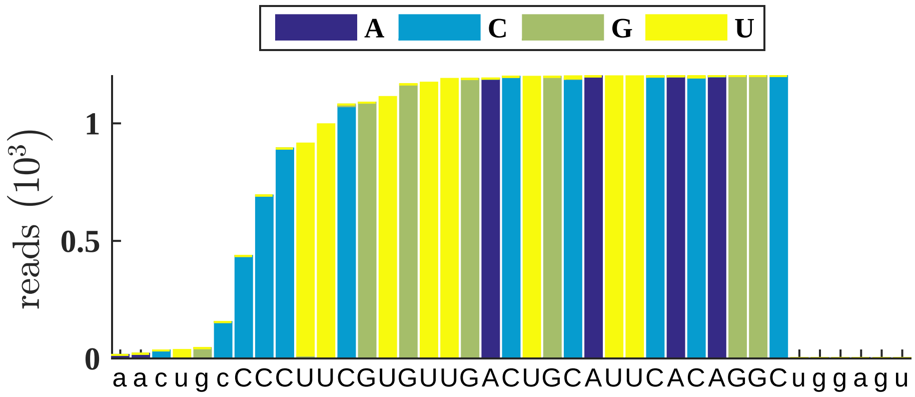

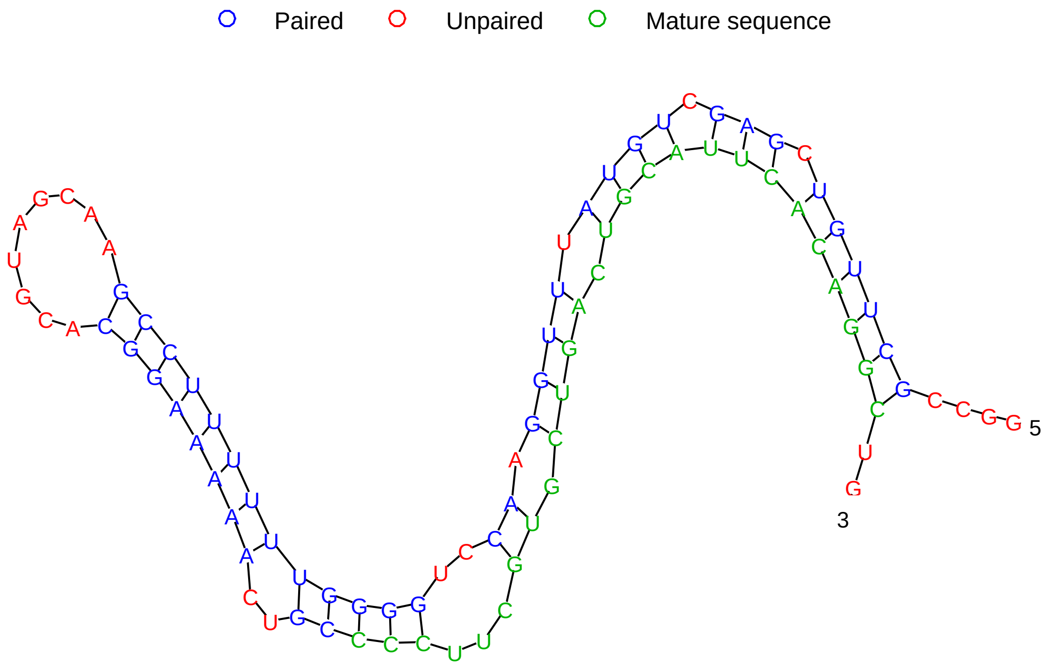

Stem loop (UMD3.1): chr7:51287993-51288084
Mature (UMD3.1): chr7:51287995-51288021
Mature seq len: 27
Total raw counts (9 samples): 1231
Average raw counts: 137
Strand: Reverse
Orientation: 3p
Minimum free energy: -31.20

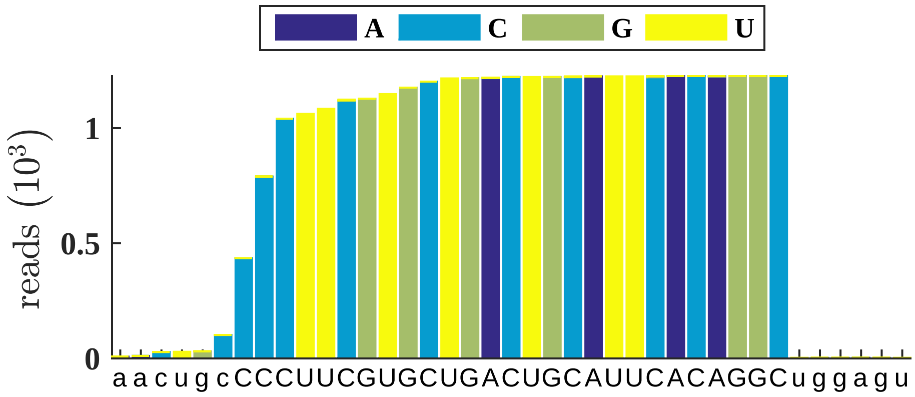

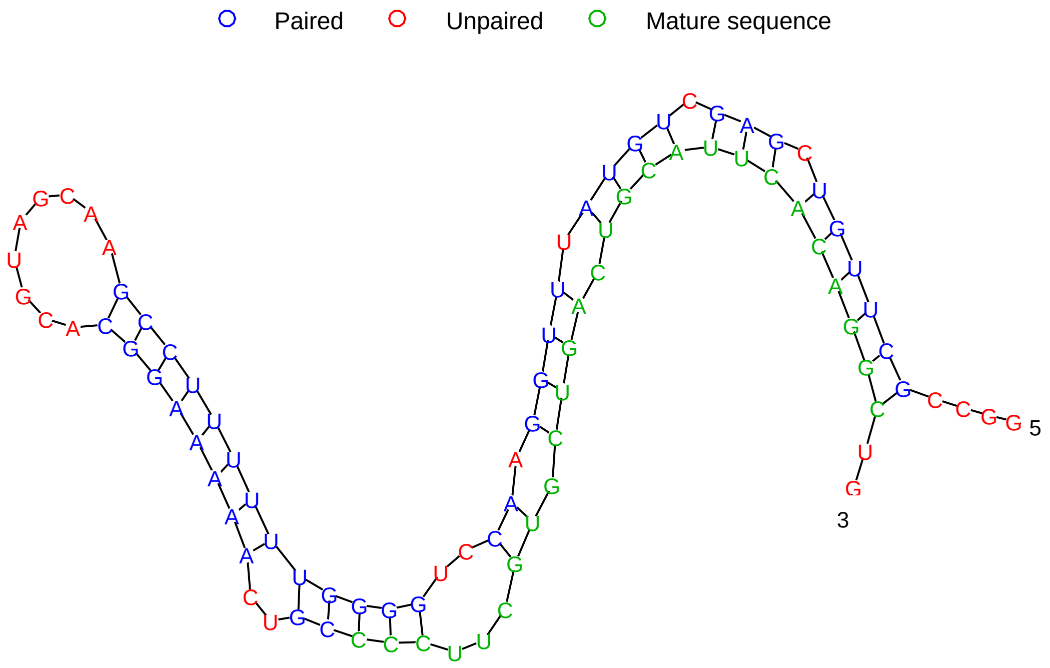

Stem loop (UMD3.1): chr7:51289387-51289478
Mature (UMD3.1): chr7:51289389-51289415
Mature seq len: 27
Total raw counts (9 samples): 1277
Average raw counts: 142
Strand: Reverse
Orientation: 3p
Minimum free energy: -31.20

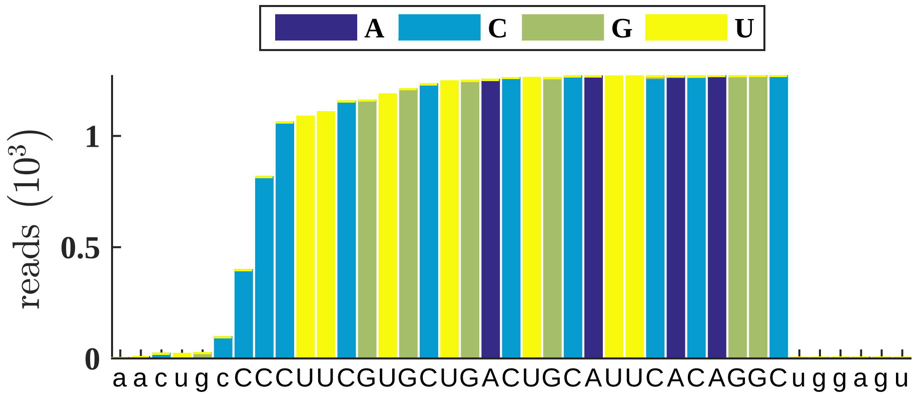

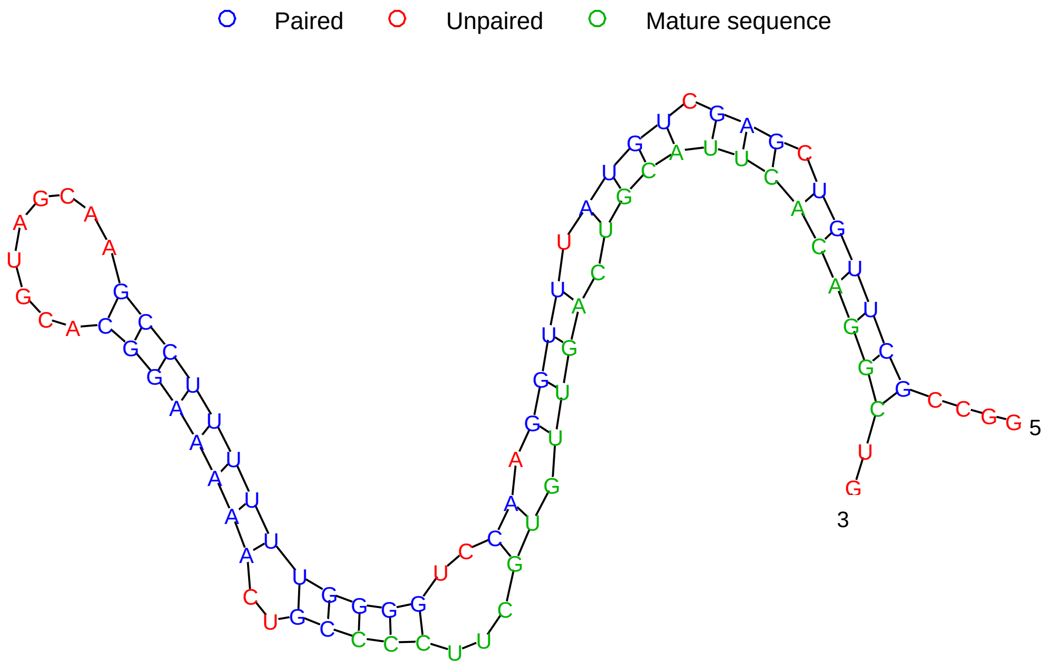

Stem loop (UMD3.1): chr7:51290443-51290534
Mature (UMD3.1): chr7:51290445-51290471
Mature seq len: 27
Total raw counts (9 samples): 1228
Average raw counts: 137
Strand: Reverse
Orientation: 3p
Minimum free energy: -28.90

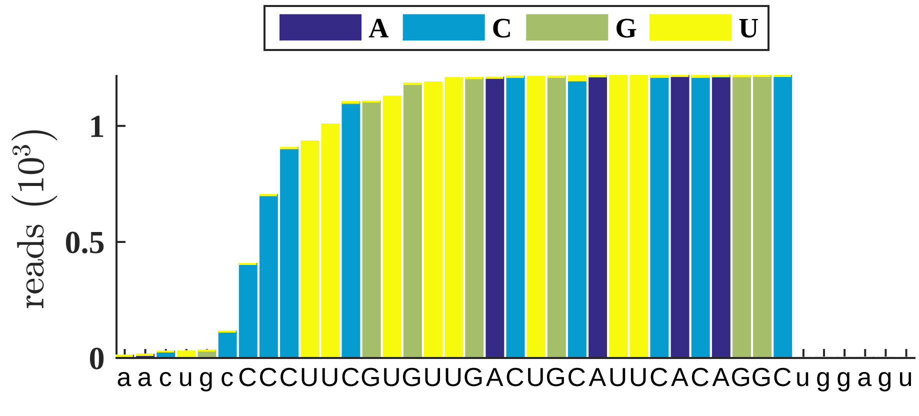

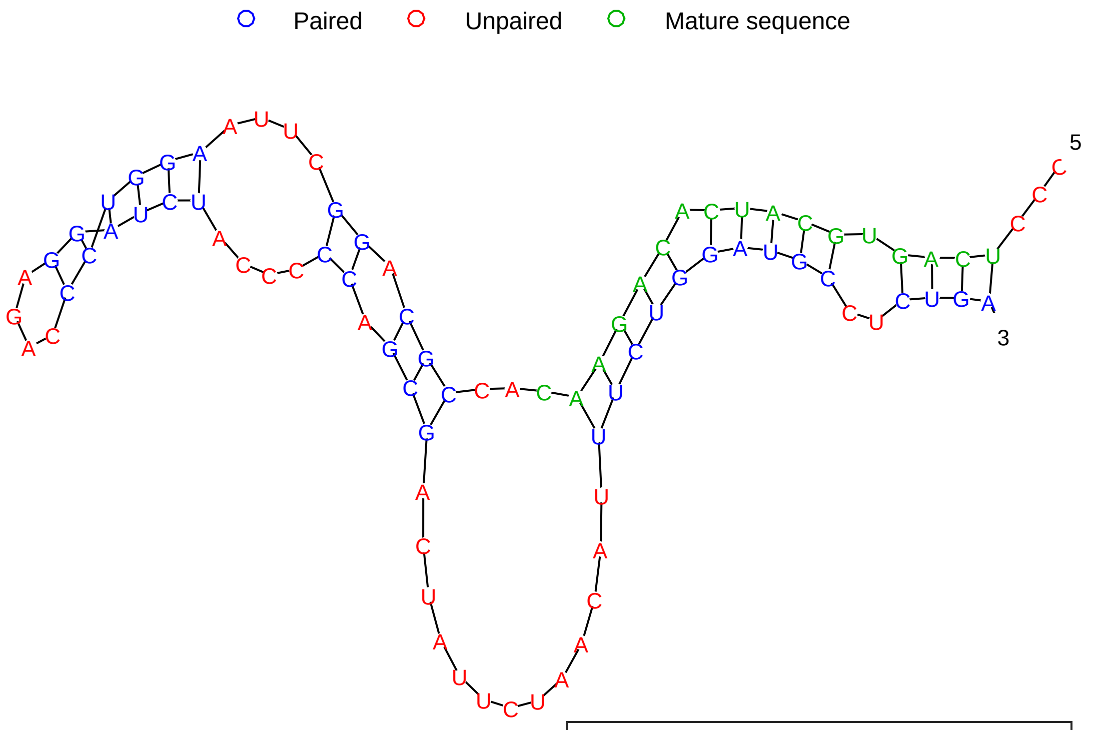

Stem loop (UMD3.1): chr7:64776446-64776532
Mature (UMD3.1): chr7:64776449-64776465
Mature seq len: 17
Total raw counts (9 samples): 582
Average raw counts: 65
Strand: Forward
Orientation: 5p
Minimum free energy: -25.30

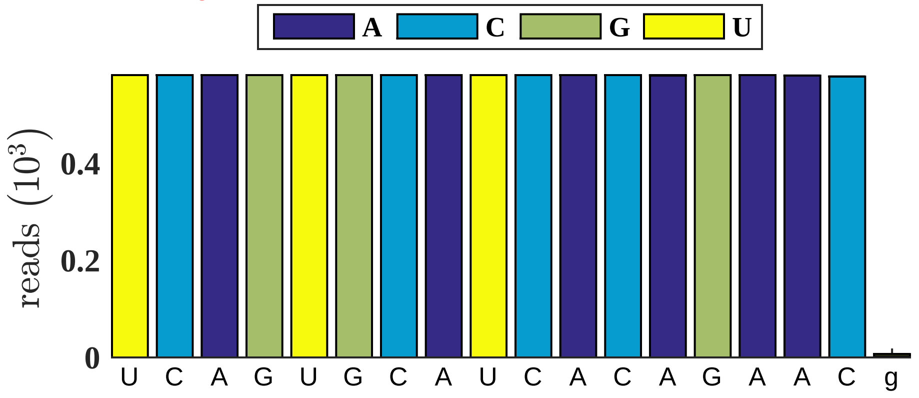

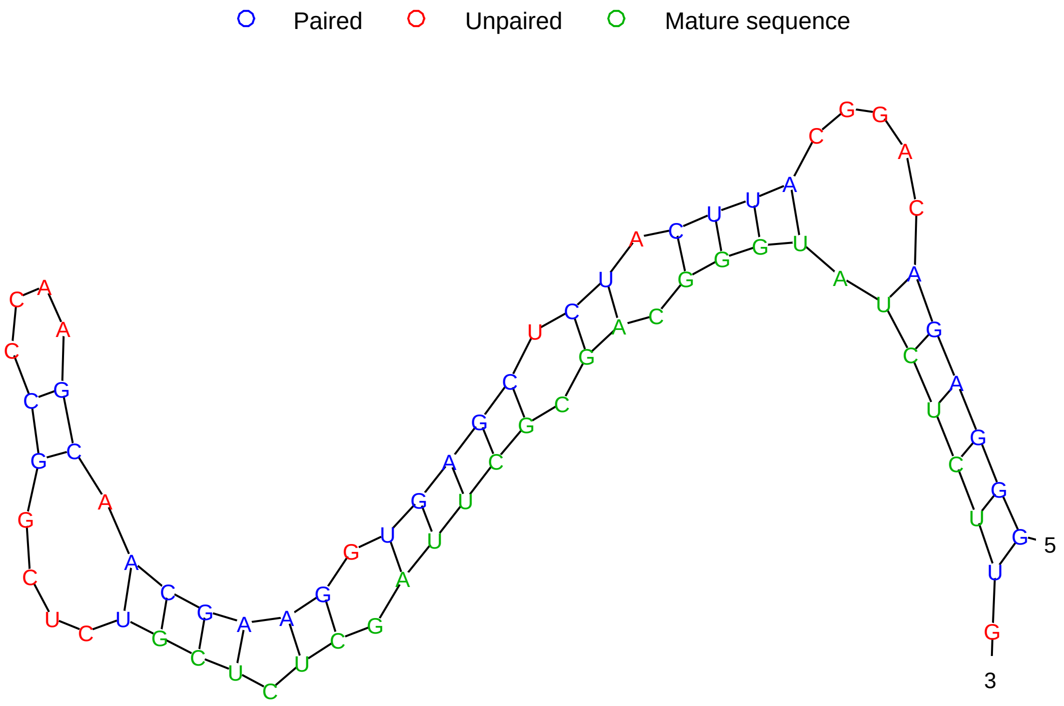

Stem loop (UMD3.1): chr7:71938629-71938701
Mature (UMD3.1): chr7:71938674-71938699
Mature seq len: 26
Total raw counts (9 samples): 7287
Average raw counts: 810
Strand: Forward
Orientation: 3p
Minimum free energy: -17.60

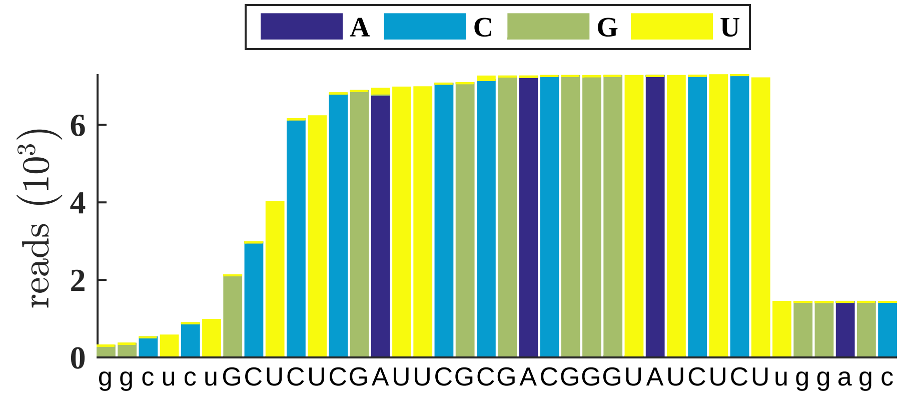

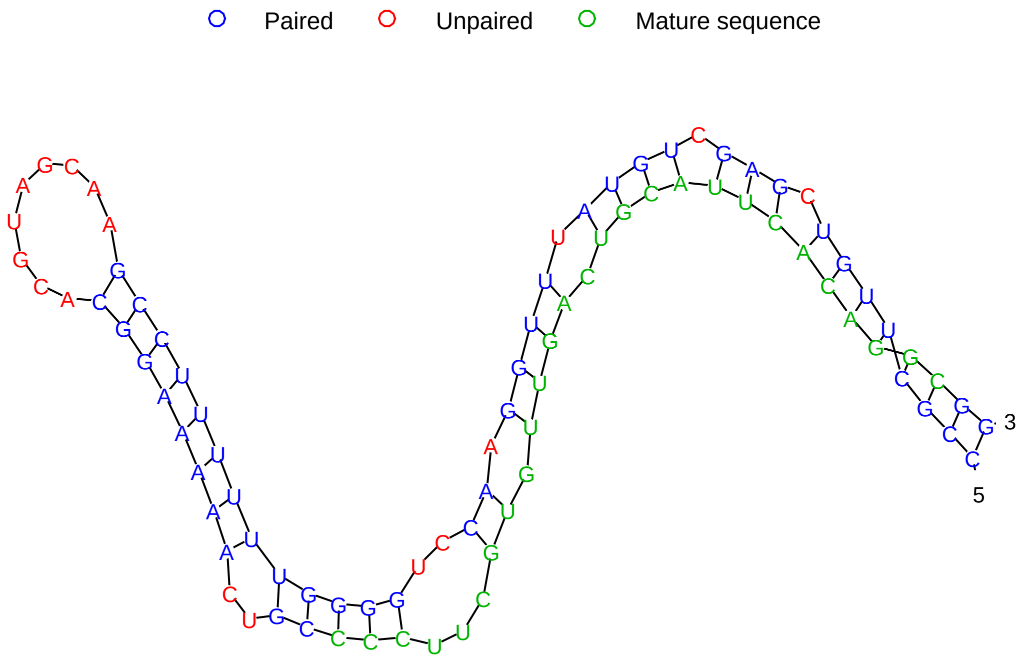

Stem loop (UMD3.1): chr7:80252211-80252300
Mature (UMD3.1): chr7:80252272-80252298
Mature seq len: 27
Total raw counts (9 samples): 1180
Average raw counts: 132
Strand: Forward
Orientation: 3p
Minimum free energy: -33.20

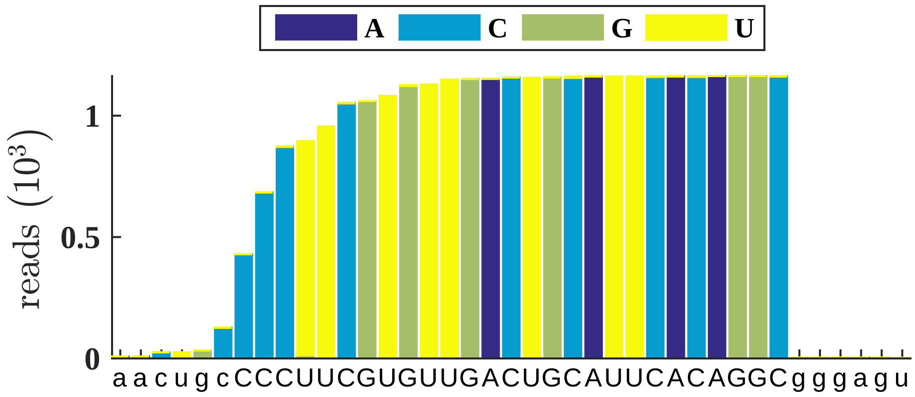

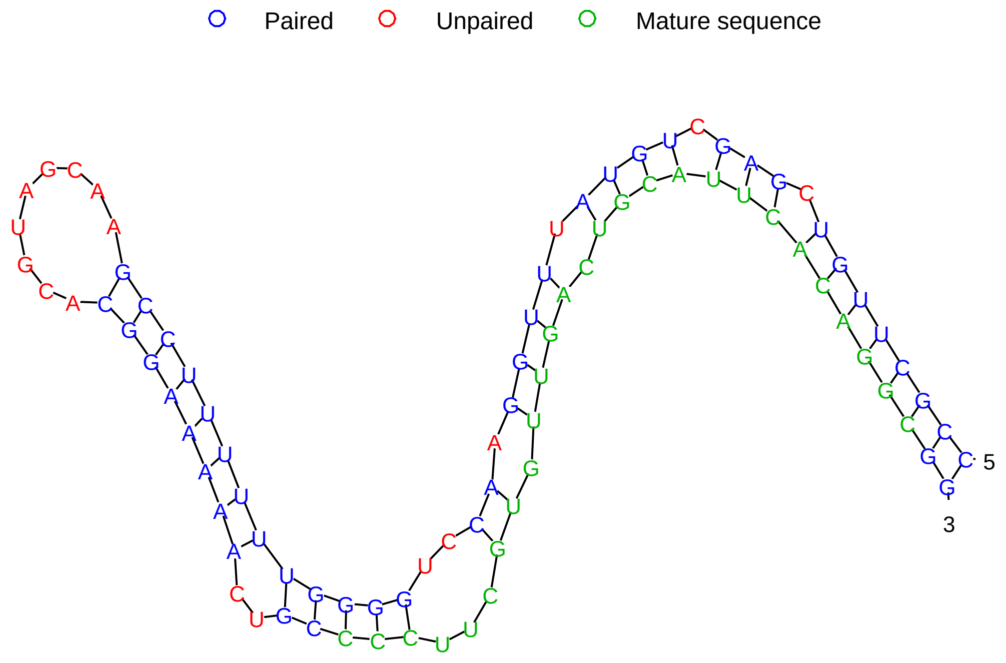

Stem loop (UMD3.1): chr7:93161632-93161721
Mature (UMD3.1): chr7:93161693-93161719
Mature seq len: 27
Total raw counts (9 samples): 1214
Average raw counts: 135
Strand: Forward
Orientation: 3p
Minimum free energy: -33.20

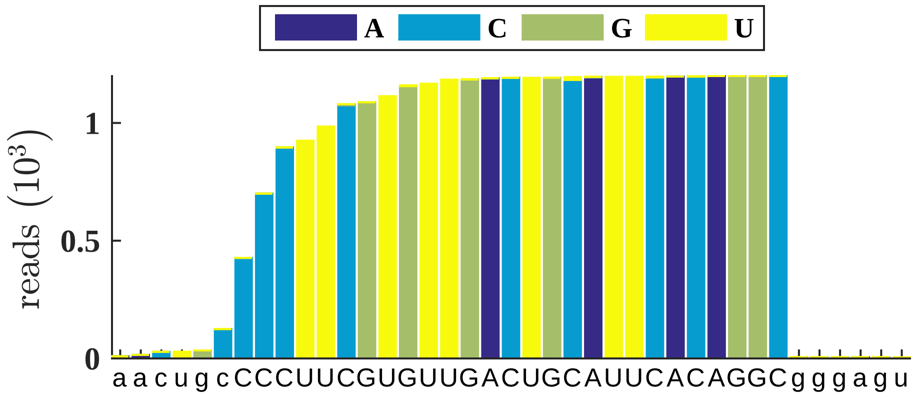

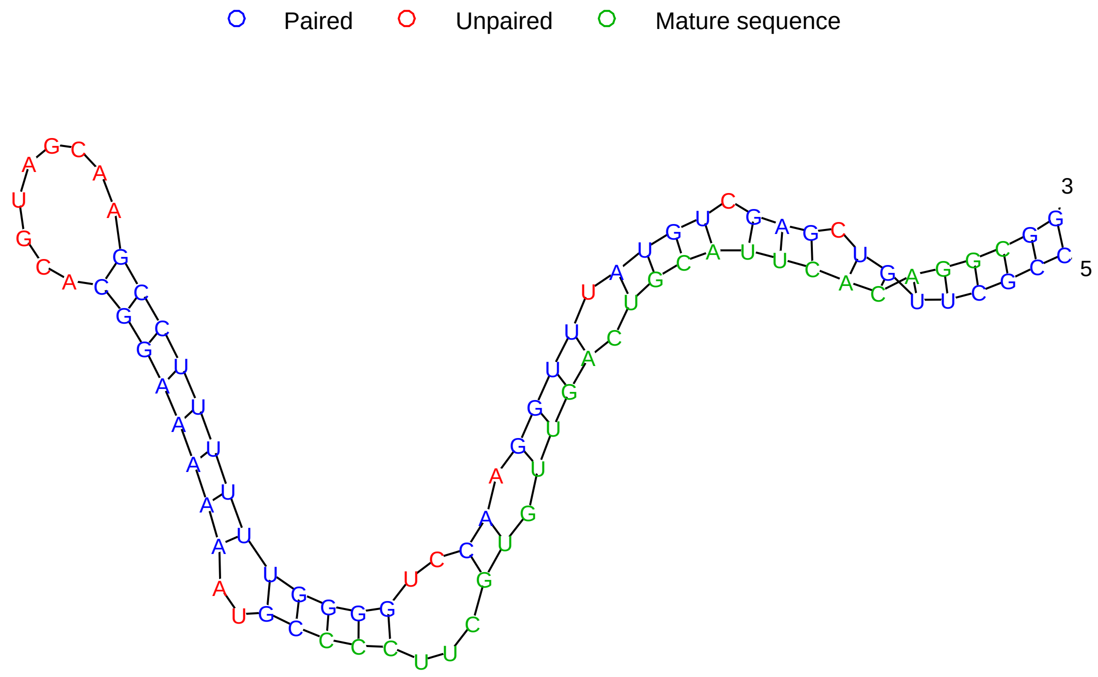

Stem loop (UMD3.1): chr7:93163032-93163121
Mature (UMD3.1): chr7:93163093-93163119
Mature seq len: 27
Total raw counts (9 samples): 1269
Average raw counts: 141
Strand: Forward
Orientation: 3p
Minimum free energy: -33.20

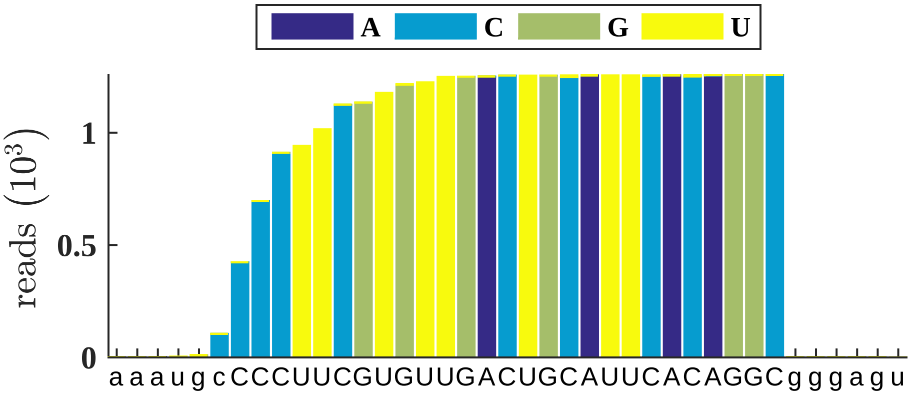

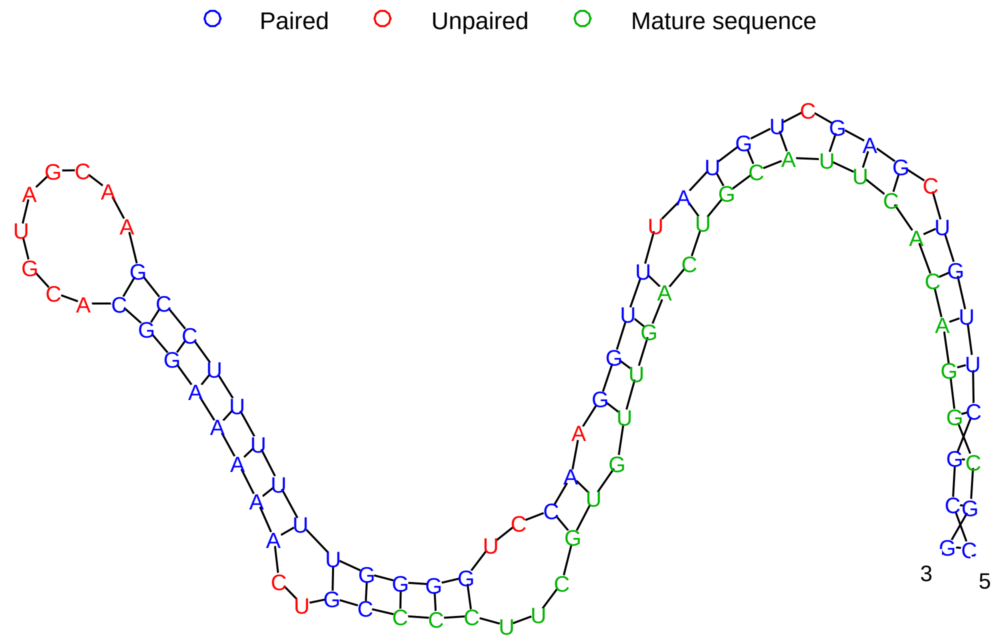

Stem loop (UMD3.1): chr7:93164704-93164793
Mature (UMD3.1): chr7:93164765-93164791
Mature seq len: 27
Total raw counts (9 samples): 1193
Average raw counts: 133
Strand: Forward
Orientation: 3p
Minimum free energy: -33.20

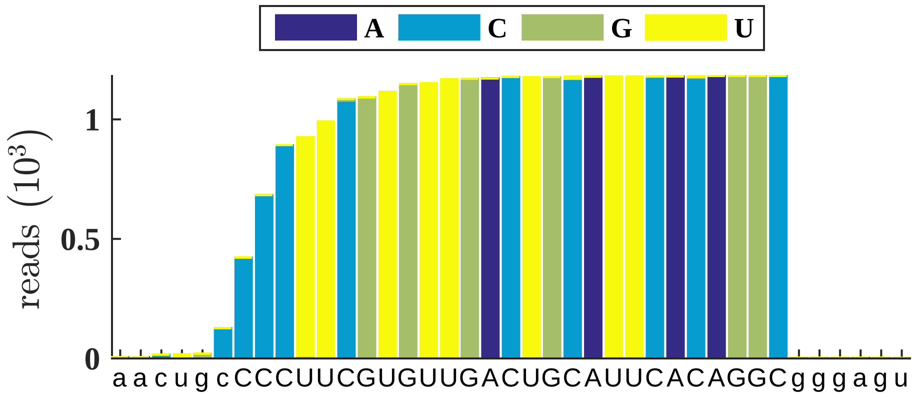

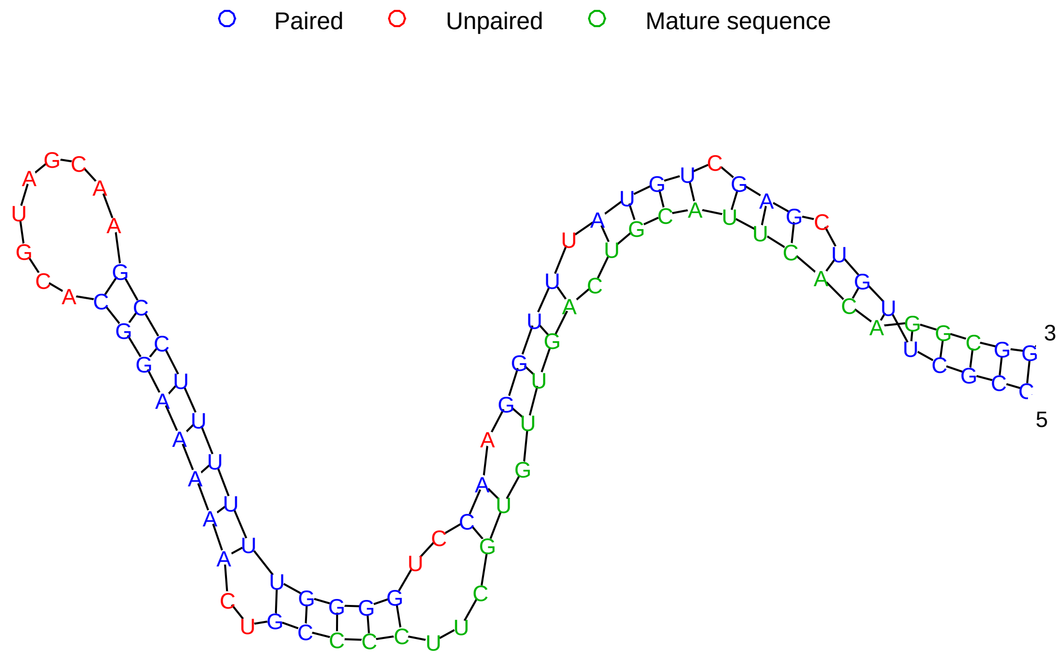

Stem loop (UMD3.1): chr8:110676305-110676394
Mature (UMD3.1): chr8:110676366-110676392
Mature seq len: 27
Total raw counts (9 samples): 1241
Average raw counts: 138
Strand: Forward
Orientation: 3p
Minimum free energy: -33.20

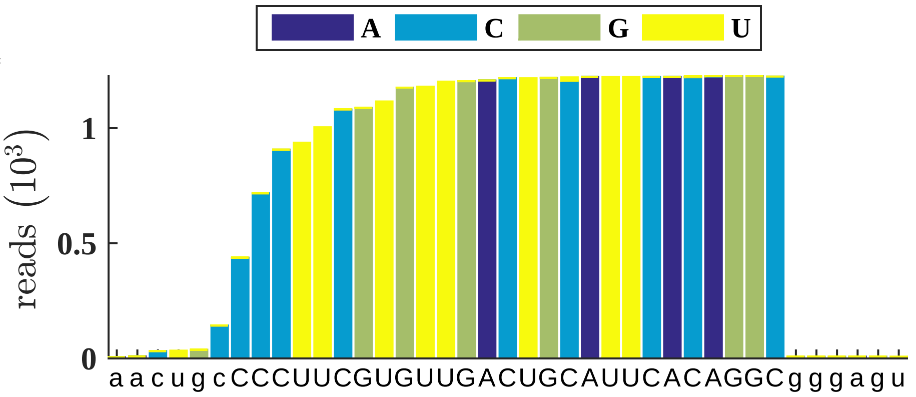

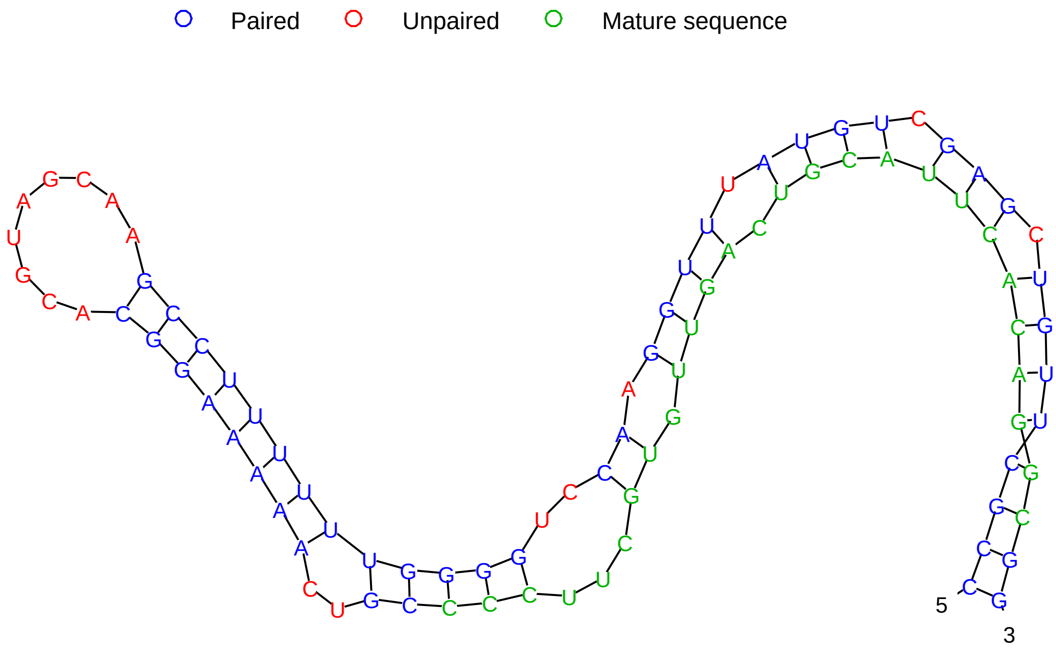

Stem loop (UMD3.1): chr8:110684999-110685088
Mature (UMD3.1): chr8:110685001-110685027
Mature seq len: 27
Total raw counts (9 samples): 1229
Average raw counts: 137
Strand: Reverse
Orientation: 3p
Minimum free energy: -33.20

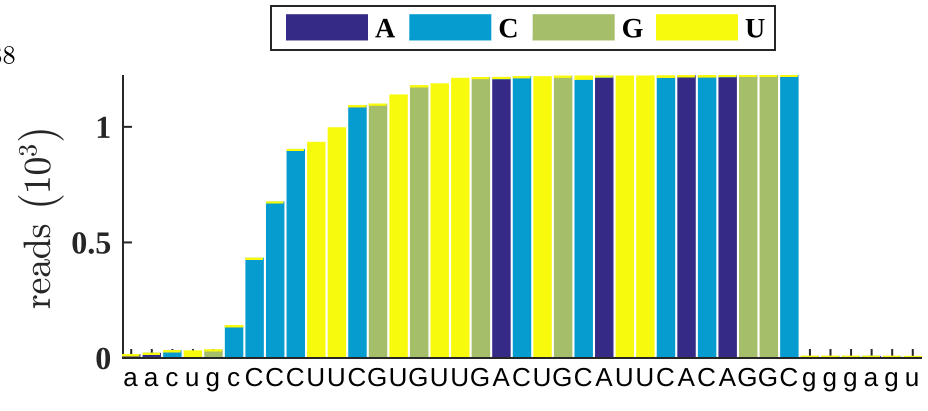

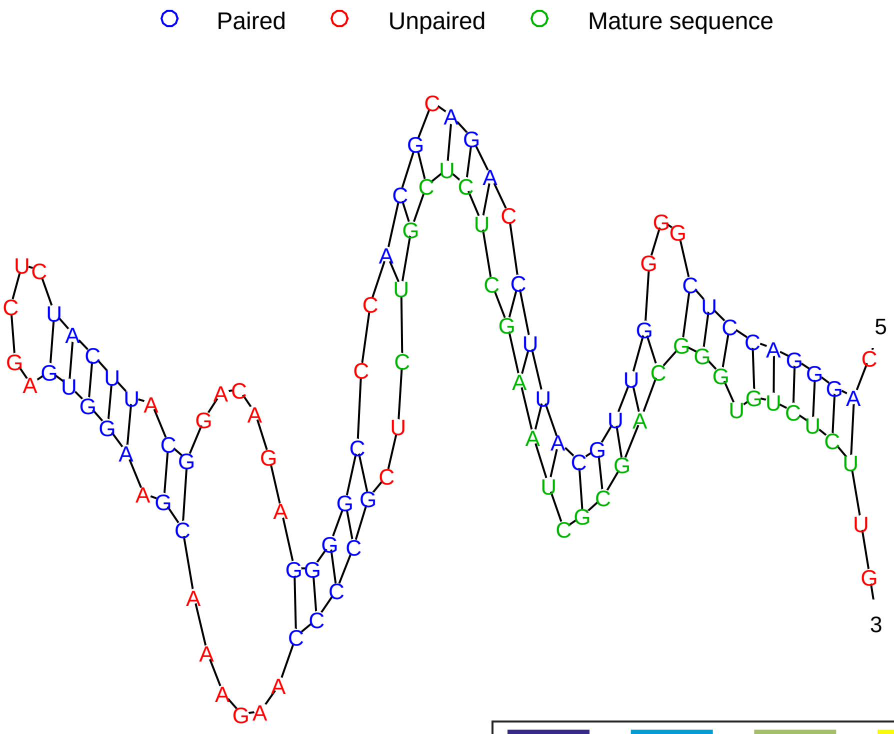

Stem loop (UMD3.1): chr8:110690124-110690230
Mature (UMD3.1): chr8:110690201-110690228
Mature seq len: 28
Total raw counts (9 samples): 2131
Average raw counts: 237
Strand: Forward
Orientation: 3p
Minimum free energy: -38.50

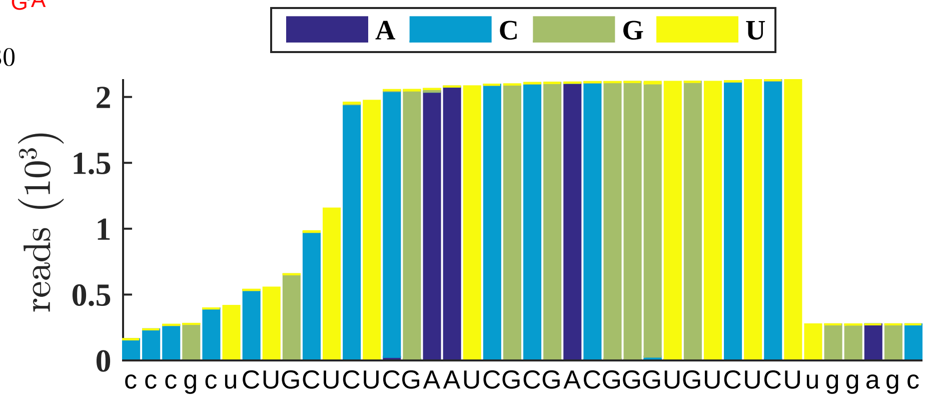

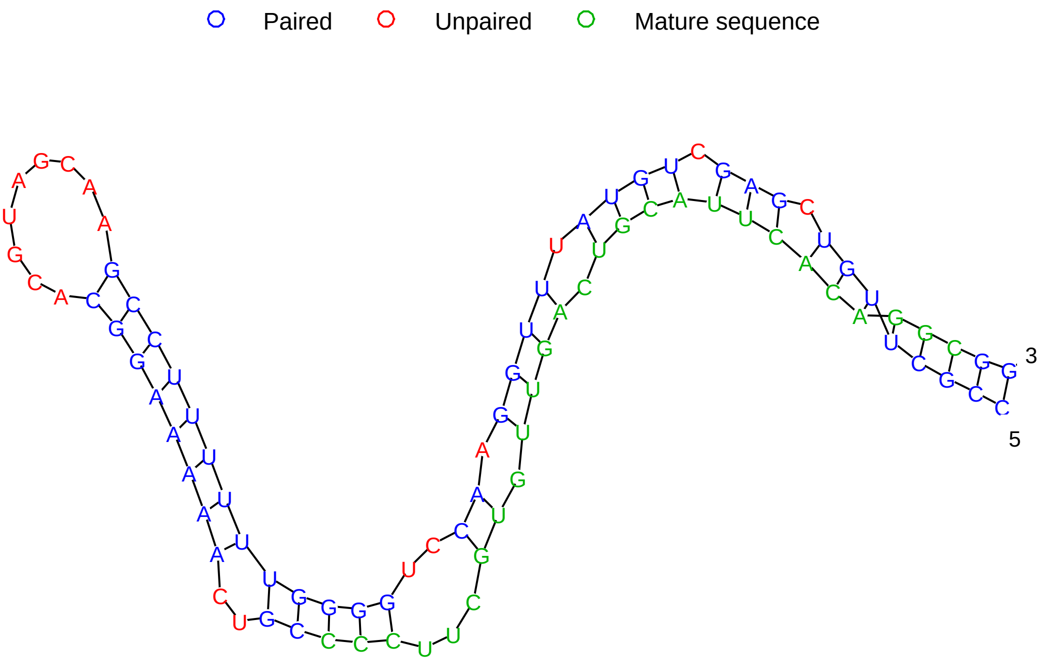

Stem loop (UMD3.1): chr8:110697128-110697217
Mature (UMD3.1): chr8:110697189-110697215
Mature seq len: 27
Total raw counts (9 samples): 1244
Average raw counts: 139
Strand: Forward
Orientation: 3p
Minimum free energy: -33.20

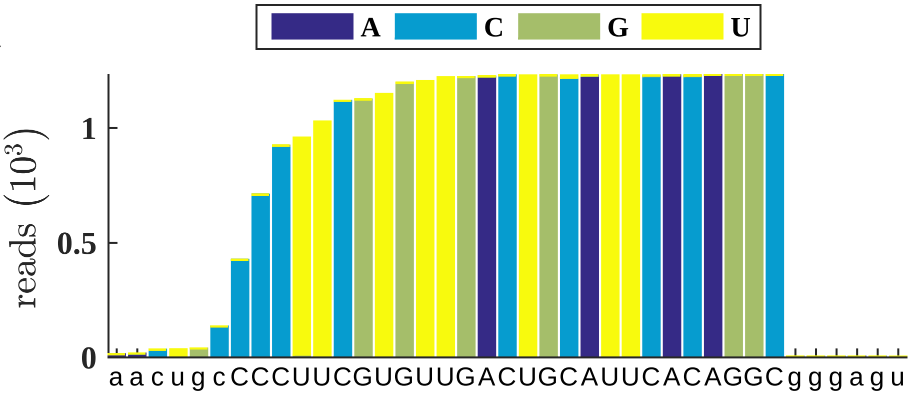

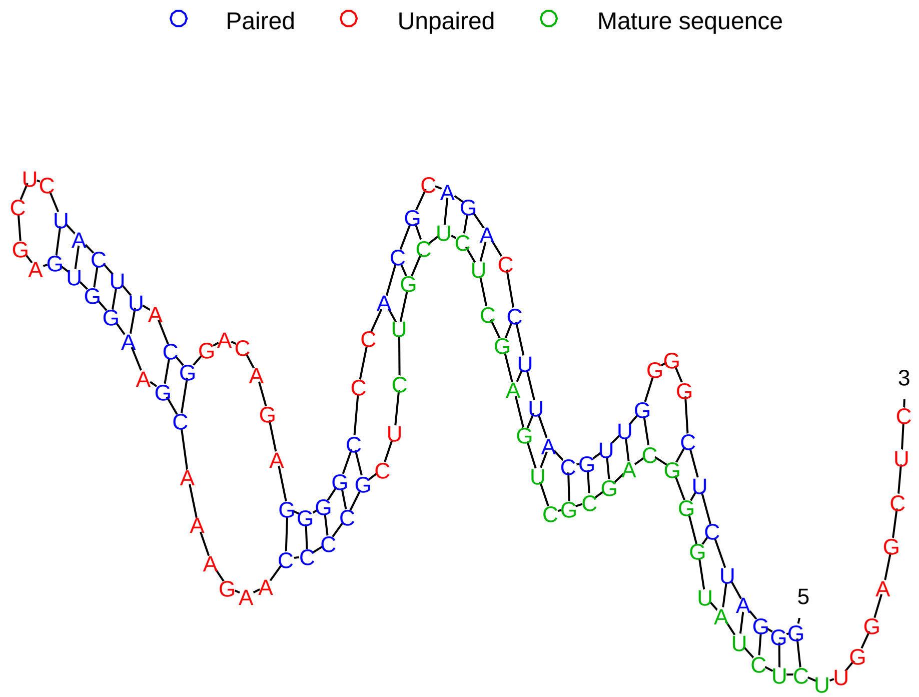

Stem loop (UMD3.1): chr8:110718497-110718607
Mature (UMD3.1): chr8:110718505-110718532
Mature seq len: 28
Total raw counts (9 samples): 13323
Average raw counts: 1481
Strand: Reverse
Orientation: 3p
Minimum free energy: -35.50

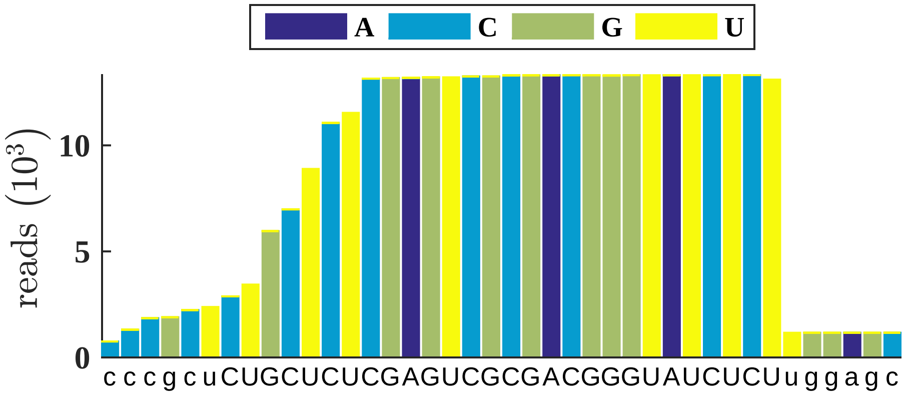

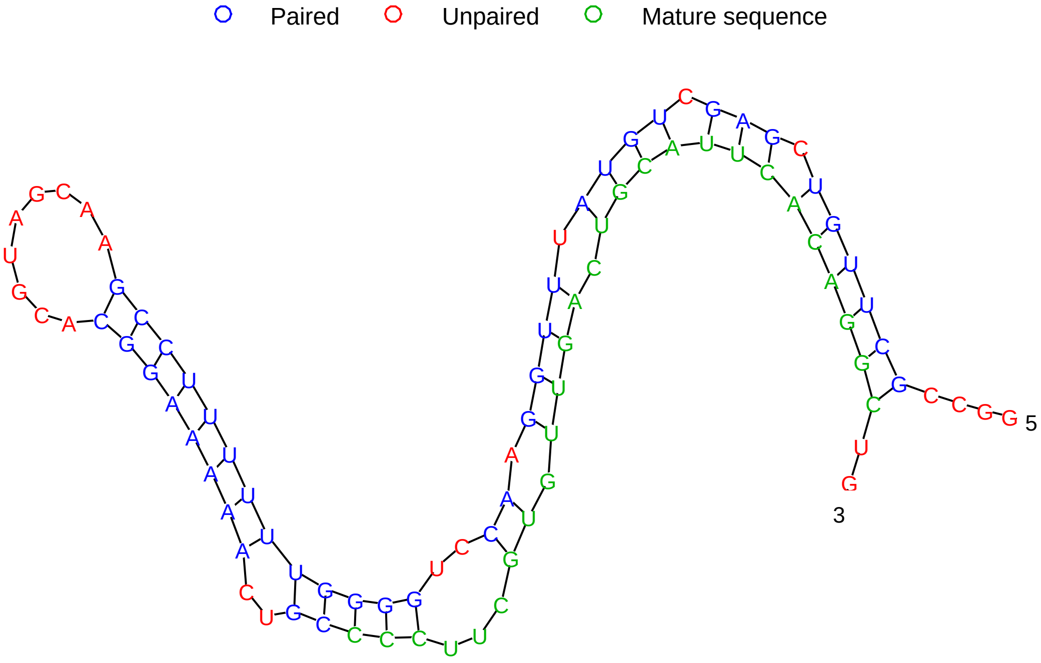

Stem loop (UMD3.1): chr8:110733923-110734014
Mature (UMD3.1): chr8:110733925-110733951
Mature seq len: 27
Total raw counts (9 samples): 1220
Average raw counts: 136
Strand: Reverse
Orientation: 3p
Minimum free energy: -28.90

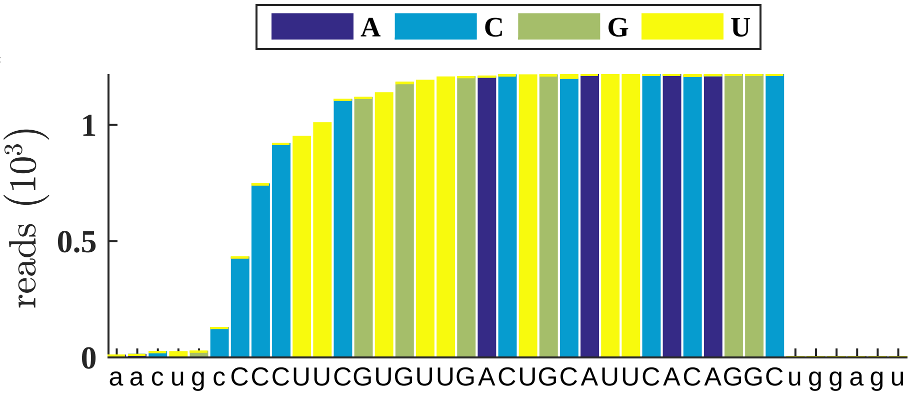

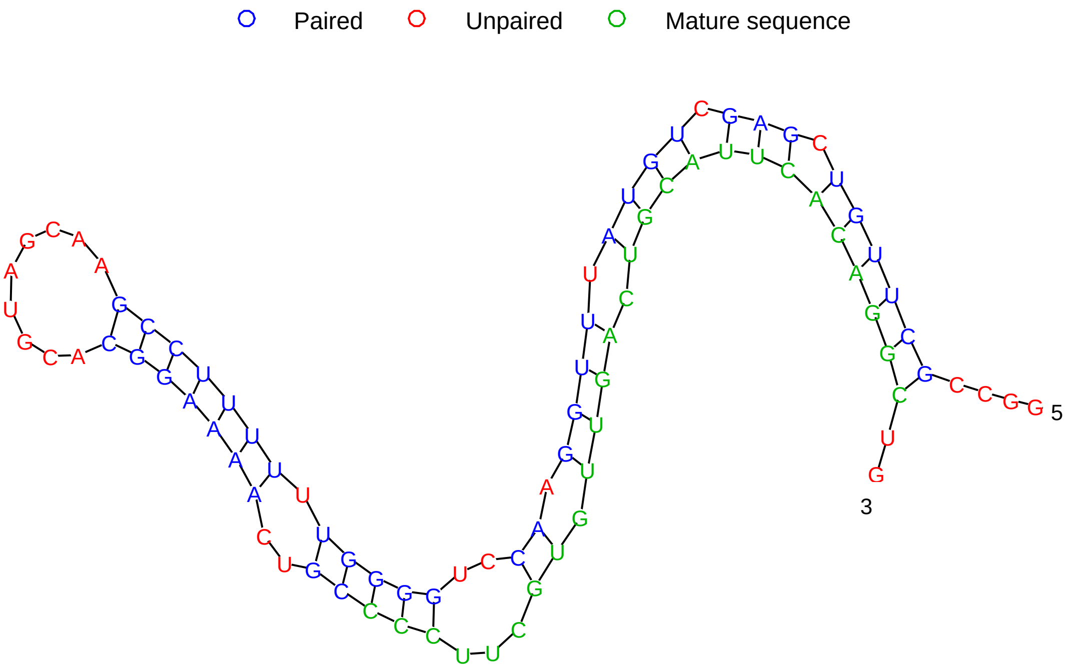

Stem loop (UMD3.1): chr8:110743074-110743164
Mature (UMD3.1): chr8:110743136-110743162
Mature seq len: 27
Total raw counts (9 samples): 1192
Average raw counts: 133
Strand: Forward
Orientation: 3p
Minimum free energy: -28.80

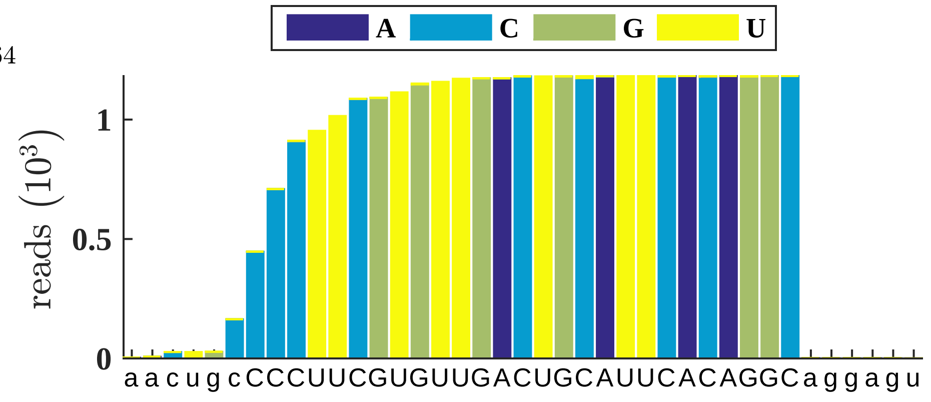

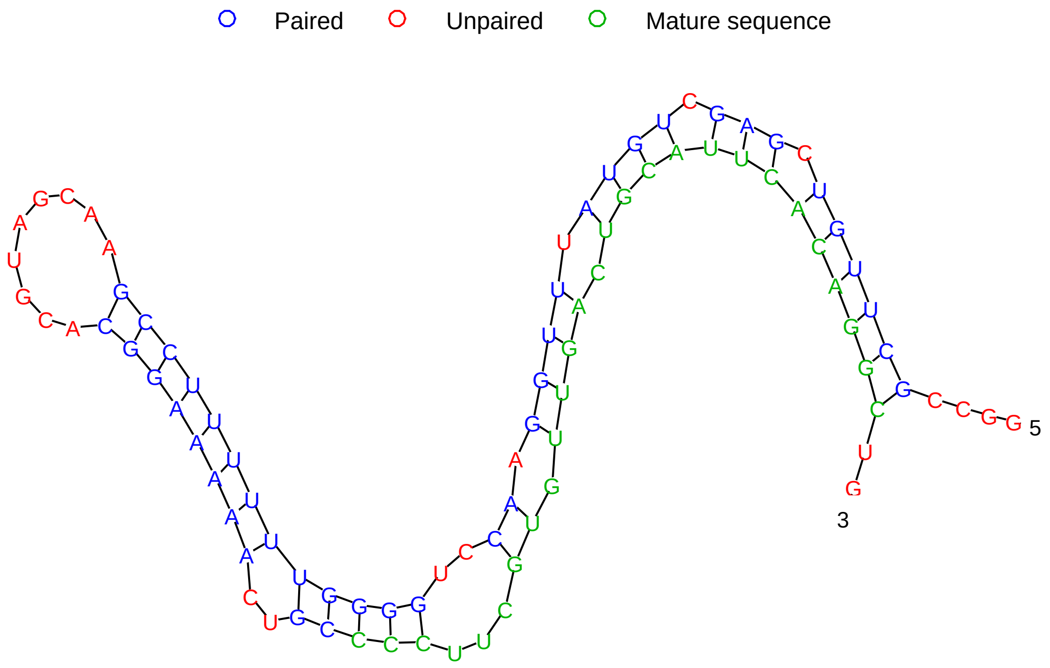

Stem loop (UMD3.1): chr8:110757056-110757147
Mature (UMD3.1): chr8:110757119-110757145
Mature seq len: 27
Total raw counts (9 samples): 1145
Average raw counts: 128
Strand: Forward
Orientation: 3p
Minimum free energy: -28.90

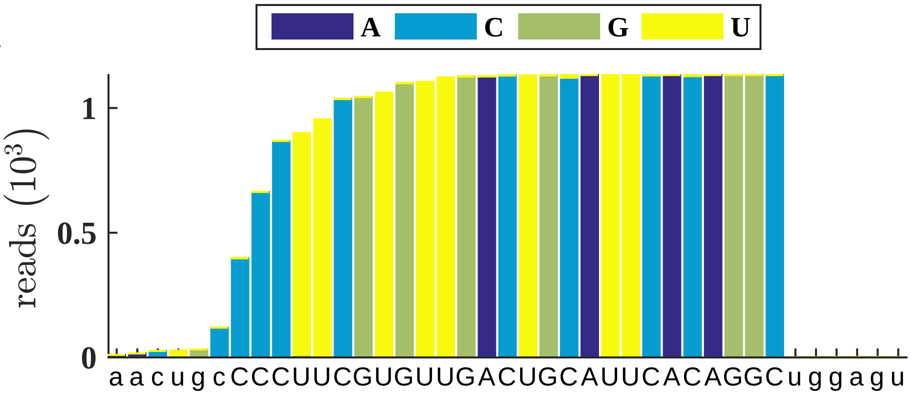

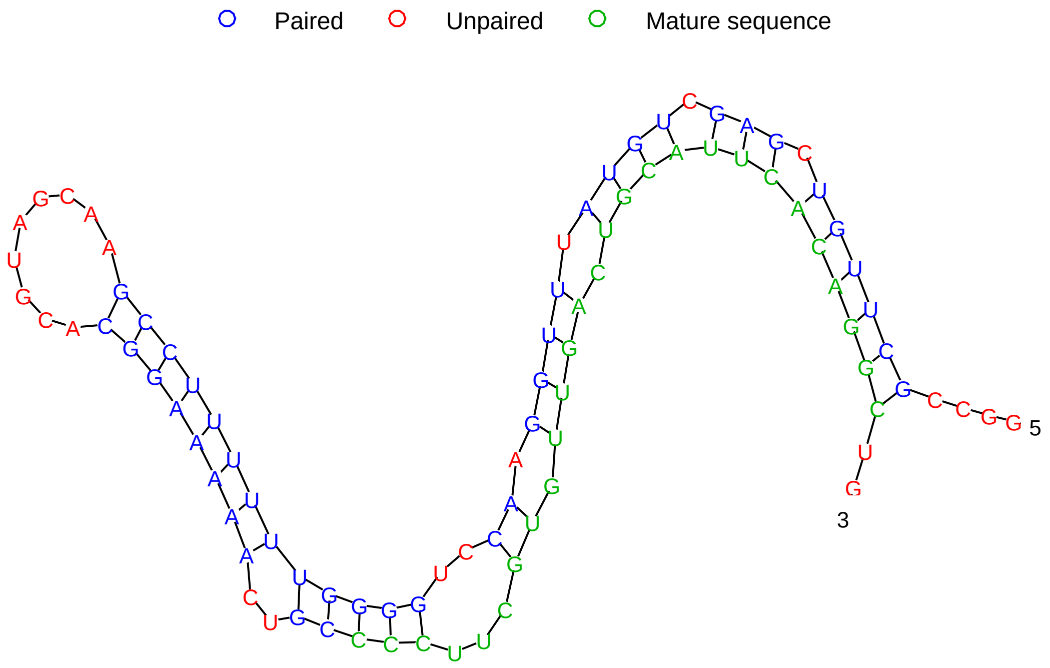

Stem loop (UMD3.1): chr8:110758928-110759019
Mature (UMD3.1): chr8:110758930-110758956
Mature seq len: 27
Total raw counts (9 samples): 1111
Average raw counts: 124
Strand: Reverse
Orientation: 3p
Minimum free energy: -28.90

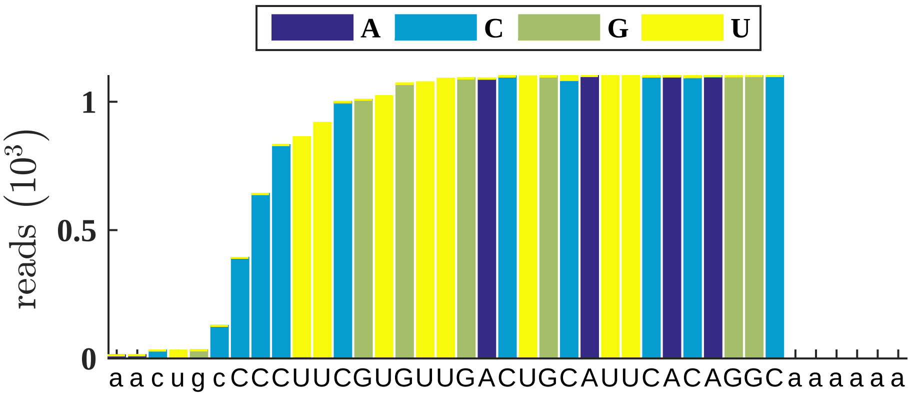

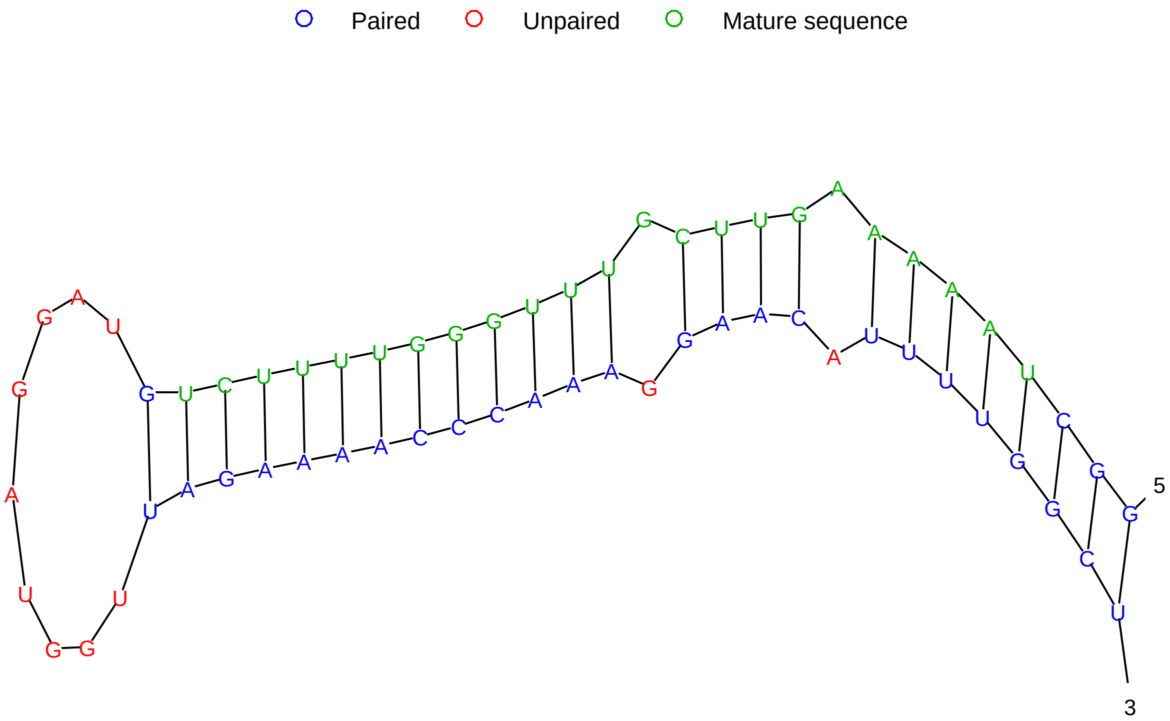

Stem loop (UMD3.1): chr8:111841830-111841892
Mature (UMD3.1): chr8:111841867-111841889
Mature seq len: 23
Total raw counts (9 samples): 811
Average raw counts: 91
Strand: Reverse
Orientation: 5p
Minimum free energy: -32.00

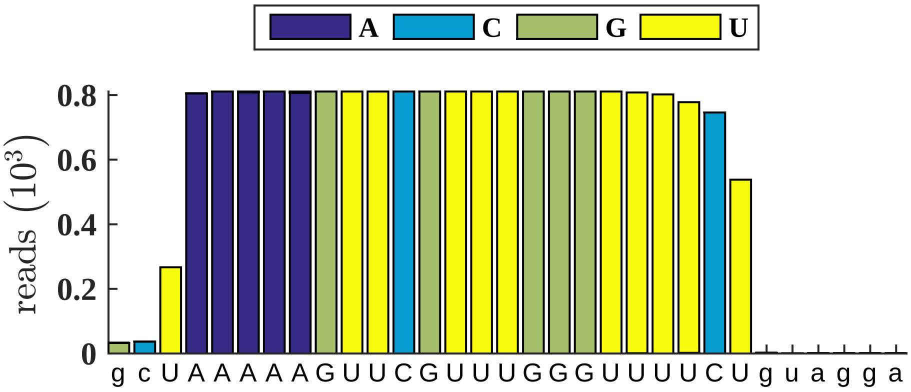

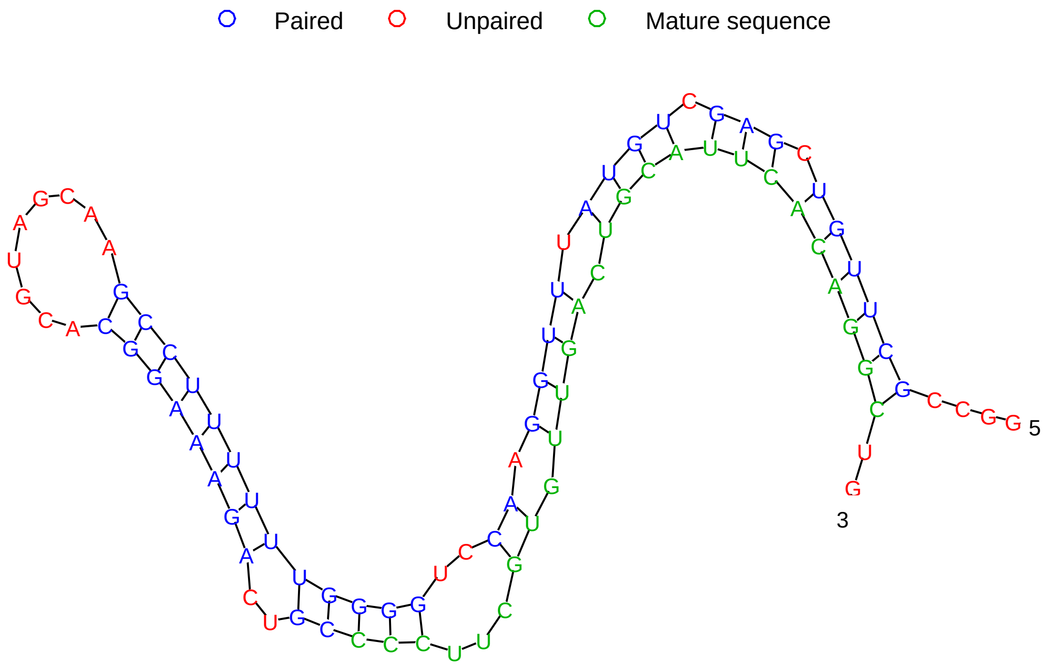

Stem loop (UMD3.1): chr8:11955733-11955824
Mature (UMD3.1): chr8:11955796-11955822
Mature seq len: 27
Total raw counts (9 samples): 1202
Average raw counts: 134
Strand: Forward
Orientation: 3p
Minimum free energy: -29.00

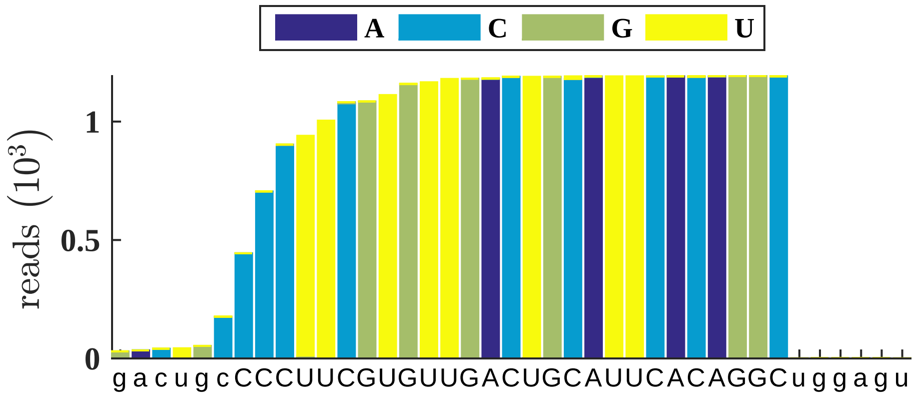

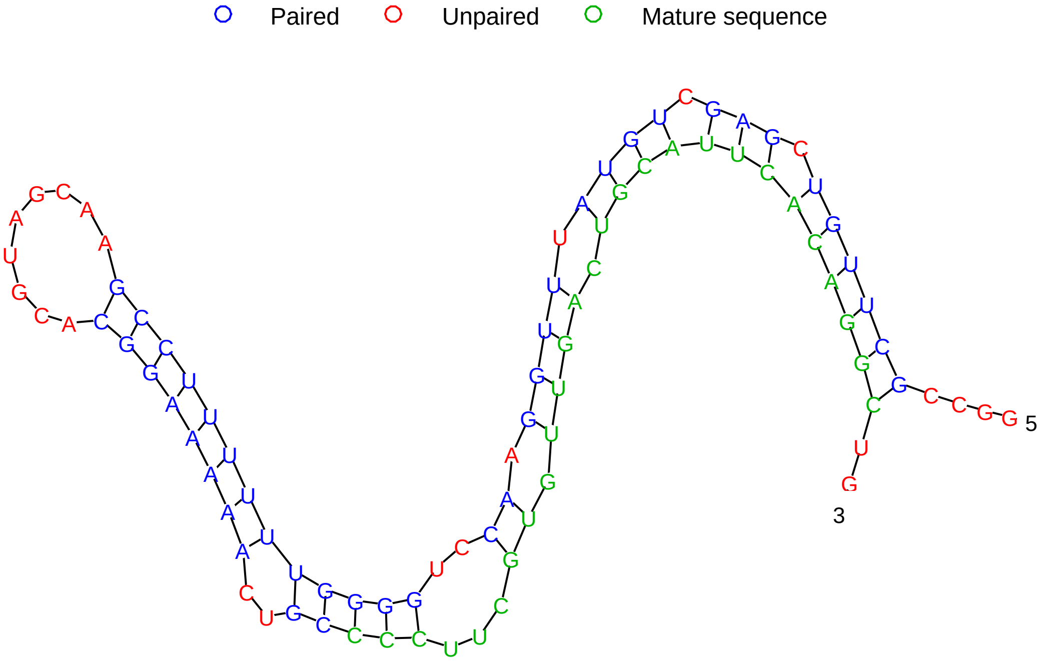

Stem loop (UMD3.1): chr8:11956503-11956594
Mature (UMD3.1): chr8:11956505-11956531
Mature seq len: 27
Total raw counts (9 samples): 1199
Average raw counts: 134
Strand: Reverse
Orientation: 3p
Minimum free energy: -28.90

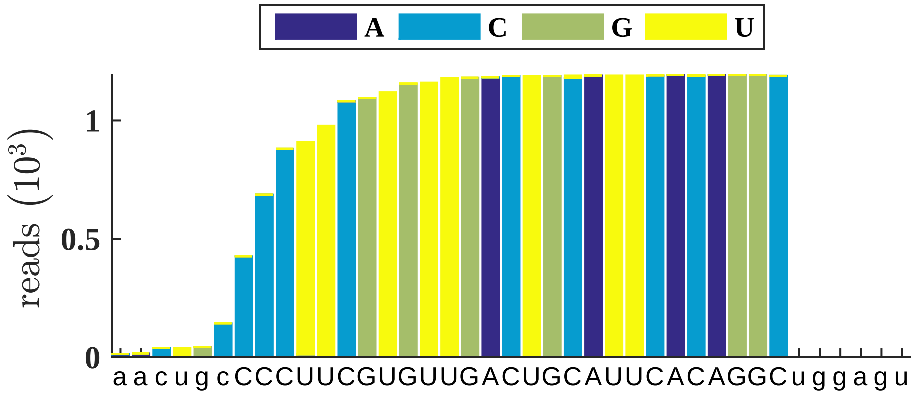

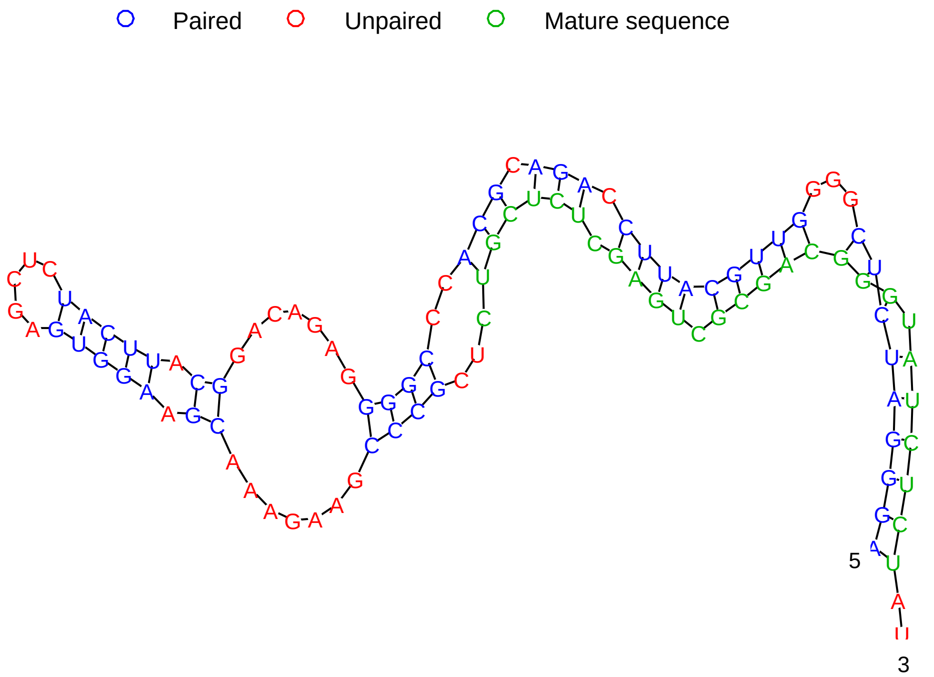

Stem loop (UMD3.1): chr8:24888252-24888357
Mature (UMD3.1): chr8:24888328-24888355
Mature seq len: 28
Total raw counts (9 samples): 11745
Average raw counts: 1305
Strand: Forward
Orientation: 3p
Minimum free energy: -34.10

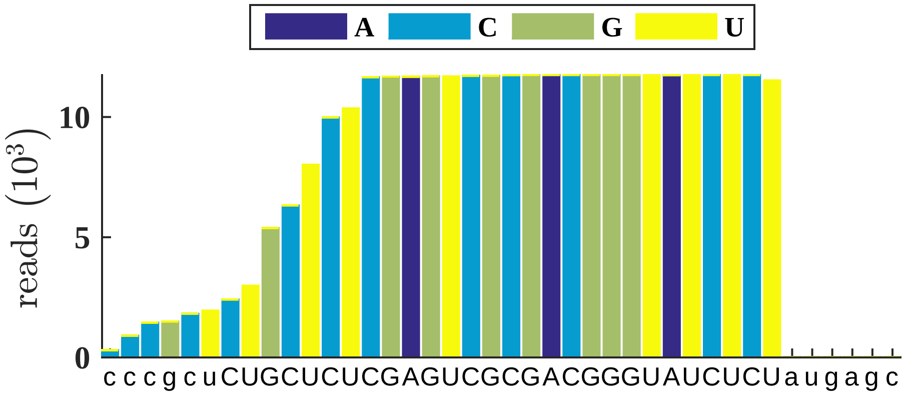

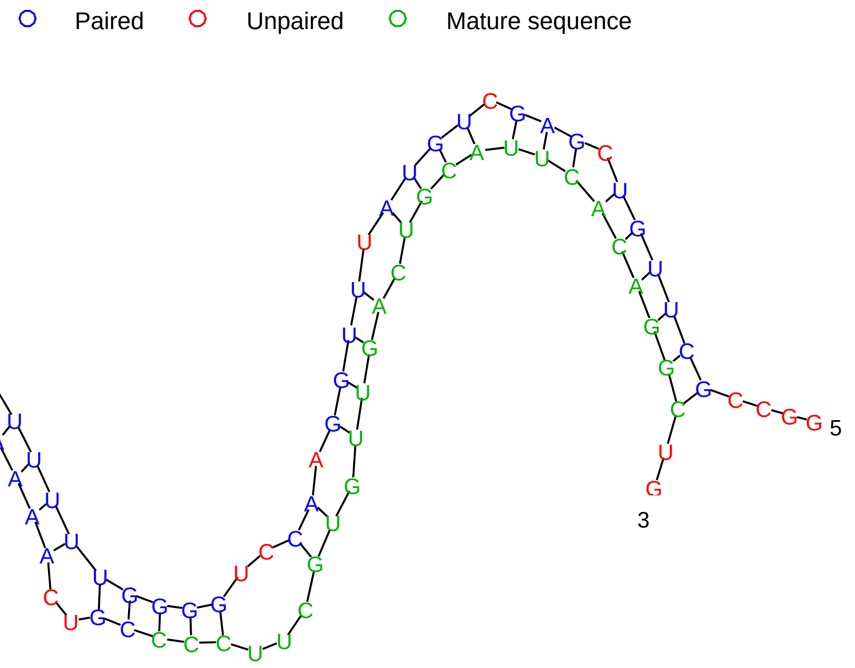

Stem loop (UMD3.1): chr8:24890361-24890452
Mature (UMD3.1): chr8:24890424-24890450
Mature seq len: 27
Total raw counts (9 samples): 1147
Average raw counts: 128
Strand: Forward
Orientation: 3p
Minimum free energy: -28.90

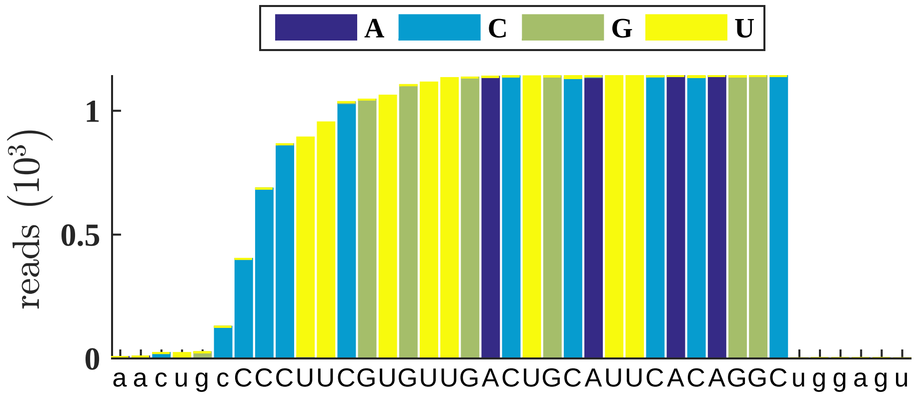

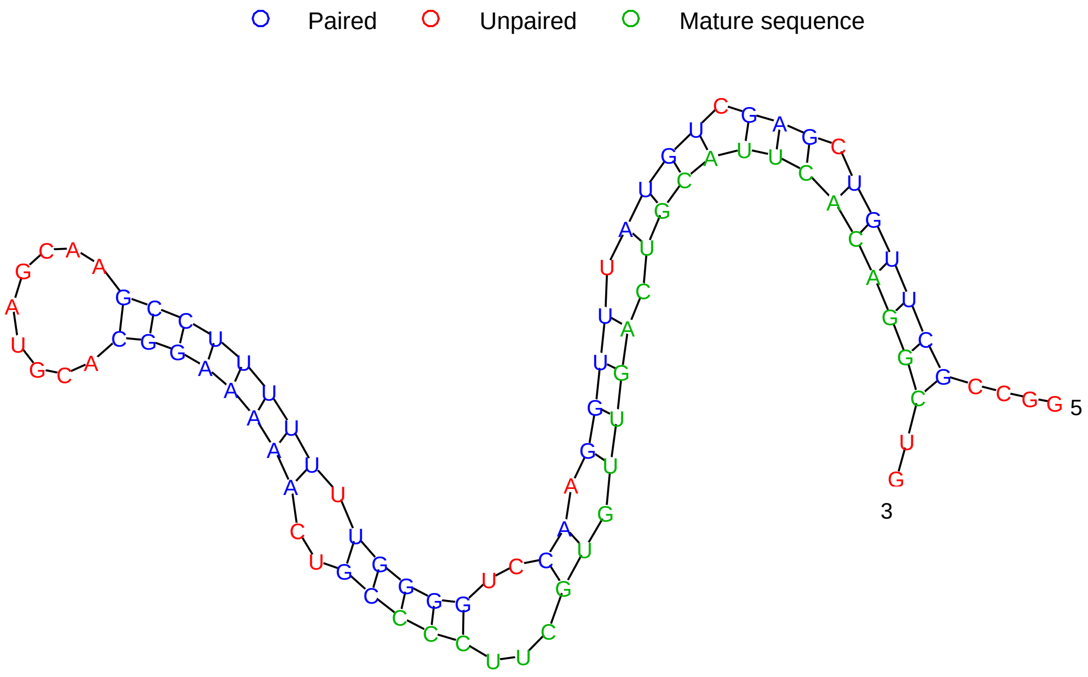

Stem loop (UMD3.1): chr8:24899765-24899857
Mature (UMD3.1): chr8:24899829-24899855
Mature seq len: 27
Total raw counts (9 samples): 1216
Average raw counts: 136
Strand: Forward
Orientation: 3p
Minimum free energy: -29.70

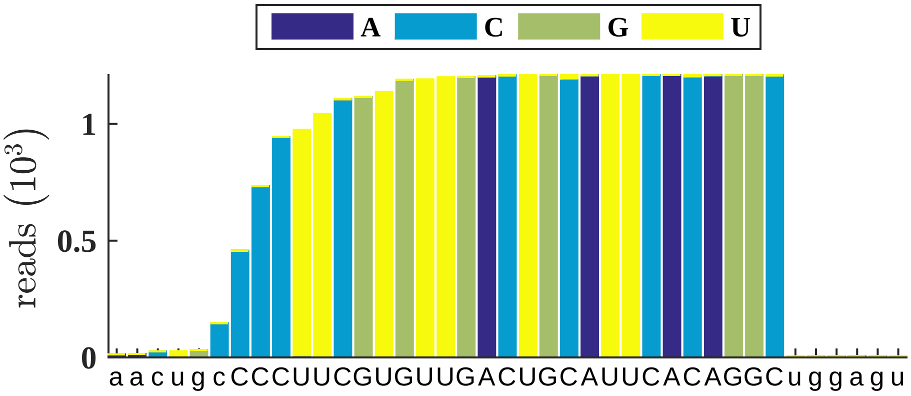

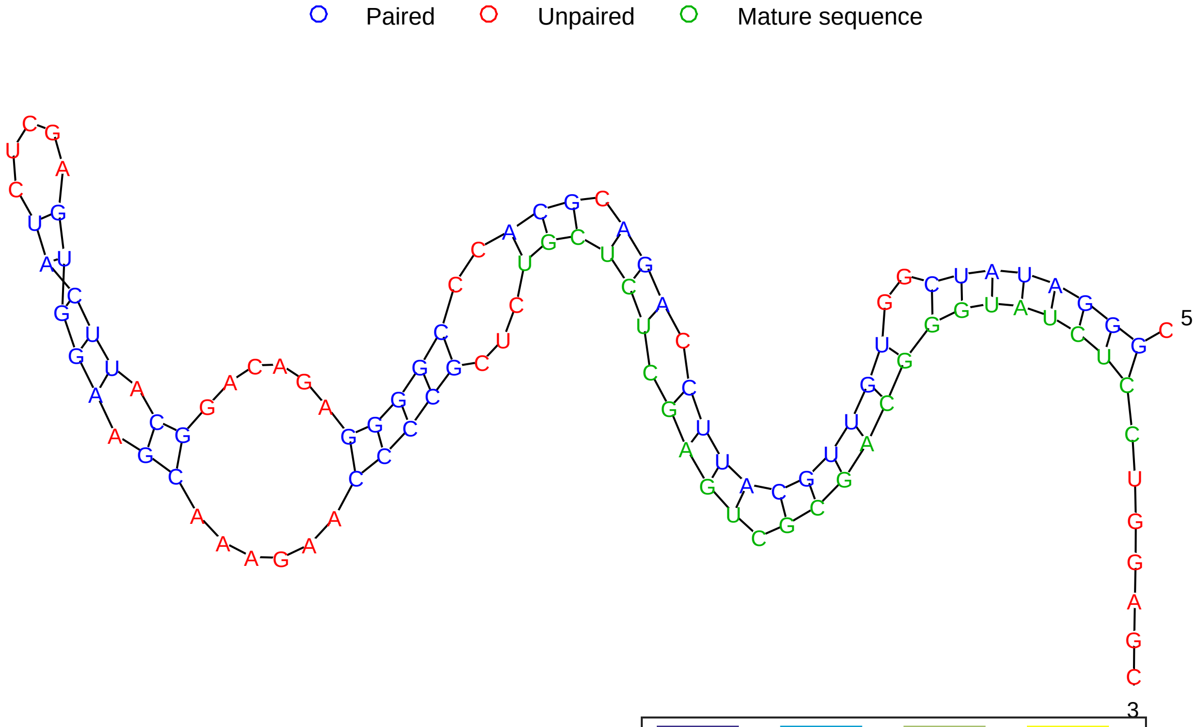

Stem loop (UMD3.1): chr8:36117586-36117695
Mature (UMD3.1): chr8:36117592-36117618
Mature seq len: 27
Total raw counts (9 samples): 1795
Average raw counts: 200
Strand: Reverse
Orientation: 3p
Minimum free energy: -38.70

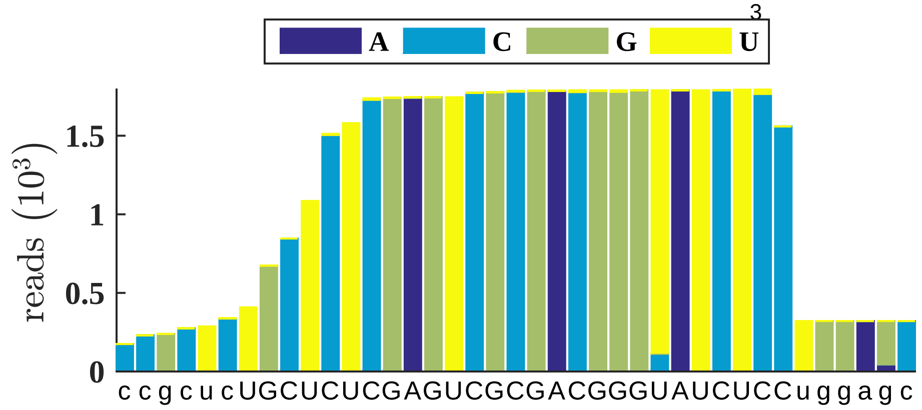

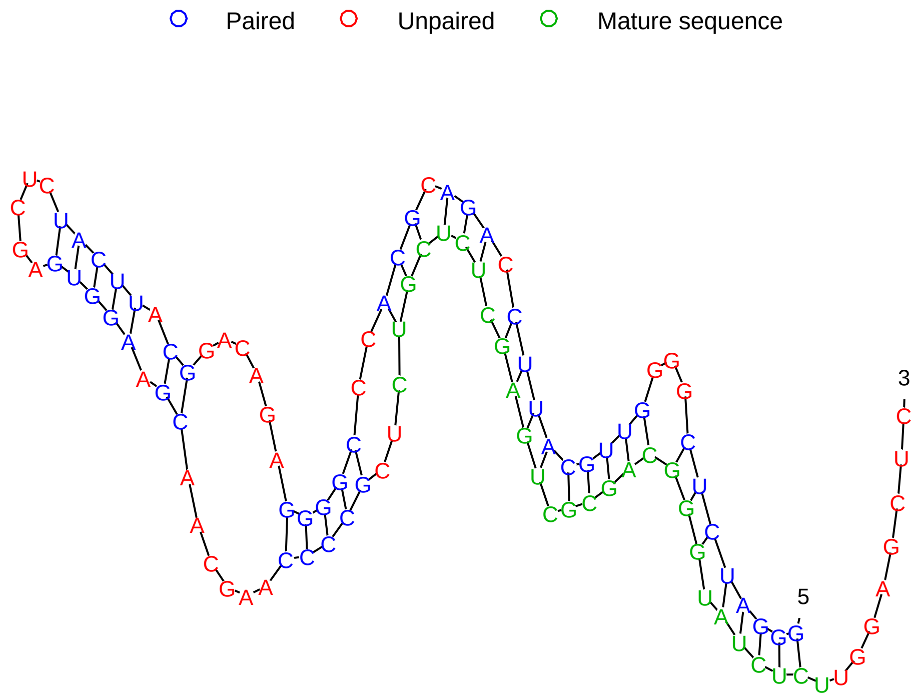

Stem loop (UMD3.1): chr8:36133016-36133126
Mature (UMD3.1): chr8:36133024-36133051
Mature seq len: 28
Total raw counts (9 samples): 13311
Average raw counts: 1479
Strand: Reverse
Orientation: 3p
Minimum free energy: -35.50

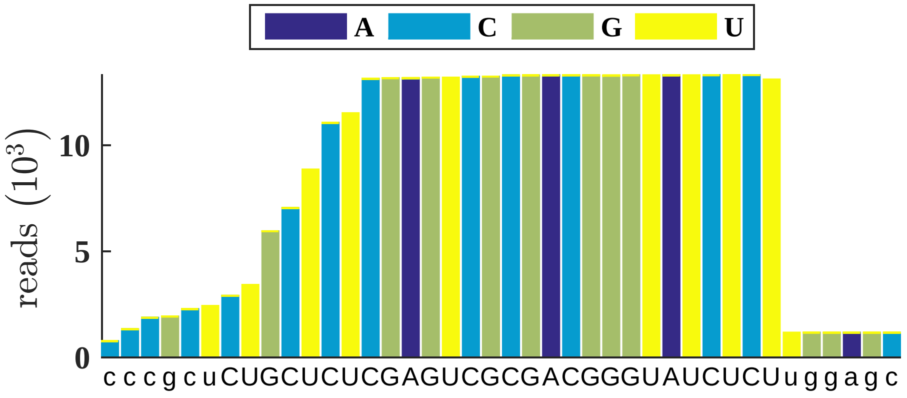

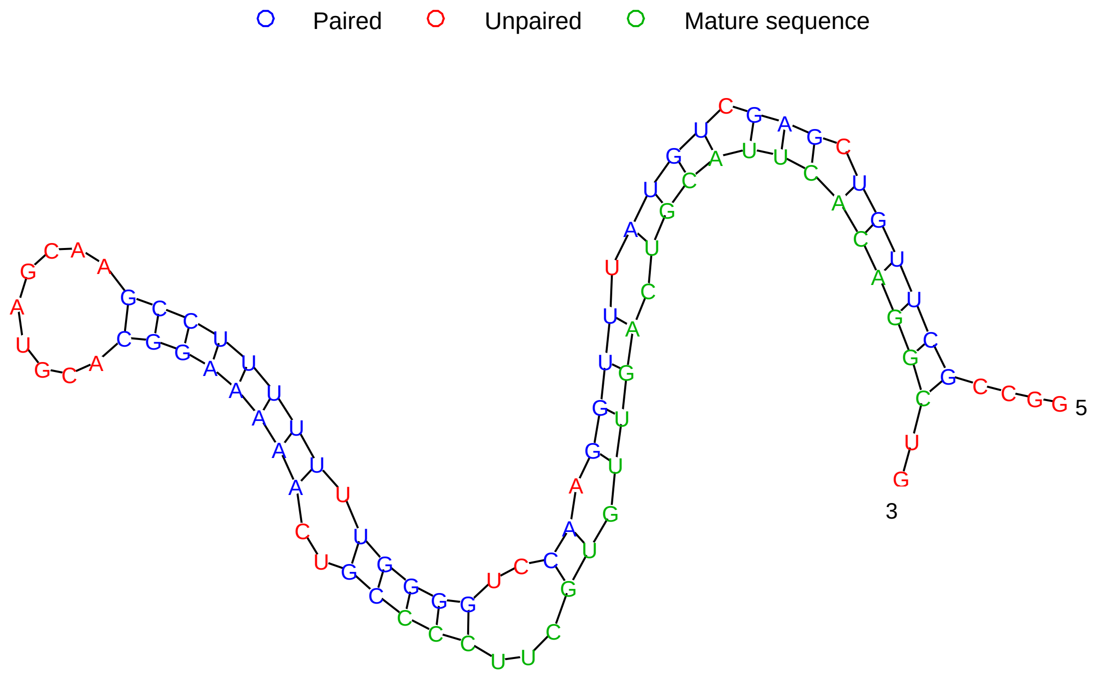

Stem loop (UMD3.1): chr8:56738512-56738604
Mature (UMD3.1): chr8:56738576-56738602
Mature seq len: 27
Total raw counts (9 samples): 1190
Average raw counts: 133
Strand: Forward
Orientation: 3p
Minimum free energy: -29.70

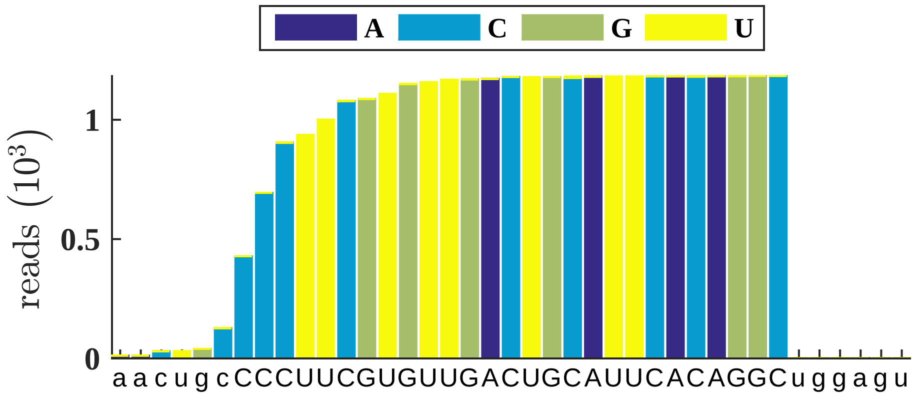

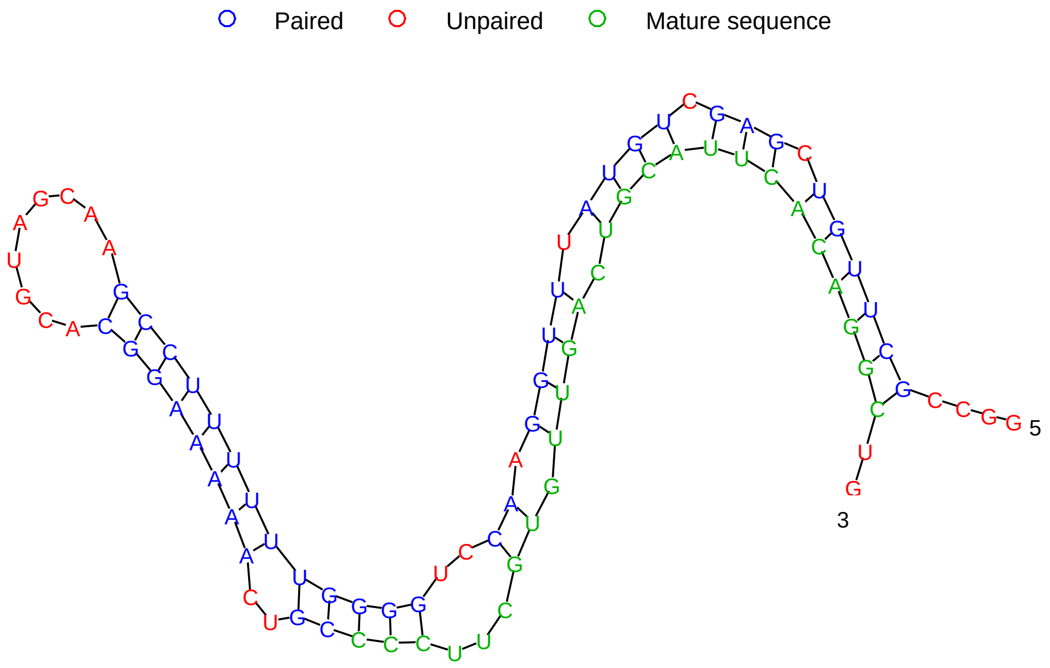

Stem loop (UMD3.1): chr8:56759886-56759977
Mature (UMD3.1): chr8:56759949-56759975
Mature seq len: 27
Total raw counts (9 samples): 1166
Average raw counts: 130
Strand: Forward
Orientation: 3p
Minimum free energy: -28.90

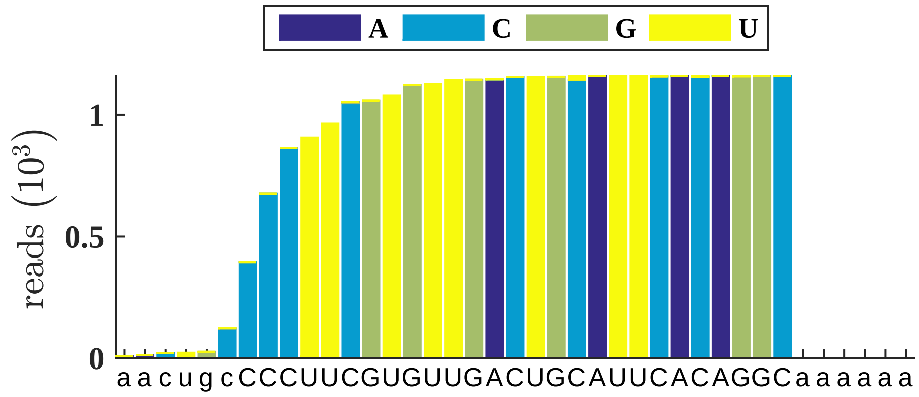

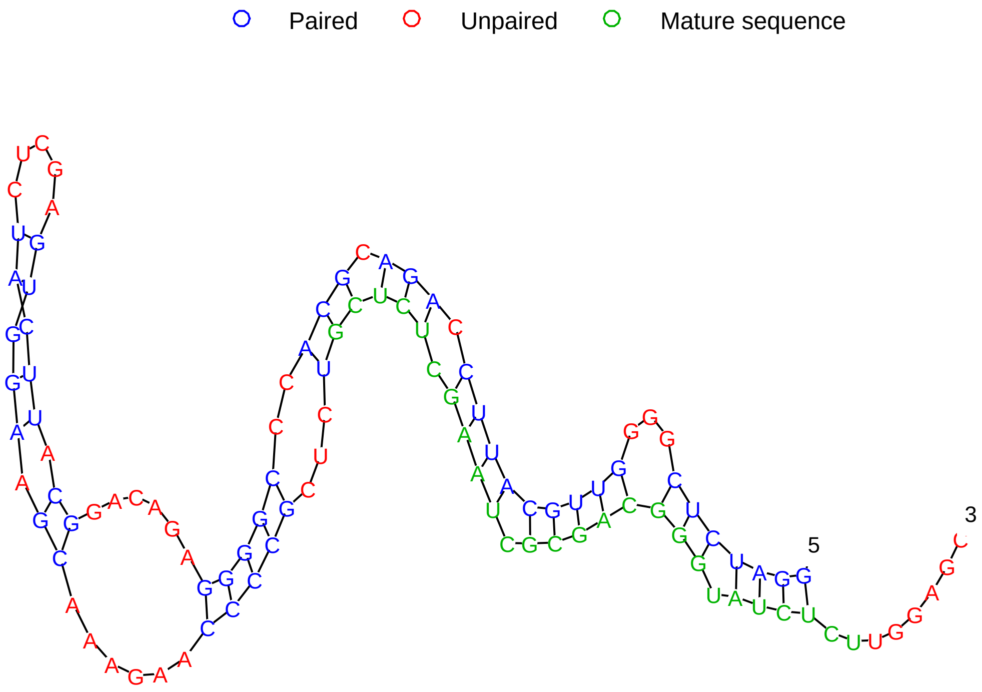

Stem loop (UMD3.1): chr8:56773321-56773428
Mature (UMD3.1): chr8:56773397-56773422
Mature seq len: 26
Total raw counts (9 samples): 4121
Average raw counts: 458
Strand: Forward
Orientation: 3p
Minimum free energy: -32.40

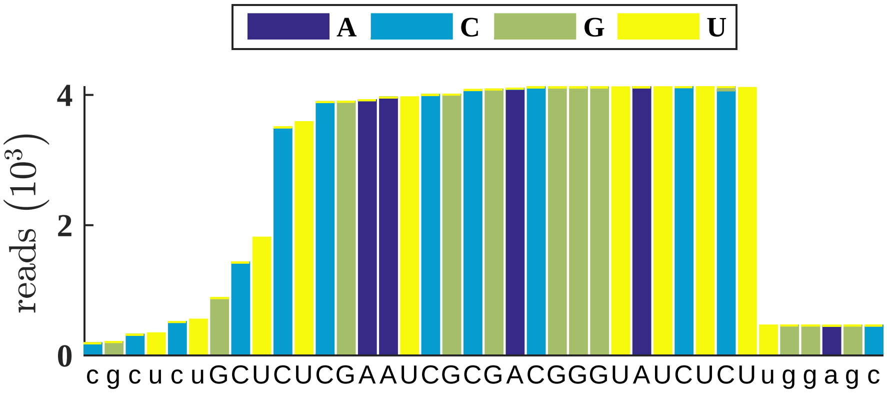

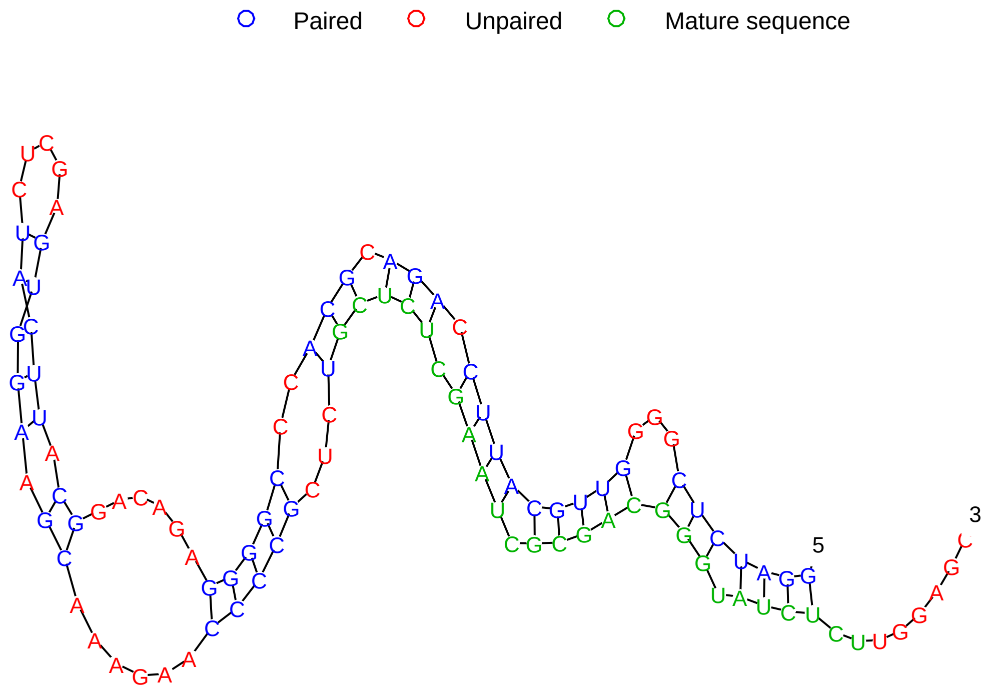

Stem loop (UMD3.1): chr8:56797985-56798092
Mature (UMD3.1): chr8:56798061-56798086
Mature seq len: 26
Total raw counts (9 samples): 3711
Average raw counts: 413
Strand: Forward
Orientation: 3p
Minimum free energy: -32.40

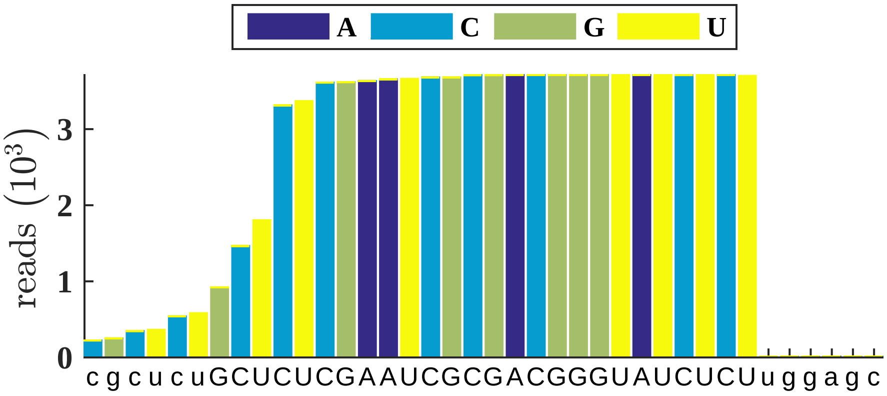

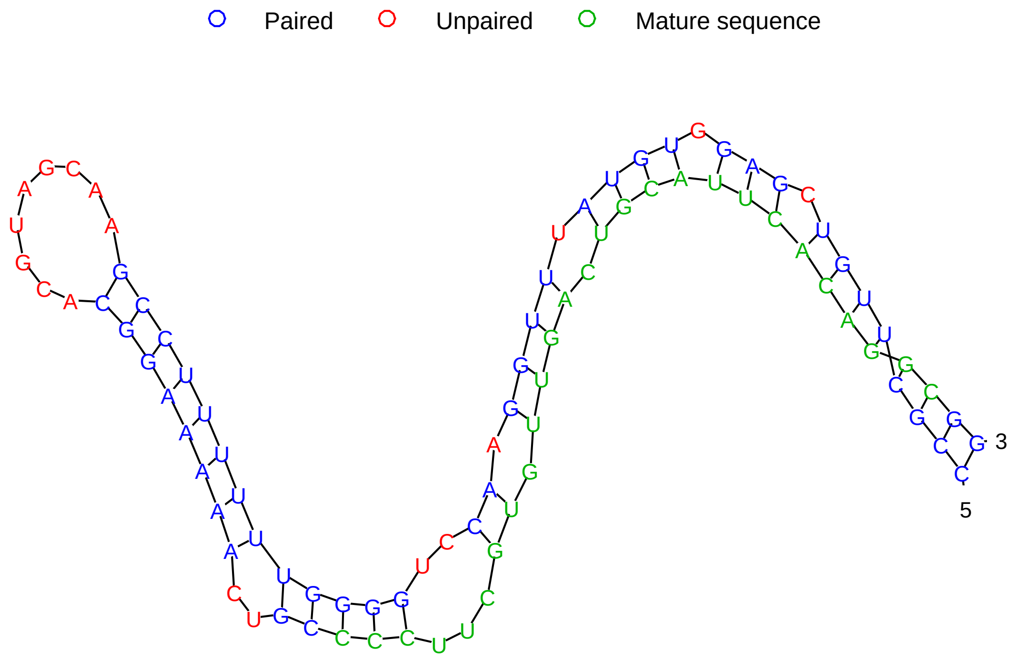

Stem loop (UMD3.1): chr8:71051072-71051161
Mature (UMD3.1): chr8:71051133-71051159
Mature seq len: 27
Total raw counts (9 samples): 1148
Average raw counts: 128
Strand: Forward
Orientation: 3p
Minimum free energy: -33.20

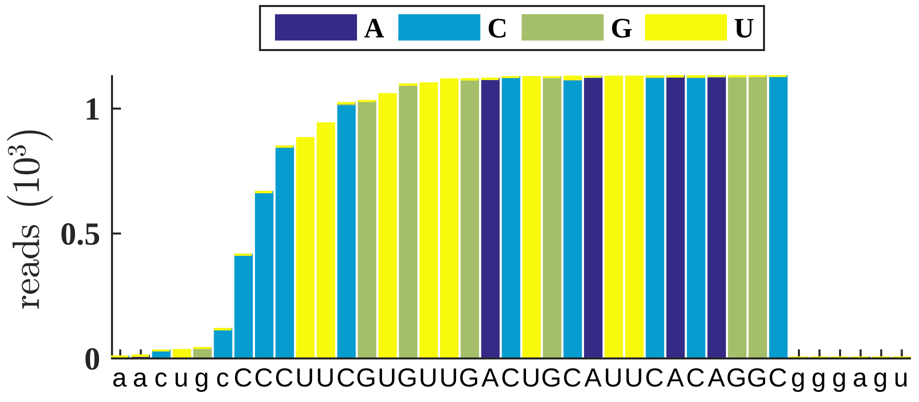

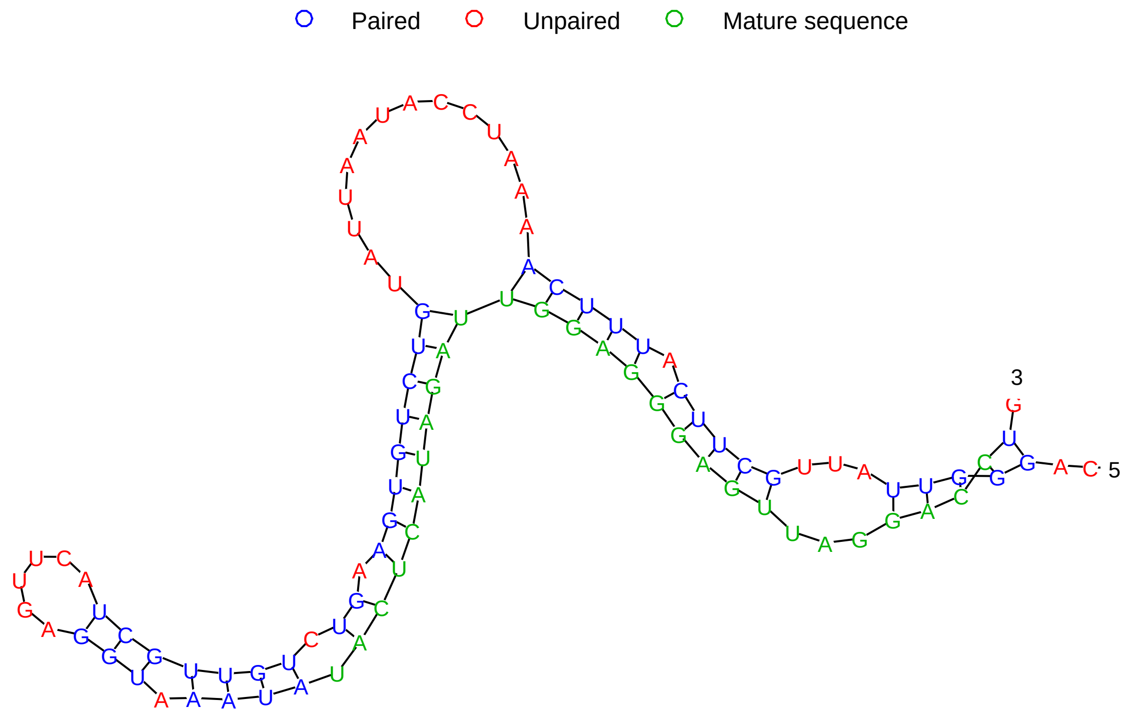

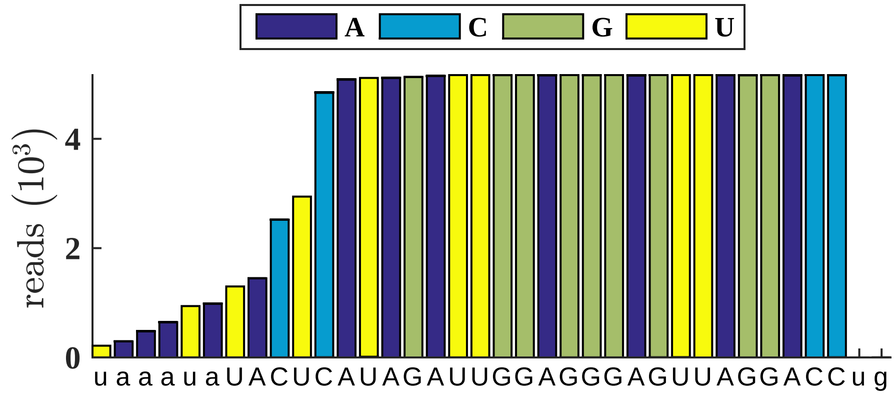

Stem loop (UMD3.1): chr8:7220773-7220870
Mature (UMD3.1): chr8:7220841-7220868
Mature seq len: 28
Total raw counts (9 samples): 5166
Average raw counts: 574
Strand: Forward
Orientation: 3p
Minimum free energy: -23.40

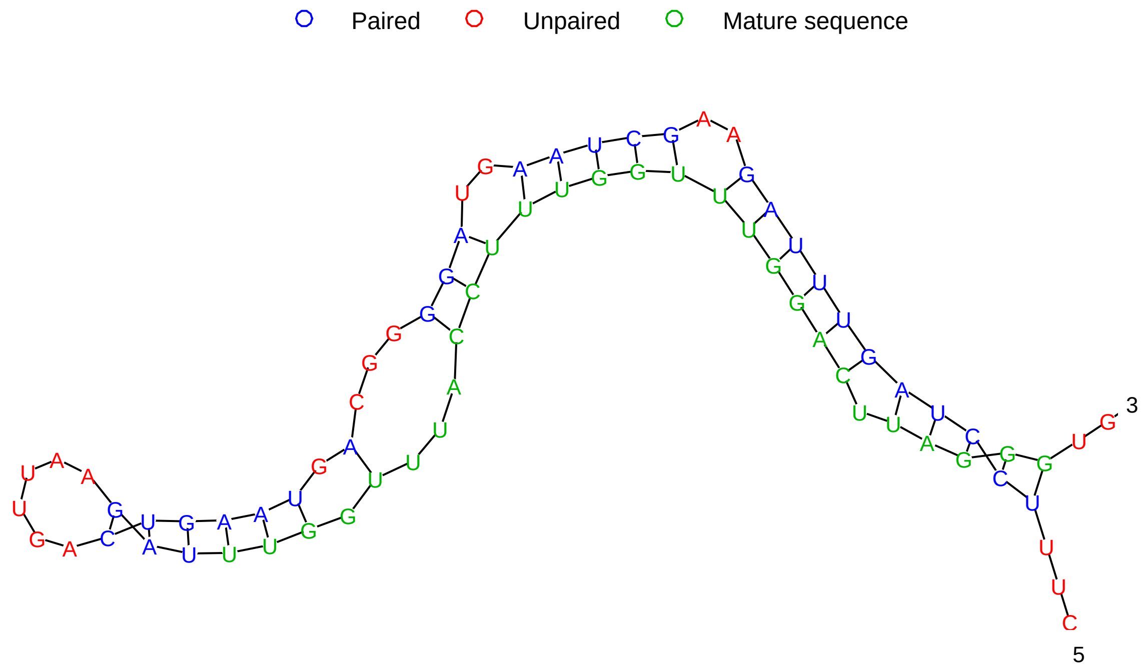

Stem loop (UMD3.1): chr8:86268737-86268812
Mature (UMD3.1): chr8:86268783-86268810
Mature seq len: 28
Total raw counts (9 samples): 824
Average raw counts: 92
Strand: Forward
Orientation: 3p
Minimum free energy: -17.90

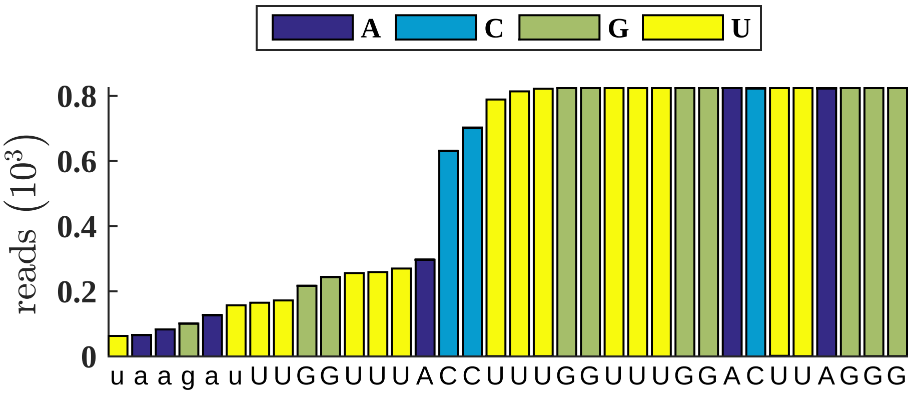

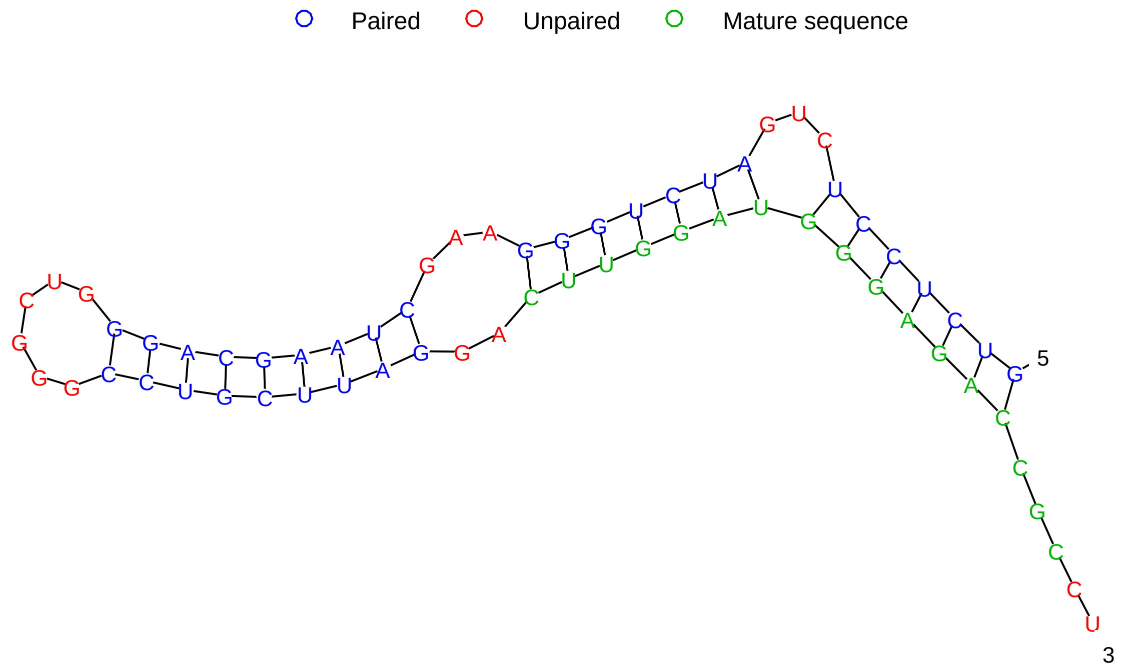

Stem loop (UMD3.1): chr8:89663120-89663184
Mature (UMD3.1): chr8:89663122-89663138
Mature seq len: 17
Total raw counts (9 samples): 2714
Average raw counts: 302
Strand: Reverse
Orientation: 3p
Minimum free energy: -31.20

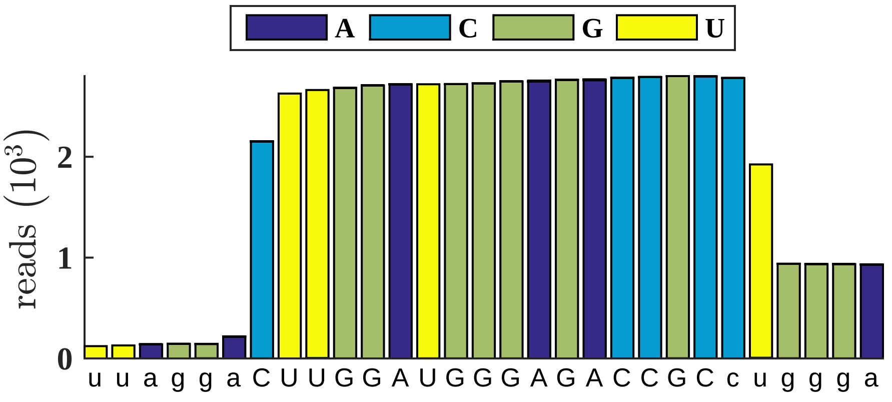

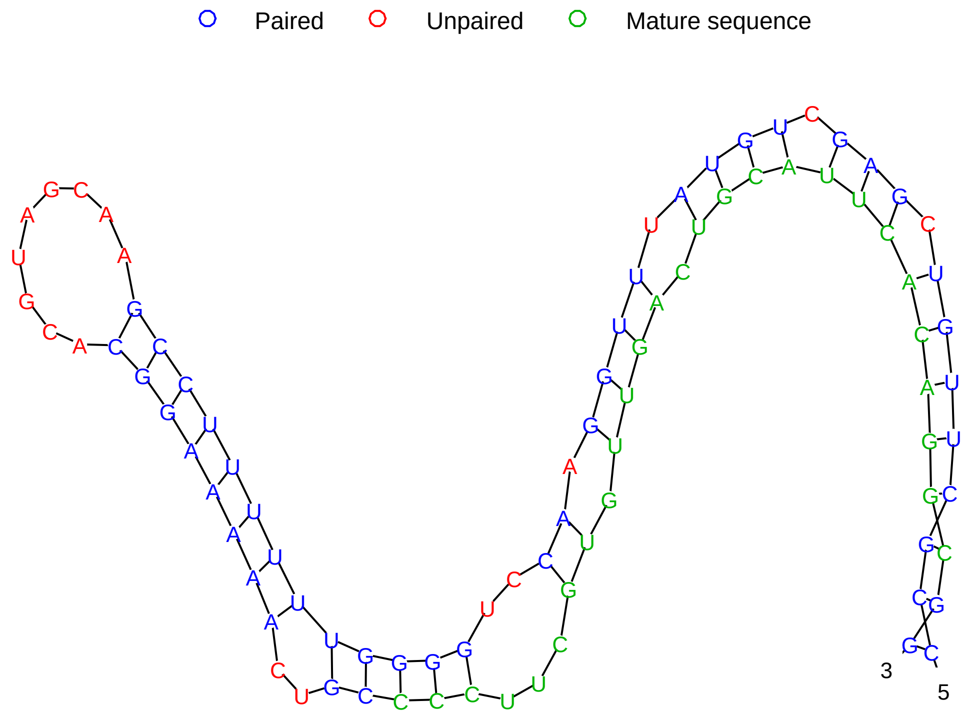

Stem loop (UMD3.1): chr8:89872663-89872752
Mature (UMD3.1): chr8:89872724-89872750
Mature seq len: 27
Total raw counts (9 samples): 1198
Average raw counts: 134
Strand: Forward
Orientation: 3p
Minimum free energy: -33.20

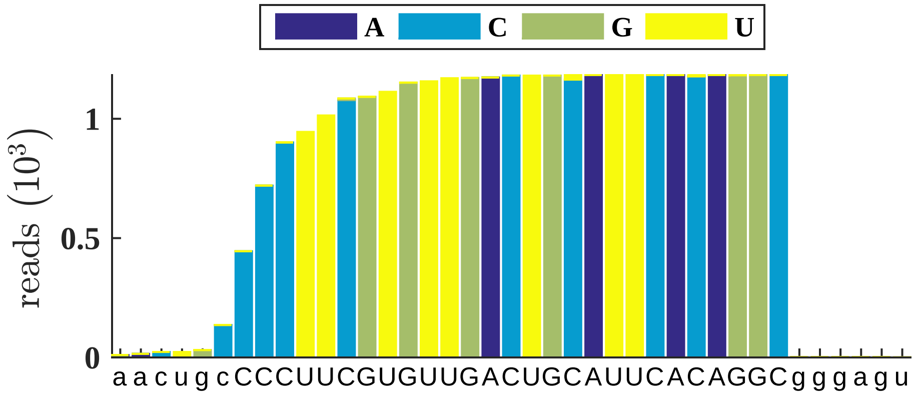

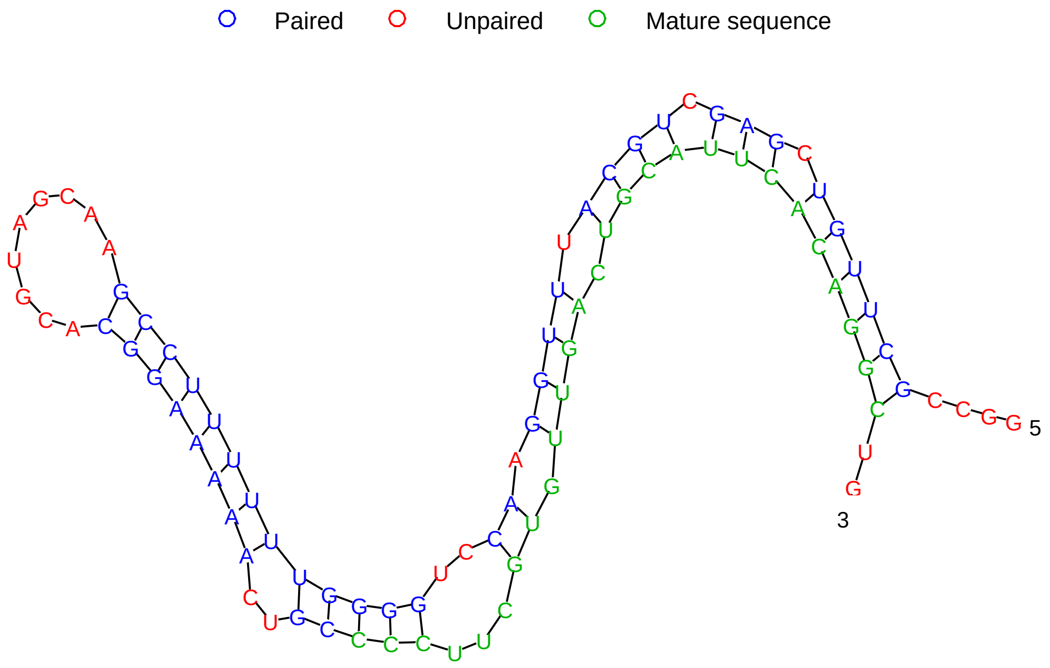

Stem loop (UMD3.1): chr8:91144099-91144190
Mature (UMD3.1): chr8:91144101-91144127
Mature seq len: 27
Total raw counts (9 samples): 1073
Average raw counts: 120
Strand: Reverse
Orientation: 3p
Minimum free energy: -30.90

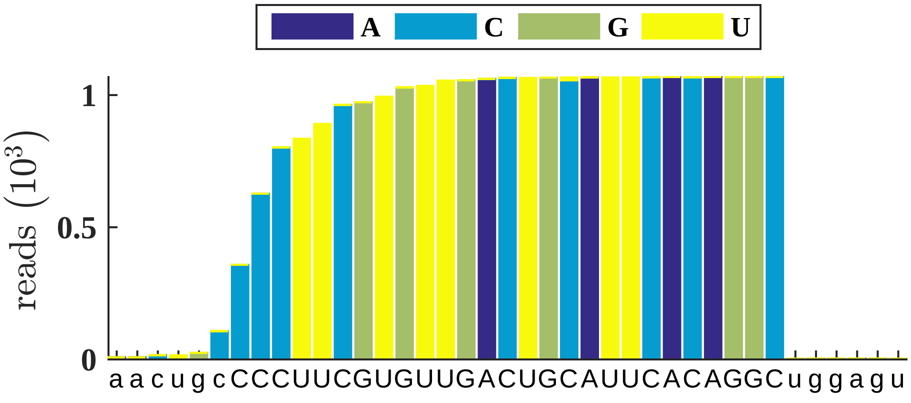

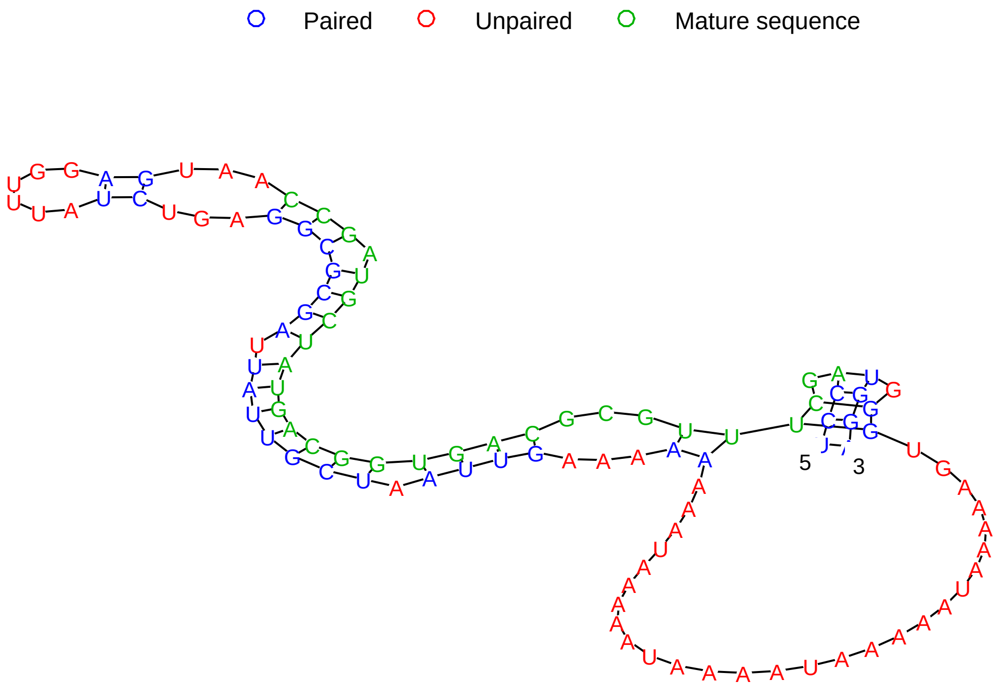

Stem loop (UMD3.1): chr9:103492645-103492751
Mature (UMD3.1): chr9:103492721-103492748
Mature seq len: 28
Total raw counts (9 samples): 746
Average raw counts: 83
Strand: Reverse
Orientation: 5p
Minimum free energy: -21.70

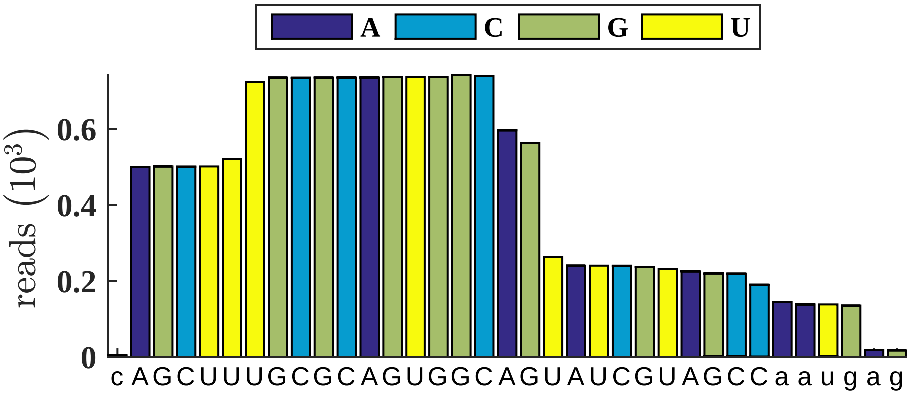

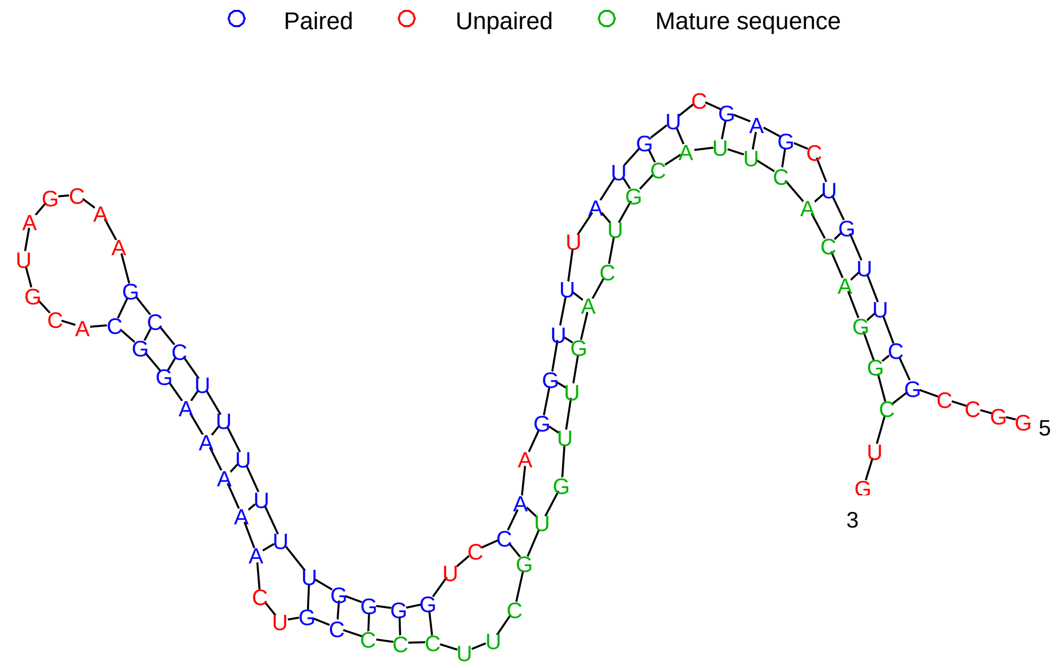

Stem loop (UMD3.1): chr9:37575814-37575905
Mature (UMD3.1): chr9:37575877-37575903
Mature seq len: 27
Total raw counts (9 samples): 1165
Average raw counts: 130
Strand: Forward
Orientation: 3p
Minimum free energy: -28.90

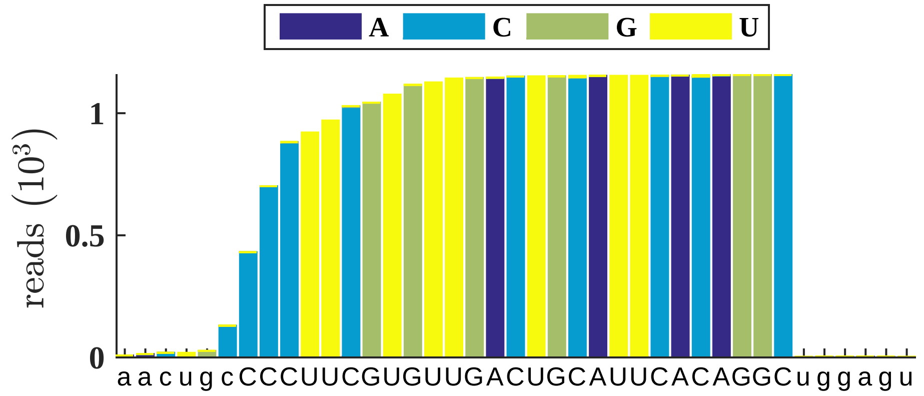

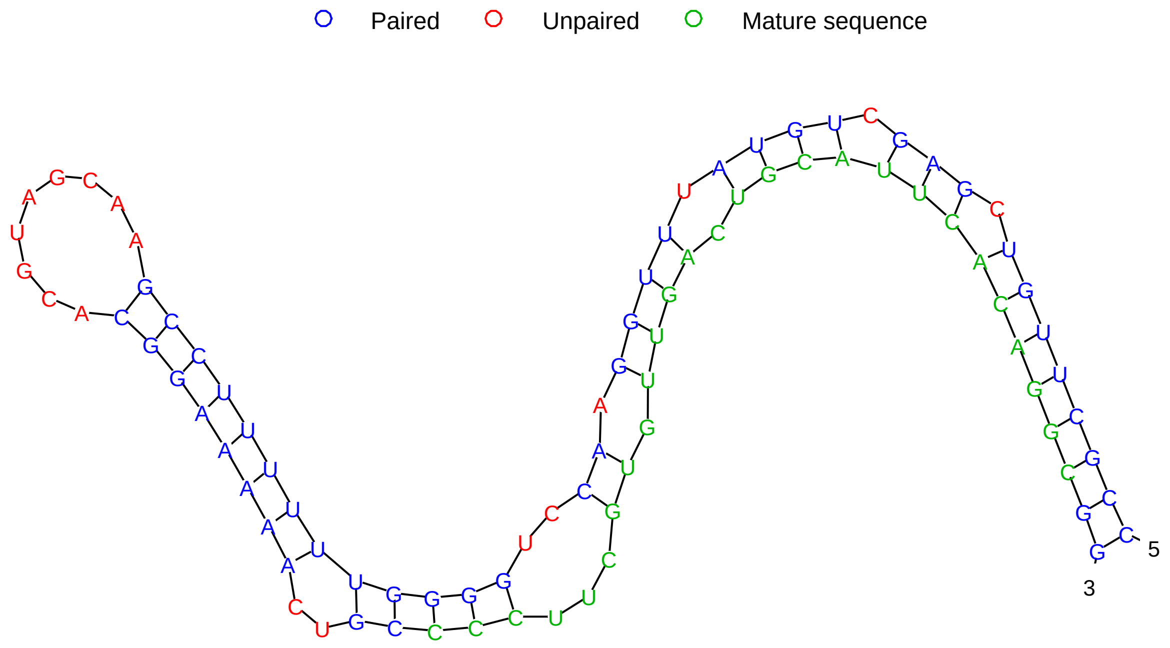

Stem loop (UMD3.1): chr9:38521723-38521812
Mature (UMD3.1): chr9:38521784-38521810
Mature seq len: 27
Total raw counts (9 samples): 1183
Average raw counts: 132
Strand: Forward
Orientation: 3p
Minimum free energy: -33.20

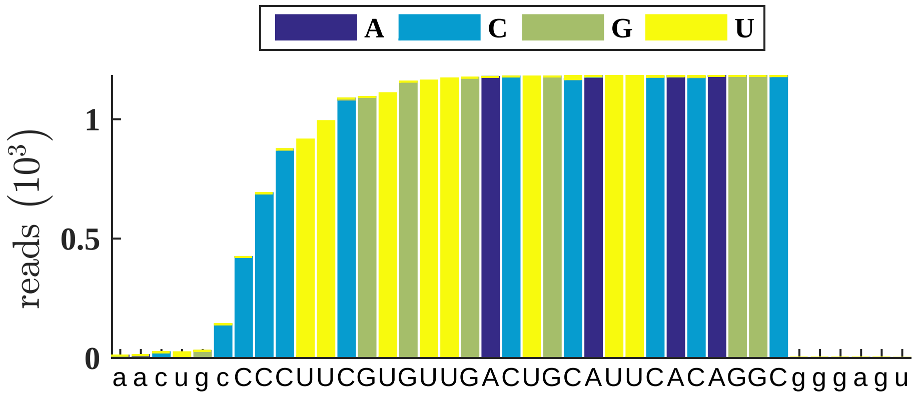

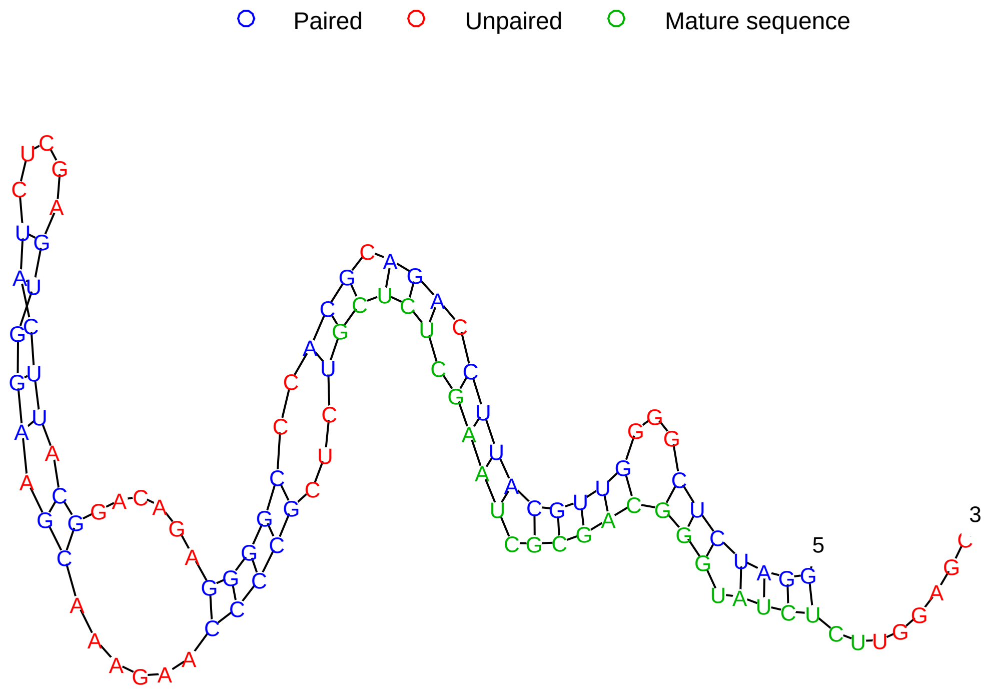

Stem loop (UMD3.1): chr9:45030053-45030160
Mature (UMD3.1): chr9:45030059-45030084
Mature seq len: 26
Total raw counts (9 samples): 3928
Average raw counts: 437
Strand: Reverse
Orientation: 3p
Minimum free energy: -32.40

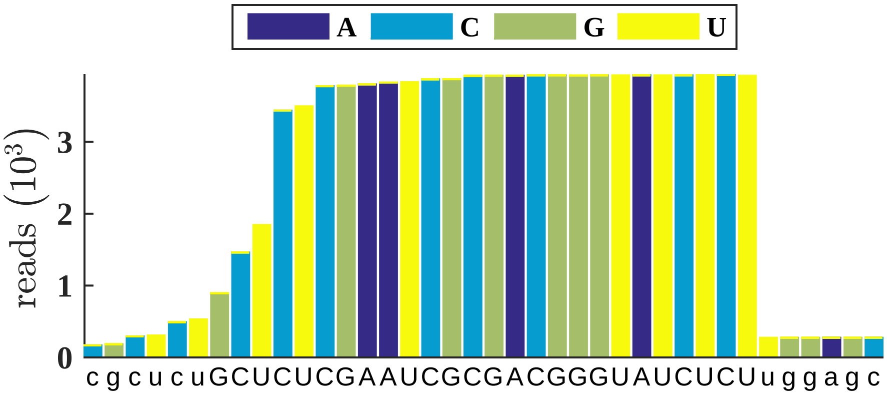

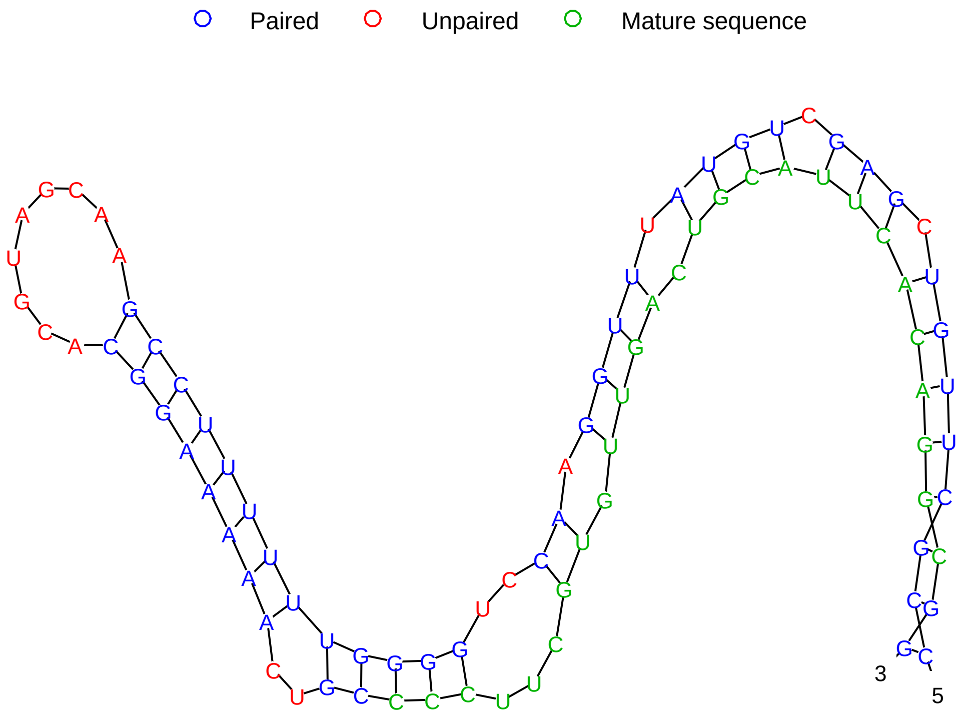

Stem loop (UMD3.1): chr9:45036153-45036242
Mature (UMD3.1): chr9:45036155-45036181
Mature seq len: 27
Total raw counts (9 samples): 1109
Average raw counts: 124
Strand: Reverse
Orientation: 3p
Minimum free energy: -33.20

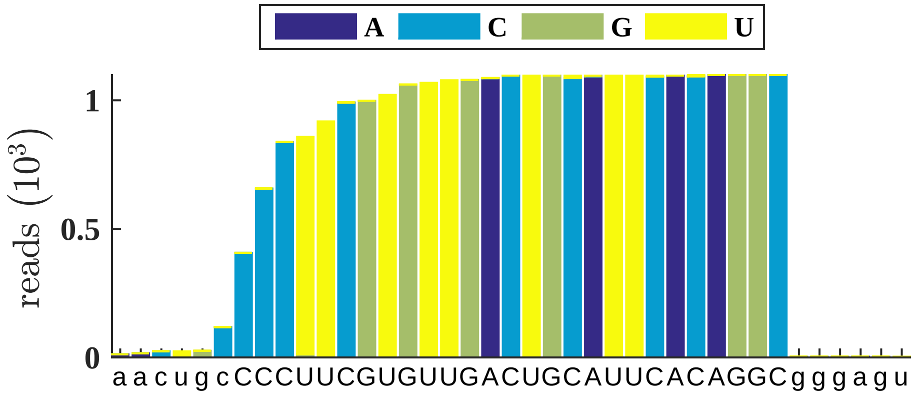

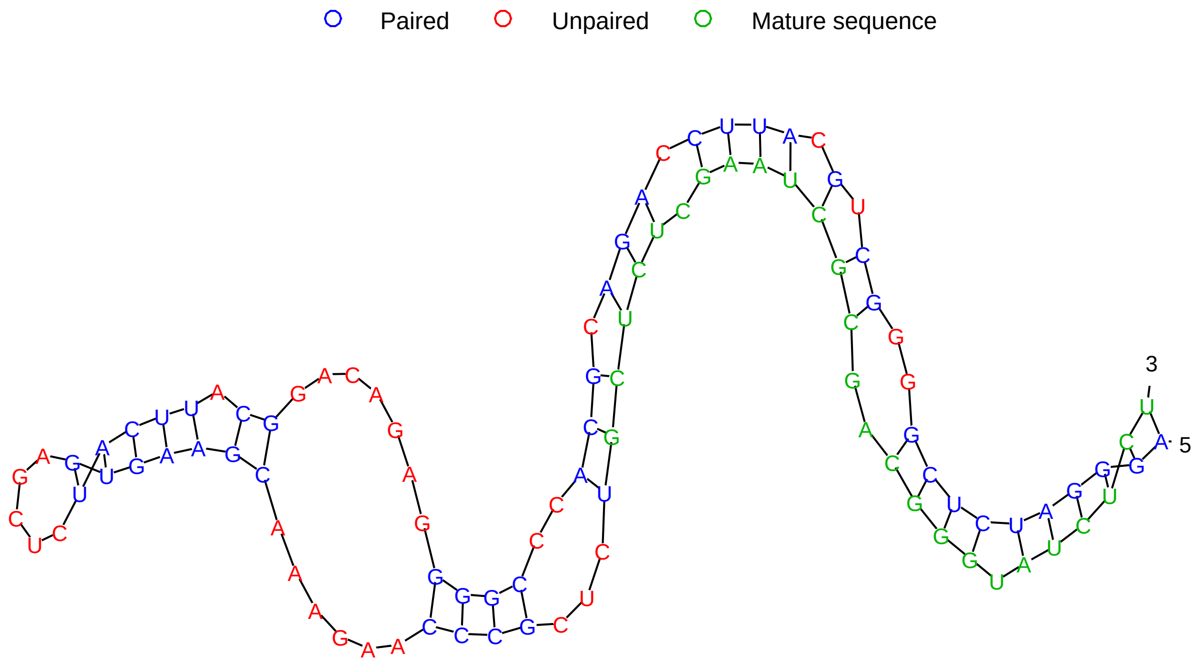

Stem loop (UMD3.1): chr9:45088035-45088136
Mature (UMD3.1): chr9:45088035-45088060
Mature seq len: 26
Total raw counts (9 samples): 3624
Average raw counts: 403
Strand: Reverse
Orientation: 3p
Minimum free energy: -33.40

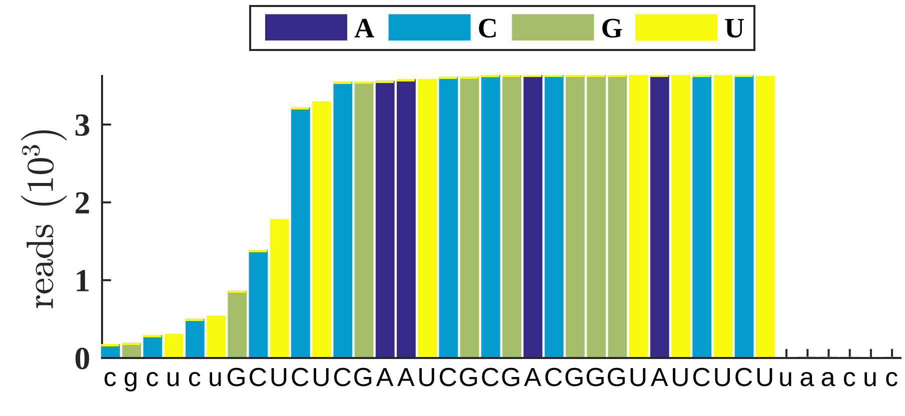

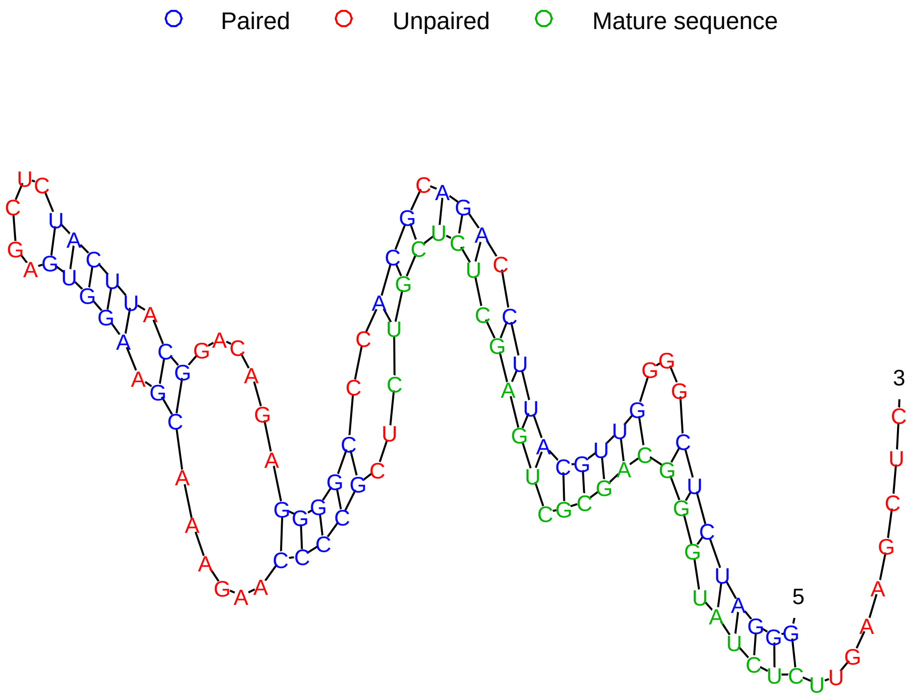

Stem loop (UMD3.1): chr9:53549501-53549611
Mature (UMD3.1): chr9:53549576-53549603
Mature seq len: 28
Total raw counts (9 samples): 12976
Average raw counts: 1442
Strand: Forward
Orientation: 3p
Minimum free energy: -35.50

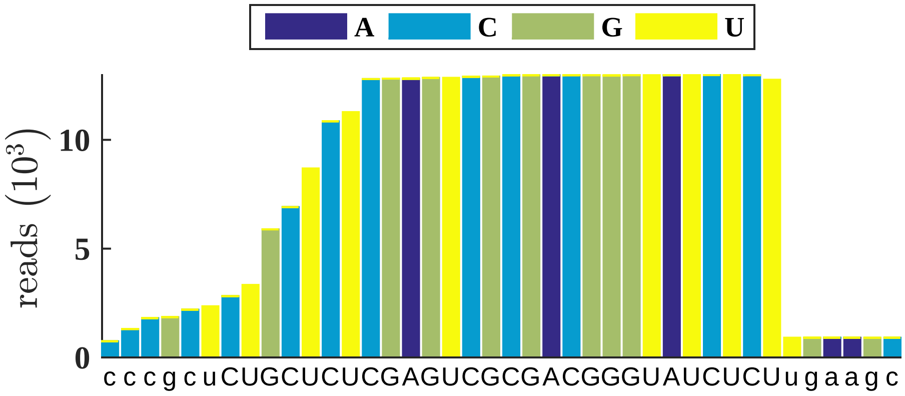

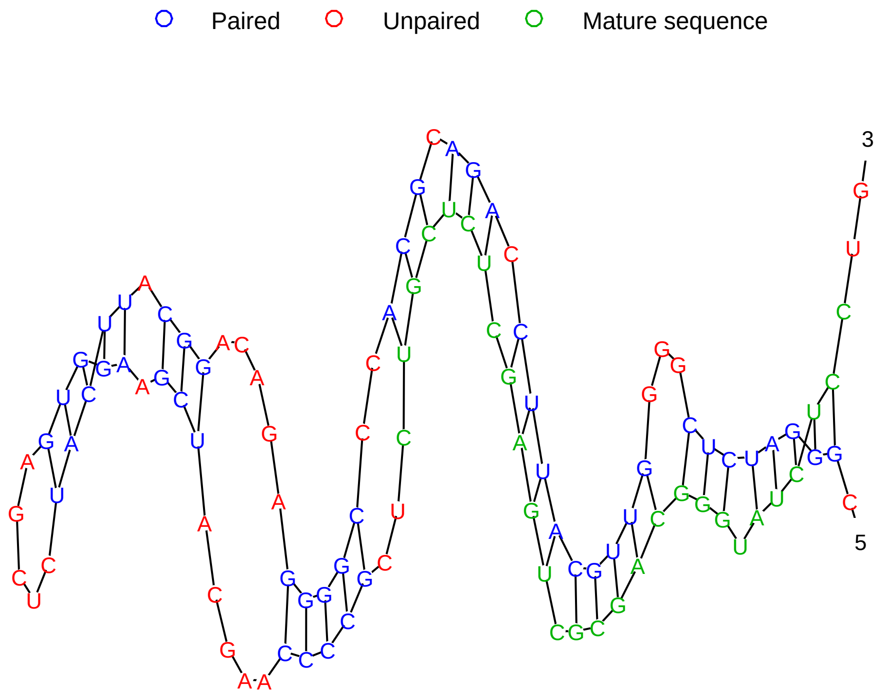

Stem loop (UMD3.1): chr9:53554905-53555010
Mature (UMD3.1): chr9:53554981-53555008
Mature seq len: 28
Total raw counts (9 samples): 1725
Average raw counts: 192
Strand: Forward
Orientation: 3p
Minimum free energy: -36.20

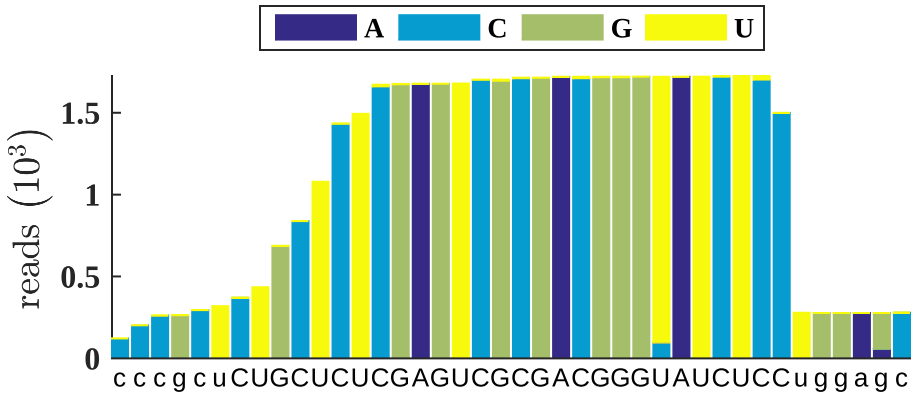

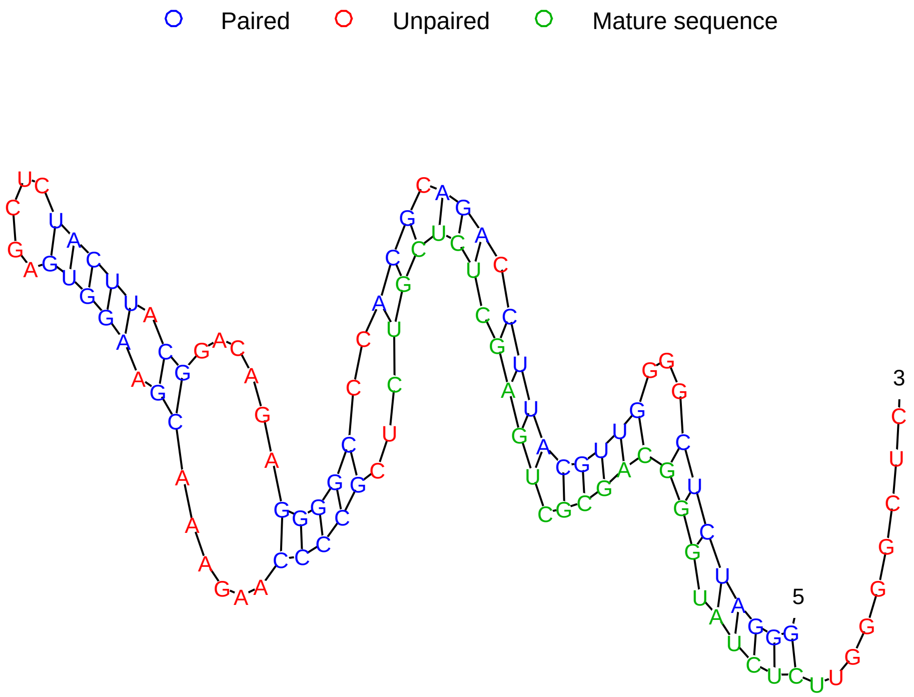

Stem loop (UMD3.1): chr9:53573398-53573508
Mature (UMD3.1): chr9:53573406-53573433
Mature seq len: 28
Total raw counts (9 samples): 12387
Average raw counts: 1377
Strand: Reverse
Orientation: 3p
Minimum free energy: -35.50

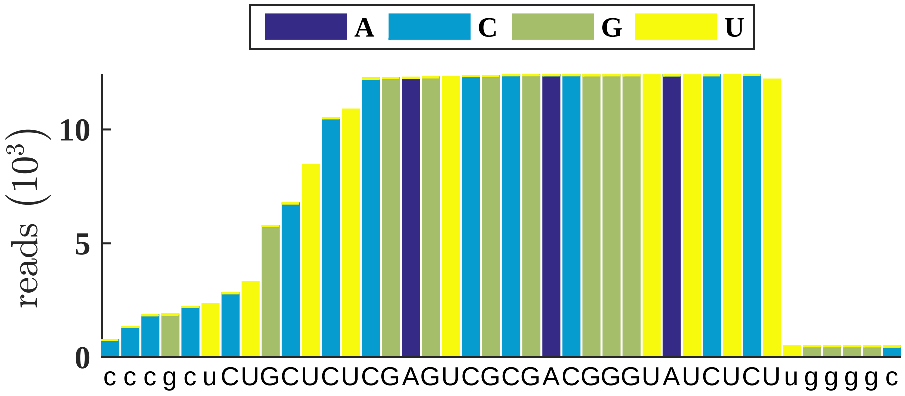

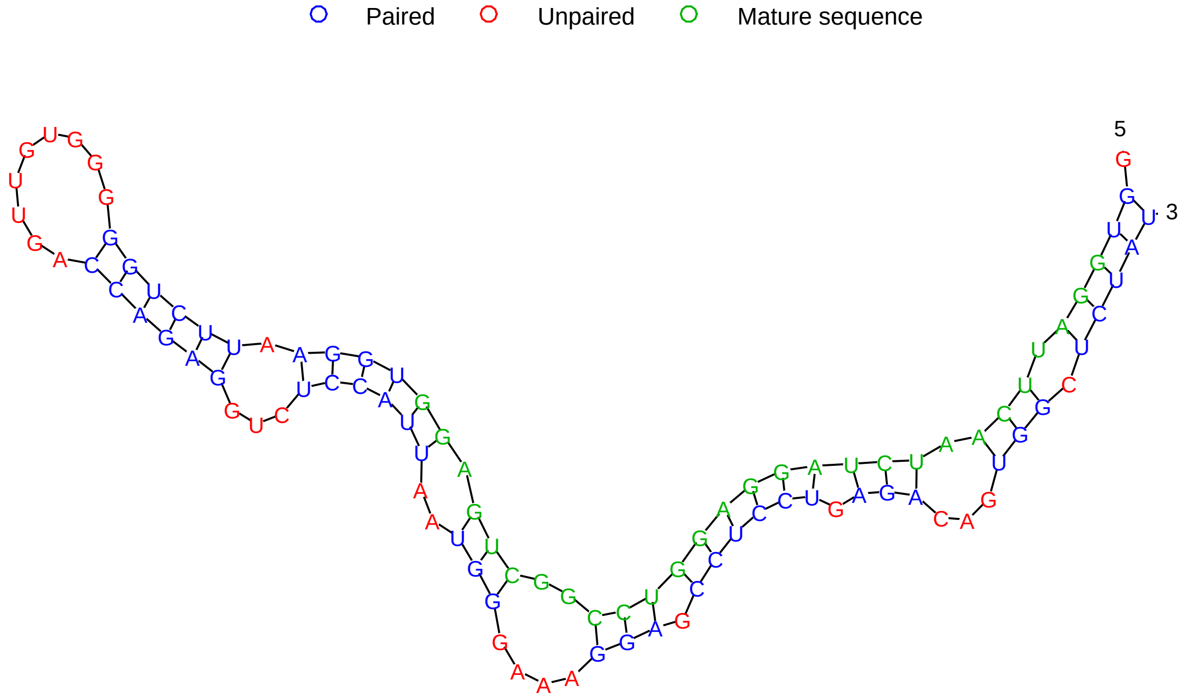

Stem loop (UMD3.1): chr9:72616253-72616353
Mature (UMD3.1): chr9:72616256-72616283
Mature seq len: 28
Total raw counts (9 samples): 949
Average raw counts: 106
Strand: Forward
Orientation: 5p
Minimum free energy: -34.80

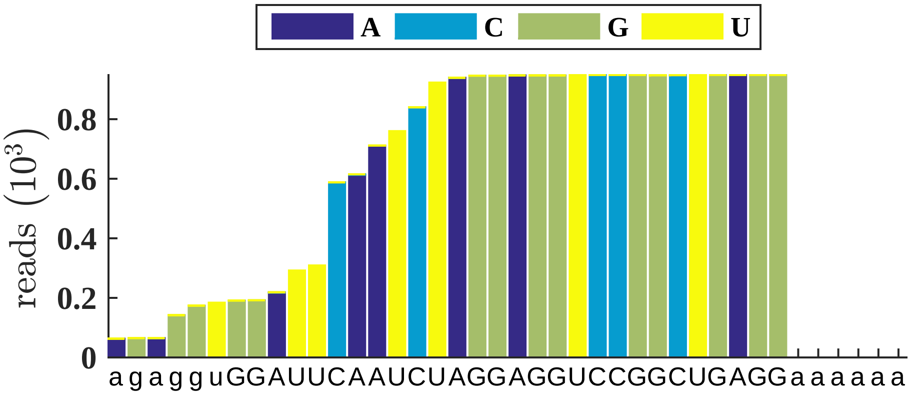

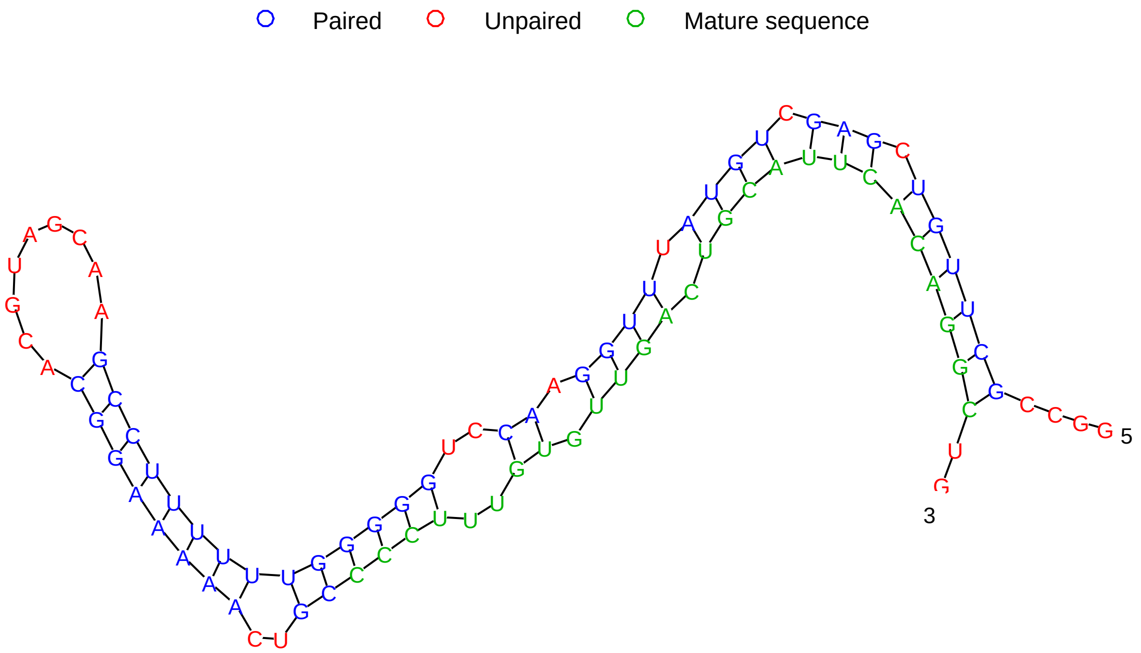

Stem loop (UMD3.1): chr9:81641299-81641391
Mature (UMD3.1): chr9:81641363-81641389
Mature seq len: 27
Total raw counts (9 samples): 1715
Average raw counts: 191
Strand: Forward
Orientation: 3p
Minimum free energy: -31.20

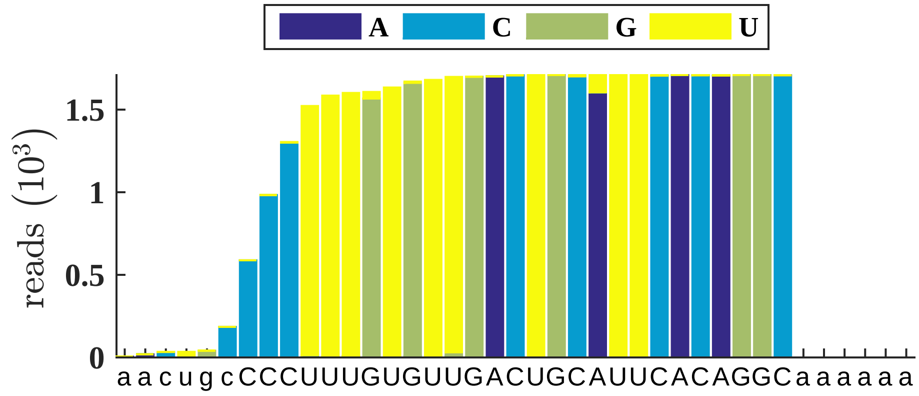

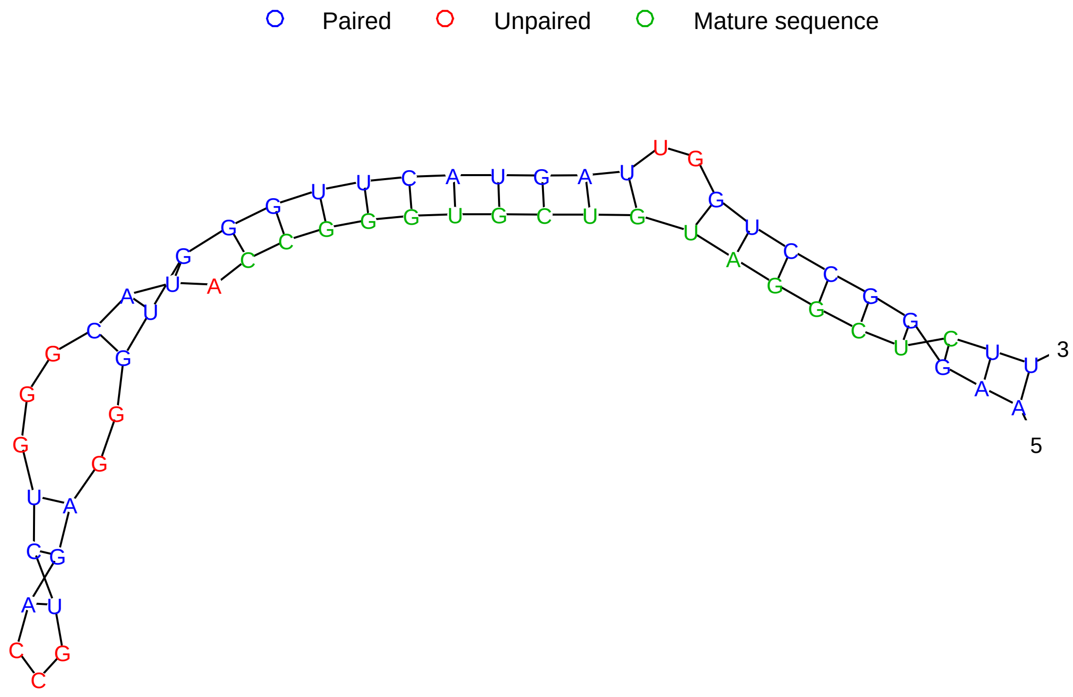

Stem loop (UMD3.1): chr9:94314511-94314571
Mature (UMD3.1): chr9:94314513-94314529
Mature seq len: 17
Total raw counts (9 samples): 5182
Average raw counts: 576
Strand: Reverse
Orientation: 3p
Minimum free energy: -26.10

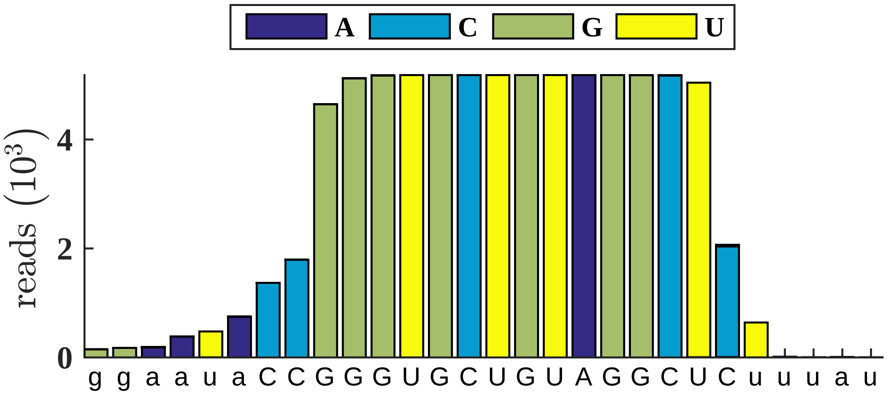

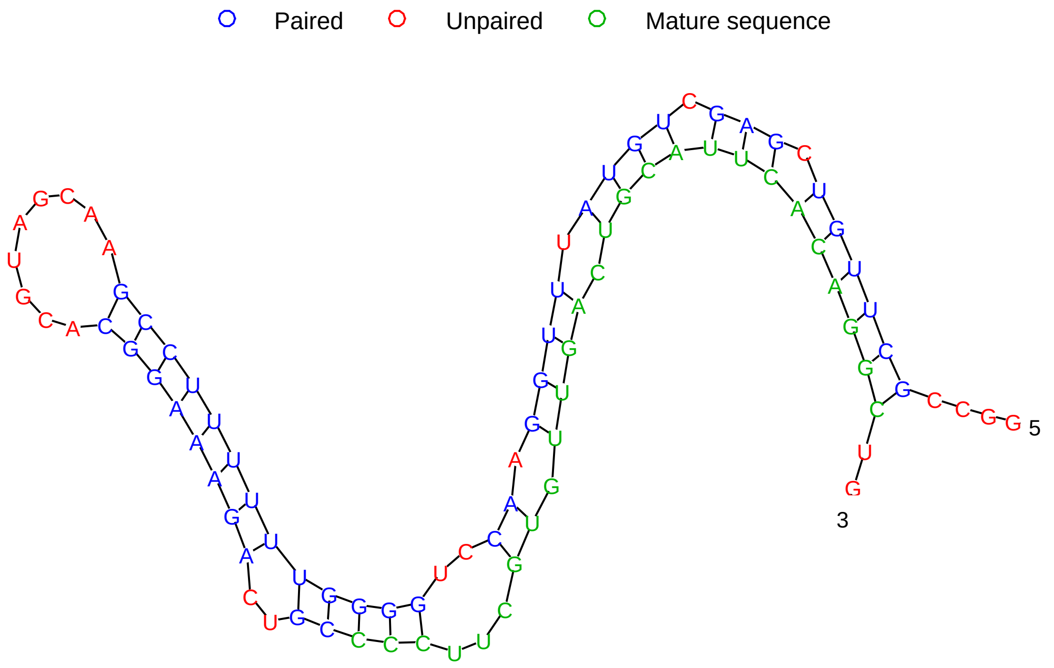

Stem loop (UMD3.1): chrX:100087844-100087935
Mature (UMD3.1): chrX:100087846-100087872
Mature seq len: 27
Total raw counts (9 samples): 1236
Average raw counts: 138
Strand: Reverse
Orientation: 3p
Minimum free energy: -29.00

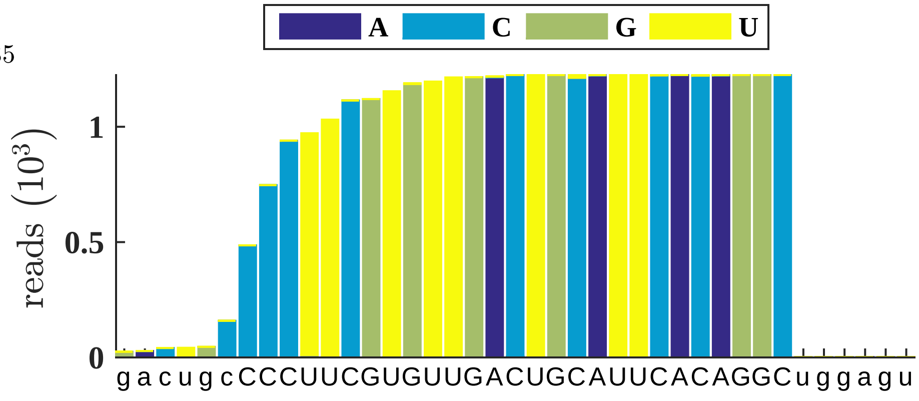

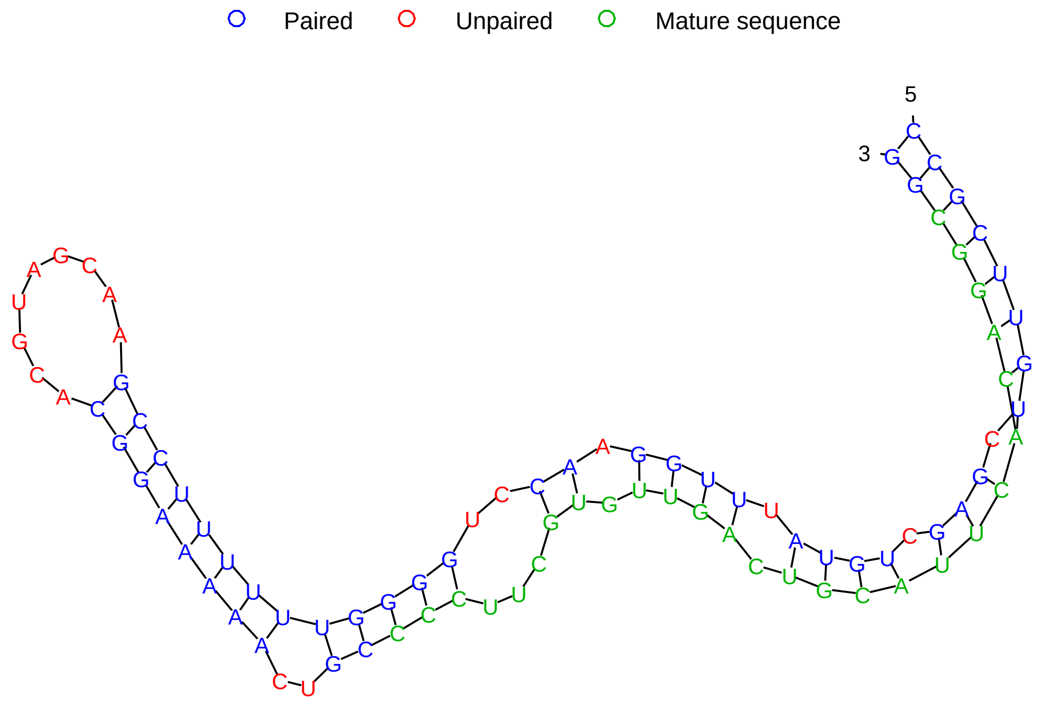

Stem loop (UMD3.1): chrX:100089450-100089539
Mature (UMD3.1): chrX:100089452-100089478
Mature seq len: 27
Total raw counts (9 samples): 1228
Average raw counts: 137
Strand: Reverse
Orientation: 3p
Minimum free energy: -33.20

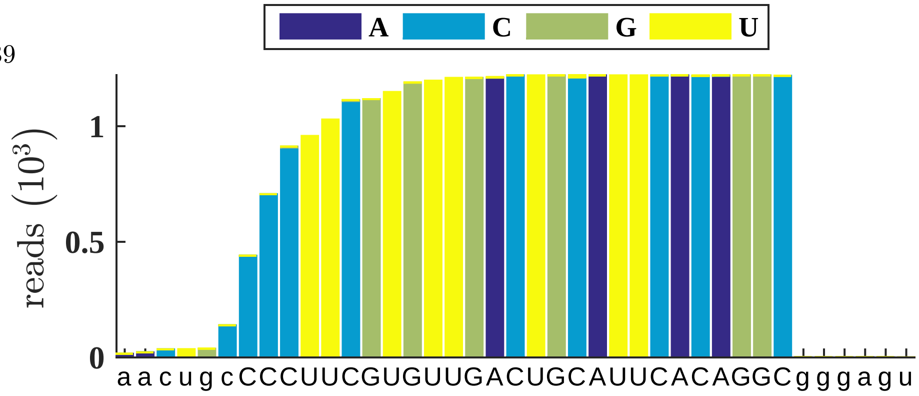

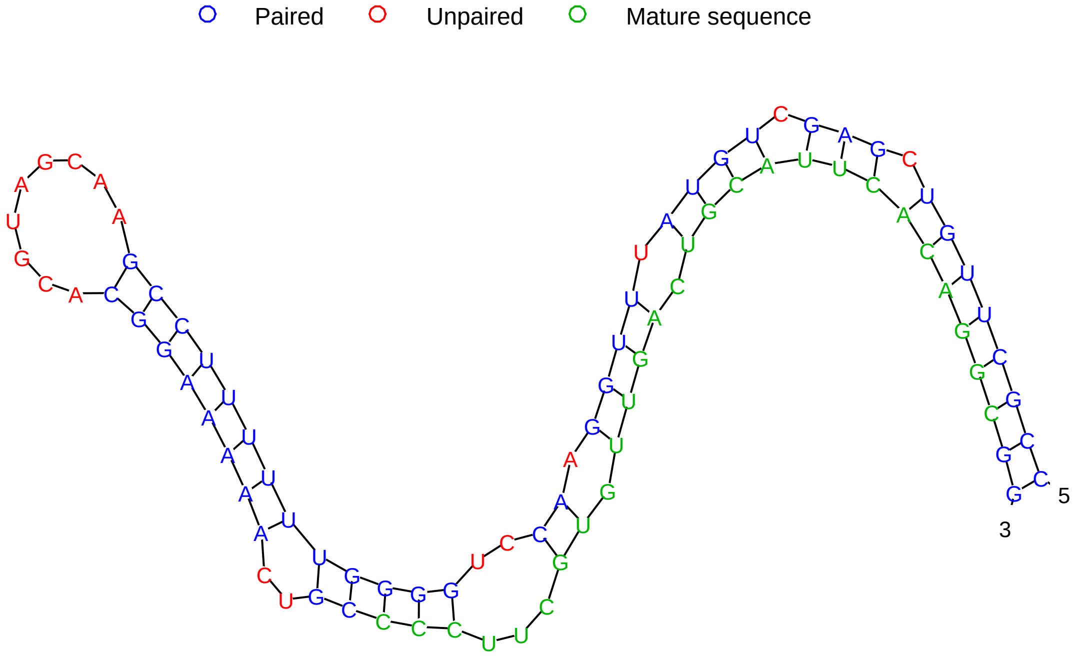

Stem loop (UMD3.1): chrX:100090839-100090928
Mature (UMD3.1): chrX:100090841-100090867
Mature seq len: 27
Total raw counts (9 samples): 1171
Average raw counts: 131
Strand: Reverse
Orientation: 3p
Minimum free energy: -33.20

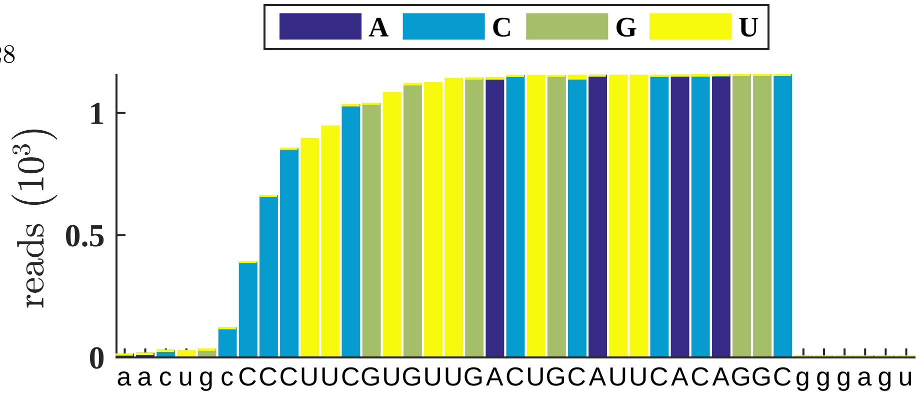

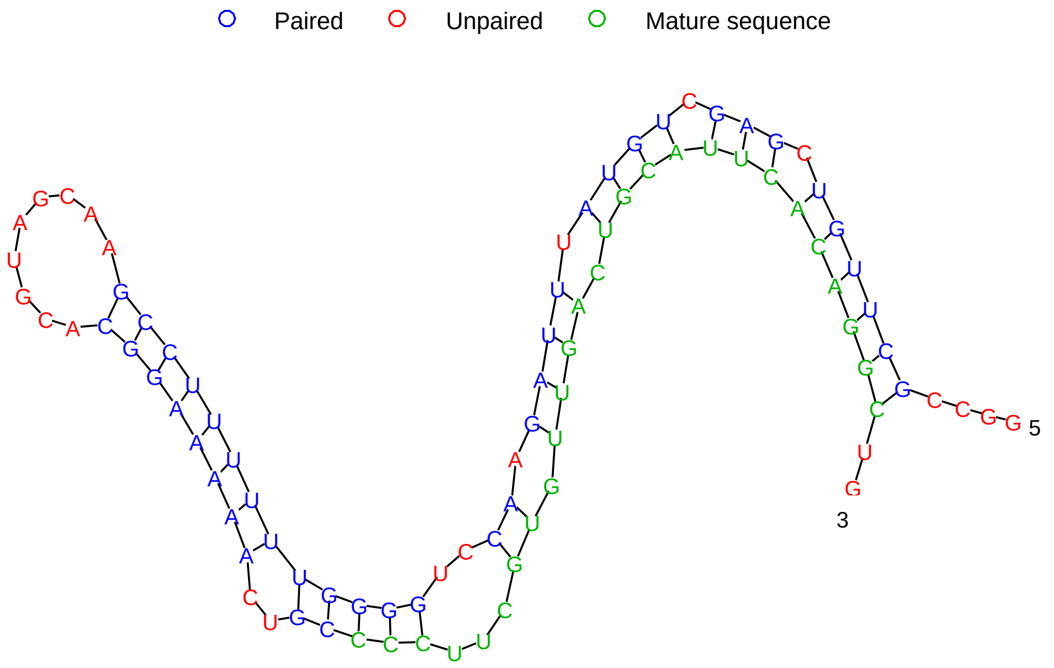

Stem loop (UMD3.1): chrX:100093177-100093268
Mature (UMD3.1): chrX:100093179-100093205
Mature seq len: 27
Total raw counts (9 samples): 1177
Average raw counts: 131
Strand: Reverse
Orientation: 3p
Minimum free energy: -30.30

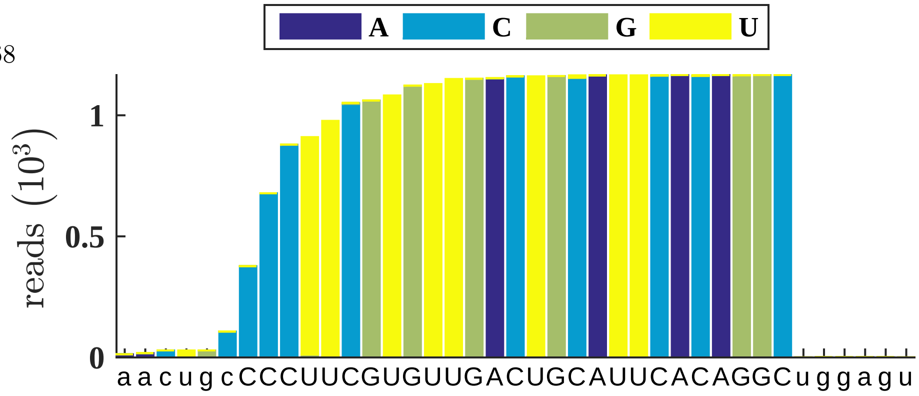

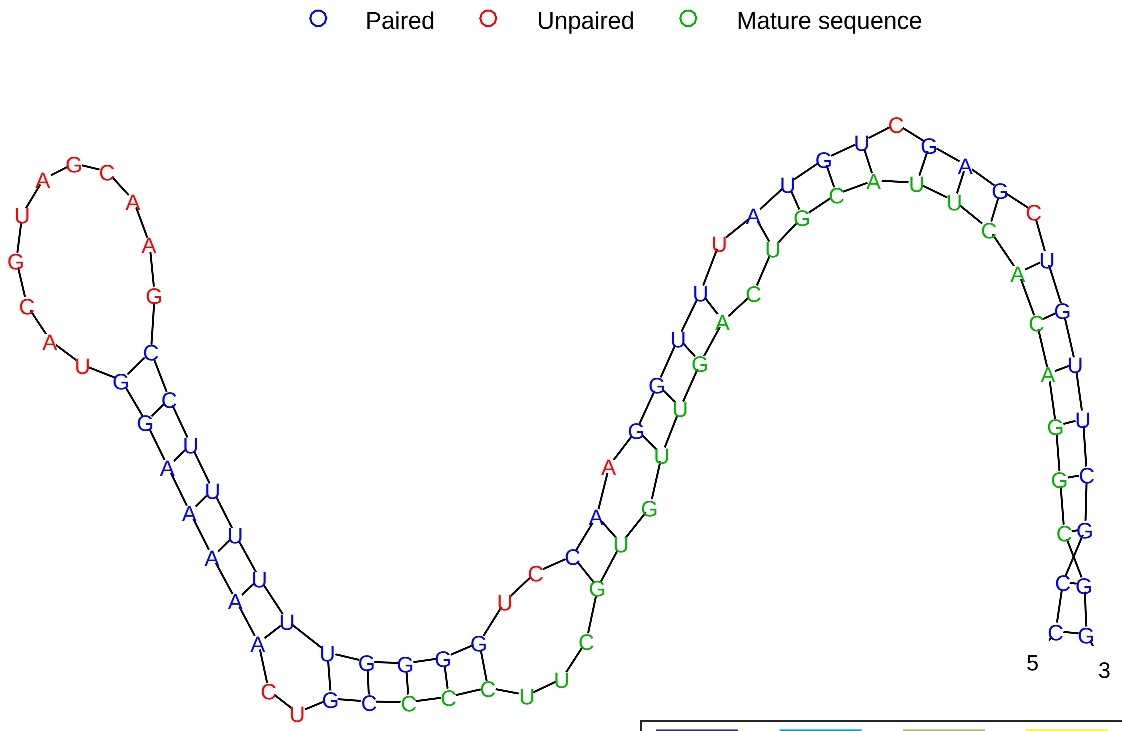

Stem loop (UMD3.1): chrX:100095068-100095157
Mature (UMD3.1): chrX:100095070-100095096
Mature seq len: 27
Total raw counts (9 samples): 1157
Average raw counts: 129
Strand: Reverse
Orientation: 3p
Minimum free energy: -31.00

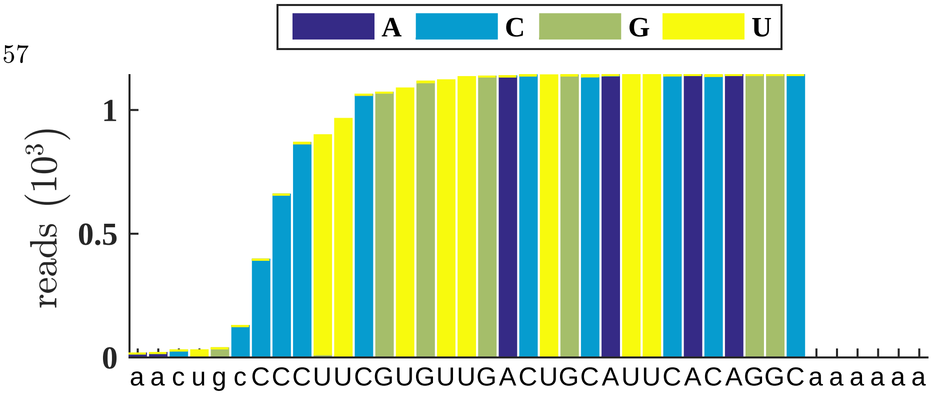

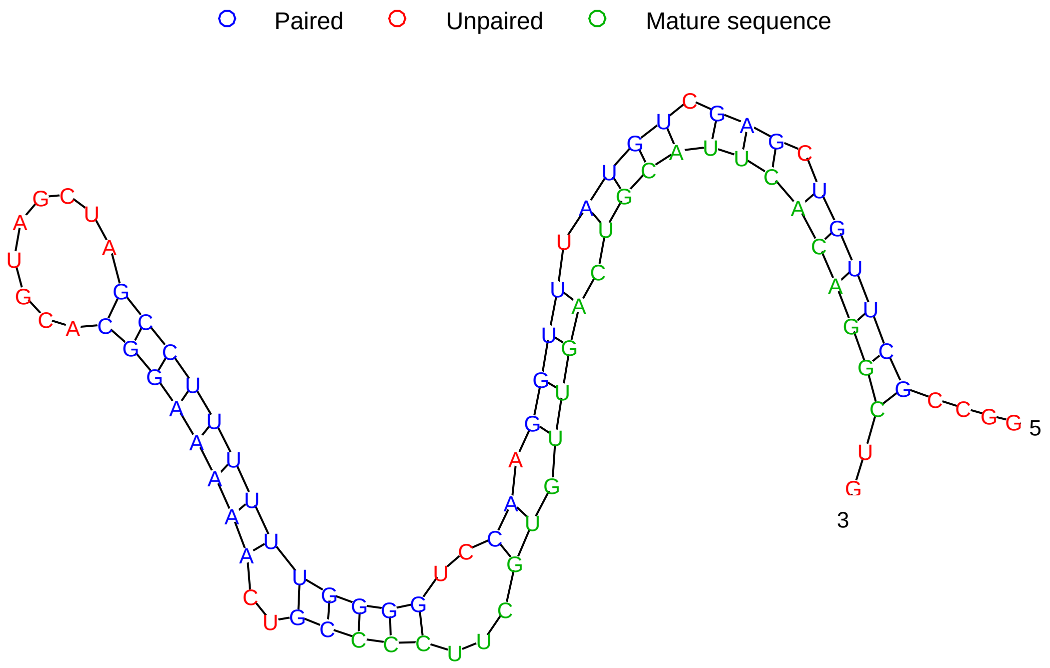

Stem loop (UMD3.1): chrX:100098825-100098916
Mature (UMD3.1): chrX:100098827-100098853
Mature seq len: 27
Total raw counts (9 samples): 1246
Average raw counts: 139
Strand: Reverse
Orientation: 3p
Minimum free energy: -28.90

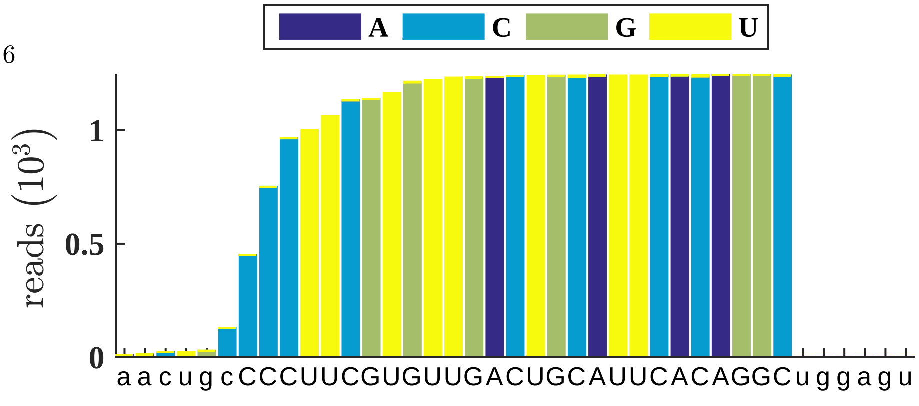

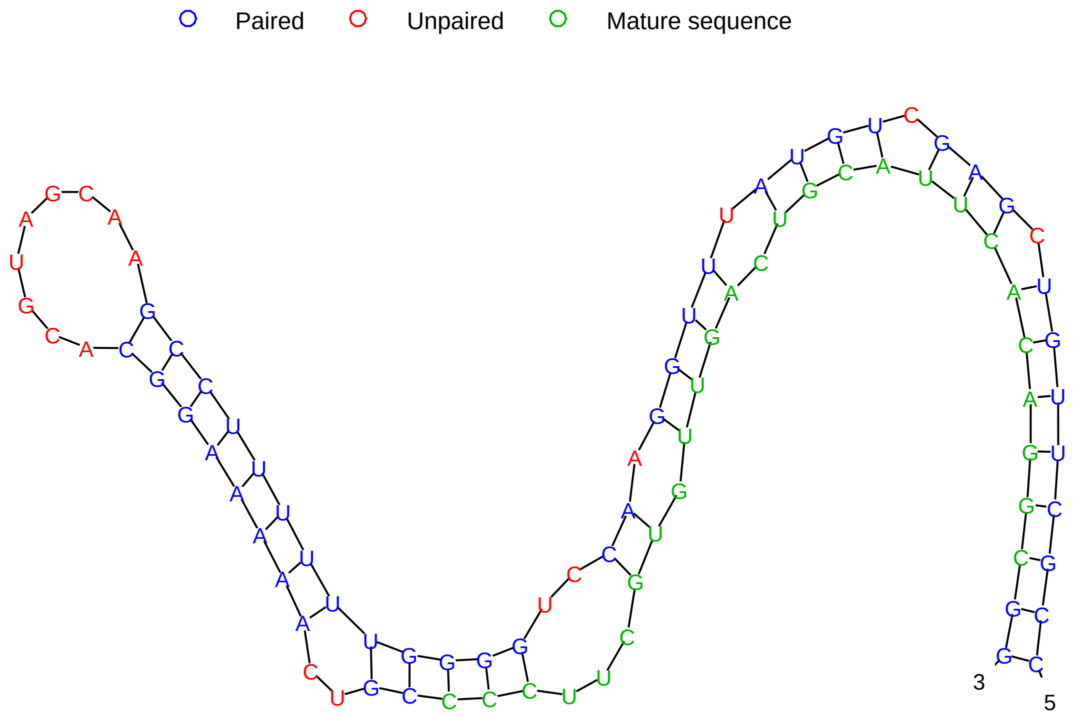

Stem loop (UMD3.1): chrX:100101280-100101369
Mature (UMD3.1): chrX:100101282-100101308
Mature seq len: 27
Total raw counts (9 samples): 1189
Average raw counts: 133
Strand: Reverse
Orientation: 3p
Minimum free energy: -33.20

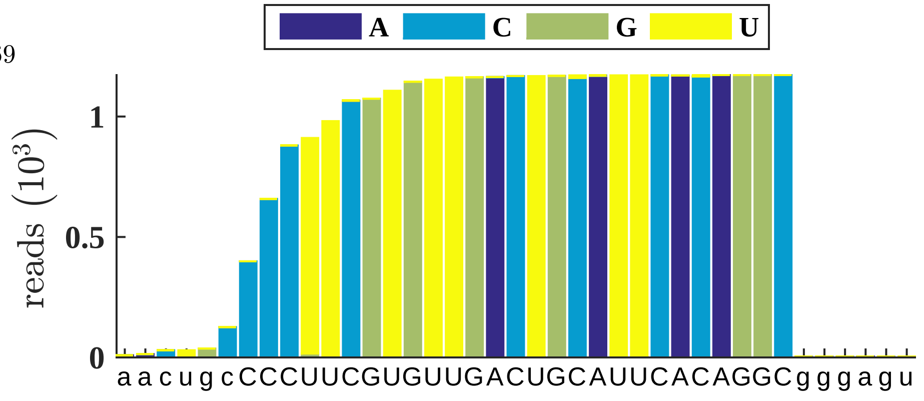

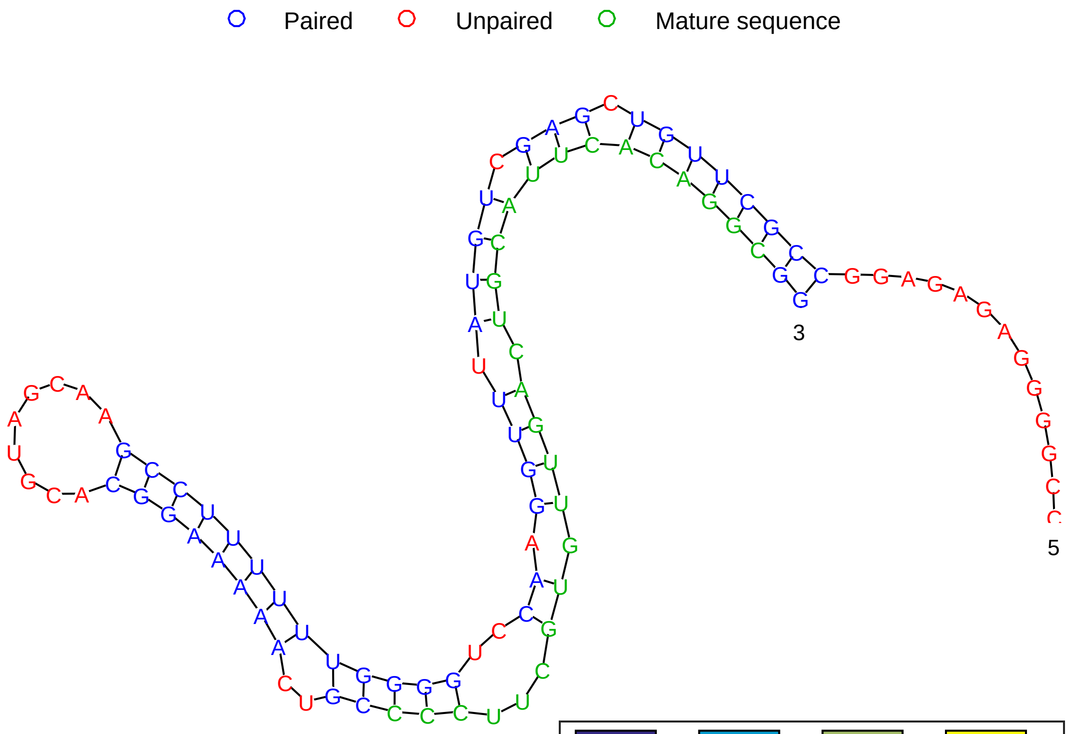

Stem loop (UMD3.1): chrX:100103800-100103902
Mature (UMD3.1): chrX:100103802-100103828
Mature seq len: 27
Total raw counts (9 samples): 1242
Average raw counts: 138
Strand: Reverse
Orientation: 3p
Minimum free energy: -33.40

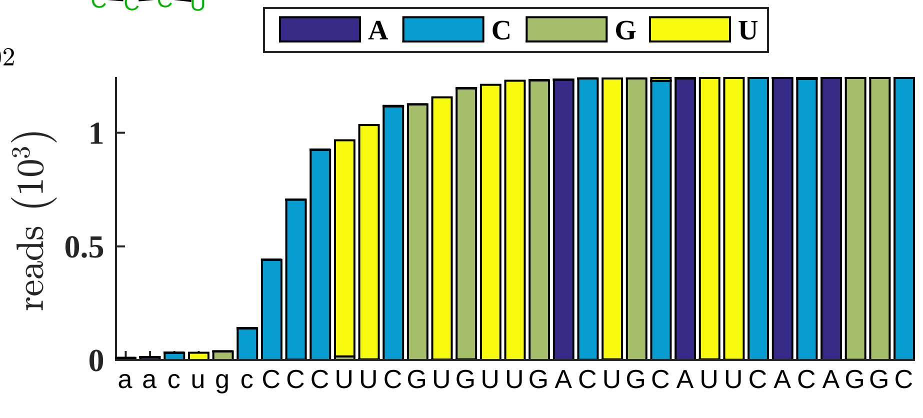

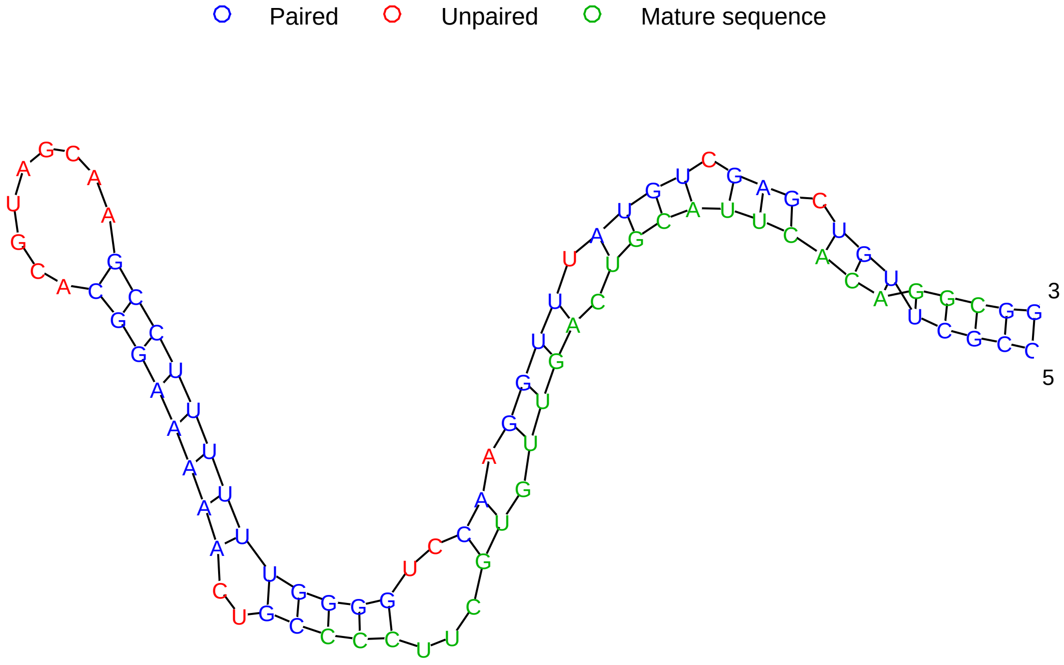

Stem loop (UMD3.1): chrX:100104770-100104859
Mature (UMD3.1): chrX:100104772-100104798
Mature seq len: 27
Total raw counts (9 samples): 1227
Average raw counts: 137
Strand: Reverse
Orientation: 3p
Minimum free energy: -33.20

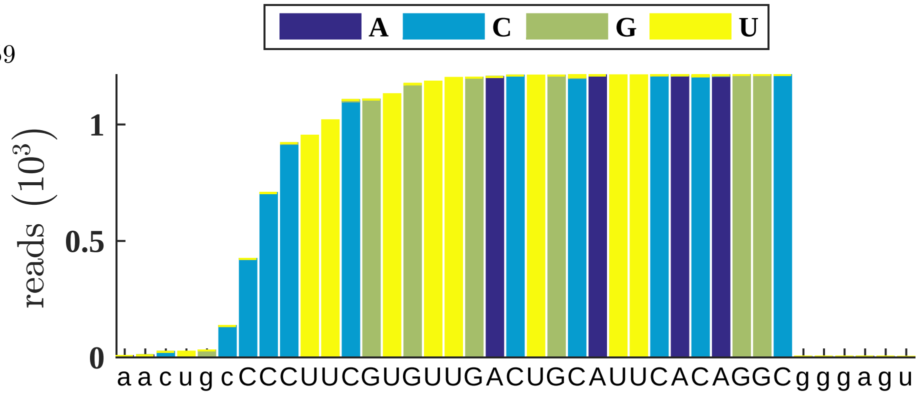

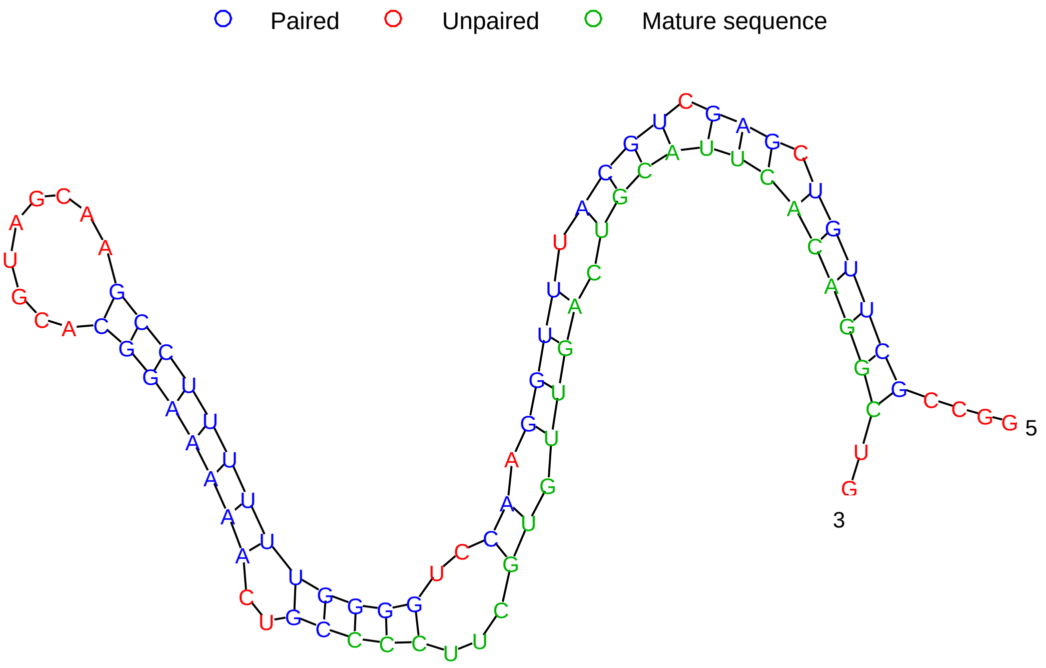

Stem loop (UMD3.1): chrX:123928434-123928525
Mature (UMD3.1): chrX:123928497-123928523
Mature seq len: 27
Total raw counts (9 samples): 1215
Average raw counts: 135
Strand: Forward
Orientation: 3p
Minimum free energy: -30.90

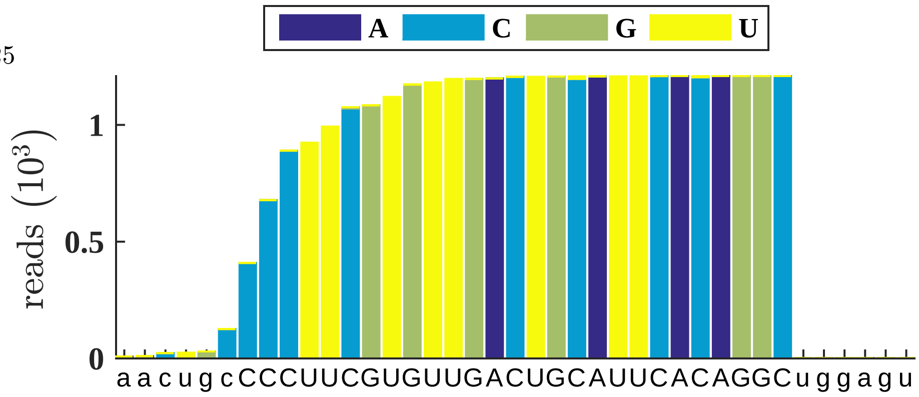

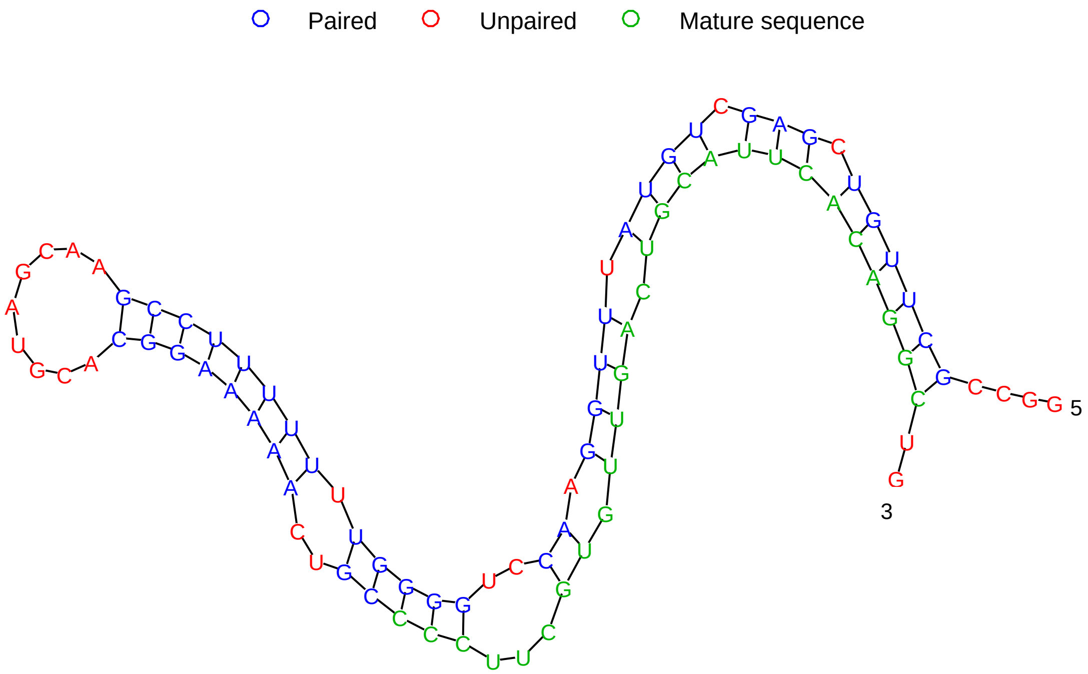

Stem loop (UMD3.1): chrX:134040094-134040186
Mature (UMD3.1): chrX:134040096-134040122
Mature seq len: 27
Total raw counts (9 samples): 1189
Average raw counts: 133
Strand: Reverse
Orientation: 3p
Minimum free energy: -29.70

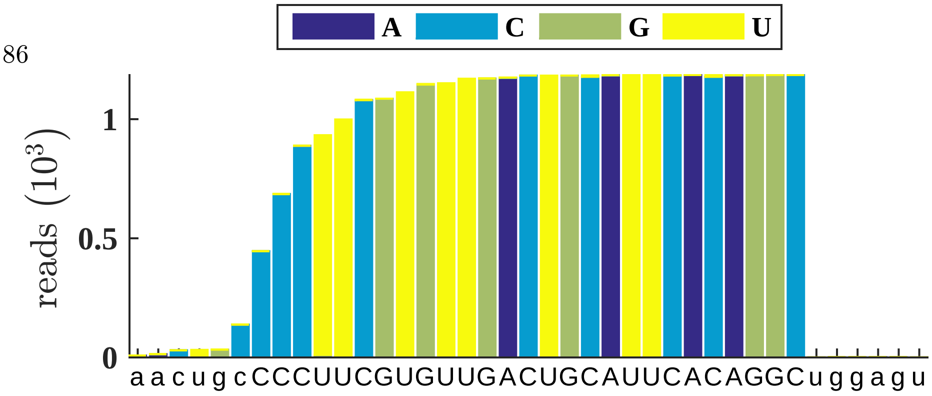

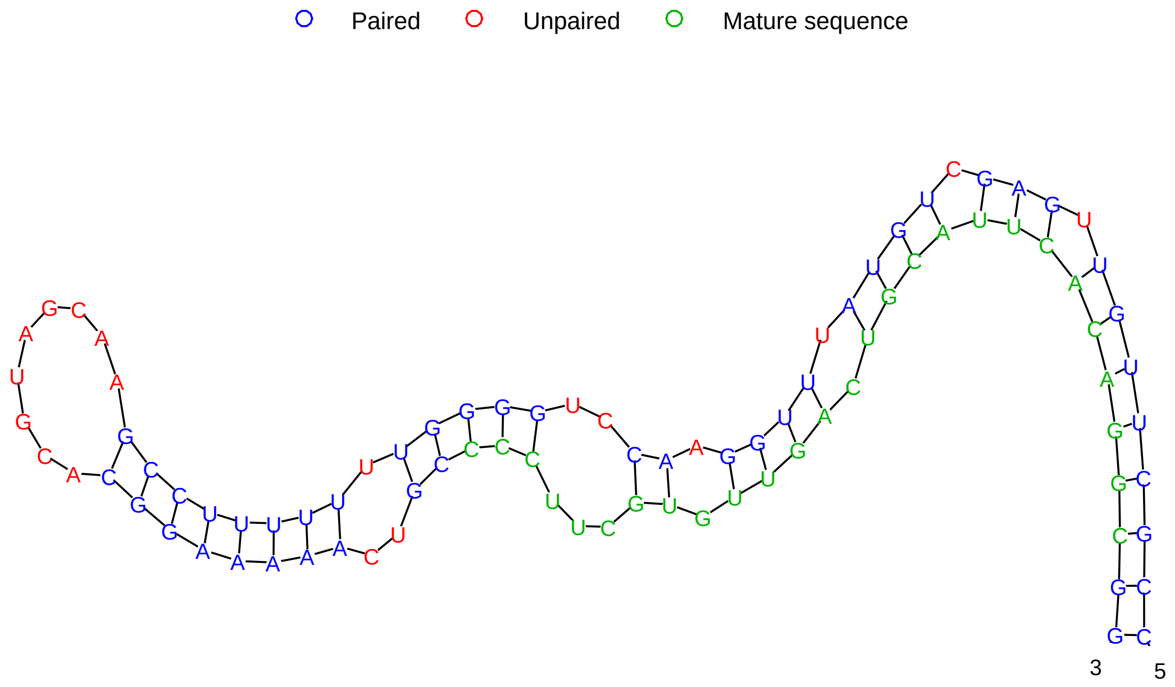

Stem loop (UMD3.1): chrX:134401421-134401511
Mature (UMD3.1): chrX:134401423-134401449
Mature seq len: 27
Total raw counts (9 samples): 1116
Average raw counts: 124
Strand: Reverse
Orientation: 3p
Minimum free energy: -34.00

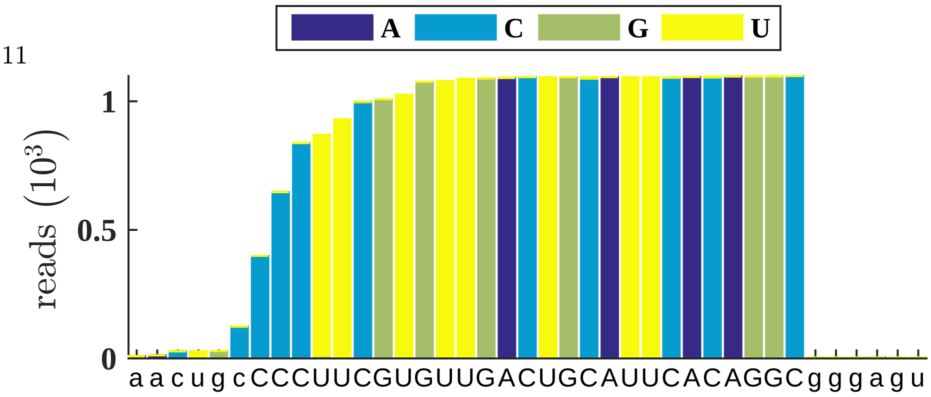

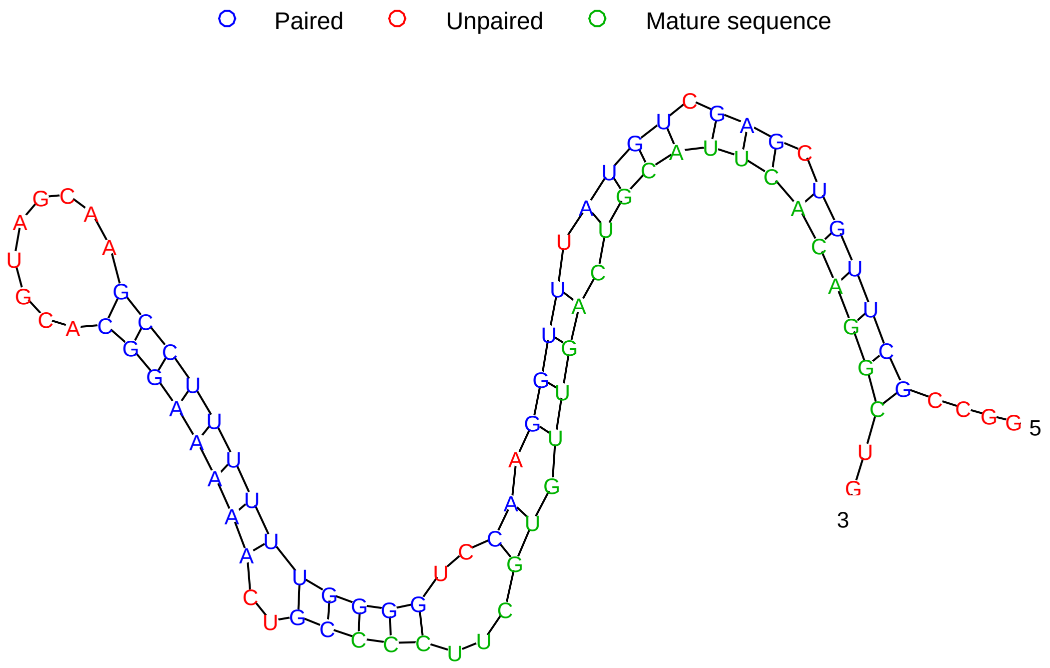

Stem loop (UMD3.1): chrX:134403067-134403158
Mature (UMD3.1): chrX:134403069-134403095
Mature seq len: 27
Total raw counts (9 samples): 1177
Average raw counts: 131
Strand: Reverse
Orientation: 3p
Minimum free energy: -28.90

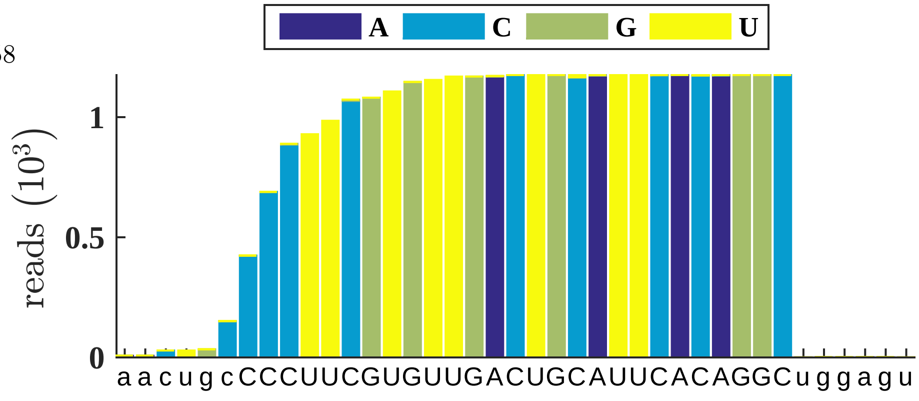

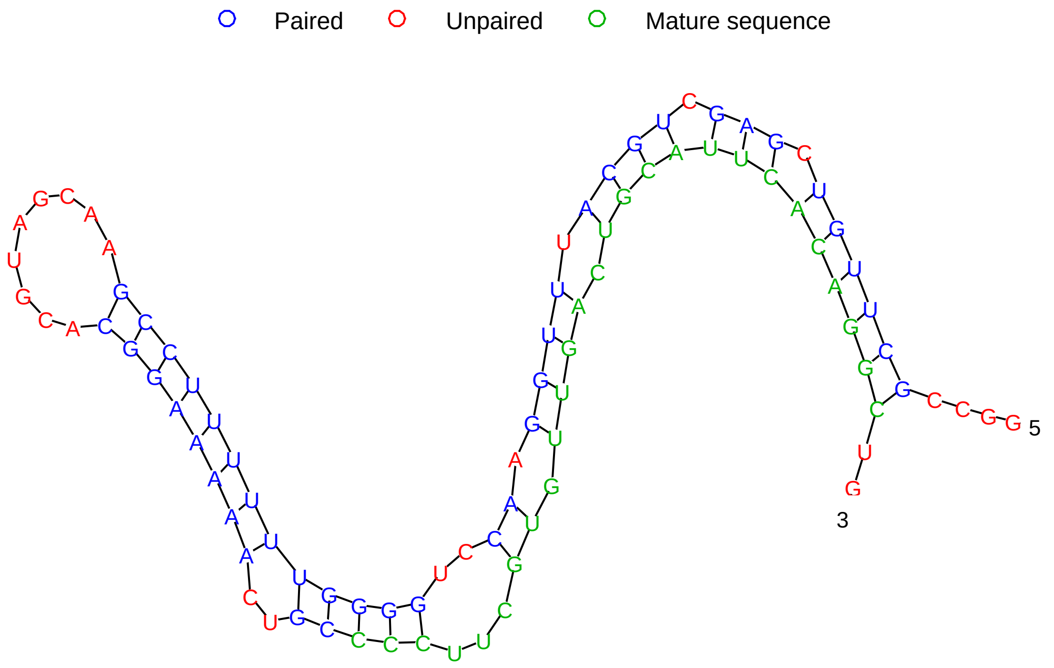

Stem loop (UMD3.1): chrX:134404457-134404548
Mature (UMD3.1): chrX:134404459-134404485
Mature seq len: 27
Total raw counts (9 samples): 1208
Average raw counts: 135
Strand: Reverse
Orientation: 3p
Minimum free energy: -30.90

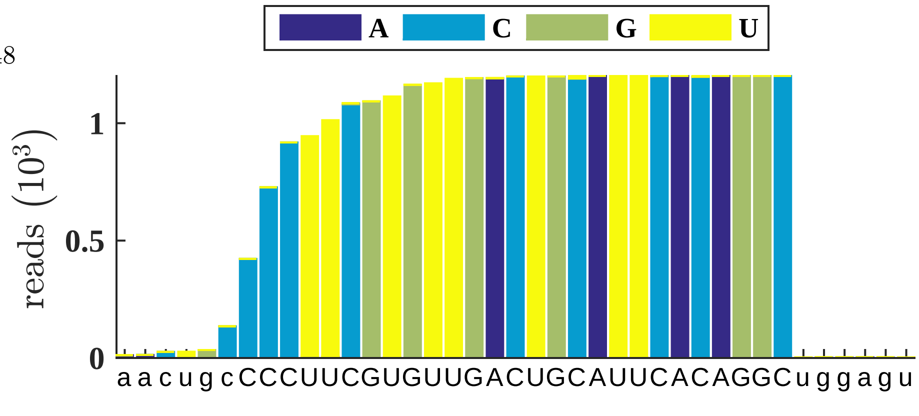

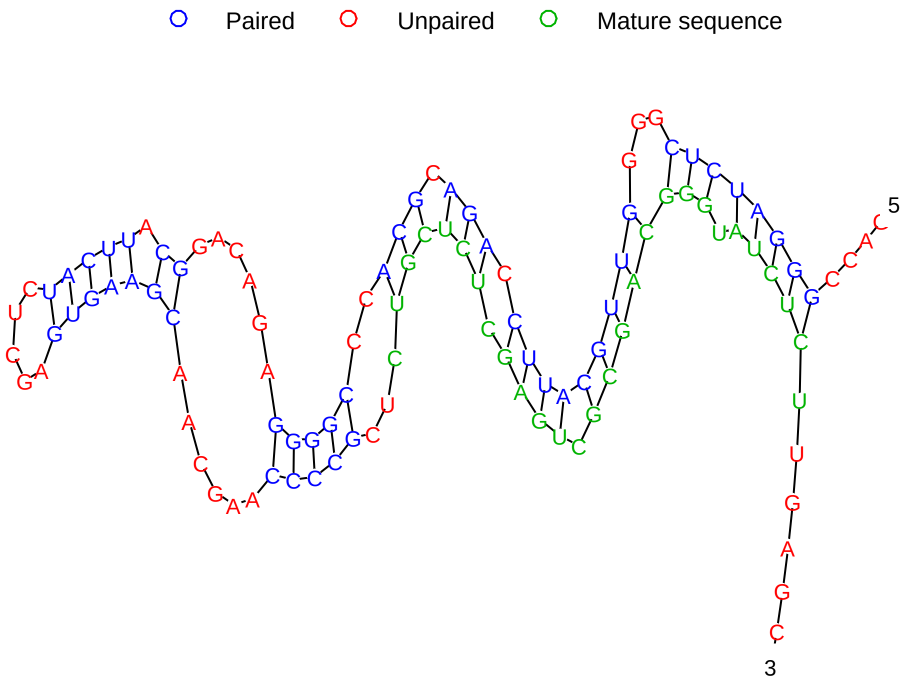

Stem loop (UMD3.1): chrX:14083281-14083391
Mature (UMD3.1): chrX:14083359-14083386
Mature seq len: 28
Total raw counts (9 samples): 12004
Average raw counts: 1334
Strand: Forward
Orientation: 3p
Minimum free energy: -35.70

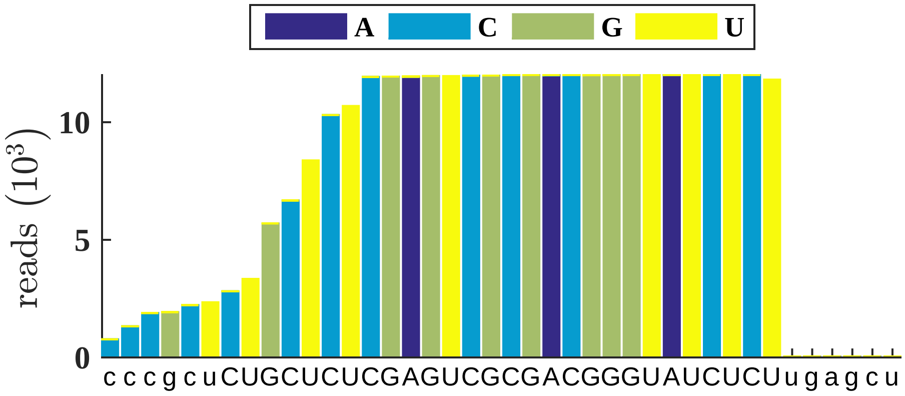

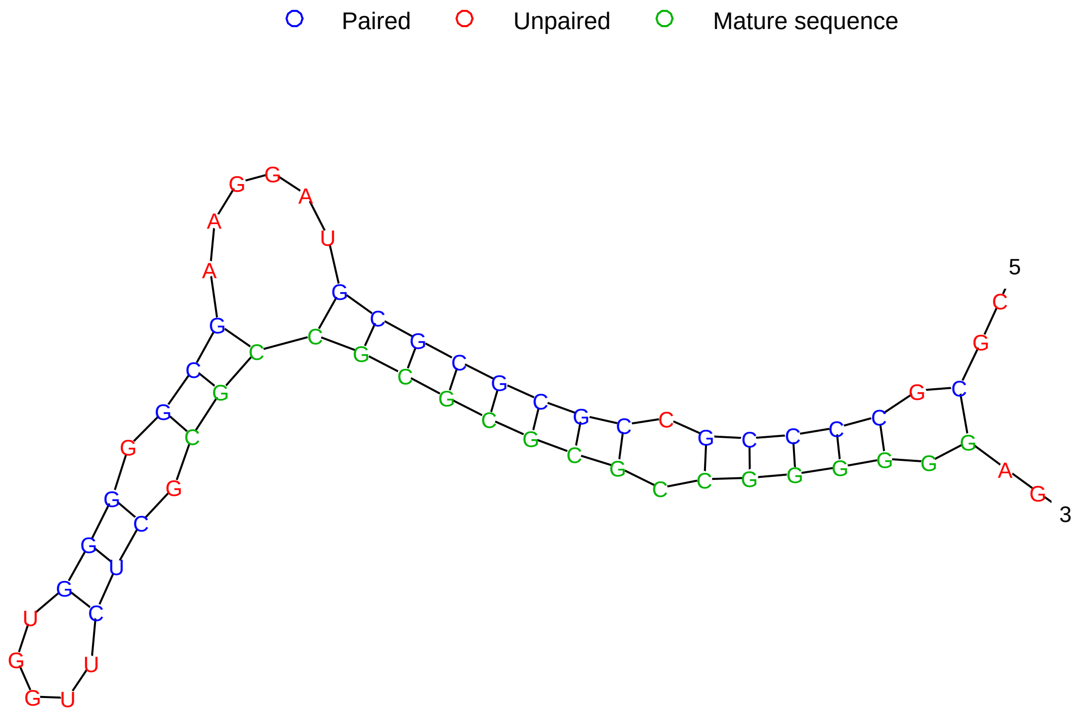

Stem loop (UMD3.1): chrX:144087397-144087457
Mature (UMD3.1): chrX:144087437-144087455
Mature seq len: 19
Total raw counts (9 samples): 875
Average raw counts: 98
Strand: Forward
Orientation: 3p
Minimum free energy: -38.20

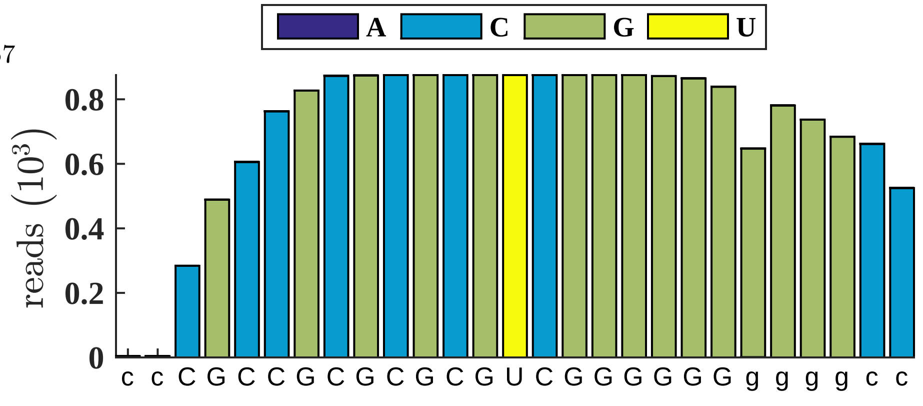

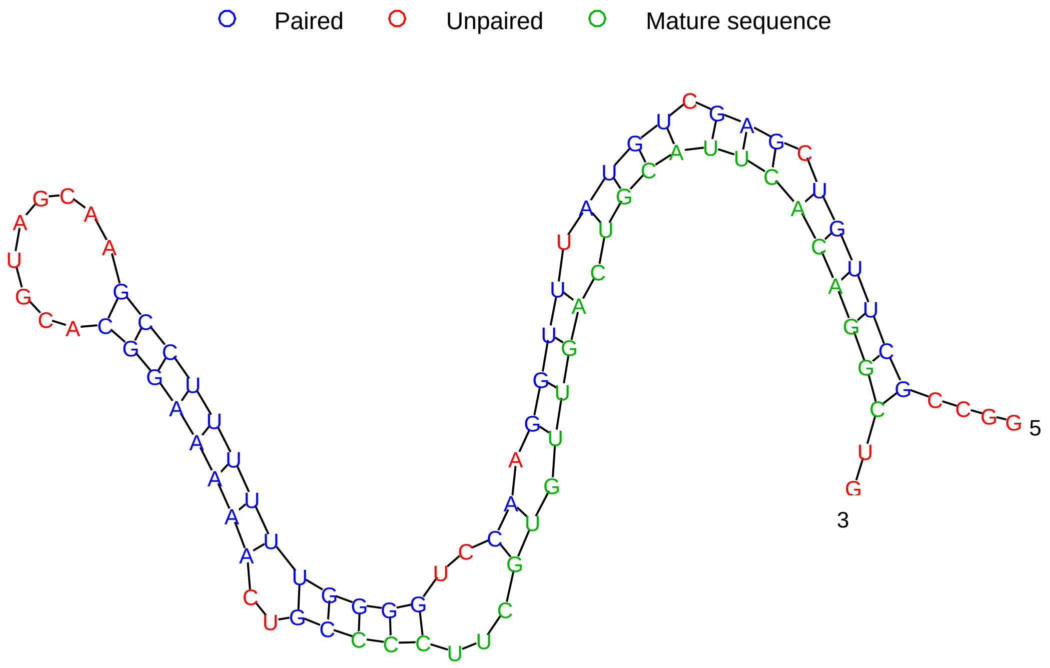

Stem loop (UMD3.1): chrX:18976208-18976299
Mature (UMD3.1): chrX:18976271-18976297
Mature seq len: 27
Total raw counts (9 samples): 1243
Average raw counts: 139
Strand: Forward
Orientation: 3p
Minimum free energy: -28.90

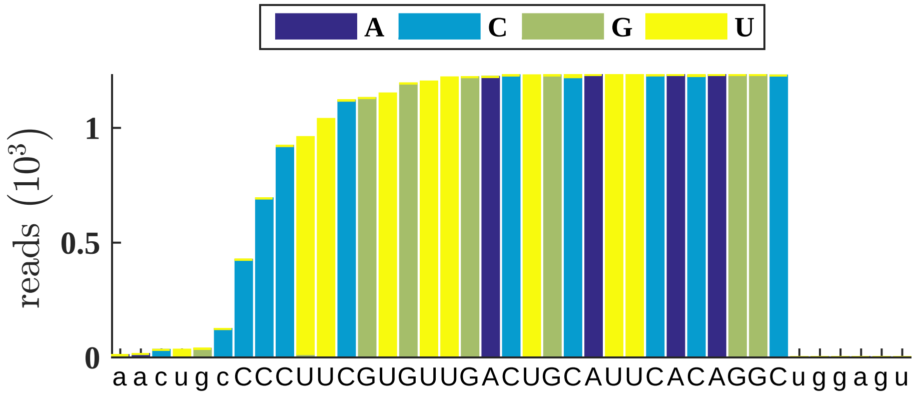

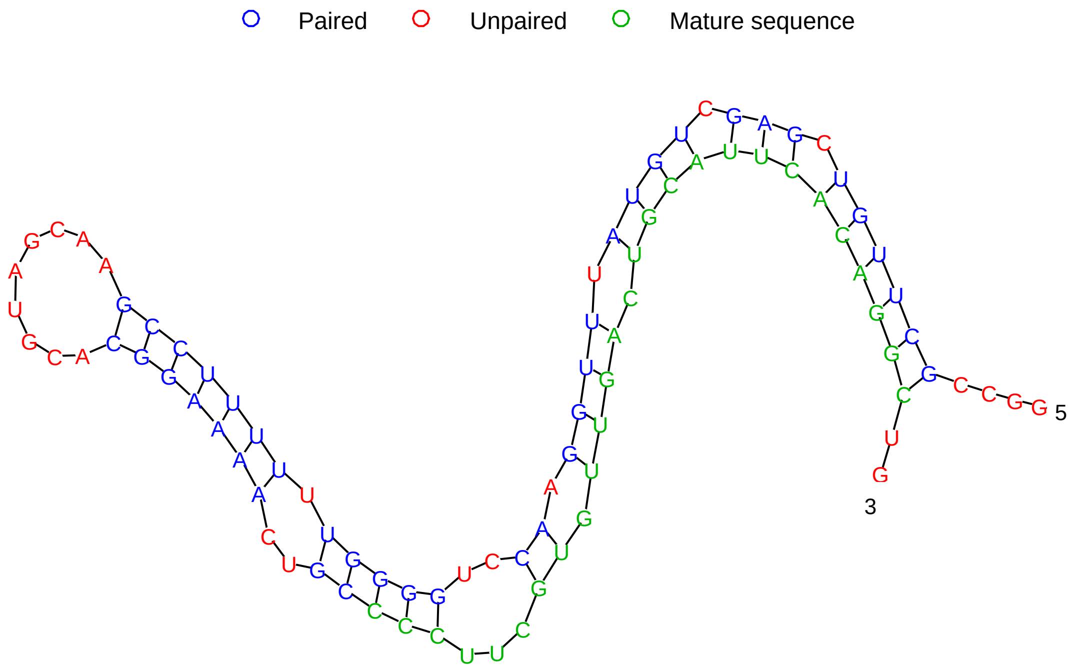

Stem loop (UMD3.1): chrX:18980325-18980415
Mature (UMD3.1): chrX:18980387-18980413
Mature seq len: 27
Total raw counts (9 samples): 1186
Average raw counts: 132
Strand: Forward
Orientation: 3p
Minimum free energy: -28.80

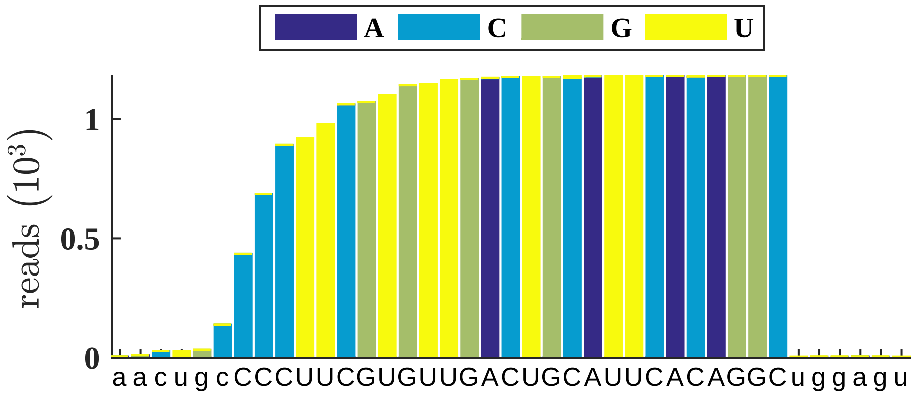

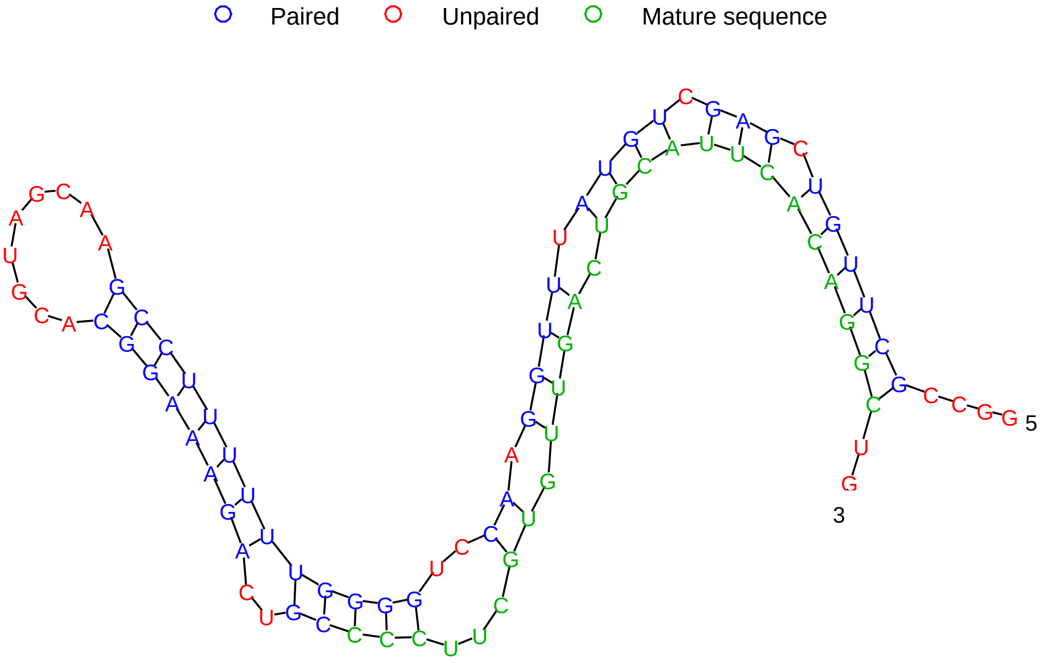

Stem loop (UMD3.1): chrX:18982521-18982612
Mature (UMD3.1): chrX:18982584-18982610
Mature seq len: 27
Total raw counts (9 samples): 1197
Average raw counts: 133
Strand: Forward
Orientation: 3p
Minimum free energy: -29.00

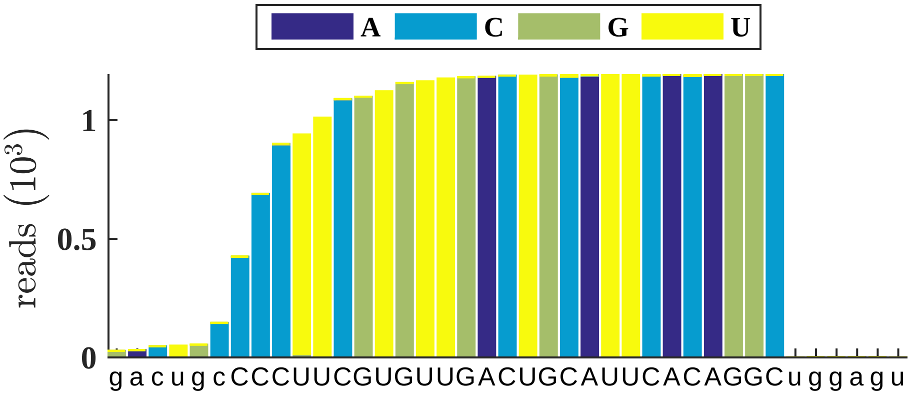

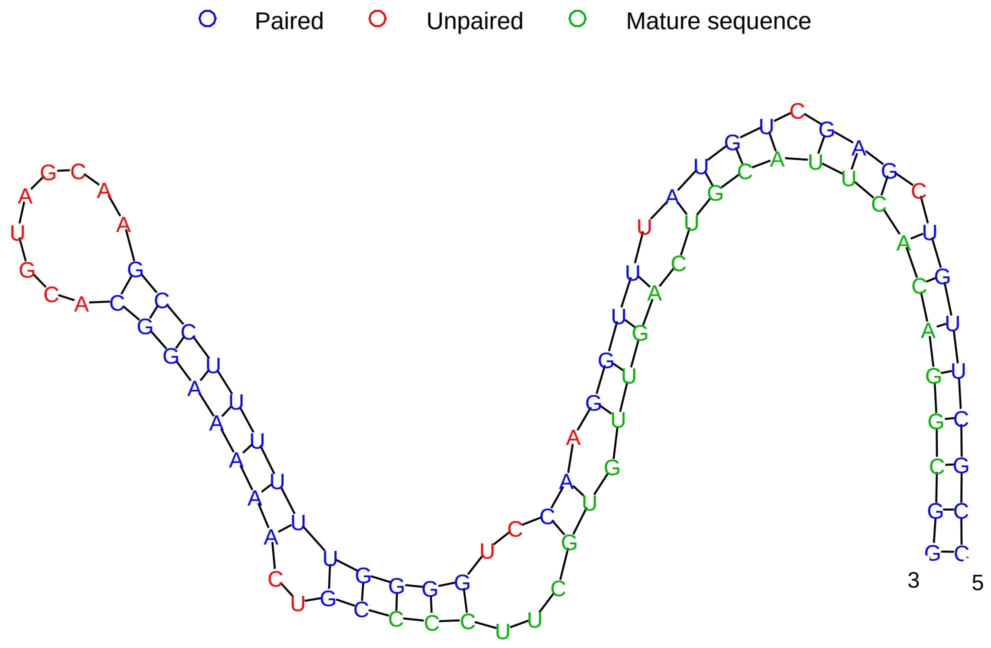

Stem loop (UMD3.1): chrX:18984352-18984441
Mature (UMD3.1): chrX:18984413-18984439
Mature seq len: 27
Total raw counts (9 samples): 1165
Average raw counts: 130
Strand: Forward
Orientation: 3p
Minimum free energy: -33.20

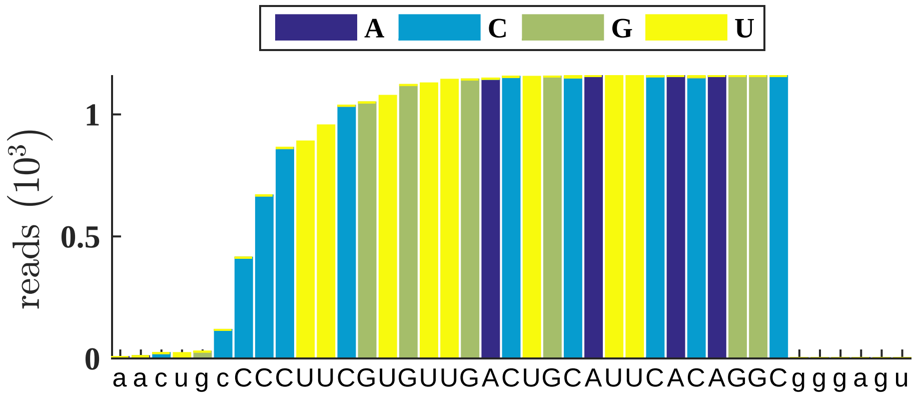

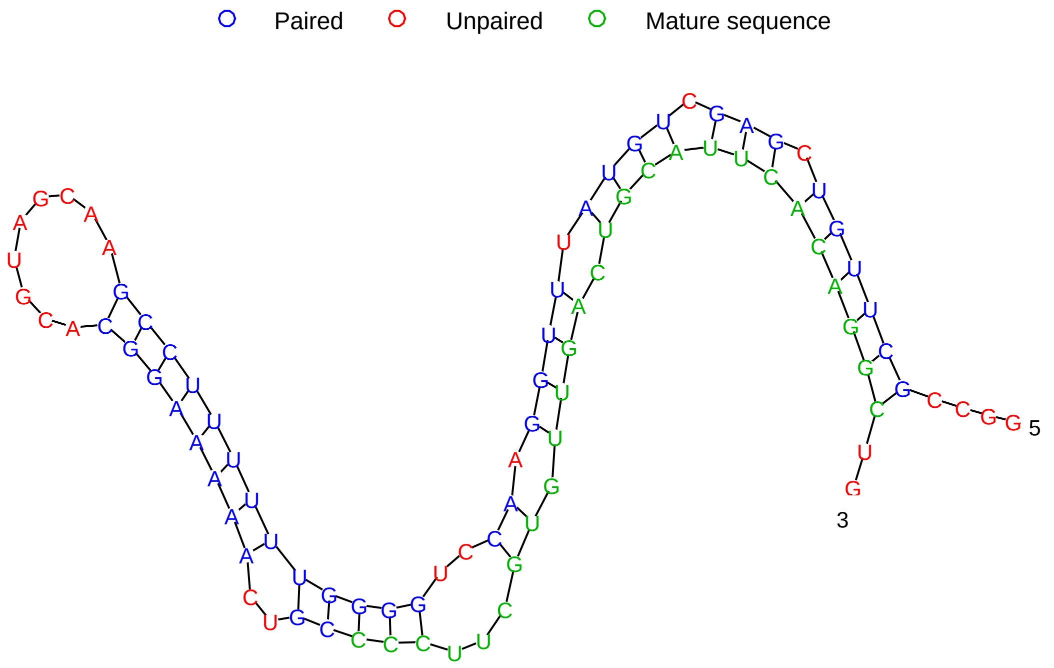

Stem loop (UMD3.1): chrX:19145283-19145374
Mature (UMD3.1): chrX:19145346-19145372
Mature seq len: 27
Total raw counts (9 samples): 1195
Average raw counts: 133
Strand: Forward
Orientation: 3p
Minimum free energy: -28.90

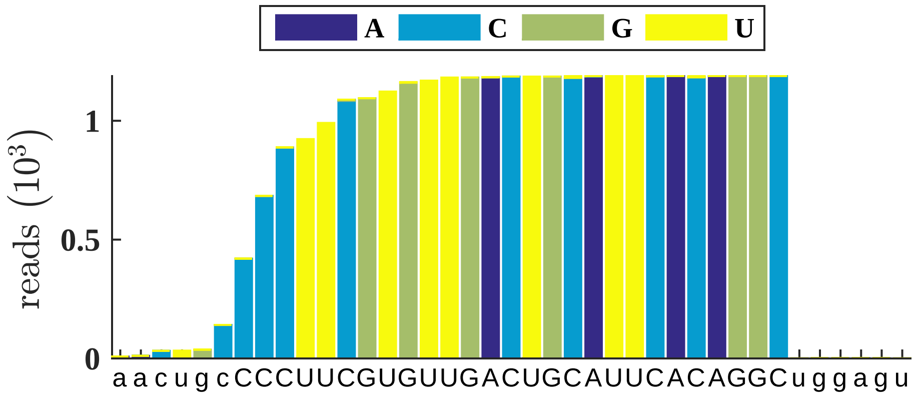

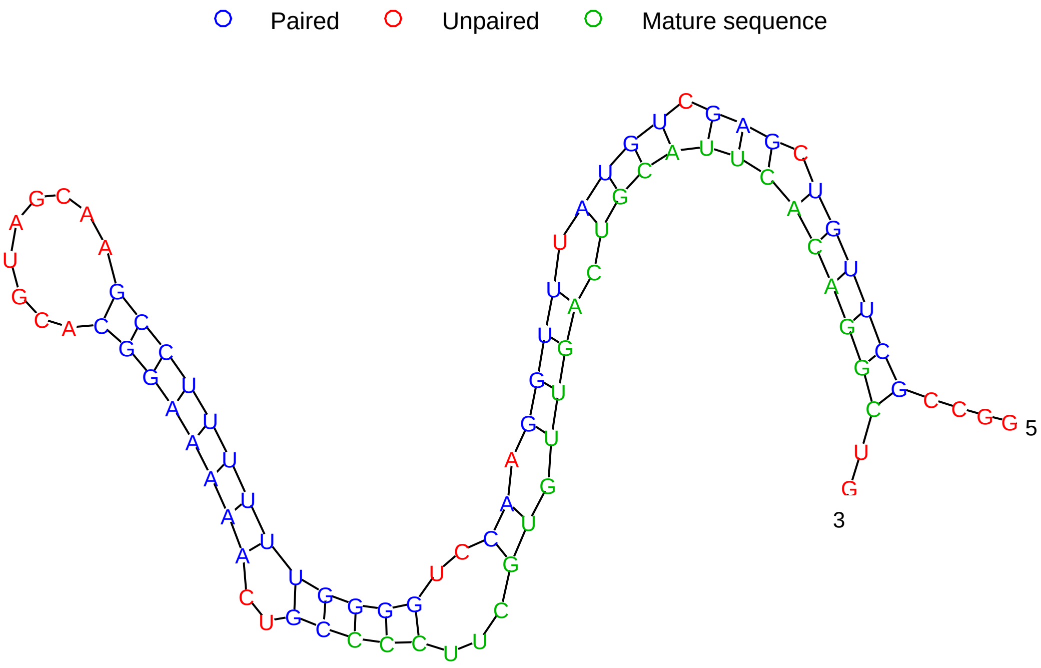

Stem loop (UMD3.1): chrX:19146668-19146759
Mature (UMD3.1): chrX:19146731-19146757
Mature seq len: 27
Total raw counts (9 samples): 1119
Average raw counts: 125
Strand: Forward
Orientation: 3p
Minimum free energy: -28.90

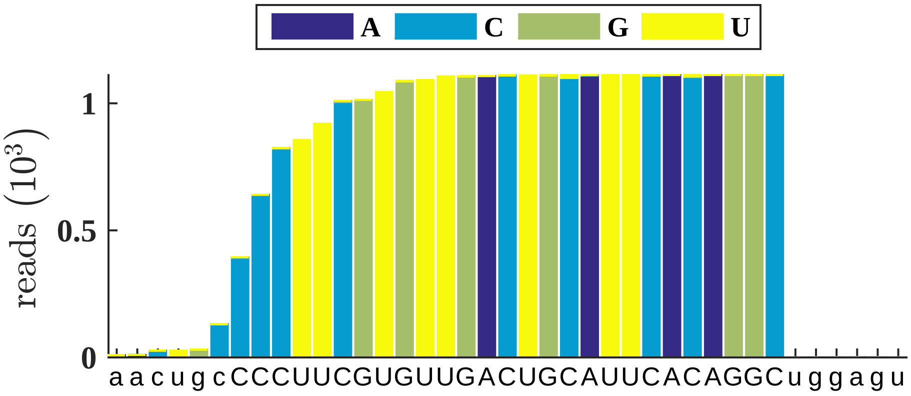

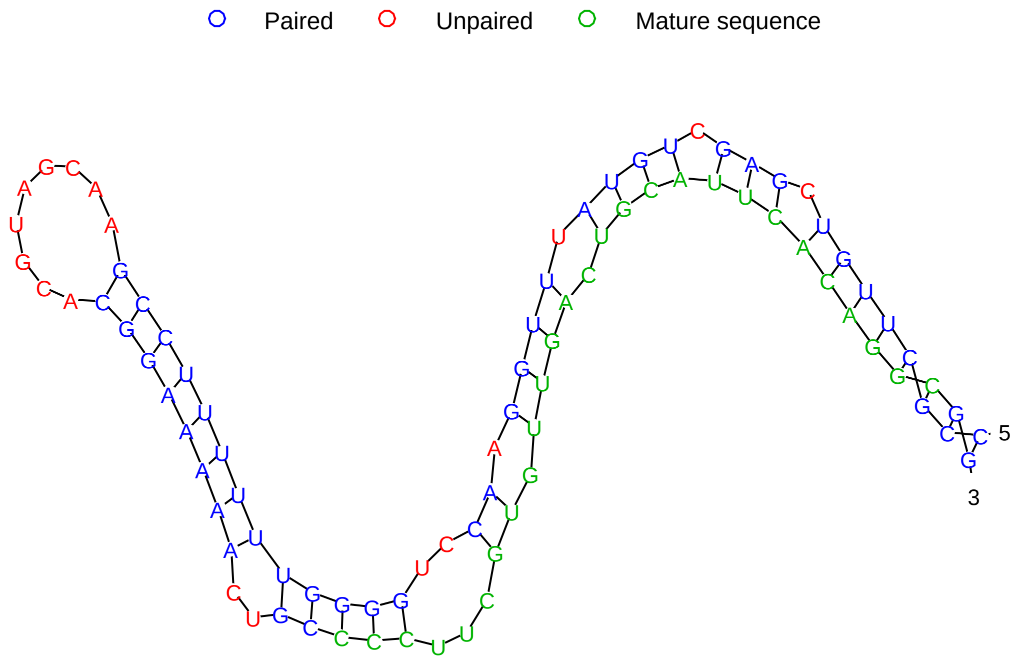

Stem loop (UMD3.1): chrX:19165397-19165486
Mature (UMD3.1): chrX:19165399-19165425
Mature seq len: 27
Total raw counts (9 samples): 1243
Average raw counts: 139
Strand: Reverse
Orientation: 3p
Minimum free energy: -33.20

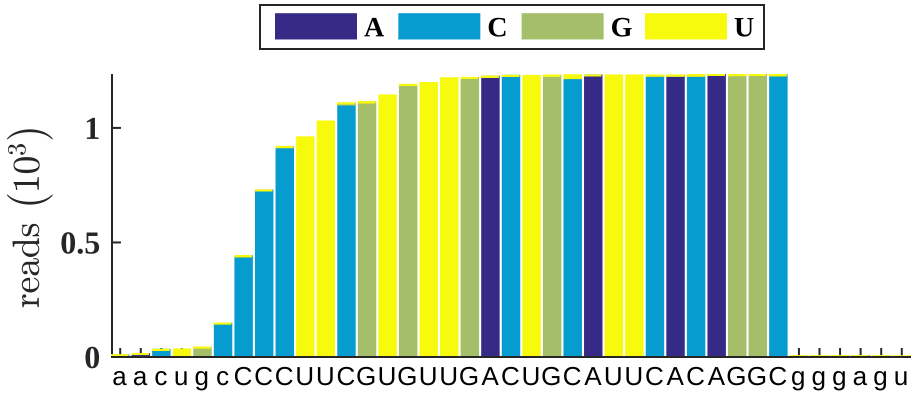

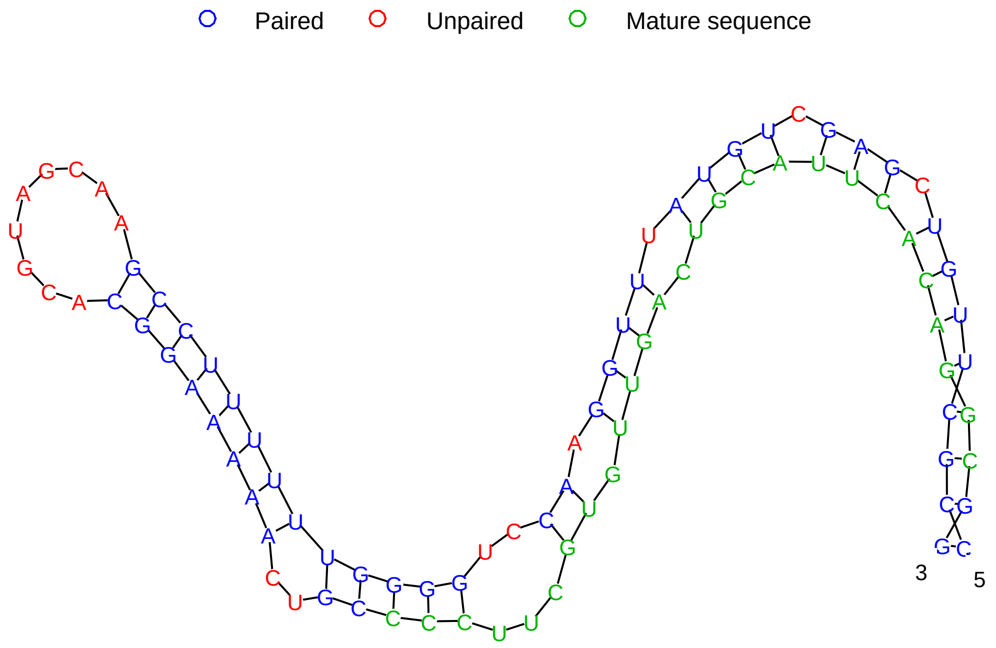

Stem loop (UMD3.1): chrX:19167094-19167183
Mature (UMD3.1): chrX:19167096-19167122
Mature seq len: 27
Total raw counts (9 samples): 1171
Average raw counts: 131
Strand: Reverse
Orientation: 3p
Minimum free energy: -33.20

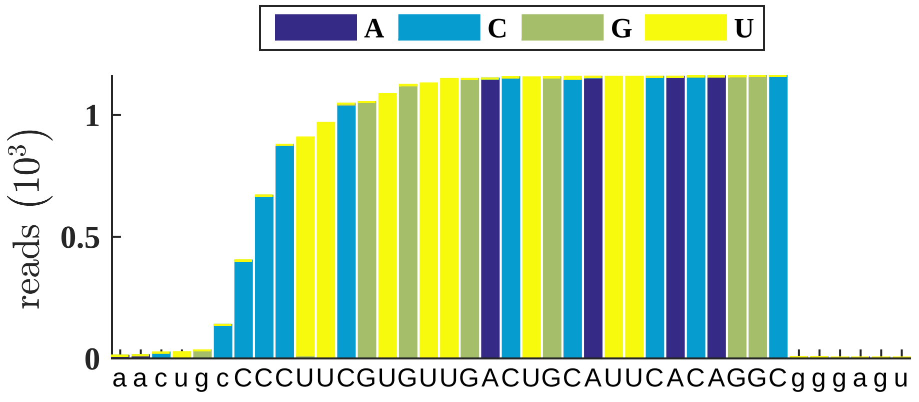

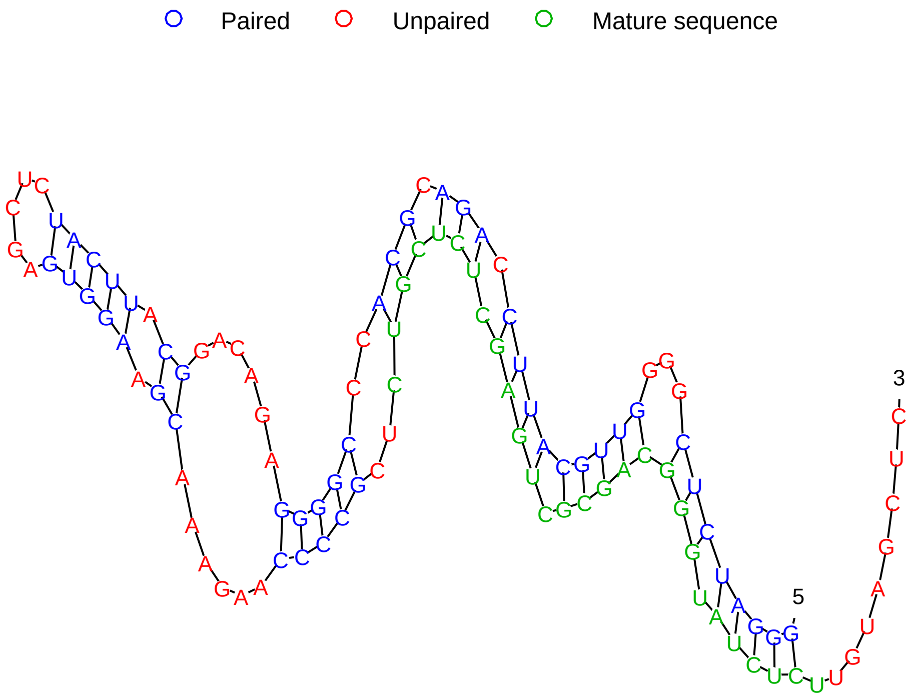

Stem loop (UMD3.1): chrX:19171683-19171793
Mature (UMD3.1): chrX:19171691-19171718
Mature seq len: 28
Total raw counts (9 samples): 12856
Average raw counts: 1429
Strand: Reverse
Orientation: 3p
Minimum free energy: -35.50

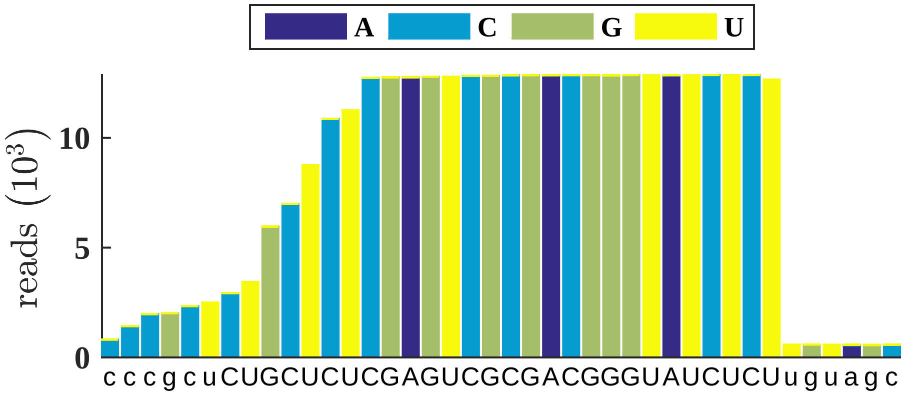

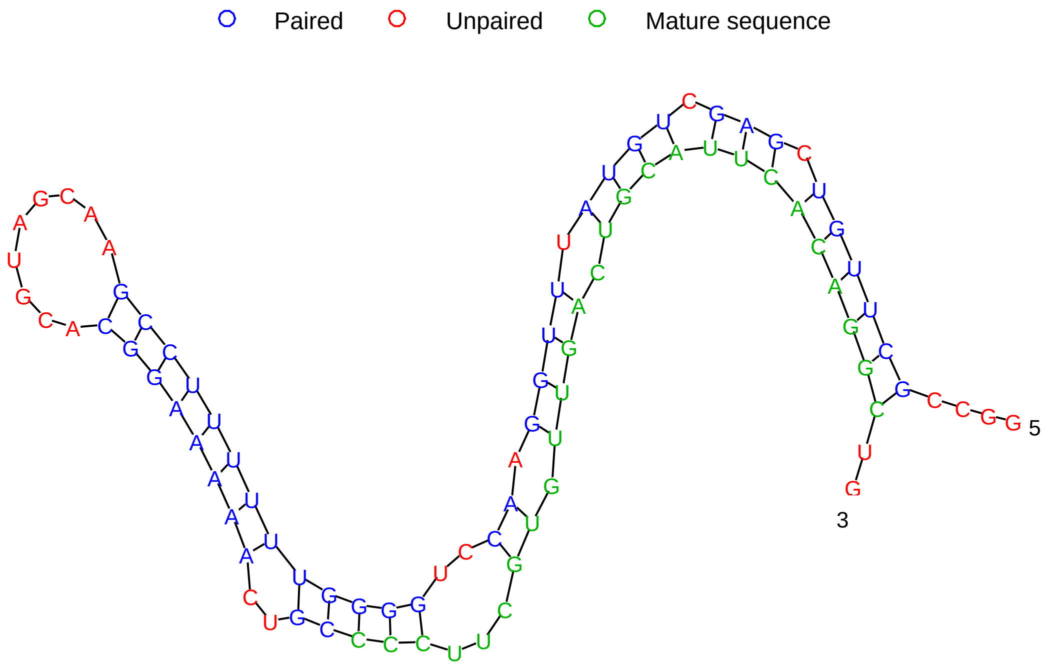

Stem loop (UMD3.1): chrX:19173617-19173708
Mature (UMD3.1): chrX:19173619-19173645
Mature seq len: 27
Total raw counts (9 samples): 1224
Average raw counts: 136
Strand: Reverse
Orientation: 3p
Minimum free energy: -28.90

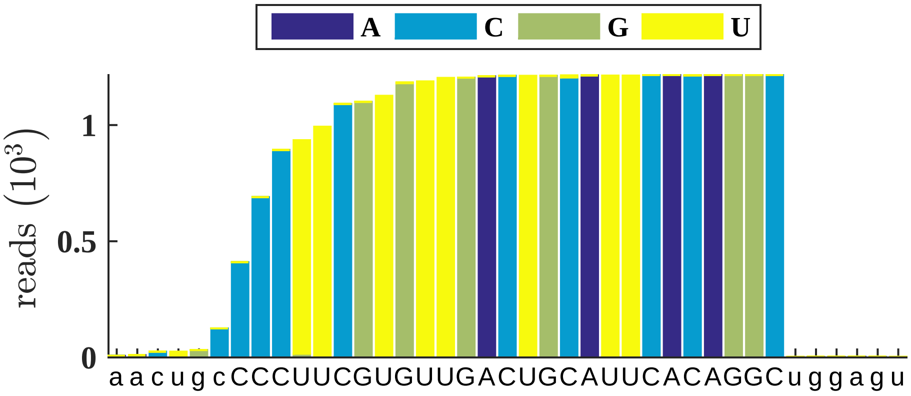

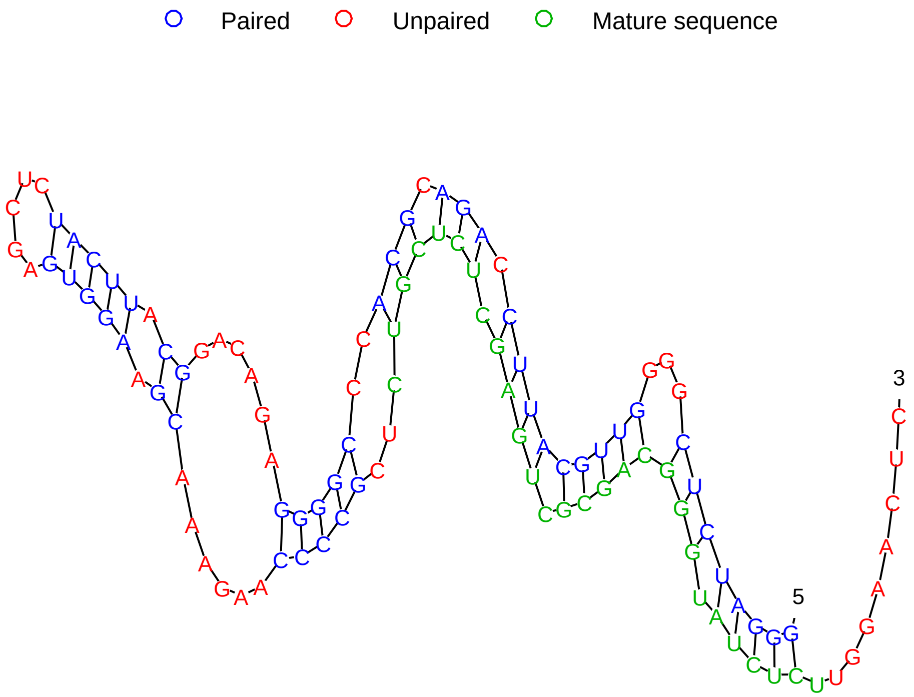

Stem loop (UMD3.1): chrX:19180532-19180642
Mature (UMD3.1): chrX:19180540-19180567
Mature seq len: 28
Total raw counts (9 samples): 14510
Average raw counts: 1613
Strand: Reverse
Orientation: 3p
Minimum free energy: -35.50

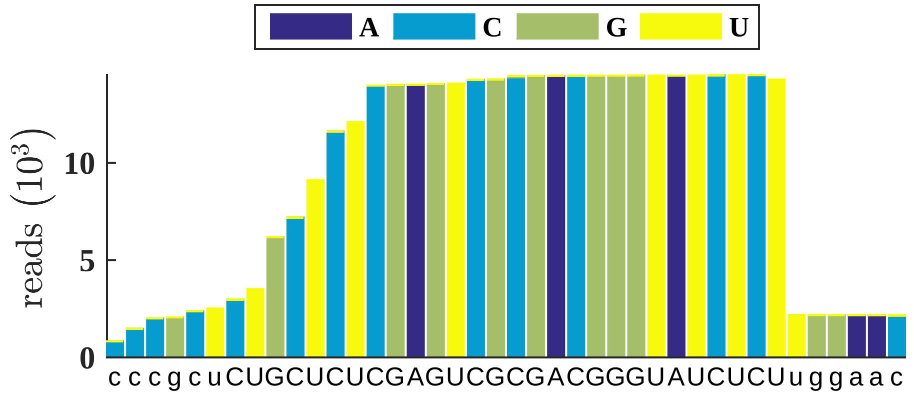

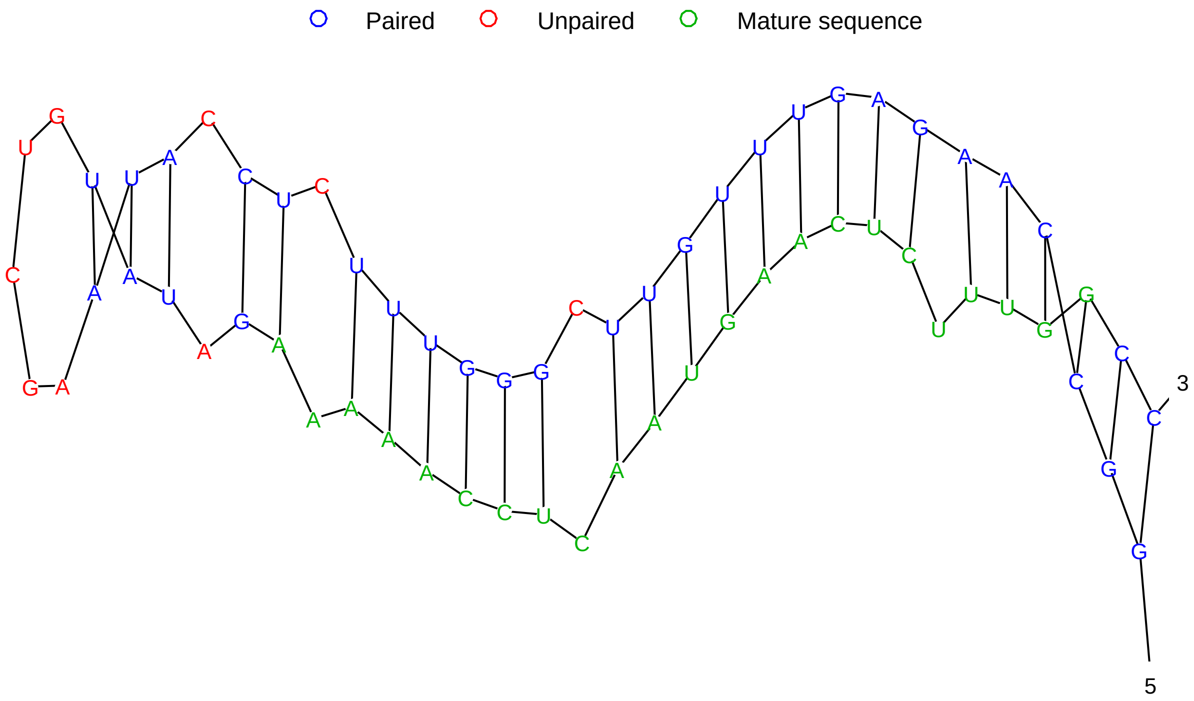

Stem loop (UMD3.1): chrX:2676189-2676252
Mature (UMD3.1): chrX:2676228-2676250
Mature seq len: 23
Total raw counts (9 samples): 997
Average raw counts: 111
Strand: Forward
Orientation: 3p
Minimum free energy: -26.00

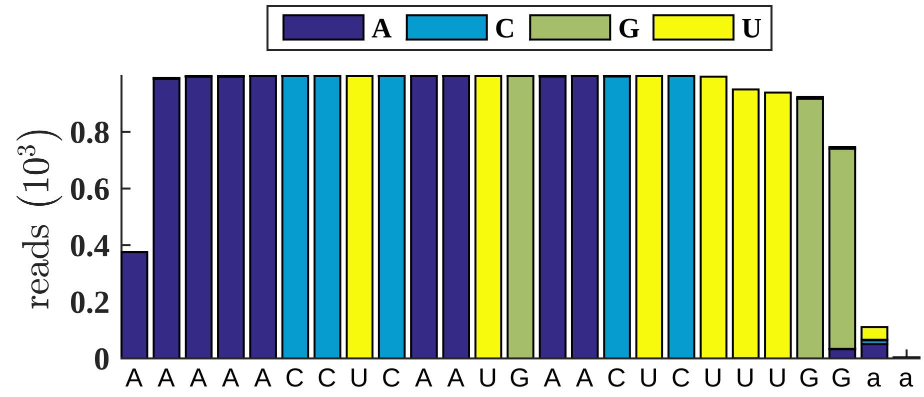

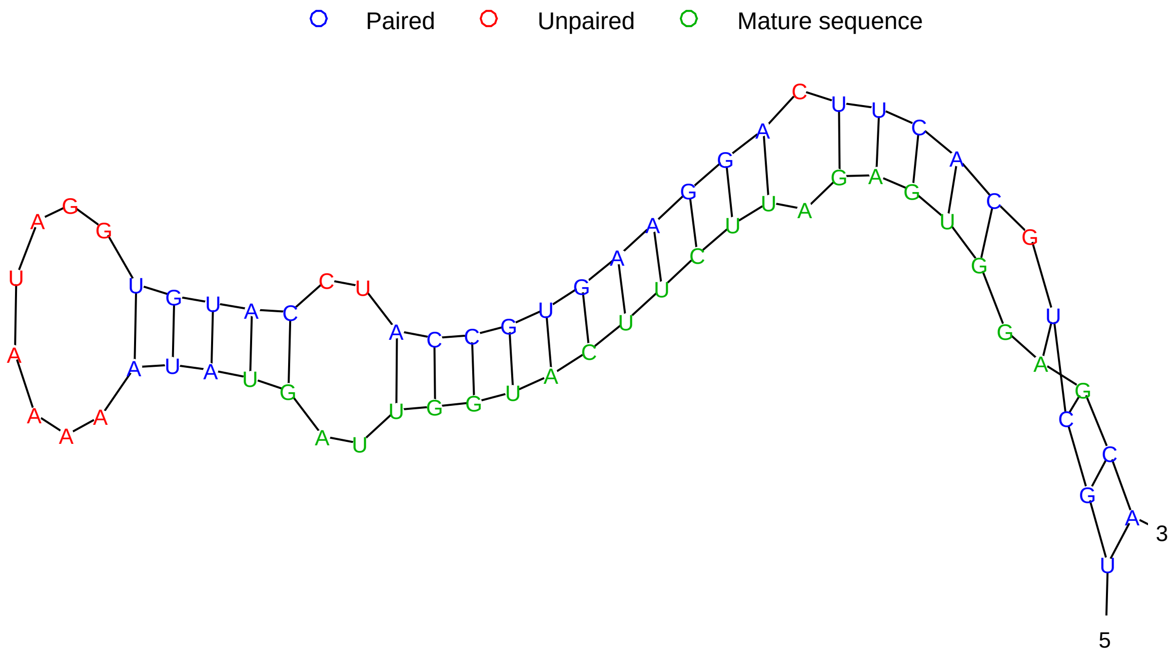

Stem loop (UMD3.1): chrX:29130206-29130271
Mature (UMD3.1): chrX:29130246-29130269
Mature seq len: 24
Total raw counts (9 samples): 409
Average raw counts: 46
Strand: Forward
Orientation: 3p
Minimum free energy: -34.40

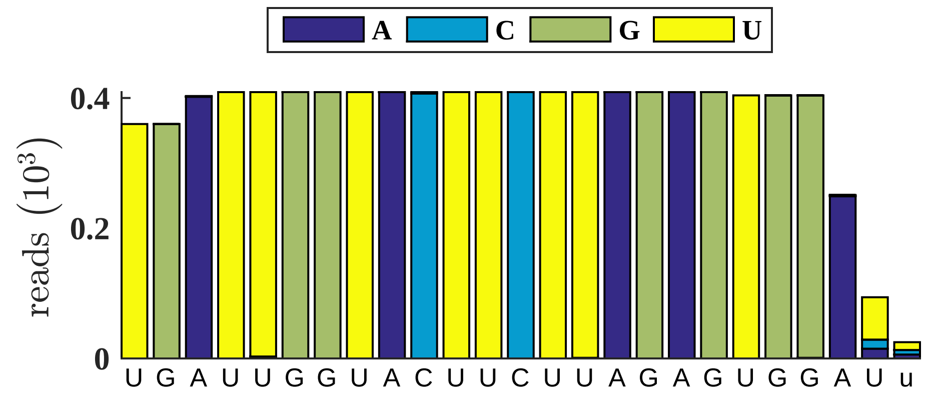

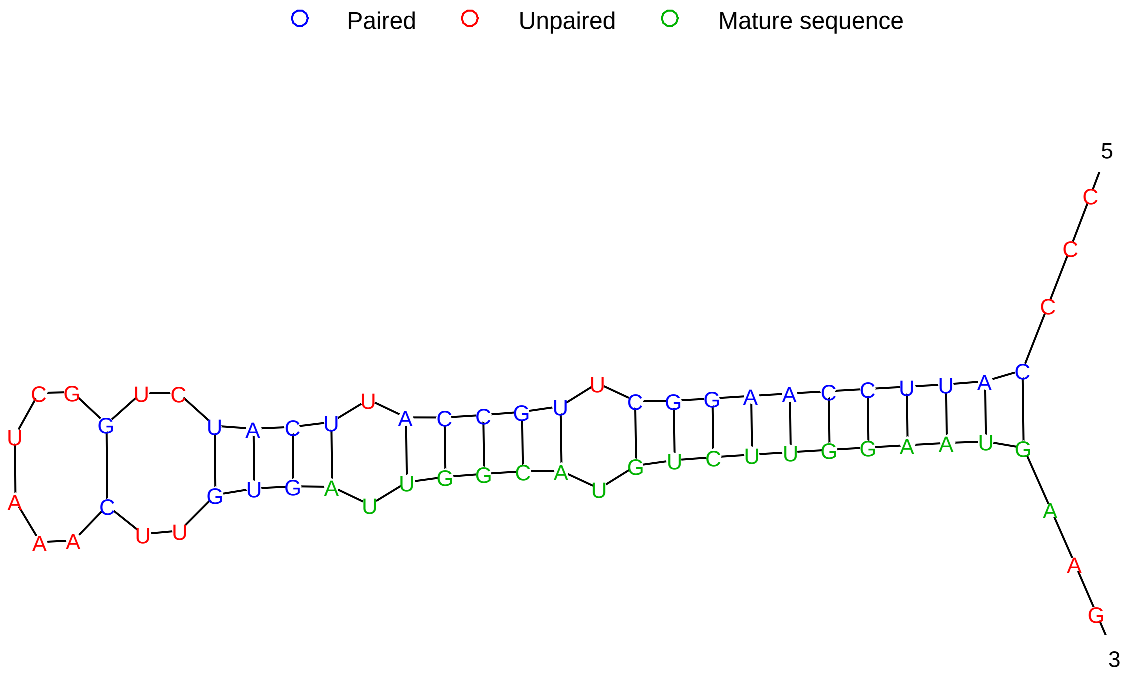

Stem loop (UMD3.1): chrX:30338369-30338430
Mature (UMD3.1): chrX:30338371-30338390
Mature seq len: 20
Total raw counts (9 samples): 4189
Average raw counts: 466
Strand: Reverse
Orientation: 3p
Minimum free energy: -31.10

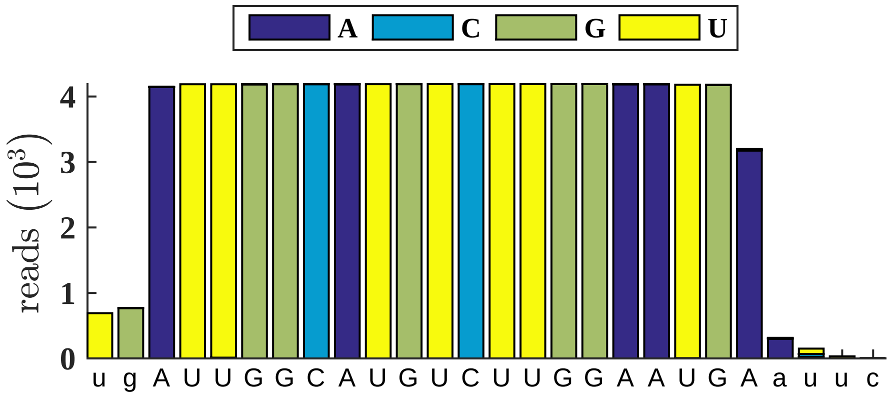

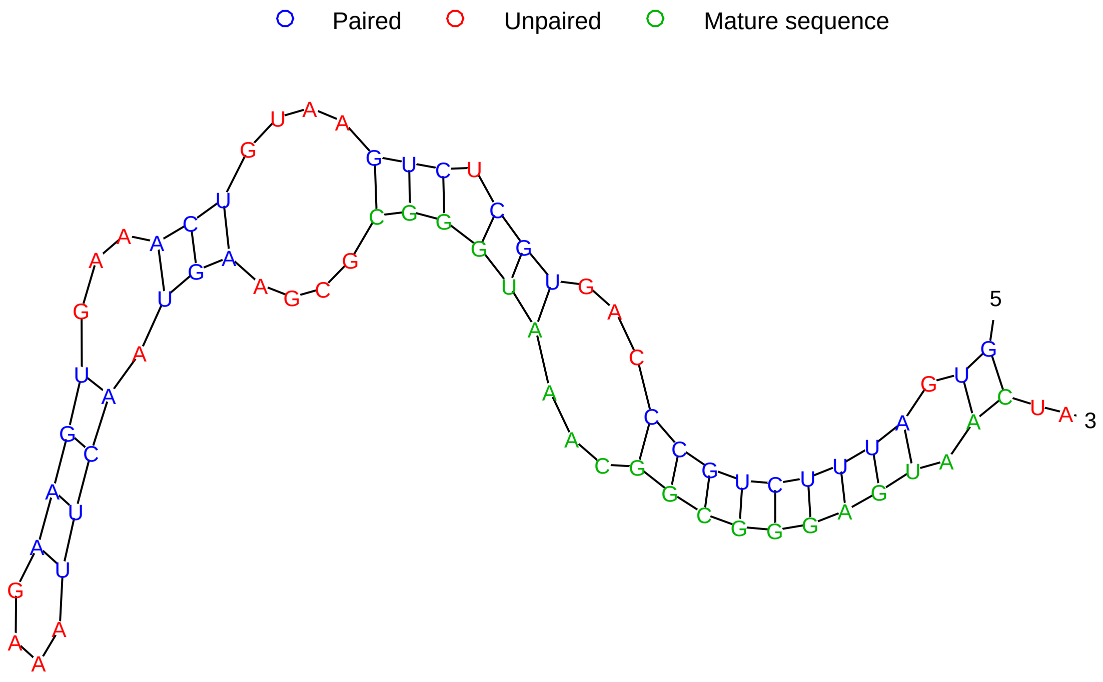

Stem loop (UMD3.1): chrX:62078659-62078733
Mature (UMD3.1): chrX:62078661-62078681
Mature seq len: 21
Total raw counts (9 samples): 2143
Average raw counts: 239
Strand: Reverse
Orientation: 3p
Minimum free energy: -21.50

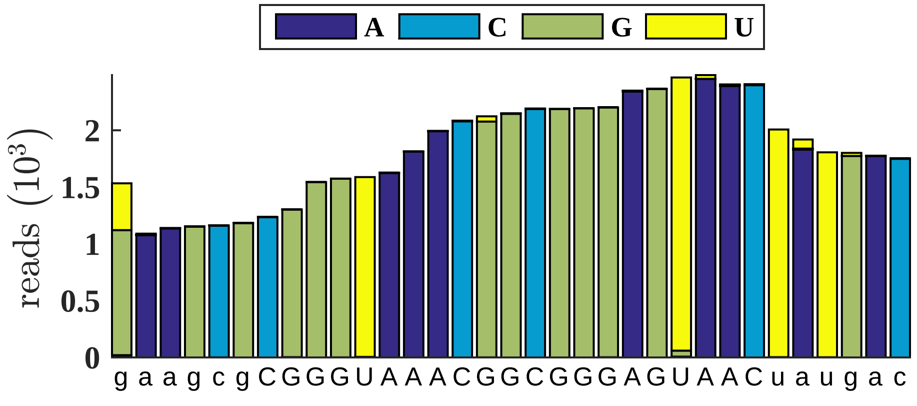

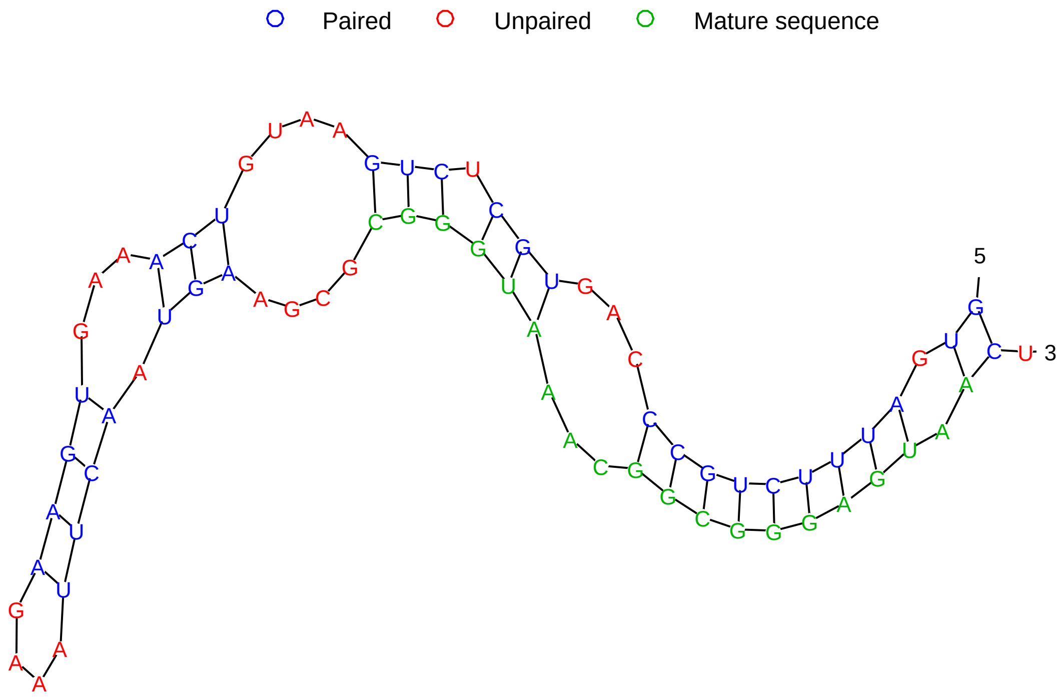

Stem loop (UMD3.1): chrX:62079639-62079712
Mature (UMD3.1): chrX:62079641-62079660
Mature seq len: 20
Total raw counts (9 samples): 2083
Average raw counts: 232
Strand: Reverse
Orientation: 3p
Minimum free energy: -21.50

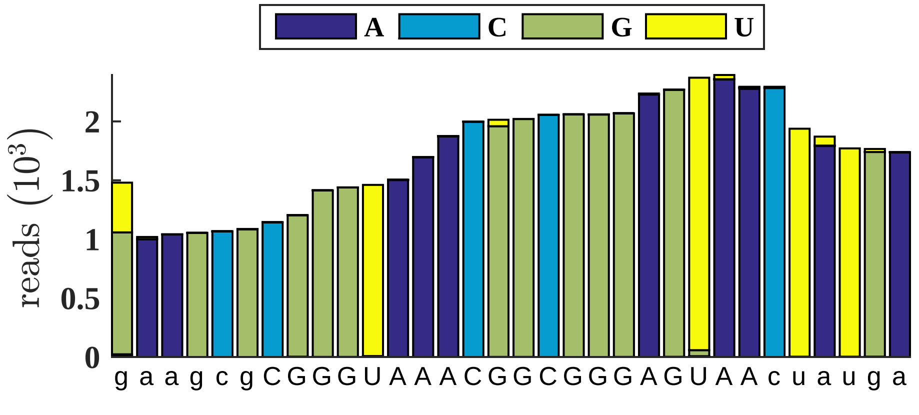

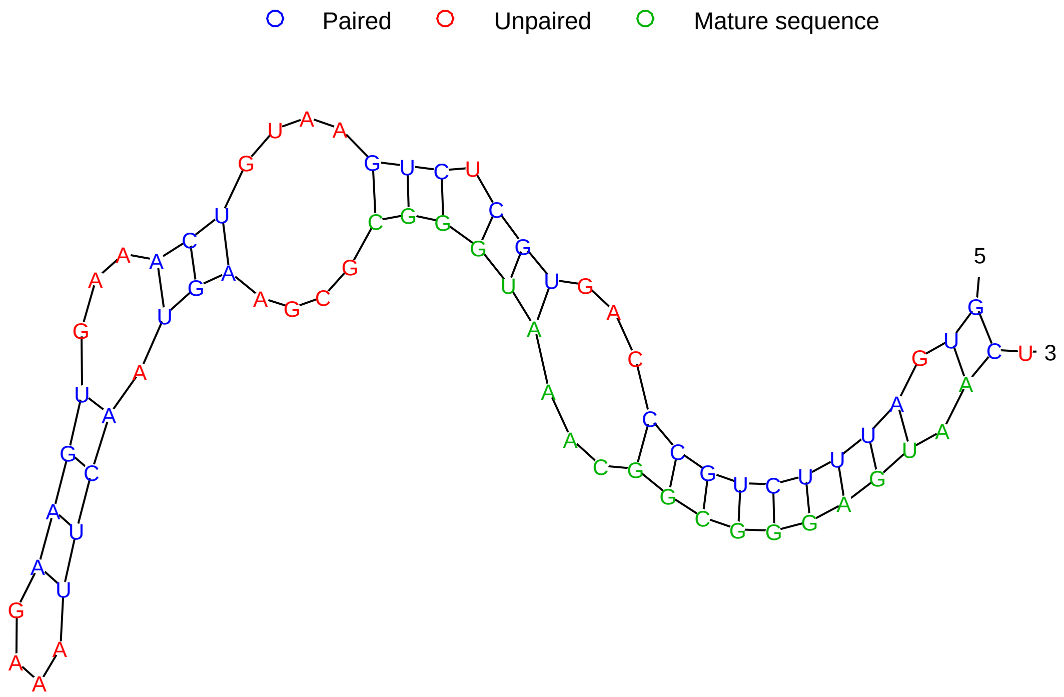

Stem loop (UMD3.1): chrX:62080683-62080756
Mature (UMD3.1): chrX:62080685-62080704
Mature seq len: 20
Total raw counts (9 samples): 1186
Average raw counts: 132
Strand: Reverse
Orientation: 3p
Minimum free energy: -21.50

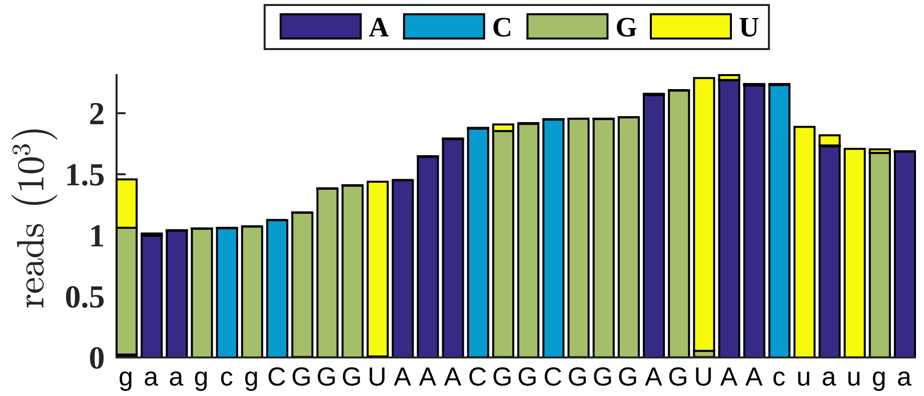

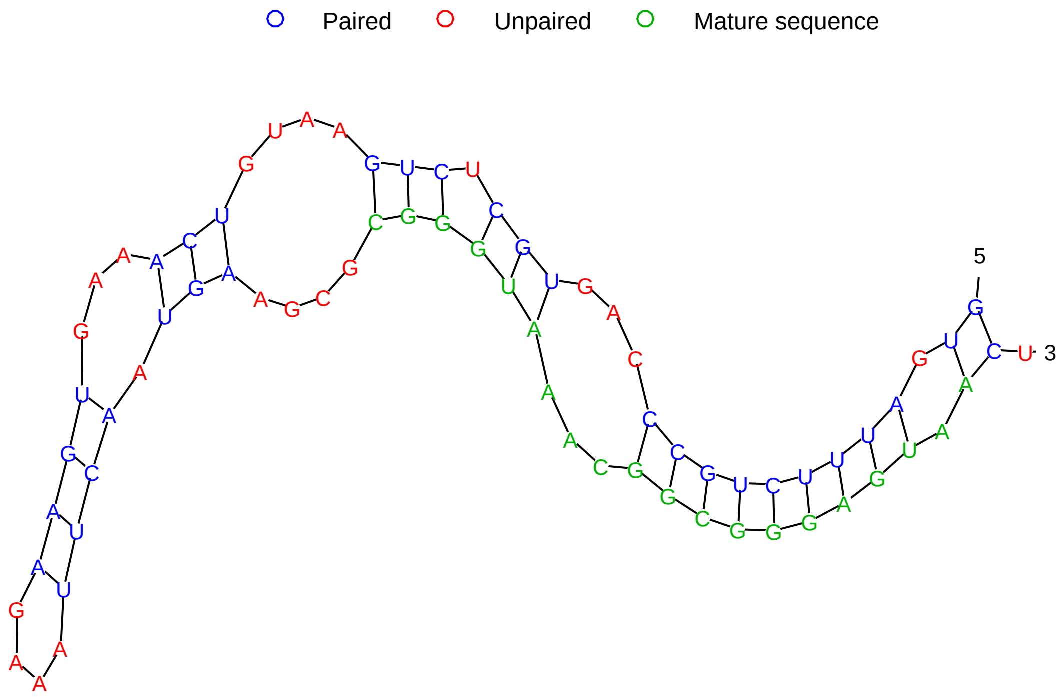

Stem loop (UMD3.1): chrX:62081819-62081892
Mature (UMD3.1): chrX:62081821-62081840
Mature seq len: 20
Total raw counts (9 samples): 1289
Average raw counts: 144
Strand: Reverse
Orientation: 3p
Minimum free energy: -21.50

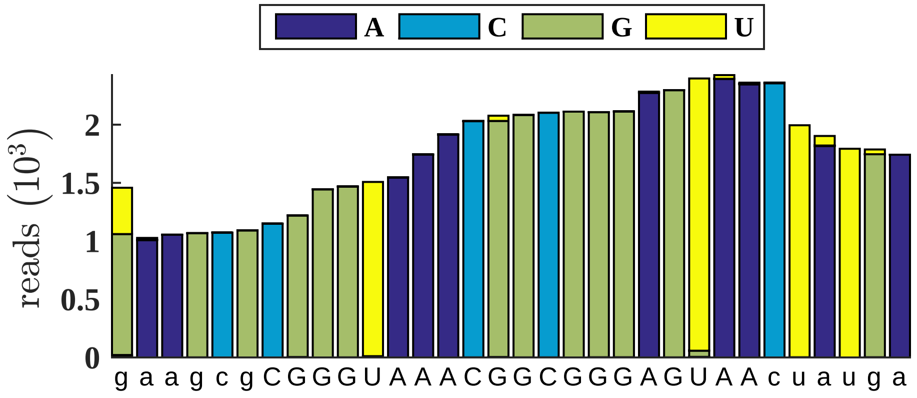

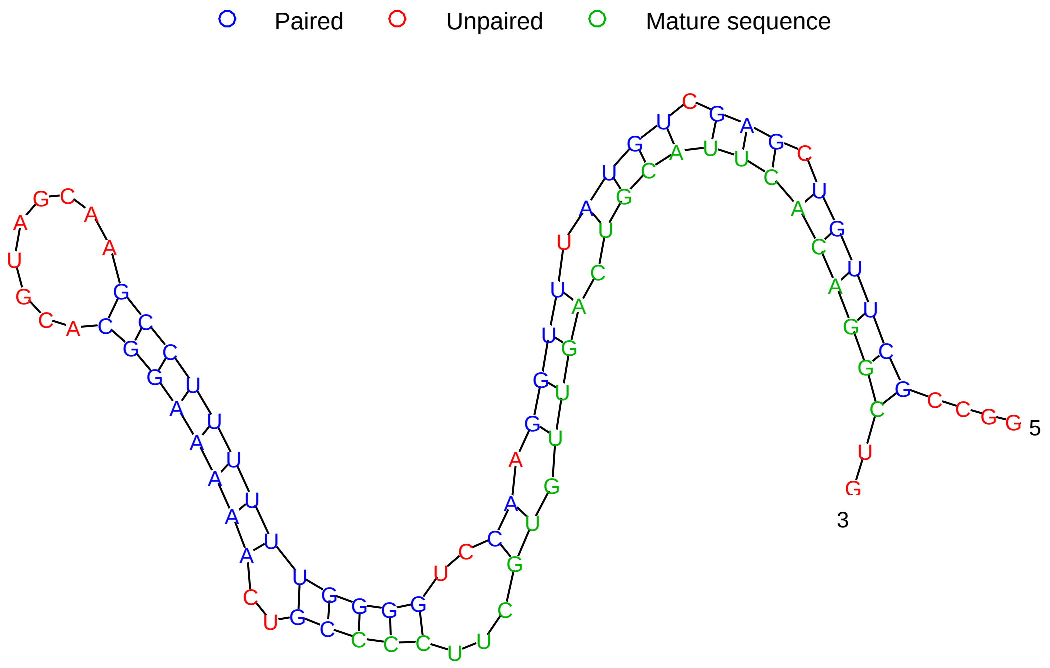

Stem loop (UMD3.1): chrX:83153396-83153487
Mature (UMD3.1): chrX:83153398-83153424
Mature seq len: 27
Total raw counts (9 samples): 1216
Average raw counts: 136
Strand: Reverse
Orientation: 3p
Minimum free energy: -28.90

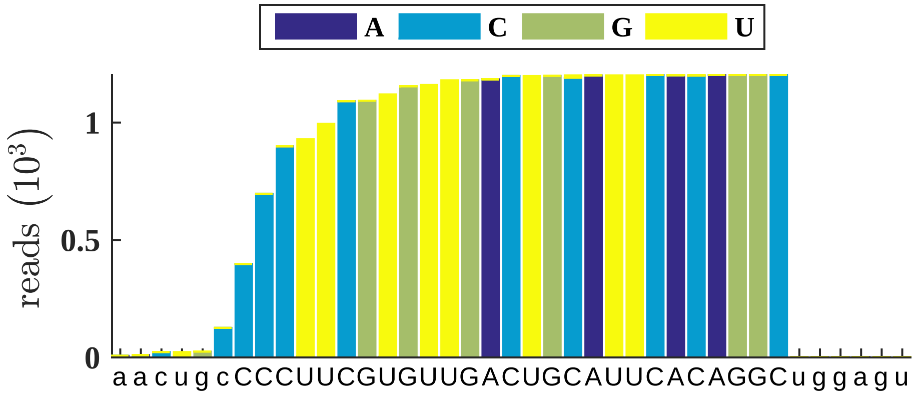

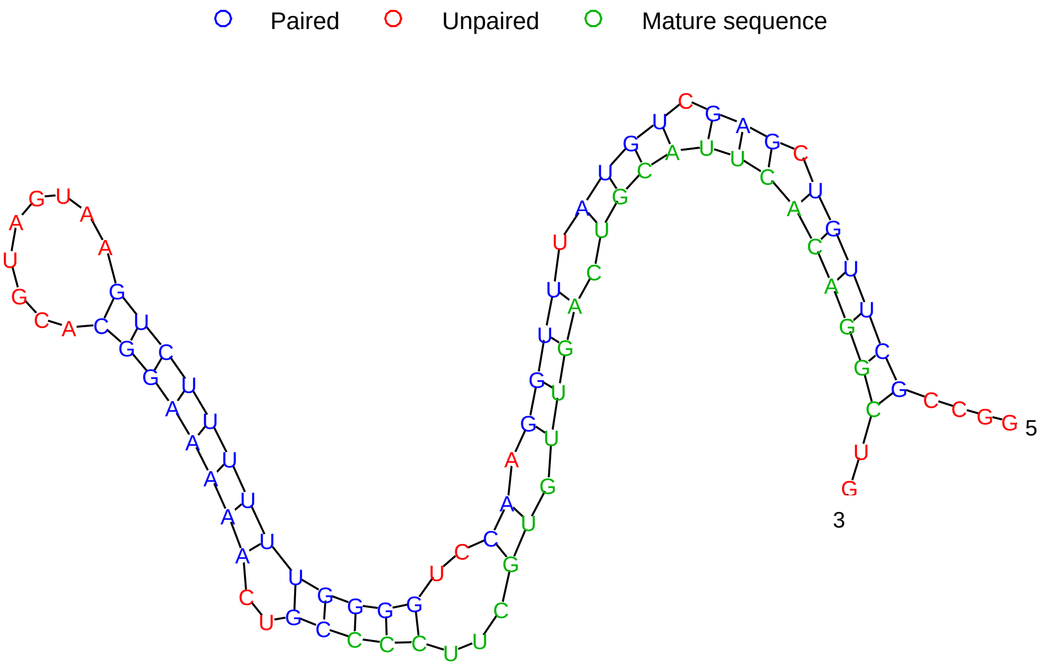

Stem loop (UMD3.1): chrX:83154763-83154854
Mature (UMD3.1): chrX:83154765-83154791
Mature seq len: 27
Total raw counts (9 samples): 1223
Average raw counts: 136
Strand: Reverse
Orientation: 3p
Minimum free energy: -26.70

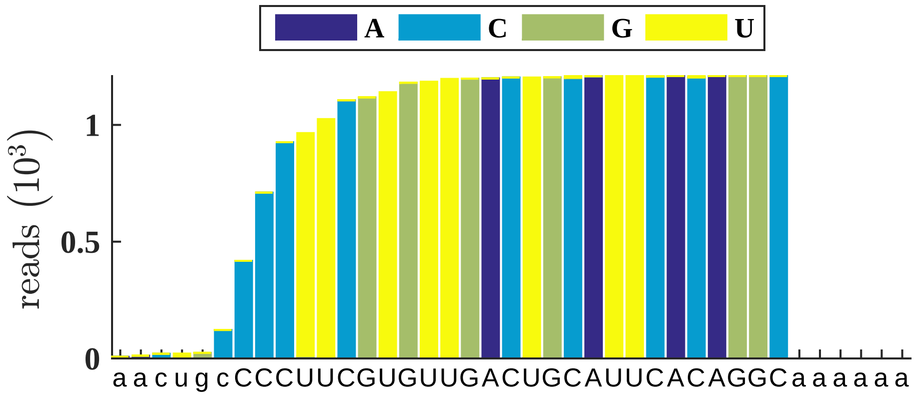

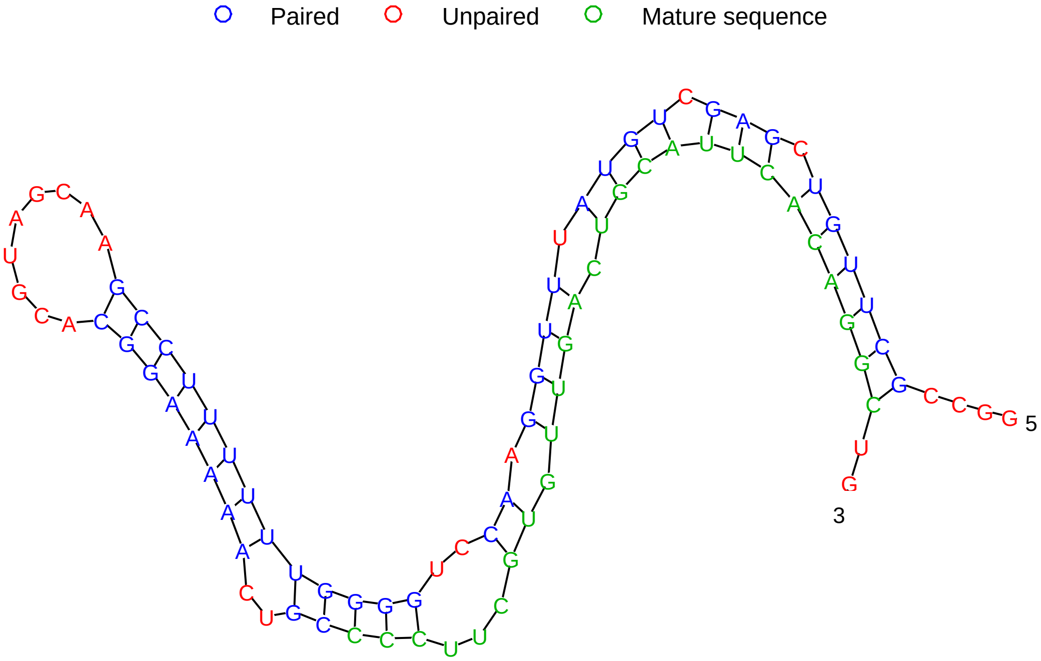

Stem loop (UMD3.1): chrX:83157691-83157782
Mature (UMD3.1): chrX:83157754-83157780
Mature seq len: 27
Total raw counts (9 samples): 1180
Average raw counts: 132
Strand: Forward
Orientation: 3p
Minimum free energy: -28.90

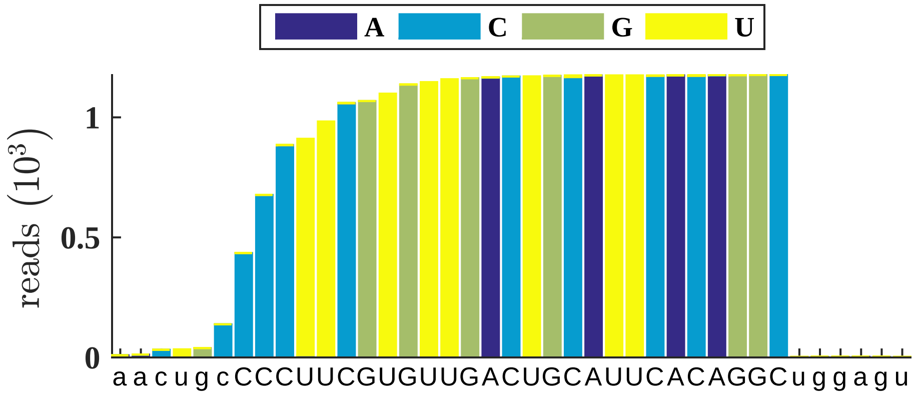

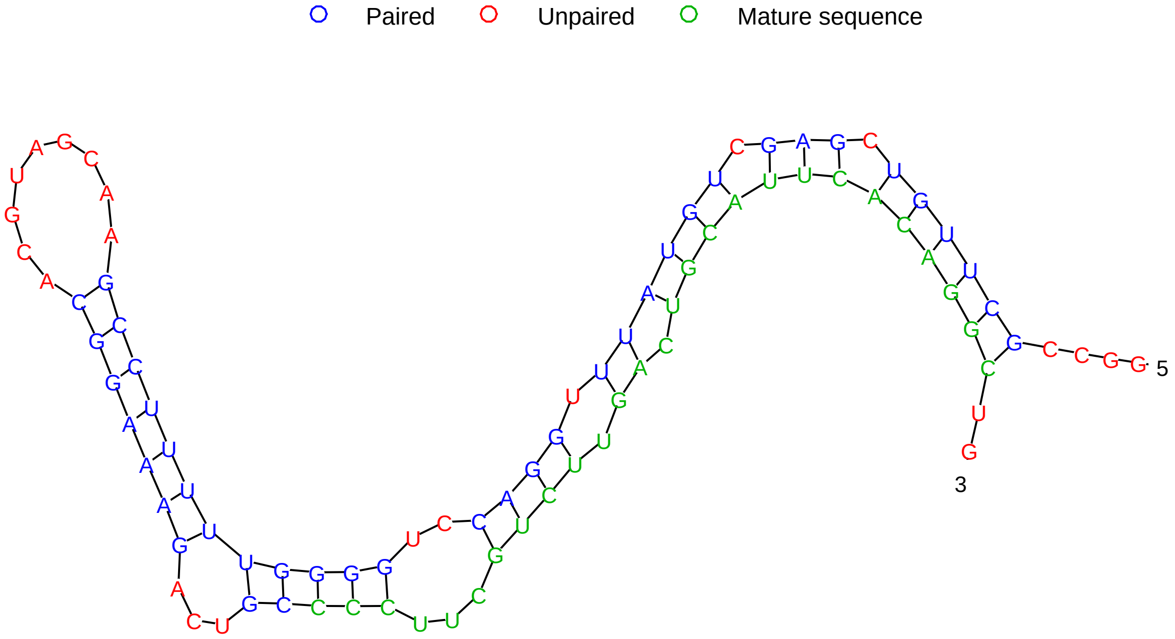

Stem loop (UMD3.1): chrX:83162225-83162314
Mature (UMD3.1): chrX:83162286-83162312
Mature seq len: 27
Total raw counts (9 samples): 4367
Average raw counts: 486
Strand: Forward
Orientation: 3p
Minimum free energy: -30.80

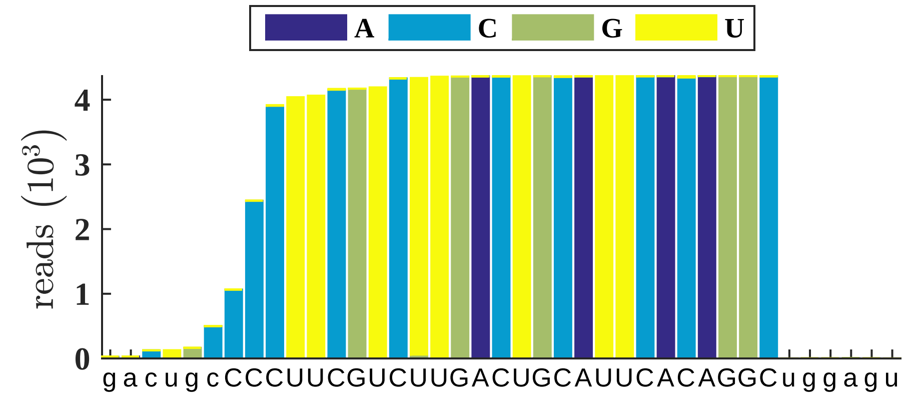

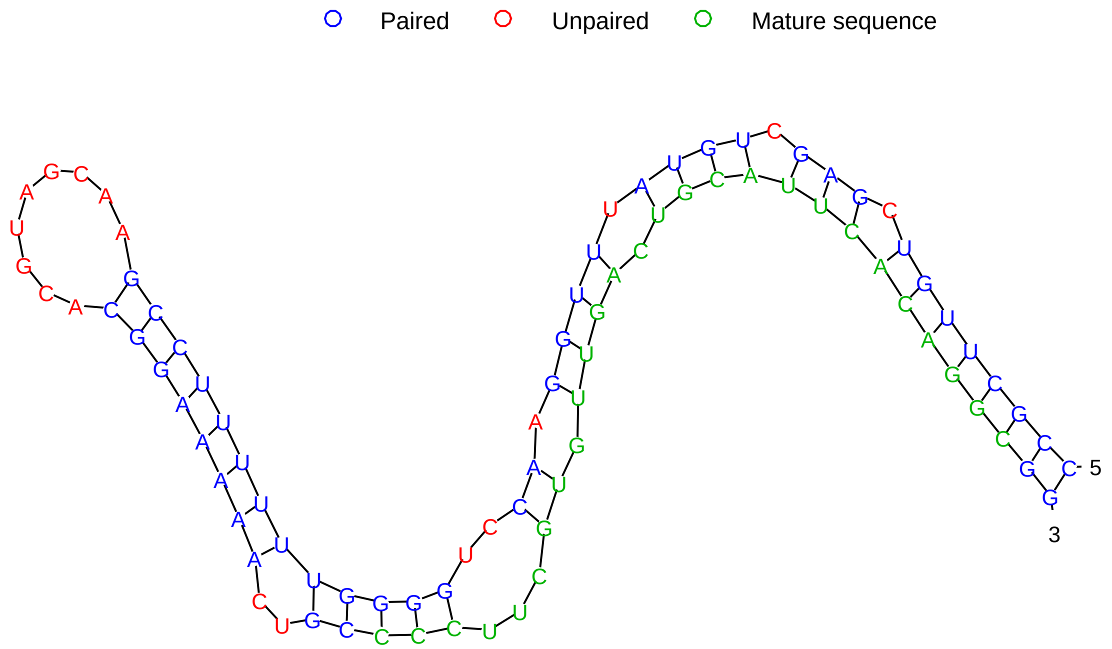

Stem loop (UMD3.1): chrX:83170860-83170949
Mature (UMD3.1): chrX:83170921-83170947
Mature seq len: 27
Total raw counts (9 samples): 1217
Average raw counts: 136
Strand: Forward
Orientation: 3p
Minimum free energy: -33.20

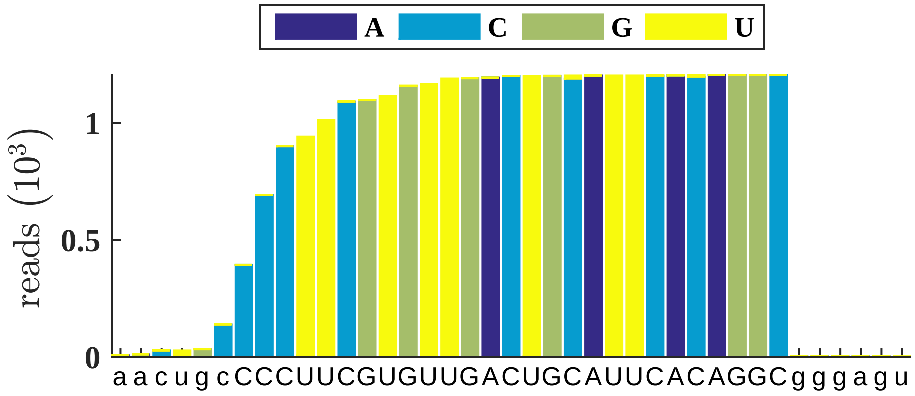

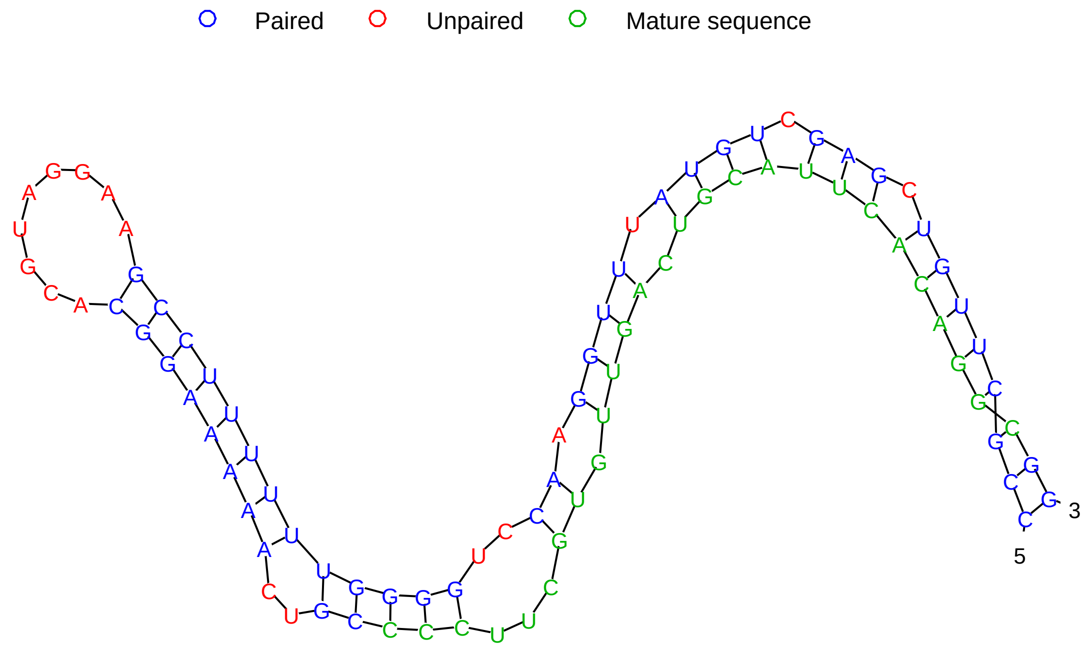

Stem loop (UMD3.1): chrX:83172152-83172241
Mature (UMD3.1): chrX:83172213-83172239
Mature seq len: 27
Total raw counts (9 samples): 1190
Average raw counts: 133
Strand: Forward
Orientation: 3p
Minimum free energy: -33.20

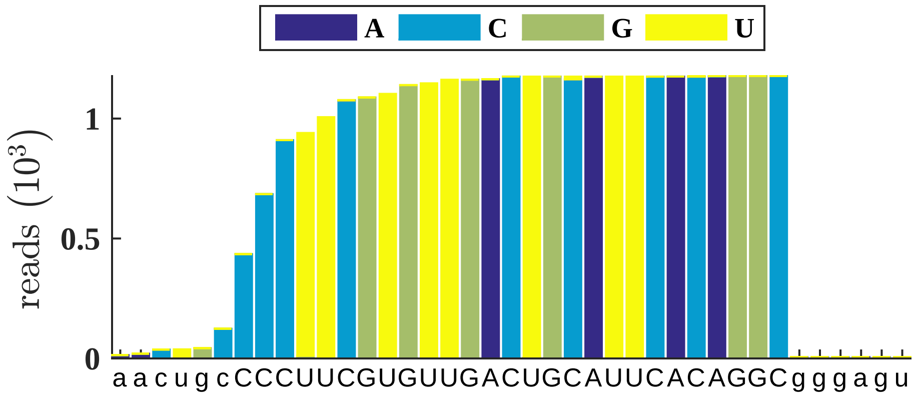

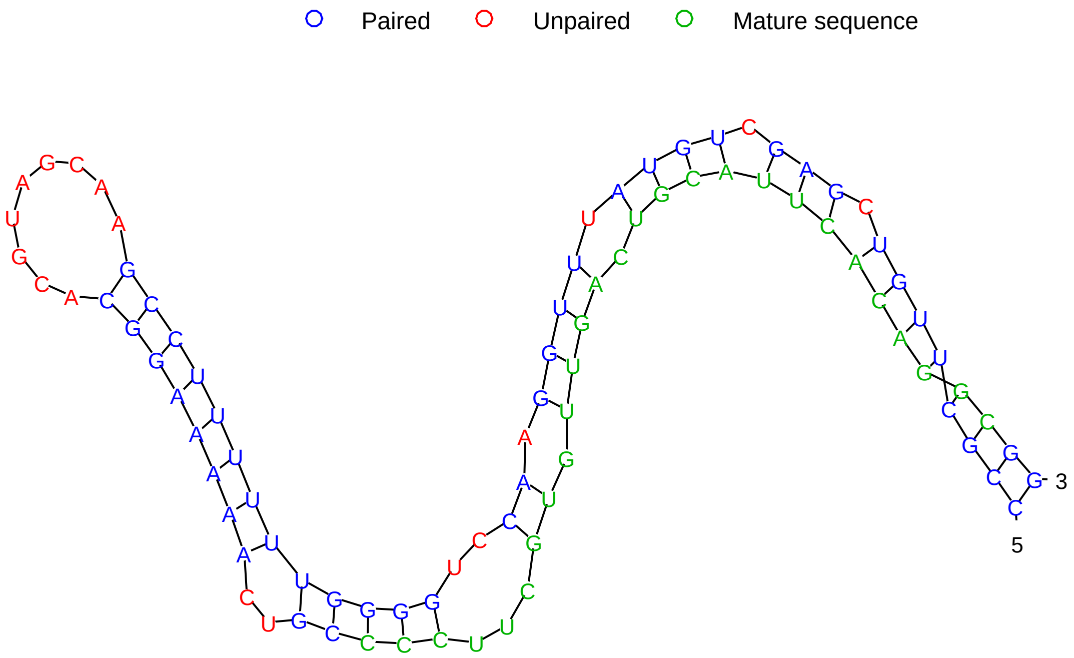

Stem loop (UMD3.1): chrX:83173405-83173494
Mature (UMD3.1): chrX:83173466-83173492
Mature seq len: 27
Total raw counts (9 samples): 1171
Average raw counts: 131
Strand: Forward
Orientation: 3p
Minimum free energy: -33.20

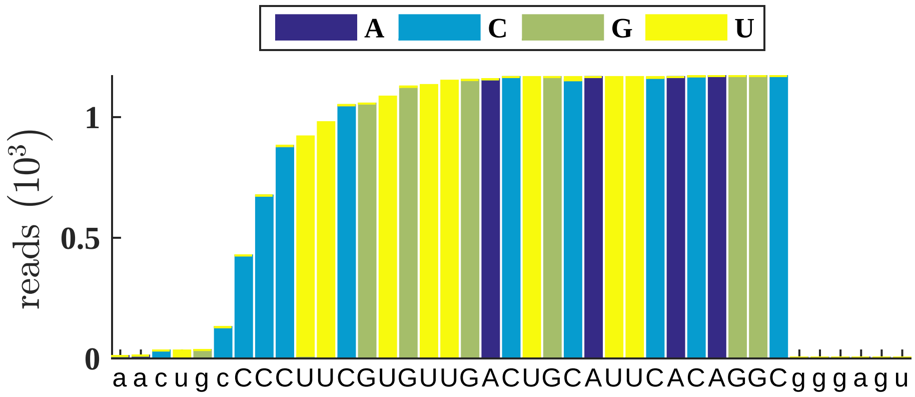

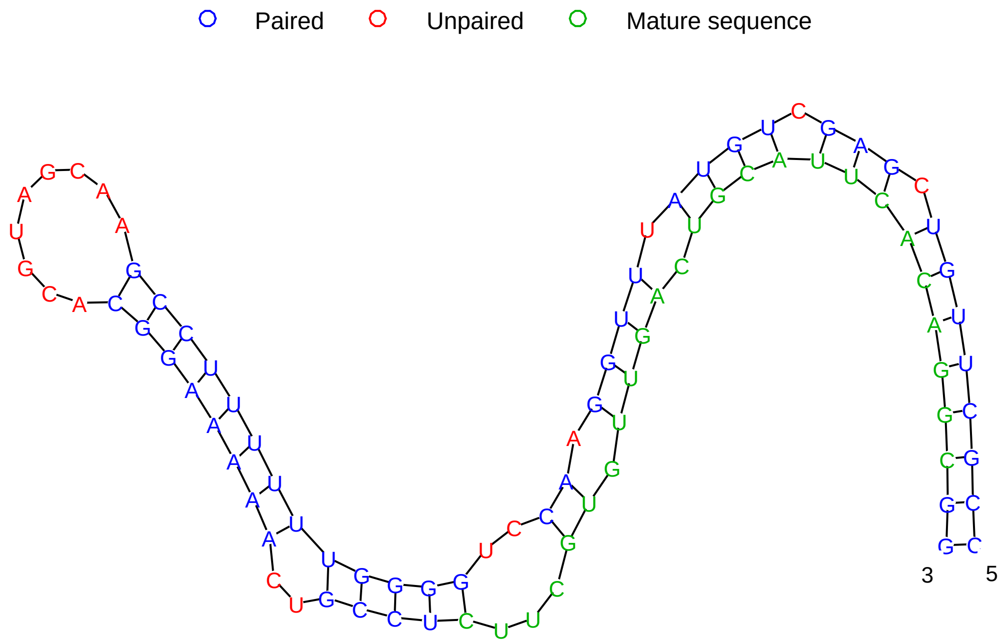

Stem loop (UMD3.1): chrX:83176015-83176104
Mature (UMD3.1): chrX:83176078-83176102
Mature seq len: 25
Total raw counts (9 samples): 650
Average raw counts: 73
Strand: Forward
Orientation: 3p
Minimum free energy: -30.20

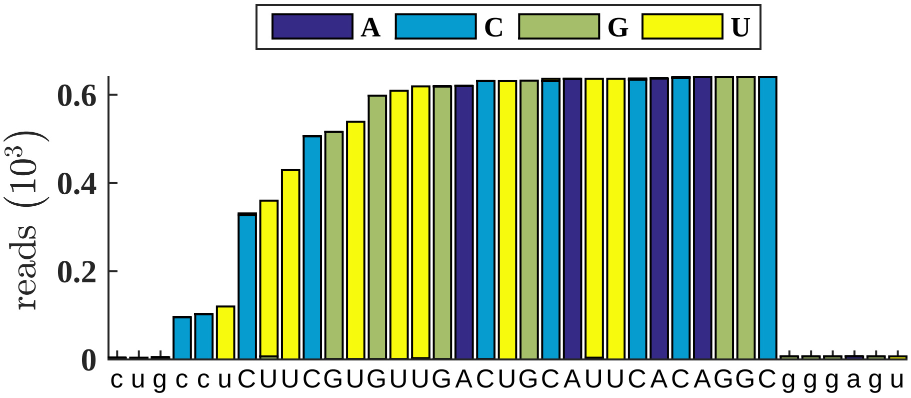

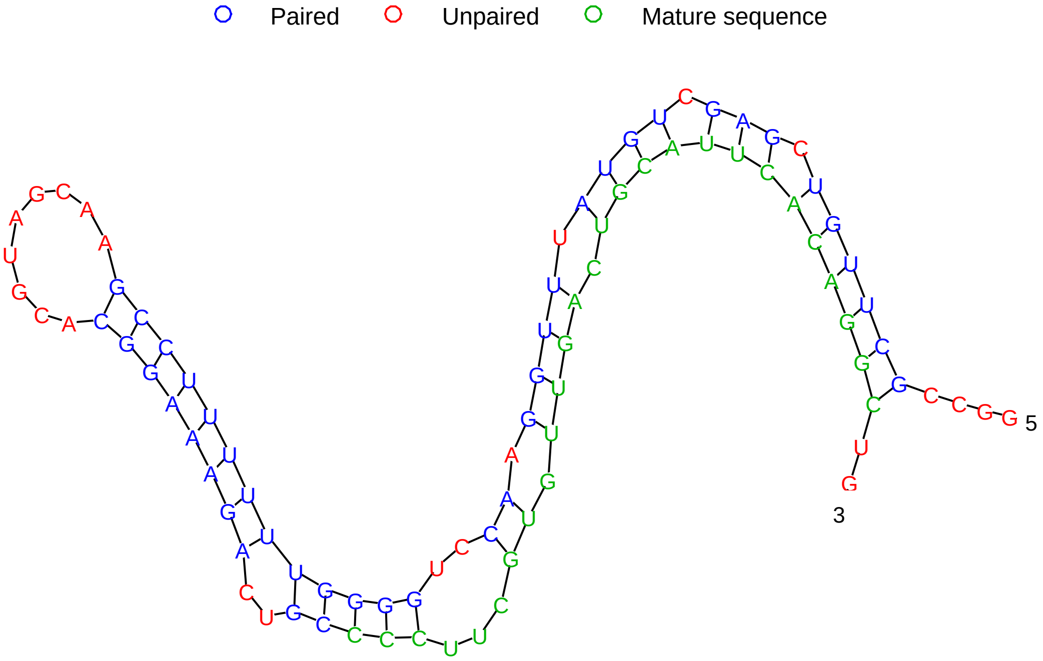

Stem loop (UMD3.1): chrX:83179798-83179889
Mature (UMD3.1): chrX:83179861-83179887
Mature seq len: 27
Total raw counts (9 samples): 1244
Average raw counts: 139
Strand: Forward
Orientation: 3p
Minimum free energy: -29.00

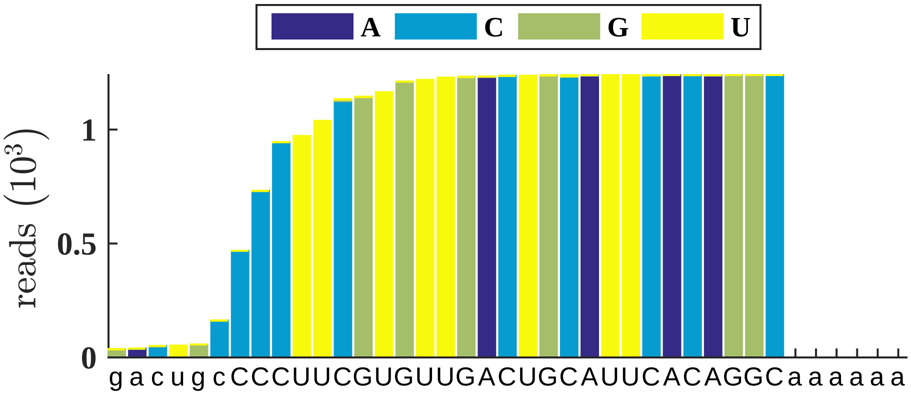

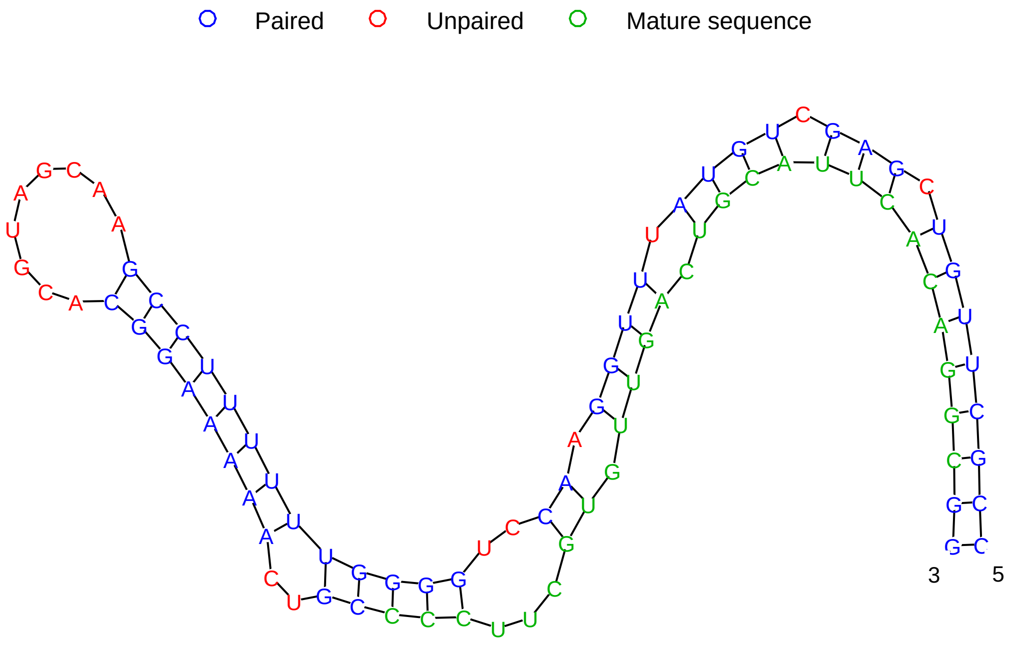

Stem loop (UMD3.1): chrX:83190047-83190136
Mature (UMD3.1): chrX:83190108-83190134
Mature seq len: 27
Total raw counts (9 samples): 1240
Average raw counts: 138
Strand: Forward
Orientation: 3p
Minimum free energy: -33.20

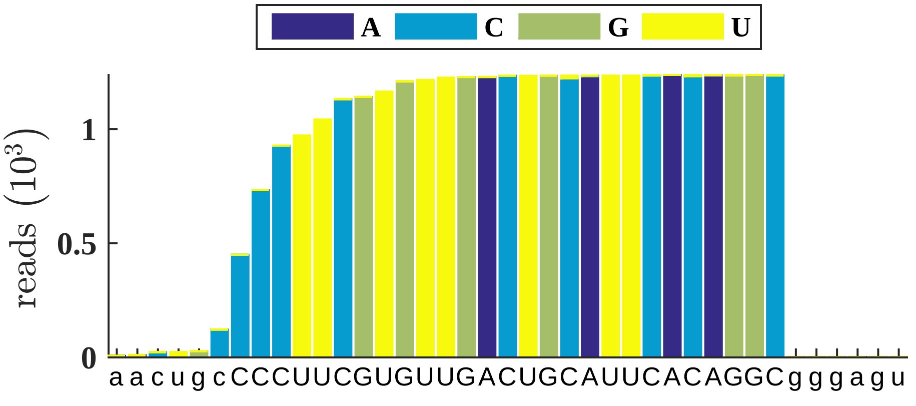

Supplement: Supplementary Table and Figures [file srep25486-s3.pdf]
